# Discovery and characterization of a fourth class of guanidine riboswitches

**DOI:** 10.1093/nar/gkaa1102

**Published:** 2020-11-25

**Authors:** Felina Lenkeit, Iris Eckert, Jörg S Hartig, Zasha Weinberg

**Affiliations:** Department of Chemistry, University of Konstanz, Universitätsstraße 10, 78457 Konstanz, Germany; Bioinformatics Group, Department of Computer Science and Interdisciplinary Centre for Bioinformatics, Leipzig University, Härtelstraße 16-18, 04107 Leipzig, Germany; Department of Chemistry, University of Konstanz, Universitätsstraße 10, 78457 Konstanz, Germany; Bioinformatics Group, Department of Computer Science and Interdisciplinary Centre for Bioinformatics, Leipzig University, Härtelstraße 16-18, 04107 Leipzig, Germany

## Abstract

Riboswitches are RNAs that specifically sense a small molecule and regulate genes accordingly. The recent discovery of guanidine-binding riboswitches revealed the biological significance of this compound, and uncovered genes related to its biology. For example, certain *sugE* genes encode guanidine exporters and are activated by the riboswitches to reduce toxic levels of guanidine in the cell. In order to study guanidine biology and riboswitches, we applied a bioinformatics strategy for discovering additional guanidine riboswitches by searching for new candidate motifs associated with *sugE* genes. Based on *in vitro* and *in vivo* experiments, we determined that one of our six best candidates is a new structural class of guanidine riboswitches. The expression of a genetic reporter was induced 80-fold in response to addition of 5 mM guanidine in *Staphylococcus aureus*. This new class, called the guanidine-IV riboswitch, reveals additional guanidine-associated protein domains that are extremely rarely or never associated with previously established guanidine riboswitches. Among these protein domains are two transporter families that are structurally distinct from SugE, and could represent novel types of guanidine exporters. These results establish a new metabolite-binding RNA, further validate a bioinformatics method for finding riboswitches and suggest substrate specificities for as-yet uncharacterized transporter proteins.

## INTRODUCTION

Riboswitches are structured, non-coding regions in the 5′-untranslated regions (UTRs) of mRNAs that regulate the downstream gene (1–4). They sense metabolites or ions to control gene expression and thereby maintain cellular homeostasis of the cognate ligand, respond to signaling molecules, or detoxify xenobiotics or ions. Riboswitches are composed of two functional components: an aptamer domain and an expression platform (5). The aptamer domain specifically senses a certain ligand. Binding usually induces a structural rearrangement in the expression platform leading to modulation of downstream events (6). This conformational change either represses (OFF-switches) or activates (ON-switches) gene expression. Gene expression is predominantly controlled by acting on transcription termination (7,8) or translation initiation (9,10). Well-known examples of riboswitch regulation include the control of co-enzyme, amino acid, and nucleotide metabolism (5). The discovery of additional riboswitch classes yields a variety of benefits. Since riboswitches are unique in their ability to directly bind small molecules and ions without the need for intermediate molecules such as proteins, they can be utilized as convenient biotechnology tools in order to control gene expression in engineered systems (11–13). The discovery of additional riboswitch classes and their associated regulatory networks will also help to understand functions of associated genes and their encoded proteins (2), and enables investigations into RNA structure and biochemistry (14).

For well over a decade, the most successful methods of discovering new riboswitch classes have been bioinformatics approaches based on a comparative strategy (1,2,15–18). The common element in such approaches is that they analyze homologous intergenic regions for ‘covariation’, which are mutations that change the primary sequence but conserve an RNA secondary structure. Such mutations are a distinct feature of structured RNAs. Good riboswitch candidates show, among other features, significant covariation and are consistently located upstream of protein-coding genes, which they are expected to regulate (18). The ligands of such candidates can often be identified by analyzing the regulated genes. However, in certain cases where the gene context is too diverse or the majority of gene functions is still unknown, this approach is limited. Riboswitch candidates whose ligands remain to be identified are called orphan riboswitches. For example, in 2004, the *ykkC-yxkD* RNA motif (15) was identified, but its ligand was unknown for well over a decade, due to challenges posed by the wide variety of associated genes and their unknown function. Being a common motif in various bacterial phyla, it was found upstream of genes encoding for multidrug efflux pumps and other transporters, urea carboxylases, purine and amino acid metabolism enzymes, among other gene products (15,19). Many of these gene classes were also associated with two additional orphan riboswitches that were identified later: the mini-*ykkC* (16) and *ykkC*-III motifs (17). Their consensus sequences did not show structural similarity between the orphan motifs, but the similar genetic contexts of all three motifs suggested the hypothesis that they sense the same ligand (16,17). After many years of efforts, guanidine was eventually revealed as the cognate ligand of the three motifs, now renamed guanidine-I, -II and -III riboswitches (20–22). The discovery of the widespread occurrence of guanidine-binding riboswitches is remarkable since, at the time, guanidine was not known to play a role in biology. Rather it has been used as a propellant, an additive in plastics, as well as a chaotropic substance in protein biochemistry. The occurrence of a riboswitch sensing guanidine suggested that it occurs naturally and is toxic at high concentrations. Accordingly, guanidine riboswitches control genes whose protein products are crucial for overcoming this toxicity. The most widely associated function, encoded by genes known as *sugE* or *emrE*, was subsequently demonstrated to export guanidine (23). Furthermore, earlier work established the existence of urea carboxylase enzymes, but riboswitch-associated genes that had been predicted to encode these enzymes have since been demonstrated to favor guanidine over urea (20). Due to the widespread occurrence of the three known guanidine riboswitch classes, it has been speculated that further guanidine riboswitches could exist (2).

Here, we exploited gene contexts of known guanidine riboswitches in combination with a discovery strategy based on comparative genomics in order to find additional classes of guanidine riboswitches. The earliest application of a similar strategy was in the discovery of the SAM-III riboswitch (also called the S_MK_-box riboswitch), which binds *S*-adenosylmethionine (SAM) (24). This riboswitch was found because certain species conspicuously lacked examples of the then-known SAM riboswitch classes. Since the known SAM riboswitch classes often occur upstream of *metK* genes, encoding SAM synthetase, a manual analysis was conducted to find conserved patterns upstream of *metK* genes in the targeted species. We previously applied a more comprehensive and automated version of this strategy by analyzing all known gene classes without regard to lineage to find *cis*-regulatory RNAs (18). We have begun a project to apply an updated version of this strategy to accommodate large sequence datasets now available. To begin, we exploited this approach to find candidate guanidine riboswitches. We demonstrate that one of our candidates is a fourth class of guanidine riboswitches that acts via transcription termination control.

## MATERIALS AND METHODS

### Bioinformatics

To analyze guanidine riboswitches, we used the bacterial and archaeal portions of version 87 of the RefSeq nucleotide database (25). We also used metagenomic and metatranscriptome data collected from a variety of sources, predominantly from IMG/M (26) and GenBank (25). Where gene annotations were not available, they were predicted with MetaProdigal (27), and conserved domains were annotated using the Conserved Domain Database (CDD) (25) version 3.16. Proteins containing matches to the COG2076 or pfam00893 models in the CDD were assumed to be SugE. The intergenic regions (IGRs) upstream of the corresponding genes were extracted, and subjected to the method described in (18) to find conserved RNA structures. Briefly, this method clusters conserved regions within IGRs using BLAST (28) and overcluster2 (18). Structured alignments were predicted by the CMfinder program (18,29) and scored using the ScoreMotif.pl script in version 0.4.1.18 of the CMfinder package (18). Manual analysis of covariation and promising alignments proceeded with the considerations outlined previously (30). In particular, while the R-scape (31) software provides a statistically well-founded measurement of covariation evidence, it is not foolproof. For example, incorrectly aligned sequences can create spurious covariation signals. As before (18), we further analyzed computer predictions by iteratively investigating potential new or alternate stems using CMfinder and R-scape, and searching for additional homologs using Infernal (32). Such homologs can reveal variation that helps to refine the structure predictions, or even leads to the conclusion that the originally proposed structure is unlikely to be conserved. Known RNAs were annotated using version 14.0 of the Rfam database (33). Motifs were drawn using R2R (34), but covariation was primarily depicted based on R-scape (31) using the -s flag. We used RNie (35) to predict Rho-independent transcription terminators.

### Oligonucleotides and chemical

All synthesized oligonucleotides were purchased from Sigma-Aldrich. [γ-^32^P]- and [α-^32^P]-ATP used for RNA labeling was purchased from Hartmann Analytic. Oligonucleotide sequences are listed (Supplementary Tables S1 and S2). Guanidine hydrochloride, urea and arginine were purchased from Roth, amino-guanidine hydrochloride and methyl-guanidine hydrochloride from Acros Organics.

### RNA oligonucleotide preparation

DNA templates for RNA synthesis were generated using T7-promoter-containing primers via overlap extension reaction using SuperScript™ II Reverse Transcriptase (Thermo Fisher). Templates were purified via Zymo DNA Clean & Concentrator™ Kit and *in vitro* transcription reactions were performed using T7 RNA polymerase (NEB). For purification of the RNA, a 10% polyacrylamide gel electrophoresis (PAGE) gel was used. After extraction of the RNA, ∼80 pmol were dephosphorylated using Shrimp Alkaline Phosphatase (NEB), following the manufacturer's instruction. ∼20 pmol of the dephosphorylated RNA were [γ-^32^P]-labeled at the 5′ terminus using T4 polynucleotide kinase (NEB) and 20 μCi [γ-^32^P]-ATP to be incubated for 1 h at 37°C. The reaction was stopped by adding a 2× urea denaturing loading buffer and purified by 10% PAGE gel. After RNA extraction and precipitation, the pellet was dissolved in water to obtain a concentration of 1 kBq/μl.

### In-line probing reaction

The in-line probing reaction was performed as previously described (36,37). In a 10 μl reaction 1 kBq of [γ-^32^P]-labeled RNA was incubated in the presence or absence of a desired ligand and with 20 mM MgCl_2_, 100 mM KCl and 50 mM Tris–HCl (pH 8.3 at 23°C) for ∼48 h. The reactions were subsequently analyzed via 10% PAGE and visualized using a phosphorimager (GE Healthcare Life Sciences). Band intensities were quantified using ImageQuant. Fraction bound values were calculated by quantification of changes in the intensity at certain positions that show modulation. To correct for loading differences between samples, the values were normalized with band intensities of a position that does not show any modulation due to ligand binding.

### Transcription termination assay

DNA templates containing the T5 promotor, the *GGAM-1* motif (explained below) RNA and the downstream natural sequence, extending through the first 31 nucleotides of the *sugE* gene, were amplified using PCR. PCR products were purified with Zymoclean™ Gel DNA Recovery Kit. A 10 μl reaction with 10 ng/μl DNA template, 1.8 mM NTPs, 2 μCi [α-^32^P]-ATP, *Escherichia coli* T5 Polymerase and the desired ligand was incubated for 8 min at 37°C. The reaction was subsequently analyzed via 10% PAGE and visualized using a phosphorimager (GE Healthcare Life Sciences). Full-length product and termination product bands were quantified with ImageQuant. Tested sequences are listed in Supplementary Table S3.

### Genetic reporter assays

*Staphylococcus aureus* RN4220 were cultivated in liquid cultures (BHI-medium) at 37°C and 200 rpm or on BHI-agar plates at 37°C. Liquid overnight cultures were grown until OD_600_ ∼6. As reporter plasmid, pCN-Pblaz-GFP was used, kindly provided by the Romby Group (University of Strasbourg, Strasbourg, France). For transformation, electrocompetent *S. aureus* cells were thawed on ice and incubated with 1 μg of non-methylated DNA in a volume of no more than 10 μL for 30 min. Cells were transferred to electroporation cuvettes with a 2 mm gap and pulsed with 1.8 kV for 2.5 ms using a Gene Pulser (BioRad). The electroporated cells were quickly resuspended in 900 μl of pre-warmed BHI medium and incubated for 2 h at 37°C under agitation. The cells were spread on BHI plates containing 10 μg/ml erythromycin and incubated overnight at 37°C. Three single colonies for each transformed plasmid were cultivated in 400 μL BHI-Medium in a 96-deepwell plate, overnight at 37°C at 1300 rpm. To 400 μl of fresh BHI-medium with and without guanidine hydrochloride in a 96-deepwell plate, 10 μl of the overnight culture were added, and technical triplicates were carried out. The plates were incubated overnight at 37°C at 1300 rpm. From each culture, 100 μl were transferred to a UV transparent flat-bottomed 96-well plate. GFP expression and OD_600_ measurements were performed using a Tecan plate reader. For GFP measurements, the excitation wavelength was set to 488 nm and emission wavelength to 535 nm. GFP expression was normalized to OD_600_.

## RESULTS

### Candidate guanidine riboswitches

**Figure 1.**
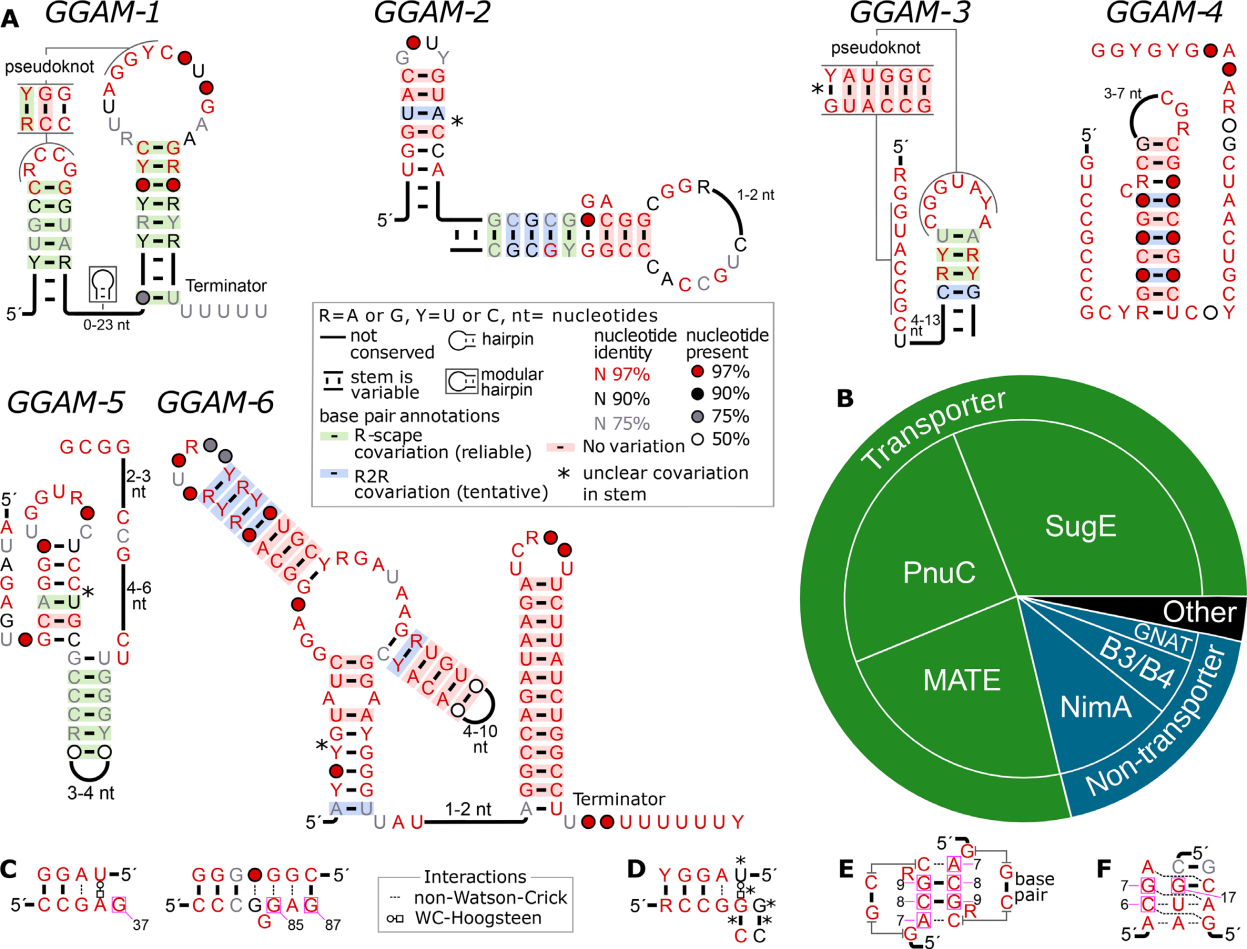
Candidate guanidine riboswitches: the *GGAM* RNA motifs. (**A**) Diagram showing conserved features of RNA sequences in *GGAM-1* to *GGAM-6*. ‘Terminator’: this stem exhibits the properties typical of Rho-independent transcription terminator hairpins. A legend (gray box) explains other symbols. (**B**) Genes frequently associated with *GGAM-1* RNAs. The six conserved protein domains most commonly encoded by genes that are immediately downstream of *GGAM-1* RNAs. Such genes are almost certainly regulated by the riboswitch, in comparison to genes that might be located in extended operons. Three domains function as transporters (green), while the other three do not (blue). Less common domains and domains that did not match the Conserved Domain Database were classified as ‘other’ (black). Additional information on the genes is available (Supplementary Table S4, Supplementary File 1). (**C**) Conserved regions around three nucleotides in the guanidine-I riboswitch that directly bind the ligand (purple boxes), and are numbered according to a previously established crystal structure (39). Non-canonical base pairs are shown as dashed lines, but a *trans* Watson-Crick-Hoogsteen interaction is shown in Leontis-Westhof Notation (40). Conservation levels are taken from a previous consensus structure (20). Most nucleotides are at least 97% conserved (red). (**D**) A possibly similar region within *GGAM-1* RNAs, depicted as if resembled the region in part C. The region's left side is the pseudoknot in part A. Asterisks indicate important incompatibilities to the guanidine-I structure (see text). (**E**) Ligand-contacting nucleotides and surrounding regions for guanidine-II riboswitches, based on previous crystal structures (41,42) and consensus studies (21,42). Annotations are like in part C. (**F**) Binding pocket of guanidine-III riboswitches, based on a previous crystal structure (43) and consensus study (22). Annotations are as in part C.

All three previously characterized guanidine riboswitches frequently occur upstream of multidrug exporters encoded by *sugE* genes, also called *emrE* (2). Therefore, we extracted intergenic regions (IGRs) upstream of such genes in all bacteria, and applied a pipeline (18) to find examples of conserved secondary structure. After a detailed analysis (30) of computationally predicted alignments, we established six candidates (Figure 1A, Table 1, Supplementary Files 1 and 2). We call these Guanidine-Gene-Associated Motifs (*GGAM*).

**Table 1.** Properties of candidate guanidine riboswitches. ‘Name’: our name for the motif. ‘Is RNA?’: our subjective judgment as to whether the motif is an RNA. ‘Y’: clear evidence. ‘y’: probably RNA. ‘?’: more borderline candidate. ‘#’: number of examples of the motifs in the databases we searched. ‘Lineage’: Taxon containing all organisms with this motif. Where the motif only occurs in a single species, the phylum is also given. In calculating lineages, genomes derived from metagenomes were ignored. ‘# R-scape cov.’: number of base pairs exhibiting statistically significant covariation, according to R-scape (31). Note: not all base pairs are shown in Figure 1, because highly variable regions are not explicitly depicted. ‘# R2R cov.’: number of base pairs that exhibit some covariation, according to R2R’s permissive test (34), but not R-scape's test

Name	Is RNA?	#	Lineage	# R-scape cov.	# R2R cov.
*GGAM-1*	Y	2,882	Phyla: Actinobacteria, Firmicutes, Fusobacteria, Proteobacteria, Spirochaetes, Synergistetes	31	4
*GGAM-2*	y	129	Phylum: Actinobacteria	5	4
*GGAM-3*	y	383	Class: Betaproteobacteria	13	1
*GGAM-4*	?	8	Species: *Methyloceanibacter superfactus* (Phylum: Proteobacteria)	0	3
*GGAM-5*	Y	328	Class: Alpha- and Betaproteobacteria	7	1
*GGAM-6*	?	8	Species: *Selenomonas ruminantium* (Phylum: Firmicutes)	0	6

Of particular interest was *GGAM-1* (Figure 1A), because it has several properties that are expected of riboswitches (18). First, it has several highly conserved nucleotides. Moreover, the motif includes sequences present in multiple phyla (Table 1, Supplementary File 1). The *GGAM-1* motif occurs most often in the phylum Firmicutes, and is also present in species from six other phyla. Since nucleotides are highly conserved, despite the RNAs being highly diverged across phyla, the RNA appears to be subject to strong biochemical constraints, which is expected of an RNA that specifically binds a small molecule. Second, the *GGAM-1* includes a potential pseudoknot. Pseudoknots are often associated with riboswitches (18). Third, *GGAM-1* RNAs consistently occur upstream of protein-coding genes, and they encode multiple non-homologous proteins (Figure 1B). This observation is strongly consistent with a *cis*-regulatory function. Finally, the *GGAM-1* motif's structure contains a predicted Rho-independent transcription terminator (6). Such terminators consist of a hairpin followed by several U nucleotides, and cause the transcription process to stop. They are a common expression platform in riboswitches.

As expected for a guanidine riboswitch, *GGAM-1* RNAs are most commonly located upstream of *sugE* genes (Figure 1B, Supplementary Table S4). However, we also noticed several gene classes apparently regulated by *GGAM-1* RNAs, some of which are rarely or never associated with previously established guanidine riboswitches (Figure 1B, Supplementary Table S4). These new gene associations could suggest additional genes with a guanidine-related function.

We considered the possibility that the *GGAM-1* motif is structurally related to one of the previously established guanidine riboswitch classes. For example, the SAM-IV riboswitch was discovered using a *de novo* bioinformatics strategy (37), and is structurally similar to the SAM-I riboswitch (37,38). The initial analysis of SAM-IV riboswitches found that they strongly conserve five of the six ligand-contacting nucleotides of SAM-I riboswitches, and that these ligand-contacting nucleotides occur in a similar context of secondary structure in SAM-I and -IV riboswitches (37). Therefore, in analyzing possible relationships between the *GGAM-1* motif and guanidine-I, -II and -III riboswitches, we looked for conservation of most of the ligand-contacting nucleotides that might occur in analogous structural contexts. In comparing structures, we took into the account that we might have missed some Watson-Crick interactions, and have made no attempt to discover non-Watson-Crick interactions.

A crystal structure of a guanidine-I riboswitch shows three nucleotides that directly contact the ligand (39) (Figure 1C). All of these nucleotides and most surrounding nucleotides are highly (at least 97%) conserved (20). The pseudoknot within the *GGAM-1* motif somewhat resembles one of these regions (Figure 1D). However, there are important deviations that make it unlikely that the regions are structurally interchangeable (Figure 1D, asterisks). First, two highly conserved nucleotides in the guanidine-I riboswitch would correspond to less conserved nucleotides in the *GGAM-1* motif (Figure 1D, black nucleotides). Moreover, two nucleotides in the *GGAM-1* motif are involved in Watson–Crick base pairs that are supported by covariation (Figure 1A), and such pairings are not observed in the guanidine-I riboswitch structure. Importantly, the *GGAM-1* nucleotide that is hypothetically analogous to the ligand-contacting G37 position (Figure 1C) is not highly conserved and is involved in a Watson-Crick pairing, which are two major deviations from the G37 position of guanidine-I. Finally, if the regions were structurally congruent, the *GGAM-1* motif would contain a U-G pair in a *trans* Watson–Crick/Hoogsteen interaction (Figure 1D). However, such interactions are very rare and do not appear capable of substituting for the U-A pair present in the guanidine-I riboswitch (40). Moreover, the other ligand-binding nucleotides in the guanidine-I riboswitch are G67 and G85 (Figure 1C). There are only two very highly conserved G nucleotide remaining in the *GGAM-1* motif, and their distances and surrounding nucleotides do not resemble those of G67 and G85. Therefore, there is insufficient evidence to suggest that *GGAM-1* RNAs and guanidine-I riboswitches share a structurally related binding pocket.

The most important nucleotides in the guanidine-II riboswitch binding pocket are two highly conserved ACG trimers that bind each other (41,42) (Figure 1E). There are no conserved ACG trimers in the *GGAM-1* motif (Figure 1A). A key part of the binding core of guanidine-III riboswitches (8) is characterized by a highly conserved CG dimer (22,43) (Figure 1F). The single CG dimer in the *GGAM-1* motif does not occur in a similar structural context to its position in the guanidine-III riboswitch: the C nucleotide in the *GGAM-1* motif likely participates in a Watson–Crick base pair, unlike the C6 position in the guanidine-III riboswitch structure, and there are no highly conserved A nucleotides on either side of this dimer, like there are in the guanidine-III riboswitch. Similarly, there is no highly conserved G nucleotide in *GGAM-1* RNAs whose structural context could resemble that of G17 (Figure 1F) in guanidine-III riboswitches. Thus, there is no model to suggest a meaningful similarity between the *GGAM-1* motif and any previously established guanidine riboswitch.

The remaining candidates *GGAM-2* to *-6* also exhibit covariation, although they are not as strong riboswitch candidates as *GGAM-1*. The *GGAM-4* and *GGAM-6* motifs do not include covariation according to R-scape's statistical test, which considers each base pair in isolation. However, these motifs do exhibit covariation in multiple base pairs that are statistically insignificant in isolation, but taken together qualitatively suggest conservation of an RNA structure. The remaining candidates had at least two base pairs that passed R-scape's test. None of the motifs other than *GGAM-1* are present in more than one phylum. Indeed, the small number of examples of *GGAM-4* and *GGAM-6* RNAs (Table 1) implies a lack of variation, which could explain the lack of statistically significant covariation.

*GGAM-6* RNAs are consistently associated with putative Rho-independent transcription terminators, but the remaining four *GGAM* motifs lack obvious expression platforms. *GGAM-2*, *-3*, *-4* and *-6* RNAs were exclusively found upstream of *sugE* genes. The *GGAM-5* motif is found upstream of a variety of genes, of which *sugE* is the most common (Supplementary File 1). These other genes are not, however, annotated with a precise biochemical function. Initial experiments showed that the motifs *GGAM-2* to *-6* did not exhibit binding to guanidine assayed by in-line and *in vitro* transcription experiments (data not shown; for sequences tested see Supplementary Table S2), and they were not further pursued. In subsequent sections, we demonstrate that the *GGAM-1* motif corresponds to a class of guanidine riboswitches.

### Guanidine binding to the *GGAM-1* motif

**Figure 2.**
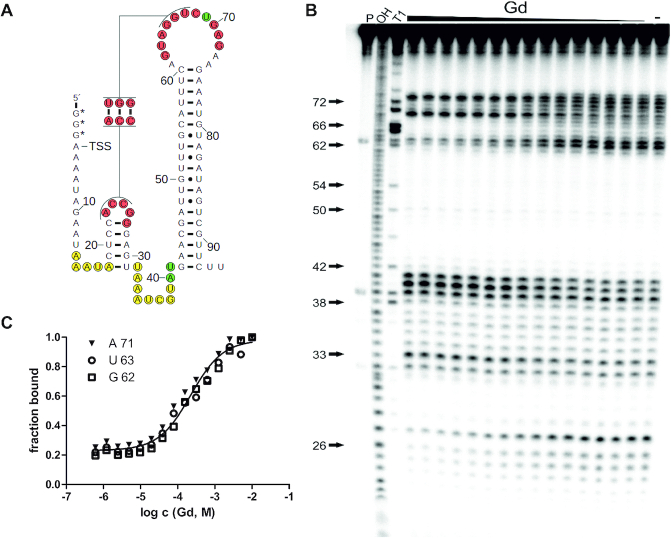
The *GGAM-1* RNA motif binds guanidine. (**A**) Sequence and secondary structure of *95 Lla* RNA construct from the 5′-UTR of the *sugE* gene of *L. lactis*. The 5′ terminus of the construct includes three non-genomic guanosine nucleotides, indicated with asterisks, to improve *in vitro* transcription efficiency. Nucleotides are numbered from the 5′ end. They are colored according to the changes in spontaneous cleavage rates (see part B) upon addition of guanidine. Red: cleavage rate decreases with guanidine. Green: cleavage increases. Yellow: cleavage is high with and without guanidine. No color: cleavage is low in both conditions. (**B**) PAGE analysis of an in-line probing reaction of 5′ ^32^P-labeled *95 Lla* RNA without (−) or with guanidine hydrochloride in a range of 0.61 μM – 10 mM. P, OH and T1 represent 5′ ^32^P-labeled RNA undergoing no reaction, digest under alkaline conditions, or digest with RNase T1, respectively. Selected nucleotide numbers from part A are indicated. (**C**) Plot of the fraction of RNA bound to ligand as a function of the logarithm (base 10) of the molar guanidine hydrochloride concentration. Fraction of RNA bound was determined based on quantification of band intensity changes at each of G 62, U 63 and A 71, normalized by the intensity of the constant band U 33. A trendline was generated using a sigmoidal dose-response curve fit (maximum value equal to 1) to determine an apparent *K*_D_ value. A mean apparent *K*_D_ of 210 μM (±20 μM) was determined in three independent experiments (Supplementary Figure S4A, B).

To test our hypothesis regarding the ligand of the *GGAM-1* riboswitch candidate, a 95-nucleotide-long RNA construct (*95 Lla*) (Figure 2A) from the 5′-UTR of the *sugE* gene of *Lactococcus lactis* was investigated in an in-line probing reaction. In-line probing relies on the inherent chemical instability of RNA and its tendency to undergo spontaneous cleavage of phosphodiester linkages (36,44). By detecting changes in spontaneous RNA degradation in response to ligand binding, the method gives information about the RNA structure and the direct binding sites of the ligand, and can be applied to determine an apparent dissociation constant (K_D_). Using this method, we confirmed that guanidine causes a concentration-dependent structural modulation of the *95 Lla* construct (Figure 2B). By quantifying the extent of spontaneous cleavage at nucleotide position G 62 over increasing concentrations of guanidine hydrochloride, a mean apparent K_D_ value of 210 μM (+/− 20 μM) was determined (Figure 2C). Additionally, we tested the *GGAM-1* motif RNA found in *Raoultibacter timonensis*. Specifically, we used the 92-nucleotide-long RNA (*92 Rti*) from the 5′-UTR of the *emrE* (i.e. *sugE*) gene of *R. timonensis* (Supplementary Figure S1A). This construct also showed a cleavage pattern that matches the predicted secondary consensus model and a modulated pattern caused by increasing guanidine hydrochloride concentration with a mean apparent K_D_ of 190 μM (+/− 30 μM) (Supplementary Figure S1B, C).

**Figure 3.**
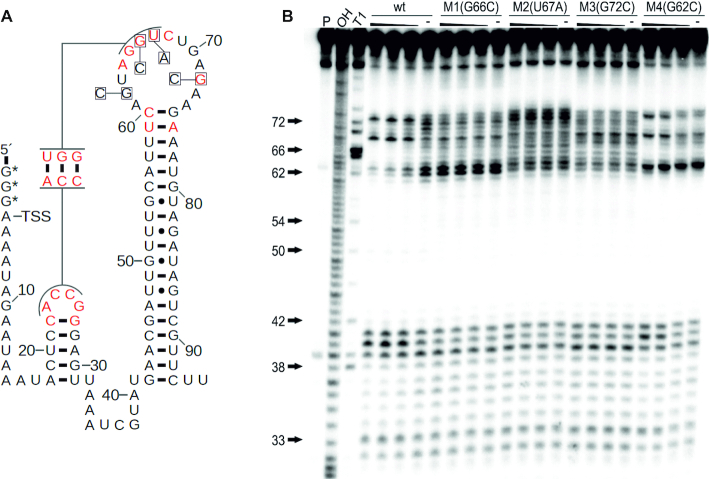
Single-nucleotide mutations of conserved nucleotides compromise guanidine binding. (**A**) Sequence and secondary structure of *95 Lla* RNA with the location of mutations in constructs M1(G66C), M2(U67A), M3(G72C) and M4(G62C). Highly conserved positions (97% nucleotide identity) are shown in red. (**B**) PAGE analysis of an in-line probing reaction of 5′ ^32^P-labeled *95 Lla* wt RNA and mutants M1(G66C), M2(U67A), M3(G72C) and M4(G62C) without (−) or with guanidine hydrochloride with concentrations of 10 mM, 1 mM and 100 μM. P, OH and T1 represent 5′ ^32^P-labeled RNA undergoing no reaction, digest under alkaline conditions, or digest with RNase T1, respectively.

For other riboswitch classes it has been shown that especially highly conserved nucleotides in the aptamer domain are often found to be directly involved in ligand binding. Hence, mutation of one of these nucleotides leads to a decreased ligand binding affinity or a complete loss of the binding function of the aptamer. To validate that the *GGAM-1* motif RNA selectively binds guanidine and to identify nucleotides that are essential for ligand binding, different mutant constructs of the *95 Lla* RNA were tested in in-line probing reactions (Figure 3A). The mutant constructs M1, M2 and M3, each carrying a single nucleotide change at a highly conserved position (97% nucleotide identity) in the second loop, completely eliminated guanidine-dependent modulation (Figure 3B). The construct M4 carries a mutation at a less conserved nucleotide position (90% nucleotide identity) and shows a greatly diminished structural modulation. However, the folding of this construct differs from the wildtype (wt) motif. These results demonstrate that binding of guanidine is dependent on the presence of the highly conserved nucleotides in the loop regions that likely form a selective binding pocket.

### Guanidine-dependent transcription termination control

**Figure 4.**
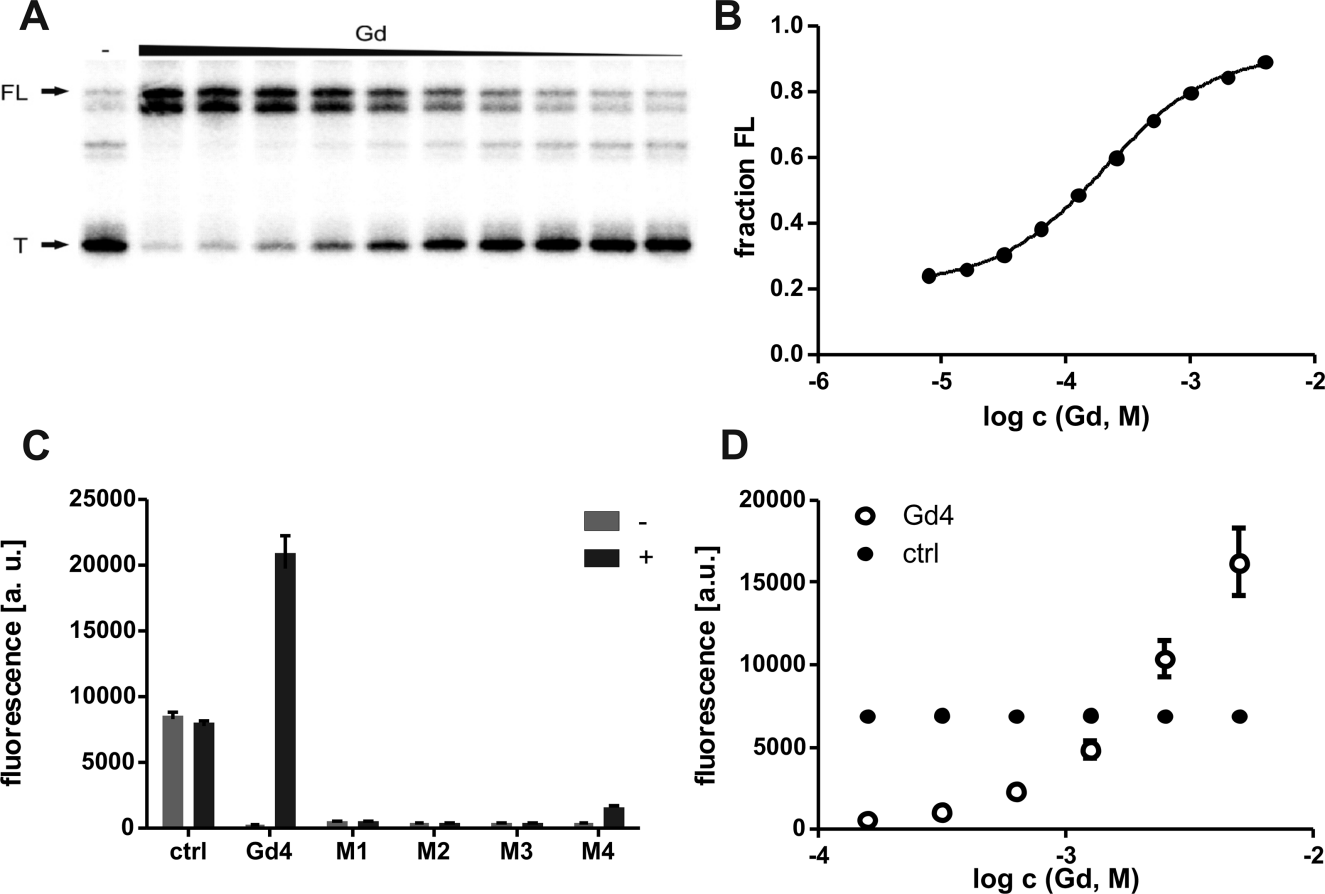
Guanidine controls gene expression via transcription termination. (**A**) PAGE analysis of a transcription termination assay of *147 Lla* RNA without (−) or with guanidine hydrochloride ranging from 7.8 μM - 4 mM. FL and T denote full length product at 147 nucleotides and termination product at 93 nucleotides, respectively. (**B**) Plot of the fraction of full length *147 Lla* product relative to the total number of transcripts (FL plus T) as a function of the guanidine hydrochloride concentration. A mean EC50 of 260 μM (±30 μM) was determined applying a sigmoidal dose-response curve fit in three independent experiments (Supplementary Figure S5A, B). (**C**) Bars show the eGFP expression in *S. aureus* without (−) and with (+) 5 mM guanidine hydrochloride, normalized to the OD_600_. Error bars represent the standard deviation from three independent experiments. A construct lacking the *GGAM-1* motif insertion eGFP (ctrl) served as a control. M1, M2, M3 and M4 represent constructs carrying single nucleotide mutations at positions hypothesized to be important for the formation of a selective binding pocket. Location of these single-nucleotide mutations are shown in Figure 3A. (**D**) Plot of dose-dependent eGFP expression in *S. aureus*. Strains were grown in media containing guanidine hydrochloride in the range 156 μM–5 mM. Fluorescence was measured and normalized to OD_600_. Error bars represent the standard deviation from three independent experiments. A construct with constitutively active eGFP (ctrl) served as control.

Almost all examples of the *GGAM-1* motif RNA are found to be associated with a Rho-independent transcription terminator. Thus, we hypothesized that the guanidine-dependent modulation observed in the binding assays would result in riboswitch-mediated control of transcription termination. To test this assumption, we monitored the transcription of a DNA template for a 147-nucleotide-long RNA construct (*147 Lla*) from *L. lactis*. This RNA construct carries the *GGAM-1* motif RNA and the following sequence context, including a Rho-independent terminator stem followed by 7 U residues, the start codon and 31 nucleotides of the *sugE* open reading frame. Using an in vitro transcription assay, the DNA template was transcribed with *E. coli* RNA polymerase in the presence or absence of guanidine hydrochloride. In accordance with our hypothesis, the yield of detected full-length transcription product increases in a concentration-dependent manner, whereas the termination product decreases in response to guanidine hydrochloride (Figure 4A), with a half maximal effective concentration EC_50_ of 260 μM (±30 μM) (Figure 4B). These data are consistent with the proposed riboswitch mechanism, in which binding of guanidine stabilizes a structure that prevents transcription termination, whereas the structure of the non-bound RNA enables formation of the intrinsic terminator and thus promotes transcription termination.

### Guanidine-dependent gene expression control

The ability of guanidine to regulate gene expression of the downstream gene *in vivo* was assessed by transforming *S. aureus* with a reporter plasmid. This plasmid carries the *GGAM-1* motif of *L. lactis* in the 5′-UTR of an *eGFP* reporter gene. Assuming that guanidine modulates the *GGAM-1* RNA motif to control transcription termination, *eGFP* expression should be increased due to guanidine addition. The reporter strain was grown in Brain Heart Infusion medium in the presence or absence of guanidine hydrochloride. To monitor the expression of the *eGFP* gene, the eGFP fluorescence intensity was measured and normalized by the optical density (OD_600_). Addition of 5 mM guanidine hydrochloride resulted in an 80-fold increase in eGFP expression (Figure 4C). Varying the amount of added guanidine showed a concentration-dependent change of gene expression (Figure 4D). A control plasmid that lacks the *GGAM-1* motif showed no influence of guanidine on eGFP expression. To verify the high selectivity of guanidine binding *in vivo*, we used plasmids carrying single-nucleotide mutations at positions that have also been investigated in binding assays and found to be important. Consistent with the in-line probing results, where M1, M2 and M3 did not show modulation due to guanidine (Figure 3B), we did not observe a change in eGFP expression for this mutants in the presence of guanidine (Figure 4C). Mutation of these highly conserved nucleotides lead to a complete loss of switching activity and they are essential for the functionality of the *GGAM-1* motif. The M4 mutation does not completely eliminate binding to guanidine, but does reduce affinity (Figure 3B). *In vivo*, the M4 mutation causes a 20-fold lower expression of eGFP compared to the wt sequence (‘Gd4’ in Figure 4C, D).

**Figure 5.**
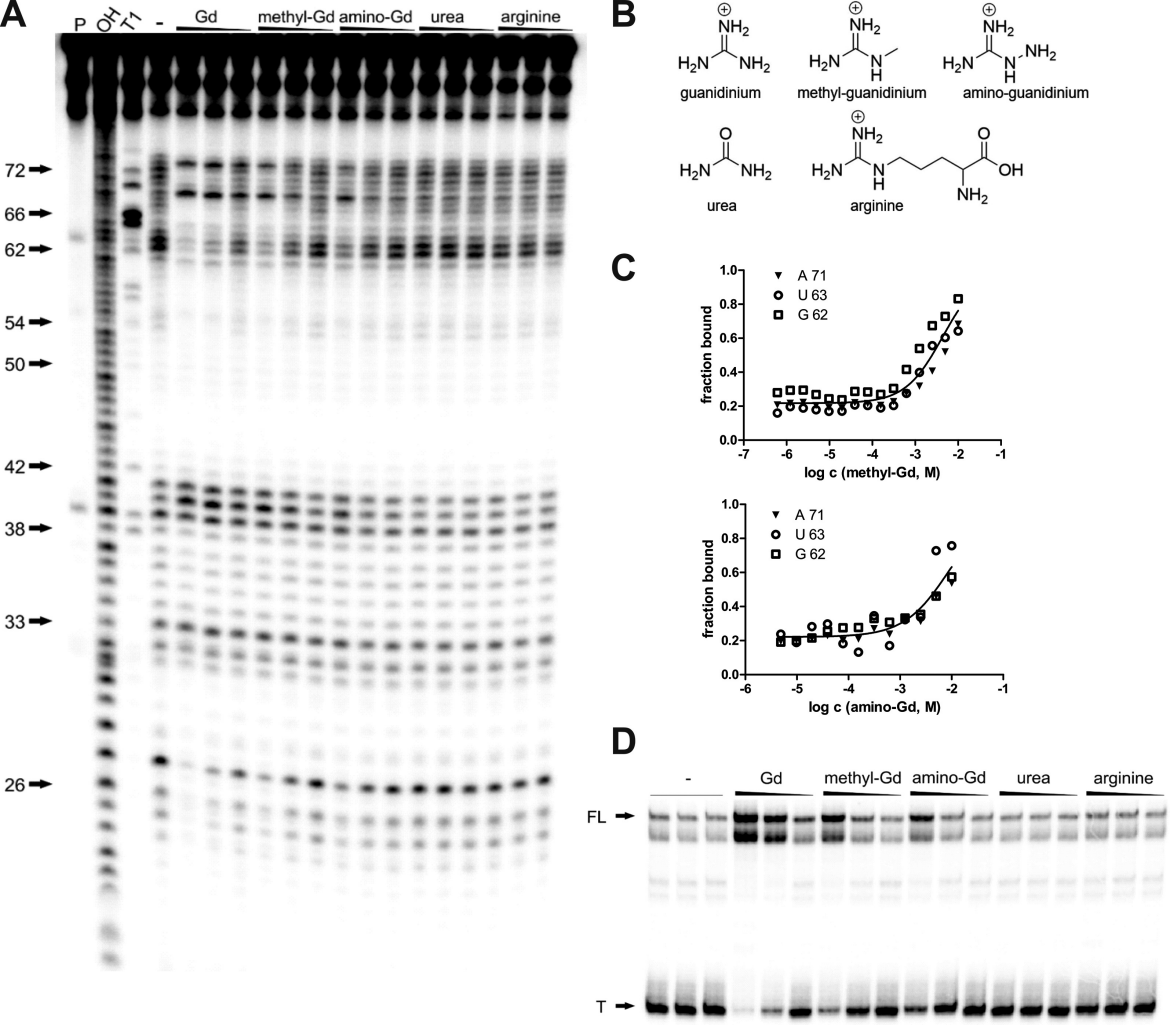
Binding and transcription control by guanidine derivates. (**A**) PAGE analysis of an in-line probing reaction of 5′ ^32^P-labeled *95 Lla* RNA without (−) or with guanidine hydrochloride, methyl-guanidine hydrochloride, amino-guanidine hydrochloride, urea or arginine with concentrations of 10 mM, 1 mM and 100 μM. P, OH and T1 have the same meaning as in Figure 2. (**B**) chemical structures of guanidine, methyl-guanidine, amino-guanidine, urea and arginine. (**C**) Plot of the fraction of RNA bound to methyl-guanidine and amino-guanidine, respectively, as a function of the logarithm (base 10) of the molar concentration. Fraction of RNA bound was determined as in Figure 2. For methyl-guanidine and amino-guanidine, a mean apparent *K*_D_ of 4.1 mM (±0.6 mM) and 7.5 mM (±0.8 mM), respectively, was determined in three independent experiments (Supplementary Figure S4C–F). The corresponding PAGE analysis of in-line probing reactions with methyl-guanidine and amino-guanidine are shown in Supplementary Figure S2. (**D**) PAGE analysis of a transcription termination assay of *147 Lla* RNA without (−) or with guanidine, methyl-guanidine, amino-guanidine, urea or arginine with concentrations of 10 mM, 1 mM and 100 μM. FL and T denote full length product at 147 nucleotides and termination product at 93 nucleotides, respectively.

To examine the ligand-binding selectivity of the riboswitch, we tested guanidine analogues in the binding assay as well as in the transcription termination assay. In both assays, only methyl-guanidine and amino-guanidine, both of which carry only small substitutes to the guanidine moiety core, were observed to bind and regulate transcription of the *GGAM-1* motif RNA (Figure 5). In in-line probing reactions with methyl- and amino-guanidine, a similar structural modulation of the *95 Lla* RNA was observed compared to guanidine (Figure 5A). However, methyl- and amino-guanidine show an increased (poorer) apparent *K*_D_ of 4.1 mM (±0.5 mM) and 7.5 mM (±0.8 mM), respectively (Figure 5C, Supplementary Figure S2). In transcription termination assays, addition of both methyl-guanidine and amino-guanidine led to an increase in full-length product in a concentration-dependent manner (Supplementary Figure S3). With urea and arginine, no binding to the RNA in in-line probing reactions was observed (Figure 5A). Also, no regulation of transcription termination was observed (Figure 5D). It seems likely that the aptamer binding pocket sterically excludes larger compounds. On the other hand, urea is a relatively small molecule that carries an oxo group instead of the imine nitrogen atom of guanidine, thus replacing a hydrogen bond donator by a hydrogen bond acceptor. Additionally, urea is neutral, whereas guanidine is positively charged under physiological pH conditions. These differences might be the reason why urea is excluded from the binding pocket. Our data indicate that the RNA motif binds guanidine with high selectivity and that binding of compounds with larger substitutions or an oxo group such as in urea is strongly discriminated against. Additionally, it has already previously shown that guanidine does not bind riboswitch classes that are already known to bind other molecules (20), providing another reason to believe that the guanidine binding of the *GGAM-1* motif is specific to the properties of this RNA.

## DISCUSSION

Our results show that *GGAM-1* RNAs bind guanidine, discriminate it from other, similar compounds, and efficiently regulate genes *in vivo*. These data fit with our bioinformatic observations that *GGAM-1* RNAs have typical properties of riboswitches, and appear to regulate guanidine-related genes. There are no meaningful similarities between *GGAM-1* RNAs and the previously established guanidine riboswitch classes, although an atomic-resolution structure could enable a more detailed comparison. We therefore propose the name guanidine-IV riboswitches for *GGAM-1* RNAs.

We were able to verify that the *GGAM-1* RNA motif regulates on the level of transcription termination (Figure 4A,B) and demonstrated that the guanidine binding is dependent on the presence of highly conserved nucleotides in the loop region. These highly conserved nucleotides likely form a selective binding pocket and mutation of a single one leads to a loss of *in vitro* modulation (Figure 3) and *in vivo* switching activity (Figure 4C) in response to guanidine. The *in vitro* and *in vivo* results together demonstrate that guanidine hydrochloride induces expression of the downstream gene via transcription termination control, indicating that the *GGAM-1* RNA motif functions as a genetic ‘ON’-switch.

Multiple riboswitches that use Rho-independent terminators to function as ON switches have been previously identified (19,45,46). A typical feature of such riboswitches is that the ligand-binding aptamer involves nucleotides that otherwise would form the 5′ side of the terminator stem. Thus, ligand binding inhibits the terminator stem, increasing gene expression. However, the proposed guanidine-IV binding structure includes the terminator stem, and ligand binding might even be expected to stabilize this stem. Since the presented *in vitro* transcription and *in vivo* reporter expression experiments establish these riboswitches as ON switches, they seem to use a new regulatory mechanism based on transcription termination. This could potentially work by steric exclusion of the formation of the full terminator stem by ligand-induced formation of a rigid structure. Guanidine binding would stabilize, for example, an extended conformation of two kissing hairpins, possibly similar to the structure of the guanidine-II riboswitch (41,42). This rigid structure could stretch the linking region between the two stems to the point that the outer base pairs of the terminator stem do not form, hence resulting in antitermination. Additional work will be needed to determine the specific regulatory mechanism that guanidine-IV riboswitches use.

The newly discovered guanidine-IV riboswitch shows similar ligand-binding characteristics to previously described classes. Some examples of the guanidine-I, -II, and III riboswitches have been reported to bind to guanidine with *K*_D_’s ranging from 25 to 300 μM (20–22), whereas the sequences tested in this work bind with dissociation constants of ∼150–250 μM. Regarding the selectivity of the interaction, the new guanidine-IV riboswitch more closely resembles the classes II and III, since these also bind to guanidine derivatives with small substitutions such as amino- and methyl-guanidine. The strong discrimination against urea and arginine is shared with all three known classes of guanidine riboswitches (20–22).

Guanidine-IV riboswitches add to an expanding set of molecule that are sensed by structurally unrelated riboswitch classes. Apart from guanidine, four riboswitch classes are currently known that bind SAM, three for preQ_1_ and two for cyclic di-GMP (47). These observations raise the question of what factors lead to multiple structural solutions to bind a given molecule. The answer could relate to the biochemistry of the ligand and RNA, cellular metabolism or other issues. Regardless of the cause, it seems reasonable to speculate that further structural classes will be found for guanidine, SAM, preQ_1_ and cyclic-di-GMP.

Some genes commonly associated with guanidine-IV riboswitches are never or very rarely observed to be regulated by guanidine-I, -II or -III riboswitches (Figure 1B, Supplementary Table S4). The guanidine-IV riboswitch thus implicates these thus far uncharacterized, new genes in guanidine biology. Two of these gene classes encode structurally distinct transporters, belonging to the PnuC and MATE families. Functional characterization of the guanidine-I riboswitch subsequently led to the result that riboswitch-associated *sugE* genes encode guanidine exporters (20,23). Thus, it seems reasonable to speculate that the MATE- and PnuC-class genes associated with guanidine-IV riboswitches also encode guanidine exporters of a new family.

Our goal in analyzing *sugE* genes was to find additional guanidine riboswitches, and this work led to the discovery of the guanidine-IV riboswitch class. However, we did not observe guanidine binding to examples of the other *GGAM* motifs. SugE proteins are diverse and form different clusters based on sequence analysis (23), and these clusters might correspond to different substrate specificities. Some have been validated as multi-drug transporters with a rather broad substrate specificity while others were recently confirmed to be specific for guanidine export (23). The *GGAM* motifs other than *GGAM-1* might associate with genes encoding multi-drug transporters, or these genes may encode SugE proteins with yet another substrate specificity. Most of these motifs only associate with *sugE* genes. The exception, *GGAM-5*, appears to regulate genes lacking a precisely predicted biochemical function. Thus, if this motif functions as a riboswitch, it might be difficult to determine the relevant ligand. It is also, however, possible that technical issues have caused false negative results in our *GGAM* experiments, as has hampered the validation of other riboswitches (2), or that the other *GGAM* motifs do not function as metabolite-binding riboswitches.

The discovery of a new class of guanidine riboswitches supports our bioinformatics approach as a viable strategy to discover novel *cis*-regulatory RNAs. In this work, we applied this strategy in a highly targeted manner, using *sugE* genes. We are currently applying this approach on a more comprehensive scale, in order to find *cis*-regulatory RNAs involved in other biological processes.

## DATA AVAILABILITY

All relevant data are available in the manuscript and supplementary materials. Alignments from the Weinberg group from papers accepted for publication are also available in the ZWD repository (https://bitbucket.org/zashaw/zashaweinbergdata/src/master).

## Supplementary Material

gkaa1102_Supplemental_Files
