# Supplementary material for: Discovery and characterization of a fourth class of guanidine riboswitches
Source: Nucleic Acids Res. 2020 Nov 25;48(22):12889–99. doi: 10.1093/nar/gkaa1102 (PMC7736828; doi:10.1093/nar/gkaa1102)
Supplement: gkaa1102_Supplemental_Files [file gkaa1102_supplemental_files.zip › G4-supplementary-text-figures-tables-revised-1018.pdf]

Supplementary Text, Figures and Tables:
Discovery and Characterization of a Fourth Class of Guanidine Riboswitches

Felina Lenkeit[1], Iris Eckert[2], Jörg S. Hartig[1*], and Zasha Weinberg[2*]

Supplementary Figures

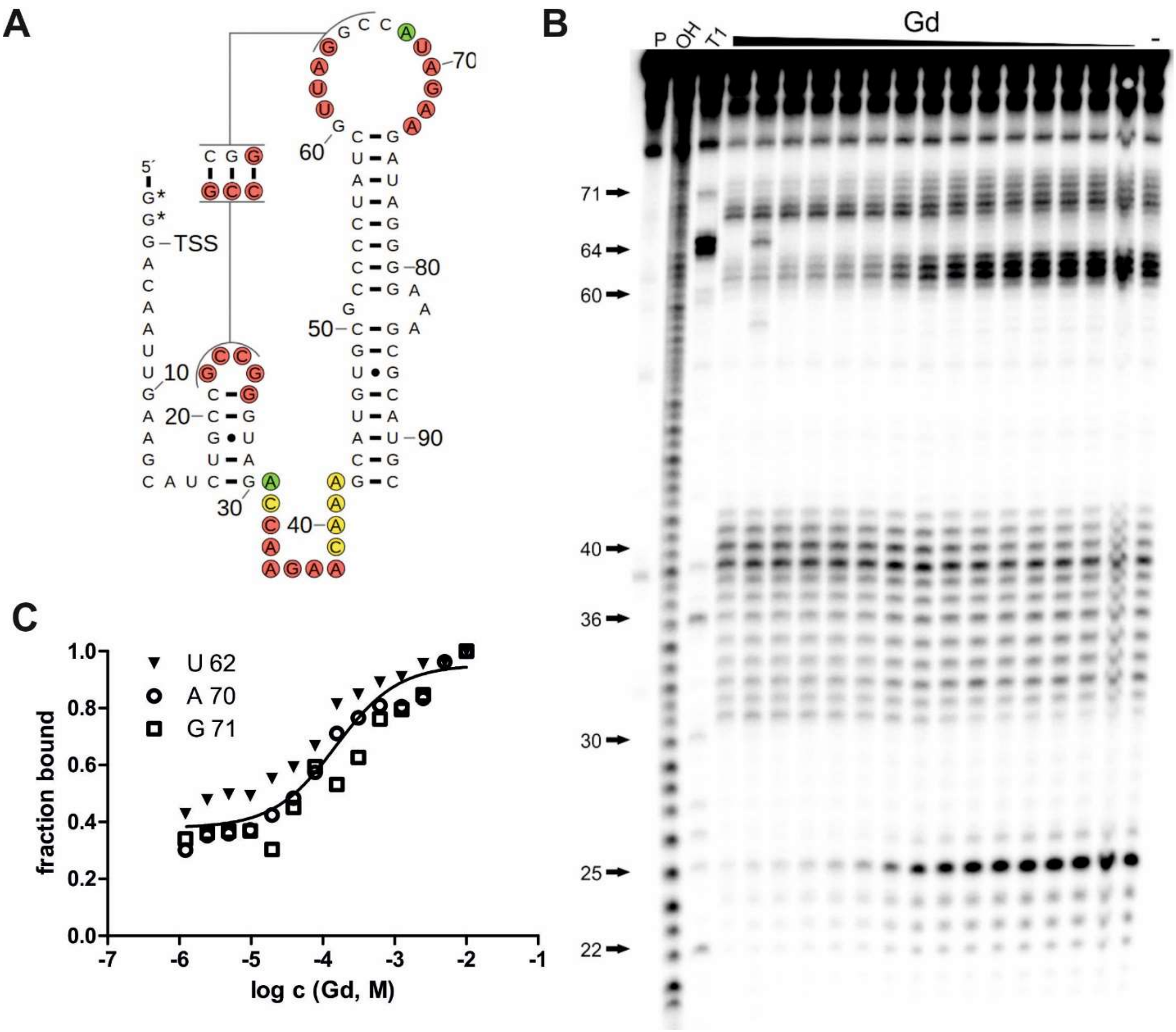

Supplemental Figure S1. Guanidine binding by *GGAM-1* motif from *Raoultibacter timonensis*. (*A*) Sequence and secondary structure of *92 Rti* RNA construct from the 5´-UTR of the *emrE* gene of *Raoultibacter timonensis*. The 5´ terminus of the construct includes two additional guanosine nucleotides to improve *in vitro* transcription efficiency. The annotations are the same as in Figure 2A. (*B*) PAGE analysis of an in-line probing reaction of 5´ ^{32}P-labeled *92 Rti* RNA without (-) or with guanidine hydrohydrochloride in a range of 0.61 µM – 10 mM. P, OH and T1 represent 5´ ^{32}P-labeled RNA undergoing no reaction, digest with RNase T1, or digest under alkaline conditions, respectively. (*C*) Plot of the fraction of RNA bound to ligand as a function of the logarithm (base 10) of the molar guanidine hydrochloride concentration. Fraction of RNA bound was determined based on quantification of band intensity changes at U 62, A 70 and G 72, normalized by the intensity of the constant band A 40. A trendline was generated using a sigmoidal dose-response curve fit (maximum value equal to 1) to determine an apparent K_D value. A mean apparent

K_D of 190 µM (standard deviation: 30 µM) was determined in three independent experiments (Supplementary Figure S4 G,H).

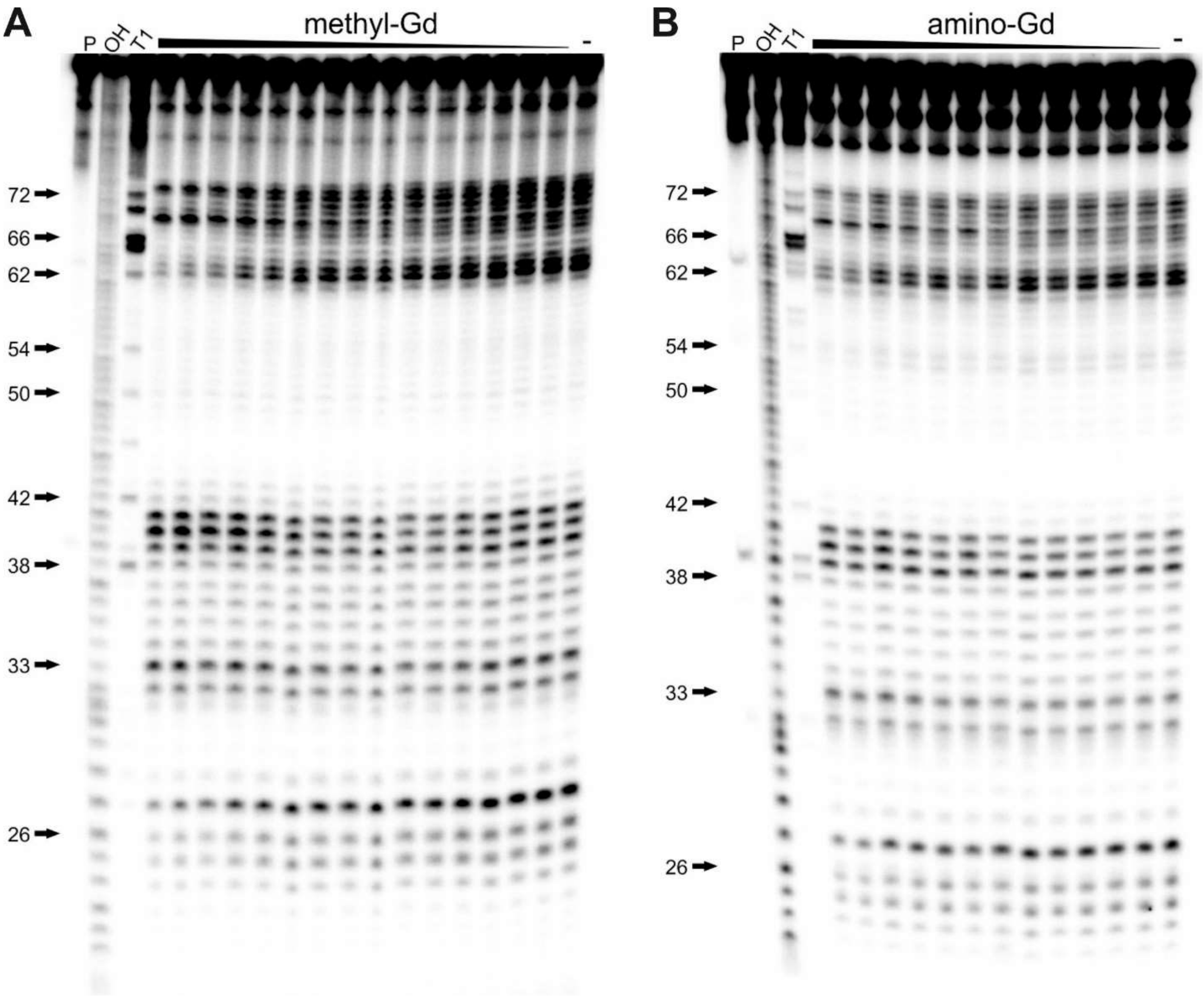

Supplemental Figure S2. Methyl-guanidine and amino-guanidine binding by the *95 Lla* motif from *L. lactis.* (*A*) PAGE analysis of an in line probing reaction of 5´ ^{32}P-labeled *95 Lla* RNA without (-) or with methyl-guanidine hydrohydrochloride in a range of 0.61 µM – 10 mM. P, OH and T1 represent 5´ ^{32}P-labeled RNA undergoing no reaction, digest with RNase T1, or digest under alkaline conditions, respectively. The plot of the fraction of RNA bound to ligand-derivate as a function of the logarithm (base 10) of the molar concentration is shown in figure 5C. (*B*) PAGE analysis of an in line probing reaction of 5´ ^{32}P-labeled *95 Lla* RNA without (-) or with amino-guanidine hydrohydrochloride in a range of 4.88 µM – 10 mM. P, OH and T1 represent 5´ ^{32}P-labeled RNA undergoing no reaction, digest with RNase T1, or digest under alkaline conditions, respectively. The plot of the fraction of RNA bound to ligand-derivate as a function of the logarithm (base 10) of the molar concentration is shown in Figure 5C.

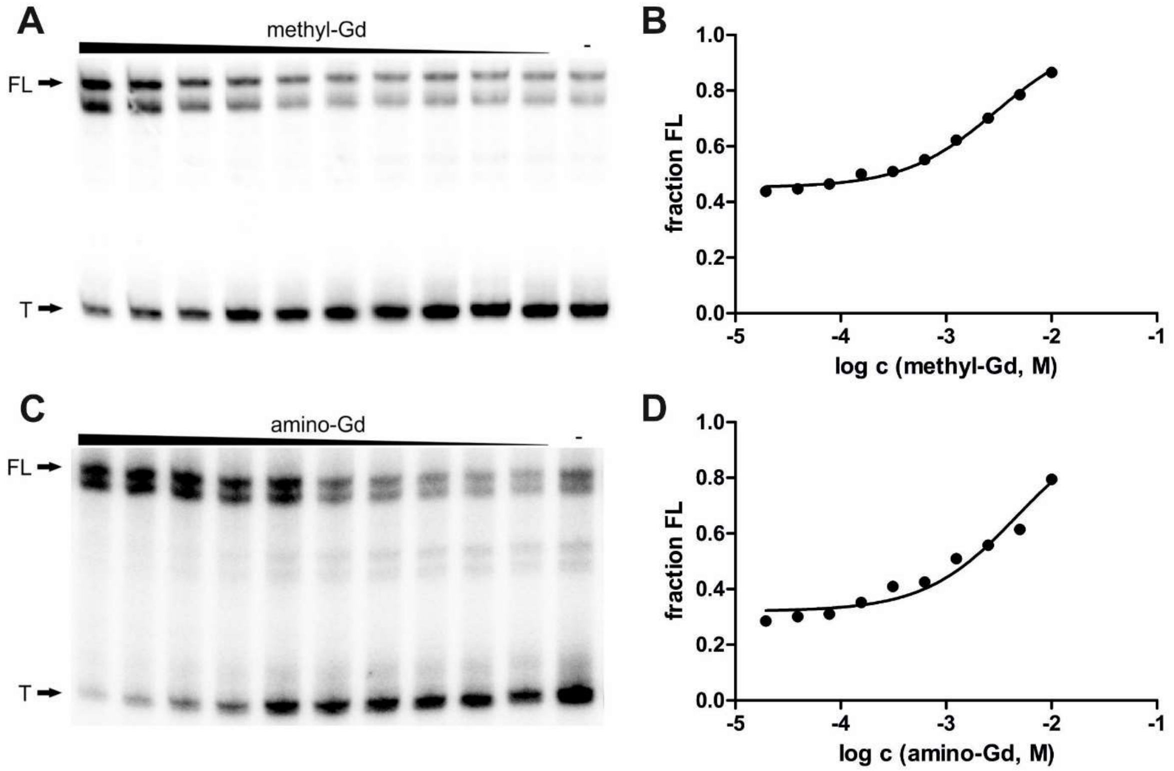

Supplemental Figure S3. Guanidine derivates prevent transcription termination less effectively than guanidine. (*A*) PAGE analysis of a transcription termination assay of *147 Lla* RNA without (-) or with methyl-guanidine hydrohydrochloride ranging from 15.6 µM - 8 mM. FL and T denote full length product at 147 nt and termination product at 93 nt, respectively. (*B*) Plot of the fraction of full length *147 Lla* product contributing to the total number of transcripts (FL plus T) as a function of the methyl-guanidine hydrochloride concentration. A mean EC_{50} of 2.7 (standard deviation: 0.7 mM) was determined applying a sigmoidal dose-response curve fit in three independent experiments (Supplementary Figure S5 C,D). (*C*) PAGE analysis of a transcription termination assay of *147 Lla* RNA without (-) or with amino-guanidine hydrohydrochloride ranging from 15.6 µM - 8 mM. FL and T denote full length product at 147 nt and termination product at 93 nt, respectively. (*D*) Plot of the fraction of full length *147 Lla* product contributing to the total number of transcripts (FL plus T) as a function of the amino-guanidine hydrochloride concentration. A mean EC_{50} of 3.8 mM (standard deviation: 0.5 mM) was determined applying a sigmoidal dose-response curve fit in three independent experiments (Supplementary Figure S5 E,F).

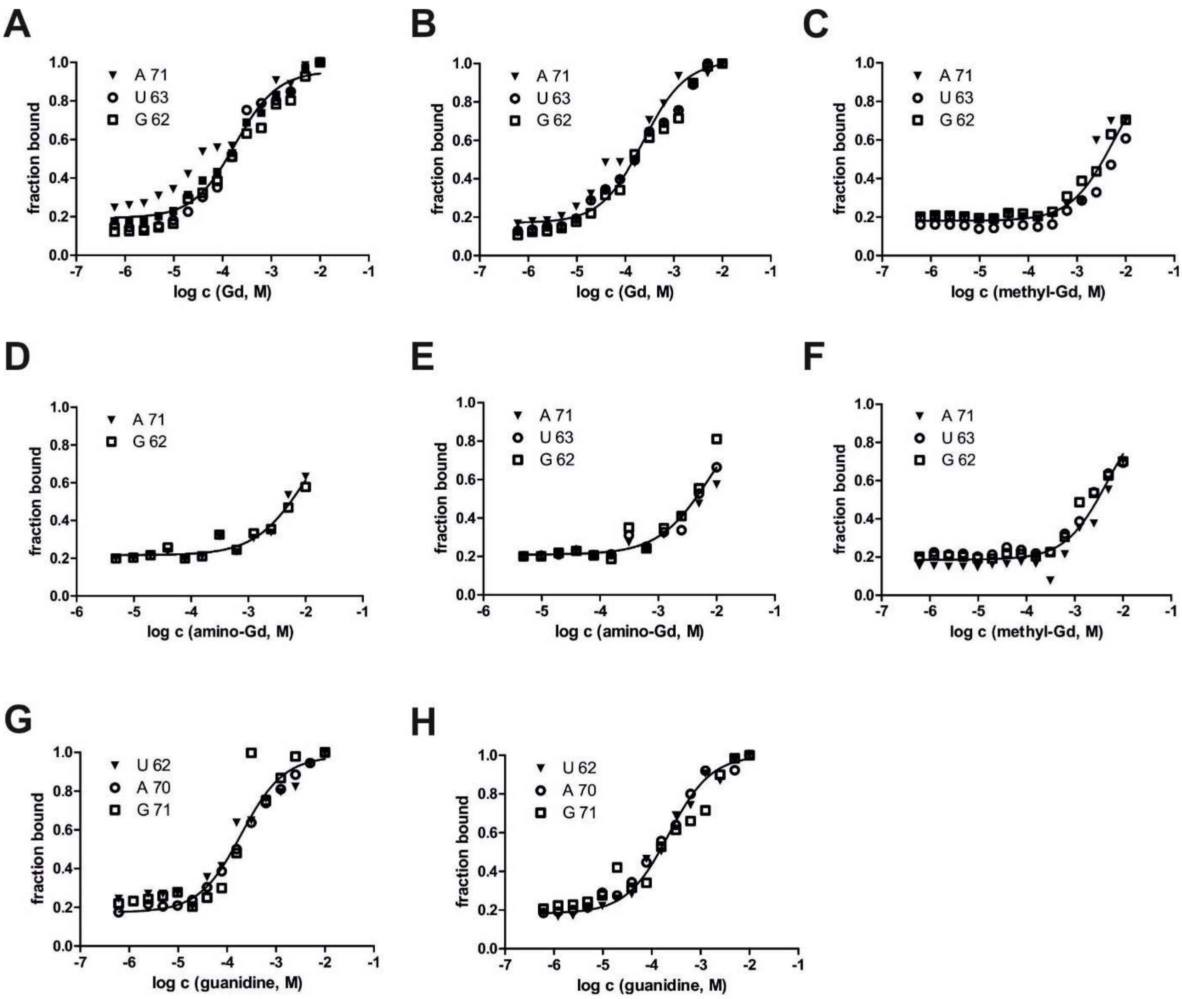

Supplementary Figure S4: Independent experiments for guanidine, methyl-guanidine and amino-guanidine binding by the *GGAM-1* motif. Data shown in this figure reflects experiments that are independent from each other and from figures in the main text.*(A), (B)*: Plot of the fraction of *95 Lla* RNA bound to guanidine as a function of the logarithm (base 10) of the molar guanidine hydrochloride concentration. Fraction of RNA bound was determined as described in Figure 2. *(C), (D)*: Plot of the fraction of *95 Lla* RNA bound to methyl-guanidine as a function of the logarithm (base 10) of the molar guanidine hydrochloride concentration. Fraction of RNA bound was determined as described in Figure 2. *(E), (F)*: Plot of the fraction of *95 Lla* RNA bound to amino-guanidine as a function of the logarithm (base 10) of the molar guanidine hydrochloride concentration. Fraction of RNA bound was determined as described in Figure 2. *(G), (H)*: Plot of the fraction of *92 Rti* RNA bound to guanidine as a function of the logarithm (base 10) of the molar guanidine hydrochloride concentration. Fraction of RNA bound was determined as described in Supplementary Figure S1.

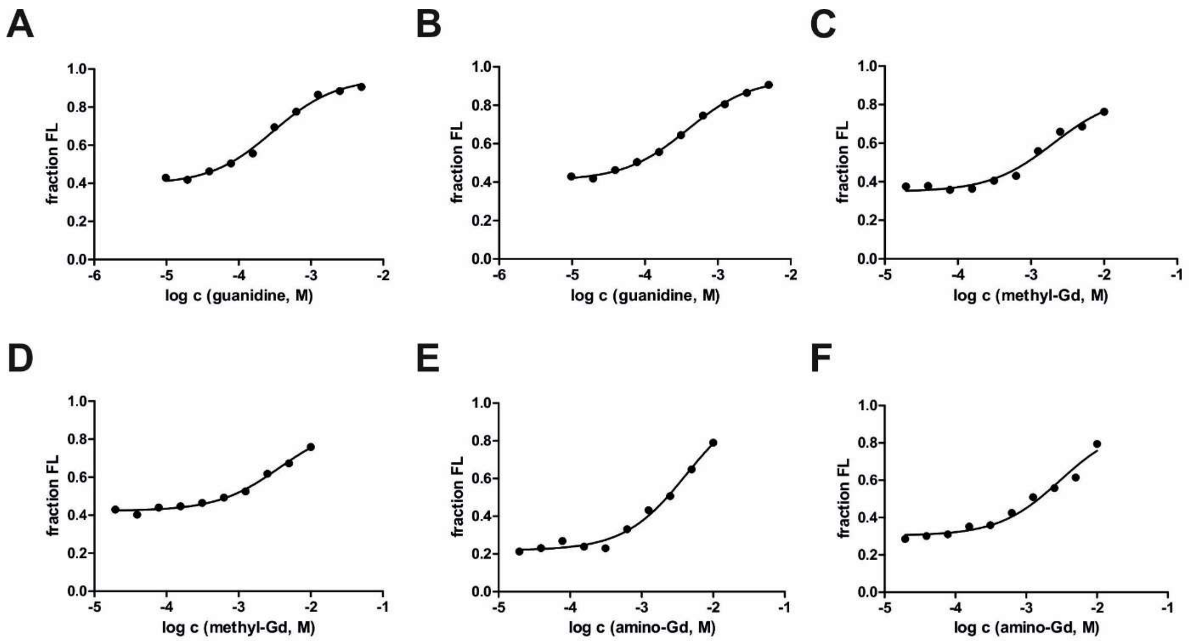

Supplementary Figure S5: Transcription termination control by guanidine and guanidine derivates, independent experiments. *(A), (B)*: Plot of the fraction of full length *147 Lla* product relative to the total number of transcripts as a function of the guanidine hydrochloride concentration. *(C), (D)*: Plot of the fraction of full length *147 Lla* product relative to the total number of transcripts as a function of the methyl-guanidine hydrochloride concentration. *(E), (F)*: Plot of the fraction of full length *147 Lla* product relative to the total number of transcripts as a function of the amino-guanidine hydrochloride concentration.

Supplementary Tables

Supplemental Table S1. Sequence of oligonucleotides used for the analysis of the *GGAM-1* motif. T7 RNA Polymerase Promotor is shown in green, T5 RNA Polymerase Promotor in blue. Lowercase letters identify guanosine nucleotides, added to enhance transcription. Nucleotides that were mutated relative to the wt sequence are shaded yellow, start codon is shaded grey.

Construct name	Sequence (5´-3´)
95 Lla	TAATACGACTCACTATAgggAAAATAGAATAAATACTCCACCGGGAGTTAAATCGTATGAACGATTGTTTGCATTTCAGTAGGTCTGAGAAGAAATGTAGATAGTCGTTCTT
95 Lla M1 (G66C)	TAATACGACTCACTATAgggAAAATAGAATAAATACTCCACCGGGAGTTAAATCGTATGAACGATTGTTTGCATTTCAGTAGCTCTGAGAAGAAATGTAGATAGTCGTTCTT
95 Lla M2 (U67A)	TAATACGACTCACTATAgggAAAATAGAATAAATACTCCACCGGGAGTTAAATCGTATGAACGATTGTTTGCATTTCAGTAGGACTGAGAAGAAATGTAGATAGTCGTTCTT
95 Lla M3 (G72C)	TAATACGACTCACTATAgggAAAATAGAATAAATACTCCACCGGGAGTTAAATCGTATGAACGATTGTTTGCATTTCAGTAGGTCTGACAAGAAATGTAGATAGTCGTTCTT
95 Lla M4 (G62C)	TAATACGACTCACTATAgggAAAATAGAATAAATACTCCACCGGGAGTTAAATCGTATGAACGATTGTTTGCATTTCACTAGGTCTGAGAAGAAATGTAGATAGTCGTTCTT
147 Lla	TCATAAAAAATTTATTTGCTTTGTGAGCGGATAACAATTATAATAAAAATAGAATAAATACTCCACCGGGAGTTAAATCGTATGAACGATTGTTTGCATTTCAGTAGGTCTGAGAAGAAATGTAGATAGTCGTTCTTTTTTTTAGCTGAGGAGGCGAAAATGACTTGGCTATATCTACTAATAGCAGGAATTT
92 Rti	TAATACGACTCACTATAggGACAATTGAAGCATCTGCCGCCGGGTAGACCAAGAACAAAGCATGTGCGCCCCTATCGTTAGGCCATAGAAGATAGGGGAAAGCGCATGC

Supplemental Table S2. Sequence of oligonucleotides used for the analysis of the *GGAM-2* to *6* motifs. Sequences were taken from *Cellulomonas fimi*, *Burkholderia ubonensis*, *Methyloceanibacter superfactus*, *Novosphingobium sp.*, *Selenomonas ruminantium*, respectively. T7 RNA Polymerase Promotor is shown in green. Lowercase letters identify guanosine nucleotides, added to enhance transcription.

Construct name	Sequence (5´-3´)
GGAM-2 (Cfi 63)	TAATACGACTCACTATAGGAATGGTACGGTCGTACCAGAACCGTGCGCG GGACGGCGGACCTGACACCCGGCGCGCAGCG
GGAM-2 (Cfi 80)	TAATACGACTCACTATAgGTCGCAGGAATGGTACGGTCGTACCAGAACC GTGCGCGGGACGGCGGACCTGACACCCGGCGCGCAGCGGTCGCCCA CC
GGAM-3 (Bub 73)	TAATACGACTCACTATAggGAUCCAUCCGGGGUACCGCUCCCGCAAGU UGAUCGCGCUCGGUACAAGCGCGAAAUCAGGAGCGGUACGCC
GGAM-3 (Bub 59)	TAATACGACTCACTATAggGAUCCAUCCGGGGUACCGCUCCCGCAAGU UGAUCGCGCUCGGUACAAGCGCGAAAUC
GGAM-4 (Msu 80)	TAATACGACTCACTATAgggAAACTAAGGTCCGCCCGCTAGGCCGGCCGC GCCTCGACGCGCCGGCTCTCGTCAATCGAACACGCGCGGTACCCATG
GGAM-4 (Msu 98)	TAATACGACTCACTATAgggCGACAGCCTCTCCTCGCGAAACTAAGGTCC GCCCGCTAGGCCGGCCGCGCCTCGACGCGCCGGCTCTCGTCAATCGA ACACGCGCGGTACCCATG
GGAM-5 (Nov 77)	TAATACGACTCACTATAggGTGGATGCGCCGAGAATAGAGTAGCAGGGTG GTGTCTCCTGCGCCCGTAAGGGTTCTTCCCAGCCACGGCGTCAA
GGAM-6 (Sru 111)	TAATACGACTCACTATAgggTCAATACTCGTATCGGACGGCAAGCAATCGC CTGCTTGCCAGATAAGGTGTCCATAGACACTGGAACGGGTTATTCAGGC CAGATAAGATCGCATTCTTATCTGGCCT

Supplemental Table S3. Sequence of the 5´-UTR of the reporter gene *eGFP* used in *S. aureus*. *GGAM-1* motif sequence is shown in bold font. Shine-Dalgarno sequence is underlined, start codon is shaded grey. Nucleotides that were mutated relative to the wt sequence are shaded yellow.

construct name	sequence (5´-3´)
Gd4 L. lactis wt	ATAAAGCAAGCATATAATATTGCGTTTCATCTTTAGAAGCGAATTTCGCC<br>AATATTATAATTATCAAAAGAGAGGGGTGGCAAACGGTATTTGGCATTAT<br>**AAAATAGAATAAATACTCCACCGGGAGTTAAATCGTATGAACGATTGT**<br>**TTGCATTTCAGTAGGTCTGAGAAGAAATGTAGATAGTCGTTCTTTTTTT**<br>**AGCT**GAAGGAGAGTGAAACCCATG
Gd4 L. lactis M1 (G66C)	ATAAAGCAAGCATATAATATTGCGTTTCATCTTTAGAAGCGAATTTCGCC<br>AATATTATAATTATCAAAAGAGAGGGGTGGCAAACGGTATTTGGCATTAT<br>**AAAATAGAATAAATACTCCACCGGGAGTTAAATCGTATGAACGATTGT**<br>**TTGCATTTCAGTAGCTCTGAGAAGAAATGTAGATAGTCGTTCTTTTTTT**<br>**AGCT**GAAGGAGAGTGAAACCCATG
Gd4 L. lactis M2 (U67A)	ATAAAGCAAGCATATAATATTGCGTTTCATCTTTAGAAGCGAATTTCGCC<br>AATATTATAATTATCAAAAGAGAGGGGTGGCAAACGGTATTTGGCATTAT<br>**AAAATAGAATAAATACTCCACCGGGAGTTAAATCGTATGAACGATTGT**<br>**TTGCATTTCAGTAGGACTGAGAAGAAATGTAGATAGTCGTTCTTTTTTT**<br>**AGCT**GAAGGAGAGTGAAACCCATG
Gd4 L. lactis M3 (G72C)	ATAAAGCAAGCATATAATATTGCGTTTCATCTTTAGAAGCGAATTTCGCC<br>AATATTATAATTATCAAAAGAGAGGGGTGGCAAACGGTATTTGGCATTAT<br>**AAAATAGAATAAATACTCCACCGGGAGTTAAATCGTATGAACGATTGT**<br>**TTGCATTTCAGTAGGTCTGACAAGAAATGTAGATAGTCGTTCTTTTTTT**<br>**AGCT**GAAGGAGAGTGAAACCCATG
Gd4 L. lactis M4 (G62C)	ATAAAGCAAGCATATAATATTGCGTTTCATCTTTAGAAGCGAATTTCGCC<br>AATATTATAATTATCAAAAGAGAGGGGTGGCAAACGGTATTTGGCATTAT<br>**AAAATAGAATAAATACTCCACCGGGAGTTAAATCGTATGAACGATTGT**<br>**TTGCATTTCACTAGGTCTGAGAAGAAATGTAGATAGTCGTTCTTTTTTT**<br>**AGCT**GAAGGAGAGTGAAACCCATG

Supplementary Table S4. The six conserved protein domains most commonly encoded by genes regulated by *GGAM-1* RNAs (related to Figure 1B). “Name”: a name for the domain, as in Figure 1B. “Description”: a brief description of the domain’s biochemical function. “Frequency among riboswitches (%)”: the percentage of riboswitches/motifs that regulate a gene with the given domain as the first gene in an operon. “*GGAM-1*”: percentages for the *GGAM-1* motif. “Guan.-I”, “Guan.-II”, “Guan.-III”: percentages for the previously discovered guanidine-I, -II and -III riboswitch classes, respectively. The percentages for “Other” are shown only for the *GGAM-1* motif. Percentages for established riboswitches that are qualitatively similar to those of the *GGAM-1* motif are underlined. “Accessions”: accessions of entries in the Conserved Domain Database that we used to classify each of the six domains in the table. The row named “Other” relates to all other accessions as well as proteins that do not match any domain in the Conserved Domain Database. A comprehensive list of genes associated with all examples of all *GGAM* motifs appears in Supplementary File 1.

		Frequency among riboswitches (%)				
Name	Description	*GGAM-1*	Guan.-I	Guan.-II	Guan.-III	Accessions
SugE	SugE/EmrE/SMR/Gdx transporter family	31	<u>17.6</u>	<u>56.5</u>	<u>82</u>	COG2076, PRK11431
PnuC	PnuC-like transporter family	25.2	0.09	0.02	0	COG3201, pfam04973. TIGR01528
MATE	Multidrug And Toxic compound Extrusion (MATE) transporter family	22.6	0.1	0.04	0	cd13143, COG0534, , pfam14667, PRK09575, TIGR00797
NimA	Pyridoxamine 5′ phosphate oxidase superfamily	10.5	0.08	0	0	COG3467, pfam01243, pfam12900
B3/B4	Phenylalanine- and lysidine-tRNA synthetase domain superfamily	5	<u>6.2</u>	0.02	0	COG3382
GNAT	GCN5-related *N*-acetyltransferases family (GNAT)	2.5	0.04	0.02	0.03	pfam00583, pfam13508
Other		3.2				
