# Supplementary material for: Discovery and characterization of a fourth class of guanidine riboswitches
Source: Nucleic Acids Res. 2020 Nov 25;48(22):12889–99. doi: 10.1093/nar/gkaa1102 (PMC7736828; doi:10.1093/nar/gkaa1102)
Supplement: gkaa1102_Supplemental_Files [file gkaa1102_supplemental_files.zip › Supplementary-File-1.pdf]

Supplementary File 1: alignments and information on downstream genes and taxonomy
Discovery and characterization of a fourth class of guanidine riboswitches

Felina Lenkeit & Iris Eckert & Jörg S.Hartig & Zasha Weinberg

Note: This supplementary file contains two alignments for the GGAM-1 motif (which we later renamed the guanidine-IV riboswitch). One alignment is called GGAM-1-curated. This alignment was hand-curated, but contains a limited number of sequences. It is appropriate for studying the secondary structure of the RNA. The other alignment is called GGAM-1, and it includes all GGAM-1 RNAs we could find. This (comprehensive) GGAM-1 alignment is appropriate for analyzing the genomic context of the GGAM-1 RNA. However, because the alignment is not hand-curated, it includes many apparent mismatches in the Rho-independent terminator's stem. In our experience with the curated alignment, inserting one gap is usually sufficient to eliminate most base pair mismatches.

Furthermore, note that the presentation and explanatory text of this supplementary data on novel RNA motifs follows the pattern of a presentation of previously found conserved RNA motifs (Weinberg, et al., 2010).

Contents

1 GGAM-1 . . . **2**
1.1 Taxa . . . 2
1.2 Notes . . . 11
1.3 Gene contexts . . . 11
1.4 Conserved domains . . . 65
1.5 Multiple-sequence alignment . . . 71
1.6 Multiple-sequence alignment curated . . . 138

2 GGAM-2 . . . **149**
2.1 Taxa . . . 149
2.2 Gene contexts . . . 149
2.3 Conserved domains . . . 153
2.4 Multiple-sequence alignment . . . 154

3 GGAM-3 . . . **159**
3.1 Taxa . . . 159
3.2 Gene contexts . . . 160
3.3 Conserved domains . . . 165
3.4 Multiple-sequence alignment . . . 166

4 GGAM-4 **178**
4.1 Taxa . . . 178
4.2 Gene contexts . . . 178
4.3 Conserved domains . . . 179
4.4 Multiple-sequence alignment . . . 179

5 GGAM-5 **180**
5.1 Taxa . . . 180
5.2 Gene contexts . . . 182
5.3 Conserved domains . . . 188
5.4 Multiple-sequence alignment . . . 189

6 GGAM-6 **205**
6.1 Taxa . . . 205
6.2 Gene contexts . . . 205
6.3 Conserved domains . . . 206
6.4 Multiple-sequence alignment . . . 206

1 GGAM-1

1.1 Taxa

The taxonomy of each organism containing a putative GGAM-1-recreated RNA is listed, with abbreviations identifying each hit (e.g., "Eco-1-1" and "Eco-1-2" might hypothetically represent two distinct RNAs in *E. coli*). The abbreviations will be used to identify each individual GGAM-1-recreated RNA in Sections 1.3 and 1.6:.

abbrev. of hits	taxonomy of species
Rma-1-1	Bacteria Actinobacteria Coriobacteriia Eggerthellales Eggerthellaceae *Raoultibacter massiliensis*
Rti-1-1	Bacteria Actinobacteria Coriobacteriia Eggerthellales Eggerthellaceae *Raoultibacter timonensis*
bMS-1-1	Bacteria bacterium MS4
Bca-1-1	Bacteria Bacteroidetes Bacteroidia Bacteroidales Bacteroidaceae *Bacteroides capillosus* ATCC 29799
Lmo-1-1 to Lmo-1-2	Bacteria Firmicutes Bacilli Bacillales Listeriaceae *Listeria monocytogenes*
Cin-1-1 to Cin-1-2	Bacteria Firmicutes Bacilli Lactobacillales Carnobacteriaceae *Carnobacterium iners*
Ime-1-1	Bacteria Firmicutes Bacilli Lactobacillales Carnobacteriaceae *Isobaculum melis*
Lna-1-1	Bacteria Firmicutes Bacilli Lactobacillales Carnobacteriaceae *Lacticigenium naphtae* DSM 19658
Tfl-1-1 to Tfl-1-2	Bacteria Firmicutes Bacilli Lactobacillales Carnobacteriaceae *Trichococcus flocculiformis*
Til-1-1	Bacteria Firmicutes Bacilli Lactobacillales Carnobacteriaceae *Trichococcus ilyis*
Tsp-1-1	Bacteria Firmicutes Bacilli Lactobacillales Carnobacteriaceae *Trichococcus sp.* DSM 22150
Eca-1-1	Bacteria Firmicutes Bacilli Lactobacillales Enterococcaceae *Enterococcus caccae*
Eca-2-1	Bacteria Firmicutes Bacilli Lactobacillales Enterococcaceae *Enterococcus caccae* ATCC BAA-1240
Efa-1-1 to Efa-1-8	Bacteria Firmicutes Bacilli Lactobacillales Enterococcaceae *Enterococcus faecalis*
Efa-2-1	Bacteria Firmicutes Bacilli Lactobacillales Enterococcaceae *Enterococcus faecalis* 02-MB-BW-10
Efa-3-1	Bacteria Firmicutes Bacilli Lactobacillales Enterococcaceae *Enterococcus faecalis* 02-MB-P-10
Efa-4-1	Bacteria Firmicutes Bacilli Lactobacillales Enterococcaceae *Enterococcus faecalis* 06-MB-S-04
Efa-5-1	Bacteria Firmicutes Bacilli Lactobacillales Enterococcaceae *Enterococcus faecalis* 06-MB-S-10
Efa-6-1	Bacteria Firmicutes Bacilli Lactobacillales Enterococcaceae *Enterococcus faecalis* 10244
Efa-7-1	Bacteria Firmicutes Bacilli Lactobacillales Enterococcaceae *Enterococcus faecalis* 20-SD-BW-06
Efa-8-1	Bacteria Firmicutes Bacilli Lactobacillales Enterococcaceae *Enterococcus faecalis* 20-SD-BW-08
Efa-9-1	Bacteria Firmicutes Bacilli Lactobacillales Enterococcaceae *Enterococcus faecalis* 20.SD.W.06
Efa-10-1	Bacteria Firmicutes Bacilli Lactobacillales Enterococcaceae *Enterococcus faecalis* 599

Efa-11-1	Bacteria Firmicutes Bacilli Lactobacillales Enterococcaceae *Enterococcus faecalis* 918
Efa-12-1	Bacteria Firmicutes Bacilli Lactobacillales Enterococcaceae *Enterococcus faecalis* ARO1/DG
Efa-13-1	Bacteria Firmicutes Bacilli Lactobacillales Enterococcaceae *Enterococcus faecalis* ATCC 10100
Efa-14-1	Bacteria Firmicutes Bacilli Lactobacillales Enterococcaceae *Enterococcus faecalis* ATCC 27275
Efa-15-1	Bacteria Firmicutes Bacilli Lactobacillales Enterococcaceae *Enterococcus faecalis* ATCC 27959
Efa-16-1 to Efa-16-2	Bacteria Firmicutes Bacilli Lactobacillales Enterococcaceae *Enterococcus faecalis* ATCC 29200
Efa-17-1	Bacteria Firmicutes Bacilli Lactobacillales Enterococcaceae *Enterococcus faecalis* ATCC 29212
Efa-18-1	Bacteria Firmicutes Bacilli Lactobacillales Enterococcaceae *Enterococcus faecalis* ATCC 35038
Efa-19-1	Bacteria Firmicutes Bacilli Lactobacillales Enterococcaceae *Enterococcus faecalis* ATCC 6055
Efa-20-1	Bacteria Firmicutes Bacilli Lactobacillales Enterococcaceae *Enterococcus faecalis* B83616-1
Efa-21-1	Bacteria Firmicutes Bacilli Lactobacillales Enterococcaceae *Enterococcus faecalis* BM4539
Efa-22-1	Bacteria Firmicutes Bacilli Lactobacillales Enterococcaceae *Enterococcus faecalis* BM4654
Efa-23-1	Bacteria Firmicutes Bacilli Lactobacillales Enterococcaceae *Enterococcus faecalis* D811610-10
Efa-24-1	Bacteria Firmicutes Bacilli Lactobacillales Enterococcaceae *Enterococcus faecalis* DAPTO 512
Efa-25-1	Bacteria Firmicutes Bacilli Lactobacillales Enterococcaceae *Enterococcus faecalis* DAPTO 516
Efa-26-1	Bacteria Firmicutes Bacilli Lactobacillales Enterococcaceae *Enterococcus faecalis* E12
Efa-43-1	Bacteria Firmicutes Bacilli Lactobacillales Enterococcaceae *Enterococcus faecalis* Efa HS0914
Efa-44-1	Bacteria Firmicutes Bacilli Lactobacillales Enterococcaceae *Enterococcus faecalis* EnGen0058
Efa-45-1	Bacteria Firmicutes Bacilli Lactobacillales Enterococcaceae *Enterococcus faecalis* EnGen0059
Efa-46-1	Bacteria Firmicutes Bacilli Lactobacillales Enterococcaceae *Enterococcus faecalis* EnGen0060
Efa-47-1	Bacteria Firmicutes Bacilli Lactobacillales Enterococcaceae *Enterococcus faecalis* EnGen0061
Efa-48-1	Bacteria Firmicutes Bacilli Lactobacillales Enterococcaceae *Enterococcus faecalis* EnGen0062
Efa-49-1	Bacteria Firmicutes Bacilli Lactobacillales Enterococcaceae *Enterococcus faecalis* EnGen0063
Efa-50-1	Bacteria Firmicutes Bacilli Lactobacillales Enterococcaceae *Enterococcus faecalis* EnGen0067
Efa-51-1	Bacteria Firmicutes Bacilli Lactobacillales Enterococcaceae *Enterococcus faecalis* EnGen0068
Efa-52-1	Bacteria Firmicutes Bacilli Lactobacillales Enterococcaceae *Enterococcus faecalis* EnGen0069
Efa-53-1	Bacteria Firmicutes Bacilli Lactobacillales Enterococcaceae *Enterococcus faecalis* EnGen0070
Efa-54-1	Bacteria Firmicutes Bacilli Lactobacillales Enterococcaceae *Enterococcus faecalis* EnGen0071
Efa-55-1	Bacteria Firmicutes Bacilli Lactobacillales Enterococcaceae *Enterococcus faecalis* EnGen0072
Efa-56-1	Bacteria Firmicutes Bacilli Lactobacillales Enterococcaceae *Enterococcus faecalis* EnGen0073
Efa-57-1	Bacteria Firmicutes Bacilli Lactobacillales Enterococcaceae *Enterococcus faecalis* EnGen0074
Efa-58-1	Bacteria Firmicutes Bacilli Lactobacillales Enterococcaceae *Enterococcus faecalis* EnGen0075
Efa-59-1	Bacteria Firmicutes Bacilli Lactobacillales Enterococcaceae *Enterococcus faecalis* EnGen0076
Efa-60-1	Bacteria Firmicutes Bacilli Lactobacillales Enterococcaceae *Enterococcus faecalis* EnGen0078
Efa-61-1	Bacteria Firmicutes Bacilli Lactobacillales Enterococcaceae *Enterococcus faecalis* EnGen0079
Efa-62-1	Bacteria Firmicutes Bacilli Lactobacillales Enterococcaceae *Enterococcus faecalis* EnGen0080
Efa-63-1	Bacteria Firmicutes Bacilli Lactobacillales Enterococcaceae *Enterococcus faecalis* EnGen0081
Efa-64-1	Bacteria Firmicutes Bacilli Lactobacillales Enterococcaceae *Enterococcus faecalis* EnGen0082
Efa-65-1	Bacteria Firmicutes Bacilli Lactobacillales Enterococcaceae *Enterococcus faecalis* EnGen0083
Efa-66-1	Bacteria Firmicutes Bacilli Lactobacillales Enterococcaceae *Enterococcus faecalis* EnGen0084
Efa-67-1	Bacteria Firmicutes Bacilli Lactobacillales Enterococcaceae *Enterococcus faecalis* EnGen0085
Efa-68-1	Bacteria Firmicutes Bacilli Lactobacillales Enterococcaceae *Enterococcus faecalis* EnGen0086
Efa-69-1	Bacteria Firmicutes Bacilli Lactobacillales Enterococcaceae *Enterococcus faecalis* EnGen0087
Efa-70-1	Bacteria Firmicutes Bacilli Lactobacillales Enterococcaceae *Enterococcus faecalis* EnGen0088
Efa-71-1	Bacteria Firmicutes Bacilli Lactobacillales Enterococcaceae *Enterococcus faecalis* EnGen0089
Efa-72-1	Bacteria Firmicutes Bacilli Lactobacillales Enterococcaceae *Enterococcus faecalis* EnGen0090
Efa-73-1	Bacteria Firmicutes Bacilli Lactobacillales Enterococcaceae *Enterococcus faecalis* EnGen0091
Efa-74-1	Bacteria Firmicutes Bacilli Lactobacillales Enterococcaceae *Enterococcus faecalis* EnGen0092
Efa-75-1	Bacteria Firmicutes Bacilli Lactobacillales Enterococcaceae *Enterococcus faecalis* EnGen0093
Efa-76-1	Bacteria Firmicutes Bacilli Lactobacillales Enterococcaceae *Enterococcus faecalis* EnGen0094
Efa-77-1	Bacteria Firmicutes Bacilli Lactobacillales Enterococcaceae *Enterococcus faecalis* EnGen0095
Efa-78-1	Bacteria Firmicutes Bacilli Lactobacillales Enterococcaceae *Enterococcus faecalis* EnGen0096
Efa-79-1	Bacteria Firmicutes Bacilli Lactobacillales Enterococcaceae *Enterococcus faecalis* EnGen0097
Efa-80-1	Bacteria Firmicutes Bacilli Lactobacillales Enterococcaceae *Enterococcus faecalis* EnGen0098
Efa-81-1	Bacteria Firmicutes Bacilli Lactobacillales Enterococcaceae *Enterococcus faecalis* EnGen0099
Efa-82-1	Bacteria Firmicutes Bacilli Lactobacillales Enterococcaceae *Enterococcus faecalis* EnGen0100
Efa-83-1	Bacteria Firmicutes Bacilli Lactobacillales Enterococcaceae *Enterococcus faecalis* EnGen0101
Efa-84-1	Bacteria Firmicutes Bacilli Lactobacillales Enterococcaceae *Enterococcus faecalis* EnGen0102
Efa-85-1	Bacteria Firmicutes Bacilli Lactobacillales Enterococcaceae *Enterococcus faecalis* EnGen0103
Efa-86-1	Bacteria Firmicutes Bacilli Lactobacillales Enterococcaceae *Enterococcus faecalis* EnGen0104
Efa-87-1	Bacteria Firmicutes Bacilli Lactobacillales Enterococcaceae *Enterococcus faecalis* EnGen0105
Efa-88-1	Bacteria Firmicutes Bacilli Lactobacillales Enterococcaceae *Enterococcus faecalis* EnGen0106
Efa-89-1	Bacteria Firmicutes Bacilli Lactobacillales Enterococcaceae *Enterococcus faecalis* EnGen0107
Efa-90-1	Bacteria Firmicutes Bacilli Lactobacillales Enterococcaceae *Enterococcus faecalis* EnGen0108
Efa-91-1	Bacteria Firmicutes Bacilli Lactobacillales Enterococcaceae *Enterococcus faecalis* EnGen0109

Efa-92-1	Bacteria Firmicutes Bacilli Lactobacillales Enterococcaceae *Enterococcus faecalis* EnGen0110
Efa-93-1	Bacteria Firmicutes Bacilli Lactobacillales Enterococcaceae *Enterococcus faecalis* EnGen0111
Efa-94-1	Bacteria Firmicutes Bacilli Lactobacillales Enterococcaceae *Enterococcus faecalis* EnGen0112
Efa-95-1	Bacteria Firmicutes Bacilli Lactobacillales Enterococcaceae *Enterococcus faecalis* EnGen0113
Efa-96-1	Bacteria Firmicutes Bacilli Lactobacillales Enterococcaceae *Enterococcus faecalis* EnGen0114
Efa-97-1	Bacteria Firmicutes Bacilli Lactobacillales Enterococcaceae *Enterococcus faecalis* EnGen0115
Efa-98-1	Bacteria Firmicutes Bacilli Lactobacillales Enterococcaceae *Enterococcus faecalis* EnGen0116
Efa-99-1	Bacteria Firmicutes Bacilli Lactobacillales Enterococcaceae *Enterococcus faecalis* EnGen0117
Efa-100-1	Bacteria Firmicutes Bacilli Lactobacillales Enterococcaceae *Enterococcus faecalis* EnGen0118
Efa-101-1	Bacteria Firmicutes Bacilli Lactobacillales Enterococcaceae *Enterococcus faecalis* EnGen0119
Efa-102-1	Bacteria Firmicutes Bacilli Lactobacillales Enterococcaceae *Enterococcus faecalis* EnGen0120
Efa-103-1	Bacteria Firmicutes Bacilli Lactobacillales Enterococcaceae *Enterococcus faecalis* EnGen0194
Efa-104-1	Bacteria Firmicutes Bacilli Lactobacillales Enterococcaceae *Enterococcus faecalis* EnGen0195
Efa-105-1	Bacteria Firmicutes Bacilli Lactobacillales Enterococcaceae *Enterococcus faecalis* EnGen0196
Efa-106-1	Bacteria Firmicutes Bacilli Lactobacillales Enterococcaceae *Enterococcus faecalis* EnGen0197
Efa-107-1	Bacteria Firmicutes Bacilli Lactobacillales Enterococcaceae *Enterococcus faecalis* EnGen0198
Efa-108-1	Bacteria Firmicutes Bacilli Lactobacillales Enterococcaceae *Enterococcus faecalis* EnGen0199
Efa-109-1	Bacteria Firmicutes Bacilli Lactobacillales Enterococcaceae *Enterococcus faecalis* EnGen0200
Efa-110-1	Bacteria Firmicutes Bacilli Lactobacillales Enterococcaceae *Enterococcus faecalis* EnGen0201
Efa-111-1	Bacteria Firmicutes Bacilli Lactobacillales Enterococcaceae *Enterococcus faecalis* EnGen0202
Efa-112-1	Bacteria Firmicutes Bacilli Lactobacillales Enterococcaceae *Enterococcus faecalis* EnGen0203
Efa-113-1	Bacteria Firmicutes Bacilli Lactobacillales Enterococcaceae *Enterococcus faecalis* EnGen0204
Efa-114-1	Bacteria Firmicutes Bacilli Lactobacillales Enterococcaceae *Enterococcus faecalis* EnGen0205
Efa-115-1	Bacteria Firmicutes Bacilli Lactobacillales Enterococcaceae *Enterococcus faecalis* EnGen0206
Efa-116-1	Bacteria Firmicutes Bacilli Lactobacillales Enterococcaceae *Enterococcus faecalis* EnGen0207
Efa-117-1	Bacteria Firmicutes Bacilli Lactobacillales Enterococcaceae *Enterococcus faecalis* EnGen0208
Efa-118-1	Bacteria Firmicutes Bacilli Lactobacillales Enterococcaceae *Enterococcus faecalis* EnGen0209
Efa-119-1	Bacteria Firmicutes Bacilli Lactobacillales Enterococcaceae *Enterococcus faecalis* EnGen0210
Efa-120-1	Bacteria Firmicutes Bacilli Lactobacillales Enterococcaceae *Enterococcus faecalis* EnGen0211
Efa-121-1	Bacteria Firmicutes Bacilli Lactobacillales Enterococcaceae *Enterococcus faecalis* EnGen0212
Efa-122-1	Bacteria Firmicutes Bacilli Lactobacillales Enterococcaceae *Enterococcus faecalis* EnGen0213
Efa-123-1	Bacteria Firmicutes Bacilli Lactobacillales Enterococcaceae *Enterococcus faecalis* EnGen0214
Efa-124-1	Bacteria Firmicutes Bacilli Lactobacillales Enterococcaceae *Enterococcus faecalis* EnGen0215
Efa-125-1	Bacteria Firmicutes Bacilli Lactobacillales Enterococcaceae *Enterococcus faecalis* EnGen0216
Efa-126-1	Bacteria Firmicutes Bacilli Lactobacillales Enterococcaceae *Enterococcus faecalis* EnGen0217
Efa-127-1	Bacteria Firmicutes Bacilli Lactobacillales Enterococcaceae *Enterococcus faecalis* EnGen0218
Efa-128-1	Bacteria Firmicutes Bacilli Lactobacillales Enterococcaceae *Enterococcus faecalis* EnGen0219
Efa-129-1	Bacteria Firmicutes Bacilli Lactobacillales Enterococcaceae *Enterococcus faecalis* EnGen0220
Efa-130-1	Bacteria Firmicutes Bacilli Lactobacillales Enterococcaceae *Enterococcus faecalis* EnGen0221
Efa-131-1	Bacteria Firmicutes Bacilli Lactobacillales Enterococcaceae *Enterococcus faecalis* EnGen0222
Efa-132-1	Bacteria Firmicutes Bacilli Lactobacillales Enterococcaceae *Enterococcus faecalis* EnGen0223
Efa-133-1	Bacteria Firmicutes Bacilli Lactobacillales Enterococcaceae *Enterococcus faecalis* EnGen0224
Efa-134-1	Bacteria Firmicutes Bacilli Lactobacillales Enterococcaceae *Enterococcus faecalis* EnGen0225
Efa-135-1	Bacteria Firmicutes Bacilli Lactobacillales Enterococcaceae *Enterococcus faecalis* EnGen0226
Efa-136-1	Bacteria Firmicutes Bacilli Lactobacillales Enterococcaceae *Enterococcus faecalis* EnGen0228
Efa-137-1	Bacteria Firmicutes Bacilli Lactobacillales Enterococcaceae *Enterococcus faecalis* EnGen0231
Efa-138-1	Bacteria Firmicutes Bacilli Lactobacillales Enterococcaceae *Enterococcus faecalis* EnGen0232
Efa-139-1	Bacteria Firmicutes Bacilli Lactobacillales Enterococcaceae *Enterococcus faecalis* EnGen0235
Efa-140-1	Bacteria Firmicutes Bacilli Lactobacillales Enterococcaceae *Enterococcus faecalis* EnGen0237
Efa-141-1	Bacteria Firmicutes Bacilli Lactobacillales Enterococcaceae *Enterococcus faecalis* EnGen0238
Efa-142-1	Bacteria Firmicutes Bacilli Lactobacillales Enterococcaceae *Enterococcus faecalis* EnGen0239
Efa-143-1	Bacteria Firmicutes Bacilli Lactobacillales Enterococcaceae *Enterococcus faecalis* EnGen0240
Efa-144-1	Bacteria Firmicutes Bacilli Lactobacillales Enterococcaceae *Enterococcus faecalis* EnGen0241
Efa-145-1	Bacteria Firmicutes Bacilli Lactobacillales Enterococcaceae *Enterococcus faecalis* EnGen0242
Efa-146-1	Bacteria Firmicutes Bacilli Lactobacillales Enterococcaceae *Enterococcus faecalis* EnGen0243
Efa-147-1	Bacteria Firmicutes Bacilli Lactobacillales Enterococcaceae *Enterococcus faecalis* EnGen0244
Efa-148-1	Bacteria Firmicutes Bacilli Lactobacillales Enterococcaceae *Enterococcus faecalis* EnGen0245
Efa-149-1	Bacteria Firmicutes Bacilli Lactobacillales Enterococcaceae *Enterococcus faecalis* EnGen0246
Efa-150-1	Bacteria Firmicutes Bacilli Lactobacillales Enterococcaceae *Enterococcus faecalis* EnGen0247
Efa-151-1	Bacteria Firmicutes Bacilli Lactobacillales Enterococcaceae *Enterococcus faecalis* EnGen0248
Efa-152-1	Bacteria Firmicutes Bacilli Lactobacillales Enterococcaceae *Enterococcus faecalis* EnGen0249
Efa-153-1	Bacteria Firmicutes Bacilli Lactobacillales Enterococcaceae *Enterococcus faecalis* EnGen0250
Efa-154-1	Bacteria Firmicutes Bacilli Lactobacillales Enterococcaceae *Enterococcus faecalis* EnGen0251
Efa-155-1	Bacteria Firmicutes Bacilli Lactobacillales Enterococcaceae *Enterococcus faecalis* EnGen0252
Efa-156-1	Bacteria Firmicutes Bacilli Lactobacillales Enterococcaceae *Enterococcus faecalis* EnGen0279

Efa-157-1	Bacteria Firmicutes Bacilli Lactobacillales Enterococcaceae *Enterococcus faecalis* EnGen0280
Efa-158-1	Bacteria Firmicutes Bacilli Lactobacillales Enterococcaceae *Enterococcus faecalis* EnGen0281
Efa-159-1	Bacteria Firmicutes Bacilli Lactobacillales Enterococcaceae *Enterococcus faecalis* EnGen0282
Efa-160-1	Bacteria Firmicutes Bacilli Lactobacillales Enterococcaceae *Enterococcus faecalis* EnGen0283
Efa-161-1	Bacteria Firmicutes Bacilli Lactobacillales Enterococcaceae *Enterococcus faecalis* EnGen0284
Efa-162-1	Bacteria Firmicutes Bacilli Lactobacillales Enterococcaceae *Enterococcus faecalis* EnGen0285
Efa-163-1	Bacteria Firmicutes Bacilli Lactobacillales Enterococcaceae *Enterococcus faecalis* EnGen0286
Efa-164-1	Bacteria Firmicutes Bacilli Lactobacillales Enterococcaceae *Enterococcus faecalis* EnGen0287
Efa-165-1	Bacteria Firmicutes Bacilli Lactobacillales Enterococcaceae *Enterococcus faecalis* EnGen0289
Efa-166-1	Bacteria Firmicutes Bacilli Lactobacillales Enterococcaceae *Enterococcus faecalis* EnGen0290
Efa-167-1	Bacteria Firmicutes Bacilli Lactobacillales Enterococcaceae *Enterococcus faecalis* EnGen0291
Efa-168-1	Bacteria Firmicutes Bacilli Lactobacillales Enterococcaceae *Enterococcus faecalis* EnGen0293
Efa-169-1	Bacteria Firmicutes Bacilli Lactobacillales Enterococcaceae *Enterococcus faecalis* EnGen0294
Efa-170-1	Bacteria Firmicutes Bacilli Lactobacillales Enterococcaceae *Enterococcus faecalis* EnGen0295
Efa-171-1	Bacteria Firmicutes Bacilli Lactobacillales Enterococcaceae *Enterococcus faecalis* EnGen0297
Efa-172-1	Bacteria Firmicutes Bacilli Lactobacillales Enterococcaceae *Enterococcus faecalis* EnGen0298
Efa-173-1	Bacteria Firmicutes Bacilli Lactobacillales Enterococcaceae *Enterococcus faecalis* EnGen0299
Efa-174-1	Bacteria Firmicutes Bacilli Lactobacillales Enterococcaceae *Enterococcus faecalis* EnGen0300
Efa-175-1	Bacteria Firmicutes Bacilli Lactobacillales Enterococcaceae *Enterococcus faecalis* EnGen0301
Efa-176-1	Bacteria Firmicutes Bacilli Lactobacillales Enterococcaceae *Enterococcus faecalis* EnGen0302
Efa-177-1	Bacteria Firmicutes Bacilli Lactobacillales Enterococcaceae *Enterococcus faecalis* EnGen0303
Efa-178-1	Bacteria Firmicutes Bacilli Lactobacillales Enterococcaceae *Enterococcus faecalis* EnGen0304
Efa-179-1	Bacteria Firmicutes Bacilli Lactobacillales Enterococcaceae *Enterococcus faecalis* EnGen0306
Efa-180-1	Bacteria Firmicutes Bacilli Lactobacillales Enterococcaceae *Enterococcus faecalis* EnGen0307
Efa-181-1	Bacteria Firmicutes Bacilli Lactobacillales Enterococcaceae *Enterococcus faecalis* EnGen0310
Efa-182-1	Bacteria Firmicutes Bacilli Lactobacillales Enterococcaceae *Enterococcus faecalis* EnGen0310 = MMH594
Efa-183-1	Bacteria Firmicutes Bacilli Lactobacillales Enterococcaceae *Enterococcus faecalis* EnGen0311
Efa-184-1	Bacteria Firmicutes Bacilli Lactobacillales Enterococcaceae *Enterococcus faecalis* EnGen0326
Efa-185-1	Bacteria Firmicutes Bacilli Lactobacillales Enterococcaceae *Enterococcus faecalis* EnGen0327
Efa-186-1	Bacteria Firmicutes Bacilli Lactobacillales Enterococcaceae *Enterococcus faecalis* EnGen0329
Efa-187-1	Bacteria Firmicutes Bacilli Lactobacillales Enterococcaceae *Enterococcus faecalis* EnGen0330
Efa-188-1	Bacteria Firmicutes Bacilli Lactobacillales Enterococcaceae *Enterococcus faecalis* EnGen0331
Efa-189-1	Bacteria Firmicutes Bacilli Lactobacillales Enterococcaceae *Enterococcus faecalis* EnGen0332
Efa-190-1	Bacteria Firmicutes Bacilli Lactobacillales Enterococcaceae *Enterococcus faecalis* EnGen0334
Efa-191-1	Bacteria Firmicutes Bacilli Lactobacillales Enterococcaceae *Enterococcus faecalis* EnGen0335
Efa-192-1	Bacteria Firmicutes Bacilli Lactobacillales Enterococcaceae *Enterococcus faecalis* EnGen0336
Efa-193-1	Bacteria Firmicutes Bacilli Lactobacillales Enterococcaceae *Enterococcus faecalis* EnGen0337
Efa-194-1	Bacteria Firmicutes Bacilli Lactobacillales Enterococcaceae *Enterococcus faecalis* EnGen0338
Efa-195-1	Bacteria Firmicutes Bacilli Lactobacillales Enterococcaceae *Enterococcus faecalis* EnGen0339
Efa-196-1	Bacteria Firmicutes Bacilli Lactobacillales Enterococcaceae *Enterococcus faecalis* EnGen0340
Efa-197-1	Bacteria Firmicutes Bacilli Lactobacillales Enterococcaceae *Enterococcus faecalis* EnGen0341
Efa-198-1	Bacteria Firmicutes Bacilli Lactobacillales Enterococcaceae *Enterococcus faecalis* EnGen0342
Efa-199-1	Bacteria Firmicutes Bacilli Lactobacillales Enterococcaceae *Enterococcus faecalis* EnGen0344
Efa-200-1	Bacteria Firmicutes Bacilli Lactobacillales Enterococcaceae *Enterococcus faecalis* EnGen0345
Efa-201-1	Bacteria Firmicutes Bacilli Lactobacillales Enterococcaceae *Enterococcus faecalis* EnGen0346
Efa-202-1	Bacteria Firmicutes Bacilli Lactobacillales Enterococcaceae *Enterococcus faecalis* EnGen0348
Efa-203-1	Bacteria Firmicutes Bacilli Lactobacillales Enterococcaceae *Enterococcus faecalis* EnGen0350
Efa-204-1	Bacteria Firmicutes Bacilli Lactobacillales Enterococcaceae *Enterococcus faecalis* EnGen0351
Efa-205-1	Bacteria Firmicutes Bacilli Lactobacillales Enterococcaceae *Enterococcus faecalis* EnGen0352
Efa-206-1	Bacteria Firmicutes Bacilli Lactobacillales Enterococcaceae *Enterococcus faecalis* EnGen0354
Efa-207-1	Bacteria Firmicutes Bacilli Lactobacillales Enterococcaceae *Enterococcus faecalis* EnGen0355
Efa-208-1	Bacteria Firmicutes Bacilli Lactobacillales Enterococcaceae *Enterococcus faecalis* EnGen0356
Efa-209-1	Bacteria Firmicutes Bacilli Lactobacillales Enterococcaceae *Enterococcus faecalis* EnGen0357
Efa-210-1	Bacteria Firmicutes Bacilli Lactobacillales Enterococcaceae *Enterococcus faecalis* EnGen0358
Efa-211-1	Bacteria Firmicutes Bacilli Lactobacillales Enterococcaceae *Enterococcus faecalis* EnGen0359
Efa-212-1	Bacteria Firmicutes Bacilli Lactobacillales Enterococcaceae *Enterococcus faecalis* EnGen0360
Efa-213-1	Bacteria Firmicutes Bacilli Lactobacillales Enterococcaceae *Enterococcus faecalis* EnGen0361
Efa-214-1	Bacteria Firmicutes Bacilli Lactobacillales Enterococcaceae *Enterococcus faecalis* EnGen0362
Efa-215-1	Bacteria Firmicutes Bacilli Lactobacillales Enterococcaceae *Enterococcus faecalis* EnGen0363
Efa-216-1	Bacteria Firmicutes Bacilli Lactobacillales Enterococcaceae *Enterococcus faecalis* EnGen0364
Efa-217-1	Bacteria Firmicutes Bacilli Lactobacillales Enterococcaceae *Enterococcus faecalis* EnGen0365
Efa-218-1	Bacteria Firmicutes Bacilli Lactobacillales Enterococcaceae *Enterococcus faecalis* EnGen0366
Efa-219-1	Bacteria Firmicutes Bacilli Lactobacillales Enterococcaceae *Enterococcus faecalis* EnGen0367
Efa-220-1	Bacteria Firmicutes Bacilli Lactobacillales Enterococcaceae *Enterococcus faecalis* EnGen0368
Efa-221-1	Bacteria Firmicutes Bacilli Lactobacillales Enterococcaceae *Enterococcus faecalis* EnGen0369

Efa-222-1	Bacteria Firmicutes Bacilli Lactobacillales Enterococcaceae *Enterococcus faecalis* EnGen0370
Efa-223-1	Bacteria Firmicutes Bacilli Lactobacillales Enterococcaceae *Enterococcus faecalis* EnGen0374
Efa-224-1	Bacteria Firmicutes Bacilli Lactobacillales Enterococcaceae *Enterococcus faecalis* EnGen0400
Efa-225-1	Bacteria Firmicutes Bacilli Lactobacillales Enterococcaceae *Enterococcus faecalis* EnGen0401
Efa-226-1	Bacteria Firmicutes Bacilli Lactobacillales Enterococcaceae *Enterococcus faecalis* EnGen0402
Efa-227-1	Bacteria Firmicutes Bacilli Lactobacillales Enterococcaceae *Enterococcus faecalis* EnGen0403
Efa-228-1	Bacteria Firmicutes Bacilli Lactobacillales Enterococcaceae *Enterococcus faecalis* EnGen0404
Efa-229-1	Bacteria Firmicutes Bacilli Lactobacillales Enterococcaceae *Enterococcus faecalis* EnGen0405
Efa-230-1	Bacteria Firmicutes Bacilli Lactobacillales Enterococcaceae *Enterococcus faecalis* EnGen0406
Efa-231-1	Bacteria Firmicutes Bacilli Lactobacillales Enterococcaceae *Enterococcus faecalis* EnGen0407
Efa-232-1	Bacteria Firmicutes Bacilli Lactobacillales Enterococcaceae *Enterococcus faecalis* EnGen0408
Efa-233-1	Bacteria Firmicutes Bacilli Lactobacillales Enterococcaceae *Enterococcus faecalis* EnGen0410
Efa-234-1	Bacteria Firmicutes Bacilli Lactobacillales Enterococcaceae *Enterococcus faecalis* EnGen0412
Efa-235-1	Bacteria Firmicutes Bacilli Lactobacillales Enterococcaceae *Enterococcus faecalis* EnGen0413
Efa-236-1	Bacteria Firmicutes Bacilli Lactobacillales Enterococcaceae *Enterococcus faecalis* EnGen0414
Efa-237-1	Bacteria Firmicutes Bacilli Lactobacillales Enterococcaceae *Enterococcus faecalis* EnGen0415
Efa-238-1	Bacteria Firmicutes Bacilli Lactobacillales Enterococcaceae *Enterococcus faecalis* EnGen0416
Efa-239-1	Bacteria Firmicutes Bacilli Lactobacillales Enterococcaceae *Enterococcus faecalis* EnGen0417
Efa-240-1	Bacteria Firmicutes Bacilli Lactobacillales Enterococcaceae *Enterococcus faecalis* EnGen0418
Efa-241-1	Bacteria Firmicutes Bacilli Lactobacillales Enterococcaceae *Enterococcus faecalis* EnGen0419
Efa-242-1	Bacteria Firmicutes Bacilli Lactobacillales Enterococcaceae *Enterococcus faecalis* EnGen0420
Efa-243-1	Bacteria Firmicutes Bacilli Lactobacillales Enterococcaceae *Enterococcus faecalis* EnGen0421
Efa-244-1	Bacteria Firmicutes Bacilli Lactobacillales Enterococcaceae *Enterococcus faecalis* EnGen0422
Efa-245-1	Bacteria Firmicutes Bacilli Lactobacillales Enterococcaceae *Enterococcus faecalis* EnGen0423
Efa-246-1	Bacteria Firmicutes Bacilli Lactobacillales Enterococcaceae *Enterococcus faecalis* EnGen0424
Efa-247-1	Bacteria Firmicutes Bacilli Lactobacillales Enterococcaceae *Enterococcus faecalis* EnGen0425
Efa-248-1	Bacteria Firmicutes Bacilli Lactobacillales Enterococcaceae *Enterococcus faecalis* EnGen0426
Efa-249-1	Bacteria Firmicutes Bacilli Lactobacillales Enterococcaceae *Enterococcus faecalis* EnGen0427
Efa-27-1	Bacteria Firmicutes Bacilli Lactobacillales Enterococcaceae *Enterococcus faecalis* ERV103
Efa-28-1	Bacteria Firmicutes Bacilli Lactobacillales Enterococcaceae *Enterococcus faecalis* ERV116
Efa-29-1	Bacteria Firmicutes Bacilli Lactobacillales Enterococcaceae *Enterococcus faecalis* ERV129
Efa-30-1	Bacteria Firmicutes Bacilli Lactobacillales Enterococcaceae *Enterococcus faecalis* ERV25
Efa-31-1	Bacteria Firmicutes Bacilli Lactobacillales Enterococcaceae *Enterococcus faecalis* ERV31
Efa-32-1	Bacteria Firmicutes Bacilli Lactobacillales Enterococcaceae *Enterococcus faecalis* ERV37
Efa-33-1	Bacteria Firmicutes Bacilli Lactobacillales Enterococcaceae *Enterococcus faecalis* ERV41
Efa-34-1	Bacteria Firmicutes Bacilli Lactobacillales Enterococcaceae *Enterococcus faecalis* ERV62
Efa-35-1	Bacteria Firmicutes Bacilli Lactobacillales Enterococcaceae *Enterococcus faecalis* ERV63
Efa-36-1	Bacteria Firmicutes Bacilli Lactobacillales Enterococcaceae *Enterococcus faecalis* ERV65
Efa-37-1	Bacteria Firmicutes Bacilli Lactobacillales Enterococcaceae *Enterococcus faecalis* ERV68
Efa-38-1	Bacteria Firmicutes Bacilli Lactobacillales Enterococcaceae *Enterococcus faecalis* ERV72
Efa-39-1	Bacteria Firmicutes Bacilli Lactobacillales Enterococcaceae *Enterococcus faecalis* ERV73
Efa-40-1	Bacteria Firmicutes Bacilli Lactobacillales Enterococcaceae *Enterococcus faecalis* ERV81
Efa-41-1	Bacteria Firmicutes Bacilli Lactobacillales Enterococcaceae *Enterococcus faecalis* ERV85
Efa-42-1	Bacteria Firmicutes Bacilli Lactobacillales Enterococcaceae *Enterococcus faecalis* ERV93
Efa-250-1	Bacteria Firmicutes Bacilli Lactobacillales Enterococcaceae *Enterococcus faecalis* F01966
Efa-251-1	Bacteria Firmicutes Bacilli Lactobacillales Enterococcaceae *Enterococcus faecalis* FL2
Efa-252-1	Bacteria Firmicutes Bacilli Lactobacillales Enterococcaceae *Enterococcus faecalis* GA2
Efa-253-1	Bacteria Firmicutes Bacilli Lactobacillales Enterococcaceae *Enterococcus faecalis* GAN13
Efa-254-1	Bacteria Firmicutes Bacilli Lactobacillales Enterococcaceae *Enterococcus faecalis* JH2-2
Efa-255-1	Bacteria Firmicutes Bacilli Lactobacillales Enterococcaceae *Enterococcus faecalis* KI-6-1-110608-1
Efa-256-1	Bacteria Firmicutes Bacilli Lactobacillales Enterococcaceae *Enterococcus faecalis* KS19
Efa-257-1	Bacteria Firmicutes Bacilli Lactobacillales Enterococcaceae *Enterococcus faecalis* LA3B-2
Efa-258-1	Bacteria Firmicutes Bacilli Lactobacillales Enterococcaceae *Enterococcus faecalis* M7
Efa-259-1	Bacteria Firmicutes Bacilli Lactobacillales Enterococcaceae *Enterococcus faecalis* MD6
Efa-260-1	Bacteria Firmicutes Bacilli Lactobacillales Enterococcaceae *Enterococcus faecalis* MN16
Efa-262-1	Bacteria Firmicutes Bacilli Lactobacillales Enterococcaceae *Enterococcus faecalis* MTmid8
Efa-261-1	Bacteria Firmicutes Bacilli Lactobacillales Enterococcaceae *Enterococcus faecalis* MTUP9
Efa-263-1	Bacteria Firmicutes Bacilli Lactobacillales Enterococcaceae *Enterococcus faecalis* NJ44
Efa-264-1	Bacteria Firmicutes Bacilli Lactobacillales Enterococcaceae *Enterococcus faecalis* NY9
Efa-265-1	Bacteria Firmicutes Bacilli Lactobacillales Enterococcaceae *Enterococcus faecalis* OG1X
Efa-266-1	Bacteria Firmicutes Bacilli Lactobacillales Enterococcaceae *Enterococcus faecalis* R508
Efa-267-1	Bacteria Firmicutes Bacilli Lactobacillales Enterococcaceae *Enterococcus faecalis* RP2S-4
Efa-268-1	Bacteria Firmicutes Bacilli Lactobacillales Enterococcaceae *Enterococcus faecalis* SLO2C-1
Efa-269-1	Bacteria Firmicutes Bacilli Lactobacillales Enterococcaceae *Enterococcus faecalis* TX0012
Efa-270-1	Bacteria Firmicutes Bacilli Lactobacillales Enterococcaceae *Enterococcus faecalis* TX0017

Efa-271-1	Bacteria Firmicutes Bacilli Lactobacillales Enterococcaceae *Enterococcus faecalis* TX0027
Efa-272-1	Bacteria Firmicutes Bacilli Lactobacillales Enterococcaceae *Enterococcus faecalis* TX0031
Efa-273-1	Bacteria Firmicutes Bacilli Lactobacillales Enterococcaceae *Enterococcus faecalis* TX0043
Efa-274-1	Bacteria Firmicutes Bacilli Lactobacillales Enterococcaceae *Enterococcus faecalis* TX0102
Efa-275-1	Bacteria Firmicutes Bacilli Lactobacillales Enterococcaceae *Enterococcus faecalis* TX0109
Efa-276-1	Bacteria Firmicutes Bacilli Lactobacillales Enterococcaceae *Enterococcus faecalis* TX0309A
Efa-277-1	Bacteria Firmicutes Bacilli Lactobacillales Enterococcaceae *Enterococcus faecalis* TX0309B
Efa-278-1	Bacteria Firmicutes Bacilli Lactobacillales Enterococcaceae *Enterococcus faecalis* TX0312
Efa-279-1	Bacteria Firmicutes Bacilli Lactobacillales Enterococcaceae *Enterococcus faecalis* TX0411
Efa-280-1	Bacteria Firmicutes Bacilli Lactobacillales Enterococcaceae *Enterococcus faecalis* TX0470
Efa-281-1	Bacteria Firmicutes Bacilli Lactobacillales Enterococcaceae *Enterococcus faecalis* TX0630
Efa-282-1	Bacteria Firmicutes Bacilli Lactobacillales Enterococcaceae *Enterococcus faecalis* TX0645
Efa-283-1	Bacteria Firmicutes Bacilli Lactobacillales Enterococcaceae *Enterococcus faecalis* TX0860
Efa-284-1	Bacteria Firmicutes Bacilli Lactobacillales Enterococcaceae *Enterococcus faecalis* TX1302
Efa-285-1	Bacteria Firmicutes Bacilli Lactobacillales Enterococcaceae *Enterococcus faecalis* TX1342
Efa-286-1	Bacteria Firmicutes Bacilli Lactobacillales Enterococcaceae *Enterococcus faecalis* TX1346
Efa-287-1	Bacteria Firmicutes Bacilli Lactobacillales Enterococcaceae *Enterococcus faecalis* TX2137
Efa-288-1	Bacteria Firmicutes Bacilli Lactobacillales Enterococcaceae *Enterococcus faecalis* TX4000
Efa-289-1	Bacteria Firmicutes Bacilli Lactobacillales Enterococcaceae *Enterococcus faecalis* TX4244
Efa-290-1	Bacteria Firmicutes Bacilli Lactobacillales Enterococcaceae *Enterococcus faecalis* UP2S-6
Efa-291-1 to Efa-291-3	Bacteria Firmicutes Bacilli Lactobacillales Enterococcaceae *Enterococcus faecalis* V583
Efa-292-1	Bacteria Firmicutes Bacilli Lactobacillales Enterococcaceae *Enterococcus faecalis* VC1B-1
Efa-293-1	Bacteria Firmicutes Bacilli Lactobacillales Enterococcaceae *Enterococcus faecalis* WKS-26-18-2
Efa-294-1 to Efa-294-3	Bacteria Firmicutes Bacilli Lactobacillales Enterococcaceae *Enterococcus faecium*
Efa-295-1	Bacteria Firmicutes Bacilli Lactobacillales Enterococcaceae *Enterococcus faecium* EnGen0038
Efa-296-1	Bacteria Firmicutes Bacilli Lactobacillales Enterococcaceae *Enterococcus faecium* EnGen0253
Efa-297-1	Bacteria Firmicutes Bacilli Lactobacillales Enterococcaceae *Enterococcus faecium* SB2C-2
Efa-298-1	Bacteria Firmicutes Bacilli Lactobacillales Enterococcaceae *Enterococcus faecium* UC7256
Efa-299-1	Bacteria Firmicutes Bacilli Lactobacillales Enterococcaceae *Enterococcus faecium* UC8668
Ega-1-1 to Ega-1-6	Bacteria Firmicutes Bacilli Lactobacillales Enterococcaceae *Enterococcus gallinarum*
Eha-1-1	Bacteria Firmicutes Bacilli Lactobacillales Enterococcaceae *Enterococcus haemoperoxidus*
Eha-2-1	Bacteria Firmicutes Bacilli Lactobacillales Enterococcaceae *Enterococcus haemoperoxidus* ATCC BAA-382
Emo-1-1	Bacteria Firmicutes Bacilli Lactobacillales Enterococcaceae *Enterococcus moraviensis*
Emo-2-1	Bacteria Firmicutes Bacilli Lactobacillales Enterococcaceae *Enterococcus moraviensis* ATCC BAA-383
Epl-1-1	Bacteria Firmicutes Bacilli Lactobacillales Enterococcaceae *Enterococcus plantarum*
Equ-1-1 to Equ-1-2	Bacteria Firmicutes Bacilli Lactobacillales Enterococcaceae *Enterococcus quebecensis*
Eri-1-1	Bacteria Firmicutes Bacilli Lactobacillales Enterococcaceae *Enterococcus rivorum*
Esa-1-1	Bacteria Firmicutes Bacilli Lactobacillales Enterococcaceae *Enterococcus saccharolyticus* 30_1
Esi-1-1	Bacteria Firmicutes Bacilli Lactobacillales Enterococcaceae *Enterococcus silesiacus*
Esp-1-1	Bacteria Firmicutes Bacilli Lactobacillales Enterococcaceae *Enterococcus sp.* 1140_ESPC
Esp-2-1	Bacteria Firmicutes Bacilli Lactobacillales Enterococcaceae *Enterococcus sp.* 255_ESPC
Esp-3-1	Bacteria Firmicutes Bacilli Lactobacillales Enterococcaceae *Enterococcus sp.* 3G1_DIV0629
Esp-4-1	Bacteria Firmicutes Bacilli Lactobacillales Enterococcaceae *Enterococcus sp.* 4G2_DIV0659
Esp-5-1	Bacteria Firmicutes Bacilli Lactobacillales Enterococcaceae *Enterococcus sp.* 5B7_DIV0075
Esp-6-1	Bacteria Firmicutes Bacilli Lactobacillales Enterococcaceae *Enterococcus sp.* HMSC061C05
Esp-7-1	Bacteria Firmicutes Bacilli Lactobacillales Enterococcaceae *Enterococcus sp.* HMSC072F02
Esp-8-1	Bacteria Firmicutes Bacilli Lactobacillales Enterococcaceae *Enterococcus sp.* HMSC073E09
Esp-9-1	Bacteria Firmicutes Bacilli Lactobacillales Enterococcaceae *Enterococcus sp.* HMSC076E04
Esp-10-1	Bacteria Firmicutes Bacilli Lactobacillales Enterococcaceae *Enterococcus sp.* HMSC078F03
Esp-11-1	Bacteria Firmicutes Bacilli Lactobacillales Enterococcaceae *Enterococcus sp.* RIT-PI-f
Ete-1-1 to Ete-1-2	Bacteria Firmicutes Bacilli Lactobacillales Enterococcaceae *Enterococcus termitis*
Eth-1-1	Bacteria Firmicutes Bacilli Lactobacillales Enterococcaceae *Enterococcus thailandicus*
Eur-1-1	Bacteria Firmicutes Bacilli Lactobacillales Enterococcaceae *Enterococcus ureasiticus*
Eur-2-1 to Eur-2-2	Bacteria Firmicutes Bacilli Lactobacillales Enterococcaceae *Enterococcus ureilyticus*
Ewa-1-1	Bacteria Firmicutes Bacilli Lactobacillales Enterococcaceae *Enterococcus wangshanyuanii*
Lag-1-1	Bacteria Firmicutes Bacilli Lactobacillales Lactobacillaceae *Lactobacillus agilis*
Lag-2-1	Bacteria Firmicutes Bacilli Lactobacillales Lactobacillaceae *Lactobacillus agilis* DSM 20509
Lal-1-1	Bacteria Firmicutes Bacilli Lactobacillales Lactobacillaceae *Lactobacillus alimentarius* DSM 20249
Lal-2-1	Bacteria Firmicutes Bacilli Lactobacillales Lactobacillaceae *Lactobacillus allii*
Lbr-1-1	Bacteria Firmicutes Bacilli Lactobacillales Lactobacillaceae *Lactobacillus brantae* DSM 23927
Lco-1-1	Bacteria Firmicutes Bacilli Lactobacillales Lactobacillaceae *Lactobacillus collinoides*
Lco-2-1	Bacteria Firmicutes Bacilli Lactobacillales Lactobacillaceae *Lactobacillus collinoides* DSM 20515 = JCM 1123
Lco-3-1	Bacteria Firmicutes Bacilli Lactobacillales Lactobacillaceae *Lactobacillus coryniformis subsp. torquens* DSM 20004 = KCTC 3535
Lcr-1-1	Bacteria Firmicutes Bacilli Lactobacillales Lactobacillaceae *Lactobacillus crispatus*
Lcr-2-1	Bacteria Firmicutes Bacilli Lactobacillales Lactobacillaceae *Lactobacillus crustorum*

Lcr-3-1	Bacteria Firmicutes Bacilli Lactobacillales Lactobacillaceae *Lactobacillus crustorum* JCM 15951
Lcu-1-1	Bacteria Firmicutes Bacilli Lactobacillales Lactobacillaceae *Lactobacillus curieae*
Lcu-2-1	Bacteria Firmicutes Bacilli Lactobacillales Lactobacillaceae *Lactobacillus curvatus*
Lde-1-1	Bacteria Firmicutes Bacilli Lactobacillales Lactobacillaceae *Lactobacillus dextrinicus* DSM 20335
Ldi-1-1	Bacteria Firmicutes Bacilli Lactobacillales Lactobacillaceae *Lactobacillus diolivorans* DSM 14421
Lfa-1-1	Bacteria Firmicutes Bacilli Lactobacillales Lactobacillaceae *Lactobacillus farciminis* KCTC 3681 = DSM 20184
Lfa-2-1 to Lfa-2-2	Bacteria Firmicutes Bacilli Lactobacillales Lactobacillaceae *Lactobacillus farraginis* DSM 18382 = JCM 14108
Lfu-1-1	Bacteria Firmicutes Bacilli Lactobacillales Lactobacillaceae *Lactobacillus fuchuensis* DSM 14340 = JCM 11249
Lga-1-1 to Lga-1-2	Bacteria Firmicutes Bacilli Lactobacillales Lactobacillaceae *Lactobacillus gallinarum*
Lgi-1-1	Bacteria Firmicutes Bacilli Lactobacillales Lactobacillaceae *Lactobacillus ginsenosidimutans*
Lhe-1-1 to Lhe-1-4	Bacteria Firmicutes Bacilli Lactobacillales Lactobacillaceae *Lactobacillus helveticus*
Lhe-2-1	Bacteria Firmicutes Bacilli Lactobacillales Lactobacillaceae *Lactobacillus helveticus* CIRM-BIA 951
Lhi-1-1	Bacteria Firmicutes Bacilli Lactobacillales Lactobacillaceae *Lactobacillus hilgardii* DSM 20176 = ATCC 8290
Lin-1-1	Bacteria Firmicutes Bacilli Lactobacillales Lactobacillaceae *Lactobacillus intestinalis* DSM 6629
Ljo-1-1 to Ljo-1-6	Bacteria Firmicutes Bacilli Lactobacillales Lactobacillaceae *Lactobacillus johnsonii*
Ljo-2-1	Bacteria Firmicutes Bacilli Lactobacillales Lactobacillaceae *Lactobacillus johnsonii* N6.2
Lki-1-1	Bacteria Firmicutes Bacilli Lactobacillales Lactobacillaceae *Lactobacillus kimchicus* JCM 15530
Lki-2-1	Bacteria Firmicutes Bacilli Lactobacillales Lactobacillaceae *Lactobacillus kisonensis* DSM 19906 = JCM 15041
Lki-3-1	Bacteria Firmicutes Bacilli Lactobacillales Lactobacillaceae *Lactobacillus kisonensis* F0435
Lli-1-1 to Lli-1-2	Bacteria Firmicutes Bacilli Lactobacillales Lactobacillaceae *Lactobacillus lindneri*
Lli-2-1 to Lli-2-4	Bacteria Firmicutes Bacilli Lactobacillales Lactobacillaceae *Lactobacillus lindneri* DSM 20690 = JCM 11027
Lna-2-1	Bacteria Firmicutes Bacilli Lactobacillales Lactobacillaceae *Lactobacillus nantensis* DSM 16982
Lno-1-1 to Lno-1-3	Bacteria Firmicutes Bacilli Lactobacillales Lactobacillaceae *Lactobacillus nodensis* DSM 19682 = JCM 14932 = NBRC 107160
Loz-1-1	Bacteria Firmicutes Bacilli Lactobacillales Lactobacillaceae *Lactobacillus ozensis* DSM 23829 = JCM 17196
Lpa-1-1 to Lpa-1-2	Bacteria Firmicutes Bacilli Lactobacillales Lactobacillaceae *Lactobacillus parafarraginis* DSM 18390 = JCM 14109
Lpa-2-1 to Lpa-2-2	Bacteria Firmicutes Bacilli Lactobacillales Lactobacillaceae *Lactobacillus parakefiri*
Lpa-3-1 to Lpa-3-2	Bacteria Firmicutes Bacilli Lactobacillales Lactobacillaceae *Lactobacillus paralimentarius* DSM 13238 = JCM 10415
Lpe-1-1	Bacteria Firmicutes Bacilli Lactobacillales Lactobacillaceae *Lactobacillus pentosiphilus*
Lpl-1-1	Bacteria Firmicutes Bacilli Lactobacillales Lactobacillaceae *Lactobacillus plantarum*
Lra-1-1	Bacteria Firmicutes Bacilli Lactobacillales Lactobacillaceae *Lactobacillus rapi* DSM 19907 = JCM 15042
Lsa-1-1	Bacteria Firmicutes Bacilli Lactobacillales Lactobacillaceae *Lactobacillus sakei*
Lsa-2-1	Bacteria Firmicutes Bacilli Lactobacillales Lactobacillaceae *Lactobacillus salivarius*
Lsa-3-1	Bacteria Firmicutes Bacilli Lactobacillales Lactobacillaceae *Lactobacillus saniviri* JCM 17471 = DSM 24301
Lse-1-1	Bacteria Firmicutes Bacilli Lactobacillales Lactobacillaceae *Lactobacillus secaliphilus*
Lsp-1-1	Bacteria Firmicutes Bacilli Lactobacillales Lactobacillaceae *Lactobacillus sp.* 2-3
Lsp-2-1	Bacteria Firmicutes Bacilli Lactobacillales Lactobacillaceae *Lactobacillus sp.* HT06-2
Lsp-3-1	Bacteria Firmicutes Bacilli Lactobacillales Lactobacillaceae *Lactobacillus sp.* UMNPBX14
Lsp-4-1	Bacteria Firmicutes Bacilli Lactobacillales Lactobacillaceae *Lactobacillus sp.* UMNPBX6
Lsp-5-1	Bacteria Firmicutes Bacilli Lactobacillales Lactobacillaceae *Lactobacillus sp. wk*B8
Lta-1-1	Bacteria Firmicutes Bacilli Lactobacillales Lactobacillaceae *Lactobacillus taiwanensis*
Lul-1-1 to Lul-1-2	Bacteria Firmicutes Bacilli Lactobacillales Lactobacillaceae *Lactobacillus ultunensis* DSM 16047
Lve-1-1	Bacteria Firmicutes Bacilli Lactobacillales Lactobacillaceae *Lactobacillus versmoldensis* DSM 14857 = KCTC 3814
Pac-1-1	Bacteria Firmicutes Bacilli Lactobacillales Lactobacillaceae Pediococcus Pediococcus acidilactici group *Pediococcus acidilactici*
Par-1-1	Bacteria Firmicutes Bacilli Lactobacillales Lactobacillaceae *Pediococcus argentinicus*
Wbo-1-1	Bacteria Firmicutes Bacilli Lactobacillales Leuconostocaceae *Weissella bombi*
Whe-1-1 to Whe-1-2	Bacteria Firmicutes Bacilli Lactobacillales Leuconostocaceae *Weissella hellenica*
Wjo-1-1	Bacteria Firmicutes Bacilli Lactobacillales Leuconostocaceae *Weissella jogaejeotgali*
Lga-2-1 to Lga-2-3	Bacteria Firmicutes Bacilli Lactobacillales Streptococcaceae *Lactococcus garvieae*
Lga-3-1	Bacteria Firmicutes Bacilli Lactobacillales Streptococcaceae *Lactococcus garvieae* 8831
Lga-4-1	Bacteria Firmicutes Bacilli Lactobacillales Streptococcaceae *Lactococcus garvieae* DCC43
Lga-5-1	Bacteria Firmicutes Bacilli Lactobacillales Streptococcaceae *Lactococcus garvieae* I113
Lga-6-1	Bacteria Firmicutes Bacilli Lactobacillales Streptococcaceae *Lactococcus garvieae* TB25
Lla-1-1 to Lla-1-13	Bacteria Firmicutes Bacilli Lactobacillales Streptococcaceae *Lactococcus lactis*
Lla-2-1	Bacteria Firmicutes Bacilli Lactobacillales Streptococcaceae *Lactococcus lactis* RTB018
Lla-3-1 to Lla-3-6	Bacteria Firmicutes Bacilli Lactobacillales Streptococcaceae *Lactococcus lactis subsp. cremoris*
Lla-4-1	Bacteria Firmicutes Bacilli Lactobacillales Streptococcaceae *Lactococcus lactis subsp. cremoris* GE214
Lla-5-1	Bacteria Firmicutes Bacilli Lactobacillales Streptococcaceae *Lactococcus lactis subsp. cremoris* HP
Lla-6-1	Bacteria Firmicutes Bacilli Lactobacillales Streptococcaceae *Lactococcus lactis subsp. cremoris* IBB477
Lla-7-1	Bacteria Firmicutes Bacilli Lactobacillales Streptococcaceae *Lactococcus lactis subsp. cremoris* KW2
Lla-8-1	Bacteria Firmicutes Bacilli Lactobacillales Streptococcaceae *Lactococcus lactis subsp. cremoris* NBRC 100676
Lla-9-1	Bacteria Firmicutes Bacilli Lactobacillales Streptococcaceae *Lactococcus lactis subsp. cremoris* UC509.9
Lla-10-1 to Lla-10-3	Bacteria Firmicutes Bacilli Lactobacillales Streptococcaceae *Lactococcus lactis subsp. lactis*
Lla-11-1	Bacteria Firmicutes Bacilli Lactobacillales Streptococcaceae *Lactococcus lactis subsp. lactis* 1AA59
Lla-13-1 to Lla-13-2	Bacteria Firmicutes Bacilli Lactobacillales Streptococcaceae *Lactococcus lactis subsp. lactis bv. diacetylactis*
Lla-14-1	Bacteria Firmicutes Bacilli Lactobacillales Streptococcaceae *Lactococcus lactis subsp. lactis bv. diacetylactis str.* LD61

Lla-15-1	Bacteria Firmicutes Bacilli Lactobacillales Streptococcaceae *Lactococcus lactis subsp. lactis bv. diacetylactis str.* TIFN2
Lla-16-1	Bacteria Firmicutes Bacilli Lactobacillales Streptococcaceae *Lactococcus lactis subsp. lactis bv. diacetylactis str.* TIFN4
Lla-12-1	Bacteria Firmicutes Bacilli Lactobacillales Streptococcaceae *Lactococcus lactis subsp. lactis* KLDS 4.0325
Lla-17-1	Bacteria Firmicutes Bacilli Lactobacillales Streptococcaceae *Lactococcus lactis subsp. tructae*
Ssp-1-1	Bacteria Firmicutes Bacilli Lactobacillales Streptococcaceae *Streptococcus sp.* X13SY08
Ssu-1-1 to Ssu-1-8	Bacteria Firmicutes Bacilli Lactobacillales Streptococcaceae *Streptococcus suis*
Ssu-2-1	Bacteria Firmicutes Bacilli Lactobacillales Streptococcaceae *Streptococcus suis* YS104
Ssu-3-1	Bacteria Firmicutes Bacilli Lactobacillales Streptococcaceae *Streptococcus suis* YS110
Ssu-4-1	Bacteria Firmicutes Bacilli Lactobacillales Streptococcaceae *Streptococcus suis* YS111
Ssu-5-1	Bacteria Firmicutes Bacilli Lactobacillales Streptococcaceae *Streptococcus suis* YS123
Ssu-6-1	Bacteria Firmicutes Bacilli Lactobacillales Streptococcaceae *Streptococcus suis* YS131
Ssu-7-1	Bacteria Firmicutes Bacilli Lactobacillales Streptococcaceae *Streptococcus suis* YS54
Ssu-8-1	Bacteria Firmicutes Bacilli Lactobacillales Streptococcaceae *Streptococcus suis* YS77
Bpu-1-1	Bacteria Firmicutes Clostridia Clostridiales Clostridiaceae *Butyricicoccus pullicaecorum*
Cbo-1-1	Bacteria Firmicutes Clostridia Clostridiales Clostridiaceae *Clostridium bornimense*
Cbo-2-1 to Cbo-2-2	Bacteria Firmicutes Clostridia Clostridiales Clostridiaceae *Clostridium botulinum*
Cbo-3-1	Bacteria Firmicutes Clostridia Clostridiales Clostridiaceae *Clostridium botulinum* B2 275
Cbu-1-1 to Cbu-1-4	Bacteria Firmicutes Clostridia Clostridiales Clostridiaceae *Clostridium butyricum*
Cbu-2-1	Bacteria Firmicutes Clostridia Clostridiales Clostridiaceae *Clostridium butyricum* 60E.3
Cbu-3-1	Bacteria Firmicutes Clostridia Clostridiales Clostridiaceae *Clostridium butyricum* AGR2140
Cbu-4-1	Bacteria Firmicutes Clostridia Clostridiales Clostridiaceae *Clostridium butyricum* CWBI1009
Cbu-5-1	Bacteria Firmicutes Clostridia Clostridiales Clostridiaceae *Clostridium butyricum* DKU-01
Cbu-6-1	Bacteria Firmicutes Clostridia Clostridiales Clostridiaceae *Clostridium butyricum* DSM 10702
Cfo-1-1 to Cfo-1-2	Bacteria Firmicutes Clostridia Clostridiales Clostridiaceae *Clostridium formicaceticum*
Cin-2-1	Bacteria Firmicutes Clostridia Clostridiales Clostridiaceae *Clostridium intestinale* URNW
Cje-1-1	Bacteria Firmicutes Clostridia Clostridiales Clostridiaceae *Clostridium jeddahense*
Cni-1-1	Bacteria Firmicutes Clostridia Clostridiales Clostridiaceae *Clostridium nigeriense*
Cpa-1-1	Bacteria Firmicutes Clostridia Clostridiales Clostridiaceae *Clostridium paraputrificum*
Cph-1-1	Bacteria Firmicutes Clostridia Clostridiales Clostridiaceae *Clostridium phytofermentans* ISDg
Csa-1-1	Bacteria Firmicutes Clostridia Clostridiales Clostridiaceae *Clostridium saccharobutylicum*
Csp-1-1	Bacteria Firmicutes Clostridia Clostridiales Clostridiaceae *Clostridium sp.* 3-3
Csp-2-1	Bacteria Firmicutes Clostridia Clostridiales Clostridiaceae *Clostridium sp.* 7_3_54FAA
Csp-3-1	Bacteria Firmicutes Clostridia Clostridiales Clostridiaceae *Clostridium sp.* ASBs410
Csp-4-1	Bacteria Firmicutes Clostridia Clostridiales Clostridiaceae *Clostridium sp.* ATCC 29733
Csp-5-1	Bacteria Firmicutes Clostridia Clostridiales Clostridiaceae *Clostridium sp.* Bc-iso-3
Csp-6-1	Bacteria Firmicutes Clostridia Clostridiales Clostridiaceae *Clostridium sp.* C105KSO13
Csp-7-1	Bacteria Firmicutes Clostridia Clostridiales Clostridiaceae *Clostridium sp.* CL-2
Csp-8-1	Bacteria Firmicutes Clostridia Clostridiales Clostridiaceae *Clostridium sp.* DSM 4029
Csp-10-1	Bacteria Firmicutes Clostridia Clostridiales Clostridiaceae *Clostridium sp.* HMb25
Csp-9-1	Bacteria Firmicutes Clostridia Clostridiales Clostridiaceae *Clostridium sp.* HMSC19A10
Csp-11-1	Bacteria Firmicutes Clostridia Clostridiales Clostridiaceae *Clostridium sp.* L74
Csp-16-1	Bacteria Firmicutes Clostridia Clostridiales Clostridiaceae *Clostridium sp.* *link*-BC1
Csp-12-1 to Csp-12-2	Bacteria Firmicutes Clostridia Clostridiales Clostridiaceae *Clostridium sp.* LS
Csp-13-1	Bacteria Firmicutes Clostridia Clostridiales Clostridiaceae *Clostridium sp.* Marseille-P2415
Csp-14-1	Bacteria Firmicutes Clostridia Clostridiales Clostridiaceae *Clostridium sp.* Marseille-P299
Csp-15-1	Bacteria Firmicutes Clostridia Clostridiales Clostridiaceae *Clostridium sp.* Marseille-P3244
Cst-1-1	Bacteria Firmicutes Clostridia Clostridiales Clostridiaceae *Clostridium sticklandii* DSM 519
Csy-1-1	Bacteria Firmicutes Clostridia Clostridiales Clostridiaceae *Clostridium symbiosum* ATCC 14940
Csy-2-1	Bacteria Firmicutes Clostridia Clostridiales Clostridiaceae *Clostridium symbiosum* WAL-14163
Csy-3-1	Bacteria Firmicutes Clostridia Clostridiales Clostridiaceae *Clostridium symbiosum* WAL-14673
Gfe-1-1	Bacteria Firmicutes Clostridia Clostridiales Clostridiaceae *Geosporobacter ferrireducens*
Mha-1-1	Bacteria Firmicutes Clostridia Clostridiales Clostridiaceae *Maledivibacter halophilus*
Nfe-1-1	Bacteria Firmicutes Clostridia Clostridiales Clostridiaceae *Natronincola ferrireducens*
Npe-1-1	Bacteria Firmicutes Clostridia Clostridiales Clostridiaceae *Natronincola peptidivorans*
Tma-1-1	Bacteria Firmicutes Clostridia Clostridiales Clostridiaceae *Tindallia magadiensis*
Cba-1-1	Bacteria Firmicutes Clostridia *Clostridiales bacterium* 1_7_47FAA
Cba-2-1	Bacteria Firmicutes Clostridia *Clostridiales bacterium* CHKCI001
Cba-3-1	Bacteria Firmicutes Clostridia *Clostridiales bacterium* CHKCI006
Cba-4-1	Bacteria Firmicutes Clostridia *Clostridiales bacterium* VE202-16
Cba-5-1	Bacteria Firmicutes Clostridia *Clostridiales bacterium* VE202-26
Cba-6-1	Bacteria Firmicutes Clostridia *Clostridiales bacterium* VE202-28
Dph-1-1	Bacteria Firmicutes Clostridia Clostridiales Defluviitaleaceae *Defluviitalea phaphyphila*
Eba-1-1	Bacteria Firmicutes Clostridia Clostridiales Eubacteriaceae *Eubacterium barkeri*
Epl-2-1	Bacteria Firmicutes Clostridia Clostridiales Eubacteriaceae *Eubacterium plexicaudatum* ASF492
Esp-12-1	Bacteria Firmicutes Clostridia Clostridiales Eubacteriaceae *Eubacterium sp.* An11

Esp-13-1	Bacteria Firmicutes Clostridia Clostridiales Eubacteriaceae *Eubacterium sp.* An3
Exy-1-1	Bacteria Firmicutes Clostridia Clostridiales Eubacteriaceae *Eubacterium xylanophilum* ATCC 35991
Gni-1-1	Bacteria Firmicutes Clostridia Clostridiales Eubacteriaceae *Garciella nitratireducens* DSM 15102
Fpl-1-1	Bacteria Firmicutes Clostridia Clostridiales *Flavonifractor plautii* ATCC 29863
Fsp-1-1	Bacteria Firmicutes Clostridia Clostridiales *Flavonifractor sp.* An9
[ae-1-1	Bacteria Firmicutes Clostridia Clostridiales Lachnospiraceae [Clostridium] aerotolerans DSM 5434
[am-1-1	Bacteria Firmicutes Clostridia Clostridiales Lachnospiraceae [Clostridium] aminophilum
[am-2-1	Bacteria Firmicutes Clostridia Clostridiales Lachnospiraceae [Clostridium] aminophilum DSM 10710
[gl-1-1	Bacteria Firmicutes Clostridia Clostridiales Lachnospiraceae [Clostridium] glycyrrhizinilyticum JCM 13369
[in-1-1	Bacteria Firmicutes Clostridia Clostridiales Lachnospiraceae [Clostridium] indolis DSM 755
[sc-1-1	Bacteria Firmicutes Clostridia Clostridiales Lachnospiraceae [Clostridium] scindens VE202-05
[sy-1-1 to [sy-1-2	Bacteria Firmicutes Clostridia Clostridiales Lachnospiraceae [Clostridium] symbiosum
[gu-1-1	Bacteria Firmicutes Clostridia Clostridiales Lachnospiraceae [Desulfotomaculum] guttoideum
Asp-1-1	Bacteria Firmicutes Clostridia Clostridiales Lachnospiraceae *Anaerostipes sp.* 3_2_56FAA
Bma-1-1	Bacteria Firmicutes Clostridia Clostridiales Lachnospiraceae *Bariatricus massiliensis*
Bsp-1-1 to Bsp-1-2	Bacteria Firmicutes Clostridia Clostridiales Lachnospiraceae *Blautia sp.* An249
Bsp-2-1	Bacteria Firmicutes Clostridia Clostridiales Lachnospiraceae *Blautia sp.* Marseille-P3087
Rgn-1-1	Bacteria Firmicutes Clostridia Clostridiales Lachnospiraceae Blautia *Ruminococcus gnavus* AGR2154
Bfo-1-1	Bacteria Firmicutes Clostridia Clostridiales Lachnospiraceae *Bryantella formatexigens* DSM 14469
Csp-17-1	Bacteria Firmicutes Clostridia Clostridiales Lachnospiraceae *Cellulosilyticum sp.* I15G10I2
Dsp-1-1	Bacteria Firmicutes Clostridia Clostridiales Lachnospiraceae *Dorea sp.* 5-2
Lph-1-1	Bacteria Firmicutes Clostridia Clostridiales Lachnospiraceae *Lachnoclostridium phytofermentans*
Lph-2-1	Bacteria Firmicutes Clostridia Clostridiales Lachnospiraceae *Lachnoclostridium phytofermentans* KNHs212
Lph-3-1	Bacteria Firmicutes Clostridia Clostridiales Lachnospiraceae *Lachnoclostridium phytofermentans* KNHs2132
Lsp-6-1	Bacteria Firmicutes Clostridia Clostridiales Lachnospiraceae *Lachnoclostridium sp.* An14
Lsp-7-1	Bacteria Firmicutes Clostridia Clostridiales Lachnospiraceae *Lachnoclostridium sp.* An169
Lsp-8-1	Bacteria Firmicutes Clostridia Clostridiales Lachnospiraceae *Lachnoclostridium sp.* An181
Lsp-9-1	Bacteria Firmicutes Clostridia Clostridiales Lachnospiraceae *Lachnoclostridium sp.* An298
Lsp-10-1	Bacteria Firmicutes Clostridia Clostridiales Lachnospiraceae *Lachnoclostridium sp.* An76
Lba-1-1	Bacteria Firmicutes Clostridia Clostridiales *Lachnospiraceae bacterium*
Lba-2-1	Bacteria Firmicutes Clostridia Clostridiales *Lachnospiraceae bacterium* 3-1
Lba-3-1	Bacteria Firmicutes Clostridia Clostridiales *Lachnospiraceae bacterium* A2
Mfo-1-1	Bacteria Firmicutes Clostridia Clostridiales Lachnospiraceae *Marvinbryantia formatexigens*
Mfa-1-1	Bacteria Firmicutes Clostridia Clostridiales Lachnospiraceae *Merdimonas faecis*
Nma-1-1	Bacteria Firmicutes Clostridia Clostridiales Lachnospiraceae *Niameybacter massiliensis*
Rpe-1-1	Bacteria Firmicutes Clostridia Clostridiales Lachnospiraceae *Robinsoniella peoriensis*
Rsp-1-1	Bacteria Firmicutes Clostridia Clostridiales Lachnospiraceae *Robinsoniella sp.* KNHs210
Tsp-2-1	Bacteria Firmicutes Clostridia Clostridiales Lachnospiraceae *Tyzzerella sp.* Marseille-P3062
Mpe-1-1	Bacteria Firmicutes Clostridia Clostridiales *Monoglobus pectinilyticus*
Oru-1-1	Bacteria Firmicutes Clostridia Clostridiales Oscillospiraceae *Oscillibacter ruminantium* GH1
Osp-1-1	Bacteria Firmicutes Clostridia Clostridiales Oscillospiraceae *Oscillibacter sp.* KLE 1728
Osp-2-1	Bacteria Firmicutes Clostridia Clostridiales Oscillospiraceae *Oscillibacter sp.* KLE 1745
Oba-1-1	Bacteria Firmicutes Clostridia Clostridiales Oscillospiraceae unclassified Oscillospiraceae *Oscillospiraceae bacterium* VE202-24
Ano-1-1	Bacteria Firmicutes Clostridia Clostridiales Peptostreptococcaceae *Acetoanaerobium noterae*
Air-1-1	Bacteria Firmicutes Clostridia Clostridiales Peptostreptococcaceae *Asaccharospora irregularis* DSM 2635
Cdi-1-1	Bacteria Firmicutes Clostridia Clostridiales Peptostreptococcaceae *Clostridium difficile* Y358
Psp-1-1	Bacteria Firmicutes Clostridia Clostridiales Peptostreptococcaceae *Proteocatella sphenisci* DSM 23131
Rma-2-1	Bacteria Firmicutes Clostridia Clostridiales Peptostreptococcaceae *Romboutsia maritimum*
Psp-2-1	Bacteria Firmicutes Clostridia Clostridiales *Pseudoflavonifractor sp.* Marseille-P3106
Asp-2-1	Bacteria Firmicutes Clostridia Clostridiales Ruminococcaceae *Anaeromassilibacillus sp.* An250
Bma-2-1	Bacteria Firmicutes Clostridia Clostridiales Ruminococcaceae *Bittarella massiliensis*
Dsp-2-1	Bacteria Firmicutes Clostridia Clostridiales Ruminococcaceae *Drancourtella sp.* An12
Dsp-3-1	Bacteria Firmicutes Clostridia Clostridiales Ruminococcaceae *Drancourtella sp.* An57
Mma-1-1	Bacteria Firmicutes Clostridia Clostridiales Ruminococcaceae *Massilimaliae massiliensis*
Pma-1-1	Bacteria Firmicutes Clostridia Clostridiales Ruminococcaceae *Provencibacterium massiliense*
[st-1-1	Bacteria Firmicutes Clostridia Clostridiales Ruminococcaceae Ruminiclostridium [Clostridium] straminisolvens JCM 21531
Ral-1-1	Bacteria Firmicutes Clostridia Clostridiales Ruminococcaceae *Ruminococcus albus* SY3
Rfl-1-1	Bacteria Firmicutes Clostridia Clostridiales Ruminococcaceae *Ruminococcus flavefaciens*
Rfl-2-1	Bacteria Firmicutes Clostridia Clostridiales Ruminococcaceae *Ruminococcus flavefaciens* ATCC 19208
Rsp-2-1	Bacteria Firmicutes Clostridia Clostridiales Ruminococcaceae *Ruminococcus sp.* FC2018
Rsp-3-1	Bacteria Firmicutes Clostridia Clostridiales Ruminococcaceae *Ruminococcus sp.* NK3A76
Csp-18-1	Bacteria Firmicutes Erysipelotrichi Erysipelotrichales Erysipelotrichaceae *Clostridium spiroforme* DSM 1552
Esp-14-1	Bacteria Firmicutes Erysipelotrichia Erysipelotrichales Erysipelotrichaceae *Erysipelatoclostridium sp.* An15
Esp-15-1	Bacteria Firmicutes Erysipelotrichia Erysipelotrichales Erysipelotrichaceae *Erysipelatoclostridium sp.* An173
Msp-1-1	Bacteria Firmicutes Erysipelotrichia Erysipelotrichales Erysipelotrichaceae *Massiliomicrobiota sp.* An105

Msp-2-1	Bacteria Firmicutes Erysipelotrichia Erysipelotrichales Erysipelotrichaceae *Massiliomicrobiota sp.* An134
Msp-3-1	Bacteria Firmicutes Erysipelotrichia Erysipelotrichales Erysipelotrichaceae *Massiliomicrobiota sp.* An142
Msp-4-1	Bacteria Firmicutes Erysipelotrichia Erysipelotrichales Erysipelotrichaceae *Massiliomicrobiota sp.* An80
Mti-1-1	Bacteria Firmicutes Erysipelotrichia Erysipelotrichales Erysipelotrichaceae *Massiliomicrobiota timonensis*
Efa-300-1	Bacteria Firmicutes Lactobacillales Enterococcaceae *Enterococcus faecalis* 62
Efa-301-1	Bacteria Firmicutes Lactobacillales Enterococcaceae *Enterococcus faecalis* AR01/DG
Efa-16-1 to Efa-16-2	Bacteria Firmicutes Lactobacillales Enterococcaceae *Enterococcus faecalis* ATCC 29200
Efa-302-1	Bacteria Firmicutes Lactobacillales Enterococcaceae *Enterococcus faecalis* ATCC 4200
Efa-303-1	Bacteria Firmicutes Lactobacillales Enterococcaceae *Enterococcus faecalis* CH188
Efa-304-1	Bacteria Firmicutes Lactobacillales Enterococcaceae *Enterococcus faecalis* D32
Efa-305-1	Bacteria Firmicutes Lactobacillales Enterococcaceae *Enterococcus faecalis* D6
Efa-306-1	Bacteria Firmicutes Lactobacillales Enterococcaceae *Enterococcus faecalis* DS5
Efa-307-1	Bacteria Firmicutes Lactobacillales Enterococcaceae *Enterococcus faecalis* E1Sol
Efa-308-1	Bacteria Firmicutes Lactobacillales Enterococcaceae *Enterococcus faecalis* Fly1
Efa-309-1	Bacteria Firmicutes Lactobacillales Enterococcaceae *Enterococcus faecalis* HH22
Efa-310-1	Bacteria Firmicutes Lactobacillales Enterococcaceae *Enterococcus faecalis* HIP11704
Efa-311-1	Bacteria Firmicutes Lactobacillales Enterococcaceae *Enterococcus faecalis* JH1
Efa-312-1	Bacteria Firmicutes Lactobacillales Enterococcaceae *Enterococcus faecalis* Merz96
Efa-313-1	Bacteria Firmicutes Lactobacillales Enterococcaceae *Enterococcus faecalis* OG1RF
Efa-314-1	Bacteria Firmicutes Lactobacillales Enterococcaceae *Enterococcus faecalis* PC1.1
Efa-315-1	Bacteria Firmicutes Lactobacillales Enterococcaceae *Enterococcus faecalis* R712
Efa-316-1	Bacteria Firmicutes Lactobacillales Enterococcaceae *Enterococcus faecalis* S613
Efa-317-1	Bacteria Firmicutes Lactobacillales Enterococcaceae *Enterococcus faecalis* T1
Efa-318-1	Bacteria Firmicutes Lactobacillales Enterococcaceae *Enterococcus faecalis* T11
Efa-319-1	Bacteria Firmicutes Lactobacillales Enterococcaceae *Enterococcus faecalis* T2
Efa-320-1	Bacteria Firmicutes Lactobacillales Enterococcaceae *Enterococcus faecalis* T3
Efa-321-1	Bacteria Firmicutes Lactobacillales Enterococcaceae *Enterococcus faecalis* T8
Efa-322-1	Bacteria Firmicutes Lactobacillales Enterococcaceae *Enterococcus faecalis* TX0104
Efa-323-1	Bacteria Firmicutes Lactobacillales Enterococcaceae *Enterococcus faecalis* TX0855
Efa-324-1	Bacteria Firmicutes Lactobacillales Enterococcaceae *Enterococcus faecalis* TX1322
Efa-325-1	Bacteria Firmicutes Lactobacillales Enterococcaceae *Enterococcus faecalis* TX2134
Efa-326-1	Bacteria Firmicutes Lactobacillales Enterococcaceae *Enterococcus faecalis* TX4248
Efa-291-1 to Efa-291-3	Bacteria Firmicutes Lactobacillales Enterococcaceae *Enterococcus faecalis* V583
Efa-327-1	Bacteria Firmicutes Lactobacillales Enterococcaceae *Enterococcus faecalis* X98
Ega-2-1	Bacteria Firmicutes Lactobacillales Enterococcaceae *Enterococcus gallinarum* EG2
Lbr-2-1	Bacteria Firmicutes Lactobacillales Lactobacillaceae *Lactobacillus brevis subsp. gravesensis* ATCC 27305
Lco-4-1	Bacteria Firmicutes Lactobacillales Lactobacillaceae *Lactobacillus coryniformis subsp. torquens* KCTC 3535
Lhe-3-1	Bacteria Firmicutes Lactobacillales Lactobacillaceae *Lactobacillus helveticus* DPC 4571
Lhi-2-1	Bacteria Firmicutes Lactobacillales Lactobacillaceae *Lactobacillus hilgardii* ATCC 8290
Lul-1-1 to Lul-1-2	Bacteria Firmicutes Lactobacillales Lactobacillaceae *Lactobacillus ultunensis* DSM 16047
Lve-2-1	Bacteria Firmicutes Lactobacillales Lactobacillaceae *Lactobacillus versmoldensis* KCTC 3814
Pac-2-1	Bacteria Firmicutes Lactobacillales Lactobacillaceae *Pediococcus acidilactici* DSM 20284
Lfa-3-1	Bacteria Firmicutes Lactobacillales *Leuconostoc fallax* KCTC 3537
Lla-18-1	Bacteria Firmicutes Lactobacillales Streptococcaceae *Lactococcus lactis subsp. cremoris* A76
Lla-19-1	Bacteria Firmicutes Lactobacillales Streptococcaceae *Lactococcus lactis subsp. cremoris* MG1363
Lla-20-1	Bacteria Firmicutes Lactobacillales Streptococcaceae *Lactococcus lactis subsp. cremoris* NZ9000
Lla-21-1	Bacteria Firmicutes Lactobacillales Streptococcaceae *Lactococcus lactis subsp. cremoris* SK11
Ali-1-1	Bacteria Firmicutes Negativicutes Selenomonadales Selenomonadaceae *Anaerovibrio lipolyticus* LB2005
Sru-1-1	Bacteria Firmicutes Negativicutes Selenomonadales Selenomonadaceae *Selenomonas ruminantium*
Pvi-1-1	Bacteria Firmicutes Negativicutes Selenomonadales Sporomusaceae *Propionispora vibrioides*
Msp-5-1	Bacteria Firmicutes Negativicutes Veillonellales Veillonellaceae *Megasphaera sp.* MJR8396C
Csp-19-1	Bacteria Fusobacteria Fusobacteriales Fusobacteriaceae *Cetobacterium sp.* ZOR0034
Fnu-1-1 to Fnu-1-3	Bacteria Fusobacteria Fusobacteriales Fusobacteriaceae *Fusobacterium nucleatum subsp. polymorphum*
Fsp-2-1	Bacteria Fusobacteria Fusobacteriales Fusobacteriaceae *Fusobacterium sp.* HMSC064B11
Fsp-3-1	Bacteria Fusobacteria Fusobacteriales Fusobacteriaceae *Fusobacterium sp.* HMSC073F01
Fva-1-1	Bacteria Fusobacteria Fusobacteriales Fusobacteriaceae *Fusobacterium varium* ATCC 27725
Bba-1-1	Bacteria Proteobacteria Betaproteobacteria *Burkholderiales bacterium* 1_1_47
Pex-1-1	Bacteria Proteobacteria Betaproteobacteria Burkholderiales Sutterellaceae *Parasutterella excrementihominis* YIT 11859
Bpi-1-1	Bacteria Spirochaetes Spirochaetales Brachyspiraceae *Brachyspira pilosicoli* P43/6/78
Csp-20-1	Bacteria Synergistetes Synergistia Synergistales Synergistaceae *Cloacibacillus sp.* An23
Ssp-2-1	Bacteria Synergistetes Synergistia Synergistales Synergistaceae *Synergistes sp.* 3_1_syn1
env-1 to env-1908	environmental samples

1.2 Notes

Superscript numbers are used to annotate any GGAM-1-recreated RNA that has special characteristics (described below). These numeric annotations will be used in Sections 1.3 and 1.6:. The meaning of the numbers are as follows.

[1] Upstream of sugE in Actinobacteria

1.3 Gene contexts

Each GGAM-1-recreated RNA (indicated by "RNA→") is listed. For each hit, the downstream genes predicted to reside in a regulated operon are listed. If the nearest downstream gene is encoding in the opposite strand (and therefore presumed to not be a part of a regulated operon), then that gene is still depicted. Some environmental sequences and some RefSeq entries lack gene annotations, and so no genes are available for such sequences. The direction of each gene is indicated with an arrow (→), and each predicted conserved domain in the gene is named. Conserved domains associated with more than one GGAM-1-recreated RNA are assigned a color; other domains are gray. Information about these conserved domains is given in Section 1.4. The accession of the sequence containing each GGAM-1-recreated RNA is given in the column named "Seq. accession". Accessions beginning with "NC_", "NS_", "NW_" or "NZ_" are contained in RefSeq. Other accession refer to environmental samples. Nucleotide coordinates are given for the 5′ and 3′ boundaries of each GGAM-1-recreated RNA. If the 5′ coordinate is greater than the 3′ coordinate, the RNA is present on the reverse-complement strand of the containing genomic DNA sequence. Each hit is denoted by an abbreviation (like "Eco-1-1") that refers to a taxonomy given in Section 1.1. (Superscript numbers refer to annotations listed in Section 1.2)

abbrev.	Seq. accession		5′ at	3′ at	genes
env-1	Ga0256832_1061013	-	1859	1760	**RNA**→ ←near_KaiC_dom (TIGR03879)
Csp-12-1	NZ_AUUU01000128.1	-	10923	10821	**RNA**→ P-II (smart00938)P-II (pfam00543)→ P-II (smart00938)GlnK (COG0347)→<br>SsuA_fam (TIGR01728)PBP2_SsuA_like_6 (cd13563)→ PRK10160 (PRK10160)TauC (COG0600)→<br>ABC_NrtD_SsuB_transporters (cd03293)AAA (smart00382)→ hypo→ hypo→<br>YcgI (COG3665)DUF1989 (pfam09347)→ YcgI (COG3665)DUF1989 (pfam09347)→<br>biotinyl_domain (cd06850)AHS1 (smart00796)AHS2 (smart00797)Biotin_carb_C (smart00878)AccC (COG0439)DU<br>GatA (COG0154)PRK08186 (PRK08186)→
env-2	HBC_scaff_10333492	-	7527	7439	**RNA**→ B3/B4 (COG3382)→
env-3	HBC_ctgs_1000109	-	7527	7439	**RNA**→ B3/B4 (COG3382)→
env-4	Pasolli2019-15408-45	+	23525	23615	**RNA**→ hypo→
Lga-2-1	NZ_CCXC01000001.1	+	541647	541745	**RNA**→ PRK11431 (PRK11431)EmrE (COG2076)→
Lga-2-2	NZ_JPUJ01000001.1	+	35693	35791	**RNA**→ PRK11431 (PRK11431)EmrE (COG2076)→
Lga-2-3	NZ_LXWL01000001.1	-	596817	596719	**RNA**→ PRK11431 (PRK11431)EmrE (COG2076)→
Lga-3-1	NZ_AFCD01000049.1	+	7317	7415	**RNA**→ PRK11431 (PRK11431)EmrE (COG2076)→
Lga-6-1	NZ_AGQX01000036.1	+	3929	4027	**RNA**→ PRK11431 (PRK11431)EmrE (COG2076)→
env-5	CXUD01001568.1	-	181	83	**RNA**→ ←hypo
env-6	CXVH01003549.1	-	158	60	**RNA**→ hypo→
env-7	DKXK01000007.1	+	26774	26872	**RNA**→ PRK11431 (PRK11431)EmrE (COG2076)→ hypo→
env-8	DKTW01000056.1	+	3313	3411	**RNA**→ PRK11431 (PRK11431)EmrE (COG2076)→
env-9	DKZV01000044.1	-	1630	1532	**RNA**→ PRK11431 (PRK11431)EmrE (COG2076)→ hypo→
env-10	DKWH01000077.1	+	26818	26916	**RNA**→ PRK11431 (PRK11431)EmrE (COG2076)→ hypo→
env-11	DMSZ01000121.1	-	1500	1402	**RNA**→ PRK11431 (PRK11431)EmrE (COG2076)→ hypo→
Lga-4-1	NZ_AMQS01000012.1	+	49893	49992	**RNA**→ PRK11431 (PRK11431)EmrE (COG2076)→
env-12	Ga0255059_10409016	-	350	240	**RNA**→ hypo→
env-13	Ga0209075_1008271	+	4662	4760	**RNA**→ PRK11431 (PRK11431)EmrE (COG2076)→
env-14	Ga0209075_1148330	-	529	431	**RNA**→ PRK11431 (PRK11431)EmrE (COG2076)→

env-15	UnmappedStool_Broad_scaffold_1128024	-	3318	3216	RNA→ P-II (smart00938)GlnK (COG0347)→ P-II (smart00938)GlnK (COG0347)→<br>PRK10160 (PRK10160)TauC (COG0600)→ ABC_NrtD_SsuB_transporters (cd03293)AAA (smart00382)→
env-16	Pasolli2019-5182-16	+	14806	14908	RNA→ GlnK (COG0347)P-II (smart00938)→ GlnK (COG0347)P-II (smart00938)→<br>TauC (COG0600)ntrB (TIGR01183)→ ABC_NrtD_SsuB_transporters (cd03293)AAA (smart00382)→
[sc-1-1	NZ_BAHS01000006.1	-	16738	16641	RNA→ vmrA (PRK09575)MATE_MepA_like (cd13143)→ hypo→<br>LytTR (smart00850)REC (smart00448)LytT (COG3279)→<br>BaeS (COG0642)YlqD (pfam11068)HATPase_c_5 (pfam14501)→
Cba-5-1	NZ_BAII02000020.1	-	328750	328653	RNA→ vmrA (PRK09575)MATE_MepA_like (cd13143)→ hypo→<br>LytTR (smart00850)REC (smart00448)LytT (COG3279)→<br>BaeS (COG0642)YlqD (pfam11068)HATPase_c_5 (pfam14501)→
env-17	longitudinal_461_92	-	113238	113141	RNA→ vmrA (PRK09575)MATE_MepA_like (cd13143)→
env-18	longitudinal_462_39	-	113257	113160	RNA→ vmrA (PRK09575)MATE_MepA_like (cd13143)→
env-19	longitudinal_463_42	+	128356	128453	RNA→ vmrA (PRK09575)MATE_MepA_like (cd13143)→
env-20	Pasolli2019-4630-5	+	56227	56324	RNA→ MATE_MepA_like (cd13143)matE (TIGR00797)→<br>LytT (COG3279)LytTR (smart00850)REC (smart00448)→<br>BaeS (COG0642)HATPase_c_5 (pfam14501)YlqD (pfam11068)→
env-21	Ga0169849_119769	+	515	612	RNA→
env-22	longitudinal_522_2334	+	607	704	RNA→ NorM (COG0534)vmrA (PRK09575)→
env-23	Ga0209064_1084087	-	570	471	RNA→ vmrA (PRK09575)MATE_MepA_like (cd13143)→
env-24	AUXO014189945.1	+	263	362	RNA→
env-25	Ga0129306_1001075	+	24863	24961	RNA→ hypo→
env-26	Ga0129306_1000008	-	274443	274344	RNA→ hypo→
env-27	Ga0129307_1000435	-	10252	10153	RNA→ hypo→
Lbr-1-1	NZ_AYZQ01000002.1	+	303894	303984	RNA→ PRK11431 (PRK11431)EmrE (COG2076)→
Lsa-3-1	NZ_BBBX01000003.1	-	102	11	RNA→
env-28	HBC_ctgs_1025501	-	653	561	RNA→ PRK11431 (PRK11431)EmrE (COG2076)→
env-29	HBC_scaff_10321086	-	653	561	RNA→ PRK11431 (PRK11431)EmrE (COG2076)→
Wbo-1-1	NZ_FMAO01000002.1	+	44769	44861	RNA→ PRK11431 (PRK11431)EmrE (COG2076)→ Acetyltransf_1 (pfam00583)Acetyltransf_7 (pfam13508)→
Wjo-1-1	NZ_CP014332.1	-	1729911	1729818	RNA→ PRK11431 (PRK11431)EmrE (COG2076)→
env-30	Ga0121203_105175	-	794	699	RNA→ vmrA (PRK09575)MATE_MepA_like (cd13143)→
Lag-1-1	NZ_CP016766.1	-	79479	79381	RNA→ hypo→
Lag-2-1	NZ_AYYP01000018.1	+	3781	3879	RNA→ PRK11431 (PRK11431)EmrE (COG2076)→
env-31	Ga0256404_1193171	-	845	745	RNA→ ←hypo
Lpl-1-1	NZ_CP017374.1	+	811587	811675	RNA→ PRK11431 (PRK11431)EmrE (COG2076)→
Lcu-1-1	NZ_CP018906.1	+	49365	49466	RNA→ PRK11431 (PRK11431)EmrE (COG2076)→ Acetyltransf_1 (pfam00583)Acetyltransf_7 (pfam13508)→
Lki-1-1	NZ_AZCX01000005.1	-	93779	93685	RNA→ PRK11431 (PRK11431)EmrE (COG2076)→
env-32	Pasolli2019-14221-99	+	3767	3861	RNA→ hypo→
Csp-20-1	NZ_NFJQ01000003.1	-	253664	253571	RNA→ hypo→
env-33	Ga0134406_1031497	+	88	180	RNA→ hypo→
env-34	Ga0172382_11199899	-	387	288	RNA→ MATE_MepA_like (cd13143)→
env-35	Ga0209064_1041410	-	714	614	RNA→ NorM (COG0534)vmrA (PRK09575)→
env-36	UMGS2022-6	-	32709	32614	RNA→ ←Acetyltransf_1 (pfam00583)Acetyltransf_7 (pfam13508)
env-37	Ga0134538_1089920	-	745	645	RNA→ NorM (COG0534)vmrA (PRK09575)→
env-38	Ga0134540_1296937	+	86	186	RNA→ MATE_MepA_like (cd13143)→
env-39	Pasolli2019-4612-48	-	7937	7833	RNA→ MATE_MepA_like (cd13143)Polysacc_synt_C (pfam14667)→
Esp-14-1	NZ_NFLA01000008.1	+	49753	49842	RNA→ vmrA (PRK09575)MATE_MepA_like (cd13143)→
Esp-15-1	NZ_NFKR01000006.1	+	41580	41669	RNA→ vmrA (PRK09575)MATE_MepA_like (cd13143)→
env-40	Pasolli2019-6746-200	-	2944	2855	RNA→ MATE_MepA_like (cd13143)matE (TIGR00797)→
env-41	OGEO01006570.1	-	2715	2624	RNA→ hypo→
env-42	OGFC01008588.1	+	2195	2286	RNA→
env-43	OGGD01053956.1	+	439	530	RNA→
env-44	OGLG01031864.1	-	219	128	RNA→
env-45	OLGD01002420.1	-	220	129	RNA→

env-46	Ga0134390_162314	-	322	231	**RNA**→ PRK11431 (PRK11431)EmrE (COG2076)→
env-47	scaffold18848_1_V1.CD-11	-	188	97	**RNA**→
env-48	DLM004_scaffold4459_8	+	538	629	**RNA**→ PRK11431 (PRK11431)EmrE (COG2076)→
env-49	DOF003_scaffold34426_6	-	597	506	**RNA**→ PRK11431 (PRK11431)EmrE (COG2076)→
env-50	OGDU01000171.1	+	5544	5635	**RNA**→ PRK11431 (PRK11431)EmrE (COG2076)→
env-51	OGER01020816.1	+	586	677	**RNA**→ PRK11431 (PRK11431)EmrE (COG2076)→
env-52	OGFL01001955.1	+	4719	4810	**RNA**→ PRK11431 (PRK11431)EmrE (COG2076)→
env-53	OGGQ01003612.1	+	4719	4810	**RNA**→ PRK11431 (PRK11431)EmrE (COG2076)→
env-54	OGHH01000922.1	+	25584	25675	**RNA**→ PRK11431 (PRK11431)EmrE (COG2076)→
env-55	OGHO01001893.1	-	13380	13289	**RNA**→ PRK11431 (PRK11431)EmrE (COG2076)→
env-56	OGHU01006248.1	+	3842	3933	**RNA**→ PRK11431 (PRK11431)EmrE (COG2076)→
env-57	OGIE01004563.1	-	8099	8008	**RNA**→ PRK11431 (PRK11431)EmrE (COG2076)→
env-58	OGIR01037761.1	-	470	379	**RNA**→ PRK11431 (PRK11431)EmrE (COG2076)→
env-59	OGJO01007888.1	-	665	574	**RNA**→ PRK11431 (PRK11431)EmrE (COG2076)→
env-60	OGKA01001763.1	+	15620	15711	**RNA**→ PRK11431 (PRK11431)EmrE (COG2076)→
env-61	OGKG01000261.1	+	4720	4811	**RNA**→ PRK11431 (PRK11431)EmrE (COG2076)→
env-62	OGLE01028308.1	-	739	648	**RNA**→ PRK11431 (PRK11431)EmrE (COG2076)→
env-63	OGLM01005782.1	-	3464	3373	**RNA**→ PRK11431 (PRK11431)EmrE (COG2076)→
env-64	OGNB01061313.1	+	165	256	**RNA**→ PRK11431 (PRK11431)EmrE (COG2076)→
env-65	PPYF01016692.1	+	185981	186072	**RNA**→ PRK11431 (PRK11431)EmrE (COG2076)→
env-66	scaffold54377_2_MH0085	-	342	251	**RNA**→ PRK11431 (PRK11431)EmrE (COG2076)→
env-67	C4928331_1_MH0086	+	2147	2238	**RNA**→ PRK11431 (PRK11431)EmrE (COG2076)→
env-68	SRS014459_C2656015	-	572	481	**RNA**→ PRK11431 (PRK11431)EmrE (COG2076)→
env-69	SRS015065_C2424287	-	572	481	**RNA**→ PRK11431 (PRK11431)EmrE (COG2076)→
env-70	SRS016267_WUGC_scaffold_26797	-	7529	7438	**RNA**→ PRK11431 (PRK11431)EmrE (COG2076)→
env-71	SRS019787_C3156274	-	637	546	**RNA**→ PRK11431 (PRK11431)EmrE (COG2076)→
Pex-1-1	NZ_GL883730.1	+	9649	9740	**RNA**→ PRK11431 (PRK11431)EmrE (COG2076)→
env-72	NOF012_scaffold58100_3	+	228	319	**RNA**→ ←hypo
env-73	OGEU01031292.1	-	1080	989	**RNA**→ hypo→
env-74	OGFF01002836.1	+	4824	4915	**RNA**→ PRK11431 (PRK11431)EmrE (COG2076)→
env-75	OGIV01059583.1	+	144	235	**RNA**→ hypo→
env-76	OIXH01075321.1	-	676	585	**RNA**→ PBP2_ProX_like (cd13606)→
env-77	OLGH01052781.1	+	582	673	**RNA**→
env-78	Ga0134492_1004968	-	9197	9106	**RNA**→ hypo→
env-79	SRS022071_C3047073	-	2078	1987	**RNA**→ PRK11431 (PRK11431)EmrE (COG2076)→
env-80	SRS075398_C2060759	+	965	1056	**RNA**→ PRK11431 (PRK11431)EmrE (COG2076)→
env-81	UnmappedStool_Broad_scaffold_557859	+	358	449	**RNA**→ PRK11431 (PRK11431)EmrE (COG2076)→
env-82	DLM009_scaffold8718_2	+	238	329	**RNA**→ PRK11431 (PRK11431)EmrE (COG2076)→
env-83	DOM016_scaffold1863_15	-	608	517	**RNA**→ PRK11431 (PRK11431)EmrE (COG2076)→
env-84	DQBB01000123.1	+	1491	1582	**RNA**→ PRK11431 (PRK11431)EmrE (COG2076)→
env-85	OGEB01010233.1	-	762	671	**RNA**→ PRK11431 (PRK11431)EmrE (COG2076)→
env-86	OGFD01014781.1	-	952	861	**RNA**→ PRK11431 (PRK11431)EmrE (COG2076)→
env-87	OGFO01000527.1	-	4454	4363	**RNA**→ PRK11431 (PRK11431)EmrE (COG2076)→
env-88	OGFW01007661.1	-	3272	3181	**RNA**→ PRK11431 (PRK11431)EmrE (COG2076)→
env-89	OGJK01007074.1	-	4144	4053	**RNA**→ PRK11431 (PRK11431)EmrE (COG2076)→
env-90	OGKZ01013169.1	+	1770	1861	**RNA**→ PRK11431 (PRK11431)EmrE (COG2076)→
env-91	OGLC01000729.1	-	21207	21116	**RNA**→ PRK11431 (PRK11431)EmrE (COG2076)→
env-92	OGUQ01002428.1	+	4733	4824	**RNA**→ PRK11431 (PRK11431)EmrE (COG2076)→
env-93	PPYE01046197.1	+	13723	13814	**RNA**→ PRK11431 (PRK11431)EmrE (COG2076)→
env-94	Pasolli2019-9262-2	+	58808	58899	**RNA**→ EmrE (COG2076)PRK11431 (PRK11431)→
env-95	scaffold23194_10_MH0045	-	758	667	**RNA**→ PRK11431 (PRK11431)EmrE (COG2076)→
env-96	SRS011061_C4867193	+	700	791	**RNA**→ PRK11431 (PRK11431)EmrE (COG2076)→

env-97	SRS012902_Baylor_scaffold_6068	-	19919	19828	**RNA**→ PRK11431 (PRK11431)EmrE (COG2076)→
env-98	SRS015578_WUGC_scaffold_29493	-	85060	84969	**RNA**→ PRK11431 (PRK11431)EmrE (COG2076)→
env-99	SRS015794_C2283587	-	639	548	**RNA**→ PRK11431 (PRK11431)EmrE (COG2076)→
env-100	SRS018351_Baylor_scaffold_31337	-	572	481	**RNA**→ PRK11431 (PRK11431)EmrE (COG2076)→
env-101	SRS019030_Baylor_scaffold_39277	+	929	1020	**RNA**→ PRK11431 (PRK11431)EmrE (COG2076)→
env-102	SRS019267_C3616456	+	954	1045	**RNA**→ PRK11431 (PRK11431)EmrE (COG2076)→
env-103	SRS022609_C3299778	+	587	678	**RNA**→ PRK11431 (PRK11431)EmrE (COG2076)→
Bba-1-1	NZ_GL383995.1	-	218123	218032	**RNA**→ PRK11431 (PRK11431)EmrE (COG2076)→ PRK11431 (PRK11431)EmrE (COG2076)→
env-104	OGDQ01019158.1	+	249	340	**RNA**→ PRK11431 (PRK11431)EmrE (COG2076)→
env-105	OGEO01010808.1	-	1712	1621	**RNA**→ PRK11431 (PRK11431)EmrE (COG2076)→
env-106	PPYE01012634.1	+	236	327	**RNA**→ PRK11431 (PRK11431)EmrE (COG2076)→
env-107	SRS011271_C3173797	+	989	1080	**RNA**→ PRK11431 (PRK11431)EmrE (COG2076)→
env-108	SRS023526_Baylor_scaffold_12981	+	3355	3446	**RNA**→ PRK11431 (PRK11431)EmrE (COG2076)→
env-109	SRS055982_C1590162	-	296	205	**RNA**→ PRK11431 (PRK11431)EmrE (COG2076)→
env-110	DQDL01000114.1	-	2925	2826	**RNA**→ PnuC (COG3201)NMN_trans_PnuC (TIGR01528)→ hypo→
					HTH_XRE (smart00530)HTH_XRE (cd00093)Zn_ribbon_2 (pfam12674)→
env-111	OIYS01019013.1	-	1297	1198	**RNA**→ PnuC (COG3201)NMN_trans_PnuC (TIGR01528)→ hypo→ hypo→
env-112	Pasolli2019-4268-276	-	1169	1070	**RNA**→ PnuC (COG3201)NMN_trans_PnuC (TIGR01528)→ HMA (cd00371)TIGR00003 (TIGR00003)→
env-113	Ga0209487_1004777	-	10067	9964	**RNA**→ NorM (COG0534)vmrA (PRK09575)→
env-114	AUXO015540661.1	-	27212	27118	**RNA**→ PRK11431 (PRK11431)EmrE (COG2076)→ YncA (COG1247)→
					MhpC (COG0596)Abhydrolase_6 (pfam12697)→ FruK_PfkB_like (cd01164)pfkB (TIGR03828)→
env-115	DGUK01000043.1	+	2475	2569	**RNA**→ PRK11431 (PRK11431)EmrE (COG2076)→ hypo→ hypo→
					MhpC (COG0596)Abhydrolase_6 (pfam12697)→ FruK_PfkB_like (cd01164)pfkB (TIGR03828)→
Rpe-1-1	NZ_JTGN01000008.1	+	9384	9489	**RNA**→ NorM (COG0534)vmrA (PRK09575)→
env-116	UMGS271-2	-	267589	267484	**RNA**→ NorM (COG0534)matE (TIGR00797)→
Rsp-1-1	NZ_JMLZ01000002.1	+	3479785	3479890	**RNA**→ NorM (COG0534)vmrA (PRK09575)→
Lsp-2-1	NZ_PQGL01000001.1	-	69521	69424	**RNA**→ PRK11431 (PRK11431)EmrE (COG2076)→
Lse-1-1	NZ_JQBW01000010.1	-	80108	80007	**RNA**→ ThrA (COG0460)PRK06349 (PRK06349)→
env-117	Ga0213827_1054387	+	201	298	**RNA**→ NorM (COG0534)vmrA (PRK09575)→
env-118	2205037665	-	1313	1211	**RNA**→ PRK11431 (PRK11431)EmrE (COG2076)→
env-119	2158322397	+	43	145	**RNA**→ PRK11431 (PRK11431)EmrE (COG2076)→
Loz-1-1	NZ_AYYQ01000036.1	-	116374	116282	**RNA**→ PRK11431 (PRK11431)EmrE (COG2076)→
env-120	Pasolli2019-15167-9	+	43124	43219	**RNA**→ EmrE (COG2076)PRK11431 (PRK11431)→
env-121	Pasolli2019-4678-0	-	10444	10348	**RNA**→ EmrE (COG2076)PRK11431 (PRK11431)→
env-122	Ga0129308_1005167	+	4322	4421	**RNA**→ NorM (COG0534)vmrA (PRK09575)→
Bpu-1-1	NZ_NFKK01000009.1	+	52063	52163	**RNA**→ vmrA (PRK09575)MATE_MepA_like (cd13143)→
env-123	DHKC01000002.1	-	2034	1934	**RNA**→ PRK11431 (PRK11431)EmrE (COG2076)→
env-124	DJEZ01000010.1	+	79622	79722	**RNA**→ PRK11431 (PRK11431)EmrE (COG2076)→
env-125	DJMZ01000096.1	-	11913	11813	**RNA**→ PRK11431 (PRK11431)EmrE (COG2076)→
env-126	DJPN01000040.1	-	12690	12590	**RNA**→ PRK11431 (PRK11431)EmrE (COG2076)→
env-127	DJSM01000087.1	-	7880	7780	**RNA**→ PRK11431 (PRK11431)EmrE (COG2076)→
env-128	DOEW01000066.1	+	4147	4247	**RNA**→ PRK11431 (PRK11431)EmrE (COG2076)→
env-129	DEDN01000004.1	-	27089	26990	**RNA**→ PRK11431 (PRK11431)EmrE (COG2076)→
env-130	Pasolli2019-9310-14	+	34573	34672	**RNA**→ EmrE (COG2076)PRK11431 (PRK11431)→
env-131	Ga0180008_1869770	+	75	178	**RNA**→
env-132	Ga0180007_10013520	-	456	353	**RNA**→ ←InsQ (COG0675)
Ali-1-1	NZ_JHYA01000006.1	-	5199	5110	**RNA**→ PRK11431 (PRK11431)EmrE (COG2076)→ Pyridox_oxidase (pfam01243)Pyridox_ox_2 (pfam12900)→
env-133	Ga0256405_10081441	-	851	762	**RNA**→ PRK11431 (PRK11431)EmrE (COG2076)→ Pyridox_oxidase (pfam01243)Pyridox_ox_2 (pfam12900)→
env-134	2158075835	-	165	72	**RNA**→ hypo→
Lin-1-1	NZ_AZGN01000003.1	+	225919	226012	**RNA**→ PRK11431 (PRK11431)EmrE (COG2076)→
Ljo-2-1	NC_022909.1	-	1477909	1477816	**RNA**→ hypo→ hypo→ hypo→
Ljo-1-1	NZ_NGOI01000008.1	+	17527	17620	**RNA**→ hypo→
Ljo-1-2	NZ_NGOH01000036.1	+	22377	22470	**RNA**→ hypo→

Ljo-1-3	NZ_NGOF01000001.1	+	86790	86883	**RNA**→ hypo→
Ljo-1-4	NZ_NGOG01000088.1	+	85702	85795	**RNA**→ hypo→
Ljo-1-5	NZ_NGOE01000010.1	+	17659	17752	**RNA**→ hypo→
Ljo-1-6	NZ_NGOD01000001.1	+	85180	85273	**RNA**→ hypo→
env-135	PPYF01000042.1	+	1032	1125	**RNA**→ hypo→ FolA (COG0262)→
env-136	OMWG01000025.1	-	19753	19658	**RNA**→ NorM (COG0534)matE (TIGR00797)→
env-137	OMZH01000025.1	-	7748	7653	**RNA**→ NorM (COG0534)matE (TIGR00797)→
env-138	Ga0051080_1004551	+	1268	1363	**RNA**→ PRK11431 (PRK11431)EmrE (COG2076)→
env-139	ADJS01004551.1	+	1268	1363	**RNA**→ PRK11431 (PRK11431)EmrE (COG2076)→
Fpl-1-1	NZ_JH417696.1	+	26681	26779	**RNA**→ NorM (COG0534)Polysacc_synt_C (pfam14667)→
Fsp-1-1	NZ_NFHC01000001.1	-	232202	232103	**RNA**→ NorM (COG0534)vmrA (PRK09575)→
env-140	Pasolli2019-15136-3	+	45724	45823	**RNA**→ NorM (COG0534)matE (TIGR00797)→
env-141	DHRS01000014.1	-	350866	350766	**RNA**→ PRK11431 (PRK11431)EmrE (COG2076)→
env-142	Ga0122811_100842	+	2599	2699	**RNA**→ PRK11431 (PRK11431)EmrE (COG2076)→
env-143	Ga0121633_111989	-	385	285	**RNA**→ PRK11431 (PRK11431)EmrE (COG2076)→
Lla-20-1	NC_017949.1	+	126527	126628	**RNA**→ PRK11431 (PRK11431)EmrE (COG2076)→
Lla-19-1	NC_009004.1	+	126527	126628	**RNA**→ PRK11431 (PRK11431)EmrE (COG2076)→
Lla-4-1	NZ_AZSI01000204.1	+	78341	78442	**RNA**→ PRK11431 (PRK11431)EmrE (COG2076)→
Lla-21-1	NC_008527.1	+	103434	103535	**RNA**→ PRK11431 (PRK11431)EmrE (COG2076)→
Lla-18-1	NC_017492.1	+	151017	151118	**RNA**→ PRK11431 (PRK11431)EmrE (COG2076)→
Lla-9-1	NC_019435.1	+	109854	109955	**RNA**→ PRK11431 (PRK11431)EmrE (COG2076)→
Lla-7-1	NC_022369.1	+	102760	102861	**RNA**→ PRK11431 (PRK11431)EmrE (COG2076)→
Lla-3-1	NZ_CP015907.1	+	98338	98439	**RNA**→ PRK11431 (PRK11431)EmrE (COG2076)→
Lla-3-2	NZ_CP015894.1	+	109856	109957	**RNA**→ PRK11431 (PRK11431)EmrE (COG2076)→
Lla-6-1	NZ_CM007353.1	+	102739	102840	**RNA**→ PRK11431 (PRK11431)EmrE (COG2076)→
Lla-3-3	NZ_CP015899.1	+	103209	103310	**RNA**→ PRK11431 (PRK11431)EmrE (COG2076)→
Lla-3-4	NZ_CP015900.1	+	137505	137606	**RNA**→ PRK11431 (PRK11431)EmrE (COG2076)→
Lla-3-5	NZ_CP015901.1	+	117426	117527	**RNA**→ PRK11431 (PRK11431)EmrE (COG2076)→
Lla-3-6	NZ_CP015909.1	+	104250	104351	**RNA**→ PRK11431 (PRK11431)EmrE (COG2076)→
Lla-5-1	NZ_JAUH01000101.1	+	3909	4010	**RNA**→ PRK11431 (PRK11431)EmrE (COG2076)→
Lla-8-1	NZ_BCVK01000019.1	-	2046	1945	**RNA**→ PRK11431 (PRK11431)EmrE (COG2076)→
Lla-1-1	NZ_LXWJ01000003.1	-	104157	104056	**RNA**→ PRK11431 (PRK11431)EmrE (COG2076)→
Lla-17-1	NZ_JXKC01000011.1	+	36689	36790	**RNA**→ PRK11431 (PRK11431)EmrE (COG2076)→
Lla-1-2	NZ_OESP01000022.1	+	36538	36639	**RNA**→ PRK11431 (PRK11431)EmrE (COG2076)→
Lla-1-3	NZ_OESD01000019.1	-	2390	2289	**RNA**→ PRK11431 (PRK11431)EmrE (COG2076)→
Lla-1-4	NZ_OESM01000013.1	-	2293	2192	**RNA**→ PRK11431 (PRK11431)EmrE (COG2076)→
Lla-1-5	NZ_OESN01000028.1	-	2286	2185	**RNA**→ PRK11431 (PRK11431)EmrE (COG2076)→
env-144	Ga0122248_100018	-	106494	106393	**RNA**→ PRK11431 (PRK11431)EmrE (COG2076)→
env-145	DCNS01000112.1	-	12539	12437	**RNA**→ PRK11431 (PRK11431)EmrE (COG2076)→
env-146	Ga0120956_100899	-	2365	2263	**RNA**→ PRK11431 (PRK11431)EmrE (COG2076)→
env-147	Ga0121497_10022	-	24854	24752	**RNA**→ PRK11431 (PRK11431)EmrE (COG2076)→
env-148	Ga0121476_100772	+	1772	1874	**RNA**→ PRK11431 (PRK11431)EmrE (COG2076)→
env-149	SRS042628_C2541337	+	114	216	**RNA**→ PRK11431 (PRK11431)EmrE (COG2076)→
Lla-12-1	NC_022593.1	+	130967	131069	**RNA**→ PRK11431 (PRK11431)EmrE (COG2076)→
Lla-10-1	NZ_CP015896.1	+	124621	124723	**RNA**→ PRK11431 (PRK11431)EmrE (COG2076)→
Lla-2-1	NZ_KV467173.1	+	68329	68431	**RNA**→ PRK11431 (PRK11431)EmrE (COG2076)→
Lla-10-2	NZ_CP015902.1	+	101618	101720	**RNA**→ PRK11431 (PRK11431)EmrE (COG2076)→
Lla-10-3	NZ_CP015906.1	+	124803	124905	**RNA**→ PRK11431 (PRK11431)EmrE (COG2076)→
Lla-15-1	NZ_ATBF01000011.1	+	30753	30855	**RNA**→ PRK11431 (PRK11431)EmrE (COG2076)→
Lla-16-1	NZ_ATBD01000155.1	+	31008	31110	**RNA**→ PRK11431 (PRK11431)EmrE (COG2076)→
Lla-14-1	NZ_AXZK01000040.1	+	31258	31360	**RNA**→ PRK11431 (PRK11431)EmrE (COG2076)→
Lla-11-1	NZ_AZQT01000020.1	+	1215	1317	**RNA**→ PRK11431 (PRK11431)EmrE (COG2076)→

Lla-13-1	NZ_LKPE01000010.1	+	68939	69041	**RNA**→ PRK11431 (PRK11431)EmrE (COG2076)→
Lla-13-2	NZ_LIWD01000027.1	-	12016	11914	**RNA**→ PRK11431 (PRK11431)EmrE (COG2076)→
Lla-1-6	NZ_NCXC01000005.1	+	47086	47188	**RNA**→ PRK11431 (PRK11431)EmrE (COG2076)→
Lla-1-7	NZ_NCWV01000005.1	+	47086	47188	**RNA**→ PRK11431 (PRK11431)EmrE (COG2076)→
Lla-1-8	NZ_OESK01000015.1	-	12944	12842	**RNA**→ PRK11431 (PRK11431)EmrE (COG2076)→
Lla-1-9	NZ_OESF01000028.1	-	14302	14200	**RNA**→ PRK11431 (PRK11431)EmrE (COG2076)→
Lla-1-10	NZ_OESH01000027.1	-	13028	12926	**RNA**→ PRK11431 (PRK11431)EmrE (COG2076)→
Lla-1-11	NZ_OESI01000023.1	-	13434	13332	**RNA**→ PRK11431 (PRK11431)EmrE (COG2076)→
env-150	Ga0256410_1039565	-	833	731	**RNA**→ PRK11431 (PRK11431)EmrE (COG2076)→
Lla-1-12	NZ_NCXG01000012.1	+	46217	46319	**RNA**→ PRK11431 (PRK11431)EmrE (COG2076)→
Lla-1-13	NZ_PDEW01000006.1	-	20610	20508	**RNA**→ PRK11431 (PRK11431)EmrE (COG2076)→
env-151	JGI990J12352_1120612	+	194	295	**RNA**→ EmrE (COG2076)→
env-152	AUXO015731993.1	+	253	354	**RNA**→ PRK11431 (PRK11431)EmrE (COG2076)→
env-153	HCF12C_16417	-	203	101	**RNA**→ hypo→
env-154	SRS057478_C1033480	+	56	152	**RNA**→ PRK11431 (PRK11431)EmrE (COG2076)→
env-155	OGDU01027475.1	+	882	978	**RNA**→ PRK11431 (PRK11431)EmrE (COG2076)→
env-156	OGGB01021667.1	+	191	287	**RNA**→ PRK11431 (PRK11431)EmrE (COG2076)→
env-157	OGGE01032672.1	+	98	194	**RNA**→ PRK11431 (PRK11431)EmrE (COG2076)→
env-158	OGGZ01012827.1	-	1432	1336	**RNA**→ PRK11431 (PRK11431)EmrE (COG2076)→
env-159	OGIA01018625.1	+	716	812	**RNA**→
env-160	OGKX01009207.1	-	2129	2033	**RNA**→ PRK11431 (PRK11431)PRK10535 (PRK10535)EmrE (COG2076)LolE (COG4591)→
env-161	OLGD01014546.1	+	1994	2090	**RNA**→ PRK11431 (PRK11431)EmrE (COG2076)→
env-162	SRS015217_WUGC_scaffold_9002	-	1096	1000	**RNA**→ PRK11431 (PRK11431)EmrE (COG2076)→
env-163	SRS051882_C2502851	-	478	382	**RNA**→ PRK11431 (PRK11431)EmrE (COG2076)→
env-164	Ga0134397_104383	-	470	374	**RNA**→ PRK11431 (PRK11431)EmrE (COG2076)→
env-165	Ga0134378_113137	+	44	140	**RNA**→ PRK11431 (PRK11431)EmrE (COG2076)→
env-166	Ga0134408_168084	+	133	229	**RNA**→
env-167	Ga0134406_1089465	+	100	196	**RNA**→
env-168	Ga0134443_1066212	-	393	297	**RNA**→ PRK11431 (PRK11431)EmrE (COG2076)→
env-169	scaffold2031_4_O2.UC-18	-	19179	19083	**RNA**→ ←PrdX_deacylase (cd04335)
env-170	SRS014459_C2387599	+	83	179	**RNA**→ PRK11431 (PRK11431)EmrE (COG2076)→
env-171	SRS058723_C6723561	-	468	372	**RNA**→ PRK11431 (PRK11431)EmrE (COG2076)→
env-172	SRS019685_C8221305	-	326	230	**RNA**→ PRK11431 (PRK11431)EmrE (COG2076)→
env-173	SRS022713_LANL_scaffold_24553	+	654	750	**RNA**→ PRK11431 (PRK11431)EmrE (COG2076)→
env-174	DLM013_scaffold33983_2	+	296	392	**RNA**→ PRK11431 (PRK11431)EmrE (COG2076)→
env-175	DOM001_scaffold16587_3	-	299	203	**RNA**→ PRK11431 (PRK11431)EmrE (COG2076)→
env-176	OGDW01018161.1	-	591	495	**RNA**→ PRK11431 (PRK11431)EmrE (COG2076)→
env-177	OGEG01030207.1	+	412	508	**RNA**→ PRK11431 (PRK11431)EmrE (COG2076)→
env-178	OGEN01001652.1	-	862	766	**RNA**→ PRK11431 (PRK11431)EmrE (COG2076)→
env-179	OGFD01010445.1	-	1116	1020	**RNA**→ PRK11431 (PRK11431)EmrE (COG2076)→
env-180	OGFY01051125.1	+	298	394	**RNA**→ PRK11431 (PRK11431)EmrE (COG2076)→
env-181	OGGU01060398.1	-	220	124	**RNA**→ PRK11431 (PRK11431)EmrE (COG2076)→
env-182	OGHF01014387.1	-	1136	1040	**RNA**→ PRK11431 (PRK11431)EmrE (COG2076)→
env-183	OGIE01001205.1	+	22373	22469	**RNA**→ PRK11431 (PRK11431)EmrE (COG2076)→
env-184	OGII01017861.1	-	823	727	**RNA**→ PRK11431 (PRK11431)EmrE (COG2076)→
env-185	OGKW01018161.1	-	591	495	**RNA**→ PRK11431 (PRK11431)EmrE (COG2076)→
env-186	OGMP01017151.1	-	1077	981	**RNA**→ PRK11431 (PRK11431)EmrE (COG2076)→
env-187	OGMW01000140.1	+	9352	9448	**RNA**→ PRK11431 (PRK11431)EmrE (COG2076)→
env-188	OGNB01034359.1	-	623	527	**RNA**→ PRK11431 (PRK11431)EmrE (COG2076)→
env-189	OGUF01010440.1	-	1198	1102	**RNA**→ PRK11431 (PRK11431)PRK10535 (PRK10535)EmrE (COG2076)LolE (COG4591)→
env-190	OIWN01003545.1	-	6749	6653	**RNA**→ PRK11431 (PRK11431)EmrE (COG2076)→
env-191	OIYD01000341.1	+	38252	38348	**RNA**→ PRK11431 (PRK11431)EmrE (COG2076)→

env-192	OIYU01004404.1	-	4042	3946	**RNA**→ PRK11431 (PRK11431)EmrE (COG2076)→
env-193	OIZB01002161.1	+	3507	3603	**RNA**→ PRK11431 (PRK11431)EmrE (COG2076)→
env-194	OLGF01005391.1	-	750	654	**RNA**→ PRK11431 (PRK11431)EmrE (COG2076)→
env-195	OLGL01007757.1	-	1457	1361	**RNA**→ PRK11431 (PRK11431)EmrE (COG2076)→
env-196	PPYF01037549.1	+	277	373	**RNA**→ PRK11431 (PRK11431)EmrE (COG2076)→
env-197	PPYF01097721.1	+	473	569	**RNA**→ PRK11431 (PRK11431)EmrE (COG2076)→
env-198	Ga0169873_101072	-	14134	14038	**RNA**→ PRK11431 (PRK11431)EmrE (COG2076)→
env-199	Ga0134391_112035	+	612	708	**RNA**→ PRK11431 (PRK11431)EmrE (COG2076)→
env-200	Ga0134410_103640	-	2312	2216	**RNA**→ PRK11431 (PRK11431)EmrE (COG2076)→
env-201	Ga0134459_111156	+	399	495	**RNA**→ PRK11431 (PRK11431)EmrE (COG2076)→
env-202	Ga0134379_103007	+	663	759	**RNA**→ PRK11431 (PRK11431)EmrE (COG2076)→
env-203	Ga0134450_118186	-	442	346	**RNA**→ PRK11431 (PRK11431)EmrE (COG2076)→
env-204	Ga0134417_100094	+	3490	3586	**RNA**→ PRK11431 (PRK11431)EmrE (COG2076)→
env-205	Ga0134394_118964	+	117	213	**RNA**→ PRK11431 (PRK11431)EmrE (COG2076)→
env-206	Ga0134418_1016814	+	748	844	**RNA**→ PRK11431 (PRK11431)EmrE (COG2076)→
env-207	Ga0134400_1003676	+	1172	1268	**RNA**→ PRK11431 (PRK11431)EmrE (COG2076)→
env-208	Ga0134381_1000046	-	22314	22218	**RNA**→ PRK11431 (PRK11431)EmrE (COG2076)→
env-209	Ga0134407_1003612	+	3774	3870	**RNA**→ PRK11431 (PRK11431)EmrE (COG2076)→
env-210	Ga0134414_1028044	+	358	454	**RNA**→ PRK11431 (PRK11431)EmrE (COG2076)→
env-211	Ga0134377_1007574	-	625	529	**RNA**→ PRK11431 (PRK11431)EmrE (COG2076)→
env-212	Ga0134371_1016250	+	162	258	**RNA**→ PRK11431 (PRK11431)EmrE (COG2076)→
env-213	Ga0134388_1000007	-	317082	316986	**RNA**→ PRK11431 (PRK11431)EmrE (COG2076)→
env-214	Ga0134444_101164	-	3810	3714	**RNA**→ PRK11431 (PRK11431)EmrE (COG2076)→
env-215	Ga0169849_100183	+	9718	9814	**RNA**→ PRK11431 (PRK11431)EmrE (COG2076)→
env-216	Ga0134385_115418	+	530	626	**RNA**→ PRK11431 (PRK11431)EmrE (COG2076)→
env-217	Ga0134440_119896	-	503	407	**RNA**→ PRK11431 (PRK11431)EmrE (COG2076)→
env-218	Ga0134442_1001066	-	9181	9085	**RNA**→ PRK11431 (PRK11431)EmrE (COG2076)→
env-219	Pasolli2019-15078-40	+	14302	14398	**RNA**→ EmrE (COG2076)PRK11431 (PRK11431)→
env-220	scaffold20031_1_V1.CD-9	+	356	452	**RNA**→ PRK11431 (PRK11431)EmrE (COG2076)→
env-221	SRS016018_WUGC_scaffold_17878	+	159	255	**RNA**→ PRK11431 (PRK11431)EmrE (COG2076)→
env-222	SRS017247_C1012204	-	458	362	**RNA**→ PRK11431 (PRK11431)EmrE (COG2076)→
env-223	SRS017521_Baylor_scaffold_51143	-	863	767	**RNA**→ PRK11431 (PRK11431)EmrE (COG2076)→
env-224	SRS018427_C2270557	+	288	384	**RNA**→ PRK11431 (PRK11431)EmrE (COG2076)→
env-225	SRS043411_WUGC_scaffold_44585	-	1187	1091	**RNA**→ PRK11431 (PRK11431)EmrE (COG2076)→
env-226	SRS053398_C2182408	-	320	224	**RNA**→ PRK11431 (PRK11431)EmrE (COG2076)→
env-227	SRS054590_LANL_scaffold_9290	-	963	867	**RNA**→ PRK11431 (PRK11431)EmrE (COG2076)→
env-228	SRS054956_C1527013	-	750	654	**RNA**→ PRK11431 (PRK11431)EmrE (COG2076)→
env-229	SRS056519_LANL_scaffold_23575	+	283	379	**RNA**→ PRK11431 (PRK11431)EmrE (COG2076)→
env-230	SRS063985_C2378642	-	230	134	**RNA**→ PRK11431 (PRK11431)EmrE (COG2076)→
Osp-2-1	NZ_KI271680.1	+	21977	22073	**RNA**→ PRK11431 (PRK11431)EmrE (COG2076)→
Osp-1-1	NZ_KI271532.1	+	41064	41160	**RNA**→ PRK11431 (PRK11431)EmrE (COG2076)→
Oba-1-1	NZ_BAIH01000109.1	+	1250	1346	**RNA**→ PRK11431 (PRK11431)EmrE (COG2076)→
env-231	SRS053214_LANL_scaffold_21121	-	992	896	**RNA**→ PRK11431 (PRK11431)EmrE (COG2076)→
env-232	OGCZ01000516.1	-	14237	14141	**RNA**→ PRK11431 (PRK11431)EmrE (COG2076)→
env-233	OGGC01041441.1	+	40	136	**RNA**→ PRK11431 (PRK11431)PRK10535 (PRK10535)EmrE (COG2076)LolE (COG4591)→
env-234	OGGX01012421.1	+	2	98	**RNA**→ PRK11431 (PRK11431)EmrE (COG2076)→
env-235	OGHJ01000104.1	+	21072	21168	**RNA**→ PRK11431 (PRK11431)EmrE (COG2076)→
env-236	OGHL01017035.1	-	308	212	**RNA**→ PRK11431 (PRK11431)EmrE (COG2076)→
env-237	OGJH01042791.1	+	399	495	**RNA**→ PRK11431 (PRK11431)EmrE (COG2076)→
env-238	OIXS01006491.1	-	537	441	**RNA**→ PRK11431 (PRK11431)EmrE (COG2076)→
env-239	OGIK01017584.1	+	1040	1136	**RNA**→ PRK11431 (PRK11431)EmrE (COG2076)→
env-240	OGLV01028700.1	+	62	158	**RNA**→ PRK11431 (PRK11431)PRK10535 (PRK10535)EmrE (COG2076)LolE (COG4591)→

env-241	OGLX01074428.1	+	62	158	**RNA**→ PRK11431 (PRK11431)EmrE (COG2076)→
env-242	OLGN01018691.1	+	1545	1641	**RNA**→
env-243	Ga0134369_100541	+	9510	9606	**RNA**→ EmrE (COG2076)→
env-244	Ga0134420_127745	+	175	271	**RNA**→ PRK11431 (PRK11431)EmrE (COG2076)→
env-245	Ga0134389_1097918	-	211	115	**RNA**→ PRK11431 (PRK11431)EmrE (COG2076)→
env-246	Ga0134427_1000584	-	16593	16497	**RNA**→ PRK11431 (PRK11431)EmrE (COG2076)→
env-247	Ga0134458_115865	+	361	457	**RNA**→ PRK11431 (PRK11431)EmrE (COG2076)→
env-248	scaffold3622_3_V1.CD-9	+	330	426	**RNA**→ PRK11431 (PRK11431)EmrE (COG2076)→
env-249	SRS019267_C3534512	-	327	231	**RNA**→ PRK11431 (PRK11431)EmrE (COG2076)→
env-250	SRS045713_WUGC_scaffold_17193	+	220	316	**RNA**→ PRK11431 (PRK11431)EmrE (COG2076)→
env-251	SRS018313_C7911232	+	402	498	**RNA**→ PRK11431 (PRK11431)EmrE (COG2076)→
env-252	OGHO01003436.1	+	3512	3608	**RNA**→ PRK11431 (PRK11431)EmrE (COG2076)→
env-253	Ga0134439_1042032	+	183	279	**RNA**→ PRK11431 (PRK11431)EmrE (COG2076)→
env-254	Ga0256408_1000801	+	1485	1584	**RNA**→ PRK11431 (PRK11431)EmrE (COG2076)→ PRK11431 (PRK11431)EmrE (COG2076)→
env-255	Ga0169775_10859	-	2638	2521	**RNA**→ PRK11431 (PRK11431)EmrE (COG2076)→ PRK11431 (PRK11431)EmrE (COG2076)→
Efa-269-1	NZ_GL456497.1	+	36505	36605	**RNA**→ PRK11431 (PRK11431)EmrE (COG2076)→ PRK11431 (PRK11431)EmrE (COG2076)→
Efa-159-1	NZ_KB947307.1	+	37203	37303	**RNA**→ PRK11431 (PRK11431)EmrE (COG2076)→ PRK11431 (PRK11431)EmrE (COG2076)→
env-256	Ga0121696_103369	-	719	619	**RNA**→ PRK11431 (PRK11431)EmrE (COG2076)→ PRK11431 (PRK11431)EmrE (COG2076)→
env-257	Ga0169765_100102	-	104396	104296	**RNA**→ PRK11431 (PRK11431)EmrE (COG2076)→ PRK11431 (PRK11431)EmrE (COG2076)→
Efa-221-1	NZ_KB944754.1	+	50245	50345	**RNA**→ PRK11431 (PRK11431)EmrE (COG2076)→ PRK11431 (PRK11431)EmrE (COG2076)→
Efa-9-1	NZ_KE351778.1	+	36451	36551	**RNA**→ PRK11431 (PRK11431)EmrE (COG2076)→ PRK11431 (PRK11431)EmrE (COG2076)→
Efa-261-1	NZ_AYOJ01000044.1	+	35402	35502	**RNA**→ PRK11431 (PRK11431)EmrE (COG2076)→ PRK11431 (PRK11431)EmrE (COG2076)→
Efa-259-1	NZ_AYLN01000022.1	+	35408	35508	**RNA**→ PRK11431 (PRK11431)EmrE (COG2076)→ PRK11431 (PRK11431)EmrE (COG2076)→
env-258	Ga0122071_109648	-	573	473	**RNA**→ PRK11431 (PRK11431)EmrE (COG2076)→ hypo→
env-259	Ga0122028_114612	+	135	235	**RNA**→
env-260	Ga0122086_106277	-	243	143	**RNA**→ hypo→
env-261	Ga0121121_11209	-	138	38	**RNA**→
env-262	DKHA01000002.1	-	18064	17964	**RNA**→ PRK11431 (PRK11431)EmrE (COG2076)→ PRK11431 (PRK11431)EmrE (COG2076)→
env-263	DBUD01000200.1	-	13108	13008	**RNA**→ PRK11431 (PRK11431)EmrE (COG2076)→ PRK11431 (PRK11431)EmrE (COG2076)→
env-264	DCRD01000003.1	+	36427	36527	**RNA**→ PRK11431 (PRK11431)EmrE (COG2076)→ PRK11431 (PRK11431)EmrE (COG2076)→
env-265	DQIL01000125.1	-	4333	4233	**RNA**→ PRK11431 (PRK11431)EmrE (COG2076)→ PRK11431 (PRK11431)EmrE (COG2076)→
env-266	Ga0129309_1000008	-	253797	253697	**RNA**→ PRK11431 (PRK11431)EmrE (COG2076)→ PRK11431 (PRK11431)EmrE (COG2076)→
env-267	Ga0129308_1000026	-	253797	253697	**RNA**→ PRK11431 (PRK11431)EmrE (COG2076)→ PRK11431 (PRK11431)EmrE (COG2076)→
env-268	Ga0121029_10018	-	166681	166581	**RNA**→ PRK11431 (PRK11431)EmrE (COG2076)→ PRK11431 (PRK11431)EmrE (COG2076)→
env-269	Ga0169876_100422	-	2647	2547	**RNA**→ PRK11431 (PRK11431)EmrE (COG2076)→ PRK11431 (PRK11431)EmrE (COG2076)→
Efa-321-1	NZ_GG698872.1	+	39125	39225	**RNA**→ PRK11431 (PRK11431)EmrE (COG2076)→ PRK11431 (PRK11431)EmrE (COG2076)→ PRK11431 (PRK11431)EmrE (COG2076)→ PRK11431 (PRK11431)EmrE (COG2076)→
Efa-327-1	NZ_GG688429.1	+	38425	38525	**RNA**→ PRK11431 (PRK11431)EmrE (COG2076)→ PRK11431 (PRK11431)EmrE (COG2076)→ PRK11431 (PRK11431)EmrE (COG2076)→ PRK11431 (PRK11431)EmrE (COG2076)→
Efa-317-1	NZ_GG670353.1	+	38670	38770	**RNA**→ PRK11431 (PRK11431)EmrE (COG2076)→ PRK11431 (PRK11431)EmrE (COG2076)→ PRK11431 (PRK11431)EmrE (COG2076)→ PRK11431 (PRK11431)EmrE (COG2076)→
Efa-303-1	NZ_GG688660.1	+	75008	75108	**RNA**→ PRK11431 (PRK11431)EmrE (COG2076)→ PRK11431 (PRK11431)EmrE (COG2076)→ PRK11431 (PRK11431)EmrE (COG2076)→ PRK11431 (PRK11431)EmrE (COG2076)→
Efa-272-1	NZ_GL454738.1	-	253084	252984	**RNA**→ PRK11431 (PRK11431)EmrE (COG2076)→ PRK11431 (PRK11431)EmrE (COG2076)→
Efa-273-1	NZ_GL454499.1	-	38027	37927	**RNA**→ PRK11431 (PRK11431)EmrE (COG2076)→ PRK11431 (PRK11431)EmrE (COG2076)→
Efa-274-1	NZ_GL455112.1	+	37290	37390	**RNA**→ PRK11431 (PRK11431)EmrE (COG2076)→ PRK11431 (PRK11431)EmrE (COG2076)→
Efa-281-1	NZ_GL454902.1	-	120229	120129	**RNA**→ PRK11431 (PRK11431)EmrE (COG2076)→ PRK11431 (PRK11431)EmrE (COG2076)→
Efa-183-1	NZ_GL455388.1	-	121887	121787	**RNA**→ PRK11431 (PRK11431)EmrE (COG2076)→ PRK11431 (PRK11431)EmrE (COG2076)→
Efa-279-1	NZ_GL456461.1	+	29274	29374	**RNA**→ PRK11431 (PRK11431)EmrE (COG2076)→ PRK11431 (PRK11431)EmrE (COG2076)→
Efa-282-1	NZ_GL457132.1	+	2580	2680	**RNA**→ PRK11431 (PRK11431)EmrE (COG2076)→ PRK11431 (PRK11431)EmrE (COG2076)→
Efa-278-1	NZ_GL456773.1	+	37140	37240	**RNA**→ PRK11431 (PRK11431)EmrE (COG2076)→ PRK11431 (PRK11431)EmrE (COG2076)→
Efa-288-1	NZ_GL476297.1	-	117402	117302	**RNA**→ PRK11431 (PRK11431)EmrE (COG2076)→ PRK11431 (PRK11431)EmrE (COG2076)→
Efa-304-1	NC_018221.1	+	297276	297376	**RNA**→ PRK11431 (PRK11431)EmrE (COG2076)→ PRK11431 (PRK11431)EmrE (COG2076)→

Efa-189-1	NZ_KB933496.1	+	75254	75354	**RNA**→ PRK11431 (PRK11431)EmrE (COG2076)→ PRK11431 (PRK11431)EmrE (COG2076)→
Efa-218-1	NZ_KB944542.1	+	44671	44771	**RNA**→ PRK11431 (PRK11431)EmrE (COG2076)→ PRK11431 (PRK11431)EmrE (COG2076)→
Efa-201-1	NZ_KB944585.1	+	41553	41653	**RNA**→ PRK11431 (PRK11431)EmrE (COG2076)→ PRK11431 (PRK11431)EmrE (COG2076)→
Efa-199-1	NZ_KB944550.1	+	39150	39250	**RNA**→ PRK11431 (PRK11431)EmrE (COG2076)→ PRK11431 (PRK11431)EmrE (COG2076)→
Efa-204-1	NZ_KB944670.1	+	43662	43762	**RNA**→ PRK11431 (PRK11431)EmrE (COG2076)→ PRK11431 (PRK11431)EmrE (COG2076)→
Efa-60-1	NZ_KB932367.1	+	40323	40423	**RNA**→ PRK11431 (PRK11431)EmrE (COG2076)→ PRK11431 (PRK11431)EmrE (COG2076)→
Efa-205-1	NZ_KB944673.1	+	43490	43590	**RNA**→ PRK11431 (PRK11431)EmrE (COG2076)→ PRK11431 (PRK11431)EmrE (COG2076)→
Efa-219-1	NZ_KB944837.1	+	44997	45097	**RNA**→ PRK11431 (PRK11431)EmrE (COG2076)→ PRK11431 (PRK11431)EmrE (COG2076)→
Efa-197-1	NZ_KB944537.1	-	2232000	2231900	**RNA**→ PRK11431 (PRK11431)EmrE (COG2076)→ PRK11431 (PRK11431)EmrE (COG2076)→
Efa-222-1	NZ_KB944799.1	+	81236	81336	**RNA**→ PRK11431 (PRK11431)EmrE (COG2076)→ PRK11431 (PRK11431)EmrE (COG2076)→
Efa-213-1	NZ_KB944562.1	+	73032	73132	**RNA**→ PRK11431 (PRK11431)EmrE (COG2076)→ PRK11431 (PRK11431)EmrE (COG2076)→
Efa-206-1	NZ_KB944712.1	+	40236	40336	**RNA**→ PRK11431 (PRK11431)EmrE (COG2076)→ PRK11431 (PRK11431)EmrE (COG2076)→
Efa-187-1	NZ_KB945049.1	+	39420	39520	**RNA**→ PRK11431 (PRK11431)EmrE (COG2076)→ PRK11431 (PRK11431)EmrE (COG2076)→
Efa-190-1	NZ_KB945028.1	+	36898	36998	**RNA**→ PRK11431 (PRK11431)EmrE (COG2076)→ PRK11431 (PRK11431)EmrE (COG2076)→
Efa-143-1	NZ_KB946408.1	+	41239	41339	**RNA**→ PRK11431 (PRK11431)EmrE (COG2076)→ PRK11431 (PRK11431)EmrE (COG2076)→
Efa-148-1	NZ_KB946498.1	+	80073	80173	**RNA**→ PRK11431 (PRK11431)EmrE (COG2076)→ PRK11431 (PRK11431)EmrE (COG2076)→
Efa-153-1	NZ_KB946670.1	+	43481	43581	**RNA**→ PRK11431 (PRK11431)EmrE (COG2076)→ PRK11431 (PRK11431)EmrE (COG2076)→
Efa-144-1	NZ_KB946412.1	+	42250	42350	**RNA**→ PRK11431 (PRK11431)EmrE (COG2076)→ PRK11431 (PRK11431)EmrE (COG2076)→
Efa-149-1	NZ_KB946507.1	+	80221	80321	**RNA**→ PRK11431 (PRK11431)EmrE (COG2076)→ PRK11431 (PRK11431)EmrE (COG2076)→
Efa-147-1	NZ_KB946489.1	+	78437	78537	**RNA**→ PRK11431 (PRK11431)EmrE (COG2076)→ PRK11431 (PRK11431)EmrE (COG2076)→
Efa-176-1	NZ_KB947270.1	+	78677	78777	**RNA**→ PRK11431 (PRK11431)EmrE (COG2076)→ PRK11431 (PRK11431)EmrE (COG2076)→
Efa-179-1	NZ_KB947290.1	+	84339	84439	**RNA**→ PRK11431 (PRK11431)EmrE (COG2076)→ PRK11431 (PRK11431)EmrE (COG2076)→
Efa-172-1	NZ_KB947208.1	+	372073	372173	**RNA**→ PRK11431 (PRK11431)EmrE (COG2076)→ PRK11431 (PRK11431)EmrE (COG2076)→
Efa-178-1	NZ_KB947334.1	+	43727	43827	**RNA**→ PRK11431 (PRK11431)EmrE (COG2076)→ PRK11431 (PRK11431)EmrE (COG2076)→
Efa-150-1	NZ_KB946519.1	+	42544	42644	**RNA**→ PRK11431 (PRK11431)EmrE (COG2076)→ PRK11431 (PRK11431)EmrE (COG2076)→
Efa-177-1	NZ_KB947204.1	+	42768	42868	**RNA**→ PRK11431 (PRK11431)EmrE (COG2076)→ PRK11431 (PRK11431)EmrE (COG2076)→
Efa-167-1	NZ_KB947300.1	+	36807	36907	**RNA**→ PRK11431 (PRK11431)EmrE (COG2076)→ PRK11431 (PRK11431)EmrE (COG2076)→
Efa-154-1	NZ_KB946615.1	+	47690	47790	**RNA**→ PRK11431 (PRK11431)EmrE (COG2076)→ PRK11431 (PRK11431)EmrE (COG2076)→
Efa-156-1	NZ_KB947325.1	+	44502	44602	**RNA**→ PRK11431 (PRK11431)EmrE (COG2076)→ PRK11431 (PRK11431)EmrE (COG2076)→
Efa-191-1	NZ_KB947479.1	+	47189	47289	**RNA**→ PRK11431 (PRK11431)EmrE (COG2076)→ PRK11431 (PRK11431)EmrE (COG2076)→
Efa-296-1	NZ_KB949589.1	+	40391	40491	**RNA**→ PRK11431 (PRK11431)EmrE (COG2076)→ PRK11431 (PRK11431)EmrE (COG2076)→
Efa-138-1	NZ_KB949667.1	+	40979	41079	**RNA**→ PRK11431 (PRK11431)EmrE (COG2076)→ PRK11431 (PRK11431)EmrE (COG2076)→
Efa-158-1	NZ_KB947337.1	+	43089	43189	**RNA**→ PRK11431 (PRK11431)EmrE (COG2076)→ PRK11431 (PRK11431)EmrE (COG2076)→
Efa-7-1	NZ_KE350878.1	+	36331	36431	**RNA**→ PRK11431 (PRK11431)EmrE (COG2076)→ PRK11431 (PRK11431)EmrE (COG2076)→
Efa-8-1	NZ_KE351694.1	-	243950	243850	**RNA**→ PRK11431 (PRK11431)EmrE (COG2076)→ PRK11431 (PRK11431)EmrE (COG2076)→
Efa-254-1	NZ_KI518257.1	-	1735572	1735472	**RNA**→ PRK11431 (PRK11431)EmrE (COG2076)→ PRK11431 (PRK11431)EmrE (COG2076)→
Efa-238-1	NZ_KI913032.1	+	2808	2908	**RNA**→ PRK11431 (PRK11431)EmrE (COG2076)→ PRK11431 (PRK11431)EmrE (COG2076)→
Efa-235-1	NZ_KI913016.1	+	38154	38254	**RNA**→ PRK11431 (PRK11431)EmrE (COG2076)→ PRK11431 (PRK11431)EmrE (COG2076)→
Efa-225-1	NZ_KI912939.1	+	43836	43936	**RNA**→ PRK11431 (PRK11431)EmrE (COG2076)→ PRK11431 (PRK11431)EmrE (COG2076)→
Efa-248-1	NZ_KI913093.1	+	333792	333892	**RNA**→ PRK11431 (PRK11431)EmrE (COG2076)→ PRK11431 (PRK11431)EmrE (COG2076)→
Efa-173-1	NZ_KB946719.1	-	2559216	2559116	**RNA**→ PRK11431 (PRK11431)EmrE (COG2076)→ PRK11431 (PRK11431)EmrE (COG2076)→
Efa-1-1	NZ_CP015410.2	+	959838	959938	**RNA**→ PRK11431 (PRK11431)EmrE (COG2076)→ PRK11431 (PRK11431)EmrE (COG2076)→
Efa-324-1	NZ_GG669020.1	-	186599	186499	**RNA**→ PRK11431 (PRK11431)EmrE (COG2076)→ PRK11431 (PRK11431)EmrE (COG2076)→
Efa-1-2	NZ_CP022712.1	+	2390244	2390344	**RNA**→ PRK11431 (PRK11431)EmrE (COG2076)→ PRK11431 (PRK11431)EmrE (COG2076)→
Efa-252-1	NZ_AYKL01000003.1	-	12921	12821	**RNA**→ PRK11431 (PRK11431)EmrE (COG2076)→ PRK11431 (PRK11431)EmrE (COG2076)→
Efa-253-1	NZ_AYLV01000004.1	-	25460	25360	**RNA**→ PRK11431 (PRK11431)EmrE (COG2076)→ PRK11431 (PRK11431)EmrE (COG2076)→
Efa-264-1	NZ_AYOL01000063.1	-	10957	10857	**RNA**→ PRK11431 (PRK11431)EmrE (COG2076)→ PRK11431 (PRK11431)EmrE (COG2076)→
Esp-2-1	NZ_JVOE01000046.1	-	43454	43354	**RNA**→ PRK11431 (PRK11431)EmrE (COG2076)→ PRK11431 (PRK11431)EmrE (COG2076)→
env-270	DIGT01000017.1	-	344919	344819	**RNA**→ PRK11431 (PRK11431)EmrE (COG2076)→ PRK11431 (PRK11431)EmrE (COG2076)→
env-271	DMGN01000013.1	+	37079	37179	**RNA**→ PRK11431 (PRK11431)EmrE (COG2076)→ PRK11431 (PRK11431)EmrE (COG2076)→
env-272	Ga0121028_13317	+	136	236	**RNA**→ hypo→
Efa-291-3	NC_004668.1	+	328333	328433	**RNA**→ PRK11431 (PRK11431)EmrE (COG2076)→ PRK11431 (PRK11431)EmrE (COG2076)→

Efa-309-1	NZ_GG668829.1	+	35735	35835	**RNA**→ PRK11431 (PRK11431)EmrE (COG2076)→ PRK11431 (PRK11431)EmrE (COG2076)→
Efa-277-1	NZ_GL454245.1	-	190598	190498	**RNA**→ PRK11431 (PRK11431)EmrE (COG2076)→ PRK11431 (PRK11431)EmrE (COG2076)→
Efa-284-1	NZ_GL454529.1	-	149860	149760	**RNA**→ PRK11431 (PRK11431)EmrE (COG2076)→ PRK11431 (PRK11431)EmrE (COG2076)→
Efa-276-1	NZ_GL454309.1	-	190649	190549	**RNA**→ PRK11431 (PRK11431)EmrE (COG2076)→ PRK11431 (PRK11431)EmrE (COG2076)→
Efa-275-1	NZ_GL455460.1	+	36384	36484	**RNA**→ PRK11431 (PRK11431)EmrE (COG2076)→ PRK11431 (PRK11431)EmrE (COG2076)→
Efa-313-1	NC_017316.1	+	257853	257953	**RNA**→ PRK11431 (PRK11431)EmrE (COG2076)→ PRK11431 (PRK11431)EmrE (COG2076)→
Efa-193-1	NZ_KB944677.1	+	41453	41553	**RNA**→ PRK11431 (PRK11431)EmrE (COG2076)→ PRK11431 (PRK11431)EmrE (COG2076)→
Efa-71-1	NZ_KB932437.1	+	1421334	1421434	**RNA**→ PRK11431 (PRK11431)EmrE (COG2076)→ PRK11431 (PRK11431)EmrE (COG2076)→
Efa-18-1	NZ_KB944641.1	+	46292	46392	**RNA**→ PRK11431 (PRK11431)EmrE (COG2076)→ PRK11431 (PRK11431)EmrE (COG2076)→
Efa-73-1	NZ_KB932455.1	+	77968	78068	**RNA**→ PRK11431 (PRK11431)EmrE (COG2076)→ PRK11431 (PRK11431)EmrE (COG2076)→
Efa-92-1	NZ_KB932450.1	+	79093	79193	**RNA**→ PRK11431 (PRK11431)EmrE (COG2076)→ PRK11431 (PRK11431)EmrE (COG2076)→
Efa-194-1	NZ_KB944930.1	+	42242	42342	**RNA**→ PRK11431 (PRK11431)EmrE (COG2076)→ PRK11431 (PRK11431)EmrE (COG2076)→
Efa-192-1	NZ_KB944666.1	+	523422	523522	**RNA**→ PRK11431 (PRK11431)EmrE (COG2076)→ PRK11431 (PRK11431)EmrE (COG2076)→
Efa-53-1	NZ_KB932423.1	-	707408	707308	**RNA**→ PRK11431 (PRK11431)EmrE (COG2076)→ PRK11431 (PRK11431)EmrE (COG2076)→
Efa-50-1	NZ_KB932414.1	+	77636	77736	**RNA**→ PRK11431 (PRK11431)EmrE (COG2076)→ PRK11431 (PRK11431)EmrE (COG2076)→
Efa-101-1	NZ_KB932476.1	+	77644	77744	**RNA**→ PRK11431 (PRK11431)EmrE (COG2076)→ PRK11431 (PRK11431)EmrE (COG2076)→
Efa-96-1	NZ_KB932537.1	+	79728	79828	**RNA**→ PRK11431 (PRK11431)EmrE (COG2076)→ PRK11431 (PRK11431)EmrE (COG2076)→
Efa-77-1	NZ_KB932495.1	+	78272	78372	**RNA**→ PRK11431 (PRK11431)EmrE (COG2076)→ PRK11431 (PRK11431)EmrE (COG2076)→
Efa-94-1	NZ_KB932517.1	+	77911	78011	**RNA**→ PRK11431 (PRK11431)EmrE (COG2076)→ PRK11431 (PRK11431)EmrE (COG2076)→
Efa-195-1	NZ_KB944963.1	+	79777	79877	**RNA**→ PRK11431 (PRK11431)EmrE (COG2076)→ PRK11431 (PRK11431)EmrE (COG2076)→
Efa-52-1	NZ_KB932418.1	+	78350	78450	**RNA**→ PRK11431 (PRK11431)EmrE (COG2076)→ PRK11431 (PRK11431)EmrE (COG2076)→
Efa-51-1	NZ_KB932415.1	+	82171	82271	**RNA**→ PRK11431 (PRK11431)EmrE (COG2076)→ PRK11431 (PRK11431)EmrE (COG2076)→
Efa-90-1	NZ_KB932557.1	+	78047	78147	**RNA**→ PRK11431 (PRK11431)EmrE (COG2076)→ PRK11431 (PRK11431)EmrE (COG2076)→
Efa-88-1	NZ_KB932426.1	+	83044	83144	**RNA**→ PRK11431 (PRK11431)EmrE (COG2076)→ PRK11431 (PRK11431)EmrE (COG2076)→
Efa-78-1	NZ_KB932503.1	+	77618	77718	**RNA**→ PRK11431 (PRK11431)EmrE (COG2076)→ PRK11431 (PRK11431)EmrE (COG2076)→
Efa-93-1	NZ_KB932472.1	+	77811	77911	**RNA**→ PRK11431 (PRK11431)EmrE (COG2076)→ PRK11431 (PRK11431)EmrE (COG2076)→
Efa-95-1	NZ_KB932530.1	+	80866	80966	**RNA**→ PRK11431 (PRK11431)EmrE (COG2076)→ PRK11431 (PRK11431)EmrE (COG2076)→
Efa-72-1	NZ_KB932441.1	+	79661	79761	**RNA**→ PRK11431 (PRK11431)EmrE (COG2076)→ PRK11431 (PRK11431)EmrE (COG2076)→
Efa-82-1	NZ_KB932542.1	+	80119	80219	**RNA**→ PRK11431 (PRK11431)EmrE (COG2076)→ PRK11431 (PRK11431)EmrE (COG2076)→
Efa-102-1	NZ_KB932435.1	+	75851	75951	**RNA**→ PRK11431 (PRK11431)EmrE (COG2076)→ PRK11431 (PRK11431)EmrE (COG2076)→
Efa-69-1	NZ_KB932552.1	+	73136	73236	**RNA**→ PRK11431 (PRK11431)EmrE (COG2076)→ PRK11431 (PRK11431)EmrE (COG2076)→
Efa-83-1	NZ_KB932571.1	+	40421	40521	**RNA**→ PRK11431 (PRK11431)EmrE (COG2076)→ PRK11431 (PRK11431)EmrE (COG2076)→
Efa-70-1	NZ_KB932430.1	+	352245	352345	**RNA**→ PRK11431 (PRK11431)EmrE (COG2076)→ PRK11431 (PRK11431)EmrE (COG2076)→
Efa-91-1	NZ_KB932445.1	+	72712	72812	**RNA**→ PRK11431 (PRK11431)EmrE (COG2076)→ PRK11431 (PRK11431)EmrE (COG2076)→
Efa-81-1	NZ_KB932525.1	+	343352	343452	**RNA**→ PRK11431 (PRK11431)EmrE (COG2076)→ PRK11431 (PRK11431)EmrE (COG2076)→
Efa-79-1	NZ_KB932512.1	+	78316	78416	**RNA**→ PRK11431 (PRK11431)EmrE (COG2076)→ PRK11431 (PRK11431)EmrE (COG2076)→
Efa-74-1	NZ_KB932463.1	+	82437	82537	**RNA**→ PRK11431 (PRK11431)EmrE (COG2076)→ PRK11431 (PRK11431)EmrE (COG2076)→
Efa-97-1	NZ_KB932563.1	+	42478	42578	**RNA**→ PRK11431 (PRK11431)EmrE (COG2076)→ PRK11431 (PRK11431)EmrE (COG2076)→
Efa-76-1	NZ_KB932488.1	+	79987	80087	**RNA**→ PRK11431 (PRK11431)EmrE (COG2076)→ PRK11431 (PRK11431)EmrE (COG2076)→
Efa-89-1	NZ_KB932548.1	+	73764	73864	**RNA**→ PRK11431 (PRK11431)EmrE (COG2076)→ PRK11431 (PRK11431)EmrE (COG2076)→
Efa-142-1	NZ_KB946399.1	+	40710	40810	**RNA**→ PRK11431 (PRK11431)EmrE (COG2076)→ PRK11431 (PRK11431)EmrE (COG2076)→
Efa-139-1	NZ_KB946180.1	+	40693	40793	**RNA**→ PRK11431 (PRK11431)EmrE (COG2076)→ PRK11431 (PRK11431)EmrE (COG2076)→
Efa-137-1	NZ_KB946659.1	+	74090	74190	**RNA**→ PRK11431 (PRK11431)EmrE (COG2076)→ PRK11431 (PRK11431)EmrE (COG2076)→
Efa-155-1	NZ_KB946584.1	+	43006	43106	**RNA**→ PRK11431 (PRK11431)EmrE (COG2076)→ PRK11431 (PRK11431)EmrE (COG2076)→
Efa-145-1	NZ_KB946415.1	+	82422	82522	**RNA**→ PRK11431 (PRK11431)EmrE (COG2076)→ PRK11431 (PRK11431)EmrE (COG2076)→
Efa-151-1	NZ_KB946563.1	+	44246	44346	**RNA**→ PRK11431 (PRK11431)EmrE (COG2076)→ PRK11431 (PRK11431)EmrE (COG2076)→
Efa-157-1	NZ_KB947183.1	+	48323	48423	**RNA**→ PRK11431 (PRK11431)EmrE (COG2076)→ PRK11431 (PRK11431)EmrE (COG2076)→
Efa-136-1	NZ_KB948663.1	+	74222	74322	**RNA**→ PRK11431 (PRK11431)EmrE (COG2076)→ PRK11431 (PRK11431)EmrE (COG2076)→
Efa-171-1	NZ_KB947100.1	+	42415	42515	**RNA**→ PRK11431 (PRK11431)EmrE (COG2076)→ PRK11431 (PRK11431)EmrE (COG2076)→
Efa-116-1	NZ_KB948606.1	+	84576	84676	**RNA**→ PRK11431 (PRK11431)EmrE (COG2076)→ PRK11431 (PRK11431)EmrE (COG2076)→
Efa-180-1	NZ_KB947167.1	+	82442	82542	**RNA**→ PRK11431 (PRK11431)EmrE (COG2076)→ PRK11431 (PRK11431)EmrE (COG2076)→
Efa-169-1	NZ_KB947149.1	+	411309	411409	**RNA**→ PRK11431 (PRK11431)EmrE (COG2076)→ PRK11431 (PRK11431)EmrE (COG2076)→

Efa-119-1	NZ_KB948869.1	+	79720	79820	**RNA**→	PRK11431 (PRK11431)EmrE (COG2076)→	PRK11431 (PRK11431)EmrE (COG2076)→
Efa-181-1	NZ_KB947132.1	+	79250	79350	**RNA**→	PRK11431 (PRK11431)EmrE (COG2076)→	PRK11431 (PRK11431)EmrE (COG2076)→
Efa-104-1	NZ_KB948306.1	+	3012983	3013083	**RNA**→	PRK11431 (PRK11431)EmrE (COG2076)→	PRK11431 (PRK11431)EmrE (COG2076)→
Efa-109-1	NZ_KB948428.1	+	80682	80782	**RNA**→	PRK11431 (PRK11431)EmrE (COG2076)→	PRK11431 (PRK11431)EmrE (COG2076)→
Efa-117-1	NZ_KB948772.1	+	73876	73976	**RNA**→	PRK11431 (PRK11431)EmrE (COG2076)→	PRK11431 (PRK11431)EmrE (COG2076)→
Efa-103-1	NZ_KB948293.1	+	78356	78456	**RNA**→	PRK11431 (PRK11431)EmrE (COG2076)→	PRK11431 (PRK11431)EmrE (COG2076)→
Efa-106-1	NZ_KB948373.1	+	77712	77812	**RNA**→	PRK11431 (PRK11431)EmrE (COG2076)→	PRK11431 (PRK11431)EmrE (COG2076)→
Efa-111-1	NZ_KB948511.1	+	79594	79694	**RNA**→	PRK11431 (PRK11431)EmrE (COG2076)→	PRK11431 (PRK11431)EmrE (COG2076)→
Efa-115-1	NZ_KB948697.1	+	77846	77946	**RNA**→	PRK11431 (PRK11431)EmrE (COG2076)→	PRK11431 (PRK11431)EmrE (COG2076)→
Efa-124-1	NZ_KB949000.1	+	78698	78798	**RNA**→	PRK11431 (PRK11431)EmrE (COG2076)→	PRK11431 (PRK11431)EmrE (COG2076)→
Efa-125-1	NZ_KB949045.1	+	74881	74981	**RNA**→	PRK11431 (PRK11431)EmrE (COG2076)→	PRK11431 (PRK11431)EmrE (COG2076)→
Efa-105-1	NZ_KB948323.1	+	682197	682297	**RNA**→	PRK11431 (PRK11431)EmrE (COG2076)→	PRK11431 (PRK11431)EmrE (COG2076)→
Efa-223-1	NZ_KB948765.1	+	74780	74880	**RNA**→	PRK11431 (PRK11431)EmrE (COG2076)→	PRK11431 (PRK11431)EmrE (COG2076)→
Efa-118-1	NZ_KB948828.1	+	77860	77960	**RNA**→	PRK11431 (PRK11431)EmrE (COG2076)→	PRK11431 (PRK11431)EmrE (COG2076)→
Efa-113-1	NZ_KB948569.1	+	77696	77796	**RNA**→	PRK11431 (PRK11431)EmrE (COG2076)→	PRK11431 (PRK11431)EmrE (COG2076)→
Efa-121-1	NZ_KB948904.1	+	42796	42896	**RNA**→	PRK11431 (PRK11431)EmrE (COG2076)→	PRK11431 (PRK11431)EmrE (COG2076)→
Efa-123-1	NZ_KB948975.1	+	77904	78004	**RNA**→	PRK11431 (PRK11431)EmrE (COG2076)→	PRK11431 (PRK11431)EmrE (COG2076)→
Efa-128-1	NZ_KB949143.1	+	74425	74525	**RNA**→	PRK11431 (PRK11431)EmrE (COG2076)→	PRK11431 (PRK11431)EmrE (COG2076)→
Efa-120-1	NZ_KB948877.1	+	78182	78282	**RNA**→	PRK11431 (PRK11431)EmrE (COG2076)→	PRK11431 (PRK11431)EmrE (COG2076)→
Efa-134-1	NZ_KB949340.1	+	74505	74605	**RNA**→	PRK11431 (PRK11431)EmrE (COG2076)→	PRK11431 (PRK11431)EmrE (COG2076)→
Efa-114-1	NZ_KB948648.1	+	79609	79709	**RNA**→	PRK11431 (PRK11431)EmrE (COG2076)→	PRK11431 (PRK11431)EmrE (COG2076)→
Efa-108-1	NZ_KB948410.1	+	74145	74245	**RNA**→	PRK11431 (PRK11431)EmrE (COG2076)→	PRK11431 (PRK11431)EmrE (COG2076)→
Efa-131-1	NZ_KB949245.1	+	78417	78517	**RNA**→	PRK11431 (PRK11431)EmrE (COG2076)→	PRK11431 (PRK11431)EmrE (COG2076)→
Efa-132-1	NZ_KB949289.1	+	73651	73751	**RNA**→	PRK11431 (PRK11431)EmrE (COG2076)→	PRK11431 (PRK11431)EmrE (COG2076)→
Efa-127-1	NZ_KB949118.1	+	72896	72996	**RNA**→	PRK11431 (PRK11431)EmrE (COG2076)→	PRK11431 (PRK11431)EmrE (COG2076)→
Efa-133-1	NZ_KB949296.1	+	81248	81348	**RNA**→	PRK11431 (PRK11431)EmrE (COG2076)→	PRK11431 (PRK11431)EmrE (COG2076)→
Efa-110-1	NZ_KB948472.1	+	80896	80996	**RNA**→	PRK11431 (PRK11431)EmrE (COG2076)→	PRK11431 (PRK11431)EmrE (COG2076)→
Efa-112-1	NZ_KB948537.1	+	72896	72996	**RNA**→	PRK11431 (PRK11431)EmrE (COG2076)→	PRK11431 (PRK11431)EmrE (COG2076)→
Efa-291-1	NZ_KE136404.1	-	189030	188930	**RNA**→	PRK11431 (PRK11431)EmrE (COG2076)→	PRK11431 (PRK11431)EmrE (COG2076)→
Efa-291-2	NZ_KE136524.1	+	773196	773296	**RNA**→	PRK11431 (PRK11431)EmrE (COG2076)→	PRK11431 (PRK11431)EmrE (COG2076)→
Efa-22-1	NZ_KI518244.1	+	76530	76630	**RNA**→	PRK11431 (PRK11431)EmrE (COG2076)→	PRK11431 (PRK11431)EmrE (COG2076)→
Efa-11-1	NZ_KK640442.1	+	2058	2158	**RNA**→	PRK11431 (PRK11431)EmrE (COG2076)→	PRK11431 (PRK11431)EmrE (COG2076)→
Efa-126-1	NZ_KB949079.1	+	79600	79700	**RNA**→	PRK11431 (PRK11431)EmrE (COG2076)→	PRK11431 (PRK11431)EmrE (COG2076)→
Efa-249-1	NZ_KI913107.1	+	78151	78251	**RNA**→	PRK11431 (PRK11431)EmrE (COG2076)→	PRK11431 (PRK11431)EmrE (COG2076)→
Efa-130-1	NZ_KB949218.1	+	79886	79986	**RNA**→	PRK11431 (PRK11431)EmrE (COG2076)→	PRK11431 (PRK11431)EmrE (COG2076)→
Efa-135-1	NZ_KB949378.1	+	79893	79993	**RNA**→	PRK11431 (PRK11431)EmrE (COG2076)→	PRK11431 (PRK11431)EmrE (COG2076)→
Efa-80-1	NZ_KB932521.1	+	80073	80173	**RNA**→	PRK11431 (PRK11431)EmrE (COG2076)→	PRK11431 (PRK11431)EmrE (COG2076)→
Efa-129-1	NZ_KB949162.1	+	691551	691651	**RNA**→	PRK11431 (PRK11431)EmrE (COG2076)→	PRK11431 (PRK11431)EmrE (COG2076)→
Efa-67-1	NZ_KB932468.1	+	72717	72817	**RNA**→	PRK11431 (PRK11431)EmrE (COG2076)→	PRK11431 (PRK11431)EmrE (COG2076)→
Efa-122-1	NZ_KB948947.1	+	78221	78321	**RNA**→	PRK11431 (PRK11431)EmrE (COG2076)→	PRK11431 (PRK11431)EmrE (COG2076)→
Efa-1-3	NZ_CP021161.1	+	367546	367646	**RNA**→	PRK11431 (PRK11431)EmrE (COG2076)→	PRK11431 (PRK11431)EmrE (COG2076)→
Efa-6-1	NZ_ASWX01000003.1	-	146627	146527	**RNA**→	PRK11431 (PRK11431)EmrE (COG2076)→	PRK11431 (PRK11431)EmrE (COG2076)→
Efa-260-1	NZ_AYKM01000002.1	-	250194	250094	**RNA**→	PRK11431 (PRK11431)EmrE (COG2076)→	PRK11431 (PRK11431)EmrE (COG2076)→
Efa-182-1	NZ_AOPW01000026.1	+	2830	2930	**RNA**→	PRK11431 (PRK11431)EmrE (COG2076)→	PRK11431 (PRK11431)EmrE (COG2076)→
Efa-258-1	NZ_AGVN01000067.1	+	5100	5200	**RNA**→	PRK11431 (PRK11431)EmrE (COG2076)→	PRK11431 (PRK11431)EmrE (COG2076)→
Efa-265-1	NZ_AFHH01000033.1	-	129244	129144	**RNA**→	PRK11431 (PRK11431)EmrE (COG2076)→	PRK11431 (PRK11431)EmrE (COG2076)→
env-273	DIJO01000008.1	+	37135	37235	**RNA**→	PRK11431 (PRK11431)EmrE (COG2076)→	PRK11431 (PRK11431)EmrE (COG2076)→
Efa-3-1	NZ_KE350810.1	-	89445	89345	**RNA**→	PRK11431 (PRK11431)EmrE (COG2076)→	PRK11431 (PRK11431)EmrE (COG2076)→
Efa-290-1	NZ_KE352657.1	-	122516	122416	**RNA**→	PRK11431 (PRK11431)EmrE (COG2076)→	PRK11431 (PRK11431)EmrE (COG2076)→
Lmo-1-1	NZ_FFHQ01000002.1	-	362945	362845	**RNA**→	PRK11431 (PRK11431)EmrE (COG2076)→	PRK11431 (PRK11431)EmrE (COG2076)→
env-274	DGLK01000038.1	-	37770	37670	**RNA**→	PRK11431 (PRK11431)EmrE (COG2076)→	PRK11431 (PRK11431)EmrE (COG2076)→
env-275	DHAF01000013.1	-	327953	327853	**RNA**→	PRK11431 (PRK11431)EmrE (COG2076)→	PRK11431 (PRK11431)EmrE (COG2076)→

env-276	DLSP01000017.1	-	21099	20999	**RNA**→ PRK11431 (PRK11431)EmrE (COG2076)→ PRK11431 (PRK11431)EmrE (COG2076)→
env-277	Ga0120771_102603	+	633	733	**RNA**→ PRK11431 (PRK11431)EmrE (COG2076)→ EmrE (COG2076)→
env-278	Ga0121731_100081	-	23179	23079	**RNA**→ PRK11431 (PRK11431)EmrE (COG2076)→ PRK11431 (PRK11431)EmrE (COG2076)→
env-279	Ga0122302_100132	-	24627	24527	**RNA**→ PRK11431 (PRK11431)EmrE (COG2076)→ PRK11431 (PRK11431)EmrE (COG2076)→
env-280	Ga0121056_100194	-	5721	5621	**RNA**→ PRK11431 (PRK11431)EmrE (COG2076)→ PRK11431 (PRK11431)EmrE (COG2076)→
env-281	Ga0169887_10007	-	120477	120377	**RNA**→ PRK11431 (PRK11431)EmrE (COG2076)→ PRK11431 (PRK11431)EmrE (COG2076)→
env-282	Ga0169743_10018	-	120059	119959	**RNA**→ PRK11431 (PRK11431)EmrE (COG2076)→ PRK11431 (PRK11431)EmrE (COG2076)→
env-283	Ga0169886_100007	-	118574	118474	**RNA**→ PRK11431 (PRK11431)EmrE (COG2076)→ PRK11431 (PRK11431)EmrE (COG2076)→
env-284	Ga0169770_10024	-	142559	142459	**RNA**→ PRK11431 (PRK11431)EmrE (COG2076)→ PRK11431 (PRK11431)EmrE (COG2076)→
env-285	Ga0121369_100005	-	380209	380109	**RNA**→ PRK11431 (PRK11431)EmrE (COG2076)→ PRK11431 (PRK11431)EmrE (COG2076)→
env-286	Pasolli2019-7962-4	+	37222	37322	**RNA**→ EmrE (COG2076)PRK11431 (PRK11431)→ EmrE (COG2076)PRK11431 (PRK11431)→
Efa-315-1	NZ_GG739867.1	+	19822	19922	**RNA**→ PRK11431 (PRK11431)EmrE (COG2076)→ PRK11431 (PRK11431)EmrE (COG2076)→
Efa-316-1	NZ_GG739732.1	-	43689	43589	**RNA**→ PRK11431 (PRK11431)EmrE (COG2076)→ PRK11431 (PRK11431)EmrE (COG2076)→
Efa-306-1	NZ_GG692880.1	-	633183	633083	**RNA**→ PRK11431 (PRK11431)EmrE (COG2076)→ PRK11431 (PRK11431)EmrE (COG2076)→<br>PRK11431 (PRK11431)EmrE (COG2076)→ PRK11431 (PRK11431)EmrE (COG2076)→
Efa-312-1	NZ_GG692918.1	+	38580	38680	**RNA**→ PRK11431 (PRK11431)EmrE (COG2076)→ PRK11431 (PRK11431)EmrE (COG2076)→<br>PRK11431 (PRK11431)EmrE (COG2076)→ PRK11431 (PRK11431)EmrE (COG2076)→
Efa-311-1	NZ_GG692707.1	+	37760	37860	**RNA**→ PRK11431 (PRK11431)EmrE (COG2076)→ PRK11431 (PRK11431)EmrE (COG2076)→<br>PRK11431 (PRK11431)EmrE (COG2076)→ PRK11431 (PRK11431)EmrE (COG2076)→
Efa-289-1	NZ_GL454710.1	-	244327	244227	**RNA**→ PRK11431 (PRK11431)EmrE (COG2076)→ PRK11431 (PRK11431)EmrE (COG2076)→
Efa-271-1	NZ_GL454383.1	-	143579	143479	**RNA**→ PRK11431 (PRK11431)EmrE (COG2076)→ PRK11431 (PRK11431)EmrE (COG2076)→
Efa-25-1	NZ_GL455713.1	-	65269	65169	**RNA**→ PRK11431 (PRK11431)EmrE (COG2076)→ PRK11431 (PRK11431)EmrE (COG2076)→
Efa-270-1	NZ_GL454195.1	-	118399	118299	**RNA**→ PRK11431 (PRK11431)EmrE (COG2076)→ PRK11431 (PRK11431)EmrE (COG2076)→
Efa-24-1	NZ_GL455798.1	-	65269	65169	**RNA**→ PRK11431 (PRK11431)EmrE (COG2076)→ PRK11431 (PRK11431)EmrE (COG2076)→
Efa-326-1	NZ_GL454422.1	+	1801	1901	**RNA**→ PRK11431 (PRK11431)EmrE (COG2076)→ PRK11431 (PRK11431)EmrE (COG2076)→
Efa-188-1	NZ_KB944998.1	+	40924	41024	**RNA**→ PRK11431 (PRK11431)EmrE (COG2076)→ PRK11431 (PRK11431)EmrE (COG2076)→
Efa-185-1	NZ_KB944969.1	+	40800	40900	**RNA**→ PRK11431 (PRK11431)EmrE (COG2076)→ PRK11431 (PRK11431)EmrE (COG2076)→
Efa-99-1	NZ_KB932596.1	+	36908	37008	**RNA**→ PRK11431 (PRK11431)EmrE (COG2076)→ PRK11431 (PRK11431)EmrE (COG2076)→
Efa-13-1	NZ_KB944872.1	+	37321	37421	**RNA**→ PRK11431 (PRK11431)EmrE (COG2076)→ PRK11431 (PRK11431)EmrE (COG2076)→
Efa-186-1	NZ_KB945011.1	+	35629	35729	**RNA**→ PRK11431 (PRK11431)EmrE (COG2076)→ PRK11431 (PRK11431)EmrE (COG2076)→
Efa-184-1	NZ_KB945020.1	+	40646	40746	**RNA**→ PRK11431 (PRK11431)EmrE (COG2076)→ PRK11431 (PRK11431)EmrE (COG2076)→
Efa-86-1	NZ_KB932584.1	+	44265	44365	**RNA**→ PRK11431 (PRK11431)EmrE (COG2076)→ PRK11431 (PRK11431)EmrE (COG2076)→
Efa-87-1	NZ_KB932588.1	+	36705	36805	**RNA**→ PRK11431 (PRK11431)EmrE (COG2076)→ PRK11431 (PRK11431)EmrE (COG2076)→
Efa-84-1	NZ_KB932576.1	+	42905	43005	**RNA**→ PRK11431 (PRK11431)EmrE (COG2076)→ PRK11431 (PRK11431)EmrE (COG2076)→
Efa-85-1	NZ_KB932580.1	+	45772	45872	**RNA**→ PRK11431 (PRK11431)EmrE (COG2076)→ PRK11431 (PRK11431)EmrE (COG2076)→
Efa-98-1	NZ_KB932592.1	+	40761	40861	**RNA**→ PRK11431 (PRK11431)EmrE (COG2076)→ PRK11431 (PRK11431)EmrE (COG2076)→
Efa-100-1	NZ_KB932600.1	+	42670	42770	**RNA**→ PRK11431 (PRK11431)EmrE (COG2076)→ PRK11431 (PRK11431)EmrE (COG2076)→
Efa-162-1	NZ_KB947461.1	+	42681	42781	**RNA**→ PRK11431 (PRK11431)EmrE (COG2076)→ PRK11431 (PRK11431)EmrE (COG2076)→
Efa-165-1	NZ_KB947438.1	+	42613	42713	**RNA**→ PRK11431 (PRK11431)EmrE (COG2076)→ PRK11431 (PRK11431)EmrE (COG2076)→
Efa-168-1	NZ_KB947418.1	+	41617	41717	**RNA**→ PRK11431 (PRK11431)EmrE (COG2076)→ PRK11431 (PRK11431)EmrE (COG2076)→
Efa-14-1	NZ_KB947427.1	+	42657	42757	**RNA**→ PRK11431 (PRK11431)EmrE (COG2076)→ PRK11431 (PRK11431)EmrE (COG2076)→
Efa-166-1	NZ_KB947485.1	+	41160	41260	**RNA**→ PRK11431 (PRK11431)EmrE (COG2076)→ PRK11431 (PRK11431)EmrE (COG2076)→
Efa-15-1	NZ_KB947434.1	+	37236	37336	**RNA**→ PRK11431 (PRK11431)EmrE (COG2076)→ PRK11431 (PRK11431)EmrE (COG2076)→
Efa-23-1	NZ_KE351058.1	-	256034	255934	**RNA**→ PRK11431 (PRK11431)EmrE (COG2076)→ PRK11431 (PRK11431)EmrE (COG2076)→
Efa-226-1	NZ_KI912957.1	+	42400	42500	**RNA**→ PRK11431 (PRK11431)EmrE (COG2076)→ PRK11431 (PRK11431)EmrE (COG2076)→
Efa-233-1	NZ_KI913002.1	+	150047	150147	**RNA**→ PRK11431 (PRK11431)EmrE (COG2076)→ PRK11431 (PRK11431)EmrE (COG2076)→
Efa-240-1	NZ_KI913051.1	+	268486	268586	**RNA**→ PRK11431 (PRK11431)EmrE (COG2076)→ PRK11431 (PRK11431)EmrE (COG2076)→
Efa-234-1	NZ_KI913011.1	+	315493	315593	**RNA**→ PRK11431 (PRK11431)EmrE (COG2076)→ PRK11431 (PRK11431)EmrE (COG2076)→
Efa-229-1	NZ_KI912975.1	+	38068	38168	**RNA**→ PRK11431 (PRK11431)EmrE (COG2076)→ PRK11431 (PRK11431)EmrE (COG2076)→
Efa-243-1	NZ_KI913061.1	+	37976	38076	**RNA**→ PRK11431 (PRK11431)EmrE (COG2076)→ PRK11431 (PRK11431)EmrE (COG2076)→
Efa-228-1	NZ_KI912967.1	+	41159	41259	**RNA**→ PRK11431 (PRK11431)EmrE (COG2076)→ PRK11431 (PRK11431)EmrE (COG2076)→
Efa-227-1	NZ_KI912960.1	+	42302	42402	**RNA**→ PRK11431 (PRK11431)EmrE (COG2076)→ PRK11431 (PRK11431)EmrE (COG2076)→
Efa-245-1	NZ_KI913083.1	-	2073067	2072967	**RNA**→ PRK11431 (PRK11431)EmrE (COG2076)→ PRK11431 (PRK11431)EmrE (COG2076)→

Efa-232-1	NZ_KI912991.1	+	35629	35729	**RNA**→ PRK11431 (PRK11431)EmrE (COG2076)→ PRK11431 (PRK11431)EmrE (COG2076)→
Efa-237-1	NZ_KI913025.1	+	41819	41919	**RNA**→ PRK11431 (PRK11431)EmrE (COG2076)→ PRK11431 (PRK11431)EmrE (COG2076)→
Efa-230-1	NZ_KI912980.1	+	43127	43227	**RNA**→ PRK11431 (PRK11431)EmrE (COG2076)→ PRK11431 (PRK11431)EmrE (COG2076)→
Efa-163-1	NZ_AJET01000004.1	+	35817	35917	**RNA**→ PRK11431 (PRK11431)EmrE (COG2076)→ PRK11431 (PRK11431)EmrE (COG2076)→
Efa-314-1	NZ_ADKN01000053.1	+	5088	5188	**RNA**→ PRK11431 (PRK11431)EmrE (COG2076)→ PRK11431 (PRK11431)EmrE (COG2076)→ PRK11431 (PRK11431)EmrE (COG2076)→ PRK11431 (PRK11431)EmrE (COG2076)→
env-287	DJUF01000022.1	-	119817	119717	**RNA**→ PRK11431 (PRK11431)EmrE (COG2076)→ PRK11431 (PRK11431)EmrE (COG2076)→
env-288	DLJC01000014.1	+	37135	37235	**RNA**→ PRK11431 (PRK11431)EmrE (COG2076)→ PRK11431 (PRK11431)EmrE (COG2076)→
env-289	DLFP01000134.1	-	18511	18411	**RNA**→ PRK11431 (PRK11431)EmrE (COG2076)→ PRK11431 (PRK11431)EmrE (COG2076)→
env-290	Ga0122174_110072	-	374	274	**RNA**→ PRK11431 (PRK11431)EmrE (COG2076)→
env-291	Ga0169866_103793	-	2428	2328	**RNA**→ PRK11431 (PRK11431)EmrE (COG2076)→ PRK11431 (PRK11431)EmrE (COG2076)→
env-292	Ga0121318_100027	+	39265	39365	**RNA**→ PRK11431 (PRK11431)EmrE (COG2076)→ PRK11431 (PRK11431)EmrE (COG2076)→
env-293	Ga0169826_100060	+	1791	1891	**RNA**→ PRK11431 (PRK11431)EmrE (COG2076)→ PRK11431 (PRK11431)EmrE (COG2076)→
Efa-305-1	NZ_GG688628.1	+	38691	38791	**RNA**→ PRK11431 (PRK11431)EmrE (COG2076)→ PRK11431 (PRK11431)EmrE (COG2076)→ PRK11431 (PRK11431)EmrE (COG2076)→ PRK11431 (PRK11431)EmrE (COG2076)→
Efa-302-1	NZ_GG670377.1	+	37294	37394	**RNA**→ PRK11431 (PRK11431)EmrE (COG2076)→ PRK11431 (PRK11431)EmrE (COG2076)→ PRK11431 (PRK11431)EmrE (COG2076)→ PRK11431 (PRK11431)EmrE (COG2076)→
Efa-287-1	NZ_GL454116.1	-	119456	119356	**RNA**→ PRK11431 (PRK11431)EmrE (COG2076)→ PRK11431 (PRK11431)EmrE (COG2076)→
Efa-325-1	NZ_GL455601.1	-	55846	55746	**RNA**→ PRK11431 (PRK11431)EmrE (COG2076)→ PRK11431 (PRK11431)EmrE (COG2076)→
Efa-46-1	NZ_KB932382.1	-	720252	720152	**RNA**→ PRK11431 (PRK11431)EmrE (COG2076)→ PRK11431 (PRK11431)EmrE (COG2076)→
Efa-216-1	NZ_KB944657.1	+	44394	44494	**RNA**→ PRK11431 (PRK11431)EmrE (COG2076)→ PRK11431 (PRK11431)EmrE (COG2076)→
Efa-203-1	NZ_KB944662.1	+	44519	44619	**RNA**→ PRK11431 (PRK11431)EmrE (COG2076)→ PRK11431 (PRK11431)EmrE (COG2076)→
Efa-65-1	NZ_KB932394.1	-	720871	720771	**RNA**→ PRK11431 (PRK11431)EmrE (COG2076)→ PRK11431 (PRK11431)EmrE (COG2076)→
Efa-68-1	NZ_KB932561.1	+	49484	49584	**RNA**→ PRK11431 (PRK11431)EmrE (COG2076)→ PRK11431 (PRK11431)EmrE (COG2076)→
Efa-44-1	NZ_KB932357.1	+	40037	40137	**RNA**→ PRK11431 (PRK11431)EmrE (COG2076)→ PRK11431 (PRK11431)EmrE (COG2076)→
Efa-59-1	NZ_KB932343.1	+	44403	44503	**RNA**→ PRK11431 (PRK11431)EmrE (COG2076)→ PRK11431 (PRK11431)EmrE (COG2076)→
Efa-58-1	NZ_KB932353.1	+	38766	38866	**RNA**→ PRK11431 (PRK11431)EmrE (COG2076)→ PRK11431 (PRK11431)EmrE (COG2076)→
Efa-54-1	NZ_KB932402.1	+	38192	38292	**RNA**→ PRK11431 (PRK11431)EmrE (COG2076)→ PRK11431 (PRK11431)EmrE (COG2076)→
Efa-57-1	NZ_KB932349.1	-	579489	579389	**RNA**→ PRK11431 (PRK11431)EmrE (COG2076)→ PRK11431 (PRK11431)EmrE (COG2076)→
Efa-64-1	NZ_KB932390.1	+	43820	43920	**RNA**→ PRK11431 (PRK11431)EmrE (COG2076)→ PRK11431 (PRK11431)EmrE (COG2076)→
Efa-56-1	NZ_KB932362.1	+	35651	35751	**RNA**→ PRK11431 (PRK11431)EmrE (COG2076)→ PRK11431 (PRK11431)EmrE (COG2076)→
Efa-62-1	NZ_KB932372.1	-	2627025	2626925	**RNA**→ PRK11431 (PRK11431)EmrE (COG2076)→ PRK11431 (PRK11431)EmrE (COG2076)→
Efa-170-1	NZ_KB947399.1	+	41218	41318	**RNA**→ PRK11431 (PRK11431)EmrE (COG2076)→ PRK11431 (PRK11431)EmrE (COG2076)→
Efa-160-1	NZ_KB947502.1	+	35483	35583	**RNA**→ PRK11431 (PRK11431)EmrE (COG2076)→ PRK11431 (PRK11431)EmrE (COG2076)→
Efa-4-1	NZ_KE351278.1	-	182670	182570	**RNA**→ PRK11431 (PRK11431)EmrE (COG2076)→ PRK11431 (PRK11431)EmrE (COG2076)→
Efa-5-1	NZ_KE351231.1	+	36337	36437	**RNA**→ PRK11431 (PRK11431)EmrE (COG2076)→ PRK11431 (PRK11431)EmrE (COG2076)→
Efa-292-1	NZ_KE352626.1	-	274123	274023	**RNA**→ PRK11431 (PRK11431)EmrE (COG2076)→ PRK11431 (PRK11431)EmrE (COG2076)→
Efa-21-1	NZ_KI518252.1	+	44230	44330	**RNA**→ PRK11431 (PRK11431)EmrE (COG2076)→ PRK11431 (PRK11431)EmrE (COG2076)→
Efa-239-1	NZ_KI913043.1	+	41222	41322	**RNA**→ PRK11431 (PRK11431)EmrE (COG2076)→ PRK11431 (PRK11431)EmrE (COG2076)→
Efa-224-1	NZ_KI912926.1	+	321882	321982	**RNA**→ PRK11431 (PRK11431)EmrE (COG2076)→ PRK11431 (PRK11431)EmrE (COG2076)→
Efa-244-1	NZ_KI913069.1	+	38756	38856	**RNA**→ PRK11431 (PRK11431)EmrE (COG2076)→ PRK11431 (PRK11431)EmrE (COG2076)→
Efa-247-1	NZ_KI913087.1	+	37753	37853	**RNA**→ PRK11431 (PRK11431)EmrE (COG2076)→ PRK11431 (PRK11431)EmrE (COG2076)→
Efa-17-1	NZ_CP008816.1	+	2394431	2394531	**RNA**→ PRK11431 (PRK11431)EmrE (COG2076)→ PRK11431 (PRK11431)EmrE (COG2076)→
Efa-10-1	NZ_JH805632.1	-	116993	116893	**RNA**→ PRK11431 (PRK11431)EmrE (COG2076)→ PRK11431 (PRK11431)EmrE (COG2076)→
Efa-1-4	NZ_CP018102.1	+	295433	295533	**RNA**→ PRK11431 (PRK11431)EmrE (COG2076)→ PRK11431 (PRK11431)EmrE (COG2076)→
Efa-63-1	NZ_KB932388.1	+	44145	44245	**RNA**→ PRK11431 (PRK11431)EmrE (COG2076)→ PRK11431 (PRK11431)EmrE (COG2076)→
Efa-55-1	NZ_KB932408.1	+	40412	40512	**RNA**→ PRK11431 (PRK11431)EmrE (COG2076)→ PRK11431 (PRK11431)EmrE (COG2076)→
Efa-300-1	NC_017312.1	+	711975	712075	**RNA**→ PRK11431 (PRK11431)EmrE (COG2076)→ PRK11431 (PRK11431)EmrE (COG2076)→
Efa-26-1	NZ_AWPI01000051.1	-	100278	100178	**RNA**→ PRK11431 (PRK11431)EmrE (COG2076)→ PRK11431 (PRK11431)EmrE (COG2076)→
env-294	DOOO01000156.1	+	5485	5585	**RNA**→ Multi_Drug_Res (pfam00893)→ PRK11431 (PRK11431)EmrE (COG2076)→
env-295	DQAP01000360.1	-	659	559	**RNA**→ PRK11431 (PRK11431)EmrE (COG2076)→ PRK11431 (PRK11431)EmrE (COG2076)→
env-296	DGFT01000096.1	+	35603	35703	**RNA**→ PRK11431 (PRK11431)EmrE (COG2076)→ PRK11431 (PRK11431)EmrE (COG2076)→
env-297	DIHA01000009.1	+	37429	37529	**RNA**→ PRK11431 (PRK11431)EmrE (COG2076)→ PRK11431 (PRK11431)EmrE (COG2076)→

env-298	DJFJ01000004.1	+	37573	37673	**RNA**→ PRK11431 (PRK11431)EmrE (COG2076)→ PRK11431 (PRK11431)EmrE (COG2076)→
env-299	OIWP01035963.1	+	99	199	**RNA**→ PRK11431 (PRK11431)EmrE (COG2076)→ PRK11431 (PRK11431)EmrE (COG2076)→
env-300	Ga0101111_1000579	-	15047	14947	**RNA**→ PRK11431 (PRK11431)EmrE (COG2076)→ PRK11431 (PRK11431)EmrE (COG2076)→
env-301	Ga0121217_10018	+	37560	37660	**RNA**→ PRK11431 (PRK11431)EmrE (COG2076)→ PRK11431 (PRK11431)EmrE (COG2076)→
env-302	Ga0120980_100656	-	804	704	**RNA**→ PRK11431 (PRK11431)EmrE (COG2076)→ PRK11431 (PRK11431)EmrE (COG2076)→
env-303	Ga0121011_100839	+	1288	1388	**RNA**→ PRK11431 (PRK11431)EmrE (COG2076)→ EmrE (COG2076)→
env-304	Ga0122016_10377	-	3451	3351	**RNA**→ PRK11431 (PRK11431)EmrE (COG2076)→ PRK11431 (PRK11431)EmrE (COG2076)→
env-305	Ga0121332_10829	-	1344	1244	**RNA**→ PRK11431 (PRK11431)EmrE (COG2076)→ PRK11431 (PRK11431)EmrE (COG2076)→
env-306	Ga0121357_100008	-	354581	354481	**RNA**→ PRK11431 (PRK11431)EmrE (COG2076)→ PRK11431 (PRK11431)EmrE (COG2076)→
env-307	Ga0122841_100082	-	1995	1895	**RNA**→ PRK11431 (PRK11431)EmrE (COG2076)→ PRK11431 (PRK11431)EmrE (COG2076)→
Efa-320-1	NZ_GG670365.1	+	38293	38393	**RNA**→ PRK11431 (PRK11431)EmrE (COG2076)→ PRK11431 (PRK11431)EmrE (COG2076)→ PRK11431 (PRK11431)EmrE (COG2076)→ PRK11431 (PRK11431)EmrE (COG2076)→
Efa-307-1	NZ_GG692679.1	+	38303	38403	**RNA**→ PRK11431 (PRK11431)EmrE (COG2076)→ PRK11431 (PRK11431)EmrE (COG2076)→ PRK11431 (PRK11431)EmrE (COG2076)→ PRK11431 (PRK11431)EmrE (COG2076)→
Efa-318-1	NZ_GG688642.1	+	37117	37217	**RNA**→ PRK11431 (PRK11431)EmrE (COG2076)→ PRK11431 (PRK11431)EmrE (COG2076)→ PRK11431 (PRK11431)EmrE (COG2076)→ PRK11431 (PRK11431)EmrE (COG2076)→
Efa-310-1	NZ_GG692639.1	+	37338	37438	**RNA**→ PRK11431 (PRK11431)EmrE (COG2076)→ PRK11431 (PRK11431)EmrE (COG2076)→ PRK11431 (PRK11431)EmrE (COG2076)→ PRK11431 (PRK11431)EmrE (COG2076)→
Efa-301-1	NZ_GG692906.1	+	38608	38708	**RNA**→ PRK11431 (PRK11431)EmrE (COG2076)→ PRK11431 (PRK11431)EmrE (COG2076)→ PRK11431 (PRK11431)EmrE (COG2076)→ PRK11431 (PRK11431)EmrE (COG2076)→
Efa-285-1	NZ_GL454582.1	-	330757	330657	**RNA**→ PRK11431 (PRK11431)EmrE (COG2076)→ PRK11431 (PRK11431)EmrE (COG2076)→
Efa-323-1	NZ_GL455653.1	+	36174	36274	**RNA**→ PRK11431 (PRK11431)EmrE (COG2076)→ PRK11431 (PRK11431)EmrE (COG2076)→
Efa-280-1	NZ_GL456536.1	-	69091	68991	**RNA**→ PRK11431 (PRK11431)EmrE (COG2076)→ PRK11431 (PRK11431)EmrE (COG2076)→
Efa-196-1	NZ_KB944832.1	+	38183	38283	**RNA**→ PRK11431 (PRK11431)EmrE (COG2076)→ PRK11431 (PRK11431)EmrE (COG2076)→
Efa-217-1	NZ_KB944687.1	+	43247	43347	**RNA**→ PRK11431 (PRK11431)EmrE (COG2076)→ hypo→ hypo→ EmrE (COG2076)→ hypo→
Efa-208-1	NZ_KB944758.1	+	42970	43070	**RNA**→ PRK11431 (PRK11431)EmrE (COG2076)→ PRK11431 (PRK11431)EmrE (COG2076)→
Efa-220-1	NZ_KB944806.1	+	38086	38186	**RNA**→ PRK11431 (PRK11431)EmrE (COG2076)→ hypo→ hypo→ EmrE (COG2076)→ hypo→
Efa-198-1	NZ_KB944693.1	+	41571	41671	**RNA**→ PRK11431 (PRK11431)EmrE (COG2076)→ hypo→ hypo→ EmrE (COG2076)→ hypo→
Efa-212-1	NZ_KB944824.1	+	320123	320223	**RNA**→ PRK11431 (PRK11431)EmrE (COG2076)→ PRK11431 (PRK11431)EmrE (COG2076)→
Efa-207-1	NZ_KB944724.1	+	41655	41755	**RNA**→ PRK11431 (PRK11431)EmrE (COG2076)→ PRK11431 (PRK11431)EmrE (COG2076)→
Efa-200-1	NZ_KB944577.1	+	40746	40846	**RNA**→ PRK11431 (PRK11431)EmrE (COG2076)→ PRK11431 (PRK11431)EmrE (COG2076)→
Efa-152-1	NZ_KB946664.1	+	45622	45722	**RNA**→ PRK11431 (PRK11431)EmrE (COG2076)→ PRK11431 (PRK11431)EmrE (COG2076)→
Efa-175-1	NZ_KB946765.1	+	37090	37190	**RNA**→ PRK11431 (PRK11431)EmrE (COG2076)→ PRK11431 (PRK11431)EmrE (COG2076)→
Efa-16-1	NZ_KB947318.1	+	44263	44363	**RNA**→ PRK11431 (PRK11431)EmrE (COG2076)→ PRK11431 (PRK11431)EmrE (COG2076)→
Efa-161-1	NZ_KB947403.1	+	43143	43243	**RNA**→ PRK11431 (PRK11431)EmrE (COG2076)→ PRK11431 (PRK11431)EmrE (COG2076)→
Efa-2-1	NZ_KE350939.1	+	9613	9713	**RNA**→ PRK11431 (PRK11431)EmrE (COG2076)→ PRK11431 (PRK11431)EmrE (COG2076)→
Efa-236-1	NZ_KI913020.1	+	39534	39634	**RNA**→ PRK11431 (PRK11431)EmrE (COG2076)→ PRK11431 (PRK11431)EmrE (COG2076)→
Efa-231-1	NZ_KI912988.1	+	327872	327972	**RNA**→ PRK11431 (PRK11431)EmrE (COG2076)→ PRK11431 (PRK11431)EmrE (COG2076)→
Efa-242-1	NZ_KI913060.1	+	325758	325858	**RNA**→ PRK11431 (PRK11431)EmrE (COG2076)→ PRK11431 (PRK11431)EmrE (COG2076)→
Efa-241-1	NZ_KI913056.1	+	36159	36259	**RNA**→ PRK11431 (PRK11431)EmrE (COG2076)→ PRK11431 (PRK11431)EmrE (COG2076)→
Efa-246-1	NZ_KI913085.1	+	44477	44577	**RNA**→ PRK11431 (PRK11431)EmrE (COG2076)→ PRK11431 (PRK11431)EmrE (COG2076)→
Efa-43-1	NZ_KL584700.1	+	33220	33320	**RNA**→ PRK11431 (PRK11431)EmrE (COG2076)→ PRK11431 (PRK11431)EmrE (COG2076)→
Efa-268-1	NZ_KE352746.1	-	247787	247687	**RNA**→ PRK11431 (PRK11431)EmrE (COG2076)→ PRK11431 (PRK11431)EmrE (COG2076)→
Efa-267-1	NZ_KE351857.1	-	234586	234486	**RNA**→ PRK11431 (PRK11431)EmrE (COG2076)→ PRK11431 (PRK11431)EmrE (COG2076)→
Efa-257-1	NZ_KE352840.1	-	127477	127377	**RNA**→ PRK11431 (PRK11431)EmrE (COG2076)→ PRK11431 (PRK11431)EmrE (COG2076)→
Efa-1-5	NZ_CP018004.1	+	260900	261000	**RNA**→ PRK11431 (PRK11431)EmrE (COG2076)→ PRK11431 (PRK11431)EmrE (COG2076)→
Efa-1-6	NZ_CP015998.1	+	2200623	2200723	**RNA**→ PRK11431 (PRK11431)EmrE (COG2076)→ PRK11431 (PRK11431)EmrE (COG2076)→
Esp-8-1	NZ_KV811994.1	-	248650	248550	**RNA**→ PRK11431 (PRK11431)EmrE (COG2076)→ PRK11431 (PRK11431)EmrE (COG2076)→
Efa-1-7	NZ_CP015883.1	+	406859	406959	**RNA**→ PRK11431 (PRK11431)EmrE (COG2076)→ PRK11431 (PRK11431)EmrE (COG2076)→
Efa-16-2	NZ_GG668756.1	+	38701	38801	**RNA**→ PRK11431 (PRK11431)EmrE (COG2076)→ PRK11431 (PRK11431)EmrE (COG2076)→
Efa-12-1	NZ_CP022488.1	+	256913	257013	**RNA**→ PRK11431 (PRK11431)EmrE (COG2076)→ PRK11431 (PRK11431)EmrE (COG2076)→
Efa-263-1	NZ_AYOK01000125.1	-	94431	94331	**RNA**→ PRK11431 (PRK11431)EmrE (COG2076)→ PRK11431 (PRK11431)EmrE (COG2076)→
Efa-251-1	NZ_AYKK01000003.1	-	89042	88942	**RNA**→ PRK11431 (PRK11431)EmrE (COG2076)→ PRK11431 (PRK11431)EmrE (COG2076)→

Esp-1-1	NZ_JWBQ01000023.1	-	92127	92027	**RNA**→ PRK11431 (PRK11431)EmrE (COG2076)→ PRK11431 (PRK11431)EmrE (COG2076)→
env-308	DJUO01000012.1	+	35980	36080	**RNA**→ PRK11431 (PRK11431)EmrE (COG2076)→ PRK11431 (PRK11431)EmrE (COG2076)→
env-309	DGRG01000017.1	+	37142	37242	**RNA**→ PRK11431 (PRK11431)EmrE (COG2076)→ PRK11431 (PRK11431)EmrE (COG2076)→
Efa-322-1	NZ_GG668945.1	-	3650	3550	**RNA**→ PRK11431 (PRK11431)EmrE (COG2076)→ PRK11431 (PRK11431)EmrE (COG2076)→
Efa-308-1	NZ_GG692665.1	+	42493	42593	**RNA**→ PRK11431 (PRK11431)EmrE (COG2076)→ PRK11431 (PRK11431)EmrE (COG2076)→
					PRK11431 (PRK11431)EmrE (COG2076)→ PRK11431 (PRK11431)EmrE (COG2076)→
Efa-33-1	NZ_JH805943.1	-	3681	3581	**RNA**→ PRK11431 (PRK11431)EmrE (COG2076)→ PRK11431 (PRK11431)EmrE (COG2076)→
Efa-29-1	NZ_JH806266.1	+	36266	36366	**RNA**→ PRK11431 (PRK11431)EmrE (COG2076)→ PRK11431 (PRK11431)EmrE (COG2076)→
Efa-49-1	NZ_KB932336.1	+	41571	41671	**RNA**→ PRK11431 (PRK11431)EmrE (COG2076)→ PRK11431 (PRK11431)EmrE (COG2076)→
Efa-45-1	NZ_KB932340.1	+	40661	40761	**RNA**→ PRK11431 (PRK11431)EmrE (COG2076)→ PRK11431 (PRK11431)EmrE (COG2076)→
Efa-215-1	NZ_KB944631.1	+	37265	37365	**RNA**→ PRK11431 (PRK11431)EmrE (COG2076)→ PRK11431 (PRK11431)EmrE (COG2076)→
Efa-210-1	NZ_KB944783.1	+	35668	35768	**RNA**→ PRK11431 (PRK11431)EmrE (COG2076)→ PRK11431 (PRK11431)EmrE (COG2076)→
Efa-75-1	NZ_KB932482.1	+	35523	35623	**RNA**→ PRK11431 (PRK11431)EmrE (COG2076)→ PRK11431 (PRK11431)EmrE (COG2076)→
Efa-141-1	NZ_KB946368.1	+	35668	35768	**RNA**→ PRK11431 (PRK11431)EmrE (COG2076)→ PRK11431 (PRK11431)EmrE (COG2076)→
Efa-146-1	NZ_KB946484.1	+	35484	35584	**RNA**→ PRK11431 (PRK11431)EmrE (COG2076)→ PRK11431 (PRK11431)EmrE (COG2076)→
Efa-107-1	NZ_KB948390.1	+	37207	37307	**RNA**→ PRK11431 (PRK11431)EmrE (COG2076)→ PRK11431 (PRK11431)EmrE (COG2076)→
Efa-250-1	NZ_KE351476.1	-	101659	101559	**RNA**→ PRK11431 (PRK11431)EmrE (COG2076)→ PRK11431 (PRK11431)EmrE (COG2076)→
Efa-38-1	NZ_JH805513.1	-	3679	3579	**RNA**→ PRK11431 (PRK11431)EmrE (COG2076)→ PRK11431 (PRK11431)EmrE (COG2076)→
Efa-27-1	NZ_JH805849.1	+	35623	35723	**RNA**→ PRK11431 (PRK11431)EmrE (COG2076)→ PRK11431 (PRK11431)EmrE (COG2076)→
Efa-39-1	NZ_JH805147.1	-	3678	3578	**RNA**→ PRK11431 (PRK11431)EmrE (COG2076)→ PRK11431 (PRK11431)EmrE (COG2076)→
Efa-41-1	NZ_JH804910.1	-	3682	3582	**RNA**→ PRK11431 (PRK11431)EmrE (COG2076)→ PRK11431 (PRK11431)EmrE (COG2076)→
Efa-40-1	NZ_JH805081.1	+	36335	36435	**RNA**→ PRK11431 (PRK11431)EmrE (COG2076)→ PRK11431 (PRK11431)EmrE (COG2076)→
Efa-42-1	NZ_JH804939.1	-	3679	3579	**RNA**→ PRK11431 (PRK11431)EmrE (COG2076)→ PRK11431 (PRK11431)EmrE (COG2076)→
Efa-36-1	NZ_JH805430.1	-	3678	3578	**RNA**→ PRK11431 (PRK11431)EmrE (COG2076)→ PRK11431 (PRK11431)EmrE (COG2076)→
Efa-31-1	NZ_JH806439.1	+	35989	36089	**RNA**→ PRK11431 (PRK11431)EmrE (COG2076)→ PRK11431 (PRK11431)EmrE (COG2076)→
Efa-28-1	NZ_JH806052.1	+	36263	36363	**RNA**→ PRK11431 (PRK11431)EmrE (COG2076)→ PRK11431 (PRK11431)EmrE (COG2076)→
Efa-35-1	NZ_JH805673.1	+	36331	36431	**RNA**→ PRK11431 (PRK11431)EmrE (COG2076)→ PRK11431 (PRK11431)EmrE (COG2076)→
Efa-30-1	NZ_JH806320.1	-	3678	3578	**RNA**→ PRK11431 (PRK11431)EmrE (COG2076)→ PRK11431 (PRK11431)EmrE (COG2076)→
Efa-32-1	NZ_JH806113.1	+	1798	1898	**RNA**→ PRK11431 (PRK11431)EmrE (COG2076)→ PRK11431 (PRK11431)EmrE (COG2076)→
Efa-37-1	NZ_JH805299.1	+	1813	1913	**RNA**→ PRK11431 (PRK11431)EmrE (COG2076)→ PRK11431 (PRK11431)EmrE (COG2076)→
Efa-34-1	NZ_JH805812.1	+	1803	1903	**RNA**→ PRK11431 (PRK11431)EmrE (COG2076)→ PRK11431 (PRK11431)EmrE (COG2076)→
Efa-140-1	NZ_KB946340.1	+	40176	40276	**RNA**→ PRK11431 (PRK11431)EmrE (COG2076)→ PRK11431 (PRK11431)EmrE (COG2076)→
Esp-10-1	NZ_KV805535.1	-	110375	110275	**RNA**→ PRK11431 (PRK11431)EmrE (COG2076)→ PRK11431 (PRK11431)EmrE (COG2076)→
Efa-262-1	NZ_AYKU01000020.1	+	19677	19777	**RNA**→ PRK11431 (PRK11431)EmrE (COG2076)→ PRK11431 (PRK11431)EmrE (COG2076)→
env-310	Ga0122765_100658	+	6216	6316	**RNA**→
env-311	Ga0121666_103615	+	1207	1307	**RNA**→ PRK11431 (PRK11431)EmrE (COG2076)→ PRK11431 (PRK11431)EmrE (COG2076)→
env-312	Ga0121235_101749	-	174	74	**RNA**→ hypo→
env-313	Ga0120787_117701	+	24	124	**RNA**→ PRK11431 (PRK11431)EmrE (COG2076)→
env-314	Ga0169827_10008	+	74769	74869	**RNA**→ PRK11431 (PRK11431)EmrE (COG2076)→ PRK11431 (PRK11431)EmrE (COG2076)→
Efa-319-1	NZ_GG692840.1	+	43227	43327	**RNA**→ PRK11431 (PRK11431)EmrE (COG2076)→ PRK11431 (PRK11431)EmrE (COG2076)→
					PRK11431 (PRK11431)EmrE (COG2076)→ PRK11431 (PRK11431)EmrE (COG2076)→
Efa-283-1	NZ_GL455529.1	+	42466	42566	**RNA**→ PRK11431 (PRK11431)EmrE (COG2076)→ PRK11431 (PRK11431)EmrE (COG2076)→
Efa-48-1	NZ_KB932318.1	-	633402	633302	**RNA**→ PRK11431 (PRK11431)EmrE (COG2076)→ PRK11431 (PRK11431)EmrE (COG2076)→
Efa-19-1	NZ_KB944851.1	+	37703	37803	**RNA**→ PRK11431 (PRK11431)EmrE (COG2076)→ PRK11431 (PRK11431)EmrE (COG2076)→
Efa-209-1	NZ_KB944765.1	+	42364	42464	**RNA**→ PRK11431 (PRK11431)EmrE (COG2076)→ PRK11431 (PRK11431)EmrE (COG2076)→
Efa-47-1	NZ_KB932321.1	+	45918	46018	**RNA**→ PRK11431 (PRK11431)EmrE (COG2076)→ PRK11431 (PRK11431)EmrE (COG2076)→
Efa-164-1	NZ_KB947341.1	-	2252165	2252065	**RNA**→ PRK11431 (PRK11431)EmrE (COG2076)→ PRK11431 (PRK11431)EmrE (COG2076)→
Efa-1-8	NZ_CP019512.1	+	270236	270336	**RNA**→ PRK11431 (PRK11431)EmrE (COG2076)→ PRK11431 (PRK11431)EmrE (COG2076)→
Efa-174-1	NZ_KB947346.1	+	49074	49174	**RNA**→ PRK11431 (PRK11431)EmrE (COG2076)→ PRK11431 (PRK11431)EmrE (COG2076)→
env-315	Ga0121387_111314	-	570	470	**RNA**→ PRK11431 (PRK11431)Multi_Drug_Res (pfam00893)→ hypo→
Efa-211-1	NZ_KB944817.1	+	74053	74153	**RNA**→ PRK11431 (PRK11431)EmrE (COG2076)→ PRK11431 (PRK11431)EmrE (COG2076)→
Efa-256-1	NZ_AYND01000079.1	+	1813	1913	**RNA**→ PRK11431 (PRK11431)EmrE (COG2076)→ PRK11431 (PRK11431)EmrE (COG2076)→
Ete-1-1	NZ_MIJY01000045.1	+	125228	125330	**RNA**→ PRK11431 (PRK11431)EmrE (COG2076)→ PRK11431 (PRK11431)EmrE (COG2076)→

Ete-1-2	NZ_JXLF01000005.1	+	125022	125124	**RNA**→ PRK11431 (PRK11431)EmrE (COG2076)→ PRK11431 (PRK11431)EmrE (COG2076)→
Ime-1-1	NZ_FOHA01000014.1	-	61187	61088	**RNA**→ PRK11431 (PRK11431)EmrE (COG2076)→ PRK11431 (PRK11431)EmrE (COG2076)→
Esp-4-1	NZ_NGLE01000003.1	+	558063	558164	**RNA**→ PRK11431 (PRK11431)EmrE (COG2076)→ PRK11431 (PRK11431)EmrE (COG2076)→
Eur-2-1	NZ_MIKC01000003.1	+	46532	46633	**RNA**→ PRK11431 (PRK11431)EmrE (COG2076)→ PRK11431 (PRK11431)EmrE (COG2076)→
Eur-2-2	NZ_NHTL01000009.1	+	45666	45767	**RNA**→ PRK11431 (PRK11431)EmrE (COG2076)→ PRK11431 (PRK11431)EmrE (COG2076)→
Ewa-1-1	NZ_CP021874.1	+	3343929	3344030	**RNA**→ PRK11431 (PRK11431)EmrE (COG2076)→ PRK11431 (PRK11431)EmrE (COG2076)→
Esi-1-1	NZ_JXLC01000028.1	-	11618	11517	**RNA**→ PRK11431 (PRK11431)EmrE (COG2076)→ PRK11431 (PRK11431)EmrE (COG2076)→
Eha-2-1	NZ_KB946315.1	+	48740	48841	**RNA**→ PRK11431 (PRK11431)EmrE (COG2076)→ PRK11431 (PRK11431)EmrE (COG2076)→
Eha-1-1	NZ_JXKS01000014.1	+	42656	42757	**RNA**→ PRK11431 (PRK11431)EmrE (COG2076)→ PRK11431 (PRK11431)EmrE (COG2076)→
Epl-1-1	NZ_MIKA01000011.1	-	30413	30312	**RNA**→ PRK11431 (PRK11431)EmrE (COG2076)→ PRK11431 (PRK11431)EmrE (COG2076)→
Emo-2-1	NZ_KB946320.1	-	109475	109374	**RNA**→ PRK11431 (PRK11431)EmrE (COG2076)→ PRK11431 (PRK11431)EmrE (COG2076)→
Emo-1-1	NZ_JXKW01000002.1	+	339507	339608	**RNA**→ PRK11431 (PRK11431)EmrE (COG2076)→ PRK11431 (PRK11431)EmrE (COG2076)→
Eur-1-1	NZ_MIJZ01000012.1	-	111756	111655	**RNA**→ PRK11431 (PRK11431)EmrE (COG2076)→ PRK11431 (PRK11431)EmrE (COG2076)→
env-316	Ga0256408_1030974	+	119	220	**RNA**→ PRK11431 (PRK11431)EmrE (COG2076)→ PRK11431 (PRK11431)EmrE (COG2076)→
Equ-1-1	NZ_MIKB01000019.1	+	4127	4228	**RNA**→ PRK11431 (PRK11431)EmrE (COG2076)→ PRK11431 (PRK11431)EmrE (COG2076)→
Equ-1-2	NZ_JXKZ01000027.1	+	3949	4050	**RNA**→ PRK11431 (PRK11431)EmrE (COG2076)→ PRK11431 (PRK11431)EmrE (COG2076)→
Eri-1-1	NZ_MIEK01000010.1	+	38595	38697	**RNA**→ PRK11431 (PRK11431)EmrE (COG2076)→ PRK11431 (PRK11431)EmrE (COG2076)→
Csp-19-1	NZ_JTLI01000022.1	+	18011	18112	**RNA**→ hypo→
env-317	Ga0134434_1010213	-	1090	994	**RNA**→ PRK11431 (PRK11431)EmrE (COG2076)→
env-318	SRS016517_C20415349	-	986	890	**RNA**→ PRK11431 (PRK11431)EmrE (COG2076)→
env-319	OJAI01003981.1	-	2569	2473	**RNA**→ PRK11431 (PRK11431)EmrE (COG2076)→
env-320	Pasolli2019-4626-0	-	56393	56297	**RNA**→ EmrE (COG2076)PRK11431 (PRK11431)→
env-321	SRS016495_C1911406	-	413	317	**RNA**→ PRK11431 (PRK11431)EmrE (COG2076)→
Mfa-1-1	NZ_MIEH01000010.1	-	11512	11416	**RNA**→ PRK11431 (PRK11431)EmrE (COG2076)→
env-322	Ga0134378_131545	-	186	90	**RNA**→
env-323	OGGQ01077014.1	-	401	305	**RNA**→ PRK11431 (PRK11431)EmrE (COG2076)→
env-324	UMGS1158-1	-	71265	71169	**RNA**→ EmrE (COG2076)PRK11431 (PRK11431)→
env-325	Ga0256404_1001375	+	57813	57913	**RNA**→ PRK11431 (PRK11431)EmrE (COG2076)→
env-326	Ga0256405_10011097	-	1432	1332	**RNA**→ PRK11431 (PRK11431)EmrE (COG2076)→
env-327	OIYU01000044.1	-	91570	91470	**RNA**→ vmrA (PRK09575)MATE_MepA_like (cd13143)→ GreA (COG0782)greA (PRK00226)→<br>PRK00942 (PRK00942)AAK_NAGK-C (cd04250)→<br>HAMP (smart00304)HATPase_c (smart00387)YesM (COG2972)→<br>HTH_ARAC (smart00342)REC (smart00448)AraC (COG2207)Response_reg (pfam00072)→
env-328	Pasolli2019-4621-6	-	91577	91477	**RNA**→ MATE_MepA_like (cd13143)matE (TIGR00797)→ GreA (COG0782)greA (TIGR01462)→<br>AAK_NAGK-C (cd04250)PLN02512 (PLN02512)→<br>YesM (COG2972)HATPase_c (smart00387)HAMP (smart00304)→<br>Response_reg (pfam00072)AraC (COG2207)HTH_ARAC (smart00342)REC (smart00448)→
env-329	UMGS564-23	-	30926	30826	**RNA**→ MATE_MepA_like (cd13143)matE (TIGR00797)→ GreA (COG0782)greA (TIGR01462)→<br>AAK_NAGK-C (cd04250)PLN02512 (PLN02512)→<br>YesM (COG2972)dCache_1 (pfam02743)HAMP (pfam00672)HATPase_c (smart00387)dCache_3 (pfam14827)HAM<br>Response_reg (pfam00072)AraC (COG2207)HTH_ARAC (smart00342)REC (smart00448)→
env-330	Ga0121678_100043	+	113621	113724	**RNA**→ PRK11431 (PRK11431)EmrE (COG2076)→ hypo→
Cje-1-1	NZ_CBYL010000036.1	-	19992	19889	**RNA**→ PRK11431 (PRK11431)EmrE (COG2076)→
env-331	Ga0169765_113060	-	775	672	**RNA**→ hypo→ PnuC (COG3201)NMN_trans_PnuC (TIGR01528)→
env-332	Pasolli2019-4606-43	+	18816	18919	**RNA**→ hypo→ PnuC (COG3201)NMN_trans_PnuC (TIGR01528)→
env-333	Pasolli2019-6185-3	+	1117	1223	**RNA**→ MATE_MepA_like (cd13143)matE (TIGR00797)→ VOC_like (cd07263)→ hypo→
env-334	UMGS620-8	+	1116	1222	**RNA**→ MATE_MepA_like (cd13143)matE (TIGR00797)→ VOC_like (cd07263)→ hypo→
Cni-1-1	NZ_LT575472.1	+	267049	267155	**RNA**→ vmrA (PRK09575)MATE_MepA_like (cd13143)→ VOC_like (cd07263)→ hypo→
env-335	HCF12C_1_1_newblercontig506168	+	100	186	**RNA**→
Lsa-2-1	NZ_CP020858.1	-	445323	445223	**RNA**→ PRK11431 (PRK11431)EmrE (COG2076)→
env-336	DBQH01000108.1	+	2586	2683	**RNA**→ NorM (COG0534)Polysacc_synt_C (pfam14667)→
env-337	AUXO016001053.1	-	889	787	**RNA**→ PRK11431 (PRK11431)EmrE (COG2076)→
env-338	DFGY01000044.1	+	8867	8969	**RNA**→ PRK11431 (PRK11431)EmrE (COG2076)→

env-339	AUXO011731176.1	-	107	5	**RNA**→
env-340	OIWK01005212.1	+	3451	3550	**RNA**→ PRK11431 (PRK11431)EmrE (COG2076)→
env-341	SRS014613_C1573645	-	1254	1159	**RNA**→ NorM (COG0534)vmrA (PRK09575)→
env-342	Pasolli2019-4274-4	-	90382	90287	**RNA**→ NorM (COG0534)matE (TIGR00797)→
env-343	Ga0208243_1019016	+	1333	1430	**RNA**→
env-344	Ga0075011_10518944	+	181	278	**RNA**→ hypo→
env-345	Ga0194136_1032286	-	1658	1561	**RNA**→ NorM (COG0534)vmrA (PRK09575)→
env-346	Ga0208242_100736	-	6314	6217	**RNA**→ NorM (COG0534)vmrA (PRK09575)→
env-347	Ga0194137_10164627	-	205	108	**RNA**→ hypo→
env-348	Ga0194138_10070084	-	911	814	**RNA**→ NorM (COG0534)vmrA (PRK09575)→
env-349	ONAY01000005.1	-	11614	11518	**RNA**→ EmrE (COG2076)PRK11431 (PRK11431)→
env-350	Ga0255062_10236642	+	333	429	**RNA**→ PRK11431 (PRK11431)EmrE (COG2076)→
env-351	Ga0256405_10003182	+	5917	6013	**RNA**→ PRK11431 (PRK11431)EmrE (COG2076)→
env-352	ONYI01000033.1	-	12012	11916	**RNA**→ EmrE (COG2076)PRK11431 (PRK11431)→
env-353	AUXO014177849.1	+	114	210	**RNA**→ hypo→
env-354	Pasolli2019-4339-114	-	4721	4625	**RNA**→ Pyridox_oxidase (pfam01243)Pyridox_ox_2 (pfam12900)→
env-355	SRS049995_C2539332	-	429	333	**RNA**→ Pyridox_oxidase (pfam01243)Pyridox_ox_2 (pfam12900)→
env-356	SRS078176_C2343304	-	4721	4625	**RNA**→ Pyridox_oxidase (pfam01243)Pyridox_ox_2 (pfam12900)→
env-357	Ga0129310_1150991	+	86	182	**RNA**→ hypo→
env-358	SRS019045_C2619271	-	326	230	**RNA**→ B3/B4 (COG3382)→
env-359	SRS021960_C2106900	-	152	56	**RNA**→
env-360	SRS022621_C3952479	+	271	367	**RNA**→
env-361	SRS045715_LANL_scaffold_92076	+	339	435	**RNA**→
env-362	SRS047824_WUGC_scaffold_22844	+	34	130	**RNA**→ B3_4 (smart00873)B3/B4 (COG3382)→
env-363	Ga0119793_1085243	+	200	296	**RNA**→ B3/B4 (COG3382)→
env-364	Ga0119815_1001973	-	778	682	**RNA**→ B3_4 (smart00873)B3/B4 (COG3382)→
env-365	Pasolli2019-6016-14	-	5708	5612	**RNA**→ B3/B4 (COG3382)B3_4 (smart00873)→
env-366	SRS020340_Baylor_scaffold_20933	+	340	436	**RNA**→ B3/B4 (COG3382)PRK00969 (PRK00969)→
env-367	SRS024447_LANL_scaffold_16049	-	264	168	**RNA**→ B3/B4 (COG3382)→
Fsp-2-1	NZ_KV821747.1	+	592	688	**RNA**→ hypo→
env-368	SRS016319_WUGC_scaffold_42533	-	286	190	**RNA**→ B3/B4 (COG3382)→
env-369	SRS018739_C3698283	+	502	598	**RNA**→ B3_4 (smart00873)B3/B4 (COG3382)→
env-370	SRS019219_WUGC_scaffold_5594	-	479	383	**RNA**→ B3_4 (smart00873)B3/B4 (COG3382)→
env-371	SRS057791_LANL_scaffold_66730	-	1122	1026	**RNA**→ B3_4 (smart00873)B3/B4 (COG3382)→
env-372	SRS019607_WUGC_scaffold_10904	+	1036	1132	**RNA**→
env-373	SRS022143_WUGC_scaffold_19361	-	197	101	**RNA**→
env-374	SRS024381_C2315002	+	18	114	**RNA**→ B3_4 (smart00873)B3/B4 (COG3382)→
env-375	SRS065335_LANL_scaffold_15199	-	679	583	**RNA**→ B3_4 (smart00873)B3/B4 (COG3382)→
env-376	Throat_LANL_C5742452	+	263	359	**RNA**→ hypo→
env-377	SRS011140_C5078888	-	202	106	**RNA**→ B3/B4 (COG3382)→
env-378	SRS014684_C3305177	+	294	390	**RNA**→ B3_4 (smart00873)B3/B4 (COG3382)→ hypo→
env-379	SRS014689_WUGC_scaffold_4557	-	2139	2043	**RNA**→ B3_4 (smart00873)B3/B4 (COG3382)→ hypo→
env-380	SRS015057_C3437814	-	479	383	**RNA**→ B3_4 (smart00873)B3/B4 (COG3382)→
env-381	SRS018591_WUGC_scaffold_37253	+	608	704	**RNA**→ B3_4 (smart00873)B3/B4 (COG3382)→
env-382	SRS019026_WUGC_scaffold_23628	+	110	206	**RNA**→ B3/B4 (COG3382)→ B3_4 (smart00873)B3/B4 (COG3382)→
env-383	SRS019027_C1976825	+	263	359	**RNA**→ B3_4 (smart00873)B3/B4 (COG3382)→ hypo→
env-384	SRS023352_C4677293	+	1113	1209	**RNA**→ B3/B4 (COG3382)→
env-385	SRS023617_Baylor_scaffold_62238	-	2274	2178	**RNA**→ B3_4 (smart00873)B3/B4 (COG3382)→
env-386	SRS051791_LANL_scaffold_53157	-	2292	2196	**RNA**→ B3_4 (smart00873)B3/B4 (COG3382)→
Fnu-1-1	NZ_NJGI01000001.1	+	350663	350758	**RNA**→ hypo→
Fnu-1-2	NZ_NJGI01000007.1	-	91417	91322	**RNA**→ hypo→
env-387	SRS019077_WUGC_scaffold_7905	-	1616	1520	**RNA**→ B3_4 (smart00873)B3/B4 (COG3382)→ hypo→

env-388	SRS013836_C4613343	+	17	113	**RNA**→ B3/B4 (COG3382)→
env-389	SRS016575_Baylor_scaffold_33602	-	345	249	**RNA**→ ←hypo
env-390	SRS043755_C2344101	+	107	203	**RNA**→ B3_4 (smart00873)B3_4 (pfam03483)→
env-391	SRS018443_Baylor_scaffold_21413	-	954	858	**RNA**→ B3_4 (smart00873)B3/B4 (COG3382)→
env-392	SRS053917_C3812292	-	837	741	**RNA**→ B3_4 (smart00873)B3/B4 (COG3382)→
env-393	Ga0119795_1002630	+	7517	7613	**RNA**→ hypo→ B3_4 (smart00873)B3_4 (pfam03483)→ B3/B4 (COG3382)→
env-394	SRS022725_LANL_scaffold_3326	-	837	741	**RNA**→ B3_4 (smart00873)B3/B4 (COG3382)→
env-395	SRS024447_C3797786	-	221	125	**RNA**→ hypo→
Fnu-1-3	NZ_NHRT01000001.1	-	1909499	1909403	**RNA**→ B3_4 (smart00873)B3/B4 (COG3382)→
env-396	HCF12C_2_8332161	-	136	35	**RNA**→
env-397	HCF12C_2_16962674	-	136	35	**RNA**→
env-398	HCF12C_2_6630403	-	136	35	**RNA**→
env-399	HCF12C_2_3273007	+	67	168	**RNA**→
env-400	HCF12C_2_10051940	+	67	168	**RNA**→
env-401	HCF12C_2_1528304	-	136	35	**RNA**→
env-402	HCF12C_2_13430193	+	67	168	**RNA**→
env-403	HCF12C_2_20225321	-	136	35	**RNA**→
env-404	HCF12C_2_11763507	-	136	35	**RNA**→
env-405	HCF12C_2_18601564	+	67	168	**RNA**→
env-406	HCF12C_2_15139683	+	67	168	**RNA**→
env-407	HCF12C_2_4977393	+	67	168	**RNA**→
env-408	OLGF01002081.1	+	3311	3410	**RNA**→ PRK11431 (PRK11431)EmrE (COG2076)→
env-409	Ga0209064_1020627	+	2226	2331	**RNA**→ hypo→
env-410	DFIO01000053.1	-	6701	6595	**RNA**→ PRK11431 (PRK11431)EmrE (COG2076)→
env-411	Ga0256406_1013474	+	2433	2539	**RNA**→ PRK11431 (PRK11431)EmrE (COG2076)→
env-412	AUXO018294670.1	-	3351	3245	**RNA**→ PRK11431 (PRK11431)EmrE (COG2076)→
env-413	DGWE01000047.1	+	2712	2818	**RNA**→ PRK11431 (PRK11431)EmrE (COG2076)→
env-414	OIZU01019392.1	-	1105	1017	**RNA**→ NimA (COG3467)Pyridox_ox_2 (pfam12900)→
Lsp-7-1	NZ_NFKT01000035.1	-	27807	27719	**RNA**→ NimA (COG3467)Pyridox_ox_2 (pfam12900)→ vmrA (PRK09575)MATE_MepA_like (cd13143)→
env-415	Pasolli2019-15278-96	+	5418	5511	**RNA**→ NimA (COG3467)Pyridox_ox_2 (pfam12900)→
env-416	Ga0119784_1113608	+	136	234	**RNA**→ PRK11431 (PRK11431)EmrE (COG2076)→
env-417	Ga0177923_1123113	+	601	698	**RNA**→ hypo→
env-418	DHKR01000117.1	-	1498	1395	**RNA**→ PnuC (COG3201)NMN_trans_PnuC (TIGR01528)→
env-419	DHMS01000020.1	-	1250	1147	**RNA**→ PnuC (COG3201)NMN_trans_PnuC (TIGR01528)→
env-420	AUXO015950200.1	+	228	325	**RNA**→ hypo→
env-421	AUXO011545968.1	-	445	350	**RNA**→ PRK11431 (PRK11431)EmrE (COG2076)→
Bca-1-1	NZ_AAXG02000015.1	-	1484	1386	**RNA**→ NorM (COG0534)vmrA (PRK09575)→ NorM (COG0534)vmrA (PRK09575)→
env-422	C3339656_1_V1.CD-9	+	20	118	**RNA**→ Pyridox_oxidase (pfam01243)Pyridox_ox_2 (pfam12900)→
env-423	UnmappedStool_Broad_C252841372	-	418	320	**RNA**→ Pyridox_oxidase (pfam01243)Pyridox_ox_2 (pfam12900)→
env-424	OIYW01054056.1	+	225	323	**RNA**→ Pyridox_oxidase (pfam01243)Pyridox_ox_2 (pfam12900)→
env-425	Ga0134388_1041368	-	412	314	**RNA**→ Pyridox_oxidase (pfam01243)Pyridox_ox_2 (pfam12900)→
Asp-2-1	NZ_NFJK01000001.1	-	155736	155638	**RNA**→ NimA (COG3467)Pyridox_ox_2 (pfam12900)→ vmrA (PRK09575)MATE_MepA_like (cd13143)→
env-426	Pasolli2019-14894-0	-	128055	127957	**RNA**→ Pyridox_oxidase (pfam01243)Pyridox_ox_2 (pfam12900)→ MATE_MepA_like (cd13143)matE (TIGR00797)→
env-427	UMGS41-0	-	128055	127957	**RNA**→ Pyridox_oxidase (pfam01243)Pyridox_ox_2 (pfam12900)→ MATE_MepA_like (cd13143)matE (TIGR00797)→
env-428	Pasolli2019-4633-116	-	2908	2808	**RNA**→ NorM (COG0534)matE (TIGR00797)→
env-429	Pasolli2019-4634-173	-	937	837	**RNA**→ NorM (COG0534)matE (TIGR00797)→
env-430	JGI20163J15578_10065710	+	516	616	**RNA**→ MATE_MepA_like (cd13143)Polysacc_synt_C (pfam14667)→
env-431	JGI20163J15578_10073597	+	1734	1834	**RNA**→ vmrA (PRK09575)MATE_MepA_like (cd13143)→
env-432	JGI20163J15578_11397692	-	280	180	**RNA**→ vmrA (PRK09575)MATE_MepA_like (cd13143)→
env-433	JGI20163J15578_10002956	+	6656	6756	**RNA**→ vmrA (PRK09575)MATE_MepA_like (cd13143)→
Bsp-1-1	NZ_NFJL01000011.1	+	90675	90764	**RNA**→ vmrA (PRK09575)MATE_MepA_like (cd13143)→

Lpe-1-1	NZ_BCMI01000024.1	-	3051	2959	**RNA**→ PRK11431 (PRK11431)EmrE (COG2076)→
Lfu-1-1	NZ_BAMJ01000038.1	-	4480	4388	**RNA**→ PRK11431 (PRK11431)EmrE (COG2076)→
Lde-1-1	NZ_AYYK01000013.1	-	40267	40175	**RNA**→ PRK11431 (PRK11431)EmrE (COG2076)→
Lfa-3-1	NZ_AEIZ01000013.1	+	27357	27449	**RNA**→ PRK11431 (PRK11431)EmrE (COG2076)→ PRK11431 (PRK11431)EmrE (COG2076)→
env-434	HBC_scaff_10331302	-	2715	2621	**RNA**→ PRK11431 (PRK11431)EmrE (COG2076)→
env-435	HBC_ctgs_1004638	-	2715	2621	**RNA**→ PRK11431 (PRK11431)EmrE (COG2076)→
env-436	Ga0121485_134321	-	220	127	**RNA**→ PRK11431 (PRK11431)EmrE (COG2076)→
env-437	DDHC01000008.1	-	20648	20555	**RNA**→ PRK11431 (PRK11431)EmrE (COG2076)→
env-438	Ga0121143_101556	+	438	531	**RNA**→ PRK11431 (PRK11431)EmrE (COG2076)→
env-439	Ga0122751_108069	-	286	193	**RNA**→ PRK11431 (PRK11431)EmrE (COG2076)→
Esp-11-1	NZ_LHOX01000015.1	-	408971	408878	**RNA**→ PRK11431 (PRK11431)EmrE (COG2076)→
env-440	DINX01000019.1	-	88938	88845	**RNA**→ PRK11431 (PRK11431)EmrE (COG2076)→
Lsp-1-1	NZ_NPNH01000007.1	+	77716	77806	**RNA**→ B3_4 (smart00873)B3/B4 (COG3382)→
Lsp-5-1	NZ_CP009531.1	-	405465	405375	**RNA**→ B3_4 (smart00873)B3_4 (pfam03483)→
Pac-2-1	NZ_GL397067.1	+	1372008	1372099	**RNA**→ PRK11431 (PRK11431)EmrE (COG2076)→
Pac-1-1	NZ_JQAQ01000002.1	-	12968	12877	**RNA**→ PRK11431 (PRK11431)EmrE (COG2076)→
Eth-1-1	NZ_CP023074.1	-	486966	486873	**RNA**→ PRK11431 (PRK11431)EmrE (COG2076)→ Acetyltransf_1 (pfam00583)Acetyltransf_7 (pfam13508)→
Lmo-1-2	NZ_CWML01000037.1	-	42653	42560	**RNA**→ PRK11431 (PRK11431)EmrE (COG2076)→
Esp-5-1	NZ_NGMR01000001.1	-	651043	650950	**RNA**→ PRK11431 (PRK11431)EmrE (COG2076)→ Acetyltransf_1 (pfam00583)Acetyltransf_7 (pfam13508)→
Efa-295-1	NZ_KB029962.1	+	1614886	1614980	**RNA**→ PRK11431 (PRK11431)EmrE (COG2076)→
Esp-3-1	NZ_NGLI01000001.1	-	205342	205248	**RNA**→ PRK11431 (PRK11431)EmrE (COG2076)→
Efa-294-1	NZ_PTWO01000043.1	-	16853	16759	**RNA**→ PRK11431 (PRK11431)EmrE (COG2076)→
Esp-9-1	NZ_KV815559.1	+	27292	27386	**RNA**→ PRK11431 (PRK11431)EmrE (COG2076)→
Esp-6-1	NZ_KV817748.1	+	10736	10830	**RNA**→ PRK11431 (PRK11431)EmrE (COG2076)→
Esp-7-1	NZ_KV833967.1	+	1996	2090	**RNA**→ PRK11431 (PRK11431)EmrE (COG2076)→
Efa-299-1	NZ_AWWN01000031.1	-	17639	17545	**RNA**→ PRK11431 (PRK11431)EmrE (COG2076)→
Efa-298-1	NZ_AWWM01000057.1	-	16745	16651	**RNA**→ PRK11431 (PRK11431)EmrE (COG2076)→
Efa-294-2	NZ_NGMJ01000001.1	-	217948	217854	**RNA**→ PRK11431 (PRK11431)EmrE (COG2076)→
Efa-294-3	NZ_NGMI01000002.1	-	9359	9265	**RNA**→ PRK11431 (PRK11431)EmrE (COG2076)→
env-441	Ga0120973_104613	+	41	135	**RNA**→ PRK11431 (PRK11431)EmrE (COG2076)→
Ega-2-1	NZ_GG670286.1	-	607329	607235	**RNA**→ PRK11431 (PRK11431)EmrE (COG2076)→
Ega-1-1	NZ_JMGP01000004.1	-	45637	45543	**RNA**→ PRK11431 (PRK11431)EmrE (COG2076)→
env-442	Ga0122310_101630	+	274	368	**RNA**→ ←hypo
env-443	Ga0122077_103095	+	82	176	**RNA**→ ←hypo
Ega-1-2	NZ_PPHK01000001.1	-	212817	212723	**RNA**→ PRK11431 (PRK11431)EmrE (COG2076)→
Ega-1-3	NZ_MJED01000006.1	+	158567	158661	**RNA**→ PRK11431 (PRK11431)EmrE (COG2076)→
env-444	DFXL01000011.1	-	560181	560087	**RNA**→ PRK11431 (PRK11431)EmrE (COG2076)→
env-445	Ga0160505_100256	-	2508	2414	**RNA**→ PRK11431 (PRK11431)EmrE (COG2076)→
env-446	Ga0169866_100168	-	28083	27989	**RNA**→ PRK11431 (PRK11431)EmrE (COG2076)→
env-447	Pasolli2019-7951-2	-	51873	51779	**RNA**→ EmrE (COG2076)PRK11431 (PRK11431)→
Esa-1-1	NZ_JH376939.1	-	1115044	1114950	**RNA**→ PRK11431 (PRK11431)EmrE (COG2076)→ hypo→ hypo→
Ega-1-4	NZ_JNLR01000003.1	-	1011533	1011439	**RNA**→ PRK11431 (PRK11431)EmrE (COG2076)→
Ega-1-5	NZ_NGMQ01000001.1	-	1127856	1127762	**RNA**→ PRK11431 (PRK11431)EmrE (COG2076)→
Ega-1-6	NZ_NSCY01000001.1	+	211008	211102	**RNA**→ PRK11431 (PRK11431)EmrE (COG2076)→
Lta-1-1	NZ_NGOW01000001.1	+	61920	62009	**RNA**→
Lcr-1-1	NZ_LJGP01000076.1	+	1740	1841	**RNA**→ PRK11431 (PRK11431)EmrE (COG2076)→ PRK00871 (PRK00871)Flavodoxin_2 (pfam02525)→ PRK00871 (PRK00871)Flavodoxin_2 (pfam02525)→
Lul-1-2	NZ_GG693253.1	-	795865	795763	**RNA**→ PRK11431 (PRK11431)EmrE (COG2076)→
Lul-1-1	NZ_AZFO01000011.1	+	27618	27720	**RNA**→ PRK11431 (PRK11431)EmrE (COG2076)→
env-448	AUXO014626460.1	-	96	1	**RNA**→
env-449	AUXO015924737.1	-	111	9	**RNA**→
env-450	Ga0129306_1000177	-	9490	9393	**RNA**→ PRK01766 (PRK01766)MATE_NorM_like (cd13131)→

env-451	Ga0129307_1000149	-	9490	9393	**RNA**→ PRK01766 (PRK01766)MATE_NorM_like (cd13131)→
env-452	Pasolli2019-6924-9	+	72522	72621	**RNA**→ MATE_NorM_like (cd13131)matE (TIGR00797)→
env-453	UMGS823-13	+	36445	36544	**RNA**→ MATE_NorM_like (cd13131)matE (TIGR00797)→
env-454	DJPM01000040.1	+	153	253	**RNA**→ PRK11431 (PRK11431)EmrE (COG2076)→ rimM (PRK00122)RimM (pfam01782)PRC (pfam05239)→ TrmD (COG0336)trmD (PRK00026)Acetyltransf_1 (pfam00583)Acetyltransf_7 (pfam13508)→ hypo→ RsmA (COG0030)ksgA (PRK00274)→
env-455	Ga0116646_1063777	+	104	204	**RNA**→ PRK11431 (PRK11431)EmrE (COG2076)→
env-456	Ga0116649_1100351	+	92	192	**RNA**→ ←hypo
env-457	DHKF01000005.1	+	17484	17584	**RNA**→ PRK11431 (PRK11431)EmrE (COG2076)→ rimM (PRK00122)RimM (pfam01782)PRC (pfam05239)→ TrmD (COG0336)trmD (PRK00026)Acetyltransf_1 (pfam00583)Acetyltransf_7 (pfam13508)→
env-458	Ga0116618_102948	-	1318	1218	**RNA**→ PRK11431 (PRK11431)EmrE (COG2076)→ rimM (PRK00122)RimM (pfam01782)PRC (pfam05239)→ TrmD (COG0336)trmD (PRK00026)→
env-459	DJEW01000044.1	-	16578	16478	**RNA**→ PRK11431 (PRK11431)EmrE (COG2076)→ rimM (PRK00122)RimM (pfam01782)PRC (pfam05239)→ TrmD (COG0336)trmD (PRK00026)Acetyltransf_1 (pfam00583)Acetyltransf_7 (pfam13508)→ hypo→ RsmA (COG0030)ksgA (PRK00274)→
env-460	Pasolli2019-15193-1	-	118649	118550	**RNA**→ EmrE (COG2076)PRK11431 (PRK11431)→ RimM (pfam01782)PRC (pfam05239)16S_RimM (TIGR02273)→ TrmD (COG0336)Acetyltransf_1 (pfam00583)trmD (TIGR00088)Acetyltransf_7 (pfam13508)→ hypo→ RsmA (COG0030)ksgA (TIGR00755)→
[1]Rti-1-1	NZ_LT964681.1	-	701232	701130	**RNA**→ PRK11431 (PRK11431)EmrE (COG2076)→
Rma-1-1	NZ_LT962670.1	+	335907	336012	**RNA**→ PRK11431 (PRK11431)EmrE (COG2076)→
env-461	Pasolli2019-7198-321	+	560	656	**RNA**→ EmrE (COG2076)PRK11431 (PRK11431)→ Acetyltransf_1 (pfam00583)Acetyltransf_7 (pfam13508)→ EcCorA_ZntB-like_u2 (cd12827)→
Lco-3-1	NZ_CP017697.1	-	2299115	2299019	**RNA**→ PRK11431 (PRK11431)EmrE (COG2076)→ Acetyltransf_1 (pfam00583)Acetyltransf_7 (pfam13508)→ EcCorA_ZntB-like_u2 (cd12827)corA (TIGR00383)→ Abhydrolase_1 (pfam00561)Abhydrolase_6 (pfam12697)→
Lco-4-1	NZ_AEOS01000032.1	-	32372	32276	**RNA**→ PRK11431 (PRK11431)EmrE (COG2076)→ PRK11431 (PRK11431)EmrE (COG2076)→ PRK11431 (PRK11431)EmrE (COG2076)→ Acetyltransf_1 (pfam00583)Acetyltransf_7 (pfam13508)→ Acetyltransf_1 (pfam00583)Acetyltransf_7 (pfam13508)→ EcCorA_ZntB-like_u2 (cd12827)corA (TIGR00383)→ EcCorA_ZntB-like_u2 (cd12827)corA (TIGR00383)→ Abhydrolase_1 (pfam00561)Abhydrolase_6 (pfam12697)→ Abhydrolase_1 (pfam00561)Abhydrolase_6 (pfam12697)→
env-462	OMZN01000075.1	-	3970	3870	**RNA**→ EmrE (COG2076)PRK11431 (PRK11431)→
env-463	Ga0129308_1000167	+	108071	108167	**RNA**→ PRK11431 (PRK11431)EmrE (COG2076)→ rimM (PRK00122)RimM (pfam01782)→ TrmD (COG0336)trmD (PRK00026)PRK05279 (PRK05279)Acetyltransf_1 (pfam00583)→ hypo→ RsmA (COG0030)ksgA (PRK00274)→ PpsA (COG0574)PRK09279 (PRK09279)→
env-464	Pasolli2019-15195-0	-	25658	25558	**RNA**→ EmrE (COG2076)PRK11431 (PRK11431)→ hypo→
env-465	OIZE01016085.1	-	1266	1171	**RNA**→ PRK11431 (PRK11431)EmrE (COG2076)→
env-466	OLGA01004984.1	-	6116	6021	**RNA**→ PRK11431 (PRK11431)EmrE (COG2076)→
env-467	OIWS01018344.1	-	166	71	**RNA**→
env-468	scaffold39640_4_V1.UC-15	-	1417	1322	**RNA**→ ←CAT (smart01059)CatA (COG4845)
env-469	DLM023_scaffold13430_3	-	2981	2886	**RNA**→ ←COG5354 (COG5354)
env-470	OIYW01005865.1	+	2167	2263	**RNA**→ PRK11431 (PRK11431)EmrE (COG2076)→
env-471	DBRU01000046.1	+	2225	2320	**RNA**→ NorM (COG0534)vmrA (PRK09575)→
env-472	OIXH01036174.1	-	753	657	**RNA**→ PRK11431 (PRK11431)EmrE (COG2076)→ rimM (PRK00122)RimM (pfam01782)→
env-473	OIYO01000658.1	-	23060	22963	**RNA**→ ←Na_pump_decarbB (TIGR01109)OAD_beta (pfam03977)
env-474	Pasolli2019-15190-12	-	78530	78433	**RNA**→ ←OAD_beta (pfam03977)Na_pump_decarbB (TIGR01109)
env-475	UMGS887-35	-	23060	22963	**RNA**→ ←OAD_beta (pfam03977)Na_pump_decarbB (TIGR01109)
env-476	OJAR01016866.1	+	713	809	**RNA**→ hypo→
env-477	OIYG01096403.1	-	273	177	**RNA**→ PRK11431 (PRK11431)EmrE (COG2076)→
env-478	Pasolli2019-15111-58	-	6689	6592	**RNA**→ EmrE (COG2076)PRK11431 (PRK11431)→
env-479	Ga0117810_1094815	+	94	191	**RNA**→ PRK11431 (PRK11431)EmrE (COG2076)→
env-480	Pasolli2019-15109-0	-	87145	87048	**RNA**→ EmrE (COG2076)PRK11431 (PRK11431)→
env-481	OIWT01057152.1	+	508	605	**RNA**→ PRK11431 (PRK11431)EmrE (COG2076)→
env-482	Pasolli2019-15110-74	+	9813	9910	**RNA**→ EmrE (COG2076)PRK11431 (PRK11431)→
env-483	Ga0134563_134821	-	437	342	**RNA**→ ←PRK11431 (PRK11431)EmrE (COG2076)

env-484	OIYT01017125.1	-	2133	2038	**RNA**→ PRK11431 (PRK11431)EmrE (COG2076)→ rimM (PRK00122)RimM (pfam01782)PRC (pfam05239)→ TrmD (COG0336)trmD (PRK00026)→
env-485	Ga0116649_1091822	-	197	102	**RNA**→ PRK11431 (PRK11431)EmrE (COG2076)→
env-486	UnmappedStool_Broad_C251980095	-	338	243	**RNA**→ PRK11431 (PRK11431)EmrE (COG2076)→
env-487	Pasolli2019-15108-16	-	18926	18831	**RNA**→ EmrE (COG2076)PRK11431 (PRK11431)→
env-488	DLM022_scaffold27765_8	-	203	108	**RNA**→ PRK11431 (PRK11431)EmrE (COG2076)→
env-489	scaffold170917_2_MH0006	+	614	709	**RNA**→ PRK11431 (PRK11431)EmrE (COG2076)→
env-490	scaffold57430_2_MH0024	-	487	392	**RNA**→ PRK11431 (PRK11431)EmrE (COG2076)→
env-491	scaffold841_4_MH0060	-	1471	1376	**RNA**→ PRK11431 (PRK11431)EmrE (COG2076)→
env-492	Ga0129313_1076656	-	145	50	**RNA**→
env-493	scaffold35295_2_MH0031	+	486	581	**RNA**→ PRK11431 (PRK11431)EmrE (COG2076)→
env-494	4491403.3_NODE_52680	+	997	1092	**RNA**→ hypo→
env-495	OIWN01000404.1	-	17710	17613	**RNA**→ vmrA (PRK09575)MATE_MepA_like (cd13143)→
env-496	UnmappedStool_Broad_scaffold_416734	-	510	413	**RNA**→ vmrA (PRK09575)MATE_MepA_like (cd13143)→
Lgi-1-1	NZ_CP012034.1	-	2549234	2549130	**RNA**→ PRK11431 (PRK11431)EmrE (COG2076)→ RhaT (COG0697)2A78 (TIGR00950)→
Lve-2-1	NZ_BACR01000012.1	+	36398	36498	**RNA**→ PRK11431 (PRK11431)EmrE (COG2076)→ PRK11431 (PRK11431)EmrE (COG2076)→
Lve-1-1	NZ_AZFA01000004.1	+	35307	35407	**RNA**→ PRK11431 (PRK11431)EmrE (COG2076)→
env-497	2205161127	+	76	177	**RNA**→
env-498	2205835708	+	474	575	**RNA**→ PRK11431 (PRK11431)EmrE (COG2076)→
env-499	2157541122	-	1889	1788	**RNA**→ PRK11431 (PRK11431)EmrE (COG2076)→ hypo→ UgpQ (COG0584)glpQ (PRK11143)→ GDPD_SaGlpQ_like (cd08601)→
Lfa-1-1	NZ_GL575018.1	+	982744	982845	**RNA**→ PRK11431 (PRK11431)EmrE (COG2076)→ hypo→ UgpQ (COG0584)glpQ (PRK11143)→
env-500	DPBB01000052.1	+	12597	12698	**RNA**→ PRK11431 (PRK11431)EmrE (COG2076)→
Lcr-2-1	NZ_CP017996.1	-	666234	666133	**RNA**→ PRK11431 (PRK11431)EmrE (COG2076)→
Lcr-3-1	NZ_AZDB01000002.1	+	80893	80994	**RNA**→ PRK11431 (PRK11431)EmrE (COG2076)→
Lna-2-1	NZ_AZFV01000007.1	+	91200	91301	**RNA**→ PRK11431 (PRK11431)EmrE (COG2076)→ hypo→ UgpQ (COG0584)glpQ (PRK11143)→ DUF3781 (pfam12636)→
env-501	2158536691	+	102	203	**RNA**→ PRK11431 (PRK11431)EmrE (COG2076)→
env-502	2157764790	-	153	52	**RNA**→ hypo→
env-503	2157527731	+	4	104	**RNA**→ PRK11431 (PRK11431)EmrE (COG2076)→
env-504	2205810991	+	9775	9876	**RNA**→ PRK11431 (PRK11431)EmrE (COG2076)→
Lpa-3-1	NZ_BAMH01000059.1	+	350	451	**RNA**→ PRK11431 (PRK11431)EmrE (COG2076)→
Lpa-3-2	NZ_AZES01000020.1	-	15348	15247	**RNA**→ PRK11431 (PRK11431)EmrE (COG2076)→
env-505	2157696259	+	6748	6849	**RNA**→ PRK11431 (PRK11431)EmrE (COG2076)→
Lno-1-1	NZ_BAMN01000045.1	+	2804	2905	**RNA**→ PRK11431 (PRK11431)EmrE (COG2076)→
Lno-1-2	NZ_AZDZ01000022.1	+	269280	269381	**RNA**→ PRK11431 (PRK11431)EmrE (COG2076)→
Lno-1-3	NZ_BCWC01000004.1	+	239079	239180	**RNA**→ PRK11431 (PRK11431)EmrE (COG2076)→
Lal-2-1	NZ_CP019323.1	+	2493863	2493964	**RNA**→ hypo→ HTH_ARSR (smart00418)HTH_ARSR (cd00090)→
env-506	2205863796	-	223	122	**RNA**→ PRK11431 (PRK11431)EmrE (COG2076)→
env-507	2158002089	-	223	122	**RNA**→ PRK11431 (PRK11431)EmrE (COG2076)→
Lal-1-1	NZ_CP018867.1	-	99819	99718	**RNA**→ PRK11431 (PRK11431)EmrE (COG2076)→ hypo→ UgpQ (COG0584)glpQ (PRK11143)→ DUF3781 (pfam12636)→
Whe-1-1	NZ_FMAW01000003.1	+	58499	58600	**RNA**→ PRK11431 (PRK11431)EmrE (COG2076)→
Whe-1-2	NZ_BBIK01000010.1	-	208580	208479	**RNA**→ PRK11431 (PRK11431)EmrE (COG2076)→
Lpa-1-1	NZ_BBAR01000058.1	-	10691	10590	**RNA**→ PRK11431 (PRK11431)EmrE (COG2076)→
Lpa-1-2	NZ_AZFZ01000057.1	+	7535	7636	**RNA**→ PRK11431 (PRK11431)EmrE (COG2076)→
Lpa-2-1	NZ_BDGB01000103.1	-	3661	3562	**RNA**→ PRK11431 (PRK11431)EmrE (COG2076)→
Lpa-2-2	NZ_NCXA01000032.1	+	23479	23578	**RNA**→ PRK11431 (PRK11431)EmrE (COG2076)→
Lra-1-1	NZ_AZEI01000022.1	-	48759	48660	**RNA**→ PRK11431 (PRK11431)EmrE (COG2076)→ hypo→ Hol_Tox (pfam16935)→
Lki-3-1	NZ_JH591048.1	+	8770	8869	**RNA**→ ←hypo
Lki-2-1	NZ_AZEB01000007.1	+	58956	59055	**RNA**→ PRK11431 (PRK11431)EmrE (COG2076)→
Ldi-1-1	NZ_AZEY01000079.1	-	85841	85742	**RNA**→ PRK11431 (PRK11431)EmrE (COG2076)→
Lhe-1-1	NZ_CP020029.1	-	1997191	1997090	**RNA**→ PRK11431 (PRK11431)EmrE (COG2076)→ hypo→

Lhe-3-1	NC_010080.1	+	55038	55139	**RNA**→ PRK11431 (PRK11431)EmrE (COG2076)→ hypo→ hypo→
Lhe-2-1	NZ_HG530772.1	+	4098	4199	**RNA**→ PRK11431 (PRK11431)EmrE (COG2076)→ hypo→ hypo→ hypo→ nudE (PRK11762)ADPRase_NUDT5 (cd03424)→
Lhe-1-2	NZ_CP016827.1	-	1377305	1377204	**RNA**→ PRK11431 (PRK11431)EmrE (COG2076)→ hypo→
Lhe-1-3	NZ_CP015498.1	+	70319	70420	**RNA**→ PRK11431 (PRK11431)EmrE (COG2076)→ hypo→
Lhe-1-4	NZ_CP015496.1	+	54243	54344	**RNA**→ PRK11431 (PRK11431)EmrE (COG2076)→ hypo→
Lfa-2-1	NZ_BAKI01000005.1	+	29375	29476	**RNA**→ PRK11431 (PRK11431)EmrE (COG2076)→ nudE (PRK11762)ADPRase_NUDT5 (cd03424)→
Lfa-2-2	NZ_AZFY01000085.1	+	29436	29537	**RNA**→ PRK11431 (PRK11431)EmrE (COG2076)→ nudE (PRK11762)ADPRase_NUDT5 (cd03424)→
env-508	Ga0214925_100152	-	9442	9340	**RNA**→ PRK11431 (PRK11431)EmrE (COG2076)→
Lbr-2-1	NZ_GG669604.1	-	529070	528968	**RNA**→ PRK11431 (PRK11431)EmrE (COG2076)→
Lhi-2-1	NZ_GG669992.1	-	451401	451299	**RNA**→ PRK11431 (PRK11431)EmrE (COG2076)→ nudE (PRK11762)ADPRase_NUDT5 (cd03424)→
Lhi-1-1	NZ_AZDF01000007.1	+	39506	39608	**RNA**→ PRK11431 (PRK11431)EmrE (COG2076)→ nudE (PRK11762)ADPRase_NUDT5 (cd03424)→
Nma-1-1	NZ_LN875035.1	-	2900439	2900337	**RNA**→ vmrA (PRK09575)MATE_MepA_like (cd13143)→ REC (smart00448)Trans_reg_C (smart00862)OmpR (COG0745)→
env-509	AUXO015873809.1	-	421	318	**RNA**→ PnuC (COG3201)NMN_trans_PnuC (TIGR01528)→
env-510	DGUM01000071.1	+	1394	1497	**RNA**→ PnuC (COG3201)NMN_trans_PnuC (TIGR01528)→
env-511	DNLK01000074.1	-	4770	4667	**RNA**→ PnuC (COG3201)NMN_trans_PnuC (TIGR01528)→
env-512	Ga0208824_1000325	-	4465	4362	**RNA**→ PnuC (COG3201)NMN_trans_PnuC (TIGR01528)→
env-513	Ga0208198_1000358	-	4465	4362	**RNA**→ PnuC (COG3201)NMN_trans_PnuC (TIGR01528)→
env-514	Ga0208694_1261288	+	92	195	**RNA**→ PnuC (COG3201)NMN_trans_PnuC (TIGR01528)→
Csp-12-2	NZ_AUUU01000057.1	-	14067	13965	**RNA**→ Acetyltransf_1 (pfam00583)Acetyltransf_7 (pfam13508)→ hypo→
Csa-1-1	NZ_CP016091.1	-	3917259	3917157	**RNA**→ Acetyltransf_1 (pfam00583)Acetyltransf_7 (pfam13508)→ hypo→
env-515	DMJQ01000025.1	-	4974	4873	**RNA**→ NorM (COG0534)vmrA (PRK09575)→
env-516	BMHB3a_c72971	+	106	205	**RNA**→ PRK11431 (PRK11431)EmrE (COG2076)→
env-517	4491686.3.4394	+	1266	1374	**RNA**→ ←hypo
env-518	UMGS1799-230	+	4862	4960	**RNA**→ Pyridox_oxidase (pfam01243)Pyridox_ox_2 (pfam12900)→
env-519	Pasolli2019-4990-89	-	15916	15818	**RNA**→ NimA (COG3467)Pyridox_ox_2 (pfam12900)→ MATE_MepA_like (cd13143)Polysacc_synt_C (pfam14667)
env-520	DBPX01000021.1	-	12416	12323	**RNA**→ hypo→
Bsp-1-2	NZ_NFJL01000021.1	+	52419	52507	**RNA**→ NimA (COG3467)Pyridox_ox_2 (pfam12900)→
env-521	2211105322	-	217	114	**RNA**→ hypo→
env-522	2157240358	-	217	114	**RNA**→ hypo→
env-523	DBOW01000057.1	+	5871	5961	**RNA**→ B3_4 (smart00873)B3/B4 (COG3382)→
env-524	Ga0116620_1000240	-	30957	30867	**RNA**→ vmrA (PRK09575)MATE_MepA_like (cd13143)→
env-525	DLLD01000014.1	-	192580	192490	**RNA**→ MATE_MepA_like (cd13143)Polysacc_synt_C (pfam14667)→
Msp-5-1	NZ_KQ958203.1	-	26182	26095	**RNA**→ PRK11431 (PRK11431)EmrE (COG2076)→
Lsa-1-1	NZ_CP025839.1	+	1692436	1692520	**RNA**→ PRK11431 (PRK11431)EmrE (COG2076)→
Lcu-2-1	NZ_LT841333.1	+	1478909	1478993	**RNA**→ hypo→
env-526	Ga0214090_10486045	-	945	861	**RNA**→ PRK11431 (PRK11431)EmrE (COG2076)→
env-527	SR_TTP_S3_1017208	+	147	250	**RNA**→ hypo→
env-528	Pasolli2019-5799-14	-	14686	14589	**RNA**→ EmrE (COG2076)PRK11431 (PRK11431)→
env-529	scaffold47472_6_MH0066	+	1193	1293	**RNA**→ PRK11431 (PRK11431)EmrE (COG2076)→
env-530	OLHE01110896.1	+	79	177	**RNA**→ vmrA (PRK09575)MATE_MepA_like (cd13143)→
env-531	UnmappedStool_Broad_scaffold_207652	+	159	257	**RNA**→ NorM (COG0534)vmrA (PRK09575)→ vmrA (PRK09575)MATE_MepA_like (cd13143)→
env-532	SRS016495_C1885865	+	393	491	**RNA**→ vmrA (PRK09575)MATE_MepA_like (cd13143)→
Csp-18-1	NZ_DS562848.1	+	316957	317055	**RNA**→ vmrA (PRK09575)MATE_MepA_like (cd13143)→
env-533	UnmappedStool_Broad_scaffold_187174	-	386	292	**RNA**→ PRK11431 (PRK11431)EmrE (COG2076)→
Csp-6-1	NZ_FBWL01000160.1	-	89305	89208	**RNA**→ vmrA (PRK09575)MATE_MepA_like (cd13143)→ REC (smart00448)Trans_reg_C (smart00862)OmpR (COG0745)→ HATPase_c (smart00387)HisKA (smart00388)BaeS (COG0642)→
env-534	Ga0169870_100614	-	13878	13790	**RNA**→ vmrA (PRK09575)MATE_MepA_like (cd13143)→
Asp-1-1	NZ_GL629688.1	+	370133	370221	**RNA**→ vmrA (PRK09575)MATE_MepA_like (cd13143)→
Cba-3-1	NZ_FCNA01000037.1	+	34293	34385	**RNA**→ vmrA (PRK09575)MATE_MepA_like (cd13143)→
env-535	UMGS1930-26	+	414	519	**RNA**→ PnuC (COG3201)NMN_trans_PnuC (TIGR01528)→

env-536	DQGD01000015.1	-	9004	8899	**RNA**→ PnuC (COG3201)NMN_trans_PnuC (TIGR01528)→
env-537	Pasolli2019-14026-10	+	24424	24529	**RNA**→ PnuC (COG3201)NMN_trans_PnuC (TIGR01528)→
Csp-11-1	NZ_LITJ01000034.1	+	63266	63367	**RNA**→ MATE_MepA_like (cd13143)Polysacc_synt_C (pfam14667)→
env-538	DGET01000051.1	+	21432	21531	**RNA**→ vmrA (PRK09575)MATE_MepA_like (cd13143)→ hypo→ hypo→
env-539	Ga0134105_1000355	+	21798	21897	**RNA**→ vmrA (PRK09575)MATE_MepA_like (cd13143)→ hypo→
env-540	Ga0134102_1000437	+	21798	21897	**RNA**→ vmrA (PRK09575)MATE_MepA_like (cd13143)→ hypo→
env-541	Ga0134100_1003380	+	7249	7348	**RNA**→ NorM (COG0534)vmrA (PRK09575)→ hypo→
env-542	Ga0208693_1001029	+	15595	15694	**RNA**→ vmrA (PRK09575)MATE_MepA_like (cd13143)→ hypo→ hypo→
env-543	Ga0208823_1002217	-	1615	1516	**RNA**→ vmrA (PRK09575)MATE_MepA_like (cd13143)→ hypo→
env-544	Ga0208564_1000507	-	14470	14371	**RNA**→ vmrA (PRK09575)MATE_MepA_like (cd13143)→ hypo→ hypo→
env-545	JGI24713J26584_10000482	-	9462	9363	**RNA**→ vmrA (PRK09575)MATE_MepA_like (cd13143)→ hypo→ hypo→
env-546	KGI_S1_ANT02_95m_c10222668	-	99	1	**RNA**→
Rma-2-1	NZ_NOJZ01000087.1	-	47095	46993	**RNA**→ vmrA (PRK09575)MATE_MepA_like (cd13143)→
env-547	UMGS826-22	-	5585	5485	**RNA**→ NorM (COG0534)matE (TIGR00797)→
env-548	OGIX01000665.1	+	17199	17297	**RNA**→ MATE_MepA_like (cd13143)Polysacc_synt_C (pfam14667)→
Csp-7-1	NZ_LM994670.1	-	2671576	2671478	**RNA**→ MATE_MepA_like (cd13143)Polysacc_synt_C (pfam14667)→
env-549	OGIN01028859.1	-	257	159	**RNA**→ MATE_MepA_like (cd13143)→
env-550	Pasolli2019-6076-22	+	51823	51926	**RNA**→ MATE_MepA_like (cd13143)matE (TIGR00797)→ CAT (pfam00302)CAT (smart01059)→
env-551	UMGS1389-19	+	51823	51926	**RNA**→ MATE_MepA_like (cd13143)matE (TIGR00797)→ CAT (pfam00302)CAT (smart01059)→
env-552	DHNI01000127.1	-	3090	2992	**RNA**→ vmrA (PRK09575)MATE_MepA_like (cd13143)→
env-553	Ga0075011_10000141	-	31924	31824	**RNA**→ NorM (COG0534)vmrA (PRK09575)→ Acetyltransf_1 (pfam00583)Acetyltransf_7 (pfam13508)→
env-554	Ga0194138_10000135	+	4530	4630	**RNA**→ NorM (COG0534)vmrA (PRK09575)→ Acetyltransf_1 (pfam00583)Acetyltransf_7 (pfam13508)→
env-555	Ga0194137_10000287	-	30676	30576	**RNA**→ NorM (COG0534)vmrA (PRK09575)→ Acetyltransf_1 (pfam00583)Acetyltransf_7 (pfam13508)→
env-556	Ga0194136_1000299	+	4529	4629	**RNA**→ NorM (COG0534)vmrA (PRK09575)→ Acetyltransf_1 (pfam00583)Acetyltransf_7 (pfam13508)→
env-557	DOPX01000027.1	-	49069	48970	**RNA**→ vmrA (PRK09575)MATE_MepA_like (cd13143)→ DUF488 (pfam04343)→ RimI (COG0456)Acetyltransf_7 (pfam13508)→
env-558	Ga0172377_10347784	+	456	555	**RNA**→ MATE_MepA_like (cd13143)→ hypo→
env-559	Ga0179952_1028037	-	387	286	**RNA**→ MATE_MepA_like (cd13143)→
env-560	Ga0208198_1086170	-	103	2	**RNA**→
env-561	Ga0214086_1578668	-	2223	2124	**RNA**→ NorM (COG0534)vmrA (PRK09575)→
env-562	Ga0170573_10897520	-	686	586	**RNA**→ vmrA (PRK09575)MATE_MepA_like (cd13143)→
Cin-1-1	NZ_FXBJ01000002.1	+	171727	171828	**RNA**→ NorM (COG0534)vmrA (PRK09575)→
Cin-1-2	NZ_FOAH01000017.1	-	33156	33055	**RNA**→ NorM (COG0534)vmrA (PRK09575)→
env-563	JGI24712J26585_10520727	+	52	151	**RNA**→ MATE_MepA_like (cd13143)→
env-564	JGI24711J26586_10547291	+	16	115	**RNA**→ ←hypo
env-565	Pasolli2019-4513-6	+	50803	50904	**RNA**→ MATE_MepA_like (cd13143)matE (TIGR00797)→
env-566	Ga0209724_1000391	-	14429	14328	**RNA**→ vmrA (PRK09575)MATE_MepA_like (cd13143)→
env-567	Ga0209511_1000053	-	61607	61506	**RNA**→ vmrA (PRK09575)MATE_MepA_like (cd13143)→
env-568	Ga0209512_1015908	-	2907	2806	**RNA**→ vmrA (PRK09575)MATE_MepA_like (cd13143)→
env-569	Ga0209100_1001493	+	11123	11224	**RNA**→ vmrA (PRK09575)MATE_MepA_like (cd13143)→
Csp-14-1	NZ_FJVE01000007.1	+	787032	787133	**RNA**→ vmrA (PRK09575)MATE_MepA_like (cd13143)→
env-570	JGI24707J26582_10003065	+	6725	6825	**RNA**→ NorM (COG0534)vmrA (PRK09575)→
env-571	JGI24708J26588_10002874	-	5472	5372	**RNA**→ NorM (COG0534)vmrA (PRK09575)→
env-572	JGI24710J26742_10163814	+	397	497	**RNA**→ MATE_MepA_like (cd13143)→
env-573	JGI24709J26583_10053322	-	1449	1349	**RNA**→ NorM (COG0534)vmrA (PRK09575)→
env-574	JGI24713J26584_10104204	+	302	401	**RNA**→
env-575	DGDR01000018.1	-	6518	6419	**RNA**→ vmrA (PRK09575)MATE_MepA_like (cd13143)→
env-576	Ga0134102_1055137	-	131	32	**RNA**→
env-577	Ga0134092_1195192	-	240	141	**RNA**→ MATE_MepA_like (cd13143)→
env-578	JGI24713J26584_10026546	-	1465	1366	**RNA**→ vmrA (PRK09575)MATE_MepA_like (cd13143)→
env-579	JGI24714J26587_10011759	+	360	459	**RNA**→ vmrA (PRK09575)MATE_MepA_like (cd13143)→
env-580	JGI24707J26582_10001983	-	15394	15294	**RNA**→ vmrA (PRK09575)MATE_MepA_like (cd13143)→

env-581	JGI24708J26588_10001999	+	367	467	RNA→ vmrA (PRK09575)MATE_MepA_like (cd13143)→
env-582	JGI24714J26587_10009638	+	3215	3315	RNA→ vmrA (PRK09575)MATE_MepA_like (cd13143)→
env-583	JGI24712J26585_10002235	-	11857	11757	RNA→ vmrA (PRK09575)MATE_MepA_like (cd13143)→
env-584	JGI24711J26586_10001266	+	4129	4229	RNA→ vmrA (PRK09575)MATE_MepA_like (cd13143)→
env-585	JGI24709J26583_10005550	+	4129	4229	RNA→ vmrA (PRK09575)MATE_MepA_like (cd13143)→
env-586	JGI24710J26742_10006634	-	5002	4902	RNA→ vmrA (PRK09575)MATE_MepA_like (cd13143)→
Lna-1-1	NZ_AUEG01000001.1	-	420373	420272	RNA→ NorM (COG0534)Polysacc_synt_C (pfam14667)→
env-587	Ga0121719_101071	+	2791	2893	RNA→ NorM (COG0534)vmrA (PRK09575)→
env-588	Ga0121666_104501	+	376	478	RNA→ vmrA (PRK09575)MATE_MepA_like (cd13143)→
env-589	Ga0213827_1003707	+	2511	2613	RNA→ vmrA (PRK09575)MATE_MepA_like (cd13143)→
env-590	Ga0213829_1003708	+	479	581	RNA→ vmrA (PRK09575)MATE_MepA_like (cd13143)→
env-591	Ga0213820_1039503	-	452	350	RNA→ vmrA (PRK09575)MATE_MepA_like (cd13143)→
env-592	DDLB01000447.1	+	944	1046	RNA→ NorM (COG0534)vmrA (PRK09575)→
env-593	DFDC01000142.1	-	52919	52817	RNA→ vmrA (PRK09575)MATE_MepA_like (cd13143)→
env-594	DEYX01000162.1	+	25739	25841	RNA→ vmrA (PRK09575)MATE_MepA_like (cd13143)→
env-595	Ga0213822_1018003	+	121	223	RNA→ vmrA (PRK09575)MATE_MepA_like (cd13143)→
env-596	Ga0213825_1001881	+	11191	11293	RNA→ vmrA (PRK09575)MATE_MepA_like (cd13143)→
env-597	Ga0213828_1007430	-	4407	4305	RNA→ vmrA (PRK09575)MATE_MepA_like (cd13143)→
env-598	Ga0213826_1035022	+	563	665	RNA→ NorM (COG0534)vmrA (PRK09575)→
Csp-3-1	NZ_JFBV01000001.1	-	4368263	4368161	RNA→ vmrA (PRK09575)MATE_MepA_like (cd13143)→
[gu-1-1	NZ_FOIP01000002.1	-	2028016	2027914	RNA→ vmrA (PRK09575)MATE_MepA_like (cd13143)→
env-599	Ga0121387_114717	+	262	364	RNA→ hypo→
[in-1-1	NZ_AZUI01000001.1	+	4426203	4426305	RNA→ vmrA (PRK09575)MATE_MepA_like (cd13143)→
Air-1-1	NZ_FQWX01000022.1	+	11260	11361	RNA→ NorM (COG0534)vmrA (PRK09575)→
Csp-5-1	NZ_LXSB01000001.1	-	1827310	1827211	RNA→ vmrA (PRK09575)MATE_MepA_like (cd13143)→
Csp-16-1	NZ_LSMZ01000063.1	-	66885	66786	RNA→ vmrA (PRK09575)MATE_MepA_like (cd13143)→
env-600	JGI24713J26584_10121863	+	79	180	RNA→ vmrA (PRK09575)MATE_MepA_like (cd13143)→
env-601	JGI24714J26587_10130813	+	312	413	RNA→ hypo→
env-602	Ga0134102_1004629	+	140	241	RNA→ vmrA (PRK09575)MATE_MepA_like (cd13143)→ DUF45 (pfam01863)WLM (pfam08325)→
env-603	Ga0134092_1022043	-	379	278	RNA→ vmrA (PRK09575)MATE_MepA_like (cd13143)→
env-604	Ga0134099_1057266	-	427	326	RNA→ vmrA (PRK09575)MATE_MepA_like (cd13143)→
env-605	Ga0134101_1001420	+	12605	12706	RNA→ vmrA (PRK09575)MATE_MepA_like (cd13143)→ DUF45 (pfam01863)WLM (pfam08325)→
env-606	Ga0134100_1001733	+	13053	13154	RNA→ vmrA (PRK09575)MATE_MepA_like (cd13143)→ DUF45 (pfam01863)WLM (pfam08325)→
env-607	Ga0134104_1001612	+	12605	12706	RNA→ vmrA (PRK09575)MATE_MepA_like (cd13143)→ DUF45 (pfam01863)WLM (pfam08325)→
env-608	Ga0209100_1032859	+	368	469	RNA→ ←hypo
Dph-1-1	NZ_JWID01000011.1	+	108771	108872	RNA→ vmrA (PRK09575)MATE_MepA_like (cd13143)→
env-609	Ga0208826_1000188	+	61003	61104	RNA→ vmrA (PRK09575)MATE_MepA_like (cd13143)→
env-610	JGI24713J26584_10083602	-	188	87	RNA→
env-611	JGI24714J26587_10274799	-	149	48	RNA→
Mha-1-1	NZ_FUZT01000005.1	-	75781	75680	RNA→ NorM (COG0534)vmrA (PRK09575)→
env-612	JGI24707J26582_10127737	-	435	334	RNA→ vmrA (PRK09575)MATE_MepA_like (cd13143)→
env-613	JGI24708J26588_10064764	+	511	612	RNA→ NorM (COG0534)vmrA (PRK09575)→
env-614	JGI24712J26585_10014756	-	2892	2791	RNA→ vmrA (PRK09575)MATE_MepA_like (cd13143)→
env-615	JGI24711J26586_10025254	+	1612	1713	RNA→ NorM (COG0534)vmrA (PRK09575)→
env-616	JGI24709J26583_10004328	-	10301	10200	RNA→ vmrA (PRK09575)MATE_MepA_like (cd13143)→
env-617	JGI24710J26742_10003561	-	10297	10196	RNA→ vmrA (PRK09575)MATE_MepA_like (cd13143)→
env-618	JGI24714J26587_10317099	-	240	139	RNA→ hypo→
env-619	JGI24712J26585_10002457	+	14105	14205	RNA→ NorM (COG0534)vmrA (PRK09575)→
env-620	JGI24711J26586_10001962	+	14089	14189	RNA→ NorM (COG0534)vmrA (PRK09575)→
env-621	JGI24709J26583_10021999	-	1030	930	RNA→ NorM (COG0534)vmrA (PRK09575)→
env-622	JGI24710J26742_10016803	-	2185	2085	RNA→ vmrA (PRK09575)MATE_MepA_like (cd13143)→
env-623	Ga0134095_1004675	-	4850	4749	RNA→ vmrA (PRK09575)MATE_MepA_like (cd13143)→

env-624	Ga0134093_1003187	+	1722	1823	**RNA**→ vmrA (PRK09575)MATE_MepA_like (cd13143)→
env-625	DFNB01000014.1	+	43448	43548	**RNA**→ vmrA (PRK09575)MATE_MepA_like (cd13143)→
[st-1-1	NZ_BAVR01000025.1	+	46451	46551	**RNA**→ vmrA (PRK09575)MATE_MepA_like (cd13143)→
Cin-2-1	NZ_KI273145.1	-	2188644	2188543	**RNA**→ UDG_like_3 (cd10035)DUF4918 (pfam16265)→ UDG_like_3 (cd10035)→
env-626	SR_TP_S2_1000049	-	53041	52943	**RNA**→ NorM (COG0534)vmrA (PRK09575)→
env-627	JGI24707J26582_10034455	-	155	53	**RNA**→
env-628	JGI24708J26588_10041023	+	1000	1102	**RNA**→ NorM (COG0534)vmrA (PRK09575)→
env-629	JGI24709J26583_10005838	-	1131	1029	**RNA**→ NorM (COG0534)vmrA (PRK09575)→
env-630	JGI24710J26742_10007004	+	6604	6706	**RNA**→ NorM (COG0534)vmrA (PRK09575)→
env-631	JGI24709J26583_10360081	-	274	172	**RNA**→ MATE_MepA_like (cd13143)→
env-632	2156806709	-	407	307	**RNA**→ vmrA (PRK09575)MATE_MepA_like (cd13143)→
Til-1-1	NZ_FJNB01000002.1	+	135547	135646	**RNA**→ NorM (COG0534)vmrA (PRK09575)→
Tsp-1-1	NZ_FNYT01000019.1	+	41998	42097	**RNA**→ NorM (COG0534)vmrA (PRK09575)→
env-633	Ga0075011_10089533	+	19	119	**RNA**→ InsQ (COG0675)tspaseT_teng_C (TIGR01766)HTH_OrfB_IS605 (pfam12323)→
env-634	Ga0194137_10317758	-	606	506	**RNA**→ InsQ (COG0675)HTH_OrfB_IS605 (pfam12323)→
env-635	Ga0194136_1082675	-	495	395	**RNA**→ InsQ (COG0675)HTH_OrfB_IS605 (pfam12323)→
Tfl-1-1	NZ_FJMZ01000044.1	-	18752	18652	**RNA**→ InsQ (COG0675)tspaseT_teng_C (TIGR01766)HTH_OrfB_IS605 (pfam12323)→ NorM (COG0534)vmrA (PRK09575)→
Tfl-1-2	NZ_FOQC01000052.1	+	2481	2581	**RNA**→ InsQ (COG0675)tspaseT_teng_C (TIGR01766)HTH_OrfB_IS605 (pfam12323)→ NorM (COG0534)vmrA (PRK09575)→
env-636	Ga0122742_100238	+	485	586	**RNA**→ vmrA (PRK09575)MATE_MepA_like (cd13143)→
env-637	Ga0134091_1240126	-	120	20	**RNA**→
env-638	Ga0134091_1242056	+	142	242	**RNA**→
env-639	Ga0134092_1247784	+	142	242	**RNA**→
env-640	Ga0208938_1000005	+	249499	249599	**RNA**→ vmrA (PRK09575)MATE_MepA_like (cd13143)→ NorM (COG0534)vmrA (PRK09575)→
env-641	JGI24713J26584_10000003	+	39952	40052	**RNA**→ MATE_MepA_like (cd13143)Polysacc_synt_C (pfam14667)→
env-642	JGI24714J26587_10000001	-	30283	30183	**RNA**→ MATE_MepA_like (cd13143)Polysacc_synt_C (pfam14667)→
env-643	Ga0134090_1010905	+	872	972	**RNA**→ MATE_MepA_like (cd13143)Polysacc_synt_C (pfam14667)→
env-644	Ga0134105_1000007	+	244371	244471	**RNA**→ MATE_MepA_like (cd13143)Polysacc_synt_C (pfam14667)→
env-645	Ga0134098_1000062	+	106272	106372	**RNA**→ MATE_MepA_like (cd13143)Polysacc_synt_C (pfam14667)→
env-646	Ga0134101_1000016	+	244371	244471	**RNA**→ MATE_MepA_like (cd13143)Polysacc_synt_C (pfam14667)→
env-647	Ga0134097_1000013	-	7270	7170	**RNA**→ MATE_MepA_like (cd13143)Polysacc_synt_C (pfam14667)→
env-648	Ga0134102_1000025	-	7270	7170	**RNA**→ MATE_MepA_like (cd13143)Polysacc_synt_C (pfam14667)→
env-649	Ga0134095_1000012	+	222166	222266	**RNA**→ MATE_MepA_like (cd13143)Polysacc_synt_C (pfam14667)→
env-650	Ga0134096_1005681	-	3694	3594	**RNA**→ MATE_MepA_like (cd13143)Polysacc_synt_C (pfam14667)→
env-651	Ga0134100_1000009	+	244371	244471	**RNA**→ MATE_MepA_like (cd13143)Polysacc_synt_C (pfam14667)→
env-652	Ga0134104_1000014	+	244371	244471	**RNA**→ MATE_MepA_like (cd13143)Polysacc_synt_C (pfam14667)→
env-653	Ga0134093_1001522	+	14561	14661	**RNA**→ MATE_MepA_like (cd13143)Polysacc_synt_C (pfam14667)→
env-654	Ga0134094_1000392	+	46985	47085	**RNA**→ MATE_MepA_like (cd13143)Polysacc_synt_C (pfam14667)→
env-655	Ga0134103_1002150	-	7270	7170	**RNA**→ MATE_MepA_like (cd13143)Polysacc_synt_C (pfam14667)→
env-656	Ga0134099_1000023	+	215904	216004	**RNA**→ MATE_MepA_like (cd13143)Polysacc_synt_C (pfam14667)→
env-657	Ga0134091_1255463	+	127	227	**RNA**→
env-658	Ga0208823_1079663	-	187	87	**RNA**→
env-659	DPOH01000093.1	-	2474	2376	**RNA**→ SUA5 (COG0009)TIGR00057 (TIGR00057)→
Tma-1-1	NZ_FOQA01000001.1	-	552850	552747	**RNA**→ NorM (COG0534)vmrA (PRK09575)→ 2A51 (TIGR00937)Chromate_transp (pfam02417)→
Nfe-1-1	NZ_FNFP01000001.1	+	763922	764020	**RNA**→ NorM (COG0534)vmrA (PRK09575)→ B3_4 (smart00873)B3/B4 (COG3382)→ hypo→
Cfo-1-1	NZ_CP020559.1	-	3019483	3019385	**RNA**→ NorM (COG0534)vmrA (PRK09575)→ B3_4 (smart00873)B3/B4 (COG3382)→ Radical_SAM (cd01335)W_rSAM_matur (TIGR04317)→ LuxQ-periplasm (pfam09308)→
Cfo-1-2	NZ_CP017603.1	-	4213701	4213603	**RNA**→ NorM (COG0534)vmrA (PRK09575)→ B3_4 (smart00873)B3/B4 (COG3382)→ Radical_SAM (cd01335)W_rSAM_matur (TIGR04317)→ LuxQ-periplasm (pfam09308)→
Npe-1-1	NZ_FOHU01000011.1	-	20872	20775	**RNA**→ CBM_17_28 (pfam03424)→ SLH (pfam00395)→ hypo→
Gfe-1-1	NZ_CP017269.1	+	5080661	5080758	**RNA**→ NorM (COG0534)vmrA (PRK09575)→

Psp-1-1	NZ_AUID01000011.1	+	64566	64663	**RNA**→ hypo→ NorM (COG0534)vmrA (PRK09575)→ hypo→ B3_4 (smart00873)B3/B4 (COG3382)→ hypo→ hypo→ hypo→ BglX (COG1472)PRK05337 (PRK05337)→ Acetyltransf_1 (pfam00583)Acetyltransf_7 (pfam13508)→ AraC_E_bind (smart00871)GyrI-like (pfam06445)→
env-660	Ga0194138_10002573	-	1946	1849	**RNA**→ B3_4 (smart00873)B3_4 (pfam03483)→ B3/B4 (COG3382)→
env-661	Ga0194137_10003642	-	1974	1877	**RNA**→ B3_4 (smart00873)B3_4 (pfam03483)→ B3/B4 (COG3382)→
env-662	Ga0194136_1012504	-	1947	1850	**RNA**→ B3_4 (smart00873)B3_4 (pfam03483)→ B3/B4 (COG3382)→
env-663	PPYF01024588.1	-	52869	52771	**RNA**→ NorM (COG0534)Polysacc_synt_C (pfam14667)→
env-664	Pasolli2019-4763-35	+	50910	51011	**RNA**→ MATE_MepA_like (cd13143)matE (TIGR00797)→
Cba-1-1	NZ_DS990260.1	-	1404248	1404147	**RNA**→ vmrA (PRK09575)MATE_MepA_like (cd13143)→ vmrA (PRK09575)MATE_MepA_like (cd13143)→
Cba-6-1	NZ_BAIK02000154.1	+	5760	5861	**RNA**→ hypo→ hypo→
env-665	Pasolli2019-4700-19	-	15837	15739	**RNA**→ MATE_MepA_like (cd13143)matE (TIGR00797)→ DUF1275 (pfam06912)→
env-666	UMGS1015-17	-	15379	15281	**RNA**→ MATE_MepA_like (cd13143)matE (TIGR00797)→ DUF1275 (pfam06912)→
env-667	OGFR01003549.1	+	2492	2590	**RNA**→ NorM (COG0534)vmrA (PRK09575)→
env-668	OGGX01020188.1	-	390	292	**RNA**→ vmrA (PRK09575)MATE_MepA_like (cd13143)→
env-669	OGKA01068969.1	+	480	578	**RNA**→
env-670	Ga0169866_100605	+	11759	11857	**RNA**→ vmrA (PRK09575)MATE_MepA_like (cd13143)→ DUF1275 (pfam06912)→ hypo→
env-671	Ga0169821_102605	-	1349	1251	**RNA**→ NorM (COG0534)vmrA (PRK09575)→ vmrA (PRK09575)MATE_MepA_like (cd13143)→
Csp-2-1	NZ_JH376512.1	+	500070	500168	**RNA**→ vmrA (PRK09575)MATE_MepA_like (cd13143)→ DUF1275 (pfam06912)→ hypo→
Csy-2-1	NZ_GL834306.1	-	471199	471101	**RNA**→ vmrA (PRK09575)MATE_MepA_like (cd13143)→ DUF1275 (pfam06912)→ hypo→
env-672	UnmappedStool_Broad_scaffold_1136494	+	2896	2994	**RNA**→
env-673	DOM022_scaffold14296_7	+	1002	1100	**RNA**→
env-674	OGEP01007292.1	-	299	201	**RNA**→ MATE_MepA_like (cd13143)→
env-675	OGGQ01003877.1	+	11914	12012	**RNA**→ NorM (COG0534)vmrA (PRK09575)→
env-676	Ga0129309_1000247	+	16343	16441	**RNA**→ vmrA (PRK09575)MATE_MepA_like (cd13143)→ DUF1275 (pfam06912)→ hypo→
env-677	Ga0129308_1001857	+	16343	16441	**RNA**→ vmrA (PRK09575)MATE_MepA_like (cd13143)→ DUF1275 (pfam06912)→ hypo→
env-678	Ga0169873_101182	+	11894	11992	**RNA**→ vmrA (PRK09575)MATE_MepA_like (cd13143)→ DUF1275 (pfam06912)→ DUF1275 (pfam06912)→ hypo→
Jsy-1-1	NZ_CYZY01000027.1	-	31260	31162	**RNA**→ vmrA (PRK09575)MATE_MepA_like (cd13143)→ DUF1275 (pfam06912)→ hypo→
env-679	OGHO01084036.1	+	120	218	**RNA**→ vmrA (PRK09575)MATE_MepA_like (cd13143)→
env-680	OIZR01000577.1	-	14117	14019	**RNA**→ vmrA (PRK09575)MATE_MepA_like (cd13143)→ DUF1275 (pfam06912)→ hypo→
env-681	Ga0169833_100600	-	13015	12917	**RNA**→ vmrA (PRK09575)MATE_MepA_like (cd13143)→ DUF1275 (pfam06912)→ hypo→
env-682	C2721239_1_V1.CD-12	-	373	275	**RNA**→ vmrA (PRK09575)MATE_MepA_like (cd13143)→
Csy-3-1	NZ_GL834360.1	+	530966	531064	**RNA**→ vmrA (PRK09575)MATE_MepA_like (cd13143)→ DUF1275 (pfam06912)→ hypo→
env-683	Ga0169773_101121	+	9908	10006	**RNA**→ MATE_MepA_like (cd13143)→
env-684	OGEO01031585.1	-	373	275	**RNA**→ vmrA (PRK09575)MATE_MepA_like (cd13143)→
env-685	OIYD01002866.1	-	3822	3724	**RNA**→ vmrA (PRK09575)MATE_MepA_like (cd13143)→ DUF1275 (pfam06912)→ hypo→
env-686	Ga0129317_1000732	-	13991	13893	**RNA**→ vmrA (PRK09575)MATE_MepA_like (cd13143)→ DUF1275 (pfam06912)→ hypo→
env-687	Ga0129310_1000493	-	31347	31249	**RNA**→ vmrA (PRK09575)MATE_MepA_like (cd13143)→ DUF1275 (pfam06912)→ hypo→
env-688	Ga0169881_111446	-	355	257	**RNA**→ vmrA (PRK09575)MATE_MepA_like (cd13143)→
Csy-1-1	NZ_KE992861.1	+	11894	11992	**RNA**→ vmrA (PRK09575)MATE_MepA_like (cd13143)→ DUF1275 (pfam06912)→ hypo→ hypo→ hypo→ PRK13882 (PRK13882)TraX (pfam05857)→ rimI (TIGR01575)Acetyltransf_1 (pfam00583)→
Cba-4-1	NZ_BAID02000078.1	-	14181	14083	**RNA**→ hypo→ hypo→ DUF1275 (pfam06912)→ hypo→
Jsy-1-2	NZ_FIZZ01000002.1	-	471509	471411	**RNA**→ vmrA (PRK09575)MATE_MepA_like (cd13143)→ DUF1275 (pfam06912)→ hypo→
Csp-10-1	NZ_PDDG01000001.1	-	202172	202074	**RNA**→ vmrA (PRK09575)MATE_MepA_like (cd13143)→ DUF1275 (pfam06912)→ hypo→
env-689	SRS053335_C2377639	+	326	426	**RNA**→
env-690	Ga0121007_101205	-	2463	2359	**RNA**→ COG3603 (COG3603)ACT_7 (pfam13840)→ Peptidases_S8_CspA-like (cd07478)→
env-691	Ga0121719_104022	-	108	4	**RNA**→
env-692	Pasolli2019-6231-124	+	1950	2056	**RNA**→ NorM (COG0534)matE (TIGR00797)→
env-693	SRS024435_LANL_scaffold_33285	-	604	504	**RNA**→ PRK11431 (PRK11431)EmrE (COG2076)→
env-694	Ga0120802_134990	+	151	250	**RNA**→
env-695	Ga0121585_100003	-	142799	142700	**RNA**→ PRK11431 (PRK11431)EmrE (COG2076)→
Jae-1-1	NZ_JHWJ01000003.1	+	130361	130460	**RNA**→ PRK11431 (PRK11431)EmrE (COG2076)→
env-696	Pasolli2019-4165-484	+	1426	1525	**RNA**→ hypo→

env-697	Ga0134523_1056541	+	473	574	**RNA**→ PnuC (COG3201)NMN_trans_PnuC (TIGR01528)→
env-698	Pasolli2019-14892-21	+	3031	3132	**RNA**→ PnuC (COG3201)NMN_trans_PnuC (TIGR01528)→
[am-2-1	NZ_JONJ01000003.1	+	172941	173041	**RNA**→ Pyridox_oxidase (pfam01243)Pyridox_ox_2 (pfam12900)→
[am-1-1	NZ_FOZC01000003.1	-	140000	139900	**RNA**→ Pyridox_oxidase (pfam01243)Pyridox_ox_2 (pfam12900)→
env-699	OLGV01057690.1	-	218	118	**RNA**→
env-700	Pasolli2019-4301-318	+	1253	1353	**RNA**→ MATE_MepA_like (cd13143)→
env-701	UMGS1413-46	+	7719	7819	**RNA**→ NorM (COG0534)matE (TIGR00797)→
env-702	AUXO015323068.1	+	206	303	**RNA**→ hypo→
env-703	AUXO017306468.1	-	761	665	**RNA**→ B3_4 (smart00873)B3/B4 (COG3382)→
env-704	RUMENNODE_3563414_33500	+	11447	11544	**RNA**→ B3_4 (smart00873)B3/B4 (COG3382)→
env-705	ACUH01000011.1	+	2966	3070	**RNA**→ NorM (COG0534)Polysacc_synt_C (pfam14667)→
env-706	Pasolli2019-15506-67	-	2943	2839	**RNA**→ NorM (COG0534)Polysacc_synt_C (pfam14667)→
Ssp-2-1	NZ_JH414692.1	+	2966	3070	**RNA**→ NorM (COG0534)Polysacc_synt_C (pfam14667)→
env-707	UnmappedStool_Broad_scaffold_980043	+	171	271	**RNA**→ vmrA (PRK09575)MATE_MepA_like (cd13143)→
env-708	Pasolli2019-4143-60	+	4403	4492	**RNA**→ PnuC (COG3201)NMN_trans_PnuC (TIGR01528)→ hypo→
env-709	Ga0209203_1405121	-	163	63	**RNA**→
env-710	Ga0208458_1005145	+	319	419	**RNA**→ PnuC (COG3201)NMN_trans_PnuC (TIGR01528)→
env-711	Ga0208459_1001335	+	415	515	**RNA**→ PnuC (COG3201)NMN_trans_PnuC (TIGR01528)→
env-712	Ga0208040_1006165	+	238	338	**RNA**→ PnuC (COG3201)NMN_trans_PnuC (TIGR01528)→
env-713	Ga0256871_1029537	+	2164	2264	**RNA**→ NMN_transporter (pfam04973)→
env-714	4491686.3.38347	+	266	366	**RNA**→ hypo→ PnuC (COG3201)NMN_trans_PnuC (TIGR01528)→
env-715	4491686.3.1003501	-	990	890	**RNA**→ hypo→ PnuC (COG3201)NMN_trans_PnuC (TIGR01528)→
env-716	DFEX01000067.1	+	795	895	**RNA**→ PnuC (COG3201)NMN_trans_PnuC (TIGR01528)→
env-717	Ga0172377_12868047	-	238	135	**RNA**→
Bpi-1-1	NC_019908.1	-	1599384	1599284	**RNA**→ vmrA (PRK09575)MATE_MepA_like (cd13143)→
env-718	Ga0134523_1020267	-	2215	2115	**RNA**→ NMN_trans_PnuC (TIGR01528)NMN_transporter (pfam04973)→
					phd_fam (TIGR01552)PhdYeFM_antitox (pfam02604)→ ParE (COG3668)→
					PRK00723 (PRK00723)Psd (COG0688)→
env-719	Ga0129309_1000659	+	4356	4453	**RNA**→ ←hypo
env-720	Ga0129308_1000878	+	4283	4380	**RNA**→ ←hypo
env-721	OGEH01000780.1	-	3127	3030	**RNA**→ PRK11431 (PRK11431)EmrE (COG2076)→ MTHFR (cd00537)fadh2 (TIGR00676)→
					MetH2 (COG1410)metH (TIGR02082)→ MetH2 (COG1410)PRK08645 (PRK08645)S-methyl_trans (pfam02574)-
Exy-1-1	NZ_JAEB01000009.1	+	120783	120880	**RNA**→ hypo→ PRK11431 (PRK11431)EmrE (COG2076)→
env-722	AUXO010394370.1	-	1036	939	**RNA**→ ←NptA (COG1283)phoU_full (TIGR02135)Na_Pi_cotrans (pfam02690)
env-723	AUXO010056474.1	+	208	305	**RNA**→ PRK11431 (PRK11431)EmrE (COG2076)→
Sru-1-1	NZ_FPJA01000004.1	-	547043	546946	**RNA**→ Pyridox_oxidase (pfam01243)Pyridox_ox_2 (pfam12900)→
env-724	Ga0139311_1308508	+	199	297	**RNA**→ ←hypo
env-725	Ga0256405_10132292	+	1486	1583	**RNA**→ Pyridox_oxidase (pfam01243)Pyridox_ox_2 (pfam12900)→
env-726	UnmappedStool_Broad_scaffold_1067525	-	819	719	**RNA**→ vmrA (PRK09575)MATE_MepA_like (cd13143)→ vmrA (PRK09575)MATE_MepA_like (cd13143)→
env-727	Pasolli2019-8652-20	-	28518	28427	**RNA**→ EmrE (COG2076)PRK11431 (PRK11431)→ Pyridox_oxidase (pfam01243)Pyridox_ox_2 (pfam12900)→
					Acetyltransf_1 (pfam00583)Acetyltransf_10 (pfam13673)→
env-728	Ga0129308_1000003	-	256414	256316	**RNA**→ HTH_XRE (smart00530)HTH_XRE (cd00093)RGG_Cterm (TIGR01716)→
Lga-5-1	NZ_AMFD01000018.1	-	67140	67042	**RNA**→ HTH_XRE (smart00530)HTH_XRE (cd00093)RGG_Cterm (TIGR01716)→
					spxA (PRK01655)ArsC_Spx (cd03032)→ SdrD_B (pfam17210)→ hypo→ hypo→ PLN00134 (PLN00134)→
env-729	Ga0256406_1014341	-	129	34	**RNA**→
env-730	DBQQ01000029.1	+	1388	1482	**RNA**→ hypo→
env-731	Pasolli2019-4681-207	-	2188	2090	**RNA**→ NimA (COG3467)Pyridox_ox_2 (pfam12900)→ MATE_MepA_like (cd13143)matE (TIGR00797)→
Esp-12-1	NZ_NFLV01000008.1	-	97797	97699	**RNA**→ NimA (COG3467)Pyridox_ox_2 (pfam12900)→ vmrA (PRK09575)MATE_MepA_like (cd13143)→
Esp-13-1	NZ_NFIR01000006.1	-	31811	31713	**RNA**→ NimA (COG3467)Pyridox_ox_2 (pfam12900)→ vmrA (PRK09575)MATE_MepA_like (cd13143)→
env-732	UnmappedStool_Broad_C251001452	-	181	84	**RNA**→ hypo→
env-733	Ga0134492_1212780	-	250	153	**RNA**→ NimA (COG3467)→
env-734	UnmappedStool_Broad_C252166642	-	399	301	**RNA**→ Pyridox_oxidase (pfam01243)Pyridox_ox_2 (pfam12900)→
env-735	Pasolli2019-14855-23	+	3142	3240	**RNA**→ NimA (COG3467)Pyridox_ox_2 (pfam12900)→ MATE_MepA_like (cd13143)matE (TIGR00797)→

env-736	UMGS364-8	+	45002	45100	**RNA**→ NimA (COG3467)Pyridox_ox_2 (pfam12900)→ MATE_MepA_like (cd13143)matE (TIGR00797)→
env-737	Ga0134452_100094	+	78601	78699	**RNA**→ NimA (COG3467)Pyridox_ox_2 (pfam12900)→ vmrA (PRK09575)MATE_MepA_like (cd13143)→
env-738	OJAG01000157.1	+	3875	3973	**RNA**→ vmrA (PRK09575)MATE_MepA_like (cd13143)→
env-739	Pasolli2019-14863-71	+	3875	3973	**RNA**→ MATE_MepA_like (cd13143)matE (TIGR00797)→
env-740	Pasolli2019-4253-9	+	64729	64826	**RNA**→ MATE_MepA_like (cd13143)matE (TIGR00797)→
env-741	SRS019601_C1986939	+	294	391	**RNA**→ NorM (COG0534)vmrA (PRK09575)→
Msp-3-1	NZ_NFLD01000025.1	-	38250	38153	**RNA**→ vmrA (PRK09575)MATE_MepA_like (cd13143)→
Msp-1-1	NZ_NFLY01000025.1	+	24860	24957	**RNA**→ vmrA (PRK09575)MATE_MepA_like (cd13143)→
Msp-4-1	NZ_NFHI01000034.1	-	13100	13003	**RNA**→ vmrA (PRK09575)MATE_MepA_like (cd13143)→
Msp-2-1	NZ_NFLH01000026.1	+	27987	28084	**RNA**→ vmrA (PRK09575)MATE_MepA_like (cd13143)→
env-742	UMGS1038-17	+	9843	9940	**RNA**→ MATE_MepA_like (cd13143)matE (TIGR00797)→
Mti-1-1	NZ_NFLJ01000003.1	+	67549	67647	**RNA**→ NimA (COG3467)Pyridox_ox_2 (pfam12900)→ vmrA (PRK09575)MATE_MepA_like (cd13143)→
env-743	Pasolli2019-6768-33	+	10810	10908	**RNA**→ NimA (COG3467)Pyridox_ox_2 (pfam12900)→ MATE_MepA_like (cd13143)matE (TIGR00797)→
env-744	Pasolli2019-4878-60	-	10549	10451	**RNA**→ MetAP1 (cd01086)SEC-C (pfam02810)met_pdase_I (TIGR00500)SWIM_PBPRA1643 (TIGR04102)→
					Radical_SAM (pfam04055)NrdG2 (TIGR02495)→
					RlmK (COG1092)Methyltransf_25 (pfam13649)PUA (smart00359)→ hypo→ WrbA (COG0655)→
env-745	Pasolli2019-4679-3	-	9283	9185	**RNA**→ MATE_MepA_like (cd13143)Polysacc_synt_C (pfam14667)→
env-746	UMGS1037-24	-	14536	14439	**RNA**→ NimA (COG3467)Pyridox_ox_2 (pfam12900)→ MATE_MepA_like (cd13143)matE (TIGR00797)→
env-747	Pasolli2019-14139-17	-	16803	16706	**RNA**→ NimA (COG3467)Pyridox_ox_2 (pfam12900)→ MATE_MepA_like (cd13143)matE (TIGR00797)→
env-748	UnmappedStool_Broad_C252521115	-	361	264	**RNA**→ Pyridox_oxidase (pfam01243)Pyridox_ox_2 (pfam12900)→
env-749	Pasolli2019-14138-124	+	4701	4798	**RNA**→ Pyridox_oxidase (pfam01243)Pyridox_ox_2 (pfam12900)→
env-750	UMGS1471-13	-	21694	21597	**RNA**→ Pyridox_oxidase (pfam01243)Pyridox_ox_2 (pfam12900)→
Bma-1-1	NZ_LT574837.1	+	569875	569971	**RNA**→ NorM (COG0534)vmrA (PRK09575)→
env-751	Pasolli2019-4006-49	+	2485	2581	**RNA**→ NorM (COG0534)matE (TIGR00797)→
env-752	UMGS354-6	-	151093	150997	**RNA**→ NorM (COG0534)matE (TIGR00797)→
env-753	scaffold13947_29_V1.UC-18	-	6532	6437	**RNA**→ PRK11431 (PRK11431)EmrE (COG2076)→
env-754	scaffold55029_2_O2.UC-23	+	110	205	**RNA**→ PRK11431 (PRK11431)EmrE (COG2076)→
env-755	Pasolli2019-5787-187	-	634	539	**RNA**→ EmrE (COG2076)PRK11431 (PRK11431)→
env-756	UMGS1712-200	+	2403	2498	**RNA**→ EmrE (COG2076)PRK11431 (PRK11431)→
Lga-1-1	NZ_NFLS01000013.1	-	21750	21648	**RNA**→ hypo→
Lga-1-2	NZ_NFLZ01000014.1	+	22928	23030	**RNA**→ hypo→
Lsp-4-1	NZ_PCZD01000004.1	-	21698	21596	**RNA**→ hypo→
Lsp-3-1	NZ_PCYW01000003.1	-	27598	27496	**RNA**→ hypo→
env-757	Ga0172377_10147895	-	206	103	**RNA**→
env-758	Ga0172382_10013609	-	1215	1112	**RNA**→ PnuC (COG3201)NMN_trans_PnuC (TIGR01528)→
env-759	Ga0129315_1079245	-	349	244	**RNA**→ ←hypo
env-760	Ga0129317_1018444	-	905	800	**RNA**→ NorM (COG0534)vmrA (PRK09575)→
env-761	Ga0116616_1059791	-	284	185	**RNA**→ hypo→
env-762	Pasolli2019-4148-66	+	10728	10830	**RNA**→ NorM (COG0534)matE (TIGR00797)→
env-763	PPYF01020042.1	-	145	56	**RNA**→
env-764	Ga0177923_1267441	-	263	167	**RNA**→ hypo→
env-765	Ga0256406_1023450	-	1415	1317	**RNA**→ EmrE (COG2076)→ RelB (COG3077)RelB_DinJ (TIGR02384)→
					toxin_Txe_YoeB (TIGR02116)COG4115 (COG4115)→ LbH_MAT_like (cd04647)Mac (pfam12464)→
env-766	Ga0256404_1042493	-	1602	1504	**RNA**→ EmrE (COG2076)→ RelB (COG3077)RelB_DinJ (TIGR02384)→
					toxin_Txe_YoeB (TIGR02116)COG4115 (COG4115)→
					LbH_MAT_GAT (cd03357)Mac (pfam12464)Hexapep_2 (pfam14602)→
env-767	Ga0256405_10046736	-	1678	1580	**RNA**→ EmrE (COG2076)→ RelB (COG3077)RelB_DinJ (TIGR02384)→
					toxin_Txe_YoeB (TIGR02116)COG4115 (COG4115)→
					LbH_MAT_GAT (cd03357)Mac (pfam12464)Hexapep_2 (pfam14602)→
env-768	OMWS01000025.1	+	30566	30665	**RNA**→ RelB (COG3077)RelB_DinJ (TIGR02384)→ YoeB_toxin (pfam06769)toxin_Txe_YoeB (TIGR02116)→
env-769	Pasolli2019-4596-148	-	4585	4489	**RNA**→ EmrE (COG2076)PRK11431 (PRK11431)→
Lsp-9-1	NZ_NFIS01000004.1	+	132453	132549	**RNA**→ PRK11431 (PRK11431)EmrE (COG2076)→
env-770	Pasolli2019-4598-1	-	24478	24382	**RNA**→ EmrE (COG2076)PRK11431 (PRK11431)→

Lsp-10-1	NZ_NFHL01000004.1	+	201513	201608	**RNA**→ PRK11431 (PRK11431)EmrE (COG2076)→
Csp-15-1	NZ_LT635549.1	-	108295	108200	**RNA**→ PRK11431 (PRK11431)EmrE (COG2076)→
env-771	AUXO013304752.1	+	806	904	**RNA**→ PRK11431 (PRK11431)EmrE (COG2076)→
env-772	Ga0256406_1001782	+	13636	13734	**RNA**→ PRK11431 (PRK11431)EmrE (COG2076)→
env-773	Pasolli2019-14846-58	+	7735	7828	**RNA**→ MATE_MepA_like (cd13143)matE (TIGR00797)→
env-774	Pasolli2019-14850-28	+	943	1040	**RNA**→ EmrE (COG2076)PRK11431 (PRK11431)→
env-775	Pasolli2019-4063-56	+	1634	1731	**RNA**→ EmrE (COG2076)PRK11431 (PRK11431)→
env-776	UnmappedStool_Broad_scaffold_892469	+	683	780	**RNA**→ PRK11431 (PRK11431)EmrE (COG2076)→
env-777	Pasolli2019-4062-37	-	501	404	**RNA**→ EmrE (COG2076)PRK11431 (PRK11431)→ hypo→
env-778	Pasolli2019-5748-5	-	25371	25274	**RNA**→ EmrE (COG2076)PRK11431 (PRK11431)→
env-779	UMGS733-4	+	79164	79261	**RNA**→ EmrE (COG2076)PRK11431 (PRK11431)→
env-780	Pasolli2019-4802-11	+	27953	28049	**RNA**→ EmrE (COG2076)PRK11431 (PRK11431)→
env-781	Ga0129309_1001406	-	11484	11384	**RNA**→ NorM (COG0534)vmrA (PRK09575)→
env-782	NOF005_scaffold31594_7	+	10535	10635	**RNA**→ NorM (COG0534)vmrA (PRK09575)→
env-783	NOM015_scaffold252_5	+	12748	12848	**RNA**→ NorM (COG0534)vmrA (PRK09575)→
env-784	OGFM01044986.1	+	302	402	**RNA**→ hypo→
env-785	AUXO015750006.1	-	143	42	**RNA**→
env-786	OGNB01074911.1	-	515	414	**RNA**→ PnuC (COG3201)→
env-787	Ga0172382_10027237	+	6051	6156	**RNA**→ PnuC (COG3201)→
env-788	Pasolli2019-4212-21	+	32081	32182	**RNA**→ PnuC (COG3201)NMN_trans_PnuC (TIGR01528)→
env-789	UMGS200-169	-	4545	4444	**RNA**→ PnuC (COG3201)NMN_trans_PnuC (TIGR01528)→
env-790	Ga0134523_1001861	+	13392	13490	**RNA**→ vmrA (PRK09575)MATE_MepA_like (cd13143)→ PRK04460 (PRK04460)RHH_1 (pfam01402)→
env-791	SRS014613_WUGC_scaffold_33931	-	915	817	**RNA**→ NorM (COG0534)vmrA (PRK09575)→
env-792	UnmappedStool_Broad_scaffold_1130971	+	440	538	**RNA**→ vmrA (PRK09575)MATE_MepA_like (cd13143)→
env-793	OGDU01022217.1	+	1395	1493	**RNA**→
env-794	BABD01006045.1	+	1062	1160	**RNA**→ vmrA (PRK09575)MATE_MepA_like (cd13143)→
env-795	DMIF01000006.1	+	14331	14429	**RNA**→ vmrA (PRK09575)MATE_MepA_like (cd13143)→ PRK04460 (PRK04460)RHH_1 (pfam01402)→
env-796	DPND01000045.1	-	16560	16462	**RNA**→ vmrA (PRK09575)MATE_MepA_like (cd13143)→ PRK04460 (PRK04460)RHH_1 (pfam01402)→
env-797	PPYF01027092.1	+	4571	4669	**RNA**→ vmrA (PRK09575)MATE_MepA_like (cd13143)→ PRK04460 (PRK04460)RHH_1 (pfam01402)→
env-798	DLVP01000082.1	-	5256	5155	**RNA**→ PnuC (COG3201)NMN_trans_PnuC (TIGR01528)→
env-799	DLBD01000073.1	-	3048	2949	**RNA**→ PnuC (COG3201)NMN_trans_PnuC (TIGR01528)→ GH36 (cd14791)Glyco_hydro_36C (pfam16874)Glyco_hydro_36N (pfam16875)→
env-800	Ga0209064_1012300	+	2432	2534	**RNA**→ PnuC (COG3201)NMN_trans_PnuC (TIGR01528)→
env-801	Ga0134563_102291	-	1261	1169	**RNA**→ PnuC (COG3201)→
env-802	Ga0209064_1235640	+	90	189	**RNA**→ hypo→
env-803	scaffold42385_2_MH0014	-	811	707	**RNA**→ PnuC (COG3201)NMN_trans_PnuC (TIGR01528)→
env-804	scaffold29813_3_MH0036	-	161	57	**RNA**→
env-805	SRS023914_C1980823	+	342	446	**RNA**→
env-806	SRS052697_C3126595	-	173	69	**RNA**→
env-807	DNZV01000036.1	-	1051	947	**RNA**→ PnuC (COG3201)NMN_trans_PnuC (TIGR01528)→ hypo→
env-808	DPVN01000012.1	-	5059	4955	**RNA**→ PnuC (COG3201)NMN_trans_PnuC (TIGR01528)→ hypo→
env-809	OGHH01032378.1	-	633	529	**RNA**→ PnuC (COG3201)NMN_trans_PnuC (TIGR01528)→
env-810	UMGS130-18	-	4943	4839	**RNA**→ PnuC (COG3201)NMN_trans_PnuC (TIGR01528)→
env-811	OGDS01003222.1	+	7517	7621	**RNA**→ PnuC (COG3201)NMN_trans_PnuC (TIGR01528)→
env-812	OGEL01003222.1	+	7517	7621	**RNA**→ PnuC (COG3201)NMN_trans_PnuC (TIGR01528)→
env-813	OGGZ01001290.1	-	996	892	**RNA**→ PnuC (COG3201)NMN_trans_PnuC (TIGR01528)→
env-814	OGIK01000500.1	-	4915	4811	**RNA**→ PnuC (COG3201)NMN_trans_PnuC (TIGR01528)→
env-815	Ga0134403_110157	-	231	127	**RNA**→ hypo→
env-816	Ga0134372_1001743	+	7230	7334	**RNA**→ hypo→
env-817	Ga0169813_114541	+	341	445	**RNA**→ PnuC (COG3201)NMN_trans_PnuC (TIGR01528)→
env-818	scaffold116602_2_MH0011	+	335	439	**RNA**→ PnuC (COG3201)NMN_trans_PnuC (TIGR01528)→
env-819	scaffold3321_2_MH0026	-	833	729	**RNA**→ PnuC (COG3201)NMN_trans_PnuC (TIGR01528)→

env-820	scaffold7369_5_V1.CD-6	+	332	436	**RNA**→ PnuC (COG3201)NMN_trans_PnuC (TIGR01528)→
env-821	SRS011302_Baylor_scaffold_23074	+	3341	3445	**RNA**→ PnuC (COG3201)NMN_trans_PnuC (TIGR01528)→
env-822	SRS018351_C2634597	+	86	190	**RNA**→ hypo→
env-823	SRS024331_C2391959	-	824	720	**RNA**→ PnuC (COG3201)NMN_trans_PnuC (TIGR01528)→
env-824	OGLI01005393.1	-	997	893	**RNA**→ PnuC (COG3201)NMN_trans_PnuC (TIGR01528)→
env-825	scaffold202452_2_MH0006	-	470	366	**RNA**→ PnuC (COG3201)NMN_trans_PnuC (TIGR01528)→
env-826	SRS016335_WUGC_scaffold_44876	+	2245	2349	**RNA**→ PnuC (COG3201)NMN_trans_PnuC (TIGR01528)→
env-827	OLGB01070317.1	-	288	184	**RNA**→ hypo→
env-828	Ga0169849_142867	-	306	202	**RNA**→ hypo→
env-829	Pasolli2019-4191-22	+	40324	40428	**RNA**→ PnuC (COG3201)NMN_trans_PnuC (TIGR01528)→
env-830	4491413.3_NODE_82482	-	5121	5017	**RNA**→ PnuC (COG3201)NMN_trans_PnuC (TIGR01528)→
env-831	4491421.3_NODE_52406	+	713	817	**RNA**→ PnuC (COG3201)NMN_trans_PnuC (TIGR01528)→
env-832	4491487.3_NODE_279	-	1017	913	**RNA**→ PnuC (COG3201)NMN_trans_PnuC (TIGR01528)→
env-833	UnmappedStool_Broad_scaffold_1119756	-	666	562	**RNA**→ PnuC (COG3201)NMN_trans_PnuC (TIGR01528)→
env-834	DQFX01000033.1	-	5142	5038	**RNA**→ PnuC (COG3201)NMN_trans_PnuC (TIGR01528)→
env-835	OGLM01021151.1	+	1585	1689	**RNA**→ PnuC (COG3201)NMN_trans_PnuC (TIGR01528)→
env-836	OGMP01005406.1	-	757	653	**RNA**→ PnuC (COG3201)NMN_trans_PnuC (TIGR01528)→
env-837	Ga0134450_102071	-	4360	4256	**RNA**→ PnuC (COG3201)NMN_trans_PnuC (TIGR01528)→
env-838	Ga0134428_107097	-	370	266	**RNA**→ PnuC (COG3201)NMN_trans_PnuC (TIGR01528)→
env-839	Ga0134399_1000014	-	4828	4724	**RNA**→ PnuC (COG3201)NMN_trans_PnuC (TIGR01528)→ hypo→
env-840	scaffold4788_1_MH0070	-	941	837	**RNA**→ PnuC (COG3201)NMN_trans_PnuC (TIGR01528)→
env-841	SRS011134_C5115507	-	827	723	**RNA**→ PnuC (COG3201)NMN_trans_PnuC (TIGR01528)→
env-842	SRS013687_C2644117	-	945	841	**RNA**→ PnuC (COG3201)NMN_trans_PnuC (TIGR01528)→
env-843	SRS014923_C3290749	-	736	632	**RNA**→ PnuC (COG3201)NMN_trans_PnuC (TIGR01528)→
env-844	SRS016095_WUGC_scaffold_30697	+	14729	14833	**RNA**→ PnuC (COG3201)NMN_trans_PnuC (TIGR01528)→
env-845	SRS019582_WUGC_scaffold_91358	-	811	707	**RNA**→ PnuC (COG3201)NMN_trans_PnuC (TIGR01528)→
env-846	SRS020233_C4262878	-	964	860	**RNA**→ PnuC (COG3201)NMN_trans_PnuC (TIGR01528)→
env-847	SRS022609_Baylor_scaffold_89528	+	3570	3674	**RNA**→ PnuC (COG3201)NMN_trans_PnuC (TIGR01528)→
env-848	NLF005_scaffold15835_1	-	954	850	**RNA**→ PnuC (COG3201)NMN_trans_PnuC (TIGR01528)→
env-849	NOM016_scaffold3623_3	-	648	544	**RNA**→ PnuC (COG3201)NMN_trans_PnuC (TIGR01528)→
env-850	OGHO01031332.1	+	344	448	**RNA**→ PnuC (COG3201)NMN_trans_PnuC (TIGR01528)→
env-851	OLFT01005769.1	+	5969	6073	**RNA**→ PnuC (COG3201)NMN_trans_PnuC (TIGR01528)→
env-852	SRS013476_C3110157	-	169	65	**RNA**→
env-853	SRS024132_C3905971	+	2534	2638	**RNA**→ PnuC (COG3201)NMN_trans_PnuC (TIGR01528)→
env-854	SRS024435_C3226101	+	2621	2725	**RNA**→ PnuC (COG3201)NMN_trans_PnuC (TIGR01528)→
env-855	Ga0134418_1043673	+	73	177	**RNA**→ PnuC (COG3201)NMN_trans_PnuC (TIGR01528)→
env-856	Ga0134523_1004605	-	4981	4877	**RNA**→ PnuC (COG3201)NMN_trans_PnuC (TIGR01528)→ hypo→ hypo→
env-857	SRS024075_LANL_scaffold_24006	+	4180	4284	**RNA**→ PnuC (COG3201)NMN_trans_PnuC (TIGR01528)→
env-858	SRS011061_C4896130	+	2516	2620	**RNA**→ PnuC (COG3201)NMN_trans_PnuC (TIGR01528)→
env-859	SRS015854_WUGC_scaffold_30461	+	4764	4868	**RNA**→ PnuC (COG3201)NMN_trans_PnuC (TIGR01528)→
env-860	SRS050925_C1944579	+	142	246	**RNA**→
env-861	SRS063040_Baylor_scaffold_32128	+	3	107	**RNA**→ PnuC (COG3201)→
env-862	Pasolli2019-4190-4	-	108107	108004	**RNA**→ PnuC (COG3201)NMN_trans_PnuC (TIGR01528)→
env-863	AUXO017224907.1	-	832	741	**RNA**→ PnuC (COG3201)NMN_trans_PnuC (TIGR01528)→
env-864	JGI20225J20221_1000065	-	261766	261675	**RNA**→ agcS (TIGR00835)Na_Ala_symp (pfam01235)→ PLPDE_III_AR (cd00430)alr (PRK00053)→
env-865	BMHB3a_c93076	-	329	238	**RNA**→ AlsT (COG1115)agcS (TIGR00835)→
env-866	Ga0139311_1002847	+	20567	20658	**RNA**→ PRK11431 (PRK11431)EmrE (COG2076)→
env-867	Ga0139361_1084275	-	281	190	**RNA**→ PRK11431 (PRK11431)EmrE (COG2076)→
env-868	Ga0134540_1100142	-	648	549	**RNA**→ hypo→
env-869	OGIV01053362.1	-	269	164	**RNA**→ MATE_MepA_like (cd13143)→
env-870	UMGS1068-154	+	392	497	**RNA**→ MATE_MepA_like (cd13143)matE (TIGR00797)→
env-871	Pasolli2019-6041-248	+	658	763	**RNA**→ MATE_MepA_like (cd13143)matE (TIGR00797)→

env-872	4491479.3_NODE_7110	-	70590	70486	**RNA**→ vmrA (PRK09575)MATE_MepA_like (cd13143)→ NorM (COG0534)vmrA (PRK09575)→
env-873	DNWG01000010.1	+	309	413	**RNA**→ vmrA (PRK09575)MATE_MepA_like (cd13143)→
env-874	OGCU01006357.1	-	1295	1191	**RNA**→ NorM (COG0534)vmrA (PRK09575)→
env-875	OGEK01002297.1	+	291	395	**RNA**→ vmrA (PRK09575)MATE_MepA_like (cd13143)→
env-876	OGJC01000205.1	+	385	489	**RNA**→ vmrA (PRK09575)MATE_MepA_like (cd13143)→
env-877	Pasolli2019-6038-116	-	7993	7889	**RNA**→ MATE_MepA_like (cd13143)matE (TIGR00797)→
Fva-1-1	NZ_GL987995.1	-	1316246	1316142	**RNA**→ vmrA (PRK09575)MATE_MepA_like (cd13143)→
Fsp-3-1	NZ_KV801673.1	-	71926	71822	**RNA**→ vmrA (PRK09575)MATE_MepA_like (cd13143)→
env-878	UnmappedStool_Broad_C253397671	+	470	561	**RNA**→ Pyridox_oxidase (pfam01243)Pyridox_ox_2 (pfam12900)→
env-879	Pasolli2019-15123-7	+	57965	58056	**RNA**→ NimA (COG3467)Pyridox_ox_2 (pfam12900)→
env-880	JGI20225J20221_1000275	-	132828	132737	**RNA**→ NimA (COG3467)Pyridox_ox_2 (pfam12900)→ hypo→
env-881	Ga0121633_100019	+	92848	92938	**RNA**→ NimA (COG3467)Pyridox_ox_2 (pfam12900)→
env-882	Ga0208826_1076971	+	928	1020	**RNA**→
env-883	Ga0208826_1273450	-	431	339	**RNA**→ PRK11431 (PRK11431)EmrE (COG2076)→
env-884	Ga0208695_1050249	-	1385	1293	**RNA**→ PRK11431 (PRK11431)EmrE (COG2076)→
env-885	JGI20225J20221_1066742	-	133	40	**RNA**→
env-886	DCDW01000051.1	-	43760	43669	**RNA**→ 2A51 (TIGR00937)Chromate_transp (pfam02417)→ 2A51 (TIGR00937)Chromate_transp (pfam02417)→
env-887	DHQX01000030.1	+	3735	3826	**RNA**→ 2A51 (TIGR00937)Chromate_transp (pfam02417)→ 2A51 (TIGR00937)Chromate_transp (pfam02417)→
env-888	scaffold57117_2_O2.UC-23	-	2642	2552	**RNA**→ Radical_SAM (cd01335)moaA_archaeal (TIGR02668)→
env-889	Pasolli2019-5787-40	-	5013	4923	**RNA**→ Radical_SAM (cd01335)moaA_archaeal (TIGR02668)→
env-890	UMGS1712-35	-	5266	5176	**RNA**→ Radical_SAM (cd01335)moaA_archaeal (TIGR02668)→
env-891	Ga0129308_1002207	+	12866	12962	**RNA**→ PnuC (COG3201)NMN_trans_PnuC (TIGR01528)→ MFS (cd06174)MFS_1_like (pfam12832)→<br>ACH1 (COG0427)AcetylCoA_hyd_C (pfam13336)→
env-892	Ga0129309_1104088	-	338	242	**RNA**→ hypo→
env-893	DOAW01000036.1	-	67220	67132	**RNA**→ NorM (COG0534)vmrA (PRK09575)→
env-894	UMGS261-1	+	66040	66128	**RNA**→ NorM (COG0534)matE (TIGR00797)→
env-895	SRS016495_C1869323	+	357	445	**RNA**→
env-896	SRS016517_C20279779	-	364	276	**RNA**→ vmrA (PRK09575)MATE_MepA_like (cd13143)→
env-897	UnmappedStool_Broad_C251758448	+	70	159	**RNA**→ vmrA (PRK09575)MATE_MepA_like (cd13143)→
env-898	Ga0075011_10829266	+	14	105	**RNA**→ hypo→
env-899	Pasolli2019-4723-13	+	33556	33647	**RNA**→ hypo→
env-900	OGGQ01100161.1	+	328	419	**RNA**→
env-901	Ga0134434_1003085	+	3995	4086	**RNA**→ Radical_SAM (cd01335)W_rSAM_matur (TIGR04317)→
env-902	UnmappedStool_Broad_C253151209	+	170	261	**RNA**→ Radical_SAM (cd01335)W_rSAM_matur (TIGR04317)→
env-903	Pasolli2019-4722-32	-	1755	1664	**RNA**→ Radical_SAM (cd01335)W_rSAM_matur (TIGR04317)→<br>XapA (COG0005)PNPH-PUNA-XAPA (TIGR01697)→
env-904	UMGS945-25	+	36770	36861	**RNA**→ Radical_SAM (cd01335)W_rSAM_matur (TIGR04317)→<br>XapA (COG0005)PNPH-PUNA-XAPA (TIGR01697)→ ATZ_TRZ_like (cd01298)Se_ssnA (TIGR03314)→
env-905	Pasolli2019-4698-19	+	31790	31879	**RNA**→ MATE_MepA_like (cd13143)matE (TIGR00797)→
env-906	Ga0209064_1143939	-	125	35	**RNA**→
env-907	Pasolli2019-6793-201	-	1233	1141	**RNA**→ NimA (COG3467)Pyridox_ox_2 (pfam12900)→ NorM (COG0534)matE (TIGR00797)→
env-908	Pasolli2019-4594-13	-	37184	37092	**RNA**→ NimA (COG3467)Pyridox_ox_2 (pfam12900)→
env-909	UMGS362-12	+	60824	60916	**RNA**→ NimA (COG3467)Pyridox_ox_2 (pfam12900)→
Cbo-2-1	NZ_JXMR01000001.1	-	2647943	2647837	**RNA**→ vmrA (PRK09575)MATE_MepA_like (cd13143)→
env-910	Ga0208938_1096150	+	348	451	**RNA**→ vmrA (PRK09575)MATE_MepA_like (cd13143)→
env-911	DBGA01000084.1	-	29477	29373	**RNA**→ NorM (COG0534)vmrA (PRK09575)→
env-912	JGI24714J26587_10172628	+	158	262	**RNA**→ MATE_MepA_like (cd13143)→
env-913	Ga0129308_1003732	-	7193	7101	**RNA**→ NimA (COG3467)Pyridox_ox_2 (pfam12900)→
env-914	Ga0121474_101121	+	1590	1681	**RNA**→ Pyridox_oxidase (pfam01243)Pyridox_ox_2 (pfam12900)→
env-915	Ga0134523_1060229	-	372	283	**RNA**→ Pyridox_oxidase (pfam01243)Pyridox_ox_2 (pfam12900)→
env-916	Ga0134538_1458069	+	206	286	**RNA**→
env-917	Ga0134540_1280486	-	236	147	**RNA**→ hypo→
env-918	OIXA01003455.1	+	12917	13009	**RNA**→ vmrA (PRK09575)MATE_MepA_like (cd13143)→

env-919	OIXG01026782.1	+	282	374	**RNA**→ NorM (COG0534)NagC (COG1940)ROK_glcA_fam (TIGR00744)Polysacc_synt_C (pfam14667)→
env-920	OLHE01001144.1	-	1543	1451	**RNA**→ vmrA (PRK09575)MATE_MepA_like (cd13143)→
env-921	Ga0134523_1001411	-	33975	33883	**RNA**→ vmrA (PRK09575)MATE_MepA_like (cd13143)→
env-922	scaffold64665_3_MH0048	-	758	666	**RNA**→ NorM (COG0534)vmrA (PRK09575)→
env-923	MNRD01000261.1	-	10432	10340	**RNA**→ vmrA (PRK09575)MATE_MepA_like (cd13143)→
env-924	OIZQ01004198.1	+	2285	2377	**RNA**→ vmrA (PRK09575)MATE_MepA_like (cd13143)→
env-925	OLFU01001169.1	+	21158	21250	**RNA**→ vmrA (PRK09575)MATE_MepA_like (cd13143)→
env-926	OLFY01002934.1	-	10558	10466	**RNA**→ vmrA (PRK09575)MATE_MepA_like (cd13143)→
env-927	Ga0121354_101943	-	391	300	**RNA**→ NimA (COG3467)Pyridox_ox_2 (pfam12900)→
env-928	Ga0208040_1087033	+	726	815	**RNA**→ NimA (COG3467)Pyridox_ox_2 (pfam12900)→
env-929	Ga0208694_1148030	-	108	19	**RNA**→
env-930	JGI24712J26585_10082144	-	993	903	**RNA**→ NorM (COG0534)vmrA (PRK09575)→
env-931	JGI24709J26583_10000883	-	12066	11976	**RNA**→ vmrA (PRK09575)MATE_MepA_like (cd13143)→
env-932	JGI24710J26742_10000044	+	17806	17896	**RNA**→ vmrA (PRK09575)MATE_MepA_like (cd13143)→
env-933	Ga0122112_114901	-	250	159	**RNA**→ NimA (COG3467)Pyridox_ox_2 (pfam12900)→
Pvi-1-1	NZ_FODY01000027.1	-	23138	23047	**RNA**→ NimA (COG3467)Pyridox_ox_2 (pfam12900)→
env-934	JGI24731J21663_1207274	-	142	51	**RNA**→
env-935	Pasolli2019-14315-64	+	8951	9041	**RNA**→ MATE_MepA_like (cd13143)Polysacc_synt_C (pfam14667)→
env-936	Ga0120841_154270	+	205	295	**RNA**→
env-937	DLWH01000354.1	-	4630	4539	**RNA**→ NimA (COG3467)Pyridox_ox_2 (pfam12900)→
env-938	Ga0121678_100005	-	138370	138279	**RNA**→ NimA (COG3467)Pyridox_ox_2 (pfam12900)→
env-939	PRSSGFe2_Sequence0000062009	+	185	276	**RNA**→ NimA (COG3467)Pyridox_ox_2 (pfam12900)→
bMS-1-1	NZ_LM994646.1	+	105984	106075	**RNA**→ NimA (COG3467)Pyridox_ox_2 (pfam12900)→
env-940	Ga0122069_103547	+	363	454	**RNA**→ NimA (COG3467)Pyridox_ox_2 (pfam12900)→
env-941	Ga0134538_1055580	+	972	1061	**RNA**→ ←hypo
Epl-2-1	NZ_KB822471.1	-	396067	395978	**RNA**→ NimA (COG3467)Pyridox_ox_2 (pfam12900)→ vmrA (PRK09575)MATE_MepA_like (cd13143)→
env-942	Pasolli2019-3971-219	+	3228	3317	**RNA**→ NimA (COG3467)Pyridox_ox_2 (pfam12900)→<br>FadR (COG2186)FCD (smart00895)HTH_GNTR (smart00345)→
env-943	UMGS1437-330	-	1337	1248	**RNA**→ NimA (COG3467)Pyridox_ox_2 (pfam12900)→<br>FadR (COG2186)FCD (smart00895)HTH_GNTR (smart00345)→
env-944	Ga0177923_1330810	-	620	531	**RNA**→ ←Transposase_20 (pfam02371)
env-945	Ga0209064_1054005	-	410	320	**RNA**→ REC (smart00448)REC (cd00156)→
env-946	DEKP01000026.1	+	21247	21349	**RNA**→ PnuC (COG3201)NMN_trans_PnuC (TIGR01528)→
env-947	Ga0256405_10143358	+	277	378	**RNA**→ PnuC (COG3201)NMN_trans_PnuC (TIGR01528)→
env-948	UMGS505-25	-	19339	19236	**RNA**→ PnuC (COG3201)NMN_trans_PnuC (TIGR01528)→ hypo→
env-949	DDJI01000082.1	+	65	168	**RNA**→ PnuC (COG3201)NMN_trans_PnuC (TIGR01528)→ hypo→
env-950	Pasolli2019-4175-0	-	107871	107768	**RNA**→ PnuC (COG3201)NMN_trans_PnuC (TIGR01528)→
env-951	DGUQ01000048.1	-	3705	3595	**RNA**→ PnuC (COG3201)NMN_trans_PnuC (TIGR01528)→
env-952	BMHB3a_c134841	+	800	900	**RNA**→ Pyridox_oxidase (pfam01243)Pyridox_ox_2 (pfam12900)→
env-953	JGI20225J20221_1011643	+	692	792	**RNA**→ NimA (COG3467)Pyridox_ox_2 (pfam12900)→
env-954	OMUY01000016.1	-	22576	22478	**RNA**→ PnuC (COG3201)NMN_trans_PnuC (TIGR01528)→
env-955	Ga0116650_1000082	+	8248	8346	**RNA**→ vmrA (PRK09575)MATE_MepA_like (cd13143)→
env-956	Ga0116619_1107154	-	266	168	**RNA**→ MATE_MepA_like (cd13143)→
env-957	DLKL01000020.1	+	6636	6734	**RNA**→ vmrA (PRK09575)MATE_MepA_like (cd13143)→
env-958	Ga0129309_1014524	-	1019	923	**RNA**→ NorM (COG0534)vmrA (PRK09575)→
env-959	Ga0129308_1005431	-	1556	1460	**RNA**→ vmrA (PRK09575)MATE_MepA_like (cd13143)→
env-960	BMHB3a_c27837	-	286	186	**RNA**→ PRK11431 (PRK11431)EmrE (COG2076)→
env-961	JGI20225J20221_1001704	-	12616	12516	**RNA**→ PRK11431 (PRK11431)EmrE (COG2076)→ B3_4 (smart00873)B3/B4 (COG3382)→ hypo→
env-962	JGI2065J20421_1010636	-	1327	1227	**RNA**→ PRK11431 (PRK11431)EmrE (COG2076)→ B3_4 (smart00873)B3/B4 (COG3382)→
env-963	UnmappedStool_Broad_scaffold_1133501	+	435	536	**RNA**→ vmrA (PRK09575)MATE_MepA_like (cd13143)→
env-964	Pasolli2019-3813-5	-	10608	10507	**RNA**→ MATE_MepA_like (cd13143)matE (TIGR00797)→
env-965	SRS042628_C2718343	+	1043	1144	**RNA**→
env-966	Pasolli2019-4803-17	-	45401	45304	**RNA**→ MATE_MepA_like (cd13143)matE (TIGR00797)→

env-967	UMGS96-1	-	110273	110176	**RNA**→ MATE_MepA_like (cd13143)matE (TIGR00797)→
env-968	OLGD01000527.1	-	66553	66456	**RNA**→ vmrA (PRK09575)MATE_MepA_like (cd13143)→
env-969	Ga0129311_1000808	-	2807	2702	**RNA**→ B3_4 (smart00873)B3/B4 (COG3382)→ hypo→
env-970	Pasolli2019-4255-18	+	1204	1304	**RNA**→ NorM (COG0534)matE (TIGR00797)→
					PLDc_SMU_988_like_2 (cd09160)PLDc_SMU_988_like_1 (cd09154)PLDc_2 (pfam13091)PLDc_N (pfam13396)→
env-971	DNTZ01000007.1	-	11982	11868	**RNA**→ NorM (COG0534)Polysacc_synt_C (pfam14667)→
env-972	OIXS01032965.1	-	407	310	**RNA**→ vmrA (PRK09575)MATE_MepA_like (cd13143)→
env-973	UMGS207-4	+	5608	5704	**RNA**→ EmrE (COG2076)PRK11431 (PRK11431)→
env-974	OLGO01000115.1	+	79513	79609	**RNA**→ PRK11431 (PRK11431)EmrE (COG2076)→
env-975	Pasolli2019-3993-0	+	133351	133447	**RNA**→ EmrE (COG2076)PRK11431 (PRK11431)→
env-976	Pasolli2019-14867-9	-	66800	66700	**RNA**→ MATE_MepA_like (cd13143)matE (TIGR00797)→
env-977	OGDY01000370.1	-	1603	1498	**RNA**→ hypo→
env-978	OIWT01001309.1	-	23160	23051	**RNA**→ B3_4 (smart00873)B3/B4 (COG3382)→ hypo→
env-979	OLGE01013253.1	+	1509	1618	**RNA**→ B3_4 (smart00873)B3/B4 (COG3382)→ hypo→
env-980	Pasolli2019-3989-56	+	2128	2237	**RNA**→ B3/B4 (COG3382)B3_4 (smart00873)→ hypo→
env-981	OLHE01045197.1	+	973	1072	**RNA**→
env-982	OLGO01029340.1	-	1016	914	**RNA**→ NorM (COG0534)vmrA (PRK09575)→
env-983	Ga0134492_1023558	+	1024	1126	**RNA**→ NorM (COG0534)Polysacc_synt_C (pfam14667)→
env-984	UnmappedStool_Broad_scaffold_379674	-	1265	1163	**RNA**→ NorM (COG0534)Polysacc_synt_C (pfam14667)→
env-985	OIYV01088865.1	-	180	78	**RNA**→ hypo→
env-986	SRS015217_C2162204	-	107	5	**RNA**→
Cba-2-1	NZ_FCNS01000038.1	+	87135	87231	**RNA**→ NimA (COG3467)Pyridox_ox_2 (pfam12900)→ vmrA (PRK09575)MATE_MepA_like (cd13143)→ hypo→
					hypo→ hypo→
env-987	Ga0134523_1276460	+	22	114	**RNA**→ PRK11431 (PRK11431)EmrE (COG2076)→
env-988	OJAI01047532.1	+	390	482	**RNA**→
env-989	Ga0134492_1095529	+	133	232	**RNA**→ vmrA (PRK09575)MATE_MepA_like (cd13143)→
env-990	Ga0224483_117344	-	357	258	**RNA**→ hypo→
env-991	Ga0172378_10029848	-	3961	3862	**RNA**→ MATE_MepA_like (cd13143)Polysacc_synt_C (pfam14667)→
env-992	Ga0172381_10186312	+	565	664	**RNA**→ NorM (COG0534)Polysacc_synt_C (pfam14667)→
env-993	Ga0172377_10593672	-	479	380	**RNA**→ vmrA (PRK09575)MATE_MepA_like (cd13143)→
env-994	NLM015_scaffold1162_5_2	+	441	540	**RNA**→ NorM (COG0534)vmrA (PRK09575)→
env-995	Ga0209694_1005629	-	3410	3311	**RNA**→ vmrA (PRK09575)MATE_MepA_like (cd13143)→ hypo→
env-996	Ga0209064_1000039	-	86575	86476	**RNA**→ vmrA (PRK09575)MATE_MepA_like (cd13143)→ hypo→
env-997	Ga0177923_1303202	+	734	833	**RNA**→ vmrA (PRK09575)MATE_MepA_like (cd13143)→
env-998	UnmappedStool_Broad_C253083550	-	369	271	**RNA**→ vmrA (PRK09575)MATE_MepA_like (cd13143)→
env-999	Ga0209694_1071632	-	326	228	**RNA**→ PRK10860 (PRK10860)TadA (COG0590)→
env-1000	Ga0209064_1071460	-	389	291	**RNA**→ PRK10860 (PRK10860)TadA (COG0590)→
env-1001	UnmappedStool_Broad_scaffold_436751	-	430	332	**RNA**→ vmrA (PRK09575)MATE_MepA_like (cd13143)→
env-1002	OIZU01007973.1	+	1703	1801	**RNA**→ NorM (COG0534)Polysacc_synt_C (pfam14667)→
env-1003	OIZW01020408.1	-	1031	933	**RNA**→ NorM (COG0534)Polysacc_synt_C (pfam14667)→
env-1004	OLGO01062501.1	+	368	466	**RNA**→ vmrA (PRK09575)MATE_MepA_like (cd13143)→
env-1005	PPYE01000123.1	-	39772	39674	**RNA**→ vmrA (PRK09575)MATE_MepA_like (cd13143)→ NimA (COG3467)Pyridox_ox_2 (pfam12900)→
env-1006	PPYF01035464.1	-	408	310	**RNA**→ vmrA (PRK09575)MATE_MepA_like (cd13143)→
env-1007	Pasolli2019-4166-37	+	18173	18271	**RNA**→ MATE_MepA_like (cd13143)matE (TIGR00797)→ NimA (COG3467)Pyridox_ox_2 (pfam12900)→
env-1008	SRS014923_WUGC_scaffold_33268	-	2329	2231	**RNA**→ vmrA (PRK09575)MATE_MepA_like (cd13143)→ NimA (COG3467)Pyridox_ox_2 (pfam12900)→
env-1009	SRS015217_C2391460	+	14489	14587	**RNA**→ NorM (COG0534)Polysacc_synt_C (pfam14667)→
env-1010	SRS023914_C2091319	+	812	910	**RNA**→ vmrA (PRK09575)MATE_MepA_like (cd13143)→
env-1011	SRS053335_C2512548	-	1858	1760	**RNA**→ vmrA (PRK09575)MATE_MepA_like (cd13143)→ NimA (COG3467)Pyridox_ox_2 (pfam12900)→
Mpe-1-1	NZ_CP020991.1	-	1054447	1054349	**RNA**→ vmrA (PRK09575)MATE_MepA_like (cd13143)→ NimA (COG3467)Pyridox_ox_2 (pfam12900)→
env-1012	UnmappedStool_Broad_scaffold_338461	+	843	943	**RNA**→ vmrA (PRK09575)MATE_MepA_like (cd13143)→
env-1013	Pasolli2019-3995-1	+	21198	21298	**RNA**→ NorM (COG0534)matE (TIGR00797)→
env-1014	UMGS452-8	+	25567	25667	**RNA**→ NorM (COG0534)matE (TIGR00797)→
env-1015	DPWY01000155.1	+	10510	10610	**RNA**→ vmrA (PRK09575)MATE_MepA_like (cd13143)→

env-1016	OIXH01000776.1	-	24132	24034	**RNA**→ vmrA (PRK09575)MATE_MepA_like (cd13143)→
env-1017	DLM022_scaffold69859_1	+	195	294	**RNA**→ PRK11440 (PRK11440)cysteine_hydrolases (cd00431)→ PRK10860 (PRK10860)TadA (COG0590)→
env-1018	OIXV01000003.1	+	151946	152045	**RNA**→ PRK11440 (PRK11440)cysteine_hydrolases (cd00431)→ PRK10860 (PRK10860)TadA (COG0590)→
env-1019	OIZW01000180.1	+	63600	63699	**RNA**→ PRK11440 (PRK11440)cysteine_hydrolases (cd00431)→ PRK10860 (PRK10860)TadA (COG0590)→
env-1020	Pasolli2019-14924-9	-	3667	3568	**RNA**→ cysteine_hydrolases (cd00431)PLN02621 (PLN02621)→ TadA (COG0590)MafB19-deam (pfam14437)→
env-1021	UMGS777-0	+	151946	152045	**RNA**→ cysteine_hydrolases (cd00431)PLN02621 (PLN02621)→ TadA (COG0590)MafB19-deam (pfam14437)→
Lba-2-1	NZ_KE159591.1	+	80566	80666	**RNA**→ vmrA (PRK09575)MATE_MepA_like (cd13143)→ NimA (COG3467)Pyridox_ox_2 (pfam12900)→
env-1022	Ga0172377_11505145	+	87	187	**RNA**→ Pyridox_oxidase (pfam01243)Pyridox_ox_2 (pfam12900)→
env-1023	Ga0129310_1034279	-	858	758	**RNA**→ ←HTH_ARSR (smart00418)HxlR (COG1733)
env-1024	SRS019397_C1467449	+	3372	3472	**RNA**→
env-1025	DOM016_scaffold22411_10	+	779	879	**RNA**→ Pyridox_oxidase (pfam01243)Pyridox_ox_2 (pfam12900)→
env-1026	NLM007_scaffold17503_1	-	3216	3116	**RNA**→ Pyridox_oxidase (pfam01243)Pyridox_ox_2 (pfam12900)→
env-1027	NOM002_scaffold3102_3	+	50629	50729	**RNA**→ Pyridox_oxidase (pfam01243)Pyridox_ox_2 (pfam12900)→
env-1028	OGDP01002140.1	-	8819	8719	**RNA**→ Pyridox_oxidase (pfam01243)Pyridox_ox_2 (pfam12900)→
env-1029	OGGB01000078.1	-	26603	26503	**RNA**→ Pyridox_oxidase (pfam01243)Pyridox_ox_2 (pfam12900)→
env-1030	OGHI01000094.1	-	12520	12420	**RNA**→ Pyridox_oxidase (pfam01243)Pyridox_ox_2 (pfam12900)→
env-1031	OGIZ01000265.1	+	21577	21677	**RNA**→ Pyridox_oxidase (pfam01243)Pyridox_ox_2 (pfam12900)→
env-1032	OGKB01000052.1	-	40401	40301	**RNA**→ Pyridox_oxidase (pfam01243)Pyridox_ox_2 (pfam12900)→
env-1033	OGKF01001281.1	-	3186	3086	**RNA**→ Pyridox_oxidase (pfam01243)Pyridox_ox_2 (pfam12900)→
env-1034	OGMW01000282.1	+	51606	51706	**RNA**→ Pyridox_oxidase (pfam01243)Pyridox_ox_2 (pfam12900)→
env-1035	OGUQ01000147.1	+	54021	54121	**RNA**→ Pyridox_oxidase (pfam01243)Pyridox_ox_2 (pfam12900)→
env-1036	OIWK01006112.1	+	1687	1787	**RNA**→ Pyridox_oxidase (pfam01243)Pyridox_ox_2 (pfam12900)→
env-1037	Ga0134403_104131	+	968	1068	**RNA**→ Pyridox_oxidase (pfam01243)Pyridox_ox_2 (pfam12900)→
env-1038	Pasolli2019-4749-1	+	72858	72958	**RNA**→ Pyridox_oxidase (pfam01243)Pyridox_ox_2 (pfam12900)→
env-1039	UMGS125-0	+	72858	72958	**RNA**→ Pyridox_oxidase (pfam01243)Pyridox_ox_2 (pfam12900)→
env-1040	OGEB01007496.1	-	149	49	**RNA**→
env-1041	OGEG01001623.1	+	14222	14322	**RNA**→
env-1042	OGJY01000523.1	+	54155	54255	**RNA**→
env-1043	OGMN01030342.1	-	149	49	**RNA**→
env-1044	OLGL01000518.1	-	149	49	**RNA**→
env-1045	OGIU01003015.1	-	146	46	**RNA**→
env-1046	OJAC01024415.1	+	7	107	**RNA**→ Pyridox_oxidase (pfam01243)Pyridox_ox_2 (pfam12900)→
env-1047	Ga0134450_108417	-	116	16	**RNA**→
env-1048	Ga0134408_106208	+	601	701	**RNA**→ ←PRK12472 (PRK12472)YkuD (pfam03734)PG_binding_4 (pfam12229)
env-1049	NOM014_scaffold16477_3	-	66744	66644	**RNA**→ Pyridox_oxidase (pfam01243)Pyridox_ox_2 (pfam12900)→
env-1050	NOM027_scaffold2601_1	+	34251	34351	**RNA**→ Pyridox_oxidase (pfam01243)Pyridox_ox_2 (pfam12900)→
env-1051	OGIQ01017670.1	+	552	652	**RNA**→ Pyridox_oxidase (pfam01243)Pyridox_ox_2 (pfam12900)→
env-1052	OGDY01024116.1	+	88	188	**RNA**→ FmnP (COG3601)ECF_trnsprt (pfam12822)→
env-1053	OGEU01019926.1	+	1258	1358	**RNA**→ ←PRK14072 (PRK14072)
env-1054	OIWT01053224.1	-	101	1	**RNA**→
env-1055	OLFS01006968.1	+	3394	3494	**RNA**→
env-1056	Ga0169781_100268	+	30915	31015	**RNA**→ Pyridox_oxidase (pfam01243)Pyridox_ox_2 (pfam12900)→
env-1057	Ga0116646_1044511	-	487	387	**RNA**→ PnuC (COG3201)→
env-1058	Ga0129310_1000588	+	8729	8829	**RNA**→ PnuC (COG3201)NMN_trans_PnuC (TIGR01528)→
env-1059	Pasolli2019-5098-25	+	2962	3062	**RNA**→ PnuC (COG3201)NMN_trans_PnuC (TIGR01528)→
env-1060	UMGS615-27	-	24875	24775	**RNA**→ Pyridox_oxidase (pfam01243)Pyridox_ox_2 (pfam12900)→ PnuC (COG3201)NMN_trans_PnuC (TIGR01528)→
env-1061	SRS020233_C4158156	-	853	753	**RNA**→ PnuC (COG3201)NMN_trans_PnuC (TIGR01528)→
env-1062	4491413.3_NODE_399	+	2163	2263	**RNA**→ PnuC (COG3201)NMN_trans_PnuC (TIGR01528)→ Pyridox_oxidase (pfam01243)Pyridox_ox_2 (pfam12900)→
env-1063	4491487.3_NODE_82858	-	754	654	**RNA**→ PnuC (COG3201)NMN_trans_PnuC (TIGR01528)→
env-1064	MNRC01000347.1	-	7014	6914	**RNA**→ PnuC (COG3201)NMN_trans_PnuC (TIGR01528)→ Pyridox_oxidase (pfam01243)Pyridox_ox_2 (pfam12900)→

env-1065	OLGH01001957.1	+	11323	11423	RNA→ PnuC (COG3201)NMN_trans_PnuC (TIGR01528)→<br>Pyridox_oxidase (pfam01243)Pyridox_ox_2 (pfam12900)→
env-1066	PPYE01046567.1	-	27565	27465	RNA→ PnuC (COG3201)NMN_trans_PnuC (TIGR01528)→<br>Pyridox_oxidase (pfam01243)Pyridox_ox_2 (pfam12900)→
env-1067	Pasolli2019-4922-7	+	5571	5671	RNA→ PnuC (COG3201)NMN_trans_PnuC (TIGR01528)→<br>Pyridox_oxidase (pfam01243)Pyridox_ox_2 (pfam12900)→
env-1068	scaffold57226_2_MH0045	+	169	269	RNA→ PnuC (COG3201)NMN_trans_PnuC (TIGR01528)→
env-1069	scaffold87392_3_MH0077	+	2879	2979	RNA→ PnuC (COG3201)→
env-1070	scaffold11558_4_MH0055	+	270	370	RNA→ PnuC (COG3201)NMN_trans_PnuC (TIGR01528)→
env-1071	Ga0117811_1066668	+	232	331	RNA→ PnuC (COG3201)NMN_trans_PnuC (TIGR01528)→
env-1072	Ga0116620_1034458	+	299	399	RNA→ hypo→
env-1073	OGHK01039176.1	-	175	76	RNA→
env-1074	OLFU01066859.1	-	453	354	RNA→
env-1075	UnmappedStool_Broad_scaffold_740423	+	50	149	RNA→ hypo→
env-1076	DOF008_scaffold28034_2	-	390	291	RNA→
env-1077	OLGF01003227.1	+	1461	1560	RNA→ PnuC (COG3201)NMN_trans_PnuC (TIGR01528)→
env-1078	SRS015133_C3315683	-	327	228	RNA→ hypo→
env-1079	SRS058723_C6777383	-	1391	1292	RNA→ Pyridox_oxidase (pfam01243)Pyridox_ox_2 (pfam12900)→<br>PnuC (COG3201)NMN_trans_PnuC (TIGR01528)→
env-1080	Pasolli2019-14184-32	-	7114	7015	RNA→ cysteine_hydrolases (cd00431)PLN02621 (PLN02621)→
env-1081	UnmappedStool_Broad_scaffold_1013163	-	364	265	RNA→ PLN02621 (PLN02621)Isochorismatase (pfam00857)→
env-1082	Ga0121064_100008	-	221600	221499	RNA→ vmrA (PRK09575)MATE_MepA_like (cd13143)→
env-1083	Ga0194137_10092412	-	1361	1260	RNA→ vmrA (PRK09575)MATE_MepA_like (cd13143)→
env-1084	Ga0247728_10007	+	294989	295090	RNA→ vmrA (PRK09575)MATE_MepA_like (cd13143)→
Ano-1-1	NZ_FUYN01000002.1	+	351755	351856	RNA→ vmrA (PRK09575)MATE_MepA_like (cd13143)→
env-1085	2211336903	-	108	7	RNA→
env-1086	Ga0075011_10184129	-	720	619	RNA→ NorM (COG0534)vmrA (PRK09575)→
env-1087	Ga0122069_100878	+	8430	8531	RNA→ vmrA (PRK09575)MATE_MepA_like (cd13143)→
env-1088	Ga0194136_1022386	-	2409	2308	RNA→ vmrA (PRK09575)MATE_MepA_like (cd13143)→ COG2865 (COG2865)→<br>COG2865 (COG2865)HATPase_c_4 (pfam13749)→
Cst-1-1	NC_014614.1	+	337374	337475	RNA→ vmrA (PRK09575)MATE_MepA_like (cd13143)→<br>W_rSAM_matur (TIGR04317)Radical_SAM (pfam04055)→ B3_4 (smart00873)B3/B4 (COG3382)→
env-1089	DCFN01000004.1	+	8739	8840	RNA→ vmrA (PRK09575)MATE_MepA_like (cd13143)→
env-1090	longitudinal_460_95	-	113409	113309	RNA→ vmrA (PRK09575)MATE_MepA_like (cd13143)→
env-1091	Pasolli2019-4799-127	+	8065	8162	RNA→ NorM (COG0534)matE (TIGR00797)→
env-1092	Pasolli2019-4718-38	-	15068	14969	RNA→ MATE_MepA_like (cd13143)matE (TIGR00797)→ cysteine_hydrolases (cd00431)PTZ00331 (PTZ00331)-
env-1093	UMGS341-39	-	15038	14939	RNA→ MATE_MepA_like (cd13143)matE (TIGR00797)→ cysteine_hydrolases (cd00431)PTZ00331 (PTZ00331)-
env-1094	DKYD01000086.1	-	2386	2287	RNA→ vmrA (PRK09575)MATE_MepA_like (cd13143)→ SLH (pfam00395)→
env-1095	Ga0129317_1014815	-	935	836	RNA→ NorM (COG0534)Polysacc_synt_C (pfam14667)→
env-1096	Ga0177923_1060928	-	5742	5642	RNA→ vmrA (PRK09575)MATE_MepA_like (cd13143)→
env-1097	Ga0209511_1244286	-	555	455	RNA→ vmrA (PRK09575)MATE_MepA_like (cd13143)→
Csp-17-1	NZ_FMMP01000011.1	-	1689	1589	RNA→ vmrA (PRK09575)MATE_MepA_like (cd13143)→
env-1098	MA40A_GDRVJ9S01AKVRT_left	+	26	126	RNA→ hypo→
env-1099	Pasolli2019-4638-0	-	32946	32847	RNA→ NorM (COG0534)matE (TIGR00797)→
env-1100	Ga0129308_1000404	+	62120	62219	RNA→ vmrA (PRK09575)MATE_MepA_like (cd13143)→ hypo→
env-1101	SRS014923_C3381573	-	8447	8348	RNA→ vmrA (PRK09575)MATE_MepA_like (cd13143)→
env-1102	Ga0177923_1013787	+	82425	82524	RNA→ vmrA (PRK09575)MATE_MepA_like (cd13143)→
env-1103	Ga0134538_1028462	-	755	656	RNA→ NorM (COG0534)vmrA (PRK09575)→
env-1104	Pasolli2019-5103-0	+	436816	436915	RNA→ MATE_MepA_like (cd13143)matE (TIGR00797)→ Asparaginase (pfam00710)Asparaginase (smart00870)-
env-1105	UMGS725-72	-	8825	8726	RNA→ MATE_MepA_like (cd13143)matE (TIGR00797)→ Asparaginase (pfam00710)Asparaginase (smart00870)-
env-1106	UnmappedStool_Broad_scaffold_65061	-	2573	2474	RNA→ vmrA (PRK09575)MATE_MepA_like (cd13143)→
env-1107	DKVI01000022.1	-	4167	4068	RNA→ vmrA (PRK09575)MATE_like (cd12082)→ PLN02621 (PLN02621)cysteine_hydrolases (cd00431)→ hypo-
env-1108	DKTY01000021.1	-	3259	3160	RNA→ vmrA (PRK09575)MATE_like (cd12082)→ PLN02621 (PLN02621)cysteine_hydrolases (cd00431)→ hypo-
env-1109	Pasolli2019-4729-14	-	3517	3417	RNA→ MATE_MepA_like (cd13143)Polysacc_synt_C (pfam14667)→

Lsp-6-1	NZ_NFLE01000016.1	-	18116	18016	**RNA**→ MATE_MepA_like (cd13143)Polysacc_synt_C (pfam14667)→
env-1110	Pasolli2019-4976-196	+	478	577	**RNA**→ MATE_MepA_like (cd13143)matE (TIGR00797)→
					BglX (COG1472)DUF1049 (pfam06305)PRK05337 (PRK05337)→
env-1111	UMGS1777-195	-	2877	2778	**RNA**→ MATE_MepA_like (cd13143)matE (TIGR00797)→
					BglX (COG1472)DUF1049 (pfam06305)PRK05337 (PRK05337)→
env-1112	Ga0121337_108427	-	206	106	**RNA**→ hypo→
Lph-1-1	NZ_JPOF01000003.1	-	784575	784475	**RNA**→ vmrA (PRK09575)MATE_MepA_like (cd13143)→
Lph-3-1	NZ_JNLG01000001.1	+	1984812	1984912	**RNA**→ vmrA (PRK09575)MATE_MepA_like (cd13143)→
Lph-2-1	NZ_JNLM01000001.1	+	2445686	2445786	**RNA**→ vmrA (PRK09575)MATE_MepA_like (cd13143)→
Cph-1-1	NC_010001.1	-	4248492	4248392	**RNA**→ vmrA (PRK09575)MATE_MepA_like (cd13143)→
env-1113	Ga0160506_101733	+	1191	1291	**RNA**→ vmrA (PRK09575)MATE_MepA_like (cd13143)→
Csp-13-1	NZ_LT732526.1	+	1670422	1670521	**RNA**→ vmrA (PRK09575)MATE_MepA_like (cd13143)→
Lba-1-1	NZ_PEDL01000021.1	-	20112	20012	**RNA**→ NorM (COG0534)vmrA (PRK09575)→
env-1114	Ga0169833_105330	-	1725	1626	**RNA**→ vmrA (PRK09575)MATE_MepA_like (cd13143)→
env-1115	DLWH01000368.1	+	3847	3946	**RNA**→ vmrA (PRK09575)MATE_MepA_like (cd13143)→
env-1116	Ga0177923_1263515	-	446	347	**RNA**→ vmrA (PRK09575)MATE_MepA_like (cd13143)→
env-1117	Ga0134540_1000036	-	47760	47660	**RNA**→ vmrA (PRK09575)MATE_MepA_like (cd13143)→
Mfo-1-1	NZ_FNBY01000011.1	+	5750	5849	**RNA**→ vmrA (PRK09575)MATE_MepA_like (cd13143)→ cysteine_hydrolases (cd00431)PTZ00331 (PTZ00331)→
Bfo-1-1	NZ_ACCL02000009.1	-	136506	136407	**RNA**→ hypo→ hypo→ vmrA (PRK09575)MATE_MepA_like (cd13143)→
					vmrA (PRK09575)MATE_MepA_like (cd13143)→ cysteine_hydrolases (cd00431)PTZ00331 (PTZ00331)→
					cysteine_hydrolases (cd00431)PTZ00331 (PTZ00331)→
env-1118	Ga0134538_1014872	-	2322	2223	**RNA**→ vmrA (PRK09575)MATE_MepA_like (cd13143)→ cysteine_hydrolases (cd00431)PTZ00331 (PTZ00331)→
					Trans_reg_C (smart00862)OmpR (COG0745)→
Pma-1-1	NZ_FWCH01000012.1	-	1990162	1990061	**RNA**→ NorM (COG0534)vmrA (PRK09575)→ Radical_SAM (cd01335)W_rSAM_matur (TIGR04317)→
env-1119	Ga0134459_144815	-	348	251	**RNA**→ hypo→
env-1120	Pasolli2019-4701-30	-	29248	29156	**RNA**→ MATE_MepA_like (cd13143)matE (TIGR00797)→
env-1121	Pasolli2019-14897-45	-	420	320	**RNA**→ MATE_MepA_like (cd13143)vmrA (PRK09575)→
env-1122	DGRE01000041.1	-	31412	31313	**RNA**→ vmrA (PRK09575)MATE_MepA_like (cd13143)→ RhaT (COG0697)EmrE (pfam13536)→
					COG1878 (COG1878)→
env-1123	SRS049959_C3774603	+	102	195	**RNA**→ PnuC (COG3201)→
env-1124	UMGS421-13	-	29407	29316	**RNA**→ hypo→
env-1125	Ga0213824_1071480	+	82	186	**RNA**→ vmrA (PRK09575)MATE_MepA_like (cd13143)→
env-1126	Ga0213829_1173523	-	180	76	**RNA**→
Lsp-8-1	NZ_NFKJ01000011.1	-	89275	89178	**RNA**→ vmrA (PRK09575)MATE_MepA_like (cd13143)→
env-1127	Pasolli2019-4620-174	+	492	589	**RNA**→ MATE_MepA_like (cd13143)matE (TIGR00797)→
Dsp-3-1	NZ_NFHY01000023.1	-	26077	25983	**RNA**→ vmrA (PRK09575)MATE_MepA_like (cd13143)→
Dsp-2-1	NZ_NFLO01000001.1	+	18864	18958	**RNA**→ vmrA (PRK09575)MATE_MepA_like (cd13143)→
env-1128	Ga0129308_1038224	+	538	642	**RNA**→ vmrA (PRK09575)MATE_MepA_like (cd13143)→
env-1129	UnmappedStool_Broad_scaffold_1023733	-	524	430	**RNA**→ vmrA (PRK09575)MATE_MepA_like (cd13143)→
Lco-1-1	NZ_JYDC01000046.1	+	77264	77356	**RNA**→ PRK11431 (PRK11431)EmrE (COG2076)→
Lco-2-1	NZ_AYYR01000067.1	+	77267	77359	**RNA**→ PRK11431 (PRK11431)EmrE (COG2076)→
env-1130	DMTF01000042.1	+	4154	4259	**RNA**→ PnuC (COG3201)NMN_trans_PnuC (TIGR01528)→
env-1131	Pasolli2019-4410-0	-	247845	247742	**RNA**→ PnuC (COG3201)NMN_trans_PnuC (TIGR01528)→
					SGNH_hydrolase (cd00229)Lipase_GDSL_2 (pfam13472)→
env-1132	Ga0172381_12431913	-	101	8	**RNA**→
env-1133	Ga0172377_10751836	+	405	499	**RNA**→ PnuC (COG3201)→
env-1134	OGHL01031433.1	-	398	297	**RNA**→ NMN_transporter (pfam04973)→
env-1135	3300000568_10029641	+	290	392	**RNA**→ PnuC (COG3201)→
env-1136	OGFK01029779.1	+	632	735	**RNA**→ hypo→
Ssp-1-1	NZ_LFYO02000002.1	-	494877	494776	**RNA**→ AAA (smart00382)LolD (COG1136)→ hypo→ hypo→ hypo→
env-1137	OIZA01062072.1	-	492	389	**RNA**→ PnuC (COG3201)→
env-1138	Pasolli2019-14251-85	+	160	263	**RNA**→ PnuC (COG3201)NMN_trans_PnuC (TIGR01528)→
env-1139	Pasolli2019-14236-10	+	2779	2888	**RNA**→ PnuC (COG3201)NMN_trans_PnuC (TIGR01528)→
					NMN_transporter (pfam04973)NMN_trans_PnuC (TIGR01528)→

env-1140	Ga0129306_1000118	+	43216	43314	**RNA**→ RimI (COG0456)Acetyltransf_7 (pfam13508)→
env-1141	Ga0129307_1000074	-	121443	121345	**RNA**→ RimI (COG0456)Acetyltransf_7 (pfam13508)→
env-1142	AUXO017690829.1	+	70	171	**RNA**→ PnuC (COG3201)→
env-1143	OGDS01006126.1	+	1808	1909	**RNA**→ PnuC (COG3201)NMN_trans_PnuC (TIGR01528)→
env-1144	OGEL01006126.1	+	1808	1909	**RNA**→ PnuC (COG3201)NMN_trans_PnuC (TIGR01528)→
env-1145	OIYG01021392.1	+	313	414	**RNA**→ PnuC (COG3201)NMN_trans_PnuC (TIGR01528)→
env-1146	UMGS172-32	+	16137	16238	**RNA**→ PnuC (COG3201)NMN_trans_PnuC (TIGR01528)→
env-1147	Pasolli2019-4406-30	-	7412	7311	**RNA**→ PnuC (COG3201)NMN_trans_PnuC (TIGR01528)→
env-1148	Pasolli2019-14936-41	-	18426	18322	**RNA**→ PnuC (COG3201)NMN_trans_PnuC (TIGR01528)→
env-1149	UMGS1397-5	+	27410	27514	**RNA**→ PnuC (COG3201)NMN_trans_PnuC (TIGR01528)→
env-1150	Pasolli2019-4144-24	-	19004	18915	**RNA**→ PnuC (COG3201)NMN_trans_PnuC (TIGR01528)→
env-1151	JGI20225J20221_1000026	+	74625	74727	**RNA**→ PRK11431 (PRK11431)EmrE (COG2076)→ PRK05451 (PRK05451)→ GGDEF (smart00267)GGDEF (cd01949)→
env-1152	JGI20225J20221_1000198	-	73566	73464	**RNA**→ PRK11431 (PRK11431)EmrE (COG2076)→ PRK05451 (PRK05451)→ GGDEF (smart00267)GGDEF (cd01949)→
env-1153	Ga0172377_10255378	+	267	369	**RNA**→ PnuC (COG3201)NMN_trans_PnuC (TIGR01528)→
env-1154	Ga0209407_1000071	-	78588	78486	**RNA**→ PnuC (COG3201)NMN_trans_PnuC (TIGR01528)→ septicolysin_like (cd12208)→
env-1155	Ga0208694_1324696	+	73	175	**RNA**→ NMN_transporter (pfam04973)→
Rfl-1-1	NZ_FPJT01000009.1	+	65708	65810	**RNA**→ PnuC (COG3201)NMN_trans_PnuC (TIGR01528)→ septicolysin_like (cd12208)→
env-1156	AUXO015907345.1	-	1633	1531	**RNA**→ PnuC (COG3201)NMN_trans_PnuC (TIGR01528)→ septicolysin_like (cd12208)→
env-1157	DGTN01000018.1	-	141614	141512	**RNA**→ PnuC (COG3201)NMN_trans_PnuC (TIGR01528)→ septicolysin_like (cd12208)→
env-1158	Ga0209604_1799201	+	48	150	**RNA**→ hypo→
env-1159	Ga0172381_12108401	-	291	188	**RNA**→ hypo→
env-1160	Ga0172377_11826832	+	201	304	**RNA**→ hypo→
env-1161	DJRT01000011.1	+	33683	33785	**RNA**→ PnuC (COG3201)NMN_trans_PnuC (TIGR01528)→
env-1162	2013407966	+	1295	1398	**RNA**→ hypo→
env-1163	KGI_S1_ANT01_95m_c10000035	-	38656	38554	**RNA**→ PnuC (COG3201)NMN_trans_PnuC (TIGR01528)→
env-1164	Ga0256405_10000612	-	55209	55103	**RNA**→ ←Nth (COG0177)DEDD_Tnp_IS110 (pfam01548)Transposase_20 (pfam02371)
env-1165	ONMD01000050.1	+	4152	4256	**RNA**→ PnuC (COG3201)NMN_trans_PnuC (TIGR01528)→
env-1166	OMSI01000057.1	+	489	593	**RNA**→ PnuC (COG3201)NMN_trans_PnuC (TIGR01528)→
env-1167	ONBS01000038.1	-	10849	10745	**RNA**→ PnuC (COG3201)NMN_trans_PnuC (TIGR01528)→
env-1168	ONDY01000052.1	+	12940	13044	**RNA**→ PnuC (COG3201)NMN_trans_PnuC (TIGR01528)→
env-1169	ONFT01000040.1	+	8200	8304	**RNA**→ PnuC (COG3201)NMN_trans_PnuC (TIGR01528)→
env-1170	ONGD01000025.1	-	15101	14997	**RNA**→ PnuC (COG3201)NMN_trans_PnuC (TIGR01528)→
env-1171	ONGI01000070.1	-	4738	4634	**RNA**→ PnuC (COG3201)NMN_trans_PnuC (TIGR01528)→
env-1172	ONIS01000048.1	+	2199	2303	**RNA**→ PnuC (COG3201)NMN_trans_PnuC (TIGR01528)→
env-1173	ONJW01000024.1	+	4638	4742	**RNA**→ PnuC (COG3201)NMN_trans_PnuC (TIGR01528)→
env-1174	ONKR01000088.1	+	4654	4758	**RNA**→ PnuC (COG3201)NMN_trans_PnuC (TIGR01528)→
env-1175	ONNM01000038.1	+	4533	4637	**RNA**→ PnuC (COG3201)NMN_trans_PnuC (TIGR01528)→
env-1176	ONPP01000112.1	+	4196	4300	**RNA**→ PnuC (COG3201)NMN_trans_PnuC (TIGR01528)→
env-1177	ONQG01000046.1	+	2668	2772	**RNA**→ PnuC (COG3201)NMN_trans_PnuC (TIGR01528)→
env-1178	ONUM01000114.1	+	185	289	**RNA**→ PnuC (COG3201)NMN_trans_PnuC (TIGR01528)→
env-1179	ONVF01000115.1	+	3471	3575	**RNA**→ PnuC (COG3201)NMN_trans_PnuC (TIGR01528)→
env-1180	ONDX01000020.1	+	12762	12866	**RNA**→
env-1181	OLFT01003292.1	+	6648	6747	**RNA**→ PnuC (COG3201)NMN_trans_PnuC (TIGR01528)→
env-1182	SRS014235_WUGC_scaffold_109454	+	10985	11084	**RNA**→ vmrA (PRK09575)MATE_MepA_like (cd13143)→
env-1183	OIYO01000045.1	-	32898	32799	**RNA**→ vmrA (PRK09575)MATE_MepA_like (cd13143)→ MET17 (COG2873)OAH_OAS_sulfhy (TIGR01326)→ Flp_Fap (pfam04964)→
env-1184	OIZV01039874.1	-	552	453	**RNA**→ vmrA (PRK09575)MATE_MepA_like (cd13143)→
env-1185	OLGM01002826.1	+	1866	1965	**RNA**→ vmrA (PRK09575)MATE_MepA_like (cd13143)→ MET17 (COG2873)OAH_OAS_sulfhy (TIGR01326)→ Flp_Fap (pfam04964)→
env-1186	OLHG01002343.1	-	841	742	**RNA**→ NorM (COG0534)vmrA (PRK09575)→
env-1187	UMGS1050-48	-	841	742	**RNA**→ NorM (COG0534)matE (TIGR00797)→

env-1188	Pasolli2019-4325-7	+	17683	17782	RNA→ MATE_MepA_like (cd13143)matE (TIGR00797)→ MET17 (COG2873)OAH_OAS_sulfhy (TIGR01326)→
					Flp_Fap (pfam04964)→
env-1189	UnmappedStool_Broad_scaffold_954874	-	699	599	RNA→ PnuC (COG3201)NMN_trans_PnuC (TIGR01528)→
env-1190	DCTO01000043.1	+	27225	27332	RNA→ PnuC (COG3201)NMN_trans_PnuC (TIGR01528)→
					LbH_MAT_GAT (cd03357)Mac (pfam12464)Hexapep_2 (pfam14602)→ EstA (COG1075)→ Ferritin_like (cd00657)
env-1191	DLKT01000044.1	+	15284	15385	RNA→ PnuC (COG3201)NMN_trans_PnuC (TIGR01528)→
env-1192	DJQI01000055.1	+	7609	7710	RNA→ PnuC (COG3201)NMN_trans_PnuC (TIGR01528)→
env-1193	Ga0116620_1069964	+	214	315	RNA→
env-1194	UnmappedStool_Broad_C252789452	-	530	430	RNA→ PRK11431 (PRK11431)EmrE (COG2076)→
env-1195	Pasolli2019-3991-6	+	68526	68626	RNA→ EmrE (COG2076)PRK11431 (PRK11431)→
env-1196	UMGS192-17	-	24668	24572	RNA→ EmrE (COG2076)PRK11431 (PRK11431)→
Cbu-1-1	NZ_CP013239.1	-	2452018	2451917	RNA→ COG3603 (COG3603)ACT_7 (pfam13840)→
Csp-9-1	NZ_KV823376.1	-	63063	62962	RNA→ COG3603 (COG3603)ACT_7 (pfam13840)→
					FldA (COG0716)NapF (COG1145)Flavodoxin_5 (pfam12724)Fer4_7 (pfam12838)→ hypo→
					SRPBCC (cd07812)Polyketide_cyc2 (pfam10604)→ nudC (PRK00241)Nudix_Hydrolase_29 (cd04688)→
					ABC_ATPase (cd00267)AAA_21 (pfam13304)AAA_23 (pfam13476)→
Cbu-2-1	NZ_KB851128.1	-	1459586	1459485	RNA→ COG3603 (COG3603)ACT_7 (pfam13840)→
					FldA (COG0716)NapF (COG1145)Flavodoxin_5 (pfam12724)Fer4_7 (pfam12838)→ hypo→ hypo→
					SRPBCC (cd07812)Polyketide_cyc2 (pfam10604)→ nudC (PRK00241)Nudix_Hydrolase_29 (cd04688)→
					ABC_ATPase (cd00267)AAA_21 (pfam13304)AAA_23 (pfam13476)→
env-1197	OGIT01000149.1	+	17733	17834	RNA→ COG3603 (COG3603)ACT_7 (pfam13840)→
					NapF (COG1145)HemG (COG4635)Flavodoxin_5 (pfam12724)Fer4_7 (pfam12838)→ hypo→
					SRPBCC (cd07812)Polyketide_cyc2 (pfam10604)→ nudC (PRK00241)Nudix_Hydrolase_29 (cd04688)→
					ABC_ATPase (cd00267)AAA_21 (pfam13304)AAA_23 (pfam13476)→
env-1198	Pasolli2019-6170-83	-	7628	7527	RNA→ COG3603 (COG3603)ACT_7 (pfam13840)→
					NapF (COG1145)HemG (COG4635)Fer4_7 (pfam12838)Flavodoxin_5 (pfam12724)→ hypo→
					SRPBCC (cd07812)Polyketide_cyc2 (pfam10604)→ Nudix_Hydrolase_29 (cd04688)nudix_YtkD (TIGR02705)→
					ABC_ATPase (cd00267)AAA_21 (pfam13304)AAA_23 (pfam13476)→
Csp-1-1	NZ_PQCS01000112.1	+	6904	7005	RNA→ COG3603 (COG3603)ACT_7 (pfam13840)→
					FldA (COG0716)NapF (COG1145)Flavodoxin_5 (pfam12724)Fer4_7 (pfam12838)→ hypo→
					SRPBCC (cd07812)Polyketide_cyc2 (pfam10604)→ nudC (PRK00241)Nudix_Hydrolase (cd02883)→
					ABC_ATPase (cd00267)AAA_21 (pfam13304)AAA_23 (pfam13476)→
Cbu-1-2	NZ_CP013352.1	-	2112132	2112031	RNA→ COG3603 (COG3603)ACT_7 (pfam13840)→
					FldA (COG0716)NapF (COG1145)Flavodoxin_5 (pfam12724)Fer4_7 (pfam12838)→ hypo→ hypo→
					nudC (PRK00241)Nudix_Hydrolase_29 (cd04688)→
					ABC_ATPase (cd00267)AAA_21 (pfam13304)AAA_23 (pfam13476)→
Cbu-1-3	NZ_CP014704.1	-	2064679	2064578	RNA→ COG3603 (COG3603)ACT_7 (pfam13840)→
					FldA (COG0716)NapF (COG1145)Flavodoxin_5 (pfam12724)Fer4_7 (pfam12838)→ hypo→ hypo→
					nudC (PRK00241)Nudix_Hydrolase_29 (cd04688)→
					ABC_ATPase (cd00267)AAA_21 (pfam13304)AAA_23 (pfam13476)→
Cbu-1-4	NZ_CP013252.1	-	2392431	2392330	RNA→ COG3603 (COG3603)ACT_7 (pfam13840)→ hypo→ hypo→ hypo→
					nudC (PRK00241)Nudix_Hydrolase_29 (cd04688)→
					ABC_ATPase (cd00267)AAA_21 (pfam13304)AAA_23 (pfam13476)→
Cbu-6-1	NZ_AQQF01000159.1	+	221001	221102	RNA→ COG3603 (COG3603)ACT_7 (pfam13840)→
					NapF (COG1145)HemG (COG4635)Flavodoxin_5 (pfam12724)Fer4_7 (pfam12838)→ hypo→
					SRPBCC (cd07812)Polyketide_cyc2 (pfam10604)→ nudC (PRK00241)Nudix_Hydrolase_29 (cd04688)→
					ABC_ATPase (cd00267)AAA_21 (pfam13304)AAA_23 (pfam13476)→
Cbu-4-1	NZ_ASPQ01000181.1	-	3405	3304	RNA→ COG3603 (COG3603)ACT_7 (pfam13840)→
					FldA (COG0716)NapF (COG1145)Flavodoxin_5 (pfam12724)Fer4_7 (pfam12838)→ hypo→
					SRPBCC (cd07812)Polyketide_cyc2 (pfam10604)→ nudC (PRK00241)Nudix_Hydrolase_29 (cd04688)→
					ABC_ATPase (cd00267)AAA_21 (pfam13304)AAA_23 (pfam13476)→
Cbu-5-1	NZ_APKZ01000022.1	+	83734	83835	RNA→ COG3603 (COG3603)ACT_7 (pfam13840)→
					NapF (COG1145)HemG (COG4635)Flavodoxin_5 (pfam12724)Fer4_7 (pfam12838)→ hypo→
Cbu-3-1	NZ_AUJN01000008.1	+	196216	196317	RNA→ COG3603 (COG3603)ACT_7 (pfam13840)→
env-1199	OIZQ01033937.1	-	903	804	RNA→ PnuC (COG3201)NMN_trans_PnuC (TIGR01528)→
env-1200	Pasolli2019-4196-50	+	15022	15121	RNA→ PnuC (COG3201)NMN_trans_PnuC (TIGR01528)→

env-1201	Pasolli2019-4375-401	+	1058	1157	**RNA**→ PnuC (COG3201)NMN_trans_PnuC (TIGR01528)→
env-1202	scaffold85453_2_MH0031	+	156	255	**RNA**→ PnuC (COG3201)→
env-1203	scaffold89794_1_V1.UC-10	-	854	755	**RNA**→ PnuC (COG3201)NMN_trans_PnuC (TIGR01528)→
env-1204	Ga0129308_1005193	-	4603	4503	**RNA**→ PnuC (COG3201)NMN_trans_PnuC (TIGR01528)→
env-1205	UMGS1815-19	-	9841	9739	**RNA**→ PnuC (COG3201)NMN_trans_PnuC (TIGR01528)→
env-1206	Ga0129309_1000751	+	15529	15631	**RNA**→ PnuC (COG3201)NMN_trans_PnuC (TIGR01528)→
env-1207	Ga0129308_1000033	-	149700	149598	**RNA**→ PnuC (COG3201)NMN_trans_PnuC (TIGR01528)→
env-1208	UMGS1940-3	+	170	272	**RNA**→ PnuC (COG3201)NMN_trans_PnuC (TIGR01528)→
env-1209	OIZX01004739.1	-	4722	4620	**RNA**→ PnuC (COG3201)NMN_trans_PnuC (TIGR01528)→
env-1210	Pasolli2019-4200-1	+	20065	20167	**RNA**→ PnuC (COG3201)NMN_trans_PnuC (TIGR01528)→
env-1211	HCE12Call500_c0161491	-	6135	6034	**RNA**→ PnuC (COG3201)NMN_trans_PnuC (TIGR01528)→ PRK10765 (PRK10765)Nitro_FMN_reductase (cd02062)→
env-1212	HCF14C_7590	-	6116	6015	**RNA**→ PnuC (COG3201)NMN_trans_PnuC (TIGR01528)→ PRK10765 (PRK10765)Nitro_FMN_reductase (cd02062)→
env-1213	HCF12C_265208	-	46448	46347	**RNA**→ PnuC (COG3201)NMN_trans_PnuC (TIGR01528)→ PRK10765 (PRK10765)Nitro_FMN_reductase (cd02062)→
env-1214	AUXO016051809.1	-	104	2	**RNA**→
env-1215	Ga0129313_1000941	+	16090	16195	**RNA**→ PnuC (COG3201)NMN_trans_PnuC (TIGR01528)→ PTZ00331 (PTZ00331)Isochorismatase (pfam00857)
env-1216	Ga0129312_1000002	-	203021	202916	**RNA**→ PnuC (COG3201)NMN_trans_PnuC (TIGR01528)→ PTZ00331 (PTZ00331)Isochorismatase (pfam00857)
env-1217	OIZF01000075.1	-	33659	33554	**RNA**→ Pyridox_oxidase (pfam01243)Pyridox_ox_2 (pfam12900)→
env-1218	OLGY01002262.1	-	7204	7099	**RNA**→ Pyridox_oxidase (pfam01243)Pyridox_ox_2 (pfam12900)→
env-1219	Pasolli2019-4554-129	-	7295	7190	**RNA**→ Pyridox_oxidase (pfam01243)Pyridox_ox_2 (pfam12900)→
env-1220	DLLG01000011.1	+	66870	66974	**RNA**→ PnuC (COG3201)NMN_trans_PnuC (TIGR01528)→
env-1221	Ga0116646_1003486	+	894	997	**RNA**→ PnuC (COG3201)NMN_trans_PnuC (TIGR01528)→
env-1222	DNUB01000281.1	-	1538	1434	**RNA**→ NMN_trans_PnuC (TIGR01528)NMN_transporter (pfam04973)→
env-1223	Ga0172378_10193755	+	655	756	**RNA**→ PnuC (COG3201)NMN_trans_PnuC (TIGR01528)→
env-1224	AUXO012025396.1	+	70	170	**RNA**→
env-1225	DLLB01000021.1	+	5815	5915	**RNA**→ PnuC (COG3201)NMN_trans_PnuC (TIGR01528)→
env-1226	Ga0116616_1014082	-	746	646	**RNA**→ PnuC (COG3201)NMN_trans_PnuC (TIGR01528)→
env-1227	ONDB01000175.1	-	8867	8762	**RNA**→ PnuC (COG3201)NMN_trans_PnuC (TIGR01528)→
env-1228	Pasolli2019-14181-120	-	1355	1254	**RNA**→ PnuC (COG3201)NMN_trans_PnuC (TIGR01528)→
env-1229	Ga0116646_1082803	-	358	257	**RNA**→ NMN_transporter (pfam04973)→
env-1230	Ga0116650_1039659	+	337	438	**RNA**→ hypo→
env-1231	UnmappedStool_Broad_scaffold_391310	-	3182	3081	**RNA**→ PnuC (COG3201)NMN_trans_PnuC (TIGR01528)→ MATE_yoeA_like (cd13138)→
env-1232	UMGS285-9	-	47723	47622	**RNA**→ PnuC (COG3201)NMN_trans_PnuC (TIGR01528)→
env-1233	SRS014613_WUGC_scaffold_32988	+	1242	1343	**RNA**→ PnuC (COG3201)NMN_trans_PnuC (TIGR01528)→ MATE_yoeA_like (cd13138)→
env-1234	SRS015960_WUGC_scaffold_28391	+	1079	1180	**RNA**→ PnuC (COG3201)NMN_trans_PnuC (TIGR01528)→
env-1235	SRS016018_C1294575	-	1533	1432	**RNA**→ PnuC (COG3201)NMN_trans_PnuC (TIGR01528)→
env-1236	SRS023914_C2101915	-	855	754	**RNA**→ PnuC (COG3201)NMN_trans_PnuC (TIGR01528)→
env-1237	SRS018817_C1941820	+	261	362	**RNA**→
env-1238	SRS050925_LANL_scaffold_63332	-	220	119	**RNA**→
env-1239	SRS020233_C3812118	+	47	148	**RNA**→
env-1240	SRS020869_C3429752	-	2392	2291	**RNA**→ PnuC (COG3201)NMN_trans_PnuC (TIGR01528)→
env-1241	SRS056259_LANL_scaffold_55180	-	721	620	**RNA**→ NMN_transporter (pfam04973)→
env-1242	Ga0116620_1000035	+	73214	73315	**RNA**→ NMN_trans_PnuC (TIGR01528)NMN_transporter (pfam04973)→
env-1243	DLLD01000013.1	-	32773	32672	**RNA**→ NMN_trans_PnuC (TIGR01528)NMN_transporter (pfam04973)→
env-1244	Ga0116646_1040897	-	574	475	**RNA**→ PnuC (COG3201)NMN_trans_PnuC (TIGR01528)→
env-1245	Pasolli2019-4400-140	-	3161	3060	**RNA**→ PnuC (COG3201)NMN_trans_PnuC (TIGR01528)→
env-1246	UnmappedStool_Broad_scaffold_483722	+	884	988	**RNA**→ PnuC (COG3201)NMN_trans_PnuC (TIGR01528)→
env-1247	OIZV01001072.1	+	8370	8472	**RNA**→ PnuC (COG3201)NMN_trans_PnuC (TIGR01528)→
env-1248	Ga0172378_12609000	-	210	108	**RNA**→ hypo→
env-1249	Ga0193963_1048239	+	99	201	**RNA**→ PnuC (COG3201)NMN_trans_PnuC (TIGR01528)→
env-1250	Ga0193964_1025987	-	1497	1395	**RNA**→ PnuC (COG3201)NMN_trans_PnuC (TIGR01528)→

env-1251	Ga0193953_1004299	+	217	319	**RNA**→ PnuC (COG3201)NMN_trans_PnuC (TIGR01528)→
env-1252	DHKH01000041.1	-	33563	33457	**RNA**→ PnuC (COG3201)NMN_trans_PnuC (TIGR01528)→ PRK11340 (PRK11340)Metallophos (pfam00149)→
env-1253	DNGF01000014.1	-	2615	2513	**RNA**→ PnuC (COG3201)NMN_trans_PnuC (TIGR01528)→
env-1254	Ga0116650_1061829	-	317	215	**RNA**→ hypo→
env-1255	OGEJ01016318.1	+	354	457	**RNA**→ PnuC (COG3201)NMN_trans_PnuC (TIGR01528)→
env-1256	Pasolli2019-4528-0	-	28365	28262	**RNA**→ PnuC (COG3201)NMN_trans_PnuC (TIGR01528)→
env-1257	UMGS1268-0	-	28365	28262	**RNA**→ PnuC (COG3201)NMN_trans_PnuC (TIGR01528)→
env-1258	OIXH01140209.1	+	156	259	**RNA**→ NMN_transporter (pfam04973)→
env-1259	Pasolli2019-5055-8	+	33856	33959	**RNA**→ PnuC (COG3201)NMN_trans_PnuC (TIGR01528)→ CysI (COG0155)PLN02431 (PLN02431)→
env-1260	Ga0129306_1001087	+	17084	17189	**RNA**→ PnuC (COG3201)NMN_trans_PnuC (TIGR01528)→
env-1261	Ga0129307_1001030	+	17084	17189	Zn_dep_PLPC (cd11009)Zn_dep_PLPC (smart00770)→<br>**RNA**→ PnuC (COG3201)NMN_trans_PnuC (TIGR01528)→<br>Zn_dep_PLPC (cd11009)Zn_dep_PLPC (smart00770)→
env-1262	AUXO018134300.1	+	61	165	**RNA**→ hypo→
env-1263	DOUO01000044.1	-	3087	2983	**RNA**→ PnuC (COG3201)NMN_trans_PnuC (TIGR01528)→
env-1264	DEUM01000013.1	+	61887	61991	**RNA**→ PnuC (COG3201)NMN_trans_PnuC (TIGR01528)→
env-1265	DKXU01000099.1	-	37001	36897	**RNA**→ PnuC (COG3201)NMN_trans_PnuC (TIGR01528)→
env-1266	Ga0134523_1029192	+	1165	1268	**RNA**→ PnuC (COG3201)NMN_trans_PnuC (TIGR01528)→
env-1267	Pasolli2019-9331-104	+	1737	1840	**RNA**→ PnuC (COG3201)NMN_trans_PnuC (TIGR01528)→
env-1268	Pasolli2019-14020-11	+	42153	42256	**RNA**→ PnuC (COG3201)NMN_trans_PnuC (TIGR01528)→
env-1269	SRS064276_C2423061	-	225	122	**RNA**→
env-1270	Ga0209075_1659383	+	27	130	**RNA**→ NMN_transporter (pfam04973)→
env-1271	O8_19_POLYDEXT_DNA_scaffold47415_2	+	263	354	**RNA**→ hypo→
Eba-1-1	NZ_FNOU01000011.1	-	50191	50091	**RNA**→ PnuC (COG3201)NMN_trans_PnuC (TIGR01528)→
env-1272	AUXO013285693.1	-	149	44	**RNA**→
env-1273	AUXO011171916.1	-	163	69	**RNA**→ hypo→
env-1274	AUXO013944861.1	-	191	90	**RNA**→
env-1275	OGMF01042976.1	-	1025	923	**RNA**→ NMN_trans_PnuC (TIGR01528)NMN_transporter (pfam04973)→ hypo→
env-1276	DGIH01000001.1	+	3583	3683	**RNA**→ PnuC (COG3201)NMN_trans_PnuC (TIGR01528)→
env-1277	DMAV01000251.1	+	3194	3294	**RNA**→ PnuC (COG3201)NMN_trans_PnuC (TIGR01528)→
env-1278	SRS011586_C3518805	+	859	964	**RNA**→ hypo→
env-1279	UnmappedStool_Broad_scaffold_1104288	-	538	433	**RNA**→ PnuC (COG3201)NMN_trans_PnuC (TIGR01528)→
env-1280	DLM008_scaffold50494_5	-	1826	1721	**RNA**→ PnuC (COG3201)NMN_trans_PnuC (TIGR01528)→
env-1281	MNTP01000050.1	-	62284	62179	**RNA**→ PnuC (COG3201)NMN_trans_PnuC (TIGR01528)→
env-1282	OIXG01112234.1	+	89	194	**RNA**→ PnuC (COG3201)→
env-1283	OLGD01001256.1	-	5827	5722	**RNA**→ PnuC (COG3201)NMN_trans_PnuC (TIGR01528)→
env-1284	Pasolli2019-4184-4	+	75942	76047	**RNA**→ PnuC (COG3201)NMN_trans_PnuC (TIGR01528)→
env-1285	UMGS1538-30	-	1221	1116	**RNA**→ PnuC (COG3201)NMN_trans_PnuC (TIGR01528)→
env-1286	scaffold19863_2_MH0045	-	490	385	**RNA**→ PnuC (COG3201)NMN_trans_PnuC (TIGR01528)→
env-1287	SRS015133_C3495561	-	1421	1316	**RNA**→ PnuC (COG3201)NMN_trans_PnuC (TIGR01528)→
env-1288	OGNQ01006323.1	-	2381	2276	**RNA**→ PnuC (COG3201)NMN_trans_PnuC (TIGR01528)→
env-1289	scaffold87131_4_V1.CD-14	-	499	394	**RNA**→
env-1290	Ga0117811_1007557	-	1900	1795	**RNA**→ PnuC (COG3201)NMN_trans_PnuC (TIGR01528)→
env-1291	Ga0134523_1001842	-	5783	5678	**RNA**→ PnuC (COG3201)NMN_trans_PnuC (TIGR01528)→
env-1292	Pasolli2019-14260-37	-	705	602	**RNA**→ PnuC (COG3201)NMN_trans_PnuC (TIGR01528)→
Cbo-1-1	NZ_HG917868.1	-	2610886	2610783	**RNA**→ PnuC (COG3201)NMN_trans_PnuC (TIGR01528)→ Beta4Glucosyltransferase (cd02511)TPR (sd00006)-
env-1293	Ga0134523_1275263	-	132	29	**RNA**→
env-1294	DLM019_scaffold11464_1	+	2293	2398	**RNA**→ NMN_transporter (pfam04973)→
env-1295	OGMF01080467.1	+	231	336	**RNA**→ NMN_transporter (pfam04973)→
env-1296	OGIE01000531.1	-	3922	3817	**RNA**→ NMN_transporter (pfam04973)→
env-1297	OGII01001290.1	-	3922	3817	**RNA**→ NMN_transporter (pfam04973)→
env-1298	Pasolli2019-14927-7	-	4124	4019	**RNA**→ NMN_transporter (pfam04973)→
env-1299	UMGS948-8	-	5411	5306	**RNA**→ NMN_transporter (pfam04973)→

env-1300	AUXO013501272.1	+	139	242	**RNA**→ PnuC (COG3201)→
env-1301	EMG_10080117	+	1350	1452	**RNA**→ PnuC (COG3201)NMN_trans_PnuC (TIGR01528)→
env-1302	Ga0172378_11817616	+	130	232	**RNA**→ hypo→
env-1303	Pasolli2019-4156-69	-	6395	6293	**RNA**→ PnuC (COG3201)NMN_trans_PnuC (TIGR01528)→
env-1304	OLGD01072815.1	+	478	585	**RNA**→ NMN_transporter (pfam04973)→
env-1305	Pasolli2019-14049-30	-	13387	13280	**RNA**→ PnuC (COG3201)NMN_trans_PnuC (TIGR01528)→
env-1306	Pasolli2019-4333-4	-	75438	75339	**RNA**→ PnuC (COG3201)NMN_trans_PnuC (TIGR01528)→
env-1307	Pasolli2019-4334-14	+	3341	3440	**RNA**→ hypo→
env-1308	OIZH01000983.1	-	1756	1657	**RNA**→ PnuC (COG3201)NMN_trans_PnuC (TIGR01528)→
env-1309	OLHA01008676.1	+	2917	3016	**RNA**→ hypo→
env-1310	UMGS508-11	+	5664	5763	**RNA**→ PnuC (COG3201)NMN_trans_PnuC (TIGR01528)→
env-1311	DKZK01000014.1	-	58560	58459	**RNA**→ vmrA (PRK09575)MATE_MepA_like (cd13143)→
env-1312	OMYH01000056.1	+	10668	10768	**RNA**→ PnuC (COG3201)NMN_trans_PnuC (TIGR01528)→
env-1313	ONJL01000138.1	-	5683	5583	**RNA**→ PnuC (COG3201)NMN_trans_PnuC (TIGR01528)→ C_GCAxxG_C_C (pfam09719)→
env-1314	ONXU01000232.1	+	2164	2264	**RNA**→ PnuC (COG3201)NMN_trans_PnuC (TIGR01528)→
Bma-2-1	NZ_LN908977.1	+	262126	262228	**RNA**→ PnuC (COG3201)NMN_trans_PnuC (TIGR01528)→
env-1315	longitudinal_413_738	+	61478	61580	**RNA**→ PnuC (COG3201)NMN_trans_PnuC (TIGR01528)→
Csp-4-1	NZ_KE993565.1	+	1390	1492	**RNA**→ PnuC (COG3201)NMN_trans_PnuC (TIGR01528)→
Csp-8-1	NZ_FQVY01000006.1	+	122504	122606	**RNA**→ PnuC (COG3201)NMN_trans_PnuC (TIGR01528)→
env-1316	AUXO010193058.1	+	89	189	**RNA**→ PLN02731 (PLN02731)PnuC (COG3201)→
env-1317	SRS023914_C1892693	+	92	188	**RNA**→ ←hypo
env-1318	SRS050752_LANL_scaffold_21620	+	219	315	**RNA**→ PnuC (COG3201)NMN_trans_PnuC (TIGR01528)→
env-1319	SRS051882_C2568373	+	232	328	**RNA**→ PnuC (COG3201)NMN_trans_PnuC (TIGR01528)→
env-1320	Pasolli2019-13983-8	-	83932	83836	**RNA**→ PnuC (COG3201)NMN_trans_PnuC (TIGR01528)→
env-1321	SRS018817_C2112822	+	78	174	**RNA**→ PnuC (COG3201)NMN_trans_PnuC (TIGR01528)→
env-1322	SRS050422_C2569824	-	223	127	**RNA**→ ←hypo
env-1323	SRS050925_C2130151	-	369	273	**RNA**→ PnuC (COG3201)→
env-1324	scaffold5459_3_MH0075	-	489	393	**RNA**→ ←hypo
env-1325	scaffold11029_2_MH0012	-	2043	1938	**RNA**→ PnuC (COG3201)NMN_trans_PnuC (TIGR01528)→
env-1326	Ga0169870_100084	-	93163	93062	**RNA**→ PnuC (COG3201)NMN_trans_PnuC (TIGR01528)→
env-1327	Ga0169737_100200	+	4998	5099	**RNA**→ PnuC (COG3201)NMN_trans_PnuC (TIGR01528)→
env-1328	Ga0169773_100178	-	19542	19441	**RNA**→ PnuC (COG3201)NMN_trans_PnuC (TIGR01528)→
env-1329	Ga0169833_100025	-	98258	98157	**RNA**→ PnuC (COG3201)NMN_trans_PnuC (TIGR01528)→
env-1330	Pasolli2019-4752-33	-	26321	26220	**RNA**→ PnuC (COG3201)NMN_trans_PnuC (TIGR01528)→
env-1331	UMGS35-33	-	26321	26220	**RNA**→ PnuC (COG3201)NMN_trans_PnuC (TIGR01528)→
env-1332	Ga0169821_100609	-	19504	19403	**RNA**→ PnuC (COG3201)NMN_trans_PnuC (TIGR01528)→
env-1333	Ga0169781_110243	+	437	538	**RNA**→ PnuC (COG3201)NMN_trans_PnuC (TIGR01528)→ hypo→
env-1334	Ga0169882_100017	-	80482	80381	**RNA**→ PnuC (COG3201)NMN_trans_PnuC (TIGR01528)→
env-1335	Ga0134379_103404	-	1855	1754	**RNA**→ PnuC (COG3201)NMN_trans_PnuC (TIGR01528)→
env-1336	Ga0134417_103994	+	657	758	**RNA**→ PnuC (COG3201)NMN_trans_PnuC (TIGR01528)→
env-1337	Ga0134394_103658	-	1715	1614	**RNA**→ PnuC (COG3201)NMN_trans_PnuC (TIGR01528)→
env-1338	Ga0134407_1052657	-	190	89	**RNA**→ hypo→
env-1339	Ga0169861_107316	-	1008	907	**RNA**→ PnuC (COG3201)NMN_trans_PnuC (TIGR01528)→
env-1340	scaffold12619_3_V1.CD-1	-	914	813	**RNA**→ PnuC (COG3201)NMN_trans_PnuC (TIGR01528)→
env-1341	SRS012902_C537160	-	1668	1567	**RNA**→ PnuC (COG3201)NMN_trans_PnuC (TIGR01528)→
env-1342	SRS016267_C4380068	+	135	236	**RNA**→ PnuC (COG3201)→
env-1343	SRS024549_C944239	-	276	175	**RNA**→ hypo→
env-1344	CAM_READ_0099806247	-	158	57	**RNA**→ hypo→
env-1345	SRS023829_C3071302	-	1668	1567	**RNA**→ PnuC (COG3201)NMN_trans_PnuC (TIGR01528)→
env-1346	SRS075398_C2057681	-	822	721	**RNA**→ PnuC (COG3201)NMN_trans_PnuC (TIGR01528)→
env-1347	UnmappedStool_Broad_scaffold_795128	+	1085	1186	**RNA**→ PnuC (COG3201)NMN_trans_PnuC (TIGR01528)→
env-1348	DOF006_scaffold9214_1	-	3488	3387	**RNA**→ PnuC (COG3201)NMN_trans_PnuC (TIGR01528)→

env-1349	DOM010_scaffold1574_13	+	367	468	**RNA**→ PnuC (COG3201)NMN_trans_PnuC (TIGR01528)→
env-1350	OGDQ01026490.1	+	223	324	**RNA**→ PnuC (COG3201)→
env-1351	OGGP01005878.1	-	809	708	**RNA**→ PnuC (COG3201)NMN_trans_PnuC (TIGR01528)→
env-1352	OGHV01046575.1	-	354	253	**RNA**→ PnuC (COG3201)→
env-1353	OGIN01000048.1	+	147486	147587	**RNA**→ PnuC (COG3201)NMN_trans_PnuC (TIGR01528)→
env-1354	OGLK01000370.1	-	3690	3589	**RNA**→ PnuC (COG3201)NMN_trans_PnuC (TIGR01528)→
env-1355	OGMN01012315.1	-	984	883	**RNA**→ PnuC (COG3201)NMN_trans_PnuC (TIGR01528)→
env-1356	OGMW01004249.1	+	4158	4259	**RNA**→ PnuC (COG3201)NMN_trans_PnuC (TIGR01528)→
env-1357	OGNA01000680.1	-	4957	4856	**RNA**→ PnuC (COG3201)NMN_trans_PnuC (TIGR01528)→
env-1358	OGNW01006073.1	+	1799	1900	**RNA**→ PnuC (COG3201)NMN_trans_PnuC (TIGR01528)→
env-1359	OLGL01017877.1	+	1261	1362	**RNA**→ hypo→
env-1360	PPYE01030207.1	-	24437	24336	**RNA**→ PnuC (COG3201)NMN_trans_PnuC (TIGR01528)→
env-1361	Pasolli2019-4753-20	-	2004	1903	**RNA**→ PnuC (COG3201)NMN_trans_PnuC (TIGR01528)→
env-1362	Pasolli2019-4859-193	-	3760	3659	**RNA**→ PnuC (COG3201)NMN_trans_PnuC (TIGR01528)→
env-1363	OBJE01012006.1	-	1551	1450	**RNA**→ PnuC (COG3201)NMN_trans_PnuC (TIGR01528)→
env-1364	OGJZ01025348.1	+	264	365	**RNA**→ NMN_transporter (pfam04973)→
env-1365	CAM_READ_0099986917	+	381	482	**RNA**→
env-1366	Pasolli2019-15305-447	+	772	874	**RNA**→ PnuC (COG3201)NMN_trans_PnuC (TIGR01528)→
Mma-1-1	NZ_FUHT01000003.1	+	706132	706236	**RNA**→ PnuC (COG3201)NMN_trans_PnuC (TIGR01528)→
env-1367	UMGS475-6	+	11654	11752	**RNA**→ NMN_transporter (pfam04973)NMN_trans_PnuC (TIGR01528)→
env-1368	SRS011405_WUGC_scaffold_22668	+	1309	1407	**RNA**→ NMN_trans_PnuC (TIGR01528)NMN_transporter (pfam04973)→
env-1369	SRS024009_C1829938	-	8636	8538	**RNA**→ NMN_trans_PnuC (TIGR01528)NMN_transporter (pfam04973)→
env-1370	SRS049995_C2637312	-	8999	8901	**RNA**→ NMN_trans_PnuC (TIGR01528)NMN_transporter (pfam04973)→
env-1371	scaffold19413_10_MH0071	-	1504	1406	**RNA**→ NMN_trans_PnuC (TIGR01528)NMN_transporter (pfam04973)→
env-1372	SRS016335_WUGC_scaffold_25085	-	3498	3400	**RNA**→ NMN_trans_PnuC (TIGR01528)NMN_transporter (pfam04973)→
env-1373	SRS049959_WUGC_scaffold_7704	-	474	376	**RNA**→ PnuC (COG3201)NMN_trans_PnuC (TIGR01528)→
env-1374	4491402.3_NODE_4434	+	1	99	**RNA**→ NMN_trans_PnuC (TIGR01528)NMN_transporter (pfam04973)→
env-1375	4491405.3_NODE_707	-	48952	48854	**RNA**→ NMN_trans_PnuC (TIGR01528)NMN_transporter (pfam04973)→
env-1376	4491423.3_NODE_2382	+	6808	6906	**RNA**→ NMN_trans_PnuC (TIGR01528)NMN_transporter (pfam04973)→
env-1377	4491477.3_NODE_69813	-	147	49	**RNA**→
env-1378	Ga0134406_1061659	+	170	268	**RNA**→ hypo→
env-1379	Ga0134371_1000091	+	16606	16704	**RNA**→ NMN_trans_PnuC (TIGR01528)NMN_transporter (pfam04973)→
env-1380	Ga0134443_1021707	+	873	971	**RNA**→ hypo→
env-1381	Ga0134523_1007078	-	6216	6118	**RNA**→ NMN_trans_PnuC (TIGR01528)NMN_transporter (pfam04973)→
env-1382	SRS011239_Baylor_scaffold_26823	-	884	786	**RNA**→ NMN_trans_PnuC (TIGR01528)NMN_transporter (pfam04973)→
env-1383	SRS018817_WUGC_scaffold_17729	+	8904	9002	**RNA**→ NMN_trans_PnuC (TIGR01528)NMN_transporter (pfam04973)→ hypo→
env-1384	SRS022609_Baylor_scaffold_41100	+	8522	8620	**RNA**→ NMN_trans_PnuC (TIGR01528)NMN_transporter (pfam04973)→ hypo→
env-1385	SRS052697_LANL_scaffold_69500	-	8764	8666	**RNA**→ NMN_trans_PnuC (TIGR01528)NMN_transporter (pfam04973)→
env-1386	Pasolli2019-14114-15	-	14019	13921	**RNA**→ NMN_transporter (pfam04973)NMN_trans_PnuC (TIGR01528)→
env-1387	scaffold44257_1_MH0066	+	528	626	**RNA**→ NMN_trans_PnuC (TIGR01528)NMN_transporter (pfam04973)→
env-1388	4491410.3_NODE_390	-	40745	40647	**RNA**→ NMN_trans_PnuC (TIGR01528)NMN_transporter (pfam04973)→
env-1389	4491412.3_NODE_1599	-	23777	23679	**RNA**→ NMN_trans_PnuC (TIGR01528)NMN_transporter (pfam04973)→
env-1390	scaffold42300_2_MH0009	+	563	661	**RNA**→ PnuC (COG3201)NMN_trans_PnuC (TIGR01528)→
env-1391	scaffold15479_4_MH0075	+	1322	1420	**RNA**→ PnuC (COG3201)NMN_trans_PnuC (TIGR01528)→
env-1392	4491417.3_NODE_57419	+	2923	3021	**RNA**→ NMN_trans_PnuC (TIGR01528)NMN_transporter (pfam04973)→
env-1393	DPOV01000014.1	-	54053	53955	**RNA**→ NMN_trans_PnuC (TIGR01528)NMN_transporter (pfam04973)→
env-1394	SRS019582_WUGC_scaffold_48116	-	1215	1117	**RNA**→ NMN_trans_PnuC (TIGR01528)NMN_transporter (pfam04973)→
env-1395	SRS020869_Baylor_scaffold_16483	+	1892	1990	**RNA**→ NMN_trans_PnuC (TIGR01528)NMN_transporter (pfam04973)→
env-1396	SRS024132_C3691383	-	317	219	**RNA**→ PnuC (COG3201)→
env-1397	SRS024435_C3166466	+	296	394	**RNA**→ NMN_trans_PnuC (TIGR01528)NMN_transporter (pfam04973)→
env-1398	SRS065504_LANL_scaffold_55561	+	1311	1409	**RNA**→ NMN_trans_PnuC (TIGR01528)NMN_transporter (pfam04973)→
env-1399	Ga0134458_138140	+	43	141	**RNA**→ PnuC (COG3201)NMN_trans_PnuC (TIGR01528)→

env-1400	C1998624_1_MH0079	+	300	398	**RNA**→ hypo→
env-1401	SRS020233_C4277441	-	15190	15092	**RNA**→ NMN_trans_PnuC (TIGR01528)NMN_transporter (pfam04973)→
env-1402	Pasolli2019-3941-1	+	13374	13476	**RNA**→ NMN_transporter (pfam04973)→
env-1403	UnmappedStool_Broad_C252733293	+	72	176	**RNA**→ PnuC (COG3201)→
env-1404	Pasolli2019-14033-2	-	68377	68273	**RNA**→ PnuC (COG3201)NMN_trans_PnuC (TIGR01528)→
env-1405	Ga0116650_1092949	+	154	258	**RNA**→ ←hypo
env-1406	Ga0116619_1016101	-	495	391	**RNA**→ PnuC (COG3201)→
env-1407	DEFH01000022.1	-	32707	32603	**RNA**→ PnuC (COG3201)NMN_trans_PnuC (TIGR01528)→
env-1408	Pasolli2019-4352-8	-	66546	66448	**RNA**→ PnuC (COG3201)NMN_trans_PnuC (TIGR01528)→ AlkD_like (cd06561)DNA_alkylation (pfam08713)→
env-1409	OIYS01008524.1	-	2865	2771	**RNA**→ PnuC (COG3201)NMN_trans_PnuC (TIGR01528)→ hypo→
env-1410	2211076460	-	145	43	**RNA**→
env-1411	2157103779	-	145	43	**RNA**→
env-1412	Pasolli2019-4154-5	-	42153	42051	**RNA**→ NMN_transporter (pfam04973)→ NMN_transporter (pfam04973)→
env-1413	OIYV01057808.1	-	570	468	**RNA**→ hypo→
env-1414	OLGD01126773.1	+	74	173	**RNA**→ PnuC (COG3201)→
env-1415	Ga0120384_1059936	-	911	807	**RNA**→ PnuC (COG3201)NMN_trans_PnuC (TIGR01528)→
env-1416	DJOQ01000002.1	-	15473	15371	**RNA**→ PnuC (COG3201)NMN_trans_PnuC (TIGR01528)→ hypo→ NhaP (COG0025)2a37 (TIGR00932)→
env-1417	DMRO01000154.1	+	346	448	**RNA**→ PnuC (COG3201)NMN_trans_PnuC (TIGR01528)→ hypo→ NhaP (COG0025)2a37 (TIGR00932)→
env-1418	Ga0116619_1025438	+	487	589	**RNA**→ PnuC (COG3201)→
[gl-1-1	NZ_BBAB01000002.1	+	121049	121152	**RNA**→ hypo→
env-1419	OIXH01006072.1	+	4128	4249	**RNA**→ PnuC (COG3201)NMN_trans_PnuC (TIGR01528)→
env-1420	UMGS1626-89	+	4128	4249	**RNA**→ PnuC (COG3201)NMN_trans_PnuC (TIGR01528)→
env-1421	UMGS1950-9	+	9122	9223	**RNA**→ PnuC (COG3201)NMN_trans_PnuC (TIGR01528)→
env-1422	OIXH01102293.1	+	418	519	**RNA**→
env-1423	Ga0129313_1000143	-	16446	16345	**RNA**→ PnuC (COG3201)NMN_trans_PnuC (TIGR01528)→
env-1424	Ga0121377_101312	-	2606	2505	**RNA**→ PnuC (COG3201)NMN_trans_PnuC (TIGR01528)→
env-1425	Pasolli2019-14214-38	-	1413	1312	**RNA**→ PnuC (COG3201)NMN_trans_PnuC (TIGR01528)→
env-1426	UMGS2055-102	+	3234	3335	**RNA**→ PnuC (COG3201)NMN_trans_PnuC (TIGR01528)→
env-1427	ONON01000115.1	+	8597	8698	**RNA**→ PnuC (COG3201)NMN_trans_PnuC (TIGR01528)→
env-1428	Ga0209694_1185009	-	257	155	**RNA**→ hypo→
env-1429	SRS016517_C20376232	-	759	656	**RNA**→ PnuC (COG3201)NMN_trans_PnuC (TIGR01528)→
env-1430	SRS016495_C1900073	-	667	564	**RNA**→ PnuC (COG3201)NMN_trans_PnuC (TIGR01528)→
env-1431	UnmappedStool_Broad_scaffold_911568	+	689	792	**RNA**→ PnuC (COG3201)NMN_trans_PnuC (TIGR01528)→
env-1432	Ga0134492_1167287	+	193	296	**RNA**→ hypo→
env-1433	Pasolli2019-14941-2	+	10900	11003	**RNA**→ PnuC (COG3201)NMN_trans_PnuC (TIGR01528)→
env-1434	SRS056519_LANL_scaffold_36973	+	49	152	**RNA**→ PnuC (COG3201)NMN_trans_PnuC (TIGR01528)→ hypo→ frr (PRK00083)Frr (COG0233)→ hypo→<br>PRK14830 (PRK14830)Cis_IPPS (cd00475)→ cdsA (PRK11624)CdsA (COG0575)→<br>Dxr (COG0743)PRK05447 (PRK05447)→
env-1435	OGMF01000078.1	-	68900	68780	**RNA**→ PnuC (COG3201)NMN_trans_PnuC (TIGR01528)→<br>HATPase_c (smart00387)YesM (COG2972)dCache_1 (pfam02743)→
env-1436	Pasolli2019-4146-1	-	65546	65426	**RNA**→ PnuC (COG3201)NMN_trans_PnuC (TIGR01528)→<br>YesM (COG2972)dCache_1 (pfam02743)HATPase_c (smart00387)→
env-1437	UMGS1883-234	+	512	632	**RNA**→ PnuC (COG3201)NMN_trans_PnuC (TIGR01528)→ His_kinase (pfam06580)dCache_1 (pfam02743)→
env-1438	Pasolli2019-125-81	-	5806	5705	**RNA**→ PnuC (COG3201)NMN_trans_PnuC (TIGR01528)→
env-1439	AUXO014244931.1	+	97	202	**RNA**→ hypo→
env-1440	UMGS1846-18	-	2086	1985	**RNA**→ PnuC (COG3201)NMN_trans_PnuC (TIGR01528)→
Cbo-3-1	NZ_AUYX01000009.1	+	457339	457440	**RNA**→ Pyridox_oxidase (pfam01243)Pyridox_ox_2 (pfam12900)→
env-1441	Pasolli2019-3951-8	-	2412	2307	**RNA**→ PnuC (COG3201)NMN_trans_PnuC (TIGR01528)→
env-1442	SRS017821_C2403080	+	350	455	**RNA**→ PnuC (COG3201)→
env-1443	Pasolli2019-4158-113	+	2602	2704	**RNA**→ PnuC (COG3201)NMN_trans_PnuC (TIGR01528)→<br>AdoMet_MTases (cd02440)Methyltransf_25 (pfam13649)→
env-1444	OGFF01023870.1	+	651	753	**RNA**→ PnuC (COG3201)NMN_trans_PnuC (TIGR01528)→
env-1445	OGHJ01019554.1	-	531	429	**RNA**→ PnuC (COG3201)→

env-1446	Ga0139311_1004220	-	9145	9043	**RNA**→ PnuC (COG3201)NMN_trans_PnuC (TIGR01528)→
env-1447	DPBR01000038.1	+	9555	9657	**RNA**→ PnuC (COG3201)NMN_trans_PnuC (TIGR01528)→
env-1448	Pasolli2019-4188-6	+	43191	43293	**RNA**→ PnuC (COG3201)NMN_trans_PnuC (TIGR01528)→
env-1449	SRS063040_C3122771	+	111	213	**RNA**→
env-1450	UnmappedStool_Broad_scaffold_503145	+	152	254	**RNA**→ PnuC (COG3201)NMN_trans_PnuC (TIGR01528)→
env-1451	Pasolli2019-14182-26	-	36123	36021	**RNA**→ PnuC (COG3201)NMN_trans_PnuC (TIGR01528)→
env-1452	SRS011302_Baylor_scaffold_6369	-	33391	33289	**RNA**→ PnuC (COG3201)NMN_trans_PnuC (TIGR01528)→
env-1453	SRS064276_C2503955	-	654	552	**RNA**→ PnuC (COG3201)NMN_trans_PnuC (TIGR01528)→
env-1454	DKZG01000008.1	-	31793	31690	**RNA**→ PnuC (COG3201)NMN_trans_PnuC (TIGR01528)→
env-1455	Pasolli2019-4120-31	+	3609	3713	**RNA**→ PnuC (COG3201)NMN_trans_PnuC (TIGR01528)→
env-1456	Ga0116646_1142095	+	91	192	**RNA**→ hypo→
env-1457	4491423.3_NODE_15031	-	739	639	**RNA**→ PnuC (COG3201)NMN_trans_PnuC (TIGR01528)→
env-1458	OJAR01000626.1	-	4197	4097	**RNA**→ PnuC (COG3201)NMN_trans_PnuC (TIGR01528)→
env-1459	Pasolli2019-15084-9	+	46180	46280	**RNA**→ PnuC (COG3201)NMN_trans_PnuC (TIGR01528)→
env-1460	OMQS01000073.1	+	1669	1769	**RNA**→ PnuC (COG3201)NMN_trans_PnuC (TIGR01528)→
env-1461	Ga0134563_119660	-	334	234	**RNA**→ NMN_transporter (pfam04973)→
env-1462	AUXO015758443.1	+	212	314	**RNA**→ PnuC (COG3201)NMN_trans_PnuC (TIGR01528)→
env-1463	Pasolli2019-14235-15	+	7034	7140	**RNA**→ PnuC (COG3201)NMN_trans_PnuC (TIGR01528)→
env-1464	UMGS731-44	-	6980	6874	**RNA**→ PnuC (COG3201)NMN_trans_PnuC (TIGR01528)→
env-1465	Ga0208297_1000372	-	58768	58669	**RNA**→ PnuC (COG3201)NMN_trans_PnuC (TIGR01528)→
env-1466	UMGS1289-1	-	97423	97322	**RNA**→ ←MqsA (cd12870)YgiT_finger (TIGR03831)
env-1467	PPYE01048950.1	-	3124	3023	**RNA**→ ←MqsA (cd12870)YgiT_finger (TIGR03831)
env-1468	Pasolli2019-4995-4	+	151889	151990	**RNA**→
env-1469	AUXO011045894.1	+	264	365	**RNA**→ PnuC (COG3201)NMN_trans_PnuC (TIGR01528)→
env-1470	rank12_10234386	-	266	165	**RNA**→ hypo→
env-1471	Ga0256404_1002593	-	10887	10786	**RNA**→ PnuC (COG3201)NMN_trans_PnuC (TIGR01528)→
env-1472	Ga0256405_10002390	+	31970	32071	**RNA**→ PnuC (COG3201)NMN_trans_PnuC (TIGR01528)→
env-1473	OGIG01015336.1	-	1672	1572	**RNA**→ PnuC (COG3201)NMN_trans_PnuC (TIGR01528)→
env-1474	SRS011134_C4789831	-	331	231	**RNA**→ hypo→
env-1475	OIWT01015674.1	-	845	745	**RNA**→ PnuC (COG3201)NMN_trans_PnuC (TIGR01528)→
env-1476	Pasolli2019-19228-38	+	3259	3360	**RNA**→ PnuC (COG3201)NMN_trans_PnuC (TIGR01528)→
env-1477	Pasolli2019-15411-108	-	6605	6504	**RNA**→ PnuC (COG3201)NMN_trans_PnuC (TIGR01528)→
env-1478	SRMUA_GNVGR9Q01D7ZK0	-	329	228	**RNA**→ PnuC (COG3201)→
env-1479	OMZF01000144.1	-	563	461	**RNA**→ PnuC (COG3201)→
env-1480	Pasolli2019-4399-343	-	1246	1144	**RNA**→ PnuC (COG3201)NMN_trans_PnuC (TIGR01528)→
env-1481	Ga0134492_1210319	+	159	265	**RNA**→ hypo→
env-1482	OMTB01000101.1	-	4536	4435	**RNA**→ ADPRase_NUDT5 (cd03424)TIGR00052 (TIGR00052)PTZ00401 (PTZ00401)→
env-1483	Pasolli2019-4141-8	+	43301	43393	**RNA**→ hypo→ hypo→ EEP-1 (cd09083)→
env-1484	UMGS1297-9	-	115677	115576	**RNA**→ PnuC (COG3201)→
env-1485	Ga0172378_11293805	-	336	233	**RNA**→ PnuC (COG3201)→
env-1486	ONIF01000093.1	+	1143	1248	**RNA**→ PnuC (COG3201)NMN_trans_PnuC (TIGR01528)→ RhaT (COG0697)→ P-type_ATPase (cd02609)ATPase_P-type (TIGR01494)→
env-1487	OIZW01045883.1	-	475	372	**RNA**→ PnuC (COG3201)NMN_trans_PnuC (TIGR01528)→
env-1488	Pasolli2019-14177-1	-	107245	107142	**RNA**→ PnuC (COG3201)NMN_trans_PnuC (TIGR01528)→
env-1489	SRS011586_C3506726	-	512	409	**RNA**→ PnuC (COG3201)NMN_trans_PnuC (TIGR01528)→
env-1490	AUXO018711526.1	-	6522	6417	**RNA**→ NMN_transporter (pfam04973)→ FAU1 (COG0212)MTHFS_bact (TIGR02727)→ AIRC (smart01001)COG1691 (COG1691)→ PRK04194 (PRK04194)DUF111 (pfam01969)→ NrdR (COG1327)nrdR (PRK00464)→ YIH1 (COG1739)DUF1949 (pfam09186)→ MA (smart00283)Tar (COG0840)→
env-1491	scaffold17545_1_MH0012	+	609	710	**RNA**→ hypo→
env-1492	OJAW01047632.1	+	629	730	**RNA**→

env-1493	Pasolli2019-14263-36	+	610	711	**RNA**→ FeoA (pfam04023)FeoA (smart00899)→ FeoA (COG1918)FeoA (smart00899)→<br>FeoB (COG0370)feoB (TIGR00437)→ P12 (pfam12669)→ MarR (COG1846)MarR_2 (pfam12802)→<br>ABC_MJ0796_LolCDE_FtsE (cd03255)AAA (smart00382)→<br>FtsX (pfam02687)ADOP (TIGR03434)lolCE (TIGR02212)→ CopZ (COG2608)PRK13748 (PRK13748)→
env-1494	AUXO016064054.1	+	100	201	**RNA**→ PnuC (COG3201)NMN_trans_PnuC (TIGR01528)→
env-1495	DFIB01000245.1	+	73	174	**RNA**→ PnuC (COG3201)NMN_trans_PnuC (TIGR01528)→
env-1496	Pasolli2019-4203-15	+	4718	4819	**RNA**→ PnuC (COG3201)NMN_trans_PnuC (TIGR01528)→
env-1497	Pasolli2019-4202-7	+	11860	11961	**RNA**→ PnuC (COG3201)NMN_trans_PnuC (TIGR01528)→
env-1498	Ga0134371_1064784	-	355	254	**RNA**→ PnuC (COG3201)→
env-1499	Ga0134443_1001566	+	8583	8684	**RNA**→ PnuC (COG3201)→
env-1500	Ga0134458_103133	+	309	410	**RNA**→ PnuC (COG3201)NMN_trans_PnuC (TIGR01528)→<br>PRK11440 (PRK11440)cysteine_hydrolases (cd00431)→
env-1501	Pasolli2019-4405-4	-	45154	45053	**RNA**→ PnuC (COG3201)NMN_trans_PnuC (TIGR01528)→
env-1502	UMGS577-5	-	4223	4122	**RNA**→ PnuC (COG3201)NMN_trans_PnuC (TIGR01528)→
env-1503	Pasolli2019-15378-306	+	1539	1640	**RNA**→ NMN_transporter (pfam04973)→
env-1504	Ga0120384_1571927	-	103	1	**RNA**→
env-1505	AUXO014969001.1	-	936	834	**RNA**→ PnuC (COG3201)NMN_trans_PnuC (TIGR01528)→
env-1506	OIYV01003147.1	-	6070	5967	**RNA**→ PnuC (COG3201)NMN_trans_PnuC (TIGR01528)→<br>Methyltransf_7 (pfam03492)Pyridox_ox_2 (pfam12900)→
env-1507	OIZF01000014.1	-	115353	115250	**RNA**→ PnuC (COG3201)NMN_trans_PnuC (TIGR01528)→<br>Methyltransf_7 (pfam03492)Pyridox_ox_2 (pfam12900)→
env-1508	UMGS127-6	-	115353	115250	**RNA**→ PnuC (COG3201)NMN_trans_PnuC (TIGR01528)→<br>Methyltransf_7 (pfam03492)Pyridox_ox_2 (pfam12900)→
env-1509	Pasolli2019-4330-0	+	11026	11129	**RNA**→ PnuC (COG3201)NMN_trans_PnuC (TIGR01528)→<br>Pyridox_oxidase (pfam01243)Pyridox_ox_2 (pfam12900)→
env-1510	DJET01000040.1	-	35448	35347	**RNA**→ PnuC (COG3201)NMN_trans_PnuC (TIGR01528)→
env-1511	OGDY01023284.1	+	568	669	**RNA**→ (big gap) ←hypo
env-1512	Pasolli2019-4393-7	+	50871	50972	**RNA**→ PnuC (COG3201)→
env-1513	UMGS738-2	-	63541	63440	**RNA**→ hypo→
env-1514	AUXO012484392.1	-	1467	1365	**RNA**→ PnuC (COG3201)NMN_trans_PnuC (TIGR01528)→
env-1515	DFID01000094.1	+	119	221	**RNA**→ PnuC (COG3201)NMN_trans_PnuC (TIGR01528)→
env-1516	Pasolli2019-4408-210	+	1130	1231	**RNA**→ PnuC (COG3201)NMN_trans_PnuC (TIGR01528)→
env-1517	DHJQ01000068.1	-	3477	3376	**RNA**→ PnuC (COG3201)NMN_trans_PnuC (TIGR01528)→
env-1518	OIYG01002780.1	-	7745	7644	**RNA**→ MarR (COG1846)MarR_2 (pfam12802)→ AAA (smart00382)MdlB (COG1132)→<br>AAA (smart00382)MdlB (COG1132)→
env-1519	OIZO01001024.1	+	5209	5310	**RNA**→ MarR (COG1846)MarR_2 (pfam12802)→ AAA (smart00382)MdlB (COG1132)→<br>AAA (smart00382)MdlB (COG1132)→
env-1520	OLHA01008183.1	-	2855	2754	**RNA**→ ←Pyridox_oxidase (pfam01243)Pyridox_ox_2 (pfam12900)
env-1521	DJOL01000120.1	-	2254	2153	**RNA**→ PnuC (COG3201)NMN_trans_PnuC (TIGR01528)→
env-1522	DCBL01000059.1	-	4614	4513	**RNA**→ PnuC (COG3201)NMN_trans_PnuC (TIGR01528)→
env-1523	Ga0172378_11641594	+	196	298	**RNA**→ hypo→
env-1524	Pasolli2019-14207-27	+	4823	4925	**RNA**→ PnuC (COG3201)NMN_trans_PnuC (TIGR01528)→
env-1525	DKVJ01000008.1	+	5653	5751	**RNA**→ PnuC (COG3201)NMN_trans_PnuC (TIGR01528)→
env-1526	DKXR01000008.1	+	972	1070	**RNA**→ PnuC (COG3201)NMN_trans_PnuC (TIGR01528)→
env-1527	DKUE01000028.1	-	46483	46385	**RNA**→ PnuC (COG3201)NMN_trans_PnuC (TIGR01528)→
env-1528	DKWD01000022.1	-	47328	47230	**RNA**→ PnuC (COG3201)NMN_trans_PnuC (TIGR01528)→
env-1529	Ga0256404_1001904	-	35267	35167	**RNA**→ PnuC (COG3201)NMN_trans_PnuC (TIGR01528)→
env-1530	Pasolli2019-14872-7	+	77130	77229	**RNA**→ PnuC (COG3201)NMN_trans_PnuC (TIGR01528)→
env-1531	UMGS263-29	+	8345	8444	**RNA**→ PnuC (COG3201)NMN_trans_PnuC (TIGR01528)→
env-1532	OIZA01001464.1	-	1791	1686	**RNA**→ PnuC (COG3201)NMN_trans_PnuC (TIGR01528)→
env-1533	Pasolli2019-4377-42	+	6509	6614	**RNA**→ PnuC (COG3201)NMN_trans_PnuC (TIGR01528)→
env-1534	UMGS1867-39	-	1791	1686	**RNA**→ PnuC (COG3201)NMN_trans_PnuC (TIGR01528)→
env-1535	Pasolli2019-14934-22	-	3727	3626	**RNA**→ PnuC (COG3201)NMN_trans_PnuC (TIGR01528)→

env-1536	DCJK01000040.1	-	23878	23778	RNA→ NMN_trans_PnuC (TIGR01528)NMN_transporter (pfam04973)→ PRK07219 (PRK07219)zf-C4_Topoisom (pfam01396)NERD (pfam08378)→
env-1537	EFB_1016762	-	8279	8179	RNA→ NMN_trans_PnuC (TIGR01528)NMN_transporter (pfam04973)→ PRK07219 (PRK07219)zf-C4_Topoisom (pfam01396)NERD (pfam08378)→
env-1538	Ga0129311_1010528	+	2066	2166	RNA→ PnuC (COG3201)NMN_trans_PnuC (TIGR01528)→
env-1539	Pasolli2019-4149-30	-	20558	20456	RNA→ PnuC (COG3201)NMN_trans_PnuC (TIGR01528)→
env-1540	O8_19_POLYDEXT_DNA_scaffold31617_3	+	7	109	RNA→ PnuC (COG3201)NMN_trans_PnuC (TIGR01528)→
env-1541	scaffold70689_1_V1.UC-15	+	555	657	RNA→
env-1542	scaffold98653_1_V1.UC-8	+	675	777	RNA→
env-1543	SRS058723_Baylor_scaffold_4767	+	159	261	RNA→
env-1544	OGEO01040827.1	-	129	27	RNA→
env-1545	OGGF01028551.1	+	35	137	RNA→ hypo→
env-1546	OGIE01018250.1	+	917	1019	RNA→ hypo→
env-1547	4491488.3_NODE_2084	+	802	904	RNA→ PnuC (COG3201)NMN_trans_PnuC (TIGR01528)→
env-1548	NLF007_C991443_1	-	859	757	RNA→ PnuC (COG3201)NMN_trans_PnuC (TIGR01528)→
env-1549	OGCY01000021.1	-	99609	99507	RNA→ PnuC (COG3201)NMN_trans_PnuC (TIGR01528)→
env-1550	OGFO01004858.1	+	754	856	RNA→ PnuC (COG3201)NMN_trans_PnuC (TIGR01528)→
env-1551	OGGE01014006.1	-	996	894	RNA→ PnuC (COG3201)NMN_trans_PnuC (TIGR01528)→
env-1552	OGMJ01009902.1	-	837	735	RNA→ PnuC (COG3201)NMN_trans_PnuC (TIGR01528)→
env-1553	DLF001_scaffold25834_1	-	897	795	RNA→ PnuC (COG3201)NMN_trans_PnuC (TIGR01528)→
env-1554	DLF002_C479785_1	+	734	836	RNA→ PnuC (COG3201)NMN_trans_PnuC (TIGR01528)→
env-1555	DLF008_C322841_1	+	733	835	RNA→ PnuC (COG3201)NMN_trans_PnuC (TIGR01528)→
env-1556	DLF013_C673952_1	+	738	840	RNA→ PnuC (COG3201)NMN_trans_PnuC (TIGR01528)→
env-1557	DLM003_C799303_1	+	734	836	RNA→ PnuC (COG3201)NMN_trans_PnuC (TIGR01528)→
env-1558	DLM007_C653239_1	+	734	836	RNA→ PnuC (COG3201)NMN_trans_PnuC (TIGR01528)→
env-1559	DLM012_C399997_1	+	509	611	RNA→ PnuC (COG3201)NMN_trans_PnuC (TIGR01528)→
env-1560	DLM013_C693903_1	-	738	636	RNA→ PnuC (COG3201)NMN_trans_PnuC (TIGR01528)→
env-1561	DLM015_C573754_1	+	740	842	RNA→ PnuC (COG3201)NMN_trans_PnuC (TIGR01528)→
env-1562	DLM028_C659924_1	-	865	763	RNA→ PnuC (COG3201)NMN_trans_PnuC (TIGR01528)→
env-1563	DOF003_C729551_1	+	740	842	RNA→ PnuC (COG3201)NMN_trans_PnuC (TIGR01528)→
env-1564	DOF007_C349829_1	-	897	795	RNA→ PnuC (COG3201)NMN_trans_PnuC (TIGR01528)→
env-1565	DOM001_scaffold46737_1	-	903	801	RNA→ PnuC (COG3201)NMN_trans_PnuC (TIGR01528)→
env-1566	DOM010_C538534_1	+	742	844	RNA→ PnuC (COG3201)NMN_trans_PnuC (TIGR01528)→
env-1567	DOM016_C502083_1	-	850	748	RNA→ PnuC (COG3201)NMN_trans_PnuC (TIGR01528)→
env-1568	DOM022_scaffold32132_2	-	862	760	RNA→ PnuC (COG3201)NMN_trans_PnuC (TIGR01528)→
env-1569	NLF010_scaffold16154_1	-	993	891	RNA→ PnuC (COG3201)NMN_trans_PnuC (TIGR01528)→
env-1570	NLM005_scaffold36742_2	+	499	601	RNA→ PnuC (COG3201)NMN_trans_PnuC (TIGR01528)→
env-1571	NLM023_scaffold48737_1	+	734	836	RNA→ PnuC (COG3201)NMN_trans_PnuC (TIGR01528)→
env-1572	NLM029_scaffold24671_2	-	862	760	RNA→ PnuC (COG3201)NMN_trans_PnuC (TIGR01528)→
env-1573	NOF001_scaffold58196_2	+	112	214	RNA→ PnuC (COG3201)NMN_trans_PnuC (TIGR01528)→
env-1574	NOF006_scaffold78_4	-	862	760	RNA→ PnuC (COG3201)NMN_trans_PnuC (TIGR01528)→
env-1575	NOM002_scaffold26215_2	-	862	760	RNA→ PnuC (COG3201)NMN_trans_PnuC (TIGR01528)→
env-1576	NOM017_C519428_1	+	742	844	RNA→ PnuC (COG3201)NMN_trans_PnuC (TIGR01528)→
env-1577	NOM019_C645318_1	+	696	798	RNA→ PnuC (COG3201)NMN_trans_PnuC (TIGR01528)→
env-1578	NOM023_scaffold3506_1	-	897	795	RNA→ PnuC (COG3201)NMN_trans_PnuC (TIGR01528)→
env-1579	NOM027_C297014_1	+	742	844	RNA→ PnuC (COG3201)NMN_trans_PnuC (TIGR01528)→
env-1580	OGDW01013381.1	+	706	808	RNA→ PnuC (COG3201)NMN_trans_PnuC (TIGR01528)→
env-1581	OGEE01009125.1	+	863	965	RNA→ PnuC (COG3201)NMN_trans_PnuC (TIGR01528)→
env-1582	OGFE01014118.1	-	873	771	RNA→ PnuC (COG3201)NMN_trans_PnuC (TIGR01528)→
env-1583	OGFJ01014138.1	+	863	965	RNA→ PnuC (COG3201)NMN_trans_PnuC (TIGR01528)→
env-1584	OGFN01009206.1	+	863	965	RNA→ PnuC (COG3201)NMN_trans_PnuC (TIGR01528)→
env-1585	OGFY01018661.1	-	1055	953	RNA→ PnuC (COG3201)NMN_trans_PnuC (TIGR01528)→
env-1586	OGGG01024619.1	+	88	190	RNA→ PnuC (COG3201)NMN_trans_PnuC (TIGR01528)→

env-1587	OGHY01016749.1	+	809	911	RNA→ PnuC (COG3201)NMN_trans_PnuC (TIGR01528)→
env-1588	OGIG01025925.1	+	437	539	RNA→ PnuC (COG3201)NMN_trans_PnuC (TIGR01528)→
env-1589	OGIP01002577.1	+	706	808	RNA→ PnuC (COG3201)NMN_trans_PnuC (TIGR01528)→
env-1590	OGIS01012853.1	-	968	866	RNA→ PnuC (COG3201)NMN_trans_PnuC (TIGR01528)→
env-1591	OGKA01015951.1	-	1055	953	RNA→ PnuC (COG3201)NMN_trans_PnuC (TIGR01528)→
env-1592	OGKC01001963.1	-	1016	914	RNA→ PnuC (COG3201)NMN_trans_PnuC (TIGR01528)→
env-1593	OGKR01015056.1	+	631	733	RNA→ PnuC (COG3201)NMN_trans_PnuC (TIGR01528)→
env-1594	OGKW01013381.1	+	706	808	RNA→ PnuC (COG3201)NMN_trans_PnuC (TIGR01528)→
env-1595	OGLI01015383.1	-	917	815	RNA→ PnuC (COG3201)NMN_trans_PnuC (TIGR01528)→
env-1596	OGLM01035592.1	+	754	856	RNA→ PnuC (COG3201)NMN_trans_PnuC (TIGR01528)→
env-1597	OGLS01011678.1	+	754	856	RNA→ PnuC (COG3201)NMN_trans_PnuC (TIGR01528)→
env-1598	OGMN01011708.1	+	658	760	RNA→ PnuC (COG3201)NMN_trans_PnuC (TIGR01528)→
env-1599	OGMV01008182.1	+	754	856	RNA→ PnuC (COG3201)NMN_trans_PnuC (TIGR01528)→
env-1600	OGMX01007195.1	+	917	1019	RNA→ PnuC (COG3201)NMN_trans_PnuC (TIGR01528)→
env-1601	OGUC01006387.1	-	1014	912	RNA→ PnuC (COG3201)NMN_trans_PnuC (TIGR01528)→
Psp-2-1	NZ_LT827110.1	-	989	887	RNA→ PnuC (COG3201)NMN_trans_PnuC (TIGR01528)→
Bsp-2-1	NZ_LT635538.1	-	989	887	RNA→ PnuC (COG3201)NMN_trans_PnuC (TIGR01528)→
Cdi-1-1	NZ_AVLH01000067.1	-	95070	94968	RNA→ PnuC (COG3201)NMN_trans_PnuC (TIGR01528)→
Tsp-2-1	NZ_FNWC01000003.1	+	3186	3288	RNA→ PnuC (COG3201)NMN_trans_PnuC (TIGR01528)→
env-1602	NLF014_scaffold14836_2	+	466	568	RNA→ PnuC (COG3201)NMN_trans_PnuC (TIGR01528)→ NMN_transporter (pfam04973)→
env-1603	OGEG01008028.1	-	1016	914	RNA→ PnuC (COG3201)NMN_trans_PnuC (TIGR01528)→
env-1604	4491405.3_NODE_15483	-	916	814	RNA→ PnuC (COG3201)NMN_trans_PnuC (TIGR01528)→
env-1605	OIXH01055700.1	-	936	834	RNA→ PnuC (COG3201)NMN_trans_PnuC (TIGR01528)→
env-1606	OIYT01020259.1	+	866	968	RNA→ PnuC (COG3201)NMN_trans_PnuC (TIGR01528)→
env-1607	4491487.3_NODE_133988	-	1781	1679	RNA→ PnuC (COG3201)NMN_trans_PnuC (TIGR01528)→
env-1608	DLF009_scaffold36663_1	+	637	739	RNA→ PnuC (COG3201)→
env-1609	DLM008_scaffold60392_2	-	1948	1846	RNA→ PnuC (COG3201)NMN_trans_PnuC (TIGR01528)→
env-1610	DOF009_scaffold31638_1	-	2306	2204	RNA→ PnuC (COG3201)NMN_trans_PnuC (TIGR01528)→
env-1611	DOF012_scaffold8565_2	+	1482	1584	RNA→ PnuC (COG3201)NMN_trans_PnuC (TIGR01528)→
env-1612	DOF014_scaffold26640_2	-	1550	1448	RNA→ PnuC (COG3201)NMN_trans_PnuC (TIGR01528)→
env-1613	DOM005_scaffold7842_1	+	4894	4996	RNA→ PnuC (COG3201)NMN_trans_PnuC (TIGR01528)→
env-1614	DOM008_scaffold12865_1	+	1040	1142	RNA→ PnuC (COG3201)NMN_trans_PnuC (TIGR01528)→
env-1615	DOM012_scaffold12088_3	-	643	541	RNA→ PnuC (COG3201)NMN_trans_PnuC (TIGR01528)→
env-1616	NLF001_scaffold39856_2	+	2465	2567	RNA→ PnuC (COG3201)NMN_trans_PnuC (TIGR01528)→
env-1617	NLF006_scaffold19110_1	+	675	777	RNA→ PnuC (COG3201)NMN_trans_PnuC (TIGR01528)→
env-1618	NLF008_scaffold14070_1	+	1410	1512	RNA→ PnuC (COG3201)NMN_trans_PnuC (TIGR01528)→
env-1619	NLF013_scaffold835_2	-	1155	1053	RNA→ PnuC (COG3201)NMN_trans_PnuC (TIGR01528)→
env-1620	NLM008_scaffold27716_1	-	3303	3201	RNA→ PnuC (COG3201)NMN_trans_PnuC (TIGR01528)→ hypo→ hypo→<br>ribD_Cterm (TIGR00227)FolA (COG0262)→
env-1621	NOF012_scaffold37889_2	+	567	669	RNA→ PnuC (COG3201)→
env-1622	NOM013_scaffold40490_1	-	2012	1910	RNA→ PnuC (COG3201)NMN_trans_PnuC (TIGR01528)→
env-1623	NOM028_C554829_1	+	662	764	RNA→ PnuC (COG3201)NMN_trans_PnuC (TIGR01528)→
env-1624	OGCV01001779.1	+	1194	1296	RNA→ PnuC (COG3201)NMN_trans_PnuC (TIGR01528)→
env-1625	OGCW01004549.1	-	1001	899	RNA→ PnuC (COG3201)NMN_trans_PnuC (TIGR01528)→
env-1626	OGCZ01002152.1	-	1578	1476	RNA→ PnuC (COG3201)NMN_trans_PnuC (TIGR01528)→ hypo→
env-1627	OGDQ01003658.1	-	2422	2320	RNA→ PnuC (COG3201)NMN_trans_PnuC (TIGR01528)→
env-1628	OGDY01012121.1	+	917	1019	RNA→ PnuC (COG3201)NMN_trans_PnuC (TIGR01528)→ hypo→ hypo→<br>ribD_Cterm (TIGR00227)FolA (COG0262)→
env-1629	OGEA01002749.1	-	2523	2421	RNA→ PnuC (COG3201)NMN_trans_PnuC (TIGR01528)→
env-1630	OGEB01002261.1	+	4761	4863	RNA→ PnuC (COG3201)NMN_trans_PnuC (TIGR01528)→
env-1631	OGEK01000960.1	+	22702	22804	RNA→ PnuC (COG3201)NMN_trans_PnuC (TIGR01528)→
env-1632	OGEM01005470.1	-	2120	2018	RNA→ PnuC (COG3201)NMN_trans_PnuC (TIGR01528)→
env-1633	OGEP01006619.1	-	2957	2855	RNA→ PnuC (COG3201)NMN_trans_PnuC (TIGR01528)→

env-1634	OGEQ01005190.1	+	2866	2968	RNA→ PnuC (COG3201)NMN_trans_PnuC (TIGR01528)→
env-1635	OGES01004621.1	-	2756	2654	RNA→ PnuC (COG3201)NMN_trans_PnuC (TIGR01528)→
env-1636	OGFA01001068.1	-	2963	2861	RNA→ PnuC (COG3201)NMN_trans_PnuC (TIGR01528)→
env-1637	OGFB01001219.1	-	1743	1641	RNA→ PnuC (COG3201)NMN_trans_PnuC (TIGR01528)→ SRPBCC_6 (cd07824)Polyketide_cyc2 (pfam10604)– TfoX (COG3070)→
env-1638	OGFF01010868.1	+	1052	1154	RNA→ PnuC (COG3201)NMN_trans_PnuC (TIGR01528)→
env-1639	OGFM01002523.1	-	934	832	RNA→ PnuC (COG3201)NMN_trans_PnuC (TIGR01528)→
env-1640	OGFR01003202.1	-	3475	3373	RNA→ PnuC (COG3201)NMN_trans_PnuC (TIGR01528)→
env-1641	OGFU01000757.1	-	1163	1061	RNA→ PnuC (COG3201)NMN_trans_PnuC (TIGR01528)→ hypo→
env-1642	OGGB01035438.1	+	575	677	RNA→ hypo→
env-1643	OGGD01004682.1	-	3492	3390	RNA→ PnuC (COG3201)NMN_trans_PnuC (TIGR01528)→
env-1644	OGGH01014707.1	+	917	1019	RNA→ PnuC (COG3201)NMN_trans_PnuC (TIGR01528)→
env-1645	OGGL01000429.1	-	9119	9017	RNA→ PnuC (COG3201)NMN_trans_PnuC (TIGR01528)→ hypo→ ArdA (pfam07275)→ ArdA (pfam07275)→
env-1646	OGGQ01018359.1	+	917	1019	RNA→ PnuC (COG3201)NMN_trans_PnuC (TIGR01528)→
env-1647	OGGU01001220.1	-	5627	5525	RNA→ PnuC (COG3201)NMN_trans_PnuC (TIGR01528)→ hypo→ hypo→
env-1648	OGGW01003370.1	+	744	846	RNA→ PnuC (COG3201)NMN_trans_PnuC (TIGR01528)→
env-1649	OGGZ01012443.1	-	1658	1556	RNA→ PnuC (COG3201)NMN_trans_PnuC (TIGR01528)→
env-1650	OGHB01001227.1	-	2422	2320	RNA→ PnuC (COG3201)NMN_trans_PnuC (TIGR01528)→
env-1651	OGHE01007671.1	+	2238	2340	RNA→ PnuC (COG3201)NMN_trans_PnuC (TIGR01528)→
env-1652	OGHJ01010609.1	-	2776	2674	RNA→ PnuC (COG3201)NMN_trans_PnuC (TIGR01528)→
env-1653	OGHO01003668.1	+	917	1019	RNA→ TolA (COG3064)PnuC (COG3201)NMN_trans_PnuC (TIGR01528)tolA_full (TIGR02794)→
env-1654	OGHP01000696.1	-	5205	5103	RNA→ PnuC (COG3201)NMN_trans_PnuC (TIGR01528)→
env-1655	OGHT01000663.1	-	2422	2320	RNA→ PnuC (COG3201)NMN_trans_PnuC (TIGR01528)→
env-1656	OGIH01007996.1	+	971	1073	RNA→ PnuC (COG3201)NMN_trans_PnuC (TIGR01528)→
env-1657	OGII01010601.1	+	714	816	RNA→ PnuC (COG3201)NMN_trans_PnuC (TIGR01528)→ hypo→ hypo→ ribD_Cterm (TIGR00227)FolA (COG0262)→
env-1658	OGIK01005718.1	-	959	857	RNA→ PnuC (COG3201)NMN_trans_PnuC (TIGR01528)→
env-1659	OGIM01001317.1	-	2419	2317	RNA→ PnuC (COG3201)NMN_trans_PnuC (TIGR01528)→
env-1660	OGIR01050648.1	+	252	354	RNA→ PnuC (COG3201)→
env-1661	OGJB01000328.1	-	6006	5904	RNA→ PnuC (COG3201)NMN_trans_PnuC (TIGR01528)→
env-1662	OGJH01012092.1	+	1404	1506	RNA→ PnuC (COG3201)NMN_trans_PnuC (TIGR01528)→
env-1663	OGJI01010997.1	+	754	856	RNA→ PnuC (COG3201)NMN_trans_PnuC (TIGR01528)→ hypo→ DUF2207 (pfam09972)→
env-1664	OGJP01000925.1	+	3793	3895	RNA→ PnuC (COG3201)NMN_trans_PnuC (TIGR01528)→
env-1665	OGJV01005666.1	-	1765	1663	RNA→ PnuC (COG3201)NMN_trans_PnuC (TIGR01528)→
env-1666	OGKE01003754.1	-	3090	2988	RNA→ PnuC (COG3201)NMN_trans_PnuC (TIGR01528)→
env-1667	OGKG01015418.1	-	2468	2366	RNA→ PnuC (COG3201)NMN_trans_PnuC (TIGR01528)→
env-1668	OGKH01004766.1	+	753	855	RNA→ PnuC (COG3201)NMN_trans_PnuC (TIGR01528)→
env-1669	OGKX01004139.1	-	2175	2073	RNA→ PnuC (COG3201)→ NMN_transporter (pfam04973)→
env-1670	OGLB01006020.1	-	2468	2366	RNA→ PnuC (COG3201)NMN_trans_PnuC (TIGR01528)→
env-1671	OGMF01009048.1	-	4487	4385	RNA→ PnuC (COG3201)NMN_trans_PnuC (TIGR01528)→
env-1672	OGMP01005687.1	+	2866	2968	RNA→ PnuC (COG3201)NMN_trans_PnuC (TIGR01528)→ SRPBCC_6 (cd07824)Polyketide_cyc2 (pfam10604)– TfoX (COG3070)→
env-1673	OGMS01000143.1	-	917	815	RNA→ PnuC (COG3201)NMN_trans_PnuC (TIGR01528)→
env-1674	OGNG01003641.1	-	995	893	RNA→ PnuC (COG3201)NMN_trans_PnuC (TIGR01528)→
env-1675	OGNU01002425.1	-	2422	2320	RNA→ PnuC (COG3201)NMN_trans_PnuC (TIGR01528)→
env-1676	OGNZ01005937.1	-	824	722	RNA→ PnuC (COG3201)NMN_trans_PnuC (TIGR01528)→
env-1677	OGUF01000531.1	-	7188	7086	RNA→ TolA (COG3064)PnuC (COG3201)NMN_trans_PnuC (TIGR01528)tolA_full (TIGR02794)→
env-1678	OGUQ01007068.1	-	985	883	RNA→ PnuC (COG3201)NMN_trans_PnuC (TIGR01528)→ hypo→
env-1679	OIZE01005736.1	-	1893	1791	RNA→ PnuC (COG3201)NMN_trans_PnuC (TIGR01528)→ hypo→ DUF523 (pfam04463)→ hypo→
env-1680	Ga0134417_150032	+	119	221	RNA→ hypo→
env-1681	Pasolli2019-5080-34	+	1078	1180	RNA→ PnuC (COG3201)NMN_trans_PnuC (TIGR01528)→
env-1682	NLM004_scaffold54727_1	+	1603	1705	RNA→ PnuC (COG3201)NMN_trans_PnuC (TIGR01528)→
env-1683	OGIO01006896.1	+	520	622	RNA→ PnuC (COG3201)NMN_trans_PnuC (TIGR01528)→

env-1684	OGMZ01003797.1	+	1366	1468	**RNA**→ PnuC (COG3201)NMN_trans_PnuC (TIGR01528)→
env-1685	OGUD01003413.1	+	111	213	**RNA**→ PnuC (COG3201)NMN_trans_PnuC (TIGR01528)→ hypo→ hypo→
env-1686	OGUJ01003289.1	-	3927	3825	**RNA**→ PnuC (COG3201)NMN_trans_PnuC (TIGR01528)→
env-1687	OIXL01000617.1	-	13174	13072	**RNA**→ PnuC (COG3201)NMN_trans_PnuC (TIGR01528)→
env-1688	OIXT01000006.1	+	2958	3060	**RNA**→ PnuC (COG3201)NMN_trans_PnuC (TIGR01528)→
env-1689	OGGX01005200.1	-	150	48	**RNA**→
env-1690	OGKB01021459.1	-	281	179	**RNA**→ PnuC (COG3201)→
env-1691	OGMQ01051617.1	-	164	62	**RNA**→
env-1692	OLGO01029303.1	-	1097	995	**RNA**→ PnuC (COG3201)NMN_trans_PnuC (TIGR01528)→
env-1693	Ga0129313_1001214	+	15235	15337	**RNA**→ PnuC (COG3201)NMN_trans_PnuC (TIGR01528)→
env-1694	Ga0129312_1016229	+	958	1060	**RNA**→ PnuC (COG3201)NMN_trans_PnuC (TIGR01528)→
env-1695	OGIQ01045534.1	+	103	205	**RNA**→ PnuC (COG3201)→
env-1696	OGJO01012264.1	+	917	1019	**RNA**→ PnuC (COG3201)→
env-1697	OGLV01003142.1	+	754	856	**RNA**→ PnuC (COG3201)NMN_trans_PnuC (TIGR01528)→
env-1698	OIWO01007600.1	-	2756	2654	**RNA**→ PnuC (COG3201)NMN_trans_PnuC (TIGR01528)→
env-1699	OIWT01004850.1	-	6066	5964	**RNA**→ PnuC (COG3201)NMN_trans_PnuC (TIGR01528)→
env-1700	OIYD01002090.1	-	2473	2371	**RNA**→ PnuC (COG3201)NMN_trans_PnuC (TIGR01528)→
env-1701	OIZP01003789.1	+	4907	5009	**RNA**→ PnuC (COG3201)NMN_trans_PnuC (TIGR01528)→
env-1702	OLFS01008167.1	-	2320	2218	**RNA**→ PnuC (COG3201)NMN_trans_PnuC (TIGR01528)→
env-1703	OLHE01020894.1	-	1154	1052	**RNA**→ PnuC (COG3201)NMN_trans_PnuC (TIGR01528)→
env-1704	PPYF01025066.1	+	4125	4227	**RNA**→ PnuC (COG3201)NMN_trans_PnuC (TIGR01528)→
env-1705	Ga0134568_104288	-	899	797	**RNA**→ PnuC (COG3201)NMN_trans_PnuC (TIGR01528)→
env-1706	OGLC01020552.1	-	219	117	**RNA**→
env-1707	OGNV01043137.1	+	509	611	**RNA**→
env-1708	Ga0129314_1048098	+	209	311	**RNA**→ PnuC (COG3201)NMN_trans_PnuC (TIGR01528)→
env-1709	Ga0121387_100429	+	6558	6660	**RNA**→ PnuC (COG3201)NMN_trans_PnuC (TIGR01528)→
env-1710	SRS013687_Baylor_scaffold_56707	+	520	622	**RNA**→ PnuC (COG3201)NMN_trans_PnuC (TIGR01528)→
env-1711	SRS048164_WUGC_scaffold_3278	-	2425	2323	**RNA**→ PnuC (COG3201)NMN_trans_PnuC (TIGR01528)→
env-1712	Ga0169873_100254	-	43919	43817	**RNA**→ PnuC (COG3201)NMN_trans_PnuC (TIGR01528)→
env-1713	Pasolli2019-4756-31	-	30880	30778	**RNA**→ PnuC (COG3201)NMN_trans_PnuC (TIGR01528)→
env-1714	UMGS275-42	+	8887	8989	**RNA**→ PnuC (COG3201)NMN_trans_PnuC (TIGR01528)→
Rfl-2-1	NZ_KI912493.1	+	22882	22984	**RNA**→ PnuC (COG3201)NMN_trans_PnuC (TIGR01528)→ HTH_YyaN (cd01109)→<br>BDH (cd08187)PRK15138 (PRK15138)→ dkgA (PRK11565)ARA1 (COG0656)→<br>PRK07116 (PRK07116)YgiN (COG1359)Flavodoxin_2 (pfam02525)→ PRK07116 (PRK07116)FldA (COG0716)→<br>decarb_PcaC (TIGR02425)YurZ (COG0599)QdoI (COG1917)Cupin_2 (pfam07883)→<br>Aldo_ket_red (cd06660)dkgA (PRK11565)PreA (COG1146)Fer4_17 (pfam13534)→ ECF_trnsprt (pfam12822)→<br>TM1506 (pfam08973)→
env-1715	Ga0209606_1020494	+	319	421	**RNA**→ PnuC (COG3201)NMN_trans_PnuC (TIGR01528)→
env-1716	Ga0179936_1177460	-	992	890	**RNA**→ PnuC (COG3201)NMN_trans_PnuC (TIGR01528)→
env-1717	Ga0179934_1037753	+	1325	1427	**RNA**→ hypo→
env-1718	Ga0209201_1140996	+	12	114	**RNA**→ PnuC (COG3201)NMN_trans_PnuC (TIGR01528)→
env-1719	DFWH01000008.1	-	112738	112637	**RNA**→ PnuC (COG3201)NMN_trans_PnuC (TIGR01528)→
env-1720	Ga0121531_100080	-	13250	13149	**RNA**→ PnuC (COG3201)NMN_trans_PnuC (TIGR01528)→
Oru-1-1	NZ_DF158897.1	-	256142	256041	**RNA**→ PnuC (COG3201)NMN_trans_PnuC (TIGR01528)→
env-1721	Pasolli2019-19227-0	+	1370	1471	**RNA**→ PnuC (COG3201)NMN_trans_PnuC (TIGR01528)→
env-1722	DKDS01000049.1	-	2363	2261	**RNA**→ PnuC (COG3201)NMN_trans_PnuC (TIGR01528)→
env-1723	scaffold2300_7_MH0040	-	5729	5628	**RNA**→ PnuC (COG3201)NMN_trans_PnuC (TIGR01528)→
env-1724	OIXH01029578.1	+	1957	2058	**RNA**→
env-1725	Pasolli2019-4407-49	+	3613	3714	**RNA**→ PnuC (COG3201)NMN_trans_PnuC (TIGR01528)→
env-1726	Ga0179940_1006214	+	325	425	**RNA**→
env-1727	AUXO017419468.1	+	1213	1314	**RNA**→ PnuC (COG3201)NMN_trans_PnuC (TIGR01528)→ hypo→ hypo→
env-1728	DETB01000025.1	-	25803	25702	**RNA**→ PnuC (COG3201)NMN_trans_PnuC (TIGR01528)→
Ral-1-1	NZ_JEOB01000002.1	-	1268540	1268439	**RNA**→ PnuC (COG3201)NMN_trans_PnuC (TIGR01528)→

env-1729	Ga0129317_1021849	-	779	677	**RNA**→ hypo→
Rgn-1-1	NZ_JAGQ01000002.1	-	297151	297049	**RNA**→ hypo→
Gni-1-1	NZ_FUWV01000009.1	-	56450	56350	**RNA**→ HTH_ARSR (smart00418)HTH_ARSR (cd00090)→ YraQ (COG0701)feoB (TIGR00437)→
env-1730	4491417.3_NODE_3891	-	9463	9362	**RNA**→ PnuC (COG3201)NMN_trans_PnuC (TIGR01528)→ NimA (COG3467)Pyridox_ox_2 (pfam12900)→
env-1731	Ga0172378_12030478	+	211	313	**RNA**→
env-1732	UnmappedStool_Broad_scaffold_985897	+	178	279	**RNA**→ PnuC (COG3201)NMN_trans_PnuC (TIGR01528)→ NimA (COG3467)Pyridox_ox_2 (pfam12900)→
env-1733	Pasolli2019-14151-52	-	4784	4683	**RNA**→ PnuC (COG3201)NMN_trans_PnuC (TIGR01528)→ NimA (COG3467)Pyridox_ox_2 (pfam12900)→ Cys_rich_KTR (pfam14205)→
env-1734	SRS011239_C2665093	+	153	254	**RNA**→ PnuC (COG3201)NMN_trans_PnuC (TIGR01528)→ NimA (COG3467)Pyridox_ox_2 (pfam12900)→
env-1735	SRS012273_C3758230	+	178	279	**RNA**→ PnuC (COG3201)NMN_trans_PnuC (TIGR01528)→ NimA (COG3467)Pyridox_ox_2 (pfam12900)→
env-1736	SRS013476_C3114979	-	1658	1557	**RNA**→ PnuC (COG3201)NMN_trans_PnuC (TIGR01528)→ NimA (COG3467)Pyridox_ox_2 (pfam12900)→
env-1737	SRS014979_WUGC_scaffold_35936	-	1691	1590	**RNA**→ PnuC (COG3201)NMN_trans_PnuC (TIGR01528)→ NimA (COG3467)Pyridox_ox_2 (pfam12900)→
env-1738	SRS042628_C2735101	-	1659	1558	**RNA**→ PnuC (COG3201)NMN_trans_PnuC (TIGR01528)→ NimA (COG3467)Pyridox_ox_2 (pfam12900)→
env-1739	SRS048870_WUGC_scaffold_51463	+	6463	6564	**RNA**→ PnuC (COG3201)NMN_trans_PnuC (TIGR01528)→ NimA (COG3467)Pyridox_ox_2 (pfam12900)→
env-1740	DOCC01000246.1	+	10073	10175	**RNA**→ PnuC (COG3201)NMN_trans_PnuC (TIGR01528)→
env-1741	Pasolli2019-4321-0	-	12267	12165	**RNA**→ PnuC (COG3201)NMN_trans_PnuC (TIGR01528)→
env-1742	Ga0134540_1004427	-	1019	917	**RNA**→ PnuC (COG3201)NMN_trans_PnuC (TIGR01528)→
env-1743	UMGS1088-1	-	36169	36067	**RNA**→ PnuC (COG3201)NMN_trans_PnuC (TIGR01528)→
env-1744	Pasolli2019-4151-115	+	2137	2239	**RNA**→ PnuC (COG3201)NMN_trans_PnuC (TIGR01528)→
env-1745	DBOQ01000035.1	-	16504	16403	**RNA**→ PnuC (COG3201)NMN_trans_PnuC (TIGR01528)→
env-1746	ONGR01000031.1	-	7471	7369	**RNA**→ PnuC (COG3201)NMN_trans_PnuC (TIGR01528)→
env-1747	Ga0129313_1033024	-	576	477	**RNA**→ PnuC (COG3201)NMN_trans_PnuC (TIGR01528)→
env-1748	Ga0129312_1087486	+	96	195	**RNA**→ PnuC (COG3201)→
env-1749	Pasolli2019-14112-19	-	1353	1255	**RNA**→ PnuC (COG3201)NMN_trans_PnuC (TIGR01528)→
env-1750	Ga0134523_1002817	-	3054	2956	**RNA**→ PnuC (COG3201)NMN_trans_PnuC (TIGR01528)→
env-1751	AUXO017873386.1	+	22	122	**RNA**→
env-1752	Pasolli2019-14185-18	+	9333	9437	**RNA**→ hypo→ hypo→
env-1753	UMGS1225-2	-	26419	26315	**RNA**→ ←Cps2a (COG1316)lytR_cpsA_psr (TIGR00350)
env-1754	Ga0129306_1001351	-	5526	5424	**RNA**→ B3_4 (smart00873)B3/B4 (COG3382)→
env-1755	Ga0129307_1001323	+	22688	22790	**RNA**→ B3_4 (smart00873)B3/B4 (COG3382)→
env-1756	Ga0122774_106203	-	600	498	**RNA**→ PnuC (COG3201)NMN_trans_PnuC (TIGR01528)→
env-1757	AUXO014726451.1	+	2285	2378	**RNA**→ PnuC (COG3201)NMN_trans_PnuC (TIGR01528)→ CBM35_mannanase-like (cd04086)Dockerin_I (cd14256)CBM_35 (pfam16990)Glyco_hydro_26 (pfam02156)→
env-1758	DJXD01000053.1	+	126216	126309	**RNA**→ PnuC (COG3201)NMN_trans_PnuC (TIGR01528)→ CBM35_mannanase-like (cd04086)Dockerin_I (cd14256)CBM_35 (pfam16990)Glyco_hydro_26 (pfam02156)→
env-1759	AUXO018506628.1	-	2515	2413	**RNA**→ B3_4 (smart00873)B3/B4 (COG3382)→
env-1760	DGGP01000081.1	-	4089	3987	**RNA**→ B3_4 (smart00873)B3/B4 (COG3382)→
env-1761	AUXO016731424.1	+	244	345	**RNA**→
env-1762	Pasolli2019-14232-6	+	9945	10046	**RNA**→ hypo→
env-1763	scaffold98082_1_MH0081	+	392	493	**RNA**→ hypo→
env-1764	AUXO017836397.1	+	737	839	**RNA**→ NMN_trans_PnuC (TIGR01528)NMN_transporter (pfam04973)→
env-1765	OLGF01007940.1	+	2118	2222	**RNA**→ PnuC (COG3201)NMN_trans_PnuC (TIGR01528)→
env-1766	SRS011302_C3355131	+	727	831	**RNA**→ hypo→
env-1767	SRS020328_Baylor_scaffold_32008	-	825	721	**RNA**→ PnuC (COG3201)NMN_trans_PnuC (TIGR01528)→
env-1768	AUXO015493828.1	+	105	206	**RNA**→ PnuC (COG3201)NMN_trans_PnuC (TIGR01528)→
env-1769	4491409.3_NODE_31037	-	897	798	**RNA**→ PnuC (COG3201)NMN_trans_PnuC (TIGR01528)→
env-1770	OGJJ01000272.1	-	4721	4622	**RNA**→ DinP (COG0389)IMS_C (pfam11799)→ YolD (pfam08863)→ hypo→ PRK10734 (PRK10734)ECM27 (COG0530)→
env-1771	Pasolli2019-4548-3	-	111784	111684	**RNA**→ (big gap) ←hypo
env-1772	OIXH01033199.1	+	1027	1130	**RNA**→ PnuC (COG3201)NMN_trans_PnuC (TIGR01528)→
env-1773	UMGS1771-78	-	5571	5470	**RNA**→ PnuC (COG3201)NMN_trans_PnuC (TIGR01528)→
env-1774	OIZX01008007.1	+	1	99	**RNA**→ ←Rve (COG3316)Zn_Tnp_IS1 (pfam03811)DDE_Tnp_IS240 (pfam13610)
env-1775	DKYJ01000046.1	+	46555	46654	**RNA**→ PnuC (COG3201)NMN_trans_PnuC (TIGR01528)→

env-1776	Pasolli2019-15005-0	+	59059	59158	**RNA**→ PnuC (COG3201)NMN_trans_PnuC (TIGR01528)→ DNA_binding_1 (pfam01035)→
env-1777	UMGS1206-0	+	89043	89142	**RNA**→ PnuC (COG3201)NMN_trans_PnuC (TIGR01528)→ DNA_binding_1 (pfam01035)→
env-1778	Ga0172382_10006886	+	15979	16079	**RNA**→ NMN_transporter (pfam04973)→
env-1779	Ga0116619_1096693	+	56	157	**RNA**→ vmrA (PRK09575)MATE_MepA_like (cd13143)→
env-1780	Ga0129306_1114541	+	265	366	**RNA**→
Rsp-3-1	NZ_JMMA01000002.1	-	2856223	2856124	**RNA**→ PnuC (COG3201)NMN_trans_PnuC (TIGR01528)→ hypo→ hypo→
env-1781	ASRN01001145.1	-	1422	1319	**RNA**→ hypo→ PnuC (COG3201)NMN_trans_PnuC (TIGR01528)→
env-1782	ASRO01000142.1	-	2587	2484	**RNA**→ hypo→ PnuC (COG3201)NMN_trans_PnuC (TIGR01528)→ RpsD (COG0522)rpsD (PRK05327)→
env-1783	ASRQ01000004.1	-	89976	89873	**RNA**→ hypo→ PnuC (COG3201)NMN_trans_PnuC (TIGR01528)→ RpsD (COG0522)rpsD (PRK05327)→
env-1784	ASRR01001748.1	+	165	268	**RNA**→ PnuC (COG3201)NMN_trans_PnuC (TIGR01528)→ hypo→
env-1785	AYRG01000160.1	+	12928	13031	**RNA**→ PnuC (COG3201)NMN_trans_PnuC (TIGR01528)→ RpsD (COG0522)rpsD (PRK05327)→
env-1786	DBSM01000043.1	-	2547	2444	**RNA**→ PnuC (COG3201)NMN_trans_PnuC (TIGR01528)→ RpsD (COG0522)rpsD (PRK05327)→
env-1787	DBSN01000066.1	-	7391	7288	**RNA**→ PnuC (COG3201)NMN_trans_PnuC (TIGR01528)→ RpsD (COG0522)rpsD (PRK05327)→
env-1788	DGAD01000041.1	-	2548	2445	**RNA**→ PnuC (COG3201)NMN_trans_PnuC (TIGR01528)→ RpsD (COG0522)rpsD (PRK05327)→
env-1789	JGI12365J12839_1000011	+	52190	52293	**RNA**→ PnuC (COG3201)NMN_trans_PnuC (TIGR01528)→
env-1790	JGI12573J12842_1000011	+	52199	52302	**RNA**→ PnuC (COG3201)NMN_trans_PnuC (TIGR01528)→
env-1791	JGI12287J12850_1000011	-	204861	204758	**RNA**→ PnuC (COG3201)NMN_trans_PnuC (TIGR01528)→
env-1792	ASRK01000262.1	+	2361	2463	**RNA**→ PnuC (COG3201)NMN_trans_PnuC (TIGR01528)→ RpsD (COG0522)rpsD (PRK05327)→
env-1793	DDQS01000002.1	-	48135	48033	**RNA**→ PnuC (COG3201)NMN_trans_PnuC (TIGR01528)→ RpsD (COG0522)rpsD (PRK05327)→
env-1794	AUXO010483333.1	+	726	827	**RNA**→ PnuC (COG3201)NMN_trans_PnuC (TIGR01528)→
env-1795	DFJW01000061.1	-	28057	27956	**RNA**→ PnuC (COG3201)NMN_trans_PnuC (TIGR01528)→
Rsp-2-1	NZ_JHXH01000004.1	+	156897	156996	**RNA**→ PnuC (COG3201)NMN_trans_PnuC (TIGR01528)→
env-1796	Ga0120384_1106142	+	384	484	**RNA**→ PnuC (COG3201)→
env-1797	Ga0256404_1003777	+	27263	27363	**RNA**→ PnuC (COG3201)NMN_trans_PnuC (TIGR01528)→
env-1798	ONJP01000006.1	-	30680	30580	**RNA**→ PnuC (COG3201)NMN_trans_PnuC (TIGR01528)→
env-1799	ONMZ01000005.1	+	26809	26909	**RNA**→ PnuC (COG3201)NMN_trans_PnuC (TIGR01528)→
env-1800	ONTE01000028.1	-	4636	4536	**RNA**→ PnuC (COG3201)NMN_trans_PnuC (TIGR01528)→
env-1801	AUXO013411786.1	-	2612	2512	**RNA**→ PnuC (COG3201)NMN_trans_PnuC (TIGR01528)→
env-1802	rank02_1001293	+	9000	9100	**RNA**→ PnuC (COG3201)NMN_trans_PnuC (TIGR01528)→
env-1803	4491686.3.1006660	-	156	56	**RNA**→ hypo→
env-1804	Pasolli2019-14198-1	+	115778	115878	**RNA**→ PnuC (COG3201)NMN_trans_PnuC (TIGR01528)→
env-1805	SRS063985_LANL_scaffold_38080	+	235	335	**RNA**→
env-1806	Ga0120384_1132356	+	83	184	**RNA**→ PnuC (COG3201)NMN_trans_PnuC (TIGR01528)→
env-1807	Ga0134540_1455609	+	50	151	**RNA**→ hypo→
env-1808	Pasolli2019-15151-201	-	2009	1907	**RNA**→ PnuC (COG3201)NMN_trans_PnuC (TIGR01528)→
env-1809	Ga0134523_1145212	-	164	62	**RNA**→
env-1810	Pasolli2019-15150-31	-	20970	20868	**RNA**→ PnuC (COG3201)NMN_trans_PnuC (TIGR01528)→
env-1811	Pasolli2019-15152-0	-	122704	122602	**RNA**→ PnuC (COG3201)NMN_trans_PnuC (TIGR01528)→
env-1812	Pasolli2019-14244-76	-	1442	1341	**RNA**→ PnuC (COG3201)NMN_trans_PnuC (TIGR01528)→
env-1813	Ga0172378_10116515	-	1457	1356	**RNA**→ PnuC (COG3201)NMN_trans_PnuC (TIGR01528)→
env-1814	Ga0172377_10911173	-	201	100	**RNA**→
env-1815	UnmappedStool_Broad_C253674136	-	1084	986	**RNA**→ PnuC (COG3201)NMN_trans_PnuC (TIGR01528)→
env-1816	NOF008_scaffold18065_24	+	1381	1480	**RNA**→ PnuC (COG3201)NMN_trans_PnuC (TIGR01528)→
env-1817	Pasolli2019-4265-5	+	32175	32274	**RNA**→ PnuC (COG3201)NMN_trans_PnuC (TIGR01528)→
env-1818	Pasolli2019-4424-47	+	8477	8579	**RNA**→ PnuC (COG3201)NMN_trans_PnuC (TIGR01528)→
env-1819	UMGS1903-222	-	738	636	**RNA**→ PnuC (COG3201)NMN_trans_PnuC (TIGR01528)→
env-1820	scaffold40924_2_V1.CD-12	-	765	666	**RNA**→ NimA (COG3467)Pyridox_ox_2 (pfam12900)→
env-1821	Ga0256405_10032937	+	1889	1990	**RNA**→ PnuC (COG3201)NMN_trans_PnuC (TIGR01528)→ GTP1 (COG0012)PRK09601 (PRK09601)→
env-1822	UMGS1469-13	+	14950	15050	**RNA**→ PnuC (COG3201)NMN_trans_PnuC (TIGR01528)→
env-1823	Pasolli2019-7254-179	+	4076	4176	**RNA**→ PnuC (COG3201)NMN_trans_PnuC (TIGR01528)→
env-1824	Pasolli2019-4138-110	+	2585	2686	**RNA**→ PnuC (COG3201)NMN_trans_PnuC (TIGR01528)→
env-1825	DKZC01000020.1	+	396	497	**RNA**→ PnuC (COG3201)NMN_trans_PnuC (TIGR01528)→

env-1826	DESG01000015.1	+	396	497	RNA→ PnuC (COG3201)NMN_trans_PnuC (TIGR01528)→
env-1827	DKUG01000039.1	+	396	497	RNA→ PnuC (COG3201)NMN_trans_PnuC (TIGR01528)→
env-1828	DKWL01000042.1	+	396	497	RNA→ PnuC (COG3201)NMN_trans_PnuC (TIGR01528)→
env-1829	DKXJ01000036.1	+	396	497	RNA→ PnuC (COG3201)NMN_trans_PnuC (TIGR01528)→
Dsp-1-1	NZ_KE159717.1	-	102723	102623	RNA→ HATPase_c (smart00387)HisKA (smart00388)BaeS (COG0642)→
env-1830	Ga0209064_1026319	+	44	144	RNA→ ←DDE_Tnp_1 (pfam01609)DDE_5 (pfam13546)
Lba-3-1	NZ_KE159636.1	-	195753	195653	RNA→ hypo→ hypo→ Pyridox_oxidase (pfam01243)Pyridox_ox_2 (pfam12900)→
env-1831	UMGS1396-39	+	6626	6728	RNA→ PnuC (COG3201)NMN_trans_PnuC (TIGR01528)→
env-1832	07_22_CONTROL_DNA_scaffold130101_1	+	3756	3856	RNA→ PnuC (COG3201)NMN_trans_PnuC (TIGR01528)→
env-1833	07_22_PECTIN_DNA_scaffold66459_9	+	606	706	RNA→ PnuC (COG3201)NMN_trans_PnuC (TIGR01528)→
env-1834	08_07_GOS_DNA_scaffold79300_8	+	418	518	RNA→ PnuC (COG3201)→
env-1835	08_19_CONTROL_DNA_scaffold79080_1	+	3756	3856	RNA→ PnuC (COG3201)NMN_trans_PnuC (TIGR01528)→
env-1836	O8_19_POLYDEXT_DNA_scaffold138457_4	+	2414	2514	RNA→ PnuC (COG3201)→
env-1837	Ga0129309_1024588	-	362	262	RNA→ ←hypo
env-1838	Pasolli2019-3580-45	+	5856	5956	RNA→ PnuC (COG3201)NMN_trans_PnuC (TIGR01528)→
env-1839	Pasolli2019-14933-16	-	8288	8187	RNA→ PnuC (COG3201)NMN_trans_PnuC (TIGR01528)→
env-1840	JGI24709J26583_10007031	+	4483	4583	RNA→ PnuC (COG3201)NMN_trans_PnuC (TIGR01528)→
env-1841	JGI24710J26742_10005476	-	5540	5440	RNA→ PnuC (COG3201)NMN_trans_PnuC (TIGR01528)→
env-1842	OLGB01016944.1	+	2244	2344	RNA→ PnuC (COG3201)NMN_trans_PnuC (TIGR01528)→
env-1843	Pasolli2019-4231-93	+	4014	4114	RNA→ PnuC (COG3201)NMN_trans_PnuC (TIGR01528)→
env-1844	SRS011405_C3518243	-	182	81	RNA→ hypo→
env-1845	Pasolli2019-4157-54	-	1698	1597	RNA→ NMN_transporter (pfam04973)→
env-1846	Ga0209694_1000392	+	14909	15010	RNA→ PnuC (COG3201)NMN_trans_PnuC (TIGR01528)→
env-1847	Ga0209064_1000233	-	60291	60190	RNA→ PnuC (COG3201)NMN_trans_PnuC (TIGR01528)→
env-1848	UMGS771-29	-	4572	4470	RNA→ PnuC (COG3201)NMN_trans_PnuC (TIGR01528)→
env-1849	UMGS1955-163	-	2469	2368	RNA→ PnuC (COG3201)NMN_trans_PnuC (TIGR01528)→
env-1850	DLQW01000010.1	+	4783	4883	RNA→ PnuC (COG3201)NMN_trans_PnuC (TIGR01528)→
env-1851	Ga0177923_1265960	+	47052	47151	RNA→ PnuC (COG3201)NMN_trans_PnuC (TIGR01528)→
env-1852	Ga0134523_1004786	-	445	344	RNA→ Pyridox_oxidase (pfam01243)Pyridox_ox_2 (pfam12900)→
env-1853	NLM031_scaffold44466_1	+	501	601	RNA→ Pyridox_oxidase (pfam01243)Pyridox_ox_2 (pfam12900)→
env-1854	OGEU01016476.1	-	684	584	RNA→ RplI (COG0359)rplI (PRK00137)Pyridox_oxidase (pfam01243)Pyridox_ox_2 (pfam12900)→
env-1855	OGFG01012389.1	+	2581	2681	RNA→ NimA (COG3467)Pyridox_ox_2 (pfam12900)→
env-1856	OGMF01022125.1	-	508	408	RNA→ Pyridox_oxidase (pfam01243)Pyridox_ox_2 (pfam12900)→
env-1857	OGLX01048382.1	+	242	342	RNA→ NimA (COG3467)Pyridox_ox_2 (pfam12900)→
env-1858	OLFU01055872.1	+	330	430	RNA→ Pyridox_oxidase (pfam01243)Pyridox_ox_2 (pfam12900)→
env-1859	SRS015065_C2311388	-	482	382	RNA→ Pyridox_oxidase (pfam01243)Pyridox_ox_2 (pfam12900)→
env-1860	Pasolli2019-4640-5	+	153306	153406	RNA→ hypo→
env-1861	DERX01000082.1	-	5432	5332	RNA→ PnuC (COG3201)NMN_trans_PnuC (TIGR01528)→
env-1862	Pasolli2019-14928-10	-	19337	19236	RNA→ PnuC (COG3201)NMN_trans_PnuC (TIGR01528)→
env-1863	UMGS1474-38	-	14944	14843	RNA→ PnuC (COG3201)NMN_trans_PnuC (TIGR01528)→
env-1864	Ga0209506_1164443	-	99	1	RNA→
env-1865	AUXO015745485.1	+	1141	1242	RNA→ PnuC (COG3201)NMN_trans_PnuC (TIGR01528)→ PRK03103 (PRK03103)IMS (pfam00817)→
env-1866	DFJO01000111.1	-	1741	1640	RNA→ PnuC (COG3201)NMN_trans_PnuC (TIGR01528)→ DinP (COG0389)PRK03103 (PRK03103)→
env-1867	Ga0256406_1091432	-	729	628	RNA→ PnuC (COG3201)NMN_trans_PnuC (TIGR01528)→
env-1868	Ga0256405_10056959	+	2046	2147	RNA→ PnuC (COG3201)NMN_trans_PnuC (TIGR01528)→<br>PolY_Pol_V_umuC (cd01700)PRK03103 (PRK03103)→
env-1869	DFHF01000037.1	+	97459	97560	RNA→ PnuC (COG3201)NMN_trans_PnuC (TIGR01528)→ DinP (COG0389)IMS_C (pfam11799)→<br>YolD (pfam08863)→
env-1870	Pasolli2019-14226-8	+	1723	1826	RNA→ PnuC (COG3201)NMN_trans_PnuC (TIGR01528)→ DUF1275 (pfam06912)→
env-1871	Ga0209075_1237601	-	100	1	RNA→
Ssu-6-1	NZ_ASBW01000158.1	+	4795	4894	RNA→ B3_4 (smart00873)B3/B4 (COG3382)→ hypo→ hypo→ hypo→ hypo→<br>glyQ (PRK09348)tRNA-synt_2e (pfam02091)→ hypo→ GlyS (COG0751)glyS (PRK01233)→<br>PRK02539 (PRK02539)YnzC (COG4224)→

Ssu-5-1	NZ_ASBV01000028.1	-	5892	5793	RNA→ B3_4 (smart00873)B3/B4 (COG3382)→ hypo→ hypo→ hypo→ hypo→ glyQ (PRK09348)tRNA-synt_2e (pfam02091)→ hypo→ GlyS (COG0751)glyS (PRK01233)→ PRK02539 (PRK02539)YnzC (COG4224)→
Ssu-3-1	NZ_ASBS01000007.1	+	7930	8029	RNA→ B3_4 (smart00873)B3/B4 (COG3382)→ hypo→ hypo→ hypo→ hypo→ glyQ (PRK09348)tRNA-synt_2e (pfam02091)→ hypo→ GlyS (COG0751)glyS (PRK01233)→ PRK02539 (PRK02539)YnzC (COG4224)→
Ssu-4-1	NZ_ASBT01000001.1	+	7930	8029	RNA→ B3_4 (smart00873)B3/B4 (COG3382)→ hypo→ hypo→ hypo→ hypo→ glyQ (PRK09348)tRNA-synt_2e (pfam02091)→ hypo→ GlyS (COG0751)glyS (PRK01233)→ PRK02539 (PRK02539)YnzC (COG4224)→
Ssu-1-1	NZ_POJI01000060.1	-	52005	51906	RNA→ B3_4 (smart00873)B3/B4 (COG3382)→ hypo→ YbcV (COG5562)→ glyQ (PRK09348)tRNA-synt_2e (pfam02091)→ GlyS (COG0751)glyS (PRK01233)→ PRK02539 (PRK02539)YnzC (COG4224)→
Ssu-1-2	NZ_PONQ01000295.1	+	18004	18103	RNA→ B3_4 (smart00873)B3/B4 (COG3382)→ hypo→ YbcV (COG5562)→ glyQ (PRK09348)tRNA-synt_2e (pfam02091)→ GlyS (COG0751)glyS (PRK01233)→ PRK02539 (PRK02539)YnzC (COG4224)→
Ssu-7-1	NZ_ALMX01000107.1	+	4675	4774	RNA→ B3_4 (smart00873)B3/B4 (COG3382)→ hypo→ YbcV (COG5562)→ glyQ (PRK09348)tRNA-synt_2e (pfam02091)→ GlyS (COG0751)glyS (PRK01233)→ PRK02539 (PRK02539)YnzC (COG4224)→ S2P-M50 (cd05709)→ hypo→
Ssu-8-1	NZ_ALNH01000024.1	-	4956	4857	RNA→ B3_4 (smart00873)B3/B4 (COG3382)→ hypo→ YbcV (COG5562)→ glyQ (PRK09348)tRNA-synt_2e (pfam02091)→ GlyS (COG0751)glyS (PRK01233)→ PRK02539 (PRK02539)YnzC (COG4224)→
Ssu-2-1	NZ_ASBO01000157.1	+	4818	4917	RNA→ hypo→ hypo→ YbcV (COG5562)→ glyQ (PRK09348)tRNA-synt_2e (pfam02091)→ GlyS (COG0751)glyS (PRK01233)→ PRK02539 (PRK02539)YnzC (COG4224)→ S2P-M50 (cd05709)→ hypo→
Ssu-1-3	NZ_POJH01000031.1	-	42473	42374	RNA→ hypo→ hypo→
Ssu-1-4	NZ_POJF01000012.1	-	157620	157521	RNA→ B3_4 (smart00873)B3/B4 (COG3382)→ hypo→ YbcV (COG5562)→
Ssu-1-5	NZ_PONK01000104.1	-	5779	5680	RNA→ B3_4 (smart00873)B3/B4 (COG3382)→ hypo→ YbcV (COG5562)→
Ssu-1-6	NZ_PONL01000038.1	-	5779	5680	RNA→ B3_4 (smart00873)B3/B4 (COG3382)→ hypo→ YbcV (COG5562)→
Ssu-1-7	NZ_POIF01000452.1	+	44241	44340	RNA→ B3_4 (smart00873)B3/B4 (COG3382)→ hypo→ glyQ (PRK09348)tRNA-synt_2e (pfam02091)→ GlyS (COG0751)glyS (PRK01233)→ PRK02539 (PRK02539)YnzC (COG4224)→ S2P-M50 (cd05709)→ hypo→
Ssu-1-8	NZ_POIG01000471.1	-	217	118	RNA→
env-1872	Ga0209694_1037457	+	243	345	RNA→ PnuC (COG3201)NMN_trans_PnuC (TIGR01528)→
env-1873	Pasolli2019-4726-6	+	9096	9191	RNA→ MATE_MepA_like (cd13143)matE (TIGR00797)→
env-1874	UMGS1839-6	+	9096	9191	RNA→ MATE_MepA_like (cd13143)matE (TIGR00797)→
env-1875	Ga0129317_1081817	+	298	396	RNA→ hypo→
env-1876	DEVR01000003.1	+	44412	44516	RNA→ vmrA (PRK09575)MATE_MepA_like (cd13143)→
env-1877	DDAM01000070.1	+	30189	30295	RNA→ vmrA (PRK09575)MATE_MepA_like (cd13143)→
env-1878	Pasolli2019-15118-61	+	11462	11552	RNA→ MATE_MepA_like (cd13143)→
env-1879	Pasolli2019-4604-14	+	39955	40043	RNA→ NimA (COG3467)Pyridox_ox_2 (pfam12900)→ MATE_MepA_like (cd13143)matE (TIGR00797)→
env-1880	DCEY01000166.1	-	1069	981	RNA→ Pyridox_oxidase (pfam01243)Pyridox_ox_2 (pfam12900)→ NorM (COG0534)vmrA (PRK09575)→
env-1881	Pasolli2019-4802-100	+	10235	10323	RNA→ NimA (COG3467)Pyridox_ox_2 (pfam12900)→
env-1882	Pasolli2019-4801-11	-	56808	56720	RNA→ MATE_MepA_like (cd13143)matE (TIGR00797)→
env-1883	UMGS604-13	+	231	319	RNA→ MATE_MepA_like (cd13143)matE (TIGR00797)→
env-1884	4491423.3_NODE_70844	+	319	408	RNA→ NimA (COG3467)Pyridox_ox_2 (pfam12900)→
env-1885	DBQZ01000034.1	+	14931	15020	RNA→ hypo→
env-1886	Pasolli2019-14931-11	+	49834	49924	RNA→ NimA (COG3467)Pyridox_ox_2 (pfam12900)→ hypo→
env-1887	SRS017821_C2242077	-	138	48	RNA→
env-1888	Pasolli2019-3951-4	-	7928	7838	RNA→ hypo→
env-1889	SRS011302_C3270899	-	463	373	RNA→ Pyridox_oxidase (pfam01243)Pyridox_ox_2 (pfam12900)→
env-1890	Pasolli2019-14194-172	+	2909	2999	RNA→ NimA (COG3467)Pyridox_ox_2 (pfam12900)→
env-1891	UMGS1037-39	+	11774	11863	RNA→ hypo→
env-1892	Pasolli2019-14249-87	+	3509	3599	RNA→ MATE_MepA_like (cd13143)matE (TIGR00797)→ NorM (COG0534)matE (TIGR00797)→
env-1893	Pasolli2019-6791-9	+	87428	87521	RNA→ MATE_MepA_like (cd13143)matE (TIGR00797)→
env-1894	UMGS1140-32	-	3253	3160	RNA→ MATE_MepA_like (cd13143)matE (TIGR00797)→
env-1895	4448808.3_Contig34458	-	291	198	RNA→ vmrA (PRK09575)MATE_MepA_like (cd13143)→

env-1896	SRS012273_C3532464	+	136	235	RNA→ hypo→
env-1897	Ga0172382_11317932	+	161	261	RNA→ vmrA (PRK09575)MATE_MepA_like (cd13143)→
env-1898	Pasolli2019-13992-1	+	232171	232272	RNA→ PnuC (COG3201)NMN_trans_PnuC (TIGR01528)→
env-1899	4491406.3_NODE_12099	+	9838	9929	RNA→ hypo→
env-1900	Ga0177923_1333981	+	266	355	RNA→
env-1901	Pasolli2019-14144-87	-	5471	5382	RNA→ NimA (COG3467)Pyridox_ox_2 (pfam12900)→
env-1902	HCF12C_358208	+	473	562	RNA→ PnuC (COG3201)NMN_trans_PnuC (TIGR01528)→
env-1903	OLGO01027006.1	-	719	621	RNA→ NorM (COG0534)vmrA (PRK09575)→
env-1904	Ga0169827_10021	+	23178	23278	RNA→ vmrA (PRK09575)MATE_MepA_like (cd13143)→
Cbo-2-2	NZ_JUWA01000161.1	+	6268	6368	RNA→ vmrA (PRK09575)MATE_MepA_like (cd13143)→
Cpa-1-1	NZ_OBIQ01000020.1	-	3899	3799	RNA→ vmrA (PRK09575)MATE_MepA_like (cd13143)→
Par-1-1	NZ_JQCQ01000020.1	-	18876	18782	RNA→ PRK11431 (PRK11431)EmrE (COG2076)→
env-1905	Ga0209694_1157506	-	351	247	RNA→ NMN_transporter (pfam04973)→
env-1906	Ga0209064_1052213	-	863	759	RNA→ PnuC (COG3201)NMN_trans_PnuC (TIGR01528)→
env-1907	DEHJ01000057.1	+	17575	17665	RNA→ NorM (COG0534)vmrA (PRK09575)→ AmyAc_euk_bac_CMD_like (cd11353)Aamy (smart00642)→<br>MetG (COG0143)PRK12267 (PRK12267)tRNA_bind_EcMetRS_like (cd02800)→<br>TatD_DNAse (cd01310)TIGR00010 (TIGR00010)→ RsmA (COG0030)ksgA (PRK00274)→ hypo→<br>TadA (COG0590)MafB19-deam (pfam14437)→ GDB1 (COG3408)GDE_N (pfam12439)→
env-1908	OMVN01000004.1	+	19879	19969	RNA→ NorM (COG0534)matE (TIGR00797)→<br>AmyAc_euk_bac_CMD_like (cd11353)Aamy (smart00642)Malt_amylase_C (pfam16657)→<br>MetG (COG0143)tRNA_bind_EcMetRS_like (cd02800)metG_C_term (TIGR00399)Anticodon_1 (pfam08264)→<br>TatD_DNAse (cd01310)TIGR00010 (TIGR00010)→ RsmA (COG0030)ksgA (TIGR00755)→ hypo→<br>TadA (COG0590)MafB19-deam (pfam14437)→ GDB1 (COG3408)GDE_N (pfam12439)→
Efa-293-1	NZ_KE352585.1	-	202711	202611	RNA→ PRK11431 (PRK11431)EmrE (COG2076)→ PRK11431 (PRK11431)EmrE (COG2076)→
Efa-297-1	NZ_KE352306.1	-	155120	155020	RNA→ PRK11431 (PRK11431)EmrE (COG2076)→ PRK11431 (PRK11431)EmrE (COG2076)→
Efa-66-1	NZ_KB932399.1	-	154423	154323	RNA→ PRK11431 (PRK11431)EmrE (COG2076)→ PRK11431 (PRK11431)EmrE (COG2076)→
Eca-2-1	NZ_KE136476.1	+	241800	241902	RNA→ PRK11431 (PRK11431)EmrE (COG2076)→ PRK11431 (PRK11431)EmrE (COG2076)→
Eca-1-1	NZ_JXKJ01000021.1	-	45987	45885	RNA→ PRK11431 (PRK11431)EmrE (COG2076)→ PRK11431 (PRK11431)EmrE (COG2076)→
Efa-286-1	NZ_GL454614.1	-	2593	2493	RNA→ PRK11431 (PRK11431)EmrE (COG2076)→ PRK11431 (PRK11431)EmrE (COG2076)→
Efa-214-1	NZ_KB944600.1	+	299098	299198	RNA→ PRK11431 (PRK11431)EmrE (COG2076)→ PRK11431 (PRK11431)EmrE (COG2076)→
Efa-20-1	NZ_KE351125.1	+	1816	1916	RNA→ PRK11431 (PRK11431)EmrE (COG2076)→ PRK11431 (PRK11431)EmrE (COG2076)→
Efa-255-1	NZ_KE350310.1	-	38192	38092	RNA→ PRK11431 (PRK11431)EmrE (COG2076)→ PRK11431 (PRK11431)EmrE (COG2076)→
Efa-266-1	NZ_JH804758.1	-	108976	108876	RNA→ PRK11431 (PRK11431)EmrE (COG2076)→ PRK11431 (PRK11431)EmrE (COG2076)→
Efa-202-1	NZ_KB944613.1	+	85524	85624	RNA→ PRK11431 (PRK11431)EmrE (COG2076)→ PRK11431 (PRK11431)EmrE (COG2076)→
Efa-61-1	NZ_KB932377.1	+	75557	75657	RNA→ PRK11431 (PRK11431)EmrE (COG2076)→ PRK11431 (PRK11431)EmrE (COG2076)→
Lli-2-1	NZ_MIYK01000016.1	-	49675	49583	RNA→ PRK11431 (PRK11431)EmrE (COG2076)→ ASCH_Ef3133_like (cd06553)ASCH (smart01022)→ hypo→<br>hypo→ hypo→ PRK10250 (PRK10250)YjbR (pfam04237)→
Lli-1-1	NZ_CP014872.1	+	354317	354409	RNA→ PRK11431 (PRK11431)EmrE (COG2076)→ ASCH_Ef3133_like (cd06553)ASCH (smart01022)→ hypo→<br>hypo→ hypo→ PRK10250 (PRK10250)YjbR (pfam04237)→
Lli-1-2	NZ_CP014907.1	+	353788	353880	RNA→ PRK11431 (PRK11431)EmrE (COG2076)→ ASCH_Ef3133_like (cd06553)ASCH (smart01022)→ hypo→<br>hypo→ hypo→ PRK10250 (PRK10250)YjbR (pfam04237)→
Lli-2-2	NZ_FUXS01000003.1	+	74318	74410	RNA→ PRK11431 (PRK11431)EmrE (COG2076)→ ASCH_Ef3133_like (cd06553)ASCH (smart01022)→ hypo→<br>hypo→ hypo→ PRK10250 (PRK10250)YjbR (pfam04237)→
Lli-2-3	NZ_BBAF01000004.1	+	73795	73887	RNA→ PRK11431 (PRK11431)EmrE (COG2076)→ ASCH_Ef3133_like (cd06553)ASCH (smart01022)→ hypo→<br>hypo→ hypo→ hypo→
Lli-2-4	NZ_JQBT01000036.1	-	12917	12825	RNA→ PRK11431 (PRK11431)EmrE (COG2076)→ ASCH_Ef3133_like (cd06553)ASCH (smart01022)→ hypo→<br>hypo→ hypo→ PRK10250 (PRK10250)YjbR (pfam04237)→

1.4 Conserved domains

Conserved domains found in protein-coding genes listed in Section 1.3 are shown below, with the first sentence in their description from the Conserved Domain Database (if any). Conserved domains associated with more than one GGAM-1-recreated RNA are assigned a color, while others are shown in gray. The symbols 'd' and 'u' (if any) to the left of the colored domain name indicate whether the domain occurs upstream or downstream of the RNA motif. Domains marked with lower-case 'd' occur (at least once) present downstream of some GGAM-1-recreated RNA in the opposite orientation. Domains marked with capital 'D' occur downstream and in the same orientation (i.e., a potential *cis*-regulatory arrangement). Domains marked with a 'u' occur upstream of the RNA in either orientation. If the 'd'/'u' symbols are missing, then the domain is downstream and in the same strand. (This mode is used for motifs predicted as *cis*-regulatory.) The number in parentheses after the colored domain name is the number of occurrences in Section 1.3.

cd00090 (2) Arsenical Resistance Operon Repressor and similar prokaryotic, metal regulated homodimeric repressors.
cd00093 (3) Helix-turn-helix XRE-family like proteins.
cd00156 (1) Signal receiver domain; originally thought to be unique to bacteria (CheY, OmpR, NtrC, and PhoB), now recently identified in eukaroytes ETR1 Arabidopsis thaliana; this domain receives the signal from the sensor partner in a two-component systems; contains a phosphoacceptor site that is phosphorylated by histidine kinase homologs; usually found N-terminal to a DNA binding effector domain; forms homodimers
cd00229 (1) SGNH_hydrolase, or GDSL_hydrolase, is a diverse family of lipases and esterases.
cd00267 (10) ATP-binding cassette transporter nucleotide-binding domain.
cd00371 (1) Heavy-metal-associated domain (HMA) is a conserved domain of approximately 30 amino acid residues found in a number of proteins that transport or detoxify heavy metals, for example, the CPx-type heavy metal ATPases and copper chaperones.
cd00430 (1) Type III Pyridoxal 5-phosphate (PLP)-Dependent Enzyme Alanine Racemase.
cd00431 (15) Cysteine hydrolases; This family contains amidohydrolases, like CSHase (N-carbamoylsarcosine amidohydrolase), involved in creatine metabolism and nicotinamidase, converting nicotinamide to nicotinic acid and ammonia in the pyridine nucleotide cycle.
cd00475 (1) Cis (Z)-Isoprenyl Diphosphate Synthases.
cd00537 (1) Methylenetetrahydrofolate reductase (MTHFR).
cd00657 (1) Ferritin-like superfamily of diiron-containing four-helix-bundle proteins.
cd01086 (1) Methionine Aminopeptidase 1.
cd01109 (1) Helix-Turn-Helix DNA binding domain of the MerR-like transcription regulators YyaN and YraB.
cd01164 (2) 1-phosphofructokinase (FruK), minor 6-phosphofructokinase (pfkB) and related sugar kinases.
cd01298 (1) TRZ/ATZ family contains enzymes from the atrazine degradation pathway and related hydrolases.
cd01310 (2) TatD like proteins; E.
cd01335 (10) Radical SAM superfamily.
cd01700 (1) umuC subunit of DNA Polymerase V.
cd01949 (2) Diguanylate-cyclase (DGC) or GGDEF domain.
cd02062 (3) Proteins of this family catalyze the reduction of flavin or nitrocompounds using NAD(P)H as electron donor in a obligatory two-electron transfer, utilizing FMN or FAD as cofactor.
cd02440 (1) S-adenosylmethionine-dependent methyltransferases (SAM or AdoMet-MTase), class I; AdoMet-MTases are enzymes that use S-adenosyl-L-methionine (SAM or AdoMet) as a substrate for methyltransfer, creating the product S-adenosyl-L-homocysteine (AdoHcy).
cd02511 (1) UDP-glucose LOS-beta-1,4 glucosyltransferase is required for biosynthesis of lipooligosaccharide.
cd02609 (1) uncharacterized subfamily of P-type ATPase transporter, similar to uncharacterized Streptococcus pneumoniae exported protein 7, Exp7.
cd02800 (2) tRNA-binding-domain-containing Escherichia coli methionyl-tRNA synthetase (EcMetRS)-like proteins.
cd02883 (1) Nudix hydrolase is a superfamily of enzymes found in all three kingdoms of life, and it catalyzes the hydrolysis of NUcleoside DIphosphates linked to other moieties, X.
cd03032 (1) Arsenate Reductase (ArsC) family, Spx subfamily; Spx is a unique RNA polymerase (RNAP)-binding protein present in bacilli and some mollicutes.
cd03255 (1) ATP-binding cassette domain of the transporters involved in export of lipoprotein and macrolide, and cell division protein.
cd03293 (3) ATP-binding cassette domain of the nitrate and sulfonate transporters.
cd03357 (3) Maltose O-acetyltransferase (MAT) and Galactoside O-acetyltransferase (GAT): MAT and GAT catalyze the CoA-dependent acetylation of the 6-hydroxyl group of their respective sugar substrates. [STAV]-X-[LIV]-[GAED]
cd03424 (6) ADP-ribose pyrophosphatase (ADPRase) catalyzes the hydrolysis of ADP-ribose and a variety of additional ADP-sugar conjugates to AMP and ribose-5-phosphate.
cd04086 (2) Carbohydrate Binding Module 35 (CBM35); appended to several carbohydrate binding enzymes, including several glycoside hydrolase (GH) family 26 mannanase domains.
cd04250 (3) AAK_NAGK-C: N-Acetyl-L-glutamate kinase - cyclic (NAGK-C) catalyzes the phosphorylation of the gamma-COOH group of N-acetyl-L-glutamate (NAG) by ATP in the second step of arginine biosynthesis found in some bacteria and photosynthetic organisms using the non-acetylated, cyclic route of ornithine biosynthesis.
cd04335 (1) This CD includes bacterial (Agrobacterium tumefaciens and Caulobacter crescentus ProX, and Clostridium sticklandii PrdX) and eukaryotic (Plasmodium falciparum N-terminal ProRS editing domain) sequences.
cd04647 (1) Maltose O-acyltransferase (MAT)-like: This family is composed of maltose O-acetyltransferase, galactoside O-acetyltransferase (GAT), xenobiotic acyltransferase (XAT) and similar proteins. [STAV]-X-[LIV]-[GAED]
cd04688 (9) Members of the Nudix hydrolase superfamily catalyze the hydrolysis of NUcleoside DIphosphates linked to other moieties, X.
cd05709 (3) Site-2 protease (S2P) class of zinc metalloproteases (MEROPS family M50) cleaves transmembrane domains of substrate proteins, regulating intramembrane proteolysis (RIP) of diverse signal transduction mechanisms.
cd06174 (1) The Major Facilitator Superfamily (MFS) is a large and diverse group of secondary transporters that includes uniporters, symporters, and antiporters.
cd06553 (6) ASC-1 homology domain, subfamily similar to Enterococcus faecalis Ef3133.
cd06561 (1) A new structural DNA glycosylase.
cd06660 (1) Aldo-keto reductases (AKRs) are a superfamily of soluble NAD(P)(H) oxidoreduc-

tases whose chief purpose is to reduce aldehydes and ketones to primary and secondary alcohols.
cd06850 (1) The biotinyl-domain or biotin carboxyl carrier protein (BCCP) domain is present in all biotin-dependent enzymes, such as acetyl-CoA carboxylase, pyruvate carboxylase, propionyl-CoA carboxylase, methylcrotonyl-CoA carboxylase, geranyl-CoA carboxylase, oxaloacetate decarboxylase, methylmalonyl-CoA decarboxylase, transcarboxylase and urea amidolyase.
cd07263 (3) uncharacterized subfamily of vicinal oxygen chelate (VOC) family.
cd07478 (1) Peptidase S8 family domain in CspA-like proteins.
cd07812 (7) START/RHO_alpha_C/PITP/Bet_v1/CoxG/CalC (SRPBCC) ligand-binding domain superfamily.
cd07824 (2) Ligand-binding SRPBCC domain of an uncharacterized subfamily of proteins.
cd08187 (1) Butanol dehydrogenase catalyzes the conversion of butyraldehyde to butanol with the cofactor NAD(P)H being oxidized in the process.
cd08601 (1) Glycerophosphodiester phosphodiesterase domain of Staphylococcus aureus and similar proteins.
cd09083 (1) Exonuclease-Endonuclease-Phosphatase domain; uncharacterized family 1.
cd09154 (1) Putative catalytic domain, repeat 1, of Streptococcus mutans uncharacterized protein SMU_988 and similar proteins.
cd09160 (1) Putative catalytic domain, repeat 2, of Streptococcus mutans uncharacterized protein SMU_988 and similar proteins.
cd10035 (2) Uncharacterized subfamily of Uracil-DNA glycosylases.
cd11009 (2) Zinc dependent phospholipase C (alpha toxin).
cd11353 (2) Alpha amylase catalytic domain found in eukaryotic and bacterial cyclomaltodextrinases and related proteins.
cd12082 (2) Multidrug and toxic compound extrusion family and similar proteins.
cd12208 (4) putative septicolysin, cholesterol-dependent cytolysin family and related proteins.
cd12827 (4) uncharacterized bacterial subfamily of the Escherichia coli CorA-Salmonella typhimurium ZntB family.
cd12870 (2) antitoxin MqsA for MqsR toxin.
cd13131 (4) Subfamily of the multidrug and toxic compound extrusion (MATE)-like proteins similar to Vibrio cholerae NorM.
cd13138 (2) Subfamily of the multidrug and toxic compound extrusion (MATE)-like proteins similar to Bacillus subtilis yoeA.
cd13143 (325) Subfamily of the multidrug and toxic compound extrusion (MATE)-like proteins similar to Streptococcus aureus MepA.
cd13563 (1) Putative substrate binding domain of sulfonate binding protein-like, a member of the type 2 periplasmic binding protein fold.
cd13606 (1) Bacterial substrate-binding protein ProX of ABC-type osmoregulated transporter and its related proteins; the type 2 periplasmic-binding protein fold.
cd14256 (2) Type I dockerin repeat domain.
cd14791 (1) glycosyl hydrolase family 36 (GH36).
COG0005 (2) Purine nucleoside phosphorylase [Nucleotide transport and metabolism]
COG0009 (1) tRNA A37 threonylcarbamoyladenosine synthetase subunit TsaC/SUA5/YrdC [Translation, ribosomal structure and biogenesis]
COG0012 (1) Ribosome-binding ATPase YchF, GTP1/OBG family [Translation, ribosomal structure and biogenesis]
COG0025 (2) NhaP-type Na+/H+ or K+/H+ antiporter [Inorganic ion transport and metabolism]
COG0030 (6) 16S rRNA A1518 and A1519 N6-dimethyltransferase RsmA/KsgA/DIM1 (may also have DNA glycosylase/AP lyase activity) [Translation, ribosomal structure and biogenesis]
COG0143 (2) Methionyl-tRNA synthetase [Translation, ribosomal structure and biogenesis]
COG0154 (1) Asp-tRNAAsn/Glu-tRNAGln amidotransferase A subunit or related amidase [Translation, ribosomal structure and biogenesis]
COG0155 (1) Sulfite reductase, beta subunit (hemoprotein) [Inorganic ion transport and metabolism]
COG0177 (1) Endonuclease III [Replication, recombination and repair]
COG0212 (1) 5-formyltetrahydrofolate cyclo-ligase [Coenzyme transport and metabolism]
COG0233 (1) Ribosome recycling factor [Translation, ribosomal structure and biogenesis]
COG0262 (4) Dihydrofolate reductase [Coenzyme transport and metabolism]
COG0336 (7) tRNA G37 N-methylase TrmD [Translation, ribosomal structure and biogenesis]
COG0347 (5) Nitrogen regulatory protein PII [Signal transduction mechanisms, Amino acid transport and metabolism]
COG0359 (1) Ribosomal protein L9 [Translation, ribosomal structure and biogenesis]
COG0370 (1) Fe2+ transport system protein B [Inorganic ion transport and metabolism]
COG0389 (3) Nucleotidyltransferase/DNA polymerase involved in DNA repair [Replication, recombination and repair]
COG0427 (1) Acyl-CoA hydrolase [Energy production and conversion]
COG0439 (1) Biotin carboxylase [Lipid transport and metabolism]
COG0456 (3) Ribosomal protein S18 acetylase RimI and related acetyltransferases [Translation, ribosomal structure and biogenesis]
COG0460 (1) Homoserine dehydrogenase [Amino acid transport and metabolism]
COG0522 (8) Ribosomal protein S4 or related protein [Translation, ribosomal structure and biogenesis]
COG0530 (1) Ca2+/Na+ antiporter [Inorganic ion transport and metabolism]
COG0534 (120) Na+-driven multidrug efflux pump [Defense mechanisms]
COG0574 (1) Phosphoenolpyruvate synthase/pyruvate phosphate dikinase [Carbohydrate transport and metabolism]
COG0575 (1) CDP-diglyceride synthetase [Lipid transport and metabolism]
COG0584 (4) Glycerophosphoryl diester phosphodiesterase [Lipid transport and metabolism]
COG0590 (9) tRNA(Arg) A34 adenosine deaminase TadA [Translation, ribosomal structure and biogenesis]
COG0596 (2) Pimeloyl-ACP methyl ester carboxylesterase [Coenzyme transport and metabolism, General function prediction only]
COG0599 (1) Uncharacterized conserved protein YurZ, alkylhydroperoxidase/carboxymuconolactone decarboxylase family [General function prediction only]
COG0600 (3) ABC-type nitrate/sulfonate/bicarbonate transport system, permease component [Inorganic ion transport and metabolism]
COG0642 (5) Signal transduction histidine kinase [Signal transduction mechanisms]
COG0655 (1) Multimeric flavodoxin WrbA [Energy production and conversion]
COG0656 (1) Aldo/keto reductase, related to diketogulonate reductase [Secondary metabolites biosynthesis, transport and catabolism]
COG0675 (6) Transposase [Mobilome: prophages, transposons]
COG0688 (1) Phosphatidylserine decarboxylase [Lipid transport and metabolism]
COG0697 (3) Permease of the drug/metabolite transporter (DMT) superfamily [Carbohydrate transport and metabolism, Amino acid transport and metabolism, General function prediction only]
COG0701 (1) Uncharacterized membrane protein YraQ, UPF0718 family [Function unknown]
COG0716 (7) Flavodoxin [Energy production and conversion]

COG0743 (1) 1-deoxy-D-xylulose 5-phosphate reductoisomerase [Lipid transport and metabolism]
COG0745 (3) DNA-binding response regulator, OmpR family, contains REC and winged-helix (wHTH) domain [Signal transduction mechanisms, Transcription]
COG0751 (10) Glycyl-tRNA synthetase, beta subunit [Translation, ribosomal structure and biogenesis]
COG0782 (3) Transcription elongation factor, GreA/GreB family [Transcription]
COG0840 (1) Methyl-accepting chemotaxis protein [Cell motility, Signal transduction mechanisms]
COG1075 (1) Triacylglycerol esterase/lipase EstA, alpha/beta hydrolase fold [Lipid transport and metabolism]
COG1092 (1) 23S rRNA G2069 N7-methylase RlmK or C1962 C5-methylase RlmI [Translation, ribosomal structure and biogenesis]
COG1115 (1) Na+/alanine symporter [Amino acid transport and metabolism]
COG1132 (4) ABC-type multidrug transport system, ATPase and permease component [Defense mechanisms]
COG1136 (1) ABC-type lipoprotein export system, ATPase component [Cell wall/membrane/envelope biogenesis]
COG1145 (10) Ferredoxin [Energy production and conversion]
COG1146 (1) NAD-dependent dihydropyrimidine dehydrogenase, PreA subunit [Nucleotide transport and metabolism]
COG1247 (1) L-amino acid N-acyltransferase YncA [Amino acid transport and metabolism]
COG1283 (1) Na+/phosphate symporter [Inorganic ion transport and metabolism]
COG1316 (1) Anionic cell wall polymer biosynthesis enzyme, LytR-Cps2A-Psr (LCP) family [Cell wall/membrane/envelope biogenesis]
COG1327 (1) Transcriptional regulator NrdR, contains Zn-ribbon and ATP-cone domains [Transcription]
COG1359 (1) Quinol monooxygenase YgiN [Energy production and conversion]
COG1410 (2) Methionine synthase I, cobalamin-binding domain [Amino acid transport and metabolism]
COG1472 (3) Periplasmic beta-glucosidase and related glycosidases [Carbohydrate transport and metabolism]
COG1691 (1) NCAIR mutase (PurE)-related protein [Nucleotide transport and metabolism]
COG1733 (1) DNA-binding transcriptional regulator, HxlR family [Transcription]
COG1739 (1) Putative translation regulator, IMPACT (imprinted ancient) protein family [General function prediction only]
COG1846 (3) DNA-binding transcriptional regulator, MarR family [Transcription]
COG1878 (1) Kynurenine formamidase [Amino acid transport and metabolism]
COG1917 (1) Cupin domain protein related to quercetin dioxygenase [General function prediction only]
COG1918 (1) Fe2+ transport system protein FeoA [Inorganic ion transport and metabolism]
COG1940 (1) Sugar kinase of the NBD/HSP70 family, may contain an N-terminal HTH domain [Transcription, Carbohydrate transport and metabolism]
COG2049 (1) Allophanate hydrolase subunit 1 [Amino acid transport and metabolism]
COG2076 (1321) Multidrug transporter EmrE and related cation transporters [Defense mechanisms]
COG2186 (2) DNA-binding transcriptional regulator, FadR family [Transcription]
COG2207 (3) AraC-type DNA-binding domain and AraC-containing proteins [Transcription]
COG2608 (1) Copper chaperone CopZ [Inorganic ion transport and metabolism]
COG2865 (2) Predicted transcriptional regulator, contains HTH domain [Transcription]
COG2873 (3) O-acetylhomoserine/O-acetylserine sulfhydrylase, pyridoxal phosphate-dependent [Amino acid transport and metabolism]
COG2972 (5) Sensor histidine kinase YesM [Signal transduction mechanisms]
COG3064 (2) Membrane protein involved in colicin uptake [Cell wall/membrane/envelope biogenesis]
COG3070 (2) Transcriptional regulator of competence genes, TfoX/Sxy family [Transcription]
COG3077 (4) Antitoxin component of the RelBE or YafQ-DinJ toxin-antitoxin module [Defense mechanisms]
COG3201 (677) Nicotinamide riboside transporter PnuC [Coenzyme transport and metabolism]
COG3279 (3) DNA-binding response regulator, LytR/AlgR family [Transcription, Signal transduction mechanisms]
COG3316 (1) Transposase (or an inactivated derivative) [Mobilome: prophages, transposons]
COG3382 (67) B3/B4 domain (DNA/RNA-binding domain of Phe-tRNA-synthetase) [General function prediction only]
COG3408 (2) Glycogen debranching enzyme (alpha-1,6-glucosidase) [Carbohydrate transport and metabolism]
COG3467 (62) Nitroimidazol reductase NimA or a related FMN-containing flavoprotein, pyridoxamine 5'-phosphate oxidase superfamily [Defense mechanisms]
COG3601 (1) Riboflavin transporter FmnP [Coenzyme transport and metabolism]
COG3603 (14) Uncharacterized protein [Function unknown]
COG3665 (2) Uncharacterized conserved protein YcgI, DUF1989 family [Function unknown]
COG3668 (1) Plasmid stabilization system protein ParE [Mobilome: prophages, transposons]
COG4115 (3) Toxin component of the Txe-Axe toxin-antitoxin module, Txe/YoeB family [Defense mechanisms]
COG4224 (10) Uncharacterized protein YnzC, UPF0291/DUF896 family [Function unknown]
COG4591 (4) ABC-type transport system, involved in lipoprotein release, permease component [Cell wall/membrane/envelope biogenesis]
COG4635 (4) Protoporphyrinogen IX oxidase, menaquinone-dependent (flavodoxin domain) [Coenzyme transport and metabolism]
COG4845 (1) Chloramphenicol O-acetyltransferase [Defense mechanisms]
COG5354 (1) Uncharacterized protein, contains Trp-Asp (WD) repeat [General function prediction only]
COG5562 (8) Prophage-encoded protein YbcV, DUF1398 family [Mobilome: prophages, transposons]
pfam00072 (3) Response regulator receiver domain.
pfam00149 (1) Calcineurin-like phosphoesterase.
pfam00302 (2) Chloramphenicol acetyltransferase.
pfam00395 (2) S-layer homology domain.
pfam00543 (1) Nitrogen regulatory protein P-II.
pfam00561 (3) alpha/beta hydrolase fold.
pfam00583 (23) Acetyltransferase (GNAT) family.
pfam00672 (1) HAMP domain.
pfam00710 (2) Asparaginase, N-terminal.
pfam00817 (1) impB/mucB/samB family.
pfam00857 (3) Isochorismatase family.
pfam00893 (2) Small Multidrug Resistance protein.
pfam01035 (2) 6-O-methylguanine DNA methyltransferase, DNA binding domain.
pfam01235 (1) Sodium:alanine symporter family.
pfam01243 (68) Pyridoxamine 5'-phosphate oxidase.

pfam01396 (2) Topoisomerase DNA binding C4 zinc finger.
pfam01402 (4) Ribbon-helix-helix protein, copG family.
pfam01548 (1) Transposase.
pfam01609 (1) Transposase DDE domain.
pfam01782 (8) RimM N-terminal domain.
pfam01863 (4) Protein of unknown function DUF45.
pfam01969 (1) Protein of unknown function DUF111.
pfam02091 (10) Glycyl-tRNA synthetase alpha subunit.
pfam02156 (2) Glycosyl hydrolase family 26.
pfam02371 (2) Transposase IS116/IS110/IS902 family.
pfam02417 (5) Chromate transporter.
pfam02525 (3) Flavodoxin-like fold.
pfam02574 (1) Homocysteine S-methyltransferase.
pfam02604 (1) Antitoxin Phd_YefM, type II toxin-antitoxin system.
pfam02626 (1) Carboxyltransferase domain, subdomain A and B.
pfam02687 (1) FtsX-like permease family.
pfam02690 (1) Na+/Pi-cotransporter.
pfam02743 (4) Cache domain.
pfam02810 (1) SEC-C motif. [HC]
pfam03424 (1) Carbohydrate binding domain (family 17/28).
pfam03483 (6) B3/4 domain.
pfam03492 (3) SAM dependent carboxyl methyltransferase.
pfam03734 (1) L,D-transpeptidase catalytic domain.
pfam03811 (1) InsA N-terminal domain.
pfam03977 (3) Na+-transporting oxaloacetate decarboxylase beta subunit.
pfam04023 (1) FeoA domain.
pfam04055 (2) Radical SAM superfamily.
pfam04237 (5) YjbR.
pfam04343 (1) Protein of unknown function, DUF488.
pfam04463 (1) Protein of unknown function (DUF523).
pfam04964 (3) Flp/Fap pilin component.
pfam04973 (61) Nicotinamide mononucleotide transporter.
pfam05239 (6) PRC-barrel domain.
pfam05857 (1) TraX protein.
pfam06305 (2) Protein of unknown function (DUF1049).
pfam06445 (1) GyrI-like small molecule binding domain.
pfam06580 (1) Histidine kinase.
pfam06769 (1) YoeB-like toxin of bacterial type II toxin-antitoxin system.
pfam06912 (21) Protein of unknown function (DUF1275).
pfam07275 (2) Antirestriction protein (ArdA).
pfam07883 (1) Cupin domain.
pfam08264 (1) Anticodon-binding domain of tRNA.
pfam08325 (4) WLM domain.
pfam08378 (2) Nuclease-related domain.
pfam08713 (1) DNA alkylation repair enzyme.
pfam08863 (2) YolD-like protein.
pfam08973 (1) Domain of unknown function (DUF1893).
pfam09186 (1) Domain of unknown function (DUF1949).
pfam09308 (2) LuxQ, periplasmic.
pfam09347 (2) Domain of unknown function (DUF1989).
pfam09719 (1) Putative redox-active protein (C_GCAxxG_C_C).
pfam09972 (1) Predicted membrane protein (DUF2207).
pfam10604 (9) Polyketide cyclase / dehydrase and lipid transport.
pfam11068 (3) YlqD protein.
pfam11799 (2) impB/mucB/samB family C-terminal domain.
pfam12229 (1) Putative peptidoglycan binding domain.
pfam12323 (5) Helix-turn-helix domain.
pfam12439 (2) Glycogen debranching enzyme N terminal.
pfam12464 (4) Maltose acetyltransferase.
pfam12636 (2) Protein of unknown function (DUF3781).
pfam12669 (1) Virus attachment protein p12 family.
pfam12674 (1) Putative zinc ribbon domain.
pfam12697 (5) Alpha/beta hydrolase family.
pfam12724 (10) Flavodoxin domain.
pfam12802 (3) MarR family.
pfam12822 (2) ECF transporter, substrate-specific component.
pfam12832 (1) MFS_1 like family.
pfam12838 (10) 4Fe-4S dicluster domain.
pfam12900 (132) Pyridoxamine 5'-phosphate oxidase.
pfam13091 (1) PLD-like domain.
pfam13304 (10) AAA domain, putative AbiEii toxin, Type IV TA system.
pfam13336 (1) Acetyl-CoA hydrolase/transferase C-terminal domain.
pfam13396 (1) Phospholipase_D-nuclease N-terminal.
pfam13472 (1) GDSL-like Lipase/Acylhydrolase family.
pfam13476 (10) AAA domain.
pfam13508 (23) Acetyltransferase (GNAT) domain.
pfam13534 (1) 4Fe-4S dicluster domain.
pfam13536 (1) Putative multidrug resistance efflux transporter.
pfam13546 (1) DDE superfamily endonuclease.
pfam13610 (1) DDE domain.
pfam13649 (2) Methyltransferase domain.
pfam13673 (1) Acetyltransferase (GNAT) domain.
pfam13749 (1) Putative ATP-dependent DNA helicase recG C-terminal.
pfam13840 (14) ACT domain.
pfam14205 (1) Cysteine-rich KTR.
pfam14437 (4) MafB19-like deaminase.
pfam14501 (3) GHKL domain.
pfam14602 (3) Hexapeptide repeat of succinyl-transferase.
pfam14667 (44) Polysaccharide biosynthesis C-terminal domain.
pfam14827 (1) Double sensory domain of two-component sensor kinase.
pfam16265 (1) Domain of unknown function (DUF4918).
pfam16657 (1) Maltogenic Amylase, C-terminal domain.
pfam16874 (1) Glycosyl hydrolase family 36 C-terminal domain.
pfam16875 (1) Glycosyl hydrolase family 36 N-terminal domain.
pfam16935 (1) Putative Holin-like Toxin (Hol-Tox).
pfam16990 (2) Carbohydrate binding module (family 35).
pfam17210 (1) SdrD B-like domain.
PLN00134 (1) fumarate hydratase; Provisional
PLN02431 (1) ferredoxin–nitrite reductase
PLN02512 (2) acetylglutamate kinase
PLN02621 (6) nicotinamidase
PLN02731 (1) Putative lipid phosphate phosphatase
PRK00026 (6) tRNA (guanine-N(1)-)-methyltransferase; Reviewed
PRK00053 (1) alanine racemase; Reviewed

PRK00083 (1) ribosome recycling factor; Reviewed
PRK00122 (7) 16S rRNA-processing protein RimM; Provisional
PRK00137 (1) 50S ribosomal protein L9; Reviewed
PRK00226 (1) transcription elongation factor GreA; Reviewed
PRK00241 (9) NADH pyrophosphatase; Reviewed
PRK00274 (4) 16S ribosomal RNA methyltransferase KsgA/Dim1 family protein; Reviewed
PRK00464 (1) transcriptional regulator NrdR; Validated
PRK00723 (1) phosphatidylserine decarboxylase; Provisional
PRK00871 (2) glutathione-regulated potassium-efflux system ancillary protein KefF; Provisional
PRK00942 (1) acetylglutamate kinase; Provisional
PRK00969 (1) hypothetical protein; Provisional
PRK01233 (10) glycyl-tRNA synthetase subunit beta; Validated
PRK01655 (1) transcriptional regulator Spx; Reviewed
PRK01766 (2) multidrug efflux protein; Reviewed
PRK02539 (10) hypothetical protein; Provisional
PRK03103 (3) DNA polymerase IV; Reviewed
PRK04194 (1) hypothetical protein; Provisional
PRK04460 (4) nickel responsive regulator; Provisional
PRK05279 (1) N-acetylglutamate synthase; Validated
PRK05327 (8) 30S ribosomal protein S4; Validated
PRK05337 (3) beta-hexosaminidase; Provisional
PRK05447 (1) 1-deoxy-D-xylulose 5-phosphate reductoisomerase; Provisional
PRK05451 (2) dihydroorotase; Provisional
PRK06349 (1) homoserine dehydrogenase; Provisional
PRK07116 (2) flavodoxin; Provisional
PRK07219 (2) DNA topoisomerase I; Validated
PRK08186 (1) allophanate hydrolase; Provisional
PRK08645 (1) bifunctional homocysteine S-methyltransferase/5,10-methylenetetrahydrofolate reductase protein; Reviewed
PRK09279 (1) pyruvate phosphate dikinase; Provisional
PRK09348 (10) glycyl-tRNA synthetase subunit alpha; Validated
PRK09575 (314) multidrug efflux pump VmrA; Reviewed
PRK09601 (1) GTP-binding protein YchF; Reviewed
PRK10160 (2) taurine transporter subunit; Provisional
PRK10250 (5) hypothetical protein; Provisional
PRK10535 (4) macrolide transporter ATP-binding /permease protein; Provisional
PRK10734 (1) putative calcium/sodium:proton antiporter; Provisional
PRK10765 (3) nitroreductase A; Provisional
PRK10860 (5) tRNA-specific adenosine deaminase; Provisional
PRK11143 (4) glycerophosphodiester phosphodiesterase; Provisional
PRK11340 (1) phosphodiesterase YaeI; Provisional
PRK11431 (1312) multidrug efflux system protein; Provisional
PRK11440 (4) putative hydrolase; Provisional
PRK11565 (2) 2,5-diketo-D-gluconate reductase A; Provisional
PRK11624 (1) CDP-diglyceride synthase; Provisional
PRK11762 (5) adenosine nucleotide hydrolase NudE; Provisional
PRK12267 (1) methionyl-tRNA synthetase; Reviewed
PRK12472 (1) hypothetical protein; Provisional
PRK13748 (1) putative mercuric reductase; Provisional
PRK13882 (1) conjugal transfer protein TrbP; Provisional
PRK14072 (1) 6-phosphofructokinase; Provisional
PRK14830 (1) undecaprenyl pyrophosphate synthase; Provisional
PRK15138 (1) aldehyde reductase; Provisional
PTZ00331 (8) alpha/beta hydrolase; Provisional
PTZ00401 (1) aspartyl-tRNA synthetase; Provisional
sd00006 (1) Tetratricopeptide repeat.
smart00267 (2) diguanylate cyclase.
smart00283 (1) Methyl-accepting chemotaxis-like domains (chemotaxis sensory transducer).
smart00304 (3) HAMP (Histidine kinases, Adenylyl cyclases, Methyl binding proteins, Phosphatases) domain.
smart00342 (3) helix_turn_helix, arabinose operon control protein.
smart00345 (2) helix_turn_helix gluconate operon transcriptional repressor.
smart00359 (1) Putative RNA-binding Domain in PseudoUridine synthase and Archaeosine transglycosylase.
smart00382 (9) ATPases associated with a variety of cellular activities.
smart00387 (7) Histidine kinase-like ATPases.
smart00388 (2) His Kinase A (phosphoacceptor) domain.
smart00418 (3) helix_turn_helix, Arsenical Resistance Operon Repressor.
smart00448 (9) cheY-homologous receiver domain.
smart00530 (3) Helix-turn-helix XRE-family like proteins.
smart00642 (2) Alpha-amylase domain.
smart00770 (2) Zinc dependent phospholipase C (alpha toxin).
smart00796 (1) Allophanate hydrolase subunit 1.
smart00797 (1) Allophanate hydrolase subunit 2.
smart00850 (3) LytTr DNA-binding domain.
smart00862 (3) Transcriptional regulatory protein, C terminal.
smart00870 (2) Asparaginase, found in various plant, animal and bacterial cells.
smart00871 (1) Bacterial transcription activator, effector binding domain.
smart00873 (58) B3/4 domain.
smart00878 (1) Biotin carboxylase C-terminal domain.
smart00895 (2) This entry represents the C-terminal ligand binding domain of many members of the GntR family.
smart00899 (2) This entry represents the core domain of the ferrous iron (Fe2+) transport protein FeoA found in bacteria. [Fe-S]
smart00938 (6) Nitrogen regulatory protein P-II.
smart01001 (1) AIR carboxylase.
smart01022 (6) The ASCH domain adopts a beta-barrel fold similar to that of the PUA domain.
smart01059 (3) Chloramphenicol acetyltransferase.
TIGR00003 (1) copper ion binding protein. [Transport and binding proteins, Cations and iron carrying compounds]
TIGR00010 (2) hydrolase, TatD family. [Unknown function, Enzymes of unknown specificity]
TIGR00052 (1) nudix-type nucleoside diphosphatase, YffH/AdpP family. [Unknown function, Enzymes of unknown specificity]
TIGR00057 (1) tRNA threonylcarbamoyl adenosine modification protein, Sua5/YciO/YrdC/YwlC family. [Protein synthesis, tRNA and rRNA base modification]
TIGR00088 (1) tRNA (guanine-N1)-methyltransferase. [Protein synthesis, tRNA and rRNA base modification]
TIGR00227 (3) riboflavin-specific deaminase C-terminal domain.
TIGR00350 (1) cell envelope-related function transcriptional attenuator common domain. [Regulatory functions, Other]
TIGR00383 (3) magnesium Mg(2+) and cobalt Co(2+) transport protein (corA). [Transport and binding proteins, Cations and iron carrying compounds]

TIGR00399 (1) methionyl-tRNA synthetase C-terminal region/beta chain. [Protein synthesis, tRNA aminoacylation]
TIGR00437 (2) ferrous iron transporter FeoB. [Transport and binding proteins, Cations and iron carrying compounds]
TIGR00500 (1) methionine aminopeptidase, type I. [Protein fate, Protein modification and repair]
TIGR00676 (1) 5,10-methylenetetrahydrofolate reductase, prokaryotic form. [Amino acid biosynthesis, Aspartate family]
TIGR00744 (1) ROK family protein (putative glucokinase). [Unknown function, General]
TIGR00755 (2) ribosomal RNA small subunit methyltransferase A. [Protein synthesis, tRNA and rRNA base modification]
TIGR00797 (75) putative efflux protein, MATE family. [Transport and binding proteins, Other]
TIGR00835 (2) amino acid carrier protein.
TIGR00932 (2) transporter, monovalent cation:proton antiporter-2 (CPA2) family. [Transport and binding proteins, Cations and iron carrying compounds]
TIGR00937 (5) chromate transporter, chromate ion transporter (CHR) family. [Transport and binding proteins, Anions]
TIGR00950 (1) Carboxylate/Amino Acid/Amine Transporter. [Transport and binding proteins, Amino acids, peptides and amines]
TIGR01109 (3) sodium ion-translocating decarboxylase, beta subunit. [Transport and binding proteins, Cations and iron carrying compounds, Energy metabolism, Other]
TIGR01183 (1) nitrate ABC transporter, permease protein. [Transport and binding proteins, Anions]
TIGR01326 (3) OAH/OAS sulfhydrylase. [Amino acid biosynthesis, Aspartate family, Amino acid biosynthesis, Serine family]
TIGR01462 (2) transcription elongation factor GreA. [Transcription, Transcription factors]
TIGR01494 (1) ATPase, P-type (transporting), HAD superfamily, subfamily IC.
TIGR01528 (668) nicotinamide mononucleotide transporter PnuC. [Transport and binding proteins, Other, Biosynthesis of cofactors, prosthetic groups, and carriers, Pyridine nucleotides]
TIGR01552 (1) prevent-host-death family protein. [Cellular processes, Toxin production and resistance, Mobile and extrachromosomal element functions, Other]
TIGR01575 (1) ribosomal-protein-alanine acetyltransferase.
TIGR01697 (2) inosine/guanosine/xanthosine phosphorylase family.
TIGR01716 (2) transcriptional activator, Rgg/GadR/MutR family, C-terminal domain. [Regulatory functions, DNA interactions]
TIGR01728 (1) ABC transporter, substrate-binding protein, aliphatic sulfonates family. [Transport and binding proteins, Other]
TIGR01766 (3) transposase, IS605 OrfB family, central region. [Mobile and extrachromosomal element functions, Transposon functions]
TIGR02082 (1) 5-methyltetrahydrofolate–homocysteine methyltransferase. [Amino acid biosynthesis, Aspartate family]
TIGR02116 (4) toxin-antitoxin system, toxin component, Txe/YoeB family. [Cellular processes, Toxin production and resistance, Mobile and extrachromosomal element functions, Other]
TIGR02135 (1) phosphate transport system regulatory protein PhoU. [Regulatory functions, Other, Transport and binding proteins, Anions]
TIGR02212 (1) lipoprotein releasing system, transmembrane protein, LolC/E family. [Protein fate, Protein and peptide secretion and trafficking]
TIGR02273 (1) 16S rRNA processing protein RimM. [Transcription, RNA processing]
TIGR02384 (4) addiction module antitoxin, RelB/DinJ family. [Cellular processes, Toxin production and resistance, Mobile and extrachromosomal element functions, Other]
TIGR02425 (1) 4-carboxymuconolactone decarboxylase. [Energy metabolism, Other]
TIGR02495 (1) anaerobic ribonucleoside-triphosphate reductase activating protein. [Purines, pyrimidines, nucleosides, and nucleotides, 2'-Deoxyribonucleotide metabolism, Protein fate, Protein modification and repair]
TIGR02668 (3) probable molybdenum cofactor biosynthesis protein A, archaeal. [Biosynthesis of cofactors, prosthetic groups, and carriers, Molybdopterin]
TIGR02705 (1) nucleoside triphosphatase YtkD. [DNA metabolism, DNA replication, recombination, and repair]
TIGR02727 (1) 5,10-methenyltetrahydrofolate synthetase. [Central intermediary metabolism, One-carbon metabolism]
TIGR02794 (2) TolA protein. [Transport and binding proteins, Other, Cellular processes, Pathogenesis]
TIGR03314 (1) putative selenium metabolism protein SsnA.
TIGR03434 (1) Acidobacterial duplicated orphan permease.
TIGR03828 (2) 1-phosphofructokinase.
TIGR03831 (2) YgiT-type zinc finger domain.
TIGR03879 (1) probable regulatory domain.
TIGR04102 (1) SWIM/SEC-C metal-binding motif protein, PBPRA1643 family.
TIGR04317 (8) tungsten cofactor oxidoreducase radical SAM maturase.

1.5 Multiple-sequence alignment

This alignment contains all predicted GGAM-1 RNAs (now renamed guanidine-IV riboswitches). For most of the sequences, we did not carefully realign the Rho-independent terminator stems. We believe that most sequences can be realigned such that there is a very small number of indels or mismatches (e.g., 1 or 2). We used this more comprehensive alignment to determine the genes regulated by GGAM-1 RNAs.

Each GGAM-1-recreated RNA is denoted by an abbreviation (like “Eco-1-1”) that refers to a taxonomy given in Section 1.1. (Superscript numbers refer to annotations listed in Section 1.2) The alignment may include sequences containing the RNA motif, as well as flanking sequence. The GGAM-1-recreated RNA itself is denoted by the line underneath marked 5′ and 3′ on either end. Nucleotides in flanking sequences (i.e., not part of the motif) are written in gray letters. Stems of predicted rho-independent transcription terminators, if any, are shaded yellow. (Note: terminator predictions have not been analyzed manually, and many are

likely to be false positives. Terminator predictions are those of the RNIE software.) Nucleotides predicted to function (as DNA) as transcription-factor binding sites, if any, are shaded in green. (But note: these predictions are manually annotated, so they might be under-predicted.) Annotated start codons, if any, are shaded green. (Note: start codons are frequently misannotated, especially in environmental samples.) Nucleotides proposed to basepair as part of the consensus structure are shaded in color when they comprise Watson-Crick or G-U pairs. Otherwise they are shaded gray. Conserved stems are also indicated at the bottom of the alignment by angle brackets, where matching $<$ and $>$ denote base-paired columns. Below these angle brackets, the symbol "2" denotes base pairs exhibiting covariation according to the statistically well-founded R-scape method. "1" denotes base pairs exhibiting covariation according to R2R's simplistic method. "0" denotes base pairs that are not observed to mutate and "?" denotes base pairs that have a significant frequency of non-canonical nucleotides for Watson-Crick or G-U pairs ($> 5\%$). Below these base pair annotation is the consensus sequence: "R" = "A" or "G", "Y" = "C" or "U", red nucleotides: nucleotide identity conserved more than 97% of the time, black nucleotides: 90%, gray nucleotides: 75%, red circle (•): nucleotide is present 97% of the time, black circle (•): 90%, gray circle (•): 75%, white circle (◦): 50%. All percentages of sequences just described (e.g. 97% conserved) assume that sequences have been weighted by the GSC algorithm implemented by the Infernal software package.

Duplicate sequences: the following putative homologs are not shown in the alignment because their sequences are identical to a homolog already shown: Asp-1-1, Asp-2-1, Bba-1-1, Bfo-1-1, Bsp-2-1, Cba-4-1, Cba-5-1, Cbo-2-2, Cbu-1-2, Cbu-1-3, Cbu-1-4, Cbu-2-1, Cbu-4-1, Cbu-5-1, Cbu-6-1, Cdi-1-1, Cfo-1-2, Cin-1-2, Cni-1-1, Cpa-1-1, Csp-1-1, Csp-10-1, Csp-16-1, Csp-18-1, Csp-2-1, Csp-8-1, Csy-1-1, Csy-3-1, Eca-1-1, Efa-1-1, Efa-1-2, Efa-1-3, Efa-1-4, Efa-1-5, Efa-1-6, Efa-1-7, Efa-1-8, Efa-10-1, Efa-100-1, Efa-101-1, Efa-102-1, Efa-103-1, Efa-104-1, Efa-105-1, Efa-106-1, Efa-107-1, Efa-108-1, Efa-109-1, Efa-11-1, Efa-110-1, Efa-111-1, Efa-112-1, Efa-113-1, Efa-114-1, Efa-115-1, Efa-116-1, Efa-117-1, Efa-118-1, Efa-119-1, Efa-12-1, Efa-120-1, Efa-121-1, Efa-122-1, Efa-123-1, Efa-124-1, Efa-125-1, Efa-126-1, Efa-127-1, Efa-128-1, Efa-129-1, Efa-13-1, Efa-130-1, Efa-131-1, Efa-132-1, Efa-133-1, Efa-134-1, Efa-135-1, Efa-136-1, Efa-137-1, Efa-138-1, Efa-139-1, Efa-14-1, Efa-140-1, Efa-141-1, Efa-142-1, Efa-143-1, Efa-144-1, Efa-145-1, Efa-146-1, Efa-147-1, Efa-148-1, Efa-149-1, Efa-15-1, Efa-150-1, Efa-151-1, Efa-152-1, Efa-153-1, Efa-154-1, Efa-155-1, Efa-156-1, Efa-157-1, Efa-158-1, Efa-16-1, Efa-16-2, Efa-160-1, Efa-161-1, Efa-162-1, Efa-163-1, Efa-164-1, Efa-165-1, Efa-166-1, Efa-167-1, Efa-168-1, Efa-169-1, Efa-17-1, Efa-170-1, Efa-171-1, Efa-172-1, Efa-173-1, Efa-174-1, Efa-175-1, Efa-176-1, Efa-177-1, Efa-178-1, Efa-179-1, Efa-18-1, Efa-180-1, Efa-181-1, Efa-182-1, Efa-183-1, Efa-184-1, Efa-185-1, Efa-186-1, Efa-187-1, Efa-188-1, Efa-189-1, Efa-19-1, Efa-190-1, Efa-191-1, Efa-192-1, Efa-193-1, Efa-194-1, Efa-195-1, Efa-196-1, Efa-197-1, Efa-198-1, Efa-199-1, Efa-2-1, Efa-20-1, Efa-200-1, Efa-201-1, Efa-203-1, Efa-204-1, Efa-205-1, Efa-206-1, Efa-207-1, Efa-208-1, Efa-209-1, Efa-21-1, Efa-210-1, Efa-212-1, Efa-213-1, Efa-214-1, Efa-215-1, Efa-216-1, Efa-217-1, Efa-218-1, Efa-219-1, Efa-22-1, Efa-220-1, Efa-221-1, Efa-222-1, Efa-223-1, Efa-224-1, Efa-225-1, Efa-226-1, Efa-227-1, Efa-228-1, Efa-229-1, Efa-23-1, Efa-230-1, Efa-231-1, Efa-232-1, Efa-233-1, Efa-234-1, Efa-235-1, Efa-236-1, Efa-237-1, Efa-238-1, Efa-239-1, Efa-24-1, Efa-240-1, Efa-241-1, Efa-242-1, Efa-243-1, Efa-244-1, Efa-245-1, Efa-246-1, Efa-247-1, Efa-248-1, Efa-249-1, Efa-25-1, Efa-250-1, Efa-251-1, Efa-252-1, Efa-253-1, Efa-254-1, Efa-255-1, Efa-257-1, Efa-258-1, Efa-259-1, Efa-26-1, Efa-260-1, Efa-261-1, Efa-262-1, Efa-263-1, Efa-264-1, Efa-265-1, Efa-266-1, Efa-267-1, Efa-268-1, Efa-27-1, Efa-270-1, Efa-271-1, Efa-272-1, Efa-273-1, Efa-274-1, Efa-275-1, Efa-276-1, Efa-277-1, Efa-278-1, Efa-279-1, Efa-28-1, Efa-280-1, Efa-281-1, Efa-282-1, Efa-283-1, Efa-284-1, Efa-285-1, Efa-287-1, Efa-288-1, Efa-289-1, Efa-29-1, Efa-290-1, Efa-291-1, Efa-291-2, Efa-291-3, Efa-292-1, Efa-294-2, Efa-294-3, Efa-296-1, Efa-297-1, Efa-298-1, Efa-299-1, Efa-3-1, Efa-30-1, Efa-300-1, Efa-301-1, Efa-302-1, Efa-303-1, Efa-304-1, Efa-305-1, Efa-306-1, Efa-307-1, Efa-308-1, Efa-309-1, Efa-31-1, Efa-310-1, Efa-311-1, Efa-312-1, Efa-313-1, Efa-314-1, Efa-315-1, Efa-316-1, Efa-317-1, Efa-318-1, Efa-319-1, Efa-32-1, Efa-320-1, Efa-321-1, Efa-322-1, Efa-323-1, Efa-324-1, Efa-325-1, Efa-326-1, Efa-327-1, Efa-33-1, Efa-34-1, Efa-35-1, Efa-36-1, Efa-37-1, Efa-38-1, Efa-39-1, Efa-4-1, Efa-40-1, Efa-41-1, Efa-42-1, Efa-43-1, Efa-44-1, Efa-45-1, Efa-46-1, Efa-47-1, Efa-48-1, Efa-49-1, Efa-5-1, Efa-50-1, Efa-51-1, Efa-52-1, Efa-53-1, Efa-54-1, Efa-55-1, Efa-56-1, Efa-57-1, Efa-58-1, Efa-59-1, Efa-6-1, Efa-60-1, Efa-61-1, Efa-62-1, Efa-63-1, Efa-64-1, Efa-65-1, Efa-67-1, Efa-68-1, Efa-69-1, Efa-7-1, Efa-70-1, Efa-71-1, Efa-72-1, Efa-73-1, Efa-74-1, Efa-75-1, Efa-76-1, Efa-77-1, Efa-78-1, Efa-79-1, Efa-8-1, Efa-80-1, Efa-81-1, Efa-82-1, Efa-83-1, Efa-84-1, Efa-85-1, Efa-86-1, Efa-87-1, Efa-88-1, Efa-89-1, Efa-9-1, Efa-90-1, Efa-91-1, Efa-92-1, Efa-93-1, Efa-94-1, Efa-95-1, Efa-96-1, Efa-97-1, Efa-98-1, Efa-99-1, Ega-1-1, Ega-1-4, Ega-1-5, Ega-1-6, Eha-1-1, Emo-1-1, Equ-1-2, Esa-1-1, Esp-1-1, Esp-10-1, Esp-12-1, Esp-2-1, Esp-5-1, Esp-6-1, Esp-7-1, Esp-8-1, Esp-9-1, Ete-1-2, Eur-2-2, Fnu-1-2, Fnu-1-3, Fsp-2-1, Fva-1-1, Lag-2-1, Lbr-2-1, Lco-2-1, Lco-3-1, Lco-4-1, Lcr-2-1, Lcr-3-1, Lfa-1-1, Lfa-2-2, Lga-1-2, Lga-2-2, Lga-2-3, Lga-3-1, Lga-6-1, Lhe-1-2, Lhe-1-3, Lhe-1-4, Lhe-2-1, Lhe-3-1, Lhi-1-1, Lhi-2-1, Ljo-1-2, Ljo-1-3, Ljo-1-4, Ljo-1-5, Ljo-1-6, Lla-1-1, Lla-1-10, Lla-1-11, Lla-1-13, Lla-1-2, Lla-1-3, Lla-1-4, Lla-1-5, Lla-1-6, Lla-1-7, Lla-1-8, Lla-1-9, Lla-10-1, Lla-10-2, Lla-10-3, Lla-11-1, Lla-12-1, Lla-13-1, Lla-13-2, Lla-14-1, Lla-15-1, Lla-16-1, Lla-17-1, Lla-18-1, Lla-19-1, Lla-2-1, Lla-3-1, Lla-3-2, Lla-3-3, Lla-3-4, Lla-3-5, Lla-3-6, Lla-5-1, Lla-6-1, Lla-7-1, Lla-8-1, Lla-9-1, Lli-1-1, Lli-1-2, Lli-2-2, Lli-2-3, Lli-2-4, Lmo-1-1, Lmo-1-2, Lno-1-2, Lno-1-3, Lpa-1-2, Lpa-2-2, Lpa-3-1, Lpa-3-2, Lph-2-1, Lph-3-1, Lsp-3-1, Lul-1-1, Lve-1-1, Mfa-1-1, Mpe-1-1, Msp-1-1, Msp-3-1, Msp-4-1, Oba-1-1, Osp-1-1, Osp-2-1, Pac-1-1, Pex-1-1, Psp-2-1, Rgn-1-1, Ssp-2-1, Ssu-1-1, Ssu-1-2, Ssu-1-5, Ssu-1-6, Ssu-2-1, Ssu-3-1, Ssu-4-1, Ssu-5-1, Ssu-7-1, Ssu-8-1, Tfl-1-1, Tfl-1-2, Tsp-1-1, Tsp-2-1, [am-1-1, [st-1-1, [sy-1-1, [sy-1-2, env-10, env-100, env-1000, env-1002, env-1003, env-1004, env-1005, env-1006, env-1007, env-1008, env-1009, env-101, env-1010, env-1011, env-1013, env-1014, env-1018, env-1019,

env-102, env-1020, env-1021, env-1027, env-1028, env-1029, env-103, env-1030, env-1031, env-1032, env-1033, env-1034, env-1035, env-1036, env-1037, env-1038, env-1039, env-1041, env-1042, env-1043, env-1044, env-105, env-1050, env-1051, env-106, env-1063, env-1064, env-1065, env-1066, env-1067, env-1068, env-1069, env-107, env-108, env-109, env-1093, env-11, env-1105, env-1108, env-1111, env-1138, env-1141, env-1144, env-1145, env-1146, env-1149, env-115, env-1152, env-1167, env-1168, env-1169, env-1170, env-1171, env-1172, env-1173, env-1174, env-1175, env-1176, env-1177, env-1178, env-1179, env-1184, env-1185, env-1186, env-1187, env-1192, env-1198, env-1201, env-1203, env-1207, env-1213, env-1216, env-1218, env-1219, env-1232, env-1233, env-1234, env-1235, env-1236, env-124, env-125, env-1256, env-1257, env-1259, env-126, env-1261, env-1265, env-1267, env-1268, env-127, env-1277, env-128, env-1280, env-1281, env-1282, env-1283, env-1284, env-1285, env-1286, env-1287, env-1295, env-1297, env-1298, env-1299, env-130, env-1314, env-1327, env-1328, env-1329, env-1330, env-1331, env-1333, env-1335, env-1336, env-1337, env-1338, env-1339, env-1340, env-1341, env-1342, env-1343, env-1346, env-1347, env-1348, env-1349, env-1350, env-1351, env-1352, env-1353, env-1354, env-1355, env-1356, env-1357, env-1358, env-1359, env-1360, env-1361, env-1362, env-1368, env-1369, env-137, env-1370, env-1373, env-1376, env-1379, env-1380, env-1381, env-1382, env-1383, env-1384, env-1385, env-1387, env-1389, env-139, env-1390, env-1391, env-1394, env-1395, env-1396, env-1397, env-1398, env-1411, env-1417, env-1418, env-142, env-1420, env-1426, env-1430, env-1432, env-1433, env-1436, env-1437, env-1451, env-1453, env-1464, env-147, env-1470, env-1472, env-148, env-1489, env-149, env-1500, env-1507, env-1508, env-1515, env-1519, env-1522, env-1526, env-1527, env-1528, env-1531, env-1533, env-1534, env-1548, env-1549, env-1550, env-1551, env-1552, env-1554, env-1555, env-1556, env-1557, env-1558, env-1559, env-156, env-1560, env-1561, env-1562, env-1563, env-1564, env-1565, env-1566, env-1567, env-1568, env-1569, env-1570, env-1571, env-1572, env-1573, env-1574, env-1575, env-1576, env-1577, env-1578, env-1579, env-1580, env-1581, env-1582, env-1583, env-1584, env-1585, env-1586, env-1587, env-1588, env-1589, env-1590, env-1591, env-1592, env-1593, env-1594, env-1595, env-1596, env-1597, env-1598, env-1599, env-16, env-1600, env-1601, env-1605, env-1606, env-1609, env-1610, env-1611, env-1612, env-1613, env-1614, env-1615, env-1616, env-1617, env-1618, env-1619, env-162, env-1620, env-1621, env-1622, env-1623, env-1624, env-1625, env-1626, env-1627, env-1628, env-1629, env-163, env-1630, env-1631, env-1632, env-1633, env-1634, env-1635, env-1636, env-1637, env-1638, env-1639, env-1640, env-1641, env-1642, env-1643, env-1644, env-1645, env-1646, env-1647, env-1648, env-1649, env-1650, env-1651, env-1652, env-1653, env-1654, env-1655, env-1656, env-1657, env-1658, env-1659, env-1660, env-1661, env-1662, env-1663, env-1664, env-1665, env-1666, env-1667, env-1668, env-1669, env-1670, env-1671, env-1672, env-1673, env-1674, env-1675, env-1676, env-1677, env-1678, env-1679, env-1680, env-1681, env-1683, env-1684, env-1685, env-1686, env-1687, env-1688, env-1694, env-1697, env-1698, env-1699, env-1700, env-1701, env-1702, env-1703, env-1704, env-1705, env-1709, env-171, env-1710, env-1714, env-1716, env-1733, env-1734, env-1735, env-1736, env-1737, env-1738, env-1739, env-174, env-175, env-1755, env-1758, env-176, env-1760, env-1767, env-177, env-1777, env-178, env-1782, env-1783, env-1784, env-1785, env-1786, env-1787, env-1788, env-179, env-1790, env-1791, env-1793, env-1795, env-1797, env-1798, env-1799, env-18, env-180, env-181, env-1817, env-182, env-1826, env-1827, env-1828, env-183, env-1833, env-1834, env-1835, env-1836, env-184, env-1841, env-1847, env-185, env-1854, env-1855, env-1856, env-186, env-1863, env-1867, env-1868, env-187, env-1874, env-188, env-1883, env-189, env-19, env-190, env-1908, env-191, env-192, env-193, env-194, env-195, env-196, env-197, env-198, env-199, env-20, env-200, env-201, env-202, env-203, env-204, env-205, env-206, env-207, env-208, env-209, env-210, env-211, env-212, env-213, env-214, env-215, env-216, env-217, env-218, env-219, env-220, env-221, env-222, env-223, env-224, env-225, env-226, env-227, env-228, env-229, env-230, env-236, env-237, env-238, env-241, env-252, env-253, env-263, env-264, env-265, env-266, env-267, env-268, env-269, env-27, env-271, env-272, env-275, env-276, env-277, env-278, env-279, env-280, env-281, env-282, env-283, env-284, env-285, env-286, env-288, env-289, env-290, env-291, env-292, env-293, env-296, env-297, env-298, env-299, env-3, env-300, env-301, env-302, env-303, env-304, env-305, env-306, env-307, env-319, env-320, env-321, env-324, env-326, env-328, env-329, env-334, env-338, env-345, env-346, env-355, env-356, env-361, env-364, env-365, env-366, env-367, env-369, env-370, env-371, env-377, env-378, env-379, env-380, env-381, env-382, env-383, env-384, env-385, env-386, env-392, env-393, env-394, env-395, env-397, env-398, env-399, env-400, env-401, env-402, env-403, env-404, env-405, env-406, env-407, env-419, env-424, env-425, env-427, env-435, env-438, env-445, env-446, env-447, env-451, env-453, env-455, env-458, env-474, env-475, env-489, env-49, env-490, env-491, env-499, env-50, env-507, env-51, env-510, env-513, env-52, env-53, env-532, env-539, env-54, env-540, env-541, env-542, env-543, env-544, env-55, env-551, env-554, env-555, env-556, env-56, env-567, env-568, env-569, env-57, env-571, env-572, env-579, env-58, env-581, env-582, env-583, env-584, env-585, env-586, env-59, env-590, env-596, env-597, env-60, env-602, env-603, env-604, env-606, env-607, env-61, env-613, env-614, env-615, env-616, env-617, env-62, env-620, env-622, env-624, env-63, env-630, env-639, env-64, env-642, env-643, env-645, env-646, env-647, env-648, env-649, env-65, env-650, env-651, env-652, env-653, env-654, env-655, env-656, env-66, env-661, env-662, env-666, env-67, env-671, env-673, env-676, env-677, env-678, env-68, env-681, env-682, env-685, env-686, env-687, env-688, env-69, env-70, env-706, env-71, env-711, env-712, env-715, env-720, env-733, env-739, env-749, env-750, env-752, env-756, env-766, env-767, env-772, env-779, env-783, env-796, env-797, env-8, env-808, env-809, env-81, env-810, env-812, env-813, env-814, env-815, env-816, env-817, env-818, env-819, env-82, env-820, env-821, env-822, env-823, env-825, env-826, env-83, env-831, env-832, env-833, env-834, env-835, env-836, env-837, env-838, env-839, env-84, env-840, env-841, env-842, env-843, env-844, env-845, env-846, env-847, env-85, env-852, env-853, env-854, env-857, env-859, env-86, env-867, env-87, env-870, env-873, env-874, env-875, env-876, env-877, env-879, env-88, env-887, env-89, env-890, env-894, env-9, env-90, env-903, env-904, env-909, env-91, env-919, env-92, env-920, env-921, env-924, env-925, env-926, env-93, env-931, env-932, env-94, env-943, env-95, env-956, env-959, env-96, env-962, env-964, env-967, env-97,

env-975, env-98, env-99, env-992, env-993, env-996

alignment positions 1 · · · 79

```
env-1      CCAAACCACCAUCAGCACCUACCUCCUCGCUCGGCAUCACGCCGUACAGCCCGGAACACAAGCUUGGCAAACCAAUCCC
Csp-12-1   ACUAAAACAAAAAUAAAUUGUAAAAAUAGUAUUGACUAUUAAUAAAUAUUUAAAUAUAAUUAAAACAUAAAAUUAAUAU
env-2      AAAGAAUUACUGUAAUUUUAAUAUAUUAUUUUUUUACAUUAAAAUACCAAUAAAUUUUAUCCUAGAAUUAUUUGUAAUA
env-4      CACAGAAUGCUUAUACUAAAAACAGUUUCUCGUCUCCAUCUUUGCGCCGUUCAGCUUUAACUUGACUUUUCGCGGGAGG
Lga-2-1    AUCUCUUUGCUGCUCAUUUCAUAUCUCGCAAGUCUAGAACUUGACCUUAGGUAGCAAAUGUGAAAUAAUGAUAAUAUAA
env-5      AUCUCUUUGCUGCUCAUUUCAUAUCUCGCAAGUCUAGAACUUGACCUUAGGUAGCAAAUGUGAAAUAAUGAUAAUAUAA
env-6      .........................UCGCAAGUCUAGAACUUGACCUUAGGUAGCAAAUGUGAAAUAAUGAUAAUAUAA
env-7      UUGUCAACUUUCCUAUGAUUUUAUAAUAUUUAAUUUAAACCUUGACCUCCUGAACUAAAUAUGAAAUAAUGAUACUAUA
Lga-4-1    UUUGAGAAACUUCCUAAAUUAUAUUUUAGGAACCUAGAGCUUGACCUCUUUACUCAAAUGUGAAAUAAUGAUACUACAA
env-12     CUUUUGGUAAGAUGCAUUUUUGGCGGGGUAAGAUUGAUUAAUGAAGAGGUGAAAUUUUCAACUGACAUCAUCACUCAUU
env-13     ACUCUGAUCCACCGCAGCUGGGAGGUGUAACAUUCACGAACUUAUUGCCCAUUUCGAGAGAUUUUGUAUCUUUGCAAUU
env-14     ............................................................................CCU
env-15     ACGCAUAAUUAUUUUAAUGUUUUUAUUGACAAGAUAAAACAAUUGUGCUAUCAUUUCCGCCAUAAGAAACACAAAUUGU
[sc-1-1    ..................................AUAAUCCGGAGUACCGCAGCGUGGUGAAGGAAUGGAUAGAGUUUG
env-17     ..................................AUAAUCCGGAGUACCGCAGCGUGGUGAAGGAAUGGAUAGAGUUUG
env-21     ..................................AUAAUCCGGAGUACCGCAGCGUGGUGAAGGAAUGGAUAGAGUUUG
env-22     AAAGACAGCAAGCAUGCGGAGGUAUGGCUGGAUCAUAAUCCGGAGUACCGCAGCGUGGUGAAGGAAUGGAUAGAGUUUG
env-23     UCGCUCCGCUCGACCUCCCCCCUAAUAAGACGGCGGAAAGAGUUUUGAAAUUCCCUUGACAUUUCCCCUGUCCUCUAGU
env-24     CUUCAAAAGAUUAUCAACUAAUAUUAUAAUGAGAAAAGCGUAGUUUUGCAUUAACAGUCUCAUUGAAGAAUUCCACCAU
env-25     .............................................................................AU
env-26     GCCCCAGGCGCUCUGGUCGCACCUCCUUGUGCUCCCGACGCGUCUUCCGCUCAGCGAAAUUCUUGAGGCAUCUACCGAA
Lbr-1-1    AUCUGCAUCGUCAGAACCUCCUCAAAGUAUGGCGCUGGCUUUAGUGUAGCAUGCUGACUAGACAAGCUGGGGCAUUCUU
Lsa-3-1    AUGCGCCAGCUUGGGAGCGCGUUGUACAGGGAUUUAAUAGUUGCUUGAGUUGUGUGACUAGACUUUUGUCAUCCAGUUU
env-28     UUUUGGUAGACUUGACUGAAUUAUAUUAAUUUUUAAGUGAAUAUAGUUCUUGAAAUUUAAACAUUAAUAAUUUAUAUUA
env-29     UUUUGGUAGACUUGACUGAAUUAUAUUAAUUUUUAAGUGAAUAUAGUUCUUGAAAUUUAAACAUUAAUAAUUUAUAUUA
Wbo-1-1    GCUUCAAAACGACUUUUGUCUCGCUCUCAGUUUGGUUAAACUUAUAUCUUGAAUUUUAAUUAUAUAUAGGUUAGAAUUU
Wjo-1-1    AAUGGCAUGUCGUAAGUUACCGUUAUUUUCAAAAGUAAAAUCUUGCAUUUCGUAGUAGGAAAUGAUUAGAUACUAGUAA
env-30     UCAUGGAUGGCUUUGCGCCGGGCGCGGGCAAAGCCUAACCCCCCUUGCCCGCCCGGCGUAACUGUGCUAUACUAGGGCU
Lag-1-1    AUCUAGUUUUCCUCCCCUAAUUAAACUCAUCUUUAUUAUACUACCCAUCCUUGCUUGCCUUAGCUAAUUUUGCUAUACU
env-31     AGGAAAACUGGUCAAUGAAUCCUGAAAUAAGUUUUCGCAGGAAGACUGCUUCCCAUGUAGAAACCUUUAUGAUAUAGUU
Lpl-1-1    GACACCGUUGACCCCACUGACAAAGUGGAUGCUUUGCGGUGAGUAAGCUUGACCAUGCAGAUGAGGUUUGCUAUAGUGG
Lcu-1-1    CAGCACUACUUCACAUUGAUGAAUCAAGUCGACUUCUAGUCAUCCUGGUUGACCUAUCAAUAAAAAUUAGUUAUAUUGA
Lki-1-1    AUUAUAGCAUUAAAUACAACGUUUCAUUUCAGCAAAUUCAAAUUGAGCUGAUUUGUCCUAGCUUCUAAGUUGUGCUAGA
env-32     UCGGAAAAUCCCGCAACUAUUUUUGCGCAAGUUCUGCGAGAGGACUUGACAGGCGUUCUUUUAUGUGAUAAAAUAGGCA
Csp-20-1   AGCCCGCGCCCUCGCGGCGCUGCGUGCGGAGCUGGCGAAGGCGGCUUGACAUUUCCGCGCCCGCGUGAUAAAAAUCCCU
env-33     ACUGUGCCGCAUUUUUAUUACUGUUAAAAUAUUUGGCUGAUUGGUUGACAGCAUUUAUUAUUUGUGGUAUAUUAACCUA
env-34     CCGUGAUCGGCGGGGCAGUCUGUCCGCCUUCCCCUCGCUUGACACCCCGGCGCUCAUCCCUUACAAUCGCCUGCGGUAA
env-35     GACAUUAUGGUAAUAUUGUACACCCCAUUUUCAAAAAAAAGAUUGAAAAAAGGAUUGAUUUCCCUCCGCGGAUAUGCUA
env-36     AAAACGCUCCUUACUAAACUAAGUUAGUAAAGAACGUUUUGGUGUAAUGUUUGACAAGGUUGUCACUUGUUGUUAAAAU
env-37     ......................CGUCAUUUAUAAAUCAUUCUUGCCUUUGAUAGGGCAUAUGCUAAAAUAAGAAUAAUU
env-38     AAAUAGUCCUGGAAGUUUUUUACGUCAUUUAUAAAUCAUUCUUUCCUUUGAUAGGGCAUAUGCUAAAAUAAGAAUAAUU
env-39     UUCUAUUUUACACCGAAAUCUCUUUUGUGAAAAGCAUUGAUUUCUUUUGCAAUCUGUGAUAGUAUAACUCUCGCAAACA
Esp-14-1   UAAAAAUGAGGUAAGAUUUAAAAAGAUAUAAGUAUAUUGAUAAAAUAGUAUUUUUAGUAUAUUGACUUAUUGUAAGAGA
Esp-15-1   UAAAAAUGAGGUAAGAUUUAAAAAGAUAUAAGUAUAUUGAUAAAAUAGUAUUUUUAGUAUAUUGACUUAUUGUAAGAGA
env-40     .......................AGAUAUAAGUAUAUUGAUAAAAUAGUAUUUUUAGUAUAUUGACUUAUUGUAAGAGA
env-41     AAAGAACGUGAAAUUUCAAAUAGUCAGAACUUUCGAGAAAUCCUCUUAACCAGAAUAACCCGCAUUUUUACUGAGUCGG
env-42     AAAGAACGUGAAAUUUCAAAUAGUCAGAACUUUCGAGAAAUCCUCUUAACCAGAAUAACCCGCAUUUUUACUGAGUCGG
env-43     AAAGAACGUGAAAUUUCAAAUAGUCAGAACUUUCGAGAAAUCCUCUUAACCAGAAUAACCCGCAUUUUUACUGAGUCGG
env-44     AAAGAACGUGAAAUUUCAAAUAGUCAGAACUUUCGAGAAAUCCUCUUAACCAGAAUAACCCGCAUUUUUACUGAGUCGG
env-45     AAAGAACGUGAAAUUUCAAAUAGUCAGAACUUUCGAGAAAUCCUCUUAACCAGAAUAACCCGCAUUUUUACUGAGUCGG
env-46     ..............................................................................G
env-47     AAAGAACGUGAAAUUUCAAAUAGUCAGAACUUUCGAGAAAUCCUCUUAACCAGAAUAACCCGCAUUUUUACUGAGUCGG
env-48     AAAGAACGUGAAAUUUCAAAUAGUCAGAACUUUCGAGAAAUCCUCUUAACCAGAAUAACCCGCAUUUUUACUGAGUCGG
env-72     AAAGAACGUGAAAUUUCAAAUAGUCAGAACUUUCGAGAAAUCCUCUUAACCAGAAUAACCCGCAUUUUUACUGAGUCGG
env-73     AAAGAACGUGAAAUUUCAAAUAGUCAGAACUUUCGAGAAAUCCUCUUAACCAGAAUAACCCGCAUUUUUACUGAGUCGG
env-74     AAAGAACGUGAAAUUUCAAAUAGUCAGAACUUUCGAGAAAUCCUCUUAACCAGAAUAACCCGCAUUUUUACUGAGUCGG
env-75     AAAGAAUGUGAAAUUUCAAAUAGUCAGAACUUUCGAGAAAUCCUCUUAACCAGAAUAACCCGCAUUUUUACUGAGUCGG
env-76     AAAGAACGUGAAAUUUCAAAUAGUCAGAACUUUCGAGAAAUCCUUUUAACGAGAAUAACCCGCAUUUUUACUGAGUCGG
env-77     AAAGAACGUGAAAUUUCAAAUAGUCAGAACUUUCGAGAAAUCCUUUUAACGAGAAUAACCCGCAUUUUUACUGAGUCGG
env-78     AAAGAACGUGAAAUUUCAAAUAGUCAGAACUUUCGAGAAAUCCUCUUAACCAGAAUAACCCGCAUUUUUACUGAGUCGG
env-79     GUUCUUGUGAAAGAACGUGAAAUUUCAAAUAGUCAGAACUUUCGAGAAAUCCUCUUAACCAGAAUAACCCGCAUGUCGG
env-80     AAAGAACGUGAAAUUUCAAAUAGUCAGAACUUUCGAGAAAUCCUUUUAACGAGAAUAACCCGCAUUUUUACUGAGUCGG
env-104    AAGAAGCGUGAAAACUUUGACGGUCAAAAUUUCUGACAAAACCUCUUUGCGAUGAUGAAACACAUCUUUAGUACGUCGG
env-110    .................................CGCUUGACAAGUGCGCUUUGAUAUCGUACAAUGUCGGCAACAAAAU
env-111    .................................CGCUUGACAAGUGCGCUUUGAUAUCGUACAAUGUCGGCAACAAAAU
env-112    UCCGCUGCUUCUCACAUUCGGAGAUUUUUGCGGUUAAUGUAUUGACAAUGCAGGAUUUAUAUGCUAUAAUAUUUUCCGA
env-113    AAUUGAUUCGUUACGUGAAUCAUUAACGACAAAUGAUUAAAUGAGCCAAAAUCCUUGAUUUGUGUUAUAGUUGUUCAUA
env-114    .................AUGUGCUUGCCUUUGGUAUUUGUUACGGAAUCGAGGCCUUGCUUGCAUAAAAGAGAAGAGAA
Rpe-1-1    AUUUGAUGUGACUGACUACUAAAGCGUGUUUUAAGUUGCAAAUUUUUUUAACAUGUGGUAAGAUCAAUCUCAUAUUAUA
env-116    AUUUGAUGUGACUGACUACUAGAGCGUGUUUUAAGUUGCAAAUUUUUUUAACAUGUGGUAAGAUCAAUCUCAUAUUAUA
Rsp-1-1    AGGUAAAUUUACGCAACCCUAGAGCGUGUUUUAAGUUGCAAAUUUUUUUAACAUGUGGUAAGAUCAAUCUCAUAUUAUA
Lsp-2-1    AGCACUAGGGGAAACCUAGAGUAUUAGUGCAGGUCUUUUUUGAUAAGAUUGCAUUUUUCAGUUAAUUUGUUAAGAUAAA
Lse-1-1    UACUGGAAUGGGACUUAAGGAUCCUGAAGAGUACGAUUGGUAAAAUGGUCGUUUGAAAUUGACGUUGAUCGUGUUAUGA
env-117    UAAAGAAAUAUCAAUAAUGUAGUUUUGUAAGAUGAAAGAUAUGAAGAAAUUUCAAAAGAAUUAAUAAAUUUAUUAGUAA
env-118    CAAGAGUUAUCCUAAACCAACGGCUUCUGCGCCCUUAGAAACAAUUUAAACAAUUUGACUUUUUAUAUUUUUGUUGAUA
env-119    ......................................AAACAAUUUAAACAAUUUGACUUUUUAUAUUUUUGUUGAUA

           ...............................................................................
           ...............................................................................
           ...............................................................................
           ...............................................................................
```

```
Loz-1-1     CUAAGCUAUUAAUAUGACAAAUGUUUCGAAAACAUUAAACUAAUCAAAUUAUAUUGAUAACUGUUGGCUAAUUUAUUAU
env-120     UGACAACAGUAUAGCAGUUUGUCCGGCCAACUGCAAGGUCGGGCAAAUUUCUCUUGACAAAGCCGCGCCUUCUGUGCUA
env-121     CGCAAGCCCCAUAGGGGGAUAGAAAUCUACUUUUUGCGGCAGAGUUUUAAAAAUUUCUUGAUUUUUCUCUUCCUUAUGA
env-122     AUCUUAUAUUAUUAUGUUAAUAUUAUGAAGGAAGAAACAGCUCAAUAUAAGGCAUUAUAUCAUAUUAAAUGAAAACCAA
Bpu-1-1     GUAAAACAUGCAAACAAAACCGCUCCGCUUUUCGGAGCGGUUUUCUUUUUCCCCUGUCUUGACAUUCCCGCACACACAU
env-123     UUUUCAAAAGAAAGCAAAAAAUUUUUUAUGAAAUCAUACAGUUAUAACUCUUAUCCACAGUUGACAAACUUUCGGCUAA
env-129     UUUUCAAAUCGGGGCAAAAAAGUUUUUAUAAAAUCAUACGGUUAUAUGCGUUAUCCACAGUUGACAAACUUUCGGUCGG
env-131     ......UAUAUAAAUUUAUAAUCAUUGUCAAAAGACUAGAAUCCAAUCUAGUCUUUUUUUGUUUUGAUUUUUGACGAAA
env-132     AAACCUUAUAUAAAUUUAUAAUCAUUGUCAAAAGACUAGAAUCCAAUCUAGUCUUUUUUUGUUUUGAUUUUUGACGAAA
Ali-1-1     GAAUGGUCCCCUUUUUUUGCUUUCAUGUUCUAUUUGCUUAUUACCCAGAUAAUUGACACUUAUUUAUGAUUUUGCUAUA
env-133     UUAUGCCGAUAUCUCCUGCGUCCUUUAGUAACUUCACCAUUUACCCAGAUAAUUGACACUUAUUUAUGAUUUUGCUAUA
env-134     ........................................ACUUAACAAAGGACAAUCCAGCAGCAGUUGUCCCGGAUA
Lin-1-1     UGCGUCUUGAAAAGGUUGAAAGCAUUGGAUGCAGAAUUGACUUAGCUAAGAAGGCUAAGAAGGACUAAUAUCAAGCUUC
Ljo-2-1     AUUUCGAUUCAUAAAUUGGUGAAGAAAAGAAAUCAUCACACGAACUUUACAAGAAUAUUUUUAUUUACUAAACUCUAAG
Ljo-1-1     AUUUCGAUUCAUAAAUUGGUGAAGAAAAGAAAUCAUCACACGAACUUUACAAGAAUAUUUUUAUUUACUAAACUCUAAG
env-135     AUUUCGAUUCAUAAAUUGUUGAAGAAAAGAAAUCAUCACACGAACUUUACAAGAAUAUUUUUAUUUACUAAACUAUAAG
env-136     AUAUCACCUAGACGUAUUUUAACACAUUAUCAUUUCAUAGCAACAGAACUGUUGACAACGCAAAUUCUUCAGGUAUAAU
env-138     CGAUAACAUCUAUUAUAACACAAAACAUGUGGAACUGACACCUUGGAUUACACGCCCUUGACGAGACAGGAAAUCUGUG
Fpl-1-1     ACAAAUGGUAAAAAAAGCUGGUACAGCCGGGGGGUUGACAGGGGGCGGUCUUUCUGUUAUCUUAAUACCCGACAGAUGA
Fsp-1-1     GCUGCGGGAAAAGCUGUCCCAGUAACGCGCCUCACACCUUGACAGGAUUCCGCUCUUCUGGUAUGCUCUUAUCCACAAG
env-140     CUGCGGGAAAAGCUGUCCCAGUAGGUUUCCUCUACACCUUGACAUUUUUCUGUCCUUCUGUUAUGCUCUUAUCCACUAG
env-141     UGUGAUAAAAAUACUAUUUAAGAGAGAUGAGACAUUAACGAGCCUAAAAGCCUAUAUUUUUUAGUUGAAAUAUGUUAGA
env-143     ..........................................................................UUAGA
Lla-20-1    CCUCUUAACUAAGCGGUUCGAGUUUAGGCUCCAAAAUUUCAAAAAAUUAUUUAGCAUUUUUUGAAAAAGUGUGAUAGAA
Lla-4-1     CCUCUUAACUAAGCGGUUCGAGUUUAGGCUCCAAAAUUUCAAAAAAUUAUUUAGCAUUUUUUGAAAAAGUAUGAUAGAA
Lla-21-1    CCUCUUAACUAAGCGGUUCGAGUUUAGGCUCCAAAAUUUCAAAAAAUUAUUUAGCAUUUUUUGAAAAAGUGUGAUAGAA
env-144     CCUCUUAACUAAGCGGUUCGAGUUUAGGCUCCAAAAUUUCAAAAAAUUAUUUAGCAUUUUUUGAAAAAGUGUGAUAGAA
env-145     GAUUCUCUGUUUUAAUAAUAGAUUUAAUCAUUCAACUCUAAAAAAUAAAUUUAGCUUUUUUAAUAAAAGUGUGAUAGAA
env-146     GAUUCUCUGUUUUAAUAAUAGAUUUAAUCAUUCAACUCUAAAAAAUAAAUUUAGCUUUUUUAAUAAAAGUGUGAUAGAA
env-150     GAUUCUCUGUUUUAAUAAUAGAUUUAAUCAUUCAACUCUAAAAAAUAAAUUUAGCUUUUUUAAUAAAAGUGUGAUAGAA
Lla-1-12    GAUUCUCUGUUUUAAUAAUAGAUUUAAUCAUUCAACUCUAAAAAAUAAAUUUAGCUUUUUUAAUAAAAGUGUGAUAGAA
env-151     ACUAUAUUUGUCUAAUUUUUUGUCUUACGAACGAGCUGAAUAAAUAGCGAUUAGAUAUUUUAGAUAAAAUGUGCUAGAA
env-152     UGUGAACAUUAUAGAAAUGCCCGGGGAAUAAAACAAUACAAAAGAGGUGACUUUGGGAGUGCUUUCGGGUAGAAUAGUG
env-153     CAGUCCGGGCUUUGAUGGUAGUUGGAGAAAAAUUUUAAGGCUAAACCAGUUAUUGCAUUUUUAUUUUUUUAUGAUAAUA
env-154     .........................GGUUUUACCGCUCUGUGGUAGAAAGGACUUGACAUUUCCCGGGAAAUCUGUAAA
env-155     .......UCCGGGAGGACCGGGAGCGGGUUUACCGCUCUGUGGUAGAAAGGACUUGACAUUUCCCGGGAAAUCUGUAAA
env-157     CGUAUUCUCCGGGAGGACCGGGAGCGGUUUUACCGCUCUGUGGUAGAAAGGACUUGACAUUUCCCGGGAAAUCUGUAAA
env-158     .......UCCGGGAGGACCGGGAGCGGUUUUACCGCUCUGUGGUAGAAAGGACUUGACAUUUCCCGGGAAAUCUGUAAA
env-159     .......UCCGGGAGGACCGGGAGCGGUUUUACCGCUCUGUGGUAGAAAGGACUUGACAUUUCCCGGGAAAUCUGUAAA
env-160     ......................................AUUGAUGUUUUAUUUUACAGAUUUCCCGGGAAAUCUGUAAA
env-161     .......UCCGGGAGGACCGGGAGCGGUUUUACCGCUCUGUGGUAGAAAGGACUUGACAUUUCCCGGGAAAUCUGUAAA
env-164     .......UCCGGGAGGACCCGGAGCGGUUUUACCGCUCUGUGGUAGAAAGGACUUGACAUUUCCCGGGAAAUCUGUAAA
env-165     .....................................CUGUGGUAGAAAGGACUUGACAUUUCCCGGGAAAUCUGUAAA
env-166     .......UCCGGGAGGACCGGGAGCGGUUUUACCGCUCUGUGGUAGAAAGGACUUGACAUUUCCCGGGAAAUCUGUAAA
env-167     .......UCCGGGAGGACCGGGAGCGGUUUUACCGCUCUGUGGUAGAAAGGACUUGACAUUUCCCGGGAAAUCUGUAAA
env-168     .......................................GUGGUAGAAAGGACUUGACAUUUCCCGGGAAAUCUGUAAA
env-169     .......UCCGGGAGGACCGGGAGCGGUUUUACCGCUCUGUGGUAGAAAGGACUUGACAUUUCCCGGGAAAUCUGUAAA
env-170     CGGAUGCUCCGGGAGGACCGGGAGCGGUUUUACCGCUCUGUGGUAGAAAGGACUUGACAUUUCCCGGGAAAUCUGUAAA
env-172     ..........GGGAGGACCGGGAGCGGUUUUACCGCUCUGUGGUAGAAAGGACUUGACAUUUCCCGGGAAAUCUGUAAA
env-173     .......UCCGGGAGGACCGGGAGCGGUUUUACCGCUCUGUGGUAGAAAGGACUUGACAUUUCCCGGGAAAUCUGUAAA
env-231     CGGAUGCUCCAGGAGGACCGGGAGCGGUUUUACCGCUCUGUGGUAGAAAGGACUUGACAUUUCCCGGGAAAUCUGUAAA
env-232     .......UCCGGGAGGACCGGGAGCGGUUUUACCGCUCUGUGGUAGAAAGGACUUGACAUUUCCCGGGAAAUCUGUAAA
env-233     .........................................GGUAGAAAGGACUUGACAUUUCCCGGGAAAUCUGUAAA
env-234     ...............................................................................
env-235     .......UCCAGGAGGACCGGGAGCGGUUUUACCGCUCUGUGGUAGAAAGGACUUGACAUUUCCCGGGAAAUCUGUAAA
env-239     .......UCCGGGAGGACCGGGAGCGGUUUUACCGCUCUGUGGGAGAAAGGACUUGACAUUUCCCGGGAAAUCUGUAAA
env-240     ...................GGGAGCGGUUUUACCGCUCUGUGGUAGAAAGGACUUGACAUUUCCCGGGAAAUCUGUAAA
env-242     .......UCCGGGAGGACCGGGAGCGGUUUUACCGCUCUGUGGUAGAAAGGACUUGACAUUUCCCGGGAAAUCUGUAAA
env-243     .......UCCGGGAGGACCGGGAGCGGUUUUACCGCUCUGUGGUAGAAAGGACUUGACAUUUCCCGGGAAAUCUGUAAA
env-244     .......UCCGGGAGGACCGGGAGCGGUUUUACCGCUCUGUGGUAGAAAGGACUUGACAUUUCCCGGGAAAUCUGUAAA
env-245     ............................UUUACCGCUCUGUGGUAGAAAGGACUUGACAUUUCCCGGGAAAUCUGUAAA
env-246     .......UCCGGGAGGACCGGGAGCGGUUUUACCGCUCCGUGGUGGAAAGGGCUUGACAUUUCCCGGGAAAUCUGUAAA
env-247     .......GGGAGGACCGGGAGCGGUUUUACCGCNNNNNNNNNNUAGAAAGGACUUGACAUUUCCCGGGAAAUCUGUAAA
env-248     .......UCCGGGAGGAUCGGGAGAUGUUCUACAGCUCCGUUGUGGAAAGGGCUUGACAUUUCCCGGGAAAUCUGUAAA
env-249     ..............................UACCGCUCUGUGGUAGAAAGGACUUGACAUUUCCCGGGAAAUCUGUAAA
env-250     .......NCCGGGAGGACCGGGAGCGGUUUUACCGCUCUGUGGUAGAAAGGACUUGACAUUUCCCGGGAAAUCUGUAAA
env-251     .......UCCGGGAGGACCGGGAGCGGUUUUACCGCUCUGUGGUAGAAAGGACUUGACAUUUCCCGGGAAAUCUGUAAA
env-254     UUUGCAAAGAAAAAGCGUUGAUGUUUCACGUUGUUAAUUUUAGUUGCAAUCUUGAGCGAAGCGGUUUAUAAUAACAACA
env-255     AAGAAAUGUCUCGAAUUCGAACUAUUAUUUUUUAGUUGAUUUUUUAAAAAAAUAAGAAUAUAAUGACCUGAGAUAUUUU
Efa-269-1   AAGAAAUGUCUCGAAUUCGAACUAUUGUUUUUUAGUUGAUUUUUUAAAAAAAUAAGAAUAUAAUAGCCUGAGAUAUUUU
Efa-159-1   AAGAAAUGUCUCGAAUUCGAACUAUUGUUUUUUAGUUGAUUUUUUAAAAAAAUAAGAAUAUAAUGACCCGAGAUAUUUU
env-256     AAGAAAUGUCUCGAAUUCGAACUAUUAUUUUUUAGUUGAUUUUUUGUAAAAAUAAGAAUAUAAUAGCCUGAGAUAAUUU
env-257     AAGAAAUGUCUCGAAUUCGAACUAUUAUUUUUUAGUUGAUUUUUAAAAAAAAUAAGAAUAUAAUAGCCUGAGAUAUUUU
env-258     ...........AAGGAGCGAGCAUUCUAAACGAGUUUGUUCAAAGUCACAAGAAAAAUCCAGUUUAACUUAGAUAUUUU
env-259     AAGAAAUGUCUCGAAUUCGAACUAUUGUUUUUUAGUUGAUUUUUUAAAAAAAUAAGAAUAUAAUGACCUGAGAUAUUUU
env-260     AAGAAAUGUCUCGAAUUCGAACUAUUAUUUUUUAGUUGAUUUUUUUAAAAAAUAAGAAUAUAAUUGCCUGAGAUAUUUU
env-261     AAGAAAUGUCUCGAAUUCGAACUAUUAUUUUUUAGUUGAUUUUUUAAAAAAAUAAGAAUAUAAUAGCCUGAGAUAUUUU
env-262     AAGAAAUGUCUCGAAUUCGAACUAUUGUUUUUUAGUUGAUUUUUUAAAAAAAUAAGAAUAUAAUGACCUGAGAUAUUUU
env-270     AAGAAAUGUCUCGAAUUCGAACUAUUAUUUUUUAGUUGAUUUUUUAAAAAAAUAAGAAUAUAAUAGCCUGAGAUAUUUU
env-273     AAGAAAUGUCUCGAAUUCGAACUAUUAUUUUUUAGUUGAUUUUUUAAAAAAAUAAGAAUAUAAUAACCAGAGAUAUUUU
env-274     AAGAAAUGUCUCGAAUUCGAACUAUUAUUUUUUAGUUGAUUUUUUUAAAAAAUAAGAAUAUAAUAGCCUGAGAUAUUUU
env-287     AAGAAAUGUCUCGAAUUCGAACUAUUGUUUUUUAGUUGAUUUUUAAAAAAAAUAAGAAUAUAAUGACCUGAGAUAUUUU

            ...............................................................................
            ...............................................................................
            ...............................................................................
            ...............................................................................
```

env-294	AAGAAAUGUCUCGAAUUCGAACUAUUAUUUUUUAGUUGAUUUUUUUUUUAAAAUAAGAAUAUAAUAGCCUGAGAUAUUUU
env-295	AAGAAAUGUCUCGAAUUCGAACUAUUAUUUUUUAGUUGAUUUUUUAAAAAAAUAAGAAUAUAAUAGCCUGAGAUAUUUU
env-308	AAGAAAUGUCUCGAAUUCGAACUAUUGUUUUUUAGUUGAUUUUUUAAAAAAAUAAGAAUAUAAUAGCCUGAGAUAUUUU
env-309	AAGAAAUGUCUCGAAUUCGAACUAUUAUUUUUUAGUUGAUUUUUUUUUAAAAAAUAAGAAUAUAAUAGCCUGAGAUAUUUU
env-310	AAGAAAUGUCUCGAAUUCGAACUAUUAUUUUUUAGUUGAUUUUUUUUUUAAAAUAAGAAUAUAAUAGCCUGAGAUAUUUU
env-311	AAGAAAUGUCUCGAAUUCGAACUAUUAUUUUUUAGUUGAUUUUUAAAAAAAAUAAGAAUAUAAUAGCCUGAGAUAUUUU
env-312	AAGAAAUGUCUCGAAUUCGAACUAUUAUUUUUUAGUUGAUUUUUUAAAAAAAUAAGAAUAUAAUAGCCUGAGAUAUUUU
env-313	....AUAUAAUAGCCUGAGAUAUUUU
env-314	AAGAAAUGUCUCGAAUUCGAACUAUUAUUUUUUAGUUGAUUUUUUAAAAAAAUAAGAAUAUAAUGACCUGAGAUAUUUU
env-315	AAGAAAUGUCUCGAAUUCGAACUAUUAUUUUUUAGUUGAUUUUUUAAAAAAAUAAGAAUAUAAUAGCCUGAGAUAUUUU
Efa-211-1	AAGAAAUGUCUCGAAUUCGAACUAUUAUUUUUUAGUUGAUUUUUUUUUUAAAAUAAGAAUAUAAUAGCCUGAGAUAUUUU
Efa-256-1	AAGAAAUGUCUCGAAUUCGAACUAUUAUUUUUUAGUUGAUUUUUUAAAAAAAUAAGAAUAUAAUAACCUGAGAUAUUUU
Ete-1-1	GAAAUUAAUCUAACAAAAGAAAAUGUCUAUUGUCAAAAGUACAAAUGUAGCGUAAAAUGAGAAUCAGUUUUAAACAAUU
Ime-1-1	GUUCUAAUUUUUAAAAUAUAUAGUUAGCGUGCAUCCUUGACUUUACCACCCAAUAUGCUAUAUUUAGAUGAAAUUCAAU
Esp-4-1	UUAAUUACGUAUACAAAUCUAUUGCUAAUUGGUUUGUUAUAGAGUAGAAUGGGAAUCAGUUUUAUAAACGUAAAGUCAA
Eur-2-1	AUAGAGAACGACUAUUGCCAAAUCAUGGAUUGUGUAGUAGGAUGAAAAGUGACGUUAAUACAUUGAAAUAAUAAAAUUA
Ewa-1-1	.....UAAAAAAAGAAUCUCUCUAUUGCCAUAUGGAUUAUUGUGGCGUAGAAUGAGAAUCAGUUUGAACAACCAAAUUA
Esi-1-1	GAGAGGCGCGCUAUUGCCAAAUCGCUUAUAGUGAGGUAGAAUGAGAAUCAGUUUUAGCCAAUUGAAACAUUAAAAUUAA
Eha-2-1	CGGAAAAAUCAACUAUUGCCAAUUCUGUUAUUAUGGAGUAGAAUGAGAGACAGUUUAAAUAGACUAAAUACUUAAUUAA
Epl-1-1	AACAAAGAACGGGGCUAUUGCCAAUUCGUUUAUUAUGGCGUAAAAUGAGAAACAGUUUUAAGAGAAUGAAUACAACUAA
Emo-2-1	AGAGGAAUUUGCUAUUGCCAAAGUAUUUAUUGUGGAGUAGAAUGAGAGACAGUUUCAGUUGAGGAAAUUACAACAUUAA
Eur-1-1	GAGAAUUUGGCUAUUGCCAAUUUGUUUAUUAUGGAGUAGAAUGAGAAACAGUUUCAAUAGAUUGAACGAAUAAAACUAA
env-316	GAGAAAAAUUCUAUUGCCAAAUUCUAGAUUGUGGAGUAGAAUGAAAAACAGUUUCAACGAAUUAAUAGAAUAAAAUUAA
Equ-1-1	AAAACUAUUUUUACCUUGCUAAAAUACUUUUUUUAGAGUAGAAUUGGAAACAGUUUAAAUAAACCGAAAACAAAAUUAA
Eri-1-1	CCAAUGGGUGCACUAGCCUAAUUCAAAAAUGAAUAAUCUUUAUUGACUUUUAAAGGAUUCGUGCUAAAAUAAACAGAAA
Csp-19-1	GAAAGAGAUUUAUAUUUUAGAAAAAUAAAUAAAAAAUUGACAGAGAUAUUUUUUUAUGAUAUAUUCUGUAUACAAAAUUA
env-317	GAACCUCCCACAUAUUCUUUCUUUAAAAUAUAGACCAAAACAUUGACAAAGGCAAUUCUAUUUGCUAAAUUAUAUUCGA
env-318	UACCUGUGAAAAUAUUCUUUCUUUAAAAUAUAGACCAAAACAUUGACAAAGGCAAUUCUAUUUGCUAAAUUAUAUUCGA
env-322	UACCUGUGAAAAUAUUCUUUCUUUAAAAUAUAGACCAAAACAUUGACAAAGGCAAUUCUAUUUGCUAAAUUAUAUUCGA
env-323	UACCUGUGAAAAUAUUCUUUCUUUAAAAUAUAGACCAAAACAUUGACAAAGGCAAUUCUAUUUGCUAAAUUAUAUUCGA
env-325	AUUUCAAUGGUUUUCUGCCGCCAAAGAUUUUACGGAAUUUGCUGUGCUGCGGUUGACAACCGCAGGUCUUUUCUGUAC
env-327	GAAAAAGAAAUGCAGGAAAAGAAAAUGCAGGAAAAAAAUGCUUGACUUCGCAUAUCUGACUGUGAUAUAAGUAGACACAA
env-330	AUUUUCAAAUACUCCUAUAAUCAAAUGCGAAUACCGGAAAGCAGAAGGAAAUAAAUUUGAUUUUACGGUUCAUGUGUGA
Cje-1-1	AUUUUCAAAUACUUCUAUAAUCAAAUGCGAAUACCGGAAAACAGAAGGAAAUAAAUUUGAUUUUGCGGUUCAUGUGUGA
env-331	AUCGUUCAUAUCACAUGAUAAGAAAUCGGGGGAAUGUUGGCUUUGGACCAAUGUUCUCCCGGUAUUGUGAAAAAGAAUU
env-332	UCGUUCAUAUCACAUGAUAAGAAAUCGGGGGAAUGUUGGCUUUGGACCAAUGUUCUCCCGGUAUUGUGAAAAAGAAUUA
env-333	GAGUGUAUUGAAAGUAUAAUAAAAUAAUAAUUAAUAGAAUCAUUGACAAAGAUAAUAUAAUAUUAUAAAAUCUUAUAAA
env-335	CCGGAAACUCCUCAUAGCUCCAUUGCUUUAUUUCCAUGAUCUUUCCUUGCUUUCCUGUUUUCUCUGUGUUAUCCUUCAU
Lsa-2-1	UUCAAGGAUGGUGAAGUGAUUACGGUUAAAGGUUAAGCUUAUAGUUCCUUGCCAAACACCUCUAGUUUUGCUAAUCUAU
env-336	CAAAAAAAAAUGAAAUCCGCUCUUGACAGCCGGGGAAUAUUAUGGUAUACUCUGACCGUGGUUGUAUUAACUAUUUAGU
env-337	UUAGACUAUAGGGGCAACAGGGUAAUCUGCGAAAAGUGAUUUCGAGCAUGCCCAUUGCCAGUGCUUUAGAUAUAUGCUA
env-339	UUAGACUAUAGGGGCAACAGGGUAAUCUGCGAAAAGUGAUUUCGAGCAUGCCCAUUGCCAGUGCUUUAGAUAUAUGCUA
env-340	UGCGCGCGUGACAAGGGAAAGGCGCGUCGCGCGAGAGAUUUUCGCCCGUUCUUGACAGGUGAUCGCGCUUUUGCUAAGA
env-341	AAGCAUAAAGCUGUAUAUUGUUAAAUUUGACAUAUAACUAAGGUUGUAAUAGAUUCGGUUAUAUGUUAUAAUACUCCCU
env-342	AAGCAUAAAGCUGUAUAUUGUUAAAUUUGACAUAUAACUAAGGUUGUAAUAGAUUCGGUUAUAUGUUAUAAUACUCCCU
env-343	..................................AACGCCUUCCAUUGGCUUUAAAGCGCAUCAAAGCAUUGGCUGAGA
env-344	..................................AACGCCUUCCAUUGGCUUUAAAGCGCAUCAAAGCAUUGGCUGAGA
env-347	..................................AACGCCUUCCAUUGGCUUUAAAGCGCAUCAAAGCAUUGGCAGAGA
env-348	..................................AACGCCUUCCAAUGGCUUUAAAGCGCAUCAAAGCAUUGGCAGAGA
env-349	......................CCGACGUGCACGAGAUAGUUAAAGAGGUUUUCCUUAAUCUUAAAUCCUGAUCUUGCU
env-350	......................CCGACGUGAACGAGAUAGUUAAAGAGGUUUUCCUUAAUCUUAAAUCCUGAUCUUGCU
env-351	......................CCGACGUGAACGAGAUAGUUAAAGAGGUUUUCCUUAAUCUUAAAUCCUGAUCUUGCU
env-352	......................CCGACGUGAACGAGAUAGUUAAAGAGGUUUUCCUUAAUCUUAAAUCCUGAUCUUGCU
env-353	......................CCGACGUGAACGAGAUAGUUAAAGAGGUUUUCCUUAAUCUUAAAUCCUGAUCUUGCU
env-354	UAAAGUUUAUUUACAUAUAAACAUUUUAAAACAAACAAAGCUUUUAAAAGAUAUUGACACAACCUCAGUAAUAUGAUAG
env-357	....AGGCAUAUAAAGAAAAAUGGCAGGACUACGAUGAAUGU
env-358	....UAUCUAUAAUGUGUUAU
env-359	.....................CAGUAUUUAAAAUUGAAGUUACAAGUAUCAGUUGUAAAGAACAUCUAUAAUGUGUUAU
env-360	GUUCUCAAUUAAUAAGGCUUUAAACUCUUAUAUUAAUUUUAGAACUGACAAACUUGAGAGAAUAUCUAUAAUGUGUUAU
env-362	....ACAAACUCGAGAGAAUAUCUAUAAUGUGUUAU
env-363	.....................CAGUAUUUAAAAUUGAAGUUACAAGUAUCAGUUGUAAAGAACAUCUAUAAUGUGUUAU
env-368	GUUCUCAAUUAAUAAGGCUUUAAACUCUUAUAUUAAUUUUUGAACUGACAAACUCGAGAGAAUAUCUAUAAUGUGUUAU
env-372	GUUCUCAAUUAAUAAGGCUUUAAACUCUUAUAUUAAUUUUUGAACUGACAAACUCAAGAGAAUAUCUAUAAUGUGUUAU
env-373	GUUCUCAAUUAAUAAGGCUUUAAACUCUUAUAUUAAUUUUUGAACUGACAAACUCGAGAGAAUAUCUAUAAUGUGUUAU
env-374	....AUCUAUAAUGUGUUAU
env-375	GUUCUCAAUUAAUGAGGCUUUAAACUCUUAUAUUAAUUUUAGAACUGACAAACUCGAGAGAAUAUCUAUAAUGUGUUAU
env-376	GUUCUCAAUUAAUAAGGCUUUAAACUCUUAUAUUAAUUUUAGAACUGACAAACUUGAGAGAAUAUCUAUAAUGUGUUAU
Fnu-1-1	.....................CAGUGUUUAAAAUUGAAGUUACAAGUUUAACUUGUAAAGAACAUUUAUAAUAUGUUAU
env-387	.......UACUUUUAAUACAGUUAUUUUAUCAUAUAUUUUUUAUAAAAUCACUGUAAUUAUUUAAUUUUUUGUGAUAUA
env-388	....AUUUUUUGUGAUAUA
env-389	..........................UUAUCAUAUAUUUUUUAUAAAAUCACUGUAAUUAUUUAAUUUUUUGUGAUAUA
env-390	UUCUCAAUACUUUUAAUACAGUUAUUUUAUCAUAUAUUUUUUAUAAAAUCACUGUAAUUAUUUAAUUUUUUGUGAUAUA
env-391	UUCUCAAUACUUUUAAUACAGUUAUUUUAUCAUAUAUUUUUUAUAAAAUCACUGUAAUUAUUUAAUUUUUUGUGAUAUA
env-396	..............CAAGGGCUUCCUUGACAAUCCAAAUUCAAUUUUAUAUAAUUCAGACAUAGUCAGCUCGGGCUGAU
env-408	GUACGCGCCCGUCCAGACGCUUUGCCAAGAAGGUGGACGCUUGACAUUCCGCCCAUUAUAAAGUAAAAUAGCUUUAUCC
env-409	CUAAAAACGUUGCAAACAAACGGGCAAUACGUUUUGCAAACGGUUGACAAUAAAAAUAAAACAUAGUAUAAUACGCUUA
env-410	UAACAAAAAUGAACCAUUCUGAUUGAAUCCUUGAUUUGCCGGGACGCAAAUUUUUUUUGCGCAAACCGCGCAAUUAUGG
env-411	UAACAAAAAUGAACCAUUCUGAUUGAAUCCUUGAUUUGCCGGGACGCAAAUUUUUUUUGCGCAAACCGCGCAAUUAUGG
env-412	....CGCAAUUAUGG
env-413	UAACAAAAAUGAACCAUUCUGAUUGAAUCCUUGAUUUGCCGGGACGCAAAUUUUUUUUGCGCAAACCACGCAAUUAUGG
env-414	GAAUUUCCUUGAGAAGUUCAUUGAGAAUAUGGACAGGAGGUUUCAAAAACUUCUUGACGGAGAUAUUUUUUUGGAGUAA
Lsp-7-1	UUUCCGCGUUGACUUUUGCAGAUCUCUGUGAUAUGAUACCAGGGUCAGAAGUGCUGCAGGCUCAACGCCGCAGCAUUUA
	....
	....
	....
	....

```
env-415    UGAAGCUGUCUUCUCUCAAGAACAGGGAGGGCCCGGCCUUUCGCCGGGCCCUCCCUGUUCUUGACUUUCCGCGGAUGG
env-416    GGGGAAUAUGCCAAUACCAUUCGCUGCGAUGCGGUGGAAGCCCCGUCUUGACAAUCACAGACACAUUGUGAUACUCUGG
env-417    ..AGGUUCUUUGCGCAGCGCUGUCCCACUGCCUUUUCCAAGAGCGGUCUUGACAAAAGUGGAGGAGUGUGUUAGAGUAC
env-418    AAAUAUCCGCCUAUAAGUCGAUUAUCGCGCUUUUUUUGCGUUAAAACGGUUGACGAAAAUCGGGCUUAUAGGUAUAAUA
env-420    ACCCCGCAGGGCCAUUUGUCGAGAGGAUACUCUCUCUCCCUCUGAUCCGUUGACAGCGCAGGGCAGCUGUGCUAUGAUA
env-421    UGCGCAGAGCGCGGAAACCGGAUCCGCAUCCUGAAAGCGGAACAGAGACGAGCCCGGGCUUCUUGACAAAGGGCGCCGG
Bca-1-1    GGAGCAUCUUGGCAUCCUGGGGUAAGGAGCAGGGGGUUGACAUGCUCCCCGCCCUGUGGUAUCCUCUCUUACGACAUUU
env-422    ............................................................AAGCAUGCUAUAAUAUCC
env-423    CGAUGCGUGUGUAAAGAUCAGUCCAUAGGAUAGCCGAAGGAUAGAAUUGACAUGCAAAGAAAAGCAUGCUAUAAUAUCC
env-426    CGAUGCGUGUGUAAAGAUCAGUCCAUAGGAUAGCCGAAGGAUAGAAUUGACAUGCAAAGAAAAGCAUGCUAUAAUAUCC
env-428    UUACCAUUGACACUCCACAUUAAUUCAUGUAUGAUACUUUCAGAAAGCAAGGCGGCAGCAAUCCAUAUGCCGUCUGGAA
env-429    UUACCAUUGACACUCCACAUUAAUUCAUGUAUGAUACCUUCAGAAAGUAAGACGGCAGCAACUCUUAUGCCGUCUGGAA
env-430    CCUUUUGUCAGUUUUGAGGCUUGAAAUUAUUCAUCACAAUUGAUACAAUUAUAGAGGACAUAGGAAAUGACUAAAUAUC
env-431    .........GAAUUCUUGGCAGAAAGCGUAAAUAUGUUGAAAUUGGCUUGCAAAGCUGAUAAAAUUAAUCAAACCAAA
env-432    ...................AUUUAUGGCACUUGCUGUAAUUGGGAAAAAACGCAGAACUGAUUAAAAAAAUAUAGUAA
env-433    ..............................................................................
Bsp-1-1    UAUCUUACUACAGGAAAAUGGGAAAAUCAAUUCUAAAGCAAAAGUCUUCUCUUGACUUUUCCGGUUCUGUGUGCUAUUU
Lpe-1-1    CUCGUUACUUUAGUGACGAGUAGUUCACAAAACACACGUUAAGUAGUCAGUAUUAGAAAUAUGUAUGCUAAACUUAUCA
Lfu-1-1    AAAAUUGCCAGUUACCAAGGCCACCGAGCAAUGACUUAUUAGGUCAAACUUGACCCCCGUAUUAAAAAUUGCUAAACUA
Lde-1-1    UGAAUAUUUAUGGUAAAACCAAUUGAAACCACCACAAAUUAGCCAGUUAACAAGUUAGUUUAAAUUUGCUAAGUUACUU
Lfa-3-1    CUUGAUAUAAUGCUUACCUAUUUCAUUACUACUUAAUCAUAAAUCACUUAACACCUUGACUCAUGUACGUUAAUUUGUA
env-434    GAUAGCUUGUAAACUACUUAUCUUGAAUAAAUCGUCUAAUUAUUUAUUGCUUUUUCCCAAAAAGUAUAGUAAGUUUUAG
env-436    CACUAUAUGCGAUUGAACAAAUGAUAACAACGAUACCAAUGAUGCUUUUUAUUGACCUUCUGUUCUAAACGUGCAAUAA
env-437    CACUAUAUGAGAAUGAACAAAUGAUAACAACGAUACCAAUGAUGCUUUCUAUUGACCUUCUGUUUUAAACGUGCAAUAA
env-439    ...UAUAUGAGAAUGAACAAAUGAUAACAACGAUACCAAUGAUGCUUUCUAUUGACCUUCUGUUUUAAACGUGCAAUAA
Esp-11-1   UAGUGUACGACACUAUAUGGGAUUGAACAAAUGAUAACAAUGAUGCUUUUAUUGACCUUCUGUUUUAAACGUGCAAUAA
env-440    CACUAUAUGAGAAUGAACAAAUGAUAACAACGAUACCAAUGAUGCUUUCUAUUGACCUUCUGUUCUAAACGUGCAAUAA
Lsp-1-1    UGGCGCAAAGAAGCAGCAUAACUGGGCAAAAUAACCUUAGGGGAAACUUGAAUUACCAUUAUUUUUAUAAUAAAAUUUA
Lsp-5-1    UUGUCAACCCUGUCAAAAAUUUGGCACGAAGAAAAGAAGGCAUAAUUAUAUUUGCAUUUGCUGGUUUUUUAUAUUAAAA
Pac-2-1    UUGUUGAGUGAAGUGAUUUUAGCAAAGCUGCGGUAACUGGCAAAGUUGCCAAUGGUUUUGAAAGUUGUUAUGAUAUCCU
Eth-1-1    UAUUCCUCUUACAAUUUUGUUGGAUAGUGUUUUUUUAUCUUUAGAAAUGCCAACCUAUUUAAAAUGUGCUAUACUGACU
Efa-295-1  CUAUGCUGUAUCCGUCAAUGCUUGAUACACCUUACUAAGAAGAAUCAGAUUGCCGAUAUCUGGAUAAGUGUGCUAUACU
Esp-3-1    UAAUGAUGUAUCUGUCAAUGCUUGAUACACCUUACUAAAAAGUAUGAGAUUGCCGAUAUCUGGAUAGGUGUGCUAUACU
Efa-294-1  CUAUGCUGUAUCCGUCAAUGCUUGAUACACCUUACUAAGAAGAAUCAGAUUGCCGAUAUCUGGAUAAGUGUGCUAUACU
env-441    .......................................UUUUUUUCAUUCCCCAAAAAAAUCCGAGUGUGCUAUACU
Ega-2-1    AUUUUUAUCCGAAAGUGCUACCCCACAUUUUUUCGAUAAGUUUUUUUCAUUCCCCAAAAAAAUCCGAGUGUGCUAUACU
env-442    AUUUUUAUCCGAAAGUGCUACCCCUCAUUUUUUCGAUAAGUUUUUUUCAUUCCCCAAAAAAAUCCGAGUGUGCUAUACU
env-443    AUUUUUAUCCGAAAGUGCUACCCCUCAUUUUUUCGAUAAGUUUUUUUCAUUCCCCAAAAAAAUCCGAGUGUGCUAUACU
Ega-1-2    AUUUUCAUCCGAAAGUGCUACCCCUCAUUUUUUCGAUAAGUUUUUUUCAUUCCCCAAAAAAAUCCGAGUGUGCUAUACU
Ega-1-3    AUUUUUAUCCGAAAGUGCUACCCCUCAUUUUUUCAAUAAGUUUUUUUCAUUCCCCAAAAAAAUCCGAGUGUGCUAUACU
env-444    AUUUUUAUCCGAAAGUGCUACCCCUCAUUUUUUCGAUAAGUUUUUUUCAUUCCCCAAAAAAAUCCGAGUGUGCUAUACU
Lta-1-1    AAUUUUAAAAAAAGAGUCGCUGGUCAACUAACAGAGAUUCACUGAAUAACUAGAAUUCUAAUAAUAAGUUUGCUAAGAU
Lcr-1-1    UAAUUUGGAAAAAGAGUCGUUGAUUAACUAACAGAGAUUCACUGAAUAACUAGAAUUCUAAUAAUAAGUUUGUUAAGAU
Lul-1-2    AUAUCUUGCAUUUUUACUUCGCCUAACUUUUUUCUAUUAUUCUCUCUUUAAUUAUAAACUUUGCUUUGCUAUACUAACU
env-448    ..................ACAGCGCUAGGGAGAUACCAGGACUGAUUGAACAGGGCAGACUUAUGUGAUAUUAUUCUG
env-449    .AAUUCAGCCGCGAGCUGCAGGAAAAAGUCAGAGAUCAGAUAAAUAAGUAGAAGUAUAUAGCUCCGGAUGUAUCCGGGU
env-450    UCAAAAUAGUUGACAACCCAGAUAUAUGAUGUUAGAAUACAUAACAGUUUUUACUCAUCGUUUAACUCAAACGAUAUAA
env-452    CAUACACUCCUAUCCAAAAACAAAAAUUAGACAAGUUCAGUAACUCAUGUUACAAUAGCAAAAUAAGUUAGUAAUUACA
env-454    UUCCUGUCACUCAUCAUUGUAACUCCUACAGACCUCUUCAGCCAUGCCAUGAAGAGCGCUUGACAAUCCAAAUUUUAUA
env-456    UUGUAAAGUGACGCGGCAAAUGCGGCGGCAGACCUCUUCAGCCAUGCCAUGAAGAGCGCUUGACAAUCCAAAUUUUAUA
env-457    UUGUAAAGUGACGCGGCAAAUGCGGCGGCAGACCUCUUCAGCCAUGCCGUGAAGAGCGCUUGACAAUCCAAAUUUUAUA
env-459    UUGUAAAGUGACGCGGCAAAUGUGGCGGCAGACCUCUUCAGCCAUGCCGUGAAGAGCGCUUGACAAUCCAAAUUUUAUA
1 env-460  ............UCGGCGAUAAUAUGAUGAAAAUGCAGAUCGUUCGGCUCGACUUUGCUUGACAAGACCUCUUCCAUA
Rti-1-1    GAUGCAUGCUGCUUGAAUGAGCAGCGGCUAUGCAGUUUUGGCGAAUACCCUAGUAUGAUGUUACCUAAGAGGGUAGAAU
Rma-1-1    GAACCUCUGACGCCUGCCGAUCAGGAGCUAUGCGGUUUUUGCGAACAGUAUAGUUAGCUGUUACCUAAAAGGGUAGAAU
env-461    UUAUUAGUGAUGAUUAGUAUUUUAUAUACAGGUACAACGCUUUCUAAAAUUUAGUUAUGUGCUAUAAUGACUAAAUAGC
env-462    CAGCAGGUGCUUUCGGCAGCUGCAGGGAAGCCGGCGGAUGCGGCUUUUUCCUUGACGCCAUUACGAGGAAGAUGAUAGG
env-463    AGCUUGUCAAAAAAGUCCGGUUUGGACUUUUUUCUCGGCCCUCCACCCCGCCGGGGCGCUUGACAGGGCCUUGUCCUUU
env-464    AUUGCCGAUGUUUUUUCUGUAAACAACAACGGCUAAUGAAAUUGCACGCAUUGGGGUCGUUGACAAAGGUCUCAAUGCG
env-465    AAAGUGCUUCUAUCUUGCCCCCCUUGGUAUUGAAAUACCGUUUGCUGAAGCUCCGCUGCUUGACAAAAGAUGUUGCUUU
env-466    AAAGUGCUUCUAUCUUGCCCCCCUUGGUAUUGAAAUACCGUUUGCUGAAGCUCCGCUGCUUGACAAAAGAUGUUGCUUU
env-467    AAAGUGCUUCUAUCUUGCCCCCCUUGGUAUUGAAAUACCGUUUGCUGAAGCUCCGCUGCUUGACAAAAGAUGUUGCUUU
env-468    GGCAUCCGGUACUUUUAUGCUGCCGCUGAUAUGGAAUCUUAUUCGCUGCGGACCCCACACUUGACAAAUGGUGCCGCCU
env-469    ............UUUAUGCUGCCGCUGAUAUGGAAUCUUAUUCGCUGCGGACCCCACACUUGACAAAUGGUGCCGCCU
env-470    UUGUCCAAGAUGCAUUGUAGUCCUAUAAGUUUUCGAGAUGUACACGGUGCGCUGCCUGCUUGACAAAAGGUGACGGAUU
env-471    ..............................ACCCCAAGCAGCUGCGGAUCUGUGCAUCGCCAUGGUGGACGAACACUG
env-472    CAUGGACUGAAAGAGAAAGACCGAGGUGGUUUUCCACCCCGGUUUUUUGCUCUCCGCGCUUGACAAGCCACGCCUUAUA
env-473    AUGGACUGAGGAACAAAAAAGACCGGAGUGGUUUUCCACCCCGGUUUUUUGCAUUUCUGUUGACAAAGCGUAUCGCGCG
env-476    GACGUUUUUCAAGGGGGUCAACUGGGCAAAAAGUAAUGGAAAGCAGGAAAAUCAUGCGCUUGACCUGGGGAGGGAAAUA
env-477    GACGUUUUUCAAGGGGGUCAACUGGGCAAAAAGUAAUGGAAAGCAGGAAAAUCAUGCGCUUGACCUGGGGAGGGAAAUA
env-478    GUCAUUUUGCGCUGCGGCUUUUAUAAGAGAGAAUUCCUCUGCUGGAUAGCGUGUGUUCUUGACAAUGCGGUGAUGUUUG
env-479    ..................................................................CGGUGAUGUUUG
env-480    GAUCAAGGGGAGAUGACCCGGAGGGGACUUUCCCUUGUCUGCGGUUCCCCGGCUGGCGGUUGACAGAAGGCGGAAAGUG
env-481    UUUAAGAGUGAUUAUUAGGAUUAUUUAUUUGUUCCUGUCUGCGGUUCCCCGGCUGGCGGUUGACAGAAGGCGGAAAGUG
env-482    GAAGUAAGCGUUUUCGGAUCAUAAAGCAAGCCCCCGGAGGAUCUCCGGGGGCUUUUUGCUUGACAAUUGCCCGGCGGUG
env-483    .....................................CUGCGGGCGCGGCACGGCUUGACAGGGCGGCUCCGCCUGUG
env-484    ....................................................................CCCCGCAUGU
env-485    CAUAGUUUUCAAGGGAUAGCGCGGCGAAAAAAUGUGCUUUAUACGAAAAAACUGCCCUUGACGAGGGAAAAUAAUUGUG
env-486    CAUAGUUUGCAAGGGAUUGCGCGGCGAAAAAAUGUGCUUUAUACGAAAAAACUGCCCUUGACGGGGGAAAAUAAUUGUG
env-487    CAUAGUUUGCAAGGGAUUGCGCGGCGAAAAAAUGUGCUUUAUACGAAAAAACUGCCCUUGACGGGGGAAAAUAAUUGUG
env-488    CAUAGUUUGCAAGGGAUUGCGCGGCGAAAAAAUGUGCUUUAUACGAAAAAACUGCCCUUGACGGGGGAAAUUAAUUGUG
env-492    CAUAGUUUGCAAGGGAUUGCGCGGCGAAAAAAUGUGCUUUAUACGAAAAAACUGCCCUUGACGGGGGAAAAUAAUUGUG

           ..............................................................................
           ..............................................................................
           ..............................................................................
           ..............................................................................
```

```
env-493    CAUAGUUUGCAAGGGAUUGCGCGGCGAAAAAAUGUGCUUUAUACGAAAAAACUGCCCUUGACGGGGGCAAAUAAUUGUG
env-494    CAUAGUUUGCAAGGGAUUGCGCGGCGAAAAAAUGUGCUUUAUACGAAAAAACUGCCCUUGACGGGGGAAAUUAAUUGUG
env-495    UCCAUGUCACCCCGCGGGAACGCAAAGCAAAAUCCGGCCGUUCCUCUUGACCUUUUUCCCUCCCUUUGCUAUAAUAAAG
env-496    UCCAUGUCACCCUGCGGGAACGCAAAGCAAAAUCCGGCCGUUCCUCUUGACCUUUUUCCCUCCCUUUGCUAUAAUAAAG
Lgi-1-1    ..............UCUUCACAUUCAUUGUUAGGAAGUUUCCAAUCACAGUUAGUGAAAAUUAAAUAUUACAUAUGAUA
Lve-2-1    ...........UUUUCACUUUUAUUGUUAGAAAAUUCCCAGUCAUCAACGUAGAAGACUAAAUUUUUUAAAUGUGCUAA
env-497    .....AGAUCUGAGCUACUUGUUCCAAUUGAUCAGCUGUCAUUUUUUGAAUCAUGUUUGAAAUCUCCUUUUAAUUUGCU
env-498    AAUCUGACUGACUUUUUCCAAUUCCAAAUCUUUCAUUUUUCGAAUCAUACUUGCCAACCUUUUUUCUAUUUGCUAAAAU
env-500    ..CAAAUAUUACUGACUACUUCAAUUUCAUCUUUAUGCAUUUUACGAAUCAUAUUUGCCACGUCCUUAUUUAUUUGCUA
Lna-2-1    .CAAAUUUCACCUACUACAUUAAUUUCAUCUUGUCUCAUCUUUCGAAUCAUGUUUGCCAUCCAUCCUUAUUUUUGCUAA
env-501    ACCAAAUCUGUCCUAUUCGAUCAAGUUCUUUUUUUGCAUUCUUCUAAUCAUAUUUGCCAAUCACCUUUUAAUUUGCUAG
env-502    .CAAAUUUUACUAACAGUAUCUAUCUCGGAAUCUUUCAUUUUACGAAUCAUAUUUGCCAACCACCUUUUAAUUUGCUAG
env-503    .............................................................................GA
env-504    .CAAAUAUCACUAACAACUUCAAUUUCUUUCGGUUGCAUUCUUCGAAUCAUGUUUGCCAUUUCUCCCCAAAUUUGCUAU
env-505    ..........CUAACAACUUCAAUUUCUUUCGGUUGCAUUCUUCGAAUCAUGUUUGCCAUUUCUCCCCAAAUUUGCUAU
Lno-1-1    ACUUGAUCAAUUUCAUCAAGUGUCAUGUUUUCAAUCAUUAUUAAACGAUCCCCUAGCCCUAAAAUAUUUAAUGUGGUAU
Lal-2-1    CAGAGUCAAUUUCAUUGGGUGUUAUUUUUUCAAUCAUUUUAAAUAUAUCCCCUAGCAAUAAAAAAUUUGAUGUGCUAU
env-506    ...................AUUCAGUCUAUCCAAAACCAACUAAACAGACUCAACUUGAAAUUAUUUAAUCAAUUUGCU
Lal-1-1    .............AUCUUUUAACAUUUAUUGUUCGCAAACAUCCACUUGUCCCUGCAGAAUAAUUUGUCACCAGCAACU
Whe-1-1    GGUACAUUUUAUUUUGCUAAUUAAUCAGUAAAUUAAAAUUUUUAAUGAUUAUGACUCAACUUAAUUUCAAGAAUAUGUU
Whe-1-2    UACAUUUUAUUUAUGUAAUGAAUAAAUAUAUUCAAAAUUUUAAUGAAUAUGACUAAACUUCAUUUCAAAAAUGUGUUAA
Lpa-1-1    CAAAUAUCUGCGGUUCCACAACUUGUCGAUAAUUUACCAAAGUUAAACUGAUUGGCAAGUCAGUUCCAAUGUGCUAUGG
Lpa-2-1    CUUGCGCAGAUUGAUGCGGUGUAUAGAGUUCGAGUGUUGCAGAAGUAAAUUCCCUUUCAAUUCCAAAUGUGCUACACUA
Lra-1-1    GAUUAACCGUAAAUUGAAGCAAAGUCAAUAAAGCGCCAAAAAGAGAUUAAUUCCCUUUGACCUAAAAAUGUGCUAUCAU
Lki-3-1    UCAAAAGGGAGAAAGGUUAAUCAGCAAAUUGAAUGAACAACCUAUCACCGUUUCUUUUACCCACAGAAUGUGCUAUGAU
Lki-2-1    UCAAAAGGGAGAAAGGUUAAUCAGCAAAUUGAAUGAACAACCUAUCACCGUUUCUUUUACCCACAGAAUGUGCUAUGAU
Ldi-1-1    UCUUAAUGGUGCUUAUGGACAGUCAAGCCUACCCCAAUCAGUUCACACUAAUUUGUUUUCUCUCCCAAAUGUGCUAUGA
Lhe-1-1    GCUGUAUUUCGGGUUCGCGUGCUGAAACAUUAAUUAAGUUACGCAAAAAAUUGGCCAGCCAUCUUCAAAUGUGAUACGC
Lfa-2-1    GCUGUAUUUCGGGUUCGUGUGCUGAAACAUUAAUUAAGUUACGCAAAAAAUUGGCCAGCCAUCUUCAAAUGUGAUACGC
env-508    UUAACGACCAACUUAGAAAACAGUAACUCACUCAUGAUAUCACUAACUAAUUGGCAAGUAAUCCCAGAUGUGAUAUUCU
Nma-1-1    ...............................ACUCAAAAAGUCGUGUUGCAAAGACAGAAAGCAUCACGAUUCAAAAAU
env-509    GGUAUGGGGAAAACUCCUUAUACUACUAUUAUACUGAAACCGGCAGAAGAAAUCAAUAGCCAUUGACAAAAAAGCUGAG
env-511    GGUAUGGGGAAAACUCCUUAUACUACUAUUAUACUGAAACCGGCAGAAGAAAUCAAUAGCCAUUGACAAAAAAGCUGAG
env-512    GGUAUGGGGAAAACUCCUUAUACUACUAUUAUACUGAAACCGGCAGAAGAAAUCAAUAGCCAUUGACAAAAAAGCUGAG
env-514    GGUAUGGGGAAAACUCCUUAUACUACUAUUAUACUGAAACCGGCAGAAGAAAUCAAUAGCCAUUGACAAAAAAGCUGAG
Csp-12-2   AAAUGGUUUAAUAAUACUAAAUCAAAGUAAGCUUACGAUUGAAAUUAUUUAUAAAAGUGUUAUAAUAAGUUUGAAAAUA
Csa-1-1    UAUGUGUAAUUAUGGCGGGGCUUUAAAAAUGUUACAUGAUUGAAAUUAUUUAUGAAGGUGUUAUAAUAAUUAUAAAAAU
env-515    UAUUUGCAAAUGAUUUUUUAGUAAAUGUUAAAAUUCUAAGGUAUAUUGACUGCCAUAAUGUCUUGUGAUAUAAUGUAAA
env-516    CAGAUGGCGAAGCUAUAUGGAGAACUUUAAAUAAUAGUUGACUUUAAAAUUCCUAUGUGAUACUAUGAGGUAAAUUAAA
env-517    UGACCGCGGUCACCGCCGGUAUCCUGAUCUCAAAGCUGCUCAAAAAAUAGCAGGCGAUCCGCUUGACAGAAGGCUCGCC
env-518    AUGGAGGAAACGGAGCGGAAUGAAGGCAGAGCUUGACAGCCGGAUCCUCUUAUGAUAAAUUUGACUAAGAUUUUUUUAU
env-519    .....UUGUCGUGAGCGGAAUGAAGGCAGAGCUUGACAGCCGGAUCCUCUUAUGAUAAAUUUGACUAAGAUUUUUUUAU
env-520    AAAGAGGCUUCACAGCGAGACUUCUUCACCCGCCGUCAGGCGGGCUUUACUGCUCUUGACAGUACAGCUCUUGUGUGCU
Bsp-1-2    AUCAGGUCUUUUUAGAAACCCUUGGAGGGAAAAUAGAGGAAUGAGCUUGACUUUUUCCGUUACAUGUGCUAGGAUGAUG
env-521    GUAAUAAAGUAAUCAUAAAAAAUACUAGUUGUAAAUCAUAAAAAAGUAUAGUAAGAUAGAUUUUGACAAACAAGCACAG
env-522    ............................................AGUAUAGUAAGAUAGAUUUUGACAAACAAGCACAG
env-523    AAGAUUAUAAACGAGACUCAGCUUGAGUAAUCUAGCGAUUUAAUAAGCAUAUUAUAAGAUGCAGUAAUAUUCUUUUUCU
env-524    GGAGUGGCAAAAACGCCACUCCCCUUUUAUACUCGCUUAUGCGUUCAAAUCCGUUGACAUUAUAAACAUAGUCUUAUAU
env-525    GGCAAAACGUCACUCUCUUUUAAUAUGCUUUUGCUCGUCCAAAUUCGUUGACAAUAUAAAUAUCGUCCUAUAUAAUCCA
Msp-5-1    UUGGACAGUAUUUUUUUCAGUGAAGGACGAAGGUGUUGACAAAGAAAUUUUUCUGUGCUAGAAUCCUUCUCAUAGCAAG
Lsa-1-1    .GGGUCUUAACAAUGGUCGUCACUUACUUAAUCGGUGGUUCGAUCGGUUAGGCUUUUCUUUGGUAACCAAAUUUGCUAU
Lcu-2-1    .GCGUCUUAACCAUGGUCGUUACCUAUUUAAUUGGUGGUUCUAUCGGCUAAGCUUCACUUUUAACCUAAAAUUUGCUAU
env-526    .GCGUCUUAACCAUGGUCGUCACCUAUUUAAUUGGUGGUUCUAUCGGCUAAGCUUCACUUUUAACCUAAAAUUUGCUAU
env-527    .......................UAGUAUUGGGGUUAUUAAAUCUUAAAAAGAAACCAAAACAAGUCGAAUAAAAAAAA
env-528    CUGGACGGCAUGUGCGAAUGGGGAGACCGGCACCGGCUCUGACCGCCUUGCCGGGAACCGUCCCCCGUGGUAAGAUACA
env-529    UGCCCAUCCUGGACGGCAUGUGCGAAUGGGGCGACCGGCACCGCCCGUGACCUUGCCCCGCCGCAGCGGGUGUGCUACA
env-530    ..UACAUAUAUGACAAUGUUGCAGUGGUAAUAAAAAAUAUUUGACCUUUUAAUUAAUAAGUAGUAGAAUGUUUGUUGGU
env-531    GAUACAUAUAUGACAAUGUUGCAGUGGUAAUAAAAAAUAUUUGACCUUUUAAUUAAUAAGUAGUAGAAUGUUUGUUGGU
env-533    UUGUACUCAGGAUACUUCCCUUUUGAGCGUUAGACUAUACUUGGUGCGCUGGCACUUGACUUGCCAAAAACACUAUGUU
Csp-6-1    AAAAGAUAAAAGAUAUGAUAUACUAAGUCCGUUCCACAUUGCGUCUGUAUGGUAAUAUUGGUGUUAUGGAACAAUAAUU
env-534    GGUCAGGGAGCUGUUUGCCGAACACGCGAAAUCGUGUACCUAGAUAUUGACUUUCUCAGUCAAUAUGAUAUUAUCUAUA
Cba-3-1    ACACCUAUCUAUACUAAUCUUAUUGACGCAAUUUGUCAAUAAAAAAAUAAAUCUUGACAGUCAGGUAAAGCCUAUGUUA
env-535    AUGUCUAUAACGUCGCGCCGCAACAUUCCCCCGAGAGUCGCGUCAAAGCUCUUGACAAUGGCGGCGAGAUGUGAUAUAC
env-536    AUGUCUAUAACGUCGCGCCGUAACAUUCCCACGAAAGCCGCGUCAAAGCUCUUGACAAUGGCGGCGAGAUGUGAUAUAC
env-537    AUGUCUAUAACGUCGCGCCGUAACAUUUCCCCGAAAGCCGCGUCAAAGCUCUUGACAAUGACGGCGAGAUGUGAUAUAC
Csp-11-1   UUUUCUAUAUUUCAAAAACUUUUAGAUUUAUGUUUCAUUGACAAAUCUCAAUUCAGUGUGAUAUCAUUUAAAGAAUUUA
env-538    ...............................................................................
env-545    ACGUGCAUGCGCUGCGUGACUAUAAUGGUUUUAGCCAUUGACAAAGUUCUAGACACCUGCUAUCUUGGUGUGGCUGACA
env-546    GUUCACCCAAGUUAAAGUGGAAUUUUUUUGUUGUGUACUUAUAUUGACACUAUCAGUUAUGUGUGUUAAUUUUAUAUAA
Rma-2-1    CUAUAUAAUAUAAAUUAAAGUGUUAAUAGGUUUAUAUAUUGAAAAACUAGUAGAUGCAUGAUAUUAUUUAAAUAUAUAA
env-547    UUUAAAUACUUAUAUGAAGGUUAAAUCUUUUUUAUACAUUGAAAAACAACUAGAUGCAUGAUACUAUUUAAAUAAUUAA
env-548    AUGCUUUGAUUUUGGAAGGUAAUUAUUUAAAUGUAAAUAGUUUAAAAAUGAAAUAUUUUAUGGUAUAAUAAAUAAAAAU
Csp-7-1    AUGCUUUGAUUUUGGAAGGUAAUUAUUUAAAUGUAAAUAGUUUAAAAAUGAAAUAUUUUAUGGUAUAAUAAAUAAAAAU
env-549    ..............................................................UUACGCAAGCUCAAAAU
env-550    AAUUAUUUUUAAUCAAUAGUAUAAAAUGGAUUUUGGGUUUUAUCUUCCCUUAUUCUUAUGCUAUACUAUAAAAGAAUUA
env-552    .................UCGGUGCAGCUUACGGUAAAGUGGCGGGCCUAUUUCAGAAGGAUGAGUGAUUGAGCCUAGUC
env-553    GAAAAAGAAGGUUUCUUUGUUCUGUAUUCCUUUUUAAGUUGACAGGGGAAGGGUUUUUUAUUAAAAUCAGAAGAUAGAA
env-557    GCAUCGGUGGGAAUCUUCUUCUUCAGCUCAUUUCUUGACAAGAAAUGACAUAUCCAUUAGAAUCAUACAAGUAAAUACA
env-558    CAUUACUGUGAUUAUAGCAAGAGAAGACUUGAAAGCGAUAUUUUGACUGUAACAUCAAAGUGGGCUAACAUGAGUUCGU
env-559    ...........................GCAGUCUAGUAUUGACGCUUUAGUAAAACUAUAUUAUUAUGAAUAAGUCUUU
env-560    AAGCUGUUAAUAAUCAGGUUUGAUAAUGCAGUCUAGUAUUGACGCUUUAGUAAAACUAUAUUAUUAUGAAUAAGUCUUU
env-561    CGAACUAUAAGCCUUUUUAAUCCUAUUUUUCGACAUUGACACAAUCCCAAGACCCAAUUAAAAUAGUAGCAUCUGAAUC

           ...............................................................................
           ...............................................................................
           ...............................................................................
           ...............................................................................
```

```
env-562    ............UAACUUGGAAGUUUAGCAUUUAGGAUUUAGCAAAAUAAAGGUUGAUUUUUCUUUAAAUAUGCUAAU
Cin-1-1    UUGUUCGAAUUUUAUAAUGAACUAGAUUUAGGCUGUACUUGACCAUCUAUGGAACUUGUAUUAUACUAUUUUGAAACGU
env-563    .............................ACAAUGAUGUAUCGAAACUCUUCCGUCCGGAGGAGUAACAAUACGUAGUG
env-564    .................................................................AACAAUACGUAGUG
env-565    GCUUGGCAAAUGUUCGUUAAGUACUCUAGGAAAAUCGUAUUGACAACUUAAAGGAACUAUAAUAAGAUUAGAAUUAAUU
env-566    UUUCCGGACUAGUAGAGAUUAUUUAUAGUAGUUGAAGUAUUGACUUCUCUUAGAAACUAUUAUAUUAUUACCAUUGUCA
Csp-14-1   CCCCAAUGCAAAAAGUUAGAGGAGAAUUGCGGAAUUGUUGUUGACUUCUUGAAAGAACUAUAAUAACAUGAAAUUAAUA
env-570    UUGUAAGUAGUUAGAAUAACCAGGUAGUGUGUUGACACCCCAAUAAAAUUAUAUUAAUAUAUAAAAUGUUUUGAUAAUA
env-573    UUGUAAGUAGUUAGAAUAACCAGGUAGUGUGUUGACACCCCAAUAAAAUUAUAUUAAUAUAUAAAAUGUUUUGAUAAUA
env-574    AGAAUAUAUAGUUGACAUUGCAUAAGUACUAUAAUAAUAUAAAUGUGGUUUAUAUAAAAACUUUAUAAUAUUCUUCUUU
env-575    AGAAUAUAUAGUUGACAUUGCAUAAGUACUAUAAUAAUAUAAAUGUGGUUUAUAUAAAAACUUUAUAAUAUUCUUCUUU
env-576    AGAAUAUAUAGUUGACAUUGCAUAAGUACUAUAAUAAUAUAAAUGUGGUUUAUAUAAAAACUUUAUAAUAUUCUUCUUU
env-577    .GAAUAUAUAGUUGACAUUGCAUAAGUACUAUAAUAAUAUAAAUGUGGUUUAUAUAAAAACUUUAUAAUAUUCUUCUUU
env-578    UCUUUUAACAAGAGGACUUGGAAGGAAAUUGAUAGUUGACAUAUGAGAAAUAAUAUAAUAAUAUUAUUAUUGUUUAUAU
env-580    GAAUGUAGCUAGUAGCAAGAUGAUUUAGUAGGACUAACAGUUGACAUUUUAGAAAAAUUAUAAUAAUAUUAUUCUUGUU
Lna-1-1    CAAAUGUUGUCUUUUUGUGGUCAUGUAAUUGAUUAUUAGGAAAACUUGAUUUAAAGCUACACUUGUCCUACAAUAGUAA
env-587    AAUGAAAUUUCAUAAAGACAGGGUUUGAGUCAGAAAGGAUUGACAAACAGUCAAAACUAUAUUAUGAUAAUUCUGGUUU
env-588    UAUGAAAUAUCAUAAAGAUAGGGUUAAAAUUACAAAGGAUUGACAAAUAGUCAAAACUAUAUUAUGAUAACUCUGGUUU
env-589    UGGUUUGAAAAGAACCUUUUGGAUAAAAAGGAUGAGUGAUUGACAAAUAAACAAAACUAUAUUAUGAUAACUCUUGUUA
env-591    UUGAAAAGAACCUUUUGGAUAAAAAGGAUGAGUGAUUGACAAAUAAACAAAACUAUAUUAUGAUAACUCUUGUUAUAAA
env-592    UAUGAAAUAUCAUAAAUAUAGGGUUAAAAUUACAAAGGAUUGACAAAUAGUCAAAACUAUAUUAUGAUAACUAUGGUUU
env-593    UAUGAAAUAUCAUAAAGAUAGGGUUAAAAUUACAAAGGAUUGACAAAUAGUCAAAACUAUAUUAUGAUAACUCUGGUUU
env-594    UAUGAAAUAUCCUAAAGAUAGGGUUAAAAUUACAAAGGAUUGACAAAUAGUCAAAACUAUAUUAUGAUAACUCUGGAUU
env-595    UUGAAAAGAACCUUUUGGAUAAAAAGGAUGAGUGAUUGACAAAUAAACAAAACUAUAUUAUGAUAACUCUUGUUAUAAA
env-598    UUGAAAAGAACCUUUUGGAUAAAAAGGAUGAGUGAUUGACAAAUAAACAAAACUAUAUUAUGAUAACUCUUGUUAUAAA
Csp-3-1    UAUGAAAUAUCCUAAAGAUAGGGUUAAAAUUACAAAGGAUUGACAAAUAGUCAAAACUAUAUUAUGAUAACUCUGGUUU
[gu-1-1    UAUGAAAUAUCAUAAAGAUAGGGUUAAAAUUACAAAGGAUUGACAAAUAGUCAAAACUAUAUUAUGAUAACUCUGGUUU
env-599    AUGGAUUAUUAUUUAUAAAGAAAAAUCAUAGGACUAAAGAUUGACAAUCAAUCAAAAUUGUAUUAUGAUAGCCUGAGUU
[in-1-1    GUAAUUUAGUUUUUUAAAGAAAAGAUUAAGCCACAAAGGAUUGACAAUCCGUUAAAACUGUAUUAUGAUAACUUGAGUU
Air-1-1    UUUUUAAUAAGUAAACUAUAGUAAAAAUGCCUGAUAAUUGACAUUUAGAGAAAAUAAUUGUACAAUUAAUAGGAUUUUA
Csp-5-1    GAACUUAUUAUUAAGUAAAAUGUGGCUGAUGCAAGUAUAUUGACAUACCGUGAAAACUAUAUUAGAAUUAAUAAUGCUU
env-600    ..AUACUAUGGAAAAAAGUAAAAAGUGACGCAGUAUUGACAAAUUCUUAAAUCUAUAUUAAUAUGCUUAUUAGUUUUGC
env-601    UAAUACUAUGGAAAAAAGUAAAAAGUGACGCAGUAUUGACAAAUUCUUAAAUCUAUAUUAAUAUGCUUAUUAGUUUUGC
env-605    UAAUACUAUGGAAAAAAGUAAAAAGUGACGCAGUAUUGACAAAUUCUUAAAUCUAUAUUAAUAUGCUUAUUAGUUUUGC
env-608    UGGCAUAGAUCGUGAAUAUGCAAAUUUAGAAUAGCACUUGUUUUAAAAUGGAGCAUAUACUAUAAUCAUAAAGAAUUAA
Dph-1-1    GACUUCUCUAGAAGAAUAGCAAAUAGAAAAUUUAAAUUGACAUUUAUUUAAAUGCAUGUUAUGCUACAUUUAAAAAUAA
env-609    UUUGAUAGCAUGGAAUAUAUUGGAGGGUUAUUUGUGGUAUUGACAUCUCGUUAAAACUAUAUUAUUAUAGCAAUUGUUU
env-610    UCAAAUGUUUAACUUCUCUAGAGAUAAUAAGAUUAAUUGACAUAUGCUAAAAAGUAUUCUAUAAUUAAAAAAAUUUCUA
env-611    UCAAAUGUUUAACUUCUCUAGAGAUAAUAAGAUUAAUUGACAUAUGCUAAAAAGUAUUCUAUAAUUAAAAAAAUUUCUA
Mha-1-1    AGUAGGACACUUUGGUAAUUAUUUUUACUGGAAUUGUAUUGACAAUUCGUUGAGAAUACAAUAUAAUUUAUUGGAUAUU
env-612    ..................................AGGUUCCUGUGAAAUAUCUUGAAAAGCAGGUUAUAUUUGCUCAAA
env-618    ................................................................GUUUUGAAUUGAAUA
env-619    ..............................................................CUUUGAAUUUCAAGGGC
env-621    CGGUGUUGAGCUGGCACGUGGGUUCGAAUCCCACCUUCUCCGCCACAGUUUAUUAAGAAAGCCUUUGAAUUUCAAGGGC
env-623    UUGCUAUCAUGGGGGAAUAUAUAGACGAAACAAUAGUAAUUAUUGACACCUUGAUAAAACAGUAUUAGUAUAGUAUUGU
env-625    UAAUAUAAUUUCUGCCAAUCUUUUUGAUUAUGCGGCUAUAUUGACAUCUUAUGAAAACUGUAUUAGAAUUAUUAAUGCU
Cin-2-1    GUAAGAAAUAGAAAUAAGGAGAAGGAUAAAUCAAGUGAAUCUUAAUUGACACUAGUUUAAAUUCAUAUUAUAAUGAAAA
env-626    GACAUUAAAUUAUAGCCAUUAAAACGUUAUCUUAGGGUAAACCGUUGACUUUAAUACAAGGUUGCAUUAAACUAUUACU
env-627    CAUAUUUAAAUUAGUGUUAUCCGAUUGAUUUUCAGGCUAUUGACAUUCAUCAAAAGCUAUCUUAUAAUUAUGUCGUUUU
env-628    CAUAUUUAAAUUAGUGUUAUCCGAUUGAUUUUCAGGCUAUUGACAUUCAUCAAAAGCUAUCUUAUAAUUAUGUCGUUUU
env-629    ..............GUGCAGUGUCAAAGAGCUCNAUAGUGGAGAAACUAGACGCACAUAAAUAAUUUGAUAUUAUUUUC
env-631    ...GAAACAAUAGUUGGAGCUGUGCCAAAGAGCUCAUUAGUAGAGAAGCUAGAAGCACAUAAAUAAUUAAUUCAACAUU
env-632    ...............................................................................
Til-1-1    AUGAGUCCAUCGUGCCAAGUAGGAAGUGCCAGGACAUUGACAACUAGUCUCCGAAAAAUUAUAAUCAAAUGAUAUCAAU
env-633    ..............................................................AAUCAAGUGAUAUCAAU
env-634    ..........................................................................UCAAU
env-635    UACUCCGGUAUCCAUUCACCCCGUGUGAAUACGGCGUUGACAAUCUUUCUCGAAAAAAAUAUAAUCAAAUGAUAUCAAU
env-636    GUAGCUAUGGAAUUUUAGGUACAAACAUUAUUAGAUAAAGAUUGACUAAAAUUAAUGAUAAAUACUACUAAAUGAAAAA
env-637    GUUAAUUUCCUUUGUAAACCACAUUAACAGGUUUUCUUGACAAUCUUUUCGAAAUAUCUUAUAAUGGCUUUGUUUACAC
env-638    GUUAAUUUCCUUUGUAAACCACAUUAACAGGUUUUCUUGACAAUCUUUUCGAAAUAUCUUAUCAUGGCUUUGUUUAAAC
env-640    GUUAAUUUCCUUUGUAAACCACAUUAACAUGUUUUCUUGACAAUCUUUUCGAAAUAUCUUAUCAUGGCUUUGUUUAAAC
env-641    GUUAAUUUCCUUUGUAAACCACAUUAACAGGUUUUCUUGACAAUCUUUUCGAAAUAUCUUAUCAUGGCUUUGUUUAAAC
env-644    GUUAAUUUCCUUUGUAAACCACAUUAACAGGUUUUCUUGACAAUCUUUUCGAAAUAUCUUAUAAUGGCUUUGUUUAAAC
env-657    GUUAAUUUCCUUUGUAAACCACAUUAACAGGUUUUCUUGACAAUCUUUUCGAAAUAUCUUAUAAUGGCUUUGUUUAAAC
env-658    GUUAAUUUCCUUUGUAAACCACAUUAACAUGUUUUCUUGACAAUCUUUUCGAAAUAUCUUAUCAUGGCUUUGUUUAAAC
env-659    ACUGUAGAUCCUGUGAAAUCACUUAGAAGAGAGUAAUAAUAUUGAAAUAUCAUUUAGUGAGUGAUAUUAUAGAGAAUAU
Tma-1-1    CAGUAAUGACACUUUCGAAAGUAGGUGGUACUUAGAUAUAUCUUGACCGAGGUGAAAAAUCCAUGUAGAAUGAUAGAAA
Nfe-1-1    AAUCUUUGAGUGAGAGUUCACUUUAAAAAAACCAUAAAUCUUGACAUAGGCCAAUAAUUCAUAUAAAAUUAUUAGAAAA
Cfo-1-1    CAACAACACAACAAUUAAUUAAAACAAUUCAUAAAGACUUGACAUAAGCUAAAGAUUAAUGUAUUUUAGUAAGAAAAUA
Npe-1-1    AUUAUUCUUAUUAAAAUACUACUAUAAAUAAUUCAUAGAUACUUGAUAUAAACUAAAGAUUGUUGUAUGAUAAUUUAAA
Gfe-1-1    AUUAUUUUUUUAAAACGACUACUAAAAAUAAUUCAUAAAGACUUGACAUAAGCUAAAUAUUAAUGUAUGAUAGUGAAAA
Psp-1-1    ...........UAGAAGACAAUUCUGAUUCUAAUUCUGACUUGACAGAUACGCCGGACUGAUGUAAUAUAAUAAAAAAA
env-660    UGAUCUCCGUGGUAUAUGAGGGAAGUUUAUAAGAAGGCUGUUGACAUUAAUAGCUCAUUGAUGUAAAAUUUCUAAAAAC
env-663    ...................................GUGAACCGCAAAAACCCUCUUGCAAAAAUCCCUCCGGUGAUAUA
env-664    UGUGACAGGGCUGGGGGCAGAAUGGUUUCUAUAGUCCUUGAAAAUAUCACAGGAUCAUGAUAGACUUUAUCAAGAUAUG
Cba-1-1    CGUGACAGGACUGGGGGCAGAAUGGUUUCUAUAGUCCUUGAAAAUAUCACAGGAUCAUGAUAGACUUUAUCAAGAUAUG
Cba-6-1    UGUGACAGGACUGGGGGCAGAAUGGUUUCUAUAGUCCUUGAAAAUAUCACAGAAUCAUGAUAGACUUUAUCAAGAUAUG
env-665    AGAGUCUGAUGCCGGAAGGAAAAAAUUUAAUUCUGUUCUUGAAAAAAAGAAUAACUUGUGGUAUUCUGAUAGCAAUUUC
env-667    GGUGUCAAUAUUAUUAAAUUACCAAGUGUGGAUUGAACUUGAAAAAUAGAAAAAACUGUGCUAUCCUAUUAAAAAAAUUA
env-668    GGUGUCAAUAUUAUUAAAUUACCAAGUGUGGAUUGAACUUGAAAAAUAGAAAAAACUGUGCUAUCCUAUUAAAAAAAUCA
env-669    GGUGUCAAUAUUAUUAAAUUACCAAGUGUGGAUUGAACUUGAAAAAUAGAAAAAACUGUGCUAUCCUAUUAAAAAAAUUA
env-670    AGGGAUGAUAAAACAAAGGAAGCGCUUGUGGAUUGAACUUGAAAAAUAGAAAAAACUGUGCUAUCCUAUAAAAAAAAUCA

           ...............................................................................
           ...............................................................................
           ...............................................................................
           ...............................................................................
```

Csy-2-1	GGUGUCAAUAUUAUUAAAUUACCAAGUGUGGAUUGAACUUGAAAAAUAGAAAAAACUGUGCUAUCCUAUUAAAAAAAUCA
env-672	GGUGUCAAUAUUAUUAAAUUACCAAGUGUGGAUUGAACUUGAAAAAUAGAAAAAACUGUGCUAUCCUAUUAAAAAAAUCA
env-674	CGGUGUCAAUAUUAUUAAAUUGCAAGUGUGGAUUGAACUUGAAAAAUAGAAAAAACUGUGCUAUCCUAUUAAAAAAAUCA
env-675	CGGUGUCAUUAUUAUUAAAUUGCAAGUGUGGAUUGAACUUGAAAAAUAGAAAAAACUGUGCUAUCAUAUUAAAAAAAUCA
env-679	GGUGUCAAUAUUAUUAAAUUACCAAGUGUGGAUUGAACUUGAAAAAUAGAAAAAACUGUGCUAUCCUAUAAAAAAAAUCA
env-680	CGGUGUCAUUAUUAUUAAAUUGCAAGUGUGGAUUGAACUUGAAAAAUAGAAAAAACUGUGCUAUCCUAUUAAAAAAAUCA
env-683	CGGUGUCAUUAUUAUUAAAUUGCAAGUGUGGAUUGAACUUGAAAAAUAGAAAAAACUGUGCUAUCAUAUUAAAAAAAUCA
env-684	GGUGUCAAUAUUAUUAAAUUACCAAGUGUGGAUUGAACUUGAAAAAUAGAAAAAACUGUGCUAUCCUAUUAAAAAAAUCA
env-689	...........................GCUAUAUUACGGGUGUGGUGUUAAGUGUAGAUGGUAUGGCGCACAGCUAACAA
env-690	ACCACAAUGCCCUGUACCCCAUUUUUUAAAAACUACUUGACCUCAAACAUAAAUUGUAGUAAAUUAUACACAACUAUUU
env-691	ACCACAAUGCCCUGUACCCCAUUUUUUAAAAACUACUUGACCUCAAACAUAAAUUGUAGUAAAUUAUACACAACUAUUU
env-692	...............AAACAUUAGUUACCAUUGAAGAAGGAAUAGAUAUAUUAACUAAUAAAUAGAAUAUAUUAAAAUUA
env-693	AACGGCCAGAGUUUAACUUUUAUAUAGAGAGGCCAUAUUUUUUUGUAUAUUAAGAUAUUUUUUGACUUUGGAAAAAUUU
env-694	CUUCAUAGGAGGAGCAUGAUUUCUCUCAUAAAGUCCUAAAAAUCCGAUUGACAAACCGUAACGAAUAAAUUAUAAUGAA
env-695	AUGCCAGCGCAUCAUCUUUCUUUUUUCAAAAUCAUAAUAAAAACCGAUUGACAAACUGCAUUAGAUAAAUUACAAUGAA
[ae-1-1	CUUUAAAAUAAUUCUUUAGUGUUUAAUUAAAUUUUCGCAAAUACCCUUUGACAAAUCCCUUUGGAUAAAUUAUAAUGAA
env-696	UGCAAAAUACGUUCCUGGAAAGACGUAAAAAACUCACGGCGAGUAUUGACUUUCUCCAAAUAAUGUGUUAUCCUCUUAC
env-697	GAGGGAAGCGGGCGCAAAAAUCCGGCUGCUCCCCUCUUGCAUUCUUCGGAAAAACGUAGUACAAUAAUCCGCGGAAAAA
env-698	GAGGGAAGCGGGCGCAAAAAUCCGGCUGCUCCCCUCUUGCAUUCUUCGGAAAAACGUAGUACAAUAAUCCGCGAAAAAA
[am-2-1	UGUCAGUCAUUAGCAUUUUUACGGAUUAGACGGUCACGUGUUCUGAACGAUGUUGAACAAAAUAGGAAAAUUGCGUAUA
env-699	UGUUAAUCUGCUAUGCUGGAUACUUUGUAUACCUUAUAAGAUAAGUCUUGACCUUAUAUUUUAAAUUUGAUAUAAUCUA
env-700	CAUAAUGUAAAUGAUGGUGACAGAAGUUAUAUAAUAUAUUUUUUAUCUGUACUUGAAUAUCUUAUUAUAUUAUGCUAUA
env-701	CAUAAUGUAAAUGAUGGUGACAGAAGUUAUAUAAUAUAUUUUUUAUCUGUACUUGAAUAUCUUAUUAUAUUAUGCUAUA
env-702	ACCUUUCUGUAUAUAAUGAUAACAAAUCGAUUAUAUCAUAUUUUUAUUGACAAGAAAAAAUGAUUUUUGUAUAAUCACG
env-703	CUGCCUUUUUAAAAUAUUUUUGCAUACACAUUAUAUCACAUAAAUCUUGACAAUAAAGAUGGGUUUUUGUAUAAUUUCC
env-704	AAAAACAACUAAUAUUAAUGAACAGCAUUCAUUAUAUCAUAUUUUUGUUGACAAUAAAUAAUGAUUUUUGUAUAAUUCA
env-705	UCUUUGGCAAACCGGCGAAUGUCCGGAACCGGAGAUUUCUGUUGUUGGCGAAUGUUUUGUGUUAGAAUAUCCGCGGUAA
env-707	GAUAAAAUAUGAAAAAAGUCAAGGGAAUGUGCGAAAAAAACAGCUUUUUUUGCAGGCUUGACACACCAUCCGGGGCAUA
env-708	UAACACGUGAUGUAAUUUACGGCAAAAAUCCAUAUUUUUACUCAAUUUCCUUGACAGGGGAAAAAUAAUGUGUUAAUAU
env-709	CACUAUAAACAGGACUUUUCUGUCUCCUCCCCAUUUUAUUUCAGCACUGUUCCGGUUUUCACUUGCACUUUUCCCCGCC
env-710	CACUAUAAACAGGACUUUUCUGUCUCCUCCCCAUUUUAUUUCAGCACUGUUCCGGUUUUCACUUGCACUUUUCCCCGCC
env-713	ACCCUGAAUGAUAUCACAGCAGAUCAGCAUUGACCGCCGCUAUGAUUUUUUUGCCGUGACUAUUGCAUUCUGAAUACAA
env-714	UUUCGCCUAAAAUCCGUACAUUUCUUCCUUCUUCUCAUUUUUCUGUGCCAUAAAUCUUUGUCUUGAAAUCUUAAUUGUC
env-716	UUUCGCCUAAAAUCCGUACAUUUCUUCCUCCUUCUCAUUUAUCUGUGCCCUAAAUCUUCGUCUUGAAAUCUUAAUUGUC
env-717	AAUUAUUCAGCAAAUUCAGAUUGGCAGCUUGAUUUCUGCCGCCAGACAAAUGCUUGAAUUUAUUAUAUACUUUUGCUAU
Bpi-1-1	.................UAGAAUAUGACUUAAAUAGUGUAGGAUUAAGUUUAAAGGACUUUAAUUAAUAUAAAAAUAAUA
env-718	....UCA
env-719	CUGGCAUAAAAGGAGAUUGAGUCUCUUAUAGAGUUUCUUGUAUUGCCAGCAACAUUUCCUUUUGCUAUAAUUUGCACUA
env-721	GAAUGAAAAAAGCCACUGGGAAGUUGCGAAAGGAUCUUGGACCCUUUAGCAAAAAAGCGGUCAUUUUGUAGGAUAUACA
Exy-1-1	UAGAGGGAAAAGCAUAUGGUAUAGAAGUUUAUACCGGAAGAUUGACAGAUUAUGAUAUUUGCUGUAACAUUUACUUAGU
env-722	....UUAUAUUAAUUUCAA
env-723	UAUACGAAAAUAAUAUUGUUAUUUUUUUUAGAAAAAUUGUAUUUUACAAAAAUAGAUAGGUUUAGUACAAUAGUGCUGC
Sru-1-1	UUACUGAGGAAAGUCACUGUGCCUGAAAACAGGCAAAUAAGAUUGACAAGGGUGUUUUUUUGCUCUAUGCUAGAUAGCA
env-724	GGGGAUUAAAGGCAUCUGGGUGCCGAAUGCAGUUUGACAUGAAUUGACAAGGUUGUUUUUUGUUUUAUGCUAUGAAGCA
env-725	CUGCGGCAAUAACGUCACUGUGCCUAAAAGCAGUAGGUGGCAUUGACAAAAUCGUUUUUUUGCCCUAUGCUAUGAAACA
env-726	AUCAUACAAUACAGUAAGGGGAAUACGAUUAUUUUAUUAAAAGCUCUUGACUCAAAUGUUUUUAUAUGCUAUAAUAUUA
env-727	UUUGUAUGGCUUUGGAUUUUUUUAAUUUAAUGAUAUAAUAUAGUUGGAAUGAAAAGAUUAAAAGUAUCAUUCCAAAUUA
env-728	CAGUCCGAUGUAUCAUAUUAUGAAUAAAAAAAUAUGCUAAACUGUAUCUUGUAAUUUUCUUUUGUAUUAAUUUUAAAAAU
Lga-5-1	UGUUACCAUGUAUCGUAUAGAAAACAAAAAAUAUGUUAGACUAUAUUUUGUAAUUUUCUUUUGUGUUAAUUUUAAAAAU
env-729	........................AAACGAUUGAAAAGGCUUUGGAAGCAAUUGUAUUAUCGUUUAACAUGUGAUAUUCU
env-730	GCCGGUUUCAUCAUGGAAACGGAAUGAUUCCAUCAAACCGUUGACACGAAAAGUACGUUAUGCUAUACUGGCUGUUACA
env-731	CUUGACAAGUAUUUCGGUUUGUGAUAAAAAUUUGUAAGAAUAUAUAACUGAGGAUCUGCUAUUGUUUUAGUGCGGGUUC
Esp-13-1	AUUUCUUGACAAGCCUUGUGGCUUAUGGUAAAAACGUAGAGGAAUAAAACGUGUGGUUCGGGCAGUUUCUUGUCCGACU
env-732	GUUUUUAGUAUGGCUGAUUUUUUUCAAGGUAUUCUGUACAGGGAAAAGGUAUUGACAAAUCAAAAAGAGAUGUUAUAAU
env-734	............................CCCCCACAUUUUAGAGCACUCCCACUUGACUUGAAUCUCAAAUCGCGGUAAA
env-735	CCACGCCUUCGAAUGGGCGGAAUAUAAAUUUAAAUACUGGGUAUUGACAUUUGGAAAAGGUGUGAUAUAAUAUAGCGAA
env-736	CCACGCCUUCGAAUGGGAGGAAUAUAAAUUUAAAUACUGGGUAUUGACAUUUGGAAAAGGUGUGAUAUAAUAUAGCGAA
env-737	CCACGCCUUCGAAUGGGCGGAAUAUAAAUUUAAAUACUGGGUAUUGACAUUUGGAAAAGGUGUGAUAUAAUAUAGCGAA
env-738	UUCCAGUAGCCGGCCGCCCCCGGCGGACGCGUGAAAAAUUGUGAUUGACAAGCCGGGAAGCGUGUGCUAAAAUGUCGGG
env-740	CCGCAACAUAUAUUUUAAUUGGGAUCGCUGGUUUUUAUUCAGCAAAAAAGAUUUGACAAUAAAAUAUGUUUGUGGUAUA
env-741	GAUGAAGAAGUUGUUUAUGUGAUUAAGAUGAAAAAAAUAAUGAGAUGUAUUGAUAUAUAUUAUGUCUUGUGAUAGAAUGC
Msp-2-1	GAUGAAGAAGUUGUUUAUGUGAUUAAGAUGAAAAAAAUAAUGAGAUGUAUUGAUAUAUAUUAUGUCUUGUGAUAGAAUGC
env-742	GAUGAAGAAGUUGUUUAUGUGAUUAAGAUGAAAAAAAUAAUGAGAUGUAUUGAUAUAUAUUAUGUCUUGUGAUAGAAUGC
Mti-1-1	UUAAAUUAUUUUGGAAUAUUAGCUGUUUAAACAAACUCUAAUCUAUUGAUAUACAAAAUAAACUGUGCUAAAAUAUCUG
env-743	UUAAAUUAUUUUGGAAUAUUGGCUGUUUAAACAAACUCUAAUCUAUUGAUAUACAAAAUAAACUGUGUUAAAAUAUCUG
env-744	ACCAUCCGGGAUUUUUGCAGAAAUUUUUGGAAAACUCAGGAAACAGGAGAAUUGACGCAUAAAUAUUUCUGUAUUAUAA
env-745	ACAUGGACGCAUUUUACCUGUUUGGUGGAUUUUUUUUACAAUUUCUUGACAAACAGGCAUUCCUGUGUUAGAGUAAACA
env-746	UAUAUUAUGCGCGCCGUUUUAAAAUGGGUAGUAAUUUGCAAAAAUAUAUUUGACAAUUUACGGCCGGUAUAUUAAAAUC
env-747	UAUAUUAUGCGCGCCGUUUUAAAAUGGGUAGUAAUUUGCAAAAAUACAUUUGACAAUUUACGGCCGGUAUAUUAAAAUC
env-748	GGAUGCGUAUGCUUUGCGCGUUCCGGACGCAUCCCUGCAAAAAUUUAAUUGACAUAUCAAAAUAGUUAUAUUAAAAUUA
Bma-1-1	CAUAAAGUAUACCAUAAUGGGAGAGUCAAGUAUCUUUAACAAAAGAUUUGACAAAAAUCCUGCCCUUAUGAUAUACUGU
env-751	....AAGUAUACCAUAAUGGGAGAGUCAAGUAUCUUUAACAAAAGAUUUGACAAAAAUCCUGCCCUUAUGAUAUACUGU
env-753	UCCUUUGCCGUAAACUUUGCUUACCUUUAUUAUAGCAUUUUAUCCGCGUAAGCGUAUUGCCAAAGCGGCGGCGGUGUGC
env-754	....CAUAUUGCCAAAGUGCGGGCAGUGUGC
env-755	GCCCUUGCCGCAAACUUUGCUACCUUUAUUAUAGCAUUUUUAUCUGCUGCGGCAUAUUGCCAAAGUGCGGGCAGUGUGC
Lga-1-1	CAAUUAUAAAUAUCCUUCCUUAAAAAUAUAAUUUCUAUAUUGAGAUAACUGUCAAACUUCUGUAAAUUUGUUAAAAUUG
Lsp-4-1	................UUAAACCUUCAGCUGAUGUUUGGUAUGAAGCUGGUUUUAGCAAGAAUUAAUAAACCUACAUUAU
env-757	AAUUCAUUAAGACUCUUUAAAAACGUUUAAUUUUAAGCGAUUUUAUUAAAAUAAAGAAUUGCUUAUGAUAUAAUAUAUU
env-758	AAUUCAUUAAGACUCUUUAAAAACGUUUAAUUUUAAGCGAUUUUAUUAAAAUAAAGAAUUGCUUAUGAUAUAAUAUAUU
env-759	....UCCGCAUCAUUCAAACC
env-760	UACGAUUGAGACGGGUUUGAUCGCUGCGCCCGAUUGGAGAAUCAGAGAUCCUUCCAUCCCUAUCCGCAUCAUUCAAACC
env-761	CGCCCUUUUGCGGAGAAAAUAAUGUUCGUGGACGAACAAGGCAAAAUCUGUUGACAAUAUAUAAUAAUUAUGGUAUCUU

```
env-762    .....................................CGCCGUCAAACAAUGCUCUUUCGGAGCUUGAAAACGCGAUGGG
env-763    AAAUAGAUUGAAAAAUCAGAAAAUAAAUAGAGUAUAAUAAAAUAAAUAAGCAAGAUUGACAACUGGAUUUCUAUGUGAU
env-764    AUGGAUUGACGUAUGUAAAGGCUGGAAUGAUGAUAAAUAACACUUGAGUCAUUCCGGUGUUAUGCUAUACUAUAAAACA
env-765    CGUACAAAUAAGCGCACGUUGCGGCGGCAUAGGGAAAAUACAAAAGCGGCGCUGGAUACGGCACCGCUUUUGUGAUAGG
env-768    CUAUGAAGCGGGGCGCAUGCUGGCAUGAUAAAGGAGGCCACGGAAAGCUGUUUUGACACCGACCAUAUUCCAUAGUAUC
env-769    .......................GUCAGCGUAAAGGUGACGCUGCUAUGGAAGUUCUGAUUGAGCUUGUAUAAUCAAUUG
Lsp-9-1    ........................GACAGCGUAAAGGUGACGCUGCUAUGGAAGUUCUGAUUGAGCUUGUAUAAUUGAAU
env-770    UAAAGGUGACGCUGCUAUGGAAGUUCUGAUCGAGCUCGUAUAAUUUAAUAUCAGAAUAUUAGUUCAGCUUAGCGAUUGG
Lsp-10-1   AGCGUAAAGGCGAUGCUGCUAUGGAGGUCCUGAUCGAGCUUGUGUAAUCAUCUGAUGAUGAUCAGUAUGAUAAAAAUAU
Csp-15-1   GCGUAAGGGCGAUGCGGCCAUGGAAGUUCUGAUCGAACUCGUUUAAAGAUCAGAAAGAGAACAGACUGAGUAAAGAAAA
env-771    CCUGGUUCUCAAUGGAGGUGAGGGUGUCGGAUUGACGAAAUGAUUCGUAUGGAUUUGAGAGAGAAUCUGUGAUAGGAUA
env-773    CCAGGACCGCCGUUCCCCACCCGGGGCGGCGGUCCUUUUCUUUUCUCUUGACAGCCGGGGUGGUUUGUGCUAUGGUAUA
env-774    GUUCUAUCAUCGGCGGCACUCCGCCCAACGGGGAUUUCAGAGCCGGUGAACCGGGCCCUUGACUUUCCUGAAAAAGGAG
env-775    AUUUAGCCAAAAUUCUUGAUGAGCAUUGCAAAUAUGCAUAAGACCACUAUUAAGGCUAUUGCCGCAGCGAUAAUAAUGU
env-776    AUUUAGCCAAAAUUCUUGAUGAGCAUUGCAAAUAUACAUAAGACCACUAUUAAGGCUAUUGCCGCAGCGAUAAUAAUGU
env-777    GGCGCAAAGGCUAGUAAAAUUUCUAGCCAUAAUGUCUUUAAAUUUCAGCCCCGCUUAUUGACCGGCAAUAAACACUGUG
env-778    UUAGCGCAGGCUUUUCCAGCAGCUAAACGCAAGUAAUAAUCUUGCUUUUUAAAAAUUGACAGCUGCAAAUUAUUCAUGU
env-780    AUUAUCUGCAUGCUCUGACCGGCGGGGUACUGCUGGACGGAUGAUCCGAAAACACUCUUGAUUUUCUUCCAAAAUGUGU
env-781    UCGAAAGCAGAAGAACCGCAAGGUUCCGGGGGUUCAAAUCCCUCCUGCUGCGCUUCAAUCCUAUUCCUCCGCAGAUCAC
env-782    UAUAAUAAGAAAAAUUUUUUUGAUAUUGUAACAUUUAUGCUAAUUGACAAAUAUAAAAUUAUAUGUUACUUUUUUCAGA
env-784    UAUAAUAAGAAAAAUUUUUUUGAUAUUGUAACAUUUAUGCUAAUUGACAAAUAUAAAAUUAUAUGUUACUUUUUUCAGA
env-785    CUUUAAUGUAUUCAAGGACGAGGACAGAAGGAUUUAUGAAUCCCUGCUCGGAUAGGUCUUGCUAUUCCGUUCUUUUCGU
env-786    ........................................................................GGAUUUCU
env-787    GCACAGCGGUCUCCAAAACCGUUAGUACGGGUUCGAAUCCUGUUAUCCCUGCCAUUUUUAAAUCAUAUGAUGUACAUCA
env-788    ...............................................UUACGCAGUCUCAGGCUGCUGAGAUCGUAAACU
env-789    AUAGGUGCAUACAGUCACGUUUAUGAAAUAUUGCUCGUAGAGAGCCUUACGCAGUCUCAGGCUGCUGAGAUCGUAAACU
env-790    CGGAAUCAAAUGUCAAUAGGAAAACAUGGAUUUUUUGCGGAACAGAACCGCUUGACUUUUUAGAGUAGAUAUGCUAUAC
env-791    CGGAAUCAAAUGUCAAUAGGAAAACAUGGAUUUUUUGCGGAACAGAACCGCUUGACUUUUUAGAGUAGAUAUGCUAUAC
env-792    CGGAAUCAAAUGUCAAUAGGAAAACAUGGAUUUUUUGCGGAACAGAACCGCUUGACUUUUUAGAGUAGAUAUGCUAUAC
env-793    CGGAAUCAAAUGUCAAUAGGAAAACAUGGAUUUUUUGCGGAACAGAACCGCUUGACUUUUUAGAGUAGAUAUGCUAUAC
env-794    CGGAAUCAAAUGUCAAUAGGAAAACAUGGAUUUUUUGCGGAACAGAACCGCUUGACUUUUUAGAGUAGAUAUGCUAUAC
env-795    CGGAAUCAAAUGUCAAUAGGAAAACAUGGAUUUUUUGCGGAACAGAACCGCUUGACUUUUUAGAGUAGAUAUGCUAUAC
env-798    .....UGUUCAAAGCCAUCUGCGACCGAUUGUAUCUGUCGGAAAAAUACGGUUGACUCUUGACAGGAUUUCUUUUUUAU
env-799    .................AAUUUGCUUCGCAGGGAUUUCCUAUUUACAGACUUGAAGCAAGAAUAUAAAUGUGUUAUAAUU
env-800    AAUAGUUUAAACCCUUCGGAAAGGGCCAGCCGUCAGUUGUUGGGAGCAGUUGGCGGCUAUUAAUGAAAAAAGCUCUGUA
env-801    GAAAAGCGAAAAGAAUAGUCGAAAGAAGCGUGUUUGCCUCCCUUGACUUUUCAUCCGGCUUCUGCUAUAAUAAGCGCAG
env-802    GGGCUCAUUCACUGAUACCUAAAACUACAUAUUCAUUUUUCUCACCCGCAGGGCAGAGAAAACAAGAAAUGAGUUAGUA
env-803    GACCAGCAGCUCAACGGCGAACCUCCGAAGGAAUGGGGAGCCUGAACGCUUGACAAAUCGGCAGCCGUGUGAUAUAAUC
env-804    GACCAGCAGUUCAACGGCGAACCUCCGAAGGAAUGGGGAGCCUGAACGCUUGACAAAUCGGCAGCCGUGUGAUAUAAUC
env-805    GACCAGCAGUUCAACGGCGAACCUCCGAAGGAAUGGGGAGCCUGAACGCUUGACAAAUCGGCAGCCGUGUGAUAUAAUC
env-806    GACCAGCAGCUCAACGGCGAACCUCCGAAGGAAUGGGGAGCCUGAACGCUUGACAAAUCGGCAACCGUGUGAUAUAAUC
env-807    GACCAGCAGUUCAACGGCGAACCUCCGAAGGAAUGGGAAGCAUGAACGCUUGACAAAUCGGCAGCCGUGUGAUAUAAUC
env-811    GACCAGCAGCUCAACGGCGAACCUCCGAAGGAAUGGGGAGCCUGAACGCUUGACAAAUCGGCAGCCGUGUGAUAUAAUC
env-824    GACCAGCAGCUCAACGGCGAACCUCCGAAGGAAUGGGGAGCCUGAACGCUUGACAAAUCGGCAGCCGUGUGAUAUAAUC
env-827    GGCCGUUCCCAAACCAAUCAUAUAUUUCCCUUCUUUAAUUGUGAAAGUAACACUAUCAAUUUACUUCAGUAUAGCAAUC
env-828    ...............................................................AGCCGUGUGAUAUAAUC
env-829    GACCAGCAGUUCAACGGCGAACCUCCGAAGGAAUGGGGAGCCUGAACGCUUGACAAAUCGGCAACCGUGUGAUAUAAUC
env-830    GACCAGCAGUUCAACGGCGAACCUCCGAAGGAAUGGGGAGCCUGAACGCUUGACAAAUCGGCAGCCGUGUGAUAUAAUC
env-848    GACCAGCAGUUCAACGGCGAACCUCCGAAGGAAUGGGGAGCCUGAACGCUUGACAAAUCGGCAGCCGUGUGAUAUAAUC
env-849    .....................ACCUCCGAAGGAAUGGGAAGCAUGAACGCUUGACAAAUCGGCAGCCGUGUGAUAUAAUC
env-850    GACCAGCAGCUCAACGGCGAGCCUCCGAAGGAAUGGGAAGCCUGAACGCUUGACAAAUCGGCAGCCGUGUGAUAUAAUC
env-851    GACCAGCAGUUCAACGGCGAACCACCUAAGGAAUGGGGAGCCUGAACGCUUGACAAAUCGGCAGCCGUGUGAUAUAAUC
env-855    ........GUUCAACGGCGAACCUCCGAAAGAAUGGGGAGCCUGAACGCUUGACAAAUCGGCAGCCGUGUGAUAUAAUC
env-856    GACCAGCAGUUCAACGGCGAACCUCCGAAAGAAUGGGGAGCCUGAACGCUUGACAAAUCGGCAGCCGUGUGAUAUAAUC
env-858    GACCAGCAGCUCAACGGCGAACCUCCGAAGGAAUGGGGAGCCUGAACGCUUGACAAAUCGGCAACCGUGUGAUAUAAUC
env-860    GACCAGCAGUUCAACGGCGAACCUCCGAAGGAAUGGGGAGCCUGAACGCUUGACAAAUCGGCAGCCGUGUGAUAUAAUC
env-861    ...............................................................................C
env-862    .......CCGAAAACUCCGAUGAAGCCGACCAGCCGCCGCAGGAAUGGGACGCUUGACAAAUCACUGAAAGUAUGCUAU
env-863    ................................................................................
env-864    UCCAAUAAGAUAUUUUUUAUACUUUUACAUCAUCUCGGGCGGGUGAUACACUGGCUGUAAUAAAAAAUCAUAAGUAUUU
env-865    UCCAAUAAGAUAUUUUUUAUACUUUUACAUCAUCUCGGGCGGGUGAUACACUGGCUGUAAUAAAAAAUCAUAAGUAUUU
env-866    GGUAAAAAAUUCAAAGAAUUAGUAAAAUAGUUUUAUGAAUUAUAAGAAAAACGGAUUGAUUUUUCAAUCCGUUUUUUUU
env-868    ................................................................................
env-869    UAGUCAUAAACUUUUUAUUUUAAAAAGGAUAUAGAAAGAUUGAUUAGAAUACCCAUUAUGUGAUAUAAUGGCAGAAGAA
env-871    UAGUCAUAAACUUUUUAUUUUAAAAAGGAUAUAGAAAAUUUGAUUAGAAUACCCAUUAUGUGAUAUAAUGGCAGAAGAA
env-872    GUUAUUUUUCUAUUUGUAGAAGAAAAGGAUAUAGAAAAGUUGAUUAGAAUAUCCAUUAUGUGAUAUAAUGGCAGAAGAA
Fsp-3-1    GUUAUUUUUCUAUUUGUAGAAGAAAAGGAUAUAGAAAAGUUGAUUAGAAUAUCCAUUAUGUGAUAUAAUGGCAGAAGAA
env-878    UAUUGAAUGGGCAUAAAACAAUACGUUUCCUGCGCUGGGAAUGGGCUUUUAUGGGCUUGACUUUUUAGGAUAGUCCUAU
env-880    CGAUUUGGAGCGAAGCCGGGAUAGUGCAGAGGCGUGAGGCAGGGACACUGUAUUGACAGCCAUGGUUUUUUUCCUUAUA
env-881    AGUUUUAAAGCAAAAUAUGCUUAAUAAGAUAAAAGAUUCGGAAAUAUCCAAUCGAUUUGACUUAAUACUGCUAAUUUGA
env-882    AAUAAAAAACCGCCUACCCUACGGUAAGCGGUUUCCCUUUCCCUUGACAACUUCUUUUCCGGCAUGCUAGACUUAAAUA
env-883    ..............................................................GCAUGCUAGACUUAAAUA
env-884    AAUAAAAAACCGCCUACCCUACGGUAAGCGGUUUCCCUUUCCCUUGACAACUUCUUUUCCGGCAUGCUAGACUUAAAUA
env-885    UCACCGCUGGCUCUAUCACGCCUUGACAUAUCUAAUUUUUAAUGCUAAACUUUUCUCAAUUUUAUAAACUGUCGGUCAU
env-886    UUUUUUGUUCUUUAUGAUUAUACGACCGAUCUGCGAUAAAGACAAGGGAAAAGAGAAAAUUUGCUUUUCAGGUGAGGGU
env-888    ................................................CCUUUGCGGAUAAAGAUUUAUUGCAGCCGCCC
env-889    ................................................CCUUUGCGGAUAAAGAUUUAUUGCAGCCGCCC
env-891    GCAGGUAGUAAACGUAUGAACUGAAUAAAGAAAAUAUUUUAUCAGCGCAGGACUUGUAUUCGAAUUUUGUUUAUGCUAG
env-892    ................................................................................
env-893    AUUUUGAGCGGAUUGGUUAAUGUGCACAAAUUUAUUUGUAAUAUUUGUUUAUAUUGACAGCCGAAAAAAAACAUGGUAC
env-895    .........................CACAAAUUUAUUUGUAAUAUUUGUUUAUAUUGACAGCCGAAAAAAAACAUGGUAC
env-896    ........................................................GACAGCCGAAAAAAAACAUGGUAC

           ................................................................................
           ................................................................................
           ................................................................................
           ................................................................................
```

```
env-897  ...........GAUUUCCCCUUUUCCUCCCUGAAAGCAUGGAGUAAAAAGGGCUUGACAGCCCGCUAUAAAUAUGCUAU
env-898  ...................................................................UAAGUAUGAUAU
env-899  GCAUCGAGCUGAGAGCAUAAAAAGGGUUGACACCCAAAGACCGGUGUGCUAUAAAUAGGGCAUAUCGGAAUCCGAUGUG
env-900  AAAUCGCCAAAAGUGCCUUUCUGAAAAAAUCUCACAAAAACCUUGACAACCCGCACCGGGUGUGCUAUAAACAUCUGAG
env-901  ............................................ACAAGAUUCUCAAUGGCUUAAAGAGUAUGGGGAUU
env-902  ............................................ACAAGAUUCUCAAUGGCUUAAAGAGUAUGGGGAUU
env-905  GCCCCAUUUUCAGACGCCUUUUUACAGAAAAACACAAAUUAUUCCCAUGAAUAUGAGUAUUGACUUAACACUCUAAUUA
env-906  UACCAACAGGAAGAAUAAAAAUUCAAGAUGCAGCCUCGUCAAAUUUUUCUUGACGAGGCGAUUUUUUUGUAUUAAACUC
env-907  CGUAUUUUCCUCAAAAUACAAAAUCGGACUUUUUCCUUAAAGGAUAUGCCUUGAUUUUCAAAUCGUUCUAUGCUAGGAU
env-908  CCCGGAAACUCAAGCAAAACCUUCCCGGAAACUCCGAGCAAAAACCUUCUUGACUUUCCUGUCUAUAUGUGCUAGGAUU
Cbo-2-1  UUUAUUUAUAAAUAUGUAAAAAUUAAAUUAAUCUUAAUCUCUUGAAAAAUUUCCCAAACUGUACUAUACUAAAAAUAAU
env-910  AGUACUGAUGUAUUAACAAUAUAUGGGAUCCACAUAAUAUUGAUUUUCUGCUUAUUUAUGCUACAAUUAAGGGAAGUUA
env-911  UAGGUCUUUCAUAUCACAGCUUCAAUAUUUCUUUCUUAAAAUUGACUUUUACAAUGAUUUCUGCUACAAUGUAAUAAAA
env-912  GCUGCCUUUAACGGCUAGAUUAAGCAUGGACUGGCAGUUGCUUUUAUUACUGGGUUUGUGUUACAAUGAGCAAGGAUUA
env-913  UAGUCUGCGGCGGCCGGAUGGAUCGCGGUCUUCAUAUUGCCAUCGGACAGACGAUAUGAUAAUAUGAAAACGAAGAAAG
env-914  ............UAAAGGCAUGUUAUGCGAUGCGCCGUUUUAGCGUUCAUUGUUACAGUUGAAAAUCCGGUAUUUUUAU
env-915  ......UCCUGCGCCGGGCCCCGGGGAAAGGCACACGGGACACGGCAGAAAUGUAGUUGACUUUUGUCCUUCCGUAUGC
env-916  .....................................................................GCUAUCGUUG
env-917  AGAAUGGUUGAAACCCUUUUGCGAUUUGGGAAAGGGUUUCAAUCAUUCUAGCUGUCGAUAAGAAUUGACUUUCGUUUGU
env-918  AUACGCACAGGAUAUGUUUACGAAUUUGUAGUAUCUCCACCUUGACUUUUCAAAACGGAUAUGCUAUAUAUGUUACGAA
env-922  ........................................CUUGACUUUUCAAAACGGAUAUGCUAUAUAUGUUACGAA
env-923  UCAGUGAUGGAUGUCUUUCCAAACUAAUAUGUAUCUCCACCUUGACUUUUCAAAACGGAUAUGCUAUAUAUGUUACGAA
env-927  ACAGGUCAUUUCUAAAAUGAGAAAUAUAAGUGAUAUUUAGCAAUCUUUAUUUAUUGUAGUUGAAAACUUAAUAUUAUUA
env-928  UUACUAUCGGGAGUGGUGUUGUUUUACUUCCAGUUGGAUAGGCCUAAACUACCUUUGCUUUUUUAUCGCAGGUAUGCUA
env-929  UUACUAUCGGGAGUGGUGUUGUUUUACUUCCAGUUGGAUAGGCCUAAACUACCUUUGCUUUUUUAUCGCAGGUAUGCUA
env-930  CCAACUUUUUUAGAGUUAGCAUAAAGCCCCUAAGCAACUCUCAAAGCACACCAUUCUUGACUUUUAUAUCUUUUCAUGG
env-933  UGAUUACGAUAUUUGAUUUCUUAUCUAUUUUUUCAAGAUACCGGGAUAAUAGCCUUUAUUUCCGGGACUAAGUAUGGUA
Pvi-1-1  AAUAAUCCCAGAGAUAGUUCCAAAGAGUCGAAAUAUGCUCUAGAUGGUCUUACCUUUACUUAUUAAUUCAACUAGGUUA
env-934  UUGGGAGUAAAGAUUGGAGUUAAGAUUAGGGAUAUAAAGCUCAUUUGCUCUUGAUUUUUCGAUUAUGUUAUGAUAUUUU
env-935  GCGCAAAUCCCAGCAUUUGAGCCACAGGGAAGCGAAUCGGUCCAACAUGACACAUAUUGACUGUUGCAUGAUUCCGUAC
env-936  AACAUGUUCCCACAAUUGGGUUGAUAGCAUAAUAUCAAUUAUGAAAAAUCUUUUAUGGUUGCAAAAUGGAGUUCUUUAU
env-937  UCGAUACCUUUACACCGAAAGUCCUUAUCUAACACCCUCUACACUCUGUAUAUUUUAUUUGACAUAUCACUGCUUUUAU
env-938  CCUGACCCCCAUAAAAUGCAAAGCGAUCGAUCCCGUCUGCGCUGUCAUCGAAUUACAGUUGAAAAAAGCAUGCGAUUAU
env-939  UCGAAUCCCGUUUGACGGAUGGCCUAUCCGCUAAGAGGCAUGAUGAGGGUGUUGCAUUUUGAUGUGCAAGUAUGCUAUA
bMS-1-1  UCGAAUCCCGUUUGACGGAUGGCCUAUCCGCUAAAAGGCAUGAUGAGAGUGUUGCAUUUUGAUGUGCAAGUAUGCUAUA
env-940  UCGAAUCCCGUUUGACGGAUGGCCUAUCCGCUAAGAGGCAUGAUGAGGGUGUUGCAUUUUGAUGUGCAAGUAUGCUAUA
env-941  UUAUAUGAUUGUAGCGCCAAAAUGGUAAAAAGACUUUUUCACCUGUUGUAUUUUGCAUUCGCACGUGUUAUAAUAAACA
Epl-2-1  CCUGUUAUAAAACUAUCGUGAUUUUGACAAAAAACUAUAUGAUCUGCUUGCACAUUUCAUCCGUAUGUGUUACACUACA
env-942  GUGGAUAUUUACGAAAACAGUUUUGUGCCCAUAGAACCAGAAUAAUUUACUUUGAAUUUUAAAUCGAAUAUGGUAUACU
env-944  ...........................................................................GUGA
env-945  CAGAAAAUUUGUUGUGGAUUUUUCUGGUCAUCUAUUUUCGCCACACGGCUACUUAUGAAGUAGUUGUGUGGCGUUUAGU
env-946  AUUUAGUAUGGCUUGAGUGAUAUCGUUGUUCGGUGAUAUCUAUUAAACGCCUGAUAGGUAUUACCAAUAUAAUAAGAAU
env-947  GUGCGAUCUAUGACGCAUUUACGAAGUAGGGCCCAAUUAAUUGAUGAUUGACUAGACGCGGCCAAUCUGUUAAAUUUCA
env-948  AGCCAAGUAAGAGCUAUCAUGUCCGAUUUCGGCGCAUAAGAUAUGUGCAGCGUAUUGACAAAGGCGGCAGCACAUGAUA
env-949  ................UCAUGUCCGAUUUCGGCGCAUAAGAUAUGCGCAGCGUAUUGACAAAGGCGGCAGCACAUGAUA
env-950  AAGCUUGUUAACGCCGUCAUGUCCGAUUUCGGCGCAUAAGAUAUGCACAGCGUAUUGACAAAGGCGGCAGCACAUGAUA
env-951  GGAAUUGCGGGAAACACUUCGUGUUACGAUUCCUAUAAGUUAUCAAUGAAGCGUUUCAACUCCUCACUCCUAAUUAAAA
env-952  GUAUUGCUGGCUUGAAAAUUUUUUAAGUGUAGAAUCAAAAUUCAGAUUUGUUGAUUUACAAGCCUAUAAUGGCAUGCUA
env-953  CUGGCAUGUAAAUGAUUGAUUUAUAAAUUCCGGGUUGCAGGUAUGUUUGAGAACUUUAAGCCGUCAAUUUACAAGGCUA
env-954  GACGAAUUGGAGCCGCCGCGGAUAACGGCGUCAAUAAAGUUUAAAUUGACUUUUUUUAAUUUCUAUGCUAUAUUAGAC
env-955  .........................CUUAUGAAACCUAUGAUGAAUUAAUGGAUGCAAUAAAAAGAGCCUUGUAAAUUG
env-957  .........................CUUAUGAAACCUAUGAUGAAUUAAUGGAUGCAAUAAAAAGAGCCUUGUAAAUUG
env-958  UUUUGAUAUUAAAAAACUGGAUAAAAGAAAAACUGUAAAGACACUUGACAAUUUGUUUUUGUUUUGAUAUUUUAGCUUU
env-960  .......................................................................AAGAAAUU
env-961  UUUCCUUUUAGCAAUCUUGUUUUAUUUUGCAAAAAAACAUUUGACUUCUGUUUCUUCCCGUGAUACUAUCAAAGAAAUU
env-963  UUUAGAAAUGCUUAUACUUUUAAAAGGUUAAAAUCAUUGCCUAUAAAUAAAAACUGAAACUUGAUAAUUAGUACUCUGU
env-965  UUUAGAAAUGCUUAUACUUUUAAAAGGUUAAAAUCAUUGCCUAUAAAUAAAAACUGAAACUUGAUAAUUAGUACUCUGU
env-966  UUCUGGGAAAGUCGAAGUUGAAAAAUUUAAAAUGCCAUGUUAUACUGAGAAAGAUAAAAAUGUGAGAUUGCGAAUGAAA
env-968  UUCCGGGAAAGUCCAAGUUGAAAAAUUUAAAAUGCCAUGUUAUACUGAAAAAGAUAAAAGUGUGAGAUUGCGAAUGAAA
env-969  AUUUUACACGUGCGAAAAAGGACAUGAAUUUUCCUGCUCGCUCUUGACAGCCGCGGCAGGUUGUGGUAUACCAAUAAUG
env-970  AUUAUUUUAUAAUUGUUGAUUUUGCCUUGUUGAAAAUCAUGCUUGACAUAUUGACUAAUUUAUUUUUGUCUGAUAUAAU
env-971  GAAGCUGCUUUUAUAUAGAACUCAAAAAAUCCUAAAGUGUUGCUUUUACCAGCAAAGUAUUGUAUACUAAUGACAUCAU
env-972  GGAAUAGGGAUUUGCCGCACCGGCACUUCCACCUCUUGACAGCCAUGCUGCUUCAUGCUAUACUAAGCAUCGCUGCAUA
env-973  UUUUAGUCUCAGUUCUUACAUAGGUUCAGCACUUAUAUAUAAAUUCGUUGACAGAUAAGGAAGAUUUUGAUAUCAUAAA
env-974  UUUUAGUCUCAGUUCUUACAUAGGUUCAGCACUUAUAUAUAAAUUCGUUGACAGAUAAGGAAGAUUUUGAUAUCAUAAA
env-976  ........................GAAGACAGCAGAAAAAUUGCCAUUGCCAAAUUUCAUCCGACAUGCUAUAAUAGAC
env-977  .......................................AUUCCUCCAAGUAUGGUAGAAUGAUUCUCAAGAACAACAA
env-978  ....................................GGCGCUUUUGUCAUUAUUCCGGAAAGAUCGUAACGAUAUUUCC
env-979  CACACAUGUCGCUCACUUAUGAGCGACGCUCCGCAAGGCGCUUUUGUCAUUAUUCCGGAAAGAUCGUAACGAUAUUUCC
env-980  CACACAUGUCGCUCACUUAUGAGCGACGCUCCGCAAGGCGCUUUUGUCAUUAUUUGGAAAUAUCGUAACGAUAUUUCC
env-981  AACCUGAACGCAAAAUUUCAGGACGUUCAGGUUAAACAGCUUGUAUCUGCCCACCGGGUAUGCUAUAAUUUCCGGGAAA
env-982  GACCUGAAGCAGGAAUUUCAAAGAACUCAGAUCAAACAGCUUGUAUCUGCCCACCGGGUAUGCUAUAAUUUCCGGGAAA
env-983  AACCUGAACGCAAAAUUUCAGGACGUUCAGGUUAAUCAGCUUGUAUCUGCCCACCGGGUAUGCUAUAAUUUCCGGGAAA
env-984  AACCUGAACGCAAAAUUUCAGGACGUUCAGGUUAAACAGCUUGUAUCUGCCCACCGGGUAUGCUAUAAUUUCCGGGAAA
env-985  AACCUGAACGCAAAAUUUCAGGACGUUCAGGUUAAUCAGCUUGUAUCUGCCCACCGGGUAUGCUAUAAUUUCCGAGAAA
env-986  AACCUGAACGCAAAAUUUCAGGACGUUCAGGUUAAACAGCUUGUAUCUGCCCACCGGGUAUGCUAUAAUUUCCGGGAAA
Cba-2-1  UUUGCUAUUGUGAUAGCUAUAAUAAAGGUUCCUUUUAAUAAACUAGUUGACUUUUUUAUUAUCCUGUGAUACACUCACA
env-987  ...........................................................CUCUGAUACCAUAUGGACGA
env-988  ACCGGGAUUAGCCGGAAAAAUCAAGUUUUUACCAUGGAAAUCUUGACAUCCCUCAGGUUCUCUGAUACCAUAUGGACGA
env-989  CAAUGAUUUCUUUUGCGUUCUAUUUUCGGGGUUGAUUUUUUACGGAGGAUUGUGUACGAUUGUUUUCGGAAAAUAAAAA
env-990  ................................AUUUUGAAAAAUGCUAUCAAGAAUAUAUAAAAACUGAACCAAUAAAU
env-991  AUUCAAUCCCUCAAUAAUCAUUUAUAUAGGUCUCUAAAAAUUUGACAUUUUGAUAAGAAUUAUUUAGAAUAUAUUUAUC

         ...............................................................................
         ...............................................................................
         ...............................................................................
         ...............................................................................
```

```
env-994    AUUUUCUGCCGCCAUGCUUAUUUUGAUAUAUAAGUCUUGCCAUUGACAUCCAAUCUGAUUUGUGCUAUAUUAUACUGGA
env-995    UGCUUUUUGGGCCCUUUUGAUAUACAAUGUAAAAGUUGAAAAAUGAAACAUAACUUUUUUGAUAUACAGUGUAGAGUUU
env-997    UCCCUACAAUAAUAAUUACAACUUCUUCACACAAACACAAAUACUUGACUUGCCAACAGAUAAAUGAUAAAAUAACAAA
env-998    CUGACAAUAAAGGGGUGAAAACGGGAAAGUUCUUCUGUUGACACUUUUAAAAGUCUUUGUUAUAAUAUGGGCGGAAAUU
env-999    UAGCAAACAGGAGGCGCUGUGUCAAGGCAGAAAUUUGGGGAUUGCAUUUUUACUCUUCCUGUGUUAUUCUAAAAAAGAA
env-1001   UUGUUAUAUAUAAAAACAGCAGGUUAAGAUAUAUGCAAUAUGGGGUUGACAUUAUAUAAGAGAUAUGGUUAAAUGUUAA
env-1012   UGAAAAGUAGGAAAGACUUUUGAGUUUAAAAAGAGUUUGCUUGCUUUGACAAGAAUGAUAGUUUAUAAUAUUAUAAACA
env-1015   AGAAAGCAACAGUGUGAGCAUACAUCGGCACUUUGACAGCAACGGGGUUCCAUGUUAGAUUGUCGGUCGUACAACUGAA
env-1016   CAACAUCAUUUGCAUAAGUAUUUAUAUGAACCGUUUGAUUUGCAGAUUGACAUUAUAAAGAUAAUAUGCUAAAAUAACU
env-1017   CCAUGUGUCGUAAAGUGACACAUGCCUCGGAUAAACGCCUAUCCAGCUGUUCUGCUGCGGCUACUGGAUUCCUGUAAUG
Lba-2-1    UAACCUGUAAAAAAUAUCUAUUUCUCUUUCCUACUGCCAUUGCAUUUUCCGCAGAUUUCAUGUAUAAUCCAACUAAUCC
env-1022   AUUCAAUUUGGAAAAACGGGGAUUAUGAUUUAGAACUUGACAGAGAUAUAAUUUCAUGAUAAUUUUUAUACAAUAACAA
env-1023   CAUCACAGCAAUGGGAUGAUUUUAAAUAACUUUUUGUAGACAAAUACAUUUUUUUAUGUUAUAAAAAGGAAGGAAAUUU
env-1024   AUAAUAUUUAUUUCAUUAAAACAUCAGUAAAAUAAUGUAGAGUAAAAAUUCUGGACAAUUUUCUUGUUUUAUGUUAUAU
env-1025   AUAAUAUUUAUUUCAUUAAAACAUCAGUAAAAUAAUGUAGAGUAAAAAUUCUGGACAAUUUUCUUGUUUUAUGUUAUAU
env-1026   AUAAUAUUUAUUUCAUUAAAACAUCAGUAAAAUAAUGUAGAGUAAAAAUUCUGGACAAUUUUCUUGUUUUAUGUUAUAU
env-1040   AUAAUAUUUAUUUCAUUAAAACAUCAGUAAAAUAAUGUAGAGUAAAAAUUCUGGACAAUUUUCUUGUUUUAUGUUAUAU
env-1045   AUAAUAUUUAUUUCAUUAAAACAUCAGUAAAAUAAUGUAGAGUAAAAAUUCUGGACAAUUUUCUUGUUUUAUGUUAUAU
env-1046   ..........................................................................UAUAU
env-1047   AUAAUAUUUAUUUCAUUAAAACAUCAGUAAAAUAAUGUAGAGUAAAAAUUCUGGACAAUUUUCUUGUUUUAUGUUAUAU
env-1048   AUAAUAUUUAUUUCAUUAAAACAUCAGUAAAAUAAUGUAGAGUAAAAAUUCUGGACAAUUUUCUUGUUUUAUGUUAUAU
env-1049   AUAAUAUUUAUUUCAUUAAAACAUCAGUAAAAUAAUGUAGAGUAAAAAUUCUGGACAAUUUUCUUGUUUUAUGUUAUAU
env-1052   AUAAUAUUUAUUUCAUUAAAACAUCAGUAAAAUAAUGUAGAGUAAAAAUUCUGGACAAUUUUCUUGUUUUAUGUUAUAU
env-1053   AUAAUAUUUAUUUCAUUAAAACAUCAGUAAAAUAAUGUAGAGUAAAAAUUCUGGACAAUUUUCUUGUUUUAUGUUAUAU
env-1054   AUAAUAUUUAUUUCAUUAAAACAUCAGUAAAAUAAUGUAGAGUAAAAAUUCUGGACAAUUUUCUUGUUUUAUGUUAUAU
env-1055   AUAAUAUUUAUUUCAUUAAAACAUCAGUAAAAUAAUGUAGAGUAAAAAUUCUGGACAAUUUUCUUGUUUUAUGUUAUAU
env-1056   AUAAUAUUUAUUUCAUUAAAACAUCAGUAAAAUAAUGUAGAGUAAAAAUUCUGGACAAUUUUCUUGUUUUAUGUUAUAU
env-1057   GAAAGCAUUAUUAUAAUAGUGGGAAGUAAGAAAAUUCUGGACAAGAAUCACAUUAUAUGUUAUAAAAUGUAGGGAAUUG
env-1058   UAUAUGGUGGAAGAUUAUAAUACAGGAGAAUACCCUCUGGACAAGCAUCAUAUUAUGUGUUAUAAACAAUAAGGAAUUG
env-1059   UGUUAUAUUCCGGAAUCAAUAUUGAAUAAAUAUAGACAGUAGACAACUUGUGCAUUUUAUGUUAUAAAGGUAACAACCA
env-1060   AACUAAUCGCAAUAUCCCACAGCAGAUAAAAUAACUAACAUCUGGACAAGGCUUCCAUUUUGUGUUAUAAAAAUAAAAA
env-1061   ..............................AUAAUGCUGGACAAUGAUUUCCUUUUAUGUUAUAUGUAAAUAACAAGCA
env-1062   AGCGAGUGAUUUCGCUGUCUGUAAAUAUAGAUAAUGCUGGACAAUGAUUUCCUUUUAUGUUAUAUGUAAAUAACAAGCA
env-1070   AGCGAGUGAUUUCGCUGUCUGUAAAUAUAGAUAAUGCUGGACAAUGACUUCCUUUUAUGUUAUAUGUAAAUAACAAGCA
env-1071   UCUUGCUGCUUCAUAAUAAGGGAAAAAACAAAUAAUGGUGGACAAGUUUUUUCUGGUAUGUUAUAACUAAUAGCAAUGA
env-1072   GAGAAUAUUUUGGUACUCAUAAAAAAAGGUUAAAUAAGUCUGGACUUUUUUCACCUUUUAUGUUAUAAAAAAUAACAAUUG
env-1073   UAUUAUAGAAUUUUAUUGAAAACAAUUUUAUUACAUUCUGGACAAAAUUGAUUGUAUAUGUUAUAAAAAUAGAAAAUUA
env-1074   ..............................AUACAUUCUGGACAAAAUUGAUUGUAUAUGUUAUAAAAAUAGAAAAUUA
env-1075   ...............................UACAUUCUGGACAAAAUUGAUUGUAUAUGUUAUAAAAAUAGAAAAUUA
env-1076   UAUUAUAGAAUUUUAUUGAAAACAAUUUUAUUACAUUCUGGACAAAAUUGAUUGUAUAUGUUAUAAAAAUAGAAAAUUA
env-1077   UAUUAUAGAAUUUUAUUGAAAACAAUUUUAUUACAUUCUGGACAAAAUUGAUUGUAUAUGUUAUAAAAAUAGAAAAUUA
env-1078   .........UCUCACGUGCGUUCGACUUGAAUACAUUCUGGACAAAAUUGAUUGUAUAUGUUAUAAAAAUAGAAAAUUA
env-1079   UAUGUAGAAUUAACACCAAAAUAUUAAAGAUAAUACUAGACAAUUCUUGUUUUGCAUGUUAUAAAUUUAUCGAAAAAUA
env-1080   AGCCUGAUUGUAUCGUGUAUCCAUGCUAUAGGGCAUUGAAAAAAGUUCUUGAUAUUUUGGAUCGUAUAGGAUAAACUUU
env-1081   AGCCUGAUUGUAUCGUGUAUCCAUGCUAUAGGGCAUUGAAAAAAGUUCUUGAUAUUUUGGAUCGUAUAGGAUAAACUUU
env-1082   GUUGCUAAAAACAGAUAAAAAGAAAUAAUUUCAAGAAAAUAUUGUAUAUAGAAUUACCCUGUGCUAUAAUUCUAAAAAA
env-1083   ..................................................GAAUUACCCUGUGCUAUAAUUCUAAAAAA
env-1084   UUGCUAAAAGCAUAUAAAAAGAAAUAAUUUCAAAAAAAUAUUGUAUAUAGAAUUACCCUGUGCUAUAAUUCUAAAAAAA
Ano-1-1    GUUGCUAAAAAUAGAUAAAAAGAAAUAAUUUCAAAAAAAUAUUGUAUAUAGAAUUACCCUGUGCUAUAAUUCUAAAAAA
env-1085   .......................................UAUUGUAUAUAGAAUUACCCUGUGCUAUAAUUCUAAAAAA
env-1086   .............................................AUAUAGAAUUACCCUGUGCUAUAAUUCUAAAAAA
env-1087   GUUGCUAAAAACAGAUAAAAAGAAAUAAUUUCAAAAAAAUAUUGUAUAUAGAAUUACCCUGUGCUAUAAUUCUAAAAAA
env-1088   CGUUGCUAAAAACAGAUAAAAGAAAUAAUUUCAAAAAAAUAUUGUAUAUAGAAUUACCCUGUGCUAUAAUUCUAAAAAA
Cst-1-1    GUUGCUAAAAGCAUAUAAAAAGAAAUAAUUUCAAAAAAAUAUUGUAUAUAGAACUACACUGUGCUAUAAUUCUAAAAAA
env-1089   CGUUGCUAAAACAUAUAAAAAGAAAAAAUUUCAAAAAAAUAUUGUAUAUAGAAUUACCCUGUGCUAUAAUUCUAAAAAA
env-1090   AGCCCGGGUAUUUCGUGGAUUCCGCAGAAGAAAGCGCUUGCAAAUUUUAUCUGCUUAUUAUAUAGUUAUACAAAACAUA
env-1091   AACUAAGGACAAUGGAUGGAACAAUGGCAGAAUUUUUAUAUUUGCAUUUUCUAAAAUUCUAUGGUAUGAUAGCCCCGUA
env-1092   AAAUUUUACAAAAAAGUGGAGUAGCAUGCCGUAAAAAUUAUGGUUGACACAGCCGGAUUCCCAUGCUAGAAUGGACGGA
env-1094   UCUGGUACAAUAUAAGCACUCGGCAAGGUAUCAUAUCAUAGUUCACGAGCCUGGUUGAGUCAAUUUCUGAUAAUAAUAA
env-1095   GCUUUUUCCGGCUGCCUGUGAUAGAAUGCACAGGAAAUGCGGUGAGACGCGUCACUGGCACGCUGCAGUGCAUUUUGCA
env-1096   UUUAUGUUGGCGUCAGCCGAAUCGCAGAAUUAACUUAAUGAUAUUGACUUUUCCCCACUUAUAUGAUAUUCUACUGGAA
env-1097   CGAACUUCACAUUAUAUUUAUUAACUGAGAAAAUAGCAUUGCCAUUUUGAUAGUUGUUAUGAUAUAAUGUUAUCAAAAU
Csp-17-1   GUAUUGUUAUAUCCGCUAAUACCCCCUAUAAAAAUGGGAUUGCCAUUUUAACAAUUGUUAUGAUAUAAUACUUAAAAAU
env-1098   .......................................................GUGAUAUGAUAAGAUAUUAGCAAC
env-1099   AGUGCCAAUGUUUACUUGACACAAACGUAGAUUUUGCUGCGAUUGACACUUAGAGCGGCUUGUGCUACAAUAUAUCGGA
env-1100   UGUUUAUUUCAUCAAAAAAACAGGAUAUUGCAGCUAUUUUCUAUUGAUUUAUCCCCACAGGCAUGGUACAAUACACAGA
env-1101   GAGUAUGUCUUAUUUUACCGGUAGGCAAAUAACGACUCCAAACGUUGACUUUUUAGCUCUUGUAUAGUAUACUAUAAAA
env-1102   AGGAAAUUUUUCAAUAACAUACGAGCAAUCCAAAUUUCCGCUAUUGUUUUUUCUCCUUCUGUAUGGUAUGAUAUAUAAA
env-1103   ACAUACAUAUAUUAUACAUACUAAGCGCUUUCUUGUAAAACUAUUGUUUUCUUUGUUUUUGUGUGAUAUCAUAUAUAGA
env-1104   CUGCUGUAUUAAGGAACCGUGUUAAAACGGAAAAAAUUAGCUGUUGCUUUUUUGGAUUCUGCAUUAUAUAAUAUACGAA
env-1106   GUCAAUCUGCGAGGAUUUGCUAUAAUAAAAACAAGGCAACAUUUUGGCAGCAGAAAAUCAAUAAGCUAAAUUAAAUUUU
env-1107   CACAAGCGAUACCGAAGACCUACACCGAAGAACAGAAAAAAUAUUUGACUUGCCUGUAAAACAGUGAUAGUAUAUCAAC
env-1109   CGUCCCACAAAAAUACGCCUCACCCGUCCCACAAAAAUACCAUUUGACUUUUCCACCCAUUCGUGCUACGCUGUACGUC
Lsp-6-1    CCUCCCCAAUAAACCAGCCUCACCCGUCCCACAAAAAUACCAUUUGACUUUUCCACCCAUUCGUGCUACGCUGUACGUC
env-1110   CUUCGACGUUUGCAACAAUGGGCCAGAUUAAAUCAUGAUUUGUUGUUGAUUUUUUCAAUUUAGCAUUAUAUAAUGCAAA
env-1112   UUCAGAGUAUUGGAUCUGGAUGGAUUGCUGCUGGUGUAAGUCGGUUCAAGUUGACAUUUAAAUAUUCAUAUGCUAAUCU
Lph-1-1    UUUAUACAACAUAGGGCAAAAGACAGCAUUGUACGUUGACAACCAGGAUAUCCUGUGCUACACUAAACGCAAAAUUGAA
Cph-1-1    UCUAAUUAUAUUAUGAAGAACAAAAUCAUACAGCGUUGACAAUCCGAAUAUCAUGUGUUACACUAAUCGCUAAAUUGAA
env-1113   CUCAAAGUAAAAGCAUGUAAAUUACAAAUUAUAAAGUUGACAAUAAAAUUACUAUGUGCUAAAAUCACCAUAAACUGAA
Csp-13-1   AUCCCUAUCUGCCGGAAAUAGUCAAUAUUAUAAAUUGACAAUAUAAACACCAUGUGCUAAACUAUUUUUGAAAAAUGAA
Lba-1-1    AAAAAUAGGUGAUUUUACAGAAUGGUAGUGUAGAUUGACAAGGAGUAAGGCAUGUGUUAAACUCAUUUAGAAAAUAGAA
env-1114   UGAAAUAUCCCGAAGAGGAAUAAAUCAUUCUAUUGACAAUAUACGUCGCAUUGUGCUAGAAUGUCGUAAAAUAAUUGAA

           ...............................................................................
           ...............................................................................
           ...............................................................................
           ...............................................................................
```

```
env-1115  UGAAAUAUCCCGAAGAGGAAUAAAUCAUUCUAUUGACAAUAUACGUCGCAUUGUGCUAGAAUGUCGUAAAAUAAUUGAA
env-1116  CAUCUAUUUUAUUGUUUCCAAUCAUUUCUUGAAAUUUUUGCUGGAAAGGUUGAUUUCAUGGUUUUUCAUGUUAUAUUA
env-1117  UUCCCACAGCUUCCUAAUUGCAUCCUCGUUUCCUAUGUCUAUCAUCGGCAUUGCGCUGAUUGACUUUUUAUGCUAAGCU
Mfo-1-1   GAAACAUCCGCCCGGCGCACAAAAGCCGGGCGGCGGAAAGAACACCGGGAGUGUCUGCAGAACAGGCAUUUCCGGUACA
env-1118  AGAAUAGAUGAAUAAAUAAUCUUUCAUACAAAAUUUUUUGCAAUUAGGAUUGAUUUAUUUGUCUUUUUAUGAUAAAUUA
Pma-1-1   AGCCGCGUAUAUCCCCCGGCGCGCGAAAAUUUCCCCACCCUCUUGCAAUUUUCGCGCGGGCCGUGCUAUAAUAAACCGA
env-1119  ...................................................................GGUAUGCUGGUA
env-1120  CCAAAGCUCUGCCGGUUCUGCGGGAAAAUGGACUUUAAAUUUUAGUUGACAGACCUGAAGUUUUCUGCUAUUCUUACAC
env-1121  UAAGGAAAAAGAUUUAAUAAAAUUCAAUAUCAUUUAUUCAUUUUGGGUAUUGCUUUUUAAUUUGUUUUGAUAUAUAAUA
env-1122  ACCCUGUAAUACAUAAAACAGGAUACAUGACAGGAAUUUGACAAAAACGAGACCCUGUGCUACACUUAUGUGCAGUAAA
env-1123  UAAAUGCGAAUACCGCCGAUGCGGCAGCUGAUUUUUAAAAAUGGUUGGCAAAUUUUUGACAACCUUUAAUUUUUAUGAU
env-1124  AGCUGGCUGUCAAACAUUUUGUACUGGGAACUGUGAAAAGAGUUGACAAGUAUCGGGUUUUUUGAUAGAGUAAACGGAG
env-1125  AAAUAUAGUUCUUAAAUAAAAAAGUCCUAAGAAUAGGACUUUUUUAUUUAUUGUAAUAUUAUUAUACCUUUGAUAUAAU
env-1126  AAAUAUAGUUCUUAAAUAAAAAAGUCCUAAGAAUAGGACUUUUUUAUUUAUUGUAAUAUUAUUAUACCUUUGAUAUAAU
Lsp-8-1   UAUCCGCACUGUCCGGAUACGAAGAAAAAUAAUUUUUUCUUGACGGGAAUUUUUUUAUUUGCUAUCCUAAUCAUGAGUU
env-1127  UAUCCGCACUGUCCGAAUACGAAGAAAAAUAAUUUUUUCUUGACGGGAAUUUUUUUAUUUGCUAUCCUAAUCAUGAGUU
Dsp-3-1   AUUAAACAGGAUAGGCAGAAAAAAGUCAACUCAUCUGAAUUUUUCCUUGACUCUUUCCUGGCAUUCUGAUAUUCUGAGU
Dsp-2-1   AUUAAACAGGAUAGGCAGAAAAAAGUCAACUCAUCUGAAUUUUUCCUUGACUCUUUCCUGGCAUUCUGAUAUUCUGAUU
env-1128  ................................GUAAUGUAAAUGAUAAACUUGACAAAAAAGGCUUGGCUGUAAAGUCA
env-1129  UUCCUGUUCUUUUCUGCCGUAUUUCCGUCAGAUUUUAAAAUAAAAUCACUUGACACCUUUCUAUACAGUUGCUAUCAUA
Lco-1-1   AUGUUUCACGUGGAACAAUCACAUUAAUACAGCCGACACCCCUUGGUUGACUCGCUGAUGGUUCUCAUGCUAUGCUUUU
env-1130  ..............................AGGAAUUUGAAUUCUUUACUCAGGACACUUCCGACAAGGAAGAAAAAUA
env-1131  UAUCACAUCUGCAGUAUGAAGUAAAGUGCGACGGUCUUCGCUUCAAAUGUAAACAGCUUGAUUUUUUUAUAUAUAAUGU
env-1132  .......AUAAACCCAAUUUAGGGGAACAAGAAAUUAAUAUGCUAACCCCCAAAUAAGGAUUUAUGUUAAAAUAGUUUU
env-1133  UGAUAAAGAGAUUUUAUAUAUGAUGUCUUGCUUUGAUUAAGUCAAUUGUUUCAAUUAUGGUUUAUGUUAAAAUAGGAAU
env-1134  CUUGUUCUAUUUUGCUUUUUUCUUUUUGCCCUGUGCAAAUGCUCUUGCAGAAAAAGGUUGCCAUUUUACGCUCUUCAUG
env-1135  UUNACUUGAAAACCUCCUGAUCUUUUUGGAAACAGACUCUCAUUACUUUAGGCUUGAAGCAGCACAAAAAAUAAAGUAU
env-1136  GGGAAAAGCGGUUUUUUUAAAAAGACCAAAGCCGGAGAAAAGAGGACUUGUAAACCGGGAGGAGAUAUGCUACAGUAUG
Ssp-1-1   CAGAAAUUGUUGUUAUCAAUUUUGAUAAAGUAGAAUAACAUAUUCAAAAAAUUUUUAGAAUAUGUUAUAAUAUCGAUUA
env-1137  AACUUAUCUAUUGUAAUCCUACACUGGCAGAGCGCAGCGAAAUGUAAGUCCCGAUUGACAUUUUGCAGAGCUUGUGAUA
env-1139  .........................................GAGUUUCGGGCAACAACGUGGACGGGUUCAUCGUUACC
env-1140  UAUCUGUGAAUAUUUUAAAUUGCUUAGCUUAGAGGUUGUAGAUAUUCUUUACAGUAGUUGUUAGUUAUGUUAUUAUAAU
env-1142  ...........CCCUAAUACUAUUAUACUGAAAUGAGCAGAAAAAUCAAUAGGCAUUGACAAAAAAGACAGGAUGUGAU
env-1143  ...........................CGGAAUAUGAAAAAAUGCUUCAUGACACAGACUGGAUAAAGCAGCUUUAAGG
env-1147  ................GGUACCUGCCGCGGAAUAUGAAAAAAUGCUUCAUGACACAGACUGGAUAAAGCAGCUUUAAGG
env-1148  CCGCCGUUUAUGGUAUAACGAACCCGGCUAGGCGGCGCUAUGGUAUAUUGUCGCCAGCGUGCCGCGGGCGGGGGGGGA
env-1150  UUUCGGUUGCUGCUGAAAAAAUGGGGAAACUCAAACCAAAAAGUAUUGACAAACCGGCGGCACAGUGUAUAAUAUACCU
env-1151  .......AAGGACACGUGGAACCAGGCGGAUCUGUAGGAAAAGUGCAGGUCGAUUGACAGGAAAUGAAGUAUAUGUUAU
env-1153  UCGUAUUUGCGAAAGGAAUAUUCAAGCCAACAUAAUUCACAGAUUUUGUUGGCUUGAAUCAUUCUUUCAUUUACUGUAU
env-1154  GGAGGAAGUGACGGUUCUCCGUGCAGGGCGGUACUUAUCUGCUGAGCUUGACUUGAUGACCGUAAAGUGGUAUAAUAUC
env-1155  ........UGACGGUUCUCCGUGCAGGGCGGUACUUAUCCGCUGAGCUUGACUUGAUGUCAGCAAAGUGGUAUAAUAUC
Rfl-1-1   GGAGGAAGUGACGGUUCUCCGUGCAGGGCGGUACUUAUUCGCUGAGCUUGACUUGAUGUCAGCAAAGUGGUAUAAUAUC
env-1156  GGAGGAAGUGACGGUUCUCCGUGCAGGGCGGUACUUAUCCGCUGAGCUUGACUUGAUGUCAGCAAAGUGGUAUAAUAUC
env-1157  GGAGGAAGUGACGGUUCUCCGUGCAGGGCGGUACUUAUCCGCUGAGCUUGACUUGAUGUCAGCAAAGUGGUAUAAUAUC
env-1158  .................................CUUAUCCGCUGAGCUUGACUUGAUGUCAGCGAAGUGGUAUAAUAUC
env-1159  ACACUAGGCUCCAAAUUGCCGCUGGGUGUCCGGGUUUUCAAAUCUUUACAUUGUUUUUGAGCUUGUAACUUUUUUCAGU
env-1160  ACACUAGGCUCCAAAUUGCCGCUGGGUGUCCGGGUUUUCAAAUCUUUACAUUGUUUUUGAGCUUGUAACUUUUUUCAGU
env-1161  GCGCCGCCGGGAUCGCGCAGCGAAAAUGCCGCCGGCAUAAAAAACACUUGCAUUUUUCGCCGGAUAGUGCUACAAUAUG
env-1162  ......................AUUGUCAGCCAGGCGAUUUAUUUGAAUAUGUAGAAGAUAUUGAUUCCUAGAGAAAUC
env-1163  AGUCACAUCUAGAUGUUUGACUUACCCAGAAAAUGAAUAGUAUUGAUUUGCUGAUCAUCUUGUGAUACAUUUAGUUUUA
env-1164  ................................ACUAUGAGUUCUACCGUGACAAGUAUUCGCUCAAAGAGAUAAAAGCG
env-1165  ..............................................UCGUCUGCUUCGGCGUGUUCAGCCUGAUCGGAG
env-1166  ..............................................UCGUCUGCUUCGGCGUGUUCAGCCUGAUCGGAG
env-1180  ..............................................UCGUCUGCUUCGGCGUGUUCAGCCUGAUCGGAG
env-1181  GGGCCGCCGGAGGACAAAUCGCCCUGAGCAGGCGGUAAAAUUCUGCUUGCUUUUUCCCUUCGGAUACAGUAUAAUGGGC
env-1182  ACGAGGAUAUUUGCCUCUGCUAUAUUACUCUUUAAUGUUAAAUUUAUUUGACAUUAAAUCUGUUGACAAUAAAAUCUUA
env-1183  AUGAGGAUAUUUGCCUCUACUAUAUUACUCUUUAAUGUUAAAUUUAUUUGACAUUAAAUCUGUUGACAAUAAAAUCUUA
env-1188  ACGAGGAUAUUUGCCUCUGCUAUAUUACUCUUUAAUGUUAAAUUUAUUUGACAUUAAAUCUGUUGACAAUAAAAUCUUA
env-1189  CUGUUAUCAAGUUUUUUUAGUAAAAAAUUUAAUUUUUUAAAAUGAUAUUGACAUUUUAUAAAAAUUAUGAUAUUAUAUA
env-1190  ...................GUAAAACAAAAAAGGGCAGGCUUGCGGUUGACAUUCUGCUUUAUAAAUGAUAUAAAUAUA
env-1191  UACAUCGAACAUGCACCUAGAAAUAGGAUUGCGAUGUAUCUUUUUUUAUGGUUGACAAGUUAUUUUUUAUUUGCUAUUU
env-1193  UACAUCGAACAUGCACCUAGAAAUAGGAUUGCGAUGUAUCUUUUUUUAUGGUUGACAAGUUAUUUUUUAUUUGCUAUUU
env-1194  .......................................CAGUUUUGGUUAAGGUAUUGCCUAAAAUCCCGUGUUGUGG
env-1195  AUACCAUAUGAUAUCUGUCCUUUUCCAGCAAAAAAACAGCAGUUUUGGUUAAGGUAUUGCCUAAAAUCUCGUGUUGUGG
env-1196  GCUCGAUAACGGGUCGCCUGUUGCUUUGUAAUGAUAAAUUGACUUUAAAGUGCCAAUAUGAUAAAGUCAUUUUAUUAAA
Cbu-1-1   AAUUAUGUAGGGAACUUUUUAGUAAAAAACAAAUUUUGUAUGGAAGAAGGAAUAUAAGUUGAUUUCUUUUCCAUAAAUG
Csp-9-1   AAUUGUAUAGGGAGCUUUUUAGUAAACAACAAAUUUUGUAUGGAAGAAGGAAUAUAAGUUGAUUUAUUUUCCAUAAAUG
env-1197  AAUUAUGUAGGGAGCUUUUUAGUAAACAACAAAUUUUGUAUGGAAGAAGGAAUACAAGUUGAUUUCUUUUCCAUAAAUG
Cbu-3-1   UAAUUAUGUAGGUAGCUUUUAGUAAAUAACAAAUUUUGUAUGGAAGAAGGAAUAUAAGUUGAUUUCUUUUCCAUAAAUG
env-1199  UGAACACACGGUAUGACGUGAUAUUCGGAUCGCAGUCGGCUUGCCGUUCCGUGUCGGUGCGCGGUCGGCGGUAUGUCCG
env-1200  AAAAUCGGAAAUGCGGCGUUUGUUAAGCAGGCAUAAAUAAACUGCAAUUGCUCCUUGACAAACCGAAAUCGUUAUGCUA
env-1202  AAAAUCGGAAAUGCGGCGUUUGUUAAGCAGGCAUAAAUAAACUGCAAUUGCUCCUUGACAAACCGAAAUCGUUAUGCUA
env-1204  UCGGAAACGCAACCUUUGUCAAAUCUGCGUAAACGCAUAACGAUUUAUAAUAUCUUGACAAGCACGAGCCGAUAUGCUA
env-1205  AUGCUUCUCACCGUAGUUUUCUGAUCUGCAGAAAUACGGCAAAUUGCCGGGGGUGACAAUCCCGACAGCUUGCCGUAUA
env-1206  UGCACAAAACGGAAACGCAUUGAAAGAAAAUUUUAUGCAUUAAAAGUAUUGAUUUUCUUGCCAGUUAUGAUACAAUAAA
env-1208  .........................................GCAUUAAAAGUAUUGAUUUUUCCGUCGACUAUGCUAUA
env-1209  CAAUUUGCUCAAAGUCGAAAAACAUUGAGAGAAAAUCUUGUGCAUUAAAAGUAUUGAUUUUCCCGUCGACUAUGCUAUA
env-1210  CAAUUUGCUCAAAGUCGAAAAACAUUGAGAGAAAAUCUUGUGCAUUAAAAGUAUUGAUUUUUCCGUCGACUAUGCUAUA
env-1211  ........AUGCUGAACAGGGAAUGUAUGCUGAGGCUUCUGAUUAUAGAAAAAUGUAAUCCUCAGGCAGCUGAAAAUGU
env-1212  ........AUGCUGAACAGGGAAUGUAUGCUGAGGCUUCUGAUUAUAGAAAAAUGUAAUCCUCAGGCAGCUGAAAAUGU
env-1214  .............................................UGGAACUUACACCCGAACAUAUUACGGGUAAACU
env-1215  AGAAUAUUUUGAUAAGCAUCAAAAGGGAUGAUAUAAAAUUAUGAAAAAUAUAUUUCAUUGACUUUAUAUUUUAAAUAUG
```

```
env-1217  UACCUGUGAACAGCAACAAUAUCACCUCUGAACAAUUACAUCAAAAUAAAUUCUGCUUGACUUUCUAUUUUUGCUAUGG
env-1220  CUCACUCAUCCGCCGUUUUUAUUCCCAUAACAUUUUCCCCAUAUCCCCCUUGACUUUCCCUCCGCGUUAUGAUAUAAUG
env-1221  UUUAUCGUACUAUGAUUUAGUAUAUCACAACUUUUUCUCAAUUUCAAUGGGGAUUUGACUUUUUCUUCUAAAUAUGAUA
env-1222  ........................CUCACGAGAUUCUGCACACUUCCUAUAAAGCUUCAAAUUUGCAUAAAUAACCGUU
env-1223  UUAAUUGUUUUAUUCCUUCCAUGUAGUAACCUCCUUGUUUUUCGUAAGCAUUGACAAAUGCUUAAAUUUUUGAUAUAAU
env-1224  ...........AAGGCUAGGAACAGCUGUAUGUGGCUUUGGCUGCAUUUGCAAUAUCAGUAUUUCGCUGGUAUAAUGCU
env-1225  ....UUUGCGGCGUACUGCUGCUCUCCAAAAAGAAAAAGUCGGAAAACACUUGACUUUUCGCCAAGUCUGUGAUAUACU
env-1226  ....UUUGCGGCGUACUGCUGCUCUCCAAAAAGAAAAAGUCGGAAAACACUUGACUUUUCGCCAAGUCUGUGAUAUACU
env-1227  CGGAUGGUGACGUCCCGGGAAGAUAGGAAGAUGCCAACAUUAAAAAGAGGCUGUGAAGAUCAGAGGAUUUUCGCAGCCU
env-1228  ACAGGCGUUACACCGCUCGAAUACAGAAAAAAUCCGGUUUAAUUAGGUAUUGACUUUUUGGAUUUAAUAAGUUAUAAUA
env-1229  GACUGUUUCCAUAUAAAAAACAAUUUUGUUUUUAUGAGCGUAAAAUUUUGACAUUAUUGAAAUUCUAUGUUACAAUAGU
env-1230  AUUAUAUUUCUUAACAAAAUUUUAUUUAUCGCGGAGCGGUUAAAUAUUUGACUUAGAAUAAAAUACGUGUUACUAUAAA
env-1231  AUUUAUAUAAACUUGGGUAUGGGGUAAAACGCGACAUGCAAAUCCCCUUGACUUUAUUUUAGAUUUAUGCUAUAAUACC
env-1237  AUUUAUAUAAACUUGGGUAUGGGGUAAAACGCGACAUGCAAAUCCCCUUGACUUUAUUUUAGAUUUAUGCUAUAAUACC
env-1238  AUUUAUAUAAACUUGGGUAUGGGGUAAAACGCGACAUGCAAAUCCCCUUGACUUUAUUUUAGAUUUAUGCUAUAAUACC
env-1239  ..................................CAUGCAAAUCCCCUUGACUUUAUUUUAGAUUUAUGCUAUAAUACC
env-1240  UUUAUAUAAACCUGUGGUAUGGGGUAAAACGCGACAUGCAAAUCCCCUUGACUUUAUUUUAGAUUUAUGCUAUAAUACC
env-1241  AUUUAUAUAAACUUGGGUAUGGGGUAAAACGCGACAUGCAAAUCCCCUUGACUUUAUUUUAGAUUUAUGCUAUAAUACC
env-1242  UAGCAAUCACACGUUAAACGAAAACAGCAAAUCCCGUCGAAAAACACCUUGACAUUGCGCCUUGUUUAUGUUAGGUUAA
env-1243  CGAACGAUUAAACGCGAUUGAAAAUCGCAAAUCACACCUAAAAACGCCUUGACAUCGAGGCGAGUUUAUGUUAGGUUAA
env-1244  CGGAGUGGUCGGCGUGAAAAUUUUUAAGCAAUAUUUCGCGUUUAAACCUUGACAAUAUCCGUGACGUAUGGUACUAUAC
env-1245  AAAAUCAAAGCGCCCGCUUCCGCGCCCGGAAAUCCGCCGUGUGACAGAGCUUGACUUUCUCAUACCUCUAUGCUAUAAU
env-1246  CACAUAUUUGAAACGAGGAAAAAAAUUUUUUCUCGGAGAAAUAUUUUUGCUUGCCAAAAGCGCGGGGGAUAUGGUACAA
env-1247  CGACACAUACAGCAAGCUUAUUUUUAAAUUUAGAGUAAGACAGCAAGAAAGUUAUUGAAAAACGGGAGAGCUUCUGAUA
env-1248  GUUUGUCAACCUAUUUUCAUAAAUAAUGGUAUAAAAGAUAAAAACGCAUUCCAGCUUGACAGUUUAGUGCUUUUAUGGU
env-1249  AAUAUUUAUGAAAAUAUAAAUACGGAGAAAUAAUAAUCUCUAUUAAACUAUUGACCAAUAAAAAAAAUAAUAUAUAAUA
env-1250  ................................................................................
env-1251  AAUAUUUAUGAAAAUAUAAAUACGGAGAAAUAAUAAUCUCUAUUAAACUAUUGACCAAUAAAAAAAAUAAUAUAUAAUA
env-1252  ..........AAGAGGUUCAUCGUGAGCUUAAUACCUCCGAGGAGUUUUCCGCCGCCUGAUAUUGACAAUUCAAUAUUG
env-1253  .........AAGCGGUUCACCGAGAGCUGAAUUCAUCGGCUGAAUACAAUUUGCAAUAAGUAUUGACAACAGCAUUUUU
env-1254  UCGUCAGAAAAUCGGUUUACCGAGAGCUGAAUUCAUCGGUUGAAUACAAUUUACAAUAAGUAUUGACAAAGGCAUGAUA
env-1255  AGAGAAUGGAAUGAACUUAUAAUUAUGCGCUCCGUGUUGUAUAUUCUUGACUUUAAAUAUCCCCUGUGAUACUAUUUUU
env-1258  .........................UUUCACUUUAUCUGGCGAGACUGGCUAAGAGAAGAAAAGAAGGAAAAUAACAAU
env-1260  CAAUACAAGAUAUAGCCGGCUGAACUAAAACAGCCGGCUUUUUCAAAAUAAUGCCCGCUCACUUGACAUACUUCAUUCA
env-1262  ....................ACGCGGAAAUGACGGUCGGCUCAAUUAAAGAUUAGUCAUAAUCUUGACAAACGCGGAAA
env-1263  .....ACCUCCGUCUUAAGAACGCGGAAAUGACGGUCGGCUCAAUUAAAGAUUAGUCAUAAUCUUGACAAACGCGGAAA
env-1264  ...............................................................UUCUUAAAAGUGUUGAA
env-1266  UAACUUGUAAAUUUGCUAUUUUAUUAUGAAGUAAUAAAUUUCAGCUAAAUUUUAUUUGACAGUAUUUUGAUAAUAUGCU
env-1269  UAACUUGUAAAUUUGCUAUUUUAUUAUGAAGUAAUAAAUUUCAGCUAAAUUUUAUUUGACAGUAUUUUGAUAAUAUGCU
env-1270  .......................................................UGAUAAUUGCAACAAUAUUUAUUAU
env-1271  ........UCAAAAAGCUGGUAGGAGUUACACCUACGGGCUACAGAAAAAAAGGAUAAAAUUUUAAGAACCUGUUCACA
Eba-1-1   ACUGCUCUUCCAAUGUAUCCCUGUCGAAGGAAAGUCAAUGCCAUUAAGCCAUUCGUGAAUUGAUUUUUUGAACAUAAGC
env-1272  GUCAUUUUCCAGUGAGUAUAGAAAUUUAUAGAAAAAAUUCCGGAAGACUUGACAGCUUUUCCGGAUUGUGGUAUAAUAA
env-1273  AGAAAUUUAUUGGUAAUUUUGCACAAAGAAUAUCAUAAACUGGAAAAUACUUGACAAAUUUGUAAAAUGAUGCUAUAAU
env-1274  UAAUAGAAAAUCUGUAACGCUGUUUUUUUGUCAAUUCUUCCGGAAUGCUUGACAGUUCCGGAAUUUUGUGCUAUAAUAG
env-1275  ACCGCUCUUUCUCUGUUAUAUUCCAUUUCGCACCUCCGGUUCAUUUUUUUAUUGACUUUUUACCUCUUAUAUGCUAUAC
env-1276  ................................................CAAACAAAGUUGAUUCUUUCAAGGAUUUUAUA
env-1278  ACUAAUCUUAUGAAGGUAGAUACUGUUAAAUGAUUCUGGAAGCAUGUUUUAAGCUUGACAUGUCAGUCUACGCAUGAUA
env-1279  ACUAAUCUUAUGAAGGUAGAUACGGUUAAAUGAUUCUGGAAGCAUGUUUUAAGCUUGACAUGUCAGUCUACGCAUGAUA
env-1288  ACUAAUCUUAUGAAGGUAGAUACGGUUAAAUGAUUCUGGAAGCAUGUUUUAAGCUUGACAUGUCAGUCUACGCAUGAUA
env-1289  ACUAAUCUUAUGAAGGUAGAUACGGUUAAAUGAUUCUGGAAGCAUGUUUUAAGCUUGACAUGUCAGUCUACGCAUGAUA
env-1290  ACUAAUCUUAUGAAGGUAGAUACGGUUAAAUGAUUCUGGAAGCAUGUUUUCAGCUUGACAUGUCAGUCUACGCAUGAUA
env-1291  ACUAAUCUUAUGAAGGUAGAUACGGUUAAAUGAUUCUGGAAGCAUGUUUUAAGCUUGACAUGUCAGUCUACUCAUGAUA
env-1292  ........................................CUCUGACAGAGCUGUGCGACCUGCUUAUUCCGUCAGCGG
Cbo-1-1   GCAUUUUGUAAAAGUUAAAAAUAAGACUGUAGCAAUAACGAUAUGUUUUAUUUAUUUACAAAGAAAUUUGUAUUUGCUA
env-1293  AUAUUCGGGUGAUAUAAUACAAAUGUUGAAUAUGUUGAAAAACCAGAAAGUUUUGUUGUAUUAAAUAUAAAAAAUUAAU
env-1294  UUCUCUCUUUUUCGACAGUAUACCAUACUCCCACGGUUUUGCCAAGGCAAAAUUUCUUGACGCGCGGCGGAUUUUCAGG
env-1296  UUCUCUCUUUUUCGACAGUAUACCAUACUCCCACGGUUUUGCCAAGACCAAACUUCUUGACGCGCGGCGGAUUUUCGGG
env-1300  AGUGAUGUUAAACCGGAUUUGAUUUGUCAAAAUAUAAGAGAAGUAUAUAUUUCUUAUUCAAAAUUUCUCAAUCAUAUGU
env-1301  CGCGCAAAAGAAUUGCAGGAGGUUAUUAAAAAAUUCAAAAUAUAGAUAGUUGCAUUGUUUUAAUAAAUAUGAUAUUUUA
env-1302  ........................................UAUUGAAUAAGAGAUUUACUUAUGAUAUAUUAAUAGCAA
env-1303  CAACUCCUUUCUACUAUUUUAUAACAAAACCGAGCAAUAAGCAAGUGUUUUUACAAAAUUCUUGACAACGCUAAAAAUA
env-1304  CCGGCGCGCUCGUGGAGAUAGAAAUCGUCGCCGCAAGAUAAAAACGCUUGACUUCCGGCGCGGCGUCGGAUAUAAUAGC
env-1305  CCGGCGCUCUCGUGGAGAUAGAAAUCGUCGCCGCAAGAUAAAAACGCUUGACUUCCGGCGCGGCGUCGGAUAUAAUAGC
env-1306  ACCGUGCUUAAAAAACUGCAUACUGUACUUGAUAAAUAUUAUGAUUGACUUUAUCGCCGAUGUAGGUUAUAAUCAUCAA
env-1307  AAAAAAUAUCCUAAGUGCGAAGCCGAACUUGAUAAAUAUUAUGAUUGACUUUAUCGCCGAUAUAGGAUAUAAUCAUCAA
env-1308  CCGUGCUUAAAAAAACUGCAUACUGCACUUGAUAAAUAUUAUGAUUGACUUUACCGCCGACAUAGGUUAUAAUCAUCAA
env-1309  ACCGUGCUUAAAAAACUGUAUACUGCACUUGAUAAAUAUUAUGAUUGACUUUAUCGCCGAUGUAGGUUAUAAUCAUCAA
env-1310  ACUGUGCUUAAAAAACUGCAUACUGCACUUGAUAAAUAUUAUGAUUGACUUUACCGUCGACAUAGGUUAUAAUCAUCAA
env-1311  ..........UGGCGGAUAUGGAAGGAAGCAGUAUUCCUCCAAAUGGGUUGCAAAAAUGAAAAAAAUGAAGUAUAGUAA
env-1312  GCGUACGCAGAGAGUACCGUUUCUUGGGGCUGCGUAAGCUUUAACAACCAAUUCAGAGUAUUAUCCGAACCAUAAUAAU
env-1313  GUAGCCUUUUCGCCAACUUACGCCAUUUACGCCAAACUUACGCCAAACGGUGCAGAAAGUUGUGCAGAGCGUAAUCAAU
Bma-2-1   AAGAGGGCGAAAAAUCCCCGCUCACCCUGCAAAAGGGGGAUUCUCGCGGCCCGCCCUUGUGUUUUGGCGGAGGCUGUGC
env-1315  AAGGGGGCGAAAAAUCCCCGCUCACCCUGCAAAAGGGGGAUUCUCGCGGCCCGCCCUUGUGUUUUGGCGGAGGCUGUGC
Csp-4-1   AAGGGGGCGAAAAAUCCCCGCUCACCCUGCAAAAGGGGGAUUCUCGCGGCCCGCCCUUGUGUUUUGGCGGAGGCUGUGC
env-1316  UUUAGUGUUGUAAUAAAAUAAUUCUUGUAAAAUAAAGGUUGUAUGUUAUAAUGACAACACAAGAAACGUCUAAAGUUAA
env-1317  UUUUCUAAAUGUUUUUUCAAAAGCGAAGUUUUAUCUUUAUUUUUUUGCUUGCCUUUUUAUUCAAAACCUGUUAUAAUCA
env-1318  .................................AUCCUUAUUUUUUGCUUGCCUUUUUAUUCAAAACCUGUUAUAAUCA
env-1319  ..........................AGUUUUAUCUUUAUUUUUUUGCUUGCCUUUUUAUUCAAAACCUGUUAUAAUCA
env-1320  CUUUUCCAAAUGUUUUUUCAAAAGCGAAAUUUUAUCCUUAUUUUUUGCUUGCCUUUUUAUUCAAAACCUGUUAUAAUCA
env-1321  ...UCUAAAUGUUUUUUCAAAAGCGAAGUUUUAUCUUUAUUUUUUUGCUUGCCUUUUUAUUCAAAACCUGUUAUAAUCA
env-1322  CUUUUCCAAAUGUUUUUUCAAAAGCGAAAUUUUAUCCUUAUUUUUUGCUUGCCUUUUUAUUCAAAACCUGUUAUAAUCA
```

```
env-1323  UUUUCUAAAUGUUUUUUCAAAAGCGAAGUUUUAUCUUUAUUUUUUUGCUUGCCUUUUUAUUCAAAACCUGUUAUAAUCA
env-1324  UUUUCUAAAUGUUUUUUCAAAAGCGAAGUUUUAUCUUUAUUUUUUUGCUUGCCUUUUUAUUCAAAACCUGUUAUAAUCA
env-1325  UGUUCGGGUUCUCCUGCGGAUAAAGAGGGCGGAAUUCCGAGCGGAUGUGUCCUUUGCCGGAUACGCUCCGAUUUGUAGC
env-1326  CUUGAUUUUUUUAGAAAGUGACAGUAUACUAUACCUCGAAAUCAGUGAUCCCUCUGUCAGAGUGGAUUCCAACUUAUUU
env-1332  CUUGAUUUUUUUAGAAAGUGACAGUAUACUAUACCUCGAAAUCAGUGAUCCCUCUGUCAGAGUGGAUUCCAACUUAUUU
env-1334  UCUCAAAAGUGCCCGUAUACUGUAUCACGAAGCGGACGCCGCAGGAGCGCCCCAUACGUUUCAGACAGCAAACUGCUGU
env-1344  UUCUCAAAAGUGCCCGUAUACUGUAUCACGAAGCGGACGCCGCAGGAGCGCCCCAUACGUUCAGACAGCAAACUGCUGU
env-1345  UCUCAAAAGUGCCCGUAUACUGUAUCACGAAGCGGACGCCGCAGGAGCGCCCCAUACGUUUCAGACAGCAAACUGCUGU
env-1363  .................UACUGUAUCACGAAGCGGACGCCGCAGGAGCGCCCCAUACGUUUCAGACAGCAAACUGCUGU
env-1364  UCUCAAAAGUGCCCGUAUACUGUAUCACGAAGCGGACGCCGCAGGAGCGCCCCAUACGUUUCAGACAGCAAACUGCUGU
env-1365  UCAAAAAGUUGCCCGUAUACUGUAUCACGAAGCGGACGCCGCAGGAGCGCCCCAUACGUUUCAGACAGCAAACUGCUGU
env-1366  GCCGGACGGGCUUUUUUGCAGGUAUCUUCUGCCCAAAAUUUCCGCUUGACAGCGCUGUUUUAGUGUGUUAUGCUGUCUG
Mma-1-1   GUAGAGUAGAAAUUAUGCACGGCAUUGCGGAUUUUUGGAAAAAUGAAGCCUUGACUUUGCUGAGGGGCUGUGCUAUAAU
env-1367  AGAUUGCGCUUGCGUCUGAGUCGGAUGAGUUUUUUUUUCAAAAGAAUAGACAAAAUCGGCGCAGUGUGUUAGUAUAAUC
env-1371  GAUUGCGCUUGCGUCUGCGUCGGAUGAGUUUUUUGUUUCAAAAGAAUAGACAAAAUCGGCGCAGUGUGUUAGUAUAAUC
env-1372  GAUUGCGCUUGCGUCUGCGUCUGAUGAGUUUUUUGUUUCAAAAGAAUAGACAAAAUCGGCGCAGUGUGUUAGUAUAAUC
env-1374  ...............................................................................
env-1375  GAUUGCGCUUGCGUCUGCGUCUGAUGAGUUUUUUGUUUCAAAAGAAUAGACAAAAUCGGCGCAGUGUGUUAGUAUAAUC
env-1377  GAUUGCGCUUGCGUCUGCGUCUGAUGAGUUUUUUGUUUCAAAAGAAUAGACAAAAUCGGCGCAGUGUGUUAGUAUAAUC
env-1378  GAUUGCGCUUGCGUCUGCGUCGGAUGAGUUUUUUGUUUCAAAAGAGUAGACAAAAUCGGCGCAGUGUGUUAGUAUAAUC
env-1386  GAUUGCGCUUGCGUCUGCGUCGGAUGAGUUUUUUGUUUCAAAAGAGUAGACAAAAUCGGCGCAGUGUGUUAGUAUAAUC
env-1388  AGAUUGCGCUUGCGUCUGAGUCGGAUGAGUUUUUUUUUCAAAAGAAUAGACAAAAUCGGCGCAGUGUGUUAGUAUAAUU
env-1392  GAUUGCGCUUGCGUCUGCGUCGGAUGAGUUUUUUGUUUCAAAAGAGUAGACAAAAUCGGCGCAGUGUGUUAGUAUAAUC
env-1393  AGAUUGCGCUUGCGUCUGAGUCGGAUGAGUUUUUUUUUCAAAAGAAUAGACAAAAUCGGCGCAGUGUGUUAGUAUAAUU
env-1399  ......................................CAAAAGAGUAGACAAAAUCGGCGCAGUGUGUUAGUAUAAUC
env-1400  GAUUGCGCUUGCGUCUGCGUCGGAUGAGUUUUUUGUUUCAAAAGAAUAGACAAAAUCGGCGCAGUGUGUUAGUAUAAUC
env-1401  ...................................UUUCAAAAGAAUAGACAAAAUCGGCGCAGUGUGUUAGUAUAAUC
env-1402  AUCCAAAGGGUUUGCGUCUCAGAGACGCAUAACGUCGGGUAAACGGUCGAUAAGAGUUGACUUAUUAUUGCUUUGAUGU
env-1403  .........CAUAUUUCAAAAAUACCGUCCCUUCGCGCGUCUUUUCGGUUGCCUUUCUCUGUUUCUUAUGAUAUAAUAC
env-1404  UUUCGCGACCAUAUUUCAAAAAAACCGUCCCUUCGCGCGUCUUUUCGGUUGCCUUUGUCUGUUUCUUAUGAUAUAAUAC
env-1405  AUGCGGGAAAAUAAAGCAGAUUCGUAAUUCGCCGGCCGAUUUGAAAAAUACCCCUUGACAACCCGUUUCCCGUCUGCUA
env-1406  AUGCGGGAAAAUAAAGCAGAUUCGUAAUUCGCCGGCCGAUUUGAAAAAUACCCCUUGACAACCCGUUUCCCGUCUGCUA
env-1407  ........................................GAUUCUGGGAAAGCAAGCUGAAACAGUUGGGCUUGACGG
env-1408  ........AACGCUUUUUCAAGUAUGUACGCCCCGUAAAAAAAACCGGAUAAGGCUUGACAAAGCCUCAAAAAGCUAAUA
env-1409  ..................................................................AUGGUAUAAUGAC
env-1410  ..........................................CUAUUGUUUUUUGAGGUGUUAUUUAUUAUAAUAGAUA
env-1412  GACGGUUUUUGUCCUGUUUUGCCAGUCCGGUAAAUUCAUUAUAAAAUUUCUUGACUUUUUGUCUUGUAUAUGAUAUUAU
env-1413  AAAAGAAAAGAAAAACACUGGAAGAAUAAAUUGUUUUGCAAAAAAAUUUCUUGACUUUUUGUCUUGUAUAUGACAUUAU
env-1414  .......AAUAUGUAACCUCACAGGUAAGAGUAUAAGCCUUUGAUAAUGGUUCUUGACUUUUCUGCAUUAUUGAAUAUA
env-1415  ..............GUUCGCUGGAGAGCGCUUGCCGCCUGAAAAACGCCGAGCUCGCGUCUUGACAAGUUCGGAAAAAU
env-1416  UAGCUUUUAUAUCGCCGCAAGUAAUGCCGAUAUACAGAAAAACUGUUGACACGGGCGUUUUAAAGUGAUAUGCUGCGCU
[gl-1-1   AUCGCUCAUAUCAUAGGAUAAGAAAUCGGGGGAAUGUUGGCUUUGCGCCAAUGUUCUCCCGGUAUUGUGAAGAAGAAUU
env-1419  ........................................AAAAAGUCCGCGAAAAAAUGGAAAAGCUCCUUAACGGGG
env-1421  ACUUCCAUCUGUGUUGCUCCAUGUAUUUUAAAUCAUCCUGUUUCUUAAUUGCUUUUUUCCUCUUCUCAUGUUACCAUAU
env-1422  AUUUCCAUCUGUGUUGCUCCAUGUAUUUUAAAUCAUCCUGUUUCUUAAUUGCUUUUUUCCUCUUCUCAUGUUACCAUAU
env-1423  AUUUCCAUCUGUGUUGCUCCAUGUAUUUUAAAUCAUCCUGUUUCUUAAUUGCUUUUUUCCUCUUCUCAUGUUACCAUAU
env-1424  .............................................................................UG
env-1425  UGCACAUCAGUGCAGGAUUCUUACUUCUUCACUAUUCACUCUUCACUUUUCACUUCCCCUCUUCCCUUUUCCCUUAUUA
env-1427  CGUUCCCGAUUUGACCCAAAAAUGCUGUACUGAUUUCCUUGCGUUUUUUCUUAAUAUAAUGUAUUAUUAUUGUAAAAUA
env-1428  ..............................................................UGGCUCCAUAUGGUAUA
env-1429  UGAUAUACGAAAACGCGAGACGGAGGAAAAUAUCGCCGGCUCCCCUGUGAAGCUGCAUUCUUUUAGAAAAUAGGCUCCA
env-1431  UGUUAUACGAAAACGCGAGACGGAGGAAAAUAUCGCCGGCUCCCCUGUGAAGCUGCAUUCUUUUAGAAAAUAGGCUCCA
env-1434  ...............................................................................
env-1435  AACAAUGACGAAAAAAAAAGCCCCAAAAUUUAUUUUUUUACUCCGUAUGUAUUGACAUUUUGAAAAACUGUGUUAAAAUA
env-1438  GAUAUAAUGUCUGCUGUCAAUCCCGCUAAGAGUAUUUUAUAGGAGCGCGAAUCCAAAUUAUUUCGGAUAAGGAUUUAA
env-1439  .............................CUGCCAAGAAUAUCCAGGUACUUGCACAGUACCCGGUACUGGUUUCCUGA
env-1440  UCCUUUUUAUUAUCUAUUAUUAUAUCAAAUUUGAAAUGAUAUACAAGAGCUUGACUUUAUAUUUUGUUGUGAUAGAAU
Cbo-3-1   UAAAAAAUAUUUUUAUAAGACAUAUAACAUAUAGAUAAAGGAGAAUUUGACUCUUUUAUUUAGAAAUGAUAUUAUUAAA
env-1441  UGCAAGCGCAGGCAGGAAUGAUCAAUAUUGUUUUUUAUAAAAAAGAAAAAAUGCUUGACAGCAGUGAAGAUCACAGUUA
env-1442  ..............................UUUUUUAUAAAAAAGAAAAAAUGCUUGACAGCAGUGAAGAUCACAGUUA
env-1443  UGCUCGCCGUGCUUAAUUCAAUGCGCGUUUUAAAAAAAUAAUGAAAAAAUAAUCUUGACAUUUUGUAAAAUAAAUGUUA
env-1444  AUGAUGCUUGCGGUGCUCAAUUCAAUGCGCGUUUUGAAAAAAUAAUCUUGACUUUUUGCAGAAUAGAUGAUAGAAUAAU
env-1445  AUGAUGCUCGCCGUGCUCAAUUCAAUGCGCGUUUUGAAAAAAUAAUCUUGACUUUUUGCAGAAUAGAUGAUAAAAUAAU
env-1446  GCUCAAAGCGGAGUUGACGGAUCGGGUCAGAAAAAACUGAUUUUGCAUUGACAUUUGAAAAAUAUAGUGCUAUAAUAAU
env-1447  ..........AUACGAGCCAAAUCAGCUUGUUUGAUGAAAACGCCGCCGAAAAAGAUUGAUGUUGACAUUUGUGCGGGA
env-1448  ..........AUAUGAGCCAAAUCAGCUUGUUUGAUGAAAACGCCGCCGAAAAAGAUUGAUGUUGACAUUUGUGCGGGA
env-1449  ..............UUUAAUGCCGUACGGUAAAUUUUAAAUAAUAAGUUAUUGACUUUUUGCAUCUGAUAAGCUAUAAU
env-1450  UGCCGGUAACUGCGUUUAAUGCCGUACGGUAAAUUUUAAAUAAUAAGUUAUUGACUUUUUGCAUCUGAUAAGCUAUAAU
env-1452  UGCCGGUAACUGCGUUUAAUGCCGUACGGUAAAUUUUAAAUAAUAAGUUAUUGACUUUUUGCAUCUGAUAAGCUAUAAU
env-1454  CGCAGCUGUGAAACGAUGCCGUUAUAUGACUUCAAAUAAAAAUCUUAAAAACAGUUGACAUAUCCGUCUCAAUUUUAUA
env-1455  UAUGAUGACGGUUGUAAUCUCUCUAUUUUUAUAGAAAUAAACCAUUGACUUGACCUGAAUUAUCAUAUCAGGUAUAAUU
env-1456  CGAAACAGGGGGGCGGAGCAUGCCCUGCGGGAAAGCUUGCCCCUUGACACGGCCUGUCCGCCGCCUUAUAAUAAGUCCC
env-1457  AAGCGGUUCUGGAAAUACGUCCGUCCGGUGAAAAAAAUCACCCCUUGACGCGGCGGCGCUGCUCGGAUAUAAUAAACAC
env-1458  AAGCGGUUCUGGAAAUAUGUCCGCCCGGUGAAAAAAAUCACCCCUUGACGCGGCGGCGCCGCUCGGGUAUAAUAAACAC
env-1459  AAGCGGUUCUGGAAAUAUGUCCGCCCGGUGAAAAAAAUCGCCCCUUGACGCGGCGGCGCUGCUCGGGUAUAAUAAACAC
env-1460  AAGCGGUUCUGGAAAUACGUCCGCCCGGUGAAAAAAAUCACCCCUUGACGCGGCGGCGCCGUUCGGGUAUAAUAAACAC
env-1461  AAGCGGUUCUGGAAAUACGUCCGCCCGGUGAAAAAAAUCACCCCUUGACGCGGCGGCACCGCUCGGGUAUAAUAAACAC
env-1462  ..............................................CAGGUCCAAUGAAUCACGCCUUUGCCUUACCAG
env-1463  .................................UUUUGCGUAAUGCCGUUUCUUUAAAAAAUUAUCAAUUUGCAAACAA
env-1465  ......................GCGUGUGCCUGUUCAUUUGGAUGGAGAGAAAACGGAAAAGGGAGCGUUAGAGGAGUC
env-1466  UUGUCACUGCGCGGAUAACCCGUACAGCAUCUGUCUCCAUCAUCAUACGCGGAUUGUUCUCUGUAAAAAGCUUUAUAAA
env-1467  UUGUCACUGCGCGGAUAACCCGUACAGCAUCUGUCUCCAUCAUCAUACGCGGAUUGUUCUCUGUAAAAAGCUUUAUAAA

          ...............................................................................
          ...............................................................................
          ...............................................................................
          ...............................................................................
```

```
env-1468  UUGUCACUGCGCGGAUAACCCGUACAGCAUCUGUCUCCAUCAUCAUACGCGGAUUGUUCUCUGUAAAAAGCUUUAUAAA
env-1469  UGACGCUAUAGUCAUAUUCUGUCUGUAAAUUCGGAAGGCUGGGUUCACCCUUGAUCUUUUGCUUUCUAUGUGUAGUAUA
env-1471  GACAAGUGACGGUUUUAUCAGAGGGCUUUGCUAUACUCUUUGUUAUAUGUCCGGUUUCCGAUCCGGCUUUUUAUAGUAU
env-1473  ACGUCCGUGCAGCUUCGUCCGCAUCACGAAAAUGCGAAAAAUUCCGCGCCGUGUUGGUGCGCGAUCGGCGGUAUGUCCG
env-1474  ..................................................................CGGCGGUAUGUCCG
env-1475  CGAGUUGUACGGAAAGAUUAUGGAACAUUGACAGAAGAUCUUUUCUUGACAACCCCCGACCGUUAUGCUAAAAUAACAA
env-1476  UCUUGUUUUUUCAAGGCUCUUUGUGCAGUGAAAAUGAAUGGUGCAAAUAUUGUAAAGCAACUAUCCCUUGUUAUGUCAU
env-1477  CCUCACGGAAUAAGCGCCUUUUCCGAAAAGAAGCGACCGUUUUGAAAGGCGGCCCGGUUCGCCGGUUCCGCAGUGGGAA
env-1478  AUCUUACUUCCGCCAAAUGGGAUCAGGCGUCUCCCGCCAACAAUCGAAUCCCAUUGACAAGCAAAAACAAUUAUGUUAC
env-1479  .......................................CCGAAUAUAAGAAAAAAGUUAAAUACAGGCUUAUACCAUUC
env-1480  AAAAACGCAAAAUAAGUUAAAAAAUCUUAAAUAAUUGCGCAAGUUUUUGUUGCGUUAUAGGUUUUUGUGUGUUACAAUA
env-1481  AAUUCAGAAAAGCUAUGGAACCGGUUUAUUUUCCCAUUUCUCUUGACAAGCUGUGGCCUUCGCGCUAUCAUACAGAAAG
env-1482  CUGCUGGCUGUCGUUACCGUCAUGAUGAUCAAAGAAAACAGUUGAAUUUUCGGGAGAAAUGUGUUAGAUUAUGAUCAUA
env-1483  .............................................UCGUCCGCCCGCACACCGAGAGGCGCGCCUGCCUU
env-1484  ..........................................GAUAAUGGUGCAAAAUAUUUUAUACCUUUUACUUCCAA
env-1485  ACUGCAAUUACCUAUAGAUAAGAAAUAAAUAUUUGAUUAUCUACAAAGCAGCACUUGACUUCUUGCACUUUAUAUGGUA
env-1486  ..............................................GAUUCUGGAAAAAGAAAUUUGAGCAGUUGGGAAU
env-1487  GUUCAUUCUAUCACAGCUCUUAAAUAUUCAUUGCGUUUUGAUAUUGACUUGCCGAUUUUUUUAUAGUAUACUGAUUAAG
env-1488  GUUCAUUCUAUCACAGCUCUUAAAUAUUCACUGCGUUUUGAUAUUGACUUGCCGAUUUUUUUAUAGUAUACUGAUUAAG
env-1490  ................................ACAGCGGAAGAAUGUGCUACGAAGUUUUAACCAGACUUUCCGCAUUUU
env-1491  UUUUGAAUAUGUACGCAAAAACAAGAGAACAUCCGUAAAAAUUUUUCAAACUGUAUUGCAAACUGUUUUUAAUAUGAUA
env-1492  UUUUGAAUAUGUACGCAAAAACAAGAGAACAUCCGUAAAAAUUUUUCAAACUGUAUUGCAAACUGUUUUUAAUAUGAUA
env-1493  UUUUGAAUAUGUACGCAAAAACAAGAGAACAUCCGUAAAAAUUUUUCAAACUGUAUUGCAAACUGUUUUUAAUAUGAUA
env-1494  UUGAAGUGGAAUUUAACUUUUCACUUCAGUUCAAGUCAACAUCAGGCAGAAAUGCUCCAUACAGUAGGUUGUUUGUGUU
env-1495  ........GAAUUUAACUUUUCACUUCAGUUCAAGUCAACAUCAGGCAGAAAUGCUCCAUACAGUAGGUUGUUUGUGUU
env-1496  ...........CUAUUGACGUCACGGAAAUUGAACGCUUAUUGAAAAGAUUUUUUAAUUGAUAAUCUUAAAAUCGGGCU
env-1497  GUUUUAAUGGUAACUUUAGGAAAGGUAUAGUCGAUCGUAUCAUGCCAAAUCCCGAAAUCUCAAUGAUGAAGACUUAGAU
env-1498  .......................................................AAAUCUCGAUGAUGAAGACUUAGAU
env-1499  GUUUUAAUGGUAACUUUCGGAAAGGUAUAGUCGAUCGUAUCAUGCCAAAUCCCGAAAUCUCGAUGAUGAAGACUUAGAU
env-1501  UAUUAUAAAAACCUCUUGACAAAACGGCCCGAACAGUGUAUACUGACACACUCCUUGCCCGGAACAAGAAAUCCGGACA
env-1502  UUAUAAAAACCUCUUGACAAAACGGCCCGAACAGCGUAUACUGACACACACUCCUUGCCCGGAACAAGAAAUCCGGACA
env-1503  GAAAAGACGAUGAAAAGACGGAAAAAGGCGGCAAUAUGGAGAGACAGAUAAAAUAGUUGCGAAAGCAGAGGAAAUAUGC
env-1504  UUCUUUCCUCUAUAAAUCGCGCAUUUCCCUCUCCUUCCCAUUUUAUUAUUCUACUGUCUCUCUUGCACUUUGAUACUCA
env-1505  GAAUAUGGUAUAAUGUGAAAGGCUUCAAACACAGCUGUGGCCUGAAAGCCCCGAUAUUGUGGUUAUUCAGGUUCAUUCA
env-1506  GUAAUUGCCCAUUUUCAGAAAGAAAAAGAUAAAUCGGAAUUUUCUAUUGAAUUUUGUAUAUGUAAAAGAUAUAAUAAAC
env-1509  GGAAUUGCCCAUUUUCAGAAAGAAAAAGAUAAAUCGGAAUUUUCUAUUGAAUUUUGUAUAUGUAAAAGAUAUAAUAAAC
env-1510  .............................AUACCGUUAAGGAAAAAAUGGAGCUUUUCGGUUCGGUUAACAAGGCAUAAU
env-1511  .............................AUACAGUUAAGGAAAAAAUGGAGCUUUUCGGUUCGGUUAACAAGGCAUAAU
env-1512  .............................AUACCGUUAAGGAAAAAAUGGAGCUUUUCGGUUCGGUUAACAAGGCAUAAU
env-1513  .............................AUACCGUUAAGGAAAAAAUGGAGCUUUUCGGUUCGGUUAACAAGGCAUAAU
env-1514  CAAUGCAGCUAUGUCAUGCUAUAAUUGUUACUAAGAAAAUCGGAAUUUAUAAUAUUGACAUUUUGCUUUGAUUAUGAUA
env-1516  CUACCCUCUUGCCUAUUCGGAUAUACAACCGCCGAUAUGUCAAAAUCUAACUUGCAUUCCGUACUUUUUUAUGUUAUAA
env-1517  CCCUAUUGACAAAUCUGUUUUUAUAGUGUAUAAUAUCAUCGUAAUUUUUGCGGCGGGUAUGCUUCGUUUUGCCGCAAAA
env-1518  UGAUUACGAAAAGCUCUUGACAAAACGGUCGGAACAGUGUAUACUUACUUAAACUUUUUGCUGCGAACAAGAGGUUCAA
env-1520  AGAAAAAAACCAAACCUUUUACCAAACCUUUAAUAAUUUACCAUAGCUGUCUUGACAAAUCCGUCGGAAUGAUGUAUGUU
env-1521  UAAAAAUCAAUUUUAAAAAUGUGAGCCUGCAAAAAGAAUAAAAUUUUGCUUGAUUUUCAGCUUGCGCUGUGCUAUAAUC
env-1523  GCAAGCACUGCUAAUUCAUUAAGGUCCGCUAUUUCCAUUAUGCUCUUCUUUGUUCUUGACUUUCACGCAGAAUUCUUGU
env-1524  UUUCGUUUUUUUCACAUGUUCCUCUUGCAUUUUCGGGGGACGUGUGUUAUAAUAUGGCACGGUGACGUUGCCACCGAAC
env-1525  AGAGGAUAAAGCGCAUGGCAGUGGUUGGCAGAAUUUAUAUGAAAUUGAAAGCGUAAACGUUUCCGAAUUUCGGAAAAUA
env-1529  CUCGGAAAAAGCAAGCGCUGCGCGGCAUAAUAACGGUUUUUGCCCGCAGAUGCUUGCAUUUGCCGGAGCAGGGUGAUAU
env-1530  UAAUAAGUACAAGUUAAACGCAAUUUAUGUGUGACUUAUUAAAUUAUAGAGAAAAGCGUUGCUUUUAUUAAGAUUAUGU
env-1532  CACGACCUAUGUGUUGCGGCGUUGUUUUAUUUGCUCACGAGAGUUAAUUACGGCUUGACAUACUCGGCGGCAAAUGCUA
env-1535  CGGCAAACCCGCCGAGAACCGUACCGGGAGGGGAAUCCCGGGAAGCUAUACGGCUCUUGACGAUGUUGCGGCCGGGUGA
env-1536  GUCUCAAAACCCGACAAGUCCACCUCUUUGCAUGAAAUCUGCAUCCGGCGGUGCAGAGGUUUAUAAAGAGGUAAAAAUC
env-1537  ...........................................................GUUUAUAAAGAGGUAAAAAUC
env-1538  GGUUUCGGGACUUCAUAAACCGAAUCAAAUUACCUCUUUACACCAAAUUUACAGCAAUCGGUGCAGAGGCGAAAAAAUC
env-1539  AAAAACAUACAAAUAAAUAACGGAAAGCUAACGCUUUCCUCGAAAGGUUGAAUCUAUCUGCGACCGCUUGCGGUCUUGA
env-1540  ...........................................................................AAAAA
env-1541  ..............................................GUAAAGAAGCAAUUGACCUGGUUAAAAUGAAAAA
env-1542  ..............................................GUAAAGAAGCAAUUGAACUGGUUAAAAUGAAAAA
env-1543  ..............................................GUAAAGAAGCAAUUGAACUGGUUAAAAUGAAAAA
env-1544  ..............................................GUAAAGAAGCAAUUGACCUGGUUAAAAUGAAAAA
env-1545  ...............................................UAAAGAAGCAAUUGAACUGGUUAAAAUGAAAAA
env-1546  ..............................................GUAAAGAAGCAAUUGACCUGGUUAAAAUGAAAAA
env-1547  ..............................................GUAAAGAAGCAAUUGAACUGGUUAAAAUGAAAAA
env-1553  ..............................................GUAAAGAAGCAAUUGACCUGGUUAAAAUGAAAAA
env-1602  ..............................................GUAAAGAAGCAAUUGACCUGGUUAAAAUGAAAAA
env-1603  ..............................................GUAAAGAAGCAAUUGACCUGGUUAAAAUGAAAAA
env-1604  ..............................................GUAAAGAAGCAAUUGAUCUGGUUAAAAUGAAAAA
env-1607  ..............................................GUAAAGAAGCAAUUGAUCUGGUUAAAAUGAAAAA
env-1608  ..............................................GUAAAGAAGCAAUUGACCUGGUUAAAAUGAAAAA
env-1682  ..............................................GUAAAGAAGCAAUUGAACUGGUUAAAAUGAAAAA
env-1689  ..............................................GUAAAGAAGCAAUUGACCUGGUUAAAAUGAAAAA
env-1690  ...........AUAUGAGGAAGCAGGAAGAAAUAAUCAGUAUAUUUGAAGCAAUAAAGUAAGAGUGAUACUAUGAAAAA
env-1691  ..............................................GUAAAGAAGCAAUUGACCUGGUUAAAAUGAAAAA
env-1692  ........................................UGACUGGUAAAGAAGCAAUUGAACUGGUUAAAAUGAAAAA
env-1693  ..............................................GUAAAGAAGCAAUUGAUCUGGUUAAAAUGAAAAA
env-1695  ..............................................GUAAAGAAGCAAUUGACCUGGUUAAAAUGAAAAA
env-1696  ..............................................GUAAAGAAGCAAUUGAUCUGGUUAAAAUGAAAAA
env-1706  ..............................................GUAAAGAAGCAAUUGAACUGGUUAAAAUGAAAAA
env-1707  ..............................................GUAAAGAAGCAAUUGAUCUGGUUAAAAUGAAAAA
env-1708  ..............................................GUAAAGAAGCAAUUGACCUGGUUAAAAUGAAAAA

          ................................................................................
          ................................................................................
          ................................................................................
          ................................................................................
```

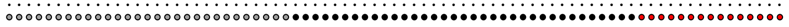

```
env-1711   .............................................GUAAAGAAGCAAUUGAACUGGUUAAAAUGAAAAA
env-1712   AACAUUAAGAAGAGCAUAAAAAAUGCGAUUAUUUGAACGUUUUAUUGCUUUUUGUUACAUCAUAGAUUAUAAUAACCAA
env-1713   AACAUUAAGAAGAGCAUAAAAAAUGCGAUUAUUUGAACGUUUUAUUGCUUUUUGUUACAUCAUAGAUUAUAAUAACCAA
Rfl-2-1    UAUCAAGAGUGUUUACGCAGAGCAGUAAAAAGCCCUUUAACGCACAGAAAUAAUUGACUUUUUCUUCUUUCUCUGCUAU
env-1715   GAAAUGCGCAGCAUAAUGUGGAUUUUCCCCAGUGCAAUGCGCUGUUACACAGAUUGACUUUUUAAGGUAUCUGUGCUAU
env-1717   GAAAUGCGCAGCAUAAUGUGGAUUUUCCCCAGUGCAAUGCGCUGUUACACAGAUUGACUUUUUAAGGUAUCUGUGCUAU
env-1718   .....................................................................UCUGUGCUAU
env-1719   UAAUCUUGUAUUUUAACAAAAGAGCAACAAGAGUUUGUUCCAGAGGGAUUGCCUUUUUUCUGAUUUUAUGUUAUAGAUA
env-1720   UUGGGAAUGGAGUUUCUCACCGCACAAUAAAGCAAAAUUUAGCGGAGCCUAUUGAUUUUUGGGGUUUUCCGGGUUAUAC
Oru-1-1    UUGGGAAUGGAGUUUCUCACCGCACAAUAAAGCAAAAUUUAGCGGAGCCUAUUGAUUUUUGGGGUUUUCCGGGUUAUAC
env-1721   UCUGUGUUGUUUUCCUUCUUUCUGUCCUAUGCAAAUACUCUUGCCGGAAAAAGCAAUUGCCAUUUUGCGCCUUUCGUGU
env-1722   AAUAGCCUGUAAUAAAUUUAUUCUUAAACCGAAAUGCCGUUAAUAUUUUUUUAUUGCUUUUUAACUCAUAAUAUGUUAC
env-1723   UCGUCGCCGUUUAUACGAAAGCGCCGCACUGCACACAAUUAAAAAUUACGGCUUGCAUUUUAUCGGCGGGUCUGAUAUA
env-1724   UCGUCGCCGUUUAUACGAAAGCGCCGCACUGCACACAAUUAAAAAUUACGGCUUGCAUUUUAUCGGCGGGUCUGAUAUA
env-1725   UCGUCGCCGUUUAUACGAAAGCGCCGCACUGCACACAAUUAAAAAUUACGGCUUGCAUUUUAUCGGCGGGUCUGAUAUA
env-1726   GUCAAUUUAAUUGCUCCUUUCAUUUUUAUACCAAAAACGUUUUUACUUGAAAACACUUGAUUUUCUGAGAUGAUUAUGU
env-1727   CAGGGAAAUUUCUUGAAUACAUCAAAACGUAUAAGGUAUUGCCAUAAGUCACAUAUUUGACAUUUUGAGCUGAAUGUGA
env-1728   GAUCGGCUUUUAUACGGUCUGUGGGAAUAGUUGGUAGUAUCUCCAUUUAAAUAUUGACUUUUUUAAUUUUUUGUGCUAU
Ral-1-1    UGGCAGAGAACUAAAAACAGCUACCCAUUAUGAUUUGCCACUUACGCCAUGUAUUGACUUUUUUAAGCCUUCGUGCUAC
env-1729   UCUCUAACUAUUCCCAACUGACAAUAAGUUUACUCUAUCGAUUUUUGAAAAUCUGUUGCUUUUUUCUUUUGUUUAUAAU
Gni-1-1    GAACGAUAAAUUUAUUUUUCCGAAAGAAAAUUGACACUAUCUUUUUAUAAAAAUAUUUAACAUUUAUUUAUAUUGUAUA
env-1730   AAGUAACUGUCAAAUAAUAAAAAAAUUCGUAGAAAAAACUUGCAAAGUAUUGACUUUAAAUUUUGAAUAUGGUAUAAUA
env-1731   GUUGGUUUCUUCAUAUAUUUUUGAAUAUCGGCUUCGGAUUAUGGUGCUUGAAAAAGUCACAAUUGUAUGAUAUUAUACU
env-1732   UGAUUGGCGAAUAAUGUAAAAUUUCGCAGAAAAAAAAACUUGCAAAGUAUUGAUUUUGAAUUUGGGAUGUGGUAUAGUUA
env-1740   AAGAGGGAAUUGAAUUUACAAGGUUGAGAAACAGUUGACAAUUUAUUUGCUGUUUGGUAAAAUAUUGAACAGCAGAUAA
env-1741   AAGAGGGAAUUGAAUUUACAAGGUUGAGAAACAGUUGACAAUUUAUUUGCUGUUUGGUAAAAUAUUGAACAAUAGAUAA
env-1742   AAGAGGGAAUUGAAUUUACAAGGUUGAGAAACAGUUGACAACUUAUCUGCUGUUUGGUAAAAUAUUGAACAAUAGAUAA
env-1743   UAAGCAUUAUAAAAACAAAGAACCACGUUGUGUAAAAGUUAGUUGAUGAUUACAUAAAUUUAUGAUAAACUGUUAAACA
env-1744   GCCGUUUGUCAAGAAAAUUUUACAUAAAUCGUGAAAGCUGACACUUAAAAGCUUGACAAAAAGCAAGAUUUGUGAUAUA
env-1745   GUUUUGUCUUCAAUUAUGUCUUAUGUGUUUUACAAAAAGUAUGAUUAAAAUUAAUUUAAUUGACUUUGUUCUCGUUAU
env-1746   CGUCAUACCACAGAAGAGUUCACAUGAACAUCUCUUAUUACGGGAGAACACUUCCGCGGCAGUUGACAAGGCGGAUCAA
env-1747   AAUACGAUUGCCGUUGGCAUUUUCGAGGAUAAGUUUGAAAAACAUGGUUGCACCCCCUUUACUUUUUCAAUGCUGUAUG
env-1748   ..............................................GUUGCACCCCCUUUACUUUUUCAAUGCUGUAUG
env-1749   AUCAAUCCCAUUUGAUAUUAGCUAUUAAAUUUUACUUUGUUAUUUUUAAAACUUGACACUGAAAAAAAAUUAUGUUAUU
env-1750   AAUAGAAAAGGGUGCGCUCGAUACAAUCAAGAAAAAUAUAUAGCUAUCUUGAAUUAAAUAAAUUUUUAUGUUAUAAUUA
env-1751   ...........................................................AGUUGUUAUAAUGCUUUAGA
env-1752   ..UGGCUGCGGAGUAUCCCGCAUAUUUCAACAGUUUUUCUUACAUGCUUUGAGAAGAUUCUCUGCGGCAGGGCUGCCGC
env-1753   ..UGGCUGCGGAGUAUCCCGCAUAUUUCAACAGUUUUUCUUACAUGCUUUGAGAAGAUUCUCUGCGGCAGGGCUGCCGC
env-1754   UGAUACCACGCAAGCCGAUGGCAAGCAAGCACCAACCGAAGCUGUUUUUUCUUGACUUAUCAAAUCAAUCAUGUUAUUA
env-1756   .............................................................................AA
env-1757   CAGGUUUUUGUGCUUUUUCACGAAUUGGAUAAAAUCAACAAUUGACUUGACAAAGUCUGACGAUUAUGUUAUAAUAAAU
env-1759   UUAAUUACGCCGAAAUAAAGCGAAAGUAAUUAUGAGACCUACAUAACCUUGACAUACCAUGCAAAGCGUGCUACUAUCU
env-1761   AAGAGUAGCAGGUGCGUAAAACGCCGGAGCAAUAAGAGCGUAAAGCGUUUUCUUGACAAACUGCUUUAUAAGUGAUAAU
env-1762   GAAGGUACGUAUUCAUUAUAUCACAAACCGCGGCGCGGCGCAACAAAAAUACUUGACAGUCCGCGCGGGGUAUGCUAUA
env-1763   GAAGGUAUAUAUCCAUUAUAUCACAAACCGCGGUGCGGCGCAACAAAAAUACUUGACAGUCCGCGCGGGGUAUGCUAUA
env-1764   GAAAGCUCCUGAAGGCGUGCUGCCGCACUCCGGGGAGGCCGACGCCGUGCAUAGCUGAUUCCCCUCGGAAGUUUUUUAG
env-1765   CGCGCCAAAAUUCUGGAAGCAUAAGCCCGCGCCGGUUCCCCCUUGACUUUUCCCGCUUCCUCGGAUAUAAUACGGUCAC
env-1766   CGCGCCAAAAUUCUGGAAGCAUAAGCCCGCGCCGGUUCCCCCUUGACUUUUCCCGCUUCCUCGGAUAUAAUACGGUCAC
env-1768   ACAAAAAGUAAAUGCUGUUACCCUGAUUAUGCAUAAAAAAAAGGCUUGACAUUGAGGGAAAGGAGUGGUAUAAUGAGGAA
env-1769   AAUUUCCUCUAUCAAGGAGUAGAACACUCUUGCAAAAUUGAAAUUUUUCCAUUAAAAUAACCGCAUAUUUGUAUGUGAA
env-1770   CAUCAAAUUGGGGAAUUGCAGAGGAACGCUCUUGCCAAAUCAAAUCUUUUCCAGUAAAAUAACCACAUAUUUGUGUGAA
env-1771   ACAGGUUUUUAUAAAAUCAAAAGAAUUCUGCAUACAUUAUAUUGACCUCUAUCUAUAGUUCUGCUACGCUAAAUUCUGU
env-1772   UUCCCGAUAAAUAAAUUAUAUCACAAACCGCCGCGCUGCGCAAUAAAGCACUUAACAAACUGCGCCCCUUGUGAUACAA
env-1773   AAAAUAUCUGUAGUUGUAUAAACAUUUUUCGUUGCAGACGUGUAUUGCAAUUUUGUGACGGAUUAUGAUAUACUAUAUC
env-1774   ...............................................................................
env-1775   GCACACCUGAGCGGAAAAAGGUAAAAAUAUACAGCCGGGGCGGGGAUUGACUUUCCGCCGCCGGCUGUGCUAUACUUGG
env-1776   GGAGUUUUUAAGCGGUAAAAACAAAACGAAUGACACACAGCUGGGGAUUGACUUUAAUCCCCGGCUGUGCUAUGCUCGG
env-1778   UAAACUGUAUGAAAAGUACCGUUUUAUCCUAUAAUUUAAAAUAGCCUGCCGCUUGACUUUUUUGUUAUUUCAUAUUAUA
env-1779   .........................CUUAGGAGACAGGCGGUUUUUUCUCUUGACAUAACAUGAUUUAGCCAUUACAAU
env-1780   UCAAGAGCGGCGGAGUGCGGAUACUCCGUUUGACAUUUUAGGAAGAAUAUGCUAUACUGAAUAAGCAGUCACAUUAAUG
Rsp-3-1    UGUGCUAUAAUAUCAGUAUCCGAUUUCGGACUAAGGAGCAAAUAACAUGGAUAACAAAGAAUAGGCGGUCAAAAAACUG
env-1781   GAUAGGUGUUUUUAUGUUGGUUCUUGCAUAAAUGAAAAAAUUAUGUAUAAUAGAAAGAAGUUAUAAAAUUUAUUUUAAA
env-1789   CGAUAGAUUUUUUAUGUUGGUUCUUGCAAAAAUAAAAAAAUUAUGUAUAAUAUAAAGAAGUUAUAAAAUUUGUUUUGAA
env-1792   ..................................ACGUACGUGAUGAAUUAGGGCUUGAUCAAUUUAGUUUUGAUUUUG
env-1794   .......UUGACAGCAGGAUAUUUUUCAAUAUUGUUGACAUCGGCGGAAAUAAGUGAUAUUAUAUAACAGUAAGCUUAC
Rsp-2-1    ACGUAUGUCUGCUGUAAAGACGUUUUGUGCCAUGUUGUUAUUUCCUUUCACUUGACAUUUUACAUAAAAUAUGAUAUAU
env-1796   .......UCCGCAGUAAAGACCUUUUGUACCAUAUCUCUUUUCCUUUCCCUUGACAUUUUUUAUAAAAUGUGAUAUAUU
env-1800   .......UCCGCAGUAAAUACCUUUUGUACCAUAUCUCUUUUCCUUUCCCUUGACAUUUUUUAUAAAAUGUGAUAUAUU
env-1801   .......UCCGCAGUAAAGACCUUUUGUACCAUAUCUCUUUUCCUUUCCCUUGACAUUUUUUAUAAAAUGUGAUAUAUU
env-1802   ACGUAUAUCCGCAGUAAAGACCUUUUGUACCAUAUCUCUUUUCCUUUCCCUUGACAUUUUUUAUAAAAUGUGAUAUAUU
env-1803   UCUGAUAUCCACCGUUAAGACACUGUGUGCCAUUUUAAUAUCUCCCUUUCGCUUGACAUUUCAGAUAAAAUGUGAUAUG
env-1804   UUUUAAGAUUGCAGUACUGCAAAUUUCACUCAUUUUUUGUUUUUUACUUGACAUUUUUCGCUUUUUAUGCUAUACUCCC
env-1805   AGAUUGCAGUACUGCAAAUUUCGCUNNNNNNNNNNNNNNNNNNNNNNNNNNNNNNNNNNNNNNNNNNNNNNNNAUACUCCC
env-1806   ...........................................UCAUCUGCUUCGGCGUGUUCAGCCUGAUUGGAGCGG
env-1807   ...............................AGAAAAACAAUCCCGGUCCGGUUGACAGACGACGGUUUUGGGGUUAAA
env-1808   CAUAUAGUCAGUAUUAUAUAUUUUUUUAAGAUCAGAUUUUCGGGCUGCCACGAAAAAAACAGUUGCAUUUUUUCUGGUU
env-1809   CAUAUAGCCAGUAUUAUAUAUUUUUUUAAGAUCAGAUUUUCGGGCCACCACGAAAAAAACAGUUGCAUUUUUUCUGGUU
env-1810   CAUAUAGCCAGUAUUAUAUAUUUUUUUAAGAUCAGAUUUUUGGGCUGCCAUCAAAAAAACUGUUGCAUUUUUUCUGGUU
env-1811   UUUGAUAUAUCCAAUAUCGUACAUCUUUUUAAUAUCAGUUUCCUCGUCCAUGAAAAAAACAGUUGCAUUUUUUCUUUCU
env-1812   UUUGCGUCAAUAUGAUAUGGCGAUAUUUUCGGCGGACAGAUCAUUUUUAUAUAAAUUUAUUAUUGAUUUUUGUCUCUUG
env-1813   UCGUAAUCAGCAGGUCGUGUGUUCAAGUCACAUCACCAGCUCCAUUCAGGGCAUUUGCGAAAGUGGACGCCCUGUAUAA
env-1814   UCGUAAUCAGCAGGUCGUGUGUUCAAGUCACAUCACCAGCUCCAUUCAGGGCAUUUGCGAAAGUGGACGCCCUGUAUAA

           ...............................................................................
           ...............................................................................
           ...............................................................................
           ...............................................................................
```

env-1815	UCUGCAGGGACUUUACGGCAAAGAGACGCACGGUAUAAGAAAUGUGUUGACUUUUUGGAUAUUAUACGGUAUACUUAUG
env-1816	AUAUACUUACAUACUUAUAAAAUUAACAAUUUAUUAUUUCAGCUAUUGACUUUUUAAUUUUAUUGUGAUAUACUAAUCG
env-1818	CACAGAAACCAAGUAACAUGACAAAUUUUCUACUGACUAUUGUAUUUCCUGCUUGACAAACGAUAAACGUCAUGUUAUA
env-1819	CACAGAAACCAAGUAACAUGACAAAUUUUCUACUGACUAUUGUAUUUCCUGCUUGACAGACGAUAAACGUCAUGUUAUA
env-1820	CUUUUUAAAGUCAUUGUAACAAAAUUUUACCACAUUUUCAAUUUUGAAAAUAUUGACUUCUCACUUUUUUCGCAGUAUA
env-1821	CCAAAAGCUGUCGAUUAUCUCUCAGCUUUUUAUAAAGCAGUUGAGUUAACCGUAUUGCCAAACCAAAAAAAUCGUGCUA
env-1822	AUUACUAUAAUGUGUUUUAUUUUUUCUUCUUCCAAAUGUUUACAAAGUUAUAUUUGACUUCAAAAAUAAAGCGUGCUAU
env-1823	GAAGGAUAUUGGAUGAGAUAAUGGCUAUAAAUCUUAUGCUGGCUAUUGACUUUUUCGCUUUGAGGCAUUAUACUUGCUG
env-1824	UUAUGCAGCACGGCCGAAUAGUAAGAAUAAUUAUUUUUCUUGCAAAGUGUUGACUUUUCUCUCUCACCGCGCUAUAAUC
env-1825	UGAUACAAGAAAAAUGAUUCUAAAACUGCAAACUUCCUAUAUUGACUUUGCUUUAGAUACAUGAUAACAUAAAUAAUUA
env-1829	UGAUACAAGAAAAAUGAUUCUAAAACUGCAAACUUCCUAUAUUGCCUUUGCUUUAGAUACAUGAUAACAUAAAUAAUUA
Dsp-1-1	....CCA
env-1830	......................................AAUCGAGAUUGACUUCUGUCCUUUUCUAUGCUAUACUUGGCA
Lba-3-1	CCGUCAAGUAGAAUGUUUUUGAGAGAAGGGAAAAUGCAAAAAUUAUUGACUUCUUUGUUUUACUGUGCUAUACUUACUG
env-1831	GGGAAUACAGAAACCGUUCGUUCGAAAAUACAGAGCAGGCAAUUUGAAGAGAGCGAUUGACUUUGCCCGCUAUAUGUGC
env-1832	.....CCUCGGUCGACGAUGUACUCGUUAUAACGUUCUGCCAAAACCUUGAAUAGUGGGAAAUUAGGGAUUUCUUUAGA
env-1837	.....CCUCGGUCGACGAUGUACUCGUUAUAACGUUCUGCCAAAACCUUGAAUAGUGGGAAAUUAGGGAUUUCUUUAGA
env-1838	AUCCUGCGGGUCUCUGUUGCUUUCCGGACAUUUUGAAACAUCCUCUUAUUGACUUUAUCUUGUAAAUGUGAUAUCAUAG
env-1839	CGGAAGUGCCAUUUCUGAUGAAAACGACGCAUCGGAUAAUAUCGAAGAACUAUUGACUUUAUUUUAGAGAUGGGUUAAA
env-1840	GUCUCUUUUUUACGUUCUAUAAAUCCAUUCAGCAAUUUUAGGUAACUAUUGACUUGCNUAUGAAGAUAUGAUAUUUUAU
env-1842	CAAACAAUGUUACAUUUCAAGAUUAAAACAAGGAUUUAAUAUUUUUUCUUGACAUUUCCCGUUUUCUGUGCUAUAAUAC
env-1843	....CAGUUCAGGUUUUCUACGGAAUAUAUCUUGUUUAC
env-1844	CCAUAUGUUUGACAACUGCAGAUUUUCGUAAAACAAAUACACCAAAUGCACUUGAAAUCUAAAGGCUUUUAUGCUAUAC
env-1845	UAUUUAAAAUCGAUAGGUCUUUAAUAAAAAAUCAAAUUUUUGCGGAAAUUGCUUGACAUUUUCUUGAUUUUUUAUAAAA
env-1846	AGGAAAAGAAAGGAAAUUUGACAGCUCUCUCUUUUUAUGAUAUGCUUUUUGAAAGAAUAUGCCAUGAGAGGCACUUCGU
env-1848	GUUAUAAUCACUUUGCAAGUUUCUUAUUGAAACAGAGCGGUUGUUAAUUCUGUAUUGUUAACGCAAGAUAAAAAUUUAU
env-1849	UUUAUUGUAGUUUAUGUAAAGCUAAAAAGUAAAAAAAUAAUGCAAAAAUCACUUGAAUUUCAAAUAAUUUUAUGCUAUAA
env-1850	CAGAUAUAAAAACGCCGCAACCGUCCGGAAGCGUGCGUAAAACGCUUGACUUCGCGGCGCACAUAUGUUAUAAUACGCA
env-1851	UUAUUAUCCGGAUAAUAAAUUUUACAAACAACUUUAUGCAAAUCAUUGACUUUUUUGAUUUGACGUGAUAUACUUGAUA
env-1852	AUAGUUUAUUCAAAUUUUAAACUAUUCUUCAUACUUUUUUAACAGUCUUGUUGACACAAAAAAAUUACUGUGGUAUAAU
env-1853	AUAGUUUAUUCAAAUUUUAAACUAUUCUUCAUACUUUUUUAACAGUCUUGUUGACGCAAAAAAAUUACUGUGGUAUAAU
env-1857	AUAGUUUAUUCAAAUUUUAAACUAUUCUUCAUACUUUUUUAACAGUCUUGUUGACGCAAAAAAAUUACUGUGGUAUAAU
env-1858	AUAGUUUAUUCAAAUUUUAAACUAUUCUUCAUACUUUUUUAACAGUCUUGUUGACACAAAAAAAUUACUGUGGUAUAAU
env-1859	.................................ACUUUUUUAACAGUCUUGUUGACACAAAAAAAUUACUGUGGUAUAAU
env-1860	....................................UGAAAGAUGCAGUAGGCAAUGUCAGCAGGAUGACUGUUAAGGUU
env-1861	GAACAUUGUUACAUCAAUUUGGGCUGUUGCCCUUGCGGCUUUGCCGCUCACUGUGCUUCGCACAGCAAAAUUGUGUUAU
env-1862	GGAAACCCGGAGAUAGACUCCAUGAUUAGGACUUGCUUUUAUAAAAAAACAUGCUAUAAUAGGCCCAUUGCUCCAACAU
env-1864	ACAAGGGAUAUACAGUGUAGCACCGCUCCGGAGCCAAGGGCAGCCAAGGCUCCGGAUAUCCCUUGACAUUUCAGCUAUG
env-1865	GCAAAGGGAUUCACUGUAUAAUUCUGCCCCGGAGCUGCUGAAAAUGCGGCUCCGGAUCUCCCUUGACAUUUUUCACGGA
env-1866	GCAAAGGGAUUCACUGUAUAAUUCUGCCCCGGAGCUGCUGAAAAUGCGGCUCCGGAUCUCCCUUGACAUUUUUCACGGA
env-1869	GCAAAGGGAUUCACUGUAUAAUUCUGCCCCGGAGCUGCUGAAAAUGCGGCUCCGGAUCUCCCUUGACAUUUUUCACGGA
env-1870	ACGUUUCUCCCGAAUCCGUACCGCUUUUCGUGUGAGUCGCCGUUUUCGCAUAUAUCGCCGGAAUAUUUCGGAAUCAUUA
env-1871	UAAUUAUUAAAAGGGGAAUUUAAUUUCCCCUUUUUCAUGUUUUGUUUGAACAAUCUUAUAUAAUUUGCUAUAAUAUCAU
Ssu-6-1	UUAGUCAUAGAUAGUUAUUUAGCUGUGUUUUUUCAAAUUUUGGAUAGUCCUUGACUUAUUUAAAGCAUUUUUAUACAAU
Ssu-1-3	AUUUUCUUGUGGCUAAUCAAGUUAUGUUUCCCCAUAUUCUGAGAUAAUGUUUGACAUUCUCUCUGGGAUUUUAUACAAU
Ssu-1-4	CUUUAUCAUGUGGCUAAACUCAAUGUACUACCUUAUGUUUGAGGUGAUAAUUGACAUUCUCUCUGGGAUUUUAUACAAU
Ssu-1-7	ACUGUAUGAUGGGUAACCUCAGGAUAGAUUUCCUAAUUUUGAGAUAAUAUUUGACAUUCUUUUAGUGAUUCUUUACAAU
Ssu-1-8	ACUGUAUGAUGGGUAACCUCAGGAUAGAUUUCCUAAUUUUGAGAUAAUAUUUGACAUUCUUUUAGUGAUUCUUUACAAU
env-1872	AGGUAUAGUGAAAUAAGAUCCUGUCCUUGACUUAUUCCCCGAUCUAUUGUAUCCUAAUAGGGAAUCGGUUUUAGCAUCC
env-1873	UUUCCGGGCAUUUUUCAUGGCAGAGCGUGGCUGUAGGCGAAUUGCCCUUGACAUCCGCGUACGAUUUGUGUAUGCUUAU
env-1875	UCUUAGACUGGUAAGCAAAUAAAUUUUAUGUAAAAAUUACAUUUUUUAUAAAUAAUAUUUUGUGAUAUAAUCAUAAAAA
env-1876	UAAAAAUCAAAUGAAAAUAUAACUUUUUCUUUUUUGACUUGACACAAGUUUAUACUUGUGAUAUAAAUAGUUUUAUUAA
env-1877	UAAAAAUCAAAUGAAAAUAUAACUUUUUCUUUUUUAUCUUGACACAAGUUUAUACUUGUGAUAUAAAUAGUUUUAUUAA
env-1878	UGACAUGAUAGAAGGGGCUGAAUCUGUAAAAUGAUAGCUUCAAAUUGACUUUUGUAUGAUCUAAUGAUAUAUUAAUGAA
env-1879	UAGAGAAGCAUUAUUAUUAUACCUUUUUACGGACAGAAUGCAAAGAAUCUUGACUUUUUAUCCUAUCUGUGAUAUUUUG
env-1880	UAGAGAAGCAUUAUUAUUAUACCUUUUUACGGACAGAAUGCAAAGAAUCUUGACUUUUUAUCCUAUCUGUGAUAUUUUG
env-1881	UACAGUAAAGGGGCUGGGAAUCCUUUAAAACAUAUCUUGACUUUGGAGCGGCUCUGUGUUAAAUUCCUAGUUGUUUGAU
env-1882	GCUUACCGUAAAGGGACUGGGAAUUCUCUAAAACUUUUCUUGACUUUGCAGAGGGCCUGUGAUAAAUUUCUAGUUGUUA
env-1884	AAAGGCCGAUUUGUUGCACUUUGUCCCUCACUAUAUGAAAAUUCGAAAAUUAUAUUGACUUACCAAGCGAUUUGUGAUA
env-1885	GGUACUCUCGUGUAUGGCAGCACGCUGGAUUUGAGGAAAAAUUUUAAAAAUCCCCCUUGACAAUUCCCCCGCUUUGUGG
env-1886	UAUAUAGGACAUGGGGGUGCGGAAGUCUUUUUGAUGCUUAUUCGCGAAUUUUGAUGUUGACUAUUCCGUCGUUUUAUGA
env-1887	GUCGUUUGCAAAUUGAGAAAAUGGUUUUUUCUUUCUGUAUUAGUGAAAUUUUUAGGUUGACUUUUAUAUCGCUUUAUGA
env-1888	GUCGUUUGCAAAUUGAGAAAAUGGUUUUUUCUUUCUGUAUUAGUGAAAUUUUUAGGUUGACUUUUAUAUCGCUUUAUGA
env-1889	....GUGUUGACUUUUACAUUGUUUUAUG
env-1890	CCCGUUUGAAAAGCGGUUUUGCCAACCAUUUUAAUCCUUAUUAGCAAAAUUUUUGUGUUGACUUUUACAUUGUUUUAUG
env-1891	ACGGAAUAUGCAGCAGUUUUGCCAAAGUUUUUUUAUCUUUAUUAUAAAAUUUUUGUGUUGACUUUUACAUAAUUUUAUA
env-1892	GGCAAUCCGAUGCACACAACGGAUUUUUCCGAACAAAAAGUCUUGACACAUGCACAAAUCGAUGAUAGAAUGAAAGAAA
env-1893	......ACGACGUUAAUUCCAUUGAACCGUUUCAUAAGACAAAAGUUCAAAAAUAAAUUUGACAUUCAAAUUUAUUCAU
env-1894	......ACGACGUUAAUUCCAUUGAACCGUUUCAUAAGACAAAAGUUCAAAAAUAAAUUUGACAUUCAAAUUUAUUCAU
env-1895	.................................AUAAGACAAAAGUUCAAAAAUAAAUUUGUACAUUCAAAUUUAUUCAU
env-1896	....AUAAAGUUGUAACAGACGGGCUCUGGACGCCCGAAGCG
env-1897	.....GACAAUUCUUCGAGCAUACGAUGCCGCGCUUCGAGGAGCUGGCGCGCCACAUCCGACGUUCACGCGCAGAGGCU
env-1898	CAAACUUAAAAGGCGUUUUAUAUAAUCAAACAAAAGAUUGCUUCAAUUUACCAAAAGCAACAGAAAAACUUAAUAAUAC
env-1899	CGGCUGAAAACGGGCGGUUUGUUGCAGUUAUUCAUAAACAAAAUAUCGAAUCCUAUAACCUUGACUUUUUGAGAUGGAU
env-1900	AUUCCAGACAAAAAAUGAAAAAAGCAUGCCGUGCUCAUUUUUGAUUUUUUCUUGAUAUUUCAAAACAGUUAUGUUAUAC
env-1901	CCUAGAGGGGUUAGUAAAGUGCUCACAGCGCAAAGGGCAAAAGCAUAUAUUGACUUUUAGCGCUAAGUGUGCUAUCCUA
env-1902	AGAGAUUUCUGUUUCCCGUCACUUCACUCCAUUCAACAGAGCAUUUUCUCCUCUUGCGAAAUUCAAACCCAUAUGGUAA
env-1903	UUGUCAAGCUCUCUUUUUUGUUCUUUUUUCAUUUGCUUACUUGUUGAUUUAUCUAUAAAGAUGUGAUAAGAUUUAGAAA
env-1904	GAACUUUUAUUUUUGUAUAAAGACAAGUCACCUAGAUGACAUUGCCUUUUUUCAAAGGUUAUGCUACAAUUUCUGGUAA
Par-1-1	UUACAGAGCGAGGCAAAGAUUCUGCUUAUUAUUGCAGUAAGUAGUCCAAAGAAUAAAGCAAAUGUGAUAAUAUUGAAUC
env-1905	....GGGCGUUAAGGCUUGACAAUUACAAAAAUUAAAUU
env-1906	...UGUUGGGAAGCGGAGAUAAGGCCGAAAUUAUUUGGGAUAGCGGGCGUUAAGGCUUGACAAUUACAAAAAUUAAAUU
	....
	....
	....
	....

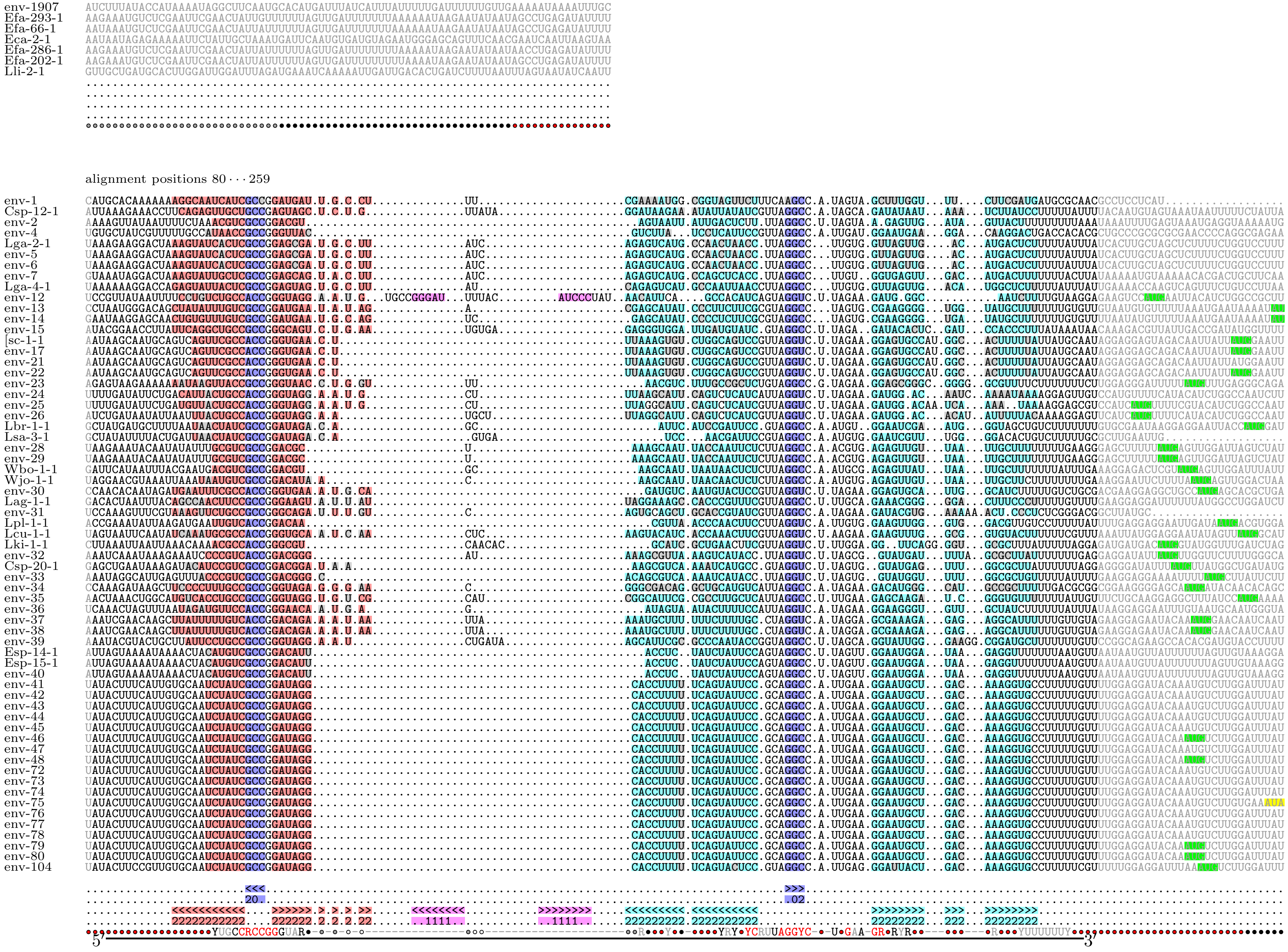
env-1907
Efa-293-1
Efa-66-1
Eca-2-1
Efa-286-1
Efa-202-1
Lli-2-1
alignment positions 80 · · · 259
env-1
Csp-12-1
env-2
env-4
Lga-2-1
env-5
env-6
env-7
Lga-4-1
env-12
env-13
env-14
env-15
[sc-1-1
env-17
env-21
env-22
env-23
env-24
env-25
env-26
Lbr-1-1
Lsa-3-1
env-28
env-29
Wbo-1-1
Wjo-1-1
env-30
Lag-1-1
env-31
Lpl-1-1
Lcu-1-1
Lki-1-1
env-32
Csp-20-1
env-33
env-34
env-35
env-36
env-37
env-38
env-39
Esp-14-1
Esp-15-1
env-40
env-41
env-42
env-43
env-44
env-45
env-46
env-47
env-48
env-72
env-73
env-74
env-75
env-76
env-77
env-78
env-79
env-80
env-104
5'
3'

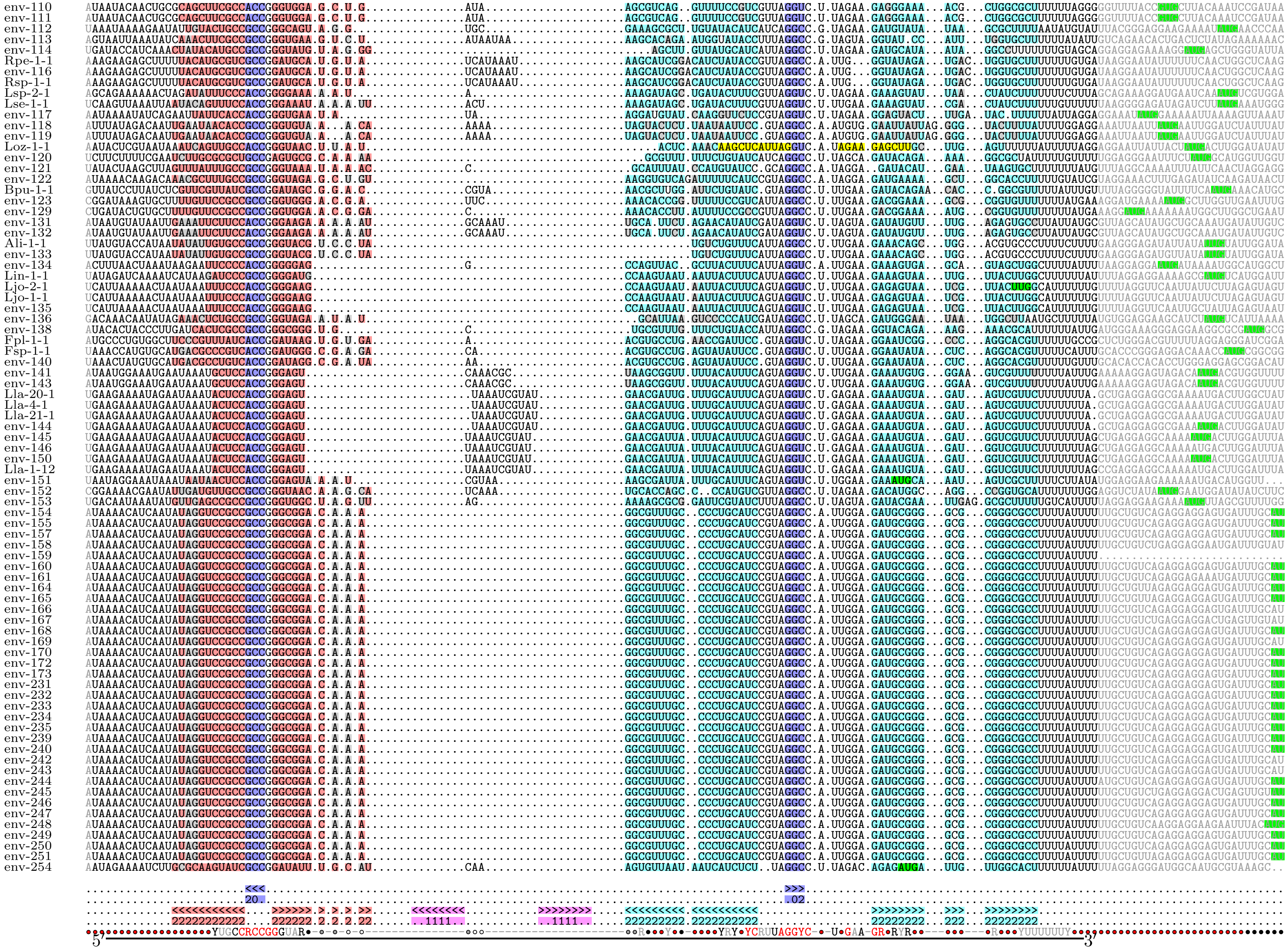
env-110
env-111
env-112
env-113
env-114
Rpe-1-1
env-116
Rsp-1-1
Lsp-2-1
Lse-1-1
env-117
env-118
env-119
Loz-1-1
env-120
env-121
env-122
Bpu-1-1
env-123
env-129
env-131
env-132
Ali-1-1
env-133
env-134
Lin-1-1
Ljo-2-1
Ljo-1-1
env-135
env-136
env-138
Fpl-1-1
Fsp-1-1
env-140
env-141
env-143
Lla-20-1
Lla-4-1
Lla-21-1
env-144
env-145
env-146
env-150
Lla-1-12
env-151
env-152
env-153
env-154
env-155
env-157
env-158
env-159
env-160
env-161
env-164
env-165
env-166
env-167
env-168
env-169
env-170
env-172
env-173
env-231
env-232
env-233
env-234
env-235
env-239
env-240
env-242
env-243
env-244
env-245
env-246
env-247
env-248
env-249
env-250
env-251
env-254
5'
3'

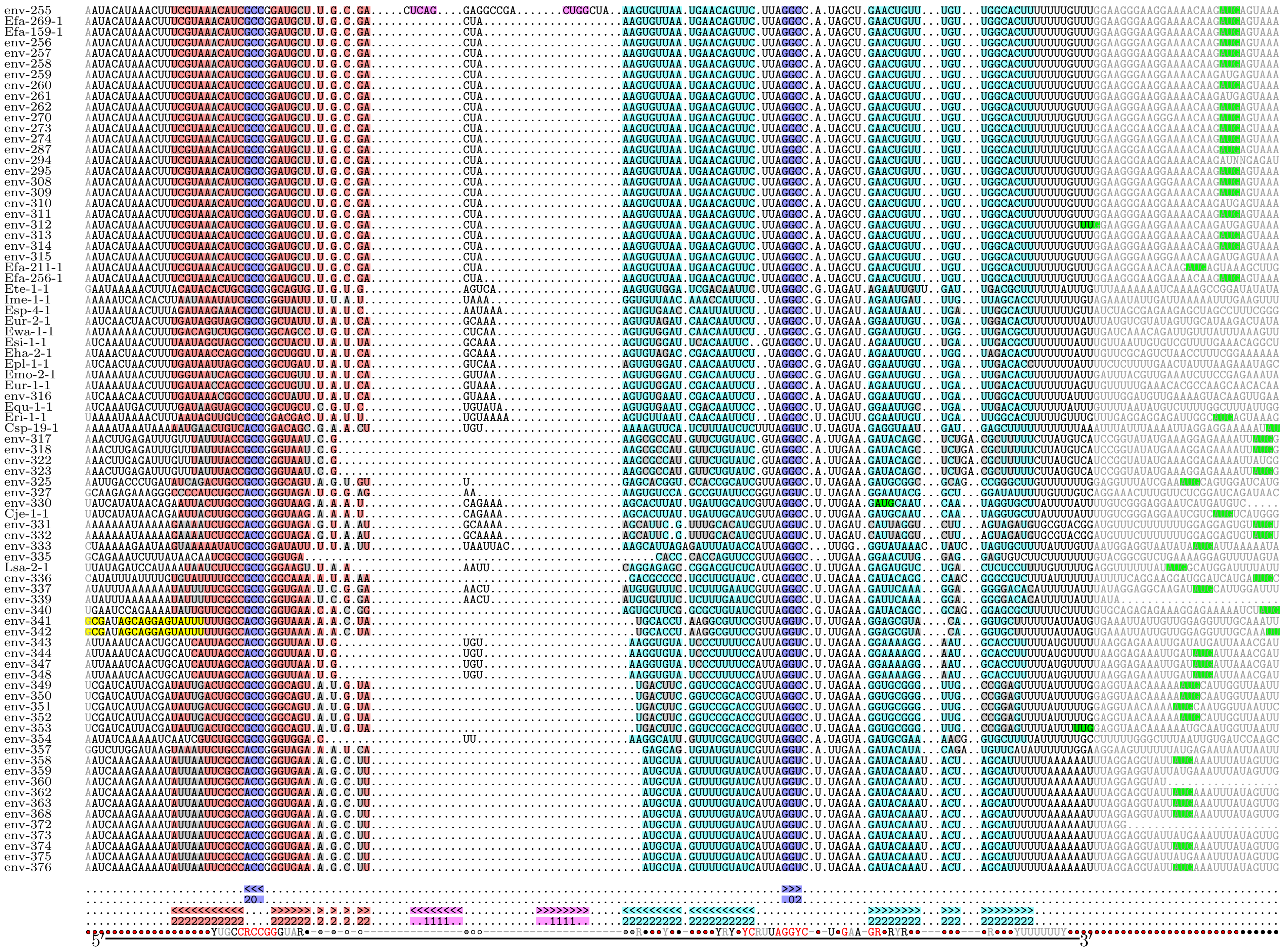
env-255
Efa-269-1
Efa-159-1
env-256
env-257
env-258
env-259
env-260
env-261
env-262
env-270
env-273
env-274
env-287
env-294
env-295
env-308
env-309
env-310
env-311
env-312
env-313
env-314
env-315
Efa-211-1
Efa-256-1
Ete-1-1
Ime-1-1
Esp-4-1
Eur-2-1
Ewa-1-1
Esi-1-1
Eha-2-1
Epl-1-1
Emo-2-1
Eur-1-1
env-316
Equ-1-1
Eri-1-1
Csp-19-1
env-317
env-318
env-322
env-323
env-325
env-327
env-330
Cje-1-1
env-331
env-332
env-333
env-335
Lsa-2-1
env-336
env-337
env-339
env-340
env-341
env-342
env-343
env-344
env-347
env-348
env-349
env-350
env-351
env-352
env-353
env-354
env-357
env-358
env-359
env-360
env-362
env-363
env-368
env-372
env-373
env-374
env-375
env-376
5'
3'

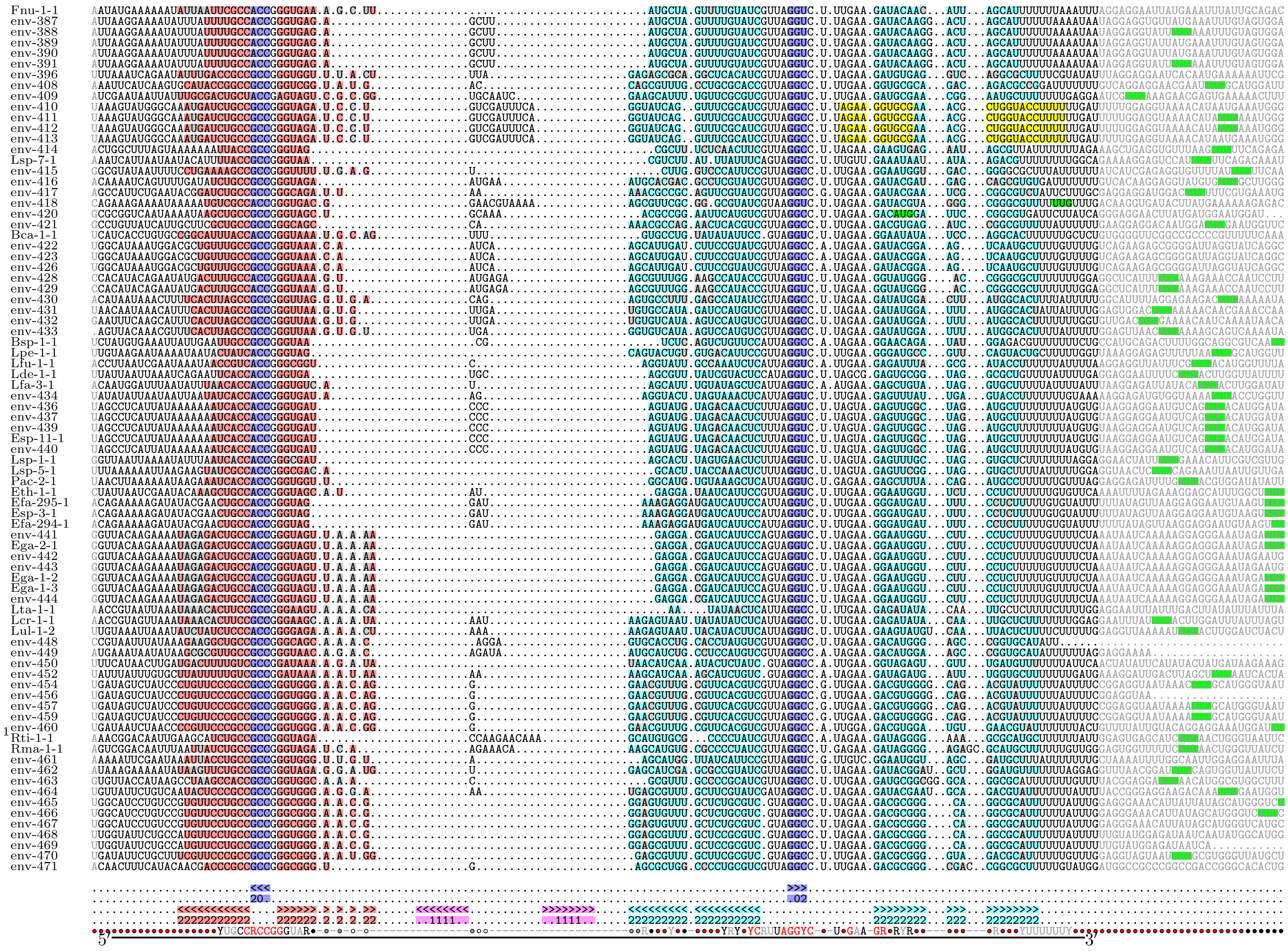
Fnu-1-1
env-387
env-388
env-389
env-390
env-391
env-396
env-408
env-409
env-410
env-411
env-412
env-413
env-414
Lsp-7-1
env-415
env-416
env-417
env-418
env-420
env-421
Bca-1-1
env-422
env-423
env-426
env-428
env-429
env-430
env-431
env-432
env-433
Bsp-1-1
Lpe-1-1
Lfu-1-1
Lde-1-1
Lfa-3-1
env-434
env-436
env-437
env-439
Esp-11-1
env-440
Lsp-1-1
Lsp-5-1
Pac-2-1
Eth-1-1
Efa-295-1
Esp-3-1
Efa-294-1
env-441
Ega-2-1
env-442
env-443
Ega-1-2
Ega-1-3
env-444
Lta-1-1
Lcr-1-1
Lul-1-2
env-448
env-449
env-450
env-452
env-454
env-456
env-457
env-459
env-460
Rti-1-1
Rma-1-1
env-461
env-462
env-463
env-464
env-465
env-466
env-467
env-468
env-469
env-470
env-471
5'
3'

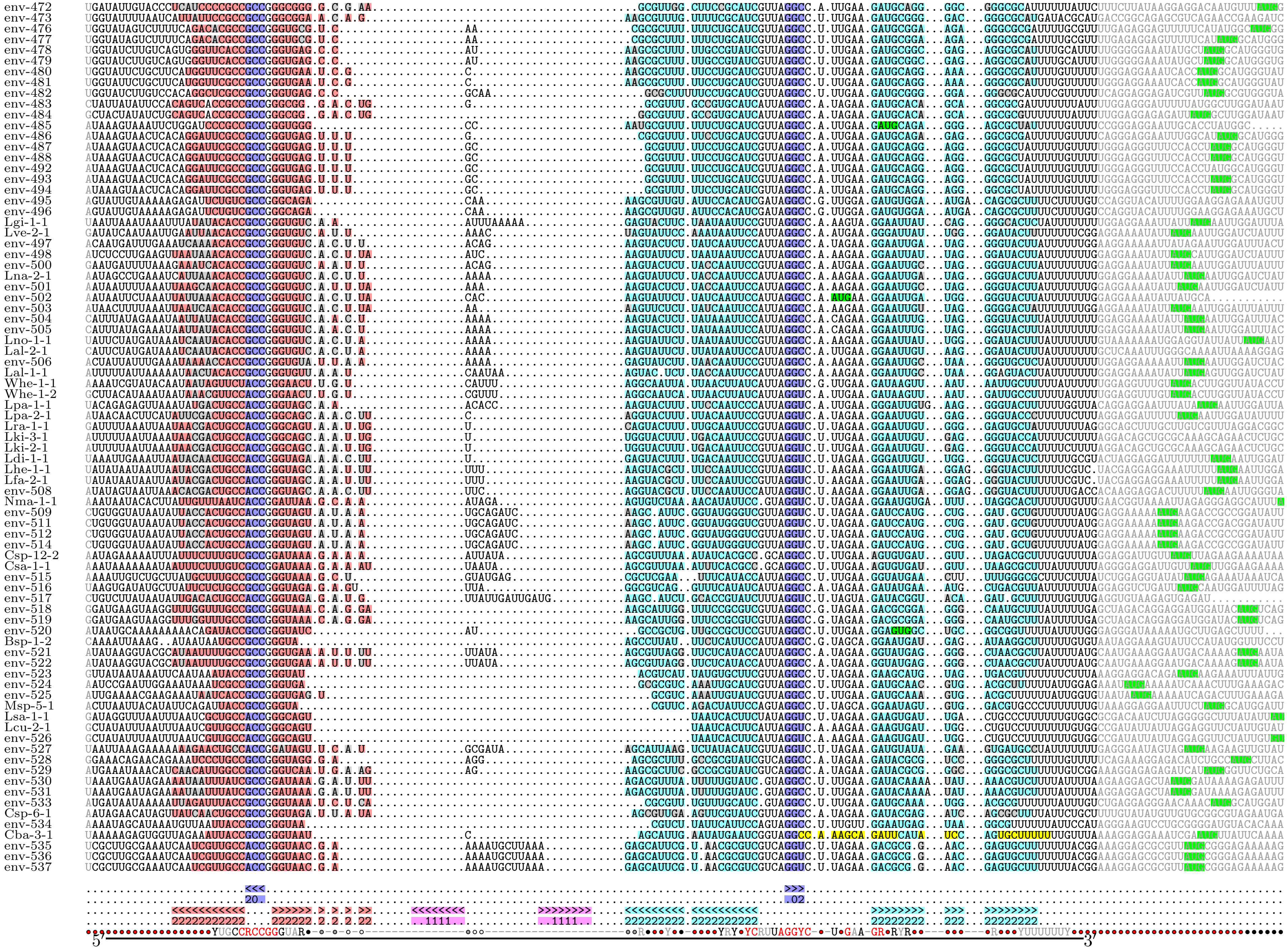
env-472
env-473
env-476
env-477
env-478
env-479
env-480
env-481
env-482
env-483
env-484
env-485
env-486
env-487
env-488
env-492
env-493
env-494
env-495
env-496
Lgi-1-1
Lve-2-1
env-497
env-498
env-500
Lna-2-1
env-501
env-502
env-503
env-504
env-505
Lno-1-1
Lal-2-1
env-506
Lal-1-1
Whe-1-1
Whe-1-2
Lpa-1-1
Lpa-2-1
Lra-1-1
Lki-3-1
Lki-2-1
Ldi-1-1
Lhe-1-1
Lfa-2-1
env-508
Nma-1-1
env-509
env-511
env-512
env-514
Csp-12-2
Csa-1-1
env-515
env-516
env-517
env-518
env-519
env-520
Bsp-1-2
env-521
env-522
env-523
env-524
env-525
Msp-5-1
Lsa-1-1
Lcu-2-1
env-526
env-527
env-528
env-529
env-530
env-531
env-533
Csp-6-1
env-534
Cba-3-1
env-535
env-536
env-537
5'
3'

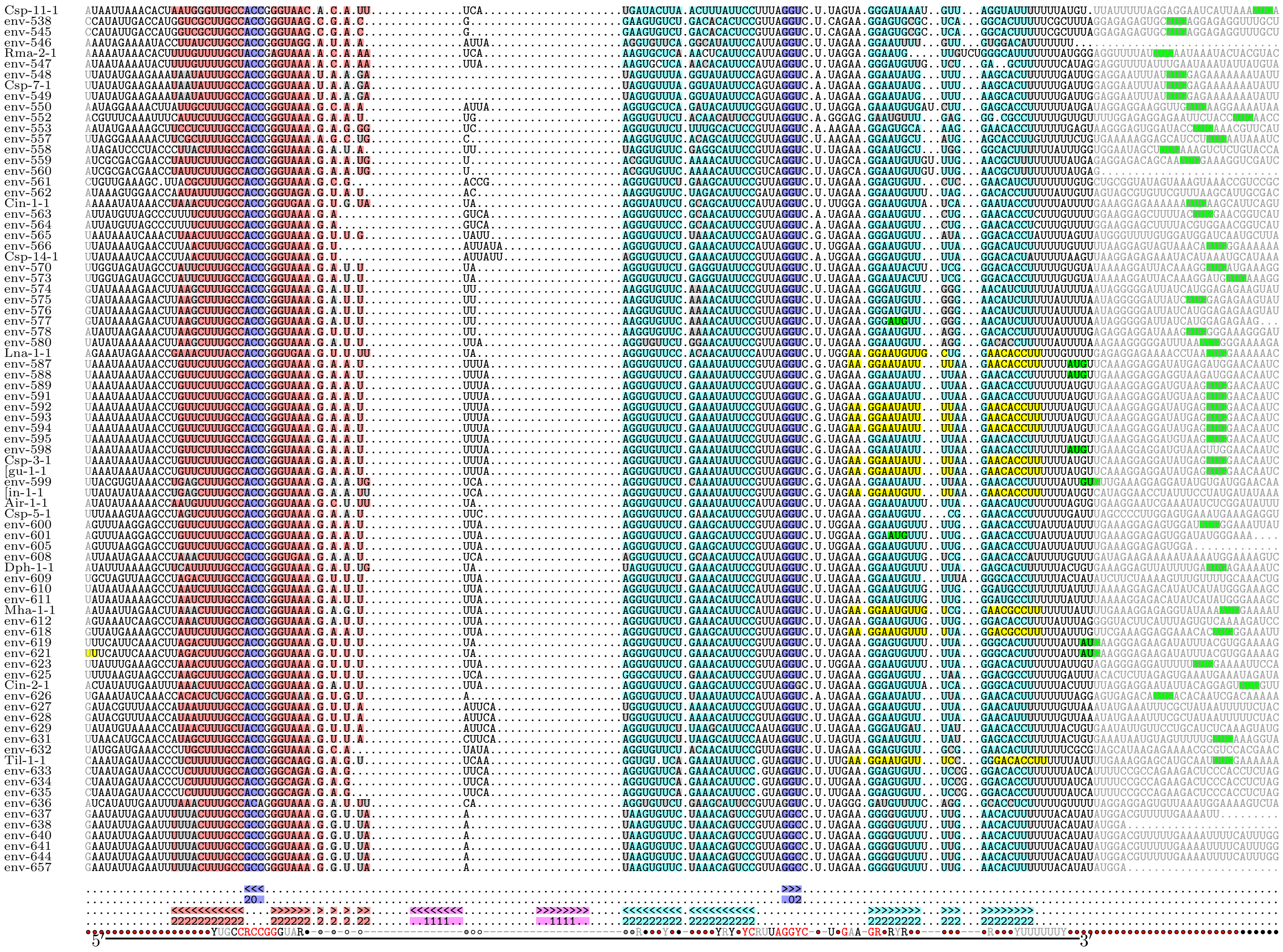
Csp-11-1
env-538
env-545
env-546
Rma-2-1
env-547
env-548
Csp-7-1
env-549
env-550
env-552
env-553
env-557
env-558
env-559
env-560
env-561
env-562
Cin-1-1
env-563
env-564
env-565
env-566
Csp-14-1
env-570
env-573
env-574
env-575
env-576
env-577
env-578
env-580
Lna-1-1
env-587
env-588
env-589
env-591
env-592
env-593
env-594
env-595
env-598
Csp-3-1
[gu-1-1
env-599
[in-1-1
Air-1-1
Csp-5-1
env-600
env-601
env-605
env-608
Dph-1-1
env-609
env-610
env-611
Mha-1-1
env-612
env-618
env-619
env-621
env-623
env-625
Cin-2-1
env-626
env-627
env-628
env-629
env-631
env-632
Til-1-1
env-633
env-634
env-635
env-636
env-637
env-638
env-640
env-641
env-644
env-657
5'
3'

```
env-658   GAAUAUUAGAAUUUUUACUUUGCCGCCGGGUAAA.G.G.U.UA..............A.........................UAAGUGUUC.UAAACAGUCCGUUAGGCC.U.UAGAA.GGGGUGUUU..UUG...AACACUUUUUUACAUAUAUGGACGUUUUUGAAAAUUUUCAUUUGG
env-659   AAAAAAAGAAUAAUUUUAUUUGCCACCGGGUAAA.U.A...................UUU.......................AAGGUAUCA.CGGGUAUUUCGUUAGGUC.U.UAGAA.GAAAUACU...UUU...GAUACCUUUUUAUUUUAAAUUAAUUAUGAAGGCAGGGAUUUUAAA
Tma-1-1   ACAUAGCUUGCACAUAUGCUUGCCACCGGGUAAG.C.G.U.A...............AAAUAA....................AAAGUGCCG.AAAGUACUUCGUUAGGUC.G.UAGAA.GAAGUGCU...UCU...GGCACUUUUUUUGUUUAGGAGGAAAUCAUGAACAAAAUAAAAUUU
Nfe-1-1   UAAAUAUAUAGCACUAUACUUGCCACCGGGUAAG.U.A.U.A...............A.........................AAAGUGCCA.AAAGUACUUCGUUAGGUC.U.UAGAA.GAAGUGGU...UUU...GGCACUUUUUUUCGUUUAGGAGGAAGCCAUGAAAAAAAUAAAAUU
Cfo-1-1   AAUUUAAUUGCACAUAUACUUGCCACCGGGUAAG.U.A.U.A...............A.........................AAAGUGUCA.AGAGUACUUCAUUAGGUC.U.UAGAA.GAAGUACU...UUU...GGCACUUUUUUAUUUAGGAGGAGGGGAUUCAUGAAAAAAUUAAGA
Npe-1-1   AAUUUUUUCGCACAUAUGCUUGCCACCGGGUAAG.U.A.U.A.........................................AAAGUGUCA.AGAGUACUUCGUUAGGUG.U.UAGAA.GAAGUGCU...UUU...GACACUUUUUUGUCAGAAUCCUGUUAGAAUCCUCUGAUAUAAUAU
Gfe-1-1   AAUAUUUUCGCACGUAUGCUUGCCACCGGGUAAG.U.A.U.A.........................................AAAGUGUCA.AGAGUACUUCAUUAGGUC.U.UAGAA.GAAGUACU...UUU...GGCACUUUUUUUUUUAUUAAGGAGGAAUCAUGAAAAAAUUUAAAUU
Psp-1-1   UAAAUUAAAGCAUGUAUGCUUGCCACCGGGUAAG.C.A.U.UU.........................................AAGUGCCA.UAAGUACUUCGUUAGGUC.A.GAGAA.GAAGUGCU...UUU...GGCACUUUUUUUUAAUUAAAGGGGAAUUUCGGAGGAAUCAUGAGA
env-660   AUAUUACAUUCACAGAUAUUUGCCACCGGGUAAA.U.A.U.C...............AUC.........................GUGUCA.AAAGUACUUCGUUAGGUC.U.UAGAA.GAGGUGCU...UUU...GGCAUUUUUGCGUUUUUACGGCUUUACAAAAAUAAGGUGAUCAGG
env-663   CAAUAGCAUAAGCAUCUGUCUGCCACCGGGUAGA.C.G.G.A...............A.........................AAAUGCCUG.AAUACAUUCCGGUAGGUC.A.UGGAA.GGAAUGUA...UUU...GGGCAUUUUUUAUUUUAUUGCAUAUUAGAAAAUACAAGGAGAUCA
env-664   AAUAAGUAAACACAGCUGCCUGCCACCGGGUAUG.C.A.G.C...............GCUU......................AUGUGCCUG.AACACAUUCCGUUAGGUC.A.AAGAA.GGAAUGUG...UCC...GGGCUUUUUUUCGGGAAAAUCCAGCUGGCGGCACUGGAUUGUGUU
Cba-1-1   AAUAAGUAAACACAGCUGCCUGCCACCGGGUAUG.C.A.G.C...............GCUU......................AUGUGCCUG.AACACAUUCCGUUAGGUC.A.AAGAA.GGAAUGUG...UCC...GGGCUUUUUUUCGGGAAAAUCCAGCCGGCGGCACUGGAUUGUGUU
Cba-6-1   AAUAAGUAAACACAGCUGCCUGCCACCGGGUAUG.C.A.G.C...............GCUU......................AUGUGCCUG.AACACAUUCCGUUAGGUC.A.AAGAA.GGAAUGUG...UCC...GGGCUUUUUUUCGGGAAAAUCCAGCCGGCGGCACUGAAUCGUGUU
env-665   AAAAUAUAAACACAAUUAUUUGCCACCGGGUAAA.U.A.A.UU........................................CAGUGUCUG.AACACACUCCGUUAGGUC.U.AAGCA.GGAGUGGG...UUC...AGGCACUUUUUAUAUCUCCCGGCAGUUGCGCACUCGCGCGAAAAU
env-667   AAACAAAAAAACACCGUUAUUUGCCACCGGGUAAA.U.A.A.U..............C.........................AAGUGUCUG.AACAUACUCCGUUAGGUC.A.AAGAA.GGAGCGUG...UUC...AGGCACUUUUUUGCUUAACGGUAUCAGGGAGGAGUAAGAAAAUGA
env-668   AAACAAAAAAACACCGUUAUUUGCCACCGGGUAAA.U.A.A.U..............C.........................AAGUGUCUG.AACAUACUCCGUUAGGUC.A.AAGAA.GGAGCGUG...UUC...AGGCACUUUUUUGCUUAACGGAAUGAUAGGCCUGUCCUGCUACAU
env-669   AAACAAAAAAACACCGUUAUUUGCCACCGGGUAAA.U.A.A.U..............C.........................AAGUGUCUG.AACAUACUCCGUUAGGUC.A.AAGAA.GGAGCGUG...UUC...AGGCACUUUUUUGCUUAACGGUAUCAGGGAGG............
env-670   AAACAAAAAAACACCGUUAUUUGCCACCGGGUAAA.U.A.A.U..............C.........................AAGUGUCUG.AACAUACUCCGUUAGGUC.A.AAGAA.GGAGCGUG...UUC...AGGCACUUUUUUGCUUAACGGUAUCAGGGAGGAGUAAGAAAAUGA
Csy-2-1   AAACAAAAAAACACCGUUAUUUGCCACCGGGUAAA.U.A.A.U..............C.........................AAGUGUCUG.AACAUACUCCGUUAGGUC.A.AAGAA.GGAGCGUG...UUC...AGGCACUUUUUUGCUUAACGGUAUCAGGGAGGAGUAAGAAAAUGA
env-672   AAACAAAAAAACACCGUUAUUUGCCACCGGGUAAA.U.A.A.U..............C.........................AAGUGUCUG.AACAUACUCCGUUAGGUC.A.AAGAA.GGAGCGUG...UUC...AGGCACUUUUUUGCUUAACGGUAUCAGGGAGGAGUAAGAAAAUGA
env-674   AAACAAAAAAACACCGUUAUUUGCCACCGGGUAAA.U.A.A.U..............C.........................AAGUGUCUG.AACAUACUCCGUUAGGUC.A.AAGAA.GGAGCGUG...UUC...AGGCACUUUUUUGCUUAACGGUAUCAGGGAGGAGUAAGAAAAUGA
env-675   AAACAAAAAAACACCGUUAUUUGCCACCGGGUAAA.U.A.A.U..............C.........................AAGUGUCUG.AACAUACUCCGUUAGGUC.A.AAGAA.GGAGCGUG...UUC...AGGCACUUUUUUGCUUAACGGUAUCAGGGAGGAGUAAGAAAAUGA
env-679   AAACAAAAAAACACCGUUAUUUGCCACCGGGUAAA.U.A.A.U..............C.........................AAGUGUCUG.AACAUACUCCGUUAGGUC.A.AAGAA.GGAGCGUG...UUC...AGGCACUUUUUUGCUUAACGGUAUCAGGGAGGAGUAAGAAAAUGA
env-680   AAACAAAAAAACACCGUUAUUUGCCACCGGGUAAA.U.A.A.U..............C.........................AAGUGUCUG.AACAUACUCCGUUAGGUC.A.AAGAA.GGAGCGUG...UUC...AGGCACUUUUUUGCUUAACGGUAUCAGGGAGGAGUAAGAAAAUGA
env-683   AAACAAAAAAACACCGUUAUUUGCCACCGGGUAAA.U.A.A.U..............C.........................AAGUGUCUG.AACAUACUCCGUUAGGUC.A.AAGAA.GGAGCGUG...UUC...AGGCACUUUUUUGCUUAACGGUAUCAGGGAGGAGUAAGAAAAUGA
env-684   AAACAAAAAAACACCGUUAUUUGCCACCGGGUAAA.U.A.A.U..............C.........................AAGUGUCUG.AACAUACUCCGUUAGGUC.A.AAGAA.GGAGCGUG...UUC...AGGCACUUUUUUGCUUAACGGUAUCAGGGAGGAGUAAGAAAAUGA
env-689   GAUGAAAUUUAAUAAAAUGCUGCCACCGGGUAGU.U.G.U..................AAUGCAUAA.................AGCAUUCG..UUUACAUAUCGUUAGGUC.U.UUGAA.GAUAUGUA...UGG...CGGAUGCUUCUUU.................................
env-690   AUAAUUAUUCACGCAGACUUUACCGCCGGGUAAA.G.U.U.U................CACACC....................AGCGUUCAG.AUUUCAUACCAUUAGGCC.A.UUGAA.GGUAUGAU...AUUC..UGGACUUUUUUUAUAAGGAGGAAAUGAAAUGGAUUUGCAGAUUAU
env-691   AUAAUUAUUCACGCAGACUUUACCGCCGGGUAAA.G.U.U.U................CACACC....................AGCGUUCAG.AUUUCAUACCAUUAGGCC.A.UUGAA.GGUAUGAU...AUUC..UGGACUUUUUUUAUAAGGAG..........................
env-692   AAUAAAUAAGCUUUUUUCUUUAUCGCCGGAUAAG.G.A.A.A...............UUAUAA....................AAGCGUUCG.GAUUUCAUACUGUAGGCC.U.UUGAAGGUAUGAAAUA.UCC...GGACGCUUUUUAUUUUUGGGAGGGAAAUAGUAAUGAAACAAAAUA
env-693   UUAAUAUAAUAAAAACAUCUUGCCGCCGGGUAAG.C.A.G.GU..............AC........................AACAUCAUG.AUUUUCUGUCGUUAGGCC.U.UAGAA.GACAGGAG...GUU...UUGAUGUUUUUUUGUUUUUAAAUUGCUGGGGAUAGAUUGAUUAUG
env-694   GCAAAUAAAUAUUAAAAGAUUGCCGCCGGGCAAU.A.A.A.GU..............U.........................AGCAUUCAG.AUAUCAUGUCGUUAGGCC.A.UUGAA.GACAUGAG...GUU...UGAGUGUUUUUUCAUUUAUAGGAGGGUAUUAAAAUGGCUUGGGU
env-695   ACAAAUUAAUAAUAAUAUAUUGCCGCCGGGCAAU.A.A.A.................GCA.......................AGCAUUCAG.AUAUCAUGUCGUUAGGCC.A.AUGAA.GACAUGAG...GUU...UGGGUGUUUUUUCAUUUAUAGGAGGAUAAAAAAUGGCUUGGGUAU
[ae-1-1   ACAAAUAAAUAAUGAUACGUUGCCGCCGGGCAAC.A.A.A.AU..............G.........................AACAUUCAA.UUAUCAUGUCGUUAGGCC.A.UAGAA.GACAUGAG...AUU...UGGGUGUUUUUUUCAUUUAAAGGAGGAUAUUAAAAUGGCAUGGAU
env-696   CGAAGUUAAUAUGUAUCGCUUGCCGCCGGGUAAG.U.A.A.AA..............CA........................GGACGCUAA.GUUUCAUAUCAUUAGGCC.U.UUGAA.GAUAUGAA...A.U...UGCAGCGUUUUUUUAUUUUCAAGCUGUUGCUUUUGCUGUUUGC..
env-697   GCGCGUUUGCGCCGUGAUACUGCCACCGGGCAGG.A.A.U.GU..............UU........................CAGGCAUUC.GUUCCGCAUCGUUAGGUC.U.UUGAA.GAUGCGGAG..GCG...GAUGCCUUUUUUGAAGGAGGAAACGGUUUUUCAUGAAAAAGUCCU
env-698   GCGCGUUUGCGCCGUGAUACUGCCACCGGGCAGG.A.A.U.GU..............UU........................CAGGCAUUC.GUUCCGCAUCGUUAGGUC.U.UUGAA.GAUGCGGAG..GCG...GAUGCCUUUUUUGAAGGAGGAAACGGUUUUUCAUGAAAAAGUCCU
[am-2-1   AUAGUUCACGUAUCAUAAACUGCCGCCGGGUAG........................CAAAACG...................CCACGUCAG.AUUUCAUGUCAUUAGGCC.G.UAGAA.GACAUGAAA..GGC...UGGCGCUUUUUCAAUUCUAAUUUCUUUAAUCAGCAAGGGGAUAGA
env-699   AGAAAAUAAAAUAAAUUGCUUGCCGCCGGGUAAG.C.U...................GAAA......................UAACGCUGU.GUCAUAUAUCGUUAGGCC.U.AUGAA.GAUAUAUG...GUAU..CAGCGUUUUAUUUUGUGUGCAUAACCAAGACGCGUAAUCGCUCGG
env-700   AUUAAAAGCAGUAUAUCAGUUGCCACCGGGUAAC.A.G...................CGUU......................UAACGCAUG.GUUCUGUAUCGUUAGGUC.U.UUGAA.GAUACAGA...AAUC..AGGCGUUUUUCUUUUUGCCUGAAAGCUACUGAAAUAUUCACAGUU
env-701   AUUAAAAGCAGUAUAUCAGUUGCCACCGGGUAAC.A.G...................CGUU......................UAACGCAUG.GUUCUGUAUCGUUAGGUC.U.UUGAA.GAUACAGA...AAUC..AGGCGUUUUUCUUUUUGACUGAAAGCUACUGAAAUAUUCACAGUU
env-702   UUGUUAAAGAAAAUAUUGCUUGCCGCCGGGUAAG.A.A.A.U...............C..........................GCAGCGCA.GAUCCAUUUCGUUAGGCC.U.UAGAA.GAGAUGGA...UAU...CGGCGCUUUUGUUUUAUCAAAAAGCGUUCUGCGCAAGAAACGAAU
env-703   GUGAAUUGUAAAUGAUGACUUGCCGCCGGGUAAG.......................CAUUU.......................GAGCGCA.GAUCUGUAUCGUUAGGCC.U.UAGAA.GAUACAGA...UAC...GGGCGCUUUUGUUUUAUCAGCUCAGUGUUCGGCGCAGGUAUUAAG
env-704   GACGAAAAGAAUAAUUGACUUGCCGCCGGGUAAG.U.A.A.G...............A.........................AGCAGCGCA.GAUCCAUUUCGUUAGGCC.U.UUGAA.GAUUUGGAU..AC....CGGC.GCUUUUAUUAUAUAUAAAAGCUCCCUGUGACAGCAAAGAA
env-705   UAAGAUGAUGCGUAACGCAUUGCCGCCGGGUAAU.G.C.G.GU..............UCUA......................AACGCGCUG.GACUCACGCCGUCAGGCC.U.UAGAA.GGGGUGAG...UCUCGGCGCG.GUUUUUUAUUUUUGCGGAGGGUGUUUGUUUAUGAGGGGAA
env-707   GUAUAAUAAGAAAAAAUUGCUGCCACCGGGUAGU.C.G.U.................GCGU......................AAGAGCACU.UGUUCGCAUCGUUAGGUC.U.UAGAA.GAUGCGCG...CAG...GCGCUCAUUUUUUGCGAAAAGGAGGAUUUCCGGCAGGCGGCAGA
env-708   AAGCACAAGUUAAUAUUAACUGCCACCGGGUAG.....................................................CAUUUG..UUUGUGUAUCGUUAGGUC.A.UUGAA.GAUAUGCA...AUG...CGAAUGCUCUAUUUUUUUGGGAGGUUUAUAUGAAAAAGAAUUUUU
env-709   AUGUGAUAUAAUAUAGUUACUGCCACCGGGUAGA.A.A.U.................GCAG......................AGCAUUUGU.UAAGUACAUCGUUAGGUC.U.UUGAA.GAUGUACU...GAG...CAAGUGCUGUUUUUUUUGGAGGUCAAUCAUGAAACUAAAGCAGCU
env-710   AUGUGAUAUAAUAUAGUUACUGCCACCGGGUAGA.A.A.U.................GCAG......................AGCAUUUGU.UAAGUACAUCGUUAGGUC.U.UUGAA.GAUGUACU...GAG...CAAGUGCUGUUUUUUUUGGAGGUCAAUCAUGAAACUAAAGCAGCU
env-713   UUAUGUUAUACUUUAUUUACUGCCACCGGGUAGA.A.A.U..G..............CAA.......................AGCAUUUGU.UAAGUACAUCGUUAGGUC.U.UUGAA.GAUGUGCU...CAG...CAAGUGCGAUUUUUUUUGGAGUGAUAUUAAUGAACUUAAA.....
env-714   AUAUGUUAUAAUAUCAGUACUGCCACCGGGUAGA.A.A.U.G...............CAA.......................AGCAUUUGU.UAAGUACAUCGUUAGGUC.U.UUGAA.GAUGUGCU...CAG...CAAAUGCUUUUUUUGUGGAGGUCGAUAUAAUGAAACUUAA.....
env-716   AUAUGUUAUAAUAUCAGUACUGCCACCGGGUAGA.A.A.U.G...............CAA.......................AGCAUUUGU.UAAGUACAUCGUUAGGUC.U.UUGAA.GAUGUGCU...CAG...CAAAUGCUUUUUUUGUGGAGAUCGAUAUAAUGAAACUUAA.....
env-717   AAUAGAAAAACAUAAAUAACUGCCACCGGGUAGU.A.G...................CAAUGCUCA.................GGCAUUUUC.CGUUUAUACCGUUAGGUC.U.UUGAA.GGUAUAA....CAG...AAAGUGCUAUUUUUUUUGGAGGCCUGAUGAAAUUUUUGACUUAUA
Bpi-1-1   AUGUAGAAAAUAAUUAAAUUUGUCGCCGGAUAAA.U.U.U.A...............UUUA......................GAACGUUUG.UAUUCAUACC.GUAGGCC.U.UAGAA.AGUAUGAA...CGC...AAACGUUUUUUUAUUAUAGGGUAUUUUUAUGAAUAAUAUACUUC
env-718   UCGUAAAUAAAUAGUGUUUCUGCCACCGGGUAGA.G.A.A.UC..............UU........................GAGCAUUCG.AGUAUGUAUCGUUAGGUC.U.UAGAA.GAUACAUG...GCG...GAUGCUUUUUUAUUGGAGGAUAUAUGAAUAUACUUGAGUCGAUUA
env-719   GAUAAAUAUGCGAUUUAUUUUACCGCCGGGUAAU.A.U.A.AA........................................ACCGCCAGA..CUUUCUAUCGAUAGGCC.U.UUGAA.GAUAGAAA...GAU...CUGGCUGUUUUUAUUUUUGAUAUCACAUUAUCUUUUUGCAAAAGU
env-721   CGUCAAUCCUAUAUUGUUUCUGCCGCCGGGUAGA.A.G.U.GG.........................................AAGGUCAG.AACCUGUAUCGUUAGGCC.A.UAGAA.GAUACAGG...GAC...UGGCGUUUUUUUGUUUCAUUGGAGGAGGAAUACGUAUGAAAAUGG
Exy-1-1   UGAAUAAAACAGACGCUCUUUGCCACCGGGUAAA.G.G.G.AG.........................................AAACGUCA.UUUUCUUAUCGUUAGGUC.U.UUGAA.GAUAAGGA...AAU...GGCGAUUUAUAUUUUUAGGAGGAUAUUUUUAUGCAGUGGAUACUA
env-722   CACAACAGCUUGACGCUCGUUGCCACCGGGUAAC.G.A.G.GG.........................................AGGCGUCA.UUUCUUUAUCGUUAGGUC.U.UAGAA.GAUAAAGA...AAU...GGCGUUAUUUUUGGGAGGGUGUCUAUGACGAAGCAUACCUUAUCA
env-723   AAAUAAUUAAAGACGCCUAUUGCCACCGGGUAAU.A.G.G.................A.........................AAGACGUCA.UUUCUUUAUCGUUAGGUC.U.UAGAA.GAUAAAGA...AGU...GGCGUAUUUUUUUUAGGAGGAAUUAUAUGGAAUGGAUAUGCUUAU
Sru-1-1   GAAUAUAAGAUGACACCUGUUGCCACCGGGUAAU.G.G.G.................A.........................AAAGCGUCA.UUUUCUUAUCGUUAGGUC.A.UGGAA.GAUAAGAA...AAU...GACGCUGUUUUUGUAGGAGGUUAAUUAUGUUUCGGAAGAUGCGAA
env-724   GAAUAUAAGUUGACACCUGUUGCCACCGGGUAAU.A.G.G.AG........................................AAAGCGUCA.UUUUCUUAUCGGUAGGUC.U.UGGCA.GAUAAGAA...AAU...GGCGCUGUUUUUGUAGGAGGCAGAAUGCAGUCCUCUUUAUUGUAU
env-725   UAAUAUGAGUUGACACCUAUUGCCACCGGGUAAU.G.G.G.AG..............A...........................AGCGUCA.UUUUCUUAUCGUUAGGUC.U.UGGCA.GAUAAGAA...GAU...GGCGCUGUUUUUUUUGGGAGGUAAAAUAUGUUUCGGAAGAUGCGGA
env-726   UUCGUUAAAUUAAUAUAUCUUGCCGCCGGGUAAG.A.A.A.................AC........................CGAACGUUG.GAUUCGUAUCAGUAGGCC.U.UUGAA.GAUACGAACU.UCC...AGCGUUUUUUAUUUGGAGGUCGUUAUGAAAAACAAGAGAAGAUUA
env-727   UAAAAAUAUUUGCAAAUAUUUGCCGCCGGGUAAG.....................................................CAUCUUU.UAAAUAUUCCAUUAGGCC.U.UUGAG.GGAAUAUU...CAA...AAGAUGCUUUUUGUAUAUAAGACAAAGGAGAAUAAUUAUGAAUUG
env-728   ACAAAAUGAAUAAAAUAUAAUUCCACCGGGAAUA.A.....................CGCU......................CAAGCGUUU.UAAGCAUUUCAUUAGGUC.U.AAGAA.GAAAUGCU...UAA...CUCACUUGAUUUUUUUUUGAGGAGAAUAAAUGAGUUAUAGCAAGU
Lga-5-1   ACAAAAACAAUAAAAUAUAAUUCCACCGGGAACA.A.....................CGCU......................CAAGCGUUU.UAAGCACUUCAUUAGGUC.A.UAGAA.GAAGUGUU...UGA...AUGACUUGUUUUUUUUAUUAAAGGAGUAGAAAUGAGUUAUAGCAA
env-729   GCCUACAGUUCAAUAAUAUCUGCCGCCGGGCAGG.U.U.U.U...............U...........................ACAUCG.GUUCCGUGUCGUUAGGCC.U.UAGAA.GAUACGGG...GUU...GCCGGUGUAUUUUUUAUUCCAGGAGGCAGAAUAUGGAAUGGAUUA
env-730   AAAGUCAAGAGCCGAUUUCUUGCCGCCGGGUAAG.......................UA........................UAAGCGCUG.GUUUCGUAUCGUUAGGCC...AUGAA.GAUACGAG...ACG...GGCGCUUAUUUUUUUGCCCGCGCCAUAUCGGCUCGGAAAUAGGAG
env-731   UCGUGCUGCAAUAAAAUGCUUGCCGCCGGGUAAG.A.G...................CA........................GAAGCGUUG.AUUUCGUAUCGUUAGGCC.A.UUGAA.GAUACGAA...GGUC..AAUGCUUUUCUUUUUGUCUUUUUCCGGUGCGUAGUGAAAAAGAUG
Esp-13-1  CCGCACUGACAUAAAAUGCUUGCCGCCGGGUAAG.A.G...................CA........................GGAGCGUUG.AUUUCGCAUCGUUAGGCC.G.UUGAA.GAUGCGAAG..GUC...AAUGCUUUUCUUUUGCCUUUUUGCAGAAAAACUUAAAAAUUUCAU
env-732   AGACACAGUUUUUGACUGCUUGCCGCCGGGUAAG.C.G.C.U...............GU..........................GCGUUG.AUUUCGUAUCGUUAGGCC.A.AUGUA.GAUACGAAA..GUC...AGCGCUUUAUUUUUGCUUUAAAUGAGGAGGAAAAUUUAUGUUUCG
env-734   AUACAGAAAAAUAUGUAAAUUGCCGCCGGGUAAG.C.U...................UUC........................GAGCAUUU.AUUUCGUAUCGUUAGGCC.U.UAGAA.GAUGCGAA...AUCA..AAUGCUUUCUUUAUUAUCUGGCUGGCACAAAGAAAGGAGCACACA
env-735   AAUGAAUCGCAAGUUUUGCUUGCCGCCGGGUAAG.C.....................UUU.......................GAAGCAUUG.AUUUCGUAUCGUUAGGCC.G.UUGGA.GAUACGAA...AAUC..AAUGCUUUUUUCUAUCGGCGACACUUUACACAUAUCAUGUGCAAG
env-736   AAUGAAUCGCAAGUUUUGCUUGCCGCCGGGUAAG.C.....................UUU.......................GAAGCAUUG.AUUUCGUAUCGUUAGGCC.G.UUGGA.GAUACGAA...AAUC..AAUGCUUUUUUCUAUCGGCGACACUUUACACAUAUCAUGUGCAAG
env-737   AAUGAAUCGCAAGUUUUGCUUGCCGCCGGGUAAG.C.....................UUU.......................GAAGCAUUG.AUUUCGUAUCGUUAGGCC.G.UUGGA.GAUACGAA...AAUC..AAUGCUUUUUUCUAUCGGCGACACUUUACACAUAUCAUGUGCAAG
env-738   GAAAUAAAUACAGUUUUGCUUGCCGCCGGGUAAG.C.U.G.UA.........................................AAGCAUUG.AUUUCAUAUCGAUAGGCC.G.UUGCA.GAUAUGAA...AGUC..AGUGCUUUAUUUUUUUGCUGUUUUCAGCCCAAAGGCGCAGGCGCU
env-740   AUGAUAAAAAAUAUUCUGCUUGCCGCCGGGUAAG.C.U.A.UA.........................................AGGCAUUG.GUUUCGUAUCGUUAGGCC.G.UUGGA.GAUACGAA...AGC...AAUGCCUUAUGUAUUUAUCGGCUGGGGGGCAGUAUAUAAGGAGG
env-741   AUAGCAGAUAUAUGAUUGUUUGCCGCCGGGUAAA.U.....................UUU........................AAGCAUUG.GUUUCGUAUCGAUAGGCC.A.UUGAA.GAUACGAC...AAUC..AGUGCUUUUCUUUUGUAAAGAUAGAAAAGGUGACAAAAGUCAAUA
Msp-2-1   AUAGCAGAUAUAUGAUUGUUUGCCGCCGGGUAAA.U.....................UUU........................AAGCAUUG.GUUUCGUAUCGAUAGGCC.A.UUGAA.GAUACGAC...AAUC..AGUGCUUUUCUUUUGUAAAGAUAGAAAAGGUGACAAAAGUCAAUA
env-742   AUAGCAGAUAUAUGAUUGUUUGCCGCCGGGUAAA.U.....................UUU........................AAGCAUUG.GUUUCGUAUCGAUAGGCC.A.UUGAA.GAUACGAC...AAUC..AGUGCUUUUCUUUUGUAAAGAUAGAAAAGGUGACAAAAGUCAAUA

          ..........................<<<.............................................................................>>>..............................................................................
          ..........................20..............................................................................02..............................................................................
          ..............<<<<<<<<<<<....>>>>>>.>.>.>.>>......<<<<<<<<..........>>>>>>>>.....<<<<<<<<<.<<<<<<<<<<.................>>>>>>>>...>>>...>>>>>>>>..................................
          ..............22222222222....222222.2.2.2.22........1111............1111.......222222222.2222222222.................22222222...222...22222222..................................
          ••••••••••••••••••YUGCCRCCGGGUAR•-•-•-○-○--------------•○○--------------------○○R•••Y••-••••YRY•YCRUUAGGYC-•-U•GAA-GR•RYR••---•••---•R•••YUUUUUUY••••••••••••••••••••••••••••••
          5'                                                                                                                                                        3'
```

```
Mti-1-1   ACAGAUAUAUCAAUAAUGUUUGCCGCCGGGUAGA.C.U.U.UA....................................AAACAUUG.GUUUCGUAUCGUUAGGCC.A.UAGAA.GAUACGAA...AAUC..AAUGUUUUUAUUUUGUACAAAAGGAGAAACAAAAGAAAAAAAAA
env-743   ACAGAUAUAUCUAUUAUGUUUGCCGCCGGGUAAA.C.U.U.UA....................................AAACAUUG.AUUUCGUAUCGUUAGGCC.A.UAGAA.GAUGCGAA...AGUC..AAUGUUUUUAUUUUUGAUGGAAGGAGAAAUAAAAAAAACAAU
env-744   UUGCCUAAAUUGUUUUUGCUUGCCGCCGGGUGAG.C.A.................UC......................AGAGCAUUG.AUUUCGUAUCGUUAGGCC.A.UGGAA.GAUACGAA...AAUC..AAUGCUUUUCUUUUUGCCGGAUUUUCGGAACAGGAAACUUCAUG
env-745   GCAAUUAAAUUACCUUUACUUGCCGCCGGGUAAG.U.G.................UUCA......................GGCAUUG.AUUUCGUAUCGUUAGGCC.A.UAGAA.GAUACGAAC..AUC..AAUGCUUUUCUUUGCUUAUUUUGCGGAAAACUAUUUUCAGGAGGU
env-746   ACACAUAUUAUAAGGAUGCCUGCCGCCGGGCAGG.C.G.U.......................................AAGCGUUG.GUUUCGUAUCAGUAGGCCGU.UAGAA.GAUGCGAA...AGUC..AAUGCUUUAUUUUUUAUUAACGUACUUUUUAACGAUAGGAGAGAAA
env-747   ACACAUAUUAUAAGGAUGCCUGCCGCCGGGCAGG.C.G.U.......................................AAGCGUUG.GUUUCGUAUCAGUAGGCCGU.UAGAA.GAUGCGAA...AGUC..AAUGCUUUAUUUUUUAUUAAGUACUUUUUAACGAUAGGAGAGAAA
env-748   GAAAAAAUAUAUGGAUAACCUGCCGCCGGGCAGG.....................CA......................AAAGCGUUG.GUUUCGCAUCAGUAGGCCGU.UAGAA.GAUGUGAAA..GUC..AAUGCUUUAUUUUUUAUCAGGCGUUUUUAGGAGGAGAAAAUUUU
Bma-1-1   GAAAAAUUAAAAACAGAAAUUACCGCCGGGUAAA.U.U.................U.......................AAAGCAUUG.GAUUCAUAUC.GUAGGCC.A.UGGAA.GAUAUGAA...UGCC..AAUGCUUUUUUAUGGUAUAGGAGGUCUCUAAAAAUAAAAAAACAUU
env-751   GAAAAAUUAAAAACAGAAAUUACCGCCGGGUAAA.U.U.................U.......................AAAGCAUUG.GAUUCAUAUC.GUAGGCC.A.UGGAA.GAUAUGAA...UGCC..AAUGCUUUUUUAUGGUAUAGGAGGUCUCUAAAAAUAAAAAAACAUU
env-753   UAUACUUACAUCGAUCUGUUUAACGCCGGUUAAG.C.A.G.GU.....................................GCCUUUA.UUUAUGUAUC.GCAGGCC.U.UAGAA.GAUACAUA...AAU...AAGGGCAUUUUUUGUUUUAACCGCAUUUUUAAGGAGGUUUUUAAU
env-754   UAUACUUACGCUGUACUGUUUAACGCCGGUUAAG.C.A.G...............GU.......................GCCUUUA.UUUAUGUAUC.GUAGGCC.U.UAGAA.GAUGCAUA...AAU...AAGGGCAUUUUUUAUUUUAAGGAGGAAAUUGAUUAUGGCAUGGUU
env-755   UAUACUUACGCUGUACUGUUUAACGCCGGUUAAG.C.A.G...............GU.......................GCCUUUA.UUUAUGUAUC.GUAGGCC.U.UAGAA.GAUGCAUA...AAU...AAGGGCAUUUUUUAUUUUAAGGAGGGUGUGAUUAAAGCAUGGUU
Lga-1-1   AACUGAAAAUACAAUAUUCUUCCCACCGGGAAAC.A.A.A.CU.............UGUA..................AGAGCAAUU.GAUACACUUCAUUAGGUC.U.UUGAA.GAAGUGUA...UCA...AUUGCUUUUUUUAGUUUUAGAAAGGAAAAAAGAUGACGUGGAUUU
Lsp-4-1   AAUCCAAUUAUAAAUAUUCUUCCCACCGGAGAAC.A.G.A.CU.............UGUA..................AGAGCAAUU.GAUACACUUCAUUAGGUC.U.UAGAA.GAAGUGUA...UUG...GUUUGCUUUUUUUAGUUUUGAAAGGAAAGAAGAUGACGUGAAUUU
env-757   CAUAAUAUAAUAAUUUAUACUGCCACCGGGUAGU.U.A.U.G..............CAAUAA.................GCAUAUCUC.UAUAUACAUCAUUAGGUC.U.UAGAA.GAUGUAUU...AAG...AUAAGCUUAUUUUUUUGUAAGGGGUAAAACUUAUGCGAAUGUUC
env-758   CAUAAUAUAAUAAUUUAUACUGCCACCGGGUAGU.U.A.U.G..............CAAUAA.................GCAUAUCUC.UAUAUACAUCAUUAGGUC.U.UAGAA.GAUGUAUU...AAG...AUAAGCUUAUUUUUUUGUAAGAGGUAAACUUAUGCGAAAAUUCU
env-759   AAGCGUGAACAGAAUAUUGCUGCCACCGGGCAGC.G.G.U.G..............CGGUUAAAAAC.............AAGCACCC...GAACACAUCGUUAGGUC.U.UAGAA.GAUGUGUU...CC....GGGUGCUUUUUCGCAAGUUUUCCAUUCGAGUCUG..........
env-760   AAGCGUGAACAGAAUAUUGCUGCCACCGGGCAGC.G.G.U.G..............CGGUUAAAAAC.............AAGCACCC...GAACACAUCGUUAGGUC.U.UAGAA.GAUGUGUU...CC....GGGUGCUUUUUCGCAAGUUUUCCAUUCGAGUCUGUUUGAAGCCAC
env-761   UUCGGAGAAAUUAAUACUGUUGCCACCGGGUAAC.G.G.A.A..............CGUA..................AAACGCACCA.UAUGCUUAUCGUUAGGUC.U.UAGAA.GAUACAUA...CA.....GAUGCUCAUUUUUUUUGGAAAGGAAGUUUUUUAAAAGAAUAUU
env-762   ACAAUAAAAUAUAAUUCAAUUACCGCCGGGUAAU.A.A.G.G..............CGCAU.................UUGCGUCGG.AAUUCAUGUCAUUAGGCC.A.UAGAA.GACAUGAA...UCC...CGACGCUUUUUAUUUAAAAGGAGAAAAAAUCAAAAACAAUAUUUU
env-763   AAUAUUUAGGGGUAAAACUUUACCGCCGGGUAUU.A..........................................CCUGU.C.AGCAUUCCAUUAGGCC.A.UAGAA.GGAGUGUU...UGA...UGGGGAUUUUUUUUGUAAAAAUAUUCAUGAAUUAAUAUUAAUAGU
env-764   AAACUAUAAUAAUUUUUAUCCGCCGCCGGGUGGA.U...................UUUU.....................CGCGUUG.UUUUCGUAUCGUCAGGCC.U.UAGAA.GAUACGGG...UUC...CGACGCUUUUUGUUUUCCGGGAUGCCCUCUGACAAACCUUAGGA
env-765   AUAGAGCAAAUGAAAUCAUCUGCCGCCGGGCAGA.....................AUC......................AUGCAUCG.GCUCCGCACCGUUAGGCC.U.UAGAA.GGUGCGGGGAGGCC...GAUGCAUUUUCUUUUGGAGGAAUGCAGGGUGAAGACGGCUUGGCU
env-768   AUAAAAUAAAUGAGAGUAUCUGCCGCCGGGCAGA.A.C.A.UC.....................................AUGCAUCG.GUCCUGCACCGUUAGGCC.U.UAGAA.GGUGCGGGAA.GCC...GGUGUAUUUUCUUUUUGCGGCAUCGAGAUAGAAGAUGUUGACA
env-769   AAUAGAAAUAACAGUUCAUUUACCGCCGGGUAAA.U.A.U.......................................AAAGCGUUA.AAUCUGUAUCGGCAGGCC.A.UAGGA.GAUACAGA...UUU...GGCGCUUUAUUUUUUACAGCAGACAGAACCGGAAUGUGCCGCCG
Lsp-9-1   AAGAAAUAUGACCAUUCAUUUACCGCCGGGUAAA.U.G.U.A......................................AAGCGUUG.AAUCUGUAUCGGCAGGCC.G.UAGGA.GAUACAGG...UUU...GGCGCUUUUUUGUUUGUAUGGAUAGAAUGCACAUGACUUUCAGUA
env-770   CGCGAGCUGAACUGUUUAUUUACCGCCGGGUAAA.U.A.U.A......................................AAGCGUUG.AAUCUGUAUCGAUAGGCC.A.UAGAA.GAUACAGA...UUU...GACGCUUUCUUUUUUAAUAUUAUUACAUAAAUGGGAAAGGAUU
Lsp-10-1  CUGAAUAAGAAAAAGAAAUUUGCCACCGGGUAAA.U.U.A.C..............A.........................GCGCUG.UGACUGUGUCGUUAGGUC.U.UAGAA.GACACAGC...UCC...ACGCGCUUUUCUUUUGCGCUGCGGGCGGGGCAGAUAGAAAAGGAG
Csp-15-1  ACAGUUAUAAAGAGAAAUUUACCGCCGGGUAAA.U...................AAAA.......................GCGUUG.AUCCUGUAUCAUUAGGCC.A.UAGAA.GAUACAGG...UUC...ACGCGCUUUUCUUUUGCGCGCGCAGGUAAAGCAUCUGAUUAUAUAU
env-771   ACGAAGAAAUAAAUAUCCUCUGCCGCCGGGCAGA.A.A.A..............CU.......................GUGCAUCG.GUUCCGUAUCGUUAGGCC.U.UAGAA.GAUACGGGGU.GACC..GA..UGUAUUUUCUUUGUUCCAGGAGGUAGAAAAAAAGAAUGGAUC
env-773   GCAAGAAAAAAGAAACUCUCUGCCGCCGGGCAGU.G...................C..........................GCGUUG.GUUCCGUAUCGUUAGGCC.U.UAGAA.GAUACGGG...CGCC..AGCGCUUUUUUGUUGCCCGGGGCAAGCCUGGAGGAAAACCACAAAA
env-774   AUAUAAUACCACUAUCAGCCCGCUGCCGAGUGGG.C.A.A.A..............C.......................AGCGCAAA.UCCUUGCAUCGUUAGGCC.A.UAGAA.GAUGCGGG...GAU...CGGCGCUGUUUUUCUAUCCUUUAGGCAUCGGCCGCAAAUCCAUGC
env-775   GCUAAGAUGAAGGCAAUGUUUGCUGCCGAGUAAA.C.A.................GA......................AGGCAUUUA.UCUUUGCAUCGUUAGGCC.U.UUGAA.GAUGCAUC...GAU...AGAUGCAUUUUUUAUUAUAAAGGGGGUAUUCUUAAAGCAUGGAUA
env-776   GCUAAGAUGAAGGCAAUGUUUGCUGCCGAGUAAA.C.A.................GA......................AGGCAUUUA.UCUUUGCAUCGUUAGGCC.U.UUGAA.GAUGCAUC...GAU...AGAUGCAUUUUUUAUUAUAAAGGGGGUAUUCUUAAAGCAUGGAUA
env-777   CUAUAAUACCCUUAAUUGCUUGCUGCCGAGUAAG.C.A.A................GAA........................GCAUUUA.UCUUUGCAUCGUUAGGCC.U.UUGAA.GAUGCAUC...GAU...AGAUGCAUUUUUUGUUUUAAAGGGGGUUUUUCUCAAAGCAUGGUU
env-778   UAUAUUUACUGUAAUUUAUUUGCUGCCGAGUAAA.U.A.A.A.....................................AUGCAUCUU.UCAACGUAUCGUUAGGCC.U.UUGCA.GAUACGUU...GCA...AGGUGCAUUUUUUAGUUUAAGGAGGAUAUGAAUUAAAGCAUGGAUU
env-780   UAUUAUAAUAUGAGUUUACUUGCUGCCGAGUAAG.U.A.A.AU......................................UGCAUUUA.UGUAUGUAUCAUUAGGCC.G.UUGAA.GAUACAUCG..UA.....AAUGCAUUUUUUUGUUUUAAGGAGGAUAUUUUUAAAGCAUGGGU
env-781   UGCGGGGGAUUAGUUAUACUUGCCGCCGGGUAAG.C.A.C.G..............UUU.....................UUACGUUGG.UUUUCGUAUCGUUAGGCC.U.UUGAA.GAUACGAGA..UAC...CAGC.UUUUUUUAUGGAGAAACUUUAGGAGGUUCUAAAAAAAUCG
env-782   AGAAAAGUAUAUAAAUUAACUGCCACCGGGUAGU.A.G.C.GU.............A.......................UAACGCUGG.AGUUCAUAUCGUUAGGUC.U.UAGAA.GAUAUGAAG..UUC...CGGCGAUUUUUUUUGUAAUAAAUAACAGAAGAAAAUAGUGGACGGC
env-784   AGAAAAGUAUAUAAAUUAACUGCCACCGGGUAGU.A.G.C.GU.............A.......................UAACGCUGG.AGUUCAUAUCGUUAGAUC.U.UAGAA.GAUAUGAAG..UUC...CGGCGAUUUUUUUUGUAAUAAAUAACAGAAGAAAAUAGUGGACGGC
env-785   GCUAUAAUGCAUUUUGUAACUGCCACCGGGUAGU.G.A.A.UG..............UAA.....................UAACAUUCG.UUUCCGUAUCAUCAGGUC.U.CAGAA.GAUACGGG...GAC...GAAUUUUUCUUUUAUGAGGCGUUUUAUGAAACAAUUAUCGCGUGC
env-786   GCAACCGAAUUCAUUGUUCUGCCACCGGGUAGG.A.A.U.G...............CCAA....................AAGCAUUCG.CCGGCGCACCGUCAGGUC.U.UAGAA.GGUGCGUC...CGC...GAAUGCUUUUUCCGUCUCAGAAGGAGUUGCUUUAAAAAAAAAUGG
env-787   UCAUACAUAUAAAUUUAUAAUGCCACCGGGUAUU.A.U.G.CA..............UGCAUU..................AAAGCAUUG.UUACUACAUCAUUAGGUC.U.UUGAA.GAUGUCGU...AAGG..GAUGUUUUUUUUUUAUGAAUUGAGGUAGCUAUGAAAAACUAAUCG
env-788   AUAUUACAGUUGAAUAGCGCUGCCGCCGGGCAGC.A.A.A.AU..............GCA.....................AUGCAUUUG.CCAAUACAUCGUUAGGCC.U.UUGAA.GAUGUAUC...GGU...AAAUGCAUUUUCUAUUUUUCAGGAGGAUUUUAAAAAACUCAAUUC
env-789   AUAUUACAGUUGAAUAGCGCUGCCGCCGGGCAGC.A.A.A.AU..............GCA.....................AUGCAUUUG.CCAAUACAUCGUUAGGCC.U.UUGAA.GAUGUAUC...GGU...AAAUGCAUUUUCUAUUUUUCAGGAGGAUUUUAAAAAACUCAAUUC
env-790   UGUUGAAAAUUAAAAAAAUCUGCCACCGGGUAGU.G.C.G.U..............G.......................AAAUGCAUU.UGUGUAUGUCGUUAGGUC.U.UAGAA.GAUGUACA...UGA...AUGCAUUUUUUUGGAGAAAGGAAGAAAUUGCUAUGACAAA.....
env-791   UGUUGAAAAUUAAAAAAAUCUGCCACCGGGUAGU.G.C.G.U..............G.......................AAAUGCAUU.UGUGUAUGUCGUUAGGUC.U.UAGAA.GAUGUACA...UGA...AUGCAUUUUUUUGGAGAAAGGAAGAAAUUGCUAUGACAAAAACCA
env-792   UGUUGAAAAUUAAAAAAAUCUGCCACCGGGUAGU.G.C.G.U..............G.......................AAAUGCAUU.UGUGUAUGUCGUUAGGUC.U.UAGAA.GAUGUACA...UGA...AUGCAUUUUUUUGGAGAAAGGAAGAAAUUGCUAAAACAAAAACCA
env-793   UGUUGAAAAUUAAAAAAAUCUGCCACCGGGUAGU.G.C.G.U..............G.......................AAAUGCAUU.UGUGUAUGUCGUUAGGUC.U.UAGAA.GAUGUACA...UGA...AUGCAUUUUUUUGGAGAAAGGAAGAAAUUGCUAUGACAAAAACCA
env-794   UGUUGAAAAUUAAAAAAAUCUGCCACCGGGUAGU.G.C.G.U..............G.......................AAAUGCAUU.UGUGUAUGUCGUUAGGUC.U.UAGAA.GAUGUACA...UGA...AUGCAUUUUUUUGGAGAAAGGAAGAAAUUGCUAUGACAAA.....
env-795   UGUUGAAAAUUAAAAAAAUCUGCCACCGGGUAGU.G.C.G.U..............G.......................AAAUGCAUU.UGUGUAUGUCGUUAGGUC.U.UAGAA.GAUGUACA...UGA...AUGCAUUUUUUUGGAGAAAGGAAGAAAUUGCUAAAACAAAAACCA
env-798   GAUAUACUUCCCGAAGAAUCUGCCACCGGGUAGA.A.A.U.................GCA......................AAAAGCAUU.UGCGCACAUCGUUAGGUC.U.UGGAA.GAUGUGCUUG.CAA...AUGCUUUCUUUUUUUGGAGGAAUCACGAAAAAGAAACCCUAUUUU
env-799   CAAGAAAGAUAAUAUAAAUCUUCCACCGGGAAGA.G.A.U.G..............C.......................AAGAGCAUC.UUAAUGUAUCGUUAGGUC.U.UAGAA.GAUACAUUU..AAG...AUGCUUUUUCUUUAGGAGAAGAGAAUAAAAAGGUUUUACAAAAA
env-800   AUUAUAAUAACUUUAUUAACUGCCACCGGGUAGU.A.A.A.A..............UGC.....................GCAAGCAUC.CGUGCAUAUCGUUAGGUC.U.UUGAA.GAUAUGUACA.UGG...AUGCUUAUUUUUAUUUUUGGAGGAUUUUUUAAAACAGAAGAAAUUA
env-801   UGAAGGUAACGUAUACAAACUGCCACCGGGUAGU.A.U...........................................AGCAUU.CCGCACAUCGUUAGGUC.U.UGGAA.GAUGUGCC...GGG...GUGCUUAUUUUUUGGAGGGAAGUCUAAAAAAGCAAUCCAUCGCAGC
env-802   UUAAGAAAAUGUUGAAUACCUGCCACCGGGCAGU.G.G.A.U..............GC......................GAAAGCAUC.CUGGCACAUCGUUAGGUC.U.UGGAA.GAUGUGCC...GGG...AUGCUUUUUUGUUUCUCUCCUCUGUUUUUCAAAUAGUAAGGAGGU
env-803   AUAUCGAAAUAAAUCAAAUCUGCCACCGGGUAGA.G.A.A.UG.............CAAUUA...................UGAAGCAUU.CGCGCGUAUCGUUAGGUC.U.UUGAA.GAUACGCA...CGG...GUGCUUUUUUGUUUGGAGGACCUGGAAAACUAAAAAAUACCUUGU
env-804   AUAUCGAAAUAAAUCAAAUCUGCCACCGGGUAGA.G.A.A.UG.............CAAUUA...................UGAAGCAUU.CGCGCGUAUCGUUAGGUC.U.UUGAA.GAUACGCA...CGG...GUGCUUUUUUGUUUGGAGGACCUGGAAAACUAUGAAAUACCUUGU
env-805   AUAUCGAAAUAAAUCAAAUCUGCCACCGGGUAGA.G.A.A.UG.............CAAUUA...................UGAAGCAUU.CGCGCGUAUCGUUAGGUC.U.UUGAA.GAUACGCA...CGG...GUGCUUUUUUGUUUGGAGGACCUGGAAAACUAUGAAAUACCUUGU
env-806   AUAUCGAAAUAAAUCAAAUCUGCCACCGGGUAGA.G.A.A.UG.............CAAUUA...................UGAAGCAUU.CGCGCGUAUCGUUAGGUC.U.UUGAA.GAUACGCA...CGG...GUGCUUUUUUGUUUGGAGGACCUGGAAAACUAUGAAAUACCUUGU
env-807   AUAUCGAAAUAAAUCAAAUCUGCCACCGGGUAGA.G.A.A.UG.............CAAUUA...................UGAAGCAUU.CGCGCGUAUCGUUAGGUC.U.UUGAA.GAUACGCA...CGG...GUGCUUUUUUGUUUGGAGGACCUGGAAAACUAAAAAAUACCUUGU
env-811   AUAUCGAAAUAAAUCAAAUCUGCCACCGGGUAGA.G.A.A.UG.............CAAUUA...................UGAAGCAUU.CGCGCGUAUCGUUAGGUC.U.UUGAA.GAUACGCA...CGG...GUGCUUUUUUGUUUGGAGGACCUGGAAAACUAAAAAAUACCUUGU
env-824   AUAUCGAAAUAAAUCAAAUCUGCCACCGGGUAGA.G.A.A.UG.............CAAUUA...................UGAAGCAUU.CGCGCGUAUCGUUAGGUC.U.UUGAA.GAUACGCA...CGG...GUGCUUUUUUGUUUGGAGGACCUGGAAAACUAAAAAAUACCUUGU
env-827   AUAUCGAAAUAAAUCAAAUCUGCCACCGGGUAGA.G.A.A.UG.............CAAUUA...................UGAAGCAUU.CGCGCGUAUCGUUAGGUC.U.UUGAA.GAUACGCA...CGG...GUGCUUUUUUGUUUGGAGGACCUGGAAAACUAAAAAAUACCUUGU
env-828   AUAUCGAAAUAAAUCAAAUCUGCCACCGGGUAGA.G.A.A.UG.............CAAUUA...................UGAAGCAUU.CGCGCGUAUCGUUAGGUC.U.UUGAA.GAUACGCA...CGG...GUGCUUUUUUGUUUGGAGGACCUGGAAAACUAAAAAAUACCUUGU
env-829   AUAUCGAAAUAAAUCAAAUCUGCCACCGGGUAGA.G.A.A.UG.............CAAUUA...................UGAAGCAUU.CGCGCGUAUCGUUAGGUC.U.UUGAA.GAUACGCA...CGG...GUGCUUUUUUGUUUGGAGGACCUGGAAAACUAAAAAAUACCUUGU
env-830   AUAUCGAAAUAAAUCAAAUCUGCCACCGGGUAGA.G.A.A.UG.............CAAUUA...................UGAAGCAUU.CGCGCGUAUCGUUAGGUC.U.UUGAA.GAUACGCA...CGG...GUGCUUUUUUGUUUGGAGGACCUGGAAAACUAAAAAAUACCUUGU
env-848   AUAUCGAAAUAAAUCAAAUCUGCCACCGGGUAGA.G.A.A.UG.............CAAUUA...................UGAAGCAUU.CGCGCGUAUCGUUAGGUC.U.UUGAA.GAUACGCA...CGG...GUGCUUUUUUGUUUGGAGGACCUGGAAAACUAAAAAAUACCUUGU
env-849   AUAUCGAAAUAAAUCAAAUCUGCCACCGGGUAGA.G.A.A.UG.............CAAUUA...................UGAAGCAUU.CGCGCGUAUCGUUAGGUC.U.UUGAA.GAUACGCA...CGG...GUGCUUUUUUGUUUGGAGGACCUGGAAAACUAAAAAAUACCUUGU
env-850   AUAUCGAAAUAAAUCAAAUCUGCCACCGGGUAGA.G.A.A.UG.............CAAUUA...................UGAAGCAUU.CGCGCGUAUCGUUAGGUC.U.UUGAA.GAUACGCA...CGG...GUGCUUUUUUGUUUGGAGGACCUGGAAAACUAAAAAAUACCUUGU
env-851   AUAUCGAAAUAAAUCAAAUCUGCCACCGGGUAGA.G.A.A.UG.............CAAUUA...................UGAAGCAUU.CGCGCGUAUCGUUAGGUC.U.UUGAA.GAUACGCA...CGG...GUGCUUUUUUGUUUGGAGGACCUGGAAAACUAAAAAAUACCUUGU
env-855   AUAUCGAAAUAAAUCAAAUCUGCCACCGGGUAGA.G.A.A.UG.............CAAUUA...................UGAAGCAUU.CGCGCGUAUCGUUAGGUC.U.UUGAA.GAUACGCA...CGG...GUGCUUUUUUGUUUGGAGGACCUGGAAAACUAAAAAAUACCUUGU
env-856   AUAUCGAAAUAAAUCAAAUCUGCCACCGGGUAGA.G.A.A.UG.............CAAUUA...................UGAAGCAUU.CGCGCGUAUCGUUAGGUC.U.UUGAA.GAUACGCA...CGG...GUGCUUUUUUGUUUGGAGGACCUGGAAAACUAAAAAAUACCUUGU
env-858   AUAUCGAAAUAAAUCAAAUCUGCCACCGGGUAGA.G.A.A.UG.............CAAUUA...................UGAAGCAUU.CGCGCGUAUCGUUAGGUC.U.UUGAA.GAUACGCA...CGG...GUGCUUUUUUGUUUGGAGGACCUGGAAAACUAAAAAAUACCUUGU
env-860   AUAUCGAAAUAAAUCAAAUCUGCCACCGGGUAGA.G.A.A.UG.............CAAUUA...................UGAAGCAUU.CGCGCGUAUCGUUAGGUC.U.UUGAA.GAUACGCA...CGG...GUGCUUUUUUGUUUGGAGGACCUGGAAAACUAUGAAAUACCUUGU
env-861   AUAUCGAAAUAAAUCAAAUCUGCCACCGGGUAGA.G.A.A.UG.............CAAUUA...................UGAAGCAUU.CGCGCGUAUCGUUAGGUC.U.UUGAA.GAUACGCA...CGG...GUGCUUUUUUGUUUGGAGGACCUGGAAAACUAAAAAAUACCUUGU
env-862   AAUCAAUGGGUAAAUAAAUCUGCCACCGGGUAGA......................GAAUGCAUAA...............UGAAGCAUU.CGCGCGUUUCGUUAGGUC.U.UUGAA.GAAACGCA...CGG...GUGCUUUUUUGUUUGGAGGAAAGCAAAAAAGAAGCUGAUAAAUUAC
env-863   ........AAUAAACAAAUCAGCCACCGGGUUGA.G.A.U.G..............C.......................AAAAGCAUC.ACGGCAUAUCGUUAGGUC.U.UGGAA.GAUAUGCA...GUG...AUGCUUUUUUGAAAGGAUGGAAAAAAAAGCUUAUAAAAAGGGA
env-864   ACAAAGACAGCUUUUGUAUCUGCCGCCGGGUAGA.............................................CAUCUUC.UGCUCAUUCCAUUAGGCC.A.UUGAA.GGAAUGAA...CUG...AAGAUGUUUUUUAUAGCAGAUAUAUCCAACAGUCUAUAUGCGC
env-865   ACAAAGACAGCUUUUGUAUCUGCCGCCGGGUAGA.............................................CAUCUUC.UGCUCAUUCCAUUAGGCC.A.UUGAA.GGAAUGAA...CUG...AAGAUGUUUUUUAUAGCAGAUAUAUCCAACAGUCUAUAUGCGC
env-866   UACUUAGGUAAAUACUUAUUUGCCGCUGGGUAAA.............................................CAUCUUA.UAUUCGUUUCGUUAGGCC.U.UAGAA.GAAACGUU...UGU...GAGGUGUUAUUUUUUAUAGUUUUUAUUAAAAGGAAUUAAUUAAAG

          ........................<<<....................................................................................>>>...................................................................
          ........................20.....................................................................................02...................................................................
          .............<<<<<<<<<<<....>>>>>>.>.>.>.>>......<<<<<<<<..........>>>>>>>>.....<<<<<<<<<.<<<<<<<<<<..................>>>>>>>>...>>>...>>>>>>>>..........................
          .............22222222222....222222.2.2.2.22........1111............1111.......222222222.2222222222..................22222222...222...22222222..........................
          5'                  YUGCCRCCGGGUAR                                         R   Y       YRY YCRUUAGGYC    U GAA GR RYR        R   YUUUUUUY                         3'
```

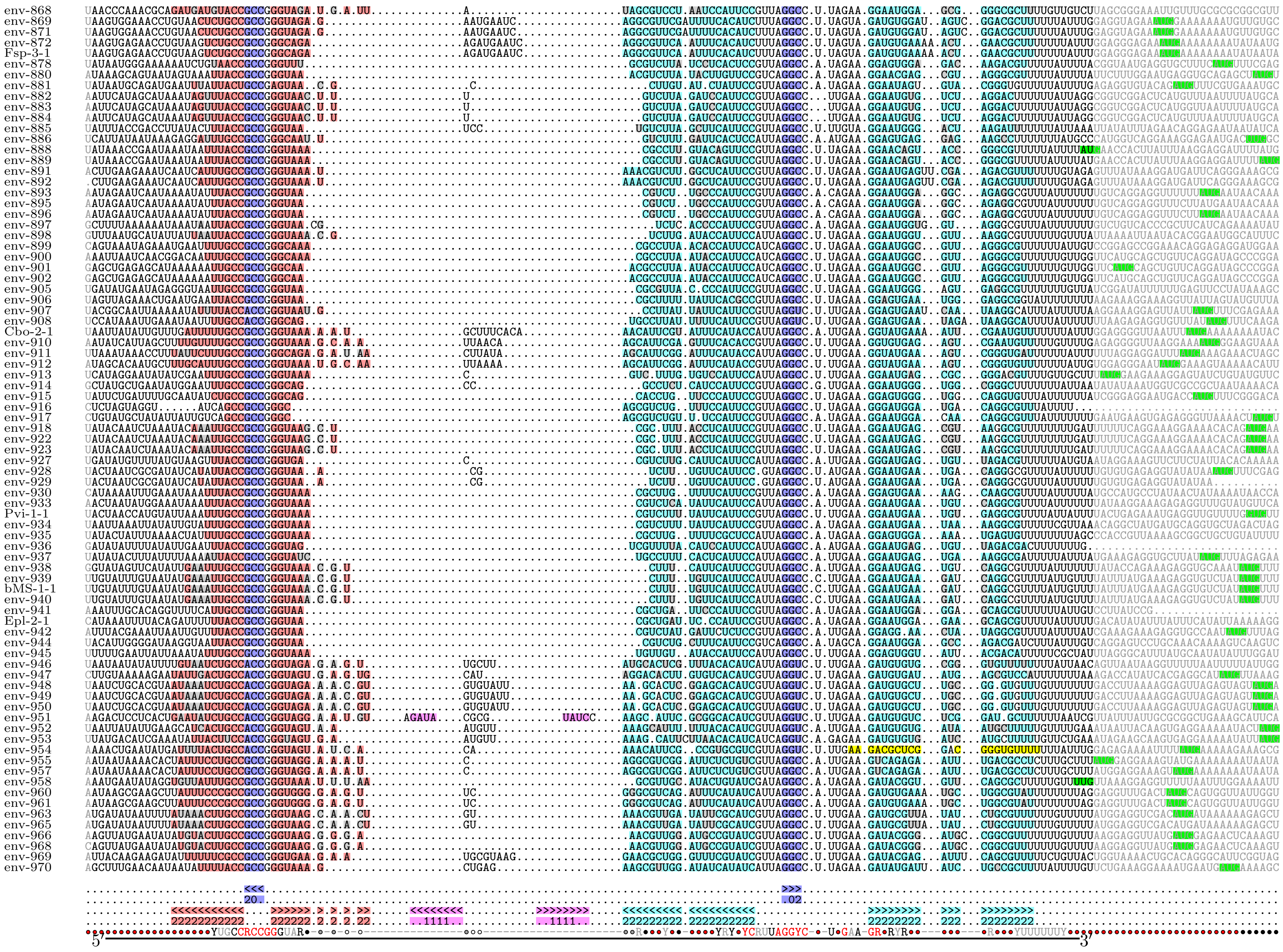
env-868
env-869
env-871
env-872
Fsp-3-1
env-878
env-880
env-881
env-882
env-883
env-884
env-885
env-886
env-888
env-889
env-891
env-892
env-893
env-895
env-896
env-897
env-898
env-899
env-900
env-901
env-902
env-905
env-906
env-907
env-908
Cbo-2-1
env-910
env-911
env-912
env-913
env-914
env-915
env-916
env-917
env-918
env-922
env-923
env-927
env-928
env-929
env-930
env-933
Pvi-1-1
env-934
env-935
env-936
env-937
env-938
env-939
bMS-1-1
env-940
env-941
Epl-2-1
env-942
env-944
env-945
env-946
env-947
env-948
env-949
env-950
env-951
env-952
env-953
env-954
env-955
env-957
env-958
env-960
env-961
env-963
env-965
env-966
env-968
env-969
env-970
5'
3'

```
env-971   AAAGUGAAACGGCACUUUUUUGCCACCGGGUAAA.A.A.A.G....ACAGGAGU...UUUG..........ACUCCAGA..GCGCCAGA...CUUUCUAUCAUUAGGUC.A.UAGAA.GAUAGGAA...G.G...UCUGGCAUUUUUUAUGCAGAAUGACGAAAAGGAGCAGAAAAUGAA
env-972   UCAAUAACAACCUUAAUUUUUAUCGCCGGAUAAA.A.A.U.U...............U.......................GAGCGUCAC.ACUUCCCAUC.GCAGGCC.U.UUGAA.GAUGGGAA...GCA...UGGCGCUCUUUUAUUUUGAGGAGGAUUGGUCAAAUUGGAACAGAC
env-973   CUGCAGAAAAUAAUACUAUUUGCCGCCGGGUAAA.......................CCA.....................GAAUGUUGG.AUUUUGUAUCAGUAGGCC.G.UUGGA.GAUACAAA...GUG...CCGGCAUUUUUUUAUUGACUGUAUUCAGCCGGAGCCUUUUCCUUA
env-974   CUGCAGAAAAUAAUACUAUUUGCCGCCGGGUAAA.......................CCA.....................GAAUGUUGG.AUUUUGUAUCAGUAGGCC.G.UUGGA.GAUACAAA...GUG...CCGGCAUUUUUUUAUUGACUGUAUUCAGCCGGAGCCUUUUCCUUA
env-976   CCACCAUAAGAUUGUUGGCUUGCCGCCGGGUAAG.C.A.U.A...............GA......................AAACGCUGG.AUUUCGUAUCGUUAGGCC.A.UUGAA.GAUACGAAA..UGC...CAGCGUUUUUCUUUUUAGGAGGUUACGAUGAACACCACACAAAAC
env-977   GGAAAAAUAACAAUUAAACUCGCCGCCGGGUGAG.A.A.G.UG..............CUAAUUU.................AAGCGACGG.AUUUCGUAUCGUUAGGCC.U.UAGAA.GAUACGGA...GUG...CGUCGCUUUUAUAAUACUGGAGGUAUAAAAAUGUUUGCAUAUUAU
env-978   UACAUAAUAAGGAAUGAUCUCGCCGCCGGGUGAG.G.A.C.G...............CUGAAAAAAAAGC............AGCGCCGG.AUUUCGUAUCGUUAGGCC.A.UUGAA.GAUGCGGA...AUG...CGGCGCUUUUUGUUUAGGCGUCUCUCCGCGCGGCGAGUGGUACAG
env-979   UACAUAAUAAGGAAUGAUCUCGCCGCCGGGUGAG.G.A.C.G...............CUGAAAAAAAAGC............AGCGCCGG.AUUUCGUAUCGUUAGGCC.A.UUGAA.GAUGCGGA...AUG...CGGCGCUUUUUGUUUAGGCGUCUCUCCGCGCGGCGAGUGGUACAG
env-980   UACAUAAUAAGGAAUGAUCUCGCCGCCGGGUGAG.G.A.C.G...............CUGAAAAAAAAGC............AGCGCCGG.AUUUCGUAUCGUUAGGCC.A.UUGAA.GAUGCGGA...AUG...CGGCGCUUUUUGUUUAGGCGUCUCUCCGCGCGGCGAGUGGUACAG
env-981   GAAACAAAUAAGAACCACACUGCCGCCGGGCAGU.U.G.C.G................CGAG...................GAGCGCCGG.AUUUCGUAUCGUUAGGCC.A.UUGAA.GAUGCGGA...AUG...CGGCGCUUUUUGUUU..............................
env-982   GAAACAAAUAAGAACCACACUGCCGCCGGGCAGU.U.G.C.G...............CGAG....................GAGCGCCGG.AUUUCGUAUCGUUAGGCC.U.UAGAA.GAUAUGAG...AUAC..CGGCGCUUGUUUUAUCGGAGGAAUGAGAAUGGAGAAGAACAGACA
env-983   GAAACAAAUAAGAACCACACUGCCGCCGGGCAGU.U.G.C.G...............CGAG....................GAGCGCCGG.AUUUCGUAUCGUUAGGCC.U.UAGAA.GAUAUGAG...AUAC..CGGCGCUUGUUUUAUCGGAGGAAUGAGAAUGGAGAAGAACAGACA
env-984   GAAACAAAUAAGAACCACACUGCCGCCGGGCAGU.U.G.C.G...............CGAG....................GAGCGCCGG.AUUUCGUAUCGUUAGGCC.U.UAGAA.GAUAUGAG...AUAC..CGGCGCUUGUUUUAUCGGAGGAAUGAGAAUGGAGAAGAACAGACA
env-985   GAAACAAAUAAGAACCACACUGCCGCCGGGCAGU.U.G.C.G...............CGAG....................GAGCGCCGG.AUUUCGUAUCGUUAGGCC.U.UAGAA.GAUAUGAG...AUAC..CGGCGCUUGUUUUAUCGGAGGAAUGAGAAUGGAGAAGAACAGACA
env-986   GAAACAAAUAAGAACCACACUGCCGCCGGGCAGU.U.G.C.G...............CGAG....................GAGCGCCGG.AUUUCGUAUCGUUAGGCC.U.UAGAA.GAUAUGAG...AUAC..CGGCGCUUGUUUUAUCGGAGG........................
Cba-2-1   AACGUUAAUAUAUAGCAACUCACCGCCGGG.GAG.U.....................AUGG.....................AGCGUUGG.AUAUCGUAUCGUUAGGCC.A.UAGAA.GAUACGAG...AUU...CGGCGCUUUUUGUUAUAUUAAGCAGAUAUAAAAGAAAAGAUUAAA
env-987   AAAAUAUAAGAAAUGAAAUUUGCCGCCGGGCAA........................UC.......................AGCGUUGA.ACCUUGUAUCCAUAGGCC.U.UAGAG.GAUACAG....AUU...UGGCGCUUUUUUUGUGAACGGCAAGACAAAACAAAUGAGAAAGAG
env-988   AAAAUAUAAGAAAUGAAAUUUGCCGCCGGGCAA........................UC.......................AGCGUUGA.ACCUUGUAUCCAUAGGCC.U.UAGAG.GAUACAG....AUU...UGGCGCUUUUUUUGUGAACGGCAAGACAAAACAAAUGAGAAAGAG
env-989   UCGGAAGAUACGUGGUUGUCUGCCACCGGGUAGG.C.A.A.C...............AG......................AAGCGUCAG.ACUUUCUAUCGUUAGGUC.U.CAGAA.GAUGGAAA...GCC...UGACCUUUUUUAUGUUGCAGGACAUAUGAAAAAGAGAACAGAGAA
env-990   UAACAGAAUCGGUAAUCCUCUGCCGCCGGGUAGA.G.G.G.................GA......................UUGCACCAG.ACUUCAUAUCGUUAGGCC.G.UUGAA.GAUAUGAA...GUUC..UGGUGUAUUUUUAUUCUAUAGGAAAGAACUUGUCACGCUAAAGCG
env-991   UAAAUAAAAGAUACGUUCUUUGCCACCGGGUGAA.G.A.A.................GUU.....................AAGCGUCAU.AUUUCCUUUC.GUAGGUC.U.UUGAA.GAUAGGAA...GUAG..GGGCGCAUUUUUUUUGGAUUAACGGAGCUGAAAACAAGCGUCCAA
env-994   AAUAUUGAACGACACUUUUUUGCCGCCGGGUAAA.G.A.G..U......................................UAAGCGUUA.GGCUUUCAUCGUUAGGCC.U.UUGAA.GAUAGAAA...GUUCU.GGCGCUAUUUUUUUGUUUAGGAGAGAUGUUAUGGAGAAAAAGACA
env-995   AAAGAUUAAACGUAGCUUUUUGCCACCGGGUAAA.A.A.G.AU..............U.......................AAGCGUCAG.ACUUUCUAUCAUUAGGUC.U.UUGAA.GAUAGAGA...GUU...UGAUGUUUUUUUGUAUAGAGAGGUAAACAGAUGAAAUCAAAAGAA
env-997   AAUAUGAACGGGUGAUUCUUUGCCGCCGGGUAAA.G.A.A.U...............GA......................AGCGCCAGA.ACCUUCUAUCGUUAGGCC.A.UUGAA.GAUAGAAG...GUC...CUGGCGCUGUUUUAUGCACACGGGCACCCAGAGGAAAUAUGUCAG
env-998   GAAUCAGACAGACCAGUUUUUACCGCCGGGUAAG.A.A.U.U...............U.......................AUGCGUCAG.GCUUUCUAUCGAUAGGCC.A.UAGAA.GAUAGAAA...GGC...UGGCGUUUUUUGUGCCGAAGUAUUUUUUAAGAGGUUCUGUUUGGA
env-999   AAAUUGAAUUGACAAUUCUUUGCCGCCGGGUAAA.G.G.G.UU......................................GUGCGUCAG.ACUUCCUAUCAGUAGGCC.A.UUGAA.GAUAGGAG...GGC...UGGCGUUUUUUGUUGCAGAAACAAAAAGGAAUGGAGGUUUACCAA
env-1001  AUUAAAUAAUUACGCUGUUUUGCCGCCGGGUAAA.A.C.A.GC..............G.......................GAGCGUCAG.ACUUUCUAUCAUUAGGUC.U.UUGAA.GAUAGAAA....GC...UGGCGCUUUUUAUGAAUAAGGAGACAAGAUAUAUGGAGCAGACAA
env-1012  GAAUAAAAAAGGCACUUUUUUGCCACCGGGUAAA.G.A.A.G...............AA......................CAGCGUCGG.ACUUUCUAUCGUUAGGUC.U.UAGAA.GAUAGAAAU..UGC...CGGCGCUUUUUUGCUGAGGAGACAGAUAUGAUUGAGAAAAAUAUU
env-1015  UAGCUCAUUGGCUUGUUCUUUGCCGCCGGGUAAA.G.A...................CAUU....................AAGCGCCGG.ACUUCAUAUCGUUAGGCC.G.UUGAA.GAUAUGAA...GACC..CGGCGCUUUUAUUGUGAGAAGAGGUUUGUCCGUGGUGCUCAAACA
env-1016  UAAAACUAAAAAUAUUUCUUUGCCGCCGGGUAAA.G.A.G.UU......................................GAGCGUCAG.AUUUUCUAUCGUUAGGCC.U.UUGAA.GAUAGAAA...UUU...GCCGCUUUAUUUUUGUAUAAAAAGGAGCAUUUAUGAACAAUCAGU
env-1017  GUUCAUAAUUGAGCGCUCUUUGCCGCCGGGUAAA.G.A.G.A...............A.......................AAGCGCCAG.GCUUUCCAUCGUUAGGCC.A.UUGAA.GAUGGAAAG..AUC...UGGCGCUAUUUUUAAUUUUAAUAGGACGGGAGUAUGUGAGAUUU
Lba-2-1   AGAAAACAACGUUGCUCUUUUGCCACCGGGUAAA.G.A.G.................AUA.....................AAACGUCAG.GCUUCCUAUCGUUAGGUC.U.UAGAA.GAUAGAAA...GUUC..UGACGUUUUAUUUGUGGAGAAAUUCCUUUACAACUACCUAAAAAU
env-1022  AAUAGUAGAUGAUACUUUUCUGCCACCGGGUAGA.A.G.A.GU..............C.......................AAUCGCUGA.ACUUUCUAUCGUUAGGUC.U.AAGAA.GAUAGAAAG..CUU...CGGCGUUUUUUAUUUACAGGAAAGAGGUAAAUCAUGUUUAGAGAA
env-1023  CACAGAUCAUGACACUUUUCUGCCACCGGGUAGA.G.A.A.G...............AU......................AUGCGUCAG.ACUUUCUAUCGUUAGGUC.U.UAGAA.GAUAGAGG...GUGC..UGGCGCUUUUUUUGAUUCAUAGGAAAUUCCUUCCUAUGAAUCAAA
env-1024  AAUAAAUCAUGACUUUUUUCUGCCACCGGGUAGA.A.A.A.AA..............AU......................AAGCGUCAG.AGUUUCUAUCGUUAGGUC.U.UAGAA.GAUAGAAA...CCA...UGGCGCUUUAUUUUUUAUGUACUAGGAAAUUCCGU..........
env-1025  AAUAAAUCAUGACUUUUUUCUGCCACCGGGUAGA.A.A.A.AA..............AU......................AAGCGUCAG.AGUUUCUAUCGUUAGGUC.U.UAGAA.GAUAGAAA...CCA...UGGCGCUUUAUUUUUUAUGUACUAGGAAAUUCCGUUGGAACUCCC
env-1026  AAUAAAUCAUGACUUUUUUCUGCCACCGGGUAGA.A.A.A.AA..............AU......................AAGCGUCAG.AGUUUCUAUCGUUAGGUC.U.UAGAA.GAUAGAAA...CCA...UGGCGCUUUAUUUUUUAUGUACUAGGAAAUUCCGUUGGAACUCCC
env-1040  AAUAAAUCAUGACUUUUUUCUGCCACCGGGUAGA.A.A.A.AA..............AU......................AAGCGUCAG.AGUUUCUAUCGUUAGGUC.U.UAGAA.GAUAGAAA...CCA...UGGCGCUUUAUUUUUUAUGUACUAGGAAAUUCCGUUGGAACUCCC
env-1045  AAUAAAUCAUGACUUUUUUCUGCCACCGGGUAGA.A.A.A.AA..............AU......................AAGCGUCAG.AGUUUCUAUCGUUAGGUC.U.UAGAA.GAUAGAAA...CCA...UGGCGCUUUAUUUUUUAUGUACUAGGAAAUUCCGUUGGAACUCCC
env-1046  AAUAAAUCAUGACUUUUUUCUGCCACCGGGUAGA.A.A.A.AA..............AU......................AAGCGUCAG.AGUUUCUAUCGUUAGGUC.U.UAGAA.GAUAGAAA...CCA...UGGCGCUUUAUUUUUUAUAAACUUAUAAAAAUCAAGAAAGGAGA
env-1047  AAUAAAUCAUGACUUUUUUCUGCCACCGGGUAGA.A.A.A.AA..............AU......................AAGCGUCAG.AGUUUCUAUCGUUAGGUC.U.UAGAA.GAUAGAAA...CCA...UGGCGCUUUAUUUUUUAUGUACUAGGAAAUUC.............
env-1048  AAUAAAUCAUGACUUUUUUCUGCCACCGGGUAGA.A.A.A.AA..............AU......................AAGCGUCAG.AGUUUCUAUCGUUAGGUC.U.UAGAA.GAUAGAAA...CCA...UGGCGCUUUAUUUUUUAUGUACUAGGAAAUUCCGUUGGAACUCCC
env-1049  AAUAAAUCAUGACUUUUUUCUGCCACCGGGUAGA.A.A.A.AA..............AU......................AAGCGUCAG.AGUUUCUAUCGUUAGGUC.U.UAGAA.GAUAGAAA...CCA...UGGCGCUUUAUUUUUUAUGUACUAGGAAAUUCCGUUGGAACUCCC
env-1052  AAUAAAUCAUGACUUUUUUCUGCCACCGGGUAGA.A.A.A.AA..............AU......................AAGCGUCAG.AGUUUCUAUCGUUAGGUC.U.UAGAA.GAUAGAAA...CCA...UGGCGCUUUAUUUUUUAUGUACUAGGAAAUUCCGUUGGAACUCCC
env-1053  AAUAAAUCAUGACUUUUUUCUGCCACCGGGUAGA.A.A.A.AA..............AU......................AAGCGUCAG.AGUUUCUAUCGUUAGGUC.U.UAGAA.GAUAGAAA...CCA...UGGCGCUUUAUUUUUUAUGUACUAGGAAAUUCCGUUGGAACUCCC
env-1054  AAUAAAUCAUGACUUUUUUCUGCCACCGGGUAGA.A.A.A.AA..............AU......................AAGCGUCAG.AGUUUCUAUCGUUAGGUC.U.UAGAA.GAUAGAAA...CCA...UGGCGCUUUAUUUUUUA............................
env-1055  AAUAAAUCAUGACUUUUUUCUGCCACCGGGUAGA.A.A.A.AA..............AU......................AAGCGUCAG.AGUUUCUAUCGUUAGGUC.U.UAGAA.GAUAGAAA...CCA...UGGCGCUUUAUUUUUUAUGUACUAGGAAAUUCCGUUGGAACUCCC
env-1056  AAUAAAUCAUGACUUUUUUCUGCCACCGGGUAGA.A.A.A.AA..............AU......................AAGCGUCAG.AGUUUCUAUCGUUAGGUC.U.UAGAA.GAUAGAAA...CCA...UGGCGCUUUAUUUUUUAUGUACUAGGAAAUUCCGUUGGAACUCCC
env-1057  AAUAGAACACGAUACUUUUCUGCCACCGGGUAGG.A.G.A.GU..............U.......................AAGCGUCAG.AUUUUCUAUCGUUAGGUC.U.UAGAA.GAUAGGAAA..UGC...UGGCGCUUUAUUUUUGGAAAAAUAGGAAGGAGCUUAGAAGCUGAU
env-1058  AAUAGAACACGAUACUCUUCUGCCACCGGGUAGG.A.G.A.GU..............U.......................AAGCGUCAG.AUUUUCUAUCGUUAGGUC.U.UAGAA.GAUAGAAA...AUAC..UGGCGCUUUCUUAUUGAAAGGAGACUAUAAUGAAUAAUCCAAUAA
env-1059  AAUAGAUUAUGAUACUUUCCUGCCACCGGGUAGG.G.G.A.GU..............U.......................AAGCGUCAG.AAUUUCUAUCGUUAGGUC.U.GAGAA.GAUAGAAAG..CUC...UGGCGCUUUAUUUUUGGAAAAAGAGGAAAAGGAGUGUAAGAGCU
env-1060  AAUAAACUACGACACUUUUCUGCCACCGGGUAGG.A.A.A.GU..............U.......................AAGCGUCAG.GAUUUCUAUCGUUAGGUC.U.UAGAA.GAUAGAAA...UUUC..UGGCGCUUUAUUUUUAAAAAGCAAAGAAAAGGAGACAAAACAUGU
env-1061  AAUAAAUUACGAUACUUUCCUGCCACCGGGUAGG.A.A.A.GU..............U.......................AAGCGUCAG.AAUUUCUAUCGUUAGGUC.U.GAGAA.GAUAGAAAG..UUU...UGGCGCUUUUUUUUACAGAAAUAAAAAGGAGUGUAAGAGAAGAUG
env-1062  AAUAAAUUACGAUACUUUCCUGCCACCGGGUAGG.A.A.A.GU..............U.......................AAGCGUCAG.AAUUUCUAUCGUUAGGUC.U.GAGAA.GAUAGAAAG..UUU...UGGCGCUUUUUUUUACAGAAAUAAAAAGGAGUGUAAGAGAAGAUG
env-1070  AAUAAAUUACGAUACUUUCCUGCCACCGGGUAGG.A.A.A.GU..............U.......................AAGCGUCAG.AAUUUCUAUCGUUAGGUC.U.GAGAA.GAUAGAAAG..UUU...UGGCGCUUUUUUUUACAGAAAUAAAAAGGAGUGUAAGAGAAGAUG
env-1071  AAUAAAUUACGGCACUUUCCUGCCACCGGGUAGG.G.A.A.GU..............U.......................AAGCGUCAG.AGUUUCUAUCGUUAGGUC.A.UAGAA.GAUAGGAA...UUU...UGGCGUUUUUUUUUUCGAGUUUUAUAAUAUGAGAGGAGUUUAAGA
env-1072  AACAAGAUGAGAUACUUUUCUGCCACCGGGUAGG.A.A.A.GU..............U.......................AAGCGUCAG.AAUUUCUAUCGUUAGGUC.A.AAGAA.GAUAGAAAU..UUC...UGGCGCUUUAUUUUGUUAUGGCGACGAGAAAAAAUUAAAAAGGAG
env-1073  AAUGUAUUACGGCUCUUUUCUGCCACCGGGUAGG.A.A.A.GG..............U.......................AAGCGUCAG.AAUUUCUAUCGUUAGGUC.U.GAGAA.GAUAGGAA...UCC...UGGCGCUUUUUAUUUGUAAUCCUGCCCGAGGGCAUUCUUGAACGC
env-1074  AAUGUAUUACGGCUCUUUUCUGCCACCGGGUAGG.A.A.A.GG..............U.......................AAGCGUCAG.AAUUUCUAUCGUUAGGUC.U.GAGAA.GAUAGGAA...UCC...UGGCGCUUUUUAUUUGUAAUCCUGCCCGAGGGCAUUCUUGAAUUU
env-1075  AAUGUAUUACGGCUCUUUUCUGCCACCGGGUAGG.A.A.A.GG..............U.......................AAGCGUCAG.AAUUUCUAUCGUUAGGUC.U.GAGAA.GAUAGGAA...UCC...UGGCGCUUUUUAUUUGUAAUCCUGCCCGAGGGCAUUCUUGAAUUU
env-1076  AAUGUAUUACGGCUCUUUUCUGCCACCGGGUAGG.A.A.A.GG..............U.......................AAGCGUCAG.AAUUUCUAUCGUUAGGUC.U.GAGAA.GAUAGGAA...UCC...UGGCGCUUUUUAUUUGUAAUCCUGCCCGAGGGCAUUCUUGAAUUU
env-1077  AAUGUAUUACGGCUCUUUUCUGCCACCGGGUAGG.A.A.A.GG..............U.......................AAGCGUCAG.AAUUUCUAUCGUUAGGUC.U.GAGAA.GAUAGGAA...UCC...UGGCGCUUUUUAUUUGUAAUCCUGCCCGAGGGCAUUCUUGAAUUU
env-1078  AAUGUAUUACGGCUCUUUUCUGCCACCGGGUAGG.A.A.A.GG..............U.......................AAGCGUCAG.AAUUUCUAUCGUUAGGUC.U.GAGAA.GAUAGGAA...UCC...UGGCGCUUUUUAUUUGUAAUCCUGCCCGAGGGCAUUCUUGAAUUU
env-1079  AAUAAAUAAAGAUACUUUCUUGCCACCGGGUAAG.A.A.A.GU..............U.......................AAGCGUCUA.AAUUUCUAUCGUUAGGUC.U.UAGAA.GAUAGAAA...UAA...GGACGCUUAUUUUUUUGAAAGAAAAGGAGAAUAAAAUAAUGUUUA
env-1080  UAAUAUAAAGGCUUAUUCCUUGCCGCCGGGUAUG.G.A.A.U...............U.......................CAGCGCCAG.ACUUUCUAUCGUUAGGCC.A.UUGAA.GAUAGAAG...GCUC..UGGCGCUUUUUUUCAAAUGUACAAACCAAACUUGAAAAUAAUCAG
env-1081  UAAUAUAAAGGCUUAUUCCUUGCCGCCGGGUAUG.G.A.A.U...............U.......................CAGCGCCAG.ACUUUCUAUCGUUAGGCC.A.UUGAA.GAUAGAAG...GCUC..UGGCGCUUUUUUUCAAAUGUACAAACCAAACUUGAAAAUAAUCAG
env-1082  AUAACUCGCAGUUGUUUCUUUGCCGCCGGGUAAA.G.A.A.AU...............CU.....................AGGCGUCAG.CUUUCCUAUCGUUAGGCC.A.UUGAA.GAUAGGAAG..UGC...UGGCGCUUUUGUUUUUGAGAUGAGAAAGAGAAGGAAGCAAUAUGA
env-1083  AUAACUCGCAGUUGUUUCUUUGCCGCCGGGUAAA.G.A.A.AU...............CU.....................AGGCGUCAG.CUUUCCUAUCGUUAGGCC.A.UUGAA.GAUAGGAAG..UGC...UGGCGCUUUUGUUUUUGAGAUGAGAAAGAGAAGGAAGCAAUAUGA
env-1084  AUAACUCGCAGUUGUUUCUUUGCCGCCGGGUAAA.G.A.A.AU...............CU.....................AGGCGUCAG.CUUUCCUAUCGUUAGGCC.A.UUGAA.GAUAGGAAG..UGC...UGGCGCUUUUGUUUUUGAGAUGAGAAAGAGAAGGAAGCAAUAUGA
Ano-1-1   AUAACUCGCAGUUGUUUCUUUGCCGCCGGGUAAA.G.A.A.AU...............CU.....................AGGCGUCAG.CUUUCCUAUCGUUAGGCC.A.UUGAA.GAUAGGAAG..UGC...UGGCGCUUUUGUUUUUGAGAUGAGAAAGAGAAGGAAGCAAUAUGA
env-1085  AUAACUCGCAGUUGUUUCUUUGCCGCCGGGUAAA.G.A.A.AU...............CU.....................AGGCGUCAG.CUUUCCUAUCGUUAGGCC.A.UUGAA.GAUAGGAAG..UGC...UGGCGCUUUUGUUUUUGAGAUGA......................
env-1086  AUAACUCGCAGUUGUUUCUUUGCCGCCGGGUAAA.G.A.A.AU...............CU.....................AGGCGUCAG.CUUUCCUAUCGUUAGGCC.A.UUGAA.GAUAGGAAG..UGC...UGGCGCUUUUGUUUUUGAGAUGAGAAAGAGAAGGAAGCAAUAUGA
env-1087  AUAACUCGCAGUUGUUUCUUUGCCGCCGGGUAAA.G.A.A.AU...............CU.....................AGGCGUCAG.CUUUCCUAUCGUUAGGCC.A.UUGAA.GAUAGGAAG..UGC...UGGCGCUUUUGUUUUUGAGAUGAGAAAGAGAAGGAAGCAAAAUGA
env-1088  AUAACUCGCAGUUGUUUCUUUGCCGCCGGGUAAA.G.A.A.AU...............CU.....................AGGCGUCAG.CUUUCCUAUCGUUAGGCC.A.UUGAA.GAUAGGAAG..UGC...UGGCGCUUUUGUUUUUGAGAUGAGAAAGAGAAGGAAGCAAUAUGA
Cst-1-1   AUAACUCGCAGUUGUUUCUUUGCCGCCGGGUAAA.G.A.A.AU...............CU.....................AGGCGUCAG.CUUUCCUAUCGUUAGGCC.A.UUGAA.GAUAGGAAG..UGC...UGGCGCUUUUGUUUUUGAGAUGAGAAAGAGAAGGAAGCAAUAUGA
env-1089  AUAACUCGCAGUUGUUUCUUUGCCGCCGGGUAAA.G.A.A.AU...............CU.....................AGGCGUCAG.CUUUCCUAUCGUUAGGCC.A.UUGAA.GAUAGGAAG..UGC...UGGCGCUUUUGUUUUUGAGAUGAGAAAGAGAAGGAAGCAAUAUGA
env-1090  AGAACUAAACGCUGUUUCUUUACCACCGGGUAAA.G.G.A.UU..............C.......................AAGCAUCAG.ACUUUCUAUCGUUAGGUC.A.UUGAA.GAUAGGAA...GUAC..UGGUGCUUUAUUUUUUUGUAAAGGAGAUAUUUUAUUAUGGAAAAC
env-1091  AUAUGAGAAAGACACUUCUUUGCCGCCGGGUAAA.G.A.G.CU......................................AAGCGUCAG.AUUUUCUAUCGAUAGGCC.U.UUGAA.GAUAGAAA....UC...UGGCGUUCUUUUUUUCUUUACCUUUGGCAGCGCAGUUUGCGGUUA
env-1092  AAUAUGGAAAGGAUGCUUUUUGCCGCCGGGUAAG.A.A.G.U...............U.......................GAGCGCCAG.GCUUUCUAUCGUUAGGCC.U.UUGAA.GAUAGAAA...GCUC..UGGCGCAUUUUUUGUCUGUCCGGGGCUUUUGCAUACGAGGAGGAA
env-1094  AAUAAUUUAAGGACACUUUUUGCCGCCGGGUAAG.G.A.G.U...............U.......................AAGCGUCAG.GCUUUCUAUCGUUAGGCC.U.UUGGA.GAUAGAAG...GUUC..UGGCGUUAUUUUUGUUUAGGAGUAGUGAGAUUAUGGAACAAAGAG
env-1095  AAUAUAAGAUGACACUUCUUUGCCGCCGGGUAAA.G.A.A.UU..............U.......................AAGCGUCAG.AAUUUCUGUCAUUAGGCC.U.UUGAA.GACAGGAA...UCC...UGGCGCUUUUUAUAUGUCGGAAGGAGAAACAUCAUGAAGGAACG

          ........................<<<..............................................................................>>>........................................................................
          ........................20...............................................................................02.........................................................................
          .............<<<<<<<<<<<....>>>>>>.>.>.>.>>......<<<<<<<<...........>>>>>>>>.....<<<<<<<<<.<<<<<<<<<<.................>>>>>>>>...>>>...>>>>>>>>.....................................
          .............22222222222....222222.2.2.2.22........1111.................1111.......222222222.2222222222.................22222222...222...22222222.....................................
          5′ ••••••••••••••••••YUGCCRCCGGGUAR•-∘-∘-∘-∘--------------∘∘∘------------------∘∘R•••Y••-••••YRY•YCRUUAGGYC-•-U•GAA-GR•RYR••---•••---•R•••YUUUUUUY••••••••••••••••••••••••••••••••• 3′
```

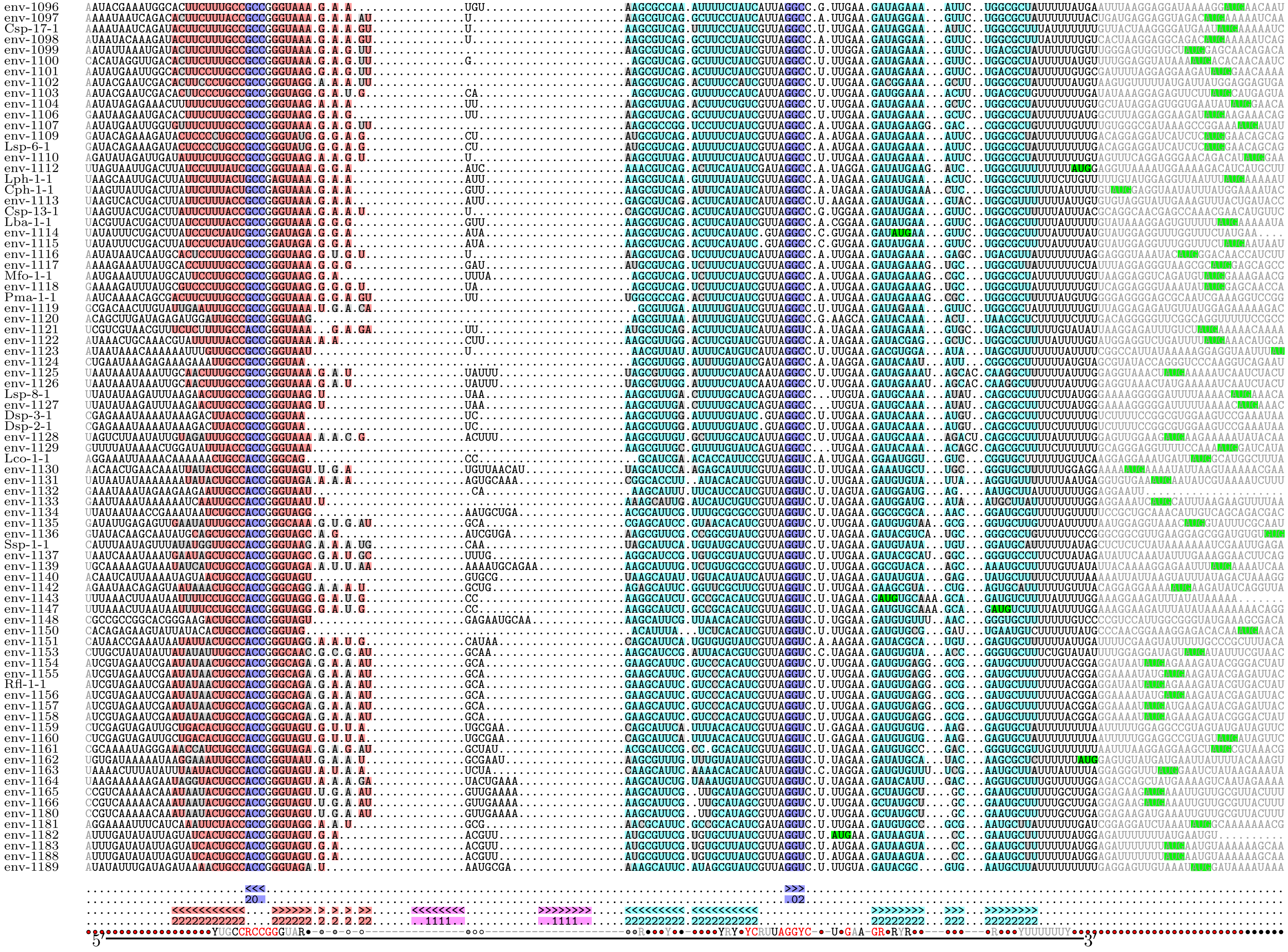
env-1096
env-1097
Csp-17-1
env-1098
env-1099
env-1100
env-1101
env-1102
env-1103
env-1104
env-1106
env-1107
env-1109
Lsp-6-1
env-1110
env-1112
Lph-1-1
Cph-1-1
env-1113
Csp-13-1
Lba-1-1
env-1114
env-1115
env-1116
env-1117
Mfo-1-1
env-1118
Pma-1-1
env-1119
env-1120
env-1121
env-1122
env-1123
env-1124
env-1125
env-1126
Lsp-8-1
env-1127
Dsp-3-1
Dsp-2-1
env-1128
env-1129
Lco-1-1
env-1130
env-1131
env-1132
env-1133
env-1134
env-1135
env-1136
Ssp-1-1
env-1137
env-1139
env-1140
env-1142
env-1143
env-1147
env-1148
env-1150
env-1151
env-1153
env-1154
env-1155
Rfl-1-1
env-1156
env-1157
env-1158
env-1159
env-1160
env-1161
env-1162
env-1163
env-1164
env-1165
env-1166
env-1180
env-1181
env-1182
env-1183
env-1188
env-1189
5'
3'

```
env-1190 UUACAAUAAAAAAGAAAAGCCGCCACCGGUGGC.A.A.U.AU..............GUUGAAAAU..............CAGCAUUCG.UUUGCGCUUCGUUAGGUC.U.UUGAA.AAAGCGCA...GGU...GAAUGCUUUUUUGAUAAGGUGAAAU[illegible]AAGCACAAUCCUUUUAA
env-1191 UUUUAUUAGUUUCAAAUAGCUGCCACCGGUAGA.U.G...................AACGAG.................GAGCGUUCU.UUUUCACAUCAUUAGGUC.U.UAGAA.GAUGUGAA...AGG...GAAGGCUUAUUUUUUUGAGGUGAAAAAG[illegible]AAAAAGGGAUUAU
env-1193 UUUUAUUAGUUUCAAAUAGCUGCCACCGGUAGA.U.G...................AACGAG.................GAGCGUUCU.UUUUCACAUCAUUAGGUC.U.UAGAA.GAUGUGAA...AGG...GAAGGCUUAUUUUUUUGAGGUGAAAAAGAUGAAAAAGGG.....
env-1194 UAUAUUAUAUUGAGUUUAUCUGCCGCCGGUAGG.U.A.A.AC..............A......................UCGCAUUUA.UCUGCGUGUCGUAAGGCC.U.UAGAA.GACGCGCUU..GGU...AAGUGCGUUUUUUAUUAUAGCGCUCUGCGUGGGGCUUUUGCAGGG
env-1195 UAUAUUAUAUUGAGUUUAUCUGCCGCCGGUAGG.U.A.A.AC..............A......................UCGCAUUUA.UCUGCGUGUCGUAAGGCC.U.UAGAA.GACGCGCUU..GGU...AAGUGCGUUUUUUAUUAUAGCGCUCUGCGUGGGGCUUUUGCAGGG
env-1196 CAUCAUAUAGAGAAAAAAUCUGCCGCCGGUAGA.U.A...................AAA......................GCAUUUG.UCAGCAUAUCGUAAGGCC.U.UAGAA.GAUGUGCA...GGC...AGGUGCCAUUUUUUUUGAUAUAUGACCUAGUCCAAUUUAUUGAAA
Cbu-1-1  UGAUAUAUUGAAUUAAAGAUUACCACCGGUAAG.A.A.U.................GUAGC..................GAGCAUUCA.UUUGUACAUCAUUAGGUC.U.UAGAA.GAUGUGCA...AAU...GGAUGCUUUUUUGCAAAUAAUAAAUUUACGAUUUAAGAAAUUGGA
Csp-9-1  UGAUAUAUUGAAUUAAAGAUUACCACCGGUAAG.A.A.U.................GUAGC..................GAGCAUUCA.UUUGUACAUCAUUAGGUC.U.UAGAA.GAUGUGCA...AAU...GGAUGCUUUUUUGCAAAUAAUAAAUUUACGAUUUAAGAAAUUGGA
env-1197 UGAUAUAUUGAAUUAAAGAUUACCACCGGUAAG.A.A.U.................GUAGC..................GAGCAUUCA.UUUGUACAUCAUUAGGUC.U.UAGAA.GAUGUGCA...AAU...GGAUGCUUUUUUGCAAAUAAUAAAUUUACGAUUUAAGAAAUUGGA
Cbu-3-1  UGAUAUAUUGAAUUAAAGAUUACCACCGGUAAG.A.A.U.................GUAGC..................GAGCAUUCA.UUUGUACAUCAUUAGGUC.U.UAGAA.GAUGUGCA...AAU...GGAUGCUUUUUUGCAAAUAAUAAAUUUACGAUUUAAGAAAUUGGA
env-1199 CCGCAGCUCAAUAUUCAAUCUGCCACCGGUAGA.......................GAUGCG.................AAAGCACCG.UCAGUACAUCGUUAGGUC.U.UUGAA.GAUGUACC...UGC...GGUGCUUUUUCGGUAAAUUUACGGAGGAUAUAAUGCUCAG.....
env-1200 AAAUAACAAUACAAUAAUUCUGCCACCGGUAGA.G.A.U.G...............CGU....................AAAGCACCG.U.GGUACAUCGUUAGGUC.U.UUGUA.GAUGUACU...CAC...GGUGCUUUUUCGGUGAAUUUACGGAGGAUAUA[illegible]UUAAAAAAGC
env-1202 AAAUAACAAUACAAUAAUUCUGCCACCGGUAGA.G.A.U.G...............CGU....................AAAGCACCG.U.GGUACAUCGUUAGGUC.U.UUGUA.GAUGUACU...CAC...GGUGCUUUUUCGGUGAAUUUACGGAGGAUAUAAUGUUAAA.....
env-1204 AAAUGACAAUAUAAUAAUUCUGCCACCGGUAGA.G.A.U.G...............CGU....................UCAGCACCG.UCAGUACAUCGUUAGGUC.U.UUGAA.GAUGUGCU...CAC...GGUGCUUUUUAUCGGAUAAAAAUAAGGAGAAACAGAU[illegible]CUCAG
env-1205 AUGAUACAGUAAAUCAUAACUGCCACCGGCAGA.U.A...................AAUGCGAA...............AAAGCAUUU.GAAGCACAUCGUUAGGUC.U.UUGAA.GAUGUGCU....CA...GAUGCUUUAUUUUUUUGAAAGGAUUCUUU[illegible]AAGAUCAAAAAC
env-1206 CUUAUACAACUUAAAACGACUGCCACCGGUAGU.......................GAAGCCU................ACAGCAUUC.GCCGUAUAUCGUUAGGUC.U.UAGAA.GAUAUACGCA.GCG...AAUGCUGUUUUUUGUAGGAGGUACUAUGAAAAUCAGAAACCCUUU
env-1208 AUAAUAUUGCAAUAUAAAACUGCCACCGGUAGU.G.A.A.G...............CUU....................ACAGCAUUC.GCCGUACAUCGUUAGGUC.U.UAGAA.GAUGUGCGCA.ACG...AAUGCUGUUUUUUGUUGGAGGAUAU[illegible]AAAAUAAGAAACCCAUU
env-1209 AUAAUAUUGCAAUAUAAAACUGCCACCGGUAGU.G.A.A.G...............CUU....................ACAGCAUUC.GCCGUACAUCGUUAGGUC.U.UAGAA.GAUGUGCGCA.ACG...AAUGCUGUUUUUUGUUGGAGGAUAUAUGAAAAUAAGAAACCCAUU
env-1210 AUAAUAUUGCAAUAUAAAACUGCCACCGGUAGU.G.A.A.G...............CUU....................ACAGCAUUC.GCCGUACAUCGUUAGGUC.U.UAGAA.GAUGUGCGCA.ACG...AAUGCUGUUUUUUGUUGGAGGAUAU[illegible]AAAAUAAGAAACCCAUU
env-1211 UUUAAUUAUUGAAUAUUAAAUCCCACCGGGAUU.G.A.A.UG..............CA.....................GGGCAUUCG.UGUACACAUCGUUAGGUC.U.UUGAA.GAUGUGUAU..AAC...GGAUGCCUUGAUUUGUUUAAUGGAUUAAUACUUGAUAUGAAGGAG
env-1212 UUUAAUUAUUGAAUAUUAAAUCCCACCGGGAUU.G.A.A.UG..............CA.....................GGGCAUUCG.UGUACACAUCGUUAGGUC.U.UUGAA.GAUGUGUAU..AAC...GGAUGCCUUGAUUUGUUUAAUGGAUUAAUACUUGAUAUGAAGGAG
env-1214 UGUAAACGAAUCAUAAUAUCAGCCGCCGGUUGA.G.A.A.UG..............UAAA...................AAGCAUUCG.CGUGCACAUCAUCAGGCC.U.UUGAA.GAUGUGUU...UGC...GGGUGCUUUUUGUUAUGA.........................
env-1215 GUAUUUUAGCUGUAUGAUACUGCCACCGGUAGA.A.U.A.A...............CGGUGCGA...............AAGCACCCG.CAAACACAUCAUUAGGUC.U.UUGAA.GAUGUGUU...UAC...GGGUGCUUUUUUUGUGUGAGGAGAUUUUUAUGUUCAAAAAGAUA
env-1217 UAUUUUGACGGUAUUGUAACUGCCACCGGUAG..A.A.C.AA..............UGGUGCGA...............AAGCACUCG.CAAACACAUCAGUAGGUC.U.UGGAA.GAUGUGUU...UAC...GAGAGCUUUUUUUGCUUUUUAUGUGAAAGGGAAUUAAAGAUAAAG
env-1220 CCUGAAGCUGAAUAUGAAUCUGCCACCGGCAGA.G.G.C.A...............GUGCAUU................AUGCGCUCG.CAAACACAUCGUUAGGUC.U.UUGAA.GAUGUGUU...UAC...GGGUGCGUUUUUUUUGGAGGUUUUU[illegible]AUAAAAAGACUUUUUGG
env-1221 GGAUAACAUCAUUAUGAAACUGCCACCGGUAGA.G.U.G.GU..............GCAAA..................GAGCACCCG.CAGACACAUCGUUAGGUC.U.UAGAA.GAUGUGUC...UAC...GGGUGCCUUUUUUAUGGAGGUUUUCGU[illegible]AGUAAAAAAGCAUUA
env-1222 CAGAAAAAAACAAACAGGUCUGCCACCGGUAGA.U.U...................AAGAAUGCAA.............AGGCAUUUG..CUGCACAUCGUUAGGUC.U.UUGAA.GAUGUGCG...GAC...AAAUGCCUUUAUUUUUUGGAGAUAUUGUGAUUAAACGCUUGAUAA
env-1223 AGAAUAAAUUAAAUAUAUGCUGCCACCGGUAGC.U.U.U.GA..............AU.....................UAGCAUUCA.UUUAUACAUCAUUAGGUC.U.UAGAA.GAUGUAUAU..UUU...GAAUGCUUUUUUGUAGGAGAAGAAUGA[illegible]AAAAUACAAAAUCCU
env-1224 UUCGAAUUGAAUAAUAAAUCUGCCACCGGUAGA.......................GAAUGCA................AGGCAUUCG.CCGGCAUAUCGUUAGGUC.U.UAGAA.GAUAUGUC...AGC...GAAUGCCUUGUCUGGUAUUUGGCCGGGGGAAAAGGAUUUGGCAAU
env-1225 GUGAAAAAUAAAAAAGACUCUGCCGCCGGCAGA.G.A.U.................GCGA...................AAGCAUUCC.CAAGCACAUCGUUAGGCC.U.UAGAA.GAUGUGCU...UGC...GAAUGCUUUUUUCGUUUUUAUACAAGGAGGAUGUUU[illegible]CGCAAG
env-1226 GUGAAAAAUAAAAAAGACUCUGCCGCCGGCAGA.G.A.U.................GCGA...................AAGCAUUCC.CAAGCACAUCGUUAGGCC.U.UAGAA.GAUGUGCU...UGC...GAAUGCUUUUUUCGUUUUUAUACAAGGAGG[illegible]UUUAUGCGCAAG
env-1227 GAAAUUUUUUAUAAUCAGAAUGCCACCGGUAUU.C.A.A.G...............AUGCAGCU...............AAGCAUCCG.UAUACACAUCGUUAGGUC.U.UUGAA.GAUGUGUA...UAC...GGAUGCUUUUUUCAGGAGGGUUAGC[illegible]AAAAUGCUGAAUUAUAU
env-1228 UAGCUAUAAUAAAAUUUAGCUGCCGCCGGUAGU.A.A.A.................UGCUG..................AUGCAUUCG.UGUGCACAUCGUUAGGUC.U.UUGUA.GAUGUGUA...UUC...GGAUGCUUUUUAAUUUUAAACGGAGAGGAAAAUGUU[illegible]AUAAA
env-1229 CGCAACUAAAUAUUGAUAUUUGCCACCGGCAAA.G.G...................AAUGUA.................AAGCAUUCA.UACGCUUAUCGUUAGGUC.U.UUGAA.GAUAAGCA...UAU...GGGUGCUUAUUUUUUUGGAGAUUUAU[illegible]AAAAAACUAAAAGCAA
env-1230 CGCAGUUAAACACGAAUAUUUGCCACCGGCAAA.A.G.A.AU..............GCC....................AAGCAUUCA.UGUACUUAUCGUUAGGUC.U.UUGAA.GAUAAGUA...UGU...GGGUGCUUAUUUUUUUGGAGGUAAAGUU[illegible]GUAAAGAAAUUAUU
env-1231 CACAAUUAAAUAUGACUAUUUGCCACCGGCAAA.A.G.A.AU..............GUA....................AAGCAUUCA.UAUACUUAUCGUUAGGUC.U.UUGAA.GAUAAGUA...UAU...GAAUGCUUAAUUUUUUGGAGGCCAU[illegible]AAAUCAUUGAAAUCGAU
env-1237 CACAAUUAAAUAUGACUAUUUGCCACCGGCAAA.A.G.A.AU..............GUA....................AAGCAUUCA.UAUACUUAUCGUUAGGUC.U.UUGAA.GAUAAGUA...UAU...GAAUGCUUAAUUUUUUGGAGGCCAUAUGAAAUCAUUGAA......
env-1238 CACAAUUAAAUAUGACUAUUUGCCACCGGCAAA.A.G.A.AU..............GUA....................AAGCAUUCA.UAUACUUAUCGUUAGGUC.U.UUGAA.GAUAAGUA...UAU...GAAUGCUUAAUUUUUUGGAGGCCAUAUGAAAUCAUUGAAAUCGAU
env-1239 CACAAUUAAAUAUGACUAUUUGCCACCGGCAAA.A.G.A.AU..............GUA....................AAGCAUUCA.UAUACUUAUCGUUAGGUC.U.UUGAA.GAUAAGUA...UAU...GAAUGCUUAAUUUUUUGGAGGCCAUAUGAAAUCAUUGAAAUCGAU
env-1240 CACAAUUAAAUAUGACUAUUUGCCACCGGCAAA.A.G.A.AU..............GUA....................AAGCAUUCA.UAUACUUAUCGUUAGGUC.U.UUGAA.GAUAAGUA...UAU...GAAUGCUUAAUUUUUUGGAGGCCAU[illegible]AAAUCAUUGAAAUCGAU
env-1241 CACAAUUAAAUAUGACUAUUUGCCACCGGCAAA.A.G.A.AU..............GUA....................AAGCAUUCA.UAUACUUAUCGUUAGGUC.U.UUGAA.GAUAAGUA...UAU...GAAUGCUUAAUUUUUUGGAGGCCAUAUGAAAUCAUUGAAAUCGAU
env-1242 UCUCAGUUGAAUAAACAAUUUGCCACCGGCAAA.A.G.A.AU..............GUU....................GAGCAUUCA.UAUACUUAUCGUUAGGUC.U.UUGAA.GAUAAGUA...UGU...GGGUGCUAUUUUUUUUGGAGGUUAUAUGAAAUCGUUCAAAGCAA
env-1243 UCUCAGUUGAAUAAAAGAUUUGCCACCGGCAAA.C.G.A.AU..............GUA....................AAGCAUUCA.GGUACUUAUCGUUAGGUC.U.UUGAA.GAUAAGUG...CGU...GGGUGCUAUUUUUUUGGAGGUUUAU[illegible]AAAUCGAUCAAAUCAA
env-1244 GGACAGUUGAAUACAAGUUUUGCCACCGGCAAG.A.A.U.................GCA....................GAGCAUUCA.UAUACUUAUCGUUAGGUC.U.UUGAA.GAUAAGUA...UGU...GGGUGCUUAUUUUUUUGUGGAGGAAACAUGAAGUAUCUCAAAUC
env-1245 CAGAAUACAAAAUAAAAAACUGCCACCGGCAGA.G.A.A.AU..............GCG....................CAGGCAUUC.GGCGCACAUCGUUAGGUC.U.UUGAA.GAUGUGUG...GGC...GGAUGCUUAUUUUUUUGGAGUGAAGAUU[illegible]AAAGCAAACGGUUU
env-1246 UAGAAAAAACAAAAAAUAUCUGCCACCGGCAGA.A.A.A.AU..............GCGGAA.................CGGCAUUCU.CGUUUACAUCGUUAGGUC.U.GAGAA.GAUGUAAA...CAG...GGAUGCCGUUUUUUGAGGUCUUU[illegible]AAAUUUUUGAUCGAUACUC
env-1247 UACUGAGAAAAAAUACUUACUGCCACCGGUAGU.C.U.G.................CUUUA..................GAGCAUUCG.UUUGUAUAUCGUUAGGUC.U.UAGAA.GAUAUGCAU..UGC...GGGUGUUUUAUUUUUGGAGGAGAUU[illegible]AAAAGCUUCACAAAAU
env-1248 AUCUUAUAAGCAAUUAAAUCUGCCACCGGUAGU.G.A.G.A...............UGCUU..................UAGCAUCCG.UUUACACAUCGUUAGGUC.U.UUGAA.GAUGUGUU...AGC...GGAUGCUUGUUUAUUUUUUAUGGAAGUUUUAUGAGUAUACA...
env-1249 A.UAUAUUAUAUAUAGAAUUUGCCACCGGCAAG.A.A.G.AA..............UGUA...................UAGCAUUCG.UUAACAUAUCGUUAGGUC.U.UUGAA.GAUGUGUUU..AGC...GUAUGCUUAUUUUUUUGGAGGUAUAU[illegible]AAAAUAAAGAAUUUGU
env-1250 ..UAUAUUAUAUAUAGAAUUUGCCACCGGCAAG.A.A.G.AA..............UGUA...................UAGCAUUCG.UUAACAUAUCGUUAGGUC.U.UUGAA.GAUGUGUUU..AGC...GUAUGCUUAUUUUUUUGGAGGUAUAU[illegible]AAAAUAAAGAAUUGGU
env-1251 A.UAUAUUAUAUAUAGAAUUUGCCACCGGCAAG.A.A.G.AA..............UGUA...................UAGCAUUCG.UUAACAUAUCGUUAGGUC.U.UUGAA.GAUGUGUUU..AGC...GUAUGCUUAUUUUUUUGGAGGUAUAU[illegible]AAAAUAAAGAAUUGGU
env-1252 ACGUGAUAUAAUACCGUCACUGCCACCGGUAGA.G.A.U.G...............CAAUAAACU..............UUGCGUCCG.UUGACACAUCAUUAGGUC.U.UUGAA.GAUGUGUC...UGC...GGGCGCUUUUUAUUUUCAGGAGGUAAAA[illegible]UCAUUAAAAAGUA
env-1253 UAAUGCUAUAAUACCGACACUGCCACCGGUAGA.G.A.U.G...............CCAAU..................AGGCGUCCG.UUGACACAUCAUUAGGUC.A.UUGAA.GAUGUGUC...UGC...GGACGCUUUUUUUAUUUAUAAGGAGGAAAAAC[illegible]GCAAAGAAA
env-1254 CCAUGCUAUAAUACCGACACUGCCACCGGUAGA.G.A.C.G...............CAAAU..................CAGCGUCCG.UUGACACAUCAUUAGGUC.U.UUGAA.GAUGUGUC...UGC...GGACGCUUUUUAUUUUGGAGGAAAAU[illegible]AAUAUUGGAAUAGAUA
env-1255 UUGAUAAUCAGAUAAUAAUCUGCCACCGGUAGU.U.G.A.AU..............GUAGA..................AAGCAUUUG.UAAGCAUAUCGUUAGGUC.U.UAGAA.GAUAUGUU...UAC...GGGUGCUUUUUUGGAGGAUAUA[illegible]ACGAAAUUAAAAACAUGGAU
env-1258 AAAUUAAAAAUAAUAUAAUCUGCCACCGGUAGA.G.G.U.UG..............CAUAA..................AAGCAUCCG.UCAAUACAUCGUUAGGUC.U.UAGAA.GGUGUAUU...UUC...GGAUGCUUUUAUGUGUAAGUCAGCCGGCUAAAAAUUUUAAGGAGU
env-1260 GAAUGAUAUAAUAAUAACACUGCCACCGGCAGA.A.U.A.UU..............GCAGAU.................AAGCAUUUG.CGUUCGCAUCGUUAGGUC.U.CAGAA.GAUGCGGGC..AGC...AAAUGCUUUUUUAUUUGAGGUGACUAA[illegible]AAGCUAUUUUCCAAA
env-1262 UAUAAUUAUAAUAAAACUACUGCCACCGGUAGA.G.A.A.U...............GUAAUACU...............GAACAUUCG.CGGGCAUAUCGUUAGGUC.U.UUGAA.GAUAUGCC....GCG..GA.UGUUUUUUGUUUUUCGGGUGUAUAAUA[illegible]AAAAAGCUUACC
env-1263 UAUAAUUAUAAUAAAACUACUGCCACCGGUAGA.G.A.A.U...............GUAAUACU...............GAACAUUCG.CGGGCAUAUCGUUAGGUC.U.UAGAA.GAUAUGCC....GCG..GA.UGUUUUUUGUUUUUCGGGUGUAUAAUA[illegible]AAAAAGCUUACC
env-1264 AAAAAGAAUAUUAGAAAUACUGCCACCGGUAGA.A.U.G.AU..............GCGGA..................AAGCAUUCG.UGAGCAUAUCGUUAGGUC.U.GAGAA.GAUAUGUGG..AGC...GGGUGCUUUUUUUUAUAAAAGGAAGGAUGGAGGAAAAG[illegible]AAAAA
env-1266 AUUCUUUUUACAAUAAAAACUGCCACCGGUAGA.A.A.A.AA..............UGCUA..................UUGCAUUUG.UUUGUGCGUCGUUAGGUC.U.UAGAA.GACGUGCA...AAU...GAAUGCUUUUUUUUGUUUAAGCC[illegible]AAAAACCGUAACAUUUUCA
env-1269 AUUCUUUUUACAAUAAAAACUGCCACCGGUAGA.A.A.A.AA..............UGCUA..................UUGCAUUUG.UUUGUGCGUCGUUAGGUC.U.UAGAA.GACGUGCA...AAU...GAAUGCUUUUUUUUGUUUAAGCUAUGAAAAACCGUAACAUUUUCA
env-1270 UAUUUAGCGAGUUGAAUAUUUGCCACCGGCAAUGU.G.A.U...............GCAUUU.................ACGCAUCCG..UUACACAUCGUUAGGUC.U.UAGAA.GAUGUGGA...AGC...GGGUGCUUAUUUUUUUGGAGGUUUCUA[illegible]AUACUACGCAAUCCA
env-1271 UAGGGCAGGUUUACAAUAUCUGCCACCGGUAGA..............................................AGCAUUC..GCAGCGUGUCGUUAGGUC.U.UUGAA.GAUACGCU...GUC...GAGUGCUUAUUUUUUUGGAGGAAUCGGA[illegible]AAGAAAAAAAUUAAA
Eba-1-1  GUGUAUAAUAAGUUAAAAAUUACCACCGGUAAG.A.A.U.................GCAC...................AAGCAUUCA.UACACACAUCGUUAGGUC.A.UGGAA.GAUGUGUG...UAU...GGGUGCUUAUUUUUUGGAGGUUAU[illegible]AAAAAAACAAGGAUGUUU
env-1272 ACCUGUAAAACUGAAUAUUCUGCCACCGGUAGA.A.....................AUACGAAUGGA............AAACAUUUG.UCUGCAUGUCGUUAGGUC.U.UUGAA.GAUUUGCA...GAG...AAAUGUUUCUUUUUGAUAUUUUUAUGCAUCCGGCUGAUAGAUAU
env-1273 UAAAAAAAUAAUUAAAUAUCUGCCACCGGUAGA.......................A......................AAACAUUUG.UCUGCAUGUCAUAAGGUC.U.UAGAA.GAGAUGCA...GGU...GAAUGUUUCUUUUUUUAGGAGGAGUGUCAAUGCAGUAUUUUA...
env-1274 AAAAAAUCAGAUUAUAAUUCUGCCACCGGCAGA.G.A.C.G...............AAAG...................AAACAUUCG.UCCGCAUGUCAUUAGGUC.U.UUGAA.GACAUGCA...GAG...GAAUGUUUCUUUUUUGGAUAGUUUUAUGCAGUUUCUGCAUUGAUA
env-1275 UAUGAUUAAGUUAAAUAAUCUGCCACCGGUAGA.......................AAUGCACG...............AAGCAUUUG.CUUGUAUAUCGUUAGGUC.U.UUGAA.GAUAUACAA..AGU...AAAUGCUUAUUUUUUUGAAAGGAUAAUUAAACAUCAUACUUUGG
env-1276 AAUGAACAAAAUAAAUAGUCUGCCACCGGUAGG.......................AAUGUAA................AAACAUUCG.UGUGCAUAUCGUUAGGUC.U.UUGAA.GAUAUGCA...UAU...GAAUGUUUAUUUUUUGGAGGUAAUU[illegible]AAAUAUUUUACAAAGG
env-1278 UAAUAAGUAAAAUUAAAUACUGCCACCGGCAGA.U.A.G.AA..............UGCAAA.................AAGCAUUCG.UCGGCGUAUCGUUAGGUC.U.UAGAA.GAUACGUUU..GGC...GGAUGCUUAUUUUUUGGAGGUUUUAUACU[illegible]AAAUUCAAAAAUC
env-1279 UAAUAAGUAAAAUUAAAUACUGCCACCGGCAGA.U.A.G.AA..............UGCAAA.................AAGCAUUCG.UCGGCGUAUCGUUAGGUC.U.UAGAA.GAUACGUUU..GGC...GGAUGCUUAUUUUUUGGAGGUUUUAUACU[illegible]AAAUUCAAAAAUC
env-1288 UAAUAAGUAAAAUUAAAUACUGCCACCGGCAGA.U.A.G.AA..............UGCAAA.................AAGCAUUCG.UCGGCGUAUCGUUAGGUC.U.UAGAA.GAUACGUUU..GGC...GGAUGCUUAUUUUUUGGAGGUUUUAUACUAUGAAAUUCAAAAAUC
env-1289 UAAUAAGUAAAAUUAAAUACUGCCACCGGCAGA.U.A.G.AA..............UGCAAA.................AAGCAUUCG.UCGGCGUAUCGUUAGGUC.U.UAGAA.GAUACGUUU..GGC...GGAUGCUUAUUUUUUGGAGGUUUUAUACUAUGAAAUUCAAAAAUC
env-1290 UAAUAAGUAAAAUUAAAUACUGCCACCGGCAGA.U.A.G.AA..............UGCAAA.................AAGCAUUCG.UCGGCGUAUCGUUAGGUC.U.UAGAA.GAUACGUUU..GGC...GGAUGCUUAUUUUUUGGAGGUUUUAUACU[illegible]AAAUUCAAAAAUC
env-1291 UAAUAAGUAAAAUUAAAUACUGCCACCGGCAGA.U.A.G.AA..............UGCAAA.................AAGCAUUCG.UCGGCGUAUCGUUAGGUC.U.UAGAA.GAUACGUUU..GGC...GGAUGCUUAUUUUUUGGAGGUUUUAUACU[illegible]AAAUUCAAAAAUC
env-1292 AAAACAAAUAAAUUAAAUACCGCCACCGGUGGU.U.A.A.U...............UGCAG..................AAGCAUUCG.UUUAUACAUCGUUAGGUC.U.UUGAA.GAUGUAUA...AAAC..GGGUGCUUUUUUUGAAAGGAGUAUCAUGAA[illegible]AAUCUGAAAAAA
Cbo-1-1  UAAUCAGUUUAAAUUAAAUCUGCCACCGGUAGG.A.A.U.G...............CAUAU..................GUGCAUUCA.CAUACACAUCGUUAGGUC.A.UAGAA.GAUGUGUAU..AGU...GGGUGCUUUUUUUAUGUAAAAAAGUAUCUGCUAUAUUAUAUAGUU
env-1293 GUAAUAAAAGGAUUAUGAACUGCCACCGGUAGU.A.A.A.................AUGCGAU................AAGCAUUCG.UUCACACAUCGUUAGGUC.U.CAGAA.GAUGUGUA...UGC...GAGUGCUUUAUUUUUUUGGAGGAUAUGGAAUGAAAAUUAAAUCUG
env-1294 UAUACUACCGCUAUACGUUCUGCCACCGGUAGA.G.A.A.AA..............UGCGA..................AAGCAUUCG.UUUGUCCAUCGUUAGGUC.U.UAGAA.GAUGGGCAAA.AAC...GAAUGCUUUUUUUGUUGGAGGAAUUCU[illegible]AACAUGCGCUGGUU
env-1296 UAUACUACCGCUAUACGUUCUGCCACCGGUAGA.G.A.A.AA..............UGCGA..................AAGCAUUCG.UUUGUCCAUCGUUAGGUC.U.UAGAA.GAUGGGCAAA.AAC...GAAUGCUUUUUUUGUUGGAGGAAUUCU[illegible]AACAUGCGCUGGUU

         .......................<<<............................................................................>>>........................................................................
         .......................20.............................................................................02.........................................................................
         .............<<<<<<<<<<<....>>>>>>.>.>.>.>>.......<<<<<<<<...........>>>>>>>>....<<<<<<<<<.<<<<<<<<<<.................>>>>>>>>...>>>...>>>>>>>>..............................
         .............22222222222....222222.2.2.2.22.........1111...............1111......222222222.2222222222.................22222222...222...22222222..............................
         •••••••••••••••••YUGCCRCCGGGUAR•-◦-◦-◦-◦--------------◦◦◦-------------------◦◦R•••Y••-••••YRY•YCRUUAGGYC-•-U•GAA-GR•RYR••---•••---R•••YUUUUUUY••••••••••••••••••••••••••••••••••••
          5'                                                                                                                                                          3'
```

```
env-1300  GUUUUUAUUAUACUGAUACCUGCCACCGGGUAGG.A.G.A.AA............UGCAAA..................CAGCAUUCA.U.UACACAUCGUUAGGUC.U.UAGAA.GAUGUGCA...UAU...GAAUGCUUGUUUUUAAAUCACAAAAAAAUUUCAGAGCAAACAGG
env-1301  AUUAAAAAAGUAUAUUUUACUGCCACCGGGUAGU.A.U.G.A.............AUGA....................GAGCAUUCA.UAGAUUCAUCGUUAGGUC.U.UAGAA.GAUGGAUCA..UAU...GGAUGCUUUCUUUUUGUGAAGAAAAAGGAGGAAAGGAUGAAAGAA
env-1302  AUAAAAUUUAAAAUAAAUACCGCCACCGGGUGGU.A.U.................AAUGUUC.................AAGCAUUUG.GAAAUACAUCGUUAGGUC.U.UAGAA.GAUGUGUU...UAC...GGAUGCUUUCUUUUUUUGGGAGGAUAUAUGCAAUCUAUAAGAAAA
env-1303  UAUGGUAUCAUAUCUUAAACUGCCACCGGGUAGA.A.A.U.G.............CAAA....................AAGCAUUUG.UUGGCGCAUCGUUAGGUC.UUUAGAA.GAUGUGCC...GCC...AAAUGUUUAUUUUUUUGGAGGAAAACAUGAUAAAGAAACUUACAA
env-1304  GGAAAAUGCGAAUAAAUGAUUGCCACCGGGUAAU.C.G.................AAAAUGCAGAUA............AAGCAUUCG.UUUGCGUAUCGUCAGGUC.U.UAGCA.GAUGCGCG...CGC...GGAUGCUUAUUUUUUUGAGGUGUUUCAUGGAAACGAAAAGAAAAC
env-1305  GGAAAAUGCGAAUAAAUGAUUGCCACCGGGUAAU.C.G.................AAAAUGCAGAUA............AAGCAUUCG.UUUGCGUAUCGUCAGGUC.U.UAGCA.GAUGCGCG...CGC...GGAUGCUUAUUUUUUUGAGGUGUUUCAUGGAAACGAAAAGAAAAC
env-1306  AAAUAAUUCAAUAAGUUAAUUGCCACCGGGUAAU.G.A.A.U.............GU......................CAGCAUUCG.AUUGCAUAUCGUUAGGUC.U.UAGAA.GAUAUGCC...GUC...GGAUGCUUUUUUGAUAACGGCUGUUUUAUAAGGAUGGACUAUGAA
env-1307  AAAUAAUUCAAUAAGUUAAUUGCCACCGGGUAAU.G.A.A.U.............GU......................CAGCAUUCG.AUUGCAUAUCGUUAGGUC.U.UAGAA.GAUAUGCC...GUC...GGAUGCUUUUUUGAUAACGGUUGUUUUAUAGGGAGAAAUUAUGAA
env-1308  AAAUAAUUCAAUAAGUUAAUUGCCACCGGGUAAU.G.A.A.U.............GU......................CAGCAUUCG.AUUGCAUAUCGUUAGGUC.U.UAGAA.GAUAUGCC...GUC...GGAUGCUUUUUUGAUAACGGUUGUUUUAUAGGGAGGAACUAUGAA
env-1309  AAAUAAUUCAAUAAGUUAAUUGCCACCGGGUAAU.G.A.A.U.............GU......................CAGCAUUCG.AUUGCAUAUCGUUAGGUC.U.UAGAA.GAUAUGCC...GUC...GGAUGCUUUUUUGAUAACGGCUGUUUUAUAAGGAUGGACUAUGAA
env-1310  AAAUAAUUCAAUAAGUUAAUUGCCACCGGGUAAU.G.A.A.U.............GU......................CAGCAUUCG.AUUGCAUAUCGUUAGGUC.U.UAGAA.GAUAUGCC...GUC...GGAUGCUUUUUUGAUAACGGCUGUUUUAUAAGGAUGGACUAUGAA
env-1311  AUAACAGAUUUUAUAGUAACUGCCACCGGGUAGU.G.A.G.UG............CAU.....................AUGCAUUCG.UUUAUACAUCGUUAGGUC.U.UGGAA.GAUGUAUA...UGC...GGAUGCAUUUUGUUGCUUUAGGCAAAGCCGCUUUGUUCUACAGAG
env-1312  CACAGAAUCGAAUAUAUAACUGCCACCGGGCAGC.G.A.................AUGCA...................AAGCAUUCG.AUUAUACAUCGUUAGGUC.U.UUGAA.GAUGUAUA...GUC...GGAUGCUUUUUUGGAGAAGAAGAUAAGAAUGAAAAGAAUAAUAAC
env-1313  CGCAUAAUUGAAUAUAUAACUGCCACCGGGUAGC.G.A.................AUGCA...................GAGCAUUCG.AUUAUACAUCAUUAGGUC.U.UUGAA.GAUGUAUG...GUC...GGAUGCUUUUUUGGAGAAGAAAAUGAAAAGAAUACUAUCGGUAAU
Bma-2-1   UAUUUUUAUAAAAGUUAUUUUGCCACCGGGCAAA.G.G.G...............UGCGAA..................AAGCAUUCG.UCUGCACAUCGUUAGGUC.U.UUGAA.GAUGUGCG...AGC...GGAUGCUUCUUUUUUUGGAGGUGCUUGCAGUGAACAGAAA.....
env-1315  UAUUUUUAUAAAAGUUAUUUUGCCACCGGGCAAA.G.G.G...............UGCGAA..................AAGCAUUCG.UCUGCACAUCGUUAGGUC.U.UUGAA.GAUGUGCG...AGC...GGAUGCUUCUUUUUUUGGAGGUGCUUACAGUGAACAGAAA.....
Csp-4-1   UAUUUUUAUAAAAGUUAUUUUGCCACCGGGCAAA.G.G.G...............UGCGAA..................AAGCAUUCG.UCUGCACAUCGUUAGGUC.U.UUGAA.GAUGUGCG...AGC...GGAUGCUUCUUUUUUUGGAAGUGCUUACAGUGAACAGAAA.....
env-1316  UUAAAUAAAUAUAUAAUAACUGCCACCGGGUAGU.G.A.A.UG............UU......................UUGCAUUUG.UUUUCACAUCAUUAGGUC.U.AAGAA.GAUGUGAU...CAC...GGAUGCAUUUUUUUCAGGGAAUAAAAUGAAAAAACAGCUUAC...
env-1317  AUAAAAAUAAAUCAUUUUGCUGCCACCGGGUAGC.A.U.A.A.............A.......................ACAUUCG.CCAGCACAUCGUUAGGUC.U.UUGAA.GAUGUGCC...CGC...GAAUGUUUUUUUUCGGAGAAAUUUCAUGAACAAAUUCAAAUCCCU
env-1318  AUAAAAAUAAAUCAUUUUGCUGCCACCGGGUAGC.A.U.A.A.............A.......................ACAUUCG.CCAGCACAUCGUUAGGUC.U.UUGAA.GAUGUGCC...CGC...GAAUGUUUUUUUUCGGAGAAAUUUCAUGAACAAAUUCAAAUCCCU
env-1319  AUAAAAAUAAAUCAUUUUGCUGCCACCGGGUAGC.A.U.A.A.............A.......................ACAUUCG.CCAGCACAUCGUUAGGUC.U.UUGAA.GAUGUGCC...CGC...GAAUGUUUUUUUUCGGAGAAAUUUCAUGAACAAAUUCAAAUCCCU
env-1320  AUAAAAAUAAAUCAUUUUGCUGCCACCGGGUAGC.A.U.A.A.............A.......................ACAUUCG.CCAGCACAUCGUUAGGUC.U.UUGAA.GAUGUGCC...CGC...GAAUGUUUUUUUUCGGAGAAAUUUCAUGAACAAAUUCAAAUCCCU
env-1321  AUAAAAAUAAAUCAUUUUGCUGCCACCGGGUAGC.A.U.A.A.............A.......................ACAUUCG.CCAGCACAUCGUUAGGUC.U.UUGAA.GAUGUGCC...CGC...GAAUGUUUUUUUUCGGAGAAAUUUCAUGAACAAAUUCAAAUCCCU
env-1322  AUAAAAAUAAAUCAUUUUGCUGCCACCGGGUAGC.A.U.A.A.............A.......................ACAUUCG.CCAGCACAUCGUUAGGUC.U.UUGAA.GAUGUGCC...CGC...GAAUGUUUUUUUUCGGAGAAAUUUCAUGAACAAAUUCAAAUCCCU
env-1323  AUAAAAAUAAAUCAUUUUGCUGCCACCGGGUAGC.A.U.A.A.............A.......................ACAUUCG.CCAGCACAUCGUUAGGUC.U.UUGAA.GAUGUGCC...CGC...GAAUGUUUUUUUUCGGAGAAAUUUCAUGAACAAAUUCAAAUCCCU
env-1324  AUAAAAAUAAAUCAUUUUGCUGCCACCGGGUAGC.A.U.A.A.............A.......................ACAUUCG.CCAGCACAUCGUUAGGUC.U.UUGAA.GAUGUGCC...CGC...GAAUGUUUUUUUUCGGAGAAAUUUCAUGAACAAAUUCAAAUCCCU
env-1325  UGUUAUGAUAUUUUGUGUUCUGCCACCGGGUAGA.A.U.................CAUGCCGGAA..............AGGCAUUCG.UCUGCACAUCGUUAGGUC.U.UUGAA.GAUGUGUA...UAC...GGAUGCCUGUUUUAUUUCGGAGGUUCCCCAGGAAAAACAAAACA
env-1326  UUUGUAUAAAUAAAAUUAUCUGCCACCGGGUAGG.A.A.U.GU............UAA.....................AAGCAUUCA.CAUACGCAUCGAUAGGUC.U.UUGAA.GAUGUGUA...UAU...GGAUGCUUUUUUGUUUAAGGAGGAAACAUGAAAUCACUUACUGCA
env-1332  UUUGUAUAAAUAAAAUUAUCUGCCACCGGGUAGG.A.A.U.GU............UAA.....................AAGCAUUCA.CAUACGCAUCGAUAGGUC.U.UUGAA.GAUGUGUA...UAU...GGAUGCUUUUUUGUUUAAGGAGGAAAUAUGAAAUCACUUACUGCA
env-1334  GCAUAAGAUCAAAAAUUAUCUGCCACCGGGUAGG.A.A.U.GU............UGA.....................AAGCAUUCA.CAUACGCAUCGAUAGGUC.U.UUGAA.GAUGUGUA...UAU...GAAUGCUUUUUAUAUAUCACCGGCAGAUAAGGAGGACAUCAUGAA
env-1344  GCAUAAGAUCAAAAAUUAUCUGCCACCGGGUAGG.A.A.U.GU............UGA.....................AAGCAUUCA.CAUACGCAUCGAUAGGUC.U.UUGAA.GAUGUGUA...UAU...GAAUGCUUUUUAUAUAUCACCGGCAGAUAAGGAGGACAUCAUGAA
env-1345  GCAUAAGAUCAAAAAUUAUCUGCCACCGGGUAGG.A.A.U.GU............UGA.....................AAGCAUUCA.CAUACGCAUCGAUAGGUC.U.UUGAA.GAUGUGUA...UAU...GAAUGCUUUUUAUAUAUCACCGGCAGAUAAGGAGGACAUCAUGAA
env-1363  GCAUAAGAUCAAAAAUUAUCUGCCACCGGGUAGG.A.A.U.GU............UGA.....................AAGCAUUCA.CAUACGCAUCGAUAGGUC.U.UUGAA.GAUGUGUA...UAU...GAAUGCUUUUUAUAUAUCACCGGCAGAUAAGGAGGACAUCAUGAA
env-1364  GCAUAAGAUCAAAAAUUAUCUGCCACCGGGUAGG.A.A.U.GU............UGA.....................AAGCAUUCA.CAUACGCAUCGAUAGGUC.U.UUGAA.GAUGUGUA...UAU...GAAUGCUUUUUAUAUAUCACCGGCAGAUAAGGAGGACAUCAUGAA
env-1365  GCAUAAGAUCAAAAAUUAUCUGCCACCGGGUAGG.A.A.U.GU............UGA.....................AAGCAUUCA.CAUACGCAUCGAUAGGUC.U.UUGAA.GAUGUGUA...UAU...GAAUGCUUUUUAUAUAUCA..........................
env-1366  CGCAACAAAAUUAUGAUAACUGCCACCGGGUAGU.....................GUAAUGCUU...............UGGCAUUCG.UCUGCACAUCGUUAGGUC.U.UUGAA.GAUGUGCA...UAC...GGGUGCUUUUUUUCGGGGGUUUUGCCAUGUCUUCCAAACU.....
Mma-1-1   AACAAAAAAUAAAUAGGCGCUGCCACCGGGUAGC.A.A.U...............GCUGAUA.................GGGCAUUCG.UUUGCACAUCGUUAGGUC.U.UUGAA.GAUGUGCAG..GAC...GGAUGCUUGUUUUUUUGGAGGUUUUUUAUGGCGAUUUUUCAUUCA
env-1367  A.....AAAAAUAUAUGAGCUGCCACCGGGUAGC.....................AAUGCGAUUU..............AAGCAUUCG.UCGGCACAUCGUUAGGUC.U.UGGAA.GAUGUGCU...UGC...GGGUGCUUUUUUUGUUGCAGGAGGUAAAAAUGAAAAAACU.....
env-1371  A.....AAAAAUAUAUGAGCUGCCACCGGGUAGC.....................AAUGCGAUUU..............AAGCAUUCG.UCGGCACAUCGUUAGGUC.U.UGGAA.GAUGUGCU...UGC...GGGUGCUUUUUUUGUUGCAGGAGGUAAAAAUGAAAAAACUCGGAA
env-1372  A.....AAAAAUAUAUGAGCUGCCACCGGGUAGC.....................AAUGCGAUUU..............AAGCAUUCG.UCGGCACAUCGUUAGGUC.U.UGGAA.GAUGUGCU...UGC...GGGUGCUUUUUUUGUUGCAGGAGGUAAAAAUGAAAAAACU.....
env-1374  ......AAAAAUAUAUGAGCUGCCACCGGGUAGC.....................AAUGCGAUUU..............AAGCAUUCG.UCGGCACAUCGUUAGGUC.U.UGGAA.GAUGUGCU...UGC...GGGUGCUUUUUUUGUUGCAGGAGGUAAAAAUGAAAAAACUCGGAA
env-1375  A.....AAAAAUAUAUGAGCUGCCACCGGGUAGC.....................AAUGCGAUUU..............AAGCAUUCG.UCGGCACAUCGUUAGGUC.U.UGGAA.GAUGUGCU...UGC...GGGUGCUUUUUUUGUUGCAGGAGGUAAAAAUGAAAAAACUCGGAA
env-1377  A.....AAAAAUAUAUGAGCUGCCACCGGGUAGC.....................AAUGCGAUUU..............AAGCAUUCG.UCGGCACAUCGUUAGGUC.U.UGGAA.GAUGUGCU...UGC...GGGUGCUUUUUUUGUUGCAGGAGGUAAAAAUGAAAAAACUCGGAA
env-1378  C.....AAAAAUAUAUGAGCUGCCACCGGGUAGC.....................AAUGCGAUUU..............AAGCAUUCG.UCGGCACAUCGUUAGGUC.U.UGGAA.GAUGUGCU...UGC...GGGUGCUUUUUUUGUUGCAGGAGGUAAAAAUGAAAAAACU.....
env-1386  C.....AAAAAUAUAUGAGCUGCCACCGGGUAGC.....................AAUGCGAUUU..............AAGCAUUCG.UCGGCACAUCGUUAGGUC.U.UGGAA.GAUGUGCU...UGC...GGGUGCUUUUUUUGUUGCAGGAGGUAAAAAUGAAAAAACUCGGAA
env-1388  A.....AAAAAUAUAUGAGCUGCCACCGGGUAGC.....................AAUGCGAUUU..............AAGCAUUCG.UCGGCACAUCGUUAGGUC.U.UGGAA.GAUGUGCU...UGC...GGGUGCUUUUUUUGUUGCAGGAGGUAAAAAUGAAAAAACUCGGAA
env-1392  C.....AAAAAUAUAUGAGCUGCCACCGGGUAGC.....................AAUGCGAUUU..............AAGCAUUCG.UCGGCACAUCGUUAGGUC.U.UGGAA.GAUGUGCU...UGC...GGGUGCUUUUUUUGUUGCAGGAGGUAAAAAUGAAAAAACUCG...
env-1393  A.....AAAAAUAUAUGAGCUGCCACCGGGUAGC.....................AAUGCGAUUU..............AAGCAUUCG.UCGGCACAUCGUUAGGUC.U.UGGAA.GAUGUGCU...UGC...GGGUGCUUUUUUUGUUGCAGGAGGUAAAAAUGAAAAAACU.....
env-1399  C.....AAAAAUAUAUGAGCUGCCACCGGGUAGC.....................AAUGCGAUUU..............AAGCAUUCG.UCGGCACAUCGUUAGGUC.U.UGGAA.GAUGUGCU...UGC...GGGUGCUUUUUUUGUUGCAGGAGGUAAAAAUGAAAAAACU.....
env-1400  A.....AAAAAUAUAUGAGCUGCCACCGGGUAGC.....................AAUGCGAUUU..............AAGCAUUCG.UCGGCACAUCGUUAGGUC.U.UGGAA.GAUGUGCU...UGC...GGGUGCUUUUUUUGUUGCAGGAGGUAAAAAUGAAAAAACUCGUAA
env-1401  A.....AAAAAUAUAUGAGCUGCCACCGGGUAGC.....................AAUGCGAUUU..............AAGCAUUCG.UCGGCACAUCGUUAGGUC.U.UGGAA.GAUGUGCU...UGC...GGGUGCUUUUUUUGUUGCAGGAGGUAAAAAUGAAAAAACU.....
env-1402  UAUAAUACUGAAAAAUAAUCUGCCACCGGGUAGA.G.A.G.AU............GCUC....................AGGCAUCCG.UUUGCACAUCGUCAGGUC.A.CAGAA.GAUGUGUA...UGC...GGAUGCCUGUUUUUAUGGAGUGUUUUUAUGCCGUGUUUAAAAAAA
env-1403  GGAAAAAUCGAAUGACAAGCUGCCACCGGGUAGC.C.A.G.AU............GCGAAC..................AAGCAUCCG.UUUACACAUCGUUAGGUC.U.UUGCA.GAUGUGUA...CGC...GGAUGCUUUUUUUCGGAGAAAUUUAUUUUUAUGCUGAAAGAAAAA
env-1404  GGAAAAAUCGAAUGACAAGCUGCCACCGGGUAGC.C.A.G.AU............GCGAAC..................AAGCAUCCG.UUUACACAUCGUUAGGUC.U.UUGCA.GAUGUGUA...CGC...GGAUGCUUUUUUUCGGAGAAAUUUAUUUUUAUGCUGAAAGAAAAA
env-1405  UACUAUACCCCGAUACUUACUGCCACCGGGCAGG.C.A.A.UG............CAGAUA..................GAGCAUUCG.UUGACGCAUCGUUAGGUC.U.UUGAA.GAUGCGUC...CGC...GGAUGCUUUUUUGUUGGAAGGGAGUU..................
env-1406  UACUAUACCCCGAUACUUACUGCCACCGGGCAGG.C.A.A.UG............CAGAUA..................GAGCAUUCG.UUGACGCAUCGUUAGGUC.U.UUGAA.GAUGCGUC...CGC...GGAUGCUUUUUUGUUGGAAGGGAGUUUUUUCAUAUGUUUACCCAA
env-1407  AAGAAGAAUAAGAUGAUUUCUGCCACCGGGUAGA.G.A.A.UG............UAUCAA..................AAACAUUCG.UAUGUACAUCGUUAGGUC.A.UAGAA.GAUGUACA...GGC...GGAUGUUUUUUUGUUUGAAGGAGGAUAAAAAUGAAAAAUCCGUUU
env-1408  UAAUACAGAAAAAUAAUAACUGCCGCCGGGUAGA.G.A.G.UA............UU........................ACACUCG.AUUGCACAUCAUUAGGUC.U.UUGAA.GAUGUGCC...GUC...GGGUGUUUUUUUUCGGGAGAAAUAAUUAUGAAUGCAAAAAAGAAC
env-1409  UUAAAAAAUUUAUAAAGAAACUGCCACCGGGUAG.....................C.......................AAGCGUUUG.UAAGCAUAUCAUUAGGUC.U.UUGAA.GAUAUGCC...GAUC..AGGCGCUUAUUUUUUUUUGAAGGAGAAUAUAUAUGAAAAAAAUCA
env-1410  AUGUUAAUAUAGAUAAAAACUGCCAACGGGUAGU.G.A.A.UG............CGUA....................AAGCAUUUG.UUUGUACAUCGUUAGGUC.U.UUGAA.GAUGUGAA...UUC...AAAUGCUUUUUCUUUUAUAACAUUAAAAGGAGAAGAACCAUGUUU
env-1412  ACAGAAAACAGAAUAUAUACUGCCACCGGGUAGU.G.A.A.UG............CGAA....................AAGCAUUUG.UAAGCACAUCAUUAGGUC.U.UAGAA.GAUGUGUA...UAC...AAAUGCUUUUUUGUUUAGCCGAAAUUUGAAAGGAGUAUCUUAUGA
env-1413  ACAGAAAACAUAAUAUAUACUGCCACCGGGUAGU.G.A.A.UG............CGAA....................AAACAUUUG.UAAGCACAUCAUUAGGUC.U.UAGAA.GAUGUGUU...UAC...GGGUGCUUUUUUAUUUGUGGGAGUUUUUUAUGAUUUAUAUAGCAA
env-1414  AUACCAAUAGAAUAUGUUGCUGCCACCGGGUAGU.G.A.G...............CGA.....................AAGCUCUCG.UUUGCACAUCGUUAGGUC.U.UAGAA.GAUGUGUA...AGC...GAGGGCUUAUUUUUUUAUGUUUUGGAGGGCUUAUGAAGAAACUGAC
env-1415  CAUAUAUCAUAUAUAGAAACUGCCACCGGGUAG.....................AAGAAUGUUAGAA............AAGCAUUCG.UUUUCACAGCGUUAGGUC.U.UUGAA.GCUGUGAA....GC...GAAUGCUUUUUUGAUGGAGAAAAUAUGAAAUUAUUGCGUUAUUUU
env-1416  UACUGUAAUUAAAAAUAUUCUGCCACCGGGUAGA.A.A.A.GU............GUCA....................AAGCACACG.UAAACACAUCGUUAGGUC.U.UUGAA.GAUGUGUU...CAC...GGGUGCUUUUUUGUCGGAGGUUUUUAUGUUAAAGAAAGUAGUGAA
[gl-1-1   AAAAAAAUAAAAAGAGAAUCUGCCACCGGGUAGA.G.U.................AAUGCAAA................AAGCAUUCG.UUUGUACAUCAUUAGGUC.U.UAGUA.GAUGUGUG...UAC...GGAUGUUUCUUUUUUGGAGGAUUGUAUGUUAAAAUUAAAGCGGCU
env-1419  AGAUAGAUUAAAAAUAAAACUGCCACCGGGUAGU.A.A.G.AAUGUACCAAACAUU.UUU.........AAUGUUU....AAGCAUUCA.UAUACGUAUCGUUAGGUC.U.UUGAA.GAUGCGUA...UGU...GGAUGCUUUUUUAUUUGUUUUUCCGUCCCGCGCAGCGGGACAGAA
env-1421  AAAAAAAUAAAUACAAGUACUGCCACCGGGUAGG.A.A.U...............GCAAG...................GAGCAUUCG.UUUACACACCAUUAGGUC.U.UUGAA.GGUGUGUA...UGC...GGAUGCUUUUUUAGGAGGUCUUAUGAAUAAAAACAAAUUAGAAAC
env-1422  AAAAAAAUAAAUACAAGUACUGCCACCGGGUAGG.A.A.U...............GCAAG...................GAGCAUUCG.UUUACACACCAUUAGGUC.U.UUGAA.GGUGUGUA...UGC...GGAUGCUUUUUUAGGAGGUCUUAUGAAUAAAAACAAAUUAGAAAC
env-1423  AAAAAAAUAAAUACAAGUACUGCCACCGGGUAGG.A.A.U...............GCAAG...................GAGCAUUCG.UUUACACACCAUUAGGUC.U.UUGAA.GGUGUGUA...UGC...GGAUGCUUUUUUAGGAGGUCUUAUGAAUAAAAACAAAUUAGA...
env-1424  UGCUAAAGUAGUUAAAACUCUACCACCGGGUAGA.A.A.A.U.............GCGA....................AAGCAUUCG.UCAACACAUCAUUAGGUC.U.UAGAA.GAUGUGUU...CAC...GGGUGCUUAUUUUUUUGGAGGUUAUUAUGAAUACUAAGUUUAAGC
env-1425  ACCCUUUUACUAAAAAUCACUGCCACCGGGUAGG.A.A.A.U.............GCAU....................UUGCAUUCG.UCUGCACAUCGUUAGGUC.U.UGGAA.GAUGUGUU...CAC...GGGUGCUUUUAUUUUUGUUUCGGAGGAAAACGGUCAUGAAGUCCG
env-1427  AAAUAAUUCAAAUAUAAAACUGCCACCGGGUAGU.....................GAAUGUAA................ACGCAUUCG.UUUUCACAUCGUUAGGUC.U.UUGAA.GAUGUGAA...UAC...GGAUGCGUUUUUUCAGGGGUAUAUAUUAUAUGAAAACAAUUAACA
env-1428  UAUAAUAUGAAUAUAGAAACUGCCACCGGGUAGG.A.A.U...............GUGAUA..................GAGCAUUCG.CUGGCGCAUCGUUAGGUC.U.UGGAA.GAUGCGCA...CGC...GGGUGCUUUUUUGUUUUGAAAGGAGUAUCUGUUCAUGGGCGCCUU
env-1429  CGGAAAAAGGAAUACGCUUCUGCCACCGGGUAGA.A.AAU.G.............CGAUA...................AAGCAUUUG.UGUGCACAUCGUUAGGUC.U.UAGAA.GAUGGGCA...CGC...GGAUGUUUAUUUUUUUGUUGUUCAGGAGGUUACUAUGUUGAGCAG
env-1431  CGGAAAAAGGAAUACGCUUCUGCCACCGGGUAGA.A.AAU.G.............CGAUA...................AAGCAUUUG.UGUGCACAUCGUUAGGUC.U.UAGAA.GAUGGGCA...CGC...GGAUGUUUAUUUUUUUGUUGUUCAGGAGGUUACUAUGUUGAGCAG
env-1434  AGAAAACCCAUAUGGAAAGCUGCCACCGGGUAGC.....................AGAGAUGCAA..............GAGCAUCCG.UUUACACAUCAGUAGGUC.U.UAGAA.GAUGUGUA...UAC...GGAUGCUUAUUUUUUUGGAGAGGUACUUUUUAUGAAAAGGAA...
env-1435  UCUGUCAUACGGAUGUAAUCUGCCACCGGGUAGA.A.A.A.UG.....UUCGGCGAAAGAAUGA...UUGCCG.....AAGCAUUUG.CCUGCACAUCGUUAGGUC.U.UUGAA.GAUGUGCG...AGC...AAAUGCUUUUUUGUUUGAAAGGGAUGGUGCAUUAUGACAAAAUAU
env-1438  ACAAUCAACUCAAUUAUAACUGCCACCGGGUAGA.A.A.A.UG............CAG.....................AAGCAUUUG.UCCGCACAUCGUUAGGUC.U.UUGAA.GAUGUGCC...CGC...AAAUGCUUAUUUUUAUAUAAAACCGCAUAUGUAUUGCGAAAGAAA
env-1439  GUAUAACUGAAUCUGAAUACUGCCACCGGGUAGU.G.A.AUCG............CUGAUA..................CAGCAUUCG.CCUGCACAUCGUUAGGUC.U.UAGCA.GAUGUGCG...UGC...GGAUGCUGUUUUGUUUGGAGGAAUACAUGGAACAGAAGCUGCGUU
env-1440  GUAGAAAAUUUUAAAUUUACUGCCACCGGGUAGG.A.A.G...............CGUU....................ACGCAUUCG.UGUACAUAUCGUUAGGUC.U.UAGAA.GAUAUGUA...CGUC..GGGUGCUUAUUUUUUUGGAGGUAUAAAAUGGGAUAUAUCACAAAA

          ........................<<<...............................................................................>>>.......................................................................
          ........................20................................................................................02.......................................................................
          ............<<<<<<<<<<<....>>>>>>.>.>.>.>>......<<<<<<<<...........>>>>>>>>....<<<<<<<<<.<<<<<<<<<<................>>>>>>>>...>>>...>>>>>>>>............................
          ............22222222222....222222.2.2.2.22........1111.............1111......222222222.2222222222................22222222...222...22222222............................
          ••••••••••••••••••YUGCCRCCGGGUAR•-○-○-○-○--------------○○○--------------------○○R•••Y••-••••YRY•YCRUUAGGYC-•-U•GAA-GR•RYR••---•••---•R•••YUUUUUUY••••••••••••••••••••••••••••••
          5'                                                                                                                                    3'
```

```
Cbo-3-1   AAUAAAAGAAGAACAAAAACUGCCACCGGGUAGG.G.A.U.................GCUAU...................AAGCAUUCA.UGUGGACAUCGUUAGGUC.A.UAGAA.GAUGUACA...UAU...GGAUGCUUGUUUUUUACAACAGCAAUAAGUUUUAUGAAGGAGAAA
env-1441  AAAUAACAUAAAAAAUAGACUGCCACCGGGUAGU.A.G.U.U...............AAAUGUGA................AAGCAUUUG.UUUGCACAUCGUUAGGUC.U.UAGAA.GAUGUGUU...UGC...AGAUGCUUGUUUUUUUGAGGAGAUUGAUUUUAUGAAUCAAAAGAA
env-1442  AAAUAACAUAAAAAAUAGACUGCCACCGGGUAGU.A.G.U.U...............AAAUGUGA................AAGCAUUUG.UUUGCACAUCGUUAGGUC.U.UAGAA.GAUGUGUU...UGC...AGAUGCUUGUUUUUUUGAGGAGAUUGAUUUUAUGAAUCAAAA...
env-1443  GAAUAUGUCAGUAAAUAAACUGCCACCGGGCAGU.A.G.A.U...............GCUU....................AAGCAUUUG.UGUGCAUAUCGUUAGGUC.U.UUGAA.GAUAUGCAC..UGC...AAAUGCUUAUUUUUUUGGAGGACGACAGAUGAAAUACUUUACAAA
env-1444  UCAGUAAUUGAAAAUUCAACUGCCGCCGGGCAGU.A.A.A.................UGCUU...................AAGCAUUUG.UGUGUAUAUCGUUAGGUC.U.UUGAA.GAUAUGCAC..UGC...AAAUGCUUAUUUUUUUGGAGGACAACAGAUGAAAUAUUUUACAAA
env-1445  UCAGUAAUUGAAAAUUCAACUGCCGCCGGGCAGU.A.A.A.................UGCUU...................AAGCAUUUG.UGUGUAUAUCGUUAGGUC.U.UUGAA.GAUAUGCAC..UGC...AAAUGCUUAUUUUUUUGGAGGACGACAGAUGAAAUAUUUUACAAA
env-1446  AAAAAUUGAAAAUUAUGAACUGCCACCGGGUAGU.G.U.A.UG..............CGUU....................CAGCAUUCG.UUCGCACAUCGUUAGGUC.U.UUGAA.GAUGUGCC...UGC...GGAUGUUUGUUUUUAGGGGACAGUAUAUGAGAGUAAAGAAAAUC
env-1447  AUAUGCUAUAAUAUGAUAACUGCCACCGGGUAGA.A.G.A.UG..............CUUA....................AAGCGUCCG.UUUACACAUCGUUAGGUC.U.UUGAA.GAUGUGUA...CGC...GGGCGUUUUUUGAAGGGAUAUUUAUAUGCAAAAAAGGAUACUGA
env-1448  AUAUGCUAUAAUAUGAUAACUGCCACCGGGUAGA.A.G.A.UG..............CUUA....................AAGCGUCCG.UUUACACAUCGUUAGGUC.U.UUGAA.GAUGUGUA...CGC...GGGCGUUUUUUGAAGGGAUAUUUAUAUGCAAAAAAGGAUACUGA
env-1449  AUAACUGUAAUAGAUUUAACUGCCACCGGGUAGU.A.A.A.AU..............GCUU....................AUGCAUUCG.UAUACACAUCGUUAGGUC.A.UAGAA.GAUGUGUG...UCC...GGGUGCAUUUUUAAUUUGAUUAUUAUAAAAGCGAGGUAAAUGUCG
env-1450  AUAACUGUAAUAGAUUUAACUGCCACCGGGUAGU.A.A.A.AU..............GCUU....................AUGCAUUCG.UAUACACAUCGUUAGGUC.A.UAGAA.GAUGUGUG...UCC...GGGUGCAUUUUUAAUUUGAUUAUUAUAAAAGCGAGGUAAAUGUCG
env-1452  AUAACUGUAAUAGAUUUAACUGCCACCGGGUAGU.A.A.A.AU..............GCUU....................AUGCAUUCG.UAUACACAUCGUUAGGUC.A.UAGAA.GAUGUGUG...UCC...GGGUGCAUUUUUAAUUUGAUUAUUAUAAAAGCGAGGUAAAUGUCG
env-1454  AAAUAUAAAAAAAUAUUUUACUGCCACCGGGUAGU.U.G.U.A...............AUUAC...................AAGCAUUCA.UUUACACGUCGUUAGGUC.U.UAGAA.GAUGUGUAA..UAU...GAAUGCUUUUUUAUUUAGGAGGAAAUAAUAUGAAACUAUUCAGGC
env-1455  GAACAAAAUGAUAUAGCAACUGCCACCGGGUAGU.G.U.G.AA..............AUGUA...................UUGCAUUCA.CUUGCACAUCGUUAGGUC.U.UUGAA.GAUGUGUCA..UGU...GGAUGCUUAUUUUUUUGAAAGGUAUGAUAUGAAGUGCAAAACCCU
env-1456  GCAAAACUGAAUUUUGUAUCUGCCACCGGGUAGA.G.A.A.UG..............CCU.....................CGGCAUUCG.AUCACGCAUCGUUAGGUC.U.UUGAA.GAUGCGCG...GUC...GGGUGCCUUUUUUGAACGGGAAAGGAGAAAGAUCAUCAUGCUGCG
env-1457  CGCAAUUUGAAUAUCGUUUCUGCCACCGGGUAGA.G.A.A.UG..............CG......................AAGCAUUCG.AUCACACAUCGUUAGGUC.U.UUGAA.GAUGUGGG...GUC...GGGUGCUUUUUUGAAUACGGAAAGGGAAUGUUUUCUAUGCUGCGC
env-1458  CGCAAUUUGAAUAUCGUUUCUGCCACCGGGUAGA.G.A.A.UG..............CG......................AAGCAUUCG.AUCACACAUCGUUAGGUC.U.UUGAA.GAUGUGGG...GUC...GGGUGCUUUUUUGAAUACGGAAAGGGAAUGUUUUCUAUGCUGCGC
env-1459  CGCAAUUUGAAUAUCGUUUCUGCCACCGGGUAGA.G.A.A.UG..............CG......................AAGCAUUCG.AUCACACAUCGUUAGGUC.U.UUGAA.GAUGUGGG...GUC...GGGUGCUUUUUUGAAUACGGAAAGGGAAUGUUUUCUAUGCUGCGC
env-1460  CGCAAUUUGAAUAUCGUUUCUGCCACCGGGUAGA.G.A.A.UG..............CG......................AAGCAUUCG.AUCACACAUCGUUAGGUC.U.UUGAA.GAUGUGGG...GUC...GGGUGCUUUUUUGAAUACGGAAAGGAAAUGUUUUCUAUGCUGCGC
env-1461  CGUAAUUUGAAUAUCGUUUCUGCCACCGGGUAGA.G.A.A.UG..............CG......................AAGCAUUCG.AUCGCACAUCGUUAGGUC.U.UUGAA.GAUGUGGG...GUC...GGGUGCUUUUUUUGAAGUGGAAAAGGAGAAAAAUUUUAUGCU...
env-1462  AUAAAUAUAAAAAAUAAAACUGCCACCGGGUAGU.......................AGAUGCUAA...............AAGCAUCCG.UUUGUACAUCGUUAGGUC.U.UAGAA.GAUGUACU...UUC...GGAUGCUUAUUUUAUGGAGGAUUAAUGAAAUACAUAUUAGAUUAU
env-1463  AUAAAAUUAAAUAAAAUAACUGCCACCGGGUAGU.A.A...................AAAUGCUUAA..............AAGCAUUCG.UGUGUACAUCGUUAGGUC.U.UUGAA.GAUGUGCAU..UAC...GAAUGCUUUUUUGAGGUUAUAAAAUGAAAUCAAAAUCCUAUUUUA
env-1465  UUAAAAUAGAAUACAGAUACUGCCACCGGGUAGU.G.A.A.UG..............AA......................AAGCAUUCG.UUUGCACAUCGUUAGGUC.U.UUGAA.GAUGUGCA....GC...GAAUGCUUUUUUAAACAACAGGGAAAGAAUUUGCUUAUGAAAAGA
env-1466  AAACAAAUACAAAAAUAAACUGCCACCGGGUAG........................GAAUGCAAA...............AAGCAUUCA.UCUGUACAUCGAUAGGUC.U.UUGAA.GAUGUGCA...AAU...GAAUGCUUUUUUACAUCCUUGCCAUUGUGCAUAAGGAGGAGUCAC
env-1467  AAACAAAUACAAAAAUAAACUGCCACCGGGUAG........................GAAUGCAAA...............AAGCAUUCA.UCUGUACAUCGAUAGGUC.U.UUGAA.GAUGUGCA...AAU...GAAUGCUUUUUUACAUCCUUGCCAUUGUGCAUAAGGAGGAGUCAC
env-1468  AAACAAAUACAAAAAUAAACUGCCACCGGGUAG........................GAAUGCAAA...............AAGCAUUCA.UCUGUACAUCGAUAGGUC.U.UUGAA.GAUGUGCA...AAU...GAAUGCUUUUUUACAUCCUUGCCAUUGUGCAUAAGGAGGAGUCAG
env-1469  GUUUAUUCAGAAUAAGUUGCUGCCACCGGGUAGU.G.A.A.U...............GCUA....................UAGCAUUCG.UUCUCGCAUCAUUAGGUC.U.UAGAA.GAUGCGAU...UAC...GGAUGCUUUUAUUUUGGAGGCUUUAAUGAGAAUCAACAGAAUAAC
env-1471  AAUUAUUUCAAAAUGAAUACUGCCACCGGGUAGU.G.A.A.UG..............CAU.....................CGGCAUUCG.CUUUCGCAUCGUUAGGUC.U.UAGAA.GAUGCGAA...UGC...GGAUGCUUUUGUUUUGGGAGAGUUUAUGAGCAGGAAGAUCACUUC
env-1473  CCGUAGCUCAAUAUUCAAUCUGCCACCGGGUAGA.G.A.U.G...............CUU.....................CGGCAUCCG.UAAACACAUCGUUAGGUC.U.UUGAA.GAUGUGUU...CGC...GGAUGCUUUUUUCUUGAAAAACUCCGCGGCACGGAUAAAUCGGAG
env-1474  CCGUAGCUCAAUAUUCAAUCUGCCACCGGGUAGA.G.A.U.G...............CUU.....................CGGCAUCCG.UAAACACAUCGUUAGGUC.U.UUGAA.GAUGUGUU...CGC...GGAUGCUUUUUUCUUGAAAAACUCCGCGGCACGGAUAAAUCGGAG
env-1475  UACAAACAAUACAAUAAUUCUGCCACCGGGUAGA.G.A.U.G...............CUU.....................CGGCAUCCG.UAAACACAUCGUUAGGUC.U.UUGAA.GAUGUGUU...CGC...GCAUGCUUUUUUCUUGAAAAAUUCCGCGGCGCGGAUAAAUCGGAG
env-1476  ACAAAAUAAAUAUUAAAAACUGCCACCGGGUAGG.A.A.U.................GCAUU...................GAGCAUUCG.UUUACACAUCGUUAGGUC.U.AAGUA.GAUGUGUA...UGC...GGAUGCUUAUUUUUUGGAGGUUAAAGUUCUAUGAAUCGAUUCAGA
env-1477  AAUCGAAAAAUAAAAAAGCCUGCCACCGGGCAGG.A.A.U.................GCGCA...................AGGCAUUCG.UAUACACAUCGUUAGGUC.U.UUGAA.GAUGUGUA...CGC...GGAUGCCUUUUUUGGAGGGAAAAAUCAUGCAGAAGAUGAAGAAAA
env-1478  CAUCUACCCCAGAUUGUAACUGCCACCGGGUAGU.G.A.A.UG..............CGA.....................UUGCAUUCG.UUUUCACAUCGUUAGGUC.U.UAGAA.GAUGUGAA...UGC...GGGUGCUAUUUUUUUGAGCGAGGUUUUUCCAUGAAGAUCCU...
env-1479  AUUUGGUAAAAGAAUUAAUCUGCCACCGGGUAGA.A.G.A.................UGCAUA..................AAGCAUCCG.UUUACACAUCGUAAGGUC.U.UAGAA.GAUGUGUA...UGC...GGGUGCUUUUUUCAGGAGAUUAUCAUGAAUAAAAUAAUAAGAUAU
env-1480  GCCGCAAACAAUAAAAUGUCUGCCACCGGGUAGA.A.A.A.U...............GCUUA...................AAGCAUUCG.UUUGCACAUCGUUAGGUC.U.UUGAA.GAUGUGUU...UGC...GAAUGCUUUUUUGAUUGUGAUUUUGAAGGGCGGUUAAUUAUAAUU
env-1481  UAUGAUUUCAUAAGAUAAGCUGCCACCGGGUAGC.A.G.A.AU..............GCUGAUU.................GUGCAUUCG.UUUACACAUCGUUAGGCC.U.UUGAA.GAUGUGUAU..AAC...GGGUGCUUCUUUUUAUUUAUACGAAGUAAAGGAGCGGUAUUGCUA
env-1482  CAGUUUGUAACAUAAAAAACUACCACCGGGUAGU.G.A.A.U...............GCAA....................GGGCAUUCG.UUUUCACAUCGUUAGGUC.A.UAGAA.GAUGUGAG...AGC...GGAUGCUUUUUUGGAGUGAGAGAGGAUAUGAAGACUCUGAGAGCG
env-1483  GAUUUUGAAAUUUAAAACACUGCCACCGGGUAG.................................................AAGCAUUCG.CUUGCACAUCGUUAGGUC.A.UAGAA.GAUGUGCG...GAC...GGAUGCUUAUUUUUUAUUUUUAAGGAGGACAACAUGAGACACUAU
env-1484  AAUAAUACCAAUUUAGAAACUGCCACCGGGUAGU.......................AGAAUGCA................GAGCAUUCG.UUUACACAUCGUUAGGUC.U.UGGAA.GAUGUGUA...GAC...GGGUGCUUAUUUUUAAUGCAAAAGGAAAUCCCGCCGGACA.....
env-1485  CAAUACACUUAAUAAAAAACUACCACCGGGUAGU.G.A.U.................GAUGCGA.................AAGCAUCCG.UAUACACAUCGUUAGGUC.U.UUGAA.GAUGUGUA...CAC...GGGUGCUUUGUUUUUUGGAGGCAUUAUGAGAAAAACAAAGCU...
env-1486  CGUAAAUGAAGAAUAAAUUCUGCCACCGGGCAGA.G.A.A.UG..............UUGAAAU.................AAACAUUCG.UUUACACAUCGUUAGGUC.U.UUGAA.GAUGUGCA...GGC...GAAUGUUUUUUGUUUGAAAGGAGAACGAAAAUGAAAAAUCCGUUU
env-1487  CAAUUAAAAUAAUAAACAACUGCCACCGGGUAGU.U.U.A.U...............GCCAA...................AAGCAUUCG.UACGCACAUCGUUAGGUC.U.UAGAA.GAUGUGCAG..AGC...GGGUGCUUGUUUUUUUGGAGGUUAAGCCAAAUGAAAAUGCACAAU
env-1488  CAAUUAAAAUAAUAAACAACUGCCACCGGGUAGU.U.U.A.U...............GCCAA...................AAGCAUUCG.UACGCACAUCGUUAGGUC.U.UAGAA.GAUGUGCAG..AGC...GGGUGCUUGUUUUUUUGGAGGUUAAGCCAAAUGAAAAUGCACAAU
env-1490  AAUAAAAGAAUAAAUGAUUCUGCCACCGGGUAGG.A.....................GAUGCAUUAUA.............AAGCAUCCG.UUUACACAUCGUUAGGUC.U.UUGAA.GAUGUGUA...UGC...GGAUGCUUUUUUUCGCGAGGAGAGAAACUGUUGUGGGGGUGCUCC
env-1491  UAAUACCACCAUAAUAAUACUGCCACCGGGCAGC.G.G.A.AU..............GAA.....................AAACAUUCG.UUUACACAUCGUUAGGUC.U.UUGAA.GAUGUGUA...UGC...GGGUGUUUUUUUGAGAAAAACAAGGUAAAAAUCAAAAGAUUCA
env-1492  UAAUACCACCAUAAUAAUACUGCCACCGGGCAGC.G.G.A.AU..............GAA.....................AAACAUUCG.UUUACACAUCGUUAGGUC.U.UUGAA.GAUGUGUA...UGC...GGGUGUUUUUUUGAGAAAAACAAGGUAAAAAUCAAAAGAUUCA
env-1493  UAAUACCACCAUAAUAAUACUGCCACCGGGCAGC.G.G.A.AU..............GAA.....................AAACAUUCG.UUUACACAUCGUUAGGUC.U.UUGAA.GAUGUGUA...UGC...GGGUGUUUUUUUGAGAAAAACAAGGUAAAAAUCAAAAGAUUCA
env-1494  AUAAUACCGAAUUAUAGUACUGCCACCGGGUAGG.G.....................AAUGUUA.................UAGCAUUCG.UUUUCGCAUCGUUAGGUC.U.UAGAA.GAUGCGGA...UUC...GGGUGCUUUAUUUUUGGAGGUGUUAAUGAACAAGAGUCAGGAGG
env-1495  AUAAUACCGAAUUAUAGUACUGCCACCGGGUAGG.G.....................AAUGUUA.................UAGCAUUCG.UUUUCGCAUCGUUAGGUC.U.UAGAA.GAUGCGGA...UUC...GGGUGCUUUAUUUUUGGAGGUGUUAAUGAACAAGAGUCAGGAGG
env-1496  ACAGCAAAAAUAAAUAGUACUGCCACCGGGUAGG.G.....................AAUGUUA.................AAGCAUUCG.UUUACGCAUCGUUAGGUC.U.UUGAA.GAUGCGUA...UUC...GGGUGCUUUUUUGGAGGUAAAAAGGUGAAAAACAAUCAAUAUUC
env-1497  AAAUAAAAACAGAAUAAUACUGCCACCGGGUAGA.G.....................AAUGUUA.................AAGCAUUCG.UUUACGCAUCGUUAGGUC.U.UUGAA.GAUGCGUA...UGC...GGGUGCUUUUUUUGGAGGUAUAAAUAUGAAAAACAAUCAAUAUU
env-1498  AAAUAAAAACAGAAUAAUACUGCCACCGGGUAGA.G.....................AAUGUUA.................AAGCAUUCG.UUUACGCAUCGUUAGGUC.U.UUGAA.GAUGCGUA...UGC...GGGUGCUUUUUUUGGAGGUAUAAAUAUGAAAAACAAUCAAUAUU
env-1499  AAAUAAAAACAGAAUAAUACUGCCACCGGGUAGA.G.....................AAUGUUA.................AAGCAUUCG.UUUACGCAUCGUUAGGUC.U.UUGAA.GAUGCGUA...UGC...GGGUGCUUUUUUUGGAGGUAUAAAUAUGAAAAACAAUCAAUAUU
env-1501  UAAUUUAAUAUGGUUAUUACUGCCACCGGGUAGU.A.G.G.AU..............GCA.....................AAGCAUUCG.UUUACACAUCGACAGGUC.U.UAGAA.GAUGUGUA...UGC...GGGUGCUUAAUUUUUUUUGGAGGUUUUCAAAUGAAAAGUGUAAA
env-1502  UAAUUUAAUAUGGUUAUUACUGCCACCGGGUAGU.A.G.G.AU..............GCA.....................AAGCAUUCG.UUUACACAUCGACAGGUC.U.UAGAA.GAUGUGUA...UGC...GGGUGCUUAAUUUUUUUUGGAGGUUUUCAAAUGAAAAGUGUAAA
env-1503  UAUCAUAUAAAAAUUAAAGCUGCCACCGGGUAGC.A.G.A.A...............UGCG....................AAGCAUUCG.UUUGCACAUCGUCAGGUC.U.UAGAA.GAUGUGCA...UGC...GGGUGCUUCUUUGUUUUUGGAGGAAUCCAUGCUAAAGAAACUGAC
env-1504  AUGUGAUAUAAUGUAGUUACUGCCACCGGGUAGA.A.A.U.................GCGAGA..................AAGCAUUUG.UUCACACAUCGUCAGGUC.U.UAGAA.GAUGUGUG...CGC...AAAUGCUUUUUUUAAGG..............................
env-1505  GAGAUAACUGAAUAUAUUACUGCCACCGGGUAGA.A.....................UAUGCGAA................AAGCAUUUG.UUCACGCAUCGUUAGGUC.U.UUGAA.GAUGUGUG...CGC...AGAUGCUUUCUUUUUUGGAGGAUAAAUAUGUUCAAACAAAUCAGA
env-1506  AAAGAUAUAAAGAAGUAAGCUGCCACCGGGUAGU.G.A.A.AU..............GCAAG...................AAGCAUUCG.UUUACACAUCGUUAGGUC.U.UAGAA.GAUGUGUA...UGC...GGAUGCUUUUCUUAACUUUUGGAGGUUAUUUGCAAGUGAGUUAUG
env-1509  AAAGAUAUAAAGAAGUAAGCUGCCACCGGGUAGU.G.A.A.AU..............GCAAG...................AAGCAUUCG.UUUACACAUCGUUAGGUC.U.UAGAA.GAUGUGUA...UGC...GGAUGCUUUUCUUAACUUUUGGAGGUUAUUUGCAAGUGAGUUAUG
env-1510  UUUAAAAAACAAUAUAUAGCUGCUACCGAGUAGC.U.G.A.AU..............GCA.....................AAGCAUUCG.UUUACACAUCGUUAGGUC.U.UAGAA.GAUGUGUA...UGC...GAAUGCUUUUUUAUCGGCUAUGAUUUAACAUUCAUAGCAUAUCC
env-1511  UUUAAAAAACAAUAUAUAGCUGCUACCGAGUAGC.U.G.A.AU..............GCA.....................AAGCAUUCG.UUUACACAUCGUUAGGUC.U.UAGAA.GAUGUGUA...UGC...GAAUGCUUUUUUAUCGGCUAUGAUUUAACAUUCAUAGCAUAUCC
env-1512  UUUAAAAAACAAUAUAUAGCUGCUACCGAGUAGC.U.G.A.AU..............GCA.....................AAGCAUUCG.UUUACACAUCGUUAGGUC.U.UAGAA.GAUGUGUA...UGC...GAAUGCUUUUUUAUCGGCUAUGAUUUAACAUUCAUAGCAUAUCC
env-1513  UUUAAAAAACAAUAUAUAGCUGCUACCGAGUAGC.U.G.A.AU..............GCA.....................AAGCAUUCG.UUUACACAUCGUUAGGUC.U.UAGAA.GAUGUGUA...UGC...GAAUGCUUUUUUAUCGGCUAUGAUUUAACAUUCAUAGCAUA...
env-1514  CUAUAUAAGUAGAAUAUAACUGCCACCGGGUAGU.G.U.A.AU..............GCUU....................UGGCAUUCG.UUUACACAUCGUUAGGUC.U.UAGAA.GAUGUGUA...UGC...GGAUGCUUAUUUUUUUGGAGGUUUUAUGAAAAUAGUUUCUAAGUA
env-1516  UACUACGCGAUAUAUGAAACUGCUACCGAGUAGU.A.G.A.AU..............GCA.....................AAGCAUUCG.UUUACACAUCAUUAGGUC.U.UAGAA.GAUGUGUA...UGC...GGAUGUUUGUUUUUUUCGGAGGCUAUGGGUUAUGUUAAACAAACU
env-1517  GCAAUUAUCGAAUAUGGUACUGCUACCGAGUAGU.A.G.A.AU..............GCA.....................GAGCAUUCG.UUUACACAUCGUUAGGUC.U.UAGAA.GAUGUGUA...UGC...GGAUGCUUAUUUUUUUGGAGGUUGGUUUUUGUGAAUAAUAUCAAA
env-1518  ACAUCAUUUAAUAUUAUUACUGCCACCGGGUAGU.A.G.A.A...............UGCA....................AAGCAUUCG.UUUACACAUCGUCAGGUC.U.UAGAA.GAUGUGUA...UAC...GGGUGCUUAUUUUUUGGAGGUUUUUUAAUGAAAGGCGUAAAAAGG
env-1520  GUUACAAACAAUAAAUUUACUGCUACCGAGUAGU.A.G.A.AU..............GCC.....................AAGCAUUCG.UUUACACAUCGUCAGGUC.U.UAGAA.GAUGUGUA...UGC...GGGUGCUUAUUUUUUUGGAGGUUUUAUUAUGAAAAAUGUAAAAAG
env-1521  CUUUCAAAGAAUAUUUUAACUGCUACCGAGUAGU.U.G.A.A...............UGCA....................AAGCAUUCG.UUUACACAUCGUUAGGUC.U.UAGAA.GAUGUGUA...UGC...GGAUGCUUAUUUUUUUAUAAGGGGAUUAUCUUAUGCUAAAAAAAC
env-1523  AUCAUAUUUAAAUAUAAAACUGCCACCGGGUAGU.A.A.A.AU..............GCGA....................AAGCAUUCG.UAUGCACAUCGUUAGGUC.A.UUGAA.GAUGUGUA...CAC...GGGUGCUUUAUUUUUUGGAGGUUACAUGAGCAAACAAUACUU...
env-1524  AAUAAAUGUAAAAAAUUAACUGCCACCGGGUAGG.A.A.A.U...............GCAAA...................AAGCAUUCG.UUUGUGCGUCGUUAGGUC.U.UUGAA.GACGCACG...CAC...GGAUGCUUUUUUUGAGGAGAGAACCUAUGACAAAAAUAUUUCGUU
env-1525  AAUAAAUAAUAAGAAAUAACUGCCACCGGGUAGU.G.A.A.U...............GU......................AAGCAUUCG.UUUGUGCAUCGUUAGGUC.U.UUGAA.GAUGCACU....GC...GGAUGCUUAUUUUUAUGAGGUGAAAAUAUGCAAAAAAUAAAAUCC
env-1529  AAUCAUCAAAGUCAAAUAACUGCCACCGGGUAGU.G.A.A.U...............GCA.....................UUGCAUUCG.UUUUCGCAUCGUUAGGUC.U.UUGAA.GAUGCGAA...UAC...GGGUGCUUAUUUUUUUGAAGGUGAGGCUUCAUAGUGAAAAGAAGG
env-1530  AGUAAACUUUAGUGGUUAACUGCCACCGGGUAGU.A.G.A.U...............GCAA....................UAGCAUCCG.UUUACACAUCGUUAGGUC.U.UUGAA.GAUGUGUG....C...GGGUGUUUAUUUUUUUGGAGGUUUUGCAUUUAUGUUCAGAAUAAA
env-1532  UAAUACCGACAAUAUAUAACUGCCACCGGGUAGU.A.U.G.AU..............GCGAAUA.................AAGCAUCCG.UUUACACAUCGUUAGGUC.A.UUGAA.GAUGUGUA...AAC...GGAUGUUUAUUUUUUUGGAGGUUUAUUAUGAAAAAUCCGCUUAAA
env-1535  UAUAAUCGAAAAAAAUAAACUGCCACCGGGUAGU.G.C.A.U...............GCGA....................GAGCAUCCG.UUUACACAUCGUUAGGUC.U.UGGAA.GAUGUGUA...CGC...GGAUGCUUAUUUUUUGGAGGAUGUCUGUAUGAACAAAGUCAAAUC
env-1536  AAACAAUUAAAUGAAAAUACUGCCACCGGGUAGU.G.A.A.U...............GCC.....................AAGCAUUCG.AUUAUACAUCGUUAGGUC.U.UAGAA.GAUGUAUG...GUC...GGAUGCUUGUUUUUUGGAGGUAUCAUGAAAAAGACAGAAUGGUUA
env-1537  AAACAAUUAAAUGAAAAUACUGCCACCGGGUAGU.G.A.A.U...............GCC.....................AAGCAUUCG.AUUAUACAUCGUUAGGUC.U.UAGAA.GAUGUAUG...GUC...GGAUGCUUGUUUUUUGGAGGUAUCAUGAAAAAGACAGAAUGGUUA
env-1538  GCAAAAUUAAAUAAAAAUCCUGCCACCGGGUAGU.G.A.A.U...............GUU.....................AAGCAUUCG.AUUAUACAUCAUUAGGUC.U.UAGAA.GAUGUAUA...UUC...GGAUGCUUAUUUUUUUGGAGGUACUAUGAAGAAAACAGAGUGUUU

          ........................<<<.............................................................................>>>........................................................................
          ........................20..............................................................................02.........................................................................
          .............<<<<<<<<<<<....>>>>>>.>.>.>.>>......<<<<<<<<...........>>>>>>>>.....<<<<<<<<<.<<<<<<<<<<.................>>>>>>>>...>>>...>>>>>>>>....................................
          .............22222222222....222222.2.2.2.22........1111.............1111.......222222222.2222222222.................22222222...222...22222222....................................
          •••••••••••••••••••YUGCCRCCGGGUAR•-•-•-○-○---------------•○○--------------------••R•••Y••-••••YRY•YCRUUAGGYC-•-U•GAA-GR•RYR••---•••---•R•••YUUUUUUY••••••••••••••••••••••••••••••
          5′ ———————————————————————————————————————————————————————————————————————————————————————————————————————————————————————————————————————————————————————————————————— 3′
```

```
env-1539  AAGAAUAAACUUAUAAAAACUGCCACCGGGUAGA.A.A.U................GCAAAA................AAGCAUUUG.UCUGCACAUCGUUAGGUC.A.UUGAA.GAUGUGUU...UGC...AAAUGCUUAUUUUUUUAGGAGGUAAACAUGAAAUAUUUUACAAAA
env-1540  AGUUGAUACAGAAUAAACGCUGCCACCGGGUAGC.A.G.A.UG.............CACA..................AAGCAUCCA.UUUACAUAUCGUUAGGUC.U.UAGAA.GAUGUGUA...UGC...GGAUGCUAAUUUUUUGGAGGUAAAUAUGGCGAAAAUUAGGAAUUA
env-1541  AGUUGAUAAAGAAUAAGCACUGCCACCGGGUAGU.A.G.A.UG.............CGUA..................AAGCAUUCG.ACUGUACAUCGUUAGGUC.U.UUGAA.GAUGUAUA...UUC...GGAUGCUUAUUUUUUUGGAGGUUAUUAUGAAAGAGUUUAAGGACU
env-1542  AGUUGAUAAAGAAUAAGCACUGCCACCGGGUAGU.A.G.A.UG.............CGUA..................AAGCAUUCG.ACUGUACAUCGUUAGGUC.U.UUGAA.GAUGUAUA...UUC...GGAUGCUUAUUUUUUUGGAGGUUAUUAUGAAAGAGUUUAAGGACU
env-1543  AGUUGAUAAAGAAUAAGCACUGCCACCGGGUAGU.A.G.A.UG.............CGUA..................AAGCAUUCG.ACUGUACAUCGUUAGGUC.U.UUGAA.GAUGUAUA...UUC...GGAUGCUUAUUUUUUUGGAGGUUAUUAUGAAAGAGUUUAAGGACU
env-1544  AGUUGAUAAAGAAUAAGCACUGCCACCGGGUAGU.A.G.A.UG.............CGUA..................AAGCAUUCG.ACUGUACAUCGUUAGGUC.U.UUGAA.GAUGUAUA...UUC...GGAUGCUUAUUUUUUUGGAGGUUAUUAUGAAAGAGUUUAAGGA..
env-1545  AGUUGAUAAAGAAUAAGCACUGCCACCGGGUAGU.A.G.A.UG.............CGUA..................AAGCAUUCG.ACUGUACAUCGUUAGGUC.U.UUGAA.GAUGUAUA...UUC...GGAUGCUUAUUUUUUUGGAGGUUAUUAUGAAAGAGUUUAAGGACU
env-1546  AGUUGAUAAAGAAUAAGCACUGCCACCGGGUAGU.A.G.A.UG.............CGUA..................AAGCAUUCG.ACUGUACAUCGUUAGGUC.U.UUGAA.GAUGUAUA...UUC...GGAUGCUUAUUUUUUUGGAGGUUAUAAGGGCGGUAAUCCCUAUCA
env-1547  AGUUGAUAAAGAAUAAGCACUGCCACCGGGUAGU.A.G.A.UG.............CGUA..................AAGCAUUCG.ACUGUACAUCGUUAGGUC.U.UUGAA.GAUGUAUA...UUC...GGAUGCUUAUUUUUUUGGAGGUUAUUAUGAAAGAGUUUAA.....
env-1553  AGUUGAUAAAGAAUAAGCACUGCCACCGGGUAGU.A.G.A.UG.............CGUA..................AAGCAUUCG.ACUGUACAUCGUUAGGUC.U.UUGAA.GAUGUAUA...UUC...GGAUGCUUAUUUUUUUGGAGGUUAUUAUGAAAGAGUUUAA.....
env-1602  AGUUGAUAAAGAAUAAGCACUGCCACCGGGUAGU.A.G.A.UG.............CGUA..................AAGCAUUCG.ACUGUACAUCGUUAGGUC.U.UUGAA.GAUGUAUA...UUC...GGAUGCUUAUUUUUUUGAGGUUAUUAUGAAAGAGUUUAAGGACUA
env-1603  AGUUGAUAAAGAAUAAGCACUGCCACCGGGUAGU.A.G.A.UG.............CGUA..................AAGCAUUCG.ACUGUACAUCGUUAGGUC.U.UUGAA.GAUGUAUA...UUC...GGAUGCUUAUUUUUUUGGAGGUUAUUAUGAAAGAGUUUAAGGACU
env-1604  AGUUGAUAAAGAAUAAGCACUGCCACCGGGUAGU.A.G.A.UG.............CGUA..................AAGCAUUCG.ACUGUACAUCGUUAGGUC.U.UUGAA.GAUGUAUA...UUC...GGAUGCUUAUUUUUUUGGAGGUUAUUAUGAAAGAGUUUAA.....
env-1607  AGUUGAUAAAGAAUAAGCACUGCCACCGGGUAGU.A.G.A.UG.............CGUA..................AAGCAUUCG.ACUGUACAUCGUUAGGUC.U.UUGAA.GAUGUAUA...UUC...GGAUGCUUAUUUUUUUGGANNNNNNNNNNNNNNNNNNNNNNNNN
env-1608  AGUUGAUAAAGAAUAAGCACUGCCACCGGGUAGU.A.G.A.UG.............CGUA..................AAGCAUUCG.ACUGUACAUCGUUAGGUC.U.UUGAA.GAUGUAUA...UUC...GGAUGCUUAUUUUUUUGGAGGUUAUUAUGAAAGAGUUUAAGGACU
env-1682  AGUUGAUAAAGAAUAAGCACUGCCACCGGGUAGU.A.G.A.UG.............CGUA..................AAGCAUUCG.ACUGUACAUCGUUAGGUC.U.UUGAA.GAUGUAUA...UUC...GGAUGCUUAUUUUUUUGGAGGUUAUUAUGAAAGAGUUUAAGGACU
env-1689  AGUUGAUAAAGAAUAAGCACUGCCACCGGGUAGU.A.G.A.UG.............CGUA..................AAGCAUUCG.ACUGUACAUCGUUAGGUC.U.UUGAA.GAUGUAUA...UUC...GGAUGCUUAUUUUUUUGGAGGUUAUUAUGAAAGAGUUUAAGGACU
env-1690  AGUUGAUAAAGAAUAAGCACUGCCACCGGGUAGU.A.G.A.UG.............CGUA..................AAGCAUUCG.ACUGUACAUCGUUAGGUC.U.UUGAA.GAUGUAUA...UUC...GGAUGCUUAUUUUUUUGGAGGUUAUUAUGAAAGAGUUUAAGGACU
env-1691  AGUUGAUAAAGAAUAAGCACUGCCACCGGGUAGU.A.G.A.UG.............CGUA..................AAGCAUUCG.ACUGUACAUCGUUAGGUC.U.UUGAA.GAUGUAUA...UUC...GGAUGCUUAUUUUUUUGGAGGUUAUUAUGAAAGGGUGUCUGCCAU
env-1692  AGUUGAUAAAGAAUAAGCACUGCCACCGGGUAGU.A.G.A.UG.............CGUA..................AAGCAUUCG.ACUGUACAUCGUUAGGUC.U.UUGAA.GAUGUAUA...UUC...GGAUGCUUAUUUUUUUGGAGGUUAUUAUGAAAGAGUUUAA.....
env-1693  AGUUGAUAAAGAAUAAGCACUGCCACCGGGUAGU.A.G.A.UG.............CGUA..................AAGCAUUCG.ACUGUACAUCGUUAGGUC.U.UUGAA.GAUGUAUA...UUC...GGAUGCUUAUUUUUUUGGAGGUUAUUAUGAAAGAGUUUAAGGACU
env-1695  AGUUGAUAAAGAAUAAGCACUGCCACCGGGUAGU.A.G.A.UG.............CGUA..................AAGCAUUCG.ACUGUACAUCGUUAGGUC.U.UUGAA.GAUGUAUA...UUC...GGAUGCUUAUUUUUUUGGAGGUUAUUAUGAAAGAGUUUAAGGACU
env-1696  AGUUGAUAAAGAAUAAGCACUGCCACCGGGUAGU.A.G.A.UG.............CGUA..................AAGCAUUCG.ACUGUACAUCGUUAGGUC.U.UUGAA.GAUGUAUA...UUC...GGAUGCUUAUUUUUUUGGAGGUUAUUAUGAAAGAGUUUAAGGACU
env-1706  AGUUGAUAAAGAAUAAGCACUGCCACCGGGUAGU.A.G.A.UG.............CGUA..................AAGCAUUCG.ACUGUACAUCGUUAGGUC.U.UUGAA.GAUGUAUA...UUC...GGAUGCUUAUUUUUUUGGAGGUUAUUAUGAAAGAGUUUAAGGACU
env-1707  AGUUGAUAAAGAAUAAGCACUGCCACCGGGUAGU.A.G.A.UG.............CGUA..................AAGCAUUCG.ACUGUACAUCGUUAGGUC.U.UUGAA.GAUGUAUA...UUC...GGAUGCUUAUUUUUUUGGAGGUUAU....................
env-1708  AGUUGAUAAAGAAUAAGCACUGCCACCGGGUAGU.A.G.A.UG.............CGUA..................AAGCAUUCG.ACUGUACAUCGUUAGGUC.U.UUGAA.GAUGUAUA...UUC...GGAUGCUUAUUUUUUUGGAGGUUAUUAUGAAAGAGUUUAAGGACU
env-1711  AGUUGAUAAAGAAUAAGCACUGCCACCGGGUAGU.A.G.A.UG.............CGUA..................AAGCAUUCG.ACUGUACAUCGUUAGGUC.U.UUGAA.GAUGUAUA...UUC...GGAUGCUUAUUUUUUUGGAGGUUAUUAUGAAAGAGUUUAAGGACU
env-1712  UGUUAAUAUAGAAUAAAAUCUGCCACCGGGUAGU.A.G.A.UG.............CGUA..................AAGCAUUCG.UUUGUACAUCGUUAGGUC.A.UUGAA.GAUGUGCA...UGC...GGAUGUUUAUUUUUUUGGAGGUUAGUAUGUCAAAGUUGAUGAAUU
env-1713  UGUUAAUAUAGAAUAAAAUCUGCCACCGGGUAGU.A.G.A.UG.............CGUA..................AAGCAUUCG.UUUGUACAUCGUUAGGUC.A.UUGAA.GAUGUGCA...UGC...GGAUGUUUAUUUUUUUGGAGGUUAGUAUGUCAAAGUUGAUGAAUU
Rfl-2-1   AAUAGAGAAAAUAUGAAAACUGCCACCGGGUAGU.A.U.A.UG.............CGAU..................AAGCAUUUG.UAUACACAUCGUUAGGUC.U.UAGAA.GAUGUGUA...CGC...AGAUGCUUUAUUUUUUUGGAGGUAUGGAACGUGUUAAAACGUAUC
env-1715  AAUAUAUCUAAUAUUGAAACUGCUACCGGGUAGG.A.U.A.UG.............CGAG..................AAGCAUUUG.UUUACACAUCGUUAGGUC.U.UAGAA.GAUGUGUA...UGC...AGAUGCUUUAUUUUUUGGAGGGAAACAAAUGAUAAAACAACUGAG
env-1717  AAUAUAUCUAAUAUUGAAACUGCUACCGGGUAGG.A.U.A.UG.............CGAG..................AAGCAUUUG.UUUACACAUCGUUAGGUC.U.UAGAA.GAUGUGUA...UGC...AGAUGCUUUAUUUUUUGGAGGGAAACAAAUGAUAAAACAACUGAG
env-1718  AAUAUAUCUAAUAUUGAAACUGCUACCGGGUAGG.A.U.A.UG.............CGAG..................AAGCAUUUG.UUUACACAUCGUUAGGUC.U.UAGAA.GAUGUGUA...UGC...AGAUGCUUUAUUUUUUGGAGGGAAACAAAUGAUAAAACAACUGAG
env-1719  UAGAUAAAGUUAAAUAUAUCUGCCACCGGGUAGA.A.A.U.G..............CGUA..................AGGCAUUCG.UUUACACAUCGUUAGGUC.U.AAGAA.GAUGUGUG...GGC...GGGUGCUUAUUUUUUUGGAGGGUAUAUGAAGAAUUUAAAAUCUCU
env-1720  UGGUCGAAAUUCAAUACGUCUGCCACCGGGUAGA.A.A.U.G..............CGUA..................AAGCAUUCG.UUUGCACAUCGUUAGGUC.U.AUGAA.GAUGUGUA...UGC...GGGUGCUUAUUUUUUUGGAGGCUAUAUGAAAAAGCUGAACGC...
Oru-1-1   UGGUCGAAAUUCAAUACGUCUGCCACCGGGUAGA.A.A.U.G..............CGUA..................AAGCAUUCG.UUUGCACAUCGUUAGGUC.U.AUGAA.GAUGUGUA...UGC...GGGUGCUUAUUUUUUUGGAGGCUAUAUGAAAAAGCUGAACGCCCU
env-1721  UAUAAUAGCUACAAUAAAUCUGCCACCGGGCAGG.A.A.U.G..............CAAA..................AAGCAUUCG.UUUACACACCGUUAGGUC.U.UAGAA.GGUGUGUA...AUC...GGAUGCUUGUUUUUUUGAUUUUUCCGCUGCAGACGGCAUCAACAG
env-1722  AAUGACCGUAAUAUAAAAACUGCCACCGGGUAGA.A.A.G.UG.............CUUA..................AAGCAUUCG.UUUGCACAUCGUUAGGUC.U.UUGAA.GAUGUGUA...UUC...GGGUGCUUAUUUUUUCGGAGGUCAUAUAUGUUAAAAAGAAUAAC
env-1723  AUAGCAGAAAAAUAUUGAUCUGCCACCGGGUAGA.A.A.U.G..............CGAA..................AAGCAUUCG.UUAACACAUCGUUAGGUC.U.UUGAA.GAUGUGUU...UGC...GGAUGCUUAUUUUUUUGGAGGUUUUAAAUUAUGAUGCGAGCUGCG
env-1724  AUAGCAGAAAAAUAUUGAUCUGCCACCGGGUAGA.A.A.U.G..............CGAA..................AAGCAUUCG.UUAACACAUCGUUAGGUC.U.UUGAA.GAUGUGUU...UGC...GGAUGCUUAUUUUUUUGGAGGUUUUAAAUUAUGAUGCGAGCUGCG
env-1725  AUAGCAGAAAAAUAUUGAUCUGCCACCGGGUAGA.A.A.U.G..............CGAA..................AAGCAUUCG.UUAACACAUCGUUAGGUC.U.UUGAA.GAUGUGUU...UGC...GGAUGCUUAUUUUUUUGGAGGUUUUAAAUUAUGAUGCGAGCUGCG
env-1726  UAUGAUAAUAACAAUAAAUCUGCCACCGGGUAGA.A.A.U.G..............CGG...................AAGCAUUCG.UUUACACAUCGUUAGGUC.U.UUGAA.GAUGUGUA...UGC...GGAUGCUUAUUUUUUGGAGGUUAAUAUGCAAAGCAACAAAACACU
env-1727  UAUAAUUAAUAUAAUAAAACUGCCACCGGGUAGA.A.A.U.G..............CUGA..................AAGCAUUUG.UUCACACAUCGUUAGGUC.U.UAGAA.GAUGUGUG...UGC...AGAUGCUUUAUUUUUUGGGAGAAAAAAUAUGAUAAAACGCAUAAA
env-1728  AAUCAAUGCAAUAUGAAAACUGCCACCAGGUAGG.A.A.U................GCUGA.................AAGCAUUUG.UUCACACAUCGUUAGGUC.U.UAGAA.GAUGUGUG...CGC...AGAUGCUUUAUUUUUUGGAGGAAUUUAUAAUGCUAAAAUACAUCA
Ral-1-1   GAUAUAAACAAUAUAAAAACUGCCACCGGGUAGA.A.A.U................GCGUA.................AAGCAUUUG.UUCACACAUCGUUAGGUC.U.UUGAA.GAUGUGUG...CAC...AGAUGCUUUAUUUUUUGGAGGAAACAGAAUGAUAAAACGUAUCAA
env-1729  AUAAUGAUUAAAAUAUAAUCUACCACCGGGUAGG.A.A.A.UG.............CGUA..................AAGCAUUCG.UUUACACAUCAUUAGGUC.U.UUGAA.GAUGUGUA...UGC...GGAUGCUUGUUUUUUGCCUAUGCGGUAGAAAUCAAAGGAGGAAAC
Gni-1-1   UUAUUAAUAAAAAUAUUAUCUGCCACCGGGUAGA.A.A.G.UG.............CGA...................AAGCAUUCG.U.UACACAUCAUUAGGUU.A.UUAAA.GAUGUGUA...UAC...GGGUGCUUAUUUUUGUUUUGGAGGGAUUGGCGUGAGACAUAAACA
env-1730  ACAAAAAAAUUAAAUUGCUCUGCCACCGGGUAGA.A.A.G.A..............GUAA..................AAGUAUUCA.UUUAUACAUCGUUAGGUC.U.UAGAA.GAUGUGUA...AAA...GGAUGCUUAUUUUUAUUUAUAUGGAGGUAUUUAUAUGUGGAGAGA
env-1731  AUAAAAUAAUAUAAUUUAACUACCACCGGGUAGU.G.A.U.G..............CUGAU.................AAGCAUUCA.UUUACACAUCGUUAGGUC.U.UAGAA.GAUGUGUA...AAU...GAGUGCUUGUUUUUUGGAGGUAAAAAAUGAGAGUUCGCAAUUCUU
env-1732  CAAAAAAUAUAUAAUCAUUCUGCCACCGGGUAGA.G.A.A.AG.............CGA...................AAGCAUUCA.UUUACACAUCGUUAGGUC.U.UAGAA.GAUGUGUA...AAG...GGAUGCUUAUUUUUUGGUUAUACGGAGGUAUUUAGAUGUUGAGAA
env-1740  GUAUAAAGUAAAUAUUAUGCUGCCACCGGGUAGC.A.A.G.AU.............GCA...................UAGCAUUCA.UAUGCACAUCGUUAGGUC.U.UAGAA.GAUGUGUGU..AGU...GAAUGCUUUUUAUAUUUUUGCUAAGGAGAUGAUUGCUAU......
env-1741  GUAUAAAGUAAAUAUUAUGCUGCCACCGGGUAGC.A.A.G.AU.............GCA...................UAGCAUUCA.UAUGCACAUCGUUAGGUC.U.UAGAA.GAUGUGUGU..AGU...GAAUGCUUUUUAUAUUUCUAUAUUAUUGUCAAGGAGAUGAU....
env-1742  GUAUAAAGUAAAUAUAAUGCUGCCACCGGGUAGC.A.A.G.AU.............GCA...................UAGCAUUCA.UAUGCACAUCGUUAGGUC.U.UAGAA.GAUGUGUA...UGUU..GAAUGCUUUUUAUAUUUUUAUAUUAUUGCUAAGGAGAGGAU....
env-1743  UAGCUAAGUAUAUAAAACUCUACCACCGGGUAGG.A.A.U.GU.............AAU...................AAGCAUUCG.CUUGCACAUCAUUAGGUC.U.UAGAA.GAUGUGUAU..AGC...GGAUGCUCUUUUUUGUUGAGGAGGAAGCAAUUAUGAGAUACAACC
env-1744  GUAUAUAAGAAAUAUCGAACUGCCACCGGGUAGG.U.A.U.G..............CGUA..................AAGCAUUUG.UUUACACAUUGUUAGGUC.U.CAGAA.GAUGUGUAA..GGC...AAGUGUUUAUUUUUUUGGAGGUAACUAUGAAAAAACUUAAGUCAU
env-1745  AUGUUAUAUUACAGGUAAACUGCCACCGGGUAGA.A.A.A.U..............GCAA..................AAGCAUUCG.UCUGCACAUCGUUAGGUC.U.UAGAA.GAUGUGCA...AAC...GGAUGCUUUUUUUAUGGGGGAUUAUUAUGAAAAUAAUUAAAACAA
env-1746  CUGUGCUAUCAUGCACUCACUGCCACCGGGUAGG.A.A.A.UG.............CUGU..................GAGCAUUCG.ACUGCACAUCGUUAGGUC.U.UAGAA.GAUGUGCG...GGC...GGAUGCUUUCUUUUUUUGGAGGAUCAUACUAUGGCGGAGAGA...
env-1747  AUAUAAUAUUC.CUGUAAACUGCCACCGGGUAG.......................GAAUGCAUU.............AAGCAUUCG..UCACACAUCGUUAGGUC.U.UAGAA.GAUGUGUG...AGC...GAAUGCUUUUUUAUUUGGAGGAAAAAGAGUGCUUAAAAGUAUACG
env-1748  AUAUAAUAUUC.CUGUAAACUGCCACCGGGUAG.......................GAAUGCAUU.............AAGCAUUCG..UCACACAUCGUUAGGUC.U.UAGAA.GAUGUGUG...AGC...GAAUGCUUUUUUAUUUGGAGGAAAAAGAGUGCUUAAAAGUAUACG
env-1749  AUAAAUUUAAAUAUAUUUGCUGCCACCGGGUAGC.A....................CAAU..................AAGCAUUUG.CGCGCGUGUCGUUAGGUC.U.UGGAA.GAUACGCC...UGC...AAAUGCUUAUUUUUUUAGGAGGUAAUAUAUGAAAAAGUUAAAGC
env-1750  CAAAAAAUUAGAAUAGUAACUGCCACCGGGUAGU.A.U..................GCG...................GAGCAUUCG.CAGGUAUAUCGUUAGGUC.U.UUGAA.GAUAUAUC...AGC...GGAUGCUAUUUGUUUGGAGGGGUAUAAUGAAUAUUAAAGAUUUUU
env-1751  GUUAUAAAAGAAUAUAUAUCUGCCACCGGGUAGA.G.A.A.U..............GCG...................GGGCAUUCG.CCGGCAUAUCGUUAGGUC.U.UAGAA.GAUAUGUC...AGC...GGAUGCUUUUUGUUUCGCUAUGGGAAACCGCAUUUGCGUUCUGCC
env-1752  AAAUCGCAUAACAAAAAUACUGCCACCGGGCAGG.A.A.A.U..............GCAAAAA...............AAGCAUUCG.UGCGCGCAUCGUUAGGUC.U.UUGAA.GAUGCGUG...GAC...GGGUGCUUUCUUUUUUCGGAGGAACGAUGCAAAAGAAACAACACG
env-1753  AAAUCGCAUAACAAAAAUACUGCCACCGGGCAGG.A.A.A.U..............GCAAAAA...............AAGCAUUCG.UGCGCGCAUCGUUAGGUC.U.UUGAA.GAUGCGUG...GAC...GGGUGCUUUCUUUUUUCGGAGGAACGAUGCAAAAGAAACAACACG
env-1754  UCUGAUUAUAAUAUUGAUACUGCCACCGGGUAGU.G....................AGCUCAU...............GUGCGCUCG.UUUACACAUCGUUAGGUC.U.UAGAA.GAUGUGUA...GAUA..GGGUGCUUUUUUACUAAUUACCAAUUCAGAAAGGAAGGCUGAACA
env-1756  AGCAAAUUGAAUAUCAUUGCUGCCACCGGGUAGC.C.A.G.AA.............AUA...................CAACAUUCG.UCUGCACAUCGUUAGGUC.U.UUGAA.GAUGUCCAU..CAC...GGGUGUUUCUUUUUUUGGAGGUUUAUAAAUGAAAAUCUGCAAUCC
env-1757  ACAGAUAAAACAAAGAAUUCUGCCGCCGGGCAGA.A....................................................AACAUUCG.UUUCCAUGUCGUUAGGCC.U.UUGAA.GACAUGCG...GAC...GGAUGUUAUUUUUUUUGAAAGAGGGAAUUUUAUGCAGCAUUUUAC
env-1759  UGACAACGAAAUAACUAAACUGCCACCGGGUAGU.G.A.G.CU.............GAU...................ACGCGCUCG.UUUCCACAUCGUUAGGUC.U.UAGAA.GAUGUGAG...GGAU..GAGCGCUUUUUGAAAGGUAGGACUGACAUGAAAAAAUUUAUAAUC
env-1761  AUCAAAAAAAAUAAUAAAACUGCCACCGGGUAGU.G.A.G.CU.............CU....................CAGCGCUCG.UUUCCACAUCGUUAGGUC.U.UAGAA.GAUGUGUG...AAAU..GUGCGCUUUUUUAAAACCUGUCUAAAGUAAAAACGGAGGUUAAGA
env-1762  ACAUGCACGUAAAUAUUAUCUCCCACCGGGGAGA.G.A..................UGCAA.................AAAGCAUCC.GCAGCACAUCGUUAGGUC.U.UUGAA.GAUGUGCCG..GCG...GGUGCUUUGUUUUUUCGGAGGUCGAAAUGGAAAAAGCAACAAAAA
env-1763  ACAUGCACGUAAAUAUUAUCUCCCACCGGGGAGA.G.A..................UGCAA.................AAAGCAUCC.GCAGCACAUCGUUAGGUC.U.UUGAA.GAUGUGCCG..GCG...GGUGCUUUGUUUUUUCGGAGGUCGAAAUGGAAAAAGCAACGAAAA
env-1764  AAAUGCUCUAUGUAUAUCUCUGCCGCCGGGCAGA.A.A.A.AU.............GCGA..................AAAGCAUUC.CGCUUACAUCGUCAGGCC.U.UUGAA.GAUGUAUG...CAG...GAUGCUUAUUUUUUUGGAGGCUGACAUGAAAAAGCUUUUACCCGA
env-1765  AGAACUCUUAUUAUAGGAACUGCCACCGGGUAGU.G.U.A.UG.............CUGU..................UAAGCAUUC.GUCGCACAUCGUUAGGUC.U.UUGAA.GAUGUGCGCC.ACG...GGUGCUUAUUUUUUAUGGAGGUUUCUCGUAUGCAAGCAUGGAAUC
env-1766  AGAACUCUUAUUAUAGGAACUGCCACCGGGUAGU.G.U.A.UG.............CUGU..................UAAGCAUUC.GUCGCACAUCGUUAGGUC.U.UUGAA.GAUGUGCGCC.ACG...GGUGCUUAUUUUUUAUGGAGGUUUCUCGUAUGCAAGCAUGGAAUC
env-1768  GUUAUUAAUAUGAAUAUUUCUGCCACCGGGUAGA.A.A.A.AU.............GCA...................AAGGCAUUC.CCUGUACAUCGUCAGGUC.U.UAGAA.GAUGUACA...GCG...AGUGCCUUUUUUGAGGUGAACUAUGUUAAACUACUUUACAAAAAC
env-1769  ACAGAAAUGUUUCACACGACUGCCACCGGGUAGG.A.A.U.G..............CAC...................AGAGCAUUC.GCCACACAUCGUUAGGUC.U.CAGAA.GAUGUGUG....CG...GAUGCUUUUUUAUGCUAUGCAUACCGCUGGCGCAUACAAAGAUCC
env-1770  GCAUGGAGAUUUCACACUACUGCCACCGGGUAGG.A.A.U.G..............CAC...................AGAGCAUUC.GCCACACAUCAUUAGGUC.U.UAGAA.GAUGUGUG....CG...GAUGCUUUUUGUUUGUUUCAGUGGUUUUAAAUUACAAAAAGGAGU
env-1771  CAAUUUCAGAAGAAAUUAUCUGCCACCGGGUAGG.A.A.A.U..............GCA...................GCUGCAUUC.AUCUCACAUCAUUAGGUC.U.UUGAA.GAUGUGUC...AUG...GAUGCUUCUUUUUUUGGAGGUAUUUUUAUAUCAUGAACAACAAAA
env-1772  UAAGCCCAUAAAAAUCAAGCUGCCACCGGGCAGC.AGA.G.AU.............GCA...................AAAGCAUCC.GCCGCACAUCGUUAGGUC.U.UGGAA.GAUGUGCCG..ACG...GGUGCUUGUUUUUUUCGGAGGUUCAAAUGACGGAAACACUAAAAA
env-1773  CAGUUUAAGUUGAAUAUAUCUGCCACCGGGUAGA.A.A..................AUGCG.................AAAGCAUUC.GCUGUAUGUCGUUAGGUC.U.UUGAA.GAUAUACAG..UCG...AAUGCUUUUUGUUCCGCUGGCGGGAAAGGAGAAUGUUAUGAACA
env-1774  .....AUAGCUUAUAUAAUCUGCCACCGGGUAGA.G.A.A.UG.............CAUA...................AAGCAUUU.GUUACACAUCAUUAGGUC.U.UUGAA.GAUGUGUU....UACA.AGUGCUUAUUUUUUUUCAAAAACGAGGUAGUGUAAAGUAAAAAUG

          .......................<<<..............................................................................>>>.........................................................................
          .......................20...............................................................................02..........................................................................
          ............<<<<<<<<<<<....>>>>>>.>.>.>.>>......<<<<<<<<..........>>>>>>>>....<<<<<<<<<.<<<<<<<<<<...............>>>>>>>>...>>>...>>>>>>>>..........................................
          ............22222222222....222222.2.2.2.22........1111............1111.....222222222.2222222222...............22222222...222...22222222..........................................
          ••••••••••••••••••YUGCCRCCGGGUAR•-○-○-○-○-------------○○○------------------○○R•••Y••-••••YRY•YCRUUAGGYC-•-U•GAA-GR•RYR••---•••---•R•••YUUUUUUY••••••••••••••••••••••••••••••••
          5'                                                                                                                                                   3'
```

```
env-1775  UUCAAAUUUGAUUAUGAUUCUGCCACCGGGUAGA.G.....................AAUG....................GAAGCAUUC.GCAGCGCAUCGUUAGGUC.U.UUGAA.GAUGCGUGA..GCG...GGUGCUUAUUUUUUGGAGGUCAAAAUGAGCCGGCCAGCGAAAAA
env-1776  UUCAAAUUUAAAUGUAAUCCUGCCACCGGGUAGG.G.....................AAUGG....................AAGCAUUC.GUUGCGCAUCGUUAGGUC.U.UUGAA.GAUGCGUGU..ACG...GGUGCUUGUUUUAUUGGAGGAAAAAUGGGCCGAAUUGCAAGAAAA
env-1778  AUAUUCGGUAAAUAUAAAACUGCCACCGGGUAGU.......................AAAUGCG.................AAAGCAUUC.GUAACACAUCGUUAGGUC.U.UAGAA.GAUGUGUC...GCG...GAUGCUUGUUUUUUUGGAGGUUACUAAAAUGCAGAUAAAGUCACU
env-1779  AGCAUCAUUCAUAGAGAAUCUGCCACCGGGUAGG.A.G.A.U...............GCA.....................UAGGCAUCC.GUUACACAUCGUUAGGUC.U.UAGAA.GAUGUGUAG..ACG...GAUGCUUUUUUAGCGGUACAGUUUUUACAUAAUAUUUGCACAAG
env-1780  CAGCAUCAAUGAAUAUAAACUGCCACCGGGUAGU.A.U.A.UG..............CGC.....................AGAGCAUUC.AAUGCACAUCGUCAGGUC.U.UAGAA.GAUGUGUG...UUG...GAUGCUUUUUUCGGAGGGAUAAAAUGCUUCGCAAAAUAACCGGAU
Rsp-3-1   CGAAAAAAAUAACUAUAAACUGCCACCGGGUAGU.A.U...................GCG.....................UAAGCAUUC.GAUACACAUCGUUAGGUC.U.UAGAA.GAUGUGUGU..UCG...GAUGCUUUUUUGGGAGGAAACGAUAUGCUCAACAGAAU......
env-1781  UAUAAAUCUAAAUACAACACUGCCACCGGGUAG........................AUAAAAUGCUU.............UAAGCAUUC.GUUGCAUAUCGUUAGGUC.U.UAGAA.GAUAUGUU...ACG...GGUGCUUUUUUUGGAGGUAAAACAAACAUGAACAUUUAUGCAUCA
env-1789  UAUAAAUCUAAAUACAACACUGCCACCGGGUAG........................AUAAAAUGCUU.............UAAGCAUUC.GUUGCAUAUCGUUAGGUC.U.UAGAA.GAUAUGUU...ACG...GGUGCUUUUUUUGGAGGUAAAAUAAACAUGAACAUUUAUGCAUCA
env-1792  AAUAAAUUAAAUAAACACACUGCCACCGGGUAGA.G.U.A.AU..............GCAU....................AAAGCAUUC.GUAACAUAUCGUUAGGUC.U.UAGAA.GAUAUGUC...ACG...GAUGCUUUUUUGGGAGGAAAAGGAAUUAUGAAUAUUUAUCGUUCA
env-1794  AUGAUGAAAGAAUAAUAAACUGCCACCGGGUAGG.U.A.A.U...............GUG.....................AAAGCAUUU.GUCGUACAGCGUUAGGUC.U.UUGAA.GCUGUACCG..GCA...AGUGCUUUUUUUGGAGGUAAAAAUGAGUAUAAAGGAUAUUUCAC
Rsp-2-1   UUUUCAUGCAAUAAUAUGACUGCCACCGGGUAGG.C.G.G.U...............GCA.....................CAAGCACUC.GUUACACAUCGUUAGGUC.U.UUGAA.GAUGUGUG...AGG...GGUG.UUGUUUUUUGGGAGGGCUCAAUGAAAUCAAAGCUAAGAUU
env-1796  UCUUUUCAGAAUAUUUUUACUGCCACCGGGUAGG.C.G.G.UG..............CA......................UCGGCACUC.GUUACACAUCGUUAGGUC.U.UUGAA.GAUGUGUA...AGG...GGUGUUAUUUUUUUGGAGGGCAUAAUGAAAUCAAAGCUAAGAUCA
env-1800  UCUUUUCAGAAUAUUUUUACUGCCACCGGGUAGG.C.G.G.UG..............CA......................UCGGCACUC.GUUACACAUCGUUAGGUC.U.UUGAA.GAUGUGUA...AGG...GGUGUUAUUUUUUUGGAGGGCAUAAUGAAAUCAAAGCUAAGAUCA
env-1801  UCUUUUCAGAAUAUUUUUACUGCCACCGGGUAGG.C.G.G.UG..............CA......................UCGGCACUC.GUUACACAUCGUUAGGUC.U.UUGAA.GAUGUGUA...AGG...GGUGUUAUUUUUUUGGAGGGCAUAAUGAAAUCAAAGCUAAGAUCA
env-1802  UCUUUUCAGAAUAUUUUUACUGCCACCGGGUAGG.C.G.G.UG..............CA......................UCGGCACUC.GUUACACAUCGUUAGGUC.U.UUGAA.GAUGUGUA...AGG...GGUGUUAUUUUUUUGGAGGGCAUAAUGAAAUCAAAGCUAAGAUCA
env-1803  UUUACUUUAGAAUAAUUUACUGCCACCGGGUAGG.C.G.G.U...............GCU.....................AUCGCACUC.GUUACACAUCGUUAGGUC.U.UUGAA.GAUGUGUG...AUG...GGUGUUAUUUUUUGAAGGUAAGGAUGAAUAAAAUGCUGAA.....
env-1804  ACCAUAAAGAAUAUUUUAACUGCCACCGGGUAGG.A.A.A.UG..............C.......................AAAGCAUUC.GCGGCACAUCGUUAGGUC.U.UUGAA.GAUGUGCAG..GCG...AAUGCUUUUUGAUUUUCUUUCCUUAAGUAAUUGGAGGAUACGAUC
env-1805  ACCAUAAAGAAUAUUUUAACUGCCACCGGGUAGG.A.A.A.UG..............C.......................AAAGCAUUC.GCGGCACAUCGUUAGGUC.U.UUGAA.GAUGUGCAG..GCG...AAUGCUUUUUGAUUUUCUUUCCUUAAGUAAUUGGAGGAUACGAUC
env-1806  UCAAAAAUAAAUAAAUAUACUGCCACCGGGUAGA.A.G.A.GU..............GUU.....................AAAGCAUUC.GUCGCAUAGCGUUAGGUC.U.UUGAA.GCUAUGCC...GCG...AAUGCUUUUUGAUUGAGGAGAAUAUGAAAUUGUUGCGUUACUUUA
env-1807  CGAAAAAAGAAUAGAAUAUCUGCCACCGGGUAGA.A.A.A.AU..............GCG.....................AAAGCAUUC.GUAGCAUAUCGUUAGGUC.U.UUGAA.GAUAUGCG...GCG...AAUGCUUUUUUUGUUUGGAGGUUUGUUUAUGAAAAAGAAAAGAUGG
env-1808  GUAUGGUAUAUCUAUACCACUGCCACCGGGUAGA.G.U.A.AU..............GCA.....................AAUGCAUUC.GGUACACAUCGUUAGGUC.U.UUGAA.GAUGUGUAU..ACG...GAUGCUUUUUUGUUUGUUGGGAGGAUUUUGUGAUGAAAGCUGCAU
env-1809  CUAUGGUAUAUCUAUACCACUGCCACCGGGUAGA.G.U.A.AU..............GCA.....................AAUGCAUUC.GGUACACAUCGUUAGGUC.U.UUGAA.GAUGUGUAU..ACG...GAUGCUUUUUUGUUUGUUGGGAGGAUUUUGUGAUGAAAGCUGCGU
env-1810  CUAUGGUAUAUCUAUACCACUGCCACCGGGUAGA.G.U.A.AU..............GCA.....................AAUGCAUUC.GGUACACAUCGUUAGGUC.U.UUGAA.GAUGUGUAU..ACG...GAUGCUUUUUUGUUUGUUGGGAGGAUUUUGUGAUGAAAGCUGCGU
env-1811  GUGUGGUAUAUCUAUACCACUGCCACCGGGUAGA.G.U.A.AU..............GCA.....................GAUGCAUUC.GGUACACAUCGUUAGGUC.U.UAGAA.GAUGUGUGC..GCG...AAUGCUUUUUUCUUUGUUGGAGGGAUUCUUGCAAUGGUUUGAUU
env-1812  AUAUGGUACAAUGAAAACACUGCCACCGGGUAGU.A.A.G.AU..............GC......................UUAGCAUUC.GGUACACAUCGUUAGGUC.U.UUGAA.GAUGUGUAU..GCG...GGUGCUUGUUUUUUUGGAGAGGGCAGUAAAUCCUGAAAAUAAAUU
env-1813  GUUUUGUAAAUAUAUAUAUCUGCCACCGGGCAGA.G.A.U.G...............CUU.....................UAAGCAUUC.AUUACUCAUCGUUAGGUC.U.UUGAA.GAUGAGCAU..AUG...GACGCUUAUUUUUUUUUUGGAGGUCGUUUUAUGAAAAAAACUAAG
env-1814  GUUUUGUAAAUAUAUAUAUCUGCCACCGGGCAGA.G.A.U.G...............CUU.....................UAAGCAUUC.AUUACUCAUCGUUAGGUC.U.UUGAA.GAUGAGCAU..AUG...GACGCUUAUUUUUUUUUUGGAGGUCUUUUUCUGAAAAAAACUAAU
env-1815  UAAACAGAAUAAAAGUAAUCUGCCACCGGGUAGA.......................AGCGA....................AAGCAUUC.GCCGCACAUCGUUAGGUC.A.UUGAA.GAUGUGCUU..GCG...AAUGCUUGUUUUUUGGAGGUGCUAUCAUGUUCCAUAUAAAAUUCA
env-1816  UAAUUUAAAUAUUAAAAAACUGCCACCGGGUAGA.U.....................AGCG....................AAAGCAUUC.AUUACACAUCAUUAGGUC.U.UUGAA.GAUGUGUAA..AUG...GAUGCUUAUUUUUAUUUUUGGAGGUAUUUAUUUAUAUGUUAUCAA
env-1818  AUGAAAAAAAUGAAUUAUGCUGCCACCGGGUAGU.A.A.A.AU..............GCAA....................AAAGCAUUC.UGUACACAUCGUAAGGUC.U.UAGAA.GAUGUGUA...UCG...AAUGCUUUUCUUUUUUAUGAGAAAGGAAUGUCAAAAAAUGAAAUC
env-1819  AUGAAAAAAAUGAAUUAUGCUGCCACCGGGUAGU.A.A.A.AU..............GCAA....................AAAGCAUUC.UGUACACAUCGUAAGGUC.U.UAGAA.GAUGUGUA...UCG...AAUGCUUUUCUUUUUUAUGAGAAAGGAAUGUCAAAAAAUGAAAUC
env-1820  GUAACUCAAAAUAUUGAAUCUGCCACCGGGUAGA.A.A.A.CA......................................AAAGCAUUU.GUUACAUAUCGUUAGGUC.U.UUGAA.GAUAUGUA...AAUA..AAUGCUUAUUUUUUUAACGACUUAUUUAGGAGGAAAUAAAGUAU
env-1821  UAAUACAUUCAUAAUAAAUCUGCCACCGGGUAGG.A.A.U.................UGCCA...................UGAGCAUUC.GCUGCACAUCGUUAGGUC.U.UUGAA.GAUGUGCA...UCG...GAUGUUUAUUUUUUUGGAGGAUAUUAUGAAGCGGUUUUAAAUUA
env-1822  CAUCAUAUAGAAAAUUAAUCUGCCACCGGGUAGA.G.A.A.................UGCA....................AUAGCAUUC.GUUGCACAUCGAUAGGUC.U.UUGAA.GAUGUGUA...AUG...GGUGCUUUUUUUGACGGAGGUUAUUAUGUUUAAGUUAUAUUCUU
env-1823  AAGUUAAAUAAGAAUUAGUCUGCCACCGGGUAGA.G.A.A.G...............CG......................AAAGCAUUC.GUUGCACAUCGUUAGGUC.U.UUGAA.GAUGUGUAU..ACG...GGUGCUUUUUUUGGAGGUGUAUAUGUUAUCAAAAAAAUGGAGAGC
env-1824  AGAACAUAAAAAAAUUAUACUGCCACCGGGCAGA.G.G.A.................UGCA....................GGCGCAUUC.GUUACACAUCGUUAGGUC.U.UUGAA.GAUGUGUAU..ACG...GGCGCUUAUUUUUUUGUUUUUAGGAGGUCGUCAUGUACGAAAAAU
env-1825  UAAAAGAAAAAUAAUUAAUCUGCCACCGGGUAGA.G.A.A.................UGCA....................AAAGCAUUC.GUUACACAUCGUUAGGUC.U.UUGAA.GAUGUGUAU..ACG...AAUGUUUAUUUUUUUGGAGGUAUCUUAUGAUAAUGAACAAGUUAA
env-1829  UAAAAGAAAAAUAAUUAAUCUGCCACCGGGUAGA.G.A.A.................UGCA....................AAAGCAUUC.GUUACACAUCGUUAGGUC.U.UUGAA.GAUGUGUAU..ACG...AAUGUUUAUUUUUUUGGAGGUAUCUUAUGAUAAUGAACAAGUUAA
Dsp-1-1   AAGUUAAAUAAAUGGUAAUCUGCCACCGGGUAGA.U.A.A.GC..............G.......................AAAGCAUUC.GUUACACAUCGUUAGGUC.A.UUGAA.GAUGUAUAA..GCG...GAUGCUUAUUUUAUUUUAGGAGUUAAUCAUGUGUAUGCGAAAGAA
env-1830  AAGUCAAUUCAAUAAUAGUCUGCCACCGGGUAGA.G.A.A.................GCG.....................AAAGCAUUC.GUUACACAUCGUUAGGUC.G.UUGAA.GAUGUGUAG..AUG...GAUGCUUUUUAUUUUUAGCAGGGAGGUAAUUAUAUGUAUUUAACU
Lba-3-1   AGGUCAAAUAGAUAAUAGGCUGCCACCGGGUAGA.A.A.A.................GCG.....................AAAGCAUUC.GUUACACAUCGUUAGGUC.U.UUGAA.GAUGUGUGG..UCG...GGUGCUUAUUUUAUUUUAGGAGGUAAUUAUAUGCACACGAAAGAG
env-1831  UAUAAUUCCCAAAAAGAUUCUGCCACCGGGUAGA.A.A.C.AU..............GCG.....................AAAGCAUUC.GCUACACAUCGUUAGGUC.U.UGGAA.GAUGUGUAU..GCG...GAUGCUUAUUUUUUUUGGAGGACUUUGAAAAUGGCAAAGAAAGGG
env-1832  ACUUAUUCAUAUAAUUGUUCUGCCACCGGGUAGA.G.A.U.G...............CG......................AAAGCAUUC.GUUACACAUCAUUAGGUC.U.UAGAA.GAUGUGUAU..ACG...GAUGCUUAUUUUUGUUGGCAAGGAGAAUUUUUUAUGUUAUUUACA
env-1837  ACUUAUUCAUAUAAUUGUUCUGCCACCGGGUAGA.G.A.U.G...............CG......................AAAGCAUUC.GUUACACAUCAUUAGGUC.U.UAGAA.GAUGUGUAU..ACG...GAUGCUUAUUUUUGUUGGCAAGGAGAAUUUUUUAUGUUAUUUACA
env-1838  CAGCAACAAAAUAAAUGGUCUGCCACCGGGUAGG.U.A.A.................GCG.....................AAAGCAUUC.GUUACACAUCGUUAGGUC.U.UUGAA.GAUGUGUAU..AAG...GAUGCUUAUUUUUGUUUUGGAGGUAUUUUAUAUGCUAUCGAAGAU
env-1839  AUUUUAAAAAAUAAUGGUUCUGCCACCGGGUAGA.A.A.A.A...............GCG.....................AAAGCAUUC.GUUACACAUCAUUAGGUC.U.UAGAA.GAUGUGUAU..ACG...GGUGCUUGUUUUUAUUUAUACGGGGGAAUUUAUAUGUUAUCUAAA
env-1840  AUAAAAUAAAAUAGUUACUCUGCCACCGGGUAGA.A.A.U.G...............UG......................AAAGCAUUC.GUUGCACAUCGUUAGGUC.U.UAGAA.GAUGUGUAU..AUG...GGUGCUUGUUUUUGUUUAUAUGGAGGUUUUUAUAUGUUAUCAAAG
env-1842  CGUCAUAAAAAUAAAUAUACUGCCACCGGGUAGG.A.A.A.U...............GC......................AAAGCAUUC.GCGGCACAUCGUUAGGUC.U.UAGAA.GAUGUGUAC..ACG...GAUGCUUUUUUUGAUUUCCGGGGCGCAAAGGAGUAAAAAGCAUGG
env-1843  GUAAACAGCAGGUAAUAUUCUGCCACCGGGUAGA.G.A.A.................GCG.....................AAAGCAUUC.GUUACACAUCGUUAGGUC.U.UAGAA.GAUGUGUAA..ACG...GAUGCUUUUAUUUUGGAGGUACAAAUGUCAUCAAAAAAACUUAAA
env-1844  UUGUAAAAAAAUAAUGAAUCUGCCACCGGGCAGA.G.A.U.G...............CUU.....................AAAGCAUCC.GUUACUCAUCGUUAGGUU.U.UUGAA.GAUGGGUAA..AUG...GGUGCUUAUUUUUUAGGAGGUCAUUUAUGAAAUUGAUUAAAUCGU
env-1845  UAGCAUUAGGUUAAUUUAUCUGCCACCGGGCAGA.G.A.U.G...............CCA.....................AAAGCAUCC.AUUACUCAUCGUUAGGUC.U.UUGAA.GAUGAGUAU..AUG...GGUGCUUUUUUUAGGAGGUUAAUAUGAAGUUAAUAAAAUCGGCUG
env-1846  GAAAUCAGAAUAAAGUAAACUGCCACCGGGUAGG.A.A.A.U...............GCA.....................AAAGCAUUC.GCGGCACAUCGUUAGGUC.U.UGGAA.GAUGUGCGG..GCG...AAUGCUUUUUUUGGAGGGAAAUUUGUUAUGCUUUCAAAAAUGCGA
env-1848  UACGCAAAAAUUAUAUUAAUUGCCACCGGGUAAU.A.A.A.A...............UGCG....................AAAGCAUUC.GUUGCACAUCAUUAGGUC.U.UUGAA.GAUGUGUAC..ACG...AGUGCUUAUUUUGUUUGGAGGUAUAUAUAAAAUGCACAA.....
env-1849  UAUGUCAAGAUAAAAGAAACUGCUACCGAGUAGU.A.G.A.AU..............GC......................AAAGCAUUC.GUUGCACAUCAUUAGGUC.U.UAGAA.GAUGUGUAU..GCG...AGUGCUUAUUUUUUUUGGAGGUUAGUUUAUGUUAAAGAAACCGGUU
env-1850  CAACAAUUCAAUGAUAACUCUGCCACCGGGCAGG.A.A.U.G...............GA......................AAAGCAUUC.AUUACUUAUCGUUAGGUC.U.UUGAA.GAUAAGUAC..AUG...GGUGCUUAUUUUUUCGGAGGUUUAUAUGAAGUCGCUUAAAUCAAU
env-1851  AGGUUAAAUAAAUAACAGUCUGCCACCGGGUAGA.......................GAUGCA..................AAAGCAUUC.GUUACACAUCAUUAGGUC.U.UUGAA.GAUGUGUA...AGC...GGAUGUUUAUUUUAUUUUUAGGAGAUAAUUAUAUGCAUUUACAAA
env-1852  UCUAACAGAUAUAAUAAGUCUGCCACCGGGUAGA.G.U.A.A...............AUC.....................GAGGCACUC.GUUACACAUCGUUAGGUC.U.UAGAA.GAUGUGUAG..UCG...UGUGCUUAUUUUUAAUUGUAUAAUUUUGAAAGGUGAAUGAAAUGU
env-1853  UCUAAAAAAUAUAAUAAGUCUGCAACCGGGUAGA.A.U.A.A...............AUC.....................AAAGCACUC.GUUACACAUCGUCAGGUC.U.UAGAA.GAUGUAUA...ACG...AGUGUACUUAUUUUUAAUUGUAUAAUUUUGAAAGGUGAAUGAAAU
env-1857  UCUAAAAAAUAUAAUAAGUCUGCAACCGGGUAGA.A.U.A.A...............AUC.....................AAAGCACUC.GUUACACAUCGUCAGGUC.U.UAGAA.GAUGUAUA...ACG...AGUGUACUUAUUUUUAAUUGUAUAAUUUUGAAAGGUGAAUGAAAU
env-1858  UCUAAAAAAUAUAAUAAGUCUGCAACCGGGUAGA.A.U.A.A...............AUC.....................AAAGCACUC.GUUACACAUCGUCAGGUC.U.UAGAA.GAUGUAUA...ACG...AGUGUACUUAUUUUUAAUUGUAUAAUUUUGAAAGGUGAAUGAAAU
env-1859  UCUAAAAAAUAUAAUAAGUCUGCAACCGGGUAGA.A.U.A.A...............AUC.....................AAAGCACUC.GUUACACAUCGUCAGGUC.U.UAGAA.GAUGUAUA...ACG...AGUGUACUUAUUUUUAAUUGUAUAAUUUUGAAAGGUGAAUGAAAU
env-1860  AAAUAGAAUUAUAUACAAUCUGCCACCGGGUAGG.......................AAUGCA..................AAAGCAUUC.GUUACACAUCGUAAGGUC.U.UAGAA.GAUGUGUAA..ACG...AAUGCUUUUCAGGUUUGGAGGGAAAUUAAUGACUCAUUACUUUAC
env-1861  AAAAUUAAAAACAAAUUAUCUGCCACCGGGUAGA.A.A.A.................ACCG....................AAUGCAUUC.GUUGCACAUCGUUAGGUC.U.UUGAA.GAUGUGUA...UUA...CGGGUGCUUUUUAUUUUGAAGAGAGGUAUUUUAUGUUAUCGGGA
env-1862  UAAAAAUUCAUAUUUAAAUCUGCCACCGGGUAGA.A.A.A.UG..............CA......................CCAGCAUUC.GUGACACAUCGUUAGGUC.U.UAGAA.GAUGUGUAA..GCG...AGUGUUGUUUUUAUUUUAGGAGGUAUGCUUUUAUGCGAAACAAGU
env-1864  CUGUGGUAUAAUUCAUUCAUCACCGCCGGGUGA..A.A.A.UG..............CUGA....................AAGCAUUUG.UCUGUACAUCGUCAGGCC.U.AAGAA.GAUGUACC...GAC...AAGUGCUUUUCUUU..............................
env-1865  AUAUGAUACAAUAUCUACAUCGCCACCGGGUGAA.A.A.U.G...............UUAA....................AAGCAUUUG.UCCGUACAUCGUUAGGUC.U.UUGAA.GAUGUACC...GAC...AAGUGCUUUUCUUUUGGAGUGAUAAUAUGUCAAAGUUUUCAGUA
env-1866  AUAUGAUACAAUAUCUACAUCGCCACCGGGUGAA.A.A.U.G...............UUAA....................AAGCAUUUG.UCCGUACAUCGUUAGGUC.U.UUGAA.GAUGUACC...GAC...AAGUGCUUUUCUUUUGGAGUGAUAAUAUGUCAAAGUUUUCAGUA
env-1869  AUAUGAUACAAUAUCUACAUCGCCACCGGGUGAA.A.A.U.G...............UUAA....................AAGCAUUUG.UCCGUACAUCGUUAGGUC.U.UUGAA.GAUGUACC...GAC...AAGUGCUUUUCUUUUGGAGUGAUAAUAUGUCAAAGUUUUCAGUA
env-1870  UUAAAAUAAAUAUAUUCGGUUGCCACCGGGUAGC.C.G.A.G...............AUGCG...................AAAGCAUUC.GUGAUACAUCAUUAGGUC.U.CAGAA.GAUGUAUGC..ACG...GGUGCUUUUUCAUUUGAGGAGGUUUAUUAUGCUGGCUUUUCUGAA
env-1871  AAAAUCAAAUAAAAACUAGCUACCACCGGGUAGC.A.A.U.AU..............GCC.....................AAGCAUAUC.GUUAUACAUCGUUAGGUC.U.UUGAA.GAUGUAUG...GCUG..GUACGCUUAUUUUU..............................
Ssu-6-1   AAAUUUAAUUAAAUAAACACUGCCACCGGGUAGU.C.A.U.................GCA.....................ACAGCAUUU.UAUGAACAUCGUUAGGUC.U.UAGAA.GAUGUUCA...UAG...GGUGCUGAUUUUUCUAUAAAAGGAGUUGGAAUAUGUCACAAUUUA
Ssu-1-3   AAAUUUAAUUAAAUAAACACUGCCACCGGGUAGU.C.A.U.................GCA.....................ACAGCAUUU.UAUGAACAUCGUUAGGUC.U.UAGAA.GAUGUUCA...UAG...GGUGCUGAUUUUUCUAUAAAGGAGUAGGGAUAUGUCGCAAUUUAU
Ssu-1-4   GAAAGCAACCAAAUAUUUACUGCCACCGGGUAGU.C.A.U.................GCA.....................ACAGCAUUU.UAUGAACAUCGUUAGGUC.U.UUGAA.GAUGUUCA...UAG...GGUGCUGAUUUUGUAUAAAAAAGGAGCAAGAAUAUGUCACAAUUU
Ssu-1-7   GAAAGCAACCAAAUAUUUACUGCCACCGGGUAGU.C.A.U.................GCA.....................ACAGCAUUU.UAUGAACAUCGUUAGGUC.U.UUGAA.GAUGUUCA...UAG...GGUGCUGAUUUUGUAUAAAAAAGGAGUAGGAGUAUGUCACAAUUU
Ssu-1-8   GAAAGCAACCAAAUAUUUACUGCCACCGGGUAGU.C.A.U.................GCA.....................ACAGCAUUU.UAUGAACAUCGUUAGGUC.U.UUGAA.GAUGUUCA...UAG...GGUGCUGAUUUUGUAUAAAAAAGGAGUAGGAGUAUGUCACAAUUU
env-1872  AAGUUUAUAUAAUAAAGUACUGCCACCGGGCAGG.A.U.A.AU..............GCAG....................AAGCAUUCA.UGGACAUAUCGGUAGGUC.U.UAGGA.GAUAUGUG...UAU...GGGUGCUUUUUGCCUUAUGGAAAGAGGGGGAAAUAUGCAGCU...
env-1873  GGGCAAAGUAAAAAAUUUUUUGCCGCCGGGUAAA.C.G...................AA......................GCGUUGA.AUUUUGUAUCGACAGGCC.U.UUGAA.GAUACAAA...AAG...CUCAGCGCUUUUUUCUGUCUAUGAAAGGAAGGCAUGUCUCUGAUG
env-1875  AGUUAAAUUAAAAUUGAUUUUGCCGCCGGGUAAA.U.U.A.AA.......................................AGCAUUGU.AAUUCAUAUCGUUAGGCC.A.UAGAA.GAUAUGAA...AUUA..UAAUGCUUUUUAUUUUUAAUAAUAUUAAUUCAAAAUUUUAUUUAG
env-1876  UUAAAAAGAUUAUUCUACUUUGCCGCCGGGCAAA.G.U.G.................UUGAUU..................AAGCGUUUUUGUUUCAUACCGAUAGGCC.U.UAGAA.GGUAUGAAG..CUA...AAGCGCUUUUUAUUUUGGAGGAAAAAGAGUGAAGAAAAAACACUC
env-1877  UUAAAAAGAUUAUGUUAUUUUGCCGCCGGGCAAA.G.U.U.UU..............AAAUA...................AAGCGUUUUUGUUUCAUACCGUUAGGCC.U.UAGAA.GGUAUGAA...GCUAA.AAGCGCUUUUUUAUUUUUGGAGGAAAUUAUGGAACAAGUAUGUAUU
env-1878  CUGAUCUAUAGACAAGAAUUUGCCGCCGGGUAAA...............................................CGCUUA.UUAUCACUCCAUUAGGCC.U.UAGAA.GGAGUGAC...AAU...AGGCGUUUUUUGUUUGCUGAGGCGGUAACCGCAGAUUGAUUAACC

          ........................<<<..............................................................................>>>.........................................................................
          ........................20...............................................................................02..........................................................................
          .............<<<<<<<<<<<....>>>>>>.>.>.>.>>......<<<<<<<<...........>>>>>>>>.....<<<<<<<<<.<<<<<<<<<<.................>>>>>>>>...>>>...>>>>>>>>......................................
          .............22222222222....222222.2.2.2.22........1111.............1111.......222222222.2222222222.................22222222...222...22222222......................................
          ●●●●●●●●●●●●●●●●●●●YUGCCRCCGGGUAR●-○-○-○-○-------------○○○------------------○○R●●●Y●●-●●●●YRY●YCRUUAGGYC-●-U●GAA-GR●RYR●●---●●●---●R●●●YUUUUUUY●●●●●●●●●●●●●●●●●●●●●●●●●●●●●●●
          5′                                                                                                                                                      3′
```

```
env-1879  ACACGGUAAUCAUAACGAAUUGCCGCCGGGUAA....................................................CGCCUU..UUUCACUCCGAUAGGCC.U.UAGAA.GGAGUGAA...GCA...GGGCGUUUAUUUUUUUGUUUAGGAGGUUUUGGAUGUUCAGAGAA
env-1880  ACACGGUAAUCAUAACGAAUUGCCGCCGGGUAA....................................................CGCCUU..UUUCACUCCGAUAGGCC.U.UAGAA.GGAGUGAA...GCA...GGGCGUUUAUUUUUUUGUUUAGGAGGUUUUGAUAUGUUCAGAGAA
env-1881  GAAGAGUUAUGAAGAAUGUUUGCCGCCGGGUAA....................................................CGCCUG.A.UCCAUUCCGAUAGGCC.U.UAGAA.GGAAUGGA...GCC...AGGCGUUUUUUUGUGGGAUAAGACCGGCGGGACAAGGAAAACUUC
env-1882  ACAAAAUUAUAAAAAAUGAUUGCCGCCGGGUAA....................................................CGCCUU..AUUCAUUCCGAUAGGCC.U.UAGAA.GGGAUGAA...UCA...AGGCGUUCUUUCUUUUGCCCGGAGAGAAAGGAAGAAACUAGAAAA
env-1884  UAUAGAAUAAAAAGAUAUGUUUCUGCCGAGAAAC.G...................................................CUUA.UUCUUACUCCAGUAGGCC.A.UAGAA.GGAGUAAA...AAU...AAGAGUCUAUUUUUUUGUGGAGGUCAAUAUGAUAUUUCGAGAAAU
env-1885  UAUCUUCUUAUAAACAGAAUUUCCGCCGGGAAAC.G.C..................................................UUA.UUUUUACUCCGUUAGGCC.A.UUGCA.GGAGUAAG...GAU...AAGCGUUUUUUGUUUUAUCGGCAUCAGUUUUAACAGAUUUUAAGG
env-1886  UAAGCUACUUUACAAUACAUUUCCGCCGGGAAA...................................................ACGCUUA.UUUUUGCUCCGUUAGGCC.A.UUGCA.GGAGUAAA...CAU...AAGUGUUUAUUUUUUUGUGGAGGUCUAUAUGCUAUUUCGAGAAAU
env-1887  UAUUAUAUUAUGAAAUAAAUUUCCGCCGGGAAA...................................................AUGUUUA.UUUUUGCUCCGUUAGGCC.A.UUGCA.GGAGUAAA...AAU...AAGCGUUUAUUUUUUUGUGGAAGUUUAUAAUGUAUUUCGAGAAAU
env-1888  UAUUAUAUUAUGAAAUAAAUUUCCGCCGGGAAA...................................................AUGUUUA.UUUUUGCUCCGUUAGGCC.A.UUGCA.GGAGUAAA...AAU...AAGCGUUUAUUUUUUUGUGGAAGUUUAUAAUGUAUUUCGAGAAAU
env-1889  AUAUUCUAUUAAAAAUGAAUUUCCGCCGGGAAA...................................................ACGCUUA.UUUUUGCUCCGUUAGGCC.G.UUGAA.GGAGUAAA...AAU...AGGCGUUUAUUUUUUUGUGGAGGGCUAUAUGGUAUUCCGAGAAAU
env-1890  AUAUUCUAUUAAAAAUGAAUUUCCGCCGGGAAA...................................................ACGCUUA.UUUUUGCUCCGUUAGGCC.G.UUGAA.GGAGUAAA...AAU...AGGCGUUUAUUUUUUUGUGGAGGGCUAUAUGGUAUUCCGAGAAAU
env-1891  AUAUUAUAUUAAAAAUAAAUUUCCGCCGGGAAA....................................................CGCUUA.UUUUUGCUCCGUUAGGCC.A.UUGCA.GGAGUAAA...AAU...AAGCGUUUUGUUUUUGUAACAUGCGGUAAGCUAAAAAUGGAUAGG
env-1892  AACCAUUGCAGGAAAUACUUUGCCGCCGGGCAAA...................................................CGCUUG.UUCCCGCUCCGUUAGGCC.G.UAGAA.GGAGUGGG...GAU...AAGCGUUUUUUACGUUCCGGGCACGCGGGGGAGUGCUUGCCCGCA
env-1893  GAUUCAAUACAAUCACUAUUUACCGCCGGGUAAU.......................C........................AAGCAUUGA.AGUUCAUAUC.GUAGGCC.U.UUGAA.GAUAUGAG...CAU...CGAUGCUUUUUUUUGGGAGGAUCAAACAAUGUUCAAAACAUUUUG
env-1894  GAUUCAAUACAAUCACUAUUUACCGCCGGGUAAU.......................C........................AAGCAUUGA.AGUUCAUAUC.GUAGGCC.U.UUGAA.GAUAUGAG...CAU...CGAUGCUUUUUUUUGGGAGGAUCAAACAAUGUUCAAAACAUU...
env-1895  GAUUCAAUACAAUCACUAUUUACCGCCGGGUAAU.......................C........................AAGCAUUGA.AGUUCAUAUC.GUAGGCC.U.UUGAA.GAUAUGAG...CAU...CGAUGCUUUUUUUUGGGUAGGAUCAAACAAUGUUCAAAACAUUUU
env-1896  CUGAAAAAAUAGAGAAAAGCUGCCACCGGGUAGC.U.G.U.................GCAA.....................AAGCACGUA.AAACCACAUCGUUAGGUC.U.UAGAA.GAUGUGGC...UU....ACGUGCUUUUAUAUUGGAAGGAGAAACAGGUAUGAAUAUUCCUUU
env-1897  CCGCCUAUCUCAAACCCCUUUGCCGCCGGGUAAA.G.....................CAACU....................AAGCACUAA.GCUUCAUAUCGUUAGGCC.U.UAGAA.GAUAUGGA...GAAC..UGGCGUUUUUGUGGAAAUUUGAUGAAGAAUGCAAGCAUUUU....
env-1898  CAAAUGUAUGGGAUACAUUUUGCCACCGGGUAAA.U.A.A.UG..............CAA......................AAGCAUUUA.UGAUCGCUUCGAUAGGUC.U.UUGAA.GAAGCGUU...CAU...AGGUGCUUUUUUAUUUAAGAAGUAAAAGCAAUAACGGAACUGCGU
env-1899  AUAUUAUAAUUUUAUCUAAUAACCGCCGGGUUUA.C.G...................U............................CUUA..UCCCCAUUUCGUUAGGCC.A.UGGAA.GGAGUGGA...GGA...CAAGGCGCUUUAUUUUUGUGAAGGAGUUACCUUAUGUUUCGAGAA
env-1900  UUACUAUCAGAAAAUAGGAUUGCCGCCGGGUAA..................................................CGUUUUG..UC.UCAUUCCAUUAGGCC.A.UAGAA.GGAAUGGA...GAA...CAAAGCGUUUUUUUUAUCUGCAUUCAGGCAAACGCAUGGUAAUCU
env-1901  GACAAAACUAAAUCAUGGAUUGCCGCCGGGUAAC.G.U..................................................CUC.AAUUCAUUCCGUUAGGCC.U.UAGAA.GGAGUGAU...GCG...UGAGGCGUUUUAUUAUGGCCAAAAAAGGGAAGGAAAGCUAUGUUU
env-1902  AAUCCCAAACAAGCGAGAAAUGCCGCCGGGUAU...................................................GCUUUUU.GC.UCAUUCCGUUAGGCC.U.UAGAA.GGAAUGUG...GCA...GGAGGCGUUUUUUUAUGCCUCCGAAAUUGAAAACCCCGCCAAUGG
env-1903  AAUAUGAAUGGAUACUUCUUUGCCGCCGGGUAAA.G.A...................AUAA.....................AGUGUCAGG.CUUUCUAUCC.UUAGGCC.U.UUGGA.G.AUAGAAA..GUU...CUGGCACUUUUUUAUUGGAUUUUAGGAGGGAGUAAAUGAAACAGA
env-1904  GUAAAUACAAGAUACAUGUUUGCCGCCGGGUAAA.U.A.U.GU..............UA.......................AGCGUCAGU.UAUCCUAUCC.GUAGGCC.UUUUGGA.GAUAGGAC...AUU...CUGGCGCUUUAUUUUUUAGGAGGACUAUAUGAAAAAUAAAUCUCA
Par-1-1   AAUAUUAAAAAUAACAAAGCCAUCACCGGGUGGC.......................A........................AAAAGCUGU.UAACAAUUCCGUUAGGUC.A.UUGAA.GGAAUUGU...UAG...GAGCUUUUUUAUUUUAUAUAGGAGGAAUUUGAAAUGAAUUGGAUU
env-1905  UAAAAUAUUAAAAAGUUAGGCGUCACCGGGCGCU.U.A.A.C...............GGAUGCG..................AAAGCAUUU.UAUCCGUGUCGUUAGGUC.U.UUGAA.GAUACGGA...UAA...AAUGCUUUUUUUCAGGAGCGGGUAUGCGUAGUUUUUCAAAAGUA
env-1906  UAAAAUAUUAAAAAGUUAGGCGUCACCGGGCGCU.U.A.A.C...............GGAUGCG..................AAAGCAUUU.UAUCCGUGUCGUUAGGUC.U.UUGAA.GAUACGGA...UAA...AAUGCUUUUUUUCAGGAGCGGGUAUGCGUAGUUUUUCAAAAGUA
env-1907  AAUGUUAAAAUAUGAAAAAUUCCCGCCGGGGAAA.U......................C............................GCAU.GAAAUAUAUCAUUAGGCC.U.UAGAA.GAUAUAUU...CCA...UGCUUUUUAUUUUUAAAUCAGGAGAAAUUAUGGAUAUAAGAUUAA
Efa-293-1 AAUACAUAAACUUUCGUAAACAUCGCCGGAUGCU.U.G.C.GA..............CUA......................AAGUGUUAA.UGAACAGUUC.UUAGGCC.A.UAGCU.GAACUGUU...UGU...UGGCACUUUUUUUGUUUGGAAGGGAAGGAAAACAAGAUGAGUAAA
Efa-66-1  AAUACAUAAACUUUCGUAAACAUCGCCGGAUGCU.U.G.C.GA..............CUA......................AAGUGUUAA.UGAACAGUUC.UUAGGCC.A.UAGCU.GAACUGUU...UGU...UGGCACUUUUUUUGUUUGGAAGGGAAGGAAAACAAGAUGAGUAAA
Eca-2-1   AUCAAAUAACUUUUGAUGAACAGCGCCGGCUGUU.U.A.U.CA..............GUAA.....................AAGUGUGAA.UCGACAAUUC.UUAGGCC.G.UAGAUGGAAUUGUU...GAU...UGACACUUUUUUUAUUGGUUUGUGAUCAGUCACAUUGUUUCUCUU
Efa-286-1 AAUACAUAAACUUUCGUAAACAUCGCCGGAUGCU.U.G.C.GA..............CUA......................AAGUGUUAA.UGAACAGUUC.UUAGGCC.A.UAGCU.GAACUGUU...UGU...UGGCACUUUUUUUGUUUGGAAGGGAAGGAAAACAAGAUGAGUAAA
Efa-202-1 AAUACAUAAACUUUCGUAAACAUCGCCGGAUGCU.U.G.C.GA..............CUA......................AAGUGUUAA.UGAACAGUUC.UUAGGCC.A.UAGCU.GAACUGUU...UGU...UGGCACUUUUUUUGUUUGGAAGGGAAGGAAAACAAGAUGAGUAAA
Lli-2-1   UAAAAUAAUUUAAUAUUAAUCGCCACCGGGCGAG.................................................AAAGCAUU.UAUAAAGUUCAUUAGGUC.A.AUGAA.GAACUUUA...UAG...GUGCUUUUAUUUUUUUAAGGAGAAUAAAUAAAAUGACGUGGAUUU
          ........................<<<...............................................................................>>>.......................................................................
          ........................20................................................................................02.......................................................................
          .............<<<<<<<<<<<....>>>>>>.>.>.>.>>......<<<<<<<<...........>>>>>>>>......<<<<<<<<<.<<<<<<<<<<.................>>>>>>>>...>>>...>>>>>>>>....................................
          .............22222222222....222222.2.2.2.22........1111.............1111........222222222.2222222222.................22222222...222...22222222....................................
          •••••••••••••••••••YUGCCRCCGGGUAR•-∘-∘-○-○---------------∘○○----------------------∘∘R•••Y••-••••YRY•YCRUUAGGYC-•-U•GAA-GR•RYR••---•••---•R•••YUUUUUUY•••••••••••••••••••••••••••••••
          5′                                                                                                                                                   3′
```

alignment positions 260 · · · 439

```
env-1     ....................................................................................................................................................................................
Csp-12-1  GUUUAAUGAAAAGUAAAAAAAAUAUCAUUGUAUUUAGCAAAGGCGCUAUGAUUUUAAAUCUUUAAAUCAUAGUGCCUUUAUUUUUUAUAUAACAAGGAGUGAAGAAAUUAUGAAAAUGAUAAAGGCAAUAAUUCGACCAGAGAAGGUAGCGGACGUGUU.....................
env-2     ACUAAAAAAUUAACUAUUUCUCCAAAAAUUUGGCAACUUAUACCCGAGCUUGAAGUCGGAGUUUAUCACCAGUGAUCAUUUUGAUCGUAAAGAUAGCAU.................................................................................
env-4     AUCCAUAAAGAAUAGCCGGUCUUCUCUCUGAAUUUCAUCGUUUGAUAACCAUAUCUGCGCCCGCUCAUGCGGGAAGAAAUGGAGCGAUGCGUAUAUGCUGUAUUCCAGAAAUGAAGAGAGGGACGAAGUAUGAAAAUCGGCAUUCAGAUUGAAUCAGAGCUUUUAUGGUACGGGGAAGAG
Lga-2-1   AACGCUAGCUCAAUAAUCUUUUCGAAAGAGGUGUAAUAUGUCGUGGAUUUUUUUAUUUCUAGCGGGAAUCUUUGAAGUUGUUUGGGC.............................................................................................
env-5     AACGCUAGCUCAAUAAUCU.................................................................................................................................................................
env-6     AACGCUAGCUCAAUAAUCUUUUCGAAAGAGG.....................................................................................................................................................
env-7     GCUCCUUAGAAGCAAGUUAAUUUUUUGAAAGAGGUAUGAUAUGGCAUGGAUUUUCUUAUUUCUUGCGGGAAUAUUUGAAGUUAUUUGGGC..........................................................................................
Lga-4-1   AAACUGACGAAAUUUUUUGGAGAAAGAGGUGUAUUAUGGCGUGGAUUUUUUUAUUUCUUGCAGGCAUUUUUGAAGUAGUUUGGGC...............................................................................................
env-12    UUAAUCAUUAUUUUUUCAAACACACUUUA.......................................................................................................................................................
env-13    GAACAUCAAUUGGUUAUUAUUAAUUAUCGGGGGAGUUUUUGAAUCACU....................................................................................................................................
env-14    GAACAUCAAUUGGUUAUUAUUAAUUAUCGGAGGGAUUUUUGAAUCACU....................................................................................................................................
env-15    UAACGUCUUUUUUGUUUUUUGUUCCGAUUGUGUAUCUGGGAAAAAGUCAGACAGUUUGAAAAUAUGAAAUUUAUUAUGAGGUGAUAUUUAUGAAAAUGAUCAAAGCAGUCGUAAGACCCGAAAAAUCAUCAGAAGUACU.........................................
[sc-1-1   AAGGAGAGACUUUUUGAAAUAUGUGAGCCUGAAUGUGAUCGG..........................................................................................................................................
env-17    AAGGAGAGACUUUUUGAAAUAUGUAAGCCUGAAUGUGAUCGG..........................................................................................................................................
env-21    AAGGAGAGACUUUUUGAAAUAUGUAAGCCUGAAUGUGAUCGGGAUGAUCGGAAUAUCC..........................................................................................................................
env-22    AAGGAGAGACUUUUUGAAAUAUGUAAGCCUGAAUGUGAUCGG..........................................................................................................................................
env-23    UCAAGCCAGCUCUUUUUCCGCUUUGCGGUGCCCCA.................................................................................................................................................
env-24    AUUAAUCGUU..........................................................................................................................................................................
env-25    UUUACUAAUCGUUACAGCAAACACCGU.........................................................................................................................................................
env-26    UUUAUUAAUCGUUACAGCAAAUACAGU.........................................................................................................................................................
Lbr-1-1   UGGUUAUGGUUAGUGAUUGCAGGUUUGUUUGAAGUUGUGUGGGC........................................................................................................................................
Lsa-3-1   ....................................................................................................................................................................................
env-28    UGAUUGCUGGCUUUUUUGAAGUUUUNUGGGC.....................................................................................................................................................
env-29    UGAUUGCUGGCUUUUUGAAGUUUUCUGGGC......................................................................................................................................................
Wbo-1-1   UAUUUAUCGCUGGCCUUUUUGAGGUUUUUGGGC...................................................................................................................................................
Wjo-1-1   CUUGGUUAUUGCAGGCUUUUUUGAAGUUUUUUGGGC................................................................................................................................................
env-30    AAGUGAACUUUUUAAGGUACGUGAGUUUGAACAUUCU...............................................................................................................................................
Lag-1-1   CUUUACUUAUUGCCGGCUUAUUUGAAGUAGUGUGGGC...............................................................................................................................................
env-31    ....................................................................................................................................................................................
Lpl-1-1   UUUAUUUAAUUAUUGCUGGAUUAUUUGAAGUGGUAUGGGC............................................................................................................................................
Lcu-1-1   GGAUUGAUUUAUUUAUCGCAGGAUUAUUUGAAGUUUUUUGGGC.........................................................................................................................................
Lki-1-1   UUAUCGCAGGACUUUUUGAGGUUUUCUGGGC.....................................................................................................................................................
env-32    CGACGAUUUGGGCGAGCUUUUUUGACAGCCU.....................................................................................................................................................
Csp-20-1  CGCGGCUCUGACCGCGAGCUUCUUCGACUGUCU...................................................................................................................................................

          ....................................................................................................................................................................................
          ....................................................................................................................................................................................
          ....................................................................................................................................................................................
          ....................................................................................................................................................................................
          •••••••••••••○○○○○○○○○○○○○○○○○○○○○○○○○----------------------------------------------------------------------------------------------------------------------------------------------
```

env-33	UUAGGUACUACUGUUACAGCAAGCUUUUUUGACAGCUU...
env-34	GCGGACAUCAAAAGCUUUUCAAAAUAUGUCUCUCU...
env-35	AUAUAAUGAAAACCUUUAUAAAGUACGUGUCCAUGAACAUCAU...
env-36	UACUUAAUUUUUGCUGGUUUGUUAGAGUGCGUAUGGUCCACAACGAUGAAAUUAAGUAAUGGUUUUCACAUUUAUAGUGGUCAAUUGCGACAAUUAUUGGGUUAGUACUUAGCUUUUUAUUUUUAGCAUUAUCUUUAAAGCGACUUGAUAUUUCACUAGCAUACCCGAUUUGGACCGGA
env-37	UAAAAAAGACUUUUUCCAAUACACCUCUUUGGGGAU...
env-38	UAAAAAAGACUUUUUCCAAUACACCUCUUUGGGGAU...
env-39	UGUUUCACUUGACUAAAAUCAGGAGGAUAUUUCCAUGGAUCAGACGAAUUCCAUUUUACGCACCUUUUUCAAAUAUGUCUCCGC...
Esp-14-1	GAAAUUAUGAUUAUGGAACAGCAAAAUUAUUUUAAAGAAUUUGCCAAGUAUACCUCGUUAAA...
Esp-15-1	AGAAAUUAUGAUUAUGGAACAGCAAAAUUAUUUUAAAGAAUUUGCCAAGUAUACCUCGUUAAA...
env-40	AGAAAUUAUGAUUAUGGAACAGCAAAAUUAUUUUAAAGAAUUUGCCAAGUAUACCUC...
env-41	CUUUUCAUUGCAGGAAUUUUUGAGAUCGUUUGGGCCGUUGCCUUGAACCCAAACUCCCGUGAAAUAGCCCGUGAUUAUUUUUAAUCGGGCUAUUUUUCUUCAACUAAUUCUCUGAUUCCUUUAAACAAAAGAUUUCAAUGACGGCAUUUUUAUGCUAUAAUAGAUCGGUAUUUUAAUAGU
env-42	CUUUUCAUUGCAGGAAUUUUUGAGAUCGUUUGGGCCGUUGCCUUGAACCCAAACUCCCGUGAAAUAGCCCGUGAUUAUUUUUAAUCGGGCUAUUUUUCUUCA...
env-43	CUUU...
env-44	CUUUUCAUUGCAGGAAUUUUUGAGAUCGUUUGGGCCGUUGCCUUGAACCCAAACUCCCGUGAAAUAGCCCGUGAUUAUUUUUAAUCGGGCUAUUUUUCU...
env-45	CUUUUCAUUGCAGGAAUUUUUGAGAUCGUUUGGGCCGUUGCCUUGAACCCAAACUCCCGUGAAAUAGCCCGUGAUUAUUUUUAAUCGGGCUAUUUUUCUU...
env-46	CUUUUCAUUGCAGGAAUUUUUGAGAUCGUUUGGGC...
env-47	CUUUUCAUUGCAGGAAUUUUUGAGAUCGUUUGGGCCGUUGCCUUGAACCCAAACUCCCGUGAAAUAGC...
env-48	CUUUUCAUUGCAGGAAUUUUUGAGAUCGUUUGGGC...
env-72	CUUUUCAUUGCAGGAAUUUUUGAGAUCGUUUGGGCCGUUGCCUUGAAAUAUGCCGAUGGCUUUAGUAAUUAGUGCGGUCACUAUUAUCGGAAUGGUCGCCAGCGUCUAUUUCUUGGCAGUAGCGAUGAGAACUAUUCCUUUAGGAACGGCUUACGCCAUUUGGACCGGUAUCGGGUGUAU
env-73	CUUUUCAUUGCAGGAAUUUUUGAGAUCGUUUGGGCCGUUGCCUUGAACCCAAACUCCCGUGAAAUAGCCCGUGAUUAUUUUUAAUCGGGCUAUUUUUCUUCAACUAAUUCUCUGAUUCCUUUAAAUAAAAGAUUUCAAUGACGGCAUUUUUAUGCUAUAAUAGAUCGGUAUUUUAAUAAU
env-74	CUUUUCAUUGCAGGAAUUUUUGAGAUCGUUUGGGCCGUUGCCUUGAAAUAUGCCGAUGGCUUUAGUAAUUAGUGCGGUCACUAUUAUCGGAAUGGUCGC...
env-75	GCCCGUGAUUAUUUUUAAUCGGGCUAUUUUUCUUCAACUAAUUCUCUGAUUCCUUUAAAUAAAAGAUUUCAAUGACGGCAUUUUUAUGCUAUAAUAGAUCGGUAUUUUAAUAAUAUAGGUCUAUUAAAAUGGGUCGCCCAAGAGUUUAUGACACUCCGCACUUCUGCCUCCAUACUUA..
env-76	CUUUUCAUUGCAGGAAUUUUUGAGAUCGUUUGGGCCGUUGCCUUGAACCCAAACUCCCGUGAAAUAGCCCGUGAUUAUUUUUAAUCGGGCUAUUUUUCUUCAACUAAUUCUCUGAUUCCUUUAAAUAAAAGAUUUCAAUGACGGCAUUUUUAUGCUAUAAUAGAUCGGUAUUUUGAUAAU
env-77	CUUUUCAUUGCAGGAAUUUUUG...
env-78	CUUUUCAUUGCAGGAAUUUUUGAGAUCGUUUGGGCCGUUGCCUUGAACCCAAACUCCCGUGAAAUAGCCCGUGAUUAUUUUUAAUCGGGCUAUUUUUCUUCAACUAAUUCUCUGAUUCCUUUAAACAAAAGAUUUCAAUGACGGCAUUUUUAUGCUAUAAUAGAUCGGUAUUUUAAUAAU
env-79	CUUUUCAUUGCAGGAAUUUUUGAGAUCGUUUGGGC...
env-80	CUUUUCAUUGCAGGAAUUUUUGAGAUCGUUUGGGC...
env-104	ACCUUUUCAUUGCAGGAAUUUUUGAAAUCGUUUGGGC...
env-110	AGAACCUGACAAAACGUGAAUGGUUGCUUUG...
env-111	AGAACCUGACGAAACGGGAAUGGGUGCUUUG...
env-112	UUCGUUCGCUGAAAAAUCUCACAAAGGGAGAGAUAUGCCU...
env-113	UCAUGGAGGAAAUCAGGGAUGGAAAUCAGAGGAGAUUUUAAAACAUUUUCUAAAUAUGUUAGUUUAAA...
env-114	UUGCUGAUUGCCGGUAUACUGGAGGUUUUCUGGGC...
Rpe-1-1	GCAGAAAGGUGGAAUUUUUAUGGAAAGUAAAAGUUUGGGAAGGGUAUUUGCAAAGUAUGUAUCUUUGAA...
env-116	GCAGAAAGGUGGAAUUUUUAUGGAAAGUAAAAGUUUGGGAAGGGUAUUUGCAAAGUAUGUAUCUUUGAA...
Rsp-1-1	GCAGAAAGGUGGAAUUUUUAUGGAAAGUAAAAGUUUGGGAAGGGUAUUUGCAAAGUAUGUAUCUUUGAA...
Lsp-2-1	UUUUUUUAGUAAUUGCUGGUUUAUGUGAGGUCUUCUGGUC...
Lse-1-1	CAAUGUUAGGUUUUGGCACUGUUGGACAAGCAGUUUGGCA...
env-117	UUAUGAAGUAUGUUAUUUUAAAUGUUAU...
env-118	UUGCUGCAGGUAUCUUUGAAGUCGUCUGGGC...
env-119	UUGCUGCAGGUAUCUUUGAAGUCGUCUGGGC...
Loz-1-1	UUAAUUAUUGCUGGGUUAUUAGAAGUAUGUUGGUC...
env-120	AUUGUUUGCGGCAGGUGCGCUGGAGGUUCUCUGGUC...
env-121	AGAUAUUAUGGCAUGGGUUUACUUAGUUUUUGCAGGGCUUCUUGAAGUUUUCUGGUC...
env-122	UAGUAAUGAGAGAUAAGAUUUCUAUGAUAAAUAUUCUAAUUUGUAUUCCUUUAUGUAGAAAUUGGAGGUGGUAUUAUUUCACAAUCAUAUUUAAAAGAAUUUACUCGAUAUGUAGUAUCAAAUAUUAUAGGAAUGAUAGGUUUUUCAGGUUAUAUUUUGGUGGAUACCUUUUUCAUUUCU
Bpu-1-1	GUUCCGGGAAUUCGCCCGGUAUGCUUCGCUCAACGCACU...
env-123	GUGAUUGCCGCCGCGUUUGAAGUCGGCUGGCC...
env-129	UUAAUCAUCGCCGCCGCGUUCGAAGU...
env-131	UUAAUUAUUCUUUGGAUUCUAGUUUCAAUCGUCUAUAUUAACUAUUUUCCAAUGAUUGUAUGCUUUUAAAAACUGGAAGCAUUUGAAAAAAUCUCAAAAUCUAUCA...
env-132	UUAAUUAUUCUUUGGAUUCUAGUUUCAAUCGUCUAUAUUAACUAUUUUCCAAUGAUUGUAUGCUUUUAAAAACUGGAAGCAUUUGAAAAAAUCUCAAAAUCUAUCAAUCAUAGUACAAAUUGAAUAGUAUAGUAGUGAACUACUCCCACCACUUAAUGAAAAAUCAUUAGAAGUGGGGGC
Ali-1-1	UAUUUGGUUAUUGCCGGCAUGUUUGAAACAGUUUGGGC...
env-133	UAUUUGGUUAUUGCCGGCAUGUUUGAAACAGUUUGGGC...
env-134	UUUCUUAUUAUUGCGGGGAUUUUGAAGCCUU...
Lin-1-1	UAUUUAAUAGUUGCUGGCAUUUUCGAAGUUGUAUGGGC...
Ljo-2-1	UCUAUUUCA...
Ljo-1-1	UCUAUUUCACGAUCAGUUUUCACUAAUAGCAUGGUUAUUUAUCGUUUUAUUAGUUAUUAGUAUAAUUGGGAUAAAAUUAACACCAGGGCAUUAAGAAAGAAAAAUGAGAAAAGUAAGUUUAUUUAUUGCGAUGAGUCUUGAUGGUUAUAUUGC...
env-135	UCUAUUUCACAAUCAGUUUUAACUAAUAAUAUGGUUAUUUAUCGUUUUAUUAGUUAUUAGGAUAAUUGGGAUAAAAUUAAUACCAGGGCAUUAAGAAAGAAAAAUGAGAAAAGUAAGUUUAUUUAUUGCGACGAGUCUUGAUGGUUAUAUUAC...
env-136	CAAAAGUUUAUUCAUUAUGUCUCAGCCAAUAUGAUUUC...
env-138	UGGAUUUGCCUGUUGGCGGCCGGAUGCCUGGAGAUGGUCUGGGU...
Fpl-1-1	GUGAAGCGGAGCUUUUUCCAGUAUGUGAGCCUGAAUGUGCUGGGCGCGCU...
Fsp-1-1	ACAUUUUUCAAGUACAUCUCCUUAAAUAUGCUGGGGGCCCU...
env-140	GGUACGUACCUUUUUUAAAUAUAUCUCGCUCAAUAUGCUGGGCGCGCUGGGGCUGUCUGGCUACAUCAUGGCCGACACCUUCUUUGUAGCCAACCGGCUGGGGGCGGACGGCCUGGCGGCCCUCAAUCUGGCCAUUCCCAUUUUCGGCCUUAUCAACGGGUUGGGCGUCCUACUGGGCAU
env-141	AUCUUAUAUUAGCAGGAUUGUUUGAAGUGGCUUGGGC...
env-143	AUCUUAUAUUAGCAGGAUUGUUUGAAGUGGCUUGGGC...
Lla-20-1	AUCU...
Lla-4-1	AUCU...
Lla-21-1	AUCU...
env-144	AUCUACUAAUAGCAGGAAUUUUUGAAGUGGUUUGGGC...
env-145	UUUACUUAUAGCGGGAACAUUUGAAGUGGCUUGGGC...
env-146	UUU...
env-150	UUUACUUAUAGCAGGAACAUUUGAAGUGGCUUGGGC...
Lla-1-12	UUU...
env-151	...
env-152	UUAUCGCAGGAAUUCUUGAGGUCACAUGGGC...
env-153	UGGCCGAUAGGGGUCUUGGUUUUAGCCAAUAUCUU...
env-154	GGCAUGGGUAUUUCUGGCAUGCGCGGGACUUCUGGAGGUGUUCUGGUC...
env-155	GGCAUGGGUAUUUCUGGCAUGCGCGGGACUUCUGGAGGUGUUCUGGUC...
env-157	GGCAUGGGUAUUUCUGGCAUGCGCGGGACUUCUGGAGGUGUUCUGGUC...
env-158	GGCAUGGGUAUUUUUGGGUGCGCGGGACUUCUGGAGGUGUUCUGGUCCUCGUUCCUGAAACU...
env-159	...
env-160	GGCAUGGGUAUUUCUGGCAUGCGCGGGACUUCUGGAGGUGUUCUGGUC...
env-161	GGCAUGGGUAUUUCUGGCAUGCGCGGGACUUCUGGAGGUGUUCUGGUC...
	...
	...
	...
	...
	●●●●●●●●●●●●●○○○○○○○○○○○○○○○○○○○○○○○○○———

```
env-164    GGCAUGGGUAUUUCUGGCAUGCGCGGGACUUCUGGAGGUGUUCUGGUC...
env-165    GGCAUGGGUAUUUCUGGCAUGCGCGGGACUUCUGGAGGUGUUCUGGUC...
env-166    GGCAUGGGUAUUUCUGGCAUGCGCGGGA...
env-167    GGCAUGGGUAUUUCUGGCGUGCGCGGGACUUCUGGAGGUGGUCUGGUCCUCGUUCCUGAAGC...
env-168    GGCAUGGGUAUUUCUGGCAUGCGCGGGACUUCUGGAGGUGUUCUGGUC...
env-169    GGCAUGGGUAUUUCUGGCAUGCGCGGGACUUCUGGAGGUGUUCUGGUCCUCGUUCCUGAAACUGUCCGAUGGCUUCACGAAGCUGGGCUGGUCCGUGAUGACCAUUGCGGGGAUGCUGGCCAGCUUCUAUUUGCUGAGCUAGGCCAUGAAGACGCUUCCCCUGGGCACUGCCUACGCCGU
env-170    GGCAUGGGUAUUUCUGGCAUGCGCGGGACUUCUGGAGGUGUUCUGGUC...
env-172    GGCAUGGGUAUUUCUGGCAUGCGCGGGACUUCUGGAGGUGUUCUGGUC...
env-173    GGCAUGGGUAUUUCUGGCAUGCGCGGGACUUCUGGAGGUGUUCUGGUC...
env-231    GGCAUGGGUAUUUCUGGCAUGCGCGGGACUUCUGGAGGUGUUCUGGUC...
env-232    GGCAUGGGUAUUUCUGGCAUGCGCGGGGCUUCUGGAGGUGUUCUGGUC...
env-233    GGCAUGGGUAUUUCUGGCAUGCGCGGGACUUCUGGAGGUGUUCUGGUC...
env-234    GGCAUGGGUAUUUCUGGCAUGCGCGGGACUUCUGGAGGUGUUCUGGUC...
env-235    GGCAUGGGUAUUUCUGGCAUGCGCGGGACUUCUGGAGGUGUUCUGGUC...
env-239    GGCAUGGGUAUUUCUGGCAUGCGCGGGACUUCUGGAGGUGUUCUGGUC...
env-240    GGCAUGGGUAUUUCUGGCAUGCGCGGGACUUCUGGAGGUGUUCUGGUC...
env-242    GGCAUGGGUAUUUCUGGCAUG...
env-243    GGCAUGGGUAUUUCUGGCAUGCGCGGGACUUCUGGAGGUGUUCUGGUCCUCGUUCCUGAAACUGUCCGAUGGCUUCACGAAGCUGGGCUGGUCCGUGAUGACCAUUGCGGGGAUGCUGGCCAGCUUCUAUUUGCUGAGCCAGGCCAUGAAGACGCUUCCCCUGGGCNNNNNNNNNNCCCC
env-244    GGCAUGGGUAUUUCUGGCAUGCGCGGGACUUCUGGAGGCGUUCUGGUC...
env-245    GGCAUGGGUAUUUCUGGCGUGCGCGGGACUUCUGGAGGUGGUCUGGUC...
env-246    GGCAUGGGUAUUUCUGGCAUGCGCGGGACUUCUGGAGGUGUUCUGGUC...
env-247    GGCAUGGGUAUUUCUGGCAUGCGCGGGACUUCUGGAGGUGUUCUGGUC...
env-248    GCAUGGGUAUUUUUGGCGUGCGCGGGACUUCUGGAGGUGGUCUGGUC...
env-249    GGCAUGGGUAUUUCUGGCAUGCGCGGGACUUCUGGAGGUGUUCUGGUC...
env-250    GGCAUGGGUAUUUCUGGCAUGCGCGGGACUUCUGGAGGUGUUCUGGUC...
env-251    GGCAUGGGUAUUUCUGGCAUGCGCGGGACUUCUGGAGGUGUUCUGGUC...
env-254    ...
env-255    GCUUGGUUAAAAGUUAUUUUUGGCGCUUUUUGUGAAGUGAU...
Efa-269-1  GCUUGGUUAAAAGUUAUUUUUGGCGCUUUUUGUGAAGUGAU...
Efa-159-1  GCUUGGUUAAAAGUUAUUUUUGGCGCUUUUUGUGAAGUGAU...
env-256    GCUUGGUUAAAAGUUAUUUUUGGCGCUUUUUGUGAAGUGAU...
env-257    GCUUGGUUAAAAGUUAUUUUUGGCGCUUUUUGUGAAGUGAU...
env-258    GCUUGGUUAAAAGUUAUUUUUGGCGCUUUUUGUGAAGUGAU...
env-259    GCUUGGUUAAAAGUUAUUUUUGGCGCUUUUUGUGAAG...
env-260    GCUUGGUUAAAAGUUAUUUUUGGCGCUUUUUGUGAAGUGAU...
env-261    GCUUGGUUA...
env-262    GCUUGGUUAAAAGUUAUUUUUGGCGCUUUUUGUGAAGUGAU...
env-270    GCUUGGUUAAAAGUUAUUUUUGGCGCUUUUUGUGAAGUGAU...
env-273    GCUUGGUUAAAAGUUAUUUUUGGCGCUUUUUGUGAAGUGAU...
env-274    GCUUGGUUAAAAGUUAUUUUUGGCGCUUUUUGUGAAGUGAU...
env-287    GCUUGGUUAAAAGUUAUUUUUGGCGCUUUUUGUGAAGUGAU...
env-294    UUUAGGGACAGUAAUUGCAAUCUUUAUCAGUUUCUAUGCGCUGAUUAAAGCAGGAGAGGAAUUGCCAGUGGGCACAGCCUAUGCUGUAUUUGUCGGCUUAGGUAGUGCUGGAACUAU...
env-295    GCUUGGUUAAAAGUUAUUUUUGGCGCUUUUUGUGAAGUGAU...
env-308    GCUUGGUUAAAAGUUAUUUUUGGCGCUUUUUGUGAAGUGAU...
env-309    GCUUGGUUAAAAGUUAUUUUUGGCGCUUUUUGUGAAGUGAU...
env-310    GCUUGAU...
env-311    ACUUGGUUAAAAGUUAUUUUUGGCGCUUUUUGUGAAGUGAU...
env-312    GCUUGGUUAAAAGUUAUUUU...
env-313    GCUUGGUUAAAAGUUAUUUUUGGCGCUUUUUGUGAAGUGAU...
env-314    GCUUGGUUAAAAGUUAUUUUUGGCGCUUUUUGUGAAGUGAU...
env-315    GCUUGGUUAAAAGUUAUUUNNNNNNNNNNNNNNNNNNNNNNNNNNNNNNNNNNNNNNNNNNNNNNNNNNNNNNNNNNNNNNNNNNNNNNNNNNNNNNNNNNNNNNNNNNNNNNNNNNNNNNNNNNNNNNNNNNNNNNNNNNNNNNNNNNNNNNUAUGCGCUGAUUAAAGCAGGAGAAGAAUUG
Efa-211-1  GUUAAAAGUUAUUUUUGGCGCUUUUUGUGAAGUGAU...
Efa-256-1  GCUUGGUUAAAAGUUAUUUUUGGCGCUUUUUGUGAAGUGAU...
Ete-1-1    AUAAUCGGCUGAAAACAGUCUCUUAGAAAAAAUACAAUGAUUGAAUGAGUAUAGCAAGCGUUCAAUCGAGACUGAACAAAAUCCAUUAUGUCUACAAAUUGAGGAGGAUUUACAUGAGUAAGGAUUGGUUAAAAUUAAUGAUUGGCGCUUUCUUUGAGGUAUU...
Ime-1-1    AGGGAGAUGAGUAGCAUGACAAAACAUUGGUUGAUGUUAAUAGUAGGCGCUUUUUUUGAGGUGUU...
Esp-4-1    AAAAAGAUAAAAAUAAAAAGGAGCAAAAAGCGCUCCUCAUAUUUUUUCUAUUUUCCAUUCAAGGCUGGACGAUUUUACUGUACUUUUAAAUUUUAAGGAGGAGUUUCUAUGAACAAAGAUUGGUUAAAAUUAAUGAUUGGUGCUUUUUUCGAAGUAUU...
Eur-2-1    CGAGUCUGCAAUGCUUUUAAAUUUAGGAGGUUUUUAGAUGAGUAAGGAUUGGAUAAAAUUAAUGAUUGGUGCAUUUUUUGAGGUUCU...
Ewa-1-1    AGGAGGAAUUGUAAUGAAUAAGGACUGGAUAAAAUUGAUCAUUGGCGCUUUUUUUGAAGUGCU...
Esi-1-1    AAAGACAUAAUGAAGAAAUGAGAGGAUGUUAAACACACCAGUAGUUGGUACAAGGAGAAGCGUUGUUACAGUUGAAUGAUCUAGCUGUAUUUUUAAAUUUUAGGAGGUAUUUUAUAUGAAUAAAGAUUGGAUAAAAUUAAUGAUCGGAGCAUUUUUUGAGGUGUU...
Eha-2-1    GACAAAAUCUGCAUGUGGCAAAGAGUACCACAACUAUUUUUUCUAUUUUCCUGUUAAGGCAGAACAAGCUCUUUCAUCUUUUAAAAUUAGGAGGUAUUUACAUGAAUAAGGAUUGGACAAAAUUAAUGAUUGGUGCAUUUUUUGAAGUAUU...
Epl-1-1    CAGUAUUGCAUGACACAAGGUGAACUAAAGCGGGACGUUGUUCCAGAUAAAAUAAGGAGGUUUUUAUAUGAACAAAGAUUGGUUAAAAUUAAUGAUUGGUGCUUUUUUUGAAGUAUU...
Emo-2-1    GAAUAAAGCUUGUAUUAAAUACAUUGAUACAUUUGUAAAUGUACAGUUAAGGUAGAAAGCGUAUUUACGGCUUUUAAAAUUAGGAGGCAUGUAUAUGAGCAAGGAUUGGAUUAAAUUAUUGAUUGGCGCUUUUUUUGAGAUUUU...
Eur-1-1    UAAAGUGAUGAGUGGUUGUUAUUUAGUUCAAGGGAAUGCAAAGUGAAACGUUAUUCCAUAUGUUAUCUAGCUUCACGGGCUAGCACUAUGUUCUGUUUCGAGUUCAACAAAUCCUAAGAGCUAAACAAGUCCGCUCGGCUUUAAAAAUUAAGGAGGUUUUUUAUAUGAACAAGGAUUGGA
env-316    GCGGAACGGAACGUUGUUACCGUUUUUAAAAUUAGGAGGUUUUUUCAUGAACAAGGAUUGGAUUAAAUUAAUGAUUGGCGCAUUUUUUGAAGUUUU...
Equ-1-1    CUAAACGUUAGGAAAAAAGUAAGGUUAGCUUGAGACAGAAAAUGUUGCAAACAUAUUUUCUUACCUUUCAAACAAUACUAAGCGAACCGAUUACGUUUUUUAAAUUAAGGAGGAUUUUUAUAUGAGUAAAGAUUGGAUAAAAUUAAUGAUUGGUGCAUUUUUUGAAGUAUU...
Eri-1-1    AUUGGUUAAAAUUAUUUUUAGGAGCAUUUUUUGAAGUAUU...
Csp-19-1   GAAUGCAAAAGAUUUUUGUAGCUGUACAGAUCACAAAUGUCCAUUACA...
env-317    AAUGGGUAUAUCUGAUUGUCGCAGGCGGCCUUGAAGUGUUCUGGUC...
env-318    AAUGGGUAUAUCUGAUUGUCGCAGGCGGCCUUGAAGUGUUCUGGUC...
env-322    AAUGGGUAUAUCUGAUUGUCGCAGGCGGCCUUGAAGUGUUCUGGUCUACCUGCCUGAAAUU...
env-323    AAUGGGUAUAUCUGCUUGUCGCAGGCGGCCUUGAAGUGUUCUGGUC...
env-325    CUGCUGAUCGCGGGGAGCCUGGAGAUCACCUGGGC...
env-327    AGUCUUUCGGGUAUCGCAAAAAGUAAGUGACCUGGCAGAAAGAGGAGCAAGGAGGAAAUAAGACAUGAAGAGAGAAUUUGGCAAAUAUGUAUCUUUGAAUAUUUUUGGAAUGCU...
env-330    ...
Cje-1-1    UUUAUUUGGUUUUUGCGGGUCUGCUGGAGGUUGUAUGGGC...
env-331    UAAAAUUAAAGCGGCUUGUGAACUAUUUUUCCAAAGGAGAAAUCUU...
env-332    UAAAAUUAAAGCGGCUUGUGAACUAUUUUUCCAAAGGAGAAAUCUU...
env-333    AUAUUUUAAAAGACUUUGCUAAAUAUGUAUCUUUAAA...
env-335    UGUCUGCA...
Lsa-2-1    GAUGAUUGCAGGAAUUUUUGAAGUAGUCUGGGC...
env-336    UUAAGGAAUUUACAAAAUGUAUCGCUUUAAGCGUUGCCGGUAUGAU...
env-337    UUCUUUUAAUCGCCGGAGUUUUAGAAGUAACAUGGGC...
```

env-339	...
env-340	GCUUGGGUCUAUCUUUUUAUUGCGGGAUUGUGUGAGGUUUUCUGGGC...
env-341	GAACAGCUUGCUAAAGGAUUUUUUUAAGUAUACAGGAUUGAACAUAGCGGGAAUGAUAGGCUUAUCUUGCUAUAUUUUGGCAGAUACAUUUUUUGUGGCACA...
env-342	GAACAGCUUGCUAAAGGAUUUUUUUAAGUAUACAGGAUUGAACAUAGC...
env-343	UACUAAAAGCC...
env-344	UACUAAAAGCCAUCAGCAUGAAUAGUCUAGGGAUGUU...
env-347	UACUAAAAGCCAUCAGCAUGAAUAGUCUAGGGAUGUU...
env-348	UACUAAAAGCCAUCAGCAUGAAUAGUCUAGGGAUGUU...
env-349	CUACUGGCGGCAGGAGCCGCAGAGGUCACCUGGGC...
env-350	CUACUGGCGGCAGGAGCCGCAGAGGUCACCUGUGC...
env-351	UACUGGCGGCAGGAGCCGCAGAGGUCACCUGGGC...
env-352	CUACUGGCGGCAGGAGCCGCAGAGGUCACCUGGGC...
env-353	CUACUGGCGGCAGGAGCCG...
env-354	ACCCGGUUUAGGCACUACGGAAAACAUCACAUCGGGCGAGGGUGCCGUGCGACAAAUAUAAAACAAAAAAAGGGGAAAAUAUAUGUUUAGAGAGAUGCGCCGAAAAAAGCAGGCUCUUAAAAGAUCCGAAUG...
env-357	CAAU...
env-358	ACAAAUCUUAUUGGAAUUUAUUUCCAGAUAGUAA...
env-359	ACAAAUCUUAUUGGAAUUUAUUUCCAG...
env-360	...
env-362	ACAAAUCUUAUUGGAAUUUAUUUCCAGAUAGUAA...
env-363	ACAAAUCUUAUUGGAAUUUAUUUCCAGAUAGUAA...
env-368	ACAAAUCUUAUUGGAAUUUAUUUCCAGAUAGUAA...
env-372	...
env-373	ACAAAUCUUAUUGGAAUUUAUUUCCAGAUAGUAAAUUAGGAGUGUUACUUUAAAAAAAUAUGCAAAAUGGAG...
env-374	ACAAAUCUUAUUGGAAUUUAUUUCCAGAUAGUAA...
env-375	ACAAAUCUUAUUGGAAUUUAUUUCCAGAUAGUAAAUUAGGAGUGUUACUUUAAAAAAAUAUGCAAAAUGGAGAAAGUACUGAUGAAAUAAAAUCAGUGUUAGAAGAAGC...
env-376	ACAAAUCUUAUUGGAAUUUAUUUCCAGAUAGUAA...
Fnu-1-1	AAAUCUUAUUGGGAAUUAUUUCCAAAUAGUAA...
env-387	UAAAUCUUAUUGGAAUUUAUUUCCAGAUAGUAAAUUAGGAGUGUUACUUUAAAAAAAUAUGCAAAAUGGAGAAAGUACUGAUGAAAUAAAAUCAGUGUUAGAAGAAGC...
env-388	UAAAUCUUAUUGGAAUUUAUUUUCAAAUAGUAA...
env-389	UAAAUCUUAUUGGAAUUUAUUUUCAAAUAGUAAAUUAGGAGUGUUACUCUUAAAGAAUAUGGAAAAUGGAGAAAGCACCAAUGAAAUAAAAAAUAUUUAACAAAGGAAGUUUUAAGUGAAAAUCCUGUUAUUGCAGUUUGGAGAGAAGCCUAUAAAAAAUUUAAAACUAAAAAAGGAGUU
env-390	UAAAUCUUAUUGGAAUUUAUUUUCAAAUAGUAAAUUAGGAGUGUUACUCUUAAAGAAUAUGGAAAAUGGAGAAAGCACCAAUGAAAUAAAAAAUAUGUUAGUGGAAGC...
env-391	UAAAUCUUAUUGGAAUUUAUUUUCAAAUAGUAA...
env-396	UAAUGG...
env-408	GAACUGGUGAUUGCGGGCGGGCUGGAGGUACUCUGGUC...
env-409	UUCAAAAGCAUAAAGCCUGUCGAAUG...
env-410	UUUAUUUGAUUUUGGCCGGCAUCUCAGAGGUCACCUGGGCCACGGCGAUGAAAUACGCGGACGGCUUCACCAAGCUUGUCCCUUCUAUAAUCACAGUCGUCGGCUACAUCGUCAGUGCCGUCUUUCUGUCCUUCGCGCUGAAAAAGCUCCCCCCUCGGCACCGCCUAUGCGAUGUGGACC
env-411	UUUAUUUGAUUUUGGCCGGCAUCUCAGAGGUCACCUGGGC...
env-412	UUUAUUUGAUUUUGGCCGGCAUCUCAGAGGUCACCUGGGC...
env-413	UUUAUUUGAUUUUGGCCGGCAUCUCAGAGGUCACCUGGGCCACGGCGAUGAAAUACGCGGACGGCUUCACCAAGCUUGUCCCUUCUAUAAUCACAGUCGUCGGCUACAUCGUCAGUGCCGUCUUUCUGUCCUUCGCGCUGAAAAAGCUCCCCCCUCGGCACCGCCUAUGCGAUGUGGACC
env-414	AAUGAGAAGAAAAAAACAGGUAAUGAGUGAGGCUGACAG...
Lsp-7-1	GCGCAGAAAAAGACAGAUGCUCUCAAAAGAAGAAUG...
env-415	GGAAAUGCGCCGCCACAAACAGCUUUUGAACGCCGAGGAGAC...
env-416	UGAUGUUAUUGCUGGCAGGAAUAUUGGAAGUGACGUGGGC...
env-417	CGUCGAAAACGGCGCUAUGGUGGGAAGUCCUUCGC...
env-418	AAUGACGAAAUAUUU...
env-420	...
env-421	UAUCUGCUGCUGGCCGCCGUGUUCGAGGUGGGCUGGGC...
Bca-1-1	GAGAGGGAUUUAUACUAUGGACGAACAGAAUCUGAACCGUACCUUUGCCCGGUACAUGUCCCUGAAUAUUCUGGGUAUGCU...
env-422	UGGAAAGGGAUGAAUCGCUAUGUUUCGAGAAAUGCGCAGGAACGGGCAAGAGCUGCCCUUGGAAGAAUG...
env-423	UGGAAAGGGAUGAAUCGCUAUGUUUCGAGAAAUGCGCAGGAACGGGCAAGAGCUGCCCUUGGAAGAAUG...
env-426	UGGAAAGGGAUGAAUCGCUAUGUUUCGAGAAAUGCGCAGGAACGGGCAAGAGCUGCCCUUGGAAGAAUGUACAGCUAUCUUAAAUCGGGGGACUUCUGGUGUGCUGGCCCUUUCGGGGGAUGACAAUUAUCCAUAUGCGGUGCCGGUCAG...
env-428	CCUCACUUUUUCUUACCUUCUGCAAAUACGU...
env-429	CCUCACUUUUUCUUACCUUCUGCAAAUACGU...
env-430	AUAGGCUUUUAAUCAAUUUUGCGAAAUACACGUUAUUUAA...
env-431	CAGUUUAUUAAAAAACUUUGCUAAAUACAC...
env-432	AAGUUUUACUUGGGAAUUUUGCGAAAUA...
env-433	ACGCUUUGCUUAAAAAUUUCAUAAAAUAUAC...
Bsp-1-1	GAUACGUAUUUAUCGGGAGGAAUUUUCUGUGAAAAAUGACAAAAACUG...
Lpe-1-1	UUACUUAUUUAUUGCAGGUCUUUUUGAAGUGGUUUGGGC...
Lfu-1-1	UUUAUUACUAGCAGGACUAUUAGAGGUGGUUUGGUC...
Lde-1-1	UAUUUAUUGCUGGUAUUUUAGAAGUAGCUUGGGC...
Lfa-3-1	AUUUAAUCAUUGCAGGUAUUUUUGAAGUUGUCUGGGC...
env-434	GUACUUAAUUUUGGGUGGCGUAUUUGAAGUGGUUUGGGC...
env-436	UAUUUAUUAUUAGGUGGGUUAUUUGAAGUUGUUUGGGC...
env-437	UAUUUAUUAUUAGGUGGGUUAUUUGAAGUUGUUUGGGC...
env-439	UAUUUAUUAUUAGGUGGGUUAUUUGAAGUUGUUUGGGC...
Esp-11-1	UAUUUAUUAUUAGGUGGGUUAUUUGAAGUUGUUUGGGC...
env-440	UAUUUAUUAUUAGGUGGGUUAUUCGAAGUUGUUUGGGC...
Lsp-1-1	AUCAAAAGUUUUGGGAAAUCUUUCCUGAAGC...
Lsp-5-1	UAAUACUUUUUGGUCUAUUUUUCCCCAAGC...
Pac-2-1	UGGUGGUUGCUGGGAUUUUGGAAGUGGUGUGGGC...
Eth-1-1	GCAUGGUUAGAAUUAAUUGUAGCAGGAGUUCUAGAAGUAUUUUGGUC...
Efa-295-1	GCAUGGAUGGAAUUAAUCAUAGCAGGGCUUUUGGAAGUAUUCUGGUC...
Esp-3-1	GCAUGGAUGGAAUUAAUCAUAGCAGGGAUUUUGGAAGUAUUCUGGUC...
Efa-294-1	GGCAUGGAUGGAAUUAAUCAUAGCAGGGCUUUUGGAAGUAUUCUGGUC...
env-441	GCUUGGCUCGAACUAUUAUUAGCUGGCGUACUGGAAGUAUUCUGGUC...
Ega-2-1	GCUUGGCUCGAACUAUUUUUAGCUGGCGUACUGGAAGUAUUCUGGUC...
env-442	GCUUGGCUCGAACUAUUAUUAGCUGGCGUACUGGAAGUAUUCUGGUCCACAACGAUGAAAUGGAGUGAAGGAUUUACCCAGCCACUUGGCUUUUUGUUGGAUUAUUAUUGAUCAGUAUUGUAGGCAUUAAAAUGACUAGCGGUCACUAACCUCAAAAAAGUUUUUUUGAAGUCUUUUAAA
env-443	GCUUGGCUCGAACUAUUAUUAGCUGGCGUACUGGAAGUAUUCUGGUCCACAACGAUGAAAUGGAGUGA...
Ega-1-2	GCUUGGCUCGAACUAUUAUUAGCUGGCGUACUAGAAGUAUUCUGGUC...
Ega-1-3	GCUUGGCUCGAACUAUUAUUAGCUGGCGUACUGGAAGUAUUCUGGUC...
env-444	GCUUGGCUCGAACUAUUAUUAGCUGGCGUACUGGAAGUAUUCUGGUC...
	...
	...
	...
	...
	●●●●●●●●●●●●○○○○○○○○○○○○○○○○○○○○○○○○○○○○○○————

	Lta-1-1	GUAAUUGCAGUAAUUUUCAAAGUUGCAUCGUCAACUACGAUAAAAUUGA
	Lcr-1-1	AAUUGCAGGAAUUUUCGAAGUUGUAUGGUC
	Lul-1-2	UAAUAUUAGCAGGAUUAUGUGAAGUUUGGUGGGC
	env-448	...
	env-449	...
	env-450	GAGUUAUUCAUGAAUACAAAUCUAUCUUUAUGGCAUAAAACAAUAAAUUUCAUACAGUU
	env-452	UGCAACACUGAAAGAUAAAAUUUUCUAUUUCAUAAAACU
	env-454	GCUCGGCAUAGCCGGGAUUUUUGAGGUCGUAUGGUC
	env-456	...
	env-457	GCUCGGCAUAGCCGGGAUUUUUGAGGUCGUAUGGUC
	env-459	GCUCGGCAUAGCCGGGAUUUUUGAGGUCGUAUGGUC
1	env-460	GGCGUGGGUAAUGCUUGGCAUCGCAGGCGUGUUUGAGGUCGUCUGGGC
	Rti-1-1	UGAUCAUCGCCGGACUGUUCGAGACGUUUUGGGC
	Rma-1-1	UGAUCAUUGCCGGGCUGUUCGAAACGUUUUGGGC
	env-461	UAUGGCGUGGAUCUACCUGAUCGUGGCGGGUUUGUUUGAAACCGUCUGGGC
	env-462	GAUGGUGGCCGGCCUGCUGGAGGUAGGAUGGGC
	env-463	AUCUUUUCAUUGCCGGCGGCCUGGAGGUAUU
	env-464	UAUAUCUUGGCAUCGCGGGGGCGAUGGAGGUGCUGUGGUC
	env-465	UGCUCGGCAUUGCCGGAAUUUUUGAGGUCGUUUGGGCGACCUGUAUGAA
	env-466	UCGGCAUUGCCGGAAUUUUUGAGGUCGUUUGGGCGACCUGUAUGAA
	env-467	UCGGCAUUGCCGGAAUUUUUGAGGUCGUUUGGGCGACCUGUA
	env-468	GGCGUGCUCGGCAUUGCCUGAAUUUUUGAGGUCGUUUGGGCGACCUGCAUGAAGUAUUUAGAGGGAUUCACAAAGCUCAGCUGGUCGCUGCUCACGUUUGCCGGCAUGGCGGUGAGCUUUUUCCUGCUGGCACGCGCGACGAAAACACUGCCGCUCGGUACGGCUUAUGCGGUCUGAACC
	env-469	...
	env-470	CGGCAUUGCCGGAAUUUUUGAGAUCGUGUGGGC
	env-471	ACAAAAACGCGCAAAAAAAGGAGGGGACCCCCGUGACCCAAGACUCCCGCCCGUUGGCGGCGCAGUUCGGCCGCUACGCGGU
	env-472	CCUGGGUAAUGCUGGGCGUCGCGGGGAUAUUUGAGGUCGUCUGGGC
	env-473	CCCCGGACUGUUAUUUUGACAGCCCGGGGAUCUGACAUGUUCAGGAUAUAUACGCAGCGUGCAGCUCUCAGCCGCCGAAUACGGCGAGGAGGAAGCCUGCGGCAACGGCGGAACCGA
	env-476	UCUUUUUGGUGAUCGCCGGUCUGACGGAGGUGUUCUGAUCCACCU
	env-477	UCUUUUUGGUGAUCGCCGGUCUGACGGAGGUGUUCUGGUC
	env-478	UUUUUGGGGAUCGCCGGGAUUAUGGAAGUGUUCUGGUC
	env-479	UUUUUGGGGAUCGCCGGGAUUAUGGAAGUGUUCUGGUC
	env-480	UUUUAGGGAUAGCCGGGCUGAUGGAGGUGUUCUGGGC
	env-481	UUUUAGGAAUCGCCGGGCUGAUGGAGGUGUUCUGGGC
	env-482	UUUUUGGGCAUCGCCGGUUUGAUGGAGGUAUUCUGGUC
	env-483	GCUUGGUAUCGCGGGAUGAACUGUUUUUUAUCCAUAAAGUAUCCUCAAAUGCGGCUGAGCCGCCCCUUCACAAAUCAUUUUCAGCAUUAUAUUAUACCAUGAGAGAGCCGCAUUGCCAAGGAAAAAUUAUGCCGAGGUCAGCUUCAGUCCGACUAUGCCGACCAGCAGCGUCAGGA
	env-484	GCUUGGCAUCGCGGGAAUAUUUGAGGUCGUGUGGGC
	env-485	...
	env-486	UAUUUUUGGGGAUUGCGGGGCUGAUGGAGGUGUUCUGGGC
	env-487	AUUUCUGGGAAUUGCCGGACUGAUGGAGGUGUUCUGGUC
	env-488	AUUUCUGGGAAUUGCCGGACUGAUGGAGGUGUUCUGGUC
	env-492	AUUUCUGGGAAUUGCCGGACU
	env-493	AUUUCUGGGAAUUGCCGGACUGAUGGAGGUGUUCUGGUC
	env-494	AUUUCUGGGAAUUGCCGGACUGAUGGAGGUGNNNNNNNN
	env-495	AUGAGCCAAAACAACAUUCUGCGGGAUUUUGCCAAAUAUUCUUCUCUCAG
	env-496	AUGAGCCAAAACAACAUUCUGCGGGAUUUUGCCAAAUAUUCUUCCCUCAG
	Lgi-1-1	UUUAAUAACAGCGGGAUUGUUCGAAGUUGUGUGGGC
	Lve-2-1	GAUCAUUGCUGGACUAUUUGAAGUAGUUUGGGC
	env-497	UGAUCACUGCCGGAUU
	env-498	AAUCAUCGCCGGACUUUUUGAGGUUGUCUGGGC
	env-500	AAUCACUGCGGGAUUGUUUGAAGUUGUCUGGGC
	Lna-2-1	UUAAUCACAGCUGGACUUUUUGAAGUCGUCUGGGC
	env-501	UAAUUACUGCCGGACUUUUUGAAGUCGUUUGGGC
	env-502	...
	env-503	AAUUACUGCUGGACUUUUUGAAGUCGUAUGGGC
	env-504	UUAAUUAUCGCUGGAAUAUUUGAAGUUGUUUGGUC
	env-505	UUAAUUAUCGCUGGAAUAUUUGAAGUUGUUUGGUC
	Lno-1-1	UGGAUUUAUUUAAUUAUCGCAGGACUAUUUGAAGUCGUUUGGGC
	Lal-2-1	AAAAUAAUAUGAAUUGGAUUUACUUAAUCAUCGCAGGAAUAUUUGAAGUAUUUUGGGC
	env-506	UUAAUUACAGCAGGAAUUUUUGAAGUCGCCUGGGC
	Lal-1-1	UUAAUUAUUGCUGGACUAUUUGAAGUAGUUUGGUC
	Whe-1-1	UAUUUUGGCUGGUGUUUUUGAAGUUGUUUGGUC
	Whe-1-2	UGUUUUGGCUGGUGUUUUUGAAGUUGUUUGGUC
	Lpa-1-1	CAUUGAUUAUUGCAGGAUUGUUUGAAGUCGUUUGGGC
	Lpa-2-1	UGAUUAUUGCAGGGCUCUUUGAGGUUGUUUGGGC
	Lra-1-1	UACGCCAAGCAGCAACCUGCACGUAAGCACCAUAAGCGAAAAUUUUGAAGCCAAAAUUUUCGCUUAUGGAGACUUACGUUCCGGUUGCUAACCGCUUUGCUCCGCUCACUUAUUUUUGGAGGAGAUUCUAUGAAUUGGAUAUUUUUAAUCAUCGCUGGUAUUUUCGAAGUUGUUUGGGC.
	Lki-3-1	ACAUAAGCACCACCAGCAAAAAUUCCAAAACCGGGAAUUCCUGCUGGCGGAGACUUAUGCUCCGAGUUCUAACCGCUUUGUUCCGCUCACUUAUUUUUAGGAGGAUAUCUAUGAAUUGGAUCACAUUGAUUAUCGCCGGACUUUUUGAAGUUGUUUGGGC
	Lki-2-1	ACAUAAGCACCACCAGCAAAAAUUCCAGAACCGGGAAUUUCCGCUGGCGGAGACUUAUGUUCCGAGUUCUAACCGCUUUGUUCCGCUCACUUAUUUUUAGGAGGAUAUCUAUGAAUUGGAUCACAUUGAUUAUCGCCGGACUUUUUGAAGUUGUUUGGGC
	Ldi-1-1	AUAUUUAAUUAUUGCAGGAUUAUUUGAGGUUGUUUGGGC
	Lhe-1-1	UAACAUUAAUCAUUGCAGGCCUAUUUGAAGUUGUUUGGGC
	Lfa-2-1	UAACAUUAAUCAUUGCAGGUCUAUUUGAAGUUGUUUGGGC
	env-508	ACAUUAAUUAUUGCUGGCUUAUUUGAGGUUGUUUGGGC
	Nma-1-1	UGAAGAACAAUCACACACUUAAAACUUUUAUGAGCUAUGUUAUUUUGAA
	env-509	UUACUUUUGGAGAGAAGCUGCUGUGGGGCGG
	env-511	UUACCUUUGGGGAGAAGCUGCUGUGGAGCGG
	env-512	UUACCUUUGGAGAGAAGCUGCUGUGGGGCGG
	env-514	UUACCUUUGGGGAGAAGCUGCUGUGGGGCGG
	Csp-12-2	UUUAGUAACUAUUAAACACUAUAAUUCAUUGUG
	Csa-1-1	UAAUUUAAUAACUAUCAAACAAUAUAAUUCUUUAUG
	env-515	CUUUUAUCACAUUUUGCAAAGUAUGUUAGUUUGAA
	env-516	UAUUGAUUGCAGGAUUCUUUGAAGUAGGGUGGGC
	env-517	...
	env-518	AUAUAUCUUUAUCUAUGACAAAUCUUCAGAAGGAGGAGAGGGG

```
env-519    AUAUAUCUUUAUCUAUGACAAAUCUUCAGAAGGAGGAGAGGGG....
env-520    ....
Bsp-1-2    GUGCCGGCAGCUGUAUCUGUAAGUGAGGUGUUUUUAUGUUUCAGGAGAUGAGAAGAAAACGGCAGGCUUUAUCACAGGAGGAAAU....
env-521    CAAAAGUAGACCAGCAAAAAGACCAUUUAUUGAAUCAAUUUAU....
env-522    CAAAAGUAGACCAGCAAAAAGACCAUUUAUUGAAUCAAUUUAU....
env-523    UUGAAGAAGAAUUCAAAGAAGUAUUUCCAGACUA....
env-524    UUUACCAAAUACGUUUCGGCAAACGU....
env-525    CUUUACGAAAUACGUAUCGGCAAACGU....
Msp-5-1    UAUUUACUUAUCGCCGGAAUUUUGGAAAUGGGCUGGGC....
Lsa-1-1    GGCAUGGUUUGAUUUAUUAAUCGCAGGAUUAGCAGAAGUUUUGUGGGC....
Lcu-2-1    GGCAUGGGUUGAUUUAUUGAUUGCAGGAUUGGCAGAAGUUUGGUGGGC....
env-526    GGCAUGGGUUGAUUUAUUGAUUGCAGGAUUGGCAGAAGUUUGGUGGGC....
env-527    AGAGUAUCUUUUAGUAAAUCUGAAAUCAUUUUAUG....
env-528    CGCCUGGCUCGACCUGCUCAUCGCAGGCAUUCUCGAAGUCGU....
env-529    UGGGUGGAUCUGAUCGCGGCGGGGCUGCUCGAAGUGGC....
env-530    AAAAAAAGAAUUUUUAAUGUAUACAAGUUUAAA....
env-531    AAAAAAAGAAUUUUUAAUGUAUACAAGUUUAAA....
env-533    CUUUUUGAUACUGGCCGGAGGAUUGGAGAUUUUCUGGGC....
Csp-6-1    AAGGAAAAGUAAUUUAUAUGAAGAAAGAGAGUUGUUUUCGAGAUUUUGUCAAAUAUUCCUCUUUAAA....
env-534    UGCAUAAGGAAGGUAUAUACCAUAUGAAAGAGAUGUCAUGUUUUAAAGUUUUUUUGAAGUAUUCUUCGCUCAA....
Cba-3-1    GAUAUGCCCAAUAUGUAUCGUUAAAUGUACUGGGAAU....
env-535    AAGUUUUAUUUUACAGGCUGCGAGCUCGCGCUGUG....
env-536    AAGUUUUAUUUUACAGGCUGCGAGCUCACGCUGUG....
env-537    AAGUUUUAUUUUACAGGCUGCGAGCUCGCGCUGUG....
Csp-11-1   GUUUAAAAAGUACAUUUCUGCGCUAUAUUUUAACAAACAUUAUAGG....
env-538    UUCGAAGUUCACUAGAUACGUGCUAGCGAGUGU....
env-545    UUCGAAGUUCACUAGAUACGUGCUAGCGAGUGU....
env-546    ....
Rma-2-1    UAAAAAACUUUUUAAAAUAUGUGAGUCUUAA....
env-547    CUAAUAAAAAAUUUCUUUAAAUAUGUGAGCCUUAAUGUUAUAGGAAUGAUAGGACUAUCUUGUUAUAUCCUUGC....
env-548    AAAAAAGUUUGCAAAAUAUGUAAGCUUAAAUAU....
Csp-7-1    AAAAAAGUUUGCCAAGUAUGUAAGCUUAAAUAU....
env-549    AAAAAAGUUUGCCAAGUAUGUAAGCUUAAAUAU....
env-550    UAUGAUUAGAAAUUUUUUAAAGUAUGUGUCGCUAAA....
env-552    AAUCUUUAUUGCGCACGUUUUCAAAAUACGUCACGUUGAACAU....
env-553    UGGCCAAGACUUUUAUGAAAUACGCCAGUCUGAAUAU....
env-557    AGUCUUUCAAACAUUUUUUAGAUAUGUGAGCCUUAAUAU....
env-558    AGAAUCUUUGCAAAAUAUGUCAGCUUAAAUGU....
env-559    UUGAAAAGUUUUCUUAAAUAUGUAAGUCUGAACGU....
env-560    ....
env-561    AUCACCAGAAGUCAGUCUAAUGGCAGCUUCGCCUCUUUCAGCGGGAUAAGCAAAGUGAGGUCAUCUAUGGAAAGAUCAAUAUUAAGAAGCUUUAUCAAGUAUGUCAGCGCAAAUAU....
env-562    UAAAGUUUUUAAAUUUAAAAAUACGAGACAUAGAGUCUUGAUUAGAUAGGAGGACUAAUUUGUUAAAUCAAUCAAUAUUCAAACAGUUUUCACAUUAUGUCAGAGCUAAUGUAUUGGGGAUGGUGGGUUUAUCGUGCUAUAUUUUAGCAGAUACUUAUUUUGUAGCAAA....
Cin-1-1    CCUUAAAAAAUUUUAUUAAGUAUACCAGUGUUAAUAU....
env-563    UAUUUAGAGAUUUUUCCAGAUACGCAAGUUUGAACAU....
env-564    UAUUUAGAGAUUUUUCCAGAUACGCAAGUUUGAACAUCUUGGGUAUGAU....
env-565    UUUUAAUUAAAAAAGGAGAAUUUUAUAGUGGAAAAAGAUAUUUUUAAAAAGUUCAUGAAAUACGUAAGUCUGAACAU....
env-566    UAUUUUUAUAAAUUUUGUAAAGUAUGUGAGUUUGAAUGU....
Csp-14-1   AGCAUAAAUGCUUAGCUCUAUAAUACAAGAGGAGGAUUACAAUGGAUAAAAAUAUUUUACAAAAGUUUGUCAAGUAUGUAAGUUUGAAUAU....
env-570    AAUGGUUAUAUUGGAAAGAUCAAUAUUUAAAUGCUUUGU....
env-573    AAUGGUUAUAUUGGAAAGAUCAAUAUUUAAAUGCUUUGUUAA....
env-574    UUUUAAGACUUUUACUAGGUGAGGAAAUUGCACAAUUAAAAAAUGUUUAGAUUUAUAUAACUUAUCCACAGAUUUUUGCGUUGAGUUGUUUACAUUCUAAUAAAAAUCGAUUUUAAUAU....
env-575    UUUUAAGACUUUUACUAGGUAUGUAAGCUUGAAUAU....
env-576    UUU....
env-577    ....
env-578    UAUUAAAGAUUUUGCUAAGUAUGUAAGUUUAAAUAU....
env-580    AUUUUUAAAGAUUUUACUAAGUAUGUAAGUUUGAAUAU....
Lna-1-1    UUUAUUCAAAAAUUUUAUAAAGUAUGCCAGCCUGAACAU....
env-587    UGUCUUAAAAGACUACAC....
env-588    UGCCUUAAAAAAACUACAC....
env-589    UGUCUUAAAAAAACUACACCAAAUAUGUAAGCCUUAAUAU....
env-591    UGUCUUAAAAAAACUACACCAAAUAUGUAAGCCUUAAUAU....
env-592    UGUCUUAAAAGACUACACCAAGUAUGUGAGUCUUAAUAU....
env-593    UGUUUUAAAAGACUACACCAAGUAUGUGAGCCUUAAUAU....
env-594    UGUUUUAAAAGACUACACCAAGUAUGUGAGUCUUAAUAU....
env-595    UGUCUUAAAAAAACUACACCAAAUAUGUAAGCCUUAAUAU....
env-598    UGUCUUAAAAAAACUACAC....
Csp-3-1    UGUCUUAAAAGACUACACCAAGUAUGUGAGCCUUAAUAU....
[gu-1-1    UGUCUUAAAAGACUACACCAAGUAUGUGAGCCUUAAUAU....
env-599    UCUGUCUUAAAAAGCUAUAU....
[in-1-1    ACCCUCCGGGGCCGGGCACUCCGGGCUAAGGAAAUGUGGUUGCAAAGGCAGCCGCACUCCGGCGGGAGAGCCUGCAAAGCAGACUUUUUAUUGUAUUAAAAGGAGGAUAUGAGAUGGAACAAUCUGUCUUAAAAAGCUAUGUCAAAUAUGUGAGCCUUAAUAU....
Air-1-1    UUUAAGUACUUUUAAUAUUUAGUUAGGAGAGUUUUUAAUUGGAAAGGAGAUGUAUAAUAUGGACAAGCCUUUAUUAAGAGAUUUUACGAAAUAUGUUAGUUUAAAUAU....
Csp-5-1    AAAGAGAAGGAAUCGAUGUCCCGAGCUUUUAUCUUAAUGGAGGGUUUUAGAUGGAGAGAUAUAUAUUUAGAGAUUUCAUAAAGUAUGUGAGCUUAAAUGU....
env-600    GUUCUAAAGGAUUUUGCUAAAUAUGUGUGUUUAAAUAU....
env-601    ....
env-605    ....
env-608    UUUACUAAAAAAACUUCACCAAAUAUGUCACCCUGAAUAUAAUGGGGAUGAUUGGGCUUUCCUGCUAUAUUCUGGGCGAUACUUAUUUUGUGUCAUAAGCCCUAAGCUUAACAGGAAUUGCGUCGUUAAAUCUUUCUAUUCCUAUCUAUAGGUGUUAAUUGGCAUCCGAGAAACUUGAGGU
Dph-1-1    UAUAUUAAGCAAAUUUUUAAAAUAUAUAACUUUAAGUAU....
env-609    UAUAGAAGAUAUAGUUCAUCUGUUUAUCGCAGCGGAACCUACUACUUUGAUUACUGCUUCCGCUGCGAAAUAUUUUGAAGGAGGAGUAUUGUAUGGAAAAGUCUUUAAUCAAAAACUUCAUAAAAUAUGUUGGUUUAAAUAU....
env-610    CUAUGCUUAGGAAUUUUGCAAAGUAUGUUAGCUUAAAUAUUUUAGGAAUGUUAGGUUU....
env-611    CUAUGCUUAGGAAUUUUGC....
Mha-1-1    UCUUUAUUAAGAAACUUUGCAAAGUAUGUCAGUUUAAAUAU....
env-612    AAGGGAUUUGAUUUUAGGGGAUUUUAUUUCGAAGGAGAGGUAUUGAAUGAAAGAAUCAAUCUUAAAAAGUUUUGCAAAGUAUGUUAGCUUGAAUGU....

           ........................................................................................
           ........................................................................................
           ........................................................................................
           ........................................................................................
           ●●●●●●●●●●●●●○○○○○○○○○○○○○○○○○○○○○○○○○------------------------------------------------------------
```

```
env-618   AUAUCCUAAAAGAUUUUGCUAAAUAUGUUAGUUUAAAUAU
env-619   GGCAUUUUUAGGAAAUUUAU
env-621   GGCAUUUUUAGGAAAUUUAU
env-623   UUUUAAAGGUAUUUAUAAAGUAUGUCAGCUUAAAUGU
env-625   AAAGUAAAGAGUCGCGAUGUUCCAUGCUUUUGCUUUGAUGGAGGAUUUCAGAUGCAGAAUAAUAUAUUUAAAAAUUUUAUAAAGUAUGUGAGCUUGAAUGU
Cin-2-1   UGCUUUUGUUUUGACAGAAUGAAUUGUAACUGGGAUGGUAUCUUUUAUGGUAAAAUUAAAAAAGGAAUCUUCAAUAUAAAAGGAUUGAUUAUAAUGGUAAAGAAGCUAAAAGACUUUGUAGAAUAUUUAGCAACUAUAGAAGU
env-626   AAUUAUUCUUUAAAUAUGCUGGUUUUAAUGU
env-627   UAUUAAAUAUGAUAAAACCCACCU
env-628   UAUUAAAUAUGAUAAAACCCACCUACUUUAGUCGAUAGAUUAGGUGAUGGAUCAAGAAAAGGGGAAUGAAAUGAAAGACACAGUUUUAAAAGACUU
env-629   UAGGUUACUUGAAAGGUAAGAAUAUUAUAAUGAAAGAUACAAUAUUAAAAGAUUUUCGAAAUAUGUAAGCUUAAAUAU
env-631   UGGUUAUUAAAAUGAAAGACACAAUUUUAAAAGACUUUUU
env-632   CGGAGAAGUUUGCAAAACGUUUUUCUUCCGGUUGUGAAUGAAUGACAUGAGAGGAAAGAUAACAGAUGGAGAAAUCACUACUUAAAAACUUUAUGAAGUAUGUCAGUCUGAACAU
Til-1-1   CAUUACUGAGGAACUUUUCAAAGUACGUCAGCCUGAAUAU
env-633   GAAUGAAAAGGGCACAGCCUAAUGAGUAGGUGGGAGAUGAUUGGCGGUAGGCGUCAGCCUAUUCUUUUAGAAAAAUCUUGUUGUUAAAGAUGGCCCUCUCGUUCGACCAAGAACAAACGUUCGUGUAUAAUAGGUGGCAGAAGGAGGUGCCUGGCGAUGAUGAUCCACAAAGCCUACAAG
env-634   GAAUGAAAAGGGCACAGCCUAAUGAGUAGGUGGGAGAUGAUUGGCGGUAGGCGUCAGCCUAUUCUUUUAGAAAAAUCUUGUUGUUAAAGAUGGCCCUCUCGUUCGACCAAGAACAAACGUUCGUGUAUAAUAGGUGGCAGAAGGAGGUGCCUGGCGAUGAUGAUCCACAAAGCCUACAAG
env-635   GAAUGAAAAGGGCACAGCCCAAUGAGUAGGUGGGAGAUGAUUGGCGGUAGGCGUCAGCCUAUUCUUUUAGAAAAAUCUUGUUGUUAAAGAUGGCCCUCUCGUUCGACCAAGAACAAACGUUCGUGUAUAAUAGGUGGCAGAAGGAGGUGCCUGGCGAUGAUGAUCCACAAAGCCUACAAG
env-636   UUUUAAAGAAUUUUAUUAAGUAUGUUAGUUUAAACAUUAUGGGGAUGAUAGGACUUUCUUGUUACAUUUUAGCAGAUACUUUUUUUAGCCAAAGCAUUAGGAGCCUCAGGUAUUGCAGCACUUAAUUGGGGACAACACCCUUAGCCUUAGUUUUAGGUGCUGAUACGACUAUAUU
env-637   ...
env-638   ...
env-640   CAAGCUUAAAAUUGAUAAGGAGAGAGAAGGAUGGAUGAAUCUGGUCUUAAACAGUUUAUUAGAUAUAUAAGUUUAAGUAU
env-641   CAAGCUUAAAAUUGAUAAGGAGAGAGAAGGAUGGAUGAAUCUGGUCUUAAACAGUUUAUUAGAUAUAUAAGUUUAAGUAU
env-644   CAAGCUUAAAAUUGAUAAGGAGAGAGAAGGAUGGAUGAAUCUGGUCUUAAACAGUUUAUUAGAUAUAUAAGUUUAAGUAU
env-657   ...
env-658   CAAGCUUAAAAUUGAUAAGGAGAGAGAAGGAUGGAUGAAUCUGGUCUUAAACAGUUUA
env-659   AUGGAAUUAAAAAUAAUUGAUAUGGAAUUUGCAGUAUGUAAGGUUGAGGAUUUAUCAAAAGUAAAUUAUAUAAUUAUAACAUAAGUAUUUUGUUUUUAUGCUUAUGUUAUAAUUAAAGGAUAGAAUAAAGAUUGUAAUUUAUGAAAAGAAAUUUUACGAAAGAAGUGUUAAACCUUGGAA
Tma-1-1   GAUUUUGUAAAGUAUGCAUCACUCAAUAUGGU
Nfe-1-1   AGAUUUUCUUAAAUAUACAUCACUCAAUAUUUU
Cfo-1-1   UUGGAUUUUUUAAAAUAUACAUCACUUAAUGUUUU
Npe-1-1   AAUCAGUAGGGACAGUUCUUUUUGGUUGCGUUUUGAAGUUUAAACAAUUAUUCUAAGAAUUGACAACAGCUAGGAACUAUCUCUGAUGAUUACCCAAUUAAAAUCUGUUAGGAGGUCUAUGUUAUGUGUAAGAAACGAGCCUUGCCUUUUCUUAUUAUCAUUACCAUGCUGCU
Gfe-1-1   GGAUUUUUUAAAAUAUAUAUCACUUAAUAUUUU
Psp-1-1   AAUAUAAAAAUGGAUUUUUUAAAGUAUACAUCGCUUAAUAUAGU
env-660   AGGCAAAGAUUAUGAAAAAAUUUACGGUAAAAGAGAGUUUUUGGGAAGUUUUUCCCGAAGC
env-663   GCUAUGCAGAAACAAAUAAGCAGAGAAUUUUAUAAGUACAUAUCCCUGAAUGU
env-664   UGAAAAUUGCUUUCCGCCGGCUGCAUAGGGAGGACAGGGAUGGGAAAAAACAGUGGAGCUUAUGAAUUUUUCAGAUAUGUGGGACUCAA
Cba-1-1   UGAAAAUUGCUUUCCGCCGGCUGCAUAGGGAGGACAGGGAUGGGAAAAAACAGCGGAGCUUAUGAAUUUUUCAGAUAUGUGGGACUCAA
Cba-6-1   UGAAAAUUGCUUUCCGCCGGCUGCAUAGGGAGGACAGGGAUGGGAAAAAACAGCGGAGCUUAUGAAUUUUUCAGAUAUGUGGGACUCAA
env-665   GUAAUAUGCCGCUAUGCGGCCGCGCUCGGCGCGAGAGUCCGGCGGACCGGACUCUUUCGUGAGAUAGGAAAGUACAGGGAAGGAGCAGAGUGAUGUUACACACAGAAGCAGUAAAAAAUAAGCGGAGCCGCAGCGGCGGUGA
env-667   UCCGCACAAGAAAAAAGAGAAAGAAAGAAGGGGGAAAAAACGACAG
env-668   ACUAGCCGAUACCUAUUUUGUUUCAAA
env-669   ...
env-670   UCCGCACAAGAAAAAAGAGAAAGAAAGAAGGGGGAAAAAACGACAG
Csy-2-1   UCCGUACAAGAAAAAAGAGAAAGAAAGAAGGGGGAAAAAACGACAG
env-672   UCCGCACAAGA
env-674   UCCGCACAAGAAAAAAGAGAAAGAAAGAAGGGGGAAAAAACGACAG
env-675   UCCGCACAAGAAAAAAGAGAAAGAAAGAAGGGGGAAAAAACGACAG
env-679   UCCGCACAAGAAAAAAGAGAAAGAAAGAAGGGGGAAAAAACGACAG
env-680   UCCGCACAAGAAAAAAGAGAAAGAAAGAAGGGGGAAAAAACGACAG
env-683   UCCGCACAAGAAAAAAGAGAAAGAAAGAAGGGGGAAACAACGACAG
env-684   UCCGCACAAGAAAAAAGAGAAAGAAAGAAGGGGGAAAAAACGACAG
env-689   ...
env-690   UGACAGGAAGUUUUCUAUAUGUAAGCUGAAAGA
env-691   ...
env-692   AUAUCUUUUAUACUUUUAUGAAAUAUUCAUCAUUAAA
env-693   GCAUGGUUAUAUUUAUUGAUUGCAUCUGUGUUUGAAAUCGGUUGGCC
env-694   AUUUUUG
env-695   UUUUGUUUAUUGCAGGUGUUUUUGAAGUAGUAUGGGC
[ae-1-1   CUUUUUGUUUAUUGCAGGAAUUGUUGAGGUUGUAUGGGC
env-696   ...
env-697   AUUUCACCCCGGCGGAACGCCUGCUCUGGGGAGGAUC
env-698   AUUUCACCCCGGCGGAACGCCUGCUGUGGGGAGGAUC
[am-2-1   AUAGAGAGGUGUAUCAUGUUCAGAAAAAUGAGACGAUUCAAACAGCAGGUGUCCGAUGACGAAUG
env-699   CUUUGCCUCGCUCGUGGUGGAUUUGGAAAGUUUGUUGACGCGGUCGAAGAAACUGCCGAUUAGUUGGCAGAGUUUUUAUAAGACACUUA
env-700   UAAAAGGAGGAUAAAAGAUGAAUGAGAAAGGACUUUUAAAAGAGUUCGGAAAGUAUGCAUCGCUUAA
env-701   UAAAAGGAGGAUAAAAGAUGAAUGAGAAAGGACUUUUAAAAGAGUUCGGAAAGUAUGCAUCGCUUAA
env-702   UAUAUAGAGGUAUAGAAGUAUGAAAAAAUUUGUAAUGGAAAAACCCUUUUUAGAACUGUUCCCUGACUG
env-703   AAAUAUUUACGGAGGUAAAAAAAUGAAGAAGUUUGUAAUGGAGAAGCCUUUCCUUGAGCUGUUCCCUGAAUG
env-704   UAACGGAGGUAAAAAAAUGAAAAAAUUCGUAAUGGA
env-705   AGCUUUUCAUGUCUUUUAUCAAAUAUGUUUCACAGAGCGU
env-707   AUGGGGAGAAUAUGUUUUUGGAGAAAGACGGCAUUUUGAAACGAUUUGC
env-708   UAAGUUAUUUCUCAAAGGGUGAGCUUGCGCUGUG
env-709   AUUCAGUUCACUCACAAUUAUUGACUACACAAUA
env-710   UUUCAGUUCACUCACAAUUAUUGACUACACAAU
env-713   ...
env-714   ...
env-716   ...
env-717   CUCUUAAAUCGCUAACCAGAUUUGACCGCACCUUGUGGACCACUUCGGUCUUUCUUAUAAUUUUUUCAUUUGUCCUGACAAACAACACUGACUAUUGGACUUUGUU
Bpi-1-1   AUCUAUUUAUUAAAUAUGUAUCACUUAAUGUGCU
env-718   AAAACUUGUCAAAAGCAGAGAAAACCAG
env-719   CUGUUUGUAGAAAUUUUAGGAGGUAUUUAUGCACAACAAAAUACUUUGGAAAGAAUUUAUUCGGUAUGCUUCUUUUGGCUUUUUUCCUCCUGUUUUGGCGUAACCGACGUAUAGCUUGCUUUUCCUGACACAGAAUUUCUCGUCUUUUUAUAUGAGCCUGUUUCUUUUUCAAAAAUGGGG
env-721   CUUGGUGUUAUCUCUUUGUGGCAGGGGCUUUUGAAUGCAUCUGGUCAGCCUGCAUGAAGCUCAGUGAAGGCUUUACCCGCCUGGGCUAUACCUUGGCUACCCU
Exy-1-1   UUAAUCUUAGCAGGAAUCUUUGAAGUAACAUGGGC
env-722   AAAAUCUAAAUCAAUGACUCUGCUAUAUUUUGACUGUGCUCAGUUAUACGCCUUA
```

```
env-723    UGAUAGCAGGAGUUUUAGAAGUGUCAUGGGC...
Sru-1-1    GAUUCAAGCAACAGCUGACAGAGGAAGACUG...
env-724    UGUAAAUUGUUCCUGCAAUGUUUGCAGGAACAGCCGGGAGG...
env-725    GAUUCAAGCAGCAGCUGCCGGAGGCAGAGUG...
env-726    ACAGAAUUUUUAAAAUAUGUGUCGCUUAA...
env-727    GUUUUAUUUAAUAAUUGCAGGAAUGUUUGAAGUAGCAUGGGC...
env-728    AUGGUGAAUCUUUUAGAACGCUAAGAAAACAGCA...
Lga-5-1    GUAUGGUGAAUCUUUUAGAACGCUGAGGAAACAGCA...
env-729    UGUUG...
env-730    GUUUAUUGUAUGUUGUUGCUUCUAGGAACUACUAUCGCGACGAGUUUUCUGGAUAGUUU...
env-731    UUAAAACAAAAAACCUGAGGCGGGAAGGAUUUUGCGGUAAAAGAUUGUAAGGAGGUAAAAAAUGUUUCAGGAAAUGAGAAGAAACAAACAGCAGCUGUCAAAAGAAGAGUG...
Esp-13-1   GUGAAAAACUAAAAAUGGAGGAGAAAAAUGUUUCGCGAGAUGAGAAGAAAAAAAGCAGCAGCUGACGCAGGAAGAAAC...
env-732    CGAAAUGAGAAGAAAAAAGCAGGAACUGCCUCAGGAAGAUGU...
env-734    UCUAUGUUUCGAGAAAUGCGCUUAAAAGAACAGCAGUUGUCCCAGGAAGAAGC...
env-735    CAUUUUAUAAGGAGAUGGAUUUUAUGUUUCGAAAUAUGCGGCGAAAAAAACAGGCUUUAUCACAGGAGGACUG...
env-736    CAUUUUAUAAGGAGAUGGAUUUUAUGUUUCGAAAUAUGCGGCGAAAAAAACAGGCUUUAUCACAGGAGGACUG...
env-737    CAUUUUAUAAGGAGAUGGAUUUUAUGUUUCGAAAUAUGCGGCG...
env-738    CCGGUUUUCCGGGCACUGCGACGCACGGCGGACUGCCGCACCAAAGCGCAGGUCCGCAAUUUUGAAGGUUAAAGUUUUGGAGGUUGAAAUGAAAACAAACUGCUUUCGGGAUUUCGCAAAAUACGCGUCCCUCAAUGU...
env-740    UUUUUAUGACUAAAUCAAGUUGUUUUCGGGAUUUUGCCAAAUAUUCGUCAUUGAA...
env-741    AAAUGUUUAAUAUAUCUAUUCAAUUGAUAAAAUGAAAGGAUAAAGAAAACAUGAAGAAAUUAGAGAGUUUACAGGAAUUUUUUAGGUAUACAUCUUUAAA...
Msp-2-1    AAAUGUUUAAUAUAUCUAUUCAAUUGAUAAAAUGAAAGGAUAAAGAAAACAUGAAGAAAUUAGAGAGUUUACAGGAAUUUUUUAGAUAUACAUCUUUAAA...
env-742    AAAUGUUUAAUAUAUCUAGGAUAUAUCUAUUCAAUUGAUAAAAUGAAAGGAUAAAGAAAACAUGAAGAAAUUAGAGAGUUUACAGGAAUUUUUUAGGUAUACAUCUUUAAA...
Mti-1-1    UGCGUCGUAGUAAACAAGCUUUAUCAAAUGAAAAAUGUAU...
env-743    GCGUCGUAAAAGACAAGCCUUAUCAAAUGAAGAAUGUAU...
env-744    GCGUCUGGUGUGUGUUCCCCGUAACAGAAGUACUGGCGGCGGCUGCAGGACCUGCGUUCUUCCUCGCCAUGCGGAAAAAAGAAAGCUGAUGUUGUACGGACUUCUUGCAUCUUUUGAAGAAUGUGGUAAAGUAUAUUUGGUACCGGUCUGGGCAGGAAAUGUACAGAUCGGAUGGAUACU
env-745    UUUUAUGGAAAAGUCAACUUGUUUUCGGGAUUUUGCAAAAUACACAUCCCUCAA...
env-746    GCUGAUGUUCAGGCCAAUGAGGAGACGCGGCCAGGAAAUGCCGCCGGAGGAAUG...
env-747    GCUGAUGUUCAGGCCAAUGAGGAGACGCGGCCAGGAAAUGCCGCCGGAGGAAUG...
env-748    GUUCAGACCAAUGCGCAGAAAAAAUCAGGCGCUCCCUUUAAAGGAAUG...
Bma-1-1    AUCGAAAAGAAUUUGCAAAAUACACCACCCUGAA...
env-751    AUCGAAAAGAAUUUGCAAAAUACACCACCCUGAA...
env-753    UUAUGGCAUGGUUCGAUUUAUUUAUAGCGGGCAUUUUUGAAGUUGUAUGGUC...
env-754    UGAUUUACUUAUUGCCGGCAUUUUCGAGGU...
env-755    UGAUUUACUUAUUGCCGGCAUUUUCGAGGUGGUGUGGUC...
Lga-1-1    ACUUAAUAUUAGCAGGAUUGUGUGAAGUUUGGUGAGC...
Lsp-4-1    UACUUAAUAUUAGCAGGAUUAUGUGAAGUUUGGUGAGC...
env-757    UUAAAUAUUAAACUAAAUAAAUUUGAACUGAGUCUAUGGAUUUUUUCUUUGGUGGUUUUAUUCAUCAGUUAUUU...
env-758    UAAAUAUUAAACUAAAUAAAUUUGAACUGAGUCUAUGGAUUUU...
env-759    ...
env-760    AUGAUCGGAAUCGCAAUAGUCGCCUCCGGCUCCGCUGCGAUUCUUGCCGACGGCGCUUCGCGCUGGGAGGCGAUGGGGGCCUCGCCCCCAUGCCCCUGCCUGAGGGACAUCGUCCCUCAGACUCCCUUCUUCACUUCGCGCUUACAGCUCGAUUCUUUCGAUGAUUCAAGGAGUGCUGCC
env-761    AAUUCAAUCAGAAAUUUGUCAAGAUUUGAGGGCUGUCU...
env-762    UAAAACUUUUUUAAAGUAUGUUUCCCUUAACAUUUU...
env-763    UGUUCUAUAUUCUAAGUUGCAAGGUAA...
env-764    CUUUAUACCGGAUUCAUACUAUUUUAUCAGGAGGCUGCCUAUGCUUUACAAAACGGGAAGAAAAAAAGAUAACCAGGCUAUUUUCCGGCU...
env-765    UUUUCU...
env-768    GGAAUAUUGCGGUUGUCAUACAAUCGUGAUACAAUAAAGGAAAAAGGAGUGAUGUCGUAUGGCGCAAGCAACGUUGAGUGUCCGUCUCGAUCGCACGGACAAAGAAAU...
env-769    UAC...
Lsp-9-1    AUUUUAAAUAUAACGUACAUAUGGGAGGACACGUAUUAUGGAAUGGGCAUUUUUAGUUAUUGCAGGCGGUCUGGAAGUAUUCUGGUC...
env-770    UGUAUUAUGGAAUGGGUAUUUUUAGUGAUUGCCGGAGGACUUGAGGUAUUCUGGUC...
Lsp-10-1   AGUGCACAUAAGAUGGAAUGGGUGUUUUUAGUGAUCGCAGGGGGCCUGGAAGUGUUCUGGUC...
Csp-15-1   UUCAAAAGAGAAGAAAGAGAGGAUGAAAUUAUGGAAUGGGUAUGUUUGAUCAUUGCAGGCGGACUGGAAGUGUUCUGGUC...
env-771    AUGUUGCUCCUUGCCGGAGCAUCAGAAAUUACAUGGGC...
env-773    CAGCGAUCGAUCCUGCGGGGAUUCAUCACGUAUGCGUCCCUCAACGC...
env-774    CCCGCACCAUAACGCGGCCGUAUUUCCAUCCAAACUUUUCAUGGGGAGGUCUUUCUAUGGCAUGGUGCUAUCUGAUUCUGGCGGGACUGCUGGAAAUUUUUUGGGC...
env-775    UAUUUGGUCGGAGCAGGCUUGUUAGAGAUUUUUUGGGC...
env-776    UAUUUGGUCGGAGCAGGCUUGUUAGAGAUUUUUUGGGC...
env-777    AUAUUUGAUAGGCGCUGGCUUGUUGGAGAUUUUUUGGGC...
env-778    UAUUUACUUUCAGCAGGAAUACUGGAAAUCUUCUGGGC...
env-780    AUUUUUAGUUUUUGCAGGACUGCUCGAAGUCUUCUGGUC...
env-781    UAUGCAAUACUUAAAAGAAUUCAUCAAGUAUACUACUUCCAA...
env-782    AGAAUUUGUGUAAUAAAAGAAUAAUGAAAUGCUAAACAAUAUUUUUAAAAUAGAAGGAUGGAAUAAUGAACAGUAACUUAAAAGAGUUUAUUAAAUAUACAGUUUUUAAUGUUUU...
env-784    AGAAUUUGAGUAAUAAAAAAAUAAUGAAAUGCUAAACAAUAUUUUUAAAAUAGAAGGAUGGAAUAAUGAACAUUGGUU...
env-785    UUUUUCCUAUUUC...
env-786    AUUGAUUCUCUCACGCUCGCUGAAAAGGUUCUCUGGUC...
env-787    CAUAUUUCACAAUCUUUGAGCUUAUAUUAUGGAUCACAAGUGUUUAACUAUAUUUAUCGUUUUUUAUAUGUUUCAAAAUAAUGAACACUUAUAUUUGAUUGCUUCUUUAAUAGGUGU...
env-788    ACUUUUCAAAACUUUUACAAAGACCGAUUUUCUGCU...
env-789    ACUUUUCAAAACUUUUACAAAGACCGAUUUUCUGCU...
env-790    ...
env-791    U...
env-792    UUCGACCACCCUCCACCCUGUCUGCUUUUCUGCACUA...
env-793    ...
env-794    ...
env-795    UUCGACCACCAUCCACCCUGUCUGCUUUUCUGCACUA...
env-798    UCCCGCGGCGAAAUCGGACUGUGGCUCGGAAG...
env-799    GGGAUAUUACAAUAUGGCUUGUGUCCGUAAC...
env-800    AAAUCAUUGUAUAGUUAUUUUUCAAAAGGGGAAAG...
env-801    GUAUUUCUCCAAGGGGGAGUGGCUCCUGUG...
env-802    UCUUUAUGAAAAAGCUGUUCCGCUAUUUUACUCCCGCUGAGAAGGCCCUCUGGCU...
env-803    AAAUUACUUUUCAAAAGGCGAGAUUGCGCUGUGGGC...
env-804    AAAUUACUUUUCAAAAGGCGAGCUUGCG...
env-805    AAAUUACUUUUCAA...
env-806    AAAUUACUUUUCAAAAGGCGAGCUUGCGCUGUGGGCGGGC...

           ...
           ...
           ...
           ...
           ●●●●●●●●●●●●○○○○○○○○○○○○○○○○○○○○○○○○○-----
```

env-807	AAAUUACUUUUCAAAAGGCGAGCUUGCGCUGUGGGC...
env-811	AAAUUACUUUUCAAAAGGCGAGCUUGCGCUGUGGGC...
env-824	AAAUUACUUUUCAAAAGGCGAGCUUGCACUGUGGGC...
env-827	AAAUUACUUUUCAAAAGGCGAGCUUGCGCUGUGGGC...
env-828	AAAUUACUUUUCAAAAGGCGAGCUUGCGCUGUGGGC...
env-829	AAAUUACUUUUCAAAAGGCGAGCUUGCGCUGUGGGC...
env-830	AAAUUACUUUUCAAAAGGCGAGCUUGCGCUGUGGGC...
env-848	AAAUUACUUUUCAAAAGGCGAGAUUGCGCUGUGGGC...
env-849	AAAUUACUUUUCAAAAGGCGAGCUUGCGCUGUGGGC...
env-850	AAAUUACUUUUCAAAAGGCGAGCUUGCGCUGUGGGC...
env-851	AAAUUACUUUUCAAAAGGCGAGCUUGCGCUGUGGGC...
env-855	AAAUUACUUUUCAAAAGGCGAGCUUGCGCUGUGGGC...
env-856	AAAUUACUUUUCAAAAGGCGAGCUUGCGCUGUGGGC...
env-858	AAAUUACUUUUCAAAAGGCGAGCUUGCGCUGUGGGC...
env-860	AAAUUACUUUUCAAAAGGCGAGCUUGCGCUGUGGG...
env-861	AAAUUACUUUUCAAAAGGCGAGCUUGCGCUGUGGGC...
env-862	UUUUCCAGGGGUGAGCUGGCACUGUGGAU...
env-863	UAUUCUGCUCUGGGCUGUAUCGGCAGCCGU...
env-864	GCAAUUAUGGAUAGAACAAAUUAUAUCUCUUAAUAAACCUUUAAAAAAACCUUUGAUAGGGAGGAGAAAAAAUGGAAUUAUUCAAUAAUGCCAUUAACAGCAUCAACAGUGUCAUCUGGAG...
env-865	GCAAUUAUGGAUAGAACAAAUUAUAUCUCUUAAUAAACCUUUAAUAAAACCUUUGAUAGGGAGGAGAAAAAAUGGAAUUAUUCAAUAAUGCCAUUAACAGCAUCAACAGUGUCAUCUGGAG...
env-866	CGUGGAUAAAUUUAGUAAUUGCGGGAUUUUUUGAAAUAUUAUGGGC...
env-868	UUGUUAAGGAGGAAAGAGGAUGAGUGGAAUGGAUUCGUAUGUGCAGGCGCUGGGGAGCCAGGCUUACAGGCAGGCUCA...
env-869	UUAAAUCUUUUAUCAAGUAUGUGACUUUAAA...
env-871	UUAAAUCUUUUAUCAAGUAUGUGACUUUAAA...
env-872	CUUAAAUCUUUUAUUAAAUAUGUAACUUUAAA...
Fsp-3-1	CUUAAAUCUUUUAUUAAAUAUGUAACUUUAAA...
env-878	AAAUGCGCAGAAAAGGGCAGGCGCUGCCUGCGGAAGAAAU...
env-880	UUAGAGAAAUGCGGAGAAAGAAUCAGCUGCUGUCGGAGGAAGCGGC...
env-881	GAAGAAGUAAACAGGCUUUAUCACACCAGGAAUG...
env-882	CACAAGGAGGAUUUAC...
env-883	CACAAGGAGGAUUUACCAUGGCUUGGUUUUAUUUACUCAUCGCCGGCAUUUUCGAGAUCACAUGGGC...
env-884	CACAAGGAGGAUUUACCAUGGCUUGGUUUUAUUUACUCAUCGCCGGCAUUUUCGAGAUCACAUGGGC...
env-885	UGUUUAGAGAA...
env-886	AAAACCUGUGAAAACCUACCUCUGGCUUUUGGCCACCAACCUGUU...
env-888	AACACCUUUACCUAUAAGGA...
env-889	AACACCUUUACCUAUAAGGAUUUAUACGAAGAAAACAGCAGAAAAGU...
env-891	CGAUGGGUGAAUGAGCAUGCCUAAGGAGGAGAGGUUCAGAUGAAAUUUCGGGAGUUAUUCAGUUAUUUUACAAAAUUUGAGUGGUGUUU...
env-892	CGAUGGUGAAUGAGCAUGCCUAAGGAGGAGAGGUUCAGAUGAAAUUUCGGGAGUUAUUCAGUUAUUUUACAAAAUUUGAGUGGUGUUU...
env-893	ACAUAUUUUUUUCAAUUUUUUCAGUAUGCAUCUUUCAA...
env-895	ACAUAUUUUUUUCAAUUUUUUCAGUAUGCAUCUUUCAAUGUGCUUGGAAUGUUAGGCCUGUCCUGCUACAUUUUGGCCGACACCUUUUUCAUUUCAAAGGGGUUGGGUGCAAAC...
env-896	ACAUAUUUUUUUCAAUUUUUUCAGUAUGCAUCUUUCAA...
env-897	UCUGGAGGUAUGUGUUAUGAGGCAAUCGGCCUGCCUGCGAGAUUUUAUCAAAUAUUCUUCUUUCAA...
env-898	AAGGAGGAUAAAAUUUUGAAGGGCUUUGAUUAUAAAGAAAUUUAUUUGGAGGACGGAAAAAGAGU...
env-899	GGAAAAAUGUGGAUUCUUUUAGGAACAACGAUUACGGCCAGCUUUUUUGACAGUCU...
env-900	CUGCCGCUGCACGUCAGAAAAAGAGGAGGAAGAAAAUGGAAUUAUUUACCUAUAAGGAAC...
env-901	CUGCCGUUGCCCGUCAGAAAAAGAG...
env-902	CUGCCGCUGCACGUCAGAAAAAGAGGAGGAAGAAAAUGGAAUUAUUUACCUAUAAGGAACUGUAUGAAGAAGAUGGAAAACGAAU...
env-905	GGUCAAAACGAUGAAAGAGAGAAAUGAACUAUGAAGUAUACAGCUUGCUUUAAAAAUUUUGUAAAAUACUCUUCACUGAA...
env-906	GAGUAA...
env-907	UGAGAAGAAAAAGGCAGGCGUUAUUGCCGGAAGAAAU...
env-908	AAUGAGGAGGAAACGGCAGGCAUUAUCGUCAGAAGAAAU...
Cbo-2-1	UUUAUAUUUAAAAUUUCUGAAAUUUAUAACUUUAAA...
env-910	AAUAUUAAGAGAACCUUUUGGAAAUAUGUUACAAUGAA...
env-911	AUCAAAAAAACCUUUAUUAAAUCUGUUUCGCUCAA...
env-912	AAAAAAACAUUUUGUAAAUAUGUCUCACUGAA...
env-913	AGAGACAUGCGCCGUUUCAAACA...
env-914	GCAAGCUGUUUUAUUAAAACGGCCGCGCGCUUUUGGCGCGGUCAAUUAAAAUACAAUUUGCGCAUCGCCAAUAGGCGGCGCAUUUGAAUUCAUUAAAAGAGGUAUCGAUAUGUUCAGAGAAAUGCGCCGCAAAAAGCAGGUUUUAUCGUUGGAAGAAAA...
env-915	UGAGAAGGAAGAACCAGCUGCUUCCCCAGGAGGAAGC...
env-916	...
env-917	UUCGUGAAAUGCGCCGUAAAAAGCAAGAACUUUCUCUGGAAGAAAG...
env-918	ACAACAUACGAUUCUCAAUGAUUUUAUAAAAUAUGCCUCUCUCAA...
env-922	ACAACAUACGAUUCUCAAUGAUUUUAUAAAAUAUGCCUCUCUCAA...
env-923	ACAACAUACGAUUCUCAAUGAUUUUAUAAAAUAUGCCUCUCUCAA...
env-927	CCCUCCGGGGGAUUGCACUGGGUUGGCAAAGAAGGUGUUGCUAUCGCAACCCAACCCAUGCGGAGAAUCUUGUAAGCAAGAUUCUUUCUUAUUUAGGAAAGAGGUGUCCAUAUGUUUAGGGAAAUGCGUCGCAAAAAACAAGCUUUAUCAUUAGAGGAAAA...
env-928	AAAUGCGCCGCAAAAAACAGCUUUUAUCUUCUAAUGACAG...
env-929	...
env-930	AAACACAUAAAAUAUCUACAGAACUUUGAAAAGCACAUAUCAAGAUCAAUGAGCCCCAAAAACAUAAAAUCGCAUAAUAACUCCAAAGACAGUUGCUUUAUGGACUUUGCAAAAUAUUCGUCUUUAAAUGUUUUGGGAAUGCUCGGUCU...
env-933	G...
Pvi-1-1	UAGAGAAAUGCGUCGCAAAAAACAGGUUUUAUCCGUAGAGGAAAC...
env-934	GGAGGAUUUGUAUGGAGUUUUA...
env-935	GCGAUUGCAUAAAUGCAAUAUGUACGUUGCGCAGCAGCAGUCUGCUCUGAAUUUGCGAGGUAGCUAUGCAAAAAUCAAAUUGUUUUCGGGAUUUCAUAAAAUAUUCUUCCUUGAA...
env-936	...
env-937	AUGCGUCGUAAAAAACAGGUUUUAUCAUUAGAGGAGAA...
env-938	AGAGAAAUGCGUCGUAAAAAACAGGCUUUGUCCUUAGAAGAAUG...
env-939	CGAGAAAUGCGCCGUAAAAAACAGGCUUUAUCCUCAGAGGAAUG...
bMS-1-1	CGAGAAAUGCGCCGCAAAAAACAGGCUUUAUCCCCAGAGGAAUG...
env-940	CGAGAAAUGCGCCGCAAAAAACAGGCUUUAUCCCCAGAGGAAUG...
env-941	...
Epl-2-1	GAGGAAUUUUCAUGUUUCGAGAAAUGAGAAGAAAAAAACAGAUGUUGUCCUGUGAACAAUG...
env-942	AAACAUGCGUCGAAGCAAACAGGUUUUAUCACAGGAAGACAG...
env-944	CCCAAAUGAAAAAAAUUGAGGGGUAAAAAAAGAGUACAACAAACCAAGCCUGCAAAAGAAGAUUUUUUGCAAGCCAUGCAAGCUGAAGAUUUUAGAGCUGUCUAAUCCUCGGCAGACACCCGCGCUUCAGCGGCGGCGAGGCAUUCCUUCA...
env-945	GAGAAAGGGAGAUAAUAGAUGAUCCGUAUUAUUAUUGCUGAGGAUGACCGAAAAUGUGCUGACCAAUU...

env-946 AGGUAGAAGCUUGUGAAGAAUAUAAAAAAAUUAUUCCGCUAUUUUUCAAAGGGCGAAAUAAU...
env-947 AGAUUUUGGAAUUACUUCAGUACAUUUGAAAAAAUGUUGUG...
env-948 AUCCGAUUAAAUCGGUAAAAAACCUUUCUUUGUUUGAAAGGUGCCU...
env-949 AUCCGAUUAAAUCGGUAAAAAACCUUUCUUUGUUUGAAAGGUGCCU...
env-950 AUCCGAUUAAAUCGGUAAAAAACCUUUCUUUGUUUGAAAGGUGCCU...
env-951 GCCUUGGAAUUUUCAAGCUUUUAGGAAGUGAAGCAUGAAACGUUUUUAUGAUAAGCUGAAUUAUUUCAGUAAUACCGAAAAAAU...
env-952 GAAUUUAGAAAAAUGAGAAGAAUUAAGCAACAGUUAAGCAACGAUGA...
env-953 GAAUUCAGAAAAAUGAGAAGGAUUAAGCAACAGUUAGGCAAUGACGA...
env-954 AAAUUAUUGUUAAAUUAUUUUUCAAACGCCGAGAA...
env-955 UAGUAAACGGAAAUAUGAAUAA...
env-957 UAGUAAACGGAAAUAUGAAUAAUGUACAAAAACA...
env-958 CGGCAGCCUUAAAAAGCUU...
env-960 AUUUGUAGCAGGUUGUUUCGAAGUCGGAUGGGC...
env-961 AUUUGUAGCAGGUUGUUUCGAAGUCGGAUGGGC...
env-963 UUUUGGGUGAGUUUGUAAAAUAUACCUCAUUUAA...
env-965 UUUUGGGUGAGUUUGUAAAAUAUACCUCAUUUAACAUCCUGGGGAUGCUAGGUAUUUCACUUUACAUUCUCGCGGACACCUUUUUCGUCUCAAAUGCGCUUGGAACGAA...
env-966 AUUUUAAAGAAUUUGUCAGAUAUGCGUCUUUGAA...
env-968 AUUUUAAAGAAUUUGUCAGAUAUGCGUCUUUGAA...
env-969 CAAUGAUAUUUAUUUGUACAGAGUGCAAAAAAGGCAUGCCCAAAGAUAAAAAUGAAAUUUAAACUUAAGCUUAAAUUCAAAUUCAAAUUCAAUCUAUUCAACACGAUGAGGAGGACAAGAAAAUGAAAAAAUUUAUUAUGGAAGCUGAUUUUUUAGAACUGUUUCCGGAAGC...
env-970 AGCUGUUUAAAAGAAUUUAUACGCUAUGCGUCUUUGAAUAG...
env-971 UUCAAAUCUCAAAGAAUUUAUCCGAUACACAACAUUAAAUGUUUU...
env-972 UAUGAGCCUUCGGGCUUUCGGGAAAUACGCUUCGCCGGG...
env-973 GCCUGCUCUGAUUGAAUUGUUUUCUCCCGGUGCACAAGGAGGUUUUUUAUGGCUUGGAUUUUUCUAUUUAUCGCCGGAGGAUUGGAGGUCUUCUGGUC...
env-974 GCCUGCUCUGAUUGAAUUGUUUUCUCCCGGUGCACAAGGAGGUUUUUUAUGGCUUGGAUUUUUCUAUUUAUCGCCGGAGGCUUGGAGGUUUUCUGGUC...
env-976 UUGAAGCAAUUUGCCAAAUAUGUUUCCUUAAA...
env-977 UGGCCAAUCGGCUUAUUGGUGCUUUCCAAUGUAUU...
env-978 CGGCUCACGGCGGGGGGAAGAGAGCGACCGCAAGGGACGACCGCAGAGAGCGAUCGCAGACAAGAAAGAUCACAGAUGAGGAGGACAAGAAGAUGAAAAAAUUUAUCAUGGACAAAGAUUUUACAGAUCUUUUUCCACAGGC...
env-979 CGGCUCACGGCGGGGGGCAGAGAGCGACCGCAAGGGACGACCGCAGAGAGCGAUCGCAGACAAGAAAGAUCACAGAUGAGGAGGACAAGAAGAUGAAAAAAUUUAUCAUGGACAAAGAUUUUACAGAUCUUUUUCCACAGGC...
env-980 CGGCUCACGGCGGGGGGCAGAGAGCGACCGCAAGGGACGACCGCAGAGAGCGAUCAAAAGGACGACUGCAGAGAAAGAUCAAAAGGACGACCGCAGAGAAAGAUCGCAGACAAGAAAGAUCACAGAUGAGGAGGACAAGAAGAUGAAAAAAUUUAUCAUGGACAAAGAUUUUACAGAUCU
env-981 ...
env-982 GAAAACUGAAUCAACGCAGUGUCAGCCAGAAAU...
env-983 GAAAACUGAAUCAACGCAGUGUCAGCCAGAAAU...
env-984 GAAAACUGAAUCAACGCAGUGUCAGCCAGAAAU...
env-985 GAAAACUGAAUCAACGCAGUGUCAGCCAGAAAU...
env-986 ...
Cba-2-1 UUUGGGAAGGGGUGAAAGAUUAAAGCAAAUUCUCUUGACGGAUCAAUAGGAACUAUUUAUAAUUCAUUCAUGCUUCUUGCUGCAUUACAGAAUCGAUACAGCAAGCAGCAAUCCGUAAUCAAUAAUCAGUUGGAGGUGCAUAUAUCAUGUUUCGUGAUAUGAGACGAAAAAAACAAUGUU
env-987 AGGGAACAAUACAUGGAAUGGAUCUUCUUAUUUGCAGCCGGACUUCUGGAGGUAUUCUGGUC...
env-988 AGGGAACAAUACAUGGAAUGGAUCUUCUUAUUUGCAGCCGGACUUCUGGAGGUAUUCUGGUCUAC...
env-989 CCUGCAAUAAGAAUAAAGUGUAUAGUUUGAAUGACAUGGAAGAGGAGAAAAUGACAGGAAUGAAGGAGUUAAAGAAAGAACUGGC...
env-990 UGAAAGUAUCUUCCUAUAGAAUAAAAAGCCCUCCGGGCAGGAUGCGCACGGAUGCAAAGGGAAUUUUAUUGCUUUGCAAUAAGCAUCCGGC...
env-991 ACCUGAAACGCACCUAAGCAAUCUUUCCCAUGAUUGCAUGAUUAAAAAAGGAGUUUGUAUGACCAAGAAAGUUGCAUUUAAAGAAUU...
env-994 GGCUUUCGUGAAUUUUUCAGGUACACGGUUUUAAGUGUUUUGGGACAUUAAGCGUAUCCUGCUAUGUUUUAGCCGAUACUUUUUUUGUUUCAAAAAGCUUGGGUUCAAAUGGACUUUCUGCCCUAAACCUUGCUAUUCCUGUUUAUAAUUUUAUUCAUGGAUCAGGUUUAAUGCUUGGAA
env-995 CUAUUCAAAGAAUUUUAUCAAUAUGUAGCUCUAAA...
env-997 CAUGCGCUGACGUGUGCCCGGCGCACAGAUAUGAAAAGUAGAAAGGAGUCGUUGUCAAAAUGGAAAAACAGUCCUGUCUGCGGGAUUUUUCACACUAUACGAUAUUAAG...
env-998 AUCGUCAAAAGUGUUAAGGGGAAACGGUAUGGAAGGAAAUCAUUA...
env-999 UGAACACAGAUGAGAUUUUUAUGAAAAAGGCGAUUGAGCUGUCUGCUUU...
env-1001 ACUGUUUAAAAGACUUUAUUCGCUAUACUUCGCUUAA...
env-1012 AAGACGGAGCUUUUUAAAUACAUAAUACUAAA...
env-1015 GCUGCUCUGCUUUCACGAAUUCGUCACGUACUCCGC...
env-1016 CUUGUUUGAAGGAAUUUGCAAGGUAUUCAUCGCUCAG...
env-1017 GUCGUUGGGGUUCUCCGCUUUGGUCUCACAGCUUUCUUCCAGGAUUGUGAUGAUUACCUUUCACGCCAUCAUCCAUGAGAUAUGGCUGGUGUAUGGCUUACAUAUCCAGCAAGAAAAAGCUUAGUUGUGUUGCUUGGAUUUGUAAUAUAUAAGAAAAUAAGGCAGGAGAAAUGGUUUAUG
Lba-2-1 CAUUUUUGUAUGAGAGGAGGCAACAAAUGAAAGAAAAAAACCUUUUUAAAGAAUUUUUUCAGUAUAUCACUUUAAA...
env-1022 AUGCGUAGAAGCAAACAGGUAUUGGCAGAAGAUGAAGU...
env-1023 AACGCUCCGCGAGGAUCGCACUGCGGCGGAAAUGAAAUAUGUCGCAGAGGCGACCCGCCGCAGGCGGAGAAUCCUGCGAGCAGGAUUCUUUUUUAUGGGGAAUUUUGACAAAUUUCAUGUAAGGAGGGCGGACAAAUGAACAAUCCGAUAAAAAAACCUGACAAAAAAAGAAUGGGGCUUG
env-1024 ...
env-1025 UAGUACACAAAAAGGCACGCCCUGCCAACGAUGCGCACUCGCGCGAUGAGGAAGUUUGUCGCAAACGACCGCGCUCGGCGCGAGAAUGUGCCAAGGCACAUUCUUAUUUAUAAACCUAUAAAAAUCAAGAAAGGGAGAGAAAAAUAAUGUUCAGACCUAUCAGGAAAAAGAAAAAUGAGA
env-1026 UAGUACACAAAAAGGCACGACCUGCCAACGAUGCGCACUCGCGCGAUGAGGAAGUUUGUCGCAAACGACCGCGCUCGGCGCGAGAAUGUGCCAAGGCACAUUCUUAUUUAUAAACCUAUAAAAAUCAAGAAAGGGAGAGAAAAAUAAUGUUCAGACCUAUCAGGAAAAAGAAAAAUGAGA
env-1040 UAGUACACAAAAAGGCACGA...
env-1045 UAGUACACAAAAAGGCA...
env-1046 GAAAAAUAAUGUUCAGACCUAUCAGGAAAAAGAAAAAUGAGAUCAGCACAGAUGAAGC...
env-1047 ...
env-1048 UAGUACACAAAAAGGCACGACCUGCCAACGAUGCGCACUCGCGCGAUGAGGAAGUUUGUCGCAAGCGACCGCGCUCGGCGCGAGAAUGUGCCAAGGCACAUUCUUUUUUAUGGUUUCUGCUCCCGUCAUCUCUUUACUUAUGGCGUGCAGAUCACCGGUAUUCCCUUGUAAAGCAGAUCA
env-1049 UAGUACACAAAAAGGCACGACCUGCCAACGAUGCGCACUCGCGCGAUGAGGAAGUUUGUCGCAAGCGACCGCGCUCGGCGCGAGAAUGUGCCAAGGCACAUUCUUAUUUAUAAACCUAUAAAAAUCAAGAAAGGGAGAGAAAAAUAAUGUUCAGACCUAUCAGGAAAAAGAAAAAUGAGA
env-1052 UAGUACACAAAAAGGCACGACCUGCCAACGAUGCGCACUCGCGCGAUGAGGAAGUUUGUCGCAAGCGACCGCGCUCGGCGCAAGAAUGUGCCAAGGCACAUUCUUUUUUACAGCAUCCUCACGAUGAACUUAACUACCUUGUAACGAUUCCAUAUCGUUGCACCAUAUUUCAUUUAGGAG
env-1053 UAGUACACAAAAAGGCACGACCUGCCAACGAUGCGCACUCGCGCGAUGAGGAAGUUUGUCGCAAACGACCGCGCUCGGUGCGAGAAUGUGCCAAGGCACAUUCUUAUUUACAUCAAAAAUCCCCGCUGGGUAAGCGGGGAAAUGUUCACAAUUCUAUUACUUUCUGUACAGAUAUUUCGG
env-1054 ...
env-1055 UAGUACACAAAAAGGC...
env-1056 UAGUACACAAAAAGGCACGAAGUUUGUCGCAAACGACCGCGCUCGGCGCGAGAAUGUGCCAAGGCACAUUCUUAUUUAUAAACCUAUAAAAAUCAAGAAAGGGAGAGAAAAAUAAUGUUCAGACCUAUCAGGAAAAAGAAAAAUGAGAUCAGCACAGAUGAAGC...
env-1057 AUGAGUAAUCCAAUCAAAAGUUUAACAAAAAAAGAAUGGGGAGUAUGGAU...
env-1058 AAGAUUUAACGAAAAAAGAAUGGAUACUUUGGGU...
env-1059 GAUAUGAGUAAUCCAAUCGCAGGUUUAACAAAAAAGGAAUGGGGGGCGUGGAU...
env-1060 UUAGGCCCAUCAGAAAAAAGAAAAAUGAAAUUGACGUAAAUGUAGC...
env-1061 GGUAAUCCAAUUGCAGAUUUAACAAAGAAGGAAUGGUGUGUGUGGAU...
env-1062 GGUAAUCCAAUUGCAGAUUUAACAAAGAAGGAAUGGUGUGUGUGGAU...
env-1070 GGUAAUCCAAUUGCAGAUUUAACAAAGAAGGAAUGGUGUGUGUGGAU...
env-1071 ACUGAUAUGAGUGAUCCAAUUGCAAGUUUAACAAGGAGAGAAUGGUGGGUGUGGAU...
env-1072 CGUAAGUUAUAUGAGUAAUCCAAUUGUAAGUUUAACUAAGAAAGAAUGGAUGGUAUGGAG...
env-1073 AGAAUAAGCAAUCCCCCGCUUCAAGUGUGUAGAGCACUAAGCGGUGG...
env-1074 UUGUUGAGGGAGUGUGUAAAGGUGCAUUUUUCUGACAAAAAAUAAAAUGUAGGAGAUGAGAAACUGUUAUGAGUAAUCCGAUUACAAGUUUAACAAAAAAAGAAUGGGGAAUCUGGAUGGGAGCGCUGCUGGUCAUCGUGGUAUCAAAUAUUUUUCAGCGAAUUUUAACAAAGGUUAGC
env-1075 UUGUUGAGGGAGUGUGUAAAGGUGCAUUUUUCUGACAAAAAAUAAAAUGUAGGAGAUGAGAAACUGUUAUGAGUAAUCCGAUUACAAGUUUAACAAAAAAAGAAUGGGGAAUCUGGAU...
env-1076 UUGUUGAGGGAGUGUGUAAAGGUGCAUUUUUCUGACAAAAAAUAAAAUGUAGGAGAUGAGAAACUGUUAUGAGUAAUCCGAUUACAAGUUUAACAAAAAAAGAAUGGGGAAUCUGGAUGGAAGCACUGCUGGUCAACGAUCACAUGGAUCCGGAAUCCGUCCGAGAGUGGAAAAGAAGUU
env-1077 UUGUUGAGGGAGUGUGUAAAGGUGCAUUUUUCUGACAAAAAAUAAAAUGUAGGAGAUGAGAAACUGUUAUGAGUAAUCCGAUUACAAGUUUAACAAAAAAAGAAUGGGGAAUCUGGAU...

...
...
...
...
●●●●●●●●●●●●●○○○○○○○○○○○○○○○○○○○○○○○○○○———————————————————————————

env-1078	UUGUUGAGGGAGUGUGUAAAGGUGCAUUUUUCUGACAAAAAAUAAAAUGUAGGAGAUGAGAAACUGUUAUGAGUAAUCCGAUUACAAGUUUAACAAAAAAAGAAUGGGGAAUCUGGAU
env-1079	GAACAAUAAGAAAAAAGAAAAAUGUACUUGAUAUAACUGAUGC
env-1080	AUCAGAAAGGAAUUGUGAAUGAUGAAAAUUGAAAAAAACAGUGCGUUGCUGGUGAUCGACAUUCAGCA
env-1081	AUCAGAAAGGAAUUGUGAAUGAUGAAAAUUGAAAAAAACAGUGCGUUGCUGGUGAUCGACAUUCAGCAGGA
env-1082	AAACGGACAUAGCAUUAAAAGACAAAUCAAUAUUAAAAAACUUUAC
env-1083	AAACGGACAUAGCAUUAAAAGACAAAUCAAUAUUAAAAAACUUUAC
env-1084	AAACGGACAUAGCAUUAAAAGACAAAUCAAUAUUAAAAAACUUUAC
Ano-1-1	AAACGGACAUAGCAUUAAAAGACAAAUCAAUAUUAAAAAACUUUAC
env-1085	...
env-1086	AAACGGACAUAGCAUUAAAAGACAAAUCAAUAUUAAAAAACUUUAC
env-1087	AAACUGACAUAGCAUUAAAAGACAAAUCAAUAUUAAAAAACUUUAC
env-1088	AAACGGACAUAGCAUUAAAAGACAAAUCAAUAUUAAAAAACUUUAC
Cst-1-1	AAACGGACAUAGCAUUAAAAGACAAAUCAAUAUUAAAAAACUUUAC
env-1089	AAACGGACAUAGCAUUAAAAGACAAAUCAAUAUUAAAAAACUUUAC
env-1090	AAAUCAUUGUUCAAAGAAUUCUUAAAAUAUUCAUCACUGAA
env-1091	AGUCUGAGAGCCGGAAGGUAGGUCAUAGGCAGAGAAAGCGGAAAGCAUUCUAAAAGAAGUGAAAAAAACAAAAAUACAGGAGAUUAUCACAUGAAAACAGAAGUUAAGCUGAAUACUUCUUACGGGAAAAAAUUUGCCCG
env-1092	AUCAUAUGUCAAACACAGGCCUUCGGGAAUUUUUCCGCCACACUGCCCUGAGCGU
env-1094	AAAGUUUUCGCGAUUUUUUUCAAUAUACAACUUUAAG
env-1095	UCGGUUUUUCGGGCUUUUGUCCGCUAUACCUUAUUAAG
env-1096	UCCCUGUUUCGGAAUUUUUCACAAUAUACAGCUUUAAGCGU
env-1097	ACUUGUUUAAAAGAAUUUGCGAGAUAUUCUAUAAUGAA
Csp-17-1	AGACUUGUUUAAGAGAAUUUGCGGGAUAUUGCAUACUAAA
env-1098	ACUUGUUUAAAAGGAUUUGCACGGUAUUCCGCUUUAAA
env-1099	AGCUUUCGUGAAUUUUUCAGAUACACGGCUUUAAG
env-1100	CUGUUUCCGUGAUUUUUCCCAUUAUACUAUCUCAAG
env-1101	AUCCAGCAUGCGGGAUUUUGCCCGCUAUACAAUUCUAAG
env-1102	UUUUAUGAGACAGCAAUCUUGUAUACGUGAUUUUAUCCGCUAUACAACUUUAAG
env-1103	UAUUCGCUGCACCACUUUGAGUGUCCUUGGUUCUUUGGG
env-1104	ACGGUCUUGUAUGGGGAAAUUUGCCCAUUAUACAAUUUUAAG
env-1106	UCUUGUAUGAGUGAUUUUAUCCGCUAUACGAUUUUAAG
env-1107	UCGGGAGACCAUCCCUGUUCGGGCAUGUCUCCUCCGGGAUCGU
env-1109	ACCUGCGCGCGGGAUUUCGCGCGCUACACCGUUUUAAG
Lsp-6-1	ACCUGCGCGCGGGAUUUCGCGCGCUACACCGUUUUAAG
env-1110	CAACAGUCUUGUAUGCGGAAUUUUACCCACUAUACAAUUUUAAG
env-1112	AAAAGAGUAUGCGAAGUA
Lph-1-1	ACGGAUUGUCUGAAGAGCUUUUUAAAAUACUCAUCCUUAAA
Cph-1-1	GGAUUGUCUUAAGAACUUUGUAAA
env-1113	GGAAAGGACAUGAUUUUUUAUGGAAAGAACAAAUUGUUUCAGAGAUUUUGCAAAGUAUGUAUCUCUCAA
Csp-13-1	ACUUGGCGACUGCCGCGAUAACCGGAGAAACUCGCAAAGCGUGUUUUUCCGGUUUUGUAUGGAGGUCUGUAUUCAAUGAAAAAUAUGACAUGCUUUAAGGAAUUUGUGAAAUACUCAUCGUUAAA
Lba-1-1	CAGCUUGUCUUAGUCAUUUUAUAAAAUACGUAUCAUUAAA
env-1114	...
env-1115	GCAAAAUGUUUUAAAGAAUUUGCGAAAUACACAUCACUGAA
env-1116	GUAUUCGGGAAUUUUCCCGAUAUACGGUCUUAAG
env-1117	GGCUUGUAUUCGGGAAUUUUCCCGGUAUACAAUUUUAAG
Mfo-1-1	UCUUGCGGCGGAGAAUUUUUCCGCUAUACGUUUUUAAG
env-1118	UCUUGUAUUCGGGAAUUUUCCCGGUAUACAGUUUUAAG
Pma-1-1	CUUGCGGGAAUUCGCCCGCUACUGCACGCAGAGCGUACUGGGGAUGCUCGGGCUGUCCUGCUAUAUCCUGGCGGACACCUAUUUCAUCGCAAA
env-1119	AGGCUUUCGUGAAUUUUUCAGGUACACGGUUUUAAGUGUUUUGGGACAUUAAGCGUAUCCUGCUAUGUUUUAGCCGAUACUUUUUUUGUUUCAAAAAGCUUGGGUUCAAAUGGACUUUCUGCCCUAAACCUUGCUAUUCCUGUUUAUAA
env-1120	UCUUACGGCCGGGAAAAAGCGAUGCUGAAAGGCGCAGGGCCCCUGGCAGACAGAGGAAAGCGCCCUGCGCUUUUCCAGUCCCAUGCAGGGAACCGGCAGCCGGUUUUUAUCAGCAAAAAUAACAGGGAGGAGACAGUACAUAUGGGAAAGAUCCACAGAGAGUUUGCAGAGUAUAUAUCG
env-1121	CGCUAUUAAAAGAAUUUUUCCAAUAUGUAAUAUUAAA
env-1122	UCUUGUCUGAAGGAAUUUUUAAGGUAUUCAUCGUUAAA
env-1123	GUCAUCCAAGUUUAAAAAUUACUUUACGAUUGGCGAAAUUGUACUUUG
env-1124	CCACCUGUGCUUGCACAGAGGUGGAUAACUGACCUUGGGACCCGCCAAAAUAUGAGGAAGAAGUGGGGCAGGGGCCCCAUGAAUUCCGAAUAUUUUGCAGAAUUGCGCAAGCACGAAGUGCGCAGCAAUUCGUCUGGUAUCAUUUGACACCCUAACUGUAAUAGGAAAGCAGAACGUUUC
env-1125	UUAAAAACAUUCUUUAAAUUCACUAGCCUAAA
env-1126	UUAAAAACAUUCUUUAAAUUCACUAGCCUAAAUGUACUGGCUAUGAU
Lsp-8-1	GACAACACUGUUUAGAGAAUUUAUAAGAUACUCAUCUUUCAG
env-1127	AGACAACACUGUUUAGAGAAUUUAUAAGAUACUCAUCUUUCAG
Dsp-3-1	CAGGAGGGUUUUAUGACGAAAAAUACACCAUUUCAGGAUUUUAUCAGGUAUUCCUUUUUCAG
Dsp-2-1	CAGGAGGGUUUUAUGACGAAAAAUACACCAUUUCAGGAUUUUAUCAGGUAUUCCUUUUUCAG
env-1128	UUAAUAAAUUUUAUAAAAUAUGCUUCCAUGAA
env-1129	CAACAAUAAAAAAAGAUUUUUUUAAGUACAUUUUCUUUAG
Lco-1-1	UUUAGUAAUUGCAGGCGUUUUCGAAGUGGUUUGGGC
env-1130	AGAUAUUUUACAGACGUCGAACUGAU
env-1131	UAAAAACUUAAAUAGAUUUGAGCUGUCACU
env-1132	...
env-1133	ACUGAAUAAACAAGAAAGUGUUUUAUGGAU
env-1134	CCGCCCCUCCGCAGAACCGGAACCCCAAGAGAAAGGACAGACUACGAAAAUGAGAGCAAAAUUGUUGUCCGGCUAUUUCACCAGAACGGAAAAAGUCCU
env-1135	CCUUUUAAAAAUCUAAGUAAAUUUGAGUGGCUUCU
env-1136	AAAAAAGAGUGGUUUGCCCUGUCACCCGGUGAGCGGGGGCUGUGGCU
Ssp-1-1	UUAGAGUUUUUCUAAAUUAAAAAUGGUUUCUUUAAAACUAUCUAACUGGUAUAAUAUCCUUAUUAUUGAGUGUAUUGAGUCCAUUCUUUAGAAUUAUUUUGAAUAUAAUAGAUUCAAAUAACUUUUUGAUUUGAUUAUUAAGAUGAAAUGAUUACCCAUUUUUUACGACCAUACAUAUGA
env-1137	AUGAAUCUCAAAAAAUCAUUUGCGGCGCUCACCACCUUCGACCGCAUCCU
env-1139	AAAUCCGAUCAGCAGGCUCACAAGGUUCGAAAAGACUCU
env-1140	AGUGAGGGGCAUGAGUCUUACCUUGAGAAAGAUAACACUUGAUGAUAUUUCAGAAUUACA
env-1142	UUUUACCCGGGGAGAGCUGAUACUCUGGGGCUC
env-1143	...
env-1147	AUAUUU
env-1148	GAAAGGGGCUUUUCAUGAAGAUCAGCCGUUGUUUCCGAAGCCUGUCAAAAUUUGAAUGGUGCCU
env-1150	CAUCGAAACUUAAUUAUUUUACCAAAGCCGAGCUUGCCCUCUG
env-1151	GGAGAUAUAAAAGGAGAAGGAAAAUAUGGCAUGGGUAGAAUUAUUAAUUGCAGGUUUUUUUGAAGUAUUAUGGGC
env-1153	CCAUUCAGAAAACUGGAUAGUUUCGAAUGGUCUCU
env-1154	UUUUCAAGGGGAGAGCUUAUGCUGUGGGG
env-1155	UUUACAAAGGGAGAGCUUAUGCUCUGGGGAGC

```
Rfl-1-1    UUUACAAGGGGAGAGCUUAUGCUGUGGGG....
env-1156   UUUACAAAUGGAGAGCUUAUGCUGUGGGGAGC....
env-1157   UUUACAAAUGGAGAGCUUAUGCUGUGGGG....
env-1158   UUUACAAGGGGAGAGCUUAUGCUGUGGGG....
env-1159   GUAACCCAUUCAAAACCUUCGUUAGGUUCGAAUGGUUUCUUUGGAUAUGCUCUAUGAUUGUGGUGGGAGGUUCCUCUCUUAUUGCAGGGAGUUUCUCCCUACU....
env-1160   GUAACCCAUUCAAAACCUUCGUUAGGUUCGAAUGGUUUCU....
env-1161   AUCUUCGUGGAUCACCUAUUUCAGUGUCGGUGAGCGUCU....
env-1162   UUGAAAAAAUAUUAUGGAUGGUAUCUGUGNNNNNNNNNNNNNNNN....
env-1163   AGAUUGACUAUUUUUCAAAAAAAGAAUUAAU....
env-1164   ACAAGAAAAUUCACACAAUUUUCAAAAUAGCGAAAAACGAGUACCCGGCUAAAGAAAAUAGCAGGAAAUAAAAAAUUAAACGAGAGCAAACUCAACGUUAUCGUUGAGUAAUUUGAAAAGAACUC....
env-1165   ACAAAAUCGGAAUGGGCUAUCUGGCUCUU....
env-1166   ACAAAAUCGGAAUGGGCUAUCUGGCUGUU....
env-1180   ACAAAAUCGGAAUGGGCUAUCUGGCUGUUUUCGUUCG....
env-1181   UGUGUAUCACUGUUUCCACUAUUUUACCAGGGGGGA....
env-1182   ....
env-1183   UUUGCAAAAUAUGUAUCACUAAAUAUUUUGGG....
env-1188   UUUGCAAAAUAUGUAUCACUCAAUAUUUUGGG....
env-1189   GAGGCUUAUUUGUUAUUUUUCAAAAGGAGAAAUUUG....
env-1190   AAAUCUUGAAAAGCACGAAUGGGCGCUGUG....
env-1191   UACAUUAUUUUAAAGUGAGUGAAAUUGUACUUUG....
env-1193   ....
env-1194   CAUAUCGUAUAUCAAGGAGGUAUAUAUAAUGGCAUGGAUCUGUUUGUAUUUGGCGGGGCUUCUGGAAGUUUUCUGGUC....
env-1195   CAUAUCGUAUAUCAAGGAGGUAUAUAUAAUGGCAUGGAUCUGUUUGUAUUUGGCGGGGCUUCUGGAAGUUUUCUGGUC....
env-1196   AUAAAUUAAUGGCAGGCAAAGGAGGUUAAAUCAUGCAGUGGAUAUACUUAAUGAUAGCAGGAAUCUUUGAAGUUGUAUGGUC....
Cbu-1-1    GGGAAUUAAAUGGAACUAAAAAUAAUAAAUCAAGAUUUUUCAGUAUGUAAAGUUAAGGA....
Csp-9-1    GGGAAUUAAAUGGAACUAAAAAUAAUAAAUCAAGAUUUUUCAGUAUGUAAAGUCAAGGA....
env-1197   GGGAAUUAAAUGGAACUAAAAAUAAUAAAUCAAGAUUUUUCAGUAUGUAAAGUCAAGGA....
Cbu-3-1    GGGAAUUAAAUGGAACUAAAAAUAAUAAAUCAAGAUUUUUCAGUAUGUAAAGUUAAGGA....
env-1199   ....
env-1200   UUAACAACUACUUCACAACCUUUGAAAAAAUACUAUG....
env-1202   ....
env-1204   AAAGCUGAAUAACUACUUUACUGCCUUUGAAAAAAUACUGUG....
env-1205   CCCGUAAAGUAUUUUUCCCGUGCGGAAAUAUGGCU....
env-1206   UUUACA....
env-1208   UUCACAGCUCACUAAAUCAGAAUGGAUUUU....
env-1209   UUCACA....
env-1210   UUCACAGCUCACUAAAUCAGAAUGGAUUUU....
env-1211   UAUGAAUAAGAUGAAAGCAUUACAAAGUUAUUUUACAAAGGGUGAAUUGCUGCUUUGGCU....
env-1212   UAUGAAUAAGAUGAAAGCAUUACAAAGUUAUUUUACAAAGGGUGAAUUGCU....
env-1214   ....
env-1215   UUGGAUUAUUUUUCAAAAGG....
env-1217   CAAUCUAAAAAACCUACUUGGAACUAACUGAUAUAAAUAUGUGAUUGGAGAGAAAAAUGUUUAGAAAAAUUCGUAAAAAGAAAAAUGAACUUGAUAUCAAUGUAGC....
env-1220   CUACUUUUCAAAGGGGGAGCUGCUUUUAUG....
env-1221   UCCUAUUUUUCCAUCGGCGAAUGGAUCUUGUG....
env-1222   GUUAUUUUACUAAAGGAGAAAUCACUCUGUGGAGCUCAUCGGUUGACUGAUUGCGGCUUCGUUUUUCAUUUUUGACAGAACAAAUUAUCUGAUUCUCUCCGCUUCGGUUAUUGGCGUAACCUCUUUGAUUUUUAAUGCUAAGGGAAAUCCAGUCGGACAAAUUCUUAUUAUCCUUUUCAG
env-1223   UUCCGCACACUAUCUAAAUUUGAAUGGUUAUU....
env-1224   AAAAACGAUAAAGAGGGAAACUGUUAUAUGGAAUCAGCAAGUGCGCUGCAGCAGGAAGAGCAGCUCAAGAAAAAGAAUAAACGGACGCCAUUUCGGGAUCUGACGCAUUUUGAAAGAGGGUUAUGGAUUGUAUCGAUGGCAGUGAUC....
env-1225   UUACGGUUUUAUUUUUCAAUGCGGGAGAUCUUAUUACUCUU....
env-1226   UUACGGUUUUAUUUUUCAAUGCGGGAGAUCUUAUU....
env-1227   AAAGGAGUACUUUACUGGAGCAGAAAUAAU....
env-1228   GCUAAAGUCUGUAAUAGGCAAUUUUUCAAGGGUUGAAAUUCU....
env-1229   UCGCAAAGUAUUUCUCGGUAGGAGAAUUAUU....
env-1230   UGCGUAUUUUUCAAGAAACGAGAUCAUGCUGUG....
env-1231   CGCAAAGUAUUUUUCGGUAGGAGAAAUCCU....
env-1237   ....
env-1238   CGCAAAGUAUUUUUCGGUAGGAGAAAUCCUGUUAUGGAGUUGUUCGGUCCUUUUAAUUAUCGUGUCGUUUUGCGUUUUCGACAGGAGAAA....
env-1239   CGCAAAGUAUUUUUCGGUAGGAGAAAUCCUGUUAUGGAGUUGUUCGGUCCUUUUAAUUAUCGUGUCGUUUUGCGUUUUCGACAGGAGAAACUAUCUGACGCUUGCAUCUUCGCUCAUAGGCGUAACGUCUCUGAUUUUUA....
env-1240   CGCAAAGUAUUUUUCGGUAGGAGAAAUCCU....
env-1241   CGCAAAGUAUUUUUCGGUAGGAGAAAUCCUGUUAUGGAGUUGUUCGGUCCUUUUAAUUAUCGUGUCGUUUUGCGUUUUCGACAGGAGAAACUAUCUGACGCUUGCAUCUUCGCUCAUAGGCGUAACGUCUCUGAUUUUUACCGCAAAAGGUAAUCCGAUAGGUCAAAUUCUGAUGAUUGU
env-1242   U....
env-1243   UGGUGAAGUACUUCUCCGUCGGAGAAAUCGU....
env-1244   GCU....
env-1245   UCUGUCAGUAAAGCUUACGGUCACGGACAUCCU....
env-1246   UGAAAACCUUUACAAAAUUCGAGCUGGC....
env-1247   UUGAAUUAGGGCUGUGGGCGGUUUCGGUAUC....
env-1248   ....
env-1249   ACAAUAACUUAUCAACAUUUGAACUUUUUUU....
env-1250   ACAAUAACUUAUCAACAUUUGAACUUUUUUU....
env-1251   ACAAUAACUUAUCAACAUUUGAACUUUUUUU....
env-1252   UUUUUCAAUCGGAGAGCUUUUGCUUUGGGGAAU....
env-1253   AGGUAUUUCACAACAGGGGAGCUUCUGCUCUGGGGAUU....
env-1254   UUGAUGGUGUAAUACUAGAUAGUGAAAAAAC....
env-1255   AAACUAUUUUUCAAAAACCGAAAUUGU....
env-1258   CCCCAAAAUGUCAAAACUAACCACAUAUUUCACAAAAUCUGAAUUACUCCUCUGGAG....
env-1260   UACGACAUCAUUCUCUGGACUGCUUCUGUAUU....
env-1262   UCAUAUUUUACGAAAUUCGAGCUUGCGCUGUGGUU....
env-1263   UCAUAUUUUACGAAAUUCGAGCUUGCGCUGUGGUU....
env-1264   CAAAAAUCCGUUUAAGCGAUUAACUAAAUUUGAGUGGGGAUU....
env-1266   AAGAUUUUACACGAACCGAAGUUUUAUU....
env-1269   AAGAUUUUACACGAACCGAAGUUUUAUUGCUUGUUUUGUCCGUACUCACAAUUAUAGCAGUUUUCUGCAUUUUUGACAGGACAAAUUAUCUUG....
env-1270   UUUAAGACGCUUACCAGGGCGGAGUGGUUGCU....
env-1271   CUACUUUACCAAAUGGGAAAUGGCACUUUGGUC....

           ....
           ....
           ....
           ....
           ●●●●●●●●●●●●●○○○○○○○○○○○○○○○○○○○○○○○○○○○−−−−
```

```
Eba-1-1    ACACAAUACUUUACCAAGGGAGAAUUGUU...
env-1272   AACAGAAAGGGAAAA...
env-1273   ...
env-1274   UAUACUAUUAUAACGUGAGUUCGAUGAAAAAAGAAUAGAAAAAGCCGCCAAAAUCUGUUAU...
env-1275   UGUUCAUCGGUCACACUUAUUAUAGGCUCAUUUCUGAUAUUCAGUCAUGACGGAUAUUUAAACCUGUGUGCCUCCCUCGUCGGAGUAACAGCACUUAUAUUCAUUGCCAAAGGAAAUCCCAUCGGUCAGCUGCUUACGGUAUUUUUUAGUAUAAUGUACGGAAUAAUAUCUUAUUCCUGC
env-1276   GAGAAGUAUUUCUCCUUACAGGUUCAGUAUU...
env-1278   CUUUUAUUGGUUUAACGAAAUUUGAAUGGUGUUU...
env-1279   CUUUUAUUGGUUUGACGAAAUUUGAAUGGUGUUU...
env-1288   CUUUUAUUGAUUUGAAUGGUGUUUAUGGACCAUAUCUGUGGUUGUCGUUACAGGUUCAUUUGUACUGUCAGGUUCGUCUGAUAUAUU...
env-1289   CUUUUAUUGGUUUGACGAAAUUUGAAUGGUGUUUAUGGACCAUAUCUGUGGUUGUCGUUACAGGUUCCUUUGUAUUGUCAGGUUCGACUGAUAUAUUAACGAUUAUAGCUUCACUAAUCGGUGUUACAGCUCUUAUUUUCGUUGCUAAGGGGUAUGUUUUUGGACAGAUUUUAACAGUAA
env-1290   CUUUUAUUGGUUUGACGAAAUUUGAAUGGUGUUU...
env-1291   CUUUUAUUGGUUUGACGAAAUUUGAAUGGUGUUU...
env-1292   UCGUUUAAAAGUCUUACCGUAUUUGAGCUUGUGCU...
Cbo-1-1    GGAGGUAACAUAUGAAAAAAUUUAAUCUCUUUGCUACGUUAACAAAACAGGAGUGGAUAUU...
env-1293   ...
env-1294   UUGGAAAGAUCUAACCGUGUUUGAGCGGGUGCU...
env-1296   UUGGAAAGAUCUAACCGUGUUUGAGCGAGUGCU...
env-1300   AUGGGGC...
env-1301   AAGUCGCCCUUUAGAGCGUUUACAAAAUUUGAUUGGGGGUU...
env-1302   AACAUUGGCUAUUUUUCAAAGGCAGAAUUAAU...
env-1303   GCUACUUUUCAAAGGGAGAAAUUGCCCUGUG...
env-1304   GGUAUUUUACCGCCUUCGAACUGGCGCUGUG...
env-1305   GGUAUUUUACCGCCUUCGAACUGGCGCUGUG...
env-1306   AGAUUUUAUAAAAGACUUUACUUUAGGCGAAAAAAAUAUUGUGGCU...
env-1307   AAAUUUUAUAAAAAAACUUUACCUUGGGUGAAAAAAAUAUUGUGGCU...
env-1308   AGAUUUUAUAAAAAAAAUUUACUUUAGCUGAAAAAAAUAUUGUGGCU...
env-1309   AGAUUUUAUAAAAAAACUUUACUUUAGGUGAAAAAAAUAUUGUGGCU...
env-1310   AGAUUUUAUAAAAAAACUUUACUUUAGGUGAAAAACUAUUGUGGCU...
env-1311   GAUUGUGUCAAGUUUUCUAUGAAAACUUGACAUUUAGCGUCAGCCCAUUUGCUGAAAGCAAAUUUGGAAUGGGCUGAUGUUCAGUGGAGGAGUUUGAAAAAAUGAUAGAAAAGAAGCUGAGAAAAUUUAUAUUUCCAAGCGUAUUUGCAAU...
env-1312   AUUAUUAAGAUACUUUAAAAAAGAGGAAAUCAU...
env-1313   AAAAUACUUUAAUAAGACGGAAAUCAU...
Bma-2-1    ...
env-1315   ...
Csp-4-1    ...
env-1316   ...
env-1317   UUUCAGUUACUUUUCAAAAGCAGAGCUGGCGCUUUGGAUUUCUUCCGUCGCCCUUAUCGUCAU...
env-1318   UUUCAGUUACUUUUCAAAAGCAGAGCUGGC...
env-1319   UUUCAGUUACUUUUCAAAAGCAGAGCUGGC...
env-1320   UUUCAGUUACUUUUCAAAAGCAGAGCUGGC...
env-1321   UUUCAGUUACUUUUCAAAAGCAGAGCUGGC...
env-1322   UUUCAGUUACUUUUCAAAAGCAGAGCUGGCGCUUUGGAUUUCUUCCGUCGCCCUUAUCGUCAU...
env-1323   UUUCAGUUACUUUUCAAAAGCAGAGCUGGC...
env-1324   UUUCAGUUACUUUUCAAAAGCAGAGCUGGCGCUUUGGGUUUCUUCCGUCGCCCUUAUCGUCAU...
env-1325   UCUUUUCCAAAAACGAACGGCUCCUAUGGUGCUUUUCGGUUGCCUUGAUUCUUGUCUCUUUCUGUAUUUUCGACAGGAAAAACUACCUGACCCU...
env-1326   CUCAAACACUAUUUUUCAAAAACAGAGAUUAU...
env-1332   CUCAAACACUAUUUUUCAAAAACAGAGAUUAU...
env-1334   AUC...
env-1344   AUC...
env-1345   AUCAUUUACCACACUCAAAAACUACUUUUCAAAAACAGAGCUGAU...
env-1363   AUCAUUUACCACACUCAAAAACUACUUUUCAAAAACAGAGCUGAU...
env-1364   AUCAUUUACCACACUCAAAAACUACUUUUCAAAAACAGAGCUGAUUCUCUGGAGUACCUCAGUCGUUUUGAUUCUGAUUGCUUUCUGUCUCUUUGACCGGACCAGUUAUAUGACCCUUUGGACCUCAUUGAUCGGAGUGACUUCUCUCAUCUUCUGUGCCAAGGGAAAUCCCAUGUGACA
env-1365   ...
env-1366   ...
Mma-1-1    UUUCGAAAUUUGACAAAAUUUGAGCGGAUUUU...
env-1367   ...
env-1371   AGAAAGGUGCUUAUUUUUCAAAAGGGGAGCUUGC...
env-1372   ...
env-1374   AGAAAGGUUCUUAUUUUUCAAAAGGGGAGCUUGC...
env-1375   AGAAAGGUUCUUAUUUUUCAAAAGGGGAGCUUGC...
env-1377   AGAAAGGUUCUUAUUUUUCA...
env-1378   ...
env-1386   AGAAAGGUUCUUAUUUUUCAAAAGGGGAGCUUGC...
env-1388   AGAAAGGUGCUUAUUUUUCAAAAGGGGAGCUUGC...
env-1392   ...
env-1393   ...
env-1399   ...
env-1400   AGAAAGGUUCUUAUUUUUCAAAAGGGGAGCUUGC...
env-1401   ...
env-1402   CUGAUUCAUUACUUUUCUUUUGGCGAAAUGAU...
env-1403   AUCAAUUAUUUUUCAAAAACGGAAAAGAUCUUAUG...
env-1404   AUCAAUUAUUUUUCAAAAACGGAAAAGAUCUUAUG...
env-1405   ...
env-1406   UGGAUCGCCCGCCUCCGCAUCCCGUGCUUCGGCCGGAC...
env-1407   AAAGCAUUGACAAAAUUGGAAUGGGGCUUGUGGCU...
env-1408   CUGUUACGCAUUUUUACCUUGAGGGACAUAUU...
env-1409   CGGCUUAUUUUUCAAAAACCGAGCUUAUUUUAUGGAU...
env-1410   AGAGAAAUGAGAAG...
env-1412   UUAUCAAAAAAAGCUUUUAACUAUUUUUCUGCUGCUGAAAAAAACCUU...
env-1413   UAACAGUUGACAAUAAAGUUAGCAGCGACUAACAUUAUGGGUGUUAAGAGAAUUGGAUCAGGGGGAUGUAACAAAUGAAAGGUUCUGAGACUAUAAAAGAAACACUGCACAAGGCGGAUUGUGU...
env-1414   CACCUAUUUUUCAAAGAGUGAGUUGACCUUGUGGGG...
env-1415   ACAACAACCGAAAAAAUCAUCUGGUGCGU...
env-1416   UUAUUUUUCAAAGGGCGAAUUGAUAUUGUG...

           ...
           ...
           ...
           ...
           ●●●●●●●●●●●●●○○○○○○○○○○○○○○○○○○○○○○○○----------------------------------------
```

```
[gl-1-1    UGUGAAUUACUUUUCCAAAGGAGAAAUCUU...
env-1419   AAGGAGAAUAUAUUUAUGAAAAAUUAUUUUACAACGGGAGAAAAGCUGCUGUGGGGCUGUUCCGC...
env-1421   UUUCACAAAUUAUUUUUCAAAAGUCGA...
env-1422   UUUCACAAAUUAUUUUUCAAAAGUCGAGAUUUUGCUGUGGUGCUGCUCUGUUAUUUUAAUCACAGUAUCCUUUUGUAUUUUUGACAGAGAAAACUAUUUAACACUAACAGCUUCUCUCAUUG...
env-1423   ...
env-1424   ACUACAUCAACUAUUUUUCCAAAGGAGAGCU...
env-1425   UAGUCAAACGCAUCACAUCUUAUUUUACGCGCGGAGAACU...
env-1427   AAUCGCU...
env-1428   UAAAAAAUCCUUAUCAUACUUUACGAAAGGGGAGCUUGC...
env-1429   ACUGAAAUCCUUGGUUCGUUAUUUUACCAAAGGUGAGCG...
env-1431   ACUGAAAUCCUUGGUUCGUUAUUUUACCAAAGGUGAGCG...
env-1434   ...
env-1435   UUCACUAAGACUGAACUGGCGCUGUGGCUGUGCUCGGC...
env-1438   GGAAACGGUAACAAACAUGAAAAACAUAUUACG...
env-1439   AUUUUUCUGCGGGAGAAUGGUGCUUGUGGGG...
env-1440   CUUUUUAAAAGCCUUUCGAGAUUUGAGCUUUG...
Cbo-3-1    AAUUAUGUUUAGAGAAAUGCGUAGAAAAAAUCAAUUACUUACAGAGAACGAAAC...
env-1441   AUCGUUUGUCACUUACUUCUCUAAGAAAGAGUAUCU...
env-1442   ...
env-1443   AAGCGAACUUGCUUUAUGGCUCGGCUCAAUGCU...
env-1444   AAGCGAGCUUAUCCUGUGGUUUGGCUCAAUGCU...
env-1445   AAGUGAGCUUAUCCUGUGGUUUGGCUCAAUGCU...
env-1446   ACUGAGUAUUUCACAAAAUUCGAGCUUGUUUU...
env-1447   GGGGCUUUACGGCACGGGAUAUCGCGCUCUG...
env-1448   GGGGCUUUACGGCACAGGAUAUCGCGCUCUG...
env-1449   UGAUAUGGAUAAGAUCUAUAAUAAGUAAUUUUUCAAAGGGAGAAUUCAUUUUGUGGGGUACUUCUGUUUGUAUGACCGUUUUGUCAUUCUUGAUUUUUGACAGGACAAAUUACCUUACGCUGACUGCUUCUCUUAUAGGUGUGACGUCU...
env-1450   UGAUAUGGAUAAGAUCUAUAAUAAGUAAUUUUUCAAAGGGAGAAUUCAU...
env-1452   UGAUAUGGAUAAGAUCUAUAAUAAGUAAUUUUUCAAAGGGAGAAUUCAUUUUGUGGGGUACUUCUGUUUGUAUGACCGUUUUGUCAUUCUUGAUUUUUGACAGGACAAAUUACCUUACGCU...
env-1454   CUUUUAAAAACCUUACAAAAUUUGAGAUUAUCCU...
env-1455   UUUCAAAAUUUUAAGAAACACGAUUGGGUGAUUUGGAU...
env-1456   CCGCGCUUAUCAGUAUUUCACCACACAGGAAAGGAUCCUUUG...
env-1457   CGCAUCAAUUCGUAUUUUACAACACAGGAAAAAGCGCUCUG...
env-1458   CGCAUCAAUUCGUAUUUUACAACACAGGAAAAAGCGCUCUG...
env-1459   CGCAUCAAUUCGUAUUUUACAACACAGGAAAAAGCGCUCUG...
env-1460   CGCAUCAAUUCGUAUUUUACAACACAGGAGAAAGCGCUCUG...
env-1461   ...
env-1462   UUUUCCAAAACAGAGAUGUAUUUGUGGAG...
env-1463   CAAAGUUUGAAAUCUUUUUAUGGUUAUU...
env-1465   AACAGAGCGUUUUUAAAAUAUUUUUCAAAAGGAGAAAUCAUACUGUUGGCAGCCUCGGUCGGGGCGAUAGUAAUAGCGUUUUUGGUGUUCGACAG...
env-1466   GAUGAAAAAACAAACUGUAUGCCUUAAAAUCUUAUUUUUCAAAAGGAAAUCUGCUUUUAUGGAGCGCAUCCACACUUUUUGUCCUGCUGUCCUUUGCUGUUUUUAACGAAGAAAAUUAUCUGAUCCUGCUCUGGUCGAUGGCUGCGAUGAAAAAUGUCGCUUAUACCUCCGUCGUUGUCU
env-1467   GAUGAAAAAACAAACUGUAUGCCUUAAAAUCUUAUUUUUCAAAAGGAAAUCUGCUUUUAUGGAGCGCAUCCACACUUUUUGUCCUGCUGUCCUUUGCUGUUUUUAACGAAGAAAAUUAUCUGAUCCUGCUCUGGUCGAUGGCUGCGAUGGAAAAUGUUGCUUAUACCUCCGUCGUUGUCU
env-1468   AUUGAAAAUGAGAAGUAGGGGGUAUCCAUUUCCUUAAUCGCAUCACAAACAAG...
env-1469   UUCCUAUUUUUCAGGGAUCGAGAAAGCCAU...
env-1471   AUAUUUUUCAGGUCUUGAGAUAGCGAUAUG...
env-1473   GUCUCAUGAUCGCAAAAAAUAAAAAAAGCCGUAUCGUAUUUUACGGCGGCGGAAAUUAUCCUUUGGGCGGUAUCCGUCCUUCUCAUCACAUCGUCGUUUUUCAUUUUCGCAAGGCGCGAAUACCUUACGCUCGCCGCGUCGCUCGUCGGCGUGACGGCUCUCAUCUUCUGCGCGAAGGGA
env-1474   GUCUCAUGAUCGCAAAAAAUAAAAAAAGCCGUAUCCUACUUUACGGCGGCGGAAAU...
env-1475   GUCUCAUGAUCGCAAAAAAUAAAAAAAGCCGUAUCGUAUUUUACGGCGGCGGAAAU...
env-1476   UCAAUGUUAAAUUAUUUUUCCAAAGGAGAGUUGCU...
env-1477   UCAUGGGUUCCUUUACUCGGGGAGAAUUGCU...
env-1478   ...
env-1479   UUUUCAAAAACGGAAAUUGUAUUAUGGAG...
env-1480   AAAAGGUUCAAUAAUUAUUUCACAAAAGGGGAAACGGCGCUUUGGAGCGUGUCGGCCUUGCUUAUCGUUAUCUCAUUCUGUGCUUUCGACAAAGGAAAUUACUUGAC...
env-1481   UGAAAAUCAAAGAUUCAUUUGCCGCAUUAUCCCGUUUUGAGUGGGCGUU...
env-1482   GCGCUCGCCUAUUUUUCAAAAACCGAAAAGGUCUUAUGGAGCGCUACGGAUUUUUGAGCUGGCGCGCCAUGGAGAAGAGGAAGGCAGGGUAAUCCGAAAAAUUCACCGCGGACGCGGCGGCUCGGAUAGACAAACGGAAGCCGCUGUGUUAUGAUAGAUAAAAAGAAAGAUUCGGAUCGG
env-1483   UUCACAAAAACCGAAAUCGCUCUCUGGAGCGUCUCCGC...
env-1484   ...
env-1485   ...
env-1486   AAGGCAUUGACGAAGUUUGAAUGGGGUUUGUGGAU...
env-1487   CCUUUUCGCAAUUUAUCUAAAUUUGAAUGGAGCCU...
env-1488   CCUUUUCGCAAUUUAUCUAAAUUUGAAUGGAGCCU...
env-1490   GUAAUAUGUACUGUUGCUGCAUUCCUUUAUUUUGACAGGGAAAGUUUUAUGACGCUUGCUGCGUCGCUUAUUGGAGUUACUUCGCUUAUCUUUAAUGCCAAGGGUAAUCCGAUAGGGCAGUUUCUCAUGGUUAUAUUCAGCCUUCUUUACGGAAUUAUUUCAUAUUCAUUUUCAUACUAC
env-1491   UCAAAGGCUUUACGCGUAGGGAGAUAGUGCUUUGGCGCGCAUCCGUUUUUCUGUCGGUUGCGGCGUUCUGUCUGAUUAUUUUUCGUUAACUUCAUCGAUCUAGGUGAGACUUCACUCAUUCUGAAUACAAAAGGCAAAUUUUGUCGAGAUUUGCGAUUAUCACCACACACCGAGAACCGU
env-1492   UCAAAGGCUUUACGCGUAGGGAGAUAGU...
env-1493   UCAAAGGCUUUACGCGUAGGGAGAUAGUGCUUUGGCGCGCAUCCGUUUUUCUGUCGGUUGCGGCGUUCUGUCUGAUUAUUUUUCGCUGACUUCAUCGAUCUAGGUGAGACUUCACUCAUUCUGAAUACAAAAGGCAAAUUUUGUCGAGAUUUGCGAUUAUCACCACACACCGAGAACCGU
env-1494   GCAAAUACUUCUCACUGUUUGAGAUUGUUUU...
env-1495   GCAAAUACUUCUCACUGUUUGAGAUUGUUUU...
env-1496   AGAAUUUUUACAAAGGGCGAAAUAGCAUU...
env-1497   CAGAAUUUUUACAAAGGGAGAAAUCGCAUU...
env-1498   CAGAAUUUUUACAAAGGGAGAAAUCGCAUU...
env-1499   CAGAAUUUUUACAAAGGGAGAAAUCGCAUU...
env-1501   AAGGUUGAUCGACUAUUUCUCAAAAGGCGAGCUCCU...
env-1502   AAGGUUGAUCGACUAUUUCUCAAAAGGCGAGCUCCU...
env-1503   CGGCUAUUUUUCAAAAGGCGAGCUCCUGCUAUG...
env-1504   ...
env-1505   UCUUACUUCUCAAACAGUGAGCUGCUGCUCUG...
env-1506   AAGGAAAAAAGAAAAAGUAUUUCACAGGUACAGAAAUCUUACUGUAGAUAGGCUCUGUAAUACUGAUUUUGGCGUCCUUUUUGAUUUUCGGCGGAAGUUCGUAUAUUUCCCUGAUAGCGUCCUUAAUAGGUGUAACCGCAUUGAUUUUCAUUGCAAAAGGCAAUCCAUUGGGGCAGUUAU
env-1509   AAGGAAAAAAGAAAAAGUAUUUCACAGGUACAGAAAUCUU...
env-1510   GGUUGAUUUCUUGUCAUUCCGAGUGACUUGCUUUCGCUGCAGUGCAGGAAAAAGCGAGCAACGGCGGGCGCCGCAAGAGUGGUAAGCGCGAAAGAAUCCGUAUUUACUUAAAAGAAGAAACGGAUUGCCGCACACCUGACGGUGGUCUCGCCUGCCGGCUCGGUCGGUGCUUCUGCUUCG
env-1511   GGUUAAUUUCUUGUCAUUCCGAGUGACGUUGCUUUCGCUGCAGUGCUGGAAAAAGCGAGCAACGGCGGACGCCGCGGUGAAAAGUUGUUCGCGCUCCAAGCAAAUAACAGCUUUUGGGCGCCGCAAGAGUGGUAAGCGCGAAAGAAUCCGUAAUUCCCCUUUAAAAGAGAAAGGAUUGCC
env-1512   GGUUAAUUUCUUGUCAUUCCGAGUGACGUUGCUUUCGCUGCAGUGCUGGAAAAAGCGAGCAACGGCGGACGCCGCGGUCAAAAAUUGUUCGCGUUCCAAGCGAAUAACAACUUUUGGGCACCGCAAGAGUGGUAAGCGCGAAAGAAUCCGUAAUACCCUUAAAAAGGAAAACGGAUUGCC
env-1513   ...
env-1514   UUUUUCAAAGGCAGAAAUACUUUUAUGGUU...
```

```
env-1516 GUUUAAUUAUUUUACAAAAAAAGAAAUCGCGCUGUG...
env-1517 AAAUUGGUCGGUUAUUUUUCUAAAGGCGAGCUUUU...
env-1518 CUGAUAACGCAAAGGCGUAAAUAUUUGGGUUUUUUUUAGGCCGGCUGUAUAUUUACCUGCCAACUUUUUUAUAAUUAUAUCUAUAACCAAACAUGGUGCAGGCUGGUUACAUCAUGCUUUUUUAUUGCUGCAUUUUAAUUUUUUAGAAAAAGGGAAGAUUUUCUGGGAAACCUAUUGACA
env-1520 ACUUAUUCAAAGAAUUGUGCGGCUUUUCCUACAUGUGCAAUUUCUUCGUCAA...
env-1521 UUUACGGUUAUUUUUCUAAAAAUGAAAUCGUGCUUUG...
env-1523 ...
env-1524 AUUUUACAAAAUUCGAGGUUGGCUUGUGGCU...
env-1525 CUAUUGUGUUAUUUUUCGUCAGAAGAAAAACU...
env-1529 AUCGGAAAACCUGCUUAUUUCUCAAAGACCGAGGUGAU...
env-1530 GGAUGCAAUAAAUUAUUUUUCGAAGGCUGAAAUAAU...
env-1532 UACUUUUCAAAGUUCGAAAUCGCGCUUUGGUG...
env-1535 UUUAUUGAAUUAUUUUACCAAAGGAGAAAUUGU...
env-1536 AUUCAUUAUUUUACCAAAACAGAAAAAAAUUUUAUAGCUGAUUUCUGCCGUCGGUAUUGUCAUAUCUUUCUUUUCCUUUGACAGGGAUAGCUAUAUCACGCUUAUGGCAUCGCUGAUUGGGGUGACUUCGUUAAUAUUUAAUGCCAAAGGAAAUCCCGCAGGACAAUUUCUAAUGAUUGUC
env-1537 AUUCAUUAUUUUACCAAAACAGAAAAAAAUUUUAUAGCUGAUUUCUGCCGUCGGUAUUGUCAUAUCUUUCUUUUCCUUUGACAGGGAUAGCUAUAUCACGCUUAUGGCAUCGCUGAUUGGGGUGACUUCGUUAAUAUUUAAUGCCAAAGGAAAUCCCGCAGGACAAUUUCUAAUGAUUGUC
env-1538 GAUUUGUUAUUUCACAAAAACGGAAAAAAAAUUUAUGGCUGUUUUCUGUCAUUGGAAUUAUUAUAGCUUUUUUGCUUUUGACAGAGAUAGUUAUCUUACGCUUAUGGCGUCAUUGAUUGGAGUGACUUCGUUAAUAUUUAACGCGAAAGGAAA...
env-1539 AGCGAGCUUGCGCUUUGGCUUGGGUCUAUGCU...
env-1540 CUUUUCAGUUGCUGAAAUUGUUUUAUGGAG...
env-1541 AUUUUUCAAAAAGUGAAAUUGUUUUGUGGAGUUCUUCUGUAAUGGUUAUUAUAAUUUCAUUCUGUGUUUUUGACAGAAUAAAUUAUAUGACUUUGUGUGCAUCGCUAAUAGGAGUAACAUCUUUGAUAUUUAACGCAAAGGGAAAUCCGUUUGGACAAUUACUUAUGGUUGUAUUCAGUU
env-1542 AUUUUUCAAAAAGUGAAAUUGUUUUGUGGAGUUCUUCUGUAAUGGUUAUUAUAAUUUCAUUCUGUGUUUUUGACAGAAUAAAUUAUAUGACUUUGUGUGCAUCGCUAAUAGGAGUAACAUCUUUGAUAUUUAACGCAAAGGGAAAUCCGUUUGGACAAUUACUUAUGGUUGUAUUCAGUU
env-1543 AUUUUUCAAAAAGUGAAAUUGUUUUGUGGAGUUCUUCUGUAAUGGUUAUUAUAAUUU...
env-1544 ...
env-1545 AUUUUUCAAAAAGUGAAAUUGUUUUGUGGAGUUCUUCUGUAAUGGUUAUUAUAAUUUCAUUCUGUGUUUUUGACAGAAUAAAUUAUAUGACUUUGUGUGCAUCGCUAAUAGGAGUAACAUCUUUGAUAUUUAACGCAAAGGGAAAUCCGUUCGGUCAACUUCUUAUGAUUCUUUUCGUAA
env-1546 AUCUUCUUUUACUGCUGAUACUGGUACAUAUCGUUUAUGUGCUGAUAAGGUGGUAUGUAAAAGGGUGUAUGAGGUCAAGAGGGUACUACUAAAUUGACACCCCAGAAAUGGGGUGUUAUAUUUUAAUAAAAAAGGUAAAAAAACUUCAUUGAUAAUAUAAGCCCCCUAUAGCCCUUUAGG
env-1547 ...
env-1553 ...
env-1602 UUUUUCAAAAAGUGAAAUUGUUUUGUGGAG...
env-1603 AUUUUUCAAAAAGUGAAAUUGUUUUGUGGAGUUCUUCUGUAAUGGUUAUUAUAAUUUCAUUCUGUGUUUUUGACAGAAUAAAUUAUAUGACUUUGUGUGCAUCGCUAAUAGGAGUAACAUCUUUGAUAUUUAACGC...
env-1604 ...
env-1607 NNNNNNNNNNNNNNNNNNNNNNNNNNNNNNNNNNNNNNNNNNNNNNNNNNNNNNNNNNNNNNNNNNNNNNNNNNAGGACUAUUUUUCAAAAAGUGAAAUUGUUUUGUGGAGUUCUUCUGUAAUGGUUAUUAUAAUUUCAUUCUGUGUUUUUGACAGAAUAAAUUAUAUGAC...
env-1608 AUUUUUCAAAAAGUGAAAUUGUUUUGUGGAG...
env-1682 AUUUUUCAAAAAGUGAAAUUGUUUUGUGGAG...
env-1689 AUUUUUCAAAAAGUGAAAU...
env-1690 AUUUUUCAAAAAGUGAAAUUGUUUUGUGGAG...
env-1691 GGGAGGGCUCCUCUUCGGGUAUGACUGGGUAGU...
env-1692 ...
env-1693 AUUUUUC...
env-1695 AUUUUUCAAAAAGUGAAAUUGUUUUGUGGGG...
env-1696 AUUUUUCAAAAAGUGAAAUUGUUUUGUGGAG...
env-1706 AUUUUUCAAAAAGUGAAAUUGUUUUGUGGAGUUCUUCUGUAAUGGUUAUUAUAAUUUCAUUCUAAGUUCUAAAUCUCUAUGGCAGCUC...
env-1707 ...
env-1708 AUUUUUC...
env-1711 AUUUUUC...
env-1712 AUUUUUC...
env-1713 AUUUUUCCAAGUCUGAAAUUGUUUUAUGGAU...
Rfl-2-1 AAAGAUUAUUUUUCAAAGGGUGAGCUUAUGCUGUGGGGCAUUUCAACGCUUUGCGUUCUUGUUUCGUUCUUCAUGUU...
env-1715 AGAAUACUUUUCAAAGACGGAAAUCUUGAUAUG...
env-1717 AGAAUACUUUUCAAAGACGGAAAUCUUGAUAUGGAGCAUUUCAAGCUUAUUGGUGAUCGUAUCAUUUUUCCUGUUCGACA...
env-1718 AGAAUACUUUUCAAAGACGGAAAUCUUGAUAUG...
env-1719 AUUUCAUUAUUUUUCUAAAGGGGAGUUGAU...
env-1720 ...
Oru-1-1 GUUUCACUAUUUUUCCAGAGGAGAACGGAC...
env-1721 GCAACUCGCCCCUCUGCUGAAUCAAAACUCCCGAAAGAAAGGAUCGGUUACAGAAAUGAAAGCAAAAUUAUUAUCCGGCUAUUUUACCAAAACAGAAAAAAACGCU...
env-1722 AGCUUAUUUUUCGGCGGGCGAGGCAUUGCUGUG...
env-1723 AAAUCCCUGUGUUCAUAUUUUUCAAAGGGCGAGAUCAUGCUUUGGUGCAUAUCGGUCUCAUUUAUACUUAUAUCGUUUUUCAUAUUCGAUCGGGAGAAUUAUCUAACGCUUACCGCCUCCCUUAUCGGAGUAACGUCGCUUAUAUUCAACGCUAAAGGGAAUCCGUUCGGGCAAUUGCUG
env-1724 AAAUCCCU...
env-1725 AAAUCCCUGUGUUCAUAUUUUUCAAAGGGCGAGAU...
env-1726 AAAUAACUACUUUUCAAAAGGAGAAUUUUCACUUUGGAUCUGCUC...
env-1727 AGAAUAUUUUUCAAAGUCUGAGCUCAUGCUUUG...
env-1728 AAGGGUAUUUUUCAAGGACCGAGAUCAUGAUAUG...
Ral-1-1 AGAAUAUUUUUCAAAGUCUGAGAUCAUGCUUUG...
env-1729 CGAGAUGUGGAGUUUAUUACUUUCAACAAUUCUAACAAGUGCUGCCGAUAGUUU...
Gni-1-1 GUUAAAAUCUUUGUUUCAUUAUUUUACAAGAGGUAAAAGCCCUAUGUGUUAGGAUAGUUGUCGUAUAGAAUGUAUAAUAGAAUUACGACAAUAACCUAAGGAGAAAAAAGUAUUCGUAAGUUUUUGUUCAAAAUUAAGGGGUCAAUGCGGGUUUUUUUGGAGAGUAAUUCUAUAAACAGA
env-1730 GAAAACAAAACCUAUACUGAAUUACUUUACAAAAGUGGA...
env-1731 UUUCAAAACUAACAGUAUUUGAAUGGUUUCUUUGGAUAGCUUC...
env-1732 AGAAAGUAAAAUCUUUACUGAACUAUUUUACAAAAACAGA...
env-1740 ...
env-1741 ...
env-1742 ...
env-1743 CAUUUAAAAUGCUUACAAGAUUCGAACGAAGAUUAUG...
env-1744 UGUCGGGAUACUUUUCCAAGGGCGAGAUUGC...
env-1745 UUACAAACUAUUUUUCAAAAUUUGAAAUUUC...
env-1746 ...
env-1747 AUCAUAUUUUUCAAAAGGAGAAAUUUGGUUAUG...
env-1748 AUCAUAUUUUUCAAAAGGAGAAAUUUGGUUAUG...
env-1749 GGAAGUUUGUUAUUUUUCUCUUUGCGAGCUUAU...
env-1750 UUAUAAGACUUACAGCAUUUGAAUGGCUGCU...
env-1751 CGCGCAUGGAAAAUGCGUAAGGCGCACUGUUUUUUGAAAGAAAGAGAUUUGAUAUGAGCAGCAAAAAUAACAGAUAUAAGCGUACCCCAUUUCGGGAUCUGACACGGUUUGAGCGAGGGCUUUGGACGCUGGCUUCGAUGGAUGACAGAUCCUAUCUCCCGAUGCUUAUGUGUUUUGUCA
env-1752 GCGUUUAUUUCAUUCCACAGUAUUUCAAUAC...
env-1753 GCGUUUAUUUCAUUCCACAGUAUUUCAAUACGGCAAAUUUGUUCACGAGCACACUUUCUGAGGAAAUAAGCUUUAUUGCCGCAAUCCUCCCCAGCAGCACAGCUCACUGUUUGCGCCCCGGCAUGCCGCAAAUAAUAUUGUGCUGCCGGUUCUUCGGACGUGGCAAGCGCGGCGCAGUCG
env-1754 CAUGAAGAAAUUUAUUAUUGGACAGGAUUUGAUGGAAAUUUUCCCUGAAGU...
env-1756 UUUUCGUCUGCUGUCAAAAUUUGAAUUGGGAUU...
env-1757 AAAAAUAGAUCUGCUAUUAUGGAGCGGAUCUAUUCU...

...
...
...
...
...
```

```
env-1759  GAACAGGAACUUCUCAGUAUUUUCCCCGAGGU......
env-1761  GAAUGAAAAAAUUUAUCAUCGAGAAGGAACUUUUGAAUAUUUUU......
env-1762  GUCUGAAGGGUUAUUUUACAAAAGCGGAGAU......
env-1763  GUCUGAAGGGUUAUUUUACAAAAGCGGAGAU......
env-1764  UUUUUC......
env-1765  CUUUCCGCAAAUUGACCGGCUUCGAAUGGGGCCU......
env-1766  CUUUCCGCAAAUUGACCGG......
env-1768  CGAAAAGAUCCUCUGGUGCAGCUCCGU......
env-1769  GGCAAUAGACUCUUUGUUCCCGGAAAAAUUUAAAGGAUGUUCAUAAAUGAUACGAGAUAAGCGCACACUACCACUAUUGGAUCCUUGGACUUCUUCUUUUUGUCAUAAACAAAUUACCAGAUAAGAAAAAGGAGUUUAUAAUCAUGAGUAAUCCCGUAAAAGAUUUUACAAAAAAAGAAU
env-1770  UUAUAAUAAUGAGUAAUCCCAUCAAAACUGAUGCAGGCGGGAGGCUGUUCAAGGCUGACCCAUAGAAAGCAAAGAGUCGCUUGCCGACUCUUUACCCAUUAUUUUUCCGGUAAGGAGGCAAAGAUGCCGCUGUCUGAUUACGAAAAUCAUACUUAUAUUGCGAUUGAUUUAAA.......
env-1771  CAACUUCCUUUUUUUCUUUUUGAUCGGGAAAAUCACCUUACUCUGACAGCAUCGUUUAUCGGAGCUGUAUCGUUAAUUUUCUGUAUAAAAGGGAAUCCUUUCGGACAAUUCCUCAUGGUUGUCUUUAGUGUUUUCUAUGGAAUCAUUUCAUUCUCCUUUUCUUAUUAUGGUGAAAUGAUU
env-1772  AGCUAACGGGCUAUUUCUCAAAAGCGGAGAU......
env-1773  GAAUAAGAAAAUUAUUCGGAAAUUUUACGAAGGGAGAACUUCU......
env-1774  AAAGAAUGGCAUAACAAUUUUACAAAAUAUCGCAGAUUUUCGCCAGAAAGAUUGUAAAUGCUCGUCUGCGAUUAGUCAAGGGUAAAAAUGAACGUGCUGACGCACGCCCUAUGACAAAUCACAGAGCGACCAUUUAAGUAUUAACUAAGCGAAAAUCAGGAUAUAAGUAACAAAGAAUUU
env-1775  CUUAACUACUUCACAAAAGCGGAGCGUGC......
env-1776  CUGAACUACUUCACAAAAGCAGAGCGGGC......
env-1778  GUCACU......
env-1779  UUGAUUUUGACAUAGGGGAGAGAAAACGAUGCUUCAAAAAUUUACAAAAUAUAUGGUACAAAGCGUUGCGGGAAUGAU......
env-1780  ACUUUUCAAAAGGAGAAACAGC......
Rsp-3-1   ......
env-1781  AUAAAAAACUUAACUUCUUUUGAAAAAGGACU......
env-1789  AUAAAAAACUUAACAUCUUUUGAAAAAGGACU......
env-1792  AUAAAGAAUUUAUCAGCAUUUGAAAGAAUAUU......
env-1794  ACGUUUUGAACUGUGUCUGUGGGGAAG......
Rsp-2-1   GAUGUACAGCUAUUUUACUUUGUUUGAAAA......
env-1796  UUAAACAGCUAUUUUACAGGAUUUGAAAA......
env-1800  UUAAACAGCUAUUUUACAGGAUUUGAAAA......
env-1801  UUAUACAGCUAUUUUACAGGAUUUGAAAA......
env-1802  UUAAACAGCUAUUUUACAGGAUUUGAAAA......
env-1803  ......
env-1804  AUAUGGCGUUACUUUCAAAAAAAGUCCUUUCCUAUUUUUCCAAGACCGAAGC......
env-1805  AUAUGGCGUUAUUUUCAAAAAAAGUCCUUUCCUAUUUUUCCA......
env-1806  CAAAAUCGGAAUGGUUUAUCUGGCUGUU......
env-1807  GAGGGCUCUCGUUUGGGCUUUACAAAAGGGGA......
env-1808  UUGGCUGGCUGAGCCGUUAUUUCUCCAAAGGGGAGCU......
env-1809  UUGGCUGGCUGAGCCGUUAUUUCUCCAAAGGGG......
env-1810  UUGGCUGGCUGAGCCGUUAUUUCUCCAAAGGGGAGCU......
env-1811  CGCUGGCUGUGCCGCUAUUUUUCCCGCGGGGAGCUGCU......
env-1812  UUCGGGCAGAUGUUUUGUGCUUUGACGCUCGGAAUAACGUUAUUUCGGUACGGCUGAAAUCAAUACUGAAUUUCGGCUUUGCAAGAAAGGAGCUAUCAUAAUGAAGAAAAAAAAUUAAAUUUUUUAAAGGCUUUUCUGUCACAGAUAUUGC......
env-1813  UU......
env-1814  UUUUAGUCAGGGUAUUUUCCAAAAGCAGAAAUUGCUGCGUGGGUCCGUUCGGUGAUCCUUAUCGCCGCAGC......
env-1815  AAUCCAUACUGCGGUAUUUUACAAAGGGAGA......
env-1816  AAAAACUAAAAUUAAUAACUGAUUACUUUACAAAAGCAGA......
env-1818  CAACAAAAAAGCAACCUCGUAUUUCUCAAAACAGGAGUGUUU......
env-1819  CAACAAAAAAGCAACCUCGUAUUUCUCAAAACAGGAGUGUUU......
env-1820  GUUUCGAGAAAUGAGAAGGAAAAGACAAUUAUUAGACUACAAUGAAUG......
env-1821  CUUUAC......
env-1822  AUUUUACAAAACAAGAAAUUAUCUUAUGGUC......
env-1823  UAUGUUUGCUUAUUUUACAAAUGGAGA......
env-1824  UCAAAUCUAUGUACCCUUCCUUUUCAAAGGGCGAGGC......
env-1825  AAUCUUUAUUUUACUACUUUACAAAAGCAGA......
env-1829  AAUCUUUAUUUUACUACUUUACAAAAGCAGA......
Dsp-1-1   CAAAAUUAUCAUUUAUUUUGCAGGUUUGAAAUU......
env-1830  CAAACUUCCCACAUUAAAAAUGGGAUUUGCUCCUUGAAAAAUCAAUACUUUAGCUCAUAAAAUAGCAAUCAGGCAGCCGCUGAACCGUACUCCAAUGCUUUUUGCAUGUACUUUGGCA......
Lba-3-1   CGAAACUCAUUAUUUGAUUAUUUUACAAAAGGAGA......
env-1831  AAUGUUUGGCUUUCAUACUUUACAAAAGGGGAAUG......
env-1832  AAAAACAAAUCGCUGCUGAAUUAUUUUACAAUGACGGA......
env-1837  AAAAACAAAUCGCUGCUGAAUUAUUUUACAAUGACGGAACUAUUAUUAUGGAGUAUAUCGGUCGCGCUUAUUACAAUGUCUUUUUUUAUUUUUGACAAAGAAAAUUAUUUGAUUUUAACCGCGUCGUUAGUAGGUGUAACUUCUUUGAUUUUCA......
env-1838  GAAAUCUAUGGUUUGUUACUUUACAAAAGGAGAAGC......
env-1839  AAGGCUAAGUCUAUGCUGAAUUAUUUUACAAAAGAGGAGCUUCUGUUAUGGUGCAUAUCGGU......
env-1840  AAAACAACAGCAUUACUGAAUUAUUUUACGAAGGGAGA......
env-1842  CAAAUUCCGCAAAAAAAUUCUAUUUUUCCCUCACUGAAUGCCUUCU......
env-1843  UCGAUCACUAAUUAUUUUACAAAAUGCGA......
env-1844  UUACGGGUUAUUUUUCAAAAGCCGAAAUAGG......
env-1845  UAAAUUAUUUUUCAAAGGGCGAAAUUUU......
env-1846  GUCCGUAUUCAUAGCUGGGGAAGGGUGGUUCU......
env-1848  ......
env-1849  AACUAUUUUUCAAGAAAUGAAAUCAUACUGUG......
env-1850  CGUAAAAUAUUUCUCCGUCGGAGAAAUCGU......
env-1851  AAUUAAA......
env-1852  UUAGAGAAAUGCGUAGAAAAAAAACAGGAAUUGAGUAAAGAAGAAUG......
env-1853  GUUUAGAGAAAUGCGUAGAAAAAAAACAGGAAUUGAGUAAAGAAGAAUG......
env-1857  GUUUAGAGAAAUGCGUAGAAAAAAAACAGGAAUUGAGUAAAGAAGAUGG......
env-1858  GUUUAGAGAAAUGCGUAGAAAAAAAACAGGAAUUGAGUAAAGAAGAAUG......
env-1859  GUUUAGAGAAAUGCGUAGAAAAAAAACAGGAAUUGAGUAAAGAAGAAUG......
env-1860  AAGAGGAGAAAUUGCAAUUUGGACUGUAUCAGU......
env-1861  AAAAACAAAUCAUUCAUCAAUUAUUUUACAAAAGCGGA......
env-1862  UCAAGUCAGCGAUCCGGUAUUUUACCAGAGCCGAAAU......
env-1864  ......
env-1865  AUUUUUCCCGCACAGAUAUACUUCUCUGGAGUUUUUCUGUCUGCAUGAUAGUUAUUUCAGCUCUUUGCUUCGGCGGAGGAAGCAUACUCGCCGU......
env-1866  AUUUUUCCCGCACAGAUAUCCUUCUCUGGAG......
env-1869  AUUUUUCCCGCACAGAUAUACUUCUCUGGAG......

          ......
          ......
          ......
          ......
          ●●●●●●●●●●●●○○○○○○○○○○○○○○○○○○○○○○○○○○○○------------------------------------
```

```
env-1870    ACGGAGUAUCGCUUCUUUUUCCCUCGGUGAACG...
env-1871    ...
Ssu-6-1     UUAUUGAUUCAUCUUUUUGGAAUUUAUUUCCAGAUGC...
Ssu-1-3     AGUUGAUACAUCGUUUUGGAAUUUAUUUCCAGAUGC...
Ssu-1-4     GUUGUUGAUUCAUCUUUUUGGAGUCUAUUUCCAGAUGC...
Ssu-1-7     GUUGUUGAUUCAUCUUUUUGGAGUCUAUUUCCAGAUGC...
Ssu-1-8     GUUGUUGAUUCAUCUUUUUGGAGUCUAUUUCCAGAUGCAAAAAUUGGAGUUCUAUUAUUGAAAGAUUAUAAAACGCCGGCGCAAUCACC...
env-1872    ...
env-1873    AAAAAAGCGACGCCAUUGCAUGAAUUUAUCAAGUACUCGACCCUGAG...
env-1875    UUUUAAG...
env-1876    UUCACUCUAUGGAAGAAUCAAAAAACGAUUCUUCAUCAUUUUUUGUGACGCCGUGAAACGGCGUAAUGGAGGAAACUAUGGAACAAGUAUGUAUUUUAAAAACUUUUAGAAAAUAUGUAACAUUAAA...
env-1877    UUAAAAACUUUUAGAAAAUAUGUAACAUUAAA...
env-1878    GGCCUAAUAUUGGAUACGCAGUCUGCUGGCUCCAAAAUAGGUUUACACAUAGAGGGGGUUCCAUAUGAGGAAUACGUCUUGUUUUAGGGAUUUUGUUCGGUAUUCUUCUCUGAA...
env-1879    AUGAGAAGAAAACGGCAGAAACUGUCAAAAGAGGAAUG...
env-1880    AUGAGGAGAAAACGCCAGAAACUGUCAAAAGAGGAAUGUGAAGAGAUCCU...
env-1881    GGAGAAAAAGACAGAGAUUACAGGAGGAUUUCAGAAAAUGUUCGGAGAAAUGAGAAGAAAAAAACAGGCUCUUCCUUUGGAGGCCUG...
env-1882    UGCAAGAAAAAUCAUGUUUUGGCAUGUUUAUAAAAUAUGCCUCCCUGAA...
env-1884    GAGGCGUAAAAAACAAGAACUAUCGCAACAAGA...
env-1885    AGAGGAUAUUUAUGGGAGUACUUUUAGCAGCGGUGUUUACGACGAGCUUUUUUGACAGCCU...
env-1886    GAGAAGGAAAAAACAGGCCCUACCGCAACGGGA...
env-1887    GAAAAACAAAAAAUCAAUU...
env-1888    GAAAAACAAAAAAUCAAUUGAAUAAACUAUAAAUAAAAUCAGCCCGCCGGGGUUUUGUUUUAUUGUGUAGUAGGCCAGCGUUACACAAACGCUGGCCUUUUUGAAUGAAAAGGAGAUGAUACAUACUAUGUGGAUACUUUUGGUAACAGUCAUAACAACAAGUUUUUUUGAUAGCUU...
env-1889    GAGAAGGAAAAAACAGGCAUUAUCGCAACAAGAGGUUGUGGAUAUUCU...
env-1890    GAGAAGGAAAAAACAGGCAUUAUCGCAACAAGA...
env-1891    GAUGAAUAAGAAAUGAAGUGAUUGAAUUUGUAAAAAGAAGCGACAAGGAUAGUAAUUGAAUAUGAAGAUAAAAUCAGCCCGCCGGGGUUCAAUUUUAUUGUGUAGUAGGCCAGCGUUACACAUACGCUGGCCUUUUUGAUUGAUAGGGAGAUGAUACUAUGUGGAUACUUUUAGGAACAG
env-1892    GAGCCCGUGCCGAAACAGGGGACGCAGAACGGUAAGCAGCGGAAAGCGAACGGGGCAGCAUAGCGGAAGUUAUGGACGGACAGGAUGAAGUUCAGGAGCAUAUAUCGUAUAAACAUAGGAAAAGGAGACCGAAAGGCGUGAAAAAUUCAGAGGAUCUGCGCGCAUAUGCGAAGUAUACAG
env-1893    UAAAUACGUUUCACUCAAUGUGCUGGGAAUGAU...
env-1894    ...
env-1895    GUAAAUACGUUUCACUCCAAUGUGCUGGGAAUGAUUGCCUUAUCCUGUUAUAUAUUGGCUGAUACCUUCUUUGUAUCCCA...
env-1896    AUCAUUAAAAAAUCUGAC...
env-1897    ...
env-1898    UUUACGCUUAUACAAAAACAAACAAAUAACUAACCAAGGAGAAUUCAACGUGAAAAAGAUUUUAAACUACUUUUCAAAAGGCGAAAUAAUAUUGUGGAG...
env-1899    AUGCGCAGAAAAAGGCAGGCGUUAUCCGCAGAAGAAAC...
env-1900    AAGCAAAUAUUACUAUUUAAGGA...
env-1901    CGAGAGAUGAGAAGAAAAAAUCAGGCGCUGACCAAAGAGGAGUG...
env-1902    ACGGGUGAGGAGGAAUGAGCAUGAAAAUUGAAAAACACGGCUACUUUACCAAAAACGAACUGCGGCUGUG...
env-1903    AAUCUUGUUUUUGGGAAUUUUCGCACUAUACGAUACUGAG...
env-1904    AUUUAAGGAAUUCGCCCGCUACUCUACAUUAAA...
Par-1-1     UAUUUAGUUGUUGCAGGAUGUUUUGAAGUUGUUUGGGC...
env-1905    UCAGCGUGUUCGAAUGGAUAAUGUGGUG...
env-1906    UCAGCGUGUUCGAAUGGAUAAUGUGGUG...
env-1907    AGAGUUACCUAAAAUAUACGACCCUUAAUAUUCU...
Efa-293-1   GCUUGGUUAAAAGUUAUUUUUGGCGCUUUUUGUGAAGUGAU...
Efa-66-1    GCUUGGUUAAAAGUUAUUUUUGGCGCUUUUUGUGAAGUGAU...
Eca-2-1     CAUUACUUGAAGUGAAAGAGUGCAGGAUGAAACAAAGCGAAACGUUGUUUUCUAUAUACUAAGGAGGUUUUUACAUGAACAAGGAUUGGAUCAAAUUAAUGAUUGGUGCUUUUUUCGAGGUGCU...
Efa-286-1   GCUUGGUUAAAAGUUAUUUUUGGCGCUUUUUGUGAAGUGAU...
Efa-202-1   GCUUGGUUAAAAGUUAUUUUUGGCGCUUUUUGUGAAGUGAU...
Lli-2-1     AUUUAAUAAUUGCAGGAAUAUUUGAGGUUGUGUGGGC...
            ...
            ...
            ...
            ...
            ●●●●●●●●●●●●●○○○○○○○○○○○○○○○○○○○○○○○○--------------------------------
```

alignment positions 440 · · · 531

```
env-1       ...
Csp-12-1    ...
env-2       ...
env-4       GGCGCUUUAAAAAUGGUCCGGAAGCGUGAUACGGAAGUGUGAGCAACACUCAAAAACACAUUGAAAAUCCUACAUUUUUACGGCAUAGACGC
Lga-2-1     ...
env-5       ...
env-6       ...
env-7       ...
Lga-4-1     ...
env-12      ...
env-13      ...
env-14      ...
env-15      ...
[sc-1-1     ...
env-17      ...
env-21      ...
env-22      ...
env-23      ...
env-24      ...
env-25      ...
env-26      ...
Lbr-1-1     ...
Lsa-3-1     ...
            ...
            ...
            ...
            ...
            --------------------------------------------------------------------------------------------
```

```
env-28     ............................................................................................
env-29     ............................................................................................
Wbo-1-1    ............................................................................................
Wjo-1-1    ............................................................................................
env-30     ............................................................................................
Lag-1-1    ............................................................................................
env-31     ............................................................................................
Lpl-1-1    ............................................................................................
Lcu-1-1    ............................................................................................
Lki-1-1    ............................................................................................
env-32     ............................................................................................
Csp-20-1   ............................................................................................
env-33     ............................................................................................
env-34     ............................................................................................
env-35     ............................................................................................
env-36     AUUGGUGCAGUUGGGUCGAUUCUAGUUGGUUUGAUCCUGUUUCACGAUCAAAUUAGCCCGUUGACUUGGUUGUUUAUUGCUCUUCUAAUCAU
env-37     ............................................................................................
env-38     ............................................................................................
env-39     ............................................................................................
Esp-14-1   ............................................................................................
Esp-15-1   ............................................................................................
env-40     ............................................................................................
env-41     AUAGGUCUAUUAAAAUGGGUCGCCCAAGAGUUUAUGACACUCCGCACUUCUGCCUCCAUACUUA............................
env-42     ............................................................................................
env-43     ............................................................................................
env-44     ............................................................................................
env-45     ............................................................................................
env-46     ............................................................................................
env-47     ............................................................................................
env-48     ............................................................................................
env-72     UGGCGCCUU...................................................................................
env-73     AUAGGUCUAUUAAAAUGGGUCGCCCAAGAGUUUAUGACACUCCGCACUUCUGCCUCCAUACUUA............................
env-74     ............................................................................................
env-75     ............................................................................................
env-76     GUAGGUCUAUUAAAAUGGGUCGCCCUAGAAUUUACGACACUCCGCACUUCUGCCUCCAUACUUA............................
env-77     ............................................................................................
env-78     AUAGGUCUAUUAAAAUGGGUCGCCCAAGAGUUUAUGACACUCCGCACUUCUGCCUCCAUACUUA............................
env-79     ............................................................................................
env-80     ............................................................................................
env-104    ............................................................................................
env-110    ............................................................................................
env-111    ............................................................................................
env-112    ............................................................................................
env-113    ............................................................................................
env-114    ............................................................................................
Rpe-1-1    ............................................................................................
env-116    ............................................................................................
Rsp-1-1    ............................................................................................
Lsp-2-1    ............................................................................................
Lse-1-1    ............................................................................................
env-117    ............................................................................................
env-118    ............................................................................................
env-119    ............................................................................................
Loz-1-1    ............................................................................................
env-120    ............................................................................................
env-121    ............................................................................................
env-122    AA..........................................................................................
Bpu-1-1    ............................................................................................
env-123    ............................................................................................
env-129    ............................................................................................
env-131    ............................................................................................
env-132    UUCUUGGGUAAAACAGACUAAUGUCUAUUAAUUUACCAAGCUAUUGCGUCUGUUCCAUACGCUUUGGUUACAUAUUUGAUUA..........
Ali-1-1    ............................................................................................
env-133    ............................................................................................
env-134    ............................................................................................
Lin-1-1    ............................................................................................
Ljo-2-1    ............................................................................................
Ljo-1-1    ............................................................................................
env-135    ............................................................................................
env-136    ............................................................................................
env-138    ............................................................................................
Fpl-1-1    ............................................................................................
Fsp-1-1    ............................................................................................
env-140    CGGCGGAGCCACCCGCUACUCCAUCUGCCGGUAUCAAGGGGAUGAGGCCAGGGCCAACCGCACCUUUACCCUGUCCCUGCUCACCGGGCUGG
env-141    ............................................................................................
env-143    ............................................................................................
Lla-20-1   ............................................................................................
Lla-4-1    ............................................................................................
Lla-21-1   ............................................................................................
env-144    ............................................................................................
env-145    ............................................................................................
env-146    ............................................................................................

           ............................................................................................
           ............................................................................................
           ............................................................................................
           ............................................................................................
           --------------------------------------------------------------------------------------------
```

```
env-150    ............................................................................................
Lla-1-12   ............................................................................................
env-151    ............................................................................................
env-152    ............................................................................................
env-153    ............................................................................................
env-154    ............................................................................................
env-155    ............................................................................................
env-157    ............................................................................................
env-158    ............................................................................................
env-159    ............................................................................................
env-160    ............................................................................................
env-161    ............................................................................................
env-164    ............................................................................................
env-165    ............................................................................................
env-166    ............................................................................................
env-167    ............................................................................................
env-168    ............................................................................................
env-169    CUGGACCGGCAUCGGGGCCGUAGGCUCCGUGGCGGUGGGCAUCCUGAUCUUCAAGGAGCCGGUGACCGCCGCCCGGAUGUUUUUCACCGUGU
env-170    ............................................................................................
env-172    ............................................................................................
env-173    ............................................................................................
env-231    ............................................................................................
env-232    ............................................................................................
env-233    ............................................................................................
env-234    ............................................................................................
env-235    ............................................................................................
env-239    ............................................................................................
env-240    ............................................................................................
env-242    ............................................................................................
env-243    UGGGCACUGCCUACGCCGUCUGGACCGGCAUCGGGGCCGUAGGCUCCGUGGCGGUGGGCAUCCUGAUCUUCAAGGAGCCGGUGACCGCCGCC
env-244    ............................................................................................
env-245    ............................................................................................
env-246    ............................................................................................
env-247    ............................................................................................
env-248    ............................................................................................
env-249    ............................................................................................
env-250    ............................................................................................
env-251    ............................................................................................
env-254    ............................................................................................
env-255    ............................................................................................
Efa-269-1  ............................................................................................
Efa-159-1  ............................................................................................
env-256    ............................................................................................
env-257    ............................................................................................
env-258    ............................................................................................
env-259    ............................................................................................
env-260    ............................................................................................
env-261    ............................................................................................
env-262    ............................................................................................
env-270    ............................................................................................
env-273    ............................................................................................
env-274    ............................................................................................
env-287    ............................................................................................
env-294    ............................................................................................
env-295    ............................................................................................
env-308    ............................................................................................
env-309    ............................................................................................
env-310    ............................................................................................
env-311    ............................................................................................
env-312    ............................................................................................
env-313    ............................................................................................
env-314    ............................................................................................
env-315    CCAGUCGGUACAGCCUA...........................................................................
Efa-211-1  ............................................................................................
Efa-256-1  ............................................................................................
Ete-1-1    ............................................................................................
Ime-1-1    ............................................................................................
Esp-4-1    ............................................................................................
Eur-2-1    ............................................................................................
Ewa-1-1    ............................................................................................
Esi-1-1    ............................................................................................
Eha-2-1    ............................................................................................
Epl-1-1    ............................................................................................
Emo-2-1    ............................................................................................
Eur-1-1    UAAAAUUGAUGAUUGGUGCUUUUUUUGAAGUAUU..........................................................
env-316    ............................................................................................
Equ-1-1    ............................................................................................
Eri-1-1    ............................................................................................
Csp-19-1   ............................................................................................
env-317    ............................................................................................
env-318    ............................................................................................
env-322    ............................................................................................

           ............................................................................................
           ............................................................................................
           ............................................................................................
           ............................................................................................
           --------------------------------------------------------------------------------------------
```

```
env-323    ..........................................................................................................
env-325    ..........................................................................................................
env-327    ..........................................................................................................
env-330    ..........................................................................................................
Cje-1-1    ..........................................................................................................
env-331    ..........................................................................................................
env-332    ..........................................................................................................
env-333    ..........................................................................................................
env-335    ..........................................................................................................
Lsa-2-1    ..........................................................................................................
env-336    ..........................................................................................................
env-337    ..........................................................................................................
env-339    ..........................................................................................................
env-340    ..........................................................................................................
env-341    ..........................................................................................................
env-342    ..........................................................................................................
env-343    ..........................................................................................................
env-344    ..........................................................................................................
env-347    ..........................................................................................................
env-348    ..........................................................................................................
env-349    ..........................................................................................................
env-350    ..........................................................................................................
env-351    ..........................................................................................................
env-352    ..........................................................................................................
env-353    ..........................................................................................................
env-354    ..........................................................................................................
env-357    ..........................................................................................................
env-358    ..........................................................................................................
env-359    ..........................................................................................................
env-360    ..........................................................................................................
env-362    ..........................................................................................................
env-363    ..........................................................................................................
env-368    ..........................................................................................................
env-372    ..........................................................................................................
env-373    ..........................................................................................................
env-374    ..........................................................................................................
env-375    ..........................................................................................................
env-376    ..........................................................................................................
Fnu-1-1    ..........................................................................................................
env-387    ..........................................................................................................
env-388    ..........................................................................................................
env-389    AGAAGUUCUAUUGAGGCAC.......................................................................................
env-390    ..........................................................................................................
env-391    ..........................................................................................................
env-396    ..........................................................................................................
env-408    ..........................................................................................................
env-409    ..........................................................................................................
env-410    GGCUUCGGCAUCAUCGGCACGAGCCUGCUCGGCGUCUGGCU.................................................................
env-411    ..........................................................................................................
env-412    ..........................................................................................................
env-413    GGCUUCGGCAUCAUCGGCACGAGCCUGCUCGGCGUCUGGCU.................................................................
env-414    ..........................................................................................................
Lsp-7-1    ..........................................................................................................
env-415    ..........................................................................................................
env-416    ..........................................................................................................
env-417    ..........................................................................................................
env-418    ..........................................................................................................
env-420    ..........................................................................................................
env-421    ..........................................................................................................
Bca-1-1    ..........................................................................................................
env-422    ..........................................................................................................
env-423    ..........................................................................................................
env-426    ..........................................................................................................
env-428    ..........................................................................................................
env-429    ..........................................................................................................
env-430    ..........................................................................................................
env-431    ..........................................................................................................
env-432    ..........................................................................................................
env-433    ..........................................................................................................
Bsp-1-1    ..........................................................................................................
Lpe-1-1    ..........................................................................................................
Lfu-1-1    ..........................................................................................................
Lde-1-1    ..........................................................................................................
Lfa-3-1    ..........................................................................................................
env-434    ..........................................................................................................
env-436    ..........................................................................................................
env-437    ..........................................................................................................
env-439    ..........................................................................................................
Esp-11-1   ..........................................................................................................
env-440    ..........................................................................................................
Lsp-1-1    ..........................................................................................................
Lsp-5-1    ..........................................................................................................

           ..........................................................................................................
           ..........................................................................................................
           ..........................................................................................................
           ..........................................................................................................
           ----------------------------------------------------------------------------------------------------------
```

```
Pac-2-1     ............................................................................................
Eth-1-1     ............................................................................................
Efa-295-1   ............................................................................................
Esp-3-1     ............................................................................................
Efa-294-1   ............................................................................................
env-441     ............................................................................................
Ega-2-1     ............................................................................................
env-442     AGACUUUUAUAAAAAUCGAAUCACUUCUUUCUACCGAAAUCUUUCUUAUUUCUACUUUUGCUUUCCUCUAGGAUAUCAUCAGACUACUGAUC
env-443     ............................................................................................
Ega-1-2     ............................................................................................
Ega-1-3     ............................................................................................
env-444     ............................................................................................
Lta-1-1     ............................................................................................
Lcr-1-1     ............................................................................................
Lul-1-2     ............................................................................................
env-448     ............................................................................................
env-449     ............................................................................................
env-450     ............................................................................................
env-452     ............................................................................................
env-454     ............................................................................................
env-456     ............................................................................................
env-457     ............................................................................................
env-459     ............................................................................................
1 env-460   ............................................................................................
Rti-1-1     ............................................................................................
Rma-1-1     ............................................................................................
env-461     ............................................................................................
env-462     ............................................................................................
env-463     ............................................................................................
env-464     ............................................................................................
env-465     ............................................................................................
env-466     ............................................................................................
env-467     ............................................................................................
env-468     GGCAUUGGCGCGCUGGGCUCCGUGAUCGUUGGCAUCAUCUUGUUCAAAGAGCCGGUGACGGUUGUGCGCCUGAUCUUUGCGGCACUGCUGCU
env-469     ............................................................................................
env-470     ............................................................................................
env-471     ............................................................................................
env-472     ............................................................................................
env-473     ............................................................................................
env-476     ............................................................................................
env-477     ............................................................................................
env-478     ............................................................................................
env-479     ............................................................................................
env-480     ............................................................................................
env-481     ............................................................................................
env-482     ............................................................................................
env-483     ............................................................................................
env-484     ............................................................................................
env-485     ............................................................................................
env-486     ............................................................................................
env-487     ............................................................................................
env-488     ............................................................................................
env-492     ............................................................................................
env-493     ............................................................................................
env-494     ............................................................................................
env-495     ............................................................................................
env-496     ............................................................................................
Lgi-1-1     ............................................................................................
Lve-2-1     ............................................................................................
env-497     ............................................................................................
env-498     ............................................................................................
env-500     ............................................................................................
Lna-2-1     ............................................................................................
env-501     ............................................................................................
env-502     ............................................................................................
env-503     ............................................................................................
env-504     ............................................................................................
env-505     ............................................................................................
Lno-1-1     ............................................................................................
Lal-2-1     ............................................................................................
env-506     ............................................................................................
Lal-1-1     ............................................................................................
Whe-1-1     ............................................................................................
Whe-1-2     ............................................................................................
Lpa-1-1     ............................................................................................
Lpa-2-1     ............................................................................................
Lra-1-1     ............................................................................................
Lki-3-1     ............................................................................................
Lki-2-1     ............................................................................................
Ldi-1-1     ............................................................................................
Lhe-1-1     ............................................................................................
Lfa-2-1     ............................................................................................

            ............................................................................................
            ............................................................................................
            ............................................................................................
            ............................................................................................
            --------------------------------------------------------------------------------------------
```

```
env-508    ...........................................................................
Nma-1-1    ...........................................................................
env-509    ...........................................................................
env-511    ...........................................................................
env-512    ...........................................................................
env-514    ...........................................................................
Csp-12-2   ...........................................................................
Csa-1-1    ...........................................................................
env-515    ...........................................................................
env-516    ...........................................................................
env-517    ...........................................................................
env-518    ...........................................................................
env-519    ...........................................................................
env-520    ...........................................................................
Bsp-1-2    ...........................................................................
env-521    ...........................................................................
env-522    ...........................................................................
env-523    ...........................................................................
env-524    ...........................................................................
env-525    ...........................................................................
Msp-5-1    ...........................................................................
Lsa-1-1    ...........................................................................
Lcu-2-1    ...........................................................................
env-526    ...........................................................................
env-527    ...........................................................................
env-528    ...........................................................................
env-529    ...........................................................................
env-530    ...........................................................................
env-531    ...........................................................................
env-533    ...........................................................................
Csp-6-1    ...........................................................................
env-534    ...........................................................................
Cba-3-1    ...........................................................................
env-535    ...........................................................................
env-536    ...........................................................................
env-537    ...........................................................................
Csp-11-1   ...........................................................................
env-538    ...........................................................................
env-545    ...........................................................................
env-546    ...........................................................................
Rma-2-1    ...........................................................................
env-547    ...........................................................................
env-548    ...........................................................................
Csp-7-1    ...........................................................................
env-549    ...........................................................................
env-550    ...........................................................................
env-552    ...........................................................................
env-553    ...........................................................................
env-557    ...........................................................................
env-558    ...........................................................................
env-559    ...........................................................................
env-560    ...........................................................................
env-561    ...........................................................................
env-562    ...........................................................................
Cin-1-1    ...........................................................................
env-563    ...........................................................................
env-564    ...........................................................................
env-565    ...........................................................................
env-566    ...........................................................................
Csp-14-1   ...........................................................................
env-570    ...........................................................................
env-573    ...........................................................................
env-574    ...........................................................................
env-575    ...........................................................................
env-576    ...........................................................................
env-577    ...........................................................................
env-578    ...........................................................................
env-580    ...........................................................................
Lna-1-1    ...........................................................................
env-587    ...........................................................................
env-588    ...........................................................................
env-589    ...........................................................................
env-591    ...........................................................................
env-592    ...........................................................................
env-593    ...........................................................................
env-594    ...........................................................................
env-595    ...........................................................................
env-598    ...........................................................................
Csp-3-1    ...........................................................................
[gu-1-1    ...........................................................................
env-599    ...........................................................................
[in-1-1    ...........................................................................

           ...........................................................................
           ...........................................................................
           ...........................................................................
           ...........................................................................
           ---------------------------------------------------------------------------
```

```
Air-1-1   ............................................................................................
Csp-5-1   ............................................................................................
env-600   ............................................................................................
env-601   ............................................................................................
env-605   ............................................................................................
env-608   AAUCAGGCAUAAAGAGCUAAAAGGAAAAACACUAUUCAGAACUAAUACUCCGGUCUGAUUAUUAACUUUGAGCAAAGUAAAGCCUACCCUCG
Dph-1-1   ............................................................................................
env-609   ............................................................................................
env-610   ............................................................................................
env-611   ............................................................................................
Mha-1-1   ............................................................................................
env-612   ............................................................................................
env-618   ............................................................................................
env-619   ............................................................................................
env-621   ............................................................................................
env-623   ............................................................................................
env-625   ............................................................................................
Cin-2-1   ............................................................................................
env-626   ............................................................................................
env-627   ............................................................................................
env-628   ............................................................................................
env-629   ............................................................................................
env-631   ............................................................................................
env-632   ............................................................................................
Til-1-1   ............................................................................................
env-633   UUUCGGAUCUACCCCAAUCAGGAACA..................................................................
env-634   UUUCGGAUCUACCCCAAUCAGGAACA..................................................................
env-635   UUUCGGAUCUACCCCAAUCAGGAACA..................................................................
env-636   ............................................................................................
env-637   ............................................................................................
env-638   ............................................................................................
env-640   ............................................................................................
env-641   ............................................................................................
env-644   ............................................................................................
env-657   ............................................................................................
env-658   ............................................................................................
env-659   ACAAAAAUUUACAAGAUACAAAGUCUUGAGGAUGAAAAAAUUAAU...............................................
Tma-1-1   ............................................................................................
Nfe-1-1   ............................................................................................
Cfo-1-1   ............................................................................................
Npe-1-1   ............................................................................................
Gfe-1-1   ............................................................................................
Psp-1-1   ............................................................................................
env-660   ............................................................................................
env-663   ............................................................................................
env-664   ............................................................................................
Cba-1-1   ............................................................................................
Cba-6-1   ............................................................................................
env-665   ............................................................................................
env-667   ............................................................................................
env-668   ............................................................................................
env-669   ............................................................................................
env-670   ............................................................................................
Csy-2-1   ............................................................................................
env-672   ............................................................................................
env-674   ............................................................................................
env-675   ............................................................................................
env-679   ............................................................................................
env-680   ............................................................................................
env-683   ............................................................................................
env-684   ............................................................................................
env-689   ............................................................................................
env-690   ............................................................................................
env-691   ............................................................................................
env-692   ............................................................................................
env-693   ............................................................................................
env-694   ............................................................................................
env-695   ............................................................................................
[ae-1-1   ............................................................................................
env-696   ............................................................................................
env-697   ............................................................................................
env-698   ............................................................................................
[am-2-1   ............................................................................................
env-699   ............................................................................................
env-700   ............................................................................................
env-701   ............................................................................................
env-702   ............................................................................................
env-703   ............................................................................................
env-704   ............................................................................................
env-705   ............................................................................................
env-707   ............................................................................................
env-708   ............................................................................................

          ............................................................................................
          ............................................................................................
          ............................................................................................
          ............................................................................................
          --------------------------------------------------------------------------------------------
```

```
env-709    ............................................................................................
env-710    ............................................................................................
env-713    ............................................................................................
env-714    ............................................................................................
env-716    ............................................................................................
env-717    ............................................................................................
Bpi-1-1    ............................................................................................
env-718    ............................................................................................
env-719    ACAAAAUACAAAAUCGGACUGAUUUUUCAGUCCGACCUUAAUUUAAUAAAAAUGCUAUUCUAUUCGCUGAUGCUCCAACCGGGCCAAAAGGU
env-721    ............................................................................................
Exy-1-1    ............................................................................................
env-722    ............................................................................................
env-723    ............................................................................................
Sru-1-1    ............................................................................................
env-724    ............................................................................................
env-725    ............................................................................................
env-726    ............................................................................................
env-727    ............................................................................................
env-728    ............................................................................................
Lga-5-1    ............................................................................................
env-729    ............................................................................................
env-730    ............................................................................................
env-731    ............................................................................................
Esp-13-1   ............................................................................................
env-732    ............................................................................................
env-734    ............................................................................................
env-735    ............................................................................................
env-736    ............................................................................................
env-737    ............................................................................................
env-738    ............................................................................................
env-740    ............................................................................................
env-741    ............................................................................................
Msp-2-1    ............................................................................................
env-742    ............................................................................................
Mti-1-1    ............................................................................................
env-743    ............................................................................................
env-744    GAAUCAGUGAAACGAAUGGAUAGGAAGAAAAAAUCAUGCGGAAAAGAAUAGGAAAAAAACGAUCCCUGCUGGUGCGGCAGUGGAAA......
env-745    ............................................................................................
env-746    ............................................................................................
env-747    ............................................................................................
env-748    ............................................................................................
Bma-1-1    ............................................................................................
env-751    ............................................................................................
env-753    ............................................................................................
env-754    ............................................................................................
env-755    ............................................................................................
Lga-1-1    ............................................................................................
Lsp-4-1    ............................................................................................
env-757    ............................................................................................
env-758    ............................................................................................
env-759    ............................................................................................
env-760    UGUGAACAAACGAAUCUUCCUGCGCUAUGU..............................................................
env-761    ............................................................................................
env-762    ............................................................................................
env-763    ............................................................................................
env-764    ............................................................................................
env-765    ............................................................................................
env-768    ............................................................................................
env-769    ............................................................................................
Lsp-9-1    ............................................................................................
env-770    ............................................................................................
Lsp-10-1   ............................................................................................
Csp-15-1   ............................................................................................
env-771    ............................................................................................
env-773    ............................................................................................
env-774    ............................................................................................
env-775    ............................................................................................
env-776    ............................................................................................
env-777    ............................................................................................
env-778    ............................................................................................
env-780    ............................................................................................
env-781    ............................................................................................
env-782    ............................................................................................
env-784    ............................................................................................
env-785    ............................................................................................
env-786    ............................................................................................
env-787    ............................................................................................
env-788    ............................................................................................
env-789    ............................................................................................
env-790    ............................................................................................
env-791    ............................................................................................
env-792    ............................................................................................

           ............................................................................................
           ............................................................................................
           ............................................................................................
           ............................................................................................
           --------------------------------------------------------------------------------------------
```

```
env-793   ..........................................................................................................
env-794   ..........................................................................................................
env-795   ..........................................................................................................
env-798   ..........................................................................................................
env-799   ..........................................................................................................
env-800   ..........................................................................................................
env-801   ..........................................................................................................
env-802   ..........................................................................................................
env-803   ..........................................................................................................
env-804   ..........................................................................................................
env-805   ..........................................................................................................
env-806   ..........................................................................................................
env-807   ..........................................................................................................
env-811   ..........................................................................................................
env-824   ..........................................................................................................
env-827   ..........................................................................................................
env-828   ..........................................................................................................
env-829   ..........................................................................................................
env-830   ..........................................................................................................
env-848   ..........................................................................................................
env-849   ..........................................................................................................
env-850   ..........................................................................................................
env-851   ..........................................................................................................
env-855   ..........................................................................................................
env-856   ..........................................................................................................
env-858   ..........................................................................................................
env-860   ..........................................................................................................
env-861   ..........................................................................................................
env-862   ..........................................................................................................
env-863   ..........................................................................................................
env-864   ..........................................................................................................
env-865   ..........................................................................................................
env-866   ..........................................................................................................
env-868   ..........................................................................................................
env-869   ..........................................................................................................
env-871   ..........................................................................................................
env-872   ..........................................................................................................
Fsp-3-1   ..........................................................................................................
env-878   ..........................................................................................................
env-880   ..........................................................................................................
env-881   ..........................................................................................................
env-882   ..........................................................................................................
env-883   ..........................................................................................................
env-884   ..........................................................................................................
env-885   ..........................................................................................................
env-886   ..........................................................................................................
env-888   ..........................................................................................................
env-889   ..........................................................................................................
env-891   ..........................................................................................................
env-892   ..........................................................................................................
env-893   ..........................................................................................................
env-895   ..........................................................................................................
env-896   ..........................................................................................................
env-897   ..........................................................................................................
env-898   ..........................................................................................................
env-899   ..........................................................................................................
env-900   ..........................................................................................................
env-901   ..........................................................................................................
env-902   ..........................................................................................................
env-905   ..........................................................................................................
env-906   ..........................................................................................................
env-907   ..........................................................................................................
env-908   ..........................................................................................................
Cbo-2-1   ..........................................................................................................
env-910   ..........................................................................................................
env-911   ..........................................................................................................
env-912   ..........................................................................................................
env-913   ..........................................................................................................
env-914   ..........................................................................................................
env-915   ..........................................................................................................
env-916   ..........................................................................................................
env-917   ..........................................................................................................
env-918   ..........................................................................................................
env-922   ..........................................................................................................
env-923   ..........................................................................................................
env-927   ..........................................................................................................
env-928   ..........................................................................................................
env-929   ..........................................................................................................
env-930   ..........................................................................................................
env-933   ..........................................................................................................
Pvi-1-1   ..........................................................................................................
env-934   ..........................................................................................................

          ..........................................................................................................
          ..........................................................................................................
          ..........................................................................................................
          ..........................................................................................................
          ----------------------------------------------------------------------------------------------------------
```

```
env-935     ............................................................................................
env-936     ............................................................................................
env-937     ............................................................................................
env-938     ............................................................................................
env-939     ............................................................................................
bMS-1-1     ............................................................................................
env-940     ............................................................................................
env-941     ............................................................................................
Epl-2-1     ............................................................................................
env-942     ............................................................................................
env-944     ............................................................................................
env-945     ............................................................................................
env-946     ............................................................................................
env-947     ............................................................................................
env-948     ............................................................................................
env-949     ............................................................................................
env-950     ............................................................................................
env-951     ............................................................................................
env-952     ............................................................................................
env-953     ............................................................................................
env-954     ............................................................................................
env-955     ............................................................................................
env-957     ............................................................................................
env-958     ............................................................................................
env-960     ............................................................................................
env-961     ............................................................................................
env-963     ............................................................................................
env-965     ............................................................................................
env-966     ............................................................................................
env-968     ............................................................................................
env-969     ............................................................................................
env-970     ............................................................................................
env-971     ............................................................................................
env-972     ............................................................................................
env-973     ............................................................................................
env-974     ............................................................................................
env-976     ............................................................................................
env-977     ............................................................................................
env-978     ............................................................................................
env-979     ............................................................................................
env-980     UUUUCCACAGGC................................................................................
env-981     ............................................................................................
env-982     ............................................................................................
env-983     ............................................................................................
env-984     ............................................................................................
env-985     ............................................................................................
env-986     ............................................................................................
Cba-2-1     UAUCGGUAGAAGAAUG............................................................................
env-987     ............................................................................................
env-988     ............................................................................................
env-989     ............................................................................................
env-990     ............................................................................................
env-991     ............................................................................................
env-994     UGGGCGGAGCCACAAAGUUUUGCGUUUGCAAAAGUCAGCA....................................................
env-995     ............................................................................................
env-997     ............................................................................................
env-998     ............................................................................................
env-999     ............................................................................................
env-1001    ............................................................................................
env-1012    ............................................................................................
env-1015    ............................................................................................
env-1016    ............................................................................................
env-1017    AAAAUUGCAGAAAGAAGCGCACUGCUUGUCAUUGACAUUCAGCAGGA.............................................
Lba-2-1     ............................................................................................
env-1022    ............................................................................................
env-1023    UGGCUGGGAUCGUUGCUCGUGGUCAUAUGUUCCAAUAUUGGGGAGAAAUGAUCACCUAUCUGGGAAUGACUAUGCCGAUGGCAAUCUGGUCA
env-1024    ............................................................................................
env-1025    UCAGCACAGAUGAAGC............................................................................
env-1026    UCAGCACAGAUGAAGC............................................................................
env-1040    ............................................................................................
env-1045    ............................................................................................
env-1046    ............................................................................................
env-1047    ............................................................................................
env-1048    UAUAAAG.....................................................................................
env-1049    UCAGCACAGAUGAAGC............................................................................
env-1052    GAUUAUCUUAUGAAACAACACAAUCUGUUAUCCAUCAAAAAUGUCGUCUUAAUGGGCAU.................................
env-1053    UACACCGUUUUCCAUCACACGCGUUG..................................................................
env-1054    ............................................................................................
env-1055    ............................................................................................
env-1056    ............................................................................................
env-1057    ............................................................................................
env-1058    ............................................................................................

            ............................................................................................
            ............................................................................................
            ............................................................................................
            ............................................................................................
            --------------------------------------------------------------------------------------------
```

```
env-1059  ............................................................................................
env-1060  ............................................................................................
env-1061  ............................................................................................
env-1062  ............................................................................................
env-1070  ............................................................................................
env-1071  ............................................................................................
env-1072  ............................................................................................
env-1073  ............................................................................................
env-1074  AAGAAUUCUCCUUCCUCUACAGGUGGGAGAUGAAUUGCAGAAAAAGAAAAACAGAACACUUGUUCUAUAAUGCGAAGUGUGUUAUACUAUAA
env-1075  ............................................................................................
env-1076  GAGAUUCAGCAAAUGACAUUGUACUGAUUGUGCUCUGGAUUAUGGCAACCAUAAAAGAUCCAUCCUAUUUUUCCGUAAUGAU..........
env-1077  ............................................................................................
env-1078  ............................................................................................
env-1079  ............................................................................................
env-1080  ............................................................................................
env-1081  ............................................................................................
env-1082  ............................................................................................
env-1083  ............................................................................................
env-1084  ............................................................................................
Ano-1-1   ............................................................................................
env-1085  ............................................................................................
env-1086  ............................................................................................
env-1087  ............................................................................................
env-1088  ............................................................................................
Cst-1-1   ............................................................................................
env-1089  ............................................................................................
env-1090  ............................................................................................
env-1091  ............................................................................................
env-1092  ............................................................................................
env-1094  ............................................................................................
env-1095  ............................................................................................
env-1096  ............................................................................................
env-1097  ............................................................................................
Csp-17-1  ............................................................................................
env-1098  ............................................................................................
env-1099  ............................................................................................
env-1100  ............................................................................................
env-1101  ............................................................................................
env-1102  ............................................................................................
env-1103  ............................................................................................
env-1104  ............................................................................................
env-1106  ............................................................................................
env-1107  ............................................................................................
env-1109  ............................................................................................
Lsp-6-1   ............................................................................................
env-1110  ............................................................................................
env-1112  ............................................................................................
Lph-1-1   ............................................................................................
Cph-1-1   ............................................................................................
env-1113  ............................................................................................
Csp-13-1  ............................................................................................
Lba-1-1   ............................................................................................
env-1114  ............................................................................................
env-1115  ............................................................................................
env-1116  ............................................................................................
env-1117  ............................................................................................
Mfo-1-1   ............................................................................................
env-1118  ............................................................................................
Pma-1-1   ............................................................................................
env-1119  ............................................................................................
env-1120  CUCAGCGUAGC.................................................................................
env-1121  ............................................................................................
env-1122  ............................................................................................
env-1123  ............................................................................................
env-1124  GUUCUGGAUUUCCUGUCACAUAAAAAAAGCUCCGUGGUGGCGCAAGAUGUCCGGUGGACAUCUUACGCCACGCGGAGUGGAACGGCGGCUGUG
env-1125  ............................................................................................
env-1126  ............................................................................................
Lsp-8-1   ............................................................................................
env-1127  ............................................................................................
Dsp-3-1   ............................................................................................
Dsp-2-1   ............................................................................................
env-1128  ............................................................................................
env-1129  ............................................................................................
Lco-1-1   ............................................................................................
env-1130  ............................................................................................
env-1131  ............................................................................................
env-1132  ............................................................................................
env-1133  ............................................................................................
env-1134  ............................................................................................
env-1135  ............................................................................................
env-1136  ............................................................................................
Ssp-1-1   CUGCUUUUUCAGCGAUUAGCUAAAAGAUGCAAGUAAUACAAGGAAAUGAUUUAGAAGGGAAAAAAUGAUAUAUAUUUAACAAUUUUGUGUC

          ............................................................................................
          ............................................................................................
          ............................................................................................
          ............................................................................................
          --------------------------------------------------------------------------------------------
```

```
env-1137  ............................................................................................
env-1139  ............................................................................................
env-1140  ............................................................................................
env-1142  ............................................................................................
env-1143  ............................................................................................
env-1147  ............................................................................................
env-1148  ............................................................................................
env-1150  ............................................................................................
env-1151  ............................................................................................
env-1153  ............................................................................................
env-1154  ............................................................................................
env-1155  ............................................................................................
Rfl-1-1   ............................................................................................
env-1156  ............................................................................................
env-1157  ............................................................................................
env-1158  ............................................................................................
env-1159  ............................................................................................
env-1160  ............................................................................................
env-1161  ............................................................................................
env-1162  ............................................................................................
env-1163  ............................................................................................
env-1164  ............................................................................................
env-1165  ............................................................................................
env-1166  ............................................................................................
env-1180  ............................................................................................
env-1181  ............................................................................................
env-1182  ............................................................................................
env-1183  ............................................................................................
env-1188  ............................................................................................
env-1189  ............................................................................................
env-1190  ............................................................................................
env-1191  ............................................................................................
env-1193  ............................................................................................
env-1194  ............................................................................................
env-1195  ............................................................................................
env-1196  ............................................................................................
Cbu-1-1   ............................................................................................
Csp-9-1   ............................................................................................
env-1197  ............................................................................................
Cbu-3-1   ............................................................................................
env-1199  ............................................................................................
env-1200  ............................................................................................
env-1202  ............................................................................................
env-1204  ............................................................................................
env-1205  ............................................................................................
env-1206  ............................................................................................
env-1208  ............................................................................................
env-1209  ............................................................................................
env-1210  ............................................................................................
env-1211  ............................................................................................
env-1212  ............................................................................................
env-1214  ............................................................................................
env-1215  ............................................................................................
env-1217  ............................................................................................
env-1220  ............................................................................................
env-1221  ............................................................................................
env-1222  CAUACUGUACGGAAACAUUUCCUACACCUCUUCUUAUUACGGAGAAAUGAUAACUUAUUUGGGAAUGACCGCACCAAUGGCUGUAUUUGCCC
env-1223  ............................................................................................
env-1224  ............................................................................................
env-1225  ............................................................................................
env-1226  ............................................................................................
env-1227  ............................................................................................
env-1228  ............................................................................................
env-1229  ............................................................................................
env-1230  ............................................................................................
env-1231  ............................................................................................
env-1237  ............................................................................................
env-1238  ............................................................................................
env-1239  ............................................................................................
env-1240  ............................................................................................
env-1241  AUUCAGUUUGCUUUACGGGUAUAUUUCGUUUGUUUUUACAUACUACGGCGAAAUGUUGACGUAUCUUUGCAUGACUAUGCCUAUGGCAAUCU
env-1242  ............................................................................................
env-1243  ............................................................................................
env-1244  ............................................................................................
env-1245  ............................................................................................
env-1246  ............................................................................................
env-1247  ............................................................................................
env-1248  ............................................................................................
env-1249  ............................................................................................
env-1250  ............................................................................................
env-1251  ............................................................................................
env-1252  ............................................................................................

          ............................................................................................
          ............................................................................................
          ............................................................................................
          ............................................................................................
          --------------------------------------------------------------------------------------------
```

```
env-1253 ............................................................................................
env-1254 ............................................................................................
env-1255 ............................................................................................
env-1258 ............................................................................................
env-1260 ............................................................................................
env-1262 ............................................................................................
env-1263 ............................................................................................
env-1264 ............................................................................................
env-1266 ............................................................................................
env-1269 ............................................................................................
env-1270 ............................................................................................
env-1271 ............................................................................................
Eba-1-1  ............................................................................................
env-1272 ............................................................................................
env-1273 ............................................................................................
env-1274 ............................................................................................
env-1275 CGCUACUACGGAGAAAUGAUUACUUAUCUCGGAAUGACCGCCCCAAUGGCGGUUAUCGCACUUAU...........................
env-1276 ............................................................................................
env-1278 ............................................................................................
env-1279 ............................................................................................
env-1288 ............................................................................................
env-1289 UUUUUGCGGUCUUUUACGGUAUUAUAUCGUUUUAUUUCAAGUAUUACGGAGAAAUGAUAACAUAUAUUUGCAUGACGUCUCCUAUAGCGGUU
env-1290 ............................................................................................
env-1291 ............................................................................................
env-1292 ............................................................................................
Cbo-1-1  ............................................................................................
env-1293 ............................................................................................
env-1294 ............................................................................................
env-1296 ............................................................................................
env-1300 ............................................................................................
env-1301 ............................................................................................
env-1302 ............................................................................................
env-1303 ............................................................................................
env-1304 ............................................................................................
env-1305 ............................................................................................
env-1306 ............................................................................................
env-1307 ............................................................................................
env-1308 ............................................................................................
env-1309 ............................................................................................
env-1310 ............................................................................................
env-1311 ............................................................................................
env-1312 ............................................................................................
env-1313 ............................................................................................
Bma-2-1  ............................................................................................
env-1315 ............................................................................................
Csp-4-1  ............................................................................................
env-1316 ............................................................................................
env-1317 ............................................................................................
env-1318 ............................................................................................
env-1319 ............................................................................................
env-1320 ............................................................................................
env-1321 ............................................................................................
env-1322 ............................................................................................
env-1323 ............................................................................................
env-1324 ............................................................................................
env-1325 ............................................................................................
env-1326 ............................................................................................
env-1332 ............................................................................................
env-1334 ............................................................................................
env-1344 ............................................................................................
env-1345 ............................................................................................
env-1363 ............................................................................................
env-1364 GCUUUUGAUGGUGCUGUUCAGCCUGCUUUACGGAAUGAUUUCCCUGAACGUUUCUUA...................................
env-1365 ............................................................................................
env-1366 ............................................................................................
Mma-1-1  ............................................................................................
env-1367 ............................................................................................
env-1371 ............................................................................................
env-1372 ............................................................................................
env-1374 ............................................................................................
env-1375 ............................................................................................
env-1377 ............................................................................................
env-1378 ............................................................................................
env-1386 ............................................................................................
env-1388 ............................................................................................
env-1392 ............................................................................................
env-1393 ............................................................................................
env-1399 ............................................................................................
env-1400 ............................................................................................
env-1401 ............................................................................................
env-1402 ............................................................................................
env-1403 ............................................................................................

         ............................................................................................
         ............................................................................................
         ............................................................................................
         ............................................................................................
         --------------------------------------------------------------------------------------------
```

```
env-1404  ............................................................................................
env-1405  ............................................................................................
env-1406  ............................................................................................
env-1407  ............................................................................................
env-1408  ............................................................................................
env-1409  ............................................................................................
env-1410  ............................................................................................
env-1412  ............................................................................................
env-1413  ............................................................................................
env-1414  ............................................................................................
env-1415  ............................................................................................
env-1416  ............................................................................................
[gl-1-1   ............................................................................................
env-1419  ............................................................................................
env-1421  ............................................................................................
env-1422  ............................................................................................
env-1423  ............................................................................................
env-1424  ............................................................................................
env-1425  ............................................................................................
env-1427  ............................................................................................
env-1428  ............................................................................................
env-1429  ............................................................................................
env-1431  ............................................................................................
env-1434  ............................................................................................
env-1435  ............................................................................................
env-1438  ............................................................................................
env-1439  ............................................................................................
env-1440  ............................................................................................
Cbo-3-1   ............................................................................................
env-1441  ............................................................................................
env-1442  ............................................................................................
env-1443  ............................................................................................
env-1444  ............................................................................................
env-1445  ............................................................................................
env-1446  ............................................................................................
env-1447  ............................................................................................
env-1448  ............................................................................................
env-1449  ............................................................................................
env-1450  ............................................................................................
env-1452  ............................................................................................
env-1454  ............................................................................................
env-1455  ............................................................................................
env-1456  ............................................................................................
env-1457  ............................................................................................
env-1458  ............................................................................................
env-1459  ............................................................................................
env-1460  ............................................................................................
env-1461  ............................................................................................
env-1462  ............................................................................................
env-1463  ............................................................................................
env-1465  ............................................................................................
env-1466  GCUUUGCCGCAUUUUUUAUCAACGAUGUUUACGGCUUCAUUAACUGAAAUAAAAUGGGAAAGCGGCAAGCGCAAAGCUGAUCGCUUUCCCGC
env-1467  GCUUUGCCGCAUUUUUUAUCAACGAUGUUUACGGCUUCAUUAACUGAAAUAAAAUGGGAAAGCGGCAGGCGCAAAGCUGAUCGCUUUCCCGC
env-1468  ............................................................................................
env-1469  ............................................................................................
env-1471  ............................................................................................
env-1473  AAUCCCGCGGGUCAGGCGCUCAUGAUAGUGUUCAGUGCGAUGUACGGCGUGAUAUCGUUCACCUUCGCGUA.....................
env-1474  ............................................................................................
env-1475  ............................................................................................
env-1476  ............................................................................................
env-1477  ............................................................................................
env-1478  ............................................................................................
env-1479  ............................................................................................
env-1480  ............................................................................................
env-1481  ............................................................................................
env-1482  AGAUGGCGCCAUGACAGAAAAGGAUUUAUGCUGGGAAACCCAAAGCAAGAGGAGGCUGCU................................
env-1483  ............................................................................................
env-1484  ............................................................................................
env-1485  ............................................................................................
env-1486  ............................................................................................
env-1487  ............................................................................................
env-1488  ............................................................................................
env-1490  GGAGAAAUGGUGACUUAUCUUGGUAUGACGAUGCCGAUGGCAAUAUUUGCACUUGUCUCAUGGCUAAAAAAUCCUUACAAUGGAAGAAGGUC
env-1491  UUUUCGUUGUCAAUCGGCACAAAACAAACGGUUCUCGGAGAUAAAAAAAUGCGUUCCGACGAAAUUGUGACAUAAUUGCAAAACGUCAAAA
env-1492  ............................................................................................
env-1493  UUUUCGUUGUCAAUCGGCACAAAACAAACGGUUCUCGGAGAUAAAAAAAAUGCGUUCCGACGAAAUUGUGACAUAAUUGCAAAACGUCAAA
env-1494  ............................................................................................
env-1495  ............................................................................................
env-1496  ............................................................................................
env-1497  ............................................................................................
env-1498  ............................................................................................
env-1499  ............................................................................................

          ............................................................................................
          ............................................................................................
          ............................................................................................
          ............................................................................................
          --------------------------------------------------------------------------------------------
```

```
env-1501  ............................................................................................
env-1502  ............................................................................................
env-1503  ............................................................................................
env-1504  ............................................................................................
env-1505  ............................................................................................
env-1506  UAACCGUGAUUUUCAGCCUGCUCUACGGCAUUAUAUC.......................................................
env-1509  ............................................................................................
env-1510  CAGAGGUGUCCACCGGACACCCGCACCGCAAUGACGUCAGCCCGCAAGAUCCGAUAUUCAAUAUUAUUUAUCAGGGAGGCUCAAAUGAAUAA
env-1511  ACAGCCGUUUACACGGUUUCGCAAUGACAGGUAAGCGAAAGUCUAUCAGCACACAUUGCGGCGCUCGCAAUGACAGGAUAUCGCAGUUUUUU
env-1512  ACACACCUGACGGUGGUCUCGCCUGCCGGCUCGGUCGGUGCUUCUGCUUCGCAGAGGUGUCCACCGGACACCCGCACCGCAAUGACGUCAGU
env-1513  ............................................................................................
env-1514  ............................................................................................
env-1516  ............................................................................................
env-1517  ............................................................................................
env-1518  AUCGCUGCAAGGAUAACUAUAAUAAUAGAUAUUCUAAUUUUAGAGUUUUUGCAAUUGGAGGAUGAAUAUGAACCAUUGGAAUUACACGGCAU
env-1520  ............................................................................................
env-1521  ............................................................................................
env-1523  ............................................................................................
env-1524  ............................................................................................
env-1525  ............................................................................................
env-1529  ............................................................................................
env-1530  ............................................................................................
env-1532  ............................................................................................
env-1535  ............................................................................................
env-1536  UUUAGUGUGCUGUAUGGCUUUAUAUCUUACACAUUCUCAUA...................................................
env-1537  UUUAGUGUGCUGUAUGGCUUUAUAUCUUACACAUUCUCAUA...................................................
env-1538  ............................................................................................
env-1539  ............................................................................................
env-1540  ............................................................................................
env-1541  UGCUGUAUGGGAUUAUUUCAUAUACCUUUUCGUAUUAUGGGGAGAUGAUUACAUAUCUUGGAAUGACAAUGCCGAUGGCAAUUUUUGCCUUA
env-1542  UGCUGUAUGGGAUUAUUUCAUAUACCUUUUCGUAUUAUGGGGAGAUGAUUACAUAUCUUGGAAUGACAAUGCCGAUGGCAAUUUUUGCCUUA
env-1543  ............................................................................................
env-1544  ............................................................................................
env-1545  UUAUAAAACAUCUUUUUCAUUUCAGUUCAUGUAUCGCUCUAACCUUUCACUGGUGGAAUAUUCUAAUAUUUCAGAAAU..............
env-1546  GCUAACAGUCAUAGGGUAGCGGAUAUAGUAUAAGUAUAUGUCAGAAAAGAGGUGUUUAUUCAUGGCAAGAAAGCCGACUGAUAAGCAGUUGU
env-1547  ............................................................................................
env-1553  ............................................................................................
env-1602  ............................................................................................
env-1603  ............................................................................................
env-1604  ............................................................................................
env-1607  ............................................................................................
env-1608  ............................................................................................
env-1682  ............................................................................................
env-1689  ............................................................................................
env-1690  ............................................................................................
env-1691  ............................................................................................
env-1692  ............................................................................................
env-1693  ............................................................................................
env-1695  ............................................................................................
env-1696  ............................................................................................
env-1706  ............................................................................................
env-1707  ............................................................................................
env-1708  ............................................................................................
env-1711  ............................................................................................
env-1712  ............................................................................................
env-1713  ............................................................................................
Rfl-2-1   ............................................................................................
env-1715  ............................................................................................
env-1717  ............................................................................................
env-1718  ............................................................................................
env-1719  ............................................................................................
env-1720  ............................................................................................
Oru-1-1   ............................................................................................
env-1721  ............................................................................................
env-1722  ............................................................................................
env-1723  AUGAUAAUUUUCAGUGUGCUGUACGGCAUUAUAUCCUUUACGUUUGCUUA..........................................
env-1724  ............................................................................................
env-1725  ............................................................................................
env-1726  ............................................................................................
env-1727  ............................................................................................
env-1728  ............................................................................................
Ral-1-1   ............................................................................................
env-1729  ............................................................................................
Gni-1-1   UCAACUUAUUGAAAAUUACAUGGAUACAAUAUAACCAUUUAUUAAUGAUAAUACAAUUUUUAAAAAUAAGGAAUCACAACAGUUCAUGAUAA
env-1730  ............................................................................................
env-1731  ............................................................................................
env-1732  ............................................................................................
env-1740  ............................................................................................
env-1741  ............................................................................................
env-1742  ............................................................................................
env-1743  ............................................................................................
env-1744  ............................................................................................

          ............................................................................................
          ............................................................................................
          ............................................................................................
          ............................................................................................
          --------------------------------------------------------------------------------------------
```

```
env-1745  ............................................................................................
env-1746  ............................................................................................
env-1747  ............................................................................................
env-1748  ............................................................................................
env-1749  ............................................................................................
env-1750  ............................................................................................
env-1751  UGUUUUUAUGCAAUGAUAUUUAUGCGUUGGUGAACUGGCAGCGGAUGGAGCAGAGACAAAAACGGGGAAAUACCGCCUAGCUGCGAAAGGGU
env-1752  ............................................................................................
env-1753  AAGUAUCUUUCUGUGUUUGUUUGCGUUGCGACCUUUUUGUGCAAUGAUAUUUACGGGGAUAUAUCAGUCGGGAGAAAAUGUGCCGCAGGCAG
env-1754  ............................................................................................
env-1756  ............................................................................................
env-1757  ............................................................................................
env-1759  ............................................................................................
env-1761  ............................................................................................
env-1762  ............................................................................................
env-1763  ............................................................................................
env-1764  ............................................................................................
env-1765  ............................................................................................
env-1766  ............................................................................................
env-1768  ............................................................................................
env-1769  GGAGCCUGUGGCU...............................................................................
env-1770  ............................................................................................
env-1771  ACGUAUCUCGGCAUGACCGCACCCAUGGCUGUCUUCUCUCUUUUUUCAUGGCUGCACAAUCCUUACUUCAGCUGUGACUUUUGCUUUUUAUU
env-1772  ............................................................................................
env-1773  ............................................................................................
env-1774  UGUUGUCGCUUAGAAUAUUUUAAUC...................................................................
env-1775  ............................................................................................
env-1776  ............................................................................................
env-1778  ............................................................................................
env-1779  ............................................................................................
env-1780  ............................................................................................
Rsp-3-1   ............................................................................................
env-1781  ............................................................................................
env-1789  ............................................................................................
env-1792  ............................................................................................
env-1794  ............................................................................................
Rsp-2-1   ............................................................................................
env-1796  ............................................................................................
env-1800  ............................................................................................
env-1801  ............................................................................................
env-1802  ............................................................................................
env-1803  ............................................................................................
env-1804  ............................................................................................
env-1805  ............................................................................................
env-1806  ............................................................................................
env-1807  ............................................................................................
env-1808  ............................................................................................
env-1809  ............................................................................................
env-1810  ............................................................................................
env-1811  ............................................................................................
env-1812  ............................................................................................
env-1813  ............................................................................................
env-1814  ............................................................................................
env-1815  ............................................................................................
env-1816  ............................................................................................
env-1818  ............................................................................................
env-1819  ............................................................................................
env-1820  ............................................................................................
env-1821  ............................................................................................
env-1822  ............................................................................................
env-1823  ............................................................................................
env-1824  ............................................................................................
env-1825  ............................................................................................
env-1829  ............................................................................................
Dsp-1-1   ............................................................................................
env-1830  ............................................................................................
Lba-3-1   ............................................................................................
env-1831  ............................................................................................
env-1832  ............................................................................................
env-1837  ............................................................................................
env-1838  ............................................................................................
env-1839  ............................................................................................
env-1840  ............................................................................................
env-1842  ............................................................................................
env-1843  ............................................................................................
env-1844  ............................................................................................
env-1845  ............................................................................................
env-1846  ............................................................................................
env-1848  ............................................................................................
env-1849  ............................................................................................
env-1850  ............................................................................................
env-1851  ............................................................................................

          ............................................................................................
          ............................................................................................
          ............................................................................................
          ............................................................................................
          --------------------------------------------------------------------------------------------
```

env-1852	....
env-1853	....
env-1857	....
env-1858	....
env-1859	....
env-1860	....
env-1861	....
env-1862	....
env-1864	....
env-1865	....
env-1866	....
env-1869	....
env-1870	....
env-1871	....
Ssu-6-1	....
Ssu-1-3	....
Ssu-1-4	....
Ssu-1-7	....
Ssu-1-8	....
env-1872	....
env-1873	....
env-1875	....
env-1876	....
env-1877	....
env-1878	....
env-1879	....
env-1880	....
env-1881	....
env-1882	....
env-1884	....
env-1885	....
env-1886	....
env-1887	....
env-1888	....
env-1889	....
env-1890	....
env-1891	UCAUAACAACCAGCUUUUUUGACAGCUU...
env-1892	CGUUGCAUGUUCUGGGCAUGAUCGGGUU...
env-1893	....
env-1894	....
env-1895	....
env-1896	....
env-1897	....
env-1898	....
env-1899	....
env-1900	....
env-1901	....
env-1902	....
env-1903	....
env-1904	....
Par-1-1	....
env-1905	....
env-1906	....
env-1907	....
Efa-293-1	....
Efa-66-1	....
Eca-2-1	....
Efa-286-1	....
Efa-202-1	....
Lli-2-1	....
	....
	....
	....
	....

1.6 Multiple-sequence alignment curated

This alignment contains a hand-curated subset of GGAM-1 RNAs (now renamed guanidine-IV riboswitches). We have carefully tried to align all stems. This alignment is therefore suitable for structural analysis and as a seed alignment for homology searches. A more comprehensive list of GGAM-1 RNAs is in the GGAM-1.sto alignment.

Each GGAM-1-curated RNA is denoted by an abbreviation (like "Eco-1-1") that refers to a taxonomy given in Section 1.1. (Superscript numbers refer to annotations listed in Section 1.2) The alignment may include sequences containing the RNA motif, as well as flanking sequence. The GGAM-1-curated RNA itself is denoted by the line underneath marked 5′ and 3′ on either end. Nucleotides in flanking sequences (i.e., not part of the motif) are written in gray letters. Stems

of predicted rho-independent transcription terminators, if any, are shaded yellow. (Note: terminator predictions have not been analyzed manually, and many are likely to be false positives. Terminator predictions are those of the RNIE software.) Nucleotides predicted to function (as DNA) as transcription-factor binding sites, if any, are shaded in green. (But note: these predictions are manually annotated, so they might be under-predicted.) Annotated start codons, if any, are shaded green. (Note: start codons are frequently misannotated, especially in environmental samples.) Nucleotides proposed to basepair as part of the consensus structure are shaded in color when they comprise Watson-Crick or G-U pairs. Otherwise they are shaded gray. Conserved stems are also indicated at the bottom of the alignment by angle brackets, where matching $<$ and $>$ denote base-paired columns. Below these angle brackets, the symbol "2" denotes base pairs exhibiting covariation according to the statistically well-founded R-scape method. "1" denotes base pairs exhibiting covariation according to R2R's simplistic method. "0" denotes base pairs that are not observed to mutate and "?" denotes base pairs that have a significant frequency of non-canonical nucleotides for Watson-Crick or G-U pairs ($>$ 5%). Below these base pair annotation is the consensus sequence: "R" = "A" or "G", "Y" = "C" or "U", red nucleotides: nucleotide identity conserved more than 97% of the time, black nucleotides: 90%, gray nucleotides: 75%, red circle (•): nucleotide is present 97% of the time, black circle (•): 90%, gray circle (•): 75%, white circle (◦): 50%. All percentages of sequences just described (e.g. 97% conserved) assume that sequences have been weighted by the GSC algorithm implemented by the Infernal software package.

alignment positions 1 · · · 138

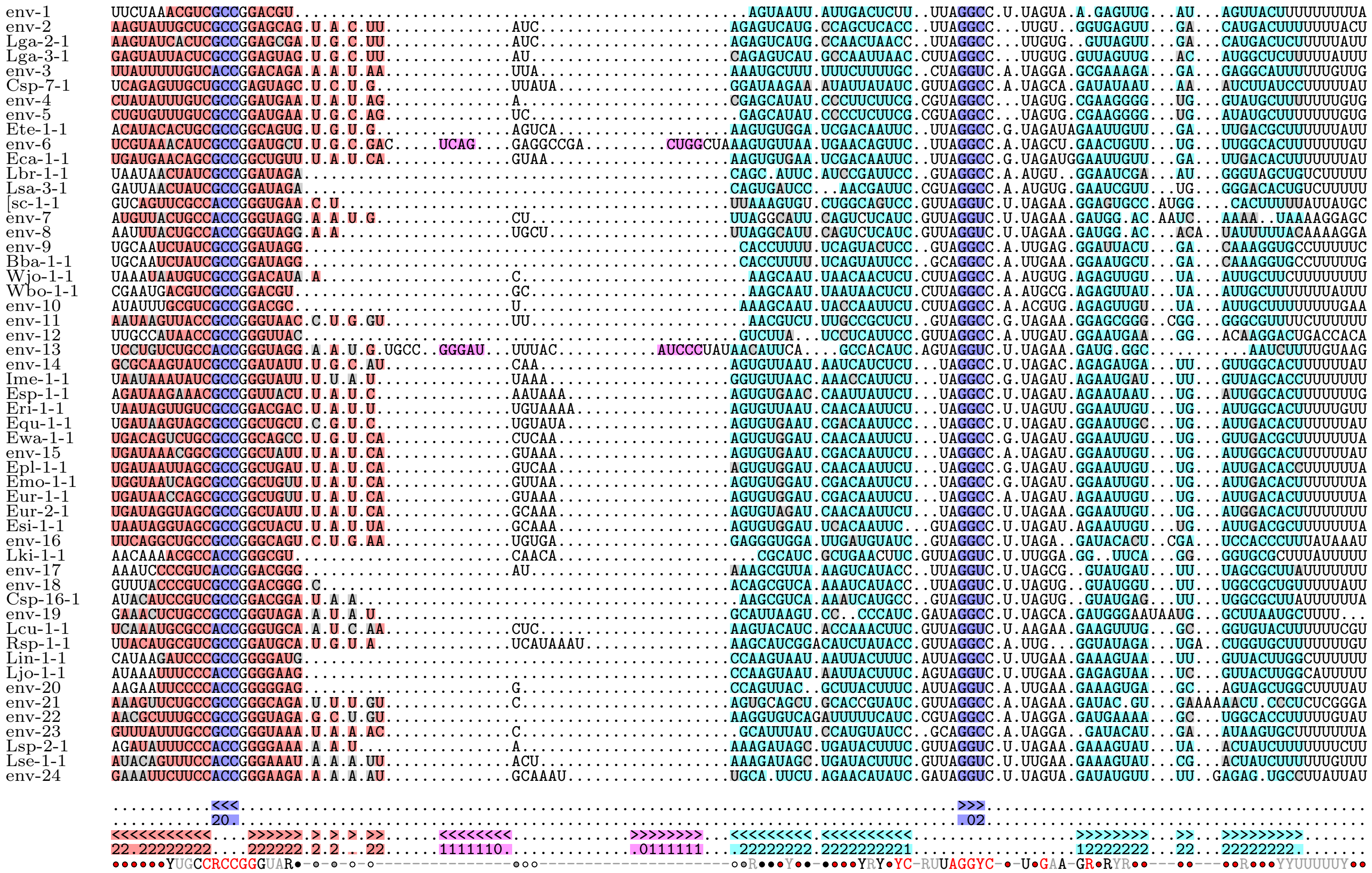

```
env-25     UGAAUAACACCGCCGGGUGUA.A...A.CA..............AAAA....................UAGUACUCU.UAAUAAUUCC..GUAGGCC.A.AUGU..GGAAUUAU...UA...GGGGUACUUUUAUUUU
env-26     CAAACUUCGCCGCCGGGUGAA.G.U.C.U...............AUAAUAA.................AAGCACAGA.AUGGUAUACC.UUUAGGCC.G.UAGUA.GGUAU.CC...AU...UUGGUGCUUUUUUAUA
Cni-1-1    GUAAAAAUAUCGCCGGAUAUU.U.U.A.UU..............UAAUUACA................AGCAUUAGA.GAUUUAUACC.AUUAGGCC...UUGG..GGUAUAAA..CUA...UCUAGUGCUUUUAUUU
Csp-15-1   AAUGAACUGUCACCGGACAGC.G.A.A.CU..............UGU.....................AAAAGUUCA.UCUUUAUCUC.UUUAGGUC.U.UAGUA.GAGGUAAU...GA...UGAGCUUUUUUUUUUU
env-27     UGUCACCUGCCGCCGGGUAGG.U.G.U.CG..............CAU.....................CGGCAUUCG.CGCCUUGCUC.AUUAGGCC.U.UUGAA.GAGCAAGA...U....CGGGUGUUUUUUUAUU
env-28     UGAAUUUCGCCACCGGGUGAA.A.U.G.CA.........................................GAUGUC.AAUGUACUCC.GUUAGGUC.U.UAGAA.GGAGUGCA...UU...GGCAUCUUUUUGUCUG
Osp-1-1    AUAGGUCCGCCGCCGGGCGGA.C.A.A.A.......................................GGCGUUUGC..CCCUGCAUC.CGUAGGCC.A.UUGGA.GAUGCGGG...GC...GCGGGCGCCUUUUAUU
env-29     UCCCCUUUGCCGCCGGGUAGA.G.G.G.AA..............C........................GGGCGACAGGCUGCAUGUC.AUUAGGCC.A.UAGAA.GACAUG.G...GCA..UGCCGCUUUUUGACGC
env-30     GCAGCUUCGCCACCGGGUGGA.G.C.U.G...............AUA.....................AGCGUCAG..GUUUUCCGUC.GUUAGGUC.U.UAGAA.GAGGGAAA...AC...GCUGGCGCUUUUUUAG
env-31     CAUCAUUAGCCACCGGGUUAA.U.G.....................UGU......................AAGGUGUA.UCCCUUUUCC.AUUAGGUC.U.UAGAA.GGAAAAGG...AA...UGCACCUUUUUAUGUU
env-32     AUAGAUGUUCCACCGGGAACA.A.U.G.A...............G..........................AUAGUA.AUACUUUUCC.AUUAGGUC.A.UAGAA.GGAAGGGU...GU...UGCUAUCUUUUUUAUU
env-33     CUAUACAUGCCGCCGGGUAUG.U.A.G.GG..........................................AGCUU.GUUAUGCAUC.AUUAGGCC.U.UAGAA.GAUGCAUA...AU...AGGCCUUUUUUUGUAG
env-34     AAUUAUUCACCACCGGGUGAA.U.A.....................UA......................AGGAUGUAU.CAAGGUUCUC.CGUAGGUC.U.UAGAAGGAGUACUU...UG...AUAC.UUUUUUAUUAG
env-35     GUUGAGCCGCCGCCGGGUGGC.U.A.G.UU..............AG......................AAAAGCGCG.GAUUCGUAUC.UUUAGGCC.U.UAGUA.GAUACGAA...UUGA.GGCGCUUUUUGUCAUU
Bpu-1-1    CGUUCGUUAUCGCCGGAUAGC.G.G.A.C...............CGUA....................AACGCUUGG.AUUCUGUAUC..GUAGGCC.U.UUGAA.GAUACAGA...ACA..CC.GGCGUUUUUAUUU
Esp-5-1    AACUACAUGUCGCCGGACAUU.........................................................ACCUC..UAUCUAUUCC.AGUAGGCC.U.UAGUU.GGAAUGGA...UA...AGAGGUUUUUUUAAUG
Lcr-1-1    UAAACACUUCCGCCGGGAAGC.A.A.A.UA..............AAU.....................AAGAGUAAU.UAUAUAUCUC.AUUAGGCC.U.UUGAA.GAGAUAUA...CA...AUUGCUCUUUUUUUGG
Lta-1-1    UAAACACUUCCGCCGGGAAGU.A.A.A.CA..............................................AA....UAUAACUC.AUUAGGCC.U.UUGAA.GAGAUAUA...CA...AUUGCUCUUUUCUUUU
Lul-1-1    AUCUAUCUCCCACCGGGGAGA.A.A.A.CU..............AAA.....................AAGAGUAAU.UACAUACUUC.AUUAGGUC.U.UUGAA.GAAGUAUG..UCA...AUUACUCUUUUCUUUU
Lsa-2-1    AAUAAUCUUCCGCCGGGAAGU.U.A.A.................AAUU....................CAGGAGAGC.CGGACGUCUC.AUUAGGCC.U.UUGAA.GAGAUGUC...UG...ACUCUCCUUUUGUUUU
1 Lag-1-1  AGCCAACUUCCGCCGGGAAGU.A.U.U.AU......................................UAGGAAAGC.CACCCGUUUC.GUUAGGCC.U.UUGCA.GAAACGGG...GG...ACUUUCCCUUUUUGUU
Rti-1-1    AAGCAUCUGCCGCCGGGUAGA.......................CCAAGAACAAA.............GCAUGUGCG...CCCCUAUC.GUUAGGCC.A.UAGAA.GAUAGGGG...AA...AGCGCAUGCUUUUUUA
env-36     AGUUAGGCGUCACCGGGCGCU.U.A.A.C...............GGAUGCG.................AAAGCAUUU.UAUCCGUGUC.GUUAGGUC.U.UUGAA.GAUACGGA...UA...AAAUGCUUUUUUUCAG
Fnu-1-1    AUUAAUUCGCCACCGGGUGAA.A.G.C.UU.........................................AUGCUA.GUUUUGUAUC.GUUAGGUC.U.UUGAA.GAUACAAC...AU...UAGCAUUUUUUUAAAU
Fsp-2-1    AUUAAUUCGCCACCGGGUGAA.A.G.C.UU.........................................AUGCUA.GUUUUGUAUC.AUUAGGUC.U.UAGAA.GAUACAAA..UAC...UAGCAUUUUUUAAAAA
Fnu-1-2    UUUAUUUUGCCACCGGGUGAG.A.......................GCUU.......................AUGCUA.GUUUUGUAUC.GUUAGGUC.U.UAGAA.GAUACAAG..GAC...UAGCAUUUUUUAAAAU
Lde-1-1    CAGAAUUCACCACCGGGUGA........................UGC........................AGCGUU.UAUCGUACUC.CAUAGGUC.U.UAGCG.GAGUGCGG...UA...GGCGCUGUUUUAUUUU
env-37     UUAUUCCUGCCGCCGGGUAGG.A.A.U.................CUGAUA..................AGCAUUCGC.GCCCAAUACC.GGUAGGCC.U.UAGCA.GGUAUUGG...GAAG.GCGGAUGCUUUUUUUG
Lla-1-1    AUAAAUACUCCACCGGGAGU..U.......................AAAUCGUAU...............GAACGAUUA.UUUACAUUUC.AGUAGGUC.U.GAGAA.GAAAUGUA...GA...UGGUCGUUCUUUUUUU
Lla-2-1    AUAAAUACUCCACCGGGAGU.........................UAAAUCGUAU.............GAACGAUUG.UUUGCAUUUC.AGUAGGUC.U.GAGAA.GAAAUGUA...GA...UAGUCGUUCUUUUUUU
env-38     AUAAUAACUCCACCGGGAGUA.A.A.U.................CGUAA...................AAGCGAUUA.UUUGCAUUUC.AGUAGGUC.U.UGGAA.GAAAUGCA...AA...UAGUCGCUUUUUCUUA
env-39     AUAAAUGCUCCACCGGGAGU........................CAAACGC.................UAAGCGGUU.UUUACAUUUC.AGUAGGUC.U.UUGAA.GAAAUGUG...GGA..AGUCGUUUUUUUUAUU
env-40     UUACCACUGCCACCGGGUAGU.A.U.A.A...............UGCAGAUC................AAGC.AUUC.GGUAUGGGUC.GUUAGGUC.U.UAGAA.GAUCCAUG...CU...GGAU.GCUGUUUUUUA
Whe-1-1    AUAAUAGUUCUACCGGGAACU.U.G.U.................CAUUU...................AGGCAAUUA.UUAACUUAUC.AUUAGGUC.G.UUGAA.GAUAAGUU...AA...UAAUUGCUUUUUAUUU
Whe-1-2    AUAAACGUUCCACCGGGAACU.U.G.U.................CGUUU...................AGGCAAUCA.UUAACUUAUC.AUUAGGUC.A.UUGAA.GAUAAGUU...AA...UGAUUGCCUUUUUAUU
env-41     CCCCAUCUGCCACCGGGUAGA.U.G.G.AG..............AA......................AAGUGUCCA.GCCGUAUUCC.GGUAGGUC.U.UAGAA.GGAAUACG...GC...UGGAUAUUUUUGUUUG
env-42     UUUGUUCCGCCGCCGGGUGGG.A.C.G.A...............UUC.....................AAACACCGG.UUUUUCCGUC.AUUAGGCC.U.UUGAA.GACGGAAA...GC...GCGGUGUUUUUUUAUG
env-43     UUUGUUCCGCCGCCGGGUGGA.A.C.G.GA..............C.......................AAACACCUU.AUUUUCCGCC.GUUAGGCC.U.UUGAA.GACGGAAA...AU...GCGGUGUUUUUUUUAU
env-44     UAUUGUUCGCCGCCGGGUGAA.C.A.C.GG......................................AGUGCUUCG.GCGCUGUAUC.GUUAGGCC.G.UAGAA.GAUACAGC...GCA..GGGAGCGCUUUUUCUU
env-45     GAUCACUCGCCGCCGGGCGGG.U.G.....................C........................UGCGUUUG.UUUCUGUACC.AUUAGGCC.G.UAGAA.GGUACAGA...AA...GAAACGCAUUUUUUAU
Lfu-1-1    AAAUAACCGUCACCGGGCGGU.......................C.........................AGGUAUU.GCCAAAUCUC.AUUAGGUC.A.UUGAA.GAGAUUUA...GC...GAUACCUUUUUUAUUU
Lfa-2-1    UAUUUAACACCACCGGGUGUC.A.......................U..........................AGCAUU.UGUAUAGCUC.AUUAGGUC.A.AUGAA.GAGCUGUA...UA...GGUGCUUUUUAUUUUA
Pac-1-1    AAGAAAUCACCACCGGGUGGU.U................................................GGCAUG.UGUAAAGCUC.AUUAGGUC.U.GAGAA.GAGCUUUA...CA...GAUGCCUUUUUUGUUU
Lsp-4-1    GAAGUAUCGCCACCGGGCGAC.A.................................................GCACU.UACCAAACUC.UUUAGGUC.U.UAGUA.GAGUUCGG...UA...GGUGCUUUUAUUUUUG
Lsp-1-1    AUUUAAUCACCACCGGGCGAU..................................................AGCACU.UAGUGAACUC.UUUAGGUC.U.UAGUA.GAGUUUGC...UA...GGUGCUCUUUUUUUAG
Esp-2-1    AAAAAAUCACCACCGGGUGAU.......................CCC........................AGUAUG.UAGACAACUC.UUUAGGUC.U.UAGUA.GAGUUGGC...UA...GAUGCUUUUUUUUAUG
env-46     UUAAUAUCACCACCGGGUGAU.A.......................AG........................AGGUACU.UAGUAAACUC.AUUAGGUC.U.UUGAA.GAGUUUAU...UG...AGUACCUUUUUUUGUA
Csp-6-1    AAUGGGUUGCCACCGGGUAAC.A.C.A.UU..............UCA.....................UGAUACUUA.ACUUUAUUCC.UUUAGGUC.U.UAGUA.GGGAUAAA..UGU...UAGGUAUUUUUUUAUG
Asp-2-1    CGCUGUUUGCCGCCGGGUAAA.C.A.....................AUCA....................AGCAUUGAU.CUUCCGUAUC.GUUAGGCC.A.UAGAA.GAUACGGA...AG....UCAAUGCUUUUGUUU
env-47     UUGAUGUUGCCGCCGGGUAAC.A.A.G.CA..............UCAAA...................UGCACCAGC.C..CCAUGUC.GUUAGGCC.U.UAGAA.GACAUGGC...AG...GCCGGUGCAUUUUUUU
env-48     UAACAAUCGCCGCCGGGUGA.........................................................CACCCA..CC.AGUUCC.GUUAGGCC...UUGAA.GGAACUUG...GA...GGAGUGUCUUCUUUUU
env-49     AUUUGACCGCCGCCGGGUGGU.U.U.A.CU..............UUA.....................GAGAGCGCA.GGCUCACAUC.GUUAGGCC.U.UAGAA.GAUGUGAG...GU...CAGGCGCUUUUCGUAU
env-50     GCAUACCGGCCGCCGGGUCGG.U.A.U.G...............AC......................CAGCGUUUG.CCUGCGCACC.GUUAGGCC.U.UUGAA.GGUGCGCA...GA...CAGACGCCGGAUUUUU
env-51     CAAUAAAUACCGCCGGGUAU..........................................................ACGUCAU.UAUGUGCUUC.GUUAGGCC.U.UAGAA.GAAGCAUG...UA...GUGACGUUUUUUUCUU
env-52     AAUUGCUUGCCGCCGGGUAAG.C.U.....................GAAA....................UAACGCUGU.GUCAUAUAUC.GUUAGGCC.U.AUGAA.GAUAUAUG...GUA..UCAGCGUUUUAUUUUG
env-53     UAUGCUUUGCCGCCGGGUAAA.G.C.U.................GUAUGAG.................CGCUCGAA...UUUCAUACC.AUUAGGCC.U.UUGAA.GGUAUGAA...CU...UUUUGGGCGCUUUCUU
env-54     UUGCGACUGCUACCGAGUAGU.C.G.C.GG..............UGCAAUC.................GAAGCAUUU.UGUUCGCGUC.GUUAGGUC.U.UUGAA.GAUGCGAA...CG...GAAUGCUUUUUUUGAG
Fpl-1-1    UCCCGUUUAUCACCGGAUAAG.U.G.U.GA..............A.......................ACGUGCCUG.AACCGAUUCC..GUAGGUC.U.UAGAA.GGAAUCGG...CC...CAGGCACGUUUUUUGC
Fsp-1-1    UGACGCCCGUCACCGGAUGGG.C.G.A.GA..............CA......................ACGUGCCUG.AGUAUAUUCC..GUAGGUC.U.UUGAA.GGAAUAUA...CU...CAGGCACGUUUUUCAU
env-55     UGACGCCUGUCACCGGAUAGG.C.G.A.UA..............AA......................ACGUGCCUG.AGUAUAUUCC..GUAGGUC.U.UUGAA.GGAAUAUA...CU...CAGGCACGUUUUUUGU
env-56     UUUGGUUUGCCGCCGGGUAAA.C.A.G.GA......................................AAGCAUUGG.UUUCCGCGUC.GUUAGGCC.G.UAGAA.GACGCGGA...GG...GCAAUGCUUUAUUUUU
env-57     AGCGCGUUGCCGCCGGGUAAC.A.G.A.C...............AGAUA...................AUGCAUCUG.CCUCCAUGUC.GUUAGGCC.A.UAGAA.GACAUGGA...AG...CCGGUGCAUAUUUUUU
env-58     AGAAGGCUGCCGCCGGGCAGC.A.A.A.C................AGGA...................GUGCACCUG.CACCUAUGUC.GUUAGGCC.U.UAGAA.GACAUGGG...AG...CCGGUGCAUAUU....
env-59     AAAAAAUUACCGCCGGGUAG.........................................................CGCU.U.UCUCAACUUC.GUUAGGCC.U.UAGAA.GAAGU.GA...GA...AUAGCGUUAUUUUUUU
env-60     UCUUGCGCGCUGCCGAGUGCG.C.A.A.AA.........................................GCGUUU.UUUCUGUAUC.AUCAGGCC.U.UAGCA.GAUACAGA...AA...AGGCGCUAUUUUUGUU
env-61     UAUUUUUUGCCACCGGGUAAA.A.A.C.UA..............U..........................GCACCU.AAGGCGUUCC.GUUAGGUC.U.UUGAA.GGAGCGUA...C....AGGUGCUUUUUUAUUA
env-62     AAAAUGUCGCCACCGGGUGAC.G.......................GAACGUAAAAA.............AGCGUUCGC.GG.GCGUAUC.GUAAGGUC.U.UAGAA.GAUACGUA...GG...GCGGGCGUUUUUUGUU
Nma-1-1    AUUGUUUAAUCACCGGAUUAA.G.C.A.A...............AUAGA...................AGUGUCUAA.AACAUAUUCC..GUAGGUC.U.UAGAA.GGAAUGUG..AUU...UUAGGCACUUUUUUGU
Csp-7-2    AUUUCUUUGUCGCCGGAUAAA.G.A.A.A...............AUUAUA..................AGCGUUUAA.AUAUCACGCC..GCAGGCC.U.UUGAA.AGUGUGAU...GU...UUAGACGCUUUUGUUU
Csa-1-1    AUUUCUUUGUCGCCGGAUAAA.G.A.A.AU..............UAAUA...................AGCGUUUAA.AUUUCACGCC..GCAGGCC.U.UUGAA.AGUGUGAU...GU...UUAAGCGCUUUAUUUU
Lpe-1-1    AUAAUACUAUCACCGGGUAG................................................CAGUACUGU.GUGACAUUCC.GUUAGGUC.U.UUGAA.GGGAUGCC...GU...UCAGUACUGCUUUUUG
env-63     GUAACUCUGCCGCCGGGUAGA.G.......................AAUGAAUC...............AGGCGUUCGA.UUUUCACAUC.UUUAGGCC.U.UAGUA.GAUGUGGA..UAG...UCGGACGCUUUUUUAU
env-64     CUGUUCCCGCCGCCGGGUGGG.A.A.C.AG..............G.......................GAACGUUUG.CGUUCACGUC.GUUAGGCC.G.UUGAA.GACGUGGG..GCA...GACGUAUUUUUUAUUU
env-65     CCGUUCCCGCCGCCGGGUGGG.A.A.C.GG..............G.......................GAACGUUUG.CGUUCACGUC.GUUAGGCC.A.UUGGA.GACGUGGA...UG...UGAACGUAUUUUUUUA
env-66     AAAGAACUGCCACCGGAUAGU.U.C.A.U...............GCGAUA..................AGCAUUAAG.UCUAUACAUC.GUUAGGUC.U.UAGAA.GAUGUAUA...GA...AGUGAUGCCUAUUUUU
Bsp-1-1    AUUGAAUUGCCGCCGGGUAA...................................................CG..UCUC.AGUCUGUUCC.AUUAGGCC.A.UAGAA.GGAACAGA...UA...UGGAGACGUUUUUUUC
env-67     GUGUUUCUGCCACCGGGUAGA.G.A.A.UC..............UU......................GAGCAUUCG.AGUAUGUAUC.GUUAGGUC.U.UAGAA.GAUACAUG...G....CGGAUGCUUUUUUAUU
env-68     UGUAUUUUGCCGCCGGGCAAA.A.U.A.AA.......................................GACGCCCC.UGCUUGUAUC..GUAGGCC.U.UAGAA.GAUACAGG...CAA..CGGGCGUCUUUAUUUU
env-69     UAUUUUUCGCCGCCGGGUGAA.U.C.G.GA..............AACU....................AUGUGUUUC.UCUUUGAAUC.GUUAGGCC.U.UAGAA.GAUUCAAA...GG...AGGGGACACAUUUUUA
env-70     UUAUUUUUGUCGCCGGAUAAA.A.A.U.AA..............AA......................AAGCAUCAA.AGCAUCUGUC..GUAGGCC.A.UAGAA.GAUAGAUG...AU...UUGGUGCUUUUUUUGA
env-71     UGACUUUUGUCGCCGGAUAAA.A.G.A.UA......................................UAACAUCAA.AUACUCUAUC..GUAGGCC.A.UUGAA.GGUAGAGU...GU...UUGAUGUUUUUUUAUU
env-72     GAGAUUCUGUCGCCGGGCAGA.......................CAA.....................AAGCGUUGU.AUUCCACAUC.GAUAGGCC.G.UUGGA.GAUGUGGA...AUG..ACAGCGCUUUUCUUUU

           ...........<<<...............................................................................>>>..........................................
           ...........20.................................................................................02..........................................
           <<<<<<<<<<<....>>>>>>.>.>.>.>>......<<<<<<<<.............>>>>>>>>...<<<<<<<<<.<<<<<<<<<<..................>>>>>>>>...>>...>>>>>>>>>.......
           22.22222222....222222.2.2...22......1111110..............0111111...22222222.2222222221..................12222222...22...22222222........
           ●●●●●●YUGCCRCCGGGUAR●-○-○-○-○---------------○○○---------------------○○R●●●Y●●-●●●●YRY●YC-RUUAGGYC-●-U●GAA-GR●RYR●●---●●---●●R●●●YYUUUUUY●●
```

```
Fva-1-1    GUAAGUCUGCCGCCGGGCAGA.......................AGAUGAAUC..............AGGCGUUCA.AUUUCACAUC.UUUAGGCC.U.UAGUA.GAUGUGAA.AAAC...UGAACGCUUUUUUUAU
Csp-8-1    AAUUACUUGCCGCCGGGUAAG.A.A.A.U...............CAGAAA.................AGCACUUAU.UGAUUGCAUC.GUUAGGCC.U.UUGAA.GAUGCAAU...CA...AUAGGUGCUUAUUUUA
env-73     ACUUCUUUGCCGCCGGGUAAA.G.A...................AUAA...................AGUGUCAGG.CUUUCUAUCC..UUAGGCC.U.UUGGA.G.AUAGAA..AGU...UCUGGCACUUUUUUAU
Cbo-2-1    ACAUGUUUGCCGCCGGGUAAA.U.A.U.GU..............UA.....................AGCGUCAGU.UAUCCUAUCC..GUAGGCC.UUUUGGA.G.AUAGGA..CAU...UCUGGCGCUUUAUUUU
env-74     UAAGUUCUGCCGCCGGGUAGA.G.G.A.UG..............U......................GAGCAUCGA.GCGCCGUAUC.GUUAGGCC.U.UAGAA.GAUACGGA..UGC...UGGAUGUUUUUUUAGG
env-75     CCUGAAAAGCCGCCGGGUUUU.U.G.A.................................................GUCUUG.GUCCCAUUCC.GUUAGGCC.U.UUGAA.GGAAUGGU...GA...CGGGGCGCUUUAUUUU
env-76     UUCACUUAGCCGCCGGGUUAG.G.U.G.A...............CAG....................AGUGCCUUUGAG.CCAUAUC.GUUAGGCC.U.UAGAA.GAUAUGGA...CU...UAUGGCACUUUUAUUU
env-77     UUCACUUAGCCGCCGGGUUAA.G.U.G.U...............UGA....................GGUGUCAUA.AGUCCAUGUC.GUUAGGCC.U.UAGAA.GAUAUGGA...UU...UAUGGCACUUUUAUUU
env-78     UUCACUUAGCCGCCGGGUUAA.G.U.G.................UUGA...................UGUGCCAUA.GAUCCAUGUC.GUUAGGCC.U.UAGAA.GAUAUGGA...UU...UAUGGCACUAUUAUUU
env-79     UUCACUUAGCCGCCGGGUUAA.G.U.G.................UUGA...................UGUGUCAUA.AGUCCAUGUC.GUUAGGCC.U.UAGAA.GAUAUGGA...UU...UAUGGCACUUUUUUUG
Lga-1-1    AUAUUCUUCCCACCGGGGAAC.A.A.A.CU..............UGUA...................AGAGCAAUU.GAUACACUUC.AUUAGGUC.U.UUGAA.GAAGUGUA...UC...AAUUGCUUUUUUUAGU
Lsp-3-1    AUAUUCUUCCCACCGGAGAAC.A.G.A.CU..............UGUA..................AGAGC.AAUU.GAUACACUUC.AUUAGGUC.U.UUGAA.GAAGUGUA...UU...GGUUUGCUUUUUUUAG
env-80     AUCAGCCCGCUGCCGAGUGGG.C.A.A.A..............................................CAGCGCAAA.UCCUUGCAUC.GUUAGGCC.A.UUGAA.GAUGCGGG...GA...UCGGCGCUGUUUUUCU
Asp-1-1    UGUUAAUUACCGCCGGGUAA...............................................CGUCU.UAUUCAUUCC.AGUAGGCC.U.UUGUU.GGAAUGAG...UA...AGGCGUUUUUUAUUCC
env-81     AUCUGUCUGCCACCGGGUAGA.C.G.G.A...............A......................AAAUGCCUG.AAUACAUUCC.GGUAGGUC.A.UGGAA.GGAAUGUA...UU...UGGGCAUUUUUUAUUU
Cba-1-1    AGCUGCCUGCCACCGGGUAUG.C.A.G.C...............GCUU...................AUGUGCCUG.AACACAUUCC.GUUAGGUC.A.AAGAA.GGAAUGUG...UC...CGGGCUUUUUUUCGGG
env-82     AUAUUACUUCCACCGGGUAGU.G.A...................AUGUU..................AAAG.CAUUCUUAACACAUC.AUCAGGUC.A.GAGAA.GAUGUGUG...AU...CAUGCUUUUUGUUCUG
env-83     GCAUCACUGCCACCGGGUAGU.A.A...................AUGUU..................AAAGCAUUU.UUUACACAUC.GUUAGGUC.A.UUGAA.GAUGUGUG...AU...AA.UGCUUUUUGUUUU
env-84     GGUCGCUUGCCACCGGGUAAG.C.G.A.C...............G......................GAAGUGUCU.GACACACUCC.GUUAGGUC.U.CAGAA.GGAGUGCG..CUC...AGGCACUUUUUCGCUU
Cba-3-1    UAGAAAUUACCGCCGGGUAAU.......................C........................AGCAUUG.AAUAUGAAUC.GGUAGGCC.A.AAGCA.GAUUCAUA...UC...CAGUGCUUUUUUUGUU
Lpa-2-1    AUUCGACUGCCACCGGGCAGC.A.A.C.UU..............C......................AGGUACUUU.UUACAAUUCC.GUUAGGUC.A.UAGAA.GGAAUUGU...GA...GGGGUACCCUUUUUCU
Lki-2-1    UAACGACUGCCACCGGGCAGC.A.A.U.UG..............U......................UGGUACUUU.UGACAAUUCC.GUUAGGUC.U.UUGAA.GGAAUUGU...GA...GGGGUACCAUUUUCUU
Lra-1-1    UAACGACUGCCACCGGGCAGU.A.A.U.UG..............U......................CAGUACUUU.UUGCAAUUCC.GUUAGGUC.U.UAGAA.GGAAUUGU...GG...GGAGUGCUAUUUUUUU
Lhe-1-1    AUACGACUGCCACCGGGUAGC.A.A.U.UU..............UUU....................AAGUACGCU.UUCCAAUUCC.GUUAGGUC.U.AAGAA.GGAAUUGA...GGA..GGGGUACUUUUUUCGU
Lbr-2-1    ACACGACUGCCACCGGGUAGC.A.A.C.UU..............UUC....................AGGUACGCU.UUCCAAUUCC.AUUAGGUC.U.AAGAA.GGAAUUGA...GGA..GGGGUACUUUUUUUGA
Ldi-1-1    AUACAACUGCCACCGGGUAGA.A.A.C.UU..............U......................GAGUACUUU.UGACAAUUCC.GUUAGGUC.U.UAGAA.GGGAUUGU...UG...GGGGUACUCUUUUUGC
Lpa-1-1    AUAUGACUGCCACCGGGUAGC.A.A...................ACACC..................AAGUACUUU.UUCCAAUCCC.AUUAGGUC.A.UUGAA.GGGAUUGU..GAA...GGGGUACUUUUUUGGU
Lco-2-1    AAUUACCUGCCGCCGGGUGGG.U.U.G.U...............A........................AGCAUGG.UUAUCAUUCC.GUUAGGUC.G.UUGUC.GGAAUGGU...AG...CGAUGCUUUAUUUUUU
env-85     AUUAUUCCGCCGCCGGGCGGA.A.C.A.G..............................................AAGCGUUUG.UUUUCGCAUC.GUUAGGCC.U.UUGAA.GAUGCGGG...GA...CGGGCGCAUGAUACGC
env-86     UAAAUUCUGCCACCGGGUAGA.A.A.U.CA..................................................GAGCAG.UGUAUGUAUC.GUUAGGUC.A.UUGAA.GAUACAUA...CAG..AUGUUCAUAUUUUUUG
env-87     AAUGAUCUGCCGCCGGGUAGA.U.C.C.U...............GUCGAUUUCA.............GGUAUCAG..GUUUCGCAUC.GUUAGGCC.U.UAGAA.GGUGCGAA...AC...GCUGGUACCUUUUUUG
env-88     UAUUGACUGCCGCCGGGCAGU.A.U.G.UA....................................UGACUUC....GGUCCGCACC.GUUAGGCC.U.UAGAA.GGUGCGGG...UU...GCCGGAGUUUUAUUUU
env-89     AACAUCUUGCCGCCGGGUAAG.C.A.G.GU..............AC.....................AACAUCAUG.AUUUUCUGUC.GUUAGGCC.U.UAGAA.GACAGGAG...GU...UUUGAUGUUUUUUUGU
[ae-1-1    GAUACGUUGCCGCCGGGCAAC.A.A.A.AU..............G......................AACAUUCAA.UUAUCAUGUC.GUUAGGCC.A.UAGAA.GACAUGAG...AU...UUGGGUGUUUUUUUCA
env-90     AAAAGAUUGCCGCCGGGCAAU.A.A.A.GU..............U......................AGCAUUCAG.AUAUCAUGUC.GUUAGGCC.A.UUGAA.GACAUGAG...GU...UUGAGUGUUUUUUUCA
env-91     AAUAUAUUGCCGCCGGGCAAU.A.A.A.................GCA....................AGCAUUCAG.AUAUCAUGUC.GUUAGGCC.A.AUGAA.GACAUGAG...GU...UUGGGUGUUUUUUCAU
Rma-1-1    AAUUAUCUGCCGCCGGGUAGA.U.C.A.................AGAAACA................AAGCAUGUG.CGCCCCUAUC.GUUAGGCC.U.GAGAA.GAUAGGGG...AGAG.CGCAUGCUUUUUUGUU
env-92     AUCAGACUGCCGCCGGGCAGU.A.G.U.GU..............U......................GAGCACGGU.CCACCGCAUC.GUUAGGCC.U.UAGAA.GAUGCGGC...GCA..GCCGGGCUUGUUUUUU
env-93     AAUAAAUCGCCGCCGGGUGA.............................................GCGCGUC.AAAUUGCAUC.GUUAGGCC.U.UUGAA.GAUGCAAC...GU...GACGCUUUUUUAUUGG
env-94     AAAUAAUCACCGCCGGGUGAG.U.........................................GCGUC.AAAUUGUAUC.GUUAGGCC.U.UUGAA.GAUGCAAA...GU...GACGCUUUUUUAUUGG
Msp-2-1    UUCAGAUUACCGCCGGGUA..............................................CGUUC.AGACUAUUCC.AGUAGGCC.U.UAGCA.GGAAUAGU...GU...GGACGUGCCCUUUUUU
env-95     UUGACACUGCCACCGGGUAGA.G.A.U.G...............UUAUUGAUUGAUG..........AAGC.AUCU.GCACCGUAUC.UUUAGGUC.U.UAGUA.GAUACGGU...AC...AGAU.GCUUUUUUGUU
Lcu-2-1    UAAUCGUUGCCACCGGGCAGU...................................................UAAUCACUUC.AUUAGGUC.A.AAGAA.GAAGUGAU...UG...GCUGUCCUUUUUUUGU
Lsa-1-1    UAAUCAUUGCCACCGGGCAGU...................................................UAAUCACUUC.UAUAGGUC.A.UAGAA.GAAGUGAU...UG...GCUGCCCUUUUUUUAU
env-96     AAUAUAAUUCCACCGGGAAUA.A.....................CGCU...................CAAGCGUUU.UAAGCAUUUC.AUUAGGUC.U.AAGAA.GAAAUGCU...UA...ACUCACUUGAUUUUUU
Lga-4-1    AAUAUAAUUCCACCGGGAACA.A.....................CGCU...................CAAGCGUUU.UAAGCACUUC.AUUAGGUC.A.UAGAA.GAAGUGUU...UG...AAUGACUUGUUUUUUU
env-97     UUUUCUUUAUCGCCGGAUAAG.G.A.A.A...............UUAUAA.................AAGCGUUCG.GAUUUCAUAC.UGUAGGCC.U.UUGAAGGUAUGAAA.UAUC...CGGACGCUUUUUAUUU
Lsp-6-1    UACAUUUUACCGCCGGGUAA..............................................CGUCUU.AU.UUAUUUC.AGUAGGCC.U.UUGUU.GAAAUAAU...AU...AAGACGUUUUUUUUGG
env-98     AAACAGAUACCGCCGGGUAUC.......................AU.......................GCCGCUG.UUGCCGCUCC.GUUAGGCC.U.UUGAA.GGAGUGGC...UG...CGGCGGUUUUUAUUUU
env-99     AAAAAUCUGCCACCGGGUAGU.G.C.G.U...............G......................AAAUGCAUU.UGUGUAUGUC.GUUAGGUC.U.UAGAA.GAUGUACA...UG...AAUGCAUUUUUUUGGA
env-100    ACAAAUCAGCCACCGGGUUGA.G.A.U.G...............C......................AAAAGCAUC.ACGGCAUAUC.GUUAGGUC.U.UUGAA.GAUAUGCA...GU...GAUGCUUUUUUUGAAA
env-101    UCAAAUCUGCCACCGGGUAGA.G.A.A.UG..............CAAUUA.................UGAAGCAUU.CGCGCGUAUC.GUUAGGUC.U.UUGAA.GAUACGCA...CG...GGUGCUUUUUUGUUUG
env-102    AUAAAUCUGCCACCGGGUAGA.......................GAAUGCAUAA.............UGAAGCAUU.CGCGCGUUUC.GUUAGGUC.U.UUGAA.GAAACGCA...CG...GGUGCUUUUUUGUUUG
env-103    GAAUACCUGCCACCGGGCAGU.G.G.A.U...............GC.....................GAAAGCAUC.CUGGCACAUC.GUUAGGUC.U.UUGAA.GAUGUGCC...GG...GAUGCUUUUUUGUUUC
env-104    UAUUAACUGCCACCGGGUAGU.A.A.A.A...............UGC....................GCAAGCAUC.CGUGCAUAUC.GUUAGGUC.U.UUGAA.GAUAUGUA.CAUG...GAUGCUUAUUUUAUUU
env-105    AAGAAUCUGCCACCGGGUAGA.A.A.U.................GCA....................AAAAGCAUU.UGCGCACAUC.GUUAGGUC.U.UGGAA.GAUGUGCU.UGCA...AAUGCUUUCUUUUUUU
env-106    UACAAACUGCCACCGGGUAGU.A.U.............................................AGCAUU.CCCGCACAUC.GUUAGGUC.U.UGGAA.GAUGUGCC...GG...GGUGCUUAUUUUUUGG
env-107    AUAAAUCUUCCACCGGGAAGA.G.A.U.G...............C......................AAGAGCAUC.UUAAUGUAUC.GUUAGGUC.U.UUGAA.GAUACAUU..UAA...GAUGCUUUUUCUUUAG
env-108    AAAUUGCUGCCACCGGGUAGU.C.G.U.................GCGU...................AAGAGCACU.UGUUCGCAUC.GUUAGGUC.U.UAGAA.GAUGCGCG...CA...GGCGCUCAUUUUUUUG
env-109    UAUUGCUUGCCGCCGGGUAAG.A.A.A.U...............C.......................GCAGCGCA.GAUCCAUUUC.GUUAGGCC.U.UAGAA.GAGAUGGA.UAUC...GGCGCUUUUGUUUUAU
env-110    AUUGACUUGCCGCCGGGUAAG.U.A.A.G...............A......................AGCAGCGCA.GAUCCAUUUC.GUUAGGCC.U.UUGAA.GAUUUGGA..UAC....CGGC.GCUUUUAUUA
Lsp-9-1    AGAAAUUUGCCACCGGGUAAA.U.U.A.C...............A.........................GCGCUG.UGACUGUGUC.GUUAGGUC.U.UAGAA.GACACAGC...UC...CAGCGCUUUUCUUUUG
env-111    GUUUAUUUACCGCCGGGUAAA.U.A.U.A...................................AAGCGUUG.AAUCUGUAUC.GAUAGGCC.A.UAGAA.GAUACAGA...UU...UGACGCUUUUCUUUUU
Lsp-8-1    AUUCAUUUACCGCCGGGUAAA.U.G.U.A...................................AAGCGUUG.AAUCUGUAUC.GGCAGGCC.G.UAGGA.GAUACAGG...UU...UGGCGCUUUUUUGUUU
env-112    GUUCAUUUACCGCCGGGUAAA.U.A.U.....................................AAAGCGUUA.AAUCUGUAUC.GGCAGGCC.A.UAGGA.GAUACAGA...UU...UGGCGCUUUAUUUUUU
Csp-12-1   GAAAAUUUACCGCCGGGUAAA.U.....................AAAA......................GCGUUG.AUCCUGUAUC.AUUAGGCC.A.UAGAA.GAUACAGG...UU...CAGCGCUUUUCUUUUG
env-113    UCAUCCCCGCCGCCGGGCGGG.G.C.G.AA.....................................GCGUUGG.CUUCCGCAUC.GUUAGGCC.A.UUGAA.GAUGCAGG...GG...CGGGCGCAUUUUUUAU
env-114    AACGACCCGCCGCCGGGCGGG.U.....................G.......................AGCGCUGG.CCCCUGCGUC.GUUAGGCC.U.UUGAA.GACGCGGG...CGA..CCGGCGCUUUUUGUAU
env-115    CAGUCACCGCCGCCGGGCGG..G.A.C.UG.....................................GGCGUUU.GCCGUGCAUC.AUUAGGCC.A.UAGAA.GAUGCACA...GC...AGGCGCGUUUUUUUAU
env-116    CAGUCACCGCCGCCGGGCGG..G.A.C.UG..............G.........................GCGUUU.GCCGUGCAUC.AUUAGGCC.A.UAGAA.GAUGCACA...GC...AGGCGCGAUUUUUUUA
env-117    UGGAUCCCGCCGCCGGGUGGG.......................CC.....................AAUGCGUUU.UUUCUGCAUC.GUUAGGCC.A.UUGAA.GAUGCAGA...GG...GAGCGCUAUUUUUGUU
env-118    CAGGAUUCGCCGCCGGGUGAG.U.U.U.................GC........................GCGUUU.UUCCUGCAUC.GUUAGGCC.A.UUGAA.GAUGCAGG...AG...GGGCGCUAUUUUUGUU
env-119    CAGGAUUCGCCGCCGGGUGAG.U.U.U.................G........................CGCGUUU.UUCCUGCAUC.GUUAGGCC.A.UUGAA.GAUGCAGA...GA...GGGCGCGAUUUUUGUU
env-120    GUGGGUUCACCGCCGGGUGAG.C.C...................AU.....................AAGCGCUUU.UUGCCGUAUC.GUUAGGCC.U.UUGAA.GAUGCGGC...GA...GAGGCGCAUUUUGCAU
env-121    CAUGGUUCGCCGCCGGGUGAA.U.C.G.................C......................AAGCGCUUU.UUCCUGCAUC.GUUAGGCC.U.UUGAA.GAUGCAGG...AA...AGGGCGCAUUUUGUUU
env-122    ACAGGCUCGCCGCCGGGUGAG.C.C...................GCAA.....................GCGCUUU.UUCCUGCAUC.GUUAGGCC.U.UUGAA.GAUGCGGG...GG...AGGGCGCAUUUCGUUU
env-123    UCAGACACGCCGCCGGGUGCG.U.C...................AA......................CGCGCUUU.UUUCUGCAUC.GUUAGGCC.U.UUGAA.GAUGCGGA...AG...AGGGCGCGAUUUUGCG
env-124    AAUCGUCUGCCGCCGGGUGGA.C.....................UU......................AAGGCAUU.GUUUCGCAUC.GUUAGGCC.A.UAGUA.GAUGCGAA...AAC..GGUGCUUUUAUUUUUU
env-125    UAUUCUCUGCCGCCGGGUAGA.G.A.GU................UUA....................GGCGUCAG..GUUUCAUAUC.AUUAGGCC.A.UAGAA.GAUAUGAA...AU...GCUGACGUUAUUUUUU
env-126    AAAACUUUACCGCCGGGUAUU.A...........................................CCUGU.C.AGCAUUCC.AUUAGGCC.A.UAGAA.GGAGUGUU...UG...AUGGGGAUUUUUUUUG
env-127    AAAUAACUGCCACCGGGUAGU.A.G...................CAAUGCUCA..............GGCAUUUUC.CGUUUAUACC.GUUAGGUC.U.UUGAA.GGUAUAA....CA...GAAAGUGCUAUUUUUU
env-128    ACGGAUCUGCCGCCGGGCAGA.U.U...................AA.....................AAACGCCGC.AGUUCGUAUC.GUUAGGCC.G.UAGAA.GAUACGAA...UC...GCGGCGUCUAUUCUUU

           ...........<<<..............................................................................>>>..........................................
           ...........20...............................................................................02...........................................
           <<<<<<<<<<<....>>>>>>.>.>.>.>>.....<<<<<<<<.............>>>>>>>>...<<<<<<<<<.<<<<<<<<<<..................>>>>>>>>...>>...>>>>>>>>>.......
           22.22222222....222222.2.2...22.....1111110..............01111111....22222222.2222222221..................12222222...22...22222222........
           ●●●●●●YUGCCRCCGGGUAR●-○-○-○-○--------------○○○--------------------○○R●●●Y●●-●●●●YRY●YC-RUUAGGYC-●-U●GAA-GR●RYR●●---●●---●●R●●●YYUUUUUY●●
```

```
env-129  UUGUUUCUGCCGCCGGGUAGA.A.G.U.GG.......................................AAGGUCAG.AACCUGUAUC.GUUAGGCC.A.UAGAA.GAUACAGG...GA...CUGGCGUUUUUUUGUU
env-130  UUUAUUUUACCGCCGGGUAAU.A.U.A.AA......................................ACCGCCAGA..CUUUCUAUC.GAUAGGCC.U.UUGAA.GAUAGAAA...GA...UCUGGCUGUUUUUAUU
env-131  UUGUAACUGCCACCGGGUAGU.G.A.A.UG..............UAA.....................UAACAUUCG.UUUCCGUAUC.AUCAGGUC.U.CAGAA.GAUACGGG...GA...CGAAUUUUUCUUUUAU
env-132  UAUCAGUUGCCACCGGGUAAC.A.G...................CGUU....................UAACGCAUG.GUUCUGUAUC.GUUAGGUC.U.UUGAA.GAUACAGA...AAU..CAGGCGUUUUUCUUUU
env-133  GAUGACUUGCCGCCGGGUAAG.......................CAUUU...................GAGCGCA...GAUCUGUAUC.GUUAGGUC.U.UAGAA.GAUACAGA...UA...CGGGCGCUUUUGUUUU
env-134  CAGACUUUACCGCCGGGUAAA.G.U.U.U...............CACACC.................AGCGUUCAG.AUUUCAUACC.AUUAGGCC.A.UUGAA.GGUAUGAU...AUU..CUGGACUUUUUUUAUA
env-135  UGAUAUCUGCCGCCGGGUAGA.......................AUGAA...................AUGCACGAC.GCCUCGUAUC.GUUAGGCC.U.UUGAA.GAUACGAU...GA...GCAGCGUGUGAUUUUU
env-136  GAGGAUUUGCCGCCGGGCAAU.U.............................................GUCUUU.GAUUCACUCC.AUUAGGCC.A.AUGAA.GGAGUGAG...GA...GAAGCCUUUUUUUAUG
env-137  UAUACUUUACCGCCGGGUAA........................UCC.......................UGUCUUA.GCUUCAUUCC.GUUAGGCC.U.UUGUA.GGAAUGGG...AC...UAAGAUUUUUUUAUUA
env-138  AAUAGUUUACCGCCGGGUAAC.U.U...................U.........................GUCUUA.GAUCCAUUCC.GUUAGGCC...UUGAA.GGAAUGUG...UC...UAGGACUUUUUUAUUA
env-139  AAAUAUUUGCCGCCGGGUAAG.................................................CAUCUUU.UAAAUAUUCC.AUUAGGCC.U.UUGAG.GGAAUAUU...CA...AAAGAUGCUUUUUGUA
env-140  AAUAAGCUGCCGCCGGGUAGC.U.....................GCAAA.....................ACGCCGG.AAUUCAUGUC.GUUAGGCC.U.UAGAA.GACAUGGA...UU...CCGGCGUGAUUCUUAU
[am-1-1  CAUAAACUGCCGCCGGGUAG........................CAAAACG.................CCACGUCAG.AUUUCAUGUC.AUUAGGCC.G.UAGAA.GACAUGAA..AGG...CUGGCGCUUUUUCAAU
env-141  GCUUCGCUGCCGCCGGGCAGC.......................CA......................AAACGCCAG.AACUCACGUC.GUUAGGCC.U.UUGAA.GACGUGAG...AU...CCGGCGUUUUUAUUUU
env-142  UACUGUUUAACGCCGGUUAAG.C.A.G.................GU........................GCCUUUA.UUUAUGUAUC..GUAGGCC.U.UAGAA.GAUGCAUA...AA...UAAGGGCAUUUUUUAU
env-143  AUCUGUUUAACGCCGGUUAAG.C.A.G.GU..........................................GCCUUUA.UUUAUGUAUC..GCAGGCC.U.UAGAA.GAUACAUA...AA...UAAGGGCAUUUUUUGU
env-144  AAUUUUUUGCCGCCGGGUAAA.C.G...................AA......................GCGUUGA...AUUUUGUAUC.GACAGGCC.U.UUGAA.GAUACAAA...AA...GCUCAGCGCUUUUUUC
env-145  GAAAAGCUGCCACCGGGUAGC.U.G.U.................GCAA....................AAGCACGUA.AAACCACAUC.GUUAGGUC.U.UAGAA.GAUGUGGC...UU....ACGUGCUUUUAUAUU
env-146  AUACUGUUGCCACCGGGUAAC.G.G.A.A...............CGUA....................AAACGUCCA.UAUGCUUAUC.GUUAGGUC.U.UUGAA.GAUACAUA...CA.....GAUGCUCAUUUUUU
env-147  UUUAUAAUGCCACCGGGUAUU.A.U.G.CA..............UGCAUU..................AAAGCAUUG.UUACUACAUC.AUUAGGUC.U.UUGAA.GAUGUCGU...AAG..GGAUGUUUUUUUUUAU
env-148  UUUUAUCCGCCGCCGGGUGGA.U.....................UUUU......................GCGUUGG.UUUUCGUAUC.GUCAGGCC.U.UAGAA.GAUACGGG...UU...CCGACGCUUUUUGUUU
env-149  AUAUUGCUGCCACCGGGCAGC.G.G.U.G...............CGGUUAAAAAC.............AAGCACCC...GAACACAUC.GUUAGGUC.U.UAGAA.GAUGUGUU...CC....GGGUGCUUUUUCGCA
env-150  AAUUAACUGCCACCGGGUAGU.A.G.C.GU..............A.......................UAACGCUGG.AGUUCAUAUC.GUUAGAUC.U.UUGAA.GAUAUGAA..GUU...CCGGCGAUUUUUUUGU
env-151  CGCCUAUUGCCACCGGGUAAU.A.G.G.................A.......................AAGACGUCA.UUUCUUUAUC.GUUAGGUC.U.UAGAA.GAUAAAGA...AG...UGGCGUAUUUUUUUUA
env-152  CGCUCGUUGCCACCGGGUAAC.G.A.G.GG.......................................AGGCGUCA.UUUCUUUAUC.GUUAGGUC.U.UAGAA.GAUAAAGA...AA...UGGCGUUAUUUUUGGG
Exy-1-1  CGCUCUUUGCCACCGGGUAAA.G.G.G.AG.......................................AAACGUCA.UUUUCUUAUC.GUUAGGUC.U.UUGAA.GAUAAGGA...AA...UGGCGAUUUAUAUUUU
env-153  CACCUAUUGCCACCGGGUAAU.G.G.G.AG..............A.........................AGCGUCA.UUUUCUUAUC.GUUAGGUC.U.UGGCA.GAUAAGAA...GA...UGGCGCUGUUUUUUUG
env-154  CACCUGUUGCCACCGGGUAAU.A.G.G.AG......................................AAAGCGUCA.UUUUCUUAUC.GGUAGGUC.U.UGGCA.GAUAAGAA...AA...UGGCGCUGUUUUUGUA
Sru-1-1  CACCUGUUGCCACCGGGUAAU.G.G.G.................A.......................AAAGCGUCA.UUUUCUUAUC.GUUAGGUC.A.UGGAA.GAUAAGAA...AA...UGACGCUGUUUUUGUA
env-155  AUAGCGCUGCCGCCGGGCAGC.A.A.A.AU..............GCA.....................AUGCAUUUG.CCAAUACAUC.GUUAGGCC.U.UUGAA.GAUGUAUC...GG...UAAAUGCAUUUUCUAU
env-156  AAUCUGUAACCGCCGGGUUU.................................................GCGUCUUA.UCCUCACUCC.GUUAGGCC.A.UAGAA.GGAGUGGA...GA...CAAGACGUUUUUAUUU
env-157  AAAAUGCUGCCACCGGGUAGU.U.G.U..................AAUGCAUAA..............AGCAUUCG..UUUACAUAUC.GUUAGGUC.U.UUGAA.GAUAUGUA...UG...GCGGAUGCUUCUUU..
env-158  CAAUCGUUGCCACCGGGUAAC.G.A...................AAAAUGCUUAAA............GAGCAUUCG.U.AACGCGUC.GUCAGGUC.U.UAGAA.GACGCG.G...AA...CGAGUGCUUUUUUUAC
Mfa-1-1  GUUUAUUUACCGCCGGGUAAU.C.G...........................................AAGCGCCAU.GUUCUGUAUC..GUAGGCC.A.UUGAA.GAUACAGC...UC....UGACGCUUUUUCUUA
env-159  UAUUUCCUGCCGCCGGGUAGG.A.A.A.U...............C.......................AGGCGUCGG.AUUCUCUGUC.GUUAGGCC.A.UUGAA.GUCAGAGA...AU...UUGACGCCUCUUUGCU
env-160  AAUAAUUUACCGCCGGGUAAA..................................................CGCCUU.GUACAGUUCC.GUUAGGCC.U.UAGAA.GGAACAGU...AC...CGGGCGUUUUUAUUUU
env-161  AUAAUUUUGCCGCCGGGUGAA.A.U.U.UU..............UUAUA...................AGCGUUAGG.UUCUCAUACC.AUUAGGCC.A.UAGAA.GGUAUGAG...GG...GCUAACGCUUAUUUUA
Bsp-1-2  AUAAUAAUGCCGCCGGGUA.................................................AGCCUUAU..UUCUCAUUCC.AUUAGGCC.G.UAGCA.GGAAUGAU...GA...GAUAAGGCUUUUUUGU
env-162  UCUACUUUGCCGCCGGGCAAA.G.U.G.................UUGAUU..................AAGCGUUUUUGUUUCAUACC.GAUAGGCC.U.UAGAA.GGUAUGAA..GCU...AAAGCGCUUUUUAUUU
env-163  AAUUUAUUACUGCCGAGUAA..C.G....................C......................
....CUUGU.AU.CUAUUCC.GUUAGGCC.A.UAGAA.GGAAUAGU...GU...ACGGGUGUUUUUAUUU
env-164  AUUCAAUUACCGCCGGGUAAU.A.A.G.G...............CGCAU...................UUGCGUCGG.AAUUCAUGUC.AUUAGGCC.A.UAGAA.GACAUGAA...UC...CCGACGCUUUUUAUUU
env-165  ACUUAUUUGCCGCUGGGUAAA.................................................CAUCUUA.UAUUCGUUUC.GUUAGGCC.U.UAGAA.GAAACGUU...UG...UGAGGUGUUAUUUUUU
env-166  UCGAAUUUGCCGCCGGGUAAA.......................C.......................
.GUC.UUUG.UGUCCAUUCC.AUUAGGCC.U.UUGAA.GGAAUGAG...CG...CGGGACGUUUUGUUGC
env-167  UUUUCAUUGCCGCCGGGUAA..................................................CGCUGAU.UC.CCAUUCC.GUUAGGCC.A.UAGAA.GGAAUGGA...GG...AGCAGCGUUUUUUAUU
Epl-2-1  GAUUUUUUACCGCCGGGUAA..................................................CGCUGAU.UC.CCAUUCC.GUUAGGCC.A.UAGAA.GGAAUGGA...GA...AGCAGCGUUUUUAUUU
env-168  AAAUAUUUGCCGCCGGGUAAA..................................................UGUUGU..AUACCAUUCC.AUUAGGCC.U.UUGAA.GGAGUGGU...AU...UACGACAUUUUUCGCU
env-169  AAAUAAUUACCGCCGGGUAA..................................................CG.UCUC.ACCCCAUUCC.GUUAGGCC.A.UAGAA.GGAAUGGU..GGU....AGGGCGUUUAUUUUU
env-170  UGAAUUUUGCCGCCGGGCAAA.................................................CGCCUUA.ACACCAUUCC.AUCAGGCC.U.UAGAA.GGAAUGGC...GU...UAGGGCGUUUUUUAUU
env-171  AAAAAAUUGCCGCCGGGCAA.................................................ACGCCUUA.AUACCAUUCC.AUCAGGCC.U.UAGAA.GGAAUGGC...GU...UAGGGCGUUUUUUGUU
env-172  GACAAUUUGCCGCCGGGCAAA.................................................CGCCUUA.AUACCAUUCC.AUCAGGCC.U.UAGAA.GGAAUGGC...GU...UAGGGCGUUUUUUGUU
env-173  UAUUAAUUACCGCCGGGUAAA.C.G..............................................UCUUG.AUACCAUUCC.AUUAGGCC.U.UAGAA.GGAAUGGU...GU...UAAGGCGUUUUUUGUU
env-174  AAAUAUUUACCGCCGGGUAA..................................................CGUCUUG.C..CCAUUCC.GUUAGGCC.A.CAGAA.GGAAUGGA...GG...CAGAGGCGUUUAUUUU
env-175  AAUGAAUUACCGCCGGGUAA..................................................CGCUUUU.UAUUCACGCC.GUUAGGCC.U.UAGAA.GGAGUGAA...UG...GGAGGCGGUAUUUUUU
env-176  UGGAAUUUGCCGCCGGGCAG........................CC.........................GCCUCU.CAUCCAUUCC.GUUAGGCC.G.UUGAA.GGAAUGGG...UG...GCGGGCUUUUUUUAUU
env-177  GUUAUUUUGCCGCCGGGCAAA.G.U.U.UU..............AAAUA..................AAGCGUUUUU.GUUUCAUACC.GUUAGGCC.U.UAGAA.GGUAUGAA...GCU..AAAAGCGCUUUUUAUU
env-178  ....AUCAGCCGCCGGGC..................................................AGCGUCUG..UUUCCAUUCC.GUUAGGCC.U.UAGAA.GGGAUGGA...UG...ACAGGCGUUUAUUUU.
env-179  UAUUGUCAGCCGCCGGGC..................................................AGCGUCUGU.U.UCCAUUCC.GUUAGGCC.A.UAGAA.GGAAUGGA...CA...ACAGGCGUUUAUUUUU
env-180  CAAUAUCUGCCGCCGGGCAG...................................................CACCUG..UUCCCAUUCC.GUUAGGCC.U.UAGAA.GGAGUGGG...UG...GCAGGUGUUUAUUUUU
Cbo-2-2  UGAUUUUUGCCGCCGGGUAAA.A.A.U.................GCUUUCACA...............AACAUUCGU.AUUUCAUACC.AUUAGGCC.A.UUGAA.GGUAUGAA..AAU...UCGAAUGUUUUUUUAU
env-181  UACAAAUUGCCGCCGGGUAAG.C.U..............................................CGCUUU.ACCUCAUUCC.GUUAGGCC.U.UAGAA.GGAAUGAG..CGU...AAGGCGUU.UUUUUUG
env-182  GGGUAAUUGCCGCCGGGUAA..................................................CGCGUUA.C.CCCAUUCC.GUUAGGCC.U.UAGAA.GGAAUGGG...AG...UGAGGCGUUUUUUUGU
env-183  UUUGUUUUGCCGCCGGGUAAA.G.C.A.A...............UUAACA..................AGCAUUCGA.GUUUCACACC.GUUAGGCC.U.UUGAA.GGUGUGAG...AG...UCGAAUGUUUUUGUUU
env-184  UAUUCUUUGCCGCCGGGCAGA.G.A.U.AA..............CUUAUA..................AGCAUUCGG.AUUUCAUACC.AUUAGGCC.U.UUGAA.GGUAUGAA...AG...UCGGGUGAUUUUUUAU
env-185  UUGCAUUUGCCGCCGGGUAAA.U.G.C.AA..............UUAAAA..................AGCAUUCGG.AUUUCAUACC.GUUAGGCC.U.UUGAA.GGUAUGAA...AG...UCGGGUGUUUUUUUAU
env-186  UUAAAAUUACCGCCGGGUAUC.................................................UGCCUUU.CACUCAUUCC.AUUAGGCC.A.UUGAA.GGAAUGAG...UG...AAAGGCGAUUUUUAUU
env-187  AUUGUUUUACCGCCGGGUAA..................................................CGUCUAU.GAUUCUCUCC.GUUAGGCC.A.UUGAA.GGAGG.AA...CU...AUAGGCGUUUUAUUUU
env-188  ACUAUUUUGCCGCCGGGUAAA.................................................CGCUUG..UUUUCGCUCC.AUUAGGCC.A.UAGAA.GGAGUGGA...AA...AUGAGUGUUUUUUUUA
env-189  GUAAGUUUACCGCCGGGUGA........................C.........................CGUCUUG.CAUUCAUUCC.AUUAGGCC.A.UUGAA.GGGAUGAG...UG...UUAGACGUUUUUUAUG
env-190  AUAAAUUUACCGCCGGGUAAA.................................................CGCUUG..UUUUCAUUCC.GUUAGGCC.A.UAGAA.GGAGUGAA...AA...GCAAGCGUUUUAUUUU
bMS-1-1  AUGAAAUUGCCGCCGGGUAAA.................................................CGUCUUU.UGUUCAUUCC.AUUAGGCC.C.UUGAA.GGAAUGAA...GA...UCAGGCGUUUUAUUGU
env-191  UUGAAUUUGCCGCCGGGUAAA.................................................CGUCUUU.CAUUCAUUCC.GUUAGGCC.A.UUGAA.GGAAUGAG...UG...UCAGGCGUUUUAUUUU
env-192  UUGUAUUUGCCGCCGGGUAAA.................................................CGUCUUU.UAUUCAUUCC.GUUAGGCC.A.UAGAA.GGAAUGAA...UA...AAAGGCGUUUUUCGUU
env-193  AUAAAUUUACCGCCGGGUAAA.................................................CGUCUCA.UAUUCAUUCC.AUUAGGCC.A.UUGAA.GGAAUGAA...UG...UCAGGCGUUUUAUUUU
Pvi-1-1  UUAAAUUUGCCGCCGGGUAAA.................................................CGUCUUU.UAUUCAUUCC.GUUAGGCC.U.UUGAA.GGAAUGAA...UG...UGAGGCGUUUUAUUAU
env-194  UUGAAUUUACCGCCGGGUAG.................................................UCGUUUUA.CAUCCAUUCC.AUUAGGCC.A.AUGAA.GGAAUGAG...UG...UUAGACGACUUUUUUU
env-195  UUGUAUCUGCCGCCGGGUAGA.................................................CAUCUUC.UGCUCAUUCC.AUUAGGCC.A.UUGAA.GGAAUGAA...CU...GAAGAUGUUUUUUAUA
env-196  AUCAUAUUACCGCCGGGUAA..A.......................CG.....................
....UCUU..UGUUCAUUCC..GUAGGCC.U.AUGAA.GGAAUGAA...UG...ACAGGGCGUUUUAUUU
env-197  AGGUAAUUACCGCCGGGUAA...................................................CGUCUG.CUUUCAUUCC.GUCAGGCC.A.UAGCA.GGAAUGGA...GC...CAGACGAUCUUUAUUU
env-198  GAUGAUGUACCGCCGGGUAGA.U.G.A.UU..............A.......................UAGCGUCCU.AAUCCAUUCC.GUUAGGCC.U.UAGAA.GGAAUGGA...GC...GGGGCGCUUUUGUUGU
env-199  GAUAUGUUUCUGCCGAGAAAC.G..............................................
.....CUUA.UUCUUACUCC.AGUAGGCC.A.UAGAA.GGAGUAAA...AA...UAAGAGUCUAUUUUUU
env-200  AAUACUUUGCCGCCGGGCAAA..................................................CGCUUG.UUCCCGCUCC.GUUAGGCC.G.UAGAA.GGAGUGGG...GA...UAAGCGUUUUUUACGU
env-201  ACAGAAUUUCCGCCGGGAAAC.G.C..............................................
...UUA.UUUUUACUCC.GUUAGGCC.A.UUGCA.GGAGUAAG...GA...UAAGCGUUUUUUGUUU

         ...........<<<...............................................................>>>..........................................
         ...........20................................................................02...........................................
         <<<<<<<<<<<....>>>>>>.>.>.>.>>......<<<<<<<<.............>>>>>>>>...<<<<<<<<<.<<<<<<<<<<..................>>>>>>>>...>>...>>>>>>>>>.......
         22.22222222....222222.2.2...22......1111110..............0111111...22222222.2222222221..................12222222...22...22222222........
         ●●●●●●YUGCCRCCGGGUAR●-○-○-○-○--------------○○○--------------------○○R●●●Y●●-●●●●YRY●YC-RUUAGGYC-●-U●GAA-GR●RYR●●---●●---●●R●●●YYUUUUUY●●
```

```
env-202  AAUAAAUUUCCGCCGGGAAA..................................................AUGUUUA.UUUUUGCUCC.GUUAGGCC.A.UUGCA.GGAGUAAA...AA...UAAGCGUUUAUUUUUU
env-203  AAUAAAUUUCCGCCGGGAAA...................................................CGCUUA.UUUUUGCUCC.GUUAGGCC.A.UUGCA.GGAGUAAA...AA...UAAGCGUUUUGUUUUU
env-204  AAUACAUUUCCGCCGGGAAA..................................................ACGCUUA.UUUUUGCUCC.GUUAGGCC.A.UUGCA.GGAGUAAA...CA...UAAGUGUUUAUUUUUU
env-205  AAUGAAUUUCCGCCGGGAAA..................................................ACGCUUA.UUUUUGCUCC.GUUAGGCC.G.UUGAA.GGAGUAAA...AA...UAGGCGUUUAUUUUUU
env-206  AAGAAUUUGCCGCCGGGUAAA..................................................CGCUUA.UUAUCACUCC.AUUAGGCC.U.UAGAA.GGAGUGAC...AA...UAGGCGUUUUUUGUUU
env-207  GAAUGUUUGCCGCCGGGUAA...................................................CGCCUG..AUCCAUUCC.GAUAGGCC.U.UAGAA.GGAAUGGA...GC...CAGGCGUUUUUUUGUG
env-208  AACGAAUUGCCGCCGGGUAA...................................................CGCCUU..UUUCACUCC.GAUAGGCC.U.UAGAA.GGAGUGAA...GC...AGGGCGUUUAUUUUUU
Bsp-2-1  AAAUGAUUGCCGCCGGGUAA...................................................CGCCUU..AUUCAUUCC.GAUAGGCC.U.UAGAA.GGGAUGAA...UC...AAGGCGUUCUUUCUUU
env-209  AAUCAUUUGCCGCCGGGUAAA.U.............................................AAACGUCUU.GGCUCAUUCC.GUUAGGCC.U.UAGAA.GGAAUGAG.UUCG...AAGACGUUUUUUUGUA
env-210  AGUAAAUUACCGCCGGGUAA.................................................ACGUCUUA.UACUUGUUCC.GUCAGGCC.A.UAGAA.GGAACGAG...CG...UAGGGCGUUUUUAUUU
env-211  AAUAUUUUACCACCGGGUAAU.G................................................CCUUAU.UAUUCAUUCC.GUUAGGUC.U.UUGAA.GGAGUGAA..UCA...AUAAGGCAUUUAUUUU
env-212  AUAAUUUUGCCACCGGGCAG.................................................UGCCUUAU.UUUUCAUUCC.GUUAGGUC.U.UUGAA.GGAGUGAA...UAG..AUAAGGCAUUUUAUUU
Ssp-2-1  AACGCAUUGCCGCCGGGUAAU.G.C.G.GU..............UCUA....................AACGCGCUG.GACUCACGCC.GUCAGGCC.U.UAGAA.GGGGUGAG...UCUCGGCGCG.GUUUUUUAUU
env-213  AAUAUACUGCCACCGGGUAGA.A.A.A.................AGUGCAAA................CGGCACCUU..AUACACAUC.GUUAGGUC.U.UUGAA.GAUGUGUA...UU...AAGGUGUUUUUUUAAU
env-214  AUUUAUUUGCUGCCGAGUAAA.U.A.A.A.......................................AUGCAUCUU.UCAACGUAUC.GUUAGGCC.U.UUGCA.GAUACGUU...GC...AAGGUGCAUUUUUUAG
env-215  GUGAUACUGCCACCGGGCAGG.A.A.U.GU..............UU......................CAGGCAUUC.GUUCCGCAUC.GUUAGGUC.U.UUGAA.GAUGCGGA..GGC...GGAUGCCUUUUUUGAA
env-216  UACAUUUUGCCACCGGGUAAA.U.A.A.UG..............CAA.....................AAGCAUUUA.UGAUCGCUUC.GAUAGGUC.U.UUGAA.GAAGCGUU...CA...UAGGUGCUUUUUUUAU
env-217  GAAAAGCCGCCACCGGGUGGC.A.A.U.AU..............GUUGAAAAU...............CAGCAUUCG.UUUGCGCUUC.GUUAGGUC.U.UUGAA.AAAGCGCA...GG...UGAAUGCUUUUUUGAU
env-218  UAUCAUCUGCCACCGGGUAGA.A.U.U.AA..............AAAAUGCAGAA.............AAGCAUUUG.UCUGUGCGCC.GUUAGGUC.U.UUGAA.GGCGUACA...AG...CAAAUGCUUUUUGUUA
env-219  UAGUAACUGCCACCGGGUAGU.......................GUGCG...................UAAGCAUAU.UGUACAUAUC.AUUAGGUC.U.UAGAA.GAUAUGUA...GA...GUAUGCUUUUUCUUUU
env-220  UAGGUACUGCCACCGGGUAGU.A.A.A.GA..............UACUGAAA................AAGCAUCUG.UAAAUGUAUC.GUUAGGUC.U.UAGAA.GAUACAUU...GA...CAGGUGCUUUGUUUUU
env-221  UUCAAUCUGCCACCGGGUAGA.......................GAUGCG..................AAAGCACCG.UCAGUACAUC.GUUAGGUC.U.UUGAA.GAUGUACC...UG...CGGUGCUUUUUCGGUA
env-222  AUAAUUCUGCCACCGGGUAGA.G.A.U.G...............CGU.....................AAAGCACCG.U.GGUACAUC.GUUAGGUC.U.UUGUA.GAUGUACU...CA...CGGUGCUUUUUCGGUG
env-223  AUAAUUCUGCCACCGGGUAGA.G.A.U.G...............CGU.....................UCAGCACCG.UCAGUACAUC.GUUAGGUC.U.UUGAA.GAUGUGCU...CA...CGGUGCUUUUUAUCGG
env-224  AUUCGGUUGCCACCGGGUAGC.C.G.A.G...............AUGCG...................AAAGCAUUC.GUGAUACAUC.AUUAGGUC.U.CAGAA.GAUGUAUG..CAC...GGGUGCUUUUUCAUUU
env-225  AACUAGCUACCACCGGGUAGC.A.A.U.AU..............GCC.....................AAGCAUAUC.GUUAUACAUC.GUUAGGUC.U.UUGAA.GAUGUAUG...GCU..GGUACGCUUAUUUUU.
env-226  AUAAAUCUGCCACCGGGUAGA.A.A.C.GU..............GUGUAUU.................AAGCACUCG..GAGCACAUC.GUUAGGUC.U.UAGAA.GAUGUGCU...UG...CGGGUGUUUGUUUUU.
env-227  GUUUAUCUGCCGCCGGGUAGG.U.A.A.AC..............A.......................UCGCAUUUA.UCUGCGUGUC.GUAAGGCC.U.UAGAA.GACGCGCU..UGG...UAAGUGCGUUUUUUAU
Lco-1-1  AAAAAACUGCCACCGGGCAG........................CC........................GCAUCGA.ACACCAUUCC.GUUAGGUC.A.UUGAA.GGAAUGGU...GU...CCGGUGCUUUUUUGUU
env-228  UCUACAUCGCCACCGGGUGAA.A.A.U.G...............UUAA....................AAGCAUUUG.UCCGUACAUC.GUUAGGUC.U.UUGAA.GAUGUACC...GA...CAAGUGCUUUUCUUUU
env-229  CAUUCAUCACCGCCGGGUGA..A.A.A.UG..............CUGA....................AAGCAUUUG.UCUGUACAUC.GUCAGGCC.U.AAGAA.GAUGUACC...GA...CAAGUGCUUUUCUUU.
env-230  AUUGUUCUGCCACCGGGUAGG.A.A.U.G...............CCAA....................AAGCAUUCG.CCGGCGCACC.GUCAGGUC.U.UAGAA.GGUGCGUC...CG...CGAAUGCUUUUUCCGU
env-231  AAAUAUCUGCCACCGGGCAGA.A.A.A.AU..............GCGGAA..................CGGCAUUCU.CGUUUACAUC.GUUAGGUC.U.GAGAA.GAUGUAAA...CA...GGGAUGCCGUUUUUUG
env-232  GAAUAUCUGCCACCGGGUAGG.A.A.U.GUA.....GAUA....CGCG.............UAUC..CAAGC.AUUC.GCGGCACAUC.GUUAGGUC.U.UUGAA.GAUGUGUC...UC...GGAU.GCUUUUUUAAU
env-233  GAAGAAUUGCCACCGGGUAAU........................CA......................AAGCAUUU.UUCAUCCAUC.GUUAGGUC.U.UAGUA.GAUGGAUG...AG....AAUGCUUAUUUUUUU
env-234  AAUCAAUUGCCACCGGGUAAU.U.............................................AAAGCAUUG.AUCAUCUGUC.GUUAGGUC.U.UAGAA.GAUGGAUG...AU....AAUGCUUAUUUUUUU
env-235  AUAUCUCUGCCGCCGGGCAGA.A.A.A.AU..............GCGA....................AAAGCAUUC.CGCUUACAUC.GUCAGGCC.U.UUGAA.GAUGUAUG...CA...GGAUGCUUAUUUUUUU
env-236  AUAUUACUGCCACCGGGUAGG.A.A.U.G...............CAUAA...................CAGCAUUCA.UGUGUGUAUC.GUUAGGUC.A.AAGAA.GAUACGCA...UG...UGAGUGCUUUUUUAUU
env-237  UGUAAUCUGCCACCGGGUAGA.G.A.G.U...............UGCUU...................AUGCACUCG.UUUACACAUC.AUUAGGUC.U.UUGAA.GAUGUGUG........CGGGUGUUUUUUUUAU
env-238  AUUAUACUGCCACCGGGUAGU.U.G.A.................UGUUAACAU...............UAGCAUCCA.AGAGCAUUUC.GUUAGGUC.U.UUGAA.GAAAUGCU...UG...CGGGUGCUUUUUUGGA
Ssu-1-1  UAAACACUGCCACCGGGUAGU.C.A.U.................GCA.....................ACAGCAUUU.UAUGAACAUC.GUUAGGUC.U.UAGAA.GAUGUUCA...UA...GGGUGCUGAUUUUUCU
env-239  GUAUCACUGCCACCGGGUAGU.G.A...................ACGUU...................AUGCGUUCG.UGUGCUUAUC.GUUAGGUC.U.AUGAA.GAUAAGUA....C...CGAAUGCUUUUUUUAU
env-240  AUUUUCCUGCCACCGGGUAGG.G.A.U.G...............CC......................AAGGCAUCU.GCCGCACAUC.GUUAGGUC.U.UAGAA.GAUGUGCA.AAGC...AGAUGUCUUUUAUUUU
env-241  AAAAAUCUGCCGCCGGGUAGA.U.A...................AAA.......................GCAUUUG.UCAGCAUAUC.GUAAGGCC.U.UAGAA.GAUGUGCA...GG...CAGGUGCCAUUUUUUU
env-242  UAUUAAAUCCCACCGGGGAUU.G.A.A.UG..............CA......................GGGCAUUCG.UGUACACAUC.GUUAGGUC.U.UUGAA.GAUGUGUA..UAA...CGGAUGCCUUGAUUUG
env-243  AAAUAGCUGCCACCGGGUAGA.U.G...................AACGAG..................GAGCGUUCU.UUUUCACAUC.AUUAGGUC.U.UAGAA.GAUGUGAA...AG...GGAAGGCUUAUUUUUU
env-244  UAAUAUCAGCCGCCGGGUUGA.G.A.A.UG...............UAAA...................AAGCAUUCG.CGUGCACAUC.AUCAGGCC.U.UUGAA.GAUGUGUU...UG...CGGGUGCUUUUUGUUA
env-245  AUAUAUUUGCCACCGGGCAAC.G.C.G.AU..............GCAA....................AAGCAUCCG.AUUACACGUC.GUUAGGUC.C.UAGAA.GAUGUGUA...AC...CGGGUGCUUUCUGUAU
env-246  AUUUUACUGCCACCGGGUAGU.A.U.C.A...............CA......................AAACAUUCG..CCGUGCGUC.GUUAGGUC.U.UUGAA.GACGCUCG...GA...CGGGUGUUUUUUUAUU
env-247  ACUAAACUGCCACCGGGUAGU.G.A.G.CU..............GAU.....................ACGCGCUCG.UUUCCACAUC.GUUAGGUC.U.UAGAA.GAUGUGAG...GGA..UGAGCGCUUUUUGAAA
env-248  AUAAAACUGCCACCGGGUAGU.G.A.G.CU..............CU......................CAGCGCUCG.UUUCCACAUC.GUUAGGUC.U.UAGAA.GAUGUGUG...AAA..UGUGCGCUUUUUUAAA
env-249  GAAUAGCUGCCACCGGGUAGC.G.A.U.GC..............UUUG....................AGGCAUCCG.UGUGCGUAUC.GUUAGGUC.U.UUGAA.GAUACGCA..UGG...CGGGUGCCUUUCUUAU
env-250  AUUUUGCUGCCACCGGGUAGC.A.U.A.A...............A.........................ACAUUCG.CCAGCACAUC.GUUAGGUC.U.UUGAA.GAUGUGCC...CG...CGAAUGUUUUUUUUCG
env-251  UCAUUGCUGCCACCGGGUAGC.C.A.G.AA..............AUA.....................CAACAUUCG.UCUGCACAUC.GUUAGGUC.U.UUGAA.GAUGUCCA..UCA...CGGGUGUUUCUUUUUU
Ssp-1-1  AUAUGGUUGCCACCGGGUAAG.A.A.A.UG..............CAA.....................UAGCAUUCA.UGUAUGCAUC.GUUAGGUC.U.UAGAA.GAUGUAUA...UG...UGGAUGCAUUUUUUAU
env-252  CCGACACUGCCACCGGGUAGA.G.A.U.G...............CCAAU...................AGGCGUCCG.UUGACACAUC.AUUAGGUC.A.UUGAA.GAUGUGUC...UG...CGGACGCUUUUUUUAU
env-253  CCGUCACUGCCACCGGGUAGA.G.A.U.G...............CAAUAAACU...............UUGCGUCCG.UUGACACAUC.AUUAGGUC.U.UUGAA.GAUGUGUC...UG...CGGGCGCUUUUUAUUU
env-254  CCGACACUGCCACCGGGUAGA.G.A.C.G...............CAAAU...................CAGCGUCCG.UUGACACAUC.AUUAGGUC.U.UUGAA.GAUGUGUC...UG...CGGACGCUUUUUAUUU
env-255  UAUGAGCUGCCACCGGGUAGC.......................AAUGCGAUUU..............AAGCAUUCG.UCGGCACAUC.GUUAGGUC.U.UGGAA.GAUGUGCU...UG...CGGGUGCUUUUUUUGU
env-256  UAAAUACCGCCACCGGGUGGU.A.U...................AAUGUUC.................AAGCAUUUG.GAAAUACAUC.GUUAGGUC.U.UAGAA.GAUGUGUU...UA...CGGAUGCUUUCUUUUU
env-257  AAGACUCUGCCGCCGGGCAGA.G.A.U.................GCGA....................AAGCAUUCC.CAAGCACAUC.GUUAGGCC.U.UAGAA.GAUGUGCU...UG...CGAAUGCUUUUUUCGU
env-258  AUAACACUGCCACCGGGCAGA.A.U.A.UU..............GCAGAU..................AAGCAUUUG.CGUUCGCAUC.GUUAGGUC.U.CAGAA.GAUGCGGG..CAG...CAAAUGCUUUUUUAUU
env-259  AUAAUACUGCCACCGGGUAGU.U.G.A.AU..............GUUGAAAA................AAGCAUUCG..UUGCAUAGC.GUUAGGUC.U.UUGAA.GCUAUGCU....G...CGAAUGCUUUUUGCUU
env-260  AUGAAUCUGCCACCGGGCAGA.G.G.C.A...............GUGCAUU.................AUGCGCUCG.CAAACACAUC.GUUAGGUC.U.UUGAA.GAUGUGUU...UA...CGGGUGCGUUUUUUUU
env-261  AGGAAAUUGCCACCGGGUAAU.G.A.A.U...............GCGAAU..................AAGCGUUUG.UUUGUAUAUC.GUUAGGUC.U.UAGAA.GAUAUGCA...UA...CAAGCGCUCUUUUUUA
env-262  UAUAAUCUGCCACCGGGUAGA.G.G.U.UG..............CAUAA...................AAGCAUCCG.UCAAUACAUC.GUUAGGUC.U.UAGAA.GGUGUAUU...UU...CGGAUGCUUUUAUGUG
env-263  AUCAGAAUGCCACCGGGUAUU.C.A.A.G...............AUGCAGCU................AAGCAUCCG.UAUACACAUC.GUUAGGUC.U.UUGAA.GAUGUGUA...UA...CGGAUGCUUUUUUCAG
env-264  UUGAUACUGCCACCGGGUAGU.G.....................AGCUCAU.................GUGCGCUCG.UUUACACAUC.GUUAGGUC.U.UAGAA.GAUGUGUA...GAU..AGGGUGCUUUUUUACU
env-265  UACUUACUGCCACCGGGUAGU.C.U.G.................CUUUA...................GAGCAUUCG.UUUGUAUAUC.GUUAGGUC.U.UAGAA.GAUAUGCA..UUG...CGGGUGUUUUUAUUUU
env-266  AUUUAGCUGCCGCCGGGUAGU.A.A.A.................UGCUG...................AUGCAUUCG.UGUGCACAUC.GUUAGGUC.U.UUGUA.GAUGUGUA...UU...CGGAUGCUUUUUAAUU
env-267  CUGACACUGCCACCGGGUAGU.G.U.U.A...............UGCGAA..................CAGCAUUCA.UUUACACAUC.GUUAGGUC.U.GAGAA.GAUGUGUG...AA...GGAGUGCUAUUUUUUU
env-268  UGUGUUCUGCCACCGGGUAGA.A.U...................CAUGCCGGAA..............AGGCAUUCG.UCUGCACAUC.GUUAGGUC.U.UUGAA.GAUGUGUA...UA...CGGAUGCCUGUUUUAU
env-269  AUGAAACUGCCACCGGGUAGA.G.U.G.GU..............GCAAA...................GAGCACCCG.CAGACACAUC.GUUAGGUC.U.UAGAA.GAUGUGUC...UA...CGGGUGCCUUUUUUAU
env-270  AUGAUACUGCCACCGGGUAGA.A.U.A.A...............CGGUGCGA................AAGCACCCG.CAAACACAUC.AUUAGGUC.U.UUGAA.GAUGUGUU...UA...CGGGUGCUUUUUUUGU
env-271  UUGUAACUGCCACCGGGUAG..A.A.C.AA..............UGGUGCGA................AAGCACUCG.CAAACACAUC.AGUAGGUC.U.UGGAA.GAUGUGUU...UA...CGAGAGCUUUUUUUGC
env-272  AAUAAUCUGCCACCGGGUAGU.U.G.A.AU..............GUAGA...................AAGCAUUUG.UAAGCAUAUC.GUUAGGUC.U.UAGAA.GAUAUGUU...UA...CGGGUGCUUUUUUGGA
Csp-2-1  GUUAUUUUGCCACCGGGCAAA.G.G.G.................UGCGAA..................AAGCAUUCG.UCUGCACAUC.GUUAGGUC.U.UUGAA.GAUGUGCG...AG...CGGAUGCUUCUUUUUU
env-273  UACUUACUGCCACCGGGCAGG.C.A.A.UG..............CAGAUA..................GAGCAUUCG.UUGACGCAUC.GUUAGGUC.U.UUGAA.GAUGCGUC...CG...CGGAUGCUUUUUUGUU
env-274  UAGAAACUGCCACCGGGUAGG.A.A.U.................GUGAUA..................GAGCAUUCG.CUGGCGCAUC.GUUAGGUC.U.UGGAA.GAUGCGCA...CG...CGGGUGCUUUUUUGUU
env-275  GAAAUACUGCCACCGGGUAGA.A.U.G.AU..............GCGGA...................AAGCAUUCG.UGAGCAUAUC.GUUAGGUC.U.GAGAA.GAUAUGUG..GAG...CGGGUGCUUUUUUUAU
env-276  UAUUUGCUGCCACCGGGUAGC.A.....................CAAU....................AAGCAUUUG.CGCGCGUGUC.GUUAGGUC.U.UGGAA.GAUACGCC...UG...CAAAUGCUUAUUUUUU
env-277  UAAUAACUGCCGCCGGGUAGA.G.A.G.UA..............UU........................ACACUCG.AUUGCACAUC.AUUAGGUC.U.UUGAA.GAUGUGCC...GU...CGGGUGUUUUUUUUCG

         ...........<<<...............................................................................>>>..........................................
         ...........20................................................................................02...........................................
         <<<<<<<<<<<....>>>>>>.>.>.>.>>......<<<<<<<<.............>>>>>>>>...<<<<<<<<<.<<<<<<<<<<..................>>>>>>>>...>>...>>>>>>>>>.......
         22.22222222....222222.2.2...22......1111110..............0111111....22222222.2222222221..................12222222...22...22222222........
         ••••••YUGCCRCCGGGUAR•-○-○-○-○---------------○○○---------------------○○R•••Y••-••••YRY•YC-RUUAGGYC-•-U•GAA-GR•RYR••---••---••R•••YYUUUUUY••
```

```
env-278   AGUUAAUUGCCACCGGGUAAU.G.A.A.U...............GU......................CAGCAUUCG.AUUGCAUAUC.GUUAGGUC.U.UAGAA.GAUAUGCC...GU...CGGAUGCUUUUUUGAU
Eba-1-1   UAAAAAUUACCACCGGGUAAG.A.A.U.................GCAC....................AAGCAUUCA.UACACACAUC.GUUAGGUC.A.UGGAA.GAUGUGUG...UA...UGGGUGCUUAUUUUUU
env-279   AUAUAACUGCCACCGGGCAGC.G.A...................AUGCA...................AAGCAUUCG.AUUAUACAUC.GUUAGGUC.U.UUGAA.GAUGUAUA...GU...CGGAUGCUUUUUUGGA
env-280   AUAUAACUGCCACCGGGUAGC.G.A...................AUGCA...................GAGCAUUCG.AUUAUACAUC.AUUAGGUC.U.UUGAA.GAUGUAUG...GU...CGGAUGCUUUUUUGGA
env-281   AUAAAUCUGCCACCGGGUAGA.......................GAAUGCA.................AGGCAUUCG.CCGGCAUAUC.GUUAGGUC.U.UAGAA.GAUAUGUC...AG...CGAAUGCCUUGUCUGG
env-282   AAACUACUGCCACCGGGUAGA.G.A.A.U...............GUAAUACU................GAACAUUCG.CGGGCAUAUC.GUUAGGUC.U.UUGAA.GAUAUGCC....G...CGGAUGUUUUUUGUUU
env-283   AUAUAUCUGCCACCGGGUAGA.G.A.A.U...............GCG.....................GGGCAUUCG.CCGGCAUAUC.GUUAGGUC.U.UAGAA.GAUAUGUC...AG...CGGAUGCUUUUUGUUU
env-284   UAGUAACUGCCACCGGGUAGU.A.U...................GCG.....................GAGCAUUCG.CAGGUAUAUC.GUUAGGUC.U.UUGAA.GAUAUAUC...AG...CGGAUGCUAUUUGUUU
env-285   UUGUAUCUGCCACCGGGUAGA.G.A.A.UG..............CCU.....................CGGCAUUCG.AUCACGCAUC.GUUAGGUC.U.UUGAA.GAUGCGCG...GU...CGGGUGCCUUUUUUGA
env-286   UCGUUUCUGCCACCGGGUAGA.G.A.A.UG..............CG......................AAGCAUUCG.AUCACACAUC.GUUAGGUC.U.UUGAA.GAUGUGGG...GU...CGGGUGCUUUUUUGAA
env-287   UCGUUUCUGCCACCGGGUAGA.G.A.A.UG..............CG......................AAGCAUUCG.AUCGCACAUC.GUUAGGUC.U.UUGAA.GAUGUGGG...GU...CGGGUGCUUUUUUUGA
env-288   AAUUAUCUGCCACCGGGUAGG.A.A.U.GU..............UAA.....................AAGCAUUCA.CAUACGCAUC.GAUAGGUC.U.UUGAA.GAUGUGUA...UA...UGGAUGCUUUUUUGUU
env-289   AAUUAUCUGCCACCGGGUAGG.A.A.U.GU..............UGA.....................AAGCAUUCA.CAUACGCAUC.GAUAGGUC.U.UUGAA.GAUGUGUA...UA...UGAAUGCUUUUUAUAU
env-290   UAAAAACUGCCACCGGGUAGA.A.A.A.AA..............UGCUA...................UUGCAUUUG.UUUGUGCGUC.GUUAGGUC.U.UAGAA.GACGUGCA...AA...UGAAUGCUUUUUUUUG
env-291   UACGUUCUGCCACCGGGUAGA.G.A.A.AA..............UGCGA...................AAGCAUUCG.UUUGUCCAUC.GUUAGGUC.U.UAGAA.GAUGGGCA.AAAA...CGAAUGCUUUUUUUGU
env-292   UAGAAUUUGCCACCGGGCAAG.A.A.G.AA..............UGUA....................UAGCAUUCG.UUAACAUAUC.GUUAGGUC.U.UUGAA.GAUGUGUU..UAG...CGUAUGCUUAUUUUUU
Cbo-1-1   UUAAAUCUGCCACCGGGUAGG.A.A.U.G...............CAUAU...................GUGCAUUCA.CAUACACAUC.GUUAGGUC.A.UAGAA.GAUGUGUA..UAG...UGGGUGCUUUUUUUAU
env-293   UGAUAACUGCCACCGGGUAGU.......................GUAAUGCUU...............UGGCAUUCG.UCUGCACAUC.GUUAGGUC.U.UUGAA.GAUGUGCA...UA...CGGGUGCUUUUUUUCG
env-294   AUUUUACUGCCACCGGGUAGU.A.U.G.A...............AUGA....................GAGCAUUCA.UAGAUUCAUC.GUUAGGUC.U.UAGAA.GAUGGAUC..AUA...UGGAUGCUUUCUUUUU
env-295   UAAAUACUGCCACCGGGCAGA.U.A.G.AA..............UGCAAA..................AAGCAUUCG.UCGGCGUAUC.GUUAGGUC.U.UAGAA.GAUACGUU..UGG...CGGAUGCUUAUUUUUU
env-296   CAAUAUCUGCCACCGGGUAGA...............................................AGCAUUC..GCAGCGUGUC.GUUAGGUC.U.UUGAA.GAUACGCU...GU...CGAGUGCUUAUUUUUU
env-297   AUAAAACUGCCACCGGGUAGU.......................GAAUGUAA................ACGCAUUCG.UUUUCACAUC.GUUAGGUC.U.UUGAA.GAUGUGAA...UA...CGGAUGCGUUUUUUCA
env-298   AACCAUCUGCCACCGGGUAGA.G.A.G.AU..............GCUAU...................ACGCAUCCG.CC.GCACAUC.GUUAGGUC.U.UAGAA.GAUGUGCC...GA...CGGGUGCGUUGUUUUU
env-299   GGAAGACUGCCACCGGGUAGU.......................GAGAAUGCAA..............AAGCAUUCG.UUAACACAUC.GUUAGGUC.U.UGGAA.GAUGUGUU..UAA...CGGGUGCUUUUUUUGU
env-300   UAGCAACUGCCACCGGGUAGU.G.U.G.AA..............AUGUA...................UUGCAUUCA.CUUGCACAUC.GUUAGGUC.U.UUGAA.GAUGUGUC..AUG...UGGAUGCUUAUUUUUU
env-301   GGAAAGCUGCCACCGGGUAGC.......................AGAGAUGCAA..............GAGCAUCCG.UUUACACAUC.AGUAGGUC.U.UAGAA.GAUGUGUA...UA...CGGAUGCUUAUUUUUU
env-302   UGUAAACUGCCACCGGGUAG........................GAAUGCAUU...............AAGCAUUCG..UCACACAUC.GUUAGGUC.U.UAGAA.GAUGUGUG...AG...CGAAUGCUUUUUUAUU
env-303   ACAGGUCUGCCACCGGGUAGA.U.U...................AAGAAUGCAA..............AGGCAUUUG..CUGCACAUC.GUUAGGUC.U.UUGAA.GAUGUGCG...GA...CAAAUGCCUUUAUUUU
env-304   GAAUAUUUGCCACCGGGCAAUGU.G.A.U...............GCAUUU..................ACGCAUCCG..UUACACAUC.GUUAGGUC.U.UAGAA.GAUGUGGA...AG...CGGGUGCUUAUUUUUU
env-305   GAUAAGCUGCCACCGGGUAGC.A.G.A.AU..............GCUGAUU.................GUGCAUUCG.UUUACACAUC.GUUAGGCC.U.UUGAA.GAUGUGUA..UAA...CGGGUGCUUCUUUUUA
env-306   AAGAAACUGCCACCGGGUAG........................C.......................AAGCGUUUG.UAAGCAUAUC.AUUAGGUC.U.UUGAA.GAUAUGCC...GAU..CAGGCGCUUAUUUUUU
env-307   AUAAAACUGCCACCGGGUAGU.A.A.G.AAUGUACCAAACAUU.UUU...........AAUGUUU...AAGCAUUCA.UAUACGUAUC.GUUAGGUC.U.UUGAA.GAUGCGUA...UG...UGGAUGCUUUUUUAUU
env-308   AAUUUACUGCCACCGGGUAGG.A.A.G.................CGUU....................ACGCAUUCG.UGUACAUAUC.GUUAGGUC.U.UAGAA.GAUAUGUA...CGU..CGGGUGCUUAUUUUUU
env-309   UAGUAACUGCCACCGGGUAGU.G.A.G.UG..............CAU.....................AUGCAUUCG.UUUAUACAUC.GUUAGGUC.U.UGGAA.GAUGUAUA...UG...CGGAUGCAUUUUGUUG
env-310   CAAGUACUGCCACCGGGUAGG.A.A.U.................GCAAG...................GAGCAUUCG.UUUACACACC.AUUAGGUC.U.UUGAA.GGUGUGUA...UG...CGGAUGCUUUUUUAGG
env-311   AAAAGCCUGCCACCGGGCAGG.A.A.U.................GCGCA...................AGGCAUUCG.UAUACACAUC.GUUAGGUC.U.UUGAA.GAUGUGUA...CG...CGGAUGCCUUUUUUGG
env-312   UAAAUACCGCCACCGGGUGGU.U.A.A.U...............UGCAG...................AAGCAUUCG.UUUAUACAUC.GUUAGGUC.U.UUGAA.GAUGUAUA...AAA..CGGGUGCUUUUUUUGA
env-313   AUUUUACUGCCACCGGGUAGU.U.G.U.A...............AUUAC...................AAGCAUUCA.UUUACACGUC.GUUAGGUC.U.UAGAA.GAUGUGUA..AUA...UGAAUGCUUUUUUAUU
env-314   AUUUAACUACCACCGGGUAGU.G.A.U.G...............CUGAU...................AAGCAUUCA.UUUACACAUC.GUUAGGUC.U.UAGAA.GAUGUGUA...AA...UGAGUGCUUGUUUUUU
env-315   CUUAAACUGCCACCGGGUAGA.A.A.U.G...............CAAA....................AAGCAUUUG.UUGGCGCAUC.GUUAGGUC.UUUAGAA.GAUGUGCC...GC...CAAAUGUUUAUUUUUU
env-316   UAUGAACUGCCACCGGGUAGU.A.A.A.................AUGCGAU.................AAGCAUUCG.UUCACACAUC.GUUAGGUC.U.CAGAA.GAUGUGUA...UG...CGAGUGCUUUAUUUUU
env-317   AAAAAACUACCACCGGGUAGU.G.A.A.U...............GCAA....................GGGCAUUCG.UUUUCACAUC.GUUAGGUC.A.UAGAA.GAUGUGAG...AG...CGGAUGCUUUUUUGGA
env-318   UAAACGCUGCCACCGGGUAGC.A.G.A.UG..............CACA....................AAGCAUCCA.UUUACAUAUC.GUUAGGUC.U.UAGAA.GAUGUGUA...UG...CGGAUGCUAAUUUUUU
[gl-1-1   GAGAAUCUGCCACCGGGUAGA.G.U...................AAUGCAAA................AAGCAUUCG.UUUGUACAUC.AUUAGGUC.U.UAGUA.GAUGUGUG...UA...CGGAUGUUUCUUUUUU
env-319   AAAAUACUGCCACCGGGCAGG.A.A.A.U...............GCAAAAA.................AAGCAUUCG.UGCGCGCAUC.GUUAGGUC.U.UUGAA.GAUGCGUG...GA...CGGGUGCUUUCUUUUU
env-320   AAAUGAUUGCCACCGGGUAAU.C.G...................AAAAUGCAGAUA............AAGCAUUCG.UUUGCGUAUC.GUCAGGUC.U.UAGCA.GAUGCGCG...CG...CGGAUGCUUAUUUUUU
env-321   UGAUAACUGCCACCGGGUAGA.A.G.A.UG..............CUUA....................AAGCGUCCG.UUUACACAUC.GUUAGGUC.U.UUGAA.GAUGUGUA...CG...CGGGCGUUUUUUUGAA
env-322   UAAAUUCUGCCACCGGGCAGA.G.A.A.UG..............UUGAAAU.................AAACAUUCG.UUUACACAUC.GUUAGGUC.U.UUGAA.GAUGUGCA...GG...CGAAUGUUUUUUGUUU
env-323   UAGAAACUGCCACCGGGUAG........................AAGAAUGUUAGAA...........AAGCAUUCG.UUUUCACAGC.GUUAGGUC.U.UUGAA.GCUGUGAA....G...CGAAUGCUUUUUUGAU
env-324   UAAUAACUGCCACCGGGUAGU.G.A.A.UG..............UU......................UUGCAUUUG.UUUUCACAUC.AUUAGGUC.U.AAGAA.GAUGUGAU...CA...CGGAUGCAUUUUUUUC
env-325   UUAAAUCUGCCACCGGGUAGU.G.A.G.A...............UGCUU...................UAGCAUCCG.UUUACACAUC.GUUAGGUC.U.UUGAA.GAUGUGUU...AG...CGGAUGCUUUGUUUAU
env-326   UGAAUACUGCCACCGGGUAGU.G.A.A.UG..............CAU.....................CGGCAUUCG.CUUUCGCAUC.GUUAGGUC.U.UAGAA.GAUGCGAA...UG...CGGAUGCUUUUGUUUU
env-327   AAUAUUCUGCCACCGGGUAGA.A.A.A.GU..............GUCA....................AAGCACACG.UAAACACAUC.GUUAGGUC.U.UUGAA.GAUGUGUU...CA...CGGGUGCUUUUUUGUC
env-328   AUUAAUCUGCCACCGGGUAGA.A.G.A.................UGCAUA..................AAGCAUCCG.UUUACACAUC.GUAAGGUC.U.UAGAA.GAUGUGUA...UG...CGGGUGCUUUUUUCAG
env-329   AAAUGUCUGCCACCGGGUAGA.A.A.A.U...............GCUUA...................AAGCAUUCG.UUUGCACAUC.GUUAGGUC.U.UUGAA.GAUGUGUU...UG...CGAAUGCUUUUUUGAU
env-330   UGAUACCUGCCACCGGGUAGG.A.G.A.AA..............UGCAAA..................CAGCAUUCA.U.UACACAUC.GUUAGGUC.U.UAGAA.GAUGUGCA...UA...UGAAUGCUUGUUUUUA
env-331   GACAAGCUGCCACCGGGUAGC.C.A.G.AU..............GCGAAC..................AAGCAUCCG.UUUACACAUC.GUUAGGUC.U.UUGCA.GAUGUGUA...CG...CGGAUGCUUUUUUUCG
env-332   AUUUAACUGCCACCGGGUAGU.A.A.A.AU..............GCUU....................AUGCAUUCG.UAUACACAUC.GUUAGGUC.A.UAGAA.GAUGUGUG...UC...CGGGUGCAUUUUUAAU
env-333   UAGAAACUGCCACCGGGUAGU.......................AGAAUGCA................GAGCAUUCG.UUUACACAUC.GUUAGGUC.U.UGGAA.GAUGUGUA...GA...CGGGUGCUUAUUUUUA
env-334   AAAACUCUACCACCGGGUAGA.A.A.A.U...............GCGA....................AAGCAUUCG.UCAACACAUC.AUUAGGUC.U.UAGAA.GAUGUGUU...CA...CGGGUGCUUAUUUUUU
env-335   UGAAUACUGCCACCGGGUAGU.G.A.AUCG..............CUGAUA..................CAGCAUUCG.CCUGCACAUC.GUUAGGUC.U.UAGCA.GAUGUGCG...UG...CGGAUGCUGUUUUGUU
env-336   AAAUCACUGCCACCGGGUAGG.A.A.A.U...............GCAU....................UUGCAUUCG.UCUGCACAUC.GUUAGGUC.U.UGGAA.GAUGUGUU...CA...CGGGUGCUUUUAUUUU
env-337   AUAAUACUGCCACCGGGCAGC.G.G.A.AU..............GAA.....................AAACAUUCG.UUUACACAUC.GUUAGGUC.U.UUGAA.GAUGUGUA...UG...CGGGUGUUUUUUUUGA
env-338   AUGAUUCUGCCACCGGGUAGG.A.....................GAUGCAUUAUA.............AAGCAUCCG.UUUACACAUC.GUUAGGUC.U.UUGAA.GAUGUGUA...UG...CGGAUGCUUUUUUUCG
env-339   AUAGUACUGCCACCGGGUAGG.G.....................AAUGUUA.................UAGCAUUCG.UUUUCGCAUC.GUUAGGUC.U.UAGAA.GAUGCGGA...UU...CGGGUGCUUUUAUUUU
env-340   AUAGUACUGCCACCGGGUAGG.G.....................AAUGUUA.................AAGCAUUCG.UUUACGCAUC.GUUAGGUC.U.UUGAA.GAUGCGUA...UU...CGGGUGCUUUUUUGGA
env-341   AUAAUACUGCCACCGGGUAGA.G.....................AAUGUUA.................AAGCAUUCG.UUUACGCAUC.GUUAGGUC.U.UUGAA.GAUGCGUA...UG...CGGGUGCUUUUUUUGG
env-342   ACGCUUCUGCCACCGGGUAGA.A.AAU.G...............CGAUA...................AAGCAUUUG.UGUGCACAUC.GUUAGGUC.U.UAGAA.GAUGGGCA...CG...CGGAUGUUUAUUUUUU
env-343   AUCGAACUGCCACCGGGUAGG.U.A.U.G...............CGUA....................AAGCAUUUG.UUUACACAUU.GUUAGGUC.U.CAGAA.GAUGUGUA..AGG...CAAGUGUUUAUUUUUU
env-344   AUAUUACUGCCACCGGGUAGA.A.....................UAUGCGAA................AAGCAUUUG.UUCACGCAUC.GUUAGGUC.U.UUGAA.GAUGUGUG...CG...CAGAUGCUUUCUUUUU
env-345   UAUAUACUGCCACCGGGUAGU.G.A.A.UG..............CGAA....................AAGCAUUUG.UAAGCACAUC.AUUAGGUC.U.UAGAA.GAUGUGUA...UA...CAAAUGCUUUUUUGUU
env-346   UAUAUACUGCCACCGGGUAGU.G.A.A.UG..............CGAA....................AAACAUUUG.UAAGCACAUC.AUUAGGUC.U.UAGAA.GAUGUGUU...UA...CGGGUGCUUUUUUAUU
env-347   UAUGAACUGCCACCGGGUAGU.G.U.A.UG..............CGUU....................CAGCAUUCG.UUCGCACAUC.GUUAGGUC.U.UUGAA.GAUGUGCC...UG...CGGAUGUUUGUUUUUU
env-348   UAAAAACUGCCAACGGGUAGU.G.A.A.UG..............CGUA....................AAGCAUUUG.UUUGUACAUC.GUUAGGUC.U.UUGAA.GAUGUGAA...UU...CAAAUGCUUUUUCUUU
env-349   AAAAAACUACCACCGGGUAGU.G.A.U.................GAUGCGA.................AAGCAUCCG.UAUACACAUC.GUUAGGUC.U.UUGAA.GAUGUGUA...CA...CGGGUGCUUUGUUUUU
env-350   GGUAAACUGCCACCGGGUAGA.A.A.A.U...............GCAA....................AAGCAUUCG.UCUGCACAUC.GUUAGGUC.U.UAGAA.GAUGUGCA...AA...CGGAUGCUUUUUUUAU
env-351   AGUAAGCUGCCACCGGGUAGU.G.A.A.AU..............GCAAG...................AAGCAUUCG.UUUACACAUC.GUUAGGUC.U.UAGAA.GAUGUGUA...UG...CGGAUGCUUUUCUUAA
env-352   UUAUUACUGCCACCGGGUAGU.A.G.G.AU..............GCA.....................AAGCAUUCG.UUUACACAUC.GACAGGUC.U.UAGAA.GAUGUGUA...UG...CGGGUGCUUUAAUUUU
env-353   AUAUAGCUGCUACCGAGUAGC.U.G.A.AU..............GCA.....................AAGCAUUCG.UUUACACAUC.GUUAGGUC.U.UAGAA.GAUGUGUA...UG...CGAAUGCUUUUUUUAU
env-354   UUAAAGCUGCCACCGGGUAGC.A.G.A.A...............UGCG....................AAGCAUUCG.UUUGCACAUC.GUCAGGUC.U.UAGAA.GAUGUGCA...UG...CGGGUGCUUCUUUGUU
env-355   AUAUAACUGCCACCGGGUAGU.G.U.A.AU..............GCUU....................UGGCAUUCG.UUUACACAUC.GUUAGGUC.U.UAGAA.GAUGUGUA...UG...CGGAUGCUUAUUUUUU
env-356   AAUUUACUGCUACCGAGUAGU.A.G.A.AU..............GCC.....................AAGCAUUCG.UUUACACAUC.GUCAGGUC.U.UAGAA.GAUGUGUA...UG...CGGGUGCUUAUUUUUU

          ...........<<<...............................................................................>>>..........................................
          ...........20.................................................................................02..........................................
          <<<<<<<<<<<....>>>>>>.>.>.>.>>......<<<<<<<<.............>>>>>>>>...<<<<<<<<<.<<<<<<<<<<..................>>>>>>>>...>>...>>>>>>>>>.......
          22.22222222....222222.2.2...22......1111110...............0111111....22222222.2222222221..................12222222...22...22222222........
          ●●●●●●YUGCCRCCGGGUAR●-○-○-○-○---------------○○○---------------------○○R●●●Y●●-●●●●YRY●YC-RUUAGGYC-●-U●GAA-GR●RYR●●---●●---●●R●●●YYUUUUUY●●
```

```
env-357   AUGGUACUGCUACCGAGUAGU.A.G.A.AU..............GCA.....................GAGCAUUCG.UUUACACAUC.GUUAGGUC.U.UAGAA.GAUGUGUA...UG...CGGAUGCUUAUUUUUU
env-358   AUGAAACUGCUACCGAGUAGU.A.G.A.AU..............GCA.....................AAGCAUUCG.UUUACACAUC.AUUAGGUC.U.UAGAA.GAUGUGUA...UG...CGGAUGUUUGUUUUUU
env-359   UUAUUACUGCCACCGGGUAGU.A.G.A.A................UGCA....................AAGCAUUCG.UUUACACAUC.GUCAGGUC.U.UAGAA.GAUGUGUA...UA...CGGGUGCUUAUUUUUU
env-360   UUUUAACUGCUACCGAGUAGU.U.G.A.A................UGCA....................AAGCAUUCG.UUUACACAUC.GUUAGGUC.U.UAGAA.GAUGUGUA...UG...CGGAUGCUUAUUUUUU
env-361   AUAUAACUGCCACCGGGUAGU.A.U.G.AU..............GCGAAUA.................AAGCAUCCG.UUUACACAUC.GUUAGGUC.A.UUGAA.GAUGUGUA...AA...CGGAUGUUUAUUUUUU
env-362   AUAAAACUGCCACCGGGUAGU.A.A.A.AU..............GCGA....................AAGCAUUCG.UAUGCACAUC.GUUAGGUC.A.UUGAA.GAUGUGUA...CA...CGGGUGCUUUAUUUUU
env-363   AAUAAACUGCCACCGGGUAGU.G.C.A.U...............GCGA....................GAGCAUCCG.UUUACACAUC.GUUAGGUC.U.UGGAA.GAUGUGUA...CG...CGGAUGCUUAUUUUUU
env-364   GGUUAACUGCCACCGGGUAGU.A.G.A.U...............GCAA....................UAGCAUCCG.UUUACACAUC.GUUAGGUC.U.UUGAA.GAUGUGUG........CGGGUGUUUAUUUUUU
env-365   AAGUUGCUGCCACCGGGUAGU.G.A.A.U...............GCUA....................UAGCAUUCG.UUCUCGCAUC.AUUAGGUC.U.UAGAA.GAUGCGAU...UA...CGGAUGCUUUUAUUUU
env-366   UUGUAACUGCCACCGGGUAGU.G.A.A.UG..............CGA.....................UUGCAUUCG.UUUUCACAUC.GUUAGGUC.U.UAGAA.GAUGUGAA...UG...CGGGUGCUAUUUUUUU
env-367   AAAUAACUGCCACCGGGUAGU.G.A.A.U...............GCA.....................UUGCAUUCG.UUUUCGCAUC.GUUAGGUC.U.UUGAA.GAUGCGAA...UA...CGGGUGCUUAUUUUUU
env-368   AAUUAACUGCCACCGGGUAGG.A.A.A.U...............GCAAA...................AAGCAUUCG.UUUGUGCGUC.GUUAGGUC.U.UUGAA.GACGCACG...CA...CGGAUGCUUUUUUUGA
env-369   AAAUAACUGCCACCGGGUAGU.G.A.A.U...............GU......................AAGCAUUCG.UUUGUGCAUC.GUUAGGUC.U.UUGAA.GAUGCACU....G...CGGAUGCUUAUUUUUA
env-370   AAAACUCUACCACCGGGUAGG.A.A.U.GU..............AAU.....................AAGCAUUCG.CUUGCACAUC.AUUAGGUC.U.UAGAA.GAUGUGUA..UAG...CGGAUGCUCUUUUUUG
Cbo-3-1   CAAAAACUGCCACCGGGUAGG.G.A.U.................GCUAU...................AAGCAUUCA.UGUGGACAUC.GUUAGGUC.A.UAGAA.GAUGUACA...UA...UGGAUGCUUGUUUUUU
env-371   AAACAACUGCCACCGGGUAGU.U.U.A.U...............GCCAA...................AAGCAUUCG.UACGCACAUC.GUUAGGUC.U.UAGAA.GAUGUGCA..GAG...CGGGUGCUUGUUUUUU
env-372   AUAAAACUGCCACCGGGUAGU.......................AGAUGCUAA...............AAGCAUCCG.UUUGUACAUC.GUUAGGUC.U.UAGAA.GAUGUACU...UU...CGGAUGCUUAUUUUAU
Mma-1-1   UAGGCGCUGCCACCGGGUAGC.A.A.U.................GCUGAUA.................GGGCAUUCG.UUUGCACAUC.GUUAGGUC.U.UUGAA.GAUGUGCA..GGA...CGGAUGCUUGUUUUUU
env-373   AAUAGACUGCCACCGGGUAGU.A.G.U.U...............AAAUGUGA................AAGCAUUUG.UUUGCACAUC.GUUAGGUC.U.UAGAA.GAUGUGUU...UG...CAGAUGCUUGUUUUUU
env-374   UAAAAACUGCCACCGGGUAGG.A.A.U.................GCAUU...................GAGCAUUCG.UUUACACAUC.GUUAGGUC.U.AAGUA.GAUGUGUA...UG...CGGAUGCUUAUUUUUU
env-375   AUUGCUCUGCCACCGGGUAGA.A.A.G.A...............GUAA....................AAGUAUUCA.UUUAUACAUC.GUUAGGUC.U.UAGAA.GAUGUGUA...AA...AGGAUGCUUAUUUUUA
Rfl-2-1   UGAAAACUGCCACCGGGUAGU.A.U.A.UG..............CGAU....................AAGCAUUUG.UAUACACAUC.GUUAGGUC.U.UAGAA.GAUGUGUA...CG...CAGAUGCUUUAUUUUU
env-376   UUGAAACUGCUACCGGGUAGG.A.U.A.UG..............CGAG....................AAGCAUUUG.UUUACACAUC.GUUAGGUC.U.UAGAA.GAUGUGUA...UG...CAGAUGCUUUAUUUUU
env-377   UAGUUACUGCCACCGGGUAGA.A.A.U.................GCGAGA..................AAGCAUUUG.UUCACACAUC.GUCAGGUC.U.UAGAA.GAUGUGUG...CG...CAAAUGCUUUUUUUAA
env-378   UAAAAACUGCCACCGGGUAGA.A.A.U.................GCAAAA..................AAGCAUUUG.UCUGCACAUC.GUUAGGUC.A.UUGAA.GAUGUGUU...UG...CAAAUGCUUAUUUUUU
env-379   AUCAUUCUGCCACCGGGUAGA.G.A.A.AG..............CGA.....................AAGCAUUCA.UUUACACAUC.GUUAGGUC.U.UAGAA.GAUGUGUA...AA...GGGAUGCUUAUUUUUU
Rgn-1-1   UAUAAUCUACCACCGGGUAGG.A.A.A.UG..............CGUA....................AAGCAUUCG.UUUACACAUC.AUUAGGUC.U.UUGAA.GAUGUGUA...UG...CGGAUGCUUGUUUUUU
env-380   UAAAAACUGCCACCGGGUAGA.A.A.G.UG..............CUUA....................AAGCAUUCG.UUUGCACAUC.GUUAGGUC.U.UUGAA.GAUGUGUA...UU...CGGGUGCUUAUUUUUU
env-381   AUAAAACUGCCACCGGGUAGA.A.A.U.G...............CUGA....................AAGCAUUUG.UUCACACAUC.GUUAGGUC.U.UAGAA.GAUGUGUG...UG...CAGAUGCUUUAUUUUU
env-382   UGAAAACUGCCACCAGGUAGG.A.A.U.................GCUGA...................AAGCAUUUG.UUCACACAUC.GUUAGGUC.U.UAGAA.GAUGUGUG...CG...CAGAUGCUUUAUUUUU
Ral-1-1   UAAAAACUGCCACCGGGUAGA.A.A.U.................GCGUA...................AAGCAUUUG.UUCACACAUC.GUUAGGUC.U.UUGAA.GAUGUGUG...CA...CAGAUGCUUUAUUUUU
env-383   AUAUAUCUGCCACCGGGUAGA.A.A.U.G...............CGUA....................AGGCAUUCG.UUUACACAUC.GUUAGGUC.U.AAGAA.GAUGUGUG...GG...CGGGUGCUUAUUUUUU
Oru-1-1   AUACGUCUGCCACCGGGUAGA.A.A.U.G...............CGUA....................AAGCAUUCG.UUUGCACAUC.GUUAGGUC.U.AUGAA.GAUGUGUA...UG...CGGGUGCUUAUUUUUU
env-384   AUUGAUCUGCCACCGGGUAGA.A.A.U.G...............CGAA....................AAGCAUUCG.UUAACACAUC.GUUAGGUC.U.UUGAA.GAUGUGUU...UG...CGGAUGCUUAUUUUUU
env-385   AUAAAUCUGCCACCGGGUAGA.A.A.U.G...............CGG.....................AAGCAUUCG.UUUACACAUC.GUUAGGUC.U.UUGAA.GAUGUGUA...UG...CGGAUGCUUAUUUUUU
env-386   AUAAAUCUGCCACCGGGCAGG.A.A.U.G...............CAAA....................AAGCAUUCG.UUUACACACC.GUUAGGUC.U.UAGAA.GGUGUGUA...AU...CGGAUGCUUGUUUUUU
Psp-2-1   UAAGCACUGCCACCGGGUAGU.A.G.A.UG..............CGUA....................AAGCAUUCG.ACUGUACAUC.GUUAGGUC.U.UUGAA.GAUGUAUA...UU...CGGAUGCUUAUUUUUU
env-387   UAAAAUCUGCCACCGGGUAGU.A.G.A.UG..............CGUA....................AAGCAUUCG.UUUGUACAUC.GUUAGGUC.A.UUGAA.GAUGUGCA...UG...CGGAUGUUUAUUUUUU
env-388   CACUCACUGCCACCGGGUAGG.A.A.A.UG..............CUGU....................GAGCAUUCG.ACUGCACAUC.GUUAGGUC.U.UAGAA.GAUGUGCG...GG...CGGAUGCUUCUUUUUU
env-389   AAAACACUGCCACCGGGUAG................................................AAGCAUUCG.CUUGCACAUC.GUUAGGUC.A.UAGAA.GAUGUGCG...GA...CGGAUGCUUAUUUUUU
env-390   AAAAUACUGCCACCGGGUAGU.G.A.A.U...............GCC.....................AAGCAUUCG.AUUAUACAUC.GUUAGGUC.U.UAGAA.GAUGUAUG...GU...CGGAUGCUUGUUUUUU
env-391   AAAAUCCUGCCACCGGGUAGU.G.A.A.U...............GUU.....................AAGCAUUCG.AUUAUACAUC.AUUAGGUC.U.UAGAA.GAUGUAUA...UU...CGGAUGCUUAUUUUUU
Gni-1-1   UAUUAUCUGCCACCGGGUAGA.A.A.G.UG..............CGA.....................AAGCAUUCG.U.UACACAUC.AUUAGGUU.A.UUAAA.GAUGUGUA...UA...CGGGUGCUUAUUUUUG
env-392   AUUAUGCUGCCACCGGGUAGC.A.A.G.AU..............GCA.....................UAGCAUUCA.UAUGCACAUC.GUUAGGUC.U.UAGAA.GAUGUGUG..UAG...UGAAUGCUUUUUAUAU
env-393   AUAAUGCUGCCACCGGGUAGC.A.A.G.AU..............GCA.....................UAGCAUUCA.UAUGCACAUC.GUUAGGUC.U.UAGAA.GAUGUGUA...UGU..UGAAUGCUUUUUAUAU
env-394   UUCAAUCUGCCACCGGGUAGA.G.A.U.G...............CUU.....................CGGCAUCCG.UAAACACAUC.GUUAGGUC.U.UUGAA.GAUGUGUU...CG...CGGAUGCUUUUUUCUU
env-395   AUAAUUCUGCCACCGGGUAGA.G.A.U.G...............CUU.....................CGGCAUCCG.UAAACACAUC.GUUAGGUC.U.UUGAA.GAUGUGUU...CG...CGCAUGCUUUUUUCUU
env-396   AAAUAACUGCCACCGGGUAGU.A.A...................AAAUGCUUAA..............AAGCAUUCG.UGUGUACAUC.GUUAGGUC.U.UUGAA.GAUGUGCA..UUA...CGAAUGCUUUUUUGAG
env-397   AAUAAACUGCCACCGGGUAG........................GAAUGCAAA...............AAGCAUUCA.UCUGUACAUC.GAUAGGUC.U.UUGAA.GAUGUGCA...AA...UGAAUGCUUUUUUACA
env-398   CAGAUACUGCCACCGGGUAGU.G.A.A.UG..............AA......................AAGCAUUCG.UUUGCACAUC.GUUAGGUC.U.UUGAA.GAUGUGCA....G...CGAAUGCUUUUUUAAA
env-399   UGUAAUCUGCCACCGGGUAGA.A.A.A.UGUU....CGGCGA..AAGAAUGA.......UUGCCG...AAGCAUUUG.CCUGCACAUC.GUUAGGUC.U.UUGAA.GAUGUGCG...AG...CAAAUGCUUUUUUGUU
env-400   UUAUAACUGCCACCGGGUAGA.A.A.A.UG..............CAG.....................AAGCAUUUG.UCCGCACAUC.GUUAGGUC.U.UUGAA.GAUGUGCC...CG...CAAAUGCUUAUUUUUA
env-401   AUGUUGCUGCCACCGGGUAGU.G.A.G.................CGA.....................AAGCUCUCG.UUUGCACAUC.GUUAGGUC.U.UAGAA.GAUGUGUA...AG...CGAGGGCUUAUUUUUU
env-402   UGAUUUCUGCCACCGGGUAGA.G.A.A.UG..............UAUCAA..................AAACAUUCG.UAUGUACAUC.GUUAGGUC.A.UAGAA.GAUGUACA...GG...CGGAUGUUUUUUUGUU
env-403   AAUAAACUGCCACCGGGCAGU.A.G.A.U...............GCUU....................AAGCAUUUG.UGUGCAUAUC.GUUAGGUC.U.UUGAA.GAUAUGCA..CUG...CAAAUGCUUAUUUUUU
env-404   AUUCAACUGCCGCCGGGCAGU.A.A.A.................UGCUU...................AAGCAUUUG.UGUGUAUAUC.GUUAGGUC.U.UUGAA.GAUAUGCA..CUG...CAAAUGCUUAUUUUUU
env-405   AAUAAUCUGCCACCGGGUAGA.G.A.G.AU..............GCUC....................AGGCAUCCG.UUUGCACAUC.GUCAGGUC.A.CAGAA.GAUGUGUA...UG...CGGAUGCCUGUUUUUA
env-406   UGAUAUUUGCCACCGGGCAAA.G.G...................AAUGUA..................AAGCAUUCA.UACGCUUAUC.GUUAGGUC.U.UUGAA.GAUAAGCA...UA...UGGGUGCUUAUUUUUU
env-407   CAAGUUUUGCCACCGGGCAAG.A.A.U.................GCA.....................GAGCAUUCA.UAUACUUAUC.GUUAGGUC.U.UUGAA.GAUAAGUA...UG...UGGGUGCUUAUUUUUU
env-408   AACAAUUUGCCACCGGGCAAA.A.G.A.AU..............GUU.....................GAGCAUUCA.UAUACUUAUC.GUUAGGUC.U.UUGAA.GAUAAGUA...UG...UGGGUGCUAUUUUUUU
env-409   AAAGAUUUGCCACCGGGCAAA.C.G.A.AU..............GUA.....................AAGCAUUCA.GGUACUUAUC.GUUAGGUC.U.UUGAA.GAUAAGUG...CG...UGGGUGCUAUUUUUUU
env-410   GAAUAUUUGCCACCGGGCAAA.A.G.A.AU..............GCC.....................AAGCAUUCA.UGUACUUAUC.GUUAGGUC.U.UUGAA.GAUAAGUA...UG...UGGGUGCUUAUUUUUU
env-411   GACUAUUUGCCACCGGGCAAA.A.G.A.AU..............GUA.....................AAGCAUUCA.UAUACUUAUC.GUUAGGUC.U.UUGAA.GAUAAGUA...UA...UGAAUGCUUAAUUUUU
env-412   AAUAAUCUGCCACCGGGUAGA.......................AAUGCACG................AAGCAUUUG.CUUGUAUAUC.GUUAGGUC.U.UUGAA.GAUAUACA..AAG...UAAAUGCUUAUUUUUU
env-413   AUAAUUCUGCCACCGGGCAGA.G.A.C.G...............AAAG....................AAACAUUCG.UCCGCAUGUC.AUUAGGUC.U.UUGAA.GACAUGCA...GA...GGAAUGUUUCUUUUUU
env-414   AAUAUUCUGCCACCGGGUAGA.A.....................AUACGAAUGGA.............AAACAUUUG.UCUGCAUGUC.GUUAGGUC.U.UUGAA.GAUUUGCA...GA...GAAAUGUUUCUUUUUU
env-415   AAAUAUCUGCCACCGGGUAGA.......................A.......................AAACAUUUG.UCUGCAUGUC.AUAAGGUC.U.UAGAA.GAGAUGCA...GG...UGAAUGUUUCUUUUUU
env-416   AGAAUUCUGCCGCCGGGCAGA.A.......................................................AACAUUCG.UUUCCAUGUC.GUUAGGCC.U.UUGAA.GACAUGCG...GA...CGGAUGUUAUUUUUUU
env-417   AAUAGUCUGCCACCGGGUAGG.......................AAUGUAA.................AAACAUUCG.UGUGCAUAUC.GUUAGGUC.U.UUGAA.GAUAUGCA...UA...UGAAUGUUUUAUUUUU
env-418   AAAAAACUGCCACCGGGCAGA.G.A.A.AU..............GCG.....................CAGGCAUUC.GGCGCACAUC.GUUAGGUC.U.UUGAA.GAUGUGUG..GGC...GGAUGCUUAUUUUUU.
env-419   UAUAUGCUGCCACCGGGUAGC.U.U.U.GA..............AU......................UAGCAUUCA.UUUAUACAUC.AUUAGGUC.U.UAGAA.GAUGUAUA..UUU...UGAAUGCUUUUUUGUA
env-420   AAAGUACUGCCACCGGGCAGG.A.U.A.AU..............GCAG....................AAGCAUUCA.UGGACAUAUC.GGUAGGUC.U.UAGGA.GAUAUGUG...UA...UGGGUGCUUUUUGCCU
env-421   AAUAAACUGCCACCGGGCAGG.A.A.A.U...............GCUG....................AGAGCAUUC.GGUUCGCUUC.GUUAGGUC.U.UUGAA.GAAGCGUA...CU...GAGUGCAUUUUUUGUU
env-422   UCAUAACUGCCACCGGGCAGA.U.A...................AAUGCGAA................AAAGCAUUU.GAAGCACAUC.GUUAGGUC.U.UUGAA.GAUGUGCU....C...AGAUGCUUUAUUUUUU
env-423   AACAGUCUGCCACCGGGUAGA.......................GAUGCA..................AAAGCAUUC.GUUACACAUC.AUUAGGUC.U.UUGAA.GAUGUGUA..AGC...GGAUG.UUUAUUUUAU
env-424   AUAAAACUGCCACCGGGUAGA.U.....................AAUGCGA.................AAAGCAUUC.AUAGCGUAUC.GUUAGGUC.U.UUGUA.GAUACGC....GU...GAAUGCUUAUUUUUUU
env-425   UAGGAACUGCCACCGGGUAGU.G.U.A.UG..............CUGU....................UAAGCAUUC.GUCGCACAUC.GUUAGGUC.U.UUGAA.GAUGUGCG.CCAC...GGGUGCUUAUUUUUUA
env-426   AAUUAUCUGCCACCGGGUAGA.A.A.A.................ACCG....................AAUGCAUUC.GUUGCACAUC.GUUAGGUC.U.UUGAA.GAUGUGUA.UUAC...GGGUGCUUUUUAUU..
env-427   UACAAUCUGCCACCGGGUAGG.......................AAUGCA..................AAAGCAUUC.GUUACACAUC.GUAAGGUC.U.UAGAA.GAUGUGUA..AAC...GAAUGCUUUUCAGGUU
env-428   GAAUAUUUGCCACCGGGCAAA.G.U.G.AU..............GCA.....................CGAGCAUCC.GUAACACAUC.GUUAGGUC.U.UUGAA.GAUGUGUA..AGC...GGGUGCUUGUUUAUUU
env-429   AAACGACUGCCACCGGGUAGU.......................GAAGCCU.................ACAGCAUUC.GCCGUAUAUC.GUUAGGUC.U.UAGAA.GAUAUACG.CAGC...GAAUGCUGUUUUUUGU
env-430   AUAAAACUGCCACCGGGUAGU.G.A.A.G...............CUU.....................ACAGCAUUC.GCCGUACAUC.GUUAGGUC.U.UAGAA.GAUGUGCG.CAAC...GAAUGCUGUUUUUUGU

          ...........<<<...............................................................................>>>..........................................
          ...........20................................................................................02...........................................
          <<<<<<<<<<<....>>>>>>.>.>.>.>>......<<<<<<<<.............>>>>>>>>...<<<<<<<<<.<<<<<<<<<<..................>>>>>>>>...>>...>>>>>>>>>.......
          22.22222222....222222.2.2...22......1111110..............0111111....22222222.2222222221..................12222222...22...22222222........
          ••••••YUGCCRCCGGGUAR•-◦-◦-◦-◦---------------◦◦◦---------------------◦◦R•••Y••-••••YRY•YC-RUUAGGYC-•-U•GAA-GR•RYR••---••---••R•••YYUUUUUY••
```

```
env-431   AUCAAGCUGCCACCGGGCAGC.AGA.G.AU..............GCA.....................AAAGCAUCC.GCCGCACAUC.GUUAGGUC.U.UGGAA.GAUGUGCC..GAC...GGGUGCUUGUUUUUUU
env-432   ACAACACUGCCACCGGGUAG........................AUAAAAUGCUU.............UAAGCAUUC.GUUGCAUAUC.GUUAGGUC.U.UAGAA.GAUAUGUU...AC...GGGUGCUUUUUUUGGA
env-433   UUUUUACUGCCACCGGGUAGG.C.G.G.UG..............CA......................UCGGCACUC.GUUACACAUC.GUUAGGUC.U.UUGAA.GAUGUGUA...AG...GGGUGUUAUUUUUUUG
env-434   AAUUUACUGCCACCGGGUAGG.C.G.G.U...............GCU.....................AUCGCACUC.GUUACACAUC.GUUAGGUC.U.UUGAA.GAUGUGUG...AU...GGGUGUUAUUUUUUGA
Rsp-2-1   AUAUGACUGCCACCGGGUAGG.C.G.G.U...............GCA.....................CAAGCACUC.GUUACACAUC.GUUAGGUC.U.UUGAA.GAUGUGUG...AG...GGGUG.UUGUUUUUUG
env-435   ACACGACUGCCACCGGGUAGG.A.A.U.G...............CAC.....................AGAGCAUUC.GCCACACAUC.GUUAGGUC.U.CAGAA.GAUGUGUG....C...GGAUGCUUUUUUAUGC
env-436   ACACUACUGCCACCGGGUAGG.A.A.U.G...............CAC.....................AGAGCAUUC.GCCACACAUC.AUUAGGUC.U.UAGAA.GAUGUGUG....C...GGAUGCUUUUUGUUUG
env-437   AUGAUUCUGCCACCGGGUAGA.G.....................AAUG....................GAAGCAUUC.GCAGCGCAUC.GUUAGGUC.U.UUGAA.GAUGCGUG..AGC...GGGUGCUUAUUUUUUG
env-438   GUAAUCCUGCCACCGGGUAGG.G.....................AAUGG...................AAGCAUUC.GUUGCGCAUC.GUUAGGUC.U.UUGAA.GAUGCGUG..UAC...GGGUGCUUGUUUUAUU
env-439   UAUUAAUUGCCACCGGGUAAU.A.A.A.A...............UGCG....................AAAGCAUUC.GUUGCACAUC.AUUAGGUC.U.UUGAA.GAUGUGUA..CAC...GAGUGCUUAUUUUGUU
env-440   AGUAAUCUGCCACCGGGUAGA.......................AGCGA...................AAGCAUUC.GCCGCACAUC.GUUAGGUC.A.UUGAA.GAUGUGCU..UGC...GAAUGCUUGUUUUUUG
env-441   UAUAAUCUGCCACCGGGUAGA.G.A.A.UG..............CAUA....................AAGCAUUU.GUUACACAUC.AUUAGGUC.U.UUGAA.GAUGUGUU....UAC.AAGUGCUUAUUUUUUU
env-442   AACACACUGCCACCGGGUAGA.G.U.A.AU..............GCAU....................AAAGCAUUC.GUAACAUAUC.GUUAGGUC.U.UAGAA.GAUAUGUC...AC...GGAUGCUUUUUUGGGA
env-443   AUUAUGCUGCCACCGGGUAGU.A.A.A.AU..............GCAA....................AAAGCAUUC.UGUACACAUC.GUAAGGUC.U.UAGAA.GAUGUGUA...UC...GAAUGCUUUCUUUUUU
env-444   AUAAAUCUGCCACCGGGUAGG.A.A.U.................UGCCA...................UGAGCAUUC.GCUGCACAUC.GUUAGGUC.U.UUGAA.GAUGUGCA...UC...GGAUGUUUAUUUUUUU
env-445   AUACCACUGCCACCGGGUAGA.G.U.A.AU..............GCA.....................AAUGCAUUC.GGUACACAUC.GUUAGGUC.U.UUGAA.GAUGUGUA..UAC...GGAUGCUUUUUUGUUU
env-446   AUACCACUGCCACCGGGUAGA.G.U.A.AU..............GCA.....................GAUGCAUUC.GGUACACAUC.GUUAGGUC.U.UAGAA.GAUGUGUG..CGC...GAAUGCUUUUUUCUUU
env-447   UUGAAUCUGCCACCGGGUAGA.A.A.A.CA..................................AAAGCAUUU.GUUACAUAUC.GUUAGGUC.U.UUGAA.GAUAUGUA...AAU..AAAUGCUUAUUUUUUU
env-448   AUUAUACUGCCACCGGGCAGA.G.G.A.................UGCA....................GGCGCAUUC.GUUACACAUC.GUUAGGUC.U.UUGAA.GAUGUGUA..UAC...GGGCGCUUAUUUUUUU
env-449   AAAACACUGCCACCGGGUAGU.A.A.G.AU..............GC......................UUAGCAUUC.GGUACACAUC.GUUAGGUC.U.UUGAA.GAUGUGUA..UGC...GGGUGCUUGUUUUUUU
Lba-3-1   AAUAGGCUGCCACCGGGUAGA.A.A.A.................GCG.....................AAAGCAUUC.GUUACACAUC.GUUAGGUC.U.UUGAA.GAUGUGUG..GUC...GGGUGCUUAUUUUAUU
env-450   AAGAUUCUGCCACCGGGUAGA.A.A.C.AU..............GCG.....................AAAGCAUUC.GCUACACAUC.GUUAGGUC.U.UGGAA.GAUGUGUA..UGC...GGAUGCUUAUUUUUUU
env-451   AUGGUUCUGCCACCGGGUAGA.A.A.A.A...............GCG.....................AAAGCAUUC.GUUACACAUC.AUUAGGUC.U.UAGAA.GAUGUGUA..UAC...GGGUGCUUGUUUUUAU
env-452   AUUGUUCUGCCACCGGGUAGA.G.A.U.G...............CG......................AAAGCAUUC.GUUACACAUC.AUUAGGUC.U.UAGAA.GAUGUGUA..UAC...GGAUGCUUAUUUUUGU
env-453   AAUAUUCUGCCACCGGGUAGA.G.A.A.................GCG.....................AAAGCAUUC.GUUACACAUC.GUUAGGUC.U.UAGAA.GAUGUGUA..AAC...GGAUGCUUUUAUUUUG
env-454   AAUAGUCUGCCACCGGGUAGA.G.A.A.................GCG.....................AAAGCAUUC.GUUACACAUC.GUUAGGUC.G.UUGAA.GAUGUGUA..GAU...GGAUGCUUUUUAUUUU
env-455   AAUGGUCUGCCACCGGGUAGG.U.A.A.................GCG.....................AAAGCAUUC.GUUACACAUC.GUUAGGUC.U.UUGAA.GAUGUGUA..UAA...GGAUGCUUAUUUUUGU
env-456   GUUACUCUGCCACCGGGUAGA.A.A.U.G...............UG......................AAAGCAUUC.GUUGCACAUC.GUUAGGUC.U.UAGAA.GAUGUGUA..UAU...GGGUGCUUGUUUUUGU
env-457   AUUAAUCUGCCACCGGGUAGA.G.A.A.................UGCA....................AAAGCAUUC.GUUACACAUC.GUUAGGUC.U.UUGAA.GAUGUGUA..UAC...GAAUGUUUAUUUUUUU
env-458   AUUAAUCUGCCACCGGGUAGA.G.A.A.................UGCA....................AUAGCAUUC.GUUGCACAUC.GAUAGGUC.U.UUGAA.GAUGUGUA...AU...GGGUGCUUUUUUUUGA
env-459   AUUAGUCUGCCACCGGGUAGA.G.A.A.G...............CG......................AAAGCAUUC.GUUGCACAUC.GUUAGGUC.U.UUGAA.GAUGUGUA..UAC...GGGUGCUUUUUUUGGA
env-460   AGUAAACUGCCACCGGGUAGG.A.A.A.U...............GCA.....................AAAGCAUUC.GCGGCACAUC.GUUAGGUC.U.UGGAA.GAUGUGCG..GGC...GAAUGCUUUUUUUGGA
env-461   AAUAUACUGCCACCGGGUAGG.A.A.A.U...............GC......................AAAGCAUUC.GCGGCACAUC.GUUAGGUC.U.UAGAA.GAUGUGUA..CAC...GGAUGCUUUUUUUGAU
env-462   UUUUAACUGCCACCGGGUAGG.A.A.A.UG..............C.......................AAAGCAUUC.GCGGCACAUC.GUUAGGUC.U.UUGAA.GAUGUGCA..GGC...GAAUGCUUUUUGAUUU
env-463   AAGAAACUGCUACCGAGUAGU.A.G.A.AU..............GC......................AAAGCAUUC.GUUGCACAUC.AUUAGGUC.U.UAGAA.GAUGUGUA..UGC...GAGUGCUUAUUUUUUU
Dsp-1-1   GGUAAUCUGCCACCGGGUAGA.U.A.A.GC..............G.......................AAAGCAUUC.GUUACACAUC.GUUAGGUC.A.UUGAA.GAUGUAUA..AGC...GGAUGCUUAUUUUAUU
env-464   AUAUAUCUGCCACCGGGCAGA.G.A.U.G...............CUU.....................UAAGCAUUC.AUUACUCAUC.GUUAGGUC.U.UUGAA.GAUGAGCA..UAU...GGACGCUUAUUUUUUU
env-465   AUGAAUCUGCCACCGGGCAGA.G.A.U.G...............CUU.....................AAAGCAUCC.GUUACUCAUC.GUUAGGUU.U.UUGAA.GAUGGGUA..AAU...GGGUGCUUAUUUUUUA
env-466   AUUUAUCUGCCACCGGGCAGA.G.A.U.G...............CCA.....................AAAGCAUCC.AUUACUCAUC.GUUAGGUC.U.UUGAA.GAUGAGUA..UAU...GGGUGCUUUUUUUAGG
env-467   AAAAAACUGCCACCGGGUAGA.U.....................AGCG....................AAAGCAUUC.AUUACACAUC.AUUAGGUC.U.UUGAA.GAUGUGUA..AAU...GGAUGCUUAUUUUUAU
env-468   AUAACUCUGCCACCGGGCAGG.A.A.U.G...............GA......................AAAGCAUUC.AUUACUUAUC.GUUAGGUC.U.UUGAA.GAUAAGUA..CAU...GGGUGCUUAUUUUUUC
env-469   GAGAAUCUGCCACCGGGUAGG.A.G.A.U...............GCA.....................UAGGCAUCC.GUUACACAUC.GUUAGGUC.U.UAGAA.GAUGUGUA..GAC...GGAUGCUUUUUUAGCG
env-470   UAUAAACUGCCACCGGGUAGU.A.U.A.UG..............CGC.....................AGAGCAUUC.AAUGCACAUC.GUCAGGUC.U.UAGAA.GAUGUGUG...UU...GGAUGCUUUUUUCGGA
Rsp-3-1   UAUAAACUGCCACCGGGUAGU.A.U...................GCG.....................UAAGCAUUC.GAUACACAUC.GUUAGGUC.U.UAGAA.GAUGUGUG..UUC...GGAUGCUUUUUUUGGG
env-471   AAUAAACUGCCACCGGGUAGG.U.A.A.U...............GUG.....................AAAGCAUUU.GUCGUACAGC.GUUAGGUC.U.UUGAA.GCUGUACC..GGC...AAGUGCUUUUUUUGGA
env-472   CAAAUUCUACCGCCGGGUAGG.A.A.U.................GCG.....................AACGCAUUC.GCCGCACAUC.GAUAGGUC.U.UUGAA.GAUGUGCC...GC...GAAUGCUUAUUUUUUG
Rfl-1-1   AUAUAACUGCCACCGGGCAGA.G.A.A.AU..............GCA.....................GAAGCAUUC.GUCCCACAUC.GUUAGGUC.U.UUGAA.GAUGUGAG..GGC...GGAUGCUUUUUUUACG
env-473   GAAUAUCUGCCACCGGGUAGA.A.A.A.AU..............GCG.....................AAAGCAUUC.GUAGCAUAUC.GUUAGGUC.U.UUGAA.GAUAUGCG...GC...GAAUGCUUUUUUGUUU
env-474   AUAUAUCUGCCACCGGGUAGA.A.A...................AUGCG...................AAAGCAUUC.GCUGUAUGUC.GUUAGGUC.U.UUGAA.GAUAUACA..GUC...GAAUGCUUUUUUGUUC
env-475   AAUAUACUGCCACCGGGUAGA.A.G.A.GU..............GUU.....................AAAGCAUUC.GUCGCAUAGC.GUUAGGUC.U.UUGAA.GCUAUGCC...GC...GAAUGCUUUUUGAUUG
env-476   AAUUAUCUGCCACCGGGUAGG.A.A.A.U...............GCA.....................GCUGCAUUC.AUCUCACAUC.AUUAGGUC.U.UUGAA.GAUGUGUC...AU...GGAUGCUUCUUUUUUU
env-477   AUAAAACUGCCACCGGGUAGU.......................AAAUGCG.................AAAGCAUUC.GUAACACAUC.GUUAGGUC.U.UAGAA.GAUGUGUC...GC...GGAUGCUUGUUUUUUU
env-478   AUAAGUCUGCAACCGGGUAGA.A.U.A.A...............AUC.....................AAAGCACUC.GUUACACAUC.GUCAGGUC.U.UAGAA.GAUGUAUA...AC...GAGUGUACUUAUUUUU
env-479   AUAAGUCUGCCACCGGGUAGA.G.U.A.A...............AUC.....................GAGGCACUC.GUUACACAUC.GUUAGGUC.U.UAGAA.GAUGUGUA..GUC...GUGUGCUUAUUUUUAA
env-480   UUAAAUCUGCCACCGGGUAGA.A.A.A.UG..............CA......................CCAGCAUUC.GUGACACAUC.GUUAGGUC.U.UAGAA.GAUGUGUA..AGC...GAGUGUUGUUUUUAUU
env-481   UAUUAUCUCCCACCGGGGAGA.G.A...................UGCAA...................AAAGCAUCC.GCAGCACAUC.GUUAGGUC.U.UUGAA.GAUGUGCC..GGC...GGGUGCUUUGUUUUUU
env-482   UUAAUACUGCCACCGGGUAGU.A.U.A.A...............UCUA....................CAAGCAUUC.AAAACACAUC.AUUAGGUC.C.UAGGA.GAUGUGUU..UUC...GAAUGCUUAUUUAUUU
env-483   AAUAAUCUGCCACCGGGUAGG.......................AAUGCUGA................ACGCAUUCG.UUUGCGCGCC.GUUAGGUC.U.UAGAA.GGCGCGCA...AA...CGGAUGCGUUUUUGUU
env-484   AUAUUUCUGCCACCGGGUAGA.A.A.A.AU..............GCA.....................AAGGCAUUC.CCUGUACAUC.GUCAGGUC.U.UAGAA.GAUGUACA...GC...GAGUGCCUUUUUUGAG
Csp-1-1   UAAAGAUUACCACCGGGUAAG.A.A.U.................GUAGC...................GAGCAUUCA.UUUGUACAUC.AUUAGGUC.U.UAGAA.GAUGUGCA...AA...UGGAUGCUUUUUUGCA
env-485   UAUACACUGCCACCGGGUAG..................................................ACAUUUA...UCUCACAUC.GUUAGGUC.U.UUGAA.GAUGUGCG...GA...UUGAAUGUCUUUUUUA
env-486   AAUUGCUUGCUGCCGAGUAAG.C.A.A.................GAA.......................GCAUUUA.UCUUUGCAUC.GUUAGGCC.U.UUGAA.GAUGCAUC...GA...UAGAUGCAUUUUUUGU
env-487   CAAUGUUUGCUGCCGAGUAAA.C.A...................GA......................AGGCAUUUA.UCUUUGCAUC.GUUAGGCC.U.UUGAA.GAUGCAUC...GA...UAGAUGCAUUUUUUAU
env-488   GUUUACUUGCUGCCGAGUAAG.U.A.A.AU...................................UGCAUUUA.UGUAUGUAUC.AUUAGGCC.G.UUGAA.GAUACAUC..GUA.....AAUGCAUUUUUUUG
env-489   UCAGUACUGCCACCGGGUAGA.A.A.U.G...............CAA.....................AGCAUUUGU.UAAGUACAUC.GUUAGGUC.U.UUGAA.GAUGUGCU...CA...GCAAAUGCUUUUUUUG
env-490   UAGUUACUGCCACCGGGUAGA.A.A.U.................GCAG....................AGCAUUUGU.UAAGUACAUC.GUUAGGUC.U.UUGAA.GAUGUACU...GA...GCAAGUGCUGUUUUUU
env-491   UAUUUACUGCCACCGGGUAGA.A.A.U..G..............CAA.....................AGCAUUUGU.UAAGUACAUC.GUUAGGUC.U.UUGAA.GAUGUGCU...CA...GCAAGUGCGAUUUUUU
env-492   UAUUAACUGCCACCGGGUAG............................................CAUUUG..UUUGUGUAUC.GUUAGGUC.A.UUGAA.GAUAUGCA...AU...GCGAAUGCUCUAUUUU
env-493   UAUUGACUGCCACCGGGUAGU.G.A.G.UG..............CAU.....................AGGACACUU.GUGUCACAUC.GUUAGGUC.U.UAGAA.GAUGUGAU...AU...GAGCGUCCAUUUUUUU
env-494   AUGCAGCUGCCACCGGGUAGC.A.G...................AUCGUGA.................AAGCGUUCG.CCGGCGUAUC.GUUAGGUC.U.UAGAA.GAUACGUC..AUG...CGGGCGCUGUUUUUUC
env-495   UUAGAUUUACCGCCGGGUAAA.U.C.U.CA.....................................CGCGUU.UGUUUGCAUC..GUAGGCC.U.UUGAA.GAUGCAAA...UG...GACGCGUUUUUUAUUU
env-496   ACCCCUUUGCCGCCGGGUAAA.G.....................CAACU...................AAGCACUAA.GCUUCAUAUC.GUUAGGCC.U.UAGAA.GAUAUGGA...GAA..CUGGCGUUUUUGUGGA
env-497   UUAAACUCGCCGCCGGGUGAG.A.A.G.UG..............CUAAUUU.................AAGCGACGG.AUUUCGUAUC.GUUAGGCC.U.UAGAA.GAUACGGA...GU...GCGUCGCUUUUAUAAU
env-498   ACUUUUUUGCCGCCGGGUAAA.G.A.G..U..................................UAAGCGUUA.GGCUUUCAUC.GUUAGGCC.U.UUGAA.GAUAGAAA.GUUC...UGGCGCUAUUUUUUUG
env-499   UGUAAAUUGCCGCCGGGUAAG.C.U...................UUC.....................GAGCAUUU.AUUUCGUAUC.GUUAGGCC.U.UAGAA.GAUGCGAA...AUC..AAAUGCUUCUUUUAUU
env-500   GAUAACCUGCCGCCGGGCAGG.......................CA......................AAAGCGUUG.GUUUCGCAUC.AGUAGGCCGU.UAGAA.GAUGUGAA..AGU...CAAUGCUUUAUUUUUU
env-501   GGAUGCCUGCCGCCGGGCAGG.C.G.U.....................................AAGCGUUG.GUUUCGUAUC.AGUAGGCCGU.UAGAA.GAUGCGAA..AGU...CAAUGCUUUAUUUUUU
env-502   GACUGCUUGCCGCCGGGUAAG.C.G.C.U...............GU.........................GCGUUG.AUUUCGUAUC.GUUAGGCC.A.AUGUA.GAUACGAA..AGU...CAGCGCUUUAUUUUUG
Esp-4-1   AAAUGCUUGCCGCCGGGUAAG.A.G...................CA......................GGAGCGUUG.AUUUCGCAUC.GUUAGGCC.G.UUGAA.GAUGCGAA..GGU...CAAUGCUUUUCUUUUG
Esp-3-1   AAAUGCUUGCCGCCGGGUAAG.A.G...................CA......................GAAGCGUUG.AUUUCGUAUC.GUUAGGCC.A.UUGAA.GAUACGAA..GGU...CAAUGCUUUUCUUUUU
env-503   CUUUACUUGCCGCCGGGUAAG.U.G...................UUCA......................GGCAUUG.AUUUCGUAUC.GUUAGGCC.A.UAGAA.GAUACGAA..CAU...CAAUGCUUUUCUUUGC
env-504   UUUUGCUUGCCGCCGGGUAAG.C.....................UUU.....................GAAGCAUUG.AUUUCGUAUC.GUUAGGCC.G.UUGGA.GAUACGAA..AAU...CAAUGCUUUUUUCUAU

          ...........<<<...............................................................................>>>..........................................
          ...........20................................................................................02...........................................
          <<<<<<<<<<<....>>>>>>.>.>.>.>>......<<<<<<<<.............>>>>>>>>...<<<<<<<<<.<<<<<<<<<<..................>>>>>>>>...>>...>>>>>>>>>.......
          22.22222222....222222.2.2...22......1111110..............0111111....22222222.2222222221..................12222222...22...22222222........
          ●●●●●●YUGCCRCCGGGUAR●-○-○-○-○---------------○○○---------------------○○R●●●Y●●-●●●●YRY●YC-RUUAGGYC-●-U●GAA-GR●RYR●●---●●---●●R●●●YYUUUUUY●●
```

```
env-505    UUUUGCUUGCCGCCGGGUGAG.C.A...................UC......................AGAGCAUUG.AUUUCGUAUC.GUUAGGCC.A.UGGAA.GAUACGAA..AAU...CAAUGCUUUUCUUUUU
env-506    UUCUGCUUGCCGCCGGGUAAG.C.U.A.UA.....................................AGGCAUUG.GUUUCGUAUC.GUUAGGCC.G.UUGGA.GAUACGAA...AG...CAAUGCCUUAUGUAUU
env-507    UUUUGCUUGCCGCCGGGUAAG.C.U.G.UA.....................................AAGCAUUG.AUUUCAUAUC.GAUAGGCC.G.UUGCA.GAUAUGAA..AGU...CAGUGCUUUAUUUUUU
Msp-1-1    GAUUGUUUGCCGCCGGGUAAA.U...................UUU.......................AAGCAUUG.GUUUCGUAUC.GAUAGGCC.A.UUGAA.GAUACGAC..AAU...CAGUGCUUUUCUUUUG
env-508    UUAUGUUUGCCGCCGGGUAAA.C.U.U.UA.....................................AAACAUUG.AUUUCGUAUC.GUUAGGCC.A.UAGAA.GAUGCGAA..AGU...CAAUGUUUUUUAUUUU
Mti-1-1    UAAUGUUUGCCGCCGGGUAGA.C.U.U.UA.....................................AAACAUUG.GUUUCGUAUC.GUUAGGCC.A.UAGAA.GAUACGAA..AAU...CAAUGUUUUUAUUUUG
env-509    GAUUUCUUGCCGCCGGGUAAG.....................UA.......................UAAGCGCUG.GUUUCGUAUC.GUUAGGCC...AUGAA.GAUACGAA...AC...GGGCGCUUAUUUUUUU
Csp-4-1    UAUCAACUGCCGCCGGGUAGA.U.U.A.UA.....................................AGCGUUGA.AGUUCGUAUC.GUUAGGCC.A.UAGAA.GAUACGAG...A....UCAGCGCUUUUAUUCU
env-510    ACUUUUUUGCCACCGGGUAAA.A.A.A.G.ACA...GGAGU...UUUG............ACUCCAGAGCGCCAGA...CUUUCUAUC.AUUAGGUC.A.UAGAA.GAUAGGAA...G....GUCUGGCAUUUUUUAU
env-511    UGAAAUUUGCCGCCGGGCAA......................UC........................AGCGUUGA.ACCUUGUAUC.CAUAGGCC.U.UAGAG.GAUACAG....AU...UUGGCGCUUUUUUUGU
env-512    GAUGGAUUGCCGCCGGGUAAG...............................................AGCGUUAA.AUUUUGUAUC.GUUAGGCC.G.AAGCA.GAUACAAA...AC...UUAACGCUCUUUUUCU
env-513    UUGAUUUUGCCGCCGGGUAAA.U.U.A.AA......................................AGCAUUGU.AAUUCAUAUC.GUUAGGCC.A.UAGAA.GAUAUGAA...AUU..AUAAUGCUUUUUAUUU
env-514    GUUGGCUUGCCGCCGGGUAAG.C.A.U.A..............GA.......................AAACGCUGG.AUUUCGUAUC.GUUAGGCC.A.UUGAA.GAUACGAA..AUG...CCAGCGUUUUUCUUUU
env-515    AUAAACUUGCCGCCGGGUAAG.C.A.A.CU.............GU.......................AAACGUUGA.UAUUCGCAUC.GUUAGGCC.U.UUGAA.GAUGCGUU..AUA...UCUGCGUUUUUUGUUU
env-516    UUAUACUUGCCGCCGGGUAAG.C.A.C.G..............UUU......................UUACGUUGG.UUUUCGUAUC.GUUAGGCC.U.UUGAA.GAUACGAG..AUA...CCAGC.UUUUUUUAUG
env-517    AUUUUUUCGCCGCCGGGUGAA.G.A.A................UGCGUAAG.................GAACGCUGG.GUUUCGUAUC.GUUAGGCC.U.UUGAA.GAUACGAG...AUU..UCAGCGUUUUUCUGUU
env-518    AUGUACUUGCCGCCGGGUAAG.G.G.G.A.......................................AACGUUG.AUGCCGUAUC.GUUAGGCC.U.UUGAA.GAUACGGG...AUG..CCGGCGUUUUUUUGUU
env-519    UGUUAUUUGCCGCCGGGUAAA.U.U.U.AA.......................................GCGUUGC.AUACUGUAUC.GAUAGGCC.A.UAGAA.GAUACGGU...GU...UCAGCGCUUUUUGUUU
env-520    AAUAUUUUACCGCCGGGUAAA.G....................CUGAG....................AAGCGUUGG.AUAUCAUAUC.AUUAGGCC.U.UAGAA.GAUAUGAU..UAU...CUGCCGCUUUUAUUUU
env-521    GUAUUUUUACCGCCGGGUAAA.A.A..................CUU......................AAGCGUUGG.ACUUCGUAUC.GUUAGGCC.A.UAGAA.GAUACGAG...GCU..CUGGCGCUUUUAUUUU
env-522    CGUUCUUUGCCACCGGGUGAA.G.A.A................GUU......................AAGCGUCAU.AUUUCCUUUC..GUAGGUC.U.UUGAA.GAUAGGAA...GUA..GGGGCGCAUUUUUUUU
env-523    AAUCCUCUGCCGCCGGGUAGA.G.G.G................GA.......................UUGCACCAG.ACUUCAUAUC.GUUAGGCC.G.UUGAA.GAUAUGAA...GUU..CUGGUGUAUUUUUAUU
env-524    AUGAUCUCGCCGCCGGGUGAG.G.A.C.G..............CUGAAAAAAAAGC.............AGCGCCGG.AUUUCGUAUC.GUUAGGCC.A.UUGAA.GAUGCGGA...AU...GCGGCGCUUUUUGUUU
env-525    ACCACACUGCCGCCGGGCAGU.U.G.C.G..............CGAG.....................GAGCGCCGG.AUUUCGUAUC.GUUAGGCC.U.UAGAA.GAUAUGAG...AUA..CCGGCGCUUGUUUUAU
env-526    ACCACACUGCCGCCGGGCAGU.U.G.C.G...............CGAG....................GAGCGCCGG.AUUUCGUAUC.GUUAGGCC.A.UUGAA.GAUGCGGA...AU...GCGGCGCUUUUUGUUU
Cba-2-1    AGCAACUCACCGCCGGG.GAG.U....................AUGG......................AGCGUUGG.AUAUCGUAUC.GUUAGGCC.A.UAGAA.GAUACGAG...AU...UCGGCGCUUUUUGUUA
env-527    AGCUUUUUGCCACCGGGUAAA.A.A.G.AU.............U........................AAGCGUCAG.ACUUUCUAUC.AUUAGGUC.U.UUGAA.GAUAGAGA...GU...UUGAUGUUUUUUUGUA
env-528    GUAGAUUUGCCGCCGGGUAAA.A.A.C.G..............ACUUU....................AAGCGUUGU.GCUUUGCAUC.AUUAGGCC.U.UUGAA.GAUGCAAA..AGAC..UCAGCGCUUUUAUUUU
env-529    GGAUAUUUACCGCCGGGUAAA...............................................AAGCGUUGG.GUUUUGUAUC.AGUAGGCC...UUGAA.GAUACAAA...ACAG.CCAGCGCUUUUCUUUU
Lsp-7-1    AAGAACUUGCCGCCGGGUAAG.U....................UAA......................AAGCGUUGA.CUUUUGCAUC.AGUAGGCC...UUGUA.GAUGCAAA...AUA..UCAGCGCUUUUCUUAU
Dsp-2-1    AAAGACUUACCGCCGGGUAA.......................UC.......................AAGCGUUGG.AUUUUGUAUC..GUAGGCC.U.UUGAA.GAUACAAA...AUG..UCAGCGCUUUUCUUUU
env-530    AAGAAAUUGCCGCCGGGUAA.................................................AGCGUUGG.AUUUUGUAUC.GAUAGGCC.A.UAGGA.GAUACAAU...AU...UCGGCGCUUUUUUAUG
env-531    GGUUGUCUGCCACCGGGUAGG.C.A.A.C..............AG.......................AAGCGUCAG.ACUUUCUAUC.GUUAGGUC.U.CAGAA.GAUGGAAA...GC...CUGACCUUUUUUAUGU
Lba-2-1    GCUCUUUUGCCACCGGGUAAA.G.A.G................AUA......................AAACGUCAG.GCUUCCUAUC.GUUAGGUC.U.UAGAA.GAUAGAAA...GUU..CUGACGUUUUAUUUGU
env-532    ACUUUUCUGCCACCGGGUAGA.A.G.A.GU.............C........................AAUCGCUGA.ACUUUCUAUC.GUUAGGUC.U.AAGAA.GAUAGAAA...GCU..UCGGCGUUUUUUAUUU
env-533    UUGAAUUUGCCGCCGGGUAAA.U.G.A.CA.......................................GCGUUGA.AUUUUGUAUC.GUUAGGCC.U.UUGAA.GAUAGAAA...GUU..CUGGCGCUAUUUUUUU
env-534    AAUUCUUUGCCGCCGGGUAAA.G.G.G.UU......................................GUGCGUCAG.ACUUCCUAUC.AGUAGGCC.A.UUGAA.GAUAGGAG...GG...CUGGCGUUUUUUGUUG
env-535    ACUUUUUUGCCACCGGGUAAA.G.A.A.G..............AA.......................CAGCGUCGG.ACUUUCUAUC.GUUAGGUC.U.UAGAA.GAUAGAAA...UUG..CCGGCGCUUUUUUGCU
env-536    UAUCCUCUAUCGCCGGAUAGA.G.G.A................AUA......................AAGCGUCAG.ACUUCAUAUC..GUAGGCC.C.GUGAA.GAUAUGAA...GUU..CUGGCGCUUUUAUUUU
env-537    GUUUCUUUGCCGCCGGGUAAA.G.A.G.UU......................................AAGCGCCGG.UCCUUCUAUC.GUUAGGCC.A.UUGAA.GAUAGAAG..GGA...CCGGCGCUGUUUUUGU
env-538    ACUUCCUGCCGCCGGGUAGG.G.A.U.G...............CA........................AGCGUCAG.GUUUUCCAUC.AUUAGGCC.U.UUGAA.GAUGGAAA...ACU..UUGACGCUGUUUUUUU
env-539    GUUUCUUUACCACCGGGUAAA.G.G.A.UU.............C........................AAGCAUCAG.ACUUUCUAUC.GUUAGGUC.A.UUGAA.GAUAGGAA...GUA..CUGGUGCUUUAUUUUU
env-540    UUUUUUCUGCCACCGGGUAGA.A.A.A.AA.............AU.......................AAGCGUCAG.AGUUUCUAUC.GUUAGGUC.U.UAGAA.GAUAGAAA...CC...AUGGCGCUUUAUUUUU
env-541    ACUUUCUUGCCACCGGGUAAA.A.A.A.GU.............U........................AAGCGUCUA.AAUUUCUAUC.GUUAGGUC.U.UAGAA.GAUAGAAA...UA...AGGACGCUUAUUUUUU
env-542    UCUUUUCUGCCACCGGGUAGG.A.A.A.GG.............U........................AAGCGUCAG.AAUUUCUAUC.GUUAGGUC.U.GAGAA.GAUAGGAA...UC...CUGGCGCUUUUUAUUU
env-543    ACUCUUCUGCCACCGGGUAGG.A.G.A.GU.............U........................AAGCGUCAG.AUUUUCUAUC.GUUAGGUC.U.UAGAA.GAUAGAAA...AUA..CUGGCGCUUUCUUAUU
env-544    ACUUUUCUGCCACCGGGUAGG.A.G.A.GU.............U........................AAGCGUCAG.AUUUUCUAUC.GUUAGGUC.U.UAGAA.GAUAGGAA...AUG..CUGGCGCUUUAUUUUU
env-545    ACUUUUCUGCCACCGGGUAGG.A.A.A.GU.............U........................AAGCGUCAG.AAUUUCUAUC.GUUAGGUC.A.AAGAA.GAUAGAAA..UUU...CUGGCGCUUUAUUUUG
env-546    ACUUUUCUGCCACCGGGUAGG.A.A.A.GU.............U........................AAGCGUCAG.GAUUUCUAUC.GUUAGGUC.U.UAGAA.GAUAGAAA...UUU..CUGGCGCUUUAUUUUU
env-547    ACUUUCCUGCCACCGGGUAGG.G.G.A.GU.............U........................AAGCGUCAG.AAUUUCUAUC.GUUAGGUC.U.GAGAA.GAUAGAAA..GCU...CUGGCGCUUUAUUUUU
env-548    ACUUUCCUGCCACCGGGUAGG.A.A.A.GU.............U........................AAGCGUCAG.AAUUUCUAUC.GUUAGGUC.U.GAGAA.GAUAGAAA..GUU...UUGGCGCUUUUUUUUA
env-549    ACUUUCCUGCCACCGGGUAGG.G.A.A.GU.............U........................AAGCGUCAG.AGUUUCUAUC.GUUAGGUC.A.UAGAA.GAUAGGAA...UU...UUGGCGCUUUUUUUUU
Pma-1-1    ACUUCUUUGCCGCCGGGUAAA.G.G.A.GU.............UU.......................UGGCGCCAG.ACUUUCUAUC.GUUAGGCC.U.UUGAA.GAUAGAAA..GCG...CUGGCGCUUUUUAUGU
env-550    UAUUCUUUACCGCCGGGUAAA.G.A.A................AUU......................GAGCGUCAG.ACUUCAUAUC.AUUAGGCC.U.AAGAA.GAUAUGAA...GUA..CUGGCGUUUUUUUAUU
Lba-1-1    UAUCCUUUACCGCCGGGUAAA.G.G.G................GUU......................AAGCGUCAG.ACUUCAUAUC.GUUAGGCC.A.CGGAA.GAUAUGAA...GUU..CUGACGCUUUUUAUUU
Csp-9-1    UAUUCUUUACCGCCGGGUAAA.G.A.A.U..............U........................CAGCGUCAG.ACUUCAUAUC.GUUAGGCC.G.UGGAA.GAUAUGAA...GUU..CUGGCGCUUUUUAUUU
Cph-1-1    UAUUCUUUACUGCCGAGUAAA.G.A.A................GUU......................AAGCGUCAG.AUUUCAUAUC.AUUAGGCC.A.UAGAA.GAUAUGAA..ACU...CUGGCGCUUUUUAUUU
Lph-1-1    UAUUCUUUACUGCCGAGUAAA.G.A.A................AUU......................AAGCGUCAA.GUUUUAUAUC.GUUAGGCC.A.UAGAA.GAUAUGAA...ACU..CUGGCGCUUUUUCUUG
env-551    ACUUCUUUGCCGCCGGGUAAA.G.A.A.UU.............U........................AAGCGUCAG.AAUUUCUGUC.AUUAGGCC.U.UUGAA.GACAGGAA...UC...CUGGCGCUUUUUAUAU
Cst-1-1    GUUUCUUUGCCGCCGGGUAAA.G.A.A.AU..............CU......................AGGCGUCAG.CUUUCCUAUC.GUUAGGCC.A.UUGAA.GAUAGGAA..GUG...CUGGCGCUUUUGUUUU
env-552    UGCUUUUUGCCGCCGGGUAAG.A.A.G.U..............U........................GAGCGCCAG.GCUUUCUAUC.GUUAGGCC.U.UUGAA.GAUAGAAA...GCU..CUGGCGCAUUUUUUGU
env-553    CACUUUUUGCCGCCGGGUAAG.G.A.G.U..............U........................AAGCGUCAG.GCUUUCUAUC.GUUAGGCC.U.UUGGA.GAUAGAAG...GUU..CUGGCGUUAUUUUUGU
env-554    ACUUCCUGCCGCCGGGUAGG.A.A.A.UU..............U........................AAGCGUCAG.ACUUUCCAUC.GUUAGGCC.U.UUGAA.GACGGAAA...GCU..UUGGCGCUAUUUUUAU
env-555    ACUUCUUUGCCGCCGGGUAAA.G.A.A................UGU......................AAGCGCCAA.AUUUUCUAUC.AUUAGGCC.G.UUGAA.GAUAGAAA...AUU..CUGGCGCUAUUUUUAU
env-556    CACUCCUUGCCGCCGGGUAAG.G.U.G.U..............U........................AAGCGUCAG.UCUUUCUAUC.AUUAGGCC.A.UUGAA.GAUAGAAA...GAG..CUGACGUUAUUUUUUU
env-557    CACCUUUUGCCGCCGGGUAAA.G.A.G................GAU......................AUGCGUCAG.UCUUUCUAUC.GUUAGGCC.A.UUGAA.GAUAGAAA..GUG...CUGGCGUUAUUUUUUC
env-558    CGUCCCUUGCCGCCGGGUAAG.G.G.G.U..............UA........................AGCGUCAG.UCUUUCUAUC.GUUAGGCC.A.UUGAA.GAUAGAAA..GUG...CUGGCGUUAUUUUUUU
Mfo-1-1    CAUUCCUUGCCGCCGGGUAAG.G.A..................UUUA.......................AGCGUCAG.UCUUUCUAUC.GUUAGGCC.A.UUGAA.GAUAGAAA..GCG...CUGGCGCUAUUUUUUU
env-559    ACUUCUUUACCGCCGGGUAAA.G.A.A.AU.............U........................AAGCGUCAG.GCUUCCUAUC.AUUAGGCC.A.UUGAA.GAUAGGAA...GUU..CUGGCGCUAUUUUUUU
Csp-13-1   ACUUCUUUGCCGCCGGGUAAA.G.A.A.GU.............U........................AAGCGUCAG.UUUUCCUAUC.GUUAGGCC.U.UUGAA.GAUAGGAA...AUU..CUGGCGCUUUAUUUUU
env-560    ACUUCUUUGCCGCCGGGUAAA.G.A.A.GU.............U........................AAGCGUCAG.GCUUUCUAUC.GUUAGGCC.A.UUGAA.GAUAGGAA...GUU..CUGGCGCUUUAUUUUU
env-561    ACUUCUUUGCCGCCGGGUAAA.G.A.G.CU......................................AAGCGUCAG.AUUUUCUAUC.GAUAGGCC.U.UUGAA.GAUAGAAA....U...CUGGCGUUCUUUUUUU
env-562    UAUUUCUUGCCGCCGGGUAAG.A.A.G.U..............U........................AAGCGUUAG.AUUUUCUAUC.AUUAGGCC.U.UUGAA.GAUAGAAA...AUU..CUGGCGCUAUUUUUUU
env-563    ACUUUCUUGCCGCCGGGUAAG.G.A.G................UU.......................AAGCGUCAG.GCUUUCUAUC.GUUAGGCC.U.UUGAA.GAUAGAAA...GCU..CUGGCGCUAUUUUUUA
env-564    UUUUUCUUGCCGCCGGGUAAG.A.A.A................UU.......................AAGCGUUAG.ACUUUCUGUC.GUUAGGCC.U.UUGAA.GAUAGAAA...GCU..CUGGCGCUAUUUUUUU
env-565    ACUUCCUUGCCGCCGGGUAAG.G.A.G.UU......................................AAGCGUCAG.ACUUUCUAUC.GUUAGGCC.U.UUGAA.GAUAGAAA...GUU..CUGACGCUAUUUUUGU
env-566    ACUUCUUUGCCGCCGGGUAAA.G.A.G.UU......................................AAGCGUCAG.GCUUUCUAUC.GUUAGGCC.U.UUGGA.GAUAGAAA...GUU..CUGACGCUAUUUUUUG
env-567    ACUUCUUUGCCGCCGGGUAAA.G.A.G.UU.............G.........................AGCGUCAG.GCUUUCUAUC.GUUAGGCC.U.UUGAA.GAUAGAAA...GUU..CUGGCGCUAUUUUUAU
env-568    UAUUCCUUGCCGCCGGGUAUG.G.A.A.U..............U........................CAGCGCCAG.ACUUUCUAUC.GUUAGGCC.A.UUGAA.GAUAGAAG...GCU..CUGGCGCUUUUUUUCA
env-569    CGCUCUUUGCCGCCGGGUAAA.G.A.G.A..............A........................AAGCGCCAG.GCUUUCCAUC.GUUAGGCC.A.UUGAA.GAUGGAAA..GAU...CUGGCGCUAUUUUUAA
env-570    AUUUCUUUGCCGCCGGGUAAA.G.A.G.UU......................................GAGCGUCAG.AUUUUCUAUC.GUUAGGCC.U.UUGAA.GAUAGAAA....U...UUGCCGCUUUAUUUUU
env-571    ACUUUUCUGCCACCGGGUAGA.G.A.A.G..............AU.......................AUGCGUCAG.ACUUUCUAUC.GUUAGGUC.U.UAGAA.GAUAGAGG...GUG..CUGGCGCUUUUUUUGA

           ...........<<<.....................................................................................>>>..........................................
           ...........20......................................................................................02..........................................
           <<<<<<<<<<<....>>>>>>.>.>.>.>>......<<<<<<<<.............>>>>>>>>>...<<<<<<<<.<<<<<<<<<<..................>>>>>>>>...>>...>>>>>>>>>.......
           22.22222222....222222.2.2...22......1111110..............0111111...22222222.2222222221..................12222222...22...22222222........
           ●●●●●●YUGCCRCCGGGUAR●-◦-◦-◦-◦--------------◦◦◦-------------------◦◦R●●●Y●●-●●●●YRY●YC-RUUAGGYC-●-U●GAA-GR●RYR●●----●●----●●R●●●YYUUUUUY●●
```

```
Lsp-5-1   ACUCCCUGCCGCCGGGUAUG.G.G.A.G..............CU.....................AUGCGUCAG.AUUUUCUAUC.GUUAGGCC.A.AUGAA.GAUAGAAA...AUU..CUGGCGCUAUUUUUUU
env-572   CAGUUUUUACCGCCGGGUAAG.A.A.U.U..............U......................AUGCGUCAG.GCUUUCUAUC.GAUAGGCC.A.UAGAA.GAUAGAAA...GG...CUGGCGUUUUUUUGUGC
Mpe-1-1   GCUGUUUUGCCGCCGGGUAAA.A.C.A.GC.............G......................GAGCGUCAG.ACUUUCUAUC.AUUAGGUC.U.UUGAA.GAUAGAAA....G...CUGGCGCUUUUUAUGA
env-573   UAUCCUUUAUCGCCGGGUAAA.G.G.A................AUC....................AAACGUCAG.ACUUCUAUC.GAUAGGCC.A.UAGGA.GAUAUGAA..GAU...CUGGCGUUUUUUUUAU
env-574   UCUCUUUUGCCGCCGGGUAAA...G.A.GA.............UU.....................AUGCGUCAG.ACUUCUAUC.AUUAGGUC.A.UAGAA.GAUAGAAA...GUG..CUGACGCUUUUUGUAU
env-575   UGUUCUUUGCCGCCGGGUAAA.G.A..................CAUU...................AAGCGCCGG.ACUUCUAUC.GUUAGGCC.G.UUGAA.GAUAUGAA...GAC..CCGGCGCUUUUAUUGU
env-576   UACUAUUUGCCGCCGGGUAAA......................CCA....................GAAUGUUGG.AUUUUGUAUC.AGUAGGCC.G.UUGGA.GAUACAAA...GUG..CCGGCAUUUUUUUAUU
env-577   GCAACUUUGCCGCCGGGUAAA.G.A.U................UAUUU..................UAGCGUUGG.AUUUUCUAUC.AAUAGGCC.U.UUGAA.GAUAGAAA..UAGCA.CCAAGGCUUUUUUAUU
env-578   AAUUUGUUGCCGCCGGGUAAU......................U.......................AACGUUAU.AUUUCAUGUC.AUUAGGCC.U.UUGAA.GACGUGGA...AU...AUAGCGUUUUUUAUU
env-579   GAUUCUUUGCCGCCGGGUAAA.G.A.A.U..............GA.....................AGCGCCAGA.ACCUUCAUAC.GUUAGGCC.A.UUGAA.GAUAGAAG...GU...CCUGGCGCUGUUUUAU
env-580   UAUUUCCCGCCGCCGGGUGGG.G.A.G.U..............UC.....................GGGCGUCAG.AUUUCAUAUC.AUUAGGCC.A.UUGAA.GAUGUGAA..AUG...CUGGCGUAUUUUUUU
Bma-1-1   CAGAAAUUACCGCCGGGUAAA.U.U..................U......................AAAGCAUUG.GAUUCAUAUC..GUAGGCC.A.UUGAA.GAUAUGAA...UGC..CAAUGCUUUUUUAUGG
env-581   UAUAUCUUGCCGCCGGGUAAG.A.A.A................AC.....................CGAACGUUG.GAUUCGUAUC.AGUAGGCC.U.UUGAA.GAUACGAA.CUUC...CAGCGUUUUUUAUUUG
env-582   CAUGGAUUGCCGCCGGGUAA..............................................CGUCUCAA..U.UCAUUCC.GUUAGGCC.U.UAGAA.GGAGUGAU...GC...GUGAGGCGUUUUAUUA
env-583   AUAGGAUUGCCGCCGGGUAA..............................................CGUUUUG..UC.UCAUUCC.AUUAGGCC.A.UAGAA.GGAAUGGA...GA...ACAAAGCGUUUUUUUU
env-584   CGAGAAAUGCCGCCGGGUAU..............................................GCUUUUU.GC.UCAUUCC.GUUAGGCC.U.UAGAA.GGAAUGUG...GC...AGGAGGCGUUUUUUUA
env-585   UAAUUUUUAUCGCCGGAUAAA.A.A.U.U..............U......................GAGCGUCAC.ACUUCCCAUC..GCAGGCC.U.UUGAA.GAUGGGAA...GC...AUGGCGCUCUUUUAUU
env-586   CACUAUUUACCGCCGGGUAAU......................C......................AAGCAUUGA.AGUUCAUAUC..GUAGGCC.U.UUGAA.GAUAUGAG...CA...UCGAUGCUUUUUUUUG
Csp-14-1  AAUAAUUUAUCGCCGGAUAAA.G.A.U.UU.............................. ......AGACGUUUA.UUUUUGUAUC..GUAGGCC.U.UUGAA.GAUACAAA..AUA...UAAACGUCUUUUAUUU
env-587   AAUCAUCUGCCGCCGGGCAGA......................AUC.....................AUGCAUCG.GCUCCGCACC.GUUAGGCC.U.UUGAA.GGUGCGGGGAGGC...CGAUGCAUUUUCUUUU
env-588   GAGUAUCUGCCGCCGGGCAGA.A.C.A.UC.....................................AUGCAUCG.GUCCUGCACC.GUUAGGCC.U.UUGAA.GGUGCGGG.AAGC...CGGUGUAUUUUCUUUU
env-589   UAUCCUCUGCCGCCGGGCAGA.A.A.A................CU......................GUGCAUCG.GUCCGUAUC.GUUAGGCC.U.UAGAA.GAUACGGG...GUGACCGAUGUAUUUUCUU..
env-590   AACUCUCUGCCGCCGGGCAGU.G....................C.........................GCGUUG.GUUCCGUAUC.GUUAGGCC.U.UAGAA.GAUACGGG...CGC...CAGCGCUUUUUUGUUG
env-591   UAAUAUCUGCCGCCGGGCAGG.U.U.U.U.............................UACAU...CG.GUUCCGUGUC.GUUAGGCC.U.UAGAA.GAUACGGG...GU...UGCCGGUGUAUUUUUU
Bpi-1-1   UUAAAUUUGUCGCCGGAUAAA.U.U.U.A..............UUUA...................GAACGUUUG.UAUUCAUACC..GUAGGCC.U.UAGAA.AGUAUGAA...CG...CAAACGUUUUUUUAUU
Csy-1-1   CGUUAUUUGCCACCGGGUAAA.U.A.A.U..............C......................AAGUGUCUG.AACAUACUCC.GUUAGGUC.A.AAGAA.GGAGCGUG...UU...CAGGCACUUUUUUGCU
env-592   AAUUAUUUGCCACCGGGUAAA.U.A.A.UU.....................................CAGUGUCUG.AACACACUCC.GUUAGGUC.U.AAGCA.GGAGUGGG...UU...CAGGCACUUUUUAUAU
Csp-5-1   UAAUAUUUGCCACCGGGUAAA.U.A.A.GA.....................................UAGUGUUUA.GGUAUAUUCC.AGUAGGUC.A.UAGAA.GGAAUAUG...UU...UAAGCACUUUUUUGAU
env-593   UUUUAUUUGCCACCGGGUAAA.U.A..................UUU....................AAGGUAUCA.CGGGUAUUUC.GUUAGGUC.U.UAGAA.GAAAUACU...UU...UGAUACCUUUUUAUUU
env-594   AGAUAUUUGCCACCGGGUAAA.U.A.U.C..............AUC.......................GUGUCA.AAAGUACUUC.GUUAGGUC.U.UAGAA.GAGGUGCU...UU...UGGCAUUUUUGCGUUU
Tma-1-1   AUAUGCUUGCCACCGGGUAAG.C.G.U.A..............AAAUAA.................AAAGUGCCG.AAAGUACUUC.GUUAGGUC.G.UAGAA.GAAGUGCU...UC...UGGCACUUUUUUUGUU
Npe-1-1   AUAUGCUUGCCACCGGGUAAG.U.A.U.A......................................AAAGUGUCA.AGAGUACUUC.GUUAGGUG.U.UAGAA.GAAGUGCU...UU...UGACACUUUUUUGUCA
Cfo-1-1   AUAUACUUGCCACCGGGUAAG.U.A.U.A..............A......................AAAGUGUCA.AGAGUACUUC.AUUAGGUC.U.UAGAA.GAAGUACU...UU...UGGCACUUUUUUAUUU
Gfe-1-1   GUAUGCUUGCCACCGGGUAAG.U.A.U.A......................................AAAGUGUCA.AGAGUACUUC.AUUAGGUC.U.UAGAA.GAAGUACU...UU...UGGCACUUUUUUUUUA
Nfe-1-1   CUAUACUUGCCACCGGGUAAG.U.A.U.A..............A......................AAAGUGCCA.AAAGUACUUC.GUUAGGUC.U.UAGAA.GAAGUGGU...UU...UGGCACUUUUUUUUCGU
Psp-1-1   GUAUGCUUGCCACCGGGUAAG.C.A.U.UU......................................AAGUGCCA.UAAGUACUUC.GUUAGGUC.A.GAGAA.GAAGUGCU...UU...UGGCACUUUUUUUUAA
env-595   AUUGCUUUGCCACCGGGUAAA.G.C.A.A..............AUUA...................AGGUGCUCA.GAUACAUUUC.GGUAGGUC.U.UAGGA.GAAAUGUG..AUCU..UGAGCACCUUUUUUAU
env-596   UCGCUUUUGCCACCGGGUAAA.A.G.C.UG.............C......................AAGGUGUUC.ACAGCAUUCC.GUUAGGUC.U.AAGAA.GGAAUGCU...AU...GAGCACCUUGUUUUUC
Til-1-1   CUCUUUUUGCCACCGGGCAAG.G.A.G.U..............UCAA...................GGUGU.UCA.GAAAUAUUCC..GUAGGUC.U.UUGAA.GGAAUGUU...UC...CGGGACACCUUUUUUA
env-597   UUUACUUUGCCGCCGGGUAAA.G.G.U.UA.............A......................UAAGUGUUC.UAAACAGUCC.GUUAGGCC.U.UAGAA.GGGGUGUU..UUU...GAACACUUUUUUACAU
env-598   UAAACUUUGCCACAGGGUAAA.G.A.U.UU.............CA.......................AGGUGUUCUGAAGCAUUCCGUUAGGUC.U.UAGG..GGAUGUUU..UCAG..GGCACCUCUUUUUGUU
env-599   CAUUCUUUGCCACCGGGUAAA.G.A.A.U..............UG.....................AGGUGUUCU.ACAA.CAUUCCGUUAGGUC.A.GGGAG.GAAUGUUU...GA...GGG.CGCCUUUUUGUU
env-600   UCCUCUUUGCCACCGGGUAAA.G.A.G.GG.............UC.....................AGGUGUUCU.UUUGCACUCC.GUUAGGUC.A.AAGAA.GGAGUGCA...AA...GGAACACCUUUUUUGA
Tfl-1-1   CUCUUUUUGCCACCGGGCAGA.G.A.G................UUCA...................AGGUGUUCA.GAAACAUUCC..GUAGGUC.U.UUGAA.GGAGUGUU...UCC..GGGACACCUUUUUUAU
Lna-1-1   GAAACUUUACCACCGGUGAA.G.U.U.UU..............UA.....................AGGUGUUCC.ACAACAUUCC.AUUAGGUC.U.UGGAA.GGAAUGUU..GCU...GGAACACCUUUUUUGUU
env-601   AUAACUUUGCUACCGGGUAAA.G.U.U.A..............AUUCA..................AGGUGUUCU.UAAGCAUUCC.AAUAGGUC.U.UAGAA.GGGAUGAU...UA...UGAACACCUUUUUACU
env-602   AUAGCUUUGCCACCGGGUAAA.G.U.U.A..............CUUCA..................AGGUGUUCU.UAAGCAUUCC.AAUAGGUC.U.UAGUA.GGAAUGUU...UA...UGAGCACCUUUUUACU
env-603   CUUGCUUUGCCACCGGGUAAA.G.C.A................UAUA...................AGGUGUUCU.ACAACAUUCC.GUUAGGUC.U.UAGAA.GGAGUGUU...GC...GGAACACUUUUUUUCG
env-604   UUAACUUUGCCACCGGGUAAA.G.U.U.G..............UAUU...................AGGUGUUCU.UAAACAUUCC.GAUAGGUC.G.UAGAA.GGGAUGUU...AU...AGGACACCUAUUUUAG
env-605   CACACUCUGCCACCGGGUAAA.G.U.G.U..............A......................AGGUGUUCU.UAAAUAUUCC.AUUAGGUC.A.UAGAA.GGAAUAUU...UU...AGAACACUUUUUUUUA
env-606   AUAAUUUUGCCACCGGGUAAA.G.U.U.A..............AUUCA..................UGGUGUUCU.AAAACAUUCC.GUUAGGUC.U.UAGAA.GGAAUGUU...UU...AGAACAUUUUUUUGUU
env-607   UAUUCUUUGCCACCGGGUAAA.G.A.U.U..............UA.....................AGGUGUUCU.GAGGUAUUCC.GUUAGGUC.U.UAGAA.GGAAUACU..UUC...GGGACACCUUUUUGUG
[st-1-1   UAAGCUUUGCCACCGGGUAAA.G.U.U.U..............UCA....................GGGCGUUCU.GAAACAUUCC.GUUAGGUC.U.UAGAA.GGGAUGUU...UA...AGGACGCCUUUUUGAU
env-608   UUAACUUUGCCACCGGGUAAA.G.U..................AUUAUA.................AGGUGUUCU.GAAACAUUCC.AUUAGGUC.U.UGGAA.GGAAUGUU...UU...AGGACAUCUUUUUUGU
Csp-11-1  UUAACUUUGCCACCGGGUAAA.G.U..................AUUAUU.................AGGUGUUCU.GAAACAUUCC.GUUAGGUC.A.UGGAA.GGGAUGUU...UU...AGGACACUAUUUUUAA
Dph-1-1   UCAUUUUUGCCACCGGGUAAA.G.A.U.UG.............UA.....................UAGUGUUCU.GAAACAUUCC.GUUAGGUC.U.UAGAA.GGAAUGUU..UUU...AGAGCACUUUUUUACU
env-609   UAAGCUUUGCCACCGGGUAAA.G.A.U.U..............UU.....................AAGGUGUUCUGGAACAUUCC.GUUAGGUC.U.UAGAA.GGAAUGUU...AG...GGACACCUUUUUAUUU
env-610   UGAGCUUUGCCACCGGGUAAA.G.A.A.UG.............UCA....................AGGUGUUCU.CAAAUAUUCC.GUUAGGUC.G.UAGAA.GGAAUAUU..UUA..AGAACACCUUUUUAUU
[in-1-1   UGAGCUUUGCCACCGGGUAAA.G.A.A.UU.............UCA....................AGGUGUUCU.GAAAUAUUCC.GUUAGGUC.G.UAGAA.GGAAUGUU..UUA..AGAACACCUUUUUUAU
[sp-1-1   UGUUCUUUGCCACCGGGUAAA.G.A.A.U..............UUUA...................AGGUGUUCU.GAAAUAUUCC.GUUAGGUC.G.UAGAA.GGAAUAUU..UUA..AGAACACCUUUUUUAU
env-611   UAAACUUUGCCGCCGGGUGAA.G.A.A.U..............UCA....................AGGUGUUCU.GCAACAUUCC.AUUAGGUC.U.UAGAA.GGAAUGUU..GUC...GGAACACCAUUUUUGU
Cin-2-1   UAAACUUUGCCACCGGGUAAA.G.A.U.U..............UUA....................AGGUGUUCU.GAAGCAUUCC.GUUAGGGC.U.UAGAA.GGGAUGUU..AUC...AGGGCACUUUUUUACU
Csp-3-1   UAGUCUUUGCCACCGGGUAAA.G.A.A.U..............UUC....................AGGUGUUCU.GAAACAUUCC.GUUAGGUC.U.UAGAA.GGAAUGUU....C...GGAACACCUUUUUUGA
env-612   UAAACUUUGCCACCGGGUAAA.G.U.U.U..............UUA....................AGGUGUUCU.GAAACAUUCC.GUUAGGUC.U.UGGAA.GGAAUGUU..UUU...AGGACACUUUUUUAUU
env-613   UAGACUUUGCCACCGGGUAAA.G.U.U.U..............UUA....................AGGUGUUCU.GAAACAUUCC.GUUAGGUC.U.UAGAA.GGAAUGUU..UUU..AGGGCACCUUUUUACU
env-614   UAGACUUUGCCACCGGGUAAA.G.U.U.U..............UA.....................AGGUGUUCU.GAAACAUUCC.GUUAGGUC.U.UAGAA.GGAGUGUU..UUU...AGGGCACUUUUUUAUU
Mha-1-1   UAAACUUUGCCACCGGGUAAA.G.A.G.U..............UUA....................AGGUGUUCU.GAAACAUUCC.AUUAGGUC.U.UAGAA.GGAAUGUU..GUC...GGAACGCCUUUUUUUA
env-615   UAAUCUUUGCCACCGGGUAAA.G.A.U.U..............UUA....................AGGUGUUCU.GAAACAUUCC.AUUAGGUC.U.UAGAA.GGAAUGUU..GUU...GGGAUGCCUUUUUUAU
env-616   UAAACUUUGCCACCGGGUAAA.G.A.U.U..............UUA....................AGGUGUUCU.GAAACAUUCC.GUUAGGUC.U.UAGAA.GGAAUGUU..GUU...GGGACACCUUUUAUUU
env-617   UAUUCUUUGCCACCGGGUAAA.G.A.A.U..............UUA....................AGGUGUUCU.GAGCAUUCC.GUUAGGUC.U.UAGAA.GGAAUGUU..UUU...AGGACGCCUUUUUAUU
env-618   UGUUCUUUGCCACCGGGUAAA.G.A.A.U..............UUA....................AGGUGUUCU.GAAGCAUUCC.GUUAGGUC.U.UGGAA.GGAAUGUU..UUU...GGAACACCUUAUUUAU
env-619   UAAGCUUUGCCACCGGGUGAA.G.A.U.U..............UU.....................AAGGUGUUC.AAAACAUUCC.GUUAGGUC.U.UAGAA.GGGAUGUU...GG...GAACAUCUUUUUAUUU
env-620   UAAGCUUUGCCACCGGGUAAA.G.U.U.U..............UU.....................AAGGUGUUC.AAAACAUUCC.GUUAGGUC.U.UAGAA.GGAAUGUU...AG...GGACACCUUUUUAUUU
Air-1-1   AAUGUUUUGCCACCGGGUAAA.G.C.U.UU.............UA.....................AGGUGUUCU.GAAAUAUUCC.AUUAGGUC.U.UAGAA.GGAAUAUU..UUU...AGAACAUCUUUUUUAG
env-621   UUUUCUUUGCCACCGGGUAAA.G.A..................GUCA...................AGGUGUUCU.GCAACAUUCC.GUUAGGUC.A.UAGAA.GGAAUGUU...CU...GGAACACCUCUUUGUU
env-622   CUUACUUUGCCACCGGGUAAA.G.A.U.A..............UU.....................UAGGUGUUC.GAGGCAUUCC.GUUAGGUC.U.UAGAA.GGAAUGCU...UG...GGGCACUUUUUUAUUU
env-623   UACGCUUUGCCACCGGGUAAA.G.C.G................ACCG...................AGGUGUUCU.GAAGCAUUCC.GUUAGGUC.U.UAGAA.GGAGUGUU...CU...CGAACAUCUUUUUUUG
env-624   AAAUAUUUGCCACCGGGUAGA.G.U.A.U..............AC.....................AAGGUGUUC.UAGACAUUCC.GAUAGGUC.U.UAGAA.GGAAUGUU..UUA...GGACACCUUUUUAUUG
env-625   UAUUCUUUGCCACCGGGUAAA.G.A.A.UG.............U......................ACGGUGUUC.AAAACAUUCC.GUCAGGUC.U.UAGCA.GGAAUGUU.GUUU...GAACGCUUUUUUUUAU
Cin-1-1   UAAACUUCGCCACCGGGUGAA.G.U.G.UA.............UA.....................AGGUAUUCU.GCAGCAUUCC.AUUAGGUC.U.UUGGA.GGAAUGUU..AUC...AGAAUACCUUUUUAUU
Rma-2-1   UUUGUUUUGCUACCGAGUAAA.A.C.A.AA.............UCA....................AAGUGCUCA.AACUCAUUCC.AUUAGGUC.U.UAGGA.GGAAUG.....UUGUCUGGGCAUUUUUUUAUG
env-626   UUUGUUUUGCUACCGGGUAAA.A.C.A.AA.............UUA....................AAGUGCUCA.AACACAUUCC.AUUAGGUC.U.UAGAA.GGGAUGUU..GUC...UGA..GCUUUUUUCAU

          ...........<<<..........................................................................>>>.........................................
          ...........20...........................................................................02..........................................
          <<<<<<<<<<<....>>>>>>.>.>.>.>>......<<<<<<<<.............>>>>>>>>...<<<<<<<<<.<<<<<<<<<<..................>>>>>>>>...>>...>>>>>>>>>.......
          22.22222222....222222.2.2...22......1111110..............0111111...22222222.2222222221..................12222222...22...22222222........
          ●●●●●●YUGCCRCCGGGUAR●-∘-∘-∘-∘--------------∘∘∘--------------------∘∘R●●●Y●●-●●●●YRY●YC-RUUAGGYC-●-U●GAA-GR●RYR●●----●●---●●●R●●●YYUUUUUY●●
```

```
Bca-1-1   CGGCAUUUACCACCGGGUAAA.U.G.C.AG..............UUU.......................GUGCCUG.UAUAUAUUCC..GUAGGUC.U.UAGAA.GGAAUAUA...UC...CAGGCACUUUUUUGCU
env-627   CUUAUCUUGCCACCGGGUAGG.A.U.A.A...............AUUA....................AGGUGUUCA.GGCAUAUUCC.AUUAGGUC.U.UAGAA.GGAAUUUU...GU...UGUGGACAUUUUUUU.
Csp-10-1  AUGACUUUGCCACCGGGUAAA.G.U...................AUGAGA..................AGCGUUUGG.AAGCCAUACC.GUUAGGUC.U.UAGAA.GGUAUGGG....A...CCGGGCGCUUUUUUUG
env-628   AUACUCCCGCCGCCGGGUGGG.A.G.G.A...............AA......................UGAGCGUUU.GCUUCGUAUC.GAUAGGCC.U.UAGAA.GAUACGAA..UGC...AGACGUAUUUUUUUAU
env-629   AUGUUCCUGCCGCCGGGUGGG.A.A.C.G.......................................GGAGCGUUU.GCUCCGCGUC..GUAGGCC.U.UAGAA.GACGCGGG....C...AGGCGCAUUUUUUAUU
env-630   GUGUUCCUGCCGCCGGGCGGG.A.A.C.G.......................................GGAGUGUUU.GCUCUGCGUC..GUAGGCC.U.UAGAA.GACGCGGG....C...AGGCGCAUUUUUUAUU
env-631   UCGUUCCCGCCGCCGGGCGGG.A.A.U.GG.......................................GAGCGUUU.GCUUCGCGUC..GUAGGCC.U.UUGAA.GACGCGGG...GU...AGACGCAUUUUUUGUU
env-632   CCUAAGCCACCGCCGGGUGGC.A.A.A.................C..........................GCGUUU.GCCCCGCAUC.GUUAGGCC.U.UUGAA.GAUGCGGC.GGGC...AGGCGCAUUUUUUUGU
env-633   AAAUCCCUGCCGCCGGGUAGG.G.A...................AGG......................AGCGCUUU.GCCGCGUAUC.GUCAGGCC.A.UAGAA.GAUACGCG...UC...CGGGCGCUUUUUUGUU
env-634   CAACAUUGGCCGCCGGGUCAA.U.G.A.AG..............AG......................AAGCGCUUC.GCCGCGUAUC.GUCAGGCC.A.UAGAA.GAUACGCG...GG...CGGGCGCUUUUUUGUC
env-635   UUUAUACUGCCACCGGGUAGU.U.A.U.G...............CAAUAA..................GCAUAUCUC.UAUAUACAUC.AUUAGGUC.U.UAGAA.GAUGUAUU........AAGAUAAGCUUAUUUU
Lal-1-1   AUAACUACACCGCCGGGUGUU.A.A.U.................CAAUAA..................AGUAC.UCU.UACCAAUUCC.AUUAGGCC.A.AAGAA.GGAAUUGC...UA...AGGAGUACUUAUUUUU
Lgi-1-1   UUAUAUACACCGCCGGGUGUC.A.A...................AUUUAAAAA...............GAGUACUUC.UAAUAAUUCC.GUUAGGCC.A.AAGUA.GGAAUUAU...CA...GGGGCACUCUAUUUUU
Lpa-3-1   AAUUAUACACCGCCGGGUGUC.A.A.C.U...............AAAA....................AAGUACUCU.UAUAAAUUCC.AUUAGGCC.A.CAGAA.GGAAUUUG...UA...GGGGUACUUUAUUUUU
env-636   AUCAAAACACCGCCGGGUGUC.A.C.U.U...............ACAG....................AAGUACUCU.UAUCAAUUCC.AUUAGGCC.A.UAGAA.GGAAUUGA...UA...GGGGUACUUAUUUUUU
env-637   UAAGCAACACCGCCGGGUGUC.A.U.U.UA..............AAA.....................AAGUACUCU.UACCAAUUCC.AUUAGGCC.A.AAGAA.GGAAUUGC...UA...GGGGUACUUAUUUUUU
Lna-2-1   CAUUAAACACCGCCGGGUGUC.A.C.U.U...............AAAA....................AAGUAUUCU.UACCAAUUCC.AUUAGGCC.A.AAGAA.GGAAUUGA...UA...GGGGUACUUAUUUUUU
env-638   UAUUAAACACCGCCGGGUGUC.A.C.U.UA..............CAC.....................AAGUAUUCU.UAUCAAUUCC.AUUAGGCC.A.AUGAA.GGAAUUGA...UG...GGGGUACUUAUUUUUU
env-639   UAAUCAACACCGCCGGGUGUC.A.C.U.UA..............AA......................AAGUUCUCU.UAUCAAUUCC.AUUAGGCC.A.AAGAA.GGAAUUGU...UA...GGGGGACUAUUUUUUU
Lcr-2-1   GAAAUCACACCGCCGGGUGUC.A.A.U.U...............ACAG....................AAGUACUCU.UACCAAUUCC.GUUAGGCC.A.AUGAA.GGAAUUGC...UA...GGGGUACUUAUUUUUU
Lno-1-1   AUCAAUACACCGCCGGGUGUC.A.C.U.A...............AAAA....................AAGUAUUCU.UAAUAAUUCC.GUUAGGCC.A.AAGAA.GGAAUUAU...UG...GGGAUACUUUAUUUUU
Lal-2-1   AUCAAUACACCGCCGGGUGUC.A.C.U.A...............AAAA....................AAGUAUUCU.UUAUAAUUCC.AUUAGGCC.A.UUGAA.GGAAUUGU...AA...GGGAUACUUUAUUUUU
Lve-1-1   GAAUUAACACCGCCGGGUGUC.A.U.U.................AAAC....................UAGUAUUCC.AAAUAAUUCC.AUUAGGCC.A.AUGAA.GGGAUUAU...UG...GGGAUACUUUUUUUUC
env-640   AUAAAACCACCGCCGGGUGUA.U.U.A.A...............AAAA....................GAGUAUCUU.UAACAAUUCC.GUUAGGCC.A.AAGAA.GGAAUUGC...UA...AGGGUGCUCUAUUUUU
Par-1-1   ACAAAGCCAUCACCGGGUGGC.......................A.......................AAAAGCUGU.UAACAAUUCC.GUUAGGUC.A.UUGAA.GGAAUUGU...UA...GGAGCUUUUUUAUUUU
Efa-1-1   UACGAACUGCCACCGGGUAG........................GAU......................AAAGAGGA.UGAUCAUUCC.AUUAGGUC.U.UUGAA.GGGAUGAU...UU...UCCUCUUUUUGUGUAU
Ega-1-1   UAGAGACUGCCACCGGGUAGU.U.A.A.AA..........................................GAGGA.CGAUCAUUCC.AGUAGGUC.U.UAGAA.GGAAUGGU...CU...UCCUCUUUUUGUUUUC
Eth-1-1   ACAAAGCUGCCACCGGGUAGC.A.U...................AU..........................GAGGA.UAAUCAUUCC.GUUAGGUC.U.UUGAA.GGAAUGGU...UC...UCCUCUUUUUUGUGUU
env-641   AUCUAAUAACCGCCGGGUUUA................................................CGUCUUA..UCCCCAUUUC.GUUAGGCC.A.UGGAA.GGAGUGGA...GG...ACAAGGCGCUUUAUUU
env-642   UAUCGCUUGCCGCCGGGUAAG.U.A.A.AA..............CA....................GGACGC..UAA.GUUUCAUAUC.AUUAGGCC.U.UUGAA.GAUAUGAA...A....UUGCAGCGUUUUUUUA
env-643   AUUGUACUGCCGCCGGGCAGU.A.G.G.................UGC.....................GAAAGCGCU.UGUAUACAUC.AUCAGGCC.G.UAGAA.GAUGUAUA...UA...AGCGCUUUUAAUAUGU
env-644   GAAAAAUUCCCGCCGGGGAAA.U......................C...........................GCAU.GAAAUAUAUC.AUUAGGCC.U.UAGAA.GAUAUAUU...CC...AUGCUUUUUAUUUUUA
Loz-1-1   AAUCAGUUGCCACCGGGUAAC.U.U.A.U............................................ACUC.AAACAAGCUC.AUUAGGUC.A.UAGAA.GAGCUUGC...UU...GAGUUUUUUAUUUUUA
Efa-2-1   UCGUAAACAUCGCCGGAUGCU.U.G.C.GA..............CUA.....................AAGUGUUAA.UGAACAGUUC..UUAGGCC.A.UAGCU.GAACUGUU...UG...UUGGCACUUUUUUUGU
Lli-1-1   UAUUAAUCGCCACCGGGCGAG................................................AAAGCAUU.UAUAAAGUUC.AUUAGGUC.A.AUGAA.GAACUUUA...UA...GGUGCUUUUAUUUUUU
Lpl-1-1   GAUGAAUUGUCACCGGACAA....................................................CGUUA.ACCCAACUUC.CUUAGGUC.A.UUGUG.GAAGUUGG...GU...GGACGUUGUCCUUUUU
Lfa-1-1   UAAUAAACACCGCCGGGUGUC.A.C.U.UA..............AUC.....................AAGUAUUCU.UAAUAAUUCC.AUUAGGCC.A.AAGAA.GGAAUUAU...UA...GGGAUACUUAUUUUUU
Eha-1-1   UGAUAACCAGCGCCGGCUGGU.U.A.U.CA..............GCAAA...................AGUGUAGAC.CGACAAUUCU...UAGGCC.G.UAGAU.AGAAUUGU...UG...GUAGACACUUUUUUUA
          ...........<<<...............................................................................>>>..........................................
          ...........20................................................................................02..........................................
          <<<<<<<<<<<....>>>>>>.>.>.>.>>......<<<<<<<<.............>>>>>>>>...<<<<<<<<<.<<<<<<<<<<..................>>>>>>>>...>>...>>>>>>>>>.......
          22.22222222....222222.2.2...22......1111110..............0111111....22222222.2222222221..................12222222...22...22222222........
          ●●●●●●YUGCCRCCGGGUAR●-●-●-○-○---------------●○○---------------------○●R●●●Y●●-●●●●YRY●YC-RUUAGGYC-●-U●GAA-GR●RYR●●---●●---●●R●●●YYUUUUUY●●
```

2 GGAM-2

2.1 Taxa

The taxonomy of each organism containing a putative GGAM-2 RNA is listed, with abbreviations identifying each hit (e.g., "Eco-1-1" and "Eco-1-2" might hypothetically represent two distinct RNAs in *E. coli*). The abbreviations will be used to identify each individual GGAM-2 RNA in Sections 2.2 and 2.4:.

abbrev. of hits	taxonomy of species
Cfi-1-1	Bacteria Actinobacteria Actinobacteridae Actinomycetales Micrococcineae Cellulomonadaceae *Cellulomonas fimi* ATCC 484
Csp-1-1	Bacteria Actinobacteria Actinobacteridae Actinomycetales Micrococcineae Cellulomonadaceae *Cellulomonas sp.* JC225
Csp-2-1	Bacteria Actinobacteria Micrococcales Cellulomonadaceae *Cellulomonas sp.* HZM
Csp-3-1	Bacteria Actinobacteria Micrococcales Cellulomonadaceae *Cellulomonas sp.* Leaf334
Csp-4-1	Bacteria Actinobacteria Micrococcales Cellulomonadaceae *Cellulomonas sp.* Leaf395
Csp-5-1	Bacteria Actinobacteria Micrococcales Cellulomonadaceae *Cellulomonas sp.* Root137
Csp-6-1	Bacteria Actinobacteria Micrococcales Cellulomonadaceae *Cellulomonas sp.* Root485
Csp-7-1	Bacteria Actinobacteria Micrococcales Cellulomonadaceae *Cellulomonas sp.* Root930
Csp-8-1	Bacteria Actinobacteria Micrococcales Cellulomonadaceae *Cellulomonas sp.* URHD0024
Csp-9-1	Bacteria Actinobacteria Micrococcales Cellulomonadaceae *Cellulomonas sp.* URHE0023
Cce-1-1	Bacteria Actinobacteria Micrococcales Promicromonosporaceae *Cellulosimicrobium cellulans*
Cce-2-1	Bacteria Actinobacteria Micrococcales Promicromonosporaceae *Cellulosimicrobium cellulans* F16
Cce-3-1	Bacteria Actinobacteria Micrococcales Promicromonosporaceae *Cellulosimicrobium cellulans* J1
Cce-4-1	Bacteria Actinobacteria Micrococcales Promicromonosporaceae *Cellulosimicrobium cellulans* J36
Cfu-1-1	Bacteria Actinobacteria Micrococcales Promicromonosporaceae *Cellulosimicrobium funkei*
Csp-10-1	Bacteria Actinobacteria Micrococcales Promicromonosporaceae *Cellulosimicrobium sp.* CUA-896
Csp-11-1	Bacteria Actinobacteria Micrococcales Promicromonosporaceae *Cellulosimicrobium sp.* I38E

Pba-1-1	Bacteria Actinobacteria Micrococcales *Promicromonosporaceae bacterium* W15
env-1 to env-109	environmental samples

2.2 Gene contexts

Each GGAM-2 RNA (indicated by "RNA→") is listed. For each hit, the downstream genes predicted to reside in a regulated operon are listed. If the nearest downstream gene is encoding in the opposite strand (and therefore presumed to not be a part of a regulated operon), then that gene is still depicted. Some environmental sequences and some RefSeq entries lack gene annotations, and so no genes are available for such sequences. The direction of each gene is indicated with an arrow (→), and each predicted conserved domain in the gene is named. Conserved domains associated with more than one GGAM-2 RNA are assigned a color; other domains are gray. Information about these conserved domains is given in Section 2.3. The accession of the sequence containing each GGAM-2 RNA is given in the column named "Seq. accession". Accessions beginning with "NC_", "NS_", "NW_" or "NZ_" are contained in RefSeq. Other accession refer to environmental samples. Nucleotide coordinates are given for the 5′ and 3′ boundaries of each GGAM-2 RNA. If the 5′ coordinate is greater than the 3′ coordinate, the RNA is present on the reverse-complement strand of the containing genomic DNA sequence. Each hit is denoted by an abbreviation (like "Eco-1-1") that refers to a taxonomy given in Section 2.1.

abbrev.	Seq. accession		5′ at	3′ at	genes
env-1	Ga0157369_10950753	+	289	350	**RNA**→ ←hypo
env-2	Ga0160505_100002	-	251075	251012	**RNA**→ PRK11431 (PRK11431)EmrE (COG2076)→ YbjK (COG3226)→
env-3	Ga0209370_1000011	+	521368	521431	**RNA**→ PRK11431 (PRK11431)EmrE (COG2076)→ YbjK (COG3226)→
Csp-2-1	NZ_JEOE01000059.1	+	2647	2709	**RNA**→ PRK11431 (PRK11431)EmrE (COG2076)→ AcrR (COG1309)→ AcrR (COG1309)→
					PRK02901 (PRK02901)OSBS (cd03320)→
					TPP_PYR_MenD (cd07037)PRK07449 (PRK07449)TPP_SHCHC_synthase (cd02009)→
					TPP_PYR_MenD (cd07037)PRK07449 (PRK07449)TPP_SHCHC_synthase (cd02009)→
env-4	Ga0160471_111600	-	786	723	**RNA**→ PRK11431 (PRK11431)EmrE (COG2076)→ YbjK (COG3226)PRK11552 (PRK11552)→
env-5	Ga0160459_102828	+	1887	1950	**RNA**→ PRK11431 (PRK11431)EmrE (COG2076)→ YbjK (COG3226)PRK11552 (PRK11552)→
env-6	Ga0160447_131657	+	122	185	**RNA**→ PRK11431 (PRK11431)EmrE (COG2076)→ YbjK (COG3226)→
env-7	Ga0164243_10004798	+	17716	17779	**RNA**→ PRK11431 (PRK11431)EmrE (COG2076)→ PRK14996 (PRK14996)AcrR (COG1309)→
					MR_MLE (smart00922)OSBS (cd03320)→
env-8	Ga0164242_10014691	+	1031	1094	**RNA**→ PRK11431 (PRK11431)EmrE (COG2076)→ PRK14996 (PRK14996)AcrR (COG1309)→
					MR_MLE (smart00922)OSBS (cd03320)→
env-9	Ga0182740_1007935	-	401	338	**RNA**→ PRK11431 (PRK11431)EmrE (COG2076)→
env-10	Ga0157372_10217055	+	761	824	**RNA**→ PRK11431 (PRK11431)EmrE (COG2076)→ PRK14996 (PRK14996)AcrR (COG1309)→
					PRK02901 (PRK02901)OSBS (cd03320)→
env-11	Ga0164241_10010842	-	1728	1665	**RNA**→ PRK11431 (PRK11431)EmrE (COG2076)→ YbjK (COG3226)→
					MR_MLE (smart00922)RspA (COG4948)→
env-12	Ga0164241_12983309	-	86	23	**RNA**→
env-13	Ga0164242_10413062	+	396	459	**RNA**→ PRK11431 (PRK11431)EmrE (COG2076)→ hypo→
Pba-1-1	NZ_DF158876.1	-	178057	177994	**RNA**→ hypo→ hypo→ MR_MLE (smart00922)OSBS (cd03320)→ hypo→
env-14	Ga0164309_10817934	-	716	655	**RNA**→ PRK11431 (PRK11431)EmrE (COG2076)→ AcrR (COG1309)PRK11552 (PRK11552)→
env-15	Ga0160446_100041	-	59705	59644	**RNA**→ PRK11431 (PRK11431)EmrE (COG2076)→ AcrR (COG1309)PRK11552 (PRK11552)→
					OSBS (cd03320)MR_MLE_C (pfam13378)→ TPP_PYR_MenD (cd07037)PRK07449 (PRK07449)→
					DegQ (COG0265)PDZ_2 (pfam13180)Trypsin_2 (pfam13365)DUF4106 (pfam13388)→
env-16	Ga0160434_100001	-	517159	517098	**RNA**→ PRK11431 (PRK11431)EmrE (COG2076)→ AcrR (COG1309)PRK11552 (PRK11552)→
					OSBS (cd03320)MR_MLE_C (pfam13378)→ TPP_PYR_MenD (cd07037)PRK07449 (PRK07449)→
					DegQ (COG0265)PDZ_2 (pfam13180)Trypsin_2 (pfam13365)DUF4106 (pfam13388)→
env-17	Ga0164307_10400753	+	951	1011	**RNA**→
env-18	Ga0164308_11065520	-	606	545	**RNA**→ PRK11431 (PRK11431)EmrE (COG2076)→ YbjK (COG3226)→
env-19	Ga0164306_10048600	+	1015	1076	**RNA**→ PRK11431 (PRK11431)EmrE (COG2076)→ AcrR (COG1309)PRK11552 (PRK11552)→
					PRK02901 (PRK02901)OSBS (cd03320)→
env-20	Ga0164243_11722168	+	92	153	**RNA**→ hypo→

Cce-2-1	NZ_KQ435288.1	+	1038007	1038068	**RNA**→ PRK11431 (PRK11431)EmrE (COG2076)→ AcrR (COG1309)RutR (TIGR03613)→<br>PRK02901 (PRK02901)OSBS (cd03320)→<br>TPP_PYR_MenD (cd07037)PRK07449 (PRK07449)TPP_SHCHC_synthase (cd02009)→<br>TPP_PYR_MenD (cd07037)PRK07449 (PRK07449)TPP_SHCHC_synthase (cd02009)→<br>DegQ (COG0265)PDZ_2 (pfam13180)Trypsin_2 (pfam13365)→ hypo→
Cce-1-1	NZ_CP021383.1	+	3046659	3046720	**RNA**→ PRK11431 (PRK11431)EmrE (COG2076)→ PRK14996 (PRK14996)AcrR (COG1309)→<br>PRK02901 (PRK02901)OSBS (cd03320)→ hypo→
env-21	Ga0160439_1069687	-	414	353	**RNA**→ PRK11431 (PRK11431)EmrE (COG2076)→
env-22	Ga0120998_107230	-	102	41	**RNA**→
Cfu-1-1	NZ_JNBQ01000002.1	+	463569	463630	**RNA**→ PRK11431 (PRK11431)EmrE (COG2076)→ AcrR (COG1309)RutR (TIGR03613)→<br>PRK02901 (PRK02901)OSBS (cd03320)→<br>TPP_PYR_MenD (cd07037)PRK07449 (PRK07449)TPP_SHCHC_synthase (cd02009)→<br>TPP_PYR_MenD (cd07037)PRK07449 (PRK07449)TPP_SHCHC_synthase (cd02009)→<br>DegQ (COG0265)PDZ_2 (pfam13180)Trypsin_2 (pfam13365)→ hypo→
Csp-11-1	NZ_LUAZ01000041.1	+	2615	2676	**RNA**→ PRK11431 (PRK11431)EmrE (COG2076)→ AcrR (COG1309)RutR (TIGR03613)→<br>PRK02901 (PRK02901)OSBS (cd03320)→<br>TPP_PYR_MenD (cd07037)PRK07449 (PRK07449)TPP_SHCHC_synthase (cd02009)→<br>DegQ (COG0265)PDZ_2 (pfam13180)Trypsin_2 (pfam13365)→
env-23	Ga0121381_100001	+	813120	813181	**RNA**→ PRK11431 (PRK11431)EmrE (COG2076)→ AcrR (COG1309)RutR (TIGR03613)→<br>PRK02901 (PRK02901)OSBS (cd03320)→<br>TPP_PYR_MenD (cd07037)PRK07449 (PRK07449)TPP_SHCHC_synthase (cd02009)→<br>DegQ (COG0265)PDZ_2 (pfam13180)Trypsin_2 (pfam13365)→
env-24	Ga0164308_10928298	-	347	288	**RNA**→ PRK11431 (PRK11431)EmrE (COG2076)→
env-25	Ga0164243_11687470	-	81	20	**RNA**→
env-26	Ga0160493_100048	-	42989	42928	**RNA**→ PRK11431 (PRK11431)EmrE (COG2076)→ PRK14996 (PRK14996)AcrR (COG1309)→<br>DUF1345 (pfam07077)→ PRK02901 (PRK02901)OSBS (cd03320)→<br>TPP_PYR_MenD (cd07037)PRK07449 (PRK07449)TPP_SHCHC_synthase (cd02009)→<br>DegQ (COG0265)PDZ_2 (pfam13180)Trypsin_2 (pfam13365)→
env-27	Ga0160498_100063	-	42989	42928	**RNA**→ PRK11431 (PRK11431)EmrE (COG2076)→ PRK14996 (PRK14996)AcrR (COG1309)→<br>DUF1345 (pfam07077)→ PRK02901 (PRK02901)OSBS (cd03320)→<br>TPP_PYR_MenD (cd07037)PRK07449 (PRK07449)TPP_SHCHC_synthase (cd02009)→<br>DegQ (COG0265)PDZ_2 (pfam13180)Trypsin_2 (pfam13365)→
env-28	Ga0160477_100084	-	42989	42928	**RNA**→ PRK11431 (PRK11431)EmrE (COG2076)→ PRK14996 (PRK14996)AcrR (COG1309)→<br>DUF1345 (pfam07077)→ PRK02901 (PRK02901)OSBS (cd03320)→<br>TPP_PYR_MenD (cd07037)PRK07449 (PRK07449)TPP_SHCHC_synthase (cd02009)→<br>DegQ (COG0265)PDZ_2 (pfam13180)Trypsin_2 (pfam13365)→
env-29	Ga0160485_109881	+	713	774	**RNA**→
env-30	Ga0164306_13161487	-	343	282	**RNA**→ PRK11431 (PRK11431)EmrE (COG2076)→
Cce-4-1	NZ_JAGJ01000018.1	+	41628	41689	**RNA**→ PRK11431 (PRK11431)EmrE (COG2076)→ RutR (TIGR03613)TetR_N (pfam00440)→<br>DUF1345 (pfam07077)→ PRK02901 (PRK02901)OSBS (cd03320)→<br>TPP_PYR_MenD (cd07037)PRK07449 (PRK07449)TPP_SHCHC_synthase (cd02009)→<br>PHA03378 (PHA03378)DegQ (COG0265)PDZ_2 (pfam13180)Trypsin_2 (pfam13365)→ hypo→
Cce-3-1	NZ_FXAA01000001.1	+	511390	511451	**RNA**→ PRK11431 (PRK11431)EmrE (COG2076)→ RutR (TIGR03613)TetR_N (pfam00440)→<br>DUF1345 (pfam07077)→ PRK02901 (PRK02901)OSBS (cd03320)→<br>TPP_PYR_MenD (cd07037)PRK07449 (PRK07449)TPP_SHCHC_synthase (cd02009)→<br>PHA03378 (PHA03378)DegQ (COG0265)PDZ_2 (pfam13180)Trypsin_2 (pfam13365)→
Csp-10-1	NZ_MKKH01000009.1	-	2198	2139	**RNA**→ hypo→ TetR_N (pfam00440)→ hypo→ hypo→
env-31	Ga0160432_102332	-	3870	3808	**RNA**→ PRK11431 (PRK11431)EmrE (COG2076)→ YbjK (COG3226)PRK11552 (PRK11552)→<br>PRK02901 (PRK02901)OSBS (cd03320)→<br>TPP_PYR_MenD (cd07037)PRK07449 (PRK07449)TPP_SHCHC_synthase (cd02009)→
env-32	Ga0164307_11501963	-	271	209	**RNA**→ PRK11431 (PRK11431)EmrE (COG2076)→
env-33	Ga0160442_100033	+	95473	95536	**RNA**→ PRK11431 (PRK11431)EmrE (COG2076)→ YbjK (COG3226)→
env-34	Ga0160440_100038	+	95473	95536	**RNA**→ PRK11431 (PRK11431)EmrE (COG2076)→ YbjK (COG3226)→
env-35	Ga0160432_100027	+	95473	95536	**RNA**→ PRK11431 (PRK11431)EmrE (COG2076)→ YbjK (COG3226)→
env-36	Ga0160452_100489	-	1068	1005	**RNA**→ PRK11431 (PRK11431)EmrE (COG2076)→ YbjK (COG3226)→
env-37	Ga0160430_100018	+	95473	95536	**RNA**→ PRK11431 (PRK11431)EmrE (COG2076)→ YbjK (COG3226)→

env-38	Ga0256799_1046593	-	517	454	RNA→ PRK11431 (PRK11431)EmrE (COG2076)→
env-39	Ga0256776_1065114	-	517	454	RNA→ PRK11431 (PRK11431)EmrE (COG2076)→
env-40	Ga0256787_1097516	-	323	260	RNA→ PRK11431 (PRK11431)EmrE (COG2076)→
env-41	Ga0256775_1025839	-	1102	1039	RNA→ PRK11431 (PRK11431)EmrE (COG2076)→ YbjK (COG3226)PRK11552 (PRK11552)→
env-42	Ga0256765_105706	-	440	377	RNA→ PRK11431 (PRK11431)EmrE (COG2076)→
env-43	Ga0256778_1002726	-	1281	1218	RNA→ PRK11431 (PRK11431)EmrE (COG2076)→ YbjK (COG3226)PRK11552 (PRK11552)→
					PRK02901 (PRK02901)OSBS (cd03320)→
env-44	Ga0256779_1090235	+	55	118	RNA→ PRK11431 (PRK11431)EmrE (COG2076)→
env-45	Ga0256796_1083137	+	118	182	RNA→ PRK11431 (PRK11431)EmrE (COG2076)→ YbjK (COG3226)PRK11552 (PRK11552)→
env-46	Ga0256778_1070514	+	269	333	RNA→ PRK11431 (PRK11431)EmrE (COG2076)→
env-47	Ga0256768_1006639	+	1346	1410	RNA→ PRK11431 (PRK11431)EmrE (COG2076)→
env-48	Ga0182740_1091939	-	756	699	RNA→ PRK11431 (PRK11431)EmrE (COG2076)→ YbjK (COG3226)PRK11552 (PRK11552)→
Csp-1-1	NZ_HE978588.1	+	1593279	1593340	RNA→ PRK11431 (PRK11431)EmrE (COG2076)→ PRK11431 (PRK11431)EmrE (COG2076)→
env-49	Ga0164308_12687686	+	39	100	RNA→ PRK11431 (PRK11431)EmrE (COG2076)→
env-50	Ga0190274_11770711	-	390	329	RNA→ PRK11431 (PRK11431)EmrE (COG2076)→
Cfi-1-1	NC_015514.1	+	1014463	1014526	RNA→ PRK11431 (PRK11431)EmrE (COG2076)→ YbjK (COG3226)→
					PRK02901 (PRK02901)OSBS (cd03320)→
					TPP_PYR_MenD (cd07037)PRK07449 (PRK07449)TPP_SHCHC_synthase (cd02009)→
					Amelogenin (smart00818)DegQ (COG0265)PDZ_2 (pfam13180)Trypsin_2 (pfam13365)→
env-51	Ga0160499_100063	+	23438	23501	RNA→ PRK11431 (PRK11431)EmrE (COG2076)→ YbjK (COG3226)PRK11552 (PRK11552)→
					PRK02901 (PRK02901)OSBS (cd03320)→
					TPP_PYR_MenD (cd07037)PRK07449 (PRK07449)TPP_SHCHC_synthase (cd02009)→
env-52	Ga0160478_100154	-	6068	6005	RNA→ PRK11431 (PRK11431)EmrE (COG2076)→ YbjK (COG3226)PRK11552 (PRK11552)→
					PRK02901 (PRK02901)OSBS (cd03320)→
					TPP_PYR_MenD (cd07037)PRK07449 (PRK07449)TPP_SHCHC_synthase (cd02009)→
env-53	Ga0121184_102312	+	220	283	RNA→ PRK11431 (PRK11431)EmrE (COG2076)→ AcrR (COG1309)PRK11552 (PRK11552)→
					PRK02901 (PRK02901)OSBS (cd03320)→
env-54	Ga0160445_113436	+	195	255	RNA→ PRK11431 (PRK11431)EmrE (COG2076)→ AcrR (COG1309)PRK11552 (PRK11552)→
env-55	Ga0121585_103339	-	433	369	RNA→ PRK11431 (PRK11431)EmrE (COG2076)→
env-56	Ga0160446_114229	-	721	658	RNA→ PRK11431 (PRK11431)EmrE (COG2076)→ YbjK (COG3226)→
env-57	Ga0120979_108451	-	315	251	RNA→ PRK11431 (PRK11431)EmrE (COG2076)→
env-58	Ga0120937_100206	+	11501	11565	RNA→ PRK11431 (PRK11431)EmrE (COG2076)→ YbjK (COG3226)→
					PRK02901 (PRK02901)OSBS (cd03320)→ MenD (COG1165)PRK07449 (PRK07449)→
env-59	Ga0121274_105479	+	549	613	RNA→ PRK11431 (PRK11431)EmrE (COG2076)→
env-60	Ga0121403_10033	-	34899	34835	RNA→ PRK11431 (PRK11431)EmrE (COG2076)→ YbjK (COG3226)→
					PRK02901 (PRK02901)OSBS (cd03320)→
					TPP_PYR_POX_like (cd07035)PRK07449 (PRK07449)TPP_SHCHC_synthase (cd02009)→
env-61	Ga0121076_111322	-	83	20	RNA→
env-62	Ga0164306_11887969	+	9	72	RNA→ PRK11431 (PRK11431)EmrE (COG2076)→
env-63	Ga0164309_10003055	+	502	565	RNA→ PRK11431 (PRK11431)EmrE (COG2076)→ hypo→ PRK02901 (PRK02901)OSBS (cd03320)→
					TPP_PYR_POX_like (cd07035)PRK07449 (PRK07449)TPP_SHCHC_synthase (cd02009)→
					DegQ (COG0265)PRK10263 (PRK10263)ARS2 (pfam04959)PDZ_2 (pfam13180)Trypsin_2 (pfam13365)→
env-64	Ga0164308_14086515	+	122	185	RNA→ PRK11431 (PRK11431)EmrE (COG2076)→
env-65	Ga0164307_11127317	+	206	270	RNA→ PRK11431 (PRK11431)EmrE (COG2076)DUF3418 (pfam11898)→
env-66	Ga0120979_100532	-	1467	1404	RNA→ PRK11431 (PRK11431)EmrE (COG2076)→ YbjK (COG3226)→
					MR_MLE (smart00922)RspA (COG4948)→
env-67	Ga0121388_104665	-	513	450	RNA→ PRK11431 (PRK11431)EmrE (COG2076)→ hypo→
env-68	Ga0190274_14164383	-	405	343	RNA→ PRK11431 (PRK11431)EmrE (COG2076)→
env-69	Ga0157370_10835390	-	574	512	RNA→ PRK11431 (PRK11431)EmrE (COG2076)→ hypo→
env-70	Ga0157369_12306528	-	297	236	RNA→ PRK11431 (PRK11431)EmrE (COG2076)→
env-71	Ga0105240_12235890	+	286	347	RNA→ PRK11431 (PRK11431)EmrE (COG2076)→
env-72	Ga0105239_15287047	-	293	232	RNA→ ←PRK08321 (PRK08321)MenB (COG0447)

Csp-3-1	NZ_LMOO01000001.1	-	1534405	1534343	**RNA**→ PRK11431 (PRK11431)EmrE (COG2076)→ YbjK (COG3226)→<br>PRK02901 (PRK02901)OSBS (cd03320)→<br>TPP_PYR_MenD (cd07037)TPP_SHCHC_synthase (cd02009)menD (TIGR00173)→<br>DegQ (COG0265)PRK14951 (PRK14951)PDZ_2 (pfam13180)Trypsin_2 (pfam13365)→
Csp-7-1	NZ_LMJI01000001.1	+	1974838	1974900	**RNA**→ PRK11431 (PRK11431)EmrE (COG2076)→ YbjK (COG3226)PRK11552 (PRK11552)→<br>PRK02901 (PRK02901)OSBS (cd03320)→<br>TPP_PYR_MenD (cd07037)TPP_SHCHC_synthase (cd02009)menD (TIGR00173)→<br>Amelogenin (smart00818)DegQ (COG0265)PDZ_2 (pfam13180)Trypsin_2 (pfam13365)→
Csp-5-1	NZ_LMFG01000001.1	+	1533192	1533254	**RNA**→ PRK11431 (PRK11431)EmrE (COG2076)→ YbjK (COG3226)PRK11552 (PRK11552)→<br>PRK02901 (PRK02901)OSBS (cd03320)→<br>TPP_PYR_MenD (cd07037)TPP_SHCHC_synthase (cd02009)menD (TIGR00173)→<br>Amelogenin (smart00818)DegQ (COG0265)PDZ_2 (pfam13180)Trypsin_2 (pfam13365)→
env-73	Ga0206353_11886671	-	671	608	**RNA**→ EmrE (COG2076)PRK11431 (PRK11431)→ YbjK (COG3226)→
env-74	Ga0164308_12900367	-	72	10	**RNA**→
env-75	Ga0190274_13680230	+	181	243	**RNA**→ PRK11431 (PRK11431)EmrE (COG2076)→
Csp-6-1	NZ_LMFC01000001.1	+	252521	252583	**RNA**→ PRK11431 (PRK11431)EmrE (COG2076)→ YbjK (COG3226)→<br>PRK02901 (PRK02901)OSBS (cd03320)→<br>TPP_PYR_MenD (cd07037)TPP_SHCHC_synthase (cd02009)menD (TIGR00173)→<br>DegQ (COG0265)PRK14951 (PRK14951)PDZ_2 (pfam13180)Trypsin_2 (pfam13365)→
env-76	Ga0164308_14137248	-	73	11	**RNA**→
env-77	FWIREl_GJ4R3DH02GNSXW	+	325	387	**RNA**→ hypo→
env-78	Ga0164307_10711663	-	351	289	**RNA**→ PRK11431 (PRK11431)EmrE (COG2076)→
Csp-4-1	NZ_LMQI01000003.1	+	194456	194519	**RNA**→ PRK11431 (PRK11431)EmrE (COG2076)→ YbjK (COG3226)→<br>PRK02901 (PRK02901)OSBS (cd03320)→<br>TPP_PYR_MenD (cd07037)TPP_SHCHC_synthase (cd02009)menD (TIGR00173)→
env-79	Ga0164308_12542235	-	408	346	**RNA**→ PRK11431 (PRK11431)EmrE (COG2076)→
env-80	Ga0164308_14334871	-	295	233	**RNA**→ PRK11431 (PRK11431)EmrE (COG2076)→
env-81	Ga0190275_12019369	+	401	464	**RNA**→ PRK11431 (PRK11431)EmrE (COG2076)→
env-82	Ga0164241_12921876	-	195	133	**RNA**→ PRK11431 (PRK11431)EmrE (COG2076)→
env-83	Ga0157372_12846580	-	238	176	**RNA**→ PRK11431 (PRK11431)EmrE (COG2076)→
env-84	Ga0160430_117334	-	767	705	**RNA**→ PRK11431 (PRK11431)EmrE (COG2076)→ PRK14996 (PRK14996)YbjK (COG3226)→
env-85	Ga0256788_1284884	-	266	204	**RNA**→ PRK11431 (PRK11431)EmrE (COG2076)→
env-86	Ga0160431_113968	-	437	375	**RNA**→ PRK11431 (PRK11431)EmrE (COG2076)→
env-87	Ga0164306_10925008	+	398	460	**RNA**→ PRK11431 (PRK11431)EmrE (COG2076)→
env-88	Ga0190274_15066833	-	329	267	**RNA**→ PRK11431 (PRK11431)EmrE (COG2076)→
env-89	Ga0206353_10028840	-	278	215	**RNA**→ EmrE (COG2076)PRK11431 (PRK11431)→
env-90	Ga0190275_13104762	+	373	435	**RNA**→ hypo→
env-91	Ga0256799_1212326	-	327	265	**RNA**→ PRK11431 (PRK11431)EmrE (COG2076)→
env-92	Ga0190274_13464751	+	81	143	**RNA**→ PRK11431 (PRK11431)EmrE (COG2076)→
Csp-9-1	NZ_JIAN01000005.1	-	382947	382885	**RNA**→ PRK11431 (PRK11431)EmrE (COG2076)→ YbjK (COG3226)→<br>PRK02901 (PRK02901)OSBS (cd03320)→<br>TPP_PYR_MenD (cd07037)TPP_SHCHC_synthase (cd02009)menD (TIGR00173)→
Csp-8-1	NZ_AUEW01000012.1	-	105430	105368	**RNA**→ PRK11431 (PRK11431)EmrE (COG2076)→ YbjK (COG3226)PRK11552 (PRK11552)→<br>PRK02901 (PRK02901)OSBS (cd03320)→<br>TPP_PYR_POX_like (cd07035)PRK07449 (PRK07449)TPP_SHCHC_synthase (cd02009)→
env-93	Ga0160439_1032782	-	676	616	**RNA**→ PRK11431 (PRK11431)EmrE (COG2076)→ YbjK (COG3226)→
env-94	Ga0160458_142033	-	294	233	**RNA**→ PRK11431 (PRK11431)EmrE (COG2076)→
env-95	Ga0160446_110248	-	568	507	**RNA**→ PRK11431 (PRK11431)EmrE (COG2076)→ YbjK (COG3226)PRK11552 (PRK11552)→
env-96	Ga0160437_1146822	+	37	98	**RNA**→ PRK11431 (PRK11431)EmrE (COG2076)→
env-97	Ga0160435_1104862	+	108	169	**RNA**→
env-98	Ga0160434_147989	+	79	140	**RNA**→ PRK11431 (PRK11431)EmrE (COG2076)→
env-99	Ga0160435_1104026	+	99	160	**RNA**→
env-100	Ga0160458_100604	-	3525	3464	**RNA**→ PRK11431 (PRK11431)EmrE (COG2076)→ betaine_BetI (TIGR03384)TetR_N (pfam00440)→<br>PRK02901 (PRK02901)OSBS (cd03320)→ TPP_PYR_MenD (cd07037)PRK07449 (PRK07449)→

env-101	Ga0160434_113199	-	759	698	**RNA**→ PRK11431 (PRK11431)EmrE (COG2076)→ betaine_BetI (TIGR03384)TetR_N (pfam00440)→
env-102	Ga0160448_107845	-	1015	954	**RNA**→ PRK11431 (PRK11431)EmrE (COG2076)→ betaine_BetI (TIGR03384)TetR_N (pfam00440)→
env-103	Ga0160446_100829	-	1023	962	**RNA**→ PRK11431 (PRK11431)EmrE (COG2076)→ betaine_BetI (TIGR03384)TetR_N (pfam00440)→
env-104	Ga0160447_106868	-	1749	1688	**RNA**→ PRK11431 (PRK11431)EmrE (COG2076)→ betaine_BetI (TIGR03384)TetR_N (pfam00440)→ OSBS (cd03320)MR_MLE_C (pfam13378)→
env-105	Ga0160451_147966	+	297	358	**RNA**→ PRK11431 (PRK11431)EmrE (COG2076)→
env-106	Ga0160449_1018056	+	1486	1547	**RNA**→ PRK11431 (PRK11431)EmrE (COG2076)→
env-107	Ga0160435_1106070	+	99	160	**RNA**→
env-108	Ga0160436_1011118	-	445	384	**RNA**→ PRK11431 (PRK11431)EmrE (COG2076)→
env-109	Ga0160439_1000093	+	6432	6493	**RNA**→ PRK11431 (PRK11431)EmrE (COG2076)→ betaine_BetI (TIGR03384)TetR_N (pfam00440)→ PRK02901 (PRK02901)OSBS (cd03320)→ TPP_PYR_MenD (cd07037)PRK07449 (PRK07449)TPP_SHCHC_synthase (cd02009)→

2.3 Conserved domains

Conserved domains found in protein-coding genes listed in Section 2.2 are shown below, with the first sentence in their description from the Conserved Domain Database (if any). Conserved domains associated with more than one GGAM-2 RNA are assigned a color, while others are shown in gray. The number in parentheses after the colored domain name is the number of occurrences in Section 2.2.

cd02009 (27) Thiamine pyrophosphate (TPP) family, SHCHC synthase subfamily, TPP-binding module; composed of proteins similar to Escherichia coli 2-succinyl-6-hydroxyl-2,4-cyclohexadiene-1-carboxylic acid (SHCHC) synthase (also called MenD).
cd03320 (37) o-Succinylbenzoate synthase (OSBS) catalyzes the conversion of 2-succinyl-6-hydroxy-2,4-cyclohexadiene-1-carboxylate (SHCHC) to 4-(2'-carboxyphenyl)-4-oxobutyrate (o-succinylbenzoate or OSB), a reaction in the menaquinone biosynthetic pathway.
cd07035 (3) Pyrimidine (PYR) binding domain of POX and related proteins.
cd07037 (27) Pyrimidine (PYR) binding domain of 2-succinyl-5-enolpyruvyl-6-hydroxy-3-cyclohexadiene-1-carboxylate synthase (MenD) and related proteins.
COG0265 (17) Periplasmic serine protease, S1-C subfamily, contain C-terminal PDZ domain [Posttranslational modification, protein turnover, chaperones]
COG0447 (1) 1,4-Dihydroxy-2-naphthoyl-CoA synthase [Coenzyme transport and metabolism]
COG1165 (1) 2-succinyl-5-enolpyruvyl-6-hydroxy-3-cyclohexene-1-carboxylate synthase [Coenzyme transport and metabolism]
COG1309 (19) DNA-binding transcriptional regulator, AcrR family [Transcription]
COG2076 (110) Multidrug transporter EmrE and related cation transporters [Defense mechanisms]
COG3226 (35) DNA-binding transcriptional regulator YbjK [Transcription]
COG4948 (2) L-alanine-DL-glutamate epimerase or related enzyme of enolase superfamily [Cell wall/membrane/envelope biogenesis, General function prediction only]
pfam00440 (9) Bacterial regulatory proteins, tetR family.
pfam04959 (1) Arsenite-resistance protein 2.
pfam07077 (5) Protein of unknown function (DUF1345).
pfam11898 (1) Domain of unknown function (DUF3418).
pfam13180 (17) PDZ domain.
pfam13365 (17) Trypsin-like peptidase domain.
pfam13378 (3) Enolase C-terminal domain-like.
pfam13388 (2) Protein of unknown function (DUF4106).
PHA03378 (2) EBNA-3B; Provisional
PRK02901 (31) O-succinylbenzoate synthase; Provisional
PRK07449 (25) 2-succinyl-5-enolpyruvyl-6-hydroxy-3-cyclohexene-1-carboxylate synthase; Validated
PRK08321 (1) naphthoate synthase; Validated
PRK10263 (1) DNA translocase FtsK; Provisional
PRK11431 (110) multidrug efflux system protein; Provisional
PRK11552 (19) putative DNA-binding transcriptional regulator; Provisional
PRK14951 (2) DNA polymerase III subunits gamma and tau; Provisional
PRK14996 (8) TetR family transcriptional regulator; Provisional
smart00818 (3) Amelogenins, cell adhesion proteins, play a role in the biomineralisation of teeth.
smart00922 (5) Mandelate racemase / muconate lactonizing enzyme, C-terminal domain.
TIGR00173 (6) 2-succinyl-5-enolpyruvyl-6-hydroxy-3-cyclohexene-1-carboxylic-acid synthase. [Biosynthesis of cofactors, prosthetic groups, and carriers, Menaquinone and ubiquinone]
TIGR03384 (6) transcriptional repressor BetI. [Regulatory functions, DNA interactions]
TIGR03613 (6) pyrimidine utilization regulatory protein R.

2.4 Multiple-sequence alignment

Each GGAM-2 RNA is denoted by an abbreviation (like “Eco-1-1”) that refers to a taxonomy given in Section 2.1. The alignment may include sequences containing the RNA motif, as well as flanking sequence. The GGAM-2 RNA itself is denoted by the line underneath marked 5′ and 3′ on either end. Nucleotides in flanking sequences (i.e., not part of the motif) are written in gray letters. Stems of predicted rho-independent transcription terminators, if any, are shaded yellow. (Note: terminator predictions have not been analyzed manually, and many are likely to be false positives. Terminator predictions are those of the RNIE software.) Nucleotides predicted to function (as DNA) as transcription-factor binding sites, if any, are shaded in green. (But note: these predictions are manually annotated, so they might be under-predicted.) Annotated start codons, if any, are shaded green. (Note: start codons are frequently misannotated, especially in environmental samples.) Nucleotides proposed to basepair as part of the consensus structure are shaded in color when they comprise Watson-Crick or G-U pairs. Otherwise they are shaded gray. Conserved stems are also indicated at the bottom of the alignment by angle brackets, where matching < and > denote base-paired columns. Below these angle brackets, the symbol “2” denotes base pairs exhibiting covariation according to the statistically well-founded R-scape method. “1” denotes base pairs exhibiting covariation according to R2R’s simplistic method. “0” denotes base pairs that are not observed to mutate and “?” denotes base pairs that have a significant frequency of non-canonical nucleotides for Watson-Crick or G-U pairs (> 5%). Below these base pair annotation is the consensus sequence: “R” = “A” or “G”, “Y” = “C” or “U”, red nucleotides: nucleotide identity conserved more than 97% of the time, black nucleotides: 90%, gray nucleotides: 75%, red circle (•): nucleotide is present 97% of the time, black circle (•): 90%, gray circle (•): 75%, white circle (◦): 50%. All percentages of sequences just described (e.g. 97% conserved) assume that sequences have been weighted by the GSC algorithm implemented by the Infernal software package.

Duplicate sequences: the following putative homologs are not shown in the alignment because their sequences are identical to a homolog already shown: Cce-3-1, env-101, env-102, env-16, env-19, env-21, env-27, env-28, env-34, env-35, env-36, env-37, env-5, env-52, env-6, env-8

alignment positions 1 · · · 180

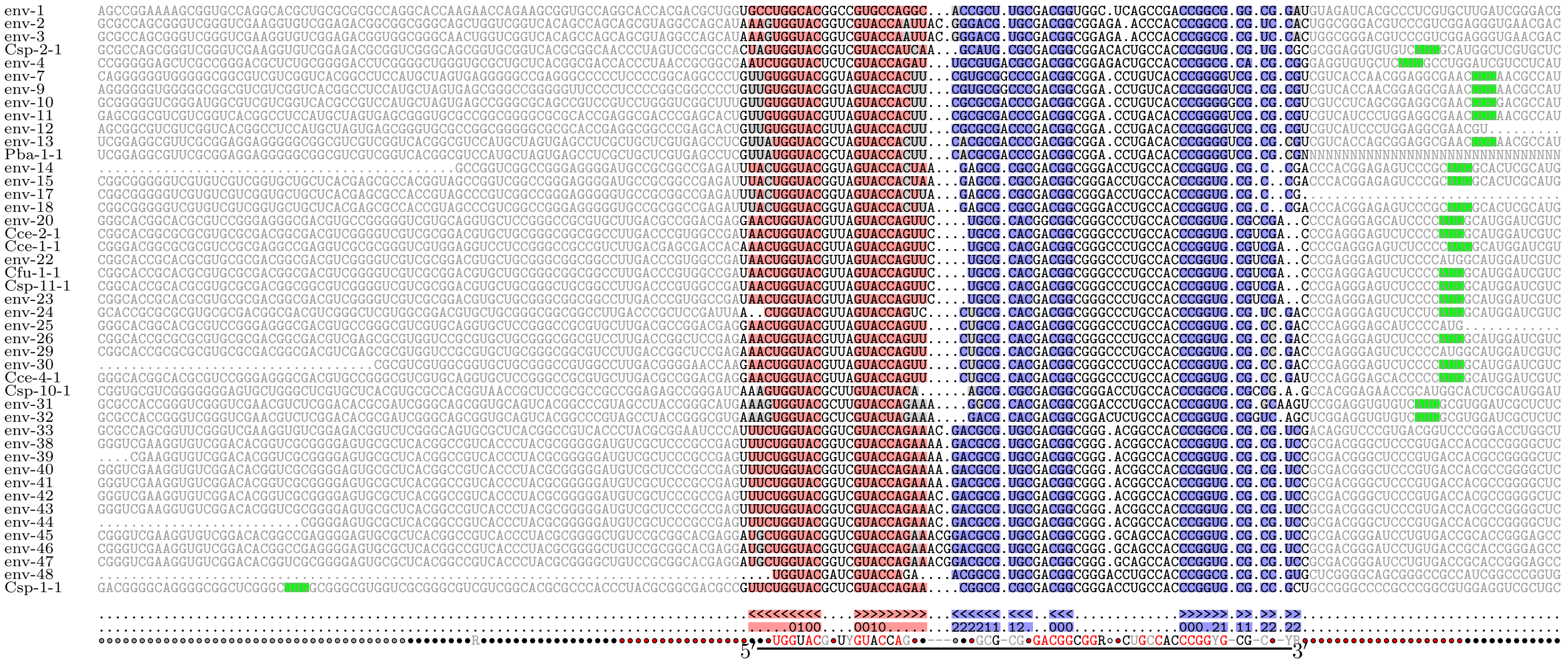

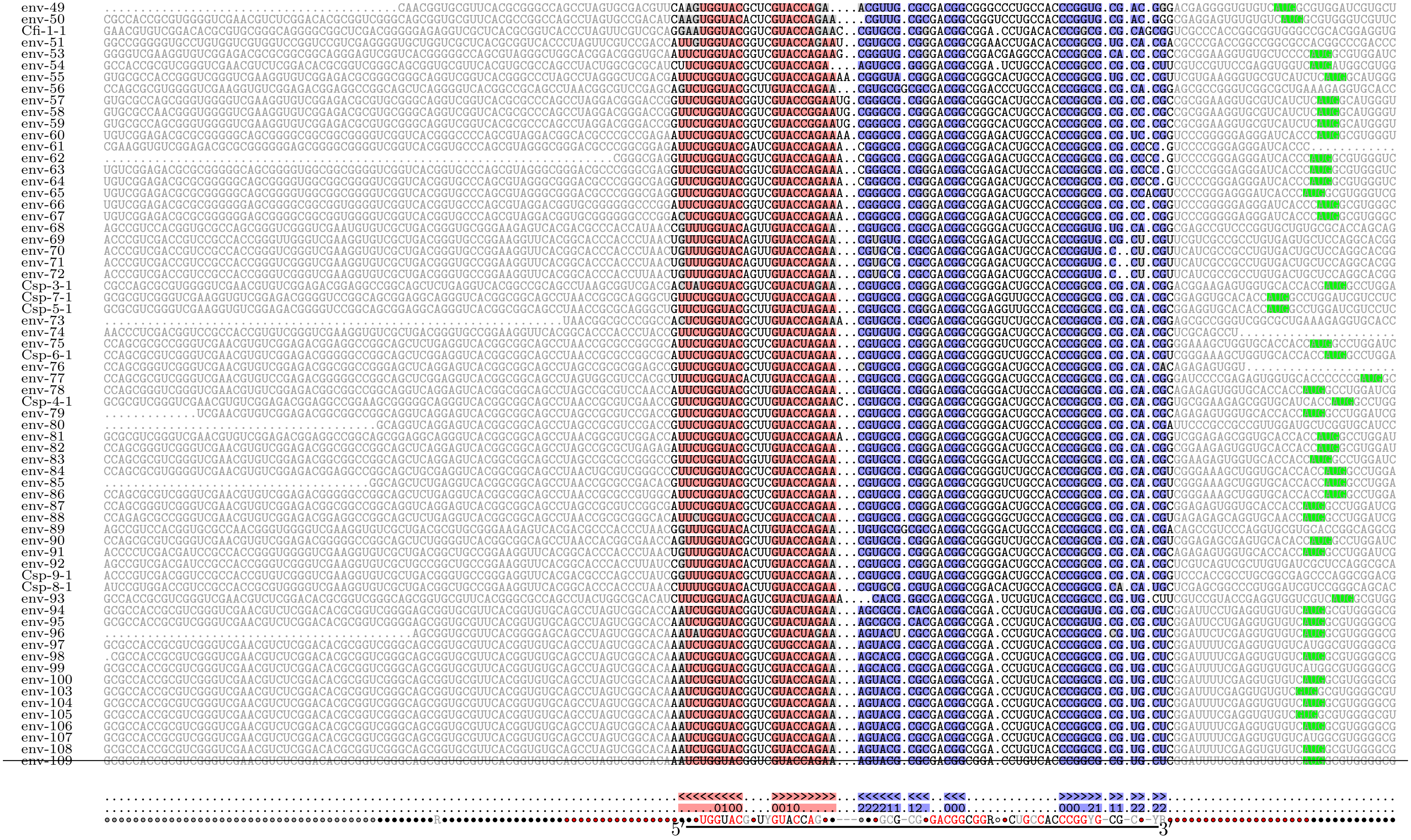

alignment positions 181 · · · 360

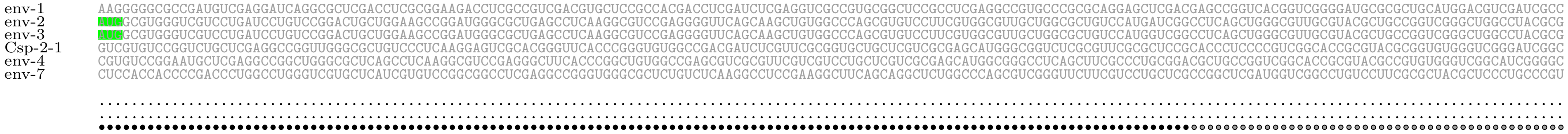

env-9	CUCCACCACCCCGACCCUGGCCUGGGUCGUGCUCAUCGUGUCCGGCGGCCUCGAGGCCGGGUGGGCGCUCUGUCUCAAGGCCUCCGAAGGCUUCAGCAGGCUCUGGCCCAGCGUCGGGUUCUUCGUCCUGCUCGCCGGCUCGAUGGUCGGCCUGUCCUUCGCGCUACGCUCCCUGCCCGU
env-10	CUCCACCACCACCACCAGCUCGACCCUCGGCUGGGUCGUCCUCAUCGCGUCCGGCGGCCUGGAGGCCGGCUGGGCGCUGUGCCUCAAGGCCUCGGAGGGCUUCAGCAGGCUCUGGCCCACCGUCGGGUUCGUCGUCCUGCUCGUCGGCUCCAUGGUCGGGCUCUCCUACGCGCUGCGGUC
env-11	CUCCACCACCCCGACCCUCGCCUGGGUCGUCCUCAUCGCGUCGGGCGGGCUCGAGGCCGGCUGGGCCCUGUGCCUCAAGGCGUCCGACGGGUUCAGCCGGCUCUGGCCCAGCAUCGGGUUCGUCGUCCUGCUGGUCGGCUCCAUGGUCGGCCUGUCCUACGCGCUACGCUCCCUGCCCGU
env-12	....
env-13	CUCCACCACCACCGGUACCGGGCUCGCCUGGGGCGUCCUCAUCGUGUCGGGAGGGUUCGAGGCCGGCUGGGCGCUGUGCCUCAAGGCCUCGCACGGUUUCAGCAGGCUCUGGCCGAGCGUCGGGUUCGUCGUCCUGCUCGCCGCCUCGAUGGUCGGUCUGUCCUAUGCGUUACGCUCCCU
Pba-1-1	NNNNNNNNNNNNNNNNNNNNNNNNNNNNNNNNNNNNNNNNNNNNNNNNNNNNNNNNNNNNNNNNNNNNNNNNNNNNNNNNNNNNNNNNNNNNNNNNNNNNNNNNNNNNNNNNNNNNNNNNNNNNNNNNNNNNNNNNNNNNNNNNNNNNNNNNNNNNNNNN
env-14	GAUCGUCCUCGUCCUGUCCGGAGCGCUCGAGGCCGGCUGGGCCCUGAGCCUCAAGGCCUCGGAAGGCUUCAGCAAGCUCUGGCCCAGCGUCUCGUUCGCCGUCCUGCUCGUCCUGAGCAUGGUCGGGCUGAGCUACGCCCUGAAGACCCUGCCCGUCGGCGUCGCGUACGGCGUCUGGGU
env-15	GAUCGUCCUCGUCCUGUCCGGAGCGCUCGAGGCCGGCUGGGCCCUGAGCCUCAAGGCCUCGGAGGGCUUCAGCAGGCUCUGGCCCAGCGUCUCGUUCGCCGUCCUGCUCGUCCUGAGCAUGGUCGGGCUGAGCUACGCCCUCAAGACGCUGCCCGUCGGCGUCGCGUACGGCGUCUGGGU
env-17	....
env-18	GAUCGUCCUCGUCCUGUCCGGAGCGCUCGAGGCCGGCUGGGCCCUGAGCCUCAAGGCCUCGGAGGGCUUCAGCAGGCUCUGGCCCAGCGUCUCGUUCGCCGUCCUGCUCGUCCUGAGCAUGGUCGGGCUGAGCUACGCCCUCAAGACGCUGCCCGUCGGCGUCGCGUACGGCGUCUGGGU
env-20	CUCAUCGCGUCCGGCAUGCUCGAGGCCGGCUGGGCGCUGAGCCUCAAGGCCUCGCACGGCUUC...
Cce-2-1	CUCAUCCUCUCCGGCAUGCUCGAGGCCGGCUGGGCCCUGAGCCUCAAGGCGUCGCACGGCUUCACCAAGCUCUGGCCCAGCGUCGCGUUCGUCGUCAUGCUCGUCCUGAGCAUGGUCGGCCUGAGCAUCGCCCUGAAGUCCUUGCCCGUCGGCGUGGCCUACGGUGUCUGGGUGGGGAUC
Cce-1-1	CCUCAUCGUGUCCGGCAUGCUCGAGGCCGGCUGGGCCCUGAGCCUCAAGGCGUCGCACGGCUUCACCAAGCUCUGGCCGAGCGUCGCGUUCGUCGUCAUGCUCGUCCUGAGCAUGGUCGGCCUCAGCAUCGCGCUGAAGUCGCUGCCCGUCGGCGUGGCCUACGGUGUCUGGGUGGGGAU
env-22	CUCAUCCUC...
Cfu-1-1	CUCAUCCUCUCCGGCAUGCUCGAGGCCGGCUGGGCCCUGAGCCUCAAGGCGUCGCACGGCUUCACCAAGCUCUGGCCCAGCGUCGCGUUCGUCGUCAUGCUCGUCCUGAGCAUGGUCGGCCUGAGCAUCGCCCUGAAGUCCCUGCCCGUCGGCGUGGCCUACGGUGUCUGGGUGGGGAUC
Csp-11-1	CUCAUCCUCUCUGGCAUGCUCGAGGCCGGCUGGGCCCUGAGCCUCAAGGCGUCGCACGGCUUCACCAAGCUCUGGCCGAGCGUCGCGUUCGUCGUCAUGCUCGUCCUGAGCAUGGUCGGCCUGAGCAUCGCCCUGAAGUCCUUGCCCGUCGGCGUGGCCUACGGUGUCUGGGUAGGGAUC
env-23	CUCAUCCUCUCCGGCAUGCUCGAGGCCGGCUGGGCCCUGAGCCUCAAGGCGUCGCACGGCUUCACCAAGCUCUGGCCCAGCGUCGCGUUCGUCGUCAUGCUCGUCCUGAGCAUGGUCGGCCUGAGCAUCGCCCUGAAGUCCUUGCCCGUCGGCGUGGCCUACGGUGUCUGGGUGGGGAUC
env-24	CUCAUCCUCUCCGGCAUGCUCGAGGCCGGCUGGGCCCUGAGCCUCAAGGCGUCGCACGGCUUCACCAGGCUCUGGCCGAGCGUCGCGUUCGUCGUCAUGCUCGUCCUGAGCAUGGUCGGCCUGAGCAUCGCCCUGAAGUCCUUGCCCGUCGGCGUGGCCUACGGUGUCUGGGUGGGGAUC
env-25	....
env-26	CUCAUCCUCUCCGGCAUGCUCGAGGCCGGCUGGGCUCUCAGCCUCAAGGCGUCGCACGGCUUCACGAAGCUGUGGCCGAGCGUCGCGUUCGUCGUCAUGCUCGUCCUGAGCAUGGUCGGCCUGAGCAUCGCCCUGAAGUCCCUGCCCGUCGGCGUGGCCUACGGUGUCUGGGUGGGGAUC
env-29	CUCAUCCUCUCCGGCAUGCUCGAGGCCGGCUGGGCUCUCAGCCUCAAGGCG...
env-30	CUCAUCGCGUCCGGCAUGCUCGAGGCCGGCUGGGCGCUGAGCCUCAAGGCGUCGCACGGCUUCACGAAGCUGUGGCCGAGCGUCGCGUUCGUCGUCAUGCUCGUCCUGAGCAUGGUCGGGCUGAGCAUCGCGCUGAAGUCGCUGCCCGUCGGCGUGGCCUACGGUGUCUGGGUGGGGAUC
Cce-4-1	CUCGUCGCGUCCGGCAUGCUCGAGGCCGGCUGGGCGCUGAGCCUCAAGGCCUCGCACGGCUUCACGCGCCUGUGGCCCAGCGUCGGGUUCGUCGUCAUGCUCGUGCUGAGCAUGGUCGGGCUGAGCAUCGCCCUGAAGUCGCUGCCCGUGGGCGUGGCCUACGGUGUCUGGGUGGGGAUC
Csp-10-1	CGUCCUCGUCCUGUCCGGCAUGCUCGAGGCGGGCUGGGCCCUGAGCCUCAAGGCGUCGGAGGGCUUCACCCGGCUCUGGCCGAGCGUGUCCUUCGUCGCCAUGCUCGCGCUGAGCAUGGUCGGGCUGAGCUAUGCCUCAGGACCCUGCCCGUGGGCGUCGCCUACGGCGUGUGGGUGGGG
env-31	GUCGUCGCUGGUCUGCUCGAGGCGGGCUGGGCCCUGCUGCUCAAGGAGUCGCACGGGUUCACCCGGCUGUGGCCGAGCGUGGGCUUCGGCGUGCUGCUCGCGCUCUCGAUGGGCGGCCUCGCGUUCGCGCUGCGGACCCUGCCCGUCGGCACGGCCUACGCGGUGUGGGUCGGCAUCGGC
env-32	GUCGUCGCUGGUCUGCUCGAGGCGGGCUGGGCCCUGCUGCUCAAGGAGUCGCACGGGUUCACCCGGCUGUGGCCGAGCGUGGGCUUCGGCGUGCUGCUCGCGCUCUCGAUGGGCGGCCUCGCGUUCGCGCUGCGGACCCUGCCCGUCGGCACGGCGUACGCGGUGUGGGUCGGCAUC...
env-33	CAGCACGGAGGUACCACCGACAUGCCAUGGUUCGUCCUCGUCGUCUCCGGCCUCCUCGAGGCCGGCUGGGCGCUGUCGCUCAAGUCCUCGCACGGCUUCACCCGGCUGGUCCCGUCGGUGCUGUUCGUCGUCCUGCUGGUCGCUGAGCAUGGGCGGCUCGCGUACGCCCUGCGCUCGCUG
env-38	GUCGGUCGAGCACGGAGGUGCUUCUCCCAUGGCAUGGCUCGUCGUCUCCGUCGUCUCCGGUCUGCUCGAGGCCGGCUGGGCGCUCGCCCUCAAGUCGUCGGACGGCUUCACCAAGCUCGUCCCCACCGUCACGUUCGUCGGCUGCUCCUGCUGAGCAUGGGCGGGCUCGCGUACGCGCUGCG
env-39	GUCGGUCGAGCACGGAGGUGCUUCUCCCAUGGCAUGGCUCGUCGUCUCCGUCGUCUCCGGUCUGCUCGAGGCCGGCUGGGCGCUCGCCCUCAAGUCGUCGGACGGCUUCACCAAGCUCGUCCCCACCGUCACGUUCGUCGGCUGCUCCUGCUGAGCAUGGGCGGGCUCGCGUACGCGCUGCG
env-40	GUCGGUCGAGCACGGAGGUGCUUCUCCCAUGGCAUGGCUCGUCGUCUCCGUCGUCUCCGGUCUGCUCGAGGCCGGCUGGGCGCUCGCCCUCAAGUCGUCGGACGGCUUCACCAAGCUCGUCCCCACCGUCACGUUCGUCGUCCUGCUCGUGCUGAGCAUGGGCGGGCUCGCGUACGCGCUGCG
env-41	GUCGGUCGAGCACGGAGGUGCUUCUCCCAUGGCAUGGCUCGUCGUCUCCGUCGUCUCCGGUCUGCUCGAGGCCGGCUGGGCGCUCGCCCUCAAGUCGUCGGACGGCUUCACCAAGCUCGUCCCCACCGUCACGUUCGUCGUCCUGCUCGUGCUGAGCAUGGGCGGGCUCGCGUACGCGCUGCG
env-42	GUCGGUCGAGCACGGAGGUGCUUCUCCCAUGGCAUGGCUCGUCGUCUCCGUCGUCUCCGGUCUGCUCGAGGCCGGCUGGGCGCUCGCCCUCAAGUCGUCGGACGGCUUCACCAAGCUCGUCCCCACCGUCACGUUCGUCGUCCUGCUCGUGCUGAGCAUGGGCGGGCUCGCGUACGCGCUGCG
env-43	GUCGGUCGAGCACGGAGGUGCUUCUCCCAUGGCAUGGCUCGUCGUCUCCGUCGUCUCCGGUCUGCUCGAGGCCGGCUGGGCGCUCGCCCUCAAGUCGUCGGACGGCUUCACCAAGCUGGUCCCCACCGUCACGUUCGUCGUCCUGCUCGUGCUGAGCAUGGGCGGGCUCGCGUACGCGCUGCG
env-44	GUCGGUCGAGCACGGAGGUGCUUCUCCCAUGGCAUGGCUCGUCGUCUCCGUCGUCUCCGGUCUGCUCGAGGCCGGCUGGGCGCUCGCCCUCAAGUCGUCGGACGGCUUCACCAAGCUCGUCCCCACCGUCACGUUCGUCGGCUGCUCCUGCUGAGCAUGGGCGGGCUCGCGUACGCGCUGCG
env-45	CGUCGGUCCGCACGGAGGUGCUUCCCCCAUGGCAUGGCUCGUCGUCUCCGUCGUCUCCGGUCUGCUCGAGGCCGGCUGGGCGCUCGCCCUCAAGUCGUCGGACGGCUUCACCAAGCUCGUCCCCACCGUCACGUUCGUCGGCUGCUCCUGCUGAGCAUGGGCGGGCUCGCGUACGCGCUGCG
env-46	CGUCGGUCCGCACGGAGGUGCUUCCCCCAUGGCAUGGCUCGUCGUCUCCGUCGUCUCCGGUCUGCUCGAGGCCGGCUGGGCGCUCGCCCUCAAGUCGUCGGACGGCUUCACCAAGCUCGUCCCCACCGUCACGUUCGUCGGCUGCUCCUGCUGAGCAUGGGCGGGCUCGCGUACGCGCUGCG
env-47	CGUCGGUCCGCACGGAGGUGCUUCCCCCAUGGCAUGGCUCGUCGUCUCCGUCGUCUCCGGUCUGCUCGAGGCCGGCUGGGCGCUCGCCCUCAAGUCGUCGGACGGCUUCACCAAGCUGGUCCCCACCGUCACGUUCGUCGUCCUGCUCGUGCUGAGCAUGGGCGGGCUCGCGUACGCGCUGCG
env-48	GUCCCGGCAGGAGGUCACUCUCAUGGCAUGGCUGAUCCUCGUGGUGUCCGGGCUGCUCGAGGCGGUCUGGGCGCUCGCGCUCAAGGAGUCGCACGGGUUCUCGCGGCUGUGGCCGUCGCUCGUGUUCGUCGUCGGGCUGGGCGCCAGCAUGGGCGGACUGGCGCUGGCGCUGCGCACGCU
Csp-1-1	CAUGGCAUGGAUGGUCCUCGUGGUGUCCGGGCUGCUCGAGGCGGUCUGGGCACUCGCCCUCAAGGAGUCGAACGGCUUCACGCGGCUGUGGCCGAGCGUCGUCUUCGUGGCCGGUCUGGCCGCCAGCAUGGGCGGCUCGCGAUCGCGCUGCGCACCCUGCCGGUCGGCACGGCGUACGC
env-49	GGUGAUCUCGGGGUUGCUCGAGGCGGGUUGGGCGCUCCUGCUCAAGGAGUCGCACGGGUUCACCCGGCUGUGGCCGAGCAUCGGCUUCGGCGUGCUCCUCGCGGCGUCGAUGACCGGGCUCGCCUUCGCGCUGCGGACGCUGCCGGUCGGGACGGCGUACGCGGUGUGGGUCGGCAUCGG
env-50	UGGUGAUCUCGGGUCUGCUCGAGGCGGGAUGGGCGCUGCUGCUCAAGGAGUCGCACGGGUUCACGCGACUGUGGCCGAGCGUCGGCUUCGGAGUCCUCCUCGCCGCGUCGAUGGCCGGGCUGGCGUUCGCGCUGCGCACGCUUCCGGUCGGGACCGCCUACGCCGUGUGGGUGGGCAUCG
Cfi-1-1	CAUCCCCAUGGCAUGGAUCGUCCUCGUCGUCUCCGGCAUGCUCGAGGCGGGCUGGGCUCUUGCGCUCAAGCAGUCGCACGGCUUCACCCGGCUCUGGCCGAGCAUCUGGUUCGUCGUGUUCGCCGUCGCGUCGUUCGCCGGGCUCAGCUUCGCGCUGCGCUCGUUGCCCGUCGGCGUCGC
env-51	GGCGGGCAACGGAGGUGCACCCCCAUGGCCUGGAUCGUCCUCAUCGUCUCCGGCGUGCUGGAGGCCGGCUGGGCGCUCAGCCUCAAGGAGUCGCACGGCUUCACGCGGCUGUGGCCCAGCGUGUGGUUCGUCGUCUUCGCCGUCGCGUCGUUCGCGGGCCUCAGCUUCGCGCUGCGCUC
env-53	GUCCUCAUCGUGUCCGGCCUGCUCGAGGCCGGCUGGGCGCUCAGCCUCAAGGCGUCGCACGGCUUCACGAGGCUGUGGCCGAGCGUCGUCUUCGUCGUGCUCAGCUGGGCGCUCGCGCUCGCCGUCGGCGUCGCGUACGGCGUCUGGGUCGGCGUGGUGCCGCGCGACAGCGAUC
env-54	GUCGUGCUGGUGGUGUCGGGUCUGCUCGAGGCGGGCUGGGCGUUGUGCCUCAAGGAGUCGCACGGGUUCACCCGCCUGUGGCCGAGCGUCGGCUUCGGCGUGCUGGCCGCGAGCAUGGCCGGGCUCGCGUUCGCGCUGCGCACGCUCCCUGUCGGCGGGGCGUACUCCGUGUGGGUG
env-55	UCGUGCUGAUCCUCUCCGGGCUGCUCGAGGCGGGCUGGGCGCUGAGCCUCAAGGCCUCCGACGGCUUCACGAAGCUGUGGCCCAGCGUCUUCGUCGCCGUGUUCGCGGUGGCGUCGUUCGCCGGCUGAGCUGGGCGCUGCGCUCGCUGCCCGUCGGCCCGCGUACGGCGUCUGGGUCG
env-56	ACCAUGGCCUGGAUCGUCCUCAUCCUCUCCGGCAUGCUCGAGGCCGGCUGGGCGCUCAGCCUCAAGGCGUCCGACGGCUUCACCAAGCUCUGGCCGAGCGUGGCGUUCGUCGUCUUCGCCGUCGCGUCGCUGGGCGGCCUGAGCUGGGCGCUCAAGUCGCUGCCCGUGGGCGUCGCGUAC
env-57	CGUCCUCAUCCUGUCCGGGCUGCUCGAGGCGGGCUGGGCCCUCAGCCUCAAGGCCUCCGACGGGUUCUCCCGGCUGUGGCCGAGCGUCUCGUUCGUCGCGUUCGCCGUGGCCUCGUUCGCCGGGCUGAGCUGGGCGCUGCGCACGCUGCCGGUCGGCACCGCGUACGGCGUCUGGGUCGG
env-58	CGUCCUCAUCCUGUCCGGGCUGCUCGAGGCGGGCUGGGCCCUCAGCCUCAAGGCCUCCGACGGGUUCUCCCGGCUGUGGCCGAGCGUCUCGUUCGUCGCGUUCGCCGUGGCCUCGUUCGCCGGGCUGAGCUGGGCGCUGCGCACGCUGCCGGUCGGCACCGCGUACGGCGUCUGGGUCGG
env-59	CGUCCUCAUCCUGUCCGGGCUGCUCGAGGCGGGCUGGGCCCUCAGCCUCAAGGCCUCCGACGGGUUCUCCCGGCUGUGGCCGAGCGUCUCGUUCGUCGCGUUCGCCGUGGCCUCGUUCGCCGGGCUGAGCUGGGCCCUGCGCACGCUGCCGGUCGGCACCGCGGCCGGCAGCUACGCGAU
env-60	CGUGCUCAUCGUGUCCGGACUGCUCGAGGCCGGCUGGGCGCUCAGCCUCAAGGCGUCGGACGGCUUCACGAAGCUGUGGCCGAGCGUGUCGUUCGGGGUGCUGGCCAUCCUGUCGUUCGCCGGCCUGAGCUGGGCGCUGCGGUCGCUGCCGGUCGGCGUCGCGUACGGCGUCUGGGUCGG
env-61	....
env-62	GUGCUCAUCGUGUCCGGACUGCUGGAGGCGGGCUGGGCGCUCAGCCUCAAGGCGUCGGACGGCUUCACGAAGCUGUGGCCGAGCGUGUCGUUCGGCGUGCUGGCGAUCCUGUCGUUCGCCGGCCUCAGCUGGGCGCUGCGGUCGCUGCCGGUCGGGGUCGCGUACGGCGUCUGGGUCGGC
env-63	GUGCUCAUCGUGUCCGGACUGCUGGAGGCGGGCUGGGCGCUCAGCCUCAAGGCGUCGGACGGCUUCACGAAGCUGUGGCCGAGCGUGUCGUUCGGCGUGCUGGCGAUCCUGUCGUUCGCCGGCCUCAGCUGGGCGCUGCGGUCGCUGCCGGUCGGGGUCGCGUACGGCGUCUGGGUCGGC
env-64	GUGCUCAUCGUGUCCGGACUGCUGGAGGCGGGCUGGGCGCUCAGCCUCAAGGCGUCGGACGGCUUCACGAAGCUGUGGCCGAGCGUGUCGUUCGGCGUGCUGGCGAUCCUGUCGUUCGCCGGCCUCAGCUGGGC..
env-65	UGCUCAUCGUGUCCGGACUGCUGGAGGCGGGCUGGGCGCUCAGCCUCAAGGCGUCGGACGGCUUCACGAAGCUGUGGCCGAGCGUGUCGUUCGGCGUGCUGGCGAUCCUGUCGUUCGCCGGCCUCAGCUGGGCGCUGCGGUCGCUGCCGGUCGGGGUCGCGUACGGCGUCUGCAGUCAUG
env-66	CGUGCUCAUCGUGUCCGGACUGCUCGAGGCGGGCUGGGCGCUCAGCCUCAAGGCGUCGGACGGCUUCACGAGGCUGUGGCCGAGCGUGUCGUUCGGCGUGCUGGCGAUCCUGUCGUUCGCCGGCCUCAGCUGGGCGCUGCGCUCGCUGCCGGUCGGCGUCGCGUACGGCGUGUGGGUCGG
env-67	CGUGCUCAUCGUGUCCGGACUGCUCGAGGCGGGCUGGGCGCUCAGCCUCAAGGCGUCGGACGGCUUCACGAGGCUGUGGCCGAGCGUGUCGUUCGGCGUGCUGGCGAUCCUGUCGUUCGCCGGCCUCAGCUGGGCGCUGCGCUCGCUGCCGGUCGGCGUCGCGUACGGCGUGUGGGUCGG
env-68	GCGGCACGGAGGUGCAUCACCAUGGCGUGGAUCGUCCUGAUCGUGUCCGGCAUGCUCGAGGCGGGCUGGGCGCUCAGCCUGAAGGCGUCGGACGGCUUCACCAAGCUGUGGCCCAGCGUCGCGUUCGUCGUGCUGCUGGUCGGCUCGAUGGGUGGGCUCGCCUGGGCGCUCAAGACGCUG
env-69	AGGUGCUCGUCGCAUGGCAUGGGUCGUACUCAUCGUGUCCGGGAUGCUCGAGGCCGGCUGGGCGCUCAGCCUCAAGGCGUCCGACGGCUUCACCAGGCUCUGGCCCAGCGUGGCCUUCGUCCUGCUGCUCGCGGGAUCCAUGGGCGGACUGAGCUGGGCGCUGAAGACGUUGCCCGUCGG
env-70	AGGUGCUCGUCGCAUGGCAUGGGUCGUACUCAUCGUGUCCGGGAUGCUCGAGGCCGGCUGGGCGCUCAGCCUCAAGGCGUCCGACGGCUUCACCAGGCUCUGGCCCAGCGUGGCCUUCGUCCUGCUGCUCGCGGGAUCCAUGGGCGGACUGAGCUGGGCGCUGAAGACGUUGCCCGUCGG
env-71	AGGUGCUCGUCGCAUGGCAUGGGUCGUACUCAUCGUGUCCGGGAUGCUCGAGGCCGGCUGGGCGCUCAGCCUCAAGGCGUCCGACGGCUUCACCAGGCUCUGGCCCAGCGUGGCCUUCGUCCUGCUGCUCGCGGGAUCCAUGGGCGGACUGAGCUGGGCGCUGAAGACGUUGCCCGUCGG
env-72	AGGUGCUCGUCGCAUGGCAUGGGUCGUACUCAUCGUGUCCGGGAUGCUCGAGGCCGGCUGGGCGCUCAGCCUCAAGGCGUCCGACGGCUUCACCAGGCUCUGGCCCAGCGUGGCCUUCAUCCUGCUGCUCGCGGGAUCCAUGGGCGGACUGAGCUGGGCGCUGAAGACGUUGCCCGUCGG
Csp-3-1	UCGUCCUCGUCCUGUCCGGCGUGCUCGAGGCCGGCUGGGCCCUCAGCCUCAAGGCGUCCGACGGCUUCACCAAGCUCUGGCCCAGCGUCCUGUUCGUCGUCCUGCUCGUCGCGUCCAUGGGCGGCUGGCUGGGCGCUGAAGACGCUGCCGGUCGGCGUCGCGUACGGCGUGUGGGUCG
Csp-7-1	GUCCUGUCCGGCAUGCUCGAGGCGGGCUGGGCGCUCAGCCUCAAGGCGUCCGACGGCUUCACCAGGCUGUGGCCCAGCGUGACGUUCGUCGUCCUGCUCGUCGCGUCCAUGGGUGGCCUCAGCUGGGCCCUGAAGUCGCUCCCGGUCGGGGUCGCCUACGGCGUCUGGGUCGGCAUCGG
Csp-5-1	GUCCUGUCCGGCAUGCUCGAGGCGGGCUGGGCGCUCAGCCUCAAGGCGUCCGACGGCUUCACCAGGCUGUGGCCCAGCGUGACGUUCGUCGUCCUGCUCGUCGCGUCCAUGGGUGGUCUCAGCUGGGCCCUGAAGUCGCUCCCGGUCGGGGUCGCCUACGGCGUCUGGGUCGGCAUCGG
env-73	ACCAUGGCCUGGAUCGUCCUCAUCCUGUCCGGCAUGCUCGAGGCCGGCUGGGCCCUGAGCCUCAAGGCGUCCGACGGGUUCAGCAAGCUCGGACCGAGCAUCGCGUUCGUCGUCUUCGCCGUCGCGUCGCUGGGUGGGCUCAGCUGGGCGCUGAAGUCGCUGCCCGUCGGCGUCGCCUAC
env-74	....
env-75	GUCCUCGUCCUGUCCGGCAUGCUCGAGGCCGGCUGGGCGCUCAGCCUCAAGGCGUCCGACGGUUUCACCAAGCUCUGGCCCAGCGUCCUGUUCGUCGUCCUGCUCGUCGCGUCCAUGGGCGGCCUGGCGUGGGCGCUGAAGACGCUGCCCGUCGGCGUCGCGUACGGCGUCUGGGUCGGC
Csp-6-1	CCGUCCUCAUCCUCUCCGGCAUGCUCGAAGCCGGCUGGGCGCUGAGCCUCAAGGCAUCCGACGGCUUCACCAGGCUCUGGCCCAGCGUGCUGUUCGUCGUCCUGCUCGUCGCCUCCAUGGGCGGCCUGGCCUGGGCGCUCAAGACGCUGCCGGUGGGCGUCGCGUACGGCGUCUGGGUCG
env-76	....
env-77	CUGGAUCGUCCUCAUCGCGUCCGGCAUGCUCGAGGCCGGCUGGGCGCUGAGCCUGAAGGCGUCCGA..
env-78	UCCUCAUCCUGUCCGGCAUGCUCGAGGCCGGCUGGGCCCUGAGCCUCAAGGCGUCCGACGGGUUCAGCAAGCUCGGACCGAGCAUCGCGUUCGUCGUCUUCGCCGUCGCGUCGCUGGGCGGCUCAGCUGGGCGCUGAAGUCGCUGCCCGUCGGCGUCGCCUACGGGGUCUGGGUGGGCA
Csp-4-1	AUCGUCCUCAUCGUGUCCGGCAUGCUCGAGGCCGGCUGGGCGCUCAGCCUCAAGGCGUCGGACGGCUUCACCAAGCUCUGGCCCAGCAUCCUGUUCGUCGUCCUGCUCGUCGCGUCCAUGGGCGGCCUGGCCUGGGCGCUGAAGACGCUGCCCGUCGGCGUCGCGUACGGCGUCUGGGUC
env-79	UCCUCAUCCUGUCCGGCAUGCUCGAGGCCGGCUGGGCGCUGAGCCUCAAGGCGUCCGACGGGUUCAGCAAGCUCGGACCGAGCAUCGCGUUCGCGGUCUUCGCCGUCGCGUCGCUGGGCGGCUCAAGUCGCUGCCCGUCGGCGUCGCCUACGGCGUCUGGGUGGGCA
env-80	GUCCGGCGGCACGGAGGUGGAUCACCAUGGCGUGGAUCGUUCUCAUCGUGUCCGGCAUGCUCGAGGCGGGCUGGGCGCUGAGCCUCAAGGCGUCCGACGGCUUCACCAAGCUGUGGCCCAGCGUCGCCUUCGUCCUGCUGCUCGUCGGCUCGAUGGGCGGACUCGCCUGGGCGCUCAAGA
env-81	CGUCCUCAUCGUGUCCGGCAUGCUCGAGGCCGGCUGGGCGCUCAGCCUCAAGGCGUCCGACGGCUUCACCCGGCUCUGGCCCAGCGUCCUGUUCGUCGUCCUGCUCGUCGCGUCGAUGGGCGGCCUUGCGUGGGCGCUCAAGACCCUGCCGGUCGGCGUC...................
env-82	CGUCCUCGUCCUGUCCGGCAUGCUCGAGGCCGGCUGGGCCCUGAGCCUCAAGGCGUCCGACGGGUUCAGCAAGCUCGGACCGAGCAUCGCGUUCGUCGUCU..
env-83	GUCCUCGUCCUGUCCGGCAUGCUCGAGGCCGGCUGGGCACUGAGCCUCAAGGCGUCCGACGGGUUCAGCAAGCUCGGACCGAGCAUCGCGUUCGUCGUCUUCGCCGUCGCGUCGCUGGUUGGGCUCAGCUGGGCGCUGAAGUCG...................................
env-84	UCGUCCUCAUCCUGUCCGGCAUGCUCGAGGCCGGCUGGGCGCUCAGCCUCAAGGCGUCCGACGGGUUCACCAAGCUCUGGCCCGAGUGUCGUUCGUCGUCCUGCUCGUCGCGUCCAUGGGCGGCCUCGCCUGGGCGCUCAAGACGCUGCCGGUGGGCGUCGCGUACGGCGUCUGGGUCG
env-85	UCGUCCUCAUCCUGUCCGGCAUGCUCGAGGCCGGCUGGGCCCUGAGCCUCAAGGCGUCCGACGGGUUCAGCAAGCUCGGACCGAGCAUCGCGUUCGUCGUCUUCGCCGUCGCGUCGCUGGGCGGCUCAGCUGGGCGCUGAAGUCGCUGCCCGUCGGCGUCGCCUACGGCGU........
env-86	UCGUCCUCAUCCUGUCCGGCAUGCUCGAGGCCGGCUGGGCGCUGAGCCUCAAGGCGUCCGACGGCUUCACCAAGCUCUGGCCGAGCGUCCUGUUCGUCGUCCUGCUCGUCGCGUCCAUGGGUGGCCUCGCCUGGGCGCUCAAGUCGCUGCCGGUCGGCGUCGCGUACGGCGUCUGGGUCG
env-87	UCCUCAUCCUGUCCGGCAUGCUCGAGGCCGGCUGGGCCCUGAGCCUCAAGGCGUCCGACGGGUUCAGCAAGCUCGGACCGAGCAUCGCGUUCGUCGUCUUCGCCGUCGCGUCGCUGGGUGGGCUCAGCUGGGCGCUGAAGUCGCUGCCCGUCGGCGUCGCCUACGGCGUCUGGGUCGGCA
env-88	UCCUCGUCCUGUCCGGGAUGCUCGAGGCCGGCUGGGCGCUCAGCCUCAAGGCCUCCGACGGCUUCACCCGGCUCUGGCCCAGCGUCCUGUUCGUCGUGCUGCUCGUCGCGUCGAUGGGCGGCCUCGCCUGGGCCCUGAAGGCGCUGCCCGUGGGCGUCGCGUACGGCGUCUGGGUCGGCA
	....
	....
	●●○○○○○○○○○○○○○○○○○○○○○○○○○○○○○○○○○○○○○○

```
env-89    GCACGGAGGUGCAGCACCAUGGCGUGGAUCGUUCUCAUCGUUUCCGGCAUGCUCGAGGCCGGCUGGGCCCUCAGCCUCAAGGCGUCCGACGGCUUCACCAAGCUCUGGCCCAGCGUCGCGUUCGUCCUGCUGCUCGCCGGCUCGAUGGGCGGCCUGGCGUGGGCGCUCAAGACCCUGCCC
env-90    GUCCUCAUCCUGUCCGGCAUGCUCGAGGCCGGCUGGGCACUCAGCCUCAAGGCGUCCGACGGCUUCACCCG.............................................................................................................
env-91    UCCUCAUCCUGUCCGGCAUGCUCGAGGCCGGCUGGGCCCUGAGCCUCAAGGCGUCCGACGGGUUCAGCAAGCUCGGACCGAGCAUCGCGUUCGUCGUCUUCGCCGUCGCGUCGCUGGGCGGCUCAGCUGGGCGCUGAAGUCGCUGCCCGUCGGCGUCGCCUACGGCGUCUGGGUGGGCA
env-92    CGGAGGUGCAUCACCAUGGCGUGGGUCGUUCUCAUCGUGUCCGGCAUGCUCGAGGCGGGCUGGGCGCUGAGCCUCAAGGCGUCCGACGGCUUCACCAAGCUCUGGCCGAGCGUCGCCUUCGUCGUCAUGCUGGCCGCCUCCAUGGGCGGCCUGGCCUGGGCGCUCAAGACGUUGCCGGUC
Csp-9-1   GAGGUGCAUCACCAUGGCAUGGGUCGUUCUCAUCGUGUCCGGCAUGCUCGAGGCGGGCUGGGCACUGAGCCUCAAGGCGUCCGACGGCUUCACCAGGCUGUGGCCGAGCGUCCUCUUCGUCGUGAUGCUGGCCGCCUCCAUGGGAGGCCUGGCCUGGUCGCUCAAGACGUUGCCGGUCGG
Csp-8-1   UGCACGGGGGUGCAUCUCUAUGGCGUGGGUCGUCCUCAUCCUGUCCGGCACGCUGGAGGCCGGCUGGGCGCUGAGCCUCAAGGCGUCCGACGGCUUCACCAAGCUCUGGCCGAGCGUGCUCUUCGUCGUCAUGCUCGUCGCGUCGAUGGGCGGCCUGGCGUGGGCGCUCAAGUCGCUGCC
env-93    AUCGUGCUGGUGGUGUCGGGUCUGCUCGAGGCCGGCUGGGCCCUGUGUCUCAAGGAGUCCCACGGAUUCACGCGAUUGUGGCCGACGGUCGGGUUCGGGGUGCUGCUCGCGGCGAGCAUGGCCGGCCUCGCGUUCGCGCUGCGCACCCUGCCGGUCGGCGGUGCGUACUCGGUGUGGGUC
env-94    UGCUGGUCGUGUCGGGUCUGCUGGAAGCGGGCUGGGCGUUGUCCCUCAAGGCCUCCGACGGGUUCAGUCGGUUGUGGCCGUCCGUCUCGUUCGUCGUGCUGCUCGCCGCGAGCAUGGCCGGGCUGGCGUUCGCGCUGCGGUCGCUGCCGGUCGGGUCCGCGUACGCGGUGUGGGUGGGGA
env-95    UGCUGGUCGUGUCGGGUCUGCUGGAAGCGGGCUGGGCGUUGUCCCUCAAGGCCUCCGACGGGUUCAGUCGGUUGUGGCCGUCCGUCUCGUUCGUCGUGCUGCUCGCCGCGAGCAUGGCCGGGCUGGCGUUCGCGCUGCGGUCGCUGCCGGUCGGGUCCGCGUACGCGGUGUGGGUGGGGA
env-96    UGCUGGUCGUGUCGGGUCUGCUGGAAGCGGGCUGGGCGUUGUCCCUCAAGGCCUCCGACGGGUUCAGUCGGUUGUGGCCGUCCGUCUCGUUCGUCGUGCUGCUCGCUGCGAGCAUGGCCGGGCUGGCGUUCGCUCUGCGGUCGCUGCCGGUCGGGUCCGCGUACGCGGUGUGGGUGGGGA
env-97    UGCUGGUCGUGUCGGGUCUGCUGGAGGCGGGCUGGGCGUUGUCCCUC.....................................................................................................................................
env-98    UGCUGGUCGUGUCGGGUCUGCUGGAAGCGGGCUGGGCGUUGUCCCUCAAGGCCUCCGACGGGUUCAGUCGGUUGUGGCCGUCCGUCUCGUUCGUCGUGCUGCUCGCCGCGAGCAUGGCCGGGCUGGCGUUCGCGCUGCGGUCGCUGCCGGUCGGGUCCGCGUACGCGGUGUGGGUGGGGA
env-99    UGCUGGUCGUGUCGGGUCUGCUGGAGGCGGGCUGGGCGUUGUCCCUCAAGGCCUCC...........................................................................................................................
env-100   UGCUGGUCGUGUCGGGUCUGCUGGAGGCGGGCUGGGCGUUGUCCCUCAAGGCCUCCGACGGGUUCAGUCGGUUGUGGCCGUCCGUCUCGUUCGUCGUGCUGCUCGCUGCGAGCAUGGCCGGACUGGCGUUCGCGCUGCGGUCGCUGCCGGUCGGGUCCGCGUACGCGGUGUGGGUGGGGA
env-103   GCUGGUCGUGUCGGGUCUGCUGGAGGCGGGCUGGGCGUUGUCCCUCAAGGCCUCCGACGGGUUCAGUCGGUUGUGGCCGUCCGUCUCGUUCGUCGUGCUGCUCGCUGCGAGCAUGGCCGGACUGGCGUUCGCGCUGCGGUCGCUGCCGGUCGGGUCCGCGUACGCGGUGUGGGUGGGGAU
env-104   UGCUGGUCGUGUCGGGUCUGCUGGAGGCGGGCUGGGCGUUGUCCCUCAAGGCCUCCGACGGAUUCAGUCGGUUGUGGCCGUCCGUCUCGUUCGUCGUGCUGCUCGCUGCGAGCAUGGCCGGACUGGCGUUCGCGCUGCGGUCGCUGCCGGUCGGGUCCGCGUACGCGGUGUGGGUGGGGA
env-105   GCUGGUCGUGUCGGGUCUGCUGGAGGCGGGCUGGGCGUUGUCCCUCAAGGCCUCCGACGGGUUCAGUCGGUUGUGGCCGUCCGUCUCGUUCGUCGUGCUGCUCGC...........................................................................
env-106   UGCUGGUCGUGUCGGGUCUGCUGGAGGCGGGCUGGGCGUUGUCCCUCAAGGCCUCCGACGGGUUCAGUCGGUUGUGGCCGUCCGUCUCGUUCGUCGUGCUGCUCGCUGCGAGCAUGGCCGGA..........................................................
env-107   UGCUGGUCGUGUCGGGUCUGCUGGAGGCGGGCUGGGCGUUGUCCCUC.....................................................................................................................................
env-108   UGCUGGUCGUGUCGGGUCUGCUGGAGGCGGGCUGGGCGUUGUCCCUCAAGGCCUCCGACGGAUUCAGUCGGUUGUGGCCGUCCGUCUCGUUCGUCGUGCUGCUCGCUGCGAGCAUGGCCGGGCUGGCGUUCGCGCUGCGGUCGCUGCCGGUCGGGUCCGCGUACGCGGUGUGGGUGGGGA
env-109   UGCUGGUCGUGUCGGGUCUGCUGGAGGCGGGCUGGGCGUUGUCCCUCAAGGCCUCCGACGGGUUCAGUCGGUUGUGGCCGUCCGUCUCGUUCGUCGUGCUGCUCGCUGCGAGCAUGGCCGGGCUCGCGUUCGCGCUGCGGUCGCUGCCGGUCGGGUCCGCGUACGCGGUGUGGGUGGGGA

          ....................................................................................................................................................................................
          ....................................................................................................................................................................................
          ●●●●●●●●●●●●●●●●●●●●●●●●●●●●●●●●●●●●●●●●●●●●●●●●●●●●●●●●●●●●●●●●●●●●●●●●●●●●●●●●●●●●●●●●●●●●●●●●●●●●●●●●●●●●●●●●●●●●●●●●●●●●●●●●●●●◉◉◉◉◉◉◉◉◉◉◉◉◉◉◉◉◉◉◉◉◉◉◉◉◉◉◉◉◉◉◉◉◉◉◉◉◉◉◉◉◉◉◉◉◉◉
```

alignment positions 361 · · · 448

```
env-1     AUCUCGUCUCCCUCGGUCUCGUUCCUUGGAUCCGAUAGAUCGCCGUUCGACCGCUCGGACAAACGUCCUGCCGUAUCUCGGAUCCCAU
env-2     GUGUGGGUCGGCAUCGGUGCCGCCACCACCGCGGUGCUCGCGAUCAUCUGGCUGGGCGAGGGAGUGUCGAUGGGCAAGAUCGUGUCGC
env-3     GUGUGGGUCGGCAUCGGUGCCGCCACCACCGCGGUGCUCGCGAUCAUCUGGCUGGGCGAGGGAGUGUCGAUGGGCAAGAUCGUGUCGC
Csp-2-1   GCGGUGACGACGGCCGUGCUCGCGAUGACCUUCCUGGGCGAGUCCGUCUCGGUGCUCAAGGUCGUCUCGAUCCUGCUGAUCAUCGGCG
env-4     CGUCCUCACCGCGGUCCUCGCGAUCGUCUGGUUCCACGAGCCCGUGACGGUGCUCAAGGUGGUCUCGAUCGUGCUCAUCAUCGCCGGC
env-7     CGGCGUCGCGUACGGCGUGUGGGUCGGGAUCGGCGCGACGGCCACGGCCCUGUUGGCGAUCCCGCUCUUCGGGGAGACUGUCACGGUG
env-9     CGGCGUCGCGUACGGCGUGUGGGUCGGGAUCGGCGCGACGGCCACGGCCCUGUUGGCGAUCCCGCUCUUCGGGGAGACUGUCACGGUG
env-10    GCUCCCCGUCGGCGUCGCCUACGGCGUCUGGGUCGGGAUCGGUGCGACCGCCACGGCCCUGUUGGCGAUCCCCCUCUUCGGGGAGACU
env-11    CGGCGUCGCGUACGGCGUGUGGGUCGGCGUGGGCGCGACCGCGACGGCCCUGUUGGCGAUCCCGCUCUUCGGGGAGACUGUCACGGUG
env-12    ........................................................................................
env-13    GCCCGUGGGUGUCGCCUACGGCGUGUGGGUGGGCGUCGGCGCCACCGCGACGGCCCUGUUGGCGAUCCCGCUCUUCGGGGAGACUGUC
Pba-1-1   NNNNNNNNNNNNNNNNNNNNNNNNNNNNNNNNNNNNNNNNNNNNNNNNNNNNNNNNNNNNNNNNNNNNNNNNNNNNNNNNNNNNNNNN
env-14    GGGCAUCGGGGCCUCGCUGACCGCUAUCCUCGCCGUCGUCCUGUUCGACGAGCCCGUCACGGCCCUCAAGGUCAUCUCCCUCGUGCUG
env-15    GGGCAUCGGGGCCUCGCUGACCGCUAUCCUCGCCGUCGUCCUGUUCGACGAGCCCGUCACGGCCCUCAAGGUCAUCUCCCUCGUGCUG
env-17    ........................................................................................
env-18    GGGCAUCGGGGCCUCGCUGACCGCUAUCCUCGCCGUCGUCCUGUUCGACGAGCCCGUCACGGCCCUCAAGGUCAUCUCCCUCGUGCUG
env-20    ........................................................................................
Cce-2-1   GGUGCCUCGCUCACCGCCGUCCUCGCCGUCGUCCUGUUCGACGAGCCCGUCACGGUCCUCAAGGUGAUCUCCCUCGUGCUGAUCGUCG
Cce-1-1   CGGUGCCUCGUUGACCGCUGUCCUCGCCGUCGUCCUGUUCGACGAGCCCGUCACGAUCCUCAAGGUGGUCUCCCUCGUGCUGAUCGUC
env-22    ........................................................................................
Cfu-1-1   GGCGCCUCGCUCACCGCCGUCCUCGCCGUCGUCCUGUUCGACGAGCCCGUCACGAUCCUCAAGGUGAUCUCCCUCGUGCUGAUCGUCG
Csp-11-1  GGUGCCUCGCUCACCGCCGUCCUCGCCGUCGUCCUGUUCGACGAGCCCGUCACGGUCCUCAAGGUGAUCUCCCUCGUGCUGAUCGUCG
env-23    GGUGCCUCGCUCACCGCCGUCCUCGCCGUCGUCCUGUUCGACGAGCCCGUCACGGUCCUCAAGGUGAUCUCCCUCGUGCUGAUCGUCG
env-24    GGUGCCUCGCUCACCGCCGUCCUCGCCGUCGUCCUGUUCGACGAGCCCGUGACCGUCCUCAAGGUGAUCUCGCUGG............
env-25    ........................................................................................
env-26    GGUGCGUCGCUCACCGCCGUCCUCGCCGUCGUCCUGUUCGACGAGCCCGUGACGGUCCUCAAGGUGAUCUCCCUCGUGCUGAUCGUCG
env-29    ........................................................................................
env-30    GGUGCCUCGCUCACCGCCGUCCUCGCCGUCGUCCUGUUCGACGAGCCCGUGACCGUCCUCAAGGUGAUCU..................
Cce-4-1   GGCGCCGCGCUGACGGCGGUCCUCGCCGUCGUCCUGUUCGACGAGCCCGUGACCGUCCUCAAGGUGAUCUCCCUCGUGCUGAUCGUCG
Csp-10-1  AUCGCGCCGCGCUGACCGCCGUCCUCGCUAUCGUGCUGUUCGGCGAGACGGUGAGCGCGCUCAAGAUCGUCUCGCUGGUCCUGAUCAU
env-31    GCGGUGACGACCGCGGUGCUCGCGAUGGUGUUCCUCGACGAGCCCGCCACGAUCGCCAAGGUGGCCUCGAUCCUGCUGAUCAUCGCGG
env-32    ........................................................................................
env-33    CCCGUCGGCACCGCCUACGCGGUGUGGGUCGGCAUCGGUGCCGUCACCACGGCCGUCCUCGCCAUGUUCUGGUUCGACGAGCCGGCCA
env-38    CACGCUGCCGGUCGGCACCGGGUACGCGGUCUGGGUCGGCAUCGGCGCCGUCACCACGGCCGUGCUCGCGAUGAUCUGGCUCGACGAG
env-39    CACGCUGCCGGUCGGCACCGGGUACGCGGUCUGGGUCGGCAUCGGCGCCGUCACCACGGCCGUGCUCGCGAUGAUCUGGCUCGACGAG
env-40    CAGCCUGCCCGUCGGCACCGGGUACGCGGUCUGGGUCGGCAUCGGCGC........................................
env-41    CAGCCUGCCCGUCGGCACCGGGUGCGCGGUCUGGGUCGGCAUCGGCGCCGUCACCACGGCCGUGCUCGCGAUGAUCUGGCUCGACGAG
env-42    CAGCCUGCCCGUCGGCACCGGGUACGCGGUCUGGGUCGGCAUCGGCGCCGUCACCACGGCCGUGCUCGCGAUGAUCUGGCUCGACGAG
env-43    CACCCUGCCCGUCGGCACCGGGUACGCGGUCUGGGUCGGCAUCGGCGCCGUCACCACGGCCGUGCUCGCGAUGAUCUGGCUCGACGAG
env-44    CACGCUGCCGGUCGGCACCGGGUACGCGGUCUGGGUCGGCAUCGGCGCCGUCACCACGGCCGUGCUCGCGAUGAUCUGGCUCGCCGAG
env-45    CACGCUGCCGGUCGGCACCGGGUACGCGGUCUGGGUCGGCAUCGGCGCCGUCACCACGGCCGUGCUCGCGAUGAUCUGGCUCGACGAG
env-46    CACGCUGCCGGUCGGCACCGGGUACGCGGUCUGGGUCGGCAUCGGCGCCGUCACCAC...............................
env-47    CACCCUGCCCGUCGGCACCGGGUACGCGGUCUGGGUCGGCAUCGGCGCCGUCACCACGGCCGUGCUCGCGAUGAUCUGGCUCGACGAG
env-48    GCCGGUCGGCACGGCGUACGCGGUGUGGGUCGGCGUCGGCGCCGUCACGACCGCGGUGCUCGCGAUGGCGUUCCUGCACGAGCCGGUG
Csp-1-1   GGUCUGGGUCGGCAUCGGGGCCGUCACCACCGCGGUCCUCGCGAUGGCGUUCCUCGACGAGCCCGUCACGGUGCUCAAGGUCGUGUCG
env-49    GGCGGUGACCACGGCGGUGCUCGCGAUGGUGUUCCUCGACGAGCCGGCCACGAUCGCGAAGGUCGUCUCGAUCCUGCUCAUCAUCGCG
env-50    GGGUCGUCACGACGGCGGUGCUCUCGAUGCUCUUCCUCGACGAGCCGGCCACGAUCACGAAGGUCGUCUCGAUCCUGCUCAUCAUCGC
Cfi-1-1   CUACGGCGUCUGGGUCGGCGUCGGCGCCGCCACCACCGCGGUCGCCGCCGCCGUCCUGCUGCACGAGCGCCUGUCGGUGCUCGCCGUG
env-51    GCUGCCCGUCGGCGUCGCCUACGGCGUCUGGGUCGGCGUCGGUGCCGCGACCACCGCGGUCGCCGCCGCGAUCAUGCUGCACGAGCGC
env-53    CUCGCCAUCCUGUGGCUCGGGGAGUCCGUGUCGGUGCUGAAGAUCGUCUCGCUCGUGCUGAUCGUCGCCGGCGUCGUCGGGCUCAACC
env-54    GGCAUCGGUGCCGUCACGACCGCGGUGCUCGCGAUGACGUUCCUCGACGAGCCCGUGACGCUGGUCAAGGUCGUCUCGAUCGGGCUCA
env-55    GCGUCGGGGCCGCCACGACGGCGAUCCUGGCGAUCGUGUGGUUCGGCGAGUCCGUGUCGGUGCUGAAGAUCGUGUCGCUGGUGCUGAU

          ........................................................................................
          ........................................................................................
          ◉◉◉◉◉◉◉◉◉◉◉◉◉◉◉◉◉◉◉◉◉◉◉◉◉◉◉◉◉◉◉◉◉◉◉◉◉◉◉◉◉◉◉◉◉◉◉◉◉◉◉◉◉◉◉◉◉◉◉◉◉◉◉◉◉◉◉◉◉◉◉◉◉◉◉◉○○○○○○○○○○○○
```

```
env-56    GGCGUGUGGGUCGGCAUCGGCGCGGCGCUGACGGCCGUGCUCGCCGUCGUCCUGUUCCACGAGACGGUCUCGGUGCUGAAGAUCGUCU
env-57    CGUCGGGGCCGCCGCCACGGCGAUCCUCGCGAUGGUGUG.................................................
env-58    CGUCGGGGCCGCCGCCACGGCGAUCCUCGCGAUGGUGUGGCUCGGCGAGUCCGUCUCGGUGCUGAAGAUCGUCUCGCUGGUGCUGAUC
env-59    CGUGUACACGGCCACCGUGGAUGCCGAUGCGACCGGCACGCUGCGCAAUGCCGUGGUG..............................
env-60    CAUCGGGGCGUCGGCGACGGCGAUCCUCGCGAUCGUGCUGUUCGGCGAGUCGGUGUCGGUGCUGAAGAUCGUGUCGCUCGUGCUGAUC
env-61    ........................................................................................
env-62    AUCGGGGCGUCGGCGACCGCGGUCCUGGCGAUCGUGCUGUUCGGCGAGUCCGUGUCGGUGCUCAAGAUCGUGUCGCUCGUGCUGAUCG
env-63    AUCGGGGCGUCGGCGACCGCGGUCCUGGCGAUCGUGCUGUUCGGCGAGUCCGUGUCGGUGCUCAAGAUCGUGUCGCUCGUGCUGAUCG
env-64    ........................................................................................
env-65    UCUGUGGUCAUGGGGUCAAACUGCCAGGGGGCGCCGCGCGGGACAAUGAGGCCAUGGCCGAUCUCGACGACCUUCGCGACCGUUUCGC
env-66    CAUCGGCGCGUCGGCGACCGCGAUCCUGGCGAUCGUGCUGUUCGGCGAGUCCGUGUCGGUGCUGAAGAUCGUGUCGCUCGUGCUCAUC
env-67    CAUCGGCGCGUCGGCGACCGCGAUCCUGGCGAUCGUGCUGUUCGGCGAGUCCGUGUCGGUGCUGAAGAUCGUGUCGCUCGUGCUCAUC
env-68    CCCGUCGGCAUCGCGUACGGCGUCUGGGUCGGCAUCGGCGCGUCGCUGACGGCCGUCCUGGCAAUCGUCAUGUUCCACGAGUCGGUGU
env-69    GGUCGCGUACGGCGUCUGGGUCGGCAUCGGUGCCGCCCUCACCGCGGUGCUCGCGAUCGUCUUCCUCGGUGAGUCGGCGUCGGUGCUC
env-70    UGUCGCGUACGGCGUCUGGGUCGG................................................................
env-71    UGUCGCGUA...............................................................................
env-72    UGUCGCGUACGGCGUCUGGG....................................................................
Csp-3-1   GCAUCGGGGCGGCCGUCACGGCCGUGCUCGCGAUCUUCGUGUUCCACGAGCCCGUCUCCGUGCUGAAGAUCGUGUCGCUCCUCCUGAU
Csp-7-1   GCGGCGCUCACCGCGGUGCUCGCGGUCGUCCUGUUCCACGAGACCGUCUCGGUGCUGAAGAUCGUGUCGCUCGUGCUCAUCGUCGCCG
Csp-5-1   GCGGCGCUCACGGCGGUGCUCGCGGUCGUCCUGUUCCACGAGACCGUCUCGGUGCUGAAGAUCGUGUCGCUCGUGCUCAUCGUCGCCG
env-73    GGCGUCUGGGUCGGCAUCGGCGCCGCGCUCACCGCGGUGCUCGCCGUCGUGCUGUUCCACGAGACCGUCUCGGUGCUGAAGAUCGUGU
env-74    ........................................................................................
env-75    AUCGGGGCGGCCCUGACCGCCGUCCUCGCGACCGUCCUGUUCCACGAGCCCGUCUCGGUCCUCA........................
Csp-6-1   GCAUCGGCGCGGCGAUCACGGCCGUCCUCGCGAUCGUCGUCUUCGACGAGGCCAUCUCCGUGCUGAAGAUCGUGUCGCUCAUCCUGAU
env-76    ........................................................................................
env-77    ........................................................................................
env-78    UCGGCGCCGCCCUCACCGCGGUCCUCGCCGUCGUCCUGUUCCACGAGACCGUCUCGGUGCUGAAGAUCGUGUCGCUC...........
Csp-4-1   GGCAUCGGGGCGGCCGUCACCGCCGUCCUCGCGAUCGUCGUCUUCGACGAGGCGGUCUCGGUGCUGAAGAUCGUCUCGCUCGCGCUGA
env-79    UCGGCGCCGCCCUCACCGCCGUGCUCGCCAUCGUCCUGUUCCACGAGACGGUGUCGGUGCUGAAGAUCGUGUCGCUCGUGCUCAUCGU
env-80    CGUUGCCCGUCGGCGUCGCGU...................................................................
env-81    ........................................................................................
env-82    ........................................................................................
env-83    ........................................................................................
env-84    GCAUCGGGGCGGCCGUCACGGCCGUCCUCGCGAUCGCCGUGUUCGACGAGCCCGUCUCCGUGCUGAAGAUCGGCUCCCUCGUCCUCAU
env-85    ........................................................................................
env-86    GCAUCGGGGCGGCCGUCACGGCCGUCCUCGCGAUCGUCGUGUUCGACGAGCCCGUCUCCGUGCUGAAGAUCGGCUCCCUCGUCCUCAU
env-87    UCGGCGCCGCGCUCACCGCGGUGCUCGCCGUCGUGCUGUUC...............................................
env-88    UCGGCGCCGCCCUGACCGCGGUCCUCGCCAUCGUCUUCCUCCACGAGACGGUCUC.................................
env-89    GUG.....................................................................................
env-90    ........................................................................................
env-91    UCGGCGCCGCCCUCACCGCGGUGCUCGCCGUCGUCCUGUUCCACGAGACCGUC...................................
env-92    GGUGUCGCGUACGGAGUCUGGGUCGGGAUCGGCGCUGCACUGACCGCGAUCUUGGCGAUCGUGUUCCUCGGCGAGUCCGUGUCCGUGC
Csp-9-1   GGUCGCGUACGGAGUCUGGGUCGGCAUCGGGGCGGCGCUGACCGCGGUCCUGGCGAUCGUGUUCCUCGGCGAGUCCGUGUCUGUGCUC
Csp-8-1   CGUCGGUGUCGCGUACGGCGUCUGGGUGGGCAUCGGCGCGGCGCUCACCGCGGUCCUCGCCAUCGUGUUCCUGGGCGAGUCGGUGUCG
env-93    GGCAUCGGCGCCGUGACCACGGCGGUGCUCGCGAUGACGUUCCUCGACGAGCCCGUGACGGUGGUCAAGGUCGUCUCGAUCCUGCUCA
env-94    UCGGUGCCGUGACCACGGCCG...................................................................
env-95    UCGGUGCCGUGACCACGGCCGUGCUGGGGAUGACGGUGCUGGGCGAGCCGGCCUCGUUGCUCAAGGUCGCCUCGAUCCUGCUGAUCAU
env-96    UCGGGGCCGUGACCACGGCCGUGCUGGGCAUGACGGUGCUGGGCGAGCCGGCCUCG................................
env-97    ........................................................................................
env-98    UCGGUGCCGUGACCACGGCCGUGCUCGGCAUGACGGUGCUGGGCGAGCCGGCCUCGUUGCUGAAGAUCGUCUCGAUCCUGCUGAUCAU
env-99    ........................................................................................
env-100   UCGGUGCCGUGACCACGGCCGUGCUCGGCAUCACGGUGCUGGGUGAGCCCGCCUCGUUGCUCAAGGUCGUCUCGAUCCUGCUGAUCAU
env-103   CGGUGCCGUGACCACGGCCGUGCUCGGCAUCACGGUGCUGGGUGAGCCCGCCUCGUUGCUCAAGGUCGUCUCGAUCCUGCUGAUCAUC
env-104   UCGGUGCCGUGACCACGGCCGUGCUCGGCAUCACGGUGCUGGGUGAGCCCGCCUCGUUGCUCAAGGUCGUCUCGAUCCUGCUGAUCAU
env-105   ........................................................................................
env-106   ........................................................................................
env-107   ........................................................................................
env-108   UCGGUGCCGUGACCACGGCCGUGCUCGGCAUGACGGUGCUGGGCGAGCCGGCCUCGUUGCUCAAGGUCGUCUCGAUCCUGCUGAUCAU
env-109   UCGGUGCCGUGACCACGGCCGUGCUCGGCAUGACGGUGCUGGGUGAGCCCGCCUCGUUGCUCAAGGUCGUCUCUAUCCUGCUGAUCAU

          ........................................................................................
          ........................................................................................
          ●●●●●●●●●●●●●●●●●●●●●●●●●●●●●●●●●●●●●●●●●●●●●●●●●●●●●●●●●●●●●●●●●●●●●●●●●●●●○○○○○○○○○○○○
```

3 GGAM-3

3.1 Taxa

The taxonomy of each organism containing a putative GGAM-3 RNA is listed, with abbreviations identifying each hit (e.g., "Eco-1-1" and "Eco-1-2" might hypothetically represent two distinct RNAs in *E. coli*). The abbreviations will be used to identify each individual GGAM-3 RNA in Sections 3.2 and 3.4:.

**abbrev. of hits**	**taxonomy of species**
[me-1-1	Bacteria Proteobacteria Betaproteobacteria Burkholderiales Burkholderiaceae Burkholderia Burkholderia cepacia complex [Pseudomonas] mesoacidophila

Bce-1-1 to Bce-1-3	Bacteria Proteobacteria Betaproteobacteria Burkholderiales Burkholderiaceae Burkholderia Burkholderia cepacia complex *Burkholderia cenocepacia*
Bce-2-1	Bacteria Proteobacteria Betaproteobacteria Burkholderiales Burkholderiaceae Burkholderia Burkholderia cepacia complex *Burkholderia cenocepacia* AU 1054
Bce-3-1	Bacteria Proteobacteria Betaproteobacteria Burkholderiales Burkholderiaceae Burkholderia Burkholderia cepacia complex *Burkholderia cenocepacia* HI2424
Bce-4-1 to Bce-4-3	Bacteria Proteobacteria Betaproteobacteria Burkholderiales Burkholderiaceae Burkholderia Burkholderia cepacia complex *Burkholderia cepacia*
Bce-5-1	Bacteria Proteobacteria Betaproteobacteria Burkholderiales Burkholderiaceae Burkholderia Burkholderia cepacia complex *Burkholderia cepacia* ATCC 25416
Bce-6-1	Bacteria Proteobacteria Betaproteobacteria Burkholderiales Burkholderiaceae Burkholderia Burkholderia cepacia complex *Burkholderia cepacia* JBK9
Bla-1-1 to Bla-1-2	Bacteria Proteobacteria Betaproteobacteria Burkholderiales Burkholderiaceae Burkholderia Burkholderia cepacia complex *Burkholderia lata*
Bpa-1-1	Bacteria Proteobacteria Betaproteobacteria Burkholderiales Burkholderiaceae Burkholderia Burkholderia cepacia complex *Burkholderia paludis*
Bps-1-1	Bacteria Proteobacteria Betaproteobacteria Burkholderiales Burkholderiaceae Burkholderia Burkholderia cepacia complex *Burkholderia pseudomultivorans*
Bpu-1-1	Bacteria Proteobacteria Betaproteobacteria Burkholderiales Burkholderiaceae Burkholderia Burkholderia cepacia complex *Burkholderia puraquae*
Bse-1-1	Bacteria Proteobacteria Betaproteobacteria Burkholderiales Burkholderiaceae Burkholderia Burkholderia cepacia complex *Burkholderia seminalis*
Bsp-1-1	Bacteria Proteobacteria Betaproteobacteria Burkholderiales Burkholderiaceae Burkholderia Burkholderia cepacia complex *Burkholderia sp.* 383
Bsp-2-1	Bacteria Proteobacteria Betaproteobacteria Burkholderiales Burkholderiaceae Burkholderia Burkholderia cepacia complex *Burkholderia sp.* MSMB1072
Bsp-3-1	Bacteria Proteobacteria Betaproteobacteria Burkholderiales Burkholderiaceae Burkholderia Burkholderia cepacia complex *Burkholderia sp.* MSMB1078WGS
Bsp-4-1	Bacteria Proteobacteria Betaproteobacteria Burkholderiales Burkholderiaceae Burkholderia Burkholderia cepacia complex *Burkholderia sp.* MSMB1459WGS
Bsp-5-1	Bacteria Proteobacteria Betaproteobacteria Burkholderiales Burkholderiaceae Burkholderia Burkholderia cepacia complex *Burkholderia sp.* MSMB1826
Bsp-6-1	Bacteria Proteobacteria Betaproteobacteria Burkholderiales Burkholderiaceae Burkholderia Burkholderia cepacia complex *Burkholderia sp.* MSMB1835
Bsp-7-1	Bacteria Proteobacteria Betaproteobacteria Burkholderiales Burkholderiaceae Burkholderia Burkholderia cepacia complex *Burkholderia sp.* MSMB2157WGS
Bsp-8-1	Bacteria Proteobacteria Betaproteobacteria Burkholderiales Burkholderiaceae Burkholderia Burkholderia cepacia complex *Burkholderia sp.* NRF60-BP8
Bst-1-1	Bacteria Proteobacteria Betaproteobacteria Burkholderiales Burkholderiaceae Burkholderia Burkholderia cepacia complex *Burkholderia stabilis*
Bte-1-1	Bacteria Proteobacteria Betaproteobacteria Burkholderiales Burkholderiaceae Burkholderia Burkholderia cepacia complex *Burkholderia territorii*
Bub-1-1	Bacteria Proteobacteria Betaproteobacteria Burkholderiales Burkholderiaceae Burkholderia Burkholderia cepacia complex *Burkholderia ubonensis*
Bvi-1-1 to Bvi-1-3	Bacteria Proteobacteria Betaproteobacteria Burkholderiales Burkholderiaceae Burkholderia Burkholderia cepacia complex *Burkholderia vietnamiensis*
Bvi-2-1	Bacteria Proteobacteria Betaproteobacteria Burkholderiales Burkholderiaceae Burkholderia Burkholderia cepacia complex *Burkholderia vietnamiensis* AU4i
Bvi-3-1	Bacteria Proteobacteria Betaproteobacteria Burkholderiales Burkholderiaceae Burkholderia Burkholderia cepacia complex *Burkholderia vietnamiensis* LMG 10929
Bsp-9-1	Bacteria Proteobacteria Betaproteobacteria Burkholderiales Burkholderiaceae *Burkholderia sp.* AU17325
Bsp-10-1	Bacteria Proteobacteria Betaproteobacteria Burkholderiales Burkholderiaceae *Burkholderia sp.* AU27893
Bsp-11-1	Bacteria Proteobacteria Betaproteobacteria Burkholderiales Burkholderiaceae *Burkholderia sp.* AU33647
Bsp-12-1	Bacteria Proteobacteria Betaproteobacteria Burkholderiales Burkholderiaceae *Burkholderia sp.* AU33803
Bsp-13-1	Bacteria Proteobacteria Betaproteobacteria Burkholderiales Burkholderiaceae *Burkholderia sp.* E168m22
Bsp-14-1	Bacteria Proteobacteria Betaproteobacteria Burkholderiales Burkholderiaceae *Burkholderia sp.* HI4860
Bsp-15-1	Bacteria Proteobacteria Betaproteobacteria Burkholderiales Burkholderiaceae *Burkholderia sp.* IDO3
Bsp-16-1	Bacteria Proteobacteria Betaproteobacteria Burkholderiales Burkholderiaceae *Burkholderia sp.* JKS000303
Bsp-17-1	Bacteria Proteobacteria Betaproteobacteria Burkholderiales Burkholderiaceae *Burkholderia sp.* K24
Bsp-18-1	Bacteria Proteobacteria Betaproteobacteria Burkholderiales Burkholderiaceae *Burkholderia sp.* KJ006
Bsp-19-1	Bacteria Proteobacteria Betaproteobacteria Burkholderiales Burkholderiaceae *Burkholderia sp.* LK4
Bsp-20-1	Bacteria Proteobacteria Betaproteobacteria Burkholderiales Burkholderiaceae *Burkholderia sp.* MSh2
Bsp-21-1	Bacteria Proteobacteria Betaproteobacteria Burkholderiales Burkholderiaceae *Burkholderia sp.* NFACC38-1
Bsp-22-1	Bacteria Proteobacteria Betaproteobacteria Burkholderiales Burkholderiaceae *Burkholderia sp.* OK233
Bsp-23-1	Bacteria Proteobacteria Betaproteobacteria Burkholderiales Burkholderiaceae *Burkholderia sp.* WP40
Bsp-24-1	Bacteria Proteobacteria Betaproteobacteria Burkholderiales Burkholderiaceae *Burkholderia sp.* WP42
Cmi-1-1	Bacteria Proteobacteria Betaproteobacteria Burkholderiales Burkholderiaceae *Caballeronia mineralivorans* PML1(12)
Pfe-1-1	Bacteria Proteobacteria Betaproteobacteria Burkholderiales Burkholderiaceae *Paraburkholderia ferrariae* NBRC 106233
Pfu-1-1	Bacteria Proteobacteria Betaproteobacteria Burkholderiales Burkholderiaceae *Paraburkholderia fungorum*
Pfu-2-1	Bacteria Proteobacteria Betaproteobacteria Burkholderiales Burkholderiaceae *Paraburkholderia fungorum* NBRC 102489
Pme-1-1	Bacteria Proteobacteria Betaproteobacteria Burkholderiales Burkholderiaceae *Paraburkholderia megapolitana*
Pph-1-1	Bacteria Proteobacteria Betaproteobacteria Burkholderiales Burkholderiaceae *Paraburkholderia phenoliruptrix* AC1100
Ptr-1-1 to Ptr-1-3	Bacteria Proteobacteria Betaproteobacteria Burkholderiales Burkholderiaceae *Paraburkholderia tropica*
Ara-1-1	Bacteria Proteobacteria Betaproteobacteria Burkholderiales Comamonadaceae *Acidovorax radicis* N35
Ara-2-1	Bacteria Proteobacteria Betaproteobacteria Burkholderiales Comamonadaceae *Acidovorax radicis* N35v
Asp-1-1	Bacteria Proteobacteria Betaproteobacteria Burkholderiales Comamonadaceae *Acidovorax sp.* 30
Asp-2-1	Bacteria Proteobacteria Betaproteobacteria Burkholderiales Comamonadaceae *Acidovorax sp.* 59
Asp-3-1	Bacteria Proteobacteria Betaproteobacteria Burkholderiales Comamonadaceae *Acidovorax sp.* Root217
Asp-4-1	Bacteria Proteobacteria Betaproteobacteria Burkholderiales Comamonadaceae *Acidovorax sp.* Root219
Asp-5-1	Bacteria Proteobacteria Betaproteobacteria Burkholderiales Comamonadaceae *Acidovorax sp.* Root267
Asp-6-1	Bacteria Proteobacteria Betaproteobacteria Burkholderiales Comamonadaceae *Acidovorax sp.* Root275
Asp-7-1	Bacteria Proteobacteria Betaproteobacteria Burkholderiales Comamonadaceae *Acidovorax sp.* Root402
Asp-8-1	Bacteria Proteobacteria Betaproteobacteria Burkholderiales Comamonadaceae *Acidovorax sp.* Root568
Asp-9-1	Bacteria Proteobacteria Betaproteobacteria Burkholderiales Comamonadaceae *Acidovorax sp.* SD340
Awa-1-1	Bacteria Proteobacteria Betaproteobacteria Burkholderiales Comamonadaceae *Acidovorax wautersii*
Hfl-1-1	Bacteria Proteobacteria Betaproteobacteria Burkholderiales Comamonadaceae *Hydrogenophaga flava* NBRC 102514
Hsp-1-1	Bacteria Proteobacteria Betaproteobacteria Burkholderiales Comamonadaceae *Hydrogenophaga sp.* A37

Hta-1-1	Bacteria Proteobacteria Betaproteobacteria Burkholderiales Comamonadaceae *Hydrogenophaga taeniospiralis* NBRC 102512
Psp-1-1	Bacteria Proteobacteria Betaproteobacteria Burkholderiales Comamonadaceae *Polaromonas sp.* A23
Vpa-1-1	Bacteria Proteobacteria Betaproteobacteria Burkholderiales Comamonadaceae *Variovorax paradoxus* EPS
Vsp-1-1	Bacteria Proteobacteria Betaproteobacteria Burkholderiales Comamonadaceae *Variovorax sp.* NFACC26
Vsp-2-1	Bacteria Proteobacteria Betaproteobacteria Burkholderiales Comamonadaceae *Variovorax sp.* NFACC27
Vsp-3-1	Bacteria Proteobacteria Betaproteobacteria Burkholderiales Comamonadaceae *Variovorax sp.* NFACC28
Vsp-4-1	Bacteria Proteobacteria Betaproteobacteria Burkholderiales Comamonadaceae *Variovorax sp.* NFACC29
Vsp-5-1	Bacteria Proteobacteria Betaproteobacteria Burkholderiales Comamonadaceae *Variovorax sp.* YR750
Vap-1-1	Bacteria Proteobacteria Betaproteobacteria Burkholderiales Comamonadaceae *Verminephrobacter aporrectodeae subsp. tuberculatae* At4
Hsp-2-1	Bacteria Proteobacteria Betaproteobacteria Burkholderiales Oxalobacteraceae *Herbaspirillum sp.* B39
Hsp-3-1	Bacteria Proteobacteria Betaproteobacteria Burkholderiales Oxalobacteraceae *Herbaspirillum sp.* RV1423
Rgu-1-1 to Rgu-1-2	Bacteria Proteobacteria Betaproteobacteria Burkholderiales *Rhizobacter gummiphilus*
Rsp-1-1	Bacteria Proteobacteria Betaproteobacteria Burkholderiales *Rhizobacter sp.* OV335
Rsp-2-1	Bacteria Proteobacteria Betaproteobacteria Burkholderiales *Rhizobacter sp.* Root1221
Ama-1-1 to Ama-1-3	Bacteria Proteobacteria Betaproteobacteria Neisseriales Chromobacteriaceae *Aquitalea magnusonii*
Aur-1-1	Bacteria Proteobacteria Betaproteobacteria Neisseriales Neisseriaceae *Amantichitinum ursilacus*
Pac-1-1	Bacteria Proteobacteria Gammaproteobacteria Pseudomonadales Pseudomonadaceae *Pseudomonas acidophila*
env-1 to env-136	environmental samples

3.2 Gene contexts

Each GGAM-3 RNA (indicated by "RNA→") is listed. For each hit, the downstream genes predicted to reside in a regulated operon are listed. If the nearest downstream gene is encoding in the opposite strand (and therefore presumed to not be a part of a regulated operon), then that gene is still depicted. Some environmental sequences and some RefSeq entries lack gene annotations, and so no genes are available for such sequences. The direction of each gene is indicated with an arrow (→), and each predicted conserved domain in the gene is named. Conserved domains associated with more than one GGAM-3 RNA are assigned a color; other domains are gray. Information about these conserved domains is given in Section 3.3. The accession of the sequence containing each GGAM-3 RNA is given in the column named "Seq. accession". Accessions beginning with "NC_", "NS_", "NW_" or "NZ_" are contained in RefSeq. Other accession refer to environmental samples. Nucleotide coordinates are given for the 5′ and 3′ boundaries of each GGAM-3 RNA. If the 5′ coordinate is greater than the 3′ coordinate, the RNA is present on the reverse-complement strand of the containing genomic DNA sequence. Each hit is denoted by an abbreviation (like "Eco-1-1") that refers to a taxonomy given in Section 3.1.

**abbrev.**	**Seq. accession**		**5′ at**	**3′ at**	**genes**
env-1	Ga0208125_100080	+	52643	53075	**RNA**→ emrE (PRK09541)EmrE (COG2076)→ hypo→ Acetyltransf_1 (pfam00583)Acetyltransf_7 (pfam13508)→
Ama-1-1	NZ_JZRC01000055.1	-	16093	15665	**RNA**→ emrE (PRK09541)EmrE (COG2076)→
Ama-1-2	NZ_BDST01000033.1	+	7366	7794	**RNA**→ emrE (PRK09541)EmrE (COG2076)→
env-2	Ga0105240_10261325	+	1	389	**RNA**→ emrE (PRK09541)EmrE (COG2076)→
Bsp-17-1	NZ_JMIK01000009.1	+	1729101	1729533	**RNA**→ emrE (PRK09541)EmrE (COG2076)→
env-3	QFRC01000102.1	+	4691	5123	**RNA**→ emrE (PRK09541)EmrE (COG2076)→
Pfu-2-1	NZ_BAYC01000032.1	+	168431	168863	**RNA**→ emrE (PRK09541)EmrE (COG2076)→
Pfu-1-1	NZ_NBSN01000003.1	-	2005208	2004776	**RNA**→ emrE (PRK09541)EmrE (COG2076)→
env-4	JCVI_SCAF_1096627189088	-	31619	31187	**RNA**→ emrE (PRK09541)EmrE (COG2076)→ RhaT (COG0697)2A78 (TIGR00950)→ hypo→
Pph-1-1	NZ_ASXI01000204.1	-	14446	14014	**RNA**→ emrE (PRK09541)EmrE (COG2076)→
env-5	Ga0255336_100070	+	127275	127707	**RNA**→ emrE (PRK09541)EmrE (COG2076)→
Bce-1-1	NZ_CP017239.1	-	3045290	3044851	**RNA**→ emrE (PRK09541)EmrE (COG2076)→
Rgu-1-1	NZ_CP015118.1	-	4117736	4117302	**RNA**→ emrE (PRK09541)EmrE (COG2076)→
Rgu-1-2	NZ_CP024645.1	-	4117739	4117305	**RNA**→ emrE (PRK09541)EmrE (COG2076)→
Hta-1-1	NZ_BCWR01000011.1	-	202778	202345	**RNA**→ emrE (PRK09541)EmrE (COG2076)→
Vpa-1-1	NC_014931.1	+	149911	150342	**RNA**→ emrE (PRK09541)EmrE (COG2076)→
					HTH_XRE (smart00530)HTH_XRE (cd00093)NBR1_like (cd14947)N_BRCA1_IG (pfam16158)→
Aur-1-1	NZ_LAQT01000005.1	-	102906	102470	**RNA**→ emrE (PRK09541)EmrE (COG2076)→
env-6	Ga0209066_10006966	-	5852	5414	**RNA**→ emrE (PRK09541)EmrE (COG2076)→
					HTH_XRE (smart00530)HTH_XRE (cd00093)NBR1_like (cd14947)N_BRCA1_IG (pfam16158)→

Vap-1-1	NZ_AFAL01000365.1	-	19662	19232	**RNA**→ emrE (PRK09541)EmrE (COG2076)→ emrE (PRK09541)EmrE (COG2076)→
Rsp-2-1	NZ_LMDI01000038.1	-	31997	31566	**RNA**→ emrE (PRK09541)EmrE (COG2076)→
Bce-1-2	NZ_CP019668.1	+	1794172	1794608	**RNA**→ emrE (PRK09541)EmrE (COG2076)→
Bce-3-1	NC_008543.1	-	2781714	2781278	**RNA**→ emrE (PRK09541)EmrE (COG2076)→
Bce-2-1	NC_008061.1	+	2485276	2485712	**RNA**→ emrE (PRK09541)EmrE (COG2076)→
Bsp-13-1	NZ_BBSK01000114.1	+	67472	67908	**RNA**→ emrE (PRK09541)EmrE (COG2076)→
Hsp-3-1	NZ_CBXX010000006.1	-	9272	8841	**RNA**→ emrE (PRK09541)EmrE (COG2076)→
env-7	Ga0105237_10008943	-	4337	3906	**RNA**→ emrE (PRK09541)EmrE (COG2076)→
Hsp-2-1	NZ_BADF01000789.1	-	28130	27699	**RNA**→ emrE (PRK09541)EmrE (COG2076)→
env-8	TB_PC08_64_1011717	-	1713	1280	**RNA**→ emrE (PRK09541)EmrE (COG2076)→
					HTH_XRE (smart00530)HTH_XRE (cd00093)NBR1_like (cd14947)N_BRCA1_IG (pfam16158)→
					oxa_formateAnti (TIGR04259)→
env-9	Ga0122799_100180	+	3408	3841	**RNA**→ emrE (PRK09541)EmrE (COG2076)→ hypo→ EamA (pfam00892)→ hypo→ hypo→
Asp-4-1	NZ_LMIJ01000014.1	-	356696	356255	**RNA**→ emrE (PRK09541)EmrE (COG2076)→
Asp-3-1	NZ_LMIH01000016.1	-	269566	269125	**RNA**→ emrE (PRK09541)EmrE (COG2076)→
env-10	Ga0209066_10001878	-	18201	17763	**RNA**→ emrE (PRK09541)EmrE (COG2076)→
Awa-1-1	NZ_FONX01000002.1	-	11717	11283	**RNA**→ emrE (PRK09541)EmrE (COG2076)→
					HTH_XRE (smart00530)HTH_XRE (cd00093)NBR1_like (cd14947)N_BRCA1_IG (pfam16158)→
Ama-1-3	NZ_LNQU01000125.1	+	2761	3189	**RNA**→ emrE (PRK09541)EmrE (COG2076)→
env-11	MERS01000011.1	+	51422	51853	**RNA**→ emrE (PRK09541)EmrE (COG2076)→
env-12	MERT01000015.1	+	51431	51862	**RNA**→ emrE (PRK09541)EmrE (COG2076)→
env-13	DONA01000025.1	+	35917	36348	**RNA**→ emrE (PRK09541)EmrE (COG2076)→
env-14	DNES01000012.1	-	300368	299937	**RNA**→ emrE (PRK09541)EmrE (COG2076)→
env-15	C687J26621_10034556	-	678	247	**RNA**→ emrE (PRK09541)EmrE (COG2076)→ hypo→
env-16	MESC01000057.1	-	1164	733	**RNA**→ emrE (PRK09541)EmrE (COG2076)→
env-17	MESF01000116.1	+	1114	1545	**RNA**→ emrE (PRK09541)EmrE (COG2076)→
env-18	Ga0208375_1037249	-	423	1	**RNA**→
env-19	Ga0194138_10426977	-	310	1	**RNA**→
env-20	Ga0194136_1626569	+	47	356	**RNA**→
env-21	Ga0209066_10062993	-	2031	1722	**RNA**→ emrE (PRK09541)EmrE (COG2076)→
env-22	Ga0180017_110563	-	260	1	**RNA**→
Bsp-20-1	NZ_JPGM01000030.1	-	63655	63220	**RNA**→ emrE (PRK09541)EmrE (COG2076)→
Bpa-1-1	NZ_JPGL01000008.1	-	61258	60823	**RNA**→ emrE (PRK09541)EmrE (COG2076)→
Bsp-8-1	NZ_LOTR01000001.1	-	442170	441735	**RNA**→ emrE (PRK09541)EmrE (COG2076)→
Pac-1-1	NZ_MTZV01000006.1	+	2018981	2019416	**RNA**→ emrE (PRK09541)EmrE (COG2076)→
Pme-1-1	NZ_FOQU01000004.1	+	390292	390727	**RNA**→ emrE (PRK09541)EmrE (COG2076)→
env-23	Ga0164242_11025267	-	382	1	**RNA**→
Ptr-1-1	NZ_MSDZ01000049.1	+	332013	332445	**RNA**→ emrE (PRK09541)EmrE (COG2076)→
Ptr-1-2	NZ_FNZM01000007.1	-	51017	50585	**RNA**→ emrE (PRK09541)EmrE (COG2076)→
Ptr-1-3	NZ_LXGI01000091.1	+	94340	94772	**RNA**→ emrE (PRK09541)EmrE (COG2076)→
Bub-1-1	NZ_CP013422.1	+	412909	413344	**RNA**→ emrE (PRK09541)EmrE (COG2076)→
Bvi-1-1	NZ_CP013455.1	+	412867	413302	**RNA**→ emrE (PRK09541)EmrE (COG2076)→
Bvi-1-2	NZ_CP013440.1	-	1799903	1799468	**RNA**→ emrE (PRK09541)EmrE (COG2076)→
Bsp-24-1	NZ_KN050723.1	+	230837	231272	**RNA**→ emrE (PRK09541)EmrE (COG2076)→
Bsp-23-1	NZ_FPJR01000001.1	-	267747	267312	**RNA**→ emrE (PRK09541)EmrE (COG2076)→
Bvi-3-1	NZ_CP009630.1	-	1783221	1782786	**RNA**→ emrE (PRK09541)EmrE (COG2076)→
Bvi-1-3	NZ_CP013433.1	+	1181732	1182167	**RNA**→ emrE (PRK09541)EmrE (COG2076)→
Bsp-18-1	NC_017921.1	+	982856	983291	**RNA**→ emrE (PRK09541)EmrE (COG2076)→ EmrE (COG2076)→
env-24	3300000558_11511866	+	1	437	**RNA**→ emrE (PRK09541)EmrE (COG2076)→
Pfe-1-1	NZ_BAYB01000038.1	+	28079	28515	**RNA**→ emrE (PRK09541)EmrE (COG2076)→
env-25	PM50_paired_qual32_contig_734	+	92	528	**RNA**→ emrE (PRK09541)EmrE (COG2076)→ PRK11478 (PRK11478)VOC_like (cd07262)→
env-26	Ga0209171_10024293	-	579	143	**RNA**→ PRK11431 (PRK11431)EmrE (COG2076)→
Bsp-22-1	NZ_OCSR01000001.1	+	2895723	2896163	**RNA**→ emrE (PRK09541)EmrE (COG2076)→

env-27	Ga0233424_10690257	-	281	1	**RNA**→
env-28	Ga0255336_109457	-	961	530	**RNA**→ emrE (PRK09541)EmrE (COG2076)→
env-29	G312J29652_10003048	-	727	296	**RNA**→ emrE (PRK09541)EmrE (COG2076)→
env-30	Ga0122097_100303	+	9306	9738	**RNA**→ PRK11431 (PRK11431)EmrE (COG2076)→
env-31	Ga0208564_1311142	+	211	388	**RNA**→
env-32	Ga0118611_113532	-	302	1	**RNA**→
env-33	Ga0118618_116904	+	3	352	**RNA**→ emrE (PRK09541)EmrE (COG2076)→
env-34	Ga0118189_101006	-	2348	1999	**RNA**→ emrE (PRK09541)EmrE (COG2076)→ emrE (PRK09541)EmrE (COG2076)→
env-35	Ga0118139_108835	+	21	370	**RNA**→ emrE (PRK09541)EmrE (COG2076)→
env-36	Ga0118335_100481	-	460	111	**RNA**→ PRK11431 (PRK11431)EmrE (COG2076)→
env-37	Ga0118355_13487	+	65	356	**RNA**→
env-38	Ga0118176_17188	-	276	1	**RNA**→
env-39	Ga0118609_113962	-	341	66	**RNA**→ emrE (PRK09541)EmrE (COG2076)→
env-40	Ga0118210_122924	-	232	1	**RNA**→
env-41	Ga0118379_115709	+	325	497	**RNA**→
env-42	Ga0118151_127626	+	114	286	**RNA**→ hypo→
env-43	Ga0118330_128175	-	225	53	**RNA**→ hypo→
env-44	Ga0118325_108579	-	215	43	**RNA**→
env-45	Ga0208628_1064142	+	240	412	**RNA**→
env-46	Ga0123390_1024131	+	230	402	**RNA**→ EmrE (COG2076)emrE (PRK09541)→
env-47	Ga0118636_118037	+	90	439	**RNA**→
env-48	Ga0118157_109833	-	346	71	**RNA**→ emrE (PRK09541)EmrE (COG2076)→
env-49	Ga0118375_105827	-	195	23	**RNA**→
env-50	Ga0118333_131724	-	217	45	**RNA**→ hypo→
env-51	Ga0118628_128146	+	36	208	**RNA**→ emrE (PRK09541)EmrE (COG2076)→
env-52	Ga0123393_1059440	+	152	324	**RNA**→ hypo→
env-53	Ga0118195_100879	+	386	735	**RNA**→ emrE (PRK09541)EmrE (COG2076)→
env-54	Ga0118196_101715	-	1180	831	**RNA**→ PRK11431 (PRK11431)EmrE (COG2076)→
env-55	Ga0118433_101172	+	958	1307	**RNA**→ PRK11431 (PRK11431)EmrE (COG2076)→
env-56	Ga0118373_101061	-	884	535	**RNA**→ PRK11431 (PRK11431)EmrE (COG2076)→
env-57	Ga0118190_103314	-	1038	689	**RNA**→ PRK11431 (PRK11431)EmrE (COG2076)→
env-58	Ga0118177_102239	+	516	865	**RNA**→ emrE (PRK09541)EmrE (COG2076)→ emrE (PRK09541)EmrE (COG2076)→
env-59	Ga0118353_102590	-	1373	1024	**RNA**→ PRK11431 (PRK11431)EmrE (COG2076)→
env-60	Ga0118383_101611	-	1713	1364	**RNA**→ emrE (PRK09541)EmrE (COG2076)→ emrE (PRK09541)EmrE (COG2076)→
env-61	Ga0207968_148482	-	161	1	**RNA**→
env-62	Ga0208885_149125	-	305	1	**RNA**→
env-63	Ga0207972_122935	-	579	225	**RNA**→ emrE (PRK09541)EmrE (COG2076)→
env-64	Ga0208125_145048	-	392	1	**RNA**→
env-65	Ga0208756_109168	-	586	154	**RNA**→ emrE (PRK09541)EmrE (COG2076)→
env-66	Ga0208758_105057	-	954	522	**RNA**→ emrE (PRK09541)EmrE (COG2076)→
env-67	Ga0207976_100202	-	3671	3239	**RNA**→ emrE (PRK09541)EmrE (COG2076)→ emrE (PRK09541)EmrE (COG2076)→
env-68	Ga0208123_104801	+	568	1000	**RNA**→ emrE (PRK09541)EmrE (COG2076)→ emrE (PRK09541)EmrE (COG2076)→
env-69	Ga0207975_101158	+	5205	5637	**RNA**→ emrE (PRK09541)EmrE (COG2076)→ emrE (PRK09541)EmrE (COG2076)→
env-70	Ga0208116_1042359	-	565	261	**RNA**→ emrE (PRK09541)EmrE (COG2076)→
Asp-8-1	NZ_LMGP01000018.1	+	262793	263225	**RNA**→ emrE (PRK09541)EmrE (COG2076)→
env-71	Ga0123397_132189	+	1827	2186	**RNA**→ emrE (PRK09541)EmrE (COG2076)→
env-72	Ga0123389_156195	-	938	579	**RNA**→ emrE (PRK09541)EmrE (COG2076)→
env-73	Ga0123396_132928	-	525	166	**RNA**→ emrE (PRK09541)EmrE (COG2076)→
env-74	Ga0208636_100239	-	1953	1521	**RNA**→ emrE (PRK09541)EmrE (COG2076)→ emrE (PRK09541)EmrE (COG2076)→
env-75	Ga0208630_117495	+	235	667	**RNA**→ emrE (PRK09541)EmrE (COG2076)→
Asp-2-1	NZ_PEET01000001.1	+	629716	630148	**RNA**→ emrE (PRK09541)EmrE (COG2076)→
Asp-1-1	NZ_PJMZ01000001.1	-	4324117	4323685	**RNA**→ emrE (PRK09541)EmrE (COG2076)→

Asp-7-1	NZ_LMDQ01000013.1	+	277382	277814	**RNA**→ emrE (PRK09541)EmrE (COG2076)→
env-76	Ga0208626_1029986	-	471	39	**RNA**→ emrE (PRK09541)EmrE (COG2076)→
env-77	Ga0118386_101399	+	666	1045	**RNA**→ emrE (PRK09541)EmrE (COG2076)→
env-78	Ga0118438_115386	+	1	380	**RNA**→ emrE (PRK09541)EmrE (COG2076)→
env-79	Ga0118349_117222	-	366	1	**RNA**→
env-80	Ga0118209_126459	+	1	342	**RNA**→
env-81	Ga0118018_110489	+	1	359	**RNA**→ emrE (PRK09541)EmrE (COG2076)→
env-82	Ga0118193_106485	-	652	273	**RNA**→ emrE (PRK09541)EmrE (COG2076)→
env-83	Ga0160495_104200	-	1094	662	**RNA**→ emrE (PRK09541)EmrE (COG2076)→
env-84	Ga0180009_10219546	+	566	842	**RNA**→
env-85	DFTY01000080.1	-	13783	13348	**RNA**→ emrE (PRK09541)EmrE (COG2076)→ HTH_XRE (cd00093)NBR1_like (cd14947)HTH_26 (pfam13443)N_BRCA1_IG (pfam16158)→
env-86	DDJC01000100.1	+	11510	11945	**RNA**→ emrE (PRK09541)EmrE (COG2076)→ HTH_XRE (cd00093)NBR1_like (cd14947)HTH_26 (pfam13443)N_BRCA1_IG (pfam16158)→
Vsp-4-1	NZ_FNVM01000008.1	+	202004	202438	**RNA**→ emrE (PRK09541)EmrE (COG2076)→
Vsp-3-1	NZ_FNUI01000012.1	+	126861	127295	**RNA**→ emrE (PRK09541)EmrE (COG2076)→
Vsp-2-1	NZ_FOOV01000005.1	+	202251	202685	**RNA**→ emrE (PRK09541)EmrE (COG2076)→
Vsp-1-1	NZ_FOKS01000008.1	-	190599	190165	**RNA**→ emrE (PRK09541)EmrE (COG2076)→
Vsp-5-1	NZ_FOBJ01000009.1	-	192511	192076	**RNA**→ emrE (PRK09541)EmrE (COG2076)→
env-87	JGI25151J46595_10269744	-	351	1	**RNA**→
Cmi-1-1	NZ_AEJF01000108.1	+	99877	100308	**RNA**→ emrE (PRK09541)EmrE (COG2076)→
[me-1-1	NZ_CP020737.1	-	988178	987747	**RNA**→ PRK11431 (PRK11431)EmrE (COG2076)→
Bce-1-3	NZ_CP007783.1	-	825679	825247	**RNA**→ PRK11431 (PRK11431)EmrE (COG2076)→ hypo→
Bps-1-1	NZ_CP013378.1	-	2143843	2143412	**RNA**→ PRK11431 (PRK11431)EmrE (COG2076)→ hypo→
Bsp-5-1	NZ_LOYN01000022.1	+	427193	427628	**RNA**→ emrE (PRK09541)EmrE (COG2076)→
Bsp-7-1	NZ_LPJG01000011.1	+	437156	437591	**RNA**→ emrE (PRK09541)EmrE (COG2076)→
Bsp-6-1	NZ_LOYP01000054.1	+	400547	400982	**RNA**→ emrE (PRK09541)EmrE (COG2076)→
Bsp-3-1	NZ_LPDI01000040.1	-	87923	87488	**RNA**→ emrE (PRK09541)EmrE (COG2076)→
Bsp-2-1	NZ_LOYC01000030.1	+	269563	269998	**RNA**→ emrE (PRK09541)EmrE (COG2076)→
Bsp-4-1	NZ_LPMU01000064.1	-	18495	18060	**RNA**→ emrE (PRK09541)EmrE (COG2076)→
Bsp-16-1	NZ_PDBZ01000001.1	+	626660	627095	**RNA**→ emrE (PRK09541)EmrE (COG2076)→
Bsp-15-1	NZ_NWSG01000010.1	-	89538	89102	**RNA**→ emrE (PRK09541)EmrE (COG2076)→
Bpu-1-1	NZ_NBYX01000006.1	-	133889	133453	**RNA**→ PRK11431 (PRK11431)EmrE (COG2076)→
Bst-1-1	NZ_CP016442.1	+	1611134	1611570	**RNA**→ PRK11431 (PRK11431)EmrE (COG2076)→
Bsp-9-1	NZ_NKFA01000003.1	-	453513	453077	**RNA**→ emrE (PRK09541)EmrE (COG2076)→
Bvi-2-1	NZ_ASSI01000024.1	-	15779	15343	**RNA**→ PRK11431 (PRK11431)EmrE (COG2076)→
Bla-1-1	NZ_LDWP01000037.1	+	52845	53281	**RNA**→ PRK11431 (PRK11431)EmrE (COG2076)→
Bla-1-2	NZ_LDWF01000088.1	-	28625	28189	**RNA**→ PRK11431 (PRK11431)EmrE (COG2076)→
Bsp-19-1	NZ_LDUL01000038.1	-	94676	94240	**RNA**→ PRK11431 (PRK11431)EmrE (COG2076)→
Bce-4-1	NZ_CP013375.1	-	1002603	1002167	**RNA**→ PRK11431 (PRK11431)EmrE (COG2076)→
Bsp-21-1	NZ_FOIG01000014.1	-	75133	74697	**RNA**→ PRK11431 (PRK11431)EmrE (COG2076)→
Bsp-1-1	NC_007510.1	-	962092	961656	**RNA**→ PRK11431 (PRK11431)EmrE (COG2076)→
Bsp-14-1	NZ_NKFO01000004.1	-	428569	428133	**RNA**→ PRK11431 (PRK11431)EmrE (COG2076)→
Bsp-11-1	NZ_NKFI01000003.1	-	428563	428127	**RNA**→ PRK11431 (PRK11431)EmrE (COG2076)→
Bsp-12-1	NZ_NKFJ01000004.1	-	447221	446785	**RNA**→ PRK11431 (PRK11431)EmrE (COG2076)→
Bsp-10-1	NZ_NKFD01000001.1	-	1013761	1013325	**RNA**→ PRK11431 (PRK11431)EmrE (COG2076)→
Bse-1-1	NZ_CP013398.1	-	949632	949196	**RNA**→ emrE (PRK09541)EmrE (COG2076)→
Bce-6-1	NZ_CP013730.1	+	3217481	3217917	**RNA**→ PRK11431 (PRK11431)EmrE (COG2076)→
env-88	Ga0208758_100009	+	98756	99191	**RNA**→ emrE (PRK09541)EmrE (COG2076)→ HmoA (COG2329)→ Acetyltransf_1 (pfam00583)Acetyltransf_7 (pfam13508)→
env-89	Ga0208125_100053	+	51410	51845	**RNA**→ emrE (PRK09541)EmrE (COG2076)→ HmoA (COG2329)→ Acetyltransf_1 (pfam00583)Acetyltransf_7 (pfam13508)→
env-90	Ga0105237_11394294	+	1	435	**RNA**→ emrE (PRK09541)EmrE (COG2076)→ hypo→

Rsp-1-1	NZ_FRCQ01000010.1	+	161720	162155	**RNA**→ emrE (PRK09541)EmrE (COG2076)→ DUF4124 (pfam13511)→
env-91	Ga0196963_10000325	+	25155	25590	**RNA**→ emrE (PRK09541)EmrE (COG2076)→ nicotinamidase_related (cd01014)PTZ00331 (PTZ00331)→
env-92	Ga0196973_1002167	-	1091	656	**RNA**→ emrE (PRK09541)EmrE (COG2076)→
env-93	Ga0209097_10295377	-	256	1	**RNA**→
env-94	Ga0208884_120217	+	421	736	**RNA**→
env-95	Ga0208398_100006	-	464348	464033	**RNA**→ emrE (PRK09541)EmrE (COG2076)→ TlpA_like_family (cd02966)Thioredoxin_8 (pfam13905)→
env-96	Ga0170573_11315038	-	732	296	**RNA**→ emrE (PRK09541)EmrE (COG2076)→
env-97	MERV01000031.1	+	181534	181970	**RNA**→ emrE (PRK09541)EmrE (COG2076)→
env-98	Ga0209542_10000009	+	181562	181998	**RNA**→ emrE (PRK09541)EmrE (COG2076)→
env-99	MERR01000006.1	+	267343	267779	**RNA**→ emrE (PRK09541)EmrE (COG2076)→
env-100	DPDC01000024.1	+	267678	268114	**RNA**→ emrE (PRK09541)EmrE (COG2076)→
env-101	PBR_1098343	+	1	254	**RNA**→
Asp-9-1	NZ_LHUP01000121.1	-	27097	26661	**RNA**→ emrE (PRK09541)EmrE (COG2076)→
env-102	Ga0208403_105257	-	388	1	**RNA**→
env-103	Ga0208398_102894	+	3884	4271	**RNA**→ emrE (PRK09541)EmrE (COG2076)→
env-104	Ga0160476_101499	-	1026	590	**RNA**→ emrE (PRK09541)EmrE (COG2076)→
env-105	Ga0160492_101358	+	7749	8185	**RNA**→ emrE (PRK09541)EmrE (COG2076)→
env-106	Ga0209066_10093987	+	354	563	**RNA**→ emrE (PRK09541)EmrE (COG2076)→
env-107	MESB01000423.1	+	7156	7592	**RNA**→ emrE (PRK09541)EmrE (COG2076)→
env-108	MESJ01000046.1	+	211200	211636	**RNA**→ emrE (PRK09541)EmrE (COG2076)→
env-109	MERW01000015.1	+	9288	9724	**RNA**→ emrE (PRK09541)EmrE (COG2076)→
env-110	Ga0208758_118006	-	210	1	**RNA**→
env-111	Ga0208125_140138	-	285	1	**RNA**→
env-112	JGI26524J50256_1000545	+	18702	19138	**RNA**→ emrE (PRK09541)EmrE (COG2076)→
env-113	JGI26523J50269_1016483	-	452	16	**RNA**→ emrE (PRK09541)EmrE (COG2076)→
Asp-5-1	NZ_LMJC01000012.1	+	278134	278570	**RNA**→ emrE (PRK09541)EmrE (COG2076)→
Ara-1-1	NZ_AFBG01000021.1	-	596152	595716	**RNA**→ emrE (PRK09541)EmrE (COG2076)→ emrE (PRK09541)EmrE (COG2076)→ emrE (PRK09541)EmrE (COG2076)→
Ara-2-1	NZ_AFBH01000016.1	+	93572	94008	**RNA**→ emrE (PRK09541)EmrE (COG2076)→
Asp-6-1	NZ_LMJH01000009.1	-	593584	593148	**RNA**→ emrE (PRK09541)EmrE (COG2076)→
env-114	Ga0208392_1131550	+	209	362	**RNA**→
env-115	Ga0187844_10008638	-	1199	763	**RNA**→ emrE (PRK09541)EmrE (COG2076)→ hypo→
env-116	Ga0187843_10006647	-	1030	594	**RNA**→ emrE (PRK09541)EmrE (COG2076)→ hypo→
env-117	Ga0187845_1013956	+	2824	3189	**RNA**→
env-118	QZKF01000013.1	-	20671	20235	**RNA**→ emrE (PRK09541)EmrE (COG2076)→
env-119	Ga0104756_1001742	+	7271	7707	**RNA**→ emrE (PRK09541)EmrE (COG2076)→
env-120	Ga0210245_100003	+	27430	27866	**RNA**→ emrE (PRK09541)EmrE (COG2076)→
env-121	Ga0210247_100115	-	48780	48344	**RNA**→ emrE (PRK09541)EmrE (COG2076)→
env-122	Ga0210246_100016	+	16990	17426	**RNA**→ emrE (PRK09541)EmrE (COG2076)→
env-123	Ga0210244_100953	-	10964	10528	**RNA**→ emrE (PRK09541)EmrE (COG2076)→
env-124	Ga0210248_100112	+	14629	15065	**RNA**→ emrE (PRK09541)EmrE (COG2076)→
env-125	Ga0247836_1446970	+	1	393	**RNA**→ emrE (PRK09541)EmrE (COG2076)→
Psp-1-1	NZ_MUNO01000012.1	-	57573	57137	**RNA**→ emrE (PRK09541)EmrE (COG2076)→
env-126	Ga0134127_14699610	-	240	1	**RNA**→
env-127	TB_PC08_64_1051016	-	329	1	**RNA**→
env-128	Ga0134125_14367156	-	194	1	**RNA**→
env-129	Ga0105239_15349733	-	352	1	**RNA**→
Hfl-1-1	NZ_BCTF01000022.1	-	26180	25743	**RNA**→ emrE (PRK09541)EmrE (COG2076)→ nitrilase (cd07197)PRK13981 (PRK13981)→
Hsp-1-1	NZ_MUNZ01000106.1	-	72062	71627	**RNA**→ emrE (PRK09541)EmrE (COG2076)→ DUF4256 (pfam14066)→
env-130	Ga0190274_10003787	+	7143	7579	**RNA**→ PRK11431 (PRK11431)EmrE (COG2076)→ LivK (COG0683)Peripla_BP_6 (pfam13458)→ hypo→
env-131	Ga0208940_1049431	-	885	450	**RNA**→ emrE (PRK09541)EmrE (COG2076)→
env-132	Ga0208197_1059241	-	930	495	**RNA**→ emrE (PRK09541)EmrE (COG2076)→

env-133	Ga0208883_103401	-	482	206	**RNA**→ emrE (PRK09541)EmrE (COG2076)→
env-134	Ga0208265_137337	+	63	339	**RNA**→
env-135	Ga0208754_100081	-	27784	27508	**RNA**→ emrE (PRK09541)EmrE (COG2076)→ HmoA (COG2329)→ Acetyltransf_1 (pfam00583)Acetyltransf_7 (pfam13508)→
env-136	Ga0208125_104047	-	813	379	**RNA**→ emrE (PRK09541)EmrE (COG2076)→ HmoA (COG2329)→
Bte-1-1	NZ_CP013365.1	-	434573	434144	**RNA**→ PRK11431 (PRK11431)EmrE (COG2076)→ HTH_XRE (smart00530)NBR1_like (cd14947)HTH_3 (pfam01381)N_BRCA1_IG (pfam16158)→
Bce-5-1	NZ_KI421535.1	+	2065048	2065484	**RNA**→ PRK11431 (PRK11431)EmrE (COG2076)→
Bce-4-2	NZ_CP022083.1	+	319544	319980	**RNA**→ PRK11431 (PRK11431)EmrE (COG2076)→
Bce-4-3	NZ_CP023518.1	-	2693239	2692803	**RNA**→ PRK11431 (PRK11431)EmrE (COG2076)→

3.3 Conserved domains

Conserved domains found in protein-coding genes listed in Section 3.2 are shown below, with the first sentence in their description from the Conserved Domain Database (if any). Conserved domains associated with more than one GGAM-3 RNA are assigned a color, while others are shown in gray. The number in parentheses after the colored domain name is the number of occurrences in Section 3.2.

cd00093 (6) Helix-turn-helix XRE-family like proteins.
cd01014 (1) Nicotinamidase_ related amidohydrolases.
cd02966 (1) TlpA-like family; composed of TlpA, ResA, DsbE and similar proteins.
cd07197 (1) Nitrilase superfamily, including nitrile- or amide-hydrolyzing enzymes and amide-condensing enzymes.
cd07262 (1) uncharacterized subfamily of vicinal oxygen chelate (VOC) family.
cd14947 (7) Functionally uncharacterized domain in neighbor of Brca1 Gene 1 and related proteins.
COG0683 (1) ABC-type branched-chain amino acid transport system, periplasmic component [Amino acid transport and metabolism]
COG0697 (1) Permease of the drug/metabolite transporter (DMT) superfamily [Carbohydrate transport and metabolism, Amino acid transport and metabolism, General function prediction only]
COG2076 (199) Multidrug transporter EmrE and related cation transporters [Defense mechanisms]
COG2329 (4) Heme-degrading monooxygenase HmoA and related ABM domain proteins [Coenzyme transport and metabolism]
pfam00583 (4) Acetyltransferase (GNAT) family.
pfam00892 (1) EamA-like transporter family.
pfam01381 (1) Helix-turn-helix.
pfam13443 (2) Cro/C1-type HTH DNA-binding domain.
pfam13458 (1) Periplasmic binding protein.
pfam13508 (4) Acetyltransferase (GNAT) domain.
pfam13511 (1) Domain of unknown function (DUF4124).
pfam13905 (1) Thioredoxin-like.
pfam14066 (1) Protein of unknown function (DUF4256).
pfam16158 (7) Ig-like domain from next to BRCA1 gene.
PRK09541 (168) multidrug efflux protein; Reviewed
PRK11431 (30) multidrug efflux system protein; Provisional
PRK11478 (1) putative lyase; Provisional
PRK13981 (1) NAD synthetase; Provisional
PTZ00331 (1) alpha/beta hydrolase; Provisional
smart00530 (5) Helix-turn-helix XRE-family like proteins.
TIGR00950 (1) Carboxylate/Amino Acid/Amine Transporter. [Transport and binding proteins, Amino acids, peptides and amines]
TIGR04259 (1) oxalate/formate antiporter.

3.4 Multiple-sequence alignment

Each GGAM-3 RNA is denoted by an abbreviation (like "Eco-1-1") that refers to a taxonomy given in Section 3.1. The alignment may include sequences containing the RNA motif, as well as flanking sequence. The GGAM-3 RNA itself is denoted by the line underneath marked 5′ and 3′ on either end. Nucleotides in flanking sequences (i.e., not part of the motif) are written in gray letters. Stems of predicted rho-independent transcription terminators, if any, are shaded yellow. (Note: terminator predictions have not been analyzed manually, and many are likely to be false positives. Terminator predictions are those of the RNIE software.) Nucleotides predicted to function (as DNA) as transcription-factor binding sites, if any, are shaded in green. (But note: these predictions are manually annotated, so they might be under-predicted.) Annotated start codons, if any, are shaded green. (Note: start codons are frequently misannotated, especially in environmental samples.) Nucleotides proposed to basepair as part of the consensus structure are shaded in color when they comprise Watson-Crick or G-U pairs. Oth-

erwise they are shaded gray. Conserved stems are also indicated at the bottom of the alignment by angle brackets, where matching < and > denote base-paired columns. Below these angle brackets, the symbol "2" denotes base pairs exhibiting covariation according to the statistically well-founded R-scape method. "1" denotes base pairs exhibiting covariation according to R2R's simplistic method. "0" denotes base pairs that are not observed to mutate and "?" denotes base pairs that have a significant frequency of non-canonical nucleotides for Watson-Crick or G-U pairs (> 5%). Below these base pair annotation is the consensus sequence: "R" = "A" or "G", "Y" = "C" or "U", red nucleotides: nucleotide identity conserved more than 97% of the time, black nucleotides: 90%, gray nucleotides: 75%, red circle (•): nucleotide is present 97% of the time, black circle (•): 90%, gray circle (•): 75%, white circle (◦): 50%. All percentages of sequences just described (e.g. 97% conserved) assume that sequences have been weighted by the GSC algorithm implemented by the Infernal software package.

Duplicate sequences: the following putative homologs are not shown in the alignment because their sequences are identical to a homolog already shown: Ara-2-1, Asp-1-1, Bce-2-1, Bce-3-1, Bce-4-2, Bce-4-3, Bla-1-2, Bsp-10-1, Bsp-11-1, Bsp-13-1, Bsp-19-1, Bsp-23-1, Bvi-1-1, Bvi-1-2, Pfu-1-1, Ptr-1-2, Rgu-1-2, Vsp-1-1, Vsp-2-1, Vsp-3-1, env-100, env-105, env-108, env-109, env-116, env-119, env-12, env-121, env-122, env-123, env-124, env-13, env-14, env-17, env-4, env-43, env-5, env-54, env-55, env-56, env-57, env-58, env-59, env-60, env-67, env-68, env-69, env-86, env-89, env-92, env-98

alignment positions 1 · · · 180

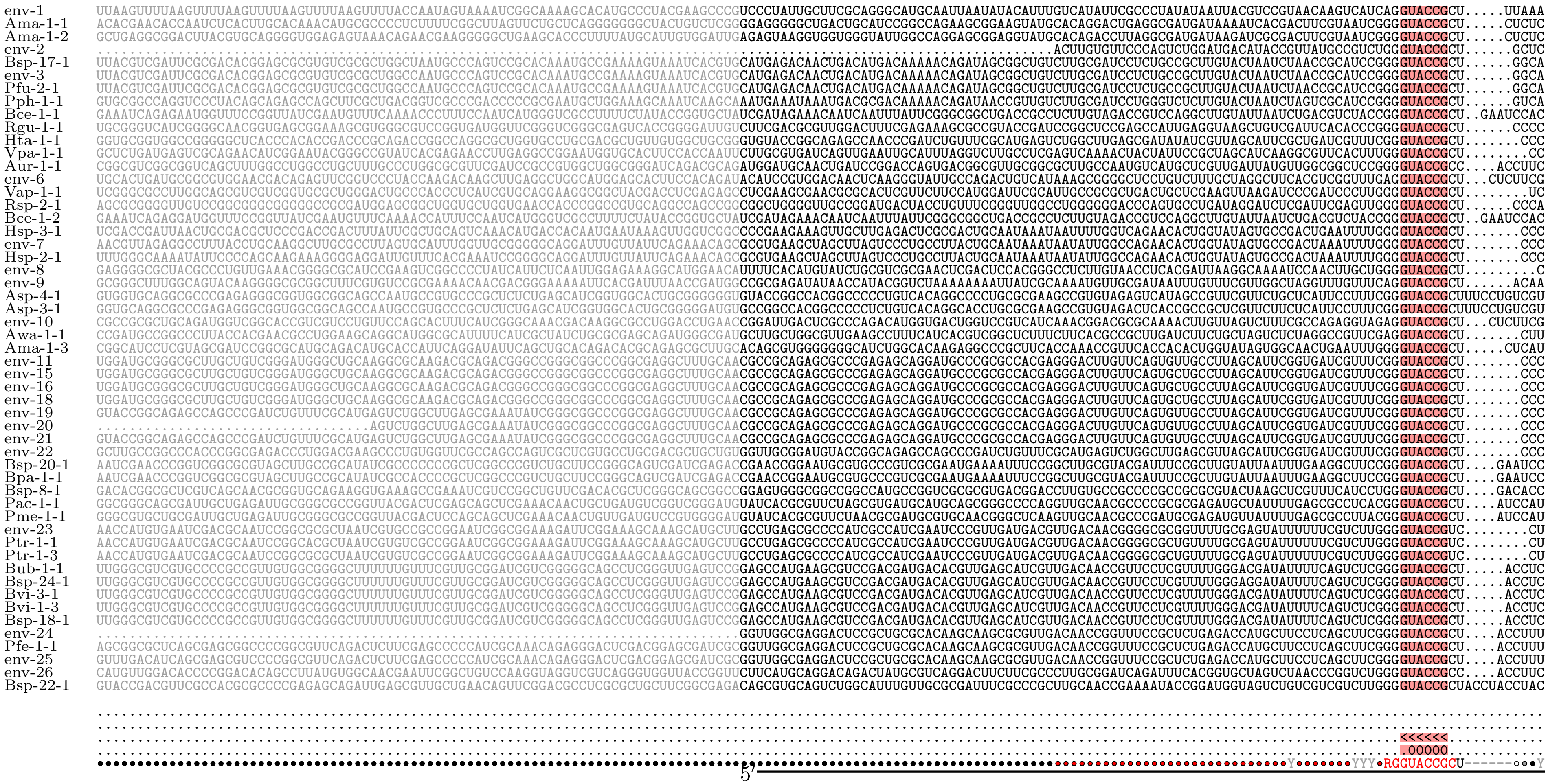

```
env-27    GCGGCGUGCCUUGUGGCGCCGUGGUGCCUCUGAGGUGGGGCCCUUCCACGAGCAGUUGGAAGAGGGCAUGGCCCAUUUCCAUCACCACGUCCAGCGGCGUUUGAGGGGCUUUGCGCCGUUUUGAUCCGGCAUCGGAUUUUUGCUACGUUGUCCCGCACAGAGGUACCGCU........CA
env-28    CCCGCCUGAUGCAUCUCCCAAUGAAAAUCUACUCCACCAACGGCACGCGAGCAAGGCCGGCAGCGUAAGCGACACACACCCAUGAAACAUAUGACAUGACGAAUACAGAUAGCGAAUCCCUUGCGAUCCUCAGUCUCCUGUAUUAACUUAAUGGCAUCCGGGGUACCGCU........GU
env-29    GAGUGCGUGGGGGUAUGGGGUAUCGGAUCAAUGAGGGGUUCAUUGCUGGUUCAGAAAAUGGGAUGGGAUGCAUCGCUAUUUCUUUGCGUGGCAGUCGAUGCAAUUCACUACGAAUUCGACAGGUUUUUUUGCCCGCAUUCCCCAAGGUGAAAUACACGCCGGGUACCGCU........UC
env-30    UUUCGAGUUGUCCCCGGACUGCUAUGGGCGCGUAGCACGCCUACGCGGGAGGCCGAAUUCGGCCAGAAGCAGUCGCUCACAGAAUCCCCAAGAAGCCGACGUAGCUCGACGGAGCCUGAGGCUUUAUAGCGCCGCUCUGAUAAGCUCCAAUGGUCAUUUGGGGUACCGCU.....CUUAG
env-31    AAGUGGUUUGAGGCGGGGGCGCCUACGGGGCCUUGAAUGUGUUGAGUGCUGCAGCAGUCGGCCAGGAGCAGUCGCUCGCAGAAUCGCCAAGAAGCGGACGUAGCGCAACUGAGCCUUAGGCCGUUGCGGUGCGCCUCUGAUAAUCUCCAGCACUGAUCCGGGGUACCGCU.....CUUAG
env-32    ............................................................................................................................GCGCGCCUCUGAUACUCUCCAAUACUGAUCCGGGGUACCGCU.....CUUAG
env-33    ..........................................................................CAGAAUCGCCAAGAAGCGGACGUGGCUCGACUGAGCCUUAGUCUGUUUCGGCGCGCCUCUGAUACUCUCCAAUACUGAUCCGGGGUACCGCU.....CUUAG
env-34    AAGUGGUUUGAGGCAGGGGCUGCAACGGGGCCUUGAAUGUGUUGAGUGCCGCGGCAAUCGGCCAGAAGCUGUCGCUCACAGAAUCGCCAAGAAGCGGACGUGGCUCGACUGAGCCUUAGUCUGUUUCGGCGCGCCUCUGAUACUCUCCAAUACUGAUCCGGGGUACCGCU.....CUUAG
env-35    ............................................................GCCAGAAGCUGUCGCUCACAGAAUCGCCAAGAAGCGGACGUGGCUCGACUGAGCCUUAGUCUGUUUCGGCGCGCCUCUGAUACUCUCCAAUACUGAUCCGGGGUACCGCU.....CUUAG
env-36    AAGUGGUUUGAGGCAGGGGCUGCAACGGGGCCUUGAAUGUGUUGAGUGCCGCGGCAAUCGGCCAGAAGCUGUCGCUCACAGAAUCGCCAAGAAGCGGACGUGGCUCGACUGAGCCUUAGUCUGUUUCGGCGCGCCUCUGAUACUCUCCAAUACUGAUCCGGGGUACCGCU.....CUUAG
env-37    ...............GGGCUGCAACGGGGCCUUGAAUGUGUUGAGUGCCGCGGCAAUCGGCCAGAAGCUGUCGCUCACAGAAUCGCCAAGAAGCGGACGUGGCUCGACUGAGCCUUAGUCUGUUUCGGCGCGCCUCUGAUACUCUCCAAUACUGAUCCGGGGUACCGCU.....CUUAG
env-38    ....................................................GGCAAUCGGCCAGAAGCUGUCGCUCACAGAAUCGCCAAGAAGCGGACGUGGCUCGACUGAGCCUUAGUCUGUUUCGGCGCGCCUCUGAUACUCUCCAAUACUGAUCCGGGGUACCGCU.....CUUAG
env-39    AAGUGGUUUGAGGCAGGGGCUGCAACGGGGCCUUGAAUGUGUUGAGUGCCGCGGCAAUCGGCCAGAAGCUGUCGCUCACAGAAUCGCCAAGAAGCGGACGUGGCUCGACUGAGCCUUAGUCUGUUUCGGCGCGCCUCUGAUACUCUCCAAUACUGAUCCGGGGUACCGCU.....CUUAG
env-40    AAGUGGUUUGAGGCAGGGGCUGCAACGGGGCCUUGAAUGUGUUGAGUGCCGCGGCAAUCGGCCAGAAGCUGUCGCUCACAGAAUCGCCAAGAAGCGGACGUGGCUCGACUGAGCCUUAGUCUGUUUCGGCGCGCCUCUGAUACUCUCCAAUACUGAUCCGGGGUACCGCU.....CUUAG
env-41    AAGUGGUUUGAGGCAGGGGCUGCAACGGGGCCUUGAAUGUGUUGAGUGCCGCGGCAAUCGGCCAGAAGCUGUCGCUCACAGAAUCGCCAAGAAGCGGACGUGGCUCGACUGAGCCUUAGUCUGUUUCGGCGCGCCUCUGAUACUCUCCAAUACUGAUCCGGGGUACCGCU.....CUUAG
env-42    AAGUGGUUUGAGGCAGGGGCUGCAACGGGGCCUUGAAUGUGUUGAGUGCCGCGGCAAUCGGCCAGAAGCUGUCGCUCACAGAAUCGCCAAGAAGCGGACGUGGCUCGACUGAGCCUUAGUCUGUUUCGGCGCGCCUCUGAUACUCUCCAAUACUGAUCCGGGGUACCGCU.....CUUAG
env-44    AAGUGGUUUGAGGCAGGGGCUGCAACGGGGCCUUGAAUGUGUUGAGUGCCGCGGCAAUCGGCCAGAAGCUGUCGCUCACAGAAUCGCCAAGAAGCGGACGUGGCUCGACUGAGCCUUAGUCUGUUUCGGCGCGCCUCUGAUACUCUCCAAUACUGAUCCGGGGUACCGCU.....CUUAG
env-45    AAGUGGUUUGAGGCAGGGGCUGCAACGGGGCCUUGAAUGUGUUGAGUGCCGCGGCAAUCGGCCAGAAGCUGUCGCUCACAGAAUCGCCAAGAAGCGGACGUGGCUCGACUGAGCCUUAGUCUGUUUCGGCGCGCCUCUGAUACUCUCCAAUACUGAUCCGGGGUACCGCU.....CUUAG
env-46    AAGUGGUUUGAGGCAGGGGCCGCAACGGGGCCUUGAAUGUGUUGAGUGCCGCGGCAAUCGGCCAGAAGCUGUCGCUCACAGAAUCGCCAAGAAGCGGACGUGGCUCGACUGAGCCUUAGUCUGUUUCGGCGCGCCUCUGAUACUCUCCAAUACUGAUCCGGGGUACCGCU.....CUUAG
env-47    AAGUGGUUUGAGGCAGGGGCUGCAACGGGGCCUUGAAUGUGUUGAGUGCCGCGGCAAUCGGCCAGAAGCUGUCGCUCACAGAAUCGCCAAGAAGCGGACGUGGCUCGACUGAGCCUUAGUCUGUUUCGGCGCGCCUCUGAUACUCUCCAAUACUGAUCCGGGGUACCGCU.....CUUAG
env-48    AAGUGGUUUGAGGCAGGGGCUGCAACGGGGCCUUGAAUGUGUUGAGUGCCGCGGCAAUCGGCCAGAAGCUGUCGCUCACAGAAUCGCCAAGAAGCGGACGUGGCUCGACUGAGCCUUAGUCUGUUUCGGCGCGCCUCUGAUACUCUCCAAUACUGAUCCGGGGUACCGCU.....CUUAG
env-49    AAGUGGUUUGAGGCAGGGGCUGCAACGGGGCCUUGAAUGUGUUGAGUGCCGCGGCAAUCGGCCAGAAGCUGUCGCUCACAGAAUCGCCAAGAAGCGGACGUGGCUCGACUGAGCCUUAGUCUGUUUCGGCGCGCCUCUGAUACUCUCCAAUACUGAUCCGGGGUACCGCU.....CUUAG
env-50    ...............GGGCUGCAACGGGGCCUUGAAUGUGUUGAGUGCCGCGGCAAUCGGCCAGAAGCUGUCGCUCACAGAAUCGCCAAGAAGCGGACGUGGCUCGACUGAGCCUUAGUCUGUUUCGGCGCGCCUCUGAUACUCUCCAAUACUGAUCCGGGGUACCGCU.....CUUAG
env-51    ..............................................GUGCCGCGGCAAUCGGCCAGAAGCUGUCGCUCACAGAAUCGCCAAGAAGCGGACGUGGCUCGACUGAGCCUUAGUCUGUUUCGGCGCGCCUCUGAUACUCUCCAAUACUGAUCCGGGGUACCGCU.....CUUAG
env-52    AAGUGGUUUGAGGCAGGGGCCGCAACGGGGCCUUGAAUGUGUUGAGUGCCGCGGCAAUCGGCCAGAAGCUGUCGCUCACAGAAUCGCCAAGAAGCGGACGUGGCUCGACUGAGCCUUAGUCUGUUUCGGCGCGCCUCUGAUACUCUCCAAUACUGAUCCGGGGUACCGCU.....CUUAG
env-53    AAGUGGUUUGAGGCAGGGGCUGCAACGGGGCCUUGAAUGUGUUGAGUGCCGCGGCAAUCGGCCAGAAGCUGUCGCUCACAGAAUCGCCAAGAAGCGGACGUGGCUCGACUGAGCCUUAGUCUGUUUCGGCGCGCCUCUGAUACUCUCCAAUACUGAUCCGGGGUACCGCU.....CUUAG
env-61    AAGUGGUUUGAGGCAGGGGCUGCAACGGGGCCUUGAAUGUGUUGAGUGCCGCGGCAAUCGGCCAGAAGCAGUCGCUCACAGAAUCGCCAAGAAGCGGACGUGGCUCGACGGAGCCUUAGGCUGUUGCGGCGCGCCUCUGAUAAUCUCCAAUACUGAUCCGGGGUACCGCU.....CUUAG
env-62    ..........................................................................................GGCGUGGAUCGACUGAGCCUUAGGCUGUUUCGGCGCGCCUCUGAUAAUCUCCAAUACUGAUCCGGGGUACCGCU.....CUUAG
env-63    .........................................................................................................................................................CGGGGUACCGCU.....CUUAG
env-64    ........................................................................................................ACUGAGCCUUAGGCUGUUUCGGCGCGCCUCUGAUAAUCUCCAAUACUGAUCCGGGGUACCGCU.....CUUAG
env-65    AAGUGGUUUGAGGCAGGGGCCGCAACGGGGCCUUGAAUGUGUUGAGUGCCGCAACAAUCGGCCAGAAGCAGUCGCUCACAGAAUCGCCAAGAAGCGGGCGUGGAUCGACUGAGCCUUAGGCUGUUUCGGCGCGCCUCUGAUAAUCUCCAAUACUGAUCCGGGGUACCGCU.....CUUAG
env-66    AAGUGGUUUGAGGCAGGGGCUGCAACGGGGCCUUGAAUGUGUUGAGUGCCGCAACAAUCGGCCAGAAGCAGUCGCUCACAGAAUCGCCAAGAAGCGGGCGUGGAUCGACUGAGCCUUAGGCUGUUUCGGCGCGCCUCUGAUAAUCUCCAAUACUGAUCCGGGGUACCGCU.....CUUAG
env-70    GGGCCGCAACGGGGCCUUGAAUGUGUUGAGUGCCGCAACAAUCGGCCAGAAGCAGUCGCUCACAGAAUCGCCAAGAAGCG...............GGCGUGGAUCGACUGAGCCUUAGGCUGUUUCGGCGCGCCUCUGAUAAUCUCCAAUACUGAUCCGGGGUACCGCU.....CUUAG
Asp-8-1   AAGUGGUUUGAGGCAGGGGCCGCAACGGGGCCUUGAAUGUGUUGAGUGCCGCAACAAUCGGCCAGAAGCAGUCGCUCACAGAAUCGCCAAGAAGCGGGCGUGGAUCGACUGAGCCUUAGGCUGUUUCGGCGCGCCUCUGAUAAUCUCCAAUACUGAUCCGGGGUACCGCU.....CUUAG
env-71    GCUCACAGAAUCGCCAAGAAGCGGACGUGGCUCGACUGAGCCUUAGUCUGUUUCGGCGCGCCUCUGAUACUCUCCAAUAC.................................................................UGAUCCGGGGUACCGCU.....CUUAG
env-72    GCUCACAGAAUCGCCAAGAAGCGGACGUGGCUCGACUGAGCCUUAGUCUGUUUCGGCGCGCCUCUGAUACUCUCCAAUAC.................................................................UGAUCCGGGGUACCGCU.....CUUAG
env-73    ..........................................................................................................................................................UGAUCCGGGGUACCGCU.....CUUAG
env-74    AAGUGGUUUGAGGCAGGGGCUGCAACGGGGCCUUGAAUGUGUUGAGUGCCGCGGCAAUCGGCCAGAAGCAGUCGCUCACAGAAUCGCCAAGAAGCGGACGUGGCUCGACGGAGCCUUAGGCUGUUGCGGCGCGCCUCUGAUAAUCUCCAAUACUGAUCCGGGGUACCGCU.....CUUAG
env-75    AAGUGGUUUGAGGCAGGGGCUGCAACGGGGCCUUGAAUGUGUUGAGUGCCGCGGCAAUCGGCCAGAAGCAGUCGCUCACAGAAUCGCCAAGAAGCGGACGUGGCUCGACGGAGCCUUAGGCUGUUGCGGCGCGCCUCUGAUAAUCUCCAAUACUGAUCCGGGGUACCGCU.....CUUAG
Asp-2-1   AAGUGGUUUGAGGCAGGGGCUGCAACGGGGCCUUGAAUGUGUUGAGUGCCGCGGCAAUCGGCCAGAAGCAGUCGCUCACAGAAUCGCCAGGAAGCGGACGUAGCUCGACUGAGCCUUGGGCUGUUUCGGCGCGCCUCUGAUACUCUCCAAUACUGAUCCGGGGUACCGCU.....CUUAG
Asp-7-1   AAGUGGUUUGAGGCAGGGGCUGCAACGGGGCCUUGAAUGUGUUGAGUGCCGCGGCAAUCGGCCAUAAGCAGUCGCUCACAGAAUCGCCAGGAAGCGGACGUGGCUCGACUGAGCCUUAGGCUGUUUCGGCGCGCCUCUGAUACUCUCCAAUACCGAUCCGGGGUACCGCU.....CUUAG
env-76    AAGUGGUUUGAGGCAGGGGCUGCAACGGGGCCUUGAAUGUGUUGAGUGCCGCGGCAAUCGGCCAGAAGCAGUCGCUCACAGAAUCGCCAAGAAGCGGACGUGGCUCGACGGAGCCUUAGGCUGUUGCGGCGCGCCUCUGAUAAUCUCCAAUACUGAUCCGGGGUACCGCU.....CUUAG
env-77    GCAAUCGGCCAGAAGCUGUCGCUCACAGAAUCGCCAAGAAGCGGACGUGGCUCGACUGAGCCUUAGUCUGUUUCGGCGCG.....................................................CCUCUGAUACUCUCCAAUACUGAUCCGGGGUACCGCU.....CUUAG
env-78    ..............................................................................................................................CCUCUGAUACUCUCCAAUACUGAUCCGGGGUACCGCU.....CUUAG
env-79    ..............................................................................................................................................CUCCAAUACUGAUCCGGGGUACCGCU.....CUUAG
env-80    ..............................................................................................................................CCUCUGAUACUCUCCAAUACUGAUCCGGGGUACCGCU.....CUUAG
env-81    .....................................................................................................................................................GAUCCGGGGUACCGCU.....CUUAG
env-82    ..............................................................................................................................CCUCUGAUACUCUCCAAUACUGAUCCGGGGUACCGCU.....CUUAG
env-83    AAGUGGUUUGAGGCAGGGGCUGCAACGGGGCCUUGAAUGUGUUGAGUGCCGCGGCAAUCGGCCAGAAGCAGUCGCUCACAGAAUCGCCAGGAAGCGGACGUAGCUCGACUGAGCCUUAGGCUGUUUCGGCGCGCCUCUGAUACUCUCCAAUACUGAUCCGGGGUACCGCU.....CUUAG
env-84    GAAGUGGUUUGAGGCAGGGGCUGCAACGGGGCCUUGAAUGUGUUGAGUGCCGCGGCAAUCGGCCAGAAGCAGUCGCUCACAGAAUCGCCAAGAAGCGGACGUGGGCGACUGAGCCUUUGGCUGUUUCGGCGCGCCUCUGAUAAUCUCCAACACUGAUCCGGGGUACCGCU.....CUUAG
env-85    CGAUGCCGGCCCGCACCACGAGCGGCUGGAAGCAGGCAUGGCCCACUUCCAUCACUACCUGUGGAACCGCAUGGGCGAUGCUUGCUGGCGUUGAAGCCCCGCAAUUGAUCGGUCUUUCUGCCAGCAUCAGCCUGAUCUUUGGUAAUCUGCCCAUCGUUCGAGGUACCGCU.......CUC
Vsp-4-1   CACGCUGAUCGUCAUCGUUGUGGCCGUGCUGAUCCUCCCGCGCCUGGCCAAGCGCAUGAGCGAACGCAAGCUGGCAAAGCUGGCGCAGAUGGACGCCUGAGUCUUUUCUUCGUCAUCAUCGGGAAACCGGAUGCUGUCAGCAUCCGGUUUUUUCGUUUUUGGGUACCGCU.....UUUCC
Vsp-5-1   CACGCUGAUCGUCAUCGUUGUGGCCGUGCUGAUCCUCCCGCGCCUGGCCAAGCGCAUGAGCGAACGCAAGCUGGCAAAGCUGGCGCAGAUGGACGCCUGAGUCUUUUUUUCGUCAUCAUCGGGAAACCGGAUGCUGUCAGCAUCCGGUUUUUUCGUUUUUGGGUACCGCU....UUUCCC
env-87    ...........................................................................................................................................GUUAGCAUCCGCAACUCAAUGGGGUACCGCU.......CCC
Cmi-1-1   CCCGCCUGAUGCAUCUUCCAAUGAAAAUCUACUCCACCAACGGCACGCGAGCAAGGCCGGCAGCGUAAGCGACACACACCCAUGAAACAUAUGACAUGACGAAUACAGAUAGCGAACCCCUUGCGAUCCUCAGUCUCCUGUAUUAACUUAAUGGCAUCCGGGGUACCGCU........GU
[me-1-1   GGUCAGCAAUGCGGUGGCGAAGGUGAAGGCGGAGAUCGUCGGCUUGUUCGAUGCGCUGGGCAAGCGGCCGGAGUGGGCGGCGGGGAGCGGAAUCGAGCCGUGAAUGUGUCGGGGCCCCGUUUGCGUUCGCCGGGCACGUGUCCUAAUCUCGUGGCAUCCGGGGUACCGCU........GC
Bce-1-3   GCACGACACGGCGCUCGUCAGCAACGCGGUGCAGAAGGUGAAGGCCGAGAUCGUCGGCUUGUUCGACACGCUCGGCCAGCGGCCGGAAUGGGCGGACGGCCGCGCGGCGCGCCGGUGAGCUUGCCUUCCCGCGCGGCAGCGACUAAGCUCGUCGCAUCCUGGGUACCGCU.......GAC
Bps-1-1   GCACGACACGGCGCUCGUCAGCAAUGCGGUGCAGAAGGUGAAGGCCGAGAUCGUCGGCUUGUUCGACACGCUCGGCCAGCGGCCGGAAUGGGCGGACGGCCGGGCGGCGCAUCGGUGAGCUUGCCUUCCCGCGCGGCAGCGACUAAGCUCGUCGCAUCCUGGGUACCGCU........GA
Bsp-5-1   GACACGGCGCUCGUCAGCAACGCGGUGCAGAAGGUGAAAGCCGAAAUCGUCGGCUGUUCGACACGCUCGGGCAGCGGCCGGAGUGGGCGGCCGGCCAUGCCGGUCGCGCGUGACGUACCUUGUGCCGCCCCGUCGCGCGUACUAAGCUCGUUUCAUUCUGGGUACCGCU.....GACAC
Bsp-7-1   GACACGGCGCUCGUCAGCAACGCGGUGCAGAAGGUGAAAGCCGAAAUCGUCGGCUGUUCGACACGCUCGGGCAGCGGCCGGAGUGGGCGGCCGGCCACGCCGGUCGCGCGUGACGGACCUUGUGCCGCCCCGCCGCGCGUACUAAGCUCGUUUCAUUCUGGGUACCGCU.....GACAC
Bsp-6-1   GACACGGCGCUCGUCAGCAACGCGGUGCAGAAGGUGAAAGCCGAAAUCGUCGGCUGUUCGACACGCUCGGGCAGCGGCCGGAGUGGGCGGCCGGACACGCCGGUCGCGCGUGACGGACCUUGUGCCGCCCCGCCGCGCGUACUAAGCUCGUUUCAUCCUGGGUACCGCU.....GACAC
Bsp-3-1   GACACGGCGCUCGUCAGCAACGCGGUGCAGAAGGUGAAAGCCGAAAUCGUCGACUGUUCGACACGCUCGGGCAGCGGCCGGAGUGGGCGACCGGACACGCCGGUCGCGCGUGACGGACCUUGUGCCGCCCCGCCGCGCGUACUAAGCUCGUUUCAUCCUGGGUACCGCU.....GACAC
Bsp-2-1   GACACGGCGCUCGUCAGCAACGCGGUGCAGAAGGUGAAAGCCGAAAUCGUCGGCUGUUCGACACGCUCGGGCAGCGGCCGGAGUGGGCGGCCGAACACGCCGGUCGCGCGUGACGGACCUUGUGCCGCCCCGCCGCGCGUACUAAGCUCGUUUCAUCCUGGGUACCGCU.....GACAC
Bsp-4-1   GACACGGCGCUCGUCAGCAACGCGGUGCAGAAGGUGAAAGCCGAAAUCGUCGGCUGUUCGACACGCUCGGGCAGCGGCCGGAGUGGGCGGCCGGACACGCCGGUCGCGCGUGACGGACCUUGUGCCGCCCCGCCGCGCGUACUAAGCUCGUUUCAUCCUGGGUACCGCU.....GACAC
Bsp-16-1  UGCAGAAGGUGAAGGCGGAGAUCGUCGCGCUGUUCGACACGCUCGGGCGCCGGCCGGAAUGGGCGGCAGGAAUGCCGGGCACGUGUAACGCGUGACGCGCAGCGUGCAUCGCCCGCGCCUUGCGCAACGCCACCGCACGCACUAAGCUCCUGUCAUCCUGGGUACCGCU.....GACAC
Bsp-15-1  UGCAGAAGGUGAAGGCGGAAAUCGUCGCGCUGUUCGACACGCUCGGACGCCGGCCGGAGUGGGCGGCAGGAAAUGCCGGGCACGCGUGACGCGCGACGCACGUCGUGCAUCGCCCGUGCCUUGCGCAACGCCGCCGCACGCGCUAAGCUCCUUUCAUCCUGGGUACCGCU....GACACC
Bpu-1-1   GCUCGUCAGCAACGCGGUGCAGAAGGUGAAAGCCGAAAUCGUCCGGUUGUUCGACACGCUCGGGCAACGGCUCGAGUGGGCGGCCGGGCAGGCCGGUGCAUGACGAGCGCGCCGCGUGCCUUGUGCUGCGCCGCCACGCGUACUAAGCUCCGUUCAUCCUGGGUACCGCU....GACACC
Bst-1-1   CGUCAGCAACGCGGUGCAGAAGGUGAAGGCCGAGAUCGUCGGCUGUUCGACACGCUCGGGCAGCGACGGCCGGAGUGGGCGGCCGGCGAUGCCGGCCGCGCGUGACUUGCGCGAUGCAUGCCUUGUGCCGCCCCGCCGCGCGUACUAAGCUGCUCGCAUCCCGGGUACCGCU....ACUCCC
Bsp-9-1   CGUCAGCAACGCGGUGCAGAAAGUGAAAACCGAGAUCGUCGGCUGUUCGACGCGCUCGGGCAGCGGCCGAGUGGGCGUCCGGGCAGGCCGACGGCGCGUGACGGGUACGCUGCGUGCCUUGUGCUGCCUCGCCGUGCGUACUAAGCUCCGUUCAUCCUGGGUACCGCU....GACACC
Bvi-2-1   UCGUCAGCAACGCGGUGCAGAAGGUGAAAGCCGAGAUCGUCCGGCUAUUCGACACGCUCGGGCAGCGACCGGAGUGGGCGGCCGGCAACACCGACCGCGCAUGACUGGCGCGCGCAUGCCUUGUAUUGCCCUUCCGCGCGUACUAAGCUGCUUUCGUUCUGGGUACCGCU....GACACC
Bla-1-1   CGUCAGCAACGCGGUACAGAAGGUGAAAGCCGAGAUCGUCCGGCUGUUCGACACGCUCGGGCAGCGACCGGAGUGGGCGGCCGGCAACACCGACCGCGCAUGACUGGCGCGGCGCAUGCCUUGUAUUGCCCUUCCGCGCGUACUAAGCUGCUUUCGUUCUGGGUACCGCU....GACACC
Bce-4-1   CGUCAGCAACGCGGUACGGAAGGUGAAAGCCGAGAUCGUCCGGCUGUUCGACACGCUCGGGCAGCGACCGGAGUGGGCGGCCGGCAACACCGACCGCGCAUGACUGGCGCGGCGCAUGCCUUGUAUUGCCCUUCCGCGCGUACUAAGCUGCUUUCGUUCUGGGUACCGCU....GACACC
Bsp-21-1  UACGGCGCUCGUCAGCAACGCGGUGCAGAAGGUGAAAGCCGAGAUCGUCGGCUGUUCGACACACUCGGGCAGCGGCCGAAUGGGCGGCCGGGCAGGCGUGACGGGCAGGCGGCAUGCGUUGCCCUGCCGCGCCGUGUGGACUAAGCUCCUUUCAUCCUGGGUACCGCU....GACACC
Bsp-1-1   CGGUGCAGAAAGUGAAAGCCGAGAUCGUCCGGCUGUUCGACACGCUCGGGCAGCGGCCCGAGUGGGCGGCCGGGCAGGCCGGCCGUGCGUGACCGGGGCGCCGCGUGCCUUGCCCCUUCCUUGUGCUGCGAAGGCGCGCGGACUAAGCUUCGUUCAUCCUGGGUACCGCU....GACACC
Bsp-14-1  CGGUGCAGAAGGUGAAAGCGGAGAUCGUCCAGCUGUUCGACACGCUCGGGCAGCGGCCGGAGUGGGCGGCCGGGCAGGCCGACGGUGCGUGACCGGGGCGCCGCGUGUCUUGUGCCUUCCUUGUGCUGCCAAGGCGCGCGUACUAAGCUCAGUUCAUCCUGGGUACCGCU....GACACC
Bsp-12-1  CGGUGCAGAAGGUGAAAGCGGAGAUCGUCCGGCUGUUCGAUGCGCUCGGGCAGCGGCCGGAGUGGGCAACCGGGCAGGCCGACGGUGCGUGACCGGGGCGCCGCGUGUCUUGUGCCUUCCUUGUGCUGCCAAGGCGCGCGUACUAAACUCAGUCCAUCCUGGGUACCGCU....GACACC
Bse-1-1   ACGCGGUGCAGAAGGUCAAGGGCGAGAUCGUCCGGCUGUUCGACACGCUCGGGCAGCGGCCGGAGUGGGCGGCCGGCAAUAACGAUCGCAGCGAUCGCACGUGACCCGUGCCGCGCUUGCUUAGUGCGGCCCCACCGCACGUACCAAGCUCCUUUCAUGGGGGGACCGCU....GACACC
Bce-6-1   UCGUCAGCAACGCGGUGCAGAAAGUGAAGGCCGAGAUCGUCCGGCUGUUCGACACGCUCGGGCAGCGGCCCGAAUGGGCGGCCGGCAAUGGCGAUCGCGCGUGACCGGCGCCGCCCGUACCUUGUGCGGCCCCGCCGCGCGUACUAAGCUCCUUCCACCUGGGUACCGCU....GACACU
env-88    ACGUUAGGCGAGGCAAUUAAGUACCAUAAAGAGGACUGAGUUUUAGAUUUGCUGGUUUUUGGCUUCUUUUAUCUCAUGGCGAACGGAAUGCUUGGAAAAAAAAUUGCCCUCCCAUUGAAUGCCUAAACCAUCCCAACUACAAUCAAGCCUAGAUUUAUAUAAGGUACCGCU......CUAA
env-90    ................................................................................AGGCGUCACCAAACCAUCUGCUCUUCCACGUUCUCAGUCGUGGUUGCUCCGAAGUCAGAAGCUAAACUGAUAGUGCUUCAGGUACCGCU.......UAC
Rsp-1-1   CGAGUUGCAAGUUCACCAGAUCCCGGUGCUGUUCGGCGGUGGGCGUCGGCUGUUCGAGGUGUUGCCGUCGCGCAUCGAGCUGGAGAUCGUUCGGGUGAUCGACACGCCGGAGGCCACACACAUCCGCUACCGCGUCCGUCGCUGAGCCGGGCUGUGCUUCAGGUACCGCU.......UAC

          ...............................................................................................................................................................................
          ...............................................................................................................................................................................
          ...............................................................................................................................................................<<<<<<...........
          ...............................................................................................................................................................00000............
          ●●●●●●●●●●●●●●●●●●●●●●●●●●●●●●●●●●●●●●●●●●●●●●●●●●●●●●●●●●●●●●●●●●●●●●●●●●●●●●●●●●●●●●●●●●●●●●●●●●●●●●●●●●●●●●●●●●●●●●●●●●●●●●●●Y●●●●●●●YYY●RGGUACCGCU-----○○●Y
                                                                                 5'
```

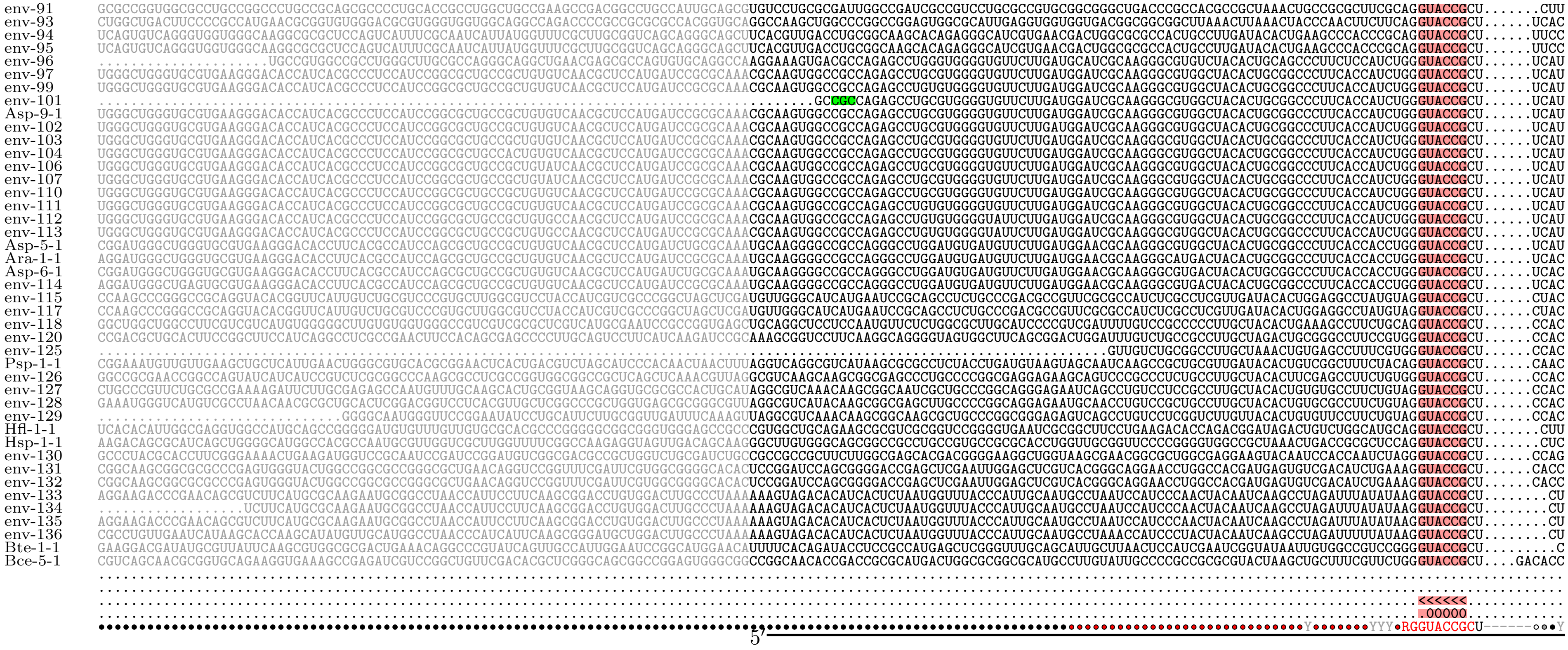

alignment positions 181 · · · 360

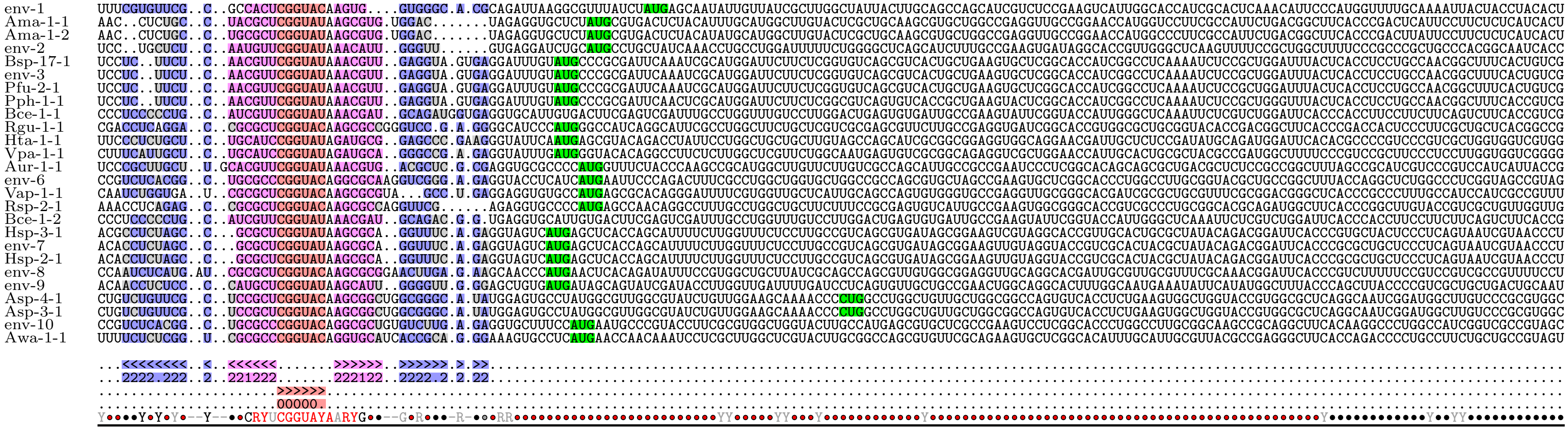

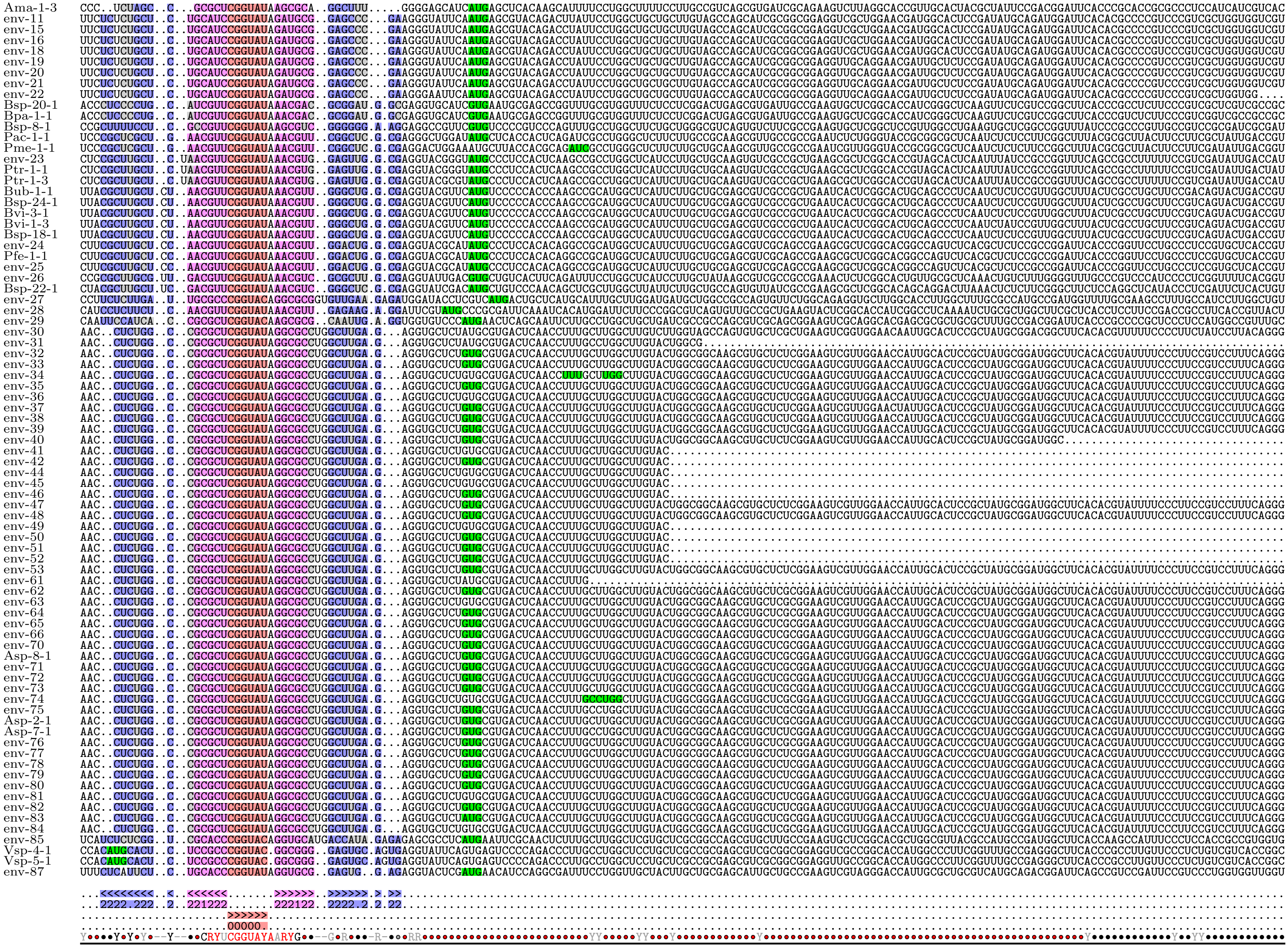
Ama-1-3
env-11
env-15
env-16
env-18
env-19
env-20
env-21
env-22
Bsp-20-1
Bpa-1-1
Bsp-8-1
Pac-1-1
Pme-1-1
env-23
Ptr-1-1
Ptr-1-3
Bub-1-1
Bsp-24-1
Bvi-3-1
Bvi-1-3
Bsp-18-1
env-24
Pfe-1-1
env-25
env-26
Bsp-22-1
env-27
env-28
env-29
env-30
env-31
env-32
env-33
env-34
env-35
env-36
env-37
env-38
env-39
env-40
env-41
env-42
env-44
env-45
env-46
env-47
env-48
env-49
env-50
env-51
env-52
env-53
env-61
env-62
env-63
env-64
env-65
env-66
env-70
Asp-8-1
env-71
env-72
env-73
env-74
env-75
Asp-2-1
Asp-7-1
env-76
env-77
env-78
env-79
env-80
env-81
env-82
env-83
env-84
env-85
Vsp-4-1
Vsp-5-1
env-87

```
Cmi-1-1    CAUCCUCUUCU..C..AACGUUCGGUAUAAACGUU..GAGGUA.G.CGAGGAUUUGUAUGCCCGCGAUUCAAAUCGCAUGGAUUCUUCUCGGCGUCAGUGUUGCCGCUGAAGUACUCGGCACCAUCGGCCUCAAAAUCUCCGCUGGCUUCACUCACCUCCUUCCGGCUGCCUUCACCAUU
[me-1-1    CAUCCUCUCCU..C..GACGUUCGGUAUAAGCGUC..GGAGGC.A.GGAGACUGUCCGUGACCUCGCUCCAAUCCGCCUGGCUUCUGCUGGGCAUCAGCGUCGCCGCCGAGGUGCUCGGCUCGAUCGGCCUAAAAUUCUCCGUCGGCUUCUCCCGCGAUGCCGUCCGCGUUGACGGUC
Bce-1-3    ACCCCUCUCCU..C..GCCGCUCGGUAUAAGCGUC..GGGGGA.A.GGCGUCCGUUCGUGUCCCCGAUCCAGUCUGCCUGGCUGCUGCUGGGCGUCAGCGUCCUCGCCGAAGUGCUCGGCUCCAUCGGCCUCAAAUACUCCGCCGGUUUUACCCGUCCUGUGCCGUCCGUCGCGACCGUC
Bps-1-1    CACCCUCUCCU..C..GCCGCUCGGUAUAAGCGUC..GGGGGA.A.GGCGUCCGUUCGUGUCCCCGAUCCAGUCUGCCUGGCUGCUGCUGGGCGUCAGCGUCCUCGCCGAAGUGCUCGGCUCCAUCGGCCUCAAAUACUCCGCCGGUUUUACCCGCCCUGUGCCGUCCGUCGCGACCGUC
Bsp-5-1    CCCACUUUUCC.UC..GCCGCUCGGUAUAAGCGUC..GGGGGA.A.GGAGGCCGUCCGUGUCCCCGUUCCAGUUUGCCUGGCUUCUGCUGGGCGUCAGUGUCCUCGCCGAAGUGCUCGGCUCCGUUGGCCUGAAGUGCUCGGCCGGUUUGUCCCGCCCGUUGCCGUCCGCGAUCGCGAUC
Bsp-7-1    CCCACUUUUCC.UC..GCCGCUCGGUAUAAGCGUC..GGGGGA.A.GGAGGCCGUCCGUGUCCCCGUUCCAGUUUGCCUGGCUUCUGCUGGGCGUCAGUGUCCUCGCCGAAGUGCUCGGCUCCGUUGGCCUGAAGUGCUCGGCCGGUUUGUCCCGCCCGUUGCCGUCCGCGAUCGCGAUC
Bsp-6-1    CCCACUUUUCC.UC..GCCGCUCGGUAUAAGCGUC..GGGGGA.A.GGAGGCCGUCCGUGUCCCCGUUCCAGUUUGCCUGGCUUCUGCUGGGCGUCAGUGUCCUCGCCGAAGUGCUCGGCUCCGUUGGCCUGAAGUGCUCGGCCGGUUUAUCCCGCCCGUUGCCGUCCGCGAUCGCGAUC
Bsp-3-1    CCCACUUCUCC.UC..GCCGCUCGGUAUAAGCGUC..GGGGGA.A.GGAGGCCGUUCGUGUCCCCGUUCCAGUUUGCCUGGCUUCUGCUGGGCGUCAGUGUCCUCGCCGAAGUGCUCGGCUCCGUUGGCCUGAAGUGCUCGGCCGGUUUGUCCCGCCCGUUGCCGUCCGCGAUCGCGAUC
Bsp-2-1    CCCACUUCUCC.UC..GCCGCUCGGUAUAAGCGUC..GGGGGA.A.GGAGGCCGUUCGUGUCCCCGUUCCAGUUUGCCUGGCUUCUGCUGGGCGUCAGUGUCCUCGCCGAAGUGCUCGGCUCCGUUGGCCUGAAGUGCUCGGCCGGUUUGUCCCGCCCGUUGCCGUCCGCGAUCGCGAUC
Bsp-4-1    CCCACUUCUCC.UC..GCCGCUCGGUAUAAGCGUC..GGGGGA.A.GGAGGCCGUUCGUGUCCCCGUUCCAGUUUGCCUGGCUUCUGCUGGGCGUCAGUGUCCUCGCCGAAGUGCUCGGCUCCGUUGGCCUGAAGUGCUCGGCCGGUUUGUCCCGCCCGUUGCCGUCCGCGAUCGCGAUC
Bsp-16-1   UACCCUUUUCC.UC..GCCGCUCGGUAUAAGCGUC..GGGGGA.A.GGAGUCCAUUCGUGUCCCCGUUCCAGUCCGCCUGGCUUCUGCUGGGCAUCAGCGUUGUCGCCGAAGUACUCGGCUCCGUCGGCCUGAAAUGCUCCGCCGGCCUGUCCCGCCCGCUACCGUCCGCCAUCACGAUC
Bsp-15-1   UGCUCUUCUCC.UC..GCCGCUCGGUAUAAGCGUC..GGGGGA.A.GGAGCCCGUUCGUGUCCCCGUUCCAGUCCGCCUGGCUUUUGCUGGGCAUCAGCGUUGUCGCCGAAGUACUCGGCUCCGUCGGCCUGAAAUGCUCCGCCGGCCUGUCCCGCCCGCUACCGUCCGCCAUCACGAUC
Bpu-1-1    CCCACUUCUCC.UC..GCCGCUCGGUAUAAGCGUC..GGACGA.A.GGAGGCUGCCCGUGUCCCCGCUCCAGUCUGCCUGGCUUCUGCUGGGCGUCAGUGUCCUCGCCGAAGUGCUCGGCUCCGUCGGCCUGAAAUACUCCGCCGGCUUUUCCCGCCCGCUCCCCUCCACGCUCACCGUC
Bst-1-1    UCCUCUUCUCC.UC..GCCGCUCGGUAUAAGCGUC..GGGGGA.A.GGAGGCCGUUCGUGUCCCCGCUCCAGUCUGCCUGGCUUCUGCUCGGCGUCAGUGUCCUCGCCGAAGUGCUCGGCUCCGUCGGCCUGAAAUUCUCGGCCGGCUUUUCCCGCCCGCUGCCGUCCGCAAUCACGAUC
Bsp-9-1    GCCACUUCUCC.UC..GCCGCUCGGUAUAAGCGUC..GGGGGA.A.GGAGGCCGUUCGUGUCCCCGCUCCAGUCUGCCUGGCUUUUGCUGGGCGUCAGUGUCUUCGCCGAAGUCCUCGGCUCCGUCGGCCUGAAAUUGUCGGCCGGCUUUUCCCGCCCGCUCCCCUCCGCGCUCACCGUC
Bvi-2-1    UCCACUUCUCC.UC..GCCGCUCGGUAUAAGCGUC..GGGGGA.A.GGAGGCCGUUCGUGUCCCCGCUCCAGUCUGCCUGGCUUCUGCUGGGCGUCAGUGUCCUCGCCGAAGUGCUCGGCUCCGUCGGCCUCAAAUACUCGGCCGGCUUUUCCCGCCCGCUCCCGUCCGCGCUCACCGUC
Bla-1-1    UCCACUUCUCC.UC..GCCGCUCGGUAUAAGCGUC..GGGGGA.A.GGAGGCCGUUCGUGUCCCCGCUCCAGUCUGCCUGGCUUCUGCUGGGCGUCAGUGUCCUCGCCGAAGUGCUCGGCUCCGUCGGCCUCAAAUACUCGGUCGGCUUUUCCCGCCCGCUCCCGUCCGCGCUCACCGUC
Bce-4-1    UCCACUUCUCC.UC..GCCGCUCGGUAUAAGCGUC..GGGGGA.A.GGAGGCCGUUCGUGUCCCCGCUCCAGUCUGCCUGGCUUCUGCUGGGCGUCAGUGUCCUCGCCGAAGUGCUCGGCUCCGUCGGCCUCAAAUACUCGGUCGGCUUUUCCCGCCCGCUCCCGUCCGCGCUCACCGUC
Bsp-21-1   UCCACUUCUCC.UC..GCCGCUCGGUAUAAGCGUC..GGGGGA.A.GGAGGCCGUUCGUGUCCCCACUUCAGUCUGCCUGGCUUCUGCUGGGCGUCAGUGUCCUCGCCGAAGUGCUCGGCUCCGUCGGCCUCAAAUACUCGGCCGGCUUUUCCCGCCCGCUCCCGUCCGCGCUCACCGUC
Bsp-1-1    CCCACUUCUCC.UC..ACCGCUCGGUAUAAGCGUC..GGGGGA.A.GGAGGCCGUUCGUGUCCCCGCUCCAGUCUGCCUGGCUUCUGCUGGGCGUCAGUGUCCUCGCCGAAGUACUCGGCUCCGUCGGCCUGAAAUAUUCGGCCGGCUUUUCCCGCCCGCUCCCCUCCGCACUCACCGUC
Bsp-14-1   CUCACUUCUCC.UC..GCCGCUCGGUAUAAGCGUC..GGGGGA.A.GGAGGCCGUUCGUGUCCCCGCUCCAGUCUGCCUGGCUUCUGCUGGGCGCCAGUGUUUUCGCCGAAGUGCUCGGCUCCGUCGGACUGAAAUACUCUGCCGGAUUUUCCCGCCCGCUCCCCUCCGCACUCACCGUC
Bsp-12-1   CUCACUUCUCC.UC..GCCGCUCGGUAUAAGCGUC..GGGGGA.A.GGAGGCCGUUCGUGUCCCCGCUCCAGUCUGCCUGGCUUCUGCUGGGCGCCAGUGUUUUCGCCGAAGUGCUCGGCUCCGUCGGACUGAAAUACUCCGCCGGAUUUUCCCGCCCGCUCCCCUCCGCACUCACCGUC
Bse-1-1    UCCGCUUCUCC.UU..GCCGCCCGAUAUAAGCGUC..GGGGGA.A.GGAGGCCGUUCGUGUCCCCGUUCCAGUCUGCCUGGCUUCUGCUGGGCGUCAGUGUUCUCGCCGAAGUGCUCGGCUCCGUCGGCCUGAAAUUUUCGGCCGUUUUUUCCCGCCCGCUGCCGUCUGCGCUCACGAUC
Bce-6-1    GCCACUUCUCU.UC..GCCGCUCGGUACAAGCGUC..GGGGGA.A.GGAGGCCGUUCGUGUCCCCGCUCCAGUCUGCCUGGCUUCUGCUGGGCGUCAGUGUUCUCGCCGAAGUGCUCGGCUCCGUCGGCCUGAAAUUGUCGGCCGGCUUUUCCCGCCCGCUGCCGUCCGCGAUCACGGUC
env-88     CUUCGCGCUCC..C..ACCACUCGGUACAAGUGGAAUGCGCGC.A.UUUAGUUGAGGUAUUUAUGAGCCCCAUCCUUGUCGCAUGGCUGCUGCUUGCCGCCAGUAUACUUUCUGAAGUUGUCGGCACCGUCGCCCUAAAACACUCCAACGGUUUUGCCAAUAUCUUACCCACCACCUGCG
env-90     CCAACUGCUAG..C..GCCACUCGGUACAAGUGGUCGGCUUGC.ACGUUCAGUACGGGUACCGUAUGAAAUCAACACUCGUGGCCUGGUCAGUUCUGGUGGCCAGCGUAGUUGCGGAGGUAGCAGGUACCGUGGCACUCAAACACUCCAGUGGGUUCACGAAGUUUCUUCCUGCUGCCAU
Rsp-1-1    CCAACUGCUAG..C..GCCACUCGGUACAAGUGGUCGGCUCGC.ACGUUCAGUAGGGGUAUCGUAUGAAAUCAACACUCGUGGCCUGGUCGGUUCUGGUGGCCAGCGUCGUUGCGGAGGUGGCAGGUACUGUGGCACUCAAACACUCCAGUGGGUUCACGAAGUUUCUUCCGGCUGCCGU
env-91     CAUGCUGCGCA..C..GCCACUCGGUACAAGUGGCCAGUGCGCCA.GUGGUGGAGAGGUAUCCCAUGAGCCCCUUGCUCACGGCCUGGCUGGUCCUGGCCGCAAGUGUCAUGUCCGAAGUCGCCGGAACCGUCGCGCUGAAGCAUUCGGCCGGUUUCACCAAGCCGCUGCCUGCGGUGCU
env-93     CGCACUGCCCG..U..GUCACUCGGUACAAGUGACAAACGGGC.AUGUCCGGCAGAGGUGCCUCAUGAAUUCAAGCCUGUGGGCCUGGCUGCUCAUUGCAGCCAGCGUCGUUGCCGAAGUUGUCGGUACGGUCGCGCUCAAGUACUCCGACGGCUUCACCAAACUCA.............
env-94     CGCACUACUCG..C..GUCACUCGGUACAAGUGACAAACGAGG.AAGUUCGGCAGAGGUGCCCAAUGAACUCAAGCCUAUUGGCCUGGCUGAUGAUCGCCGCCAGCGUUGUUGCAGAAGUGGUCGGAACCGUGGCGCUCAAGCACUCGGAUGGCUUCUCCAAGCUCCUCCCCUCAGGCAC
env-95     CGCACUACUCG..C..GUCACUCGGUACAAGUGACAAACGAGG.AAGUUCGGCAGAGGUGCCCAAUGAACUCAAGCCUAUUGGCCUGGCUGAUGAUCGCCGCCAGCGUUGUUGCAGAAGUGGUCGGAACCGUGGCGCUCAAGCACUCGGAUGGCUUCUCCAAGCUCCUCCCCUCAGGCAC
env-96     CGUACUGCCCG..U..GUCACUCGGUACAAGUGACAAACGGGC.AUGUUCGGCCAAGGUGCCCCAUGAAUCCAAACCUAUCGGCCUGGCUGCUCGUCGCAGCCAGCGUUAUUGCAGAAGUUGCCGGAACCGUCGCGCUCAAGUACUCCGAUGGCUUCUCCAGACUCCUCCCGUCCGGCAU
env-97     CGUACUGCCCG..U..GUCACUCGGUACAAGUGACAAACGGGC.AUGUUCGGCCAAGGUGCCCCAUGAAUCCAAACGUAUCGGCCUGGCUGCUCGUCGCAGCCAGCGUCAUUGCAGAAGUUGCCGGAACCGUCGCGCUCAAGUACUCCGAUGGCUUCUCCAGACUCCUCCCGUCCGGCAU
env-99     CGUACUGCCCG..U..GUCACUCGGUACAAGUGACAAACGGGC.AUGUUCGGCCAAGGUGCCCCAUGAAUCCAAACGUAUCGGCCUGGCUGCUCGUCGCAGCCAGCGUCAUUGCAGAAGUUGCCGGAACCGUCGCGCUCAAGUACUCCGAUGGCUUCUCCAGACUCCUCCCGUCCGGCAU
env-101    CGUACUGCCCG..U..GUCACUCGGUACAAGUGACAAACGGGC.AUGUUCGGCCAAGGUGCCCCAUGAAUCCAAACCUAUCGGCCUGGCUGCUCGUCGCAGCCAGCGUCAUUGCAGAAGUUGCCGGAACCGUCGCGCUCAAGUACUCCGAUGGCUUUUCCAGACUCCUCCCGU.......
Asp-9-1    CGUACUGCCCG..U..GUCACUCGGUACAAGUGACAAACGGGC.AUGUUCGGCCAAGGUGCCCCAUGUAUCCAAACCUAUCGGCCUGGCUGCUCGUCGCAGCCAGCGUCAUUGCAGAAGUUGCCGGAACCGUCGCGCUCAAGUACUCCGAUGGCUUCUCCAGACUCCUCCCGUCAGGCAU
env-102    CGUACUGCCCG..U..GUCACUCGGUACAAGUGACAAACGGGC.AUGUUCGGCCAAGGUGCCCCAUGAAUCCAAACCUAUCGGCCUGGCUGCUCGUCGCAGCCAGCGUCAUUGCAGAAGUUGCCGGAACCGUCGCGCUCAAGUACUCCGAUGGCUUCUCCAGACUCCUCCCGUCCGGUAU
env-103    CGUACUGCCCG..U..GUCACUCGGUACAAGUGACAAACGGGC.AUGUUCGGCCAAGGUGCCCCAUGAAUCCAAACCUAUCGGCCUGGCUGCUCGUCGCAGCCAGCGUCAUUGCAGAAGUUGCCGGAACCGUCGCGCUCAAGUACUCCGAUGGCUUCUCCAGACUCCUCCCGUCCGGUAU
env-104    CGUACUGCCCG..U..GUCACUCGGUACAAGUGACAAACGGGC.AUGUUCGGCCAAGGUGCCCCAUGAAUCCAAACCUAUCGGCCUGGCUGCUCGUCGCAGCCAGCGUCAUUGCAGAAGUUGCCGGAACCGUCGCGCUCAAGUACUCCGAUGGCUUCUCCAGACUCCUCCCGUCAGGCAU
env-106    CGUACUGCCCG..U..GUCACUCGGUACAAGUGACAAACGGGC.AUGUUCGGCCAAGGUGCCCCAUGAAUCCAAACCUAUCGGCCUGGCUGCUCGUCGCAGCCAGCGUCAUUGCAGAAGUU.................................................
env-107    CGUACUGCCCG..U..GUCACUCGGUACAAGUGACAAACGGGC.AUGUUCGGCCAAGGUGCCCCAUGAAUCCAAACCUAUCGGCCUGGCUGCUCGUCGCAGCCAGCGUCAUUGCAGAAGUUGCCGGAACCGUCGCGCUCAAGUACUCCGAUGGCUUCUCCAGACUCCUCCCGUCCGGCAU
env-110    CGUACUGCCCG..U..GUCACUCGGUACAAGUGACAAACGGGC.AUGUUCGGCCAAGGUGCCCCAUGAAUCCAAACCUAUCGGCCUGGCUGCUCGUCGCAGCCAGCGUCAUUGCAGAAGUU.................................................
env-111    CGUACUGCCCG..U..GUCACUCGGUACAAGUGACAAACGGGC.AUGUUCGGCCAAGGUGCCCCAUGAAUCCAAACCUAUCGGCCUGGCUGCUCGUCGCAGCCAGCGUCAUUGCAGAAGUUGUCGGAACCGUCGCGCUCAAGUACUCCGAUGGCUUCUCCAGACUCCUCCCGUCCGGCAU
env-112    CGUACUGCUCG..U..GUCACUCGGUACAAGUGACAAACGGGC.AUGUUCGGCCAAGGUGCCCCAUGAAUCCAAACCUAUCGGCCUGGCUGCUCGUCGCAGCCAGCGUCAUUGCAGAAGUUGCCGGAACCGUCGCGCUCAAGUACUCCGAUGGCUUCUCCAGACUCCUCCCGUCCGGCAU
env-113    CGUACUGCUCG..U..GUCACUCGGUACAAGUGACAAACGGGC.AUGUUCGGCCAAGGUGCCCCAUGAAUCCAAACCUAUCGGCCUGGCUGCUCGUCGCAGCCAGCGUCAUUGCAGAAGUUGCCGGAACCGUCGCGCUCAAGUACUCCGAUGGCUUCUCCAGACUCCUCCCGUCCGGCAU
Asp-5-1    CGUACUGCCCG..U..GUCACUCGGUACAAGUGACAAACGGGC.AUGUUCGGCAGAGGUGCCCCAUGAAUCCAAACCUAUCGGCCUGGCUGCUUGUCGCAGCCAGCGUCAUUGCAGAAGUUGUCGGAACGGUCGCGCUCAAGUACUCCGAUGGCUUUUCCAGACCCCUCCCAUCCGGCAU
Ara-1-1    CGUACUGCCCG..U..GUCACUCGGUACAAGUGACAAACGGGC.AUGUUCGGCAGAGGUGCCCCAUGAAUCCAAACCUAUCGGCCUGGCUGCUUGUCGCAGCCAGCGUCAUUGCAGAAGUUGUCGGAACGGUCGCGCUCAAGUGCUCCGAUGGCUUUUCCAGACCCCUCCCAUCCGGCAU
Asp-6-1    CGUACUGCCCG..U..GUCACUCGGUACAAGUGACAAACGGGC.AUGUUCGGCAGAGGUGCCCCAUGAAUCCAAACCUAUCGGCCUGGCUGCUUGUCGCAGCCAGCGUCAUUGCAAAAGUUGUCGGAACGGUCGCGCUCAAGUGCUCCGAUGGCUUUUCCAGACCCCUCCCAUCCGGCAU
env-114    CGUACUGCCCG..U..GUCACUCGGUACAAGUGACAAACGGGC.AUGUUCGGCAGAGGUGCCCCA...................................................................................................................
env-115    CGCACUGCUCG..U..GUCACUCGGUACAAGUGACAAGCGAGC.AUGUUCGGCAGAGGUGCCACAUGAACUCCAGCCUAUCGGCCUGGCUGCUCAUUGCAGUCAGCGUCAUCGCGGAAGUCGUCGGAACAGUCGCGCUCAAGCAUUCGGACGGCUUCUCCAGACUCUUACCCUCUGGUGC
env-117    CGCACUGCUCG..U..GUCACUCGGUACAAGUGACAACCGAGC.AUGUUCGGCAGAGGUGCCACAUGAACUCCAGCCUAUCGGCCUGGCUGCUCAUUGCAGUCAGCGUCAUCGCGGAAGUCGUCGGAACAGUCGCGCUCAAGCAUUCGGACGGCUUCUCCAGACUCUUACCCUCUGGUGC
env-118    CGCACUGCUCG..U..GUCACUCGGUACAAGUGACCAACGCGC.AAGUCCGGAAGAGGUGCCUCAUGAACCCAAGCCUGUCGGCCUGGCUGCUCGUCGCCAUCAGCGUCGUCGCAGAAGUUGUCGGAACCGUCGCGCUCAGAUACUCCGACGGCUUCUCCAGACUCCUCCCUUCUGGCGC
env-120    CGCACUGCUCG..U..GUCACUCGGUACAAGUGACAAACGAGC.AAGUACGGCAGAGGUGCCACAUGAACCCAAGCCUGCUGGCCUGGCUGCUCGUUGCAACCAGCGUCAUCGCAGAAGUUGUCGGAACGAUUGCGCUCAAGCACUCCGAUGGCUUCUCCAAACUCUUUCCAUCUGUCGC
env-125    CGCACUGCUCG..U..GUCACUCGGUACAAGUGACAAGCGAGC.AAGUACGGCAGAGGUGCCCCAUGAACCCAAGCCUGUUGGCCUGGCUGCUCGUUGCAACCAGCGUCAUCGCAGAAGUUGUCGGAACGGUUGCGCUCAAGCACUCCGAUGGCUUCUCCAAACUCUUCCCAUCUGCCGC
Psp-1-1    CGCACUGCUCG..U..GUCACUCGGUACAAGUGACAAACGGGC.AAGUUCGGCAGAGGUGCCCAAUGAAAUCAAGCCUGUUGGCCUGGCUGCUCAUCGCAGUCAGCGUCAUCGCAGAAGUCGUCGGAACAGUUGCGCUCAAGCACUCGGAUGGCUUUUCCAAACUCUUCCCUUCUGGAGC
env-126    CGCACUGCUCG..U..GUCACUCGGUACAAGUGACAAGCGAGC.AAGUUCGGCAGAGGUACCACAUGAACCCAAGCCUGUCAGCCUGGCUGCUCGUCGCAACCAGCGUCGUCGCAGAAGUUGUCGGAACGGUCGCGCUCAAGUACUCCGAU...........................
env-127    CGCACUGCUCG..U..GUCACUCGGUACAAGUGACAAGCGAGC.AAGUUCGGCAGAGGUGCCUCAUGAAUCCAAGCCUAUCGGCCUGGCUGCUCGUCGCAGCGAGCGUCAUCGCAGAAGUUGUCGGAACGGUCGCGCUCAAGUACUCCGAUGGCUUCAGAAGUGGCUCGGUUCGUCUGAA
env-128    CGCACUGCUCG..U..GUCACUCGGUACAAGUGACAAGCGAGC.AAGUUCGGCAGAGGUGCCACAUGAACCCAAGCCUGUCGGGCUGGCUGCUCGUCGCAACCAG...................................................................
env-129    CGCACUGCUCG..U..GUCACUCGGUACAAGUGACAAGCGAGC.AAGUUCGGCACAGGUGCCUCAUGAAUCCAAGCCUAUCGGCCUGGCUGCUCGUCACAGCGAGCGUCAUCGCAGAAGUUGUCGGAACGGUCGCGCUCAAGUACUCCGAUGGCUUCUUCAAACUCCUCCCAUCUGCCAC
Hfl-1-1    CGCACUGCCCG..CUGGUCACUCGGUACAAGUGACCAGCGGGU.GGGUUCGACAGGGGUAUUUCAUGAAUGUAAGUCUCACGGGGUGGUGGUUCGUCGCGGUCAGCGUCGUCGCAGAGGUGGCCGGAACAGUGGCGCUCAAGCACUCGGACGGGAUGACCCGUCCUCUCGCAGCGGGCGC
Hsp-1-1    CCAACUGUUGG..C..CCCACUCGGUACAAGUGGCCGGCUGGC.AUGUUCGGUCGAGGUGCGCAUGAAAUCAGAGCUGAUGGCUUGGGUGGUGCUUUCUGCCAGUGUGGUCUCUGAGGUGGCAGGUACCGUGGCGCUCAGGUACUCCAGCGGCUUCACCAAGCUCUUGCCCACGGCCCUU
env-130    GUCAAUGCUCG..C..UCCACUCGGUACAAGUGGAAUGCGGGC.AUGGUUGCCGGAGGUAUCCAUGAAUUCCAGCGCAUUUGCCUGGUUUUUGCUCGGGAUCAGCGUCAUAUCGGAAGUUGCAGGAACCGUGGCGCUCAAGCAUUCGGAUGGUUUUUCCAGGUUCGCCCCCGCGGCCGCG
env-131    AACACUGCUCG..A..GCCGCUCGGUACAAGCGGCCGCUGAGC.A.CCUUGGAGUAGGUACCCAUGAGUCCCCUUCUCGCGGCCUGGCUGCUGCUUGCCGUCAGUGUCGCCUGUGAAGUCGUCGGCAACGUCGCCCUGCGAUACGCCGACGGAUUCACCAGACCGAUCGCGGCGACUGCU
env-132    AACACUGCUCG..A..GCCGCUCGGUACAAGCGGCCGCUGAGC.A.UCUUGGAGUAGGUACCCAUGAGUCCCCUUCUCGCGGCCUGGCUGCUGCUUGCCGUCAGUGUCGCCUGUGAAGUCGUCGGCAACGUCGCCCUGCGAUACGCCGACGGAUUCACCAGACCGAUCGCGGCGACUGCU
env-133    AACUUCGCGCUCCC..ACCACUCGGUACAAGUGGC..GUGCGC.GUAUUUAGUUGAGGUAUUUAUGAGCCCCAUCCUUGUCGCAUGGCUGCUGCUUGCCGCCAGCAUACUUUCUGAAGUUGUCGGCACCGUCGCCCUAAAACACUCCAACGGUUUUGCCAAUAUCUUACCCACCACCUGC
env-134    AACUUCGCGCUCCC..ACCACUCGGUACAAGUGGC..GUGCGC.GUAUUUAGUUGAGGUAUUUAUGAGCCCCAUCCUUGUCGCAUGGCUGCUGCUUGCCGCCAGCAUACUUUCUGAAGUUGUCGGCACCGUCGCCCUAAAACACUCCAACGGUUUUGCCAAUAUCUUACCCACCACCUGC
env-135    AACUUCGCGCUCCC..ACCACUCGGUACAAGUGGC..GUGCGC.GUAUUUAGUUGAGGUAUUUAUGAGCCCCAUCCUUGUCGCAUGGCUGCUGCUUGCCGCCAGCAUACUUUCUGAAGUUGUCGGCACCGUCGCCCUAAAACACUCCAACGGUUUUGCCAAUAUCUUACCCACCACCUGC
env-136    AACUUCGCGCUCCC..ACCACUCGGUACAAGUGGC..GUGCGC.GUAUUUAGUUGAGGUAUUUAUGAGCCCCAUCCUUGUCGCAUGGCUGCUGCUUGCAGCCAGCAUUCUUUCUGAAGUUGUCGGCACCGUCGCACUAAAGCACUCCAACGGUUUUGCCAAUCUGUUACCCACCACCUGC
Bte-1-1    CCGAAAAUCUG..C..AGCGCUCGGUACAAGCGC...GCAAUC.U.GGAGCGGCAUUCCAUGGACGCCACAUCUCCCCUCCUUAGCUGGUUGUUUGUUGCCGCCAGCGUCGUCGCCGAAGUCGCCGGCACUGUUGGCCUUCGAUAUGCCGACGGAUUCACUCGACCGCUUCCCUCAAUGA
Bce-5-1    UCCACUUCUCC.UC..GCCGCUCGGUAUAAGCGUC..GGGGGA.A.GGAGGCCGUUCGUGUCCCCGCUCCAGUCUGCCUGGCUUCUGCUGGGCGUCAGUGUCCUCGCCGAAGUGCUCGGCUCCGUCGGCCUCAAAUACUCGGCCGGCUUUUCCCGCCCGCUCCCGUCCGCGCUCACCGUC
           ...<<<<<<<<..<..<<<<<<.......>>>>>>..>>>>>>.>.>>......................................................................................................................................
           ...2222.222..2..221222.......222122..2222.2.2.22......................................................................................................................................
           .......................>>>>>>.........................................................................................................................................................
           .......................00000..........................................................................................................................................................
           Y●●●Y●Y●Y●--Y--●●CRYUCGGUAYAARYG●●--G●R●●●-R-●○●RR●●●●●●●●●●●●●●●●●●●●●●●●●●●●●●●●●●●●●●●YY●●●●●YY●●●Y●●●●●●●●●●●●●Y●●●●●●●●●●●●●●●●●●●●●●●●●●●●●●●●●●●●●●●●●●●●●●●●●●●●●●●●●●Y●●●●●●●●●●●●Y●●YY●●●●●●●●●●●
```

alignment positions 361 · · · 540

```
....................................................................................................................................................................................
....................................................................................................................................................................................
....................................................................................................................................................................................
....................................................................................................................................................................................
●●Y●●●●●●●●●●●●●●●●●●●●●●●●●●●●●●●●●●●●●●●●●●●●●●●●●●○○○○○○○○○○○○○○○○○○○○○○○○○○○○○○○○○○○○○○○○○○○○○○○○○○○○○○○○○○○○○○○○○○○○○○○○○○○○○○○○○○○○○○○○○○○○○○○○○○○○○○○○○○○○○○○○○○
                                                                                                                                                                              3′
```

```
env-1     UGGGGCCGCCAGUUGCUUUUUAAUGGCGAUCUGGCUGAUGUCGCUGUCACUCAAAUAUAUUGAAAUGGGCAUCACCUAUGCAGUGUGGGCAGGCAGCGCCACGGCCAUUCUGGCGCUGGUAGGCAUUGCGUUUUAUAGUGAGUCCCUCAGCGUGAUCAAACUGGUCGGCAUGGUGAUGGU
Ama-1-1   GGUCUUUGCUACACCGCAGCCAUCUGGCUUAUGGCUGUAUCUGUGAGAAAUCUUGAAGUCGGCUUGGCUUAUGCCGUGUGGGCGGGCAGUGGCACCGCCGUAACCGCACUGCUCGGAAUCAUCUUGUUUGGAGAGUCAGCCAGCCUACUUCGGCUCUCUGGCUUGGGACUCAUCAUUUCU
Ama-1-2   GGUCUUUGCUACACCGCAGCCAUCUGGCUUAUGGCUGUAUCUGUGCGGAAUCUUGCAGUCGGCUUGGCUUAUGCCGUGUGGGCGGGCAGUGGUACCGCCGUAACCGCACUGCUCGGAAUCAUUUUGUUUGAAGAGUCAGCCAGCCUACUUCGGCUCUCUGGCUUGGGACUCAUCAUUUCU
env-2     AUUGCCUGCUACCUCAGCGCGAUCUGGCUGAUGGCGCUUUCCACCAAACAACUGGAAAUUGGCCUCGCUUACGCCGUGUGGGCUGGUGCCUGCACCGCGUUGACGGCGGUGGUGGGGAUUACCUGGUUUGGCGAAUCCGUGUCAUCGGUCAAAUUGCUGGGUCUAGGCCUUGCGAUUGCC
Bsp-17-1  CUUGCUAUACGGGUGCAGUCUGGCUCAUGGCGAUCUCCACCAAACAUAUUGAGGUCGGGAUGGCCUAUGCGGUGUGGGCAGGAGCCAGCACCGCGCUGACUGCUCUUAUCGGCAUCGCGUGGUUUGGUGAAUCCAUCACCACCCUCAAACUGCUUGGUCUCGCCUUGGCCGUCGUUAGCC
env-3     CUUGCUAUACGGGUGCAGUCUGGCUCAUGGCGAUCUCCACCAAACAUAUUGAGGUCGGGAUGGCCUAUGCGGUGUGGGCAGGAGCCAGCACCGCGCUGACUGCUCUUAUCGGCAUCGCGUGGUUUGGUGAAUCCAUCACCACCCUCAAACUGCUUGGUCUCGCCUUGGCCGUCGUUAGCC
Pfu-2-1   CUUGCUAUACGGGUGCAGUCUGGCUCAUGGCGAUCUCCACCAAACAUAUUGAGAUCGGGAUGGCCUAUGCGGUGUGGGCAGGAGCCAGCACCGCGCUGACUGCUCUUAUCGGCAUCGCGUGGUUUGGUGAAUCCAUCACCACCCUCAAACUGCUUGGUCUCGCCUUGGCCGUCGUUAGCC
Pph-1-1   CUUGCUAUACGGGGGCAGUGUGGCUCAUGGCGAUCUCCACCAAACAUAUUGAGAUCGGUAUGGCCUAUGCCGUGUGGGCUGGAGCCAGCACCGCGCUGACUGCUCUUGCCGGCAUCACGUGGUUUGGUGAAUCCAUCACCACCCUCAAACUGCUUGGUCUCGCCUUGGCUGUCGUUAGCC
Bce-1-1   UUUGCUACGCCGGCGCCGUUUGGCUCAUGUCCAUUACGACAUUAAACGUCGAGGUGGGUCUGGCCUACGCCGCGUGGGCCGGCGCCAGUACCGCCAUCACCGCCGGACUCGGAAUAUUGCUCUUCGGCGAAUCUGUUUCGGUAUCGAAGCUCUGUGGCCUCGCGAUGGCUGCAGGUAGCC
Rgu-1-1   CCUGCUAUGCCGGCGCCAUCUGGUGGAUGGCCGUCGCCGUGCGGCACCUGGAGGUCGGGUUGACCUAUGCGAUCUGGGCGGGUUGCGGGACGGCGUUGACGGCGCUGGUCGGCAUCGUGUGGUUCGGGGAGUCGGCGGGGAUGUUGCGCCUCGCUGGCCUGGCGAUGAUCGUGGGUGGGG
Hta-1-1   CCUCGUAUGCGGCGGCCAUUUGGCUCAUGUCCUUGGCCGUGAGGCACUUGGAAGUCGGGUUGGCGUAUGCGGUGUGGGCGGGAAGCGGUACCGCGUUGACGGCAGCCCUCGGCAUCCUCUGGUUCUCCGAGUCGAUGACGCUCUUGCGGAUUGCGGGAGUCGUCAUGAUCGUCAUUGGCG
Vpa-1-1   UCUCCUAUGCAAUCGCAAUUUGGCUUAUGGCGAUUGCGGUUAGGCACUUGGAAAUAGGAAUGGCGUAUGCAAUUUGGGCCGGUGGCGGAGCCGCUCUGAUCGCGCUGACCGGUGUCGUCUGGUUCGGCGAAUCCAUGACGCCCUCACGAGCCAUUGGCGUCACCAUGAUCAUCAUCGGGG
Aur-1-1   CGCUGUGCUACAUCCUGGCGAUCUGGCUGAUGGCGCUGGCCACCAAACGUCUGGAAAUGAGCCUGGCCUACGCCGUGUGGGCGGGCGGCAGCGCCGCGAUGACCACGCUGAUUGGCGUGGUCUGCUUUGGCGACAGCAUGGGCGGGCUGAAGGCGCUGGGGCUGUGCACCACCAUUGCGG
env-6     UGACCUGCUACGCCUGCGCCAUCUGGCUGAUGUCUGUCGCAGUCAAACGUCUCGAAAUGGGCUUGACCUACGCAGUGUGGGCCGGCGCCGGAACGGCGCUCACGGCGAUACUUGGCAUGAUCUGGUUUGGGGAAACAAGCUCGGCGCUGCGUUUUGCGGGCCUCGGCUGUAUUGUGGUCG
Vap-1-1   UCCUGUCGUAUGGUGUGGCGAUCUGGCUCAUGUCCCUCGCGGUGAGACAGCUCGAAGUGGGUCUUACCUACGCCGUGUGGGCCGGCAGCGGGACAGCGCUCAUCGCGCUGAUCGGGAUCCUCUGCUUCAAUGAGUCGGCCCAUGCCAUGCGCUUGCUGGGCUUCGUCUUCAUCGUGCUGG
Rsp-2-1   GCGCUUGUUAUGCGGGCGCCAUCUGGCUCAUGGCGAUCGCGGUGCGCCACCUCGAGAUGGGGCUGACCUACGCGGUGUGGGCGGGCAGUGGCACGGCGCUCACCGCAUUGCUGGGCAUCCUGUGGUUCGGUGAAUCCGCGAGUGCACUGCGCCUUGCCGGGUUGGGUUCAUCGUUGUCG
Bce-1-2   UCGUUUGCUACGCCGGCGCCGUUUGGCUCAUGUCCAUUACGACAUUAAACGUCGAGGUGGGUCUGGCCUACGCCGCGUGGGCCGGCGCCAGUACCGCCAUCACCGCCGGACUCGGAAUAUUGCUCUUCGGCGAAUCUGUUUCGGUAUCGAAGCUCUGUGGCCUCGCGAUGGCUGCAGGUA
Hsp-3-1   UAGCUAUGCAAGUGCCAUUUGGCUCAUGUCAGUAUCUGUUCGCCACCUUGAAGCUGGGUUGGCAUACGCAGUGUGGGCAGGAUGCGGAACGGCACUAAUUGCCCUCCUAGGAAUUUUCUGCUUCGGAGAAUCAACGGCACCGCUUCGAUUGGUAGGUAUUGCAUUAAUCAUUAGCGGAGU
env-7     UAGUUAUGCAAGCGCUAUUUGGCUCAUGUCAGUAUCUGUUCGCCACCUUGAAGCUGGAUUGGCAUACGCAGUGUGGGCAGGAUGUGGAACGGCGCUAAUUGCCCUCAUAGGAAUUUUCUGUUUCGGAGAAUCAACGGCGCCACUUCGAUUGGUAGGUAUUGCAUUAAUCAUUAGCGGAGU
Hsp-2-1   UAGUUAUGCAAGCGCUAUUUGGAUCAUGUCAGUAUCUGUUCGCCACCUUGAAGCUGGAUUGGCAUACGCAGUGUGGGCAGGAUGUGGAACGGCGCUAAUUGCCCUCAUAGGAAUUUUCUGUUUCGGAGAAUCAACGGCGCCACUUCGAUUGGUAGGUAUUGCAUUAAUCAUUAGCGGAGU
env-8     GUUGUAUGGCGCAGCAAUUUGGCUCAUGUCCCUGGCGGUGAGGCGCCUCGCAAUUGGUCUCGCAUAUGCCGUGUGGGCUGGCAGCAGCACGGCGCUCAUUGCACUGGUGGGGAUACUCUACUUCAAUGAGUCGGCCCAUGCUGUACGCCUACUUGGUCUCGCCUUCAUCGUGAUAGGGGU
env-9     CUUCUACGUAGCUGCUGUUGGCUUAAUGAGUAUCGCAGCUAAGUCAAUUGAUAUAAGCACUCUUUACCCAAUAUGGGCCGCAGCCAGCACUGUGCUCAUAUCUUUGAGUGGAUGCGUUCUCUUCAGCGAAUCCAUAACAUCUAACAAAGCGCUGGGCAUGCUAUUUCCGUCUCAGGUAU
Asp-4-1   GCCCUCGGUGGCCGCUGGGGCCUGCUACGUGCUGGCGGUGUGGUGGAUGUCACUGGCCAUGAAGCACAUCGGCAUGGGCACCACCUAUGCGGUGUGGGCAGGCAGCGGACUGGCGUUGACGGCGCUGCUGGGGAUGGCGCUGUUUGGUGAGCCGCUCUCGGGCAUUCGGUUCGCCGGACU
Asp-3-1   GCCCUCGGUGGCCGCUGGGGCCUGCUACGUGCUGGCGGUGUGGUGGAUGUCACUGGCCAUGAAGCACAUCGGCAUGGGCACCACCUAUGCGGUGUGGGCAGGCAGCGGACUGGCGUUGACGGCGCUGCUGGGGAUGGCGCUGUUUGGUGAGCCGCUCUCGGGCAUUCGGUUCGCCGGACU
env-10    GGUCUGCUACGCCAGUGCCAUCUGGCUGAUGUCGGUCGCCGUCAAACACCUUGAAAUGGGCUUGGCGUAUGCCGUAUGGGCCGGUGCCGGUACCGCAGUCACGGCGAUCUUGGGCAUGCUCUGGUUUGAUGAGGCGCGCUCGGCCUUGCGGUUUGCGGGCCUGGGGUGCAUCGUGCUUGG
Awa-1-1   GAUCUGCUAUGGCAGCGCUAUUUGGUUGAUGUCCCUUUCGAUCAAGCAUCUUGAAAUGGGCCUUACCUAUGCCGUAUGGGCUGGCUGCGGCACUGCUCUCACGGCGGCCUUGGGCAUUAUCUGGUUCGACGAAUCCUCCGGGCCCCUCAGGCUGGCAGGCCUGGGCUGCAUCGUUCUGGG
Ama-1-3   CCUUUGUUAUGCAAGUGCCAUUUGGCUCAUGUCCGUAUCCGUUCGCCAUCUUGAAGUCGGCUUGGCAUAUGCGGUAUGGGCCGGAUUCGGAACGGUACUCAUUGCCCUUCUGGGAAUUCUCUGCUUCGGAGAGUCAACGGCACCGCUUCGACUGAUUGGCAUUGCCUUAAUCGUUGGCGG
env-11    GGCCUCGUAUGCGGCGGCCAUUUGGCUCAUGUCCAUGGCCGUGAGGCACUUGGAAGUCGGGUUGGCGUAUGCGGUGUGGGCGGGAAGCGGCACCGCGUUGACGGCAGCCCUCGGCAUCCUCUGGUUCUCCGAGUCGAUGACGCUCUUGCGGAUUGCGGGAGUCGUCAUGAUCGUCAUUGG
env-15    GGCCUCGUAUGCGGCGGCCAUUUGGCUCAUGUCCAUGGCCGUGAGGCACUUGGAAGUCGGGUUGGCGUAUGCGGUGUGGGCGGGAAGCGGCACCGCGUUGACGGCAGCCCUCGGCAUCCUCUGGUUCUCCGAGUCGAUGACGCUCUUGCGGAUUGCGGGAGUCGUCAUGAUCGUCAUUGG
env-16    GGCCUCGUAUGCGGCGGCCAUUUGGCUCAUGUCCAUGGCCGUGAGGCACUUGGAAGUCGGGUUGGCGUAUGCGGUGUGGGCGGGAAGCGGCACCGCGUUGACGGCAGCCCUCGGCAUCCUCUGGUUCUCCGAGUCGAUGACGCUCUUGCGGAUUGCGGGAGUCGUCAUGAUCGUCAUUGG
env-18    GGCCUCGUAUGCGGCGGCCAUUUGGCUCAUGUCCAUGGCCGUGAGGCACUUGGAAGUCGGGUUGGCGUAUGCGGUGUGGGCGGGAAGCGGCACCGCGUUGACGGCAGCCCUCGGCAUCCUCUGGUUCUCCGAGUCGAUGACGCUCUUGCGGAUUGCGGG......................
env-19    GGCCUCGUAUGCGGCGGCCAUUUGGCUCAUGUCCAUGGCCGUGAAG.............................................................................................................................
env-20    GGCCUCGUAUGCGGCGGCCAUUUGGCUCAUGUCCAUGGCCGUGAAG.............................................................................................................................
env-21    GGCCUCGUAUGCGGCGGCCAUUUGGCUCAUGUCCAUGGCCGUGAAG...................................................................................................................CACUUGGAAGUC
env-22    ...........................................................................................................................................................................................
Bsp-20-1  CGCCUGCUAUGCCGGCGCCGUCUGGCUCAUGUCCAUCGCAACGUUGCACGUCGAGGUCGGCCUCGCCUACGCGGUCUGGGCCGGCGCGAGUACGGCCGCCACUGCCGGGCUCGGGAUCCUGUUCUUCGGCGAGUCGCUUUCGGCGCUCAAGCUUUGCGGCCUCGCGAUGGCCGCGGGGAG
Bpa-1-1   CUGCUAUGCCGGCGCCGUCUGGCUCAUGUCCAUCGCAACGUUGCACGUCGAGGUCGGCCUCGCCUACGCGGUCUGGGCCGGCGCGAGUACGGCCGCCACUGCCGGGCUCGGGAUCCUGUUCUUCGGCGAGUCGCUUUCGGCGCUCAAGCUUUGCGGCCUCGCGAUGGCCGCGGGGAGCCU
Bsp-8-1   CGUCUGCUAUGCAUGUGCGGUAUGGCUGAUGGCGCUCGCGACGAAGCGAAUCGAAAUGGGGCUCGCGUAUGCGAGCUGGGCCGCUGCCAGCACGGCGGCCACGGCCAUGAUCGGCAUCGUCUGCUUCGACGAAUCGGUGUCGUCGCUGAAGCUCGUCGGCAUCGCGCUGACCGUCUGCGC
Pac-1-1   CGUGUGCUAUGCGACUGCCGUCUGGCUCAUGGCGCUCGCGACAAAGCGAAUCGAAAUGGGCAUGGCGUAUGCGGCCUGGGCGGGCAGUAGUGCUGCCUUGACAGCGGUGAUCGGAAUCGUGUGGUUCGGGGAAGCGCUGAGUCUCGUCAAAGCAAUCGGAAUCGGUACCGCCAUAUGCGG
Pme-1-1   CGCGUGCUAUGCGACCGCAGUCUGGCUCAUGGCGCUAGCAACAAAGCGAAUCGAAAUGGGCAUGGCGUAUGCGGCCUGGGCGGGCAGUAGUGCUGCGUUGACCGCGGCGAUCGGGAUUAUCUGGUUCGGCGAGGCCCUGAGCCUUGUCAAGACAAUCGGAAUCGGCACCGCGAUAUGCGG
env-23    CGCGUGCUAUGCCGUGGCCAUCUAUCUGAUGGCGGUCGCGACGAAAAGCAUCGAAAUGGGCAUGGCUUACGCCGCGUGGGCCGGCAGCAGCGCGGCCCUCACGGCGGUCAUCGGCAU...........................................................................
Ptr-1-1   CGCGUGCUAUGCCGUGGCCAUCUAUCUGAUGGCGGUCGCGACGAAAAGCAUCGAAAUGGGCAUGGCUUACGCUGCGUGGGCCGGCAGCAGCGCGGCCCUCACGGCGAUCAUCGGCAUGCUGUGGUUUGGCGAAUCGGUGAGCGCCGUCAAAGCCAUCGGGCUCGUGACGGCGAUUUCGGG
Ptr-1-3   CGCGUGCUAUGCCGUGGCCAUCUAUCUGAUGGCGGUCGCGACGAAAAGCAUCGAAAUGGGCAUGGCUUACGCCGCGUGGGCCGGCAGCAGCGCGGCCCUCACGGCGAUCAUCGGCAUGCUGUGGUUUGGCGAAUCGGUGAGCGCCGUCAAAGCCAUCGGGCUCGUGACGGCGAUUUCGGG
Bub-1-1   GACGUUCUACGUGGCAGCUGUCAUUCUGAUGGCCGUCGCGACGAAGAGAAUCGAAAUGAGUUUGGCAUACGCAGCCUGGGCUGGAAGCAGCGUCGUGCUGACGGUAGUGAUAGGUUUCGUCUGGUUUGGAGAGUCGAUGAACGUCGUUAAGGCGAUUGGAUUGAUCGCUGCGAUAUCCGG
Bsp-24-1  GACGUUCUACGUGGCAGCUGUCAUUCUGAUGGCCGUCGCGACGAAGAGAAUCGAAAUGAGUUUGGCAUACGCAGCCUGGGCUGGAAGCAGCGCCGUGCUGACGGUAGUGAUAGGUUUCGUCUGGUUUGGAGAGUCGAUGAACGUCGUUAAGGCGAUUGGAUUGAUCGCUGCGAUAUCCGG
Bvi-3-1   GACGUUCUACGUGGCAGCUGUCAUUCUGAUGGCCGUCGCGACGAAGAGAAUCGAAAUGAGUUUGGCAUACGCAGCCUGGGCUGGAAGCAGCGCCGUGCUGACGGUAGUGAUAGGUUUCGUCUGGUUUGGAGAGUCGAUGAACGUCGUUAAGGCGAUUGGAUUGAUCGCUGCGAUAUCCGG
Bvi-1-3   GACGUUCUACGUGGCAGCUGUCAUUCUGAUGGCCGUCGCGACGAAGAGAAUCGAAAUGAGUUUGGCAUACGCAGCCUGGGCUGGAAGCAGCGCCGUGCUGACGGUAGUGAUAGGUUUCGUCUGGUUUGGAGAGUCGAUGAACGUCGUUAAGGCGAUUGGAUUGAUCGCUGCGAUAUCCGG
Bsp-18-1  GACGUUCUACGUGGCAGCUGUCAUUCUGAUGGCCGUCGCGACGAAGAGAAUCGAAAUGAGUUUGGCAUACGCAGCCUGGGCUGGAAGCAGCGCUGUGCUGACCGUAGUGAUAGGUUUCGUCUGGUUUGGAGAGUCGAUGAACGUCGUUAAGGCGAUUGGAUUGAUCGCUGCGAUAUCCGG
env-24    GAUCUGCUACGUCACGGCCGUCUUUCUGAUGGCUGUCGCAACGAAGCGAAUCGAAAUGGGUAUGGCCUACGCGGCCUGGGCGGGAAGCAGUGCUGCCUUGACGGCAGUGAUAGGUGUGGUCUGGUUCGAGGAAUCGCUCAACGCCCUCAAGGCCAUGGGGUUGUGCAUGGCCAUAUGCGG
Pfe-1-1   GAUCUGCUACGUCACGGCCGUCUUUCUGAUGGCUGUCGCAACGAAGCGAAUCGAAAUGGGUAUGGCCUACGCGGCCUGGGCGGGAAGCAGUGCUGCCUUGACGGCAGUGAUAGGUGUGGUCUGGUUCGAGGAAUCGCUCAACGCCCUCAAGGCCAUGGGGUUGUGCAUGGCCAUAUGCGG
env-25    GAUCUGCUACGUCACGGCCGUCUUUCUGAUGGCUGUCGCAACGAAGCGAAUCGAAAUGGGUAUGGCCUACGCGGCCUGGGCGGGAAGCAGUGCUGCCUUGACGGCAGUGAUAGGUGUGGUCUGGUUCGAGGAAUCGCUCAACGCCCUCAAGGCCAUGGGGUUGUGCAUGGCCAUAUGCGG
env-26    CACCUUUUACAUCGCCGCCGUCUGGUUAAUGUCGCUGGCUACCAAACGCCUUGAAAUGAGCACGGCAUACGCCGCAUGGGCAGGCACCAGUGCGGCUCUAACCGCAUUCAUUGGCAUUACGUGCUUUGGUGAGGCCAUUGCCCCUCUGAAACUGAUCGGGCUAUCCAUGGCGAUCUGCGG
Bsp-22-1  CGUCUGCUACAUGGCCGCCGUGUGGCUGAUGGCGCUUGCCACCAAACGGCUUGAAAUGGGCACGGCAUACGCUGCAUGGGCAGGUACAAGCGCGGCGCUAACCGCGUUGAUUGGAAUCGUGUGUUUCGCCGAGACCAUUUCACCGCUCAAACUGAUUGGUCUGGGCACGGCGGUGCUCGG
env-27    GGUGCUGUGUUACG.............................................................................................................................................................................
env-28    UGCUAUACGGGUGCAGUGUGGCUCAUGGCGAUCUCGACUAAACAUAUCGAGAUCGGCAUGGCCUAUGCCGUGUGGGCCGGAGCUAGCACAGCCCUGACCGCUCUUGCCGGCAUCGCGUGGUUUGGCGAAUCCGUCACCGCCCUCAAGCUGCUGGGUCUAGCCUUGGCCGUCGUUAGCCUG
env-29    CUGUCGUAUGACGCGACCAUCUGGCUGAUGUCCCUGGCGGUCGGGCAGCUUGAAGUGGGCCUCACCUAUGCAGUGUGGGCCGGUAGCGGCACGGCACUCACGGCCUUGGUCGGGAUGUUGCAUUUCAAUGAGUCGGCGCAUGCCCUGCGCUUGCCGGGCUUUGCCUUCAUCGUGGCCGGU
env-30    CUUUGUUAUUCAGCCGCUAUCUGGCUUAUGGCAAUUGCCGUCCGGCAUAUGGAAGUCGGACUUACCUAUGCGGUAUGGGCGGGAAGCGGAACAGCUUUGACGGCGGUGCUCGGAAUGUUCUGGUUUGGUGAGUCGGCGAGCUUCCUUCGUCUGGCCGGUCUAAGCUUCAUUGUUUUGGGC
env-31    ...........................................................................................................................................................................................
env-32    CUUUGUUAUUCCGCCGCUAUCUGGCUUAUUGCAAUCGCCGUCCGGCAUAUGGAAGUCGGACUUACCUAUGCGGUAUGGGCGGGAA......................................................................................
env-33    CUUUGUUAUUCCGCCGCUAUCUGGCUUAUGGCAAUCGCCGUCCGGCAUAUGGAAGUCGGACUUACCUAUGCGGUAUGGGCGGGAA...........................................................................GCGGAACAGCUU
env-34    CUUUGUUAUUCCGCCGCUAUCUGGCUUAUGGCAAUCGCCGUCCGGCAUAUGGAAGUCGGACUUACCUAUGCGGUAUGGGCGGGAA...........................................................................GCGGAACAGCUU
env-35    CUUUGUUAUUCCGCCGCUAUCUGGCUUAUGGCAAUCGCCGUCCGGCAUAUGGAAGUCGGACUUACCUAUGCGGUAUGGGCGGGAA...........................................................................GCGGAACAGCUU
env-36    CUUUGUUAUUCCGCCGCUAUCUGGCUUAUGGCAAUCGCCGUCCGGCAUAUGGAAGUCGGACUUACCUAUGCGGUAUGGGCGGGAA...........................................................................GCGGAACAGCUU
env-37    CUUUGUUAUUCCGCCGCUAUCUGGCUU................................................................................................................................................................
env-38    CUUUGUUAUUC................................................................................................................................................................................
env-39    CUUUGUUAUUC................................................................................................................................................................CGCCGCUAUCUG
env-40    ...........................................................................................................................................................................................
env-41    ...........................................................................................................................................................................................
env-42    .........................................................................................................................................................................UGGCGGCAAGCG
env-44    .........................................................................................................................................................................UGGCGGCAAGCG
env-45    .........................................................................................................................................................................UGGCGGCAAGCG
env-46    .........................................................................................................................................................................UGGCGGCAAGCG
env-47    CUUUGUUAUUCCGCCGCUAUCUGGCUUAUGGCAAUCGCCGUCCGGCAUAUGGAAGUCGGACUUACCUAUGCGGUAUGGGCGGGAA......................................................................................
env-48    CUUUGUUAUUC................................................................................................................................................................CGCCGCUAUCUG
env-49    .........................................................................................................................................................................UGGCGGCAAGCG
env-50    .........................................................................................................................................................................UGGCGGCAAGCG
env-51    .........................................................................................................................................................................UGGCGGCAAGCG
env-52    .........................................................................................................................................................................UGGCGGCAAGCG
env-53    CUUUGUUAUUCCGCCGCUAUCUGGCUUAUGGCAAUCGCCGUCCGGCAUAUGGAAGUCGGACUUACCUAUGCGGUAUGGGCGGGAA...........................................................................GCGGAACAGCUU
env-61    ...........................................................................................................................................................................................
env-62    CUUUGUUAUUCCGCCGCUAUCUGGCUUAUGGCAAUCGCCGUCCGGCAUAUGGAAGU...................................................................................................................

          ...........................................................................................................................................................................................
          ...........................................................................................................................................................................................
          ...........................................................................................................................................................................................
          ...........................................................................................................................................................................................
                                                                                                                                                                                       3′
```

env-63	CUUUGUUAUUCCGCCGCUAUCUGGCUUAUGGCAAUCGCCGUCCGGCAUAUGGAAGUCGGACUUACCUAUGCGGUAUGGGCGGGAAGCGGAACAGCUUUGACGGCGGUGCUCGGAAUGCUCUGGUUUGGUGAGUCGGCGAGCUUCCUUCGACUGUCCGGUCUUAGCCUCAUUGUUUUGGGC
env-64	CUUUGUUAUUCCGCCGCUAUCUGGCUUAUGGCAAUCGCCGUCCGGCAUAUGGAAGUCGGACUUACCUAUGCGGUAUGGGCGGGAAGCGGAACAGCUUUGACGGCGGUGCUCGGAAUGCUCUGGUUUGGUGAGUCGGCGAGCUUCCUUCGACUGU..........................
env-65	CUUUGUUAUUCCGCCGCUAUCUGGCUUAUGGCAAUCGCCGUCCGGCAUAUGGAAGUCGGACUUACCUAUGCGGUAUGGGCGGGAAGCGGAACAGCUUUGACGGCGGUGCUCGGAAUGCUCUGGUUUGGUGAGUCGGCGAGCUUCCUUCGACUGUCCGGUCUUAGCCUCAUUGUUUUGGGC
env-66	CUUUGUUAUUCCGCCGCUAUCUGGCUUAUGGCAAUCGCCGUCCGGCAUAUGGAAGUCGGACUUACCUAUGCGGUAUGGGCGGGAAGCGGAACAGCUUUGACGGCGGUGCUCGGAAUGCUCUGGUUUGGUGAGUCGGCGAGCUUCCUUCGACUGUCCGGUCUUAGCCUCAUUGUUUUGGGC
env-70	CUUUGUUAUUCCGCCGCUAUCUGGCUUAUGGCAAUCGCCGUCCGGCAUAUGGAAGU..CGGACUUACCUA
Asp-8-1	CUUUGUUAUUCCGCCGCUAUCUGGCUUAUGGCAAUCGCCGUCCGGCAUAUGGAAGUCGGACUUACCUAUGCGGUAUGGGCGGCAAGCGGAACAGCUUUGACGGCGGUGCUCGGAAUGCUCUGGUUUGGUGAGUCGGCGAGCUUCCUUCGACUGUCCGGUCUUAGCCUCAUUGUUUUGGGC
env-71	CUUUGUUAUUCCGCCGCUAUCUGGCUUAUGGCAAUCGUCGUCCGGCAUAUGGAAGUCGGACUUACCUAUGCGGUAUGGGCGGGAAGCGGAACAGCUUUGACGGCGGUGCUCGGAAUGAUCUGGUUUGGUGAGUCGGCGAGCUUCCUUCGACUGGCCGGUCUAAGCUUCAUUGUUUUGGGC
env-72	CUUUGUUAUUCCGCCGCUAUCUGGCUUAUGGCAAUCGUCGUCCGGCAUAUGGAAGUCGGACUUACCUAUGCGGUAUGGGCGGGAAGCGGAACAGCUUUGACGGCGGUGCUCGGAAUGAUCUGGUUUGGUGAGUCGGCGAGCUUCCUUCGACUGGCCGGUCUAAGCUUCAUUGUUUUGGGC
env-73	CUUUGUUAUUCCGCCGCUAUCUGGCUUAUGGCAAUCGUCGUCCGGCAUAUGGAAGUCGGACUUACCUAUGCGGUAUGGGCGGGAAGCGGAACAGCUUUGACGGCGGUGCUCGGAAUGAUCUGGUUUGGUGAGUCGGCGAGCUUCCUUCGACUGGCCGGUCUAAGCUUCAUUGUUUUGGGC
env-74	CUUUGUUAUUCCGCGGCUAUCUGGCUUAUGGCAAUCGCCGUCCGGCAUAUGGAAGUCGGACUUACCUAUGCGGUAUGGGCGGGAAGCGGAACAGCUUUGACGGCGGUGCUCGGAAUGCUCUGGUUUGGUGAGUCGGCGAGCUUCCUUCGACUGGCCGGUCUAAGCUUCAUUGUUUUGGGC
env-75	CUUUGUUAUUCCGCGGCUAUCUGGCUUAUGGCAAUCGCCGUCCGGCAUAUGGAAGUCGGACUUACCUAUGCGGUAUGGGCGGGAAGCGGAACAGCUUUGACGGCGGUGCUCGGAAUGCUCUGGUUUGGUGAGUCGGCGAGCUUCCUUCGACUGGCCGGUCUAAGCUUCAUAGUUUUGGGC
Asp-2-1	CUUUGUUAUUCCGCCGCUAUCUGGCUUAUGGCAAUCGCCGUCCGGCAUAUGGAAGUCGGACUUACCUAUGCGGUAUGGGCGGGAAGCGGAACAGCUUUGACGGCGGUGCUCGGAAUGCUCUGGUUUGGUGAGUCGGCGAGCUUCCUUCGACUGGCCGGUCUUAGCUCCAUUGUUUUGGGC
Asp-7-1	CUUUGUUAUUCCGCCGCUAUCUGGCUUAUGGCAAUCGCCGUCCGGCAUAUGGAAGUCGGACUUACCUAUGCGGUAUGGGCGGGAAGCGGAACAGCUUUGACGGCGGUGCUCGGAAUGCUCUGGUUUGGUGAGUCGGCGAGCUUCCUUCGACUGGCCGGUCUAAGCUUCAUUGUUUUGGGC
env-76	CUUUGUUAUUCCGCCGCUAUCUGGCUUAUGGCAAUCGCCGUCCGGCAUAUGGAAGUCGGACUUACCUAUGCGGUAUGGGCGGGAAGCGGAACAGCUUUGACGGCGGUGCUCGGAAUGCUCUGGUUUGGUGAGUCGGCGAGCUUCCUUCGACUGGCCGGUCUAAGCUUCAUUGUUUUGGGC
env-77	CUUUGUUAUUCCGCCGCUAUCUGGCUUAUGGCAAUCGCCGUCCGGCAUAUGGAAGUCGGACUUACCUAUGCGGUAUGGGCGGGAAGCGGAACAGCUUUGACGGCGGUGCUCGGAAUGCUCUGGUUUGGUGAGUCGGCGAGCUUCCUUCGACUGGCCGGUCUAAGCUUCAUUGUUUUGGGC
env-78	CUUUGUUAUUCCGCCGCUAUCUGGCUUAUGGCAAUCGCCGUCCGGCAUAUGGAAGUCGGACUUACCUAUGCGGUAUGGGCGGGAAGCGGAACAGCUUUGACGGCGGUGCUCGGAAUGCUCUGGUUUGGUGAGUCGGCGAGCUUCCUUCGACUGGCCGGUCUAAGCUUCAUUGUUUUGGGC
env-79	CUUUGUUAUUCCGCCGCUAUCUGGCUUAUGGCAAUCGCCGUCCGGCAUAUGGAAGUCGGACUUACCUAUGCGGUAUGGGCGGGAAGCGGAACAGCUUUGACGGCGGUGCUCGGAAUGCUCUGGUUUGGUGAGUCGGCGAGCUUCCUUCGACUGGCCGGUCUAAGC...............
env-80	CUUUGUUAUUCCGCCGCUAUCUGGCUUAUGGCAAUCGCCGUCCGGCAUAUGGAAGUCGGACUUACCUAUGCGGUAUGGGCGGGAAGCGGAACAGCUUUGACGGCGGUGCUCGGAAUGCUCUGGUUUGGUG..
env-81	CUUUGUUAUUCCGCCGCUAUCUGGCUUAUGGCAAUCGCCGUCCGGCAUAUGGAAGUCGGACUUACCUAUGCGGUAUGGGCGGGAAGCGGAACAGCUUUGACGGCGGUGCUCGGAAUGCUCUGGUUUGGUGAGUCGGCGAGCUUCCUUCGACUGGCCGGUCUAAGCUUCAUUGUUUUGGGC
env-82	CUUUGUUAUUCCGCCGCUAUCUGGCUUAUGGCAAUCGCCGUCCGGCAUAUGGAAGUCGGACUUACCUAUGCGGUAUGGGCGGGAAGCGGAACAGCUUUGACGGCGGUGCUCGGAAUGCUCUGGUUUGGUGAGUCGGCGAGCUUCCUUCGACUGGCCGGUCUAAGCUUCAUUGUUUUGGGC
env-83	CUUUGUUAUUCCGCCGCUAUCUGGCUUAUGGCAAUCGCCGUCCGGCAUAUGGAAGUCGGACUUACCUAUGCGGUAUGGGCGGGAAGCGGAACAGCUUUGACGGCGGUGCUAGGAAUGCUCUGGUUUGGCGAGUCGGCGAGCUUCCUUCGACUGACCGGUCUAAGCUUCAUUGUUUUGGGC
env-84	CUUUGUUAUUCC..
env-85	CUCUGCUAUGGCGCGGCCAUCUGGCUGAUGUCCAUCUCCAUCAAGCACCUUGAGAUGGGCCUGACCUACGCAGUGUGGGCCGGGAGCGGCACCGCCCUCACCGCGGCCCUGGGCAUCGUCUGGUUUGGCGAGUCCUUCAGCGCCCUGAGGAUGGCCGGCCUGGGUUGCAUCGUGCUGGGG
Vsp-4-1	GCCUGCUAUGCCGCCGCGAUCUGGCUCAUGUCGCUCGCGGUGCGCGAGCUCGGCGUGGGGUUGAGCUAUGCGGUCUGGGCCGGCAGCGGCACGGUGCUGAUCGCCGUGCUCGGCAUGAUCUGGCUCGGCGAGCCCGUCACGCUGCCGCGGAUCGCCGGAGUGGCGGCGAUCGUCGCGGGC
Vsp-5-1	GCCUGCUAUGCCGCCGCGAUCUGGCUCAUGUCGCUCGCGGUGCGCGAGCUCGGCGUGGGGUUGAGCUAUGCGGUCUGGGCCGGCAGCGGCACGGUGCUGAUCGCCGUGCUCGGCAUGAUCUGGCUCGGCGAGCCCGUCACGCUGCCGCGGAUCGCCGGAGUGGCGGCGAUCGUCGCGGGC
env-87	GUUGCCUAUGCGAUCGCAAUCUGGUUGAUGUCGCUAUCGGUCAGACACUUGGAAGUGGGGACGGCCUAUGCAGUCUGGGCGGGGGCUGGCACUGCGUUGAUUGCCCUUGUCGGCAUCAUCUGGUUUCACGAAACAGUCACCUUCGC..................................
Cmi-1-1	ACUUGCUAUACGGGCGCAGUGUGGCUCAUGGCGAUCUCGACCAAACAUAUCGAGAUCGGCAUGGCCUACGCCGUGUGGGCCGGAGCUAGCACAGCCCUGACCGCUCUUGCCGGCAUCGCGUGGUUUGGCGAAUCCGUCACCGCCCUCAAGCUACUGGGUCUAGCCUUGGCCGUCGUUAGC
[me-1-1	GUCUGCUACGCGUGCGCCGUGUGGCUGAUGGCGCUCGCCACGAAGCGCCUCGAAAUGGGGCUCGCCUACGCAACCUGGGCGGGCGCCAGCACUGCGGCAACGGCCGUCAUCGGCAUCGUCUGCUUCGGCGAGUCCGCUUCGCCGAUCAAGCUGGCCGGCGUCGCGCUGGCCGUCUGGGGG
Bce-1-3	GUCUGCUAUGCGUGCGCGGUCUGGCUGAUGGCGCUCGCCACCCGGCGGCUCGAAAUGGGGCUCGCCUAUGCGAGCUGGGCGGCCGCGAGCACGGCCGCCACGGCCGUGAUCGGCAUCGUCUGCUUCGACGAAUCCGCAUCGUCGCUGAAACUCGCGGGCAUCGCGCUGACCGUCUGCGCG
Bps-1-1	GUCUGCUAUGCGUGCGCGGUCUGGCUGAUGGCGCUCGCCACCCGGCGGCUCGAAAUGGGGCUCGCCUAUGCGAGCUGGGCGGCCGCGAGCACGGCCGCCACGGCCCUGAUCGGCAUCGUCUGCUUCGACGAAUCCGCAUCGUCGCUGAAACUCGCUGGCAUCGCGCUGACCGUCUGCGCG
Bsp-5-1	GUCUGCUAUGCAUGUGCGGUAUGGCUGAUGGCGCUCGCGACGAAGCGAAUCGAAAUGGGGCUCGCGUAUGCGAGCUGGGCCGCUGCCAGCACGGCGGCCACGGCCAUGAUCGGCAUCGUCUGCUUCGACGAAUCGGUGUCGUCGCUGAAGCUCGUCGGCAUCGCGCUGACCGUCUGCGCG
Bsp-7-1	GUCUGCUAUGCAUGUGCGGUAUGGCUGAUGGCGCUCGCGACGAAGCGAAUCGAAAUGGGGCUCGCGUAUGCGAGCUGGGCCGCUGCCAGCACGGCGGCCACGGCCAUGAUCGGCAUCGUCUGCUUCGACGAAUCGGUGUCGUCGCUGAAGCUCGUCGGCAUCGCGCUGACCGUCUGCGCG
Bsp-6-1	GUCUGCUAUGCAUGUGCGGUAUGGCUGAUGGCGCUCGCGACGAAGCGAAUCGAAAUGGGGCUCGCGUAUGCGAGCUGGGCCGCUGCCAGCACGGCGGCCACGGCCAUGAUCGGCAUCGUCUGCUUCGACGAAUCGGUGUCGUCGCUGAAGCUCGUCGGCAUCGCGCUGACCGUCUGCGCA
Bsp-3-1	GUCUGCUAUGCAUGUGCGAUAUGGCUGAUGGCGCUCGCGACGAAGCGAAUCGAAAUGGGGCUCGCGUAUGCGAGCUGGGCCGCUGCCAGCACGGCGGCCACGGCCAUGAUCGGCAUCGUCUGCUUCGACGAAUCGGUGUCGUCGCUGAAGCUCGUCGGCAUCGCGCUGACCGUCUGCGCA
Bsp-2-1	GUCUGCUAUGCAUGUGCGGUAUGGCUGAUGGCGCUCGCGACGAAGCGAAUCGAAAUGGGGCUCGCGUAUGCGAGCUGGGCCGCUGCCAGCACGGCGGCCACGGCCAUGAUCGGCAUCGUCUGCUUCGACGAAUCGGUGUCGUCGCUGAAGCUCGUCGGCAUCGCGCUGACCGUCUGCGCA
Bsp-4-1	GUCUGCUAUGCAUGUGCGGUAUGGCUGAUGGCGCUCGCGACGAAGCGAAUCGAAAUGGGGCUCGCGUAUGCGAGCUGGGCCGCUGCCAGCACGGCGGCCACGGCCAUGAUCGGCAUCGUCUGCUUCGACGAAUCGGUGUCGUCGCUGAAGCUCGUCGGCAUCGCGCUGACCGUCUGCGCA
Bsp-16-1	GUCUGCUAUGCGAGUGCCGUCUGGCUGAUGGCGCUCGCGACGAAGCGCCUCGAAAUGGGGCUCGCCUAUGCGAGCUGGGCCGCCGCCAGCACGGCGGCUACCGCCGUCAUCGGCAUCGUCUGCUUCGAUGAAUCGGUGUCGUCGCUGAAGCUCGUCGGCAUCGCGUUGACCCUCUGCGCC
Bsp-15-1	GUCUGCUAUGCGAGUGCCGUCUGGCUGAUGGCGCUCGCGACGAAGCGCGUCGAAAUGGGGCUCGCCUAUGCGAGCUGGGCCGCCGCCAGCACGGCGGCCACCGCCGUCAUCGGCAUCGUCUGCUUCGAUGAAUCGGUAUCGUCGCUGAAGCUCGUCGGCAUCGCGUUGACCGUCUGCGCC
Bpu-1-1	GUCUGUUACGCGUGUGCUGUCUGGCUGAUGGCGCUCGCGACCAAGCGGCUCGAAAUGGGGCUCGCGUAUGCAAGCUGGGCCGCCGCCAGCACCGCGGCCACAGCCGUGAUCGGCAUCGUCUGCUUCGACGAAUCGGUGUCGUCGCUGAAGCUCGCCGGCAUCGCGCUGACCGUCUGUGCG
Bst-1-1	GUCUGUUAUGCGUGCGCCGUCUGGUUGAUGGCGCUCGCGACGAAGCGUCUCGAACUGGGGCUCGCGUAUGCGAGCUGGGCCGCCGCCAGCACGGCGGCCACAGCCGUGAUCGGUAUCGUCUGCUUCGAUGAGUCGGUGUCGUCGCUGAAGCUCGCGGGCAUCGCGCUGACCGUCUGUGCG
Bsp-9-1	GUCUGUUAUGCCUGCGCGGUGUGGCUGAUGGCGCUCGCGACGAAGCGCCUCGAAAUGGGGCUCGCGUAUGCGAGCUGGGCCGCCGCCAGCACCGCGGCCACGGCCGUGAUCGGCAUCCUCUGCUUCGAUGAAUCGGUGUCGUCGCUGAAGCUCGCCGGCAUCGCGCUGACCGUCUGUGCG
Bvi-2-1	GUCUGUUAUGCGUGCGCCGUCUGGCUGAUGGCGCUCGCGACGAAGCGGCUCGAAAUGGGGCUCGCGUACGCGAGCUGGGCCGCCGCCAGCACGGCGGCCACGGCCGUGAUCGGCAUCGUCUGCUUCGACGAAUCGGUGUCGUCGCUGAAGCUCGCCGGCAUCGCGCUGACCGUUUGUGCG
Bla-1-1	GUCUGUUAUGCCUGCGCCGUCUGGCUGAUGGCGCUCGCGACGAAGCGGCUCGAAAUGGGGCUCGCGUACGCGAGCUGGGCCGCCGCCAGCACCGCGGCCACGGCCGUGAUCGGCAUCGUCUGCUUCGACGAAUCGGUGUCGUCGCUGAAGCUCGCCGGCAUCGCGCUGACCGUUUGUGCG
Bce-4-1	GUCUGUUAUGCGUGCGCCGUCUGGCUGAUGGCGCUCGCGACGAAGCGGCUCGAAAUGGGGCUCGCGUACGCGAGCUGGGCCGCCGCCAGCACCGCGGCCACGGCCGUGAUCGGCAUCGUCUGCUUCGACGAAUCGGUGUCGUCGCUGAAGCUCGCCGGCAUCGCGCUGACCGUUUGUGCG
Bsp-21-1	GUCUGUUAUGCCUGCGCCGUCUGGCUGAUGGCGCUCGCGACGAAGCGGCUCGAAAUGGGGCUCGCGUACGCGAGCUGGGCUGCCGCCAGCACCGCGGCCACGGCCGUGAUCGGCAUCGUCUGCUUCGACGAAGCGGUGUCGUCGCUGAAGCUCGCCGGCAUCGCGCUGACCGUUUGUGCG
Bsp-1-1	GUCUGUUACGCAUGCGCGGUCUGGCUGAUGGCGCUCGCGACGAAACGCCUCGAAAUGGGGCUCGCAUAUGCAAGCUGGGCCGCCGCCAGCACCGCGGCCACGGCUGUGAUCGGCAUCGUCUGCUUCGACGAAUCGACAUCGUCGCUGAAGCUCGCCGGCAUCGCGCUGACCGUGUGUGCG
Bsp-14-1	GUCUGUUACGCAUGCGCGGUCUGGCUGAUGGCGCUCGCGACGAAGCGCCUCGAACUGGGGCUCGCCUAUGCGAGCUGGGCCGCCGCCAGCACCGCGGCCACGGCCGUGAUCGGUAUCGUCUGCUUCAAUGAAUCGGCGUCGUCGCUGAAGCUCGCCGGCAUCGCGCUGACCGUCUGUGCG
Bsp-12-1	GUCUGUUACGCAUGCGCGGUCUGGCUGAUGGCGCUCGCGACGAAGCGCCUCGAACUGGGGCUGGCCUAUGCGAGCUGGGCCGCCGCCAGCACCGCGGCCACGGCCGUGAUCGGUAUCGUCUGCUUCAAUGAAUCGGUGUCGUCGCUGAAGCUCGCCGGCAUCGCGCUGACCGUCUGUGCG
Bse-1-1	GCCUGCUAUGCCUGCGCCGUAUGGCUGAUGGCGCUCGCGACGAAGCGGCUCGAACUGGGGCUCGCGUACGCGAGCUGGGCCGCCGCCAGCACCGCGGCCACCGCCGUGAUCGGCAUCGUCUGCGUCGACGAGUGGGUGUCGUCGCUGAAGCUCGCCGGCCUCGCGCUGACCGUCUGCACG
Bce-6-1	GUCUGCUAUGCGUGCGCCGUGUGGCUGAUGGCGCUCGCGACGAAGCGGCUCGAAAUGGGGCUCGCCUAUGCAAGCUGGGCCGCUGCCAGCACGGCGGCCACCGCCGUGAUCGGCAUCGUCUGCUUCGACGAAUCGGUGUCGUCGCUGAAGCUCGCCGGCAUCGCGCUGACCGUCUGCGCG
env-88	CUGCAGCCAGCUUUUUACUGGCGAUAUGGCUGAUGUCACUCUCGUUAAAACAUAUUGAGAUGAGCAUCACCUAUGCCGUGUGGGCAGGUAGCGCGACGGCAAUUUAGCGCUGGUUGGCAUCACCCUCUACGAUGAAUCCAUGUCGCAGUUUAAGCUGGUCGGCAUGCUACUGGUCAUCG
env-90	AGCAGGGUUCUUCUAUGUGGUCGCAAUCUGGCUCAUGGCCAUAGCGAUGCGGCAACUAGAAAUGGGAGUCACGUAUGCAGUUUGGGCUGCGAGCGGAACAGCAGCCAUUGCUUUGGUCGGCAUCGCUUUCUACGGGGAGCAAACUUCAGCUCUCAAGCUCGCAGGUCUGGCGCUCGUAGU
Rsp-1-1	CGCGGGUUUCUUCUAUGUGAUCGCAAUCUGGCUUAUGGCCAUAGCGAUGCGGCAACUGGAAAUGGGAGUCACCUAUGCAGUGUGGGCUGCGAGCGGAACAGCAGCCAUUGCGUUGGUCGGCAUCGCUUUCUACGGGGAGCAAACUUCGGCUCUCAAGCUCGCAGGUCUGGUGCUCGUGGU
env-91	GGCCGGCGGCUGCUACGUCGCCGCGGUGUGGCUGAUGGCCGUGGCCAUGCGGCAGCUUGAAAUGGGCAUCGCCUACGCGGUGUGGGCCGCGGGCGGAACGGCGAUCACCGUCCUCAUCGGCAUCCUUCGUUAUGGCGAGCAGCUGUCGAUCCACAAGCUCGCCGGCGUGGGCCUGGUGCU
env-93	....
env-94	AGCGGCAGCCAGCUAUGCGCUCGCGAUCUGGCUGAUGGCGGUUGCCA...
env-95	AGCGGCAGCCAGCUAUGCGCUCGCGAUCUGGCUGAUGGCGGUUGCCA...UGCGGCAGCUUG
env-96	GGCCGCUGCGUGCUACGCGCUCGCCAUCUGGCUCAUGGCCGUGGCCAUGCGCCAGCUUGACAUGGGCAUCACCUACGCGGUCUGGGCCGCCAGCGGCACGGCGGCCACGGCCGUGGUGGGGAUAGCGCUGCUGGGCGAGCCCGUGGACACCCUCAAGGUGCUGGGCCUGGUGUGCGUGGU
env-97	GGCAGCUGCGUGCUACGCACUCGCCAUCUGGCUCAUGGCCGUGGCCAUGCGCCAGCUCGACAUGGGCCUGACCUAUGCAGUCUGGGCGGCCAGCGGCACGGCGGCCGCAGCCGUGGUGGGGAUGGCGCUGUUUGGCGAGCCAGUGGGUGUCAUCAAAGUAGUGGGUCUGGUGUGCGUGGU
env-99	GGCAGCUGCGUGCUACGCACUCGCCAUCUGGCUCAUGGCCGUGGCCAUGCGCCAGCUCGACAUGGGCCUCACCUAUGCAGUCUGGGCGGCCAGCGGCACGGCGGCCGCAGCCGUGGUGGGGAUGGCGCUGUUUGGCGAGCCAGUGGGUGUCAUCAAAGUAGUGGGUCUGGUGUGCGUGGU
env-101	....
Asp-9-1	GGCAGCUGCGUGCUACGCACUCGCCAUCUGGCUCAUGGCCGUGGCCAUGCGCCAGCUCGACAUGGGCCUCACCUAUGCAGUCUGGGCGGCCAGCGGCACGGCGGCCGCAGCCGUGGUGGGGAUGGCGCUGUUUGGCGAGCCAGUGGGUGUCAUCAAAGUAGUGGGUCUGGUGUGCGUGGU
env-102	GGCAGCUGCGUGCUACGCACUCGCCAUCUGGCUCAUGGCCGUGGCCAUGCGCCAGCUCGACAUGGGCCUCACCUAUGCAGUCUGGGCGGCCAGCGGCACGGCGGCCGCAGCCGUGGUGG...
env-103	GGCAGCUGCGUGCUACGCACUCGCCAUCUGGCUCAUGGCCGUGGCCAUGCGCCAGCUCGACAUGGGCCUCACCUAUGCAGUCUGGGCGGCCAGCGGCACGGCGGCCGCAGCCGUGGUGG...GGAUGGCGCUGU
env-104	GGCAGCUGCGUGCUACGCACUCGCCAUCUGGCUCAUGGCCGUGGCCAUGCGCCAGCUCGACAUGGGCCUCACCUAUGCAGUCUGGGCGGCCAGCGGCACGGCGGCCGCAGCCGUGGUGGGGAUGGCGCUGUUUGGCGAGCCAGUGGGUGUCAUCAAAGUAGUGGGUCUGGUGUGCGUGGU
env-106	....GCCGGAACCGUC
env-107	GGCAGCUGCGUGCUACGCACUCGCCAUCUGGCUCAUGGCCGUGGCCAUGCGCCAGCUCGACAUGGGCCUCACCUAUGCAGUCUGGGCGGCCAGCGGCACGGCGGCGGCAGCCGUGGUGGGGAUGGCGCUGUUUGGCGAGCCAGUGGGUGUCAUCAAAGUAGUGGGUCUGGUGUGCGUGGU
env-110	....
env-111	GGCAGCUGCGUGCUAC..
env-112	GGCAGCUGCGUGCUACGCACUCGCCAUCUGGCUCAUGGCCGUGGCCAUGCGCCAGCUCGACAUGGGCCUCACCUAUGCAGUCUGGGCGGCCAGCGGCACGGCGGCCGCAGCCGUGGUGGGGAUGGCGCUGUUUGGCGAGCCAGUGGGUGUCAUCAAAGUAGUGGGUCUGGUGUGCGUGGU
env-113	GGCAGCUGCGUGCUACGCACUCGCCAUCUGGCUCAUGGCCGUGGCCAUGCGCCAGCUCGACAUGGGCCUCACCUAUGCAGUCUGGGCGGCCAGCGGCACGGCGGCCGCAGCCGUGGUGGGGAUGGCGCUGUUUGGCGAGCCAGUGGGUGUCAUCAAAGUAGUGGGUCUGGUGUGCGUGGU
Asp-5-1	GGCCGCUGCGUGCUACGCGCUCGCCAUCUGGCUCAUGGCGGUGGCCAUGCGCCAACUGGACAUGGGCAUCACCUAUGCGGUCUGGGCCGCAAGCGGCACGGCGGCCACGGCCAUCGUGGGGAUCGCACUGUUUGGCGAGCCAGUGGACACCAUCAAGGCACUGGGCCUGGUUUGCGUGGU
Ara-1-1	GGCCGCUGCGUGCUACGCGCUCGCCAUCUGGCUCAUGGCGGUGGCCAUGCGCCAACUGGACAUGGGCAUCACCUAUGCGGUCUGGGCCGCAAGCGGCACGGCGGCCACGGCCAUCGUGGGGAUUGCACUGUUUGGCGAGCCAGUGGACACCAUCAAGGCACUGGGCCUGGUUUGCGUGGU
Asp-6-1	GGCCGCUGCGUGCUACGCGCUCGCCAUCUGGCUCAUGGCGGUGGCCAUGCGCCAACUGGACAUGGGCAUCACCUAUGCGGUCUGGGCCGCAAGCGGCACGGCGGCCACGGCCAUCGUGGGGAUUGCACUGUUUGGCGAGCCAGUGGACACCACCAAGGCACUGGGCCUGGUUUGCGUGGU
env-114	....
env-115	AGCCGCAGCGUCCUACGCGCUUGCCAUCUGGUUCAUGGCAGUUGCCAUGCGGCAACUGGAUAUGGGGAUCACGUACGCUGUCUGGGCGGCAAGCGGAACUGCGGCCACGGCUCUCGUCGGCGUCAUGUUCUUCGGGGAGCAGAUGGGCGGCAUCAAGGCUCUUGGCCUUGCUUGUGUCAU
env-117	AGCCGCAGCGUCCUACGCGCUUGCCAUCUGGUUCAUGGCAGUUGCCAUGCGGCAACUGGAUAUGGGGAUCACGUACGCUGUCUGGGCGGCAAGCGGA...
env-118	AGCGGCGGCUUGCUACGCGCUGGCCAUCUGGCUCAUGGCUGUUGCCAUGCGCCAGCUCGACAUGGGCAUCACCUACGCCGUCUGGGCCGCAAGCGGUACGGCGGCCACCGCGCUGGUUGGCAUCGCGUUUUUCGGGGAGCACAUGGGCUCGGUCAAGGCGCUGGGCCUGGUGUGCGUGGU
env-120	AGCGGCUGCUUGUUACGCACUUGCCAUCUGGCUCAUGGCCGUUGCCAUGCGGCACCUCGACAUGGGCGUCACUUACGCAGUUUGGGCCGCAAGCGGUACUGCAGCAACGGCUCUGGUCGGAAUCGCUGUCUACGGAGAGCAUCUGGGCAUUCUCAAAGCGCUGGGCUUGGUCUGCAUCGU
env-125	AGCGGCUGCUUGUUACGCGCUUGCCAUCUGGCUCAUGGCCGUUGCCAUGCGGCACCUCGACAUGGGAAUCACUUAUGCAGUCUGGGCCGCAAGCGGUACUGCGGGAACGGCUCUGGUCGGAAUCGCGGUCUUCGGCGAGCAACUGGGCAUCCUCAAGGCGCUGGGCUUGGUCUGCAUCGU
Psp-1-1	GGCAGCGGCGUGUUACGCGCUUGCCAUUUGGCUCAUGGCAGUUGCCAUGCGGCAACUCGAGAUGGGCAUUACUUAUGCAGUCUGGGCCGCAAGCGGCACUGCAGCAACCGCCCUUGUCGGCAUCGCGUUCUACGGGGAGCAGAUGGGUUACGUCAAGGCGCUAGGUCUUGUUUGCGUAGU
	....
	....
	....
	....
	●●Y●●○○
	3′

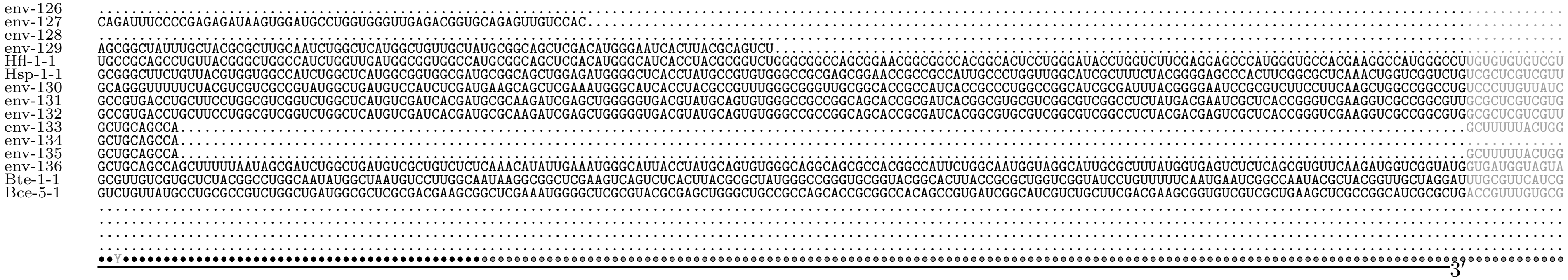

alignment positions 541 · · · 720

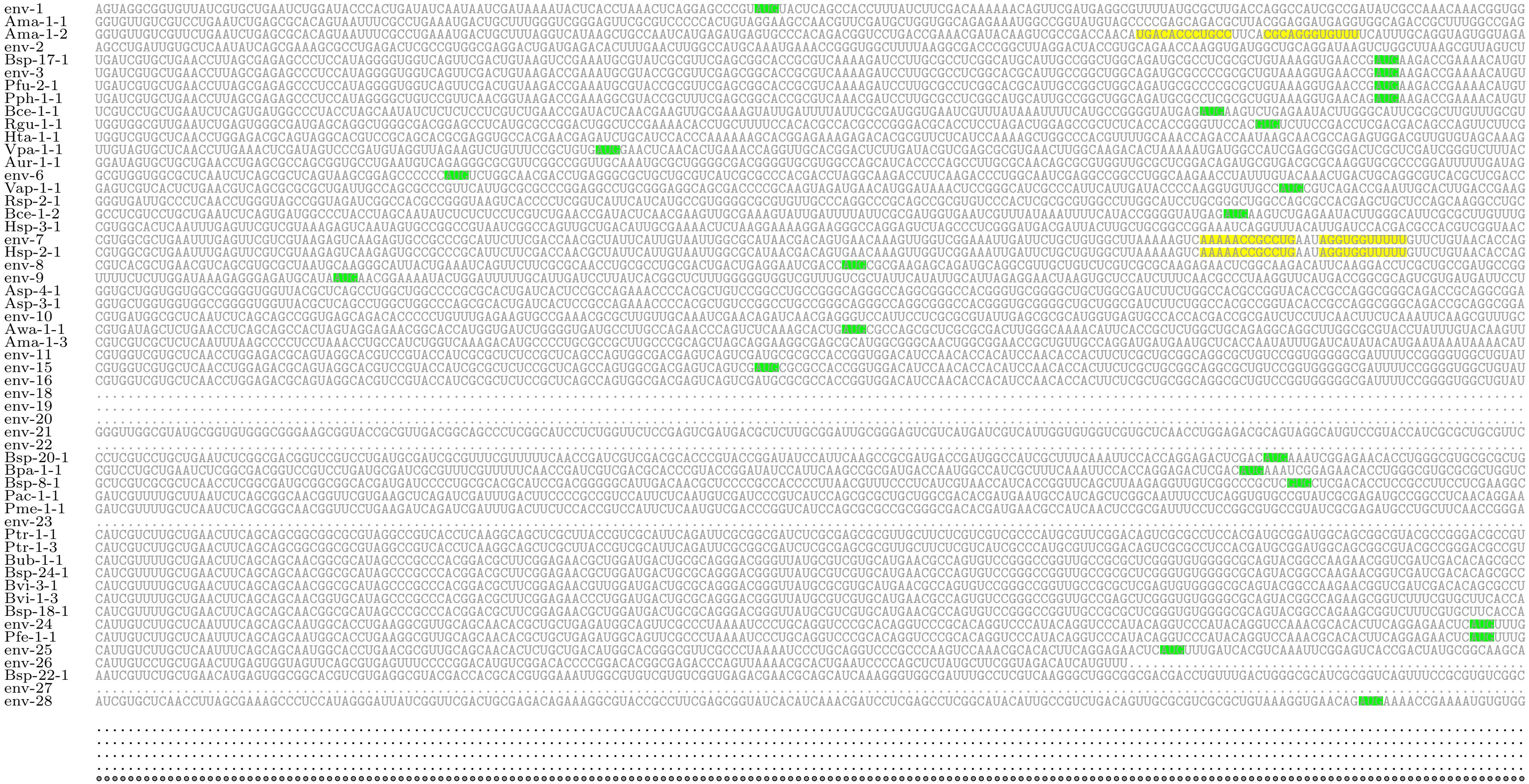

```
env-29   GUCGUCAUUCUGAAUAUCAGUGCGCGCUGAUGGUGGCAUGUGGGCGCGCCAUCAUAAUGGACUGCCAUUUUUUCCAGAAUGGACGUUUGGAUUCAGUCUAUUCUGGGUAAAAGAUAGCCUGCCCGAUGGCCAAAUGGCGCGGCAACUCUCGGAUGGCCACGCUGAUUGCAACGGGCCGUC
env-30   GUCAUUGCUUUGAACUUGAGUGCUCGAUAAGUCGCCGGACACGCAUUUUCAAAGACUGAUGCACGCUUCAGCGACCGACUUCAAACGGCGCAUUGCAGUCGUCCAUCCCUCCGAUAAGCCGUCAUUGGCGAUGCGCCGACUGACAGACCGCUUUUGGGCGGUGCGGCAGACGGCUCUGCG
env-31   ........................................................................................................
env-32   ........................................................................................................
env-33   UGACGGCGGUGCUCGGAAUGCUC.................................................................................
env-34   UGACGGCGGUGCUCGGAAUGCUCUGGUUUGGUGAGUCGGCGAGCUUCCUUCGACUGGCCGGUCUAAGCUUCAUUGUUUUGGGCGUCAUUGCUUUGAACUUGAGUGCCCGAUAGGUCGCCGGACACGCAUUUUCAAAGACUGAUUCACGCUUCAGCGAGCGGCUACAAAGGGACCGAAGCG
env-35   UGACGGCGGUGCUCGGAAUGCUCUGGUUUGGUGAGUCGGCGAGCUUCCUUCGACUGGCCGGUCUAAGCUUCAUUGUUUUGGGCGUCAUUGCUUUGAACUUGAGUGCCCGAUAGGUCGCCGGACACGCAUUUUCAAAGACUGAUUCACGCUUCAGCGAGCGGCUACAAAGGGACCGAAGCG
env-36   UGACGGCGGUGCUCGGAAUGCUCUGGUUUGGUGAGUCGGCGAGCUUCCUUCGACUGGCCGGUCUAAGCUUCAUUGUUUUGGGCGUCAUUGCUUUGAAC.......................................
env-37   ........................................................................................................
env-38   ........................................................................................................
env-39   GCUUAUGGCAAUCGCCGUCCGGCAUAUGGAAGUCGGACUUACCUAUGCGGUAU...................................................
env-40   ........................................................................................................
env-41   ........................................................................................................
env-42   UGCUCUCGGAAGUCGUUGGAACCAUUGCACUCCGCUAUGC................................................................
env-44   UGCUCUCGGAAGUCGUUGGAACCAUUGCAC..........................................................................
env-45   UGCUCGCGGAAGUCGUUGGAACCAUUGCAC..........................................................................
env-46   UGCUCGCGGAAGUCGUUGGAACCAUUGCACUCCGCUAUGCGGAUGGCUUCACACGUAUUUUCCCUUCCGUCCUUUCAGGGCUUUGUUAUUCCGCCGCUAUCUGGCUUAUGGCAAUCGUCGUCCGGCAUAUGGAAGUCGGACUUACCUAUGCG.........................
env-47   ........................................................................................................
env-48   GCUUAUGGCAAUCGCCGUCCGGCAUAUGGAAGUCGGACUUACCUAUGCGGUAUGGGCG..............................................
env-49   UGCUCUCGGA..............................................................................................
env-50   UGCUCUCGGAAGUCGUUGGAACCAUUGCACUC........................................................................
env-51   UGCUCUCGGAAGUCGUUGGAGCCAUUGCACUCCGCUAUGCGGAUGGCUUCACACGUAUUUUCCCUUCCGUCCUUUCAGGGCUUUGUUAUUCCGCCGCUAUCUGGCUUAUGGCAAUCGCCGUCCGGCAUAUGGAAGUCGGACUUACCUAUGCGGUAUGGGCGGGAAGCGGAACAGCUUUGA
env-52   UGCUCGCGGAAGUCGUUGGAACCAUUGCACUCCGCUAUGCGGAUGGCUUC......................................................
env-53   UGACGGCGGUGCUCGGAAUGCUCUGGUUUGGUGAGUCGGCGAGCUUCCUUCGACUGGCCGGUCUAAGCUUCAUUGUUUUGGGCGUCAUUGCUUUGAACUUGAGUGCCCGAUAGGUCGCCGGACACGCAUUUUCAAAGACUGAUUCACGCUUCAGCGAGCGGCUACAAAGGGACCGAAGCG
env-61   ........................................................................................................
env-62   ........................................................................................................
env-63   GUCAUUGCUUUGAACUUGAGUGCCCGAUAAGUCGCCGGACACGCAUUUUCAAAGACGGAUUCACGCUUCAGCGAGCGGCUACAAAGGGACCGAAGCGGACUUGUUUGCUCUAAGCCGAGCAGCGUCAAGGCCCCAAUGCCGUUAAGCCAGAACGCCGUUUUUGCGUCCUGGGUAUGCGAU
env-64   ........................................................................................................
env-65   GUCAUUGCUUUGAACUUGAGUGCCCGAUAAGUCGCCGGACACGCAUUUUCAAAGACGGAUUCACGCUUCAGCGAGCGGCUACAAAGGGACCGAAGCGGACUUGUUUGCUCUAAGCCGAGCAGCGUCAAGGCCCCAAUGCCG...............................
env-66   GUCAUUGCUUUGAACUUGAGUGCCCGAUAAGUCGCCGGACACGCAUUUUCAAAGACGGAUUCACGCUUCAGCGAGCGGCUACAAAGGGACCGAAGCGGACUUGUUUGCUCUAAGCCGAGCAGCGUCAAGGCCCCAAUGCCGUUAAGCCAGAACGCCGUUUUUGCGUCCUGGGUAUGCGAU
env-70   UGCGGUAUGGGCGGCAAGCGGAACAGCUUUGACGGCGGUGCUCGGAAUGCUCUGGUUUGGUGAGUCGGCGAGCUUCCUUCGACUGGCCGGUCUAAGCUUCAUUGUUUUGGGCGUCAUUGCUUUGAACUUGAGUGCCCGAUAAGUCGCCGGACACGCAUUUUCAAAGACGGAUUCACGCUU
Asp-8-1  GUCAUUGCUUUGAACUUGAGUGCCCGAUAAGUCGCCGGACACGCAUUUUCAAAGACGGAUUCACGCUUCAGCGAGCGGCUACAAAGGGACCGAAGCGGACUUGUUUGCUCUAAGCCGAGCAGCGUCAAGGCCCCAAUGCCGGCAAGCCAGAACGCCGUUUUUGCGUCCUGGGUAUGCGAU
env-71   GUCAUUGCUUUGAACUUGAGUGCCCGAUAGGUCGCCGGACACGCAUUUUCAAAGACUGAUUCACGCUUCAGGGAACGGCUGCAAAGGGACCGAAGCGGACUUGUUUGCCUUAAGCCGAGCAGCUUCGAGGACCCAAUGCCGGCAAGCCAGAACCCCGUUUUUGCUUUGGUGUUGCGUCCU
env-72   GUCAUUGCUUUGAACUUGAGUGCCCGAUAGGUCGCCGGACACGCAUUUUCAAAGACUGAUUCACGCUUCAGGGAACGGCUGCAAAGGGACCGAAGCGGACUUGUUUGCCUUAAGCCGAGCAGCUUCGAGGACCCAAUGCCGGCAAGCCAGAACCCCGUUUUUGCUUUGGUGUUGCGUCCU
env-73   GUCAUUGCUUUGAACUUGAGUGCCCGAUAGGUCGCCGGACACGCAUUUUCAAAGACUGAUUCACGCUUCAGGGAACGGCUGCAAAGGGACCGAAGCGGACUUGUUUGCCUUAAGCCGAGCAGCUUCGAGGACCCAAUGCCGGCAAGCCAGAAC..........................
env-74   GUCAUUGUUUUGAACUUGAGUGCCCGAUAAGUCGCCGGACACGCAUUUUCAAAGACUGAUUCACGCUUCAGCGAGCGGCUGCAAAGGGACCGAAGCGGACUUGUUUGCCUUAAGCCGAGCAGCGUCGAGGACCCAAUGCCGGCAAGCCAGAACCCCGUUUUUGCGUCGAUGUUGCGUCCU
env-75   GUCAUUGCUUUGAACUUGAGUGCCCGAUAAGUCGCCGGACACGCAUUUUCAAAGACGGAUUCACGCUUCAGCGAGCGGCUACAAAGGGACCGAAGCGGACUUGUUUGCUCUAAGCCG.................................................
Asp-2-1  GUCAUUGCUUUGAACUUGAGUGCCCGAUAAGUCGCCGGACACGCAUUUUCAAAGACUGAUUCACGCUUCGGCGAACGGCUGCAAAGGGACCGAAGCGGACUUGUUUGCUCUAAGCCGAGCAGCGUCAAGGCCCCAAUGCCGUUAAGCCAGAACACCGUUUUUGCGUCGGUGUUGCGUCCU
Asp-7-1  GUCAUUGCUUUGAACUUGAGUGCCCGAUAGGUCGCCGGACACGCAUUUUCAAAGACUGAUUCACGCUUCAGGGAACGGCUGCAAAGGGACCGAAGCGGACUUGUUUGCCUCAAGCCGAGCAGCUUCGAGGACCCAAUGCCGGCAAGCCAGAACCCCGUUUUUGCUUUGGUGUUGCGUCCU
env-76   GUCAUUGUUUUGAACUUGAGUGCCCG..............................................................................
env-77   GUCAUUGCUUUGAACUUGAGUGCCCGAUAGGUCGCCGGACACGCAUUUUCAAAGACUGAUUCACGCUUCAGCGAGCGGCUACAAAGGGACCGAAGCGGACUUUUUUGCCUUAAGCCGAGCAGCUUCGAGGACUCAAUGCCGGCAAGCCAGAACCCCGUUUUUGCUUUGGUGUUGCGUCCU
env-78   GUCAUUGCUUUGAACUUGAGUGCCCGAUAGGUCGCCGGACACGCAUUUUCAAAGACUGAUUCACGCUUCAGCGAGCGGCUACAAAGGGACCGAAGCGGACUUUUUUGCCUUAAGCCGAGCAGCUUCGAGGACUCAAUGCCGGCAAGCCAGAACCCCGUUUUUGCUUUGGUGU........
env-79   ........................................................................................................
env-80   ........................................................................................................
env-81   GUCAUUGCUUUGAACUUGAGUGCCCGAUAGGUCGCCGGACACGCAUUUUCAAAGACUGAUUCUUUGAAAAUGCGUGUCCGGCGACCUAUCGGGCACUCAAGUUCAAAGCAAUGA......................................
env-82   GUCAUUGCUUUGAACUUGAGUGCCCGAUAGGUCGCCGGACACGCAUUUUCAAAGACUGAUUCACGCUUCAGCGAGCGGCUACAAAGGGACCGAAGCGGACUUUUUUGCCUUAAGCCGAGCAGCUUCGAGGACUCAAUGCCGGCAAGCCAGAACCCCGUUUUUGCUUUGGUGUUGCGUCCU
env-83   GUCAUUGCUUUGAACUUGAGUGCCCGAUAAGUCGCUGGACACGCAUUUUCAAAGACUGAUUCACGCUUCGGCGAACGGCUGCAAAGGGACCGAAGCGGACUUGUUUGCUCUAAGCCGAGCAGCGUCAAGGCCCCAAUGCCGUUAAGCCAGAACACCGUUUUUGCGUCGGUGUUGCGUCCU
env-84   ........................................................................................................
env-85   GUGAUCGCUGAACCUCAGCAGUCACUGAAGCGCGAAUCCAUACAACGAAGAUCAGGGAUGAUGCC[illegible]CUUGAGCAAUGCCUUCAGGCGCUGAUGCGCCAGCGUUCGCGCGACCUUGGCAAGACGUUCACCGUGCUGGCCGCGGAGGCUGGACUGGCGCGAACCUACUUGUACAAACU
Vsp-4-1  GUGAUCGUGCUCAACCUCGAGGCGCGCUAGCGGCUCGGUGCCGCGGGCGUCUCGACCAGGUCGUCGUCGAUGUGCGGAUCGGCCGGCUGCCCGCGCAGCCUGCGCACGGUGCGCCUGAUCCAGUGCUCGAUGCUCGUCGAUGUAGGUGAACACGGCCGGCACCACCAGCAGGCUCAGCACG
Vsp-5-1  GUGAUCGUGCUCAACCUCGAGGCGCGCUAGCGGCUCGGUGCCGCGGGCGUCUCGACCAGGUCGUCGUCGAUGUGCGGAUCGGCCGGCUGCCCGCGCAGCCUGCGCACGGUGCGCCUGAUCCAGUGCUCGAUGCUCGUCGAUGUAGGUGAACACGGCCGGCACCACCAGCAGGCUCAGCACG
env-87   ........................................................................................................
Cmi-1-1  CUGAUUGUGCUCAGCCUUAGCGAAAGCCCUCCAUAGGGAUUAUCGGUUCGACUGCGAGACGAAAAGCGUACCGCCUUCGAGCGGUAUCACAUCAAACGAUCCUCGAGCCUGGGCAUGCAUUCAUCCUCGUCCUUGAGCCGGCUUUUGGCGGAUUGUUCGGUUACCUUUUGGGCAGUGAU
[me-1-1  AUCUUCGCGCUCAAUCUCGGCGAUGGCUCGCAUUAGCGUUCCGGCAUCCGGUUCGAUCCGCGCGCCCGGAUGCAAUCGCUAACCGCGGCGGCGGUCGCGAUGCAGCGCCUCGUGAUCGCACCGAAAUCGGCCUGGAUGACGGCGAAGAUGAACGCGGUGGACAUGCCGAACAGCAGCAC
Bce-1-3  CUCGUCGCGCUCAAUCUCGGCGAUGGGGCGGCGCGAUGAGCGCGAUAAAGGUGAUGAAGGCGCCGGCCGUUGCGCGCAUGCCGUUCGCGGCCGCGCGCGAGGCUCGCAUCGCUCGCGUGGUUCCCGAUGCGGGCCUCAGGUGCGCGCGCAUGCCGCGCACGGUUUGCGAGCACGCUCGCA
Bps-1-1  CUCGUCGCGCUCAAUCUCGGCGAUGGGCGGCGCGAUGAGCGCGAUAAAGGUGAUGAAGGCGCCGGCCGUUGCGCGCAUGCCGUUCGCGGCCGCGCGCGAGGCUCGCAUCGCUCGCGUGGUUCCCGAUGCGGGCCUCAGGUGCGCGCGCAUGCCGCGCACGGUUUGCGAGCACGCUCGCA
Bsp-5-1  CUCGUCGCGCUCAACCUCGGCGAUGCGGCGGCACGAUGAUCCCCUGCGCACGCAUCCGACGGGGGCAUUGACAACGCUCCCCGCCGACCCUUAACGUUUCCCUCAUCGUAACCAUCACCGGUUCAGCUUAAGAGGUUGUCGGCCCGCUC[illegible]CUCGACACCUCCGCCUUCCUCGAAGGCC
Bsp-7-1  CUCGUCGCGCUCAACCUCGGCGAUGCGGCGGCACGAUGAUCCCCUGCGCACGCAUCCGACGGGGGGCAUUGACAACGCUCCCCGCCACCCCUUAACGUUUCCCUCAUCGUAACCAUCACCGGUUCAGCUUAAGAGGUUGUCGGCCCGCUC[illegible]CUCGACACCUCCGCCUUCCUCGAAGGC
Bsp-6-1  CUCGUCGCGCUCAAUCUCGGCGAUGCGGCGGCACGUUGAUCCAUGCGCACGCAUCCGGCGGGGGCAUUGACAACGCUCCCCGCCAACCCUUAACGUUUCCCUCAUCGUAACCAUCACCGGUUCAGCUUAAGAGGUUGUCGGCCCGCUC[illegible]CUCGACACCUCCGCCUUCCUCGAAGGCCU
Bsp-3-1  CUCGUCGCACUCAAUCUCGGCGAUGCGGCGGCACGAUGAUCCAUACGCACGCAUCCAGCGGGGGCAUUGACAACGCUCACCGCCCCCCUUAACGUUUCCCUCAUCGUAACCAUCACCGGUUCAGCUUAAGAGGUUGUCGGCCCGCUC[illegible]CUCGACACCUCCGCCUUCCUCGAAGGCCUG
Bsp-2-1  CUCGUCGCACUCAAUCUCGGCGAUGCGGCGGCACGAUGAUCCAUACGCACGCAUCCGGCGGGGGCAUUGACAACGCUCACCGCCACCCCUUAACGUUUCCCUCAUCGUAACCAUCACCGGUUCAGCUUAAGAGGUUGUCGGCCCGCUC[illegible]CUCGACACCUCCGCCUUCCUCGAAGGCCU
Bsp-4-1  CUCGUCGCACUCAAUCUCGGCGAUGCGGCGGCACGAUGAUCCAUACGCACGCAUCCGGCGGGGGCAUUGACAACGCUCACCGCCACCCCUUAACGUUUCCCUCAUCGUAACCAUCACCGGUUCAGCUUAAGAGGUUGUCGGCCCGCUC[illegible]CUCGACACCUCCGCCUUCCUCGAAGGCCU
Bsp-16-1 CUCGUCGCGCUCAAUCUCGGCGACGCGGCGCCGCGCGGCUGAUCGCAUCUGCGACGCGCUCGUCGCGGCGACAGGUGGGCGGUGGCGCGUCGCUCGGCAUGGCGCGCCGAACAUUGACAACGCCCCCCGCCGCCCCUUAAUGUGGGUCGCAUCGUAAUCAUCACCGGUUCAGCUUAAGAGGUUG
Bsp-15-1 CUCGUCGCGCUCAAUCUCGGCGACGCGGCGCCGCGCGGCUGAUCGGCAUGCGCGGCGCUCGUCGCCGCAACGCGGCGGCAGGCGGCAGAUGACGGGUGCGCGAGCGCACCGUGCGGCAUGGUGCGCCGAACAUUGACAACGCCCUCCGCCGCCCCUUAAUGUGGGUCGCAUCGUAACCAUCACC
Bpu-1-1  CUCGUCGCGCUCAACCUCGGCGAGGCGGCGCGAUGAUCGGUGCGCACGCGUCCGGCGGGGCAUUGAGCAUUGACAACGCCCCCGGCGCCCCUUAACGUUGCUUCCAUCGCAACCAUCACCGGUUCAGCUUAAGAGGUUGUCGGCCCGCUC[illegible]CUCGACACCUCUGCCUUCAUCGAAGG
Bst-1-1  CUCGUCGCACUCAACCUCGGCGAUGCAGCGGCGCACUGACUUCGAUCGGCGCCAACCGUCGGCGCGGAUGUCGAGCGUCGGUGGCGGGAGGCAUUGACAACGCCCCCCGCCGCCCCUUAACGUUGCUCGCAUCGUAACCAUCACCGGUUCAGCUUAAGAGGUUGUCGGCCCGCUC[illegible]
Bsp-9-1  CUCGUUGCGCUCAACCUCGGCGAAGCGGCCACGCACUGACCGGUGCCGGGCCGGUCAUCGGCGCAUGGGCGCCUGGCGGGGCAUUGACAACACCCCGGGCGCCCCUUAACGUUUCGUCAUCGUAACCAUUACCGGUUCAGCUUAAGAGGUUGUCGGCCCGCUC[illegible]CUCGACACCUCU
Bvi-2-1  CUCGUCGCGCUCAACCUCGGCGAGGCGGCGACGCACUGACCGGUGGCGGGCCGGUCGUCGACGCAUGGGCGCCAGGGGCAUUGACAACGCCCCCGCGCGGCCCUUAACGUUUCGUCAUCGUAACCAUCACCGGUUCAGCUUAAGAGGUUGUCGGCCCGCUC[illegible]CUCGACACCUCUGCC
Bla-1-1  CUCGUCGCGCUCAACCUCGGUGAGGCGGCGACGCGCUGACCGGUGGCGGGCCGGUCGUCGACGCAUGGGCGCCAGUGGCAUUGACAAUGCCCCGCGCGGCCCUUAACGUUUCGUCAUCGUAACCAUCACCGGUUCAGCUUAAGAGGUUGUCGGCCCGCUC[illegible]CUCGACACCUCUGCC
Bce-4-1  CUCGUCGCGCUCAACCUCGGUGAGGCGGCAACGCGCUGACCGCUUUGGGUGUCAGCGGCCGAUACCGCGCGAAACGCGCGAUGCGCGCGGCAUUGACAGCUCAAAACGAUGCCGGUGCUGCGGCGUGCGAGCGAAACUGCGUCGACGGUUGCGCCAUCGGCGAGCCGCGUGAACGG
Bsp-21-1 CUCGUCGCGCUCAACCUCGGCGAGGCGGCGACGCACUGACCGGUGGCGGGCCGGUCGUCGACGCCUGGGCGCCAGGGGGCAUUGACACCGCCCCCGCGCGCCCCUUAACGUUUCGUCAUCGUAACCAUCACCGGUUCAGCUUAAGAGGUUGUCGGCCCGCUC[illegible]CUCGACACCUCCGC
Bsp-1-1  CUUGUCGCGCUCAACCUCGGCGACGCGGCGCACUGACCGCGAUCACCACCGGUUGCCGGCACGCGGGCAUCGGGGAGACAUUGACAACGCCCCCCGACGCCCCUUAACGUUGCUCCCAUCGUAACCAUCACCGGUUCAGCUUAAGAGGUUGUCGGCCCGCUC[illegible]CUCGACACCUCU
Bsp-14-1 CUGGUCGCGCUCAAUCUCGGGGAAGCGGCUACGCACUGAUCGUGACCGCCGCGCGGUUGACGGCACACGGGCGCCGGGGGCAUUGACAACCUCCCCCGGCGCCCCUUAACGUUGCUCGCAUCGUAACCAUCACCGGUUCAGCUUAAGAGGUUGUCGGCCCGCUC[illegible]CUCGACACUUCUGC
Bsp-12-1 CUGGUCGCGCUCAAUCUCGGGGAAGCGGCUACGCACUGACCGUGACCGCCGCGCGGUUGACGGCACACGGGCGCCGGGGGCAUUGACAACCUCCCCCGGCGCCCCUUAACGUUGCUCGCAUCGUAACCAUCACCGGUUCAGCUUAAGAGGUUGUCGGCCCGCUC[illegible]CUCGACACCUCUGC
Bse-1-1  CUCGUCGCGCUCAACCUCGGCGAUGCAGCGGCGCGGUGAUCGGCGCACGCGCAGGCGCCGCGGCGCGGCAUUGACAACGCCCCCGGCGCCCCCCUUAACGUUGCUCACAUCGUAACCAUCACCGGUUCAGCUUAAGAGGUUGUCGGCCCGCUC[illegible]CUCGACACCUCUGCCUUCCUCGAAGGCCUG
Bce-6-1  CUCGUCACGCUCAACCUCGGCGAUGCGGCAGCGCGAUGAGCGAUGCGCAGGCGCCCGGCGGGGCAUUGACAACGCCCCCCGGCGCCCCUUAACGUUGCUCCCAUCGUAACCAUCACCGGUUCAGCUUAAGAGGUUGUCGGCCCGCUC[illegible]CUCGACACCUCUGCCUUCAUCGAAGGCCUG
env-88   GCGGUGUUAUCGUGCUGAAUCUGGAUACCCACUGAUAUCAAUAUAUCGAUAAAAUACGUACCAAAACUCAGGAACCCGU[illegible]UAUUCAGCCACCUUUAUCUUCGACAAAAAACAAUUCGAUGAGGCGUUUUAUGCGCUUGACCAGGCCAUCGCCGACAUUGCCAAACAAACGGUGGGUUA
env-90   UUCCGGCGUAGUCCUGCUAAACCUAGAGGCCAGCCGUGACGCUAACCCUUCAAUCAACCGGACGUGCCCGGCAAGCCGGGUCACGCCGGUUAUCUCAAACGUUGGGCGUCACGGUGACGCGUUGGAAGAACC[illegible]CUGAGCGCACUGUUGCAGGCUCCGCCGCUUUCUGACGUUCGU
Rsp-1-1  UUUCGGCGUAGUCCUGCUGAACCUGGAGGCCAGCCGUGACGCUGGCCCUUCAAUCGAGGGGACAAACACCGGCUUGCCGGUGUUUGCCCUCUCAUCUAAAACCUUGGGCGCUGCAAGUGCAUUCA[illegible]CCGAUCGAGAAGAUCUACGCCAUGAAAACGCCGUUCGGUUUACUGCUGUUG
env-91   GCUGGGGGUCAUCAUGCUGAACCUGAGCGGCCGCGAUGAAGCUUGAACGCAGCCGCCGCGCCGCGGCCCGCC[illegible]ACCGGCCGCAACGGUGCCAUCCGCAUGGCCGACGGGACGCCGACGAUGCUGAUCGAUGCCCCCGAAUUCCCAGGCCCACCCACCACCGCCCAUUCGCCAAGGAU
env-93   ........................................................................................................
env-94   ........................................................................................................

         ........................................................................................................
         ........................................................................................................
         ........................................................................................................
         ........................................................................................................
         ........................................................................................................
```

```
env-95    ACAUGGGUGUGACCUACGCCGUCUGGGCCGCGAGCGGCACCGCAGCCACCGCCCUCGUCGGCAUCGCGUUCUACGGCGAGCAGAUGGGCGGCAUCAAGGCCUUGGGGCUUGUUUGCGUGGUCGGCGGCGUCGUCAUGCUCAAUCUCGGUAGUCACCGAUGACACCCAGAGAAACGUAGUG
env-96    GGGAGGCGUUGUCAUGCUCCACCUCGGAAGCCCUCAGUGAUGGCUUCUGGUGUUGAUCAAGGCCCACACCAACUCCACCCCGGCCGUCAGCAGAUCGCCCGUGAAUGAGCUGUCAUCGGCAUCGCUGGUGCUGGUAAACGCGCGCUUUUGCCGCUCGCGCUCGGCGCGCGUGCGGUCACG
env-97    GGGAGGCGUUGUCAUGCUCCACCUCGGAAGCCCUCAGUGAUGGCUUCUGGUGUUGAUCAAGGCCCACACCAACUCCACCCCGGCCGUCAGCAGGUCGCCCGUGAAUGAGCUGUCGCUCGCAUCACUGGCGCUGGUGAACGCGCGCUUUUGCCGCUCGCGCUCGGCGCGGCUGCGGUCGCG
env-99    GGGAGGCGUUGUCAUGCUCCACCUCGGAAGCCCUCAGUGAUGGCUUCUGGUGUUGAUCAAGGCCCACACCAACUCCACCCCGGCCGUCAGCAGAUCGCCUGUGAAUGAGCUGUCGCUCGCAUCACUGGCGCUGGUGAACGCGCGCUUUUGCCGCUCGCGCUCGGCGCGGCUGCGGUCGCG
env-101   ..........................................................................................................................................................................................
Asp-9-1   GGGAGGCGUUGUCAUGCUCCACCUCGGAAGCCCUCAGUGAUGGCUUCUGGUGUUGAUCAAGGCCCACACCAAUUCCACCCCGGCCGUCAGCAGAUCGCCCGUGAAUGAGCUGUCGCUCGCAUCACUGGCGCUGGUGAACGCGCGCUUUUGCCGCUCGCGUUCGGCGCGGCUGCGGUCGCG
env-102   ..........................................................................................................................................................................................
env-103   UUGGCGAGCCAGUGGGUGUCAUCAAAGUAGUGGGUCUGGUGUGCGUGGUGGGAGGCGUUGUCAUGCUCCACCUCGGAAGCCCUCAGUGAUGGCUUCUGGUGUUGAUCAAGGCCCACACCAAUUCCACCCCGGCCGUCAGCAGAUCGCCCGUGAAUGAGCUGUCGCUCGCAUCACUGGCGC
env-104   GGGAGGCGUUGUCAUGCUCCACCUCGGAAGCCCUCAGUGAUGGCUUCUGGUGUUGAUCAAGGCCCACACCAAUUCCACCCCGGCCGUCAGCAGAUCGCCCGUGAAUGAGCUGUCGCUCGCAUCACUGGCGCUGGUGAACGCGCGCUUUUGCCGCUCGCGUUCGGCGCGGCUGCGGUCGCG
env-106   GCGCUCAAGUACUCCGAUGGCUUCUCCAGACUCCUCCCGUCCGGCAUGGCAGCUGCGUGCUACGCACUCGCCAUCUGGCUCAUGGCCGUGGCCAUGCGCCAGCUCGACAUGGGCCUCACCUAUGCAGUCUGGGCGGCCAGCGGCACGGCGGCCGCAGCCGUGGUGGGAUGGCGCUGUUU
env-107   GGGAGGCGUUGUCAUGCUCCACCUCGGAAGCCCUCAGUGAUGGCUUCUGGUGUUGAUCAAGGCCCACACCAACUCCACCCCGGCCGUCAGCAGAUCGCCCGUGAAUGAGCUGUCGCUCGCAUCAUUGGCGCUGGUGAACGCGCGCUUUUGCCGCUCGCGCUCGGCGCGGCUGCGGUCGCG
env-110   ..........................................................................................................................................................................................
env-111   ..........................................................................................................................................................................................
env-112   GGGAGGCGUUGUCAUGCUCCACCUCGGAAGCCCUCAGUGAUGGCUUCUGGUGUUGAUCAAGGCCCACACCAAUUCCACCCCGGCCGUCAGCAGAUCGCCCGUGAAUGAGCUGUCGCUCGCAUCACUGGCGCUGGUGAACGCGCGCUUUUGCCGCUCGCGUUCGGCGCGGCUGCGGUCGCG
env-113   GGG.......................................................................................................................................................................................
Asp-5-1   GGGAGGCGUUGUGAUGCUCCACCUUGGAAACCCUCAGUGAUGGCUCCUGGUGUUGAUCACCGCCCACACCAAGGCCACCCCGGCCGACAGCAGGUCGCCCGUGAAUGAACUGUCAUCGGCAUCGCUGGUGCCGGUAAACGCCCGCUUUUGCCGCUCACGCUCGGCGCGCGUGCGGUCGCG
Ara-1-1   GGGAGGCGUUGUGAUGCUCCACCUUGGAAACCCUCAGUGAUGGCUCCUGGUGUUGAUCACCGCCCACACCAAGGCCACCCCGGCCGACAGCAGGUCGCCCGUGAAUGAACUGUCAUCGGCAUCGCUGGUGCCGGUAAACGCCCGCUUUUGCCGCUCACGCUCGGCGCGCGUGCGGUCGCG
Asp-6-1   GGGAGGCGUUGUGAUGCUCCACCUUGGAAACCCUCAGUGAUGGCUCCUGGUGUUGAUCACCGCCCACACCAAGGCCACCCCGGCCGACAGCAGGUCGCCCGUGAAUGAACUGUCAUCGGCAUCGCUGGUGCCGGUAAACGCCCGCUUUUGCCGCUCACGCUCGGCGCGCGUGCGGUCGCG
env-114   ..........................................................................................................................................................................................
env-115   UGGCGGCGUCGUCAUGCUAAAUCUCGGCGCUCAGCGAUGAUGCCUAACCCAUAUUUCAAUCAUGCCCGCUACGGCAAGAGCCGCAAGGCCCGCCUUAGGCACAUGGAGCAUCAUCUCGGCCCGGGCUUACGGCGCUUCCCAUUGAGCUGACGCGCCUUCUAAAAUGCAUGAGAACGAUGC
env-117   ..........................................................................................................................................................................................
env-118   GGCCGGCGUCGUCAUGCUCAAUCUGGGCGGUCAACGUUGACGCCCGAAGGGACUCCUCCGUCCGCGUCACCCCGGCUACGAGGCCCUGGCCGGCGGCUGCCAGCGGAAGAACAUCGGGUAGACCUUCUCCACCACCGGGCCGUCGAAGUCCCAGGCCAGGCACUGGCCCAGGUGGCCGG
env-120   UGGCGGCGUCGUCAUGCUCAAUCUCGGCAACCAGCAAUGACGCCUGAGCUUCGCUCAAGCCGUUCAGCUUGAACGUUGAACGUCCGCUUCAAAAGUACCCGACCGCCUGCUACUGGACGACCGCAAACACCGUAGCCCAGUGAAAGCAUUGAAUCAUUCGUGAAUCAAGCGAACUGAGCA
env-125   UGGCGGC...................................................................................................................................................................................
Psp-1-1   UGCGGGCGUCGUGAUGCUCAAUCUUGGUGGUCAGCGAUGACUAUUAGCCCUUGGUACGGACGCCCACAAGCGCAAGAAGCUGACGGAUAGAUAGGCUUCCUGGGAAGCGCCUGCAGUCGUAUAUCAGGCAUCUAUGGCGGGCUCUGCCGUGGGACGCGUGUCCGGUUUAUCCCCAAGCUG
env-126   ..........................................................................................................................................................................................
env-127   ..........................................................................................................................................................................................
env-128   ..........................................................................................................................................................................................
env-129   ..........................................................................................................................................................................................
Hfl-1-1   GGUGGGUGUUGCGCUGCUCAACCUCGGCGAACGGGCUUGACACCCAUCAACCGACCCAUCGACCCCGUCACAGCCCAUGGCCGCUAGUCUCCCACUCCGAAUCGCCGCCGCGCAGUCCUCGUCCCUCCCCGGUGAUGUGCGUGGGAACCUGUGCCGACACCUGCCGUUCGUCGAAGCCGC
Hsp-1-1   CUGGGCGUGGUCUUGCUGAACCUGGGGGCCAAGGCGUGACGCCCGGCCCCUUUGCAGGACCAUCCAAGGAAGGCCCGUGGCGCCGACGCCGGGCUCCCUUGAACCACAAACCGCUGAAAGGACCGUUAUGGAACUGCUUGACACCCUGAAACGACGCUUCGAAAAACACAUGGAUCGGCA
env-130   AUCGGCAUUGUCCUGCUGAAUCUCGGCGCCAGGCAGUAACGCAGUCCCAGGCGGUAUCCUUCCACCCAACGGGCAGUCAAGACCAUAACGAAGGACCGACAUGAGCAUGAAACCAGGAAUCGUCGCGGCGUUGGGUCUUGCCACAGUACUUGCCGGCCUUGAGGCCCGCGCCGCAGAGCC
env-131   CUGGGUGUGAUCGUGCUGAGCUCGGGGGCCGGGUAGCCGCGCCCGCAUGCGCUCAGGCCAUCGUCGUCGGUUCCGCCUUCGGAGUCGCCGCGCGUGCUGCCGUCCAUGAGCGCCGAUGACGAGGAACGGACGAAGCGGUCGAGGACACGUCGCCAGAACGCGAACAGGUGCCCCACGGUG
env-132   CUGGGUGUGAUCGUGCUGAGCUCGGGGGCCGGGUAGCCGCGCCCGCAUGCGCUCAGGCCAUCGUCGUCGGUUCCGCCUUCGGAGUCGCCGCGCGUGCUGCCGUCCAUGAGCGCCGAUGACGAGGAACGGACGAAGCGGUCGAGGACACGUCGCCAGAACGCGAACAGGUGCCCCACGGUG
env-133   CGAUAUGGCUGAUGUCACUGUCACUUAAACAUAUUGAAAUGGGCAUUACCUACGCGGUGUGGGCAGGCAGCGCCACAGCCAUUCUGGCAAUGGUGGGCAUUGCGCUUUAUGGUGAGUCUCUUAGCGUGUUCAAGAUGGUCGGUAUGGUGAUGGUAGUAGGCGGUGUUAUCGUGCUGAAUC
env-134   ..........................................................................................................................................................................................
env-135   CGAUAUGGCUGAUGUCACUGUCACUUAAACAUAUUGAAAUGGGCAUUACCUACGCGGUGUGGGCAGGCAGCGCCACAGCCAUUCUGGCAAUGGUGGGCAUUGCGCUUUAUGGUGAGUCUCUUAGCGUGUUCAAGAUGGUCGGUAUGGUGAUGGUAGUAGGCGGUGUUAUCGUGCUGAAUC
env-136   GGCGGUGUUAUCGUGCUGAAUCUGGAUACCCACUGACAUCAAUAAUCGAUAAAAUACUUACCAAAACUCAGGAGCCCCUAUGUAUUCAGCCACCUUUAUCUUCGACAAAAAACAGUUCGAUGAGGCGUUUUAUGCGCUUGACCAGGCCAUCGCCGAUAUCGCCAAACAAACGGCGGCUA
Bte-1-1   UAGUAGGCGUGGCUACAUUGAACCUCGGUUCUUGAUCAUGACGGCGUUAUCAAAGCCCGGCGAAUCGGUUCCGGUCUCACGCGGAAGAAAUGAGCGAACGAUCUCGCGAGUCGGGCAAAUGGAUGCUCUGCUGUCUCGCCGUGCCCGUGAACUUGGCAAAACCGUCAAGGAAUUGGCUAC
Bce-5-1   CUCGUCGCGCUCAACCUCGGCGAGGCGGCGACGCACUGACCGGUGGCCUGCCGGUCGUCGACGCAUGGGCGCCAGGUGGCAUUGACACCGCCCCCGCGCGCCCCUUAACGUUUCGUUCAUCGUAACCAUCACCGGUUCAGCUUAAGAGGUUGUCGGCCCGCUCGUGCUCGACACCUCCGC
          ..........................................................................................................................................................................................
          ..........................................................................................................................................................................................
          ..........................................................................................................................................................................................
          ..........................................................................................................................................................................................
          oooooooooooooooooooooooooooooooooooooooooooooooooooooooooooooooooooooooooooooooooooooooooooooooooooooooooooooooooooooooooooooooooooooooooooooooooooooooooooooooooooooooooooooooooooooooooooooooo
```

alignment positions 721 ··· 828

```
env-1     GUUAUAUAGGCGAGGAAGCCUGGGAAAACCCUGAAACUGGGCGCAUUUCCAAUGUCUAUUACUGGGAAAACAUGGAUGGCUUGCAGGCGUUGAUGAAGCACCCUAAAC
Ama-1-1   UUACUGCCUACCAAGAAGACAAAUCACCAGAUAGUGGGAUACGUUAAUGUUCCUGAUUCAAGCCAAGAGAAGCCAUGACUCUGGCGGCAUUCAGCUUGCAGUCCAUGU
Ama-1-2   GGACGGCGAACCGGGACUUGACUACCCUCCUCCAUCACAGGGAACGGUAGCAGGAAGGAAGCGUUAGAUUUUUGUGCGCCGGUGACAAAAAAAGCGCAAAUAGUGCCA
env-2     GCGCCGACGCGAAUCACCAGCUUGCCGUUGUUCUUGCCCUGCAGUAGCCCGAUAAAUGCCUGCGGCGCAUUUUCCAGUCCGUCGACCACGUCUUCGCGAUAUUUGAUU
Bsp-17-1  GGGCAACCGCACUGGUUGGUGUGACGAUCCAAUGGGGUUGGUCGUUCGUCGCCAUCCACGAAUCCCUAACGACCUUGAGCGCCACUGCGUUCAAUGCAUUCCGCUUUC
env-3     GGGCAACCGCACUGGUUGGUGUGACGAUCCUAUGGGGUUGGUCGUUCGUCGCCAUCCACGAAUCCCUAACGACCUUGAGCGCCACUGCGUUCAAUGCAUUCCGCUUUC
Pfu-2-1   GGGCAACCGCACUGGUUGGUGUGACGAUCCUAUGGGGUUGGUCGUUCGUCGCCAUCCACGAAUCCCUAACGACCUUGAGCGCCACUGCGUUCAAUGCAUUCCGCUUUC
Pph-1-1   GGGCAACCGCACUGGUUGGCGUGACGAUCCUAUGGGGCUGGUCGUUCGUCGCCAUCCACGAAUCCCUAUCGACCUUGAGCGCCACUGCGUUCAAUGCAUUCCGUUUUC
Bce-1-1   GACCAUACUCUGGGGCUGGUCAUUUGUCGCAAUUCAUCAAUCUCUAGGGUUCGUCAGUGCAUCAACGUUUAACGCAUAUCGCUUCCUGAUCGGCGCAGCGGCGAUGUU
Rgu-1-1   AACACGCCCCCGUGUCGCUGUGGCUCGAGGACCUCAGCGCGAUCAAGGCGCUGUUCGAUGCGUGGCGAGCCGCGGGCGUGUCGGAUGUCGAAGCGCACUUUCGCGAUC
Hta-1-1   CACACAACGCUGUCGCAAGGCCCGUGCGUUAUGUCGUGAUCGAUACAGGAGCCCGUCAUGUCCCAACCGUUGUACAGCCCGUUGCCCAACGCCCGCCCGUGCGUGGUC
Vpa-1-1   AAGCUUGCCGACGGGACGAUUCGCGAUCCUUCGGUACACACGUUGCUUCGUCUUGCCGCGGCAAUGGAAGUCUCGCCAAUUGCGCUAAUUCGACUCUAUGGAGACAUA
Aur-1-1   CGCUCGAUCAGCGACUCCAGCGCGGCGAUGGCGGAGUGGUCGGCCAGGUGCAGGUGGCGGCAAUCCAGCGUGACGUCGUGCGGGUCAGCGGCGGGUUCAAACAUCGCC
env-6     CCUCCGUCGGCACUUUGGUGCGACUGGCCGGUGCGCUCGAAGUGUCGCCCAUCGCCCUGCUACGGCACUACGCGCAAUUAGCCAUGCCGGAGCAACGAGCCCCCCGCA
Vap-1-1   AAGACCGCGCGGUCUUGGACGAUAUAUGCAGCAAGGGGCAGCAUCGUGCGCGCGAGGUCAGGCGCGCGCACGUGCUGUCCUGCCUGGACCGUGGCGUGCCGGAGUCCC
Rsp-2-1   UGCCCGGGCGCCGGGUGGUCCGGGAACGUGGGCACCACGGUGACGAUCUGGUCGCCGCGCUCGGCGCCGUCGUGGUCCAGCGGCCAGCCCUGGCCGCGCAGGCGGUAC
Bce-1-2   CGUGACCAUACUCUGGGGCUGGUCAUUUGUCGCAAUUCAUCAAUCUCUAGGGUUCGUCAGUGCAUCAACGUUUAACGCAUAUCGCUUCCUGAUCGGCGCAGCGGCGAU
Hsp-3-1   CGCGAGGGCGCGUUGACUAUUGAACGUGCCACUCUUAUCCCAAGCGACACCCCGUCCUUGAGCAAAGGCUGCACGGUACUUGCGCAUCAUAUUGUGCGGGUCGCGGGC
env-7     GAAUAGCAAGGCCGCUAUAGGCGAGAAGCCGACUUCCACUGACUAAAACAAACGCCCGCAUUGCGCGGGCGUUGCUAUAUUCAGAUCAUGCGCGGGAUUUAUUAUUCA
Hsp-2-1   GAAUAGCAAGGCCGCUAUAGGCGAGAAGCCGACUUCCACUGACUAAAACAAACGCCCGCAUUGCGCGGGCGUUGCUAUAUUCAGAUCAUGCGCGGGAUUUAUUAUUCA
env-8     UGUGGCACGCACCUAUCUCUACAAGCUACUGGCCGGCAAAACGCAUGAUCCCUCGAUUCGGACGCUGAUUCGAUUGGCGGCUGCCAUUGAGGUUUCUCCCAUACUGCU
env-9     UUUGUAUACGGGCGAUUGAAAAAAACAGCACUACAUCAUUAUAUCCAAGCUGGAAUUGCCGGUGGNNNNNNNNNNUUGCGCUGCUGAUAUUAAAAGUUAAGCCCACGU
Asp-4-1   UGGCCUCGACGGGGUUGCGCGCGUGCAGCAGCACGAAGUCGGCAUGGCAGCCGACGGCAAGGCCGUAGCCUUCCAGGCCCAGCAGUUUUGCGGGAUUGGUGGUCACCG
Asp-3-1   UGGCCUCGACGGGGUUGCGCGCGUGCAGCAGCACGAAGUCGGCAUGGCAGCCGACGGCAAGGCCGUAGCCUUCCAGGCCCAGCAGUUUUGCGGGAUUGGUGGUCACCG
env-10    UGCGCUGAGUUGCGCCACCAGAAAAUCCACCAUGGUCCUGAGCUUCGGGGGCACCACGCGCUGCGGUUGCCACAAAGCCCAGGCUGUCGCACCAAAUGCGGGCUGAGG
Awa-1-1   GGCAGCCGAUAGCGGUGUGGACCCGUCUAUUGGCACGCUGGCCCGGUUGGCCCAUGCGCUGCAGGUAUCUCCUGUUGCCGUGUUACGGCACUAUGUGGCGUUGGGCAU
Ama-1-3   GGUUUUCGAUUUUAUGGAUAUUAUUUUACAAAGUUUGAUCACAAAGGACGAGAAUGACCAGAACAAGGACACCACCCGUAGCAGCGCACCCGCUGUUCAGCUUGCAGG
env-11    CCGCCCGUCUGGGGCCGGGCGCGCCAGUGCGCGCUUCGUUGACUGACUCGCGGCCACUGUUUGAGCAGAGAGAGCGCAGCGAACGGCGCGAGUUUGGCCGCGCCGGCC
env-15    CCGCCCGUCUGGGGCCGGGCGCGCCAGUGCGCGCUUCGUUGACUGACUCGCGGC......................................................
env-16    CCGCCCGUCUGGGGCCGGGCGCGCCAGUGCGCGCUUCGUUGACUGACUCGCGGCCACUGUUUGAGCAGAGAGAGCGCAGCGAACGGCGCGAGUUUGGCCGCGCCGGCC
env-18    ............................................................................................................

          ............................................................................................................
          ............................................................................................................
          ............................................................................................................
          ............................................................................................................
          oooooooooooooooooooooooooooooooooooooooooooooooooooooooooooooooooooooooooooooooooooooooooooooooooooooooooooo
```

```
env-19     ....................................................................................................
env-20     ....................................................................................................
env-21     GCUGCGCUCUCUCCGCUCAGACAGUGGCGACGAGUCAGUCGAAGAUGCGCGCUGCGCGCGCGGCACAACCCCUGCGCUUCUCGGCGCCUCACAAGGCGCGCUGCGGCC
env-22     ....................................................................................................
Bsp-20-1   GUCUGCGUGACCGUACUCUGGGGCUGGUCGUUCGUCGCGAUCCAUGAAUCCCUCGCGGCCGUCAGCGCAUCGGCAUUCAAUGCGUAUCGCUUCCUGAUCGGCGCCGCG
Bpa-1-1    UGCGUGACCGUACUCUGGGGCUGGUCGUUCGUCGCGAUCCAUGAAUCCCUCGCGGCCGUCAGCGCAUCGGCAUUCAAUGCGUAUCGCUUCCUGAUCGGCGCCGCGGCG
Bsp-8-1    CUGCUGCUUGGCGCCGGCCUGUUCACGUCGGUCGGCCCGAAGGACGCCUUCGUCAUCAAGCGCUCGAUUUCGAGCCCUCACCUGCUCUCCAUCGCCCUCGUCUGCGCG
Pac-1-1    ACCUGGCGGUCGCUUCUUCGACCGUCAUGUUGUGCAACGGUGCCCAAUGCUCUAGAUACGAUUGCCGCCGACCGGUCAUCACCGGGCCGGGCAAUACGCUGUUGACCU
Pme-1-1    ACCUGGCGGUCGCUUCUUCGACCGUCAUGUUGUGCAGCGGCGCCCAAUGCUCGAGAUACGAUUGCCGCCGACCAGUCAUCACAGGGCCGGGCAAUACGCUGUUGACCU
env-23     ....................................................................................................
Ptr-1-1    CACGCGCCUCGAAGCCCGUUCGCGCGCGAGCGAUCCACGCCGCGCCGAGCGCGCGAUCGCGCGGGACGCCAUUGCCGGUCAGAAAGCGCAUGCCGAUGUCGUACUGCA
Ptr-1-3    CACGCGCCUCGAAGCCCGUUCGCGCGCGAGCGAUCCACGCCGCGCCGAGCGCGCGAUCGCGCGGGACGCCAUUGCCGGUCAGAAAGCGCAUGCCGAUGUCGUACUGCA
Bub-1-1    UAGAUCGCUGACAAUCGGGGCGCAUUCACCAAAUUGGUAGUUGCGGUCCUGAACGCUCCGGUACGUAGAAUUUUGAAUUUCACGUGGUGGCGCAUGCAUGACUACCUA
Bsp-24-1   UAGAUCGCUGACAAUCGGGGCGCAUUCACCAAAUUGGUAGUUGCGGUCCUGAACGCUCCGGUACGUAGAAUUUUGAAUUUCACGUGGUGGCGCAUGCAUGACUACCUA
Bvi-3-1    AGAUCGCUGACAAUCGGGGCGCAUUCACCAAAUUGGUAGUUGCGGUCCUGAACGCUCCGGUACGUAGAAUUUUGAAUUUCACGUGGUGGCGCAUGCAUGACUACCUAA
Bvi-1-3    CCCAGCGCCUGCUUGUCAUCCCGAAUCAGCCACCCGGACGCACAAUUGGAAGCGCAUGGAGCAAGAUCGUUGCCACAGAAAGGCUCCGUCGAUGGGCGAAUUUUGUCU
Bsp-18-1   CCCAGCGCCUGCUUGUCAUCCCGAAUCAGCCACCCGGACGCACAAUUGGAAGCGCAUGGAGCAAGAUCGUUGCCACAGAAAGGCUCCGUCGAUGGGCGAAUUUUGUCU
env-24     AUCACGUCAAAUUCGGAGUCACCGACUAUGCGGCAAGCAAGGCGUUCUAUCUCGCAGCGCUCGAACCGCUGGGCGUAGCGGUGGUCUCCGAGGGCGACCCGACCUACG
Pfe-1-1    AUCACGUCAAAUUCGGAGUCACCGACUAUGCGGCAAGCAAGGCGUUCUAUCUCGCAGCGCUCGAACCGCUGGGCGUAGCGGUGGUCUCCGAGGGCGACCCGACCUACG
env-25     AGGCGUUCUAUCUCGCAGCGCUCGAACCGCUGGGCGUAGCGGUGGUCUCCGAGGGCGACCCGACCUACGGUGUGGAGUUGAGCCCGCCGGGCCAGCCUUCGCUGUGUC
env-26     ....................................................................................................
Bsp-22-1   AGUCGGCGGGAAUACGCGCCAACUGCCGUCAUCGUGCCUGAAGAAGAACAAUGCCCGCGCUCCGGCUGGUGACGACGUCUCGACGCACACGUAGCGUCGCCCAUUCCA
env-27     ....................................................................................................
env-28     GCAACCGCCUUGGUUGGCGUGACAAUCCUUUGGGGCUGGUCGUUCGUCUCCAUCCAUGAAUCGCUGUCAACUUUGAGCGCCACUGCGUUCAAUGCAUUCCGCUUUCUC
env-29     AUGGGGAGCGAUACCCGGCCGGAGUAAAUACGUAAUGAAAAUUAAUCGUCUCGAAACAUUCAGCAACAGGCACGUUGGCUUUGUGCGUGCCACCACCGAUACC.....
env-30     CCCACAGCAGUCUCUGGCACGCGUACAAAGCAGACAUUUGCGCAUUCGUGCCUCCGGUGUUGGAUUUCGCUGAGAUCUGACCCAGGUUUUCCAUCAGAUACUGCCCCA
env-31     ....................................................................................................
env-32     ....................................................................................................
env-33     ....................................................................................................
env-34     GACUUUUUUGCCUUAAGCCGAGCAGCUUCGAGGACUCAAUGCCGGCAAGCCAGAACCCCGUUUUUGCUUUGGUGUUGCGUCCUUGGGGCGCGAUCCAAACANNNNNNN
env-35     GACUUUUUUGCCUUAAGCCGAGCAGCUUCGAGGACUCAAUGCCGGCAAGCCAGAACCCCGUUUUUGCUUUGGUGUUGCGUCCUUGGGGCGCGAUCCAAACAGGCCCUA
env-36     ....................................................................................................
env-37     ....................................................................................................
env-38     ....................................................................................................
env-39     ....................................................................................................
env-40     ....................................................................................................
env-41     ....................................................................................................
env-42     ....................................................................................................
env-44     ....................................................................................................
env-45     ....................................................................................................
env-46     ....................................................................................................
env-47     ....................................................................................................
env-48     ....................................................................................................
env-49     ....................................................................................................
env-50     ....................................................................................................
env-51     CGGCGGUGCUCGGAAUGCUCUGGUUUGGUGAGUCGGCGAGCUUCCUUCGACUGGCC................................................
env-52     ....................................................................................................
env-53     GACUUUUUUGCCUUAAGCCGAGCAGCUUCGAGGACUCAAUGCCGGCAAGCCAGAACCCCGUUUUUGCUUUGGUGUUGCGUCCUUGGGGCGCGAUCCAAACAGGCCCUA
env-61     ....................................................................................................
env-62     ....................................................................................................
env-63     CCAAACAAGCCCUACGCAGCCAAAACAUCCUG............................................................................
env-64     ....................................................................................................
env-65     ....................................................................................................
env-66     CCAAACAAGCCCUACGCAGCCAAAACAUCCUGCAACGCCCGCGUGGCAGCCACCGCCCCCAGGCUCGACUCGGUAAACCCGCCAUGCAGCACCACCCCGGUUGAUGCC
env-70     CAGCGAGCGGCUACAAAGGGACCGAAGCGGACUUGUUUGCUCUAAGCCGAGCAGCGUCAAGGCCCCAA........................................
Asp-8-1    CCAAACAAGCCCUACGCAGCCAAAACAUCCUGCAACGCCCGCGUGGCAGCCACCGCCCCCAGGCUCGACUCGGUAAACCCGCCAUGCAGCACCACCCCGGUUGAUGCC
env-71     UGGGGCGCGAUCCAAACAGGUCCUACGCCGCCAGCACAUCCUGCAGCGCCCGCGUGGC..................................................
env-72     UGGGGCGCGAUCCAAACAGGUCCUACGCCGCCAGCACAUCCUGCAGCGCCCGCGUGGCAGCCACCGCCCCCAGGCUGGAUUCGGUGAAGCCGCCAUGCAGCACCACUC
env-73     ....................................................................................................
env-74     GGGUAUGCGAUCCAAACAGGCCCUACGCCGCCAGCACAUCCUGCAGCGCCCGCGUGGCAGCCACCGCCCCCAGGCUGGAUUCGGUGAAGCCGCCAUGCAGCACCACCC
env-75     ....................................................................................................
Asp-2-1    GGGUAUGCGAUCCAAACAGGCCCUACGCAGCCAAAACAUCCUGCAGCGCCCGCGUGGCAGCCACCGCCCCCAGGCUCGACUCGGUAAACCCGCCAUACAGCACCACCC
Asp-7-1    UGGGGCGCGAUCCAAACAGGUCCUACGCCGCCAGCACAUCCUGCAGCGCCCGCGUGGCAGCCACCGCCCCCAGGCUGGAUUCGGUGAAGCCGCCAUGCAGCACCACUC
env-76     ....................................................................................................
env-77     UGGGGCGCGAUCCAAACAGGCCCUACGCCGCCCGCACAUCCUGCAGCGCCCGCGUGGCAGCCACCGCCCCCAGGCUGGAUUCGGUAAACCCGCCAUGCAGCACCACCC
env-78     ....................................................................................................
env-79     ....................................................................................................
env-80     ....................................................................................................
env-81     ....................................................................................................
env-82     UGGGGCGCGAUCCAAACAGGCCCUACGCCGCCAGCACAUCCUGCAGCGCCCGCGUGGCAGCCACCGCCCCCAGGCUGGAU............................
env-83     GGGUAUGCGAUCCAAACAGGCCCUACGCAGCCAAAACAUCCUGCAGCGCCCGCGUGGCAGCCACCGCCCCCAGGCUCGACUCGGUAAACCCGCCAUGCAGCACCACCC
env-84     ....................................................................................................
env-85     GGCUUCGGACACCGUCAUGGACCCGUCCAUCGGCACACUGAUGCGCCUGGCCCGUGCCUUGGAGGUGUCGCCUGUGGCGGUAUUCAGGCACUACGUGAGCUUGGGCAU
Vsp-4-1    GUGGAGGUGAUCAGCCCGCCGAUCACCGCCAUCGCCAUCGGCGAGCGGAAGCUCGAAUCCGCCGUGCCGAAGCCCACCGCAAUGGGCAGCAUGCCCGCGCCCAUGGCC
Vsp-5-1    GUGGAGGUGAUCAGCCCGCCGAUCACCGCCAUCGCCAUCGGCGAGCGGAAGCUUGAAUCCGCCGUGCCGAAGCCCACCGCAAUGGGCAGCAUGCCCGCGCCCAUGGCC
env-87     ....................................................................................................
Cmi-1-1    CGCCUGAGCGUCAUCAAUCUUUUCAUAGCAGUCCUGAUCAUUAUCGCCAUGGUCAUGACCGAGUACAGGCCAAGGAUGCGUGCCGUGUCGAGUUAGGAUAGUGUGACU
[me-1-1    GCCGCACGACGACUCGAUCGCCCCCAGCAUCCGCCAGUUGCCGGCCAGCGCGAGACCCGACGCGCCGCGCGUCGCCAUCGAAUCGAUCGAGUAGAACAGCGCGUCGCC
Bce-1-3    UGGCGCGUGCUCGCCCCGUUCGUCGCGGCGAUGAUGCUCGGCUUCGAGAUCCGCUUCGCGCUCGACGCACGUGAAGCCGGUCACGCCGGUCAGCGGCCGGCUAUCGCG
Bps-1-1    UGGCGCGUGCUCGCCCCGUUCGUCGCGGCGAUGAUGCUCGGCUUCGAGAUCCGCUUCGCGCUCGACGCACGUGAAGCCGGUCACGCCGGUCAGCGGCCGGCUAUCGCG
Bsp-5-1    UGCUGCUUGGCGCCGGCUUGUUCACGUCGGUCGGCCCGAAGGACGCCUUCGUCAUCAAGCGCUCGAUUUCGAGCCCUCACCUGCUCUCCAUCGCCCUCGUCUGCGCGG
Bsp-7-1    CUGCUGCUUGGCGCCGGCCUGUUCACGUCGGUCGGCCCGAAGGACGCCUUCGUCAUCAAGCGCUCGAUUUCGAGCCCUCACCUGCUCUCCAUCGCCCUCGUCUGCGCG

           ....................................................................................................
           ....................................................................................................
           ....................................................................................................
           ....................................................................................................
           oooooooooooooooooooooooooooooooooooooooooooooooooooooooooooooooooooooooooooooooooooooooooooooooooooo
```

```
Bsp-6-1    GCUGCUUGGCGCCGGCCUGUUCACGUCGGUCGGUCCGAAGGACGCCUUCGUCAUCAAGCGCUCGAUUGCGAGCCCUCACCUGCUCUCCAUCGCCCUCGUCUGCGCGGG
Bsp-3-1    CUGCUUGGCGCCGGCCUGUUCACGUCGGUCGGUCCGAAGGACGCCUUCGUCAUCAAGCGCUCGAUUUCGGGCCCUCACCUGCUCUCCAUCGCCCUCGUCUGCGCGGGC
Bsp-2-1    GCUGCUUGGCGCCGGCCUGUUCACGUCGGUCGGUCCGAAGGACGCCUUCGUCAUCAAGCGCUCGAUUUCGGGCCCUCACCUGCUCUCCAUCGCCCUCGUCUGCGCGGG
Bsp-4-1    GCUGCUUGGCGCCGGCCUGUUCACGUCGGUCGGUCCGAAGGACGCCUUCGUCAUCAAGCGCUCGAUUUCGGGCCCUCACCUGCUCUCCAUCGCCCUCGUCUGCGCGGG
Bsp-16-1   UCGGCCCGCUCGUGCUCGACACCUCUGCCUUCCUCGAAGGUCUGCUGCUUGGCGCCGGCCUGUUCACGUCGGUCGGCCCCAAGGACGCCUUCGUCAUCAUGCGCUCGA
Bsp-15-1   GGUUCAGCUUAAGAGGUUGUCGGCCCGCUCGUGCUCGACACCUCUGCCUUCCUGGAAGGUCUGCUGCUUGGCGCCGGCCUGUUCACGUCGGUCGGCCCCAAGGACGCC
Bpu-1-1    CCUGCUGCUUGGCGCCGGACUGUUCACGUCCGUCGGCCCCAAGGACGCGUUCGUCAUCAAGCGCUCGAUCUCGAGCCCCCAUCUGCUUUCCAUCGCCCUCGUCUGCGC
Bst-1-1    CUCGACACCUCCGCCUUCCUCGAAGGCCUGCUGCUUGGCGCCGGCCUGUUCACGUCGGUCGGCCCCAAAGACGCCUUCGUCAUCAAGCGCUCGAUCUCGAGCCCCCAU
Bsp-9-1    GCCUUCGCCGAAGGCCUGCUGCUUGGCGCCGCCCUGUUCACGUCGGUCGGCCCCAAGGACGCCUUCGUCAUCAAGCGCUCGAUCUCGAGCCCCCAUCUGCUCUCCAUC
Bvi-2-1    UUCAUCGAAGGCCUGCUGCUUGGCGCCGGCCUGUUCACGUCGGUCGGCCCCAAGGACGCCUUCGUCAUCAAGCGCUCGAUCUCGAGCCCCCAUCUCCUCUCCAUCGCC
Bla-1-1    UUCAUCGAAGGCCUGCUGCUUGGCGCCGGCCUGUUCACGUCGGUCGGCCCCAAGGACGCCUUCGUCAUCAAGCGCUCGAUCUCGAGCCCCCAUCUCUUCUCCAUCGCC
Bce-4-1    AUCGAACGCGAACGGAUUGACUUCCGCACCGCCGAGCCCGAGCGCAGCGACGACGUGCAGCGAGAACAGGUGGCCGCCAUGUGGCCAGAACGCGUCGCGCCGCCAGCC
Bsp-21-1   CUUCAUCGAAGGCCUGCUGCUUGGCGCCGGCCUGUUCACGUCGGUCGGCCCCAAGGACGCCUUCGUCAUCAAGCGCUCGAUCUCGAGCCCCCAUCUCCUCUCCAUCGC
Bsp-1-1    GCCUUCAUCGAAGGCCUGCUGCUUGGCGCCGGACUGUUCACGUCCGUCGGCCCCAAGGACGCAUUCGUCAUCAAGCGCUCGAUCUCGAGCCCCCAUCUGCUUUCCAUU
Bsp-14-1   CUUCCUCGAAGGCCUGCUGCUUGGCGCCGGACUGUUCACGUCCGUCGGCCCCAAGGACGCUUUCGUCAUCAAACGCUCGAUCUCGAGCCCCCAUCUGCUUUCCAUCGC
Bsp-12-1   CUUCCUCGAAGGCCUGCUGCUUGGCGCCGGACUGUUCACGUCCGUCGGCCCCAAGGACGCUUUCGUCAUCAAGCGCUCGAUCUCGAGCCCCCAUCUGCUUUCCAUCGC
Bse-1-1    CUGCUUGGCGCCGGCCUGUUCACGUCGGUCGGCCCCAAGGACGCCUUCGUCAUCAAGCGCUCGCUCUCGAGCCCUCACCUGCUCUCCAUCGCCCUCGUCUGCGCAGGC
Bce-6-1    CUGCUUGGCGCCGGCCUGUUCACGUCGGUCGGCCCCAAGGACGCCUUCGUCAUCAAGCGCUCGAUCUCGAGCCCCCAUCUACUCGCCAUCGCCCUCGUCUGCGCAGGC
env-88     CAUAGGCGAGGAAGCCUGGGAAAACCCUGAAACCGGGCGCAUUUCCAAUGUCUACUAUUGGGAAAGCAUGGAUGGCUUGCAGGCUUUGAUGAAGCACCCCAAGCAUCU
env-90     CUCGCCAGUGAUUACAUCCAACUCGUGUUUGGUGAGUACACCGCGAAUCUGUACAACGACCUUAGCUUCAAGGAUCGCGC............................
Rsp-1-1    ACUGCGAUUUGGUUUCCUUGCCAUGCAAUAGAUGUCUACAAAUGGGCUGACGCAAAAGGCCAAGUCCACUACGGCGACGCCGUCCCGGAGGGAAAGGCAAGCUCCGCC
env-91     GGCGUGUGGGAGCAGGCCUGGCGCUUCGAUGCACGCCAGCCACCCAGGAUCUCGGCCGAGCGGCGCCGCGCCCUGCGGGCCGGCAGCGCCGGCGCAGUGCCCGACGCA
env-93     ............................................................................................................
env-94     ............................................................................................................
env-95     UGUUGAUGUCCGGCCCGGAUACACAGGCGUACAUGUUCCGGGCCAGAUGGGGCAAAGGCGGCCAAGAAUGCGCUCAUUCGCAUCCGUCAAGGCCCCGCAAGGCACCAU
env-96     CUCCAGGGCCACCAGCGCAUCGCCGAUGUCUACCGGGCUGGAAGGCAUGGCGCGUGGGCUGCUGGCUCUGGCAUAGCCUUCCAUGGCGCGCACCACGGCCUCG.....
env-97     CUCCAGGGCCACCAGGGCGUCGCCGAUGUCCACCGAGGGGGUGGCGUGGGUGGUGCGCUCGCUGCUGGCCUUGGCGUAGCCUUCCAUGGCGCGCACCACGGCGUCGGG
env-99     CUCCAGGGCCACCAGGGCGUCGCCGAUGUCCACCGAGGGGGUGGCGUGGAUGGUGCGCUCGCUGCUGGCCUUGGCGUAGCCCUCCAUGGCGCGCACCACGGCGUCGGG
env-101    ............................................................................................................
Asp-9-1    CUCCAGGGCCACCAGGGCGUCGCCGAUGUCCACCGGGGGGGUGGCGUGGGUGGUGCGCUCGCUGCUGGCCUUGGCGUAGCCCUCCAUGGCGCGCACCACGGCGUCGGG
env-102    ............................................................................................................
env-103    UGGUGAACGCGCGCUUUUGCCGCUCGCGUUCGGCGCGGCUGCGGUCGCGCUCCAGGGCCACCAGGGCGUCGCCGAUGUCCACCGAGGGGGUGGCGUGGGUGGUGCGCU
env-104    CUCCAGGGCCACCAGGGCGUCGCCGAUGUCCACCGGGGGGGUAGCGUGGGUGGUGCGCUCGCUGCUGGCCUUGGCGUAGCCCUCCAUGGCGCGCACCACGGCGUCGGG
env-106    GGCGAGCCAGUGGGUGUCAUCAAAGUAGUGGGUCUGGUGUGCGUGGUGGGAGGCGUUGUCAUGCUCCACCUCGGAAGCCCUCAGUGAUGGCUUCUGGUGUUGAUCAAG
env-107    CUCCAGGGCCACCAGGGCGUCGCCGAUGUCCACCGAGGGGGUGGCGUGGGUGGUGCGCUCGGUGCUGGCCUUGGCGUAGCCCUCCAUGGCGCGCACCACGGCGUCGGG
env-110    ............................................................................................................
env-111    ............................................................................................................
env-112    CUCCAGGGCCACCAGGGCGUCGCCGAUGUCCACCGAGGGGGUGGCGUGGGUGGUGCGCUCGCUGCUGGCCUUGGCGUAGCCUUCCAUGGCGCGCACCACGGCGUCGGG
env-113    ............................................................................................................
Asp-5-1    CUCCAGGGCCACCAGCGCGUCGCCGAUAUCCACCGGGCUGGAGGGCAUGGCGCGUGGACUGCUGGCUUUGGCAUAGCCCUCCAUGGCGCGCACCACGGCCUCGGGGUC
Ara-1-1    CUCCAGGGCCACCAGCGCGUCGCCGAUGUCCACCGGGCUGGAGGGCAUGGCGCGCGGACUGCUGGCUUUGGCAUAGCCCUCCAUGGCGCGCACCACGGCCUCGGGGUC
Asp-6-1    CUCCAGGGCCACCAGCGCGUCGCCGAUGUCCACCGGGCUGGAGGGCAUGGCGCGCGGACUGCUGGCUUUGGCAUAGCCCUCCAUGGCGCGCACCACGGCCUCGGGGUC
env-114    ............................................................................................................
env-115    GUGCUUGGUGAUGACCCGUCCUGGCCGCGCCCUCUACCGGGUUCAGCCGAGCGGGUCGGCUGGGCCGACCGCCAUCGAUGCUCACAGCCCGGCUCGCGGCUCGCGGCU
env-117    ............................................................................................................
env-118    CGGGCGGCCGUCGGCCGGUGGGGCGAACGACGCCGAGCGGGUCUGGAAUGCCGCGGUGGCCAGGUUGAACAGCAGCUCGCGCAGGUGGGUGCAGCCCUGGAUGCCGCC
env-120    CCCCUCGACAGAUCACCCAUCGGAAGAACGCCGGCAGCGAAACAGGCCGGUCGCAUCCACUCGGGCCAUCGCCGGCAUGUGCCGUGGCGCAAGGCGCUGCCAGUCCAG
env-125    ............................................................................................................
Psp-1-1    CAAGCCAACCUCGCAAUGCACCCGUGGAAUCCAAGGCACUAAUGCGGUGCAGUGUGAGGUGGGGCUGACGGAGGCGCAGAUCGGUACGCUGAGGGAGCGCGGUGUUGU
env-126    ............................................................................................................
env-127    ............................................................................................................
env-128    ............................................................................................................
env-129    ............................................................................................................
Hfl-1-1    UGCGGCAGAAGGUGCGUCGCUCAUCCUGUUUCCCGAGCUGUCGCUCACCGGCUACGAGUUGCCGCUUCUGAACCGCCAUGUCCUGCCGGCGGACGCUCCGGUGCUAGA
Hsp-1-1    CAAGACGAUCAAGUGGGCGCAGGUUCUGGCGCGACUGGAGGGCCGUCCCGGGGCGCUGGAGGUGCUGCGUGAAAUGGAGGCGACCGGUGGCGAACCGGACGUGGUGGU
env-130    CAUCCGCAUCGGCGUGGUGAACGAGAUUUCCGGCGUGCAGGCCCAGGCCGGGGAGUACACGCUCUACGGCAUCCGGCUCGCUCUGGAGGAAAUCAACAAGGCCGGCGG
env-131    GCGCCCAUGACCACGGUGCCGACUCCGACUCGGCCACCGAGCAGCGCGCCGGUGCAGGCCAUGCCGAUCUCCAUCCCGACGCGAGUCCGGGCCGGGGCGACACCGAGU
env-132    GCGCCCAUGACCACGGUGCCGACUCCGACUCGGCCACCGAGCAGCGCGCCGGUGCAGGCCAUGCCGAUCUCCAUCCCGACGCGAGUCCGGGCCGGGGCGACACCGAGU
env-133    UGGAUACCCACUG...............................................................................................
env-134    ............................................................................................................
env-135    UGGAUACCCACUGAUAUCAAUAAUCGAUAAAAUGCUUACCUAAAAUCAGGAGUCUGUAUGUAUUCAGCCACUUUUAUCUUCGACAAAAAACAGUUCGAUGAGGCGUUU
env-136    CAUAGGCGAGGAAGCCUGGGAAAACCCUGAAACCGGGCGCAUUUCCAAUGUCUACUACUGGGAAAGCAUGGAUGGCUUGCAGGCUUUGAUGAAACACCCCAAACACCU
Bte-1-1    CGAUGCCGGCUUGACGCGCACAUAUCUCUACAAGCUCGCUUCAGGCGACACUCGAGAUCCCUCGAUCAGAACCCUGAUCCGGUUGGCCGUUGCGCUCAAGAUAUCGCC
Bce-5-1    CUUCAUCGAAGGCCUGCUGCUUGGCGCCGGCCUGUUCACGUCGGUCGGCCCCAAGGACGCCUUCGUCAUCAAGCGCUCGAUCUCGAGCCCCCAUCUCCUCUCCAUCGC
           ............................................................................................................
           ............................................................................................................
           ............................................................................................................
           ............................................................................................................
           oooooooooooooooooooooooooooooooooooooooooooooooooooooooooooooooooooooooooooooooooooooooooooooooooooooooooooo
```

4 GGAM-4

4.1 Taxa

The taxonomy of each organism containing a putative GGAM-4 RNA is listed, with abbreviations identifying each hit (e.g., "Eco-1-1" and "Eco-1-2" might hypothetically represent two distinct RNAs in *E. coli*). The abbreviations will be used to identify each individual GGAM-4 RNA in Sections 4.2 and 4.4:.

abbrev. of hits	taxonomy of species
Msu-1-1	Bacteria Proteobacteria Alphaproteobacteria Rhizobiales *Methyloceanibacter superfactus*
env-1 to env-7	environmental samples

4.2 Gene contexts

Each GGAM-4 RNA (indicated by "RNA→") is listed. For each hit, the downstream genes predicted to reside in a regulated operon are listed. If the nearest downstream gene is encoding in the opposite strand (and therefore presumed to not be a part of a regulated operon), then that gene is still depicted. Some environmental sequences and some RefSeq entries lack gene annotations, and so no genes are available for such sequences. The direction of each gene is indicated with an arrow (→), and each predicted conserved domain in the gene is named. Conserved domains associated with more than one GGAM-4 RNA are assigned a color; other domains are gray. Information about these conserved domains is given in Section 4.3. The accession of the sequence containing each GGAM-4 RNA is given in the column named "Seq. accession". Accessions beginning with "NC_", "NS_", "NW_" or "NZ_" are contained in RefSeq. Other accession refer to environmental samples. Nucleotide coordinates are given for the 5′ and 3′ boundaries of each GGAM-4 RNA. If the 5′ coordinate is greater than the 3′ coordinate, the RNA is present on the reverse-complement strand of the containing genomic DNA sequence. Each hit is denoted by an abbreviation (like "Eco-1-1") that refers to a taxonomy given in Section 4.1.

abbrev.	Seq. accession		5′ at	3′ at	genes
Msu-1-1	NZ_LPWF01000033.1	+	92831	93271	**RNA→** PRK11431 (PRK11431)EmrE (COG2076)→ PaaN-DH (TIGR02278)FkbR2 (cd03451)→
env-1	Ga0114923_10107181	-	484	40	**RNA→** PRK11431 (PRK11431)EmrE (COG2076)→
env-2	ASMM170b_GJFD58A01BWWI0	-	201	1	**RNA→**
env-3	Ga0114930_10262156	-	180	1	**RNA→**
env-4	Ga0114921_10231115	+	1	416	**RNA→** PRK11431 (PRK11431)EmrE (COG2076)→ PaaN-DH (TIGR02278)FkbR2 (cd03451)→
env-5	Ga0114922_10932069	+	49	494	**RNA→** PRK11431 (PRK11431)EmrE (COG2076)→ FkbR2 (cd03451)→
env-6	Ga0114921_10117330	+	1627	1791	**RNA→**
env-7	Ga0114930_10512263	-	340	1	**RNA→**

4.3 Conserved domains

Conserved domains found in protein-coding genes listed in Section 4.2 are shown below, with the first sentence in their description from the Conserved Domain Database (if any). Conserved domains associated with more than one GGAM-4 RNA are assigned a color, while others are shown in gray. The number in parentheses after the colored domain name is the number of occurrences in Section 4.2.

cd03451 (3) FkbR2 is a Streptomyces hygroscopicus protein with a hot dog fold that belongs to a conserved family of proteins found in prokaryotes and archaea but not in eukaryotes.
COG2076 (4) Multidrug transporter EmrE and related cation transporters [Defense mechanisms]
PRK11431 (4) multidrug efflux system protein; Provisional
TIGR02278 (2) phenylacetic acid degradation protein paaN.

4.4 Multiple-sequence alignment

Each GGAM-4 RNA is denoted by an abbreviation (like "Eco-1-1") that refers to a taxonomy given in Section 4.1. The alignment may include sequences containing the RNA motif, as well as flanking sequence. The GGAM-4 RNA itself is denoted by the line underneath marked 5′ and 3′ on either end. Nucleotides in flanking sequences (i.e., not part of the motif) are written in gray letters. Stems of predicted rho-independent transcription terminators, if any, are shaded yellow. (Note: terminator predictions have not been analyzed manually, and many are likely to be false positives. Terminator predictions are those of the RNIE soft-

ware.) Nucleotides predicted to function (as DNA) as transcription-factor binding sites, if any, are shaded in green. (But note: these predictions are manually annotated, so they might be under-predicted.) Annotated start codons, if any, are shaded green. (Note: start codons are frequently misannotated, especially in environmental samples.) Nucleotides proposed to basepair as part of the consensus structure are shaded in color when they comprise Watson-Crick or G-U pairs. Otherwise they are shaded gray. Conserved stems are also indicated at the bottom of the alignment by angle brackets, where matching < and > denote base-paired columns. Below these angle brackets, the symbol "2" denotes base pairs exhibiting covariation according to the statistically well-founded R-scape method. "1" denotes base pairs exhibiting covariation according to R2R's simplistic method. "0" denotes base pairs that are not observed to mutate and "?" denotes base pairs that have a significant frequency of non-canonical nucleotides for Watson-Crick or G-U pairs (> 5%). Below these base pair annotation is the consensus sequence: "R" = "A" or "G", "Y" = "C" or "U", red nucleotides: nucleotide identity conserved more than 97% of the time, black nucleotides: 90%, gray nucleotides: 75%, red circle (•): nucleotide is present 97% of the time, black circle (•): 90%, gray circle (•): 75%, white circle (◦): 50%. All percentages of sequences just described (e.g. 97% conserved) assume that sequences have been weighted by the GSC algorithm implemented by the Infernal software package.

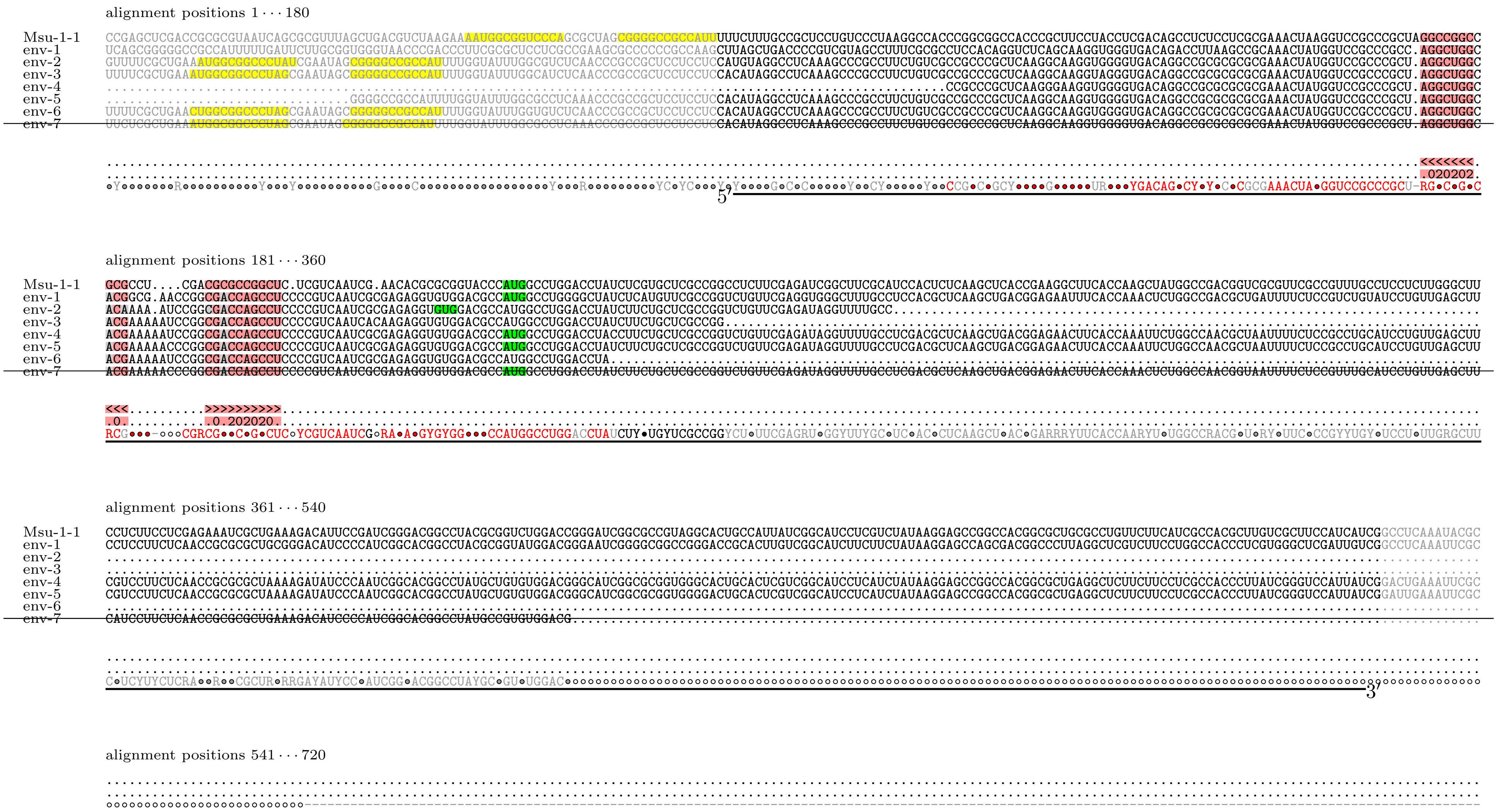

```
Msu-1-1  AACACCGACACAAUGACCAAUCCAGACAAGAUCGACCGCACCAUCACCGGCAACUAUUUCGAGGACUUCAAGGUCGGCCAGGAAAUCGUGCAUGCCACGCCGCGCACCGUCACCGUGGGCGAUACGAGCGUCUAUACCGCGCUCUAUGGCGGCCGCUUCGCCGUGCAGUCGUCGGACGCC
env-1    AAGCUCCCAAUGACCGAUACCAAAGC..........................................................................................................................................................
env-2    ....................................................................................................................................................................................
env-3    ....................................................................................................................................................................................
env-4    AACCACGAGCACAUGACCAACAUAAAAAGCGACAAGAUUGACCGGACUGUCACCGGCAAUUAUUUCGAGGACUUCAAACUCGGCCAGGAGAUCGUGCACGCAACGCCGCGCACCGUGACCGUGGGCGAUGUCAGCCUGUACACCGCGCUCUAUGGUGGGCGCUUCGCCGUGCAGUCCUCA
env-5    AACCACGAGCACAUGACCAACAUAAAAAGCGACAAGAUCGACCGGACUGUCACCGGCAAUUAUUUCGAGGACUUCAAACUCGGCCAGGAGAUUGUGCACGCAACGCCGCGCACCGUGACCGUGGGCGAUGUCAGCCUGUACACCGCGCUCUAUGGUGGGCGCUUCGCCGUGCAGUCCUCA
env-6    ....................................................................................................................................................................................
env-7    ....................................................................................................................................................................................

         ....................................................................................................................................................................................
         ....................................................................................................................................................................................
         oooooooooooooooooooooooooo----------------------------------------------------------------------------------------------------------------------------------------------------------
```

alignment positions 721 · · · 827

```
Msu-1-1  UUCGCGCAGUCCAUCGGCUAUCCGAAGAGCCCCAUCGACGACAUUCUCGUGUUCCACUUCGUCAUCGGCAAGACGGUGCCAGACAUCUCGCGCAACGCCAUCGCCAA
env-1    ...........................................................................................................
env-2    ...........................................................................................................
env-3    ...........................................................................................................
env-4    GAUGCCUUCGCCCAGAGCAUCGGCUACCCACGCUCGCCCAUUGACGACAUCCUGGUGUUCCACUUCGUGAUCGGCAAGACGGUGCCCGACAUCUCGCGCAACGCCAU
env-5    GAUGCCUUCGCCCAGAGCA........................................................................................
env-6    ...........................................................................................................
env-7    ...........................................................................................................

         ...........................................................................................................
         ...........................................................................................................
         -----------------------------------------------------------------------------------------------------------
```

5 GGAM-5

5.1 Taxa

The taxonomy of each organism containing a putative GGAM-5 RNA is listed, with abbreviations identifying each hit (e.g., "Eco-1-1" and "Eco-1-2" might hypothetically represent two distinct RNAs in *E. coli*). The abbreviations will be used to identify each individual GGAM-5 RNA in Sections 5.2 and 5.4:.

**abbrev. of hits**	**taxonomy of species**
Bsp-1-1	Bacteria Proteobacteria Alphaproteobacteria Caulobacterales Caulobacteraceae *Brevundimonas sp.* SH203
Kvu-1-1 to Kvu-1-3	Bacteria Proteobacteria Alphaproteobacteria Rhodobacterales Rhodobacteraceae *Ketogulonicigenium vulgare*
Kvu-2-1	Bacteria Proteobacteria Alphaproteobacteria Rhodobacterales Rhodobacteraceae *Ketogulonicigenium vulgare* WSH-001
Kvu-3-1	Bacteria Proteobacteria Alphaproteobacteria Rhodobacterales Rhodobacteraceae *Ketogulonicigenium vulgare* Y25
Osp-1-1	Bacteria Proteobacteria Alphaproteobacteria Rhodobacterales Rhodobacteraceae *Oceanicola sp.* S124
Psa-1-1	Bacteria Proteobacteria Alphaproteobacteria Rhodobacterales Rhodobacteraceae *Paracoccus sanguinis*
Psp-1-1	Bacteria Proteobacteria Alphaproteobacteria Rhodobacterales Rhodobacteraceae *Phaeobacter sp.* 11ANDIMAR09
Rma-1-1	Bacteria Proteobacteria Alphaproteobacteria Rhodobacterales Rhodobacteraceae *Rhodobacter maris*
Rsu-1-1	Bacteria Proteobacteria Alphaproteobacteria Rhodobacterales Rhodobacteraceae *Rhodovulum sulfidophilum*
Ral-1-1	Bacteria Proteobacteria Alphaproteobacteria Rhodobacterales Rhodobacteraceae *Roseovarius albus*
Ysp-1-1	Bacteria Proteobacteria Alphaproteobacteria Rhodobacterales Rhodobacteraceae *Yangia sp.* SAOS 153D
Bro-1-1 to Bro-1-2	Bacteria Proteobacteria Alphaproteobacteria Rhodospirillales Acetobacteraceae *Belnapia rosea*
Efl-1-1	Bacteria Proteobacteria Alphaproteobacteria Sphingomonadales Erythrobacteraceae *Erythrobacter flavus*
Esp-1-1	Bacteria Proteobacteria Alphaproteobacteria Sphingomonadales Erythrobacteraceae *Erythrobacter sp.* HI0020
Esp-2-1 to Esp-2-2	Bacteria Proteobacteria Alphaproteobacteria Sphingomonadales Erythrobacteraceae *Erythrobacter sp.* HI0038
Esp-3-1	Bacteria Proteobacteria Alphaproteobacteria Sphingomonadales Erythrobacteraceae *Erythrobacter sp.* SAORIC-644
Npa-1-1	Bacteria Proteobacteria Alphaproteobacteria Sphingomonadales Sphingomonadaceae *Novosphingobium panipatense*
Nsp-1-1	Bacteria Proteobacteria Alphaproteobacteria Sphingomonadales Sphingomonadaceae *Novosphingobium sp.* B-7
Nsp-2-1	Bacteria Proteobacteria Alphaproteobacteria Sphingomonadales Sphingomonadaceae *Novosphingobium sp.* HII-3
She-1-1	Bacteria Proteobacteria Alphaproteobacteria Sphingomonadales Sphingomonadaceae *Sphingobium herbicidovorans*
She-2-1 to She-2-2	Bacteria Proteobacteria Alphaproteobacteria Sphingomonadales Sphingomonadaceae *Sphingobium herbicidovorans* NBRC 16415
Sph-1-1	Bacteria Proteobacteria Alphaproteobacteria Sphingomonadales Sphingomonadaceae *Sphingobium phenoxybenzoativorans*
Squ-1-1	Bacteria Proteobacteria Alphaproteobacteria Sphingomonadales Sphingomonadaceae *Sphingobium quisquiliarum* P25
Ssp-1-1	Bacteria Proteobacteria Alphaproteobacteria Sphingomonadales Sphingomonadaceae *Sphingobium sp.* C100
Ssp-2-1	Bacteria Proteobacteria Alphaproteobacteria Sphingomonadales Sphingomonadaceae *Sphingobium sp.* DC-2

Ssp-3-1 to Ssp-3-2	Bacteria Proteobacteria Alphaproteobacteria Sphingomonadales Sphingomonadaceae *Sphingobium sp.* IP1
Ssp-4-1	Bacteria Proteobacteria Alphaproteobacteria Sphingomonadales Sphingomonadaceae *Sphingobium sp.* YR657
Sxe-1-1	Bacteria Proteobacteria Alphaproteobacteria Sphingomonadales Sphingomonadaceae *Sphingobium xenophagum* QYY
Sya-1-1	Bacteria Proteobacteria Alphaproteobacteria Sphingomonadales Sphingomonadaceae *Sphingobium yanoikuyae*
Sya-2-1	Bacteria Proteobacteria Alphaproteobacteria Sphingomonadales Sphingomonadaceae *Sphingobium yanoikuyae* ATCC 51230
Sya-3-1	Bacteria Proteobacteria Alphaproteobacteria Sphingomonadales Sphingomonadaceae *Sphingobium yanoikuyae* XLDN2-5
Sdo-1-1	Bacteria Proteobacteria Alphaproteobacteria Sphingomonadales Sphingomonadaceae *Sphingomonas dokdonensis*
Spa-1-1 to Spa-1-2	Bacteria Proteobacteria Alphaproteobacteria Sphingomonadales Sphingomonadaceae *Sphingomonas paucimobilis*
Ssp-5-1	Bacteria Proteobacteria Alphaproteobacteria Sphingomonadales Sphingomonadaceae *Sphingomonas sp.* Ag1
Ssp-6-1	Bacteria Proteobacteria Alphaproteobacteria Sphingomonadales Sphingomonadaceae *Sphingomonas sp.* CCH20-B6
Ssp-7-1	Bacteria Proteobacteria Alphaproteobacteria Sphingomonadales Sphingomonadaceae *Sphingomonas sp.* CCH5-D11
Ssp-8-1 to Ssp-8-2	Bacteria Proteobacteria Alphaproteobacteria Sphingomonadales Sphingomonadaceae *Sphingomonas sp.* Cra20
Ssp-9-1	Bacteria Proteobacteria Alphaproteobacteria Sphingomonadales Sphingomonadaceae *Sphingomonas sp.* IBVSS2
Ssp-10-1	Bacteria Proteobacteria Alphaproteobacteria Sphingomonadales Sphingomonadaceae *Sphingomonas sp.* Leaf20
Ssp-11-1	Bacteria Proteobacteria Alphaproteobacteria Sphingomonadales Sphingomonadaceae *Sphingomonas sp.* OV641
Ssp-12-1	Bacteria Proteobacteria Alphaproteobacteria Sphingomonadales Sphingomonadaceae *Sphingomonas sp.* Root710
Swi-1-1	Bacteria Proteobacteria Alphaproteobacteria Sphingomonadales Sphingomonadaceae *Sphingomonas wittichii*
Ssp-13-1	Bacteria Proteobacteria Alphaproteobacteria Sphingomonadales Sphingomonadaceae *Sphingopyxis sp.* C-1
Ssp-14-1	Bacteria Proteobacteria Alphaproteobacteria Sphingomonadales Sphingomonadaceae *Sphingopyxis sp.* H005
Ssp-15-1	Bacteria Proteobacteria Alphaproteobacteria Sphingomonadales Sphingomonadaceae *Sphingopyxis sp.* H012
Ssp-16-1	Bacteria Proteobacteria Alphaproteobacteria Sphingomonadales Sphingomonadaceae *Sphingopyxis sp.* H038
Ssp-17-1	Bacteria Proteobacteria Alphaproteobacteria Sphingomonadales Sphingomonadaceae *Sphingopyxis sp.* H053
Ssp-18-1	Bacteria Proteobacteria Alphaproteobacteria Sphingomonadales Sphingomonadaceae *Sphingopyxis sp.* H077
Ssp-19-1	Bacteria Proteobacteria Alphaproteobacteria Sphingomonadales Sphingomonadaceae *Sphingopyxis sp.* H080
Ssp-20-1	Bacteria Proteobacteria Alphaproteobacteria Sphingomonadales Sphingomonadaceae *Sphingopyxis sp.* H085
Ssp-21-1	Bacteria Proteobacteria Alphaproteobacteria Sphingomonadales Sphingomonadaceae *Sphingopyxis sp.* H093
Ssp-22-1	Bacteria Proteobacteria Alphaproteobacteria Sphingomonadales Sphingomonadaceae *Sphingopyxis sp.* H115
Ssp-23-1	Bacteria Proteobacteria Alphaproteobacteria Sphingomonadales Sphingomonadaceae *Sphingopyxis sp.* LC81
Bce-1-1	Bacteria Proteobacteria Betaproteobacteria Burkholderiales Burkholderiaceae Burkholderia Burkholderia cepacia complex *Burkholderia cepacia*
Bst-1-1	Bacteria Proteobacteria Betaproteobacteria Burkholderiales Burkholderiaceae Burkholderia Burkholderia cepacia complex *Burkholderia stabilis*
Bsp-2-1	Bacteria Proteobacteria Betaproteobacteria Burkholderiales Burkholderiaceae *Burkholderia sp.* H160
Cpa-1-1	Bacteria Proteobacteria Betaproteobacteria Burkholderiales Burkholderiaceae *Cupriavidus pauculus*
Pba-1-1	Bacteria Proteobacteria Betaproteobacteria Burkholderiales Burkholderiaceae *Paraburkholderia bannensis* NBRC 103871
Peb-1-1	Bacteria Proteobacteria Betaproteobacteria Burkholderiales Burkholderiaceae *Paraburkholderia eburnea*
Pno-1-1	Bacteria Proteobacteria Betaproteobacteria Burkholderiales Burkholderiaceae *Paraburkholderia nodosa* DSM 21604
Ptr-1-1 to Ptr-1-3	Bacteria Proteobacteria Betaproteobacteria Burkholderiales Burkholderiaceae *Paraburkholderia tropica*
env-1 to env-256	environmental samples

5.2 Gene contexts

Each GGAM-5 RNA (indicated by "RNA→") is listed. For each hit, the downstream genes predicted to reside in a regulated operon are listed. If the nearest downstream gene is encoding in the opposite strand (and therefore presumed to not be a part of a regulated operon), then that gene is still depicted. Some environmental sequences and some RefSeq entries lack gene annotations, and so no genes are available for such sequences. The direction of each gene is indicated with an arrow (→), and each predicted conserved domain in the gene is named. Conserved domains associated with more than one GGAM-5 RNA are assigned a color; other domains are gray. Information about these conserved domains is given in Section 5.3. The accession of the sequence containing each GGAM-5 RNA is given in the column named "Seq. accession". Accessions beginning with "NC_", "NS_", "NW_" or "NZ_" are contained in RefSeq. Other accession refer to environmental samples. Nucleotide coordinates are given for the 5′ and 3′ boundaries of each GGAM-5 RNA. If the 5′ coordinate is greater than the 3′ coordinate, the RNA is present on the reverse-complement strand of the containing genomic DNA sequence. Each hit is denoted by an abbreviation (like "Eco-1-1") that refers to a taxonomy given in Section 5.1.

abbrev.	Seq. accession		5′ at	3′ at	genes
env-1	2200709793	-	1077	645	**RNA**→ DoxX (pfam07681)→
env-2	Ga0041910_1005008	+	1178	1611	**RNA**→ AcrA (COG0845)HlyD_3 (pfam13437)→ PRK09579 (PRK09579)AcrB (COG0841)→
env-3	Ga0066256_1000093	+	298410	298843	**RNA**→ AcrA (COG0845)HlyD_3 (pfam13437)→ PRK09579 (PRK09579)AcrB (COG0841)→ TolC (COG1538)outer_NodT (TIGR01845)→

env-4	Ga0164287_1035651	-	438	27	**RNA**→ hypo→
Ral-1-1	NZ_FWFX01000002.1	-	56138	55705	**RNA**→ DoxX_2 (pfam13564)→
env-5	Ga0122799_111629	+	1	267	**RNA**→
env-6	Ga0121274_101118	+	654	1088	**RNA**→ DoxX_2 (pfam13564)→
Spa-1-1	NZ_JFYY01000042.1	+	5862	6295	**RNA**→ AcrA (COG0845)HlyD_3 (pfam13437)→ PRK09579 (PRK09579)AcrB (COG0841)→ TolC (COG1538)outer_NodT (TIGR01845)→
env-7	Ga0164243_10814917	+	1	383	**RNA**→ PRK11556 (PRK11556)AcrA (COG0845)→
env-8	Ga0164243_11646301	+	1	298	**RNA**→
env-9	Ga0164243_10389726	-	174	1	**RNA**→
env-10	Ga0164243_11601753	-	158	1	**RNA**→
env-11	Ga0164243_11663834	+	137	273	**RNA**→
env-12	Ga0164243_10432238	+	1	383	**RNA**→ AcrA (COG0845)HlyD_3 (pfam13437)→
env-13	Ga0164242_10552363	+	15	447	**RNA**→ AcrA (COG0845)RND_mfp (TIGR01730)→
env-14	Ga0164242_10056649	-	2121	1689	**RNA**→ AcrA (COG0845)HlyD_3 (pfam13437)→ PRK09579 (PRK09579)AcrB (COG0841)→
env-15	Ga0187893_10176203	-	1342	1132	**RNA**→ PBP2_CrgA_like (cd08422)HTH_1 (pfam00126)HTH_20 (pfam12840)→
env-16	Ga0187892_10242559	+	721	931	**RNA**→
env-17	Ga0160437_1004473	-	3365	2932	**RNA**→ AcrA (COG0845)HlyD_3 (pfam13437)→ PRK09579 (PRK09579)AcrB (COG0841)→
env-18	JGI24741J21665_1021010	+	275	708	**RNA**→ DoxX (COG2259)→
env-19	JGI24740J21852_10003992	-	6171	5738	**RNA**→ DoxX (COG2259)→
env-20	Ga0105240_10621691	-	817	384	**RNA**→ DoxX (COG2259)→
env-21	JGI24736J21556_1001379	+	3165	3598	**RNA**→ DoxX (COG2259)→
env-22	JGI24736J21556_1002379	-	3127	2694	**RNA**→ DoxX (COG2259)→
env-23	Ga0157370_10392951	-	1019	586	**RNA**→ DoxX (COG2259)→
env-24	Ga0105239_10680212	+	441	874	**RNA**→ DoxX (COG2259)→
env-25	Ga0157371_10479976	-	208	1	**RNA**→
env-26	JGI24739J22299_10066217	+	200	633	**RNA**→ DoxX (COG2259)→
env-27	Ga0105238_10512111	-	907	474	**RNA**→ DoxX (COG2259)→
env-28	Ga0157373_10314914	-	821	388	**RNA**→ DoxX (COG2259)→
env-29	Ga0157369_10533629	+	259	692	**RNA**→ DoxX (COG2259)→
env-30	Ga0157372_10511047	+	528	961	**RNA**→ DoxX (COG2259)→
Cpa-1-1	NZ_PJRP01000011.1	+	136700	137126	**RNA**→ YjbI (COG1357)Pentapeptide_4 (pfam13599)→
Ysp-1-1	NZ_NTHN01000173.1	-	62744	62311	**RNA**→ ←YkuD (pfam03734)
env-31	topACOD_F95O9CU02GCKK0	+	1	372	**RNA**→
env-32	Ga0164287_1071499	-	448	16	**RNA**→ hypo→
env-33	Ga0182024_15249143	-	305	1	**RNA**→
env-34	Ga0182024_10282561	-	305	1	**RNA**→
env-35	Pasolli2019-11528-383	-	3777	3346	**RNA**→ DoxX_2 (pfam13564)→
Bsp-1-1	NZ_BDMM01000010.1	+	10829	11257	**RNA**→ hypo→
env-36	SwRhRL2b_contig_3233759	-	1052	619	**RNA**→ AcrA (COG0845)HlyD_3 (pfam13437)→
Ssp-11-1	NZ_FNZB01000004.1	+	163344	163771	**RNA**→ emrE (PRK09541)EmrE (COG2076)→
Ssp-7-1	NZ_LSKH01000232.1	+	1408	1835	**RNA**→ emrE (PRK09541)EmrE (COG2076)→
Nsp-2-1	NZ_PPSM01000021.1	+	42884	43311	**RNA**→ emrE (PRK09541)EmrE (COG2076)→
Rsu-1-1	NZ_MSYQ01000001.1	-	3659520	3659087	**RNA**→ emrE (PRK09541)EmrE (COG2076)→
env-37	Ga0208939_1002464	+	361	789	**RNA**→ emrE (PRK09541)EmrE (COG2076)→
env-38	Ga0208822_1002099	-	12565	12137	**RNA**→ emrE (PRK09541)EmrE (COG2076)→
env-39	Ga0208460_10036363	+	357	785	**RNA**→ emrE (PRK09541)EmrE (COG2076)→
Sya-3-1	NZ_AFXE01000076.1	+	2240	2673	**RNA**→ AcrA (COG0845)HlyD_3 (pfam13437)→ AcrA (COG0845)HlyD_3 (pfam13437)→ AcrA (COG0845)HlyD_3 (pfam13437)→ PRK09579 (PRK09579)AcrB (COG0841)→ PRK09579 (PRK09579)AcrB (COG0841)→ TolC (COG1538)outer_NodT (TIGR01845)→ TolC (COG1538)outer_NodT (TIGR01845)→ hypo→ hypo→ TolC (COG1538)outer_NodT (TIGR01845)→ TolC (COG1538)outer_NodT (TIGR01845)→
env-40	QFQL01000020.1	+	248	681	**RNA**→ AcrA (COG0845)HlyD_3 (pfam13437)→ PRK09579 (PRK09579)AcrB (COG0841)→ TolC (COG1538)outer_NodT (TIGR01845)→ hypo→

Sya-1-1	NZ_CP023741.1	+	4204246	4204679	**RNA**→ AcrA (COG0845)HlyD_3 (pfam13437)→ PRK09579 (PRK09579)AcrB (COG0841)→ TolC (COG1538)outer_NodT (TIGR01845)→
Sya-2-1	NZ_JH992904.1	+	1954279	1954712	**RNA**→ AcrA (COG0845)HlyD_3 (pfam13437)→ PRK09579 (PRK09579)AcrB (COG0841)→ TolC (COG1538)outer_NodT (TIGR01845)→
Ssp-4-1	NZ_FRCG01000010.1	-	192274	191841	**RNA**→ AcrA (COG0845)HlyD_3 (pfam13437)→ PRK09579 (PRK09579)AcrB (COG0841)→ TolC (COG1538)outer_NodT (TIGR01845)→
env-41	Ga0121342_100008	+	8135	8568	**RNA**→ AcrA (COG0845)HlyD_3 (pfam13437)→ PRK09579 (PRK09579)AcrB (COG0841)→ TolC (COG1538)outer_NodT (TIGR01845)→
Ssp-3-1	NZ_NOIW01000005.1	-	99930	99497	**RNA**→ AcrA (COG0845)HlyD_3 (pfam13437)→ PRK09579 (PRK09579)AcrB (COG0841)→ TolC (COG1538)outer_NodT (TIGR01845)→
env-42	Ga0172375_10701175	+	248	638	**RNA**→
env-43	Ga0265750_1058614	-	599	218	**RNA**→ MFS (cd06174)efflux_EmrB (TIGR00711)→
env-44	Ga0182024_12872952	-	511	107	**RNA**→ ←hypo
env-45	JGI12635J15846_11006785	-	474	42	**RNA**→ MFS (cd06174)PRK14995 (PRK14995)→
env-46	Ga0182024_14185875	-	388	1	**RNA**→
env-47	JGI26338J50219_1144781	-	258	1	**RNA**→
env-48	Ga0209171_10504426	+	239	599	**RNA**→
env-49	Ga0182024_10371454	+	1588	1861	**RNA**→
env-50	Ga0182024_10039438	+	7624	7814	**RNA**→
env-51	Ga0182024_11529562	-	292	1	**RNA**→
env-52	Ga0265753_1261941	-	284	1	**RNA**→
env-53	Ga0209171_10008215	+	4254	4688	**RNA**→ hypo→
env-54	Ga0182024_12799710	-	519	138	**RNA**→ hypo→
env-55	Ga0182024_10211869	-	617	183	**RNA**→ hypo→
env-56	Ga0265754_1011246	+	286	717	**RNA**→
env-57	Ga0265750_1147222	+	106	437	**RNA**→
env-58	JGI12635J15846_12919899	-	280	1	**RNA**→
env-59	Ga0182024_10246818	-	2049	1615	**RNA**→ hypo→
env-60	Ga0182024_12309640	+	434	585	**RNA**→
env-61	Ga0182024_10026525	-	1145	711	**RNA**→ PRK14995 (PRK14995)MFS_1 (pfam07690)→
env-62	Ga0182024_13255656	-	166	1	**RNA**→
env-63	Ga0182024_11260806	+	670	860	**RNA**→
env-64	Ga0105238_11113836	-	599	170	**RNA**→ emrE (PRK09541)EmrE (COG2076)→
Ssp-12-1	NZ_LMIB01000015.1	+	377116	377545	**RNA**→ DUF1772 (pfam08592)→
env-65	Ga0209607_1502072	+	40	371	**RNA**→
env-66	Ga0157371_10187098	+	541	971	**RNA**→ ←hypo
Ssp-10-1	NZ_LMKN01000012.1	-	44119	43689	**RNA**→ hypo→
Bst-1-1	NZ_CP016444.1	-	192575	192145	**RNA**→ YjbI (COG1357)Pentapeptide_4 (pfam13599)→ hypo→
env-67	JGI25321J50212_10416229	+	73	245	**RNA**→
She-1-1	NZ_CP020541.1	+	8212	8641	**RNA**→ ←TolC (COG1538)outer_NodT (TIGR01845)
She-2-1	NZ_JFZA02000024.1	-	6173	5744	**RNA**→ emrE (PRK09541)EmrE (COG2076)→
She-2-2	NZ_BCZD01000033.1	+	3341	3770	**RNA**→ emrE (PRK09541)EmrE (COG2076)→
Ssp-13-1	NZ_BBRO01000001.1	+	720934	721364	**RNA**→ emrE (PRK09541)EmrE (COG2076)→
env-68	APTF_F3GY48O02IV6IJ	-	208	1	**RNA**→ hypo→
env-69	Ga0164242_10375249	+	674	1050	**RNA**→
Ptr-1-1	NZ_MSDZ01000069.1	+	360281	360711	**RNA**→ YjbI (COG1357)Pentapeptide_4 (pfam13599)→
Ptr-1-2	NZ_FNZM01000003.1	+	469877	470307	**RNA**→ YjbI (COG1357)Pentapeptide_4 (pfam13599)→
Ptr-1-3	NZ_LXGI01000075.1	-	24713	24283	**RNA**→ YjbI (COG1357)Pentapeptide_4 (pfam13599)→
Ssp-22-1	NZ_LNSA01000002.1	+	18637	19070	**RNA**→ emrE (PRK09541)EmrE (COG2076)→
Bsp-2-1	NZ_ABYL01000019.1	-	8125	7694	**RNA**→ YjbI (COG1357)Pentapeptide_4 (pfam13599)→ YjbI (COG1357)Pentapeptide_4 (pfam13599)→
env-70	Ga0172382_10004210	+	3766	4201	**RNA**→ DUF1772 (pfam08592)→
Kvu-2-1	NC_017384.1	-	818678	818249	**RNA**→ DoxX_2 (pfam13564)→
Kvu-3-1	NC_014625.1	-	1224074	1223645	**RNA**→ DoxX_2 (pfam13564)→ DoxX_2 (pfam13564)→

Kvu-1-1	NZ_CP012908.1	-	1209058	1208629	**RNA**→ DoxX_2 (pfam13564)→
Kvu-1-2	NZ_CP016592.1	-	1208880	1208451	**RNA**→ DoxX_2 (pfam13564)→
Kvu-1-3	NZ_CP017622.1	-	1363121	1362692	**RNA**→ DoxX_2 (pfam13564)→
env-71	Ga0160483_1014032	+	500	862	**RNA**→ emrE (PRK09541)EmrE (COG2076)→
env-72	Ga0160496_1031206	-	741	379	**RNA**→ emrE (PRK09541)EmrE (COG2076)→
env-73	Ga0160479_100005	+	169912	170341	**RNA**→ emrE (PRK09541)EmrE (COG2076)→
env-74	Ga0160487_100468	+	19862	20291	**RNA**→ emrE (PRK09541)EmrE (COG2076)→
env-75	Ga0160504_1077870	-	179	1	**RNA**→
Psp-1-1	NZ_LIKT01000001.1	-	114585	114152	**RNA**→ DoxX_2 (pfam13564)→
Psa-1-1	NZ_JRKQ01000003.1	-	64422	63989	**RNA**→ emrE (PRK09541)EmrE (COG2076)→
env-76	Ga0196970_1030827	-	824	397	**RNA**→ DUF1772 (pfam08592)→ hypo→
env-77	Ga0196960_10628459	-	193	1	**RNA**→ hypo→
env-78	Ga0196960_10130910	-	690	263	**RNA**→ DUF1772 (pfam08592)→
Ssp-8-1	NZ_CP024923.1	+	4321884	4322313	**RNA**→ emrE (PRK09541)EmrE (COG2076)→ emrE (PRK09541)EmrE (COG2076)→
Osp-1-1	NZ_AFPM01000170.1	+	33600	34033	**RNA**→ hypo→
env-79	Ga0117992_103150	-	496	66	**RNA**→ emrE (PRK09541)EmrE (COG2076)→
env-80	Ga0118081_104263	+	532	962	**RNA**→ emrE (PRK09541)EmrE (COG2076)→
Rma-1-1	NZ_OBMT01000041.1	-	2092	1660	**RNA**→ emrE (PRK09541)EmrE (COG2076)→
Spa-1-2	NZ_JFYY01000015.1	+	135986	136414	**RNA**→ DUF1772 (pfam08592)→
Squ-1-1	NZ_ATHO01000005.1	+	2820	3248	**RNA**→ DUF1772 (pfam08592)→
env-81	Ga0209066_11538086	+	1	295	**RNA**→
Sph-1-1	NZ_MINO01000126.1	-	5520	5090	**RNA**→ DUF1772 (pfam08592)→
Pno-1-1	NZ_JAFA01000010.1	+	272973	273404	**RNA**→ YjbI (COG1357)Pentapeptide_4 (pfam13599)→
Bce-1-1	NZ_LDWR01000042.1	-	160484	160054	**RNA**→ YjbI (COG1357)Pentapeptide_4 (pfam13599)→
env-82	Ga0160473_101437	+	4938	5367	**RNA**→ RpoE (COG1595)sigma70-ECF (TIGR02937)→<br>PRK09774 (PRK09774)FecR (pfam04773)DUF4880 (pfam16220)→
env-83	Ga0160461_1000679	-	8145	7716	**RNA**→ RpoE (COG1595)sigma70-ECF (TIGR02937)→<br>PRK09774 (PRK09774)FecR (pfam04773)DUF4880 (pfam16220)→
env-84	Ga0160449_1008947	-	1106	677	**RNA**→ RpoE (COG1595)sigma70-ECF (TIGR02937)→ FecR (COG3712)→
env-85	Ga0194138_10550947	+	228	423	**RNA**→
env-86	3300001592_10003349	+	11524	11957	**RNA**→ emrE (PRK09541)EmrE (COG2076)→
env-87	Ga0121620_10003	-	27382	26950	**RNA**→ emrE (PRK09541)EmrE (COG2076)→
env-88	Ga0121620_10010	+	9083	9515	**RNA**→ emrE (PRK09541)EmrE (COG2076)→
env-89	Ga0208135_135518	-	330	1	**RNA**→
env-90	DPOLF_F46JATG01ATV7N	+	17	377	**RNA**→
env-91	GRANPEH02GI0PN	-	197	1	**RNA**→
env-92	DNIF01000230.1	-	4908	4548	**RNA**→ emrE (PRK09541)EmrE (COG2076)→
env-93	Ga0122817_100324	-	1836	1476	**RNA**→ emrE (PRK09541)EmrE (COG2076)→
env-94	Ga0208412_1002716	+	477	837	**RNA**→ emrE (PRK09541)EmrE (COG2076)→
env-95	Ga0196958_10122145	-	233	1	**RNA**→
env-96	Ga0196960_10540688	-	144	1	**RNA**→
env-97	CEVJ01187520.1	-	1089	668	**RNA**→ emrE (PRK09541)EmrE (COG2076)→
env-98	CEVM01194738.1	+	1	393	**RNA**→ emrE (PRK09541)EmrE (COG2076)→
env-99	CETM01256083.1	-	279	1	**RNA**→
env-100	CEQH01146501.1	-	1250	820	**RNA**→ emrE (PRK09541)EmrE (COG2076)→
env-101	CEQM01235672.1	-	433	3	**RNA**→ emrE (PRK09541)EmrE (COG2076)→
env-102	CEVK01106931.1	+	481	911	**RNA**→ emrE (PRK09541)EmrE (COG2076)→
env-103	CETG01056918.1	+	598	889	**RNA**→
env-104	CEQO01025250.1	-	1306	876	**RNA**→ emrE (PRK09541)EmrE (COG2076)→
env-105	CESC01032633.1	+	594	1024	**RNA**→ emrE (PRK09541)EmrE (COG2076)→
env-106	CESG01137323.1	-	3249	2819	**RNA**→ emrE (PRK09541)EmrE (COG2076)→
env-107	CESL01240785.1	-	3251	2821	**RNA**→ emrE (PRK09541)EmrE (COG2076)→
env-108	CETA01149805.1	-	1250	820	**RNA**→ emrE (PRK09541)EmrE (COG2076)→

env-109	CETR01088801.1	-	1363	933	**RNA**→ emrE (PRK09541)EmrE (COG2076)→
env-110	CETS01152879.1	+	481	911	**RNA**→ emrE (PRK09541)EmrE (COG2076)→
env-111	CETT01151249.1	+	737	1167	**RNA**→ emrE (PRK09541)EmrE (COG2076)→
env-112	CEVI01225922.1	-	3251	2821	**RNA**→ emrE (PRK09541)EmrE (COG2076)→
env-113	DENQ01000161.1	-	2185	1755	**RNA**→ emrE (PRK09541)EmrE (COG2076)→
env-114	DFPC01000033.1	-	3227	2797	**RNA**→ emrE (PRK09541)EmrE (COG2076)→
env-115	DEYG01000029.1	-	3228	2798	**RNA**→ emrE (PRK09541)EmrE (COG2076)→
env-116	NYUG01000021.1	-	3303	2873	**RNA**→ emrE (PRK09541)EmrE (COG2076)→
env-117	PBAK01000050.1	-	3379	2949	**RNA**→ emrE (PRK09541)EmrE (COG2076)→
env-118	CETO01085124.1	+	598	1028	**RNA**→ emrE (PRK09541)EmrE (COG2076)→
env-119	CESY01269054.1	+	540	919	**RNA**→
env-120	CETU01098223.1	+	1	422	**RNA**→ emrE (PRK09541)EmrE (COG2076)→
env-121	CEVO01120844.1	+	1	369	**RNA**→ emrE (PRK09541)EmrE (COG2076)→
Ssp-9-1	NZ_NFUR01000004.1	+	102600	103029	**RNA**→ DUF1772 (pfam08592)→
Ssp-2-1	NZ_JNAC01000024.1	-	27373	26945	**RNA**→ DUF1772 (pfam08592)→
env-122	Ga0121624_102190	-	2336	1907	**RNA**→ emrE (PRK09541)EmrE (COG2076)→
Npa-1-1	NZ_MSQB01000031.1	-	49201	48772	**RNA**→ emrE (PRK09541)EmrE (COG2076)→
env-123	QFPX01000023.1	+	2723	3152	**RNA**→ emrE (PRK09541)EmrE (COG2076)→
Pba-1-1	NZ_BAYA01000010.1	+	43002	43433	**RNA**→ YjbI (COG1357)Pentapeptide_4 (pfam13599)→
env-124	Ga0208611_13739	-	490	100	**RNA**→ DoxX_2 (pfam13564)→
env-125	Ga0208734_101020	+	1638	2028	**RNA**→ DoxX_2 (pfam13564)→
env-126	Ga0122302_100145	-	16761	16329	**RNA**→ emrE (PRK09541)EmrE (COG2076)→
env-127	saf2_BPHP10386_g1	+	482	915	**RNA**→
env-128	Ga0209265_1082389	+	659	910	**RNA**→
Ssp-3-2	NZ_NOIW01000211.1	-	328	1	**RNA**→
env-129	Ga0209055_1492684	+	1	397	**RNA**→
Ssp-6-1	NZ_LSJC01000019.1	+	9345	9777	**RNA**→ COG4584 (COG4584)→ AAA (smart00382)IstB_IS21 (pfam01695)→
Peb-1-1	NZ_PQGA01000006.1	+	269522	269953	**RNA**→ YjbI (COG1357)Pentapeptide_4 (pfam13599)→
env-130	Ga0121496_104043	+	383	812	**RNA**→ emrE (PRK09541)EmrE (COG2076)→
env-131	Ga0121500_100115	-	19143	18714	**RNA**→ emrE (PRK09541)EmrE (COG2076)→
env-132	Ga0122302_100599	-	5605	5176	**RNA**→ emrE (PRK09541)EmrE (COG2076)→
Sxe-1-1	NZ_AKIB01000052.1	+	7257	7687	**RNA**→ DUF1772 (pfam08592)→ GstA (COG0625)GST_C_2 (pfam13410)GST_N_3 (pfam13417)→
Ssp-23-1	NZ_JNFD01000001.1	-	100147	99717	**RNA**→ emrE (PRK09541)EmrE (COG2076)→
Esp-2-1	NZ_LWEW01000536.1	-	492	61	**RNA**→ emrE (PRK09541)EmrE (COG2076)→
Esp-1-1	NZ_LWEO01000125.1	-	43661	43230	**RNA**→ emrE (PRK09541)EmrE (COG2076)→
Esp-2-2	NZ_LWEW01000003.1	+	22841	23272	**RNA**→ emrE (PRK09541)EmrE (COG2076)→
env-133	Ga0209717_1905796	+	1	282	**RNA**→
Efl-1-1	NZ_CP022528.1	+	1207408	1207839	**RNA**→ emrE (PRK09541)EmrE (COG2076)→
env-134	Ga0105664_1120711	+	1	382	**RNA**→ emrE (PRK09541)EmrE (COG2076)→
env-135	CEVA01354125.1	-	545	114	**RNA**→ emrE (PRK09541)EmrE (COG2076)→
env-136	CESR01307506.1	+	485	916	**RNA**→ emrE (PRK09541)EmrE (COG2076)→
env-137	CETT01270190.1	-	854	423	**RNA**→ emrE (PRK09541)EmrE (COG2076)→
env-138	CEVI01194233.1	+	176	607	**RNA**→ emrE (PRK09541)EmrE (COG2076)→
env-139	CEQH01089969.1	+	2810	3241	**RNA**→ emrE (PRK09541)EmrE (COG2076)→
env-140	CEQM01085564.1	-	896	465	**RNA**→ emrE (PRK09541)EmrE (COG2076)→
env-141	CESC01056768.1	+	2806	3237	**RNA**→ emrE (PRK09541)EmrE (COG2076)→
env-142	CESG01139675.1	-	12710	12279	**RNA**→ emrE (PRK09541)EmrE (COG2076)→
env-143	CESL01158797.1	+	2850	3281	**RNA**→ emrE (PRK09541)EmrE (COG2076)→
env-144	CETA01029321.1	+	2906	3337	**RNA**→ emrE (PRK09541)EmrE (COG2076)→
env-145	CETG01101007.1	+	370	801	**RNA**→ emrE (PRK09541)EmrE (COG2076)→
env-146	CETO01078326.1	-	7445	7014	**RNA**→ emrE (PRK09541)EmrE (COG2076)→
env-147	CETR01051202.1	-	1880	1449	**RNA**→ emrE (PRK09541)EmrE (COG2076)→

env-148	CETS01095889.1	+	2810	3241	**RNA**→ emrE (PRK09541) EmrE (COG2076)→
env-149	CETU01110819.1	+	122	553	**RNA**→ emrE (PRK09541) EmrE (COG2076)→
env-150	CEVM01098399.1	+	2868	3299	**RNA**→ emrE (PRK09541) EmrE (COG2076)→
env-151	DFPC01000003.1	+	81400	81831	**RNA**→ emrE (PRK09541) EmrE (COG2076)→
env-152	DEYG01000003.1	+	97360	97791	**RNA**→ emrE (PRK09541) EmrE (COG2076)→
env-153	NYUA01000180.1	-	12764	12333	**RNA**→ emrE (PRK09541) EmrE (COG2076)→
env-154	DLVK01000441.1	-	1177	746	**RNA**→ emrE (PRK09541) EmrE (COG2076)→
env-155	Ga0206126_10785699	+	1	389	**RNA**→
env-156	Ga0206123_10000002	-	198382	197994	**RNA**→ DUF1772 (pfam08592)→
env-157	CEQK01018135.1	-	321	1	**RNA**→
env-158	DDCE01000033.1	-	3501	3072	**RNA**→ DUF1772 (pfam08592)→
env-159	DLYG01000068.1	-	72692	72263	**RNA**→ DUF1772 (pfam08592)→
env-160	CEQK01004732.1	-	155	1	**RNA**→
env-161	CESK01033754.1	-	590	436	**RNA**→ DUF1772 (pfam08592)→
env-162	CETU01063367.1	-	2846	2464	**RNA**→ DUF1772 (pfam08592)→
env-163	CEVM01075768.1	+	3042	3424	**RNA**→ hypo→
env-164	CEVQ01010938.1	+	2411	2793	**RNA**→ hypo→
env-165	CEVJ01028928.1	-	895	466	**RNA**→ DUF1772 (pfam08592)→
env-166	CEVI01085365.1	-	231	1	**RNA**→
env-167	CEVO01079008.1	-	231	1	**RNA**→
env-168	PBAK01000036.1	-	13940	13511	**RNA**→ DUF1772 (pfam08592)→
env-169	CEOS01055084.1	+	888	1177	**RNA**→
env-170	NZUA01000001.1	-	89699	89270	**RNA**→ DUF1772 (pfam08592)→
env-171	DFLW01000042.1	-	30039	29610	**RNA**→ DUF1772 (pfam08592)→
env-172	CETR01200015.1	-	649	229	**RNA**→ DUF1772 (pfam08592)→
env-173	NZVT01000042.1	+	2940	3369	**RNA**→ DUF1772 (pfam08592)→
env-174	Ga0209603_1547893	-	341	1	**RNA**→
env-175	CESC01059182.1	-	659	230	**RNA**→ DUF1772 (pfam08592)→
Esp-3-1	NZ_POYF01000006.1	+	180820	181249	**RNA**→ DUF1772 (pfam08592)→
env-176	CEQF01045462.1	+	792	1221	**RNA**→ DUF1772 (pfam08592)→
env-177	DJKK01000219.1	-	938	509	**RNA**→ DUF1772 (pfam08592)→
env-178	DMDZ01000293.1	+	8852	9281	**RNA**→ DUF1772 (pfam08592)→
env-179	CEQG01239325.1	+	1	421	**RNA**→ DUF1772 (pfam08592)→
env-180	CEQX01139862.1	-	301	1	**RNA**→
env-181	CERQ01037211.1	+	150	450	**RNA**→ hypo→
env-182	CERC01118729.1	-	165	1	**RNA**→
env-183	CEVK01004900.1	+	2406	2579	**RNA**→ hypo→
env-184	CETG01036650.1	-	174	1	**RNA**→
env-185	CETZ01212572.1	+	409	582	**RNA**→ hypo→
env-186	CEQO01119467.1	+	407	717	**RNA**→
env-187	CESG01020729.1	+	3855	4165	**RNA**→ DUF1772 (pfam08592)→
env-188	CESL01018578.1	+	4776	5086	**RNA**→ DUF1772 (pfam08592)→
env-189	DFPC01000024.1	+	59745	60055	**RNA**→ DUF1772 (pfam08592)→
env-190	DEYG01000050.1	-	16129	15819	**RNA**→ DUF1772 (pfam08592)→
env-191	NYUG01000024.1	+	71507	71817	**RNA**→ DUF1772 (pfam08592)→
env-192	CETO01019877.1	-	174	1	**RNA**→
env-193	DMBR01000067.1	-	174	1	**RNA**→
env-194	CETA01029931.1	+	7330	7640	**RNA**→ DUF1772 (pfam08592)→
env-195	CETS01042976.1	+	2445	2755	**RNA**→ DUF1772 (pfam08592)→
env-196	CEVI01077033.1	-	231	58	**RNA**→
Ssp-1-1	NZ_AYOY01000124.1	+	39718	40148	**RNA**→ DUF1772 (pfam08592)→
Nsp-1-1	NZ_APCQ01000267.1	-	9495	9065	**RNA**→ emrE (PRK09541) EmrE (COG2076)→

env-197	DDTH01000007.1	-	149651	149221	**RNA**→ emrE (PRK09541)EmrE (COG2076)→
Ssp-15-1	NZ_LNRS01000003.1	+	320239	320669	**RNA**→ emrE (PRK09541)EmrE (COG2076)→
Ssp-18-1	NZ_LNRV01000010.1	-	155982	155552	**RNA**→ emrE (PRK09541)EmrE (COG2076)→
env-198	Ga0196958_10540293	+	132	456	**RNA**→
Ssp-16-1	NZ_LNRT01000002.1	+	233230	233660	**RNA**→ emrE (PRK09541)EmrE (COG2076)→
Ssp-19-1	NZ_LNRW01000010.1	-	70902	70472	**RNA**→ emrE (PRK09541)EmrE (COG2076)→
Ssp-20-1	NZ_LNRX01000016.1	+	22888	23318	**RNA**→ emrE (PRK09541)EmrE (COG2076)→
Ssp-14-1	NZ_LNRR01000004.1	-	155963	155533	**RNA**→ emrE (PRK09541)EmrE (COG2076)→
Ssp-21-1	NZ_LNRY01000017.1	-	79908	79478	**RNA**→ emrE (PRK09541)EmrE (COG2076)→
Ssp-17-1	NZ_LNRU01000005.1	+	320239	320669	**RNA**→ emrE (PRK09541)EmrE (COG2076)→
env-199	Ga0134125_11812937	-	311	1	**RNA**→
env-200	Ga0187845_1313006	+	175	493	**RNA**→
Sdo-1-1	NZ_NBBI01000004.1	+	240591	241021	**RNA**→ DUF1772 (pfam08592)→
env-201	Ga0187843_10209865	-	821	449	**RNA**→ DUF1772 (pfam08592)→
Bro-1-1	NZ_FMZX01000075.1	-	1779	1346	**RNA**→ DUF1772 (pfam08592)→
Bro-1-2	NZ_FMXZ01000037.1	+	556	989	**RNA**→ DUF1772 (pfam08592)→
env-202	Ga0190275_11237537	-	236	1	**RNA**→
env-203	Ga0209082_1006005	-	2110	1679	**RNA**→ DUF1772 (pfam08592)→
env-204	Ga0209097_10682820	-	356	1	**RNA**→
env-205	CETS01114686.1	+	336	506	**RNA**→
env-206	JGI25317J35169_1001159	-	8289	8119	**RNA**→ DUF1772 (pfam08592)→
env-207	PBXY01000025.1	+	15198	15368	**RNA**→ DUF1772 (pfam08592)→
env-208	JGI25319J35699_1104244	+	72	242	**RNA**→ ←hypo
env-209	JGI25322J35698_1137920	-	219	49	**RNA**→ hypo→
env-210	Ga0103682_10253602	-	417	1	**RNA**→
env-211	Ga0209082_1013676	+	1255	1685	**RNA**→ DUF1772 (pfam08592)→
env-212	Ga0160435_1081981	+	64	359	**RNA**→
env-213	Ga0160435_1025067	-	732	302	**RNA**→ DUF1772 (pfam08592)→
env-214	Ga0163151_10002143	+	1304	1734	**RNA**→ DUF1772 (pfam08592)→
env-215	Ga0163155_10067101	-	2087	1657	**RNA**→ DUF1772 (pfam08592)→
env-216	Ga0163153_10000089	+	117466	117896	**RNA**→ DUF1772 (pfam08592)→
env-217	Ga0163147_10002825	+	4974	5404	**RNA**→ DUF1772 (pfam08592)→
env-218	Ga0163150_10001261	+	13582	14012	**RNA**→ DUF1772 (pfam08592)→
env-219	Ga0163154_10000056	-	11287	10857	**RNA**→ DUF1772 (pfam08592)→
env-220	Ga0163152_10000176	+	79023	79453	**RNA**→ DUF1772 (pfam08592)→
env-221	Ga0163146_10035506	-	2645	2215	**RNA**→ DUF1772 (pfam08592)→
env-222	Ga0163145_1023008	+	512	942	**RNA**→ DUF1772 (pfam08592)→
env-223	Ga0163148_10689377	+	100	435	**RNA**→
env-224	Ga0163149_10292039	+	1	419	**RNA**→ DUF1772 (pfam08592)→
env-225	Ga0160433_100449	+	16518	16948	**RNA**→ DUF1772 (pfam08592)→
env-226	Ga0160483_1006491	-	2340	1910	**RNA**→ DUF1772 (pfam08592)→
Ssp-8-2	NZ_CP024923.1	-	4318198	4317768	**RNA**→ DUF1772 (pfam08592)→ DUF1772 (pfam08592)→
env-227	JGI24738J21930_10067538	+	1	420	**RNA**→ DUF1772 (pfam08592)→
env-228	Ga0157369_10320354	+	1043	1473	**RNA**→ DUF1772 (pfam08592)→
env-229	JGI24741J21665_1004404	+	180	610	**RNA**→ DUF1772 (pfam08592)→
env-230	JGI24740J21852_10030792	+	156	586	**RNA**→ DUF1772 (pfam08592)→
env-231	Ga0105238_10409569	-	1164	734	**RNA**→ DUF1772 (pfam08592)→
env-232	Ga0105239_10508938	+	206	636	**RNA**→ DUF1772 (pfam08592)→
env-233	Ga0157370_10206583	+	182	612	**RNA**→ DUF1772 (pfam08592)→
env-234	Ga0157372_10387809	-	1391	961	**RNA**→ DUF1772 (pfam08592)→
env-235	Ga0105240_10987719	-	715	285	**RNA**→ DUF1772 (pfam08592)→
env-236	Ga0105237_10519749	-	1009	579	**RNA**→ DUF1772 (pfam08592)→

env-237	Ga0157371_10000024	+	169516	169946	**RNA**→ DUF1772 (pfam08592)→
env-238	JGI24735J21928_10043982	+	398	828	**RNA**→ DUF1772 (pfam08592)→
env-239	JGI24737J22298_10081992	-	881	451	**RNA**→ DUF1772 (pfam08592)→
env-240	JGI24736J21556_1153182	-	313	1	**RNA**→
env-241	Ga0157373_10236538	-	1092	819	**RNA**→ DUF1772 (pfam08592)→
env-242	JGI24739J22299_10083349	+	499	929	**RNA**→ hypo→
env-243	JGI24735J21928_10498135	+	90	363	**RNA**→
env-244	QFQO01000651.1	+	1125	1555	**RNA**→ DUF1772 (pfam08592)→
Swi-1-1	NZ_JQMC01000041.1	-	3232	2802	**RNA**→ DUF1772 (pfam08592)→
Ssp-5-1	NZ_LAZX01000089.1	+	80498	80928	**RNA**→ DUF1772 (pfam08592)→
env-245	Ga0160428_1196590	-	359	1	**RNA**→
env-246	Ga0196960_10001097	-	7201	6769	**RNA**→ DUF1772 (pfam08592)→
env-247	Ga0196960_11297697	-	289	1	**RNA**→
env-248	ABLY01071752.1	+	1	112	**RNA**→
env-249	ABLY01334457.1	-	101	1	**RNA**→
env-250	ABLY01092354.1	-	111	1	**RNA**→
env-251	ABLY01071353.1	+	1	112	**RNA**→
env-252	ABLY01677343.1	-	60	1	**RNA**→ hypo→
env-253	ABLW01150054.1	+	1	101	**RNA**→
env-254	ABLY01333258.1	-	101	1	**RNA**→
env-255	Ga0209066_10205972	+	438	869	**RNA**→ emrE (PRK09541)EmrE (COG2076)→ smpB (PRK05422)SmpB (pfam01668)→
env-256	Ga0160429_1364045	+	34	464	**RNA**→

5.3 Conserved domains

Conserved domains found in protein-coding genes listed in Section 5.2 are shown below, with the first sentence in their description from the Conserved Domain Database (if any). Conserved domains associated with more than one GGAM-5 RNA are assigned a color, while others are shown in gray. The number in parentheses after the colored domain name is the number of occurrences in Section 5.2.

cd06174 (2) The Major Facilitator Superfamily (MFS) is a large and diverse group of secondary transporters that includes uniporters, symporters, and antiporters.
cd08422 (1) The C-terminal substrate binding domain of LysR-type transcriptional regulator CrgA and its related homologs, contains the type 2 periplasmic binding domain.
COG0625 (1) Glutathione S-transferase [Posttranslational modification, protein turnover, chaperones]
COG0841 (13) Multidrug efflux pump subunit AcrB [Defense mechanisms]
COG0845 (18) Multidrug efflux pump subunit AcrA (membrane-fusion protein) [Cell wall/membrane/envelope biogenesis, Defense mechanisms]
COG1357 (11) Uncharacterized protein YjbI, contains pentapeptide repeats [Function unknown]
COG1538 (13) Outer membrane protein TolC [Cell wall/membrane/envelope biogenesis]
COG1595 (3) DNA-directed RNA polymerase specialized sigma subunit, sigma24 family [Transcription]
COG2076 (94) Multidrug transporter EmrE and related cation transporters [Defense mechanisms]
COG2259 (12) Uncharacterized membrane protein YphA, DoxX/SURF4 family [Function unknown]
COG3712 (1) Periplasmic ferric-dicitrate binding protein FerR, regulates iron transport through sigma-19 [Inorganic ion transport and metabolism, Signal transduction mechanisms]
COG4584 (1) Transposase [Mobilome: prophages, transposons]
pfam00126 (1) Bacterial regulatory helix-turn-helix protein, lysR family.
pfam01668 (1) SmpB protein.
pfam01695 (1) IstB-like ATP binding protein.
pfam03734 (1) L,D-transpeptidase catalytic domain.
pfam04773 (2) FecR protein.
pfam07681 (1) DoxX.
pfam07690 (1) Major Facilitator Superfamily.
pfam08592 (76) Domain of unknown function (DUF1772).
pfam12840 (1) Helix-turn-helix domain.
pfam13410 (1) Glutathione S-transferase, C-terminal domain.
pfam13417 (1) Glutathione S-transferase, N-terminal domain.
pfam13437 (16) HlyD family secretion protein.
pfam13564 (12) DoxX-like family.
pfam13599 (11) Pentapeptide repeats (9 copies).
pfam16220 (2) Domain of unknown function (DUF4880).
PRK05422 (1) SsrA-binding protein; Validated
PRK09541 (94) multidrug efflux protein; Reviewed
PRK09579 (13) multidrug efflux protein; Reviewed
PRK09774 (2) fec operon regulator FecR; Reviewed

PRK11556 (1) multidrug efflux system subunit MdtA; Provisional
PRK14995 (2) methyl viologen resistance protein SmvA; Provisional
smart00382 (1) ATPases associated with a variety of cellular activities.
TIGR00711 (1) drug resistance transporter, EmrB/QacA subfamily. [Cellular processes, Toxin production and resistance, Transport and binding proteins, Other]
TIGR01730 (1) RND family efflux transporter, MFP subunit. [Transport and binding proteins, Unknown substrate]
TIGR01845 (13) efflux transporter, outer membrane factor (OMF) lipoprotein, NodT family. [Cellular processes, Detoxification, Transport and binding proteins, Porins]
TIGR02937 (3) RNA polymerase sigma factor, sigma-70 family.

5.4 Multiple-sequence alignment

Each GGAM-5 RNA is denoted by an abbreviation (like "Eco-1-1") that refers to a taxonomy given in Section 5.1. The alignment may include sequences containing the RNA motif, as well as flanking sequence. The GGAM-5 RNA itself is denoted by the line underneath marked $5'$ and $3'$ on either end. Nucleotides in flanking sequences (i.e., not part of the motif) are written in gray letters. Stems of predicted rho-independent transcription terminators, if any, are shaded yellow. (Note: terminator predictions have not been analyzed manually, and many are likely to be false positives. Terminator predictions are those of the RNIE software.) Nucleotides predicted to function (as DNA) as transcription-factor binding sites, if any, are shaded in green. (But note: these predictions are manually annotated, so they might be under-predicted.) Annotated start codons, if any, are shaded green. (Note: start codons are frequently misannotated, especially in environmental samples.) Nucleotides proposed to basepair as part of the consensus structure are shaded in color when they comprise Watson-Crick or G-U pairs. Otherwise they are shaded gray. Conserved stems are also indicated at the bottom of the alignment by angle brackets, where matching $<$ and $>$ denote base-paired columns. Below these angle brackets, the symbol "2" denotes base pairs exhibiting covariation according to the statistically well-founded R-scape method. "1" denotes base pairs exhibiting covariation according to R2R's simplistic method. "0" denotes base pairs that are not observed to mutate and "?" denotes base pairs that have a significant frequency of non-canonical nucleotides for Watson-Crick or G-U pairs ($> 5\%$). Below these base pair annotation is the consensus sequence: "R" = "A" or "G", "Y" = "C" or "U", red nucleotides: nucleotide identity conserved more than 97% of the time, black nucleotides: 90%, gray nucleotides: 75%, red circle (•): nucleotide is present 97% of the time, black circle (•): 90%, gray circle (•): 75%, white circle (◦): 50%. All percentages of sequences just described (e.g. 97% conserved) assume that sequences have been weighted by the GSC algorithm implemented by the Infernal software package.

Duplicate sequences: the following putative homologs are not shown in the alignment because their sequences are identical to a homolog already shown: Esp-2-2, Kvu-1-1, Kvu-1-2, Kvu-1-3, Kvu-3-1, Ptr-1-2, She-2-1, She-2-2, Ssp-14-1, Ssp-17-1, Ssp-19-1, Ssp-20-1, Ssp-21-1, Ssp-4-1, env-105, env-106, env-107, env-108, env-109, env-110, env-111, env-112, env-113, env-114, env-115, env-116, env-117, env-123, env-140, env-141, env-142, env-143, env-144, env-145, env-146, env-147, env-148, env-149, env-150, env-151, env-152, env-153, env-154, env-159, env-164, env-167, env-177, env-178, env-188, env-189, env-19, env-190, env-191, env-193, env-207, env-215, env-216, env-217, env-218, env-219, env-220, env-221, env-222, env-230, env-231, env-232, env-233, env-234, env-27, env-28, env-29, env-3, env-30, env-38, env-39, env-41, env-83, env-84, env-88, env-93, env-94

alignment positions 1 · · · 180

```
env-1    GGCCAAGGCGCCGGUUCGAGCGCGAGCGCUCCUGGCGGCGAUGGCGGGCUUCAAUCGGUUGCGGACGUGGCGAGCAGGACCGCGUCCGCCGCCGCCGUAGGACAUCCUGUCCAAUCCACCUGGAUCCCUGAGCCGAGCUAAGGACGCGUCGCGCGAAAAAUGAGUAGCAGCGUGGUGUCU
env-2    GAAAAAGCGCCGUUCGCGGCGUCUUUCGCGCCAUGGUUAGAGUCCGGGGCGCACAGGGGUCAAGGGCGUUCGUCAUGAAUGACGCGGGUCCCGUGCCGGCGGGCCGUUCGGCCGAUCUUGCGAAUCGCUCGCGGGGCGCGUAGCAGGGGGCGCCGAAAACGGAGUGGCAGGGUGGUGUCU
env-4    ......................................................................................................GUCACAUGGCCAUCUUGCGAAUCGCUCGCAGGGGGCGUAGGGGGUGGGCGCCGAAAACAGAGUAGCAGGGUGGUGUCU
Ral-1-1  CAUGGCGGACUCCCGGAAAUCUAGUCAGCUAGGGGCAUAGACGCGCCGCGCGCGCGAUACAAGUGUGAUACAACCGAACCACACCCUUCAGAACGUGAAUUUGUGACCAAACGAACUUGACGGAAAUCUAAAUCAACGAUACCCAAACAAUGCCGAAAAUAGAGUAGCAGGGUGGUGUCU
env-5    ............................................................................................................UGCCUAAUCCAUCCGGCCCCCGAGCCGAGCUAAGGACGCGUCGCGCGAAAAGUGAGUAGCAGGGUGGUGUCU
env-6    UCAAACUUAAAGGAAUCAGAUAACCAUUGAAAUGGUAAUGUUAUUUUUUGGGGUGACAAUCUUAUCAAACACGUAUCCGUAUUUUAUUAAAAUCUUUUAAUACUGUUAAAUUUAACCUUGCCAGUUUUCAACUAUUCCUUAUAAUCCAGCUGUCGAAAAAAGAGUUGCGGGGUGGUGUCU
Spa-1-1  UUGGUUACAUCGAGAUCUUUUCCAGAGCCGGGGGUGCUGACUGGCCUGCGAUCCUCGACCUCCCCACAUAAGGCGGCGCGCUGUCCCAAAAAAAAUGGGAACAAUCCCAAAAUCGGUUGCAAAUCACUUUCAACAAUCCUAUAAGGUUAUCGCCGAAAACAGAGUAGCAGGGUGGUGAUU
env-7    ...................................................................................................................................AACUACGCUAGGCGGCGCGGGCCGAAAACAGAGUGGCAGGGUGGUGUCU
env-8    ..............................................................................................CAGCGAUUCGGGCGCGAAUUGCUUGAUGGCCGCUUGCAAAUACGCUAGGCGGCGCGGGUCGAAAACAGAGUGGCAGGGUGGUGUCU
env-9    GCGCCGCGCGCUGCGGACUCUGCGAAGGAGCCGGCAUGACCUUGUUUUUCCACGCGGAAAUACCGGAUCGGGAAAGGUCCGCCGGUGGCCGGAACGGCGAUUCGGGCGCGAAUUGCUUGAUAGCCGUUCGCAACUACGCUAGGCGGCGCGGGCCGAAAACAGAGUGGCAGGGUGGUGUCU
env-10   GCGCCGCGCGCUGCGGACUCUGCGAAGGAGCCGGCAUGACUUUGUUUCUCCACGCGAAAAUACCGGAUCGGGAAAGGUCCACCGGCGGCCGGAACGGUGAUUCGGGCGCGAAUUGCUUGAUGGCCGUUCGCAACUACGCUAGGCGGCGCGGGCCGAAAACAGAGUGGCAGGGUGGUGUCU
env-11   GCGCCUUGCGUUGCAGAACCUGCGAAGGAGCCGGCAUGACUUUGUUUUUCCAUGUUAAAAUUCAUAACCGGGAUGGGGGUGCCGGCGUCCGGAGCAGCGAUUCGGGCGCGAAUUGCUUGAUGGCCGCUUGCAAAUACGCUAGGCGGCGCGGGUCGAAAACAGAGUGGCAGGGUGGUGUCU
env-12   ...................................................................................................................................AACUACGCUAGGCGGCGCGGGCCGAAAACAGAGUGGCAGGGUGGUGUCU
env-13   ..................................................................CCGACAUGCCUUCCGCUUAUUCGUUCUCUCCCCGGCGCGAGAAUCGCGCCUUGAAAACGGUUCGCAACAAGGUUAGGAAACCGGGGCCGAAAACAGAGUGGCAGGGUGGUGUCU
env-14   CCGUCACGGGGCACGGGCCCGCGCAUUCAGGAGCCGACAUGCCUUCUUCGCCUUGGUUUUGCCUGCCUUGCCGCCGGCGUUCCGUGCCAGUCGUUCCCCGGUAUCGGAAUCGCCACUUGAAAGCGGUUCGCAACAAGGCUAGGGAAUCGGAGCCGAAAACAGAGUGGCAGGGUGGUGUCU
env-15   GUGACCACAUCGUGCGGGAGCGCGUCCCAAGCGACCACUUCGGCCCGCUUCAGCUCCUCUGACAGUCCUGGCAUGUCGGGCAGCCUGUUCAACCAGAUCAGGUCCUGCUCGGUGAUGCGGAUAGGUUUCUCUCGGUUCAUCGCGUCGAUUCCUCCUCGAUAGAGUUGCAGGUUGGUGU..
env-16   GUGACCACAUCGUGCGGGAGCGCGUCCCAAGCGACCACUUCGGCCCGCUUCAGCUCCUCUGACAGUCCUGGCAUGUCGGGCAGCCUGUUCAACCAGAUCAGGUCCUGCUCGGUGAUGCGGAUAGGUUUCUCUCGGUUCAUCGCGUCGAUUCCUCCUCGAUAGAGUUGCAGGUUGGUGU..
env-17   UUUACCUCAACUAAUGUUGAGGUUUUAUAGAGGCACUCCCUGAUUCGCAAGGGAGGCGAAAUUUGCAUGAUCCUGUCACCGACCAUUUCCGGCCCCGCGCUGCGCGCCGGAAUUUGUUGCAAGCCGCUCGCAGCAACCCUAUUCCAAUUGCGCCAGAAAUAGAGUGGCAGGGUCGUGUCU
env-18   AAUCCGAACAAUGAUGCCCGUCAUUCACUUCGAUGGCGGGUCGAAUAGUGAAUGACGAAAUCCAGUGGCGAGCCGACAUGGAACUUAUGCCCGAGUGGUAGGACAAUUUGUCCCUUUUCCCAAUUAAGCUCUCGCAGCUAUGUCGCCGACGCGCGAAAAAUGAGUUGCAGGGUGGUGUCU

         ......................................................................................................................................................................<<<<<<.......>
         ......................................................................................................................................................................02..........
         ooooooooooooooooooooooooooooooooooooooooooooooooooooooooooooooooooooooooooooooooooooooooooooooooooooooooooooooo•••••••••••R•••••••••••••••••••••A•••••••••GCCGAAAUAGAGU•GCAGG•UGGUR•CU
                                                                                 5'
```

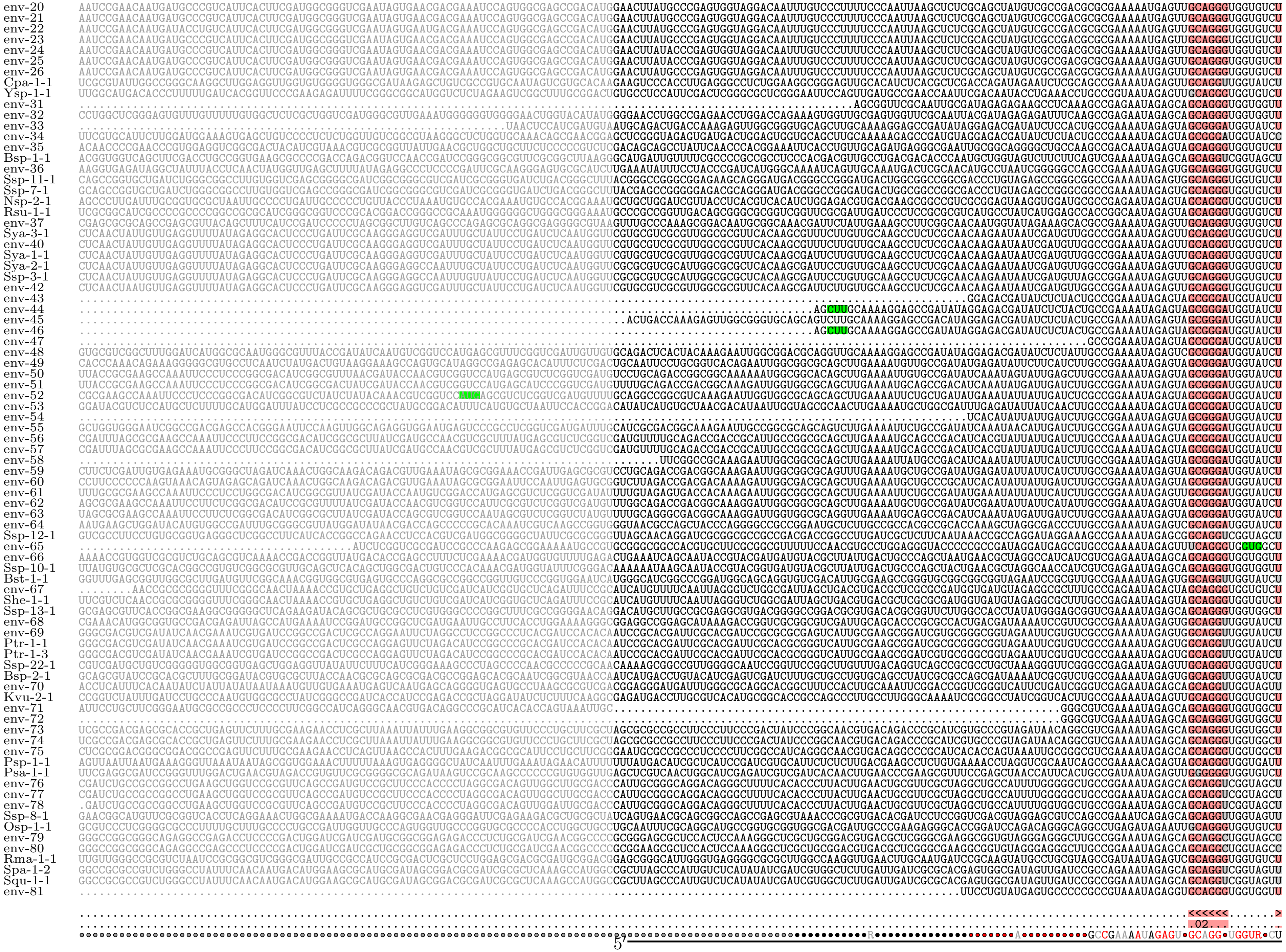

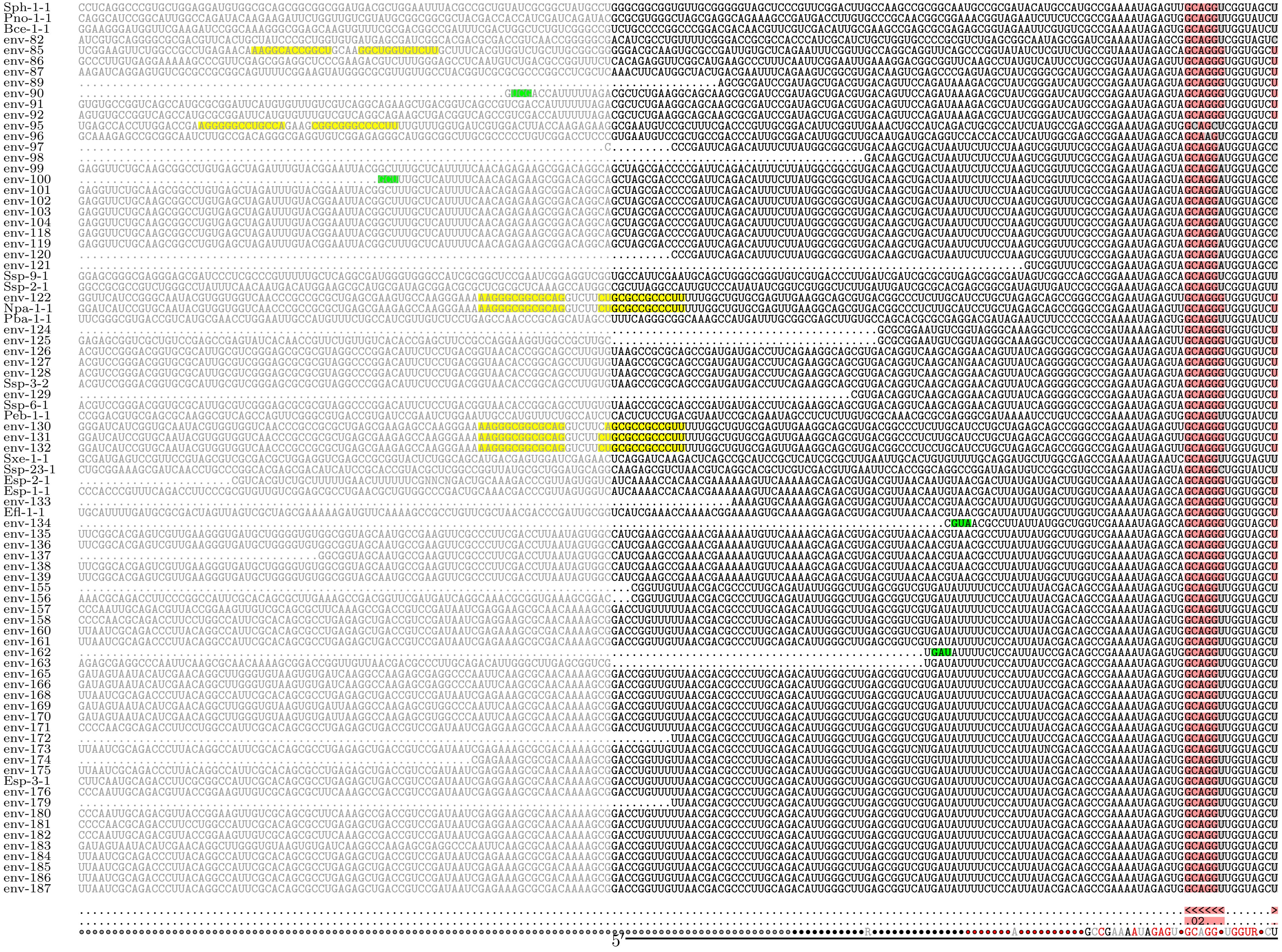
Sph-1-1
Pno-1-1
Bce-1-1
env-82
env-85
env-86
env-87
env-89
env-90
env-91
env-92
env-95
env-96
env-97
env-98
env-99
env-100
env-101
env-102
env-103
env-104
env-118
env-119
env-120
env-121
Ssp-9-1
Ssp-2-1
env-122
Npa-1-1
Pba-1-1
env-124
env-125
env-126
env-127
env-128
Ssp-3-2
env-129
Ssp-6-1
Peb-1-1
env-130
env-131
env-132
Sxe-1-1
Ssp-23-1
Esp-2-1
Esp-1-1
env-133
Efl-1-1
env-134
env-135
env-136
env-137
env-138
env-139
env-155
env-156
env-157
env-158
env-160
env-161
env-162
env-163
env-165
env-166
env-168
env-169
env-170
env-171
env-172
env-173
env-174
env-175
Esp-3-1
env-176
env-179
env-180
env-181
env-182
env-183
env-184
env-185
env-186
env-187
5′

```
env-192   UUAAUCGCAGACCCUUACAGGCCAUUCGCACAGCGCCUGAGAGCUGACCGUCCGAUAAUCGAGAAAGCGCGACAAAAGCGGACCGGUUGUUAACGACGCCCUUGCAGACAUUGGGCUUGAGCGGUCAUGAUAUUUUCUCCAUUAUACGACAGCCGAAAAUAGAGUGGCAGGUUGGUAGCU
env-194   UUAAUCGCAGACCCUUACAGGCCAUUCGCACAGCGCCUGAGAGCUGACCGUCCGAUAAUCGAGAAAGCGCGACAAAAGCGGACCGGUUGUUAACGACGCCCUUGCAGACAUUGGGCUUGAGCGGUCAUGAUAUUUUCUCCAUUAUACGACAGCCGAAAAUAGAGUGGCAGGUUGGUAGCU
env-195   UUAAUCGCAGACCCUUACAGGCCAUUCGCACAGCGCCUGAGAGCUGACCGUCCGAUAAUCGAGAAAGCGCGACAAAAGCGGACCGGUUGUUAACGACGCCCUUGCAGACAUUGGGCUUGAGCGGUCAUGAUAUUUUCUCCAUUAUACGACAGCCGAAAAUAGAGUGGCAGGUUGGUAGCU
env-196   UUAAUCGCAGACCCUUACAGGCCAUUCGCACAGCGCCUGAGAGCUGACCGUCCGAUAAUCGAGAAAGCGCGACAAAAGCGGACCGGUUGUUAACGACGCCCUUGCAGACAUUGGGCUUGAGCGGUCAUGAUAUUUUCUCCAUUAUACGACAGCCGAAAAUAGAGUGGCAGGUUGGUAGCU
Ssp-1-1   CCGAGAUCGCCGCCAUUGACGCGCAGUUAGCCAGCCGCGUCACGGAUUCGGAGCCACAAGCGCAUUGAGGCGCGUUUGUUGUAACGGGCUGCGACGCGGCGCAAGCGCUUAAGCUCUUGAUUCAUCACGCGUGAGCGGCGAUAGUUGGCCAGCCGAAAAUAGAGCAGCAGGUCGGUAGUC
Nsp-1-1   CCGGUGACCAGGACGUCGACUGAGGGCCAUUGCGCCAAUGGCAGCUAUCUGCGAUUUUCUGCCAGAAGCUGCCUUUCAGGAAGCGACCCCAUUUCGGACAUUUUCCUGAUGGUCGCGUGACAAGCUGAUUGAGCUUUCCUAAGUUGGUUUCGCCGAGCAUAGAGUAGCAGGAUGGUAGCC
env-197   AUCUGGCAGGGUGAAACAUAAGAGAGGCGAGUAGGAAAGUUUUUUGUGAGUAGGUUCUUGGGUGUGUCUGGAACAUCUGCUUUCGAUCCGUCUCAGGCAUUUUUUCUGAUGGUCGCGUGACAACCCGAUUGGGUUUUCCUAAGUCGGUCCGGCCGAGAAUAGAGUAGCAGGAUGGUAGCC
Ssp-15-1  GUUUGCGCAAGGCGAUCAACCUGCCCGGCACGAGCGACAUCAUCCGCACCGUGCGCUCGGCGGGUUAUGCCCUCGAUUCGGGCAAGAGCGUCUGACGUCGGACGGCGCCGGCCCGGUUGAUUCUCACCGUCAGGCCGGAUAGGUCCGGCGUGCCGAGAAUAGAGUAGCAGGCUGGUAUCU
Ssp-18-1  GUUUGCGCAAGGCGAUCAACCUGCCCGGCACGAGCGACAUCAUCCGCACCGUGCGCUCGGCGGGUUAUGCCCUCGAUUCGGGCAAGAGCGUCUGACGUCGGACGGCGCCGGCCCGGUUGAUUCUCACCGUCAGGCCGGAUAGGUCCGGCGUGCCGAGAAUAGAGUAGCAGGCUGGUAUCU
env-198   CACGUAACUGAGCUUCCAUCGCCAGAGUGAGCGUUUCGCGUCUAGGCUGGUGUCCGCUUUCCACCCAUUGCGGACAUUAGCCGAGUGUCCGCUUUCGACCCCUUGCGGACAUUCGGUUGAAAGCUGCAGCAUAUCACUCCAUCUAGAGUGGGCCGGAAAUAGAGUAGCAGGUCGGUAGCU
Ssp-16-1  GUUUGCGCAAGGCGAUCAACCUGCCCGGCACGAGCGACAUCAUCCGCACCGUGCGCUCGGCGGGUUAUGCCCUCGAUUCGGGCAAGAGCGUCUGACGUCGGACGGCGCCGGCCCGGUUGAUUCUCACCGUCAGGCCGGAUAGGUCCGGCGUGCCGAGAAUAGAGUAGCAGGCUGGUAUCU
env-199   UGCCGCGCAAGUGACAUGCGGGGAUGGCUACCGUGCGAAGCAGGUUCUCGAGUGCUUCCAACGGCAGCUUUCCACCCGUUCCGGAUGUUCCGUAAGUCAUGAUCUUUACGUCAUCGUUGCAGGUAUGAGGGGAAGGGGUUAUGAUCAGCUUGCCGGAAAUAGAGUUGCAGGUUGGUAGCU
env-200   CAGCUGGAGGAGAUCGCAAAGGACAUGGCGGGACGUUGAGUAAGCCAGAUGGACGGUCGUUAUGGUCAACCGUCUCUCGAUCCCGGCGUCUGCGGCGCGCCGAUGUCCGCUUACGCUUGCAGUUCUGCCGACUUCGCGUCAUGCUGAGGGUGCCGAAAAUAGAGUAGCGGGUCGGUAGCU
Sdo-1-1   GAGCAGUCGAGAACAGAUUUUGGGAAAUGCGAUACUCAGGACGCUCCUAGGUGUAAUCGGCGAGGUCACCUUCCCGUUUUGAGUUCGUUUCGGGAAAGACGAGAUGCGACUUACGCUUGCAGUUUCGCGGACGCCACGUCAUGCUGAGGGUGCCGAAAAUAGAGUAGCAGGUCGGUAGCU
env-201   ..........................................................................................................................................UCAUGCUGAGGGUGCCGAAAAUAGAGUAGCGGGUCGGUAGCU
Bro-1-1   GUUAGGCUUGAGUGGGGAGCAAUCCGGGGCGGACCUGCACCUUUCACUCCGCCCCGCGCCAAGCCGAACGUUUAAGGCCUAUUGCUGCACUCGAUCCGAAGCAGCCCUGCGAGAUGGUGCUCUGGUGGUCGGCAUCCGGCAUCAUGGCCCAGCCGGAAAUAGAGUUGCAGGUUGGUAGCU
Bro-1-2   GUUAGGCUUGAGUGGGGAGCAAUCCGGGGCGGACCUGCACCUUUCACUCCGCUCCGCGCCAAGCCGAACGUUUAAGGCCUAUUGCUGCACUCGAUCCGAAGCAGCCCUGCGAGAUGGUGCUCUGGUGGUCGGCAUCCGGCAUCAUGGCUCAGCCGGAAAUAGAGUUGCAGGUUGGUAGCU
env-202   ACGUACGGAACUGGGUUCGUCUGGACGAGCAGCUGAGGACUGGAGCGGCAAGUAUCCUGGGCCUGCGUACGGAUAGCCGCCGGCCAACUUCCGACCCAAGGCGGCCAUUCGACGCCUUUUAAUUCUGACCAUCGCAGGCCAUUCCGCGAACGCCGAAAAUAGAGUUGCAGGUCGGUAGCU
env-203   CUCGUGCUCAAAUUGGCGCUGGAUCGGGUGGCGGAUUUUUAUCGGUUCGAGUGAAUUGAGACGAAUCUCGACGCGCGAAAGCGGAACGACCGCAGGUCUGGAUUUACUGGCAUCGGUUGCAACAUUCUUGAGUGCCUGCUAUUCGAUUUUCGUCGAAAAUAGAGUGGCAGGUCGGUAGCU
env-204   ..................................................................................................................................................ACCGCGUCGAAAAUAGAGUGGCAGGUCGGUAUCU
env-205   CGGGAUUAGCGGGCGGCGGUCGGUGGUGGUCGUGGAAUAUCGGCUAUGGGGAGGGCCGACGCGAUGCGGUAUAAAGUCGUUCGGCAGCAGUCACCCUCUUGCAGACAUUCCGAGCCUUGCAAUUGACAUCAUUCCCGGUCAUUUUUCGGUUGCCGAAAAUAGAGUUGCAGGUCGGUAGCU
env-206   CGGGAUUAGCGGGCGGCGGUCGGUGGUGGUCGUGGAAUAUCGGCUAUGGGGAGGGCCGACGCGAUGCGGUAUAAAGUCGUUCGGCAGCAGUCACCCUCUUGCAGACAUUCCGAGCCUUGCAAUUGACAUCAUUCCCGGUCAUUUUUCGGUUGCCGAAAAUAGAGUUGCAGGUCGGUAGCU
env-208   .........CGGGCGGCGGUCGGUGGUGGUCGUGGAAUAUCGGCUAUGGGGAGGGCCGACGCGAUGCGGUAUAAAGUCGUUCGGCAGCAGUCACCCUCUUGCAGACAUUCCGAGCCUUGCAAUUGACAUCAUUCCCGGUCAUUUUUCGGUUGCCGAAAAUAGAGUUGCAGGUCGGUAGCU
env-209   CGGGAUUAGCGGGCGGCGGUCGGUGGUGGUCGUGGAAUAUCGGCUAUGGGGAGGGCCGACGCGAUGCGGUAUAAAGUCGUUCGGCAGCAGUCACCCUCUUGCAGACAUUCCGAGCCUUGCAAUUGACAUCAUUCCCGGUCAUUUUUCGGUUGCCGAAAAUAGAGUUGCAGGUCGGUAGCU
env-210   GGGAUUAGCGGGCGGCGGACGGUGGUGGUCGUGGAAUAUCGCCUAAGGGGAAGGGCCGACGCGAUGCGGUAUAAAAUCGUUCGGCAGCAGUCACCCUCUUGCAGACAUUCCGAGCCUUGCAAUUGACAUCAUUUCCGGUCAUUUUUCGGUUGCCGAAAAUAGAGUUGCAGGUCGGUAGCU
env-211   GGUGCUGGGCUAUGCGGUGCUGCGGCUGGUCACACGAGUGCGCGAUGAGGCGGACGAUCCGUAUGAUCCGGUGGUGUGAGGGCGAGCCUUUCGGCCCCCGUCUAUCGUCAGGUGCCUUGCCAAUGUCAUCAUCACGAGGCAUCGUGCUCUAGCCGAAAAUAGAGUGGCAGGUCGGUAGCU
env-212   .................AGGGGGCGGGUUGAGGCUGAGCAGUGCUGGAGGUGGUGAGGCCAGCCUGGUCGCACCGAGUAGGUGACCGCUUGCGCCCACAAGCAGCCAUUCGCCUCCUUGCAAUUGACGCCGUGGGAGGGCAUCCUGCGGAUGCCGAAGAUAGAGUGGCCGGUCGGUAGCU
env-213   UGAUCGCCGGGAUCAGGGCGGAGCUAAAUGGAGGCGUUGCUCAGAAAGAGGCCACGUAGAUCACGGAGUUUUCUGAUGCCAACGACUGCUGUCGCCAAAAGCAGCCAUUCGCCGCCUUGCAACUGACGCCGUCAGAGGGCAUCCUGCGGACGCCGAAAAUAGAGUGGCAGGUCGGUAGCU
env-214   GUGUUCGGAUCACCGACGUUCGUAGUCGACGACGAGAUAUGGUUCGGCGAGGAACGGCUUGCCGAAGUCGAGCGGCGGCGCUUGCUGGUUAAUCGUGAUCGAGGGCAGAUACGCCUUGCAGUUCUUAUCGUUGCACGGCAUCUUGCAAGGGCCGAAAAUAGAGUUGCAGGUCGGUAGCU
env-223   GUGUUCGGAUCACCGACGUUCGUAGUCGACGACGAGAUAUGGUUCGGCGAGGAACGGCUUGCCGAAGUCGAGCGGCGGCGCUUGCUGGUUAAUCGUGAUCGAGGGCAGAUACGCCUUGCAGUUCUUAUCGUUGCACGGCAUCUUGCAAGGGCCGAAAAUAGAGUUGCAGGUCGGUAGCU
env-224   ............................................................................................AUCGUGAUCGAGGGCAGAUACGCCUUGCAGUUCUUAUCGUUGCACGGCAUCUUGCAAGGGCCGAAAAUAGAGUUGCAGGUCGGUAGCU
env-225   CGACGCCGCUGAAGAAAAGAACAGCCCUUCGACAAGCUCAGGGCGAACGGUGGUGAGGGGGCAGGCCGGUCCAGACAGGCCGGGCUUCGAACCGUCAUUGCGAAUAGUGGCUGUUCGAAUUUGUCCACGCCGCCUAGACCAUUUCCCAGAUGCCGAAAAUAGAGUUGCAGGUCGGUAGCU
env-226   GCCAAAUGUUCUGACGACGGACAGGGCGGCACCUGCCGGACGAGUAAGGAAAGUGAAACAGCGCCUUCCGGCGCUAUCGGCGAUGGGUGACGACGCAAUUGCUGACGCUCGGCGACUUGCAACCUUUAUCGUCUUAGGCCAUUUUCCGGAUGCUGAAAAUAGAGUUGCAGGUCGGUAGCU
Ssp-8-2   AUCUACCCACCAUUCUUGCCAUUCGGAUGCAACGAUCGGCGCAACGAGCCAAAAGCCGGCCGGCAGGUAUCGGAUGCUGUCGAAAGGCGAGCGCCCAGGUGCGGGCAUCCGACGGCUUGCAAUUGUCGUCGGCACCGACCAUUUUCCAGAUGCCGAAAAUAGAGUUGCAGGUCGGUAGCU
env-227   ...........................................................................................CGGCCCAUUUGCAGACAUUCGGACCUUGCAAUUCGCCCCGUCACCGAUCAUUCUGGAAAUGCCGAAAAUAGAGUUGCAGGUCGGUAGCU
env-228   UCGAGAAGCUGUUCGGACGGAAAUAGAGAAGGUUAGACGACUUCAUCUAUAGUUGCGUCUGAGAUCGAGCGGCCAGGUUGAAUGAUCCCGUCGGCCCAUUUGCAGACAUUCGGACCUUGCAAUUCGCCCCGUCACCGAUCAUUCUGGAAAUGCCGAAAAUAGAGUUGCAGGUCGGUAGCU
env-229   UCGAGAAGCUGUUCGGACGGAAAUAGAGAAGGUUAGACGACUUCAUCUAUAGUUGCGUCUGAGAUCGAGCGGCCAGGUUGAAUGAUCCCGUCGGCCCAUUUGCAGACAUUCGGACCUUGCAAUUCGCCCCGUCACCGAUCAUUCUGGAAAUGCCGAAAAUAGAGUUGCAGGUCGGUAGCU
env-235   UCGAGAAGCUGUUCGGACGGAAAUAGAGAAGGUUAGACGACUUCAUCUAUAGUUGCGUCUGAGAUCGAGCGGCCAGGUUGAAUGAUCCCGUCGGCCCAUUUGCAGACAUUCGGACCUUGCAAUUCGCCCCGUCACCGAUCAUUCUGGAAAUGCCGAAAAUAGAGUUGCAGGUCGGUAGCU
env-236   UCGAGAAGCUGUUCGGACGGAAAUAGAGAAGGUUAGACGACUUCAUCUAUAGUUGCGUCUGAGAUCGAGCGGCCAGGUUGAAUGAUCCCGUCGGCCCAUUUGCAGACAUUCGGACCUUGCAAUUCGCCCCGUCACCGAUCAUUCUGGAAAUGCCGAAAAUAGAGUUGCAGGUCGGUAGCU
env-237   UUGAGAAGCUGUUCGGACGGAAAUAGAGAAGGUUAGACGACUUCAUCUAUAGUUGCGUCUGAGAUCGAGCGGCCAGGUUGAAUGAUCCCGUCGGCCCAUUUGCAGACAUUCGGACCUUGCAAUUCGCCCCGUCACCGAUCAUUCUGGAAAUGCCGAAAAUAGAGUUGCAGGUCGGUAGCU
env-238   UCGAGAAACUGUUCGGUAGAAAAUAGAGAAGGUUAGACGACUUCAUCUAUAGUUGCGCCUGAGGUCGAGCGGCCAGGUUGAAUGAUCCCGUCGGCCCAUUUGCGGACAUUCGGACCUUGCAAUUCGCCCCGUCACCGAUCAUUCUGGAAAUGCCGAAAAUAGAGUUGCAGGUCGGUAGCU
env-239   ..GAGAAACUGUUCGGUAGAAAAUAGAGAAGGUUAGACGACUUCAUCUAUAGUUGCGCCUGAGGUCGAGCGGCCAGGUUGAAUGAUCCCGUCGGCCCAUUUGCGGACAUUCGGACCUUGCAAUUCGCCCCGUCACCGAUCAUUCUGGAAAUGCCGAAAAUAGAGUUGCAGGUCGGUAGCU
env-240   .......CUGUUCGGUAGAAAAUAGAGAAGNUCUAGACGACUUCAUCUAUAGUUGNGNCUGAGGUCNAGCGGCCAGGUUGAAUGAUUCCGUCGGCCCAUUUGCAGACAUUCGGACCUUGCAAUUCGCCCCGUCACCGAUCAUUCUGGAAAUGCCGAAAAUAGAGUUGCAGGUCGGUAGCU
env-241   UCGAGAAGCUGUUCGGACGGAAAUAGAGAAGGUUAGACGACUUCAUCUAUAGUUGCGUCUGAGAUCGAGCGGCCAGGUUGAAUGAUCCCGUCGGCCCAUUUGCAGACAUUCGGACCUUGCAAUUCGCCCCGUCACCGAUCAUUCUGGAAAUGCCGAAAAUAGAGUUGCAGGUCGGUAGCU
env-242   UCGAGAAACUGUUCGGUAGAAAAUAGAGAAGGUUAGACGACUUCAUCUAUAGUUGCGCCUGAGGUCGAGCGGCCAGGUUGAAUGAUCCCGUCGGCCCAUUUGCAGACAUUCGGACCUUGCAAUUCGCCCCGUCACCGAUCAUUCUGGAAAUGCCGAAAAUAGAGUUGCAGGUCGGUAGCU
env-243   UCGAGAAGCUGUUCGGACGGAAAUAGAGAAGGUUAGACGACUUCAUCUAUAGUUGCGUCUGAGAUCGAGCGGCCAGGUUGAAUGAUCCCGUCGGCCCAUUUGCAGACAUUCGGACCUUGCAAUUCGCCCCGUCACCGAUCAUUCUGGAAAUGCCGAAAAUAGAGUUGCAGGUCGGUAGCU
env-244   GACGAGAUUCCCGAGGAUGUUUGGCCUAAGCAACCUGCAGAAUCGGAAUGACAUCACGCCUCGGCUUUUCGAGAUCGCGACGGCAGCUAGCGGUUCACUUGAAGAUAUUCGAUGCCUUGUAAUCGGCACUGCCCCGGGCCAUUUUACGGAUGCCGAAAAUAGAGUUGCAGGUCGGUAGCU
Swi-1-1   CAAUUGUGGUCGACCAUCGCGGCCAGAGCGACGCGGUCCAGGGAACGCACUUUCGCUCGGGAGGGCGGAUGGCUCCAAGUUCGCCGGCUCGGUUCCGGUCGGCAACAUGCAACGCCUUGCAAUUGUCACCGUCGCGCGCCAUUUUCCGGAUGCCGAAAAUAGAGUUGCAGGUCGGUAGUU
Ssp-5-1   CUGGCGCGGGGAAGUGAGCGGCCGCUCGCGCGCCGGCAGGAUGGGGGCGGCGCGGGUGGCAGAUUCAUCGAAGCGGAUGGACGGGAAGCGCCCCACGAGCAGGCAUUCGCCGCCUUGUAUUUGGCGCCCUCGCGGGGCAUCCUGCGGACGCCGAAAAUAGAGUGGCAGGUCGGUAGCU
env-245   AUAAGCUGAGAACAACGCGGAGGUUGCUGCGCCGUGGCUUGGGACGAGAAGCCAUCCACAGACGAGGAACGCCGAGACGCGGUGCUGCGGCGGAUGUUGACGACGCCGAAGCGACCAGCCCAACCGCAUUCAGUCGGCCGCGUCCUUACCUCUGCCAAAUAGAGUUGCGGGUUGGUAGCU
env-246   GCGCCGCACGUGAAGCCAUGGGGCAUGUACGAAUUCGCGGUCGGCGAUCCGGACGGCGUGCUGGUCCGGGUAGGCCGGCCGAUCGGCUGACGCCCGGCCGCAGCCCGCGCGCGGAUUGAAUUCCAGAGUUCCCUGCGUCAUGAUGGGCGCUGCCGGAAAUAGAGCAGCAGGUCGGUAGUU
env-247   ......................................................................................................AGUCCUGCAGGAUGUUGCUCUGUUGGUCGGCAUCGGACAUCAUGGCCUUGCCGAAAAUAGAGUUGCAGGUUGGUAGCU
env-248   ................................................................................................................................GGCUGCCUGACUAUGUCGCGCAUGCCGAGAAUAGAAUUGCAGGUUGGUAGUC
env-249   ......................................................................................................................GCAAAUCGGCGGCUGCCUGACUAUGUCGCGCAUGCCGAGAAUAGAAUUGCAGGUUGGUAGUC
env-250   .....................................................................................................................................CCUGACUAUGUCGCGCAUGCCGAGAAUAGAAUUGCAGGUUGGUAGUC
env-251   ......................................................................................................................................................UGCCGAGAAUAGAAUUGCAGGUUGGUAGUC
env-252   .............................................................................................................................................................GAUAGAAUUGCAGGUUGGUAGUC
env-253   ................................................................................................................AUACGUGCAAAUCGGCGGCUGCCUGACUAUGUCGCGCAUGCCGAGAAUAGAAUUGCAGGUUGGUAGUC
env-254   ...................................................................................................................CGUGCAAAUCGGCGGCUGCCUGACUAUGUCGCGCAUGCCGAGAAUAGAAUUGCAGGUUGGUAGUC
env-255   GAAGCCCAGCAAACCGGAGGUUUGCGCCCGGCGUCUGAGGGCAUGAACAAGCCACUACCGGCGGUCGAAGCAUUCGGCCGCUGGUAUGAGGCCACGUCCCGCGCGGGACGGUCGCCUUGAGCCGAUCCGGCUUCCGCACUAUCUAGCGGAAGCCGGAAAUAGAGUUGCAGGUUGGUAGCU
env-256   ...............................................CACCAAGCGGGUCGUUGGCUAGUGCUUGCCUCAGUUUGGCUGCUCAACCUCAAGCGGUCCUAAGGCGCAUUGCCAUGUCCACUGGCAUCCGGCAUCGUGGCUACGCCGGAAAUAGAGUGGCAGGUUGGUAGUU
          ......................................................................................................................................................................<<<<<<.......>
          .......................................................................................................................................................................02..........
          ○○○○○○○○○○○○○○○○○○○○○○○○○○○○○○○○○○○○○○○○○○○○○○○○○○○○○○○○○○○○○○○○○○○○○○○○○○○○○○○○○○○○○○○○○○○○○○○○○○○●●●●●●●●●●●R●●●●●●●●●●●●●●●●●●●●●A●●●●●●●●●●●GCCGAAAUAGAGU●GCAGG●UGGUR●CU
                                                                                          5′
```

alignment positions 181 · · · 360

```
env-1     CCCGCAGCCGG..UGACC.....UCGGCUUCGUCCCA..GCCACAGCGCCUAACCCCUGCGUCGUCGGGGAGAGGCUCCGUACGACCUGUGCUUGGAGAUGCGUCCUAUGCCUUCCGCCUUGGCUGGCCUUCUGGCGUCGAACUGGUUUGCGGUGCUCGCGCGCGUCGUUCUGACUUUCG
env-2     CCUGCGCUUCCU.UCUG.....AGGGGGUUCUGCUUC..GUCCACGGCGUCGACCUGAGUGUUUUCAGGCUGGCGCGCGUGAUUUUCCCCAUCGGGCCUGACGCGUGCUUCCCUGACGAGAAGGGGGAUAGGACGAUGGGUGAUCGACAGCGGGACGCGGCAUGACCGAAACGACGC
env-4     CCUGCGCUUCCU.UCUG.....AGGGAGUUCUGCUUC..GUCCACGGCGUCGACCUGAGUAGGUUUCCAGGGUUGGCGCGCGUGUUUUCCCAAGAACGGCCCUCGCGUGCUUCCCUGACGAGAAGGGGGUAUUGGGAUGGGCAAUCGAAAGCGGGCCACGGCCGGCGCGGGAGGACCCA
Ral-1-1   CCUGCGCUGACC.AAAU.....GGUCGGUUCUACUUC..GACCACGGCGCAACCCUUAACUUUCGGGGGCGGCGCGUGGGAUAUCAUAUCACUCUCAGCCUGUAAGCACCCUGAUCUGAUUGAUAUGGCGAGACCACAAACAUGAUACACUUUUGAUCUGGUUCCUUGUUGCCUUCUUU
env-5     CCUGCAGCCGG..UUCCCU....CCGGCUUCGUCCCA..GCCGCAGCGCCUAACCCCUGCGUCGUCGGGGAGAGGCCCGUUGGACGUAUGCUCGGAGACACGUCUUAUGCCUUCCAUCUUGGCUAGCCUUCUGGCGUCGAACUGGUUCGCGGUGCUGGCGCGCGUCGUUCUGACGUUCGU
env-6     CCCGUACCUGC..AAUAAAC...GCAGGAUCUUCGUG..GCCACGGCGUUAACCCAUUACAUACUGGGGCAAACGCUGGUGCUUCUUUUUCCGCAUCCUUCGUGUGCCCCUUUUCGGAGGCUCUAUGGAUUACUUCUCUCUCGUCAAUAUUCUUUCCUGGCUUUUAGGUCUCUUUUUCU
Spa-1-1   CCUGCGCUAUCU.UCCA.....AGAGAGUUCUGCCUU..GUCCUCGGCGUCAACCUGGCAAUUCGUCGGGGUUGGCGCAGAGGCUUCCGUUUGUCGGUCUCCCCGUGCGCCUGCCCCGAUUGGGGCAUUCGCGCAAAUGGUCUCAAUCACAAAAACUCCGCUGCUGGAGCCGCUUACAA
env-7     CCUGCGUCCGUC.CCCU.....GACGGAUUCUGCUUA..GUCCUCGGCGUCAACCUGGGCAUUGGUUCGGGGCUGGCGCGGGGUGGCUUUCCGGAUUUUCGGUUCCAUCGCGUGCUUCCCCAACAGGAGGAAGUCACAAAGAUGCGUUUCAGCAAGAUCAGCGCACCGACUGCGAGGGGG
env-8     CCUGCGUCCGUC.CUUU.....GACGGAUUCUGCUUA..GUCCUCGGCGUCAACCUGAGCAUUGGUUCGGGGCUGGCGCGGGGUGGCUUGCCGGGUUUCGGUUCCAUCGCGUGCUUCCCCAACAGGAGGAAGUCACAAAGAUGCGUUUCAGCACAAUCAGCGCAGUACGAGUGACGGCA
env-9     CCUGCGUCCGUC.CCCU.....GACGGAUUCUGCUUA..GUCCUCGGCGUCAACCUGGGCAUUGGUUCGGGGCUGGCGCGGG..................................................................................................
env-10    CCUGCGUCCGUC.CCCU.....GACGGAUUCUGCUUA..GUCCUCGGCGUCAACCUGGGCAUUGGU..................................................................................................................
env-11    CCUGCGUCCGUC.CUUU.....GACGGAUUCUGCUUA..GUCCUC.......................................................................................................................................

          >>>>><<<<<<<<........>>>>>>>>.......................................................................................................................................................
          ..20.1222221.........1222221.........................................................................................................................................................
          CCUGCGCCCR○--●●●●------○YGGGUUCUUCYC●--GCC●CGGCGYY●R●C●●●●●●●●●●●●●R●●R●●●●●●●●●●●●●●●●●●●●●●●●●●●●●●●●●●●●●●●●●●●●●●●●●○○○○○○○○○○○○○○○○○○○○○○○○○○○○○○○○○○○○○○○○○○○○○○○○○○○○○○○○○○○
```

```
env-12    CCUGCGUCCGUC.CCCU.....GACGGAUUCUGCUUA..GUCCUCGGCGUCAACCUGGGCAUUGGUUCGGGGCUGGCGCGGGUGGCUUUCCGGAUUUUCGGUUCCAUCGCGUGCUUCCCCACUAAAAGGGAAGUCACAAAGAUGCGUUUCAGCACAAUCAGCGUACCGGCCGCAGAGGCA
env-13    CCUGCGCCGGCU.UCCG.....GGCCGGUUCUGCAC...GUCCACGGCGUCGACCUGAAUAUGCAUUCGGGGAUGGCGCACGUGGCACGCCGAUCCGGCCCCAUCGUGCGUCCUCUCUGGGAAGGGGGACCGCGCGGAUGUUUUUCGAUCAGCCCGUAUCGGCGUGCCGGGGGACGGCGG
env-14    CCUGCGCUGGCC.UCCG.....GGCCGGUUCUGCAC...GUCCGCGGCGUCGACUGAACAUGGCUUCGGGGAUGGCGCACGUGGCAGGCCGUUUCGGCCCCAUGGCGCGCUUUCCCUGAAAUGGGGGACCGCGCAGAUGCUUUUCGAUCAGACCCUGUUGGCGUGCCGGGGGACGCGG
env-15    CUUGCCAUCGC..UCCUCU....GCGGUCUCAUCCCU..GCCAAGCGCGCAACCCAGACGGGCGCAGCUGCCCGCACGACCGGAGUUGAAUGCCGCAGGCUCUCGCCGGAACUCUGCGCGA......................................................
env-16    CUUGCCAUCGC..UCCUCU....GCGGUCUCAUCCCU..GCCAAGCGCGCAACCCAGACGGGCGCAGCUGCCCGCACGACCGGAGUUGAAUGCCGCAGGCUCUCGCCGGAACUCUGCGCGA......................................................
env-17    CCUGCGCUUGC..CUUGCU....GCAAGUUCUUCCUU..GCCGCGGCGUCGAUCCGAUCUUUCAUCGGGGCAUGGCGCUCACAUUUCGUGGUUUCCGCUUCCGCACGCCCUGUCCCCAGUAAAGGACAGCCCGUGCGCGCAUAUUCUCGCGACUUUUCCUGCCUGACCCGGACCGACCGC
env-18    CCUGCGGCCGG..CUUAGC....CCGGCUUCUUCCUG..GCCUCUGCGCCUAACCCUGCGUCGUCGGGGAAAGGCUCUGCACGACGCUAUGAAAGGAGACAGGAACAUAUGCCCAUCGCAAUCCACCGCAUCCUCACAUCCCGCUGGUUCAACUUUCUGGCGCGCACGGUCCUGACAUUU
env-20    CCUGCGGCCGG..CUUAGC....CCGGCUUCUUCCUG..GCCUCUGCGCCUAACCCUGCGUCGUCGGGGAAAGGCUCUGCACGACGCUAUGAAAGGAGACAGGAACAUAUGCCCAUCGCAAUCCACCGCAUCCUCACAUCCCGCUGGUUCAACUUUCUGGCGCGCACGGUCCUGACAUUU
env-21    CCUGCGGCCGG..CUUAGC....CCGGCUUCUUCCUG..GCCUCUGCGCCUAACCCUGCGUCGUCGGGGAAAGGCUCUGCACGACGCUAUGAAAGGAGACAGGAACAUAUGCCCAUCGCAAUCCACCGCAUCCUCACAUCCCGCUGGUUCAACUUUCUGGCGCGCACGGUCCUGACAUUU
env-22    CCUGCGGCCGG..CUUAGC....CCGGCUUCUUCCUG..GCCUCUGCGCCUAACCCUGCGUCGUCGGGGAAAGGCUCUGCACGACGCUAUGAAAGGAGACAGGAACAUAUGCCCAUCGCAAUCCACCGCAUCCUCACAUCCCGCUGGUUCAACUUUCUGGCGCGCACGGUCCUGACAUUU
env-23    CCUGCGGCCGG..CUUAGC....CCGGCUUCUUCCUG..GCCUCUGCGCCUAACCCUGCGUCGUCGGGGAAAGGCUCUGCACGACGCUAUGAAAGGAGACAGGAACAUAUGCCCAUCGCAAUCCACCGCAUCCUCACAUCCCGCUGGUUCAACUUUCUGGCGCGCACGGUCCUGACAUUU
env-24    CCUGCGGCCGG..CUUAGC....CCGGCUUCUUCCUG..GCCUCUGCGCCUAACCCUGCGUCGUCGGGGAAAGGCUCUGCACGACGCUAUGAAAGGAGACAGGAACAUAUGCCCAUCGCAAUCCACCGCAUCCUCACAUCCCGCUGGUUCAACUUUCUGGCGCGCACGGUCCUGACAUUU
env-25    CCUGCGGCCGG..CUUAGC....CCGGCUUCUUCCUG..GCCUCUGCGCCUAACCCUGCGUCGUCGGGGAAAGGCUCUGCACGACGCUAUGAAAGGAGACAGGAACAUAUGCCCAU.........................................................
env-26    CCUGCGGCCGG..CUUAGC....CCGGCUUCUUCCUG..GCCUCUGCGCCUAACCCUGCGUCGUCGGGGAAAGGCUCUGCACGACGCUAUGAAAGGAGACAGGAACAUAUGCCCAUCGCAAUCCACCGCAUCCUCACAUCCCGCUGGUUCAACUUUCUGGCGCGCACGGUCCUGACAUUU
Cpa-1-1   CCUGCGCCC....AUCUA.......GGGUUCUUCC....GUCCACGGCGUCAACCUUGAACAAGCAUGCUUGAGGAGGCGCACGUGGUCAAAUCCUUGUGUGCCCUCACCCAAGAGGAUUUUGCACACAUGCCAUCCGUUUUCUCACCGAUUCCCCAAGCCGGCGAUGAGGCUAUCACU
Ysp-1-1   CCUGAGCCUGUC.UUGC.....GACAGGUUCUUCUUC..GCCACCGCGUCAAACCUGAAUUGAUCAGGGGGGCGCUUCGACGUGGUUCACGGCCUUUCCUGAUCGUCACGAAAUGACGGACCGUUGAACUUCGGCCGGGAAAGCGAGAGCGCAAUGCAUCCCUGCCUCACCUCCUAUUUU
env-31    CCUGCGCUCACC.UUCG.....GGUGAGUUCUACGA...GCCCACGGCGUCAACCUUACCAAUCAUGGUUCGGACGGCGCGUGGUUCUUUUCUCCAUUGCGCGUCCCCGGACGGAGGAUGUGCGCAGAUGGUGUUCUGCAAGAAACGGACAUGACAGCGGGUGUCGGGAGGAUGGCAU
env-32    CCUGCGCUCACC.UUCG.....GGUGAGUUCUACAA...GCCCACGGCGUCAACCUUACCAAUCAUGGUUCGGACGGCGCGUGGCCUUUUCCCCAUCGCGUCCCUCCGAACGGGAGGAAGCCGCGCAGAUGGUUUUCUGCAAGGAAACGGAAAUGGAAGCGGGCGCCGGGGUGACGGCAU
env-33    CCCGCAAUCGUCCCUC.....GGAUGAUCUCUUCACA..GCCACGGCGCAACCUGACCAAUGUCGGGAAUGCGCAUAACGUGGCUGUUCCGCGUUUAUGGUGCAUCCCGACGAGAAGGAUGUACCCAUGGCAAUCACGAAACAGCGAAUUCUCCCUAAUACCCUUCCAUACUCCAACAAA
env-34    CCCGCAAUCGUCCCUC.....GGAUGAUCUCUUCACA..GCCACGGCGCAACCUGACCAAUGUCGGGAAUGCGCAUAACGUGGCUGUUUCGCGUUUAUGGUGCAUCCCGACGAGAAGGAUGUACCCAUGGCAAUCACGAAACAGCGAAUUCUCCCUAAUACCCUUCCAUACUCCAACAAA
env-35    CCUGCGCCUGC..UUUC.....GCAGGUUCUUCCCA...GCCACGGCGUCGAUCUGAACAAGCGUUCGGACUGGCGCGUGGACCGCUUGUCGCCCCGCACUCUCGUCCGACGCGCACUGGAGUGUAUCACGUGCUCGAACUGUCUGCGUCCAAUGUCAUCUCCUACCUGGUGACCCUG
Bsp-1-1   CCUGCGCCCG...CUAG.......CGGGUCUUCACA...GCCACGGCGUCAACCCGAUUCAGAUCGGGAAGGCGCAGGCUAUUCGCGGCGCCCUCUCAGCUGAAUUUCAGAAGAGAGAAUAUCCAUAUGCCCCGUGCGAUCACUGUGACGAAGGUCGUUCAUGCUCUGGCCGGCACCAUC
env-36    CCUGCGCUGGCC.GCAA.....GGCCGGUUCUUCUCA..GCCACGGCGUCGACCUGAACUUUUGUCCGGGGAUGGCGCGUGGAUUUUGCUUUAUCCUAUCCUUCCGACCGUCCCGCUGUGGAGGCAAAACUGUGCGUUUCUUCUUUCCUCUUGUACGGAAUGGACCAGUCGUGGCCAUCG
Ssp-11-1  CCUGCGCCC....CGAC........GGGUUCUUCCCU..GCCACGGCGUUCAACCUCAUCGGGUGACGGCGCGUGGCGCGAUCCGCGUCCGUCGCGAAGGAGCGCGUGUGAAAUAUCUGUAUCUGGCGUCCGCGAUCAUCGCCGAGGUCAUCGGCACCAGUUUCAUGAAGGAAUCGGACG
Ssp-7-1   CCUGCGCCC....CGAC........GGGUUCUUCCCU..GCCACGGCGUUCAACCCAUCGGGUGACGGCGCGCGGCGCGAUCGCGCCCGUCGCGAAGGAGCGCGUGUGAAAUAUCUGUAUCUGGCGUCCGCGAUCAUCGCAGAGGUCAUCGGCACCAGUUUCAUGAAGGAAUCGGACG
Nsp-2-1   CCUGCGCCC....GUAA........GGGUUCUUCCCA..GCCACGGCGUCAACCCCGGAUGGGCUGACGGCGCGUGACCUGUCGCUGCGCUCCGUCAUGGCCAGGAGCGAUUAUGAAUUAUCUUUACUUGACUGGUGCGAUUGUGGCCGAAGUCAUUGGCACCAGCUUCAUGAAGCAGU
Rsu-1-1   CCUGCGCCCGUC.UUGC.....GACGGGUUCUUCGCA..GCCACGGUGUUCGACCUGAUCCGAUCGGGACGGCACUCUGGCUUGUCUAUGGGCUUCCCGAUCCUCACUACAGACAGGCCGCUCGGUUGCGGUCGGGAAAGCGCAGACCUCAUGCCCUCUUACCUUGUGUCUUAUUCCGC
env-37    CCUGCGCCU....GACAA.......AGGUUCUUCCCA..GCCACGGCGUCAUGUCAACUUCGGAGCGUCUCUUCGCAGACGCUUCUUGUGCAAGGACGCUGAAACACAUGCAAUACCUAUAUCUCGCUAUCGCAGUCGCUGCAGAGGUGAUCGCCACAAGCUUCAUGAAGUCUUCGGAGGG
Sya-3-1   CCUGCGCUUGCC.AGGU.....GGCAAGUUCUUCUCC..GCCGCGGCGUCAACCCGAACCUGUUUCGGGGCAUGACGCGCGGUGUUUACGGCUCUUGCCGACGCACGUCCUGUCCCAGUGAAGGAUAGCCCGUGCGCGCAGACGAUUGUGACCCUUCCCCAUUCUCCCAUGAGCGACUGU
env-40    CCUGCGCUUGCC.AGGU.....GGCAAGUUCUUCUCC..GCCGCGGCGUCAACCCGAACCUGUUUCGGGGCAUGACGCGCGGUGUUUACGGCUCUUGCCGACGCACGUCCUGUCCCAGUGAAGGAUAGCCCGUGCGCGCAGACGAUUGUGACCCUUCCCCAUUCUCCCAUGAGCGACUGU
Sya-1-1   CCUGCGCUUGCC.AGGU.....GGCAAGUUCUUCUCC..GCCGCGGCGUCAACCCGAACCUGUUUCGGGGCAUGACGCGCGGUGUUUACGGCUCUUGCCGACGCACGUCCUGUCCCAGUGAAGGAUAGCCCGUGCGCGCAGACGAUUGUGACCCUUCCCCAUUCUCCCAUGAGCGACUGU
Sya-2-1   CCUGCGCUUGCC.AGGU.....GGCAAGUUCUUCUCC..GCCGCGGCGUCAACCCGAACCUGUUUCGGGGCAUGGCGCGCGGUAUUUGCGGCUCUUGCCGACGCACGUCCUGUCCCAGCGAAGGAUAGCCCGUGCGCGCAGACGAUUGUGACCCCUCCCCAUUCUCCGAUGAGCGACUGU
Ssp-3-1   CCUGCGCUUGCC.AGGU.....GGCAAGUUCUUCUCC..GCCGCGGCGUCAACCCGAACCUGUUUCGGGGCAUGGCGCGCGGUAUUUGCGGCUCUUGCCGACGCACGUCCUGUCCCAGCGAAGGAUAGCCCGUGCGCGCAGACGAUUGUGACCCCUCCCCAUUCUCCGAUGAGCGACUGU
env-42    CCUGCACUUGCC.AGGU.....GGCAAGUUCUUCUCC..GCCGCGGCGUCAACCCGAACCUGUUUCGGGGCAUGGCGCGCGGUAUUUGCGGCUCUUGCCGACGCACGUCCUGUCCCAGCGAAGGAUAGCCCGUGCGCGCAGACGAUUGUGACCCCUCCCCAUUCUCCGAUGAGCGACUGU
env-43    CCCGCAGUCAUCUCUU.....GGAUGAUCUCUUCACA..GCCACGGCGCAAACUGACCAACGGUCGGGAAUGCGCAUAACGUGGCUGUUUCGCGUUCAUGGUGCAUCCCGACGAGAAGGAUGUACGCGUGGCCAUCAGAAAACAACAUAUUCUUGCUAAUACCCUUCCAUACUCCAAUAA
env-44    CCCGCAGUCAUCCCUU.....GGAUGAUCUCUUCACA..GCCACGGCGCAACCUGACCAACGGUCGGGAAUGCGCAUAACGUGGCUGUUUCGCGUUCAUGGUGCAUCCCGACGAGAAGGAUGUACGCGUGGCCAUCAGAAAGCAACAUACUCUCGCUACUACCCUUCCAUACUCCAAUAA
env-45    CCCGCAGUCAUCCCUU.....GGAUGAUCUCUUCACA..GCCACGGCGCAACCUGACCAACGGUCGGGAAUGCGCAUAACGUGGCUUUUUCGCGUUCAUGGUGCAUCCCGACGAGAAGGAUGUACGCGUGGCCAUCAGGAAACAACAUAUUCUCGCUAAUACCCUUCCAUACUCCAAUAA
env-46    CCCGCAGUCAUCCCUU.....GGAUGAUCUCUUCACA..GCCACGGCGCAACCUGACCAAUGGUCGGGAAUGCGCAUAACGUGGCUGUUUCGCGUUCAUGGUGCAUCCCGACGAGAAGGAUGUACGCGUGGCCAUCAGAAAACAACAUAUUCUCGCUAAUACCCUUCCAUACUCCAAUAA
env-47    CCCGCGAUCAUC.CCUCA....GAUGAUCUCUUCACA..GCCACGGCGCAACCUGACUAACGGUCGGGAGUGCGCAUAACGUGGCUGUUUCGCGUUUAUGGUGCAUCCCGACGAGAAGGAUGUACGCAUGGCGAUCACGAAACAACAAAUUCUCCCUAAUACCCUUCCAUACUCCAAUAA
env-48    CCCGCAAUCAUCCCUU.....GGAUGAUCUCUUCACA..GCCACGGCGCAACCUGACCAAUUGUCGGGAAUGCGCAUAACGUGGCUGUUUCGCGUUUAUGGUGCAUCCCGACGAGAAGGAUGUACGCAUGGCAAUCACGAAACAGCGAAUUCUCCCAAAUACCCUUCCAUACUCCAAUAA
env-49    CCCGCGGUCAUC.CCUCA....GAUGAUCUCUUCACA..GCCACGGCGCGACCUGACCAACAGUCGGGAAUGCGCAUGACGUGGCUGUUUUCGCGUUCACGGUGCAUCCCGACGAGAAGGAUGUGCGCGUGGCUAUCACGAAACAGAAUAUUCUCCUUAGCGCCCUGCUCUGGACUAGCA
env-50    CCCGCGGUCAUC.CCUCA....GAUGAUCUCUUCACA..GCCACGGCGCAACCUGACCAACAGUCGGGAAUGCGCAUAACGUGGCUGUUUUCGCGUUC.......................................................................................
env-51    CCCGCGGUCAUC.CCUCA....GAUGAUCUCUUCACA..GCCACGGCGCAACCUGACCAAACAGUCGGGAAUGCGCAUAACGUGGCUGUUUUCGCGUUCACGGUGCAUCCCGACGAGAAGGAUGUGCGCGUGGCUAUCACGAAACAGAAUAUUCUCCUUAGCGCCCUGCUCUGGACUAGC
env-52    CCCGCGGUCAUC.CCUCA....GAUGAUCUCUUCACA..GCCACGGCGCAACCUGACCAACAGUCGGGGAAUGCGCAUAACGUGGCUGUUUUCGCGUUCACGGUGCAUCCCGACUAGAAGGAUGUGCGCGUGGCUAUCUCGAAACCGAAUUUUCUCCUGAGCGCCCUGCUCUGGGCUAGC
env-53    CCUGCGGUCAUC.CCUCA....GAUGAUUUCUUCACA..GCCACGGCGCAACCUGACCAAACUGUCGGGAAUGCGCAUAACGUGGCUGUUUUCGCGUUCACGGUGCAUCCCGACGAGAAGGAUGUGCGCGUGGCCAUCUCGAAACAGAAUAUUCUCCUGAGCGCCCUGCUCUGGGCUAGC
env-54    CCCGCGGUCAUC.CCUCA....GAUGAUCUCUUCACA..GCCACGGCGCAACCUGACCAAACGGUCGGGAAUGCGCAUAACGUGGCUGUUUUCGCGUUCACGGUGCAUCCCGACGAGAAGGAUGUGCGCGUGGCUAUCACGAAACAAAAUAUUCUCCUGAGCGCCCUGCUCUGGGCUAGC
env-55    CCCGCGGUCAUC.CCUCA....GAUGAUUUCUUCACA..GCCACGGCGCAACCUGACCCAACGGUCGGGAAUGCGCAUAACGUGGCUGUUUUCGCGUUCACGGUGCAUCCCGACAAGAAGGAUGUGCGCGUGGCCAUCUCGAAACAGAAUAUUCUCCAGGGCGCCCUGCUCUGGGCUAGC
env-56    CCCGCGGUCAUC.CCUCA....GAUGAUUUCUUCACA..GCCACGGCGCAACCUGACCAACAGUCGGGGAAUGCGCAUAACGUGGCUGUUUUCGCGUUCGCGGUGCAUCCCGACGAGAAGGAUGUGCGCGUGGCUAUCUCGAAACAGGAUUUUCUUCUGAGCGCCCUGCUCUGGGCUAGC
env-57    CCCGCGGUCAUC.CCUCA....GAUGAUUUCUUCACA..GCCACGGCGCAACCUGACCAACAGUCGGGGAAUGCGCAUAACGUGGCUGUUUUCGCGUUCACGGUGCAUCCCGACGAGAAGGAUGUGCGCGUGGCUAUCACGAAACAGAAUAUUCUCCUGAGCGCCCUGCUCUGGACUAGC
env-58    CCCGCGGUCAUC.CCUCA....GAUGAUCUCUUCACA..GCCACGGCGCAACCUGACCAACAGUCGGGGAAUGCGCAUAACGUGGCUGUUUUCGCGUUCACGGUGCAUCCCGAUGAGAAGGAUGUGCGCGUGGCCAUCUCGAAACAGAGUUUUCUCCUGAGCGCCCUGCUCUGGGCUAGC
env-59    CCCGCGAUCAUC.CCUCA....GAUGAUCUCUUCACA..GCCACGGCGCAACCUGACAACAGUCGGGGAUGCGCAUAACGUGGCUGUUUCGCGUUCACGGUGCAUCCCGACGAGAAGGAUGUACACGUGGCCAUCUCGAAACAGAAUAUUCUCCUUCGCGCCCUGAUCUGGACUAGUACG
env-60    CCCGCGGUCAUC.CCUCA....GAUGAUCUCUUCACA..GCCACGGCGCAACCUGACCA..................................................................................................................
env-61    CCCGCGAUCAUC.CCUCA....GAUGAUCUCUUCACA..GCCACGGCGCAACCUGACCAACAGUCGGGAAUGCGCAUAACGUGGCUGUUUCGCGUUCAUGGUGCAUCCCGACGAGAAGGAUGUACGCAUGGCGAUCACGAAGCAGCAAAUUCUCCCUAAGACCCUUCCACACUCCAAUA
env-62    CCCGCGGUCAUC.CCUCA....GAUGAUCUCUUCACA..GCCACGGCGCAACCUGACCAAACUGUCGGGAAUG....................................................................................................
env-63    CCCGCGGUCAUC.CCUCA....GAUGAUCUCUUCACA..GCCACGGCGCAACCUGACCAACAGUCGGGAAUGCGCAUAACGUGGCUGUUUUCGCGUUC.......................................................................................
env-64    CCUGCCCCCG...UUCG.......UGGGGCCGUGACC..GCCACGGCGUCACUUCAACCGGGGGACGCCUUCGCUUUCGCCAGGACGUUCCUCCUCCAGGACGCUGCGUCAUGAAAUACCUCUUCUCUGAUGCUCGCAAUCGUGGGCGAAGCGAUUGGCACGACCUUCCUGAAAGAGUCGGA
Ssp-12-1  CCUGCACCCC...UCAU.......GGGGUUCUUCCCA..GCCACGGCGUCACCCGGAUUCGGGAGGCGCGCACUUGGGUUUGGCGUCUCCUCGAACCAACAUCGAGGAUCGGUCAUGACAUUCGCUCUCGUCGCCCUGAUCAGCGCCACGCUGUUCGCCGGAGCCGCCUCCUAUAUCAGU
env-65    UCUGCGUCCGC..ACC.......GCGGAAUCUUCUCG..GCCACGGCGUCAAGACGCUCUACAUCGAACCGGGAGGUCGUGGGAGACCCGCUAUUGCGAGAGCUUCAACUCAACGCUUCGCGAUGAGCUUCUCGCAAGGGAGAUCUUCUACAAGAUGAAGGAAGCCAGAACGCUGAUUG
env-66    CCUGCACCUGC..GUA.......GCAGGUUCUUCUCA..GCCCCGACGUCGACGUCUGCGGACGCACGAAGCGGGGGAUAGAGUGGGAAAUGUUGCUGUUGCGUCAGCGUGGCUCCUCGGAGCCAUCUUCAUCGUUUUCGCUAUCAGCCACGUAGCGUCCGUCAAAAGCGUCGGUCUUCU
Ssp-10-1  CCUGCACCUGC..GCA.......GCGGGUUCUUCUCA..GCCCCGACGUCGACGUCUGCGGACGCACGAAGUGGGGGAUGAAGUGGGAAAUGUUGCUGUUGCGUCAGCGUGGCUCCUCGGAGCCAUCUUCAUCGUUUUCGCUAUCAGCCACGUAGCGUCCGUCAGAAGCGUCGGGCUUCU
Bst-1-1   CCUGCGCCC....GAUCCA......GGGUUCUAUGUAU.GCCGCGGCGUCAACCUUGGUUUCACGAAUCAAGGGGGCGCACUGGGCAUGACACUGCGCGCCCCCACCCAGGGGGAUUUGUACCGAUGCGCACACACGUCAAAGAUCCGUCGACCGGCAACGGCGCCAUGUUCAGCGA
env-67    CCUGCGUUCGC..UAC.......GCGGAUCUUCCCU...GCCACGGCGUUAACUUGAAAUGGUUCAGGGCGCUUCGCAUAACAU.....................................................................................
She-1-1   CCUGCGUUCGC..UAC.......GCGGAUCUUCCCU...GCCACGGCGUUAACUUGAAAUGGUUCAGGGCGCUUCGCAUAACAUGCUGGCCCGCUGAUCCGACGAGCGGAGCCGAACAUGAAUUACGUCUAUCUUGCCAUAGCGAUCAUUUCCGAGUGAUCGGCACCACGUUUAUGA
Ssp-13-1  CCUGCGCCCGC..AUU.......GCGGGUUCUUCUCA..GCCUCGGCGUCAACUGGCUUGGGUUCGGUGUGACGCGGUGAGGCAACGCGCUCAGGCCACCUGCAAAGCGGAGCGUGAAAUGAACUAUUUUCUUCUUGGACUUGCCAUUGUCGCCGAAGUGCGUCGGCACAUCUUUCAUGAA
env-68    CCUGCGCCCC...GCGA.......GGGGUUCUAUGC...GCCACGGCGUCCACCUUGAUUUCACCGAGUCGAGGGGACGCAUCCGUGGUCGAUCCGAUGCGUGCCCGCGACCGAGGAGU.....................................................
env-69    CCUGCGCCCC...GCGA.......GGGGUUCUAUGCAU.GCCACGGCGUCAACCUGACUUUCACAAGUUGAGGGGGCGCGCGAGGGUAUGGACUGCGCGCCCCGCACGAAUGGGGAUGUGUACCGAUGCGCAAUCACGCAAACGAUCCGGCGGCCGGCAACGGCGUAGUGUUCAGCGACA
Ptr-1-1   CCUGCGCCCC...GCGA.......GGGGUUCUAUGCAU.GCCACGGCGUCAACCUGACUUUCACAAGUUGAGGGGGCGCGCGAGGGUAUGGACUGCGCGCCCCGCACGAAUGGGGAUGUGUACCGAUGCGCAAUCACGCAAACGAUCCGGCGGCCGGCAACGGCGUAGUAUUCAGCGACA
Ptr-1-3   CCUGCGCCCC...GCGA.......GGGGUUCUAUGCAU.GCCACGGCGUCAACCUGACUUUCACAAGUUGAGGGGGCGCGCGAGGGUAUGGACUGCGCGCCCCGCACGAAUGGGGAUGUGUACCGAUGCGCAAUCACGCAAACGAUCCGGCGGCCGGCAACGGCGUAGUAUUCAGCGACA
Ssp-22-1  CCUGCGCUCCCU.UUUG.....AGGGGGUUCUUCUCC..GCCACGGCGUUCGACCAAUUGGGUGGGAACGCUGCCACGACCCGUCGCGGCUUUCCCCGCAGGAGCGUGAAAAUGCACUAUAUCUAUCUGCUGGCCGCUAUCGUCUUCGAGGUGUUGGCAACAUCCUUCCUCAAACAAGCC
Bsp-2-1   CCUGCGCCCG...AGUGC......CGGGUUCUAUAUAU.GCCACGGCGUCAUCCUUUACUUCACCAAGUGGAGGGGACGCAUCGUGGUCAAUCCUCUGCGCCCCUCUGUGCUUAGGAGUUUGACAAUGCGCAGCAACACAGGGGUGCAACCGACCAUCAUGGUGCGGUUCUUGCCGAAA
env-70    CCUGCACACGUC.AAUCGC...GACGGGAUCUUCCCA..GCCACGGCGCCAACCCGACCAGUUUCGGGGAGGCGCGGCUGGCUCUUGGGCUAUUCGCAUCUCUCCUCGAUGGAAAAUGAGGAGAUCGUCUUACUGCUAUCCUUGUUUGUACUCGUGACCGCGACCCUGUUCACCGGCGCUGC
Kvu-2-1   CCUGCACCCG...GGUA.......CGGGAUCUUCUUC..GCCACGGCGUCACACAGGCUUUGGUGUUGGCGCAGGCUUUGACGAUGUCAGCGCAGCCGGAUUAUGAAGGAGACCCGAUGUAUUUAUGCAAUCUUGCCGAAUUCGGCCUUUUCCUUGGUGCCACAUGGCUGGGCUGGG
env-71    CCUGCGCCCG...CAUG.......CGGGUUCUUCUCA..GCCUCGGCGUCCAACGAACCCGGUUCGAAGCGACGCAUGGCUUGCUGCUCCGAACCCAUCGGAGUCCGCCAUGAACUAUCUCUACCUUGGCAUUGCGAUCGCCGCCGAAGUGCUCGGCACCAGCUUCAUGAAACAGUCCGA
env-72    CCUGCGCCCG...CAUG.......CGGGUUCUUCUCA..GCCUCGGCGUCCAACGAACCCGGUUCGAAGCGACGCAUGGCUUGCUGCUCCGAACCCAUCGGAGUCCGCCAUGAACUAUCUCUACCUUGGCAUUGCGAUCGCCGCCGAAGUGCUCGGCACCAGCUUCAUGAAACAGUCCGA
env-73    CCUGCGCCCG...CAUG.......CGGGUUCUUCUCA..GCCUCGGCGUCCAACGAACCCGGUUCGAAGCGACGCAUGGCUUGCUGCUCCGAACCCAUCGGAGUCCGCCAUGAACUAUCUCUUCCUUGGUAUCGCGAUCGCCGCCGAAGUGCUCGGCACCAGCUUCAUGAAACAGUCCGA
env-74    CCUGCGCCCG...CAUG.......CGGGUUCUUCUCA..GCCUCGGCGUCCAACGAACCCGGUUCGAAGCGACGCAUGGCUUGCUGCUCCGAACCCAUCGGAGUCCGCCAUGAACUAUCUCUUCUUGGUAUCGCGAUCGCCGCCGAAGUGCUCGGCACCAGCUUCAUGAAACAGUCCGA
env-75    CCUGCGCCCG...CAUG.......CGGGUUCUUCUCA..GCCUCGGCGUCCAACGAACCCGGUUCGAAGCGACGCAUGGCUUGCCGCUCCG..............................................................................
Psp-1-1   CCUGCGCCAAUC.AUCA.....GGUUGGUUCUGCUGG..ACCGCGGCGUGCCCUUAACUUUUCGAGGGGCGCGCUUGUGGUCCUCUGACGGGCUCCUUGUACGCAUCCUCGAACAAUUCGAAAGAUGAGUACGACAUGACCCAAGCCCGAUCUGGUUUCUGAGCAUUUUCUUCCUGA
Psa-1-1   CCUGCGCCCGUC.CGGU.....GACGGGUUCUUCCCA..GCCACGGCGUCGAACCUGAUCCGAUCGAGGAAGGCGCUUAGACCUGGUUCUGGCCUUUCCCGAUCUCCAUUUUUAUCGGACCGCCGGACGGCGGCCGGGAAAGCGCAAACACCAUGCAGUUCACCUUCGUCUCCUACCUCGC
env-76    CCUGCGCCC....CUCG........GGGUUCUUCCCA..GCCACGGCGUCAACCCGAUUCGUUCGGGAUGGCGCUACUGUGCUGAAGCGCCGCCGCCUCGGACGAUCCUGAUUGGAGAUUGUCCCAUGUACGCCGGAUUGAUGACCCUCACGCUCGCAUCGAUGUUCGCUGGUGCUGCCCUCU
env-77    CCUGCGCCC....CUCG........GGGUUCUUCCCA..GCCACGGCGUCAACCCGAUUCGUUCGGGAUGGCGCUACUGUGCUGAAGCGCCGCCGCCUCGGACGAUCCUG.........................................................
env-78    CCUGCGCCC....CUCG........GGGUUCUUCCCA..GCCACGGCGUCAACCCGAUUCGUUCGGGAUGGCGCUACUGUGCUGAAGCGCCGCCUCGGACGAUCCUGAUUGGAGAUUGUCCCAUGUACGCCGGAUUGAUGACCCUCACGCUCGCAUCGAUGUUCGCUGGUGCUGCCCUCU
Ssp-8-1   CCUGCGCCCG...CAC.......GCGGGUUCGAUUU...GCCGCGGCGUCAACCGACCUGGAUCGGCCAUGCGCGCGUGGACACCGACUCUGUUUCGCCCGCCGUCACGAUCCCGGUUCUGAAAGUGGAACGGAGAAUAUACGUGUGGCAAGCUUACGGCUUUUUAAUCGGUGCAAUCA

          >>>>><<<<<<<<.........>>>>>>>>.....................................................................................................................................................
          ..20.1222221.........1222221........................................................................................................................................................
          CCUGCGCCCRo--•••o------oYGGGUUCUUCYC•--GCC•CGGCGYY•R•C•••••••••••••R••R••••••••••••••••••••••••••••••••••••••••••••••••••••••••••••••••••••••••••••••••••••••••••••••••••••
```

```
Osp-1-1    CCUGAACCCGC..CGCGGC....GCGGGGUCGUCUCC..GUCUCGGCCUCCAUCCCCAGCGAUCGCGCGGGGGAGCGGACAACGUUCCGGUCCCUUCCGAUCCCUGGCCUGCCAGGGUCGCCACGGAGGCCAGAAAGGGUCCGAUAUCCUGUAAAUUCAGGCCGCAGCCUAUCUCGCCC
env-79     CCCGCGCCCG...CAUGU......CGGGUUCUUCCCU..GCCUCGGCGUCGACCUGACCCGGUCAGCGGCGCUGCGCUCAAACAAGCCCGCCCGCUGAUCCAGCGCACGGAGCGUCCCCUGAACUACGUCUUUCUCGCCAUCGCGAUCCUCUCCGAGGUGAUCGGCACCACGUUCAUGAA
env-80     CCUGCGCCCG...CAUGU......CGGGGUCUUCCCU..GCCUCGGCGUCGACCUGACCCGGUCAGCGGCGCUGCGCUCAAACAAGCCCGCCCGCUGAUCCAGCGCACGGAGCGUCCCCUGAACUACGUCUUUCUCGCCAUCGCGAUCCUCUCCGAGGUGAUCGGCACCACGUUCAUGAA
Rma-1-1    CCUGCGCCCGUU.CUA......AACGGGUUCUUCCCA..GCCACGGCGUCAAACCUGAUCUGAUCAGGAAGGCGCAAUGUCUGUGGACCCGGCUUUCCCGAUCAUCACCCUCUUGCGGACCGCCAUCGUGCGGCCGGGAAAGCGCAAUCGUCAUGCAAGUGCAUAUCGUUUCAUAUCUUG
Spa-1-2    CCUGCACCCC...AUC........GGGGUUCUUCCCA..GCCGCGGCGUCACCCCAAAUUCGGGGAGGCGCUGUCCCGUGGGUUGGCGCUUCCUCGAACCAGGCGAGGAACGGUUAUGAUAAUCUCACUUGUUGCUCUGCUCAGCGCGACGCUUUUCACUGGCGCAGCUUUCUACAUCAC
Squ-1-1    CCUGCACCCC...AUC........GGGGUUCUUCCCA..GCCGCGGCGUCACCCCAAAUUCGGGGAGGCGCUGUCCCGUGGGUUGGCGCUUCCUCGAACCAGGCGAGGAACGGUUAUGAUAAUCUCACUUGUUGCUCUGCUCAGCGCGACGCUUUUCACUGGCGCAGCUUUCUACAUCAC
env-81     CCUGCGCCUGUC.UGA......GACGGGUUCUUCUCU..GCCUCGGCGUCAACCUGAUCCGUUCGGGAAUGGCGCUUUGGACCCCCAGUGGCCGGCUCCGAACGACAAAUUGGAAGCCGCGCAUGCCGCCUAUCAUGACCGCGUACAUCGCCCUUGCAUUUGCCAUCGUGCUGGAGGUGA
Sph-1-1    CCUGCGCCCCG..AUA.......CGGGGUUCUUCCCA..GCCACGGCGUUGACCCAACCUGCUUGGGAAGACGCUGGCUAUGGAUUGGCGUCUUCCCGGCUCAUGCCGAGGAUAUUCAUCUUGCCGAUGUCGAUCGCCGCUCUUGUCUGCGCCACGCUUUUCACGGGCGCGGCGGGAUAU
Pno-1-1    CCUGCGCCCAG..UUG.......UUGGGUUCUAAUUCC.GCCGCGGCGUCAACCUUACUUUCACCAAGUUGAGGGGACGCGCUGUGGAGAAGACGCUGCGCACCCCUCAGAAUGAGGAGUCUGCACAGAUGCGCAGCAAUACAAAAGACCCGUCAACCGGAGCCGGAGCGCUGUUCAGCG
Bce-1-1    CCUGCGCCCC...GCGA.......GGGGUUCUAACCAU.GCCGCGGCGUCAACCUUGCUUUCACGAGUCAAGGGGGGCGCACUGUGGUCAUGACGCUGCGCGCCUCCCAUCCAUGGGGAAUUGCACCGAUGCGCACACACGUAAAAGAUCCGUCGACCGGCAACGGGGCGGUGUUCAGCG
env-82     CCUGCAUUC....CCAAGC......GGGUUCUUCCCA..GCCACGGCGUCAUCCGAGAUACGAGAGGCGCCGUCCUGUCGGUUGGUGCCUGCGCGGUGUCGUUCGCAAGAUAAUCCGCGACAUCGCUCGACGCUGUAUGUCACGGAAAUCCGAAGCUCGAUUGUCCCAGGUUGGAAGGGG
env-85     CCUGCGCCCGUC.UCA......GACGGGUUCUUCCCU..GCCUCGGCGUCAACCUGAUCCGUUCGGGAGUGGCGCUUGGGAUUCCAUGGUCGCUUCCGUAUCAUA...........................................................................
env-86     CCUGCGCCCGUC.AGAU.....GACGGGUUCUUCCCA..GCCACGGCGUCUAACCUGAUCUGAUCAGGAAGGCGCCAUGAUUUGGUUCUGGUCCUUCCCGAUCCUCACCAAAUGACGGACCGUUCAAUUGCGGCCGGGAAAGAGCAGACGUUAUGCAGCCUUAUCUUGUGUCCUAUUUUG
env-87     CCUGCGUCCGCA.UAU......UGCGGGUUCUUCCCU..GCCACGGCGUCAACCUGACCAUGUCGGGAGGACGCCUUGUCACCACAGGGCCGUGCUUUCCGACGAUAAAUGGAGCGCACAUGAAUUACAUUCUACUCGCUGUCGCGAUUGUGUCCGAAGUCAUCGGCACCACCUUCAUGA
env-89     CCUGCGCCCGC..AAAUC.....GCGGGUUCUUCCCU..GCCACGGCGUCAACCUGACCCUGUCGGGAGGACGCCUUGUGAUAACAGGGCCGUGCCUUCCGACGAGAAACCGGAGCGCACAUGAAUUACGUUCUUCUCGCGGUAGCGAUCAUAUCCGAAGUGAUCGGAACCACGUUCAUG
env-90     CCUGCGCCCGC..AAAUC.....GCGGGUUCUUCCCU..GCCACGGCGUCAACCUGACCCUGUCGGGAGGACGCCUUGUGAUAACAGGGCCGUGCCUUCCGACGAGAAACCGGAGCGCACAUGAAUUACGUUCUUCUCGCGGUAGCGAUCAUAUCCGAAGUGAUCGGAACCACGUUCAUG
env-91     CCUGCGCCCGC..AAAUC.....GCGGGUUCUUCCCU..GCCACGGCGUCAACCUGACCCUGUCGGGAGGACGCCUUGUGAUAACAGGGCCGUGCCUUCCGACGAG..........................................................................
env-92     CCUGCGCCCGC..AAAUC.....GCGGGUUCUUCCCU..GCCACGGCGUCAACCUGACCCUGUCGGGAGGACGCCUUGUGAUAACAGGGCCGUGCCUUCCGACGAGAAACCGGAGCGCACAUGAAUUACGUUCUUCUCGCGGUAGCGAUCAUAUCCGAAGUGAUCGGAACCACGUUCAUG
env-95     CCUACGCCCG...CUAACAA....CGGGUUCUUCCCA..GCCACGGCGUCAACCGAACCUCGUUCGGGAGGCGCGUGGGCAUCGGUUCCGCAUCUCCUUGGGCGAACCUCACAGGAGACAACUUAUAGCCGUUCCGCUGUUU......................................
env-96     CCUGCGCCCGU..UACC......ACGGGUUCUUCCCA..GCCACGGCGUCGAAC..............................................................................................................................
env-97     CCUGCGCCC....UCAUGUC.....GGGUUCUUCCCU..GCCACGGCGUCAUCUGAUCGAAAGGGGAGGCGCUUGACCAUCGGGCCAACGCUCCCCAGAAACCAAGGAGCGUUUUAUGCAGUAUCUCUAUCUCGCGAUCGCCAUCAUUGGUGAAGUCUUCGCGACAUCGUUCCUCCGCCA
env-98     CCUGCGCCC....UCAUGUC.....GGGUUCUUCCCU..GCCACGGCGUCAUCUGAUCGAAAGGGGAGGCGCUUGACCAUCGGGCCAACGCUCCCCAGAAACCAAGGAGCGUUUUAUGCAGUAUCUCUAUCUCGCGAUCGCCAUCAUUGGUGAAGUCUUCGCGACAUCGUUCCUCCGCCA
env-99     CCUGCGCCC....UCAUGUC.....GGGUUCUUCCCU..GCCACGGCGUCAUCUGAUCGAAAGGGGAGGCGCUUGACCAUCGGGCCAACGCUCCCCAGAAACCAAGGAGCGUUUUAUGCAGUAUCUCUAUCUCGCGAUCGCCAUCAUUGGUGAAGUCUUCGCGACAUCGUUCCUCCGCCA
env-100    CCUGCGCCC....UCAUGUC.....GGGUUCUUCCCU..GCCACGGCGUCAUCUGAUCGAAAGGGGAGGCGCUUGACCAUCGGGCCAACGCUCCCCAGAAACCAAGGAGCGUUUUAUGCAGUAUCUCUAUCUCGCGAUCGCCAUCAUUGGUGAAGUCUUCGCGACAUCGUUCCUCCGCCA
env-101    CCUGCGCCC....UCAUGUC.....GGGUUCUUCCCU..GCCACGGCGUCAUCUGAUCGAAAGGGGAGGCGCUUGACCAUCGGGCCAACGCUCCCCAGAAACCAAGGAGCGUUUUAUGCAGUAUCUCUAUCUCGCGAUCGCCAUCAUUGGUGAAGUCUUCGCGACAUCGUUCCUCCGCCA
env-102    CCUGCGCCC....UCAUGUC.....GGGUUCUUCCCU..GCCACGGCGUCAUCUGAUCGAAAGGGGAGGCGCUUGACCAUCGGGCCAACGCUCCCCAGAAACCAAGGAGCGUUUUAUGCAGUAUCUCUAUCUCGCGAUCGCCAUCAUUGGUGAAGUCUUCGCGACAUCGUUCCUCCGCCA
env-103    CCUGCGCCC....UCAUGUC.....GGGUUCUUCCCU..GCCACGGCGUCAUCUGAUCGAAAGGGGAGGCGCUUGACCAUCGGGCCAACGCUCCCCAGAAACCAAGGAGCGUUUUAUGCAGUAUCUCUAUCUCGCGAUCGCCAUCAUUGGUGAAGUCUUCGCGACAUCGUUCCUCCGCCA
env-104    CCUGCGCCC....UCAUGUC.....GGGUUCUUCCCU..GCCACGGCGUCAUCUGAUCGAAAGGGGAGGCGCUUGACCAUCGGGCCAACGCUCCCCAGAAACCAAGGAGCGUUUUAUGCAGUAUCUCUAUCUCGCGAUCGCCAUCAUUGGUGAAGUCUUCGCGACAUCGUUCCUCCGCCA
env-118    CCUGCGCCC....UCAUGUC.....GGGUUCUUCCCU..GCCACGGCGUCAUCUGAUCGAAAGGGGAGGCGCUUGACCAUCGGGCCAACGCUCCCCAGAAACCAAGGAGCGUUUUAUGCAGUAUCUCUAUCUCGCGAUCGCCAUCAUUGGUGAAGUCUUCGCGACAUCGUUCCUCCGCCA
env-119    CCUGCGCCC....UCAUGUC.....GGGUUCUUCCCU..GCCACGGCGUCAUCUGAUCGAAAGGGGAGGCGCUUGACCAUCGGGCCAACGCUCCCCAGAAACCAAGGAGCGUUUUAUGCAGUAUCUCUAUCUCGCGAUCGCCAUCAUUGGUGAAGUCUUCGCGACAUCGUUCCUCCGCCA
env-120    CCUGCGCCC....UCAUGUC.....GGGUUCUUCCCU..GCCACGGCGUCAUCUGAUCGAAAGGGGAGGCGCUUGACCAUCGGGCCAACGCUCCCCAGAAACCAAGGAGCGUUUUAUGCAGUAUCUCUAUCUCGCGAUCGCCAUCAUUGGUGAAGUCUUCGCGACAUCGUUCCUCCGCCA
env-121    CCUGCGCCC....UCAUGUC.....GGGUUCUUCCCU..GCCACGGCGUCAUCUGAUCGAAAGGGGAGGCGCUUGACCAUCGGGCCAACGCUCCCCAGAAACCAAGGAGCGUUUUAUGCAGUAUCUCUAUCUCGCGAUCGCCAUCAUUGGUGAAGUCUUCGCGACAUCGUUCCUCCGCCA
Ssp-9-1    CCUGCACCCC...AAGC.......GGGGUUCUUCCCA..GCCACGGCGUCACCCGAAACUCGGGAGGCGCGGUCGUGCGGGUUGGCGCCUCCUCGAACCAAGCGAGGAACGGCUAUGAUAAUUUCACUUGUUGCUUUGCUCGGUGCAACGCUUUUCACUGGCGCAGCGCUCUAUAUCACC
Ssp-2-1    CCUGCACCCC...ACC........GGGGUUCUUCCCA..GCCGCGGCGUCACCCCAAUUCGGGGAGGCGCUGUCCCGUGGGUUGGCGCUUCCUCGAACCAGGCGAGGAAGGGUUAUGAUAAUCUCACUUGUUGCUCUGCUCAGCGCGACGCUUUUCACUGGCGCAGCUUUCUACAUCACG
env-122    CCUGCGCCC....CCGAGA......GGGUUCUUCCCA..GCCACGGCGUCAACCUGACCAUGUCGGGGCGACGCUUCGUGAAGGCGUGCGUUCCGAUCGAAAAGAGGAACGCGCGUGAAUUAUCUGCUGCUUGCCGCCGCCAUCGUCGCCGAAGUGAUCGGCACCAGUUUCAUGAAGCAG
Npa-1-1    CCUGCGCCC....CCGAGA......GGGUUCUUCCCA..GCCACGGCGUCAACCUGACCAUGUCGGGGCGACGCUUCGUGAAGGCGUGCGUUCCGAUCGAAAAGAGGAACGCGCGUGAAUUAUCUGCUGCUUGCCGCCGCCAUCGUCGCCGAAGUGAUCGGCACCAGUUUCAUGAAGCAG
Pba-1-1    CCUGCGCCCA...CUUGC......UGGGUUCUUACUAC.GCCGCGGCGUCAACCUUACUUUGAACAAGUCGAGGGGGCGCGCUGUGGACAAGACGCUGCGCCCUCUCACGAUGAGGAGUCUGUACCGAUGCGCAGCCAUACCCAGGAACCCGCCCCCGCAGAAGGCACGCUAUUCAGCGA
env-124    CCUGCGCCCGC..ACC.......GCGGGUUCUUCCCA..GCCACGGCGUUGAUCUGACGACGGUCGGGUUGGCGCACGCGUAUGGGGAUCGCCUCUACCCGUUGCCGGUCCGCGCAACGGAGUGGGGCAUGUGCAUAAGCUGACUUUUCGAGACUUCGGCGCGUUGGCCUUGAGCCUGUU
env-125    CCUGCGCCCGC..ACC.......GCGGGUUCUUCCCA..GCCACGGCGUUGAUCUGACGACGGUCGGGUUGGCGCACGCGUAUGGGGAUCGCCUCUACCCGUUGCCGGUCCGCGCAACGGAGUGGGGCAUGUGCAUAAGCUGACUUUUCGAGACUUCGGCGCGUUGGCCUUGAGCCUGUU
env-126    CCUGCGCCCGC..AUAUC.....GUGGGUUCUUCCCU..GCCGCGGCGUAAACCUGACCAUGUCGGGAAGACGCCUUGUUAUGACAGGGCCGUGCUUUCCGAUGGAAAAACGGAGCGCACAUGAAUUACGUCCUACUCGCGGUGGCGAUUGUGUCUGAAGUGAUCGGCACAACUUUCAUG
env-127    CCUGCGCCCGC..AUAUC.....GUGGGUUCUUUCCCU.GCCGCGNCGUUCAACUGAACAUGUCGGGAAGACGCCUUGNUAUGACCAGCUCGUGCUUUCCCGAUGGAAAAUGCUGAUUCCGAGGAAGGCCGCCCCCUCAUCCCAAAUGAAUGCCGCCCCCCUGUCCCAAAAAUACUCGCC
env-128    CCUGCGCCCGC..AUAUC.....GUGGGUUCUUCCCU..GCCGCGGCGUCAACCUGACCAUGUCGGGAAGACGCCUUGUUAUGACAGGGCCGUGCUUUCCGAUGGAAAAAUGCUGAUUCCGAGGGAAGCCGCCCCCUCAUUCCGAAAUGAUGCAGCCCCCC...................
Ssp-3-2    CCUGCGCCCGC..AUAUC.....GUGGGUUCUUCCCU..GCCGCGGCGUCAACCUGACCAUGUCGGGAAGACGCCUUGUUAUGACAGGGCCGUGCUUUCCGAUGGAAAAAUGCUGAUUCCGAGGGAAGCCGCCCCCUCAUUCCGAAAUGAUGCCGCCCCCCUGUUCCGAGAAUUACUCGC
env-129    CCUGCGCCCGC..AUAUC.....GUGGGUUCUUCCCU..GCCGCGGCGUCAACCUGACCAUGUCGGGAAGACGCCUUGUUAUGACAGGGCCGUGCUUUCCGAUGGAAAAAUGCUGAUUCCGAGGGAAGCCGCCCCCUCAUUCCGAAAUGAUGCCGCCCCCCUGUUCCGAGAAUUACUCGC
Ssp-6-1    CCUGCGCCCGC..AUAUC.....GUGGGUUCUUCCCU..GCCGCGGCGUCAACCUGACCAUGUCGGGAAGACGCCUUGUUAUGACAGGGCCGUGCUUUCCGAUGGAAAAAUGCUGAUUCCGAGGGAAGCCGCCCCCUCAUUCCGAAAUGAUGCCGCCCCCCUGUUCCGAGAAUUACUCGC
Peb-1-1    CCUGCGCCCAA..UCA.......UUGGGUUCUUACUAC.GCCGCGGCGUCAACCUUACUUUGACCAAGUUGAGGGGACGCGCUGUGGACCAGACGCUGCGCGCCCCUCCCUGAUGAGGAGUCUGCACAGAUGCGCAGCAAUAUCCAGGGAAUCCAGGGAAUCCAGGCAAUCCAGGCAAUC
env-130    CCUGCGCCC....CCGAGA......GGGUUCUUCCCA..GCCACGGCGUCAACCUGACCAUGUCGGGGCGACGCUUCGUGAAGGCGUGCGUUCCGAUCGAAAAAAAGGAACGCGCGUGAAUUAUCUGCUGCUUGCCGCCGCCAUCGUUGCCGAAGUGAUCGGCACCAGUUUCAUGAAGCA
env-131    CCUGCGCCC....CCGAGA......GGGUUCUUCCCA..GCCACGGCGUCAACCUGACCAUGUCGGGGCGACGCUUCGUGAAGGCGUGCGUUCCGAUCGAAAAAGAGGAACGCGCGUGAAUUAUCUGCUGCUUGCCGCCGCCAUCGUCGCUGAAGUGAUCGGCACCAGUUUCAUGAAGCA
env-132    CCUGCGCCC....CCGAGA......GGGUUCUUCCCA..GCCACGGCGUCAACCUGACCAUGUCGGGGCGACGCUUCGUGAAGGCGUGCGUUCCGAUCGAAAAAGAGGAACGCGCGUGAAUUAUCUGCUGCUUGCCGCCGCCAUCGUUGCCGAAGUGAUCGGCACCAGUUUCAUGAAGCA
Sxe-1-1    CCUGCAUCCGC..UUU.......GCGGAUUCUUCGCA..GCAGCGGCGUCGAACCGAGAAUCUCGGGGCGGCGCAACUGGCGUGGCGUCGCCUCGGAAAUGCCGAGGAUCCAAUUAUGAGCCUGGUUUCUGUUAUCGCGCUCGUUUCCGCGACACUUUUUGCCGGUGCCGCGCUGUAUAU
Ssp-23-1   CCUGCGCCCG...CUUGA......CGGGUUCUUCUCC..GCCACGGCGCCAACCAGCCUUUGCGGCAGGUGGCGCUCAAUUGCAGUUGCCGCCCCGCCGCCACCGACGACGGAGCAUCGCCAUGCAAUAUAUCUACCUCGCCAUCGCCAUCGUUGCAGAGGUCAUUGCAACAAGCUUUAU
Esp-2-1    CCUGCGCCCGC..UACA......GCUGGUUCUUCUCU..GCCUCGGCGUCAAUCGAACCAAGUUCGGUGUGACGCGAUGAGGUUUCGCGCUCCCGGACCAAAUGUGGAGCGCGAGUAUGAAAUACGUUUAUUUGAGUUUGGCCAUAAUCGCUGAAGUGAUCGGCACCACCUUCAUGAAGC
Esp-1-1    CCUGCGCCCGC..UACA......GCUGGUUCUUCUCU..GCCUCGGCGUCAAUCGAACCAAGUUCGGUGUGACGCGAUGAGGUUUCGCGCUCCCGGACCAAAUGUGGAGCGCGAGUAUGAAAUACGUUUAUUUGAGUUUGGCCAUAAUCGCUGAAGUGAUCGGCACCACCUUCAUGAAGC
env-133    CCUGCGCCCGU..UUCA......GCGGGUUCUUCUCU..GCCUCGGCGUCAAUCGAACUGGGUUCGGUGUGACGCGAUGAGGUUUCGCGCUCCCGGACCAAAUGUGGAGCGCGAGUAUGAAAUACGUUUAUUUGAGUUUGGCCAUAAUCGCUGAAGUGAUCGGCACCACCUUCAUGAAGC
Efl-1-1    CCUGCGCCCGC..GUCA......GCGGGUUCUUCUCU..GCCUCGGCGUCAAUCGAACCAGGUUCGGAGUGACGCGAUGAGGUUUCGCGCUCCCGGACCAAAUACGGAGCGCGAGCAUGAACUAUGUUUAUCUGAUUUUGGCCAUAAUUGCCGAAGUGAUCGGCACCACCUUUAUGAAGC
env-134    CCUGCGCCCGC..UUCA......GCGGGUUCUUCUCU..GCCUCGGCGUCAAUCGAACCGGGUUCGGUGUGACGCGAUGAGGUUUCGCGCUCCCGAACCAAAUGUGGAGCGAGAGCAUGAACUACGUUUAUUUGAGUUUGGCCAUAAUCGCCGAAGUGAUCGGCACCACCUUUAUGAAGC
env-135    CCUGCGCCCGC..UUCA......GCGGGUUCUUCUCU..GCCUCGGCGUCAAUCGAACCGGUUCGGUGUGACGCGAUGAGGUUUCGCGCUCCCGGACCAAAUGUGGAGCGAGAGCAUGAACUACGUUUAUUUGAGUUUGGCUAUAAUCGCCGAAGUGAUCGGCACCACCUUUAUGAAGCA
env-136    CCUGCGCCCGC..UUCA......GCGGGUUCUUCUCU..GCCUCGGCGUCAAUCGAACCGGUUCGGUGUGACGCGAUGAGGUUUCGCGCUCCCGGACCAAAUGUGGAGCGAGAGCAUGAACUACGUUUAUUUGAGUUUGGCUAUAAUCGCCGAAGUGAUCGGCACCACCUUUAUGAAGCA
env-137    CCUGCGCCCGC..UUCA......GCGGGUUCUUCUCU..GCCUCGGCGUCAAUCGAACCGGUUCGGUGUGACGCGAUGAGGUUUCGCGCUCCCGGACCAAAUGUGGAGCGAGAGCAUGAACUACGUUUAUUUGAGUUUGGCUAUAAUCGCCGAAGUGAUCGGCACCACCUUUAUGAAGCA
env-138    CCUGCGCCCGC..UUCA......GCGGGUUCUUCUCU..GCCUCGGCGUCAAUCGAACCGGUUCGGUGUGACGCGAUGAGGUUUCGCGCUCCCGGACCAAAUGUGGAGCGAGAGCAUGAACUACGUUUAUUUGAGUUUGGCUAUAAUCGCCGAAGUGAUCGGCACCACCUUUAUGAAGCA
env-139    CCUGCGCCCGC..UUCA......GCGGGUUCUUCUCU..GCCUCGGCGUCAAUCGAACCGGUUCGGUGUGACGCGAUGAGGUUUCGCGCUCCCGGACCAAAUGUGGAGCGAGAGCAUGAACUACGUUUAUUUGAGUUUGGCUAUAAUCGCCGAAGUGAUCGGCACCACCUUUAUGAAGCA
env-155    CCUGCACCCA...CAUC.......UGGGUUCUUCUCA..GCCGCGGCGUUGACCCAACUUGUUUGGGGAGGCGCUGGCUGUUUUUUUUCGCGUCUCCCCGGCUCAUGCCGAGGAACCCUGAUGUCGCUUUUCUAUAUCAUUGCACUGGUGUCCGCCGCCUUGUUUGCCGGUGCGGCAGCU
env-156    CCUGCACCCA...CAUC.......UGGGUUCUUCUCA..GCCGCGGCGUUGACCCAACUUGUUUGGGGAGGCGCUGGCUGUUUUUUUUCGCGUCUCCCCGGCUCAUGCCGAGGAACCCUGAUGUCGCUUUUCUAUAUCAUUGCACUGGUGUCCGCCGCCUUGUUUGCCGGUGCGGCAGCU
env-157    CCUGCACCC....ACAUCA......GGGUUCUUCUCA..GCCGCGGCGUUGACCCAACUUGUUUGGGGAGGCGCUGGCUGUUUUUUUUCGCGUCUCCCCGGCUCAUGCCGAGGAAUCCUAAAUGUCGCUUUUCUAUAUCAUUGCACUGGUGUCCGCCGCCUUGUUUGCCGGUGCGGCAGCU
env-158    CCUGCACCC....ACAUCC......GGGUUCUUCUCA..GCCGCGGCGUUGACCCAACUUGUUUGGGGAGGCGCUGGCUGUUUUUUUUCGCGUCUCCCCGGCUCAUGCCGAGGAACCCUAAAUGUCGCUUUUCUAUAUCAUUGCACUGGCGUCCGCCGCCUUGUUUGCCGGUGCGGCAGCU
env-160    CCUGCACCC....ACAUCA......GGGUUCUUCUCA..GCCGCGGCGUUGACCCAACUUGUUUGGG...................................................................................................................
env-161    CCUGCACCC....ACAUCA......GGGUUCUUCUCA..GCCGCGGCGUUGACCCAACUUGUUUGGG...................................................................................................................
env-162    CCUGCACCC....AUAUCC......GGGUUCUUCUCA..GCCGCGGCGUUGACCCAAUUUGUUUGGGGAGGCGCUGGCUGUUUUUUUCGCGUCUCUCCGGCUCAUGCCGAGGAACCCUGAUGUCGCUUUUCUAUAUCAUUGCACUGGCGUCUGCCGCGUUGUUUGCCGGUGCGGCAAGUU
env-163    CCUGCACCC....AUAUCC......GGGUUCUUCUCA..GCCGCGGCGUUGACCCAAUUUGUUUGGGGAGGCGCUGGCUGUUUUUUUCGCGUCUCUCCGGCUCAUGCCGAGGAACCCUGAUGUCGCUUUUCUAUAUCAUUGCACUGGCGUCUGCCGCGUUGUUUGCCGGUGCGGCAAGUU
env-165    CCUGCACCC....AUAUCC......GGGUUCUUCUCA..GCCGCGGCGUUGACCCAACUUGUUUGGGGAGGCGCUGGCUGUUUUUUUCGCGUCUCUCCGGCUCAUGCCGAGGAACCCUGAUGUCGCUUUUCUAUAUCAUUGCACUGGCGUCUGCCGCGUUGUUUGCCGGUGCGGCAAGUU
env-166    CCUGCACCC....AUAUCC......GGGUUCUUCUCA..GCCGCGGCGUUGACCCAAUUUGUUUGGGGAGGCGCUGGCUGUUUUUUUCGCGUCUCUCCGGCUCAUGCCGAGGAACCCUGAUGUCGCUUUUCUAUAUCAUUGCA.....................................
env-168    CCUGCACCC....AUAUCC......GGGUUCUUCUCA..GCCGCGGCGUUGACCCAAUUUGUUUGGGGAGGCGCUGGCUGUUUUUUUCGCGUCUCCCCGGCUCAUGCCGAGGAACCCUGAUGUCGCUUUUCUAUAUCAUUGCACUGGUGUCCGCCGCCUUGUUUGCCGGUGCGGCAGCUU
env-169    CCUGCACCCA...CAUU.......UGGGUUCUUCUCA..GCCGCGGCGUUGACCCAACCUGUGUGGGGAGGCGCUGGCUGUUUUUUCGCGUCUCUCCGGCUCAUGCCGAGGAACCCUGAUGUCGCUUUUCUACAUCAUUGCACUGGUGUCUGCCGCGUUGUUUGCCGGUGCGGCAGCUUA
env-170    CCUGCACCCA...CAUU.......UGGGUUCUUCUCA..GCCGCGGCGUUGACCCAACCUGUGUGGGGAGGCGCUGGCUGUUUUUUCGCGUCUCUCCGGCUCAUGCCGAGGAACCCUGAUGUCGCUUUUCUACAUCAUUGCACUGGUGUCUGCCGCGUUGUUUGCCGGUGCGGCAGCUUA
env-171    CCUGCACCCA...CAUC.......UGGGUUCUUCUCA..GCCGCGGCGUUGACCCAACCUGUUUGGGGAGGCGCUGGCUGUUUUUUCGCGUCUCUCCGGCUCCUGCCGAGGAACCCUGAUGUCGCUUUUCUAUAUCAUUGCACUGGUGUCUGCCGCGUUGUUUGCCGGUGCGGCAAGUUA
env-172    CCUGCACCCA...CAUC.......UGGGUUCUUCUCA..GCCGCGGCGUUGACCCAACUUGUUUGGGGAGGCGCUGGCUGUUUUUUUCGCGUCUCCCCGGCUCAUGCCGAGGAACCCUGAUGUCGCUUUUCUAUAUCAUUGCACUGGUGUCCGCCGCCUUGUUUGCCGGUGCGGCAGCUU
env-173    CCUGCACCCA...CAUC.......UGGGUUCUUCUCA..GCCGCGGCGUUGACCCAACUUGUUUGGGGAGGCGCUGGCUGUUUUUUCGCGUCUCUCCGGCUCCUGCCGAGGAACCCUGAUGUCGCUUUUCUAUGUCAUUGCACUGGCGUCCGCCGCCUUGUUUGCCGGUGCGGCAGCUUA
env-174    CCUGCACCCA...CAUC.......UGGGUUCUUCUCA..GCCGCGACGUUGACCCAACUUGUUUGGGGAGGCGCUGGCUGUUUUUUUCGCGUCUCCCCGGCUCAUGCCGAGGAACCCUGAUGUCGCUUUUCUAUAUCAUUGCACUGGUGUCCGCCGCCUUGUUUGCCGGUGCGGCAGCUU
env-175    CCUGCACCCA...CAUC.......UGGGUUCUUCUCA..GCCGCGGCGUUGACCCAACUUGUUUGGGGAGGCGCUGGCUGUUUUUUUCGCGUCUCCCCGGCUCAUGCCGAGGAACCCUGAUGUCGCUUUUCUAUAUCAUUGCACUGGUGUCCGCCGCCUUGUUUGCCGGUGCGGCAGCUU
Esp-3-1    CCUGCACCCA...CAUC.......UGGGUUCUUCUCA..GCCGCGGCGUUGACCCAACUUGUUUGGGGAGGCGCUGGCUGUUUUUUUCGCGUCUCCCCGGCUCAUGCCGAGGAACCCUGAUGUCGCUUUUCUAUAUCAUUGCACUGGUGUCCGCCGCCUUGUUUGCCGGUGCGGCAAGUU
env-176    CCUGCACCCA...CAUC.......UGGGUUCUUCUCA..GCCGCGGCGUUGACCCAAUUUGUUUGGGGAGGCGCUGGCUGUUUUUUUUCGCGUCUCCCCGGCUCAUGCCGAGGAAUCCUAAAUGUCGCUUUUCUAUAUCAUUGCACUGGUGUCCGCCGCCUUGUUUGCCGGUGCGGCAGCU
env-179    CCUGCACCCA...CAUC.......UGGGUUCUUCUCA..GCCGCGGCGUUGACCCAAUUUGUUUGGGGAGGCGCUGGCUGUUUUUUUUCGCGUCUCCCCGGCUCAUGCCGAGGAAUCCUAAAUGUCGCUUUUCUAUAUCAUUGCACUGGUGUCCGCCGCCUUGUUUGCCGGUGCGGCAGCU
env-180    CCUGCACCCA...CAUC.......UGGGUUCUUCUCA..GCCGCGGCGUUGACCCAAUUUGUUUGGGGAGGCGCUGGCUGUUUUUUUUCGCGUCUCCCCGGCUCAUGCCGAGGAAUCCUAAAUGUCGCUUUUCUAUAUCAUUGCACUGGUGUCCGCCGCCUUGUUUGCCGGUGCGGCAGCU

           >>>>><<<<<<<<........>>>>>>>>.......................................................................................................................................................
           ..20.1222221.........1222221.......................................................................................................................................................
           CCUGCGCCCR○--●●●○------○YGGGUUCUUCYC●--GCC●CGGCGYY●R●C●●●●●●●●●●●●●●R●●R●●●●●●●●●●●●●●●●●●●●●●●●●●●●●●●●●●●●●●●●●●●●●●●●○○○○○○○○○○○○○○○○○○○○○○○○○○○○○○○○○○○○○○○○○○○○○○○○○○○○○○○○○○○
```

```
env-181    CCUGCACCCA...CAUC.......UGGGUUCUUCUCA..GCCGCGGCGUUGACCCAAUUUGUUUGGGGAGGCGCUGGCUGUUUUUUUCGCGUCUCCCGGCUCAUGCCGAGGAAUCCUAAAUGUCGCUUUUCUAUAUCAUUGCACUGGUGUCCGCCGCCUUGUUUGCCGGUGCGGCAGCU
env-182    CCUGCACCCA...CAUC.......UGGGUUCUUCUCA..GCCGCGGCGUUGACCCAAUUUGUUUGGGGAGGCGCUGG.................................................................................................
env-183    CCUGCACCCA...CAUC.......UGGGUUCUUCUCA..GCCGCGGCGUUGACCCAACUUGUUUGGGGAGGCGCUGGCUGUUUUUU.......................................................................................
env-184    CCUGCACCCA...CAUC.......UGGGUUCUUCUCA..GCCGCGGCGUUGACCCAACUUGUUUGGGGAGGCGCUGGCUGUUUUUU.......................................................................................
env-185    CCUGCACCCA...CAUC.......UGGGUUCUUCUCA..GCCGCGGCGUUGACCCAACUUGUUUGGGGAGGCGCUGGCUGUUUUUU.......................................................................................
env-186    CCUGCACCCA...CAUC.......UGGGUUCUUCUCA..GCCGCGGCGUUGACCCAACUUGUUUGGGGAGGCGCUGGCUGUUUUUUUCGCGUCUCCCGGCUCAUGCCGAGGAACCCUGAUGUCGCUUUUCUAUAUCAUUGCACUGGUGUCCGCCGCCUUGUUUGCCGGUGCGGCAGCUU
env-187    CCUGCACCCA...CAUC.......UGGGUUCUUCUCA..GCCGCGGCGUUGACCCAACUUGUUUGGGGAGGCGCUGGCUGUUUUUUUCGCGUCUCCCGGCUCAUGCCGAGGAACCCUGAUGUCGCUUUUCUAUAUCAUUGCACUGGUGUCCGCCGCCUUGUUUGCCGGUGCGGCAGCUU
env-192    CCUGCACCCA...CAUC.......UGGGUUCUUCUCA..GCCGCGGCGUUGACCCAACUUGUUUGGGGAGGCGCUGGCUGUUUUUU.......................................................................................
env-194    CCUGCACCCA...CAUC.......UGGGUUCUUCUCA..GCCGCGGCGUUGACCCAACUUGUUUGGGGAGGCGCUGGCUGUUUUUUUCGCGUCUCCCGGCUCAUGCCGAGGAACCCUGAUGUCGCUUUUCUAUAUCAUUGCACUGGUGUCCGCCGCCUUGUUUGCCGGUGCGGCAGCUU
env-195    CCUGCACCCA...CAUC.......UGGGUUCUUCUCA..GCCGCGGCGUUGACCCAACUUGUUUGGGGAGGCGCUGGCUGUUUUUUUCGCGUCUCCCGGCUCAUGCCGAGGAACCCUGAUGUCGCUUUUCUAUAUCAUUGCACUGGUGUCCGCCGCCUUGUUUGCCGGUGCGGCAGCUU
env-196    CCUGCACCCA...CAUC.......UGGGUUCUUCUCA..GCCGCGGCGUUGACCCAACUUGUUUGGGGAGGCGCUGGCUGUUUUUU.......................................................................................
Ssp-1-1    CCUGCGCCCC...CAAGC......GGGGUUCUUCCCA..GCCGCGGCGUCACCCGAAACCCGGGGGCGCGGUCAUAUGGGUUGGCGCCUCCUCGAACAAGACGAGGAACGGUUAUGAUAAUUUCACUUGUCGCUUUGCUCAGCGCAACGCUUUUCACUGGCGCGGCGAUCUACAUCAGCU
Nsp-1-1    CCUGCGCCCG...CAUGU......CGGACUCUUCCCU..GCCACGGCGUCACCUGAUCGAAAGGGUAGGCGCUUGACCAAUGGGCCAACGCUCCCUCCAAACUCAAGGAGCGUUUCAUGCAGUAUCUCUACCUUGCGAUUGCCAUCAUUGGUGAAGUGUUCGCGCAUCGUUACUCCGCC
env-197    CCUGCGCCCG...CAUGU......CGGGUUCUUCCCU..GCCACGGCGUCACCUGAUCGAGAGGGUAGGCGCUUGACCAUCGGGCCGACGCUCCCUCCGAGCCUAAGGAGCGUUUCAUGCAGUAUCUCUAUCUUGCGAUUGCCAUCAUUGGUGAAGUGUUCGCGCAUCGUUCCUCCGCC
Ssp-15-1   CCUGCGCCCGU..UUA.......GCGGGUUCUUCUCC..GCCACGGCGUCGACCAGCCUUUUGCGGCGGGGCGGCAACUGCAAUUCGCGCCACCCCCCGCCGCUACCGACGACGGAGCAUGGCCAUGCAAUAUAUCUACCUCGCCAUCGCCAUCGUUGCAGAGGUCGUUGCGACCAGUU
Ssp-18-1   CCUGCGCCCGU..UUA.......GCGGGUUCUUCUCC..GCCACGGCGUCGACCAGCCUUUUGCGGCGGGUGGCGCGGAAUUGCAGAUAUAUUGCAUGGCGAUGCCCCGCCGCUACCGACGACGGAGCAUCGCCAUGCAAUAUAUCUACCUCGCCAUCGCCAUCGUUGCAGAGGUCGUUG
env-198    CCUGCGCCCGCU.CGAU......AGCGGGUUCUUCCCA..GCCACGGCGUCAACCGAACGUUCGGGAGGCGCGUGGGCAUUGGUUUCCUCAUCCUCUCGGACGACAUCAAGAGGACACCACCAUGCCAGUUGCCCUAUUCGCUCUCAUCGCCGCGACGCUGUUCGCUGGUGCUGCGCUCUA
Ssp-16-1   CCUGCGCCCGU..UUA.......GCGGGUUCUUCUCC..GCCACGGCGUCGACCAGCCUUUUGCGGCGGGUGGCGCGGAAUUGCAGUUGCCGCCCCCGCCGCUACCGACGACGGAGCAUCGCCAUGCAAUAUAUCUACCUCGCCAUCGCCAUCGUUGCAGAGGUCGUUGCGACCAGUUUCA
env-199    CCUGCAUCCGUC.GCAUA....GACGGGUUCUUCCCA..GCCACGGCGUUACCCGGAACAGUUCAGGGAGGCGCGGCAGUUGGCUUCCAUUGCUCGCUUCAUGGAACGAAGACCAAGGGGGCCGACUAGUGGUAUUUUCUUUAGCUGCACUCGUUUUCGCCACGCUGUUUGCUGGCGCGG
env-200    CCUGCGCCCGU..CUA.......ACGGGUUCUUCCCA..GCCACGGCGUUGACCCAAUACGUUGGGAAGACGCUGGCUAUGGGGUUUGCGUCUUCCCGGCUGAUGCCGAGGAAUGCUCAUGAAUGUCAUUGCCAUCAUCGCCCUGACCUGUGCGGCUCUGUUCACGGGCGCGGCGGAUA
Sdo-1-1    CCUGCGCCCGU..CCA.......ACGGGUUCUUCCCA..GCCACGGCGUUGACCCAAUACGUUGGGAAGGCGCUGGCUAUGGGGUUCGCGUCUUCCCGGCUGAUGCCGAGGAAUGCUCAUGAAUAUUAUUGCCAUUGUCGCCCUGACCUGUGCGGCUCUGUUCACGGGCGCGGCGGAUA
env-201    CCUGCGCCCGU..CUA.......ACGGGUUCUUCCCA..GCCGCGGCGUUGACCCAAUACGUUGGGAAGACGCUGGCUAUGGGGUUUGCGUCUUCCCGGCUGAUGCCGAGGAAUGCUCAUGAAUGUCAUUGCCAUCAUCGCCCUGACCUGUGCGGCUCUGUUCACGGGCGCGGCGGAUA
Bro-1-1    CCUGCGCCCGUC.CUUG.....GACGGGCUCUUCUCA..GCCUCCGCGUCAACCCGACCAAUAUCGGGAAGGCGCAUCAGGCGUGGUCGCGUCUCCUGACAGCAAACCAGAGGAGAAUUCAGGUGGUCCCUAGCCUUGUUGCCCUUGUCAUUGCGGUCCUCUUUGCCGGGGCCGCGCUCU
Bro-1-2    CCUGCGCCCGUC.CUUG.....GACGGGCUCUUCUCA..GCCUCGGCGUCAACCCGACCAAUAUCGGGAAGGCGCAUCAGGCGUGGUCGCGUCUCCUGACAGCAAACCAGAGGAGAAUUCAGGUGGUCCCUAGCCUUGUUGCCCUUGUCAUUGCGGUCCUCUUUGCCGGGGCCGCGCUCU
env-202    CCUGCGCCCG...UUCAU......CGGGUUCUUCCCA..GCCACGGCGUCACCCAACUCGUUGGGAGGGCGCUGACAUGGGUUUCGCGUCUUCCCGGCUGAUGCCGAGGAACUGCAAUGUUUCAACUUUCGAUAGCGGCUCUCAUUU...................................
env-203    CCUGCGCCCGC..UUAG......GCGGGUUCUUCCCG..GCCACGGCGUUGACCUGAACAUGUUCGGGGAGGCGUUCGGGCACGUUUUCGAAAGUCUCCUCAAUUGCACUUUGGAGACGAUCAUGCACAAUAUUGCGAUCUUGGCCCUGAUUGCCACGACGCUCUUCGCAGGUGUGGCGC
env-204    CCUGCGCCCGU..UUCA......GCGGGUUCUUCUUG..GCCACGGCGUUGACCCGAACAUGUUCGGGGAGGCGUUCGGGUGAGUUUUCGAAAGUCUCCUCGCAUUCACCUUGGAGACUGCCAUGUAUAUUAUUCCCAUCAUUGCCCUGAUUGCUGCAACGAGUUUCGCCAGUGCUGCGC
env-205    CCUGCGCCCG...UUCAU......CGGGUUCUUCCCA..GCCACGGCGUCAACCCAACUCGUUGGGAAGGCGCUGCUGUGCG.........................................................................................
env-206    CCUGCGCCCG...UUCAU......CGGGUUCUUCCCA..GCCACGGCGUCAACCCAACUCGUUGGGAAGGCGCUGCUGUGCG.........................................................................................
env-208    CCUGCGCCCG...UUCAU......CGGGUUCUUCCCA..GCCACGGCGUCAACCCAACUCGUUGGGAAGGCGCUGCUGUGCG.........................................................................................
env-209    CCUGCGCCCG...UUCAU......CGGGUUCUUCCCA..GCCACGGCGUCAACCCAACUCGUUGGGAAGGCGCUGCUGUGCG.........................................................................................
env-210    CCUGCGCGCG...UUCAU......CGGGUUCUUCCCA..GCCACGGCGUCAACCCAACUCGUUGGGAAGGCGCUGCUGUGUGUUUCGCGCCUUCCCGGCUGAUGCCGAGGAAUGUUCAUGUUUCAGCUCUCGAUAGCUGCCCUUCUUUUCGCCGCGCUGUUUGCUGGCGCUGCACUCUAU
env-211    CCUGCGCCCG...UUCAC......CGGGUUCUUCCCA..GCCCCGGCGUCAACCCAACCUGUUUGGGGAGGCGCUGCCUUGGGUUUCGCGCCUUCCCGGCUGAUGCCGAGGAUUUUCGAUGAUCCAACUCUCGAUCGCUGCGCUCGUUUUUGCCACGCUGUUUGCCGGGCCGCCUUGUA
env-212    CCUGCGCCCGU..UCA.......ACGGGUUCUUCCCA..GCCACGGCGUCAACCCAACUCGUUGGGAAGGCGCUGACUUGGGGUUCGGGCCUUCCCGGCUGAUGCCGAGAAAUUUCUAUGUUUCAGGUCGCGAUAUCUGCUCUGUUUUUGCCAUGCUUUUCGCUGGCGCGGCACUUUA
env-213    CCUGCGCCC....UUUCAUC.....GGGUUCUUCCCA..GCCACGGCGUCAACCCAACUCGUUUGGGAAGGCGCUGACUUGGGUUUCGCGCCUUCCCGGCUGAUGCCGAGGAAUUUCUAUGUUUCAGAUUGCGAUAGCUGCUCUUGUUUUUGCCACGCUCUUCGCUGGCGCUGCACUUUA
env-214    CCUGCGCCCG...UUCAU......CGGGUUCUUCCCA..GCCACGGCGUCGAUCCAACUCGUUUGGGAAGGCGCUGACUUGGGUUUCGCGCCUUCCCGGCUAAUGCCGAGGAUUUUCGAUGUUUGAACUCUCGAUAGCUGCUCUCGUUUUUGCAACGCUGUUCGCAUCCGCUGCAAUGUA
env-223    CCUGCGCCCG...UUCAU......CGGGUUCUUCCCA..GCCACGGCGUCGAUCCAACUCGUUUGGGAAGGCGCUGACUUGGGUUUCGCGCCUUCCCGGCUAAUGCCGAGGAUUUUCGAUGUUUGAACUCUCGAUAGCUGCUCUCGUUUUUGCAACGCUGUUCGCAUCCGCUGCAAUGUA
env-224    CCUGCGCCCG...UUCAU......CGGGUUCUUCCCA..GCCACGGCGUCGAUCCAACUCGUUUGGGAAGGCGCUGACUUGGGUUUCGCGCCUUCCCGGCUAAUGCCGAGGAUUUUCGAUGUUUGAACUCUCGAUAGCUGCUCUCGUUUUUGCAACGCUGUUCGCAUCCGCUGCAAUGUA
env-225    CCUGCGCCCG...UUCAU......CGGGUUCUUCCCA..GCCACGGCGUCAACCCAACUGGUUUGGGAAGGCGCUGACAUGGGUUUUGCGCUUUCCCGGCUGAUGCCGAGGACCUUCGAUGCUUCAACUCUCGAUAGCUGCUCUCGUUUUCGCCACGCUGUUCGCCGGCGCCGCGCUCUA
env-226    CCUGCGCCCG...UUGAU......CGGGUUCUUCCCA..GCCACGGCGUCAACCCAACUGGUUUGGGAAGGCGCUGACAUGGGGUUUGCGCUUUCCCGGCUGAUGCCGAGGACCUUCGAUGCUUCAACUCUCGAUAGCUGCUCUCGUCUUCGCCACGUUGUUCGCCGGCGCCGCGCUCUA
Ssp-8-2    CCUGCGCCCG...UUCAU......CGGGUUCUUCCCA..GCCGCGGCGUCAACCCAACGUGUUUGGGAAGGCGCUGACAUGGGUAUCGCGCCUUCCCGGCCAAUGCCGAGGAAUUUCGAUGUUUCAACUCUCGAUAGCUGCACUCGUUUUCGCUACGCUGUUCGCAGGCGCGGCACUCUA
env-227    CCUGCGCCCG...UUCAU......CGGGUUCUUCCCA..GCCGCGGCGUCCACCCAACUCGUUUGGGAGGGCGCUGACAUGGGUCUCGCGUCCUCCCGGCUGAUGCCGAGGAACAUUUAUGCUUCAACUCUCGAUAGCGGCUCUCGUUUUCGCCACGCUGUUCGCCGGUGCCGCAUUCUA
env-228    CCUGCGCCCG...UUCAU......CGGGUUCUUCCCA..GCCGCGGCGUCCACCCAACUCGUUUGGGAGGGCGCUGACAUGGGUCUCGCGUCCUCCCGGCUGAUGCCGAGGAACAUUUAUGCUUCAACUCUCGAUAGCGGCUCUCGUUUUUGCCACGCUGUUCGCCGGUGCCGCACUCUA
env-229    CCUGCGCCCG...UUCAU......CGGGUUCUUCCCA..GCCGCGGCGUCCACCCAACUCGUUUGGGAGGGCGCUGACAUGGGUCUCGCGUCCUCCCGGCUGAUGCCGAGGAACAUUUAUGCUUCAACUCUCGAUAGCGGCUCUCGUUUUUGCCACGCUGUUCGCCGGUGCCGCACUCUA
env-235    CCUGCGCCCG...UUCAU......CGGGUUCUUCCCA..GCCGCGGCGUCCACCCAACUCGUUUGGGAGGGCGCUGACAUGGGUCUCGCGUCCUCCCGGCUGAUGCCGAGGAACAUUUAUGCUUCAACUCUCGAUAGCGGCUCUCGUUUUUGCCACGCUGUUCGCCGGUGCCGCACUCUA
env-236    CCUGCGCCCG...UUCAU......CGGGUUCUUCCCA..GCCGCGGCGUCCACCCAACUCGUUUGGGAGGGCGCUGACAUGGGUCUCGCGUCCUCCCGGCUGAUGCCGAGGAACAUUUAUGCUUCAACUCUCGAUAGCGGCUCUCGUUUUUGCCACGCUGUUCGCCGGUGCCGCACUCUA
env-237    CCUGCGCCCG...UUCAU......CGGGUUCUUCCCA..GCCGCGGCGUCCACCCAACUCGUUUGGGAGGGCGCUGACAUGGGUCUCGCGUCCUCCCGGCUGAUGCCGAGGAACAUUUAUGCUUCAACUCUCGAUAGCGGCUCUCGUUUUUGCCACGCUGUUCGCCGGUGCCGCACUCUA
env-238    CCUGCGCCCG...UUCAU......CGGGUUCUUCCCA..GCCGCGGCGUCCACCCAACUCGUUUGGGAGGGCGCUGACAUGGGUCUCGCGUCCUCCCGGCUGAUGCCGAGGAACAUUUAUGCUUCAACUCUCGAUAGCGGCUCUCGUUUUUGCCACGCUGUUCGCCGGUGCCGCACUCUA
env-239    CCUGCGCCCG...UUCAU......CGGGUUCUUCCCA..GCCGCGGCGUCCACCCAACUCGUUUGGGAGGGCGCUGACAUGGGUCUCGCGUCCUCCCGGCUGAUGCCGAGGAACAUUUAUGCUUCAACUCUCGAUAGCGGCUCUCGUUUUCGCCACGCUGUUCGCCGGUGCCGCAUUCUA
env-240    CCUGCGCCCG...UUCAU......CGGGUUCUUCCCA..GCCGCGGCGUCCACCCAACUCGUUUGGGAGGGCGCUGACAUGGGUCUCGCGUCCUCCCGGCUGAUGCCGAGGAACAUUUAUGCUUCAACUCUCGAUAGCGGCUCUCGUUUUCGCCACGCUGUUCGCCGGUGCCGCAUUCUA
env-241    CCUGCGCCCG...UUCAU......CGGGUUCUUCCCA..GCCGCGGCGUCCACCCAACUCGUUUGGGAGGGCGCUGACAUGGGUCUCGCGUCCUCCCGGCUGAUGCCGAGGAACAUUUAUGCUUCAACUCUCGAUAGCGGCUCUCGUUUUCGCCACGCUGUUCGCCGGUGCCGCAUUCUA
env-242    CCUGCGCCCG...UUCAU......CGGGUUCUUCCCA..GCCGCGGCGUCCACCCAACUCGUUUGGGAGGGCGCUGACAUGGGUCUCGCGUCCUCCCGGCUGAUGCCGAGGAACAUUUAUGCUUCAACUCUCGAUAGCGGCUCUCGUUUUCGCCACGCUGUUCGCCGGUGCCGCAUUCUA
env-243    CCUGCGCCCG...UUCAU......CGGGUUCUUCCCA..GCCGCGGCGUCCACCCAACUCGUUUGGGAGGGCGCUGACAUGGGUCUCGCGUCCUCCCGGCUGAUGCCGAGGAACAUUUAUGCUUCAACUCUCGAUAGCGGCUCUCGUUUUCGCCACGCUGUUCGCCGGUGCCGCAUUCUA
env-244    CCUGCGCCCG...UUCAU......CGGGUUCUUCUCA..GCCACGGCGUCGACCCAACUUGUUUGGGAAGGCGCUGACAUGGGUUUCGCGUCUUCCCGCUGAUGCCGAGGAAGUUCGAUGUUUCAUCUCUCGAUAGCCGCUCUCGUUUUUGCCACGCUGUUCUCCGGCGCCGCGCUCUA
Swi-1-1    CCUGCGCCCG...UUCGC......CGGGUUCUUCCCA..GCCACGGCGUCAACCCAACGUGUUUGGGAAGGCGCUGACAUGGGUUUCGCGCUUUCCCGGCCGAUGCUGAGGAUUUUCGAUGUUUCAACUCUCGAUCGCCGCUCUCGUUUUUGCCACGCUGUUCACGGGCGCUGCACUCUA
Ssp-5-1    CCUGCGCCCG...CUCAU......CGGGUUCUUCCCA..GCCGCGGCGUCAACCCAACGCGUUUGGGAAGGCGCUGACACAUGAGGUUCGCGUCUUCCCGGCUGAUGCUGAGGAAUGAUCAUGGUUCAACUUGCGACCGCUGCUCUCGUUCUUGCCACGCUCUUCGCGGGCGCUGCACUC
env-245    CCUGCGCCCG...CCCUCGGA...CGGGUUCUUCUCA..GCCUCGGCGUCUCAACCCGAUCGGUAUCGGGAGAGGCGCAUCAGGCGUGAUCGCGUCUCCCGUAGCGCACCAGAGGAAAAUUCAAUUGGUCCCUGGCCUCGUUGCUCUUGCUGUUGCGACCCUCUUUGCUGGGGCCGCGCU
env-246    CCUGCGCCCGCG.CCC.......CGCGGGUUCUUCCAA..GCCACGGCGUCGAACCGAUCAAUCGUUCGGGGAGACGCUGCAUGUGGUUUCCAGCCUCGGCGCGCCUCCUCGAUCUUUGCAAUGAGGAGCAUCUCGAUGGCAUUCCCCCUGGCUGCCCUGGUCUUCGCGACGCUGUUUGCG
env-247    CCUGCGCCCGU..CCUUGA....ACGGGUUCUUCUCA..GCCUCGGCGUCAACCCGACCAACCAUCGGGAAGGCGCAUCAGGCGUGGUCGCGUCUUCCGACAGUGAACCAGAGGAGAAUUCAGGUGGUCCCUGGCCUUGUUGCCCUCGUCAUCGCUACCCUUUUUGCCGGAGCCGCACUU
env-248    CCUGCGCCCGU..CCCA......GUGGGUUCUUUCAAACGCCCGCGGCGUCAACCNCCCGAAAAAUGG..........................................................................................................
env-249    CCUGCGCCCGU..CCCA......GUGGGUUCUUCAAC..GCCGCGGCGU.............................................................................................................................
env-250    CCUGCGCCCGU..CCCA......GUGGGUUCUUCAAC..GCCGCGGCGUCAACCACCCGAAAAUGGUGGGGUGG..................................................................................................
env-251    CCUGCGCCCGU..CCCA......GUGGGUUCUUCAAC..GCCGCGGCGUCAACCACCCGAAAAUGGUGGGGUGGCGCUGGUCAACGACUGGA..............................................................................
env-252    CCUGCGCCCGU..CCCA......GUGGGUUCUUCAAC..GCCGCGGC...............................................................................................................................
env-253    CCUGCGCCCGU..CCCA......GUGGGUUCUUCAAC..GCCG...................................................................................................................................
env-254    CCUGCGCCCGU..CCCA......GUGGGUUCUUCAAC..GCCGCGG................................................................................................................................
env-255    CCUGCGCCCUU..CGCG......AUGGGUUCUUCUCC..GCCUCGGCCAGCCCAGCGCCCUGCUGGUCGCCGAUUGCAGGAGGCCCCCAUGCCCUAUGUCUACCUGGCCAUCGCCAUCGUCGUGGAAGUCAUCGCGACCUCCUUCCUGAAGCGCUCCGACGGCUUCUCGAAACUGGUGC
env-256    CCUGCACCCGUC.CUCC......GACGGGCUCUUCUCA..GCCUCGGCGUCUCAACCUGAUCGGUAUCGGGAGAGGCGCAUCAGGCGUGGUCGCGCCUCCCGUAGCGCACCAGAGGAGAAUUCAGUUGGUCCCUGGCCUCAUUGCUCUCGUCGUUGCGACCCUCUUUGCUGGGGCCGCACU
           >>>>><<<<<<<<.........>>>>>>>>......................................................................................................................................................
           ..20..1222221.........1222221.......................................................................................................................................................
           CCUGCGCCCR○--●●●○------○YGGGUUCUUCYC●--GCC●CGGCGYY●R●C●●●●●●●●●●●●●R●●R●●●●●●●●●●●●●●●●●●●●●●●●●●●●●●●●●●●●●●●●●●●●●●●●●●●○○○○○○○○○○○○○○○○○○○○○○○○○○○○○○○○○○○○○○○○○○○○○○○○○○○○○○○○○○○○○○○○
```

alignment positions 361 · · · 540

```
env-1      UGUUCUGGAGCGCUGGACUGGCUGGCGUCCUAGACUUUCCUGAGAAGGUCGCCUCGAUGCGAGGCGUUGGUCUUGCGCCGCCUGAGCUUUACGCCGUCGCUGUGACCGCGGUCAACUGGGCGGGUCAGCACUUAUCGUCUUCAACCGGCAGGCAUGGUUGGGCGCCGGGGCCUUGGCUG
env-2      CCGAUUUUCUCACGCAGGACGAAGGCCGUGGGCGCCAAGGGCGCGCCAUCCUCAUAACGGUUGCGGUGCUGGCGCUCGUGCUGGUGGCUGUUCCUGUGGCGCAGCUUGCGCGCUGGACCCACGCAAGGCUGGCAGCAGCAGGCGGUGCCGGUAGCGGCGAUGCGCCUCACACCACGCG
env-4      CGGCAUGACCGAGGCUUCCCCGGAAUUUCUCGCGCAGGAAGACGGCCGCGGGCGCCAGUGGCGCGCCAUCCUCAUAACGGUUGCGGUGCUGGCGCUGGUGCUGGGCGGUCUGUUCCUCUGGCGGACCCUGCGGGCCGGGCCGGGGCAGGCCUGGCAGCAACGGGCGGUGCCGGUGGCGGC
Ral-1-1    GUCGUUGGCACGCUGACAAACGGAAUUGGCCCUAAGAAAAUUCGCGAAAGUUACGCAAAAUGGGGAUACCCCUCUUGGUUUAGAUAUGUCACCUCAGCCUUGGAAUUUGCUGUCGCAGUUCUGCUUAUCUUUCCUGAAACCCGCCUCUUUGGUGCAGGCCUUGGAAUGAUGAUUAUGCUG
env-5      GUUCUGGAGCGCCGGAUUGGCU...........................................................................................................................................................

           ..........................................................................................................................................................................................
           ..........................................................................................................................................................................................
           ○○○○○○○○○○○○○○○○○○○○○○○○○○○○○○○○○○○○○○○○○○○○○○○○○○○○○○○○○○○○○○○○○○○○○○○○○○○○○○○○○○○○○○○○○○○○○○○○○○○○○○○○○○○○○○○○○○○○○○○○○○○○○○○○○○○○○○○○○○○○○○○○○○○○○○○○○○○○○○○○○○○○○○○○○○○○
                                                                                                                                                                                    3′
```

```
env-6     UUGUCGGUGCCAUCAUUAAUUCAUUGGCUCCAGGGAAGAUACGUGAGGACUAUCAUCGAUGGGGUUACCCCGCAUGGUUCCGGUUUAUCACAGCAUUGUUAGAACUGAUUGCGGCUAUUCUGAUUGUUAGCUACCGCUAUAAACUGCCGGGAUUAGCCUUAGCCGUUCUGGUGAUGGGUG
Spa-1-1   AAGCGGUGGCAGCCCUUUCCGUUUCCGGCCUGAUUUCGGUCCUCGUCGCGGGUUUCACGGACGAAUUUGCCCGAAUCUGGGUGAUCACUUUCCUGCUGGGCUGGCCGCUCGGCUCCCUCUUUGAUCGGGCGGUCAGCCGUCUUGAAAGGGGGAUGGCAUGAGCGCCGCCGAUCAGGAACU
env-7     AUGGCAUGAGCGACGCUGCCCGGGACAUGCCGGCGCCGGAGAAGGGCUCCGGCCGCCAGCUUCGCACGAUCCUCAUUACGGUUGUCGUGCUGAUCGCCGUGCUCGGCGGGCUUUUCCUAUGGCGGACAUUGCGUUCGGGCCGGCAGCAGAGCUGGCCGCAGCAGGCGACGGCAGUGGCCG
env-8     UGACCGAUGCCGUUCGGGAUAGGCCGGCACCGGCAGAGGG.........
env-9     .........
env-10    .........
env-11    .........
env-12    AGGGCAUGAGCGACGCUGUCCGGGAUGCGCCGGCGGAAGGCUCGGGGCGUCAGCUUCGCACGAUCCUCAUCACGGUCUUGGUGCUGGCCGCUGUGCUCGGCGGGCUGUUCCUGUGGCGGAUGCUGCGCUCGGGCCAGCCGCAGAACUGGCAACAGCAGGCGACGGCAGUGGCCGCGACGG
env-13    CAUGACGGAUCCGUCAGCGGCCGCACCGGAGCGCGGCACCGUGCCCGCGCAUCAGCUUCGCACGAUUGUCGGCACGGUCAUUGUCCUCGCCAUGAUAUUGGGCGGUCUGUUCCUGUGGCGGAUGGCGCGGUCGGCCCGGCGCAGAACUGGCAGGCGCAGGCCUUGCCGGUCGCGGCAAC
env-14    CAUGACGGAUCUGCCCCAGACCGCACCGGAGCGGAGCACCGCGUUCCCGUCGCAGCUUCGCGCGAUCGUCGGUACGAUCAUCGUCCUGGCCGUGAUAUUGGGCGGCCUGUUCCUCUGGCGGAUGGCGCGUUCCGGUCCGGCGCAGAACUGGCAGGCGCAGGCGCUGCCGGUCGCGGCAGC
env-15    .........GGGCAAGCCAUGCCGGGA
env-16    .........
env-17    AACGCCGAUCAUCGCUGAUCCCGUGCGCCCUGCGUCCUACCUCUCCUCUUUUUUCGUAACGGACAUUCGAUGAGCAUCGAUCCUUUCAGCCUUGCCGUUCUCCUGUGGCCCCGCCUUGUCGGUGCGAAGCGCACGGAAGCCCUGGCAUGACCGACCACGCCUCCAUGACCGACCAUGACC
env-18    GUUUUCUGGAGCGCAGGUCUCGCUGGCAUCUUCGAUUUUUCCGGGAAAGUCAUUGAGAUGGAAAUGGUCGGUCUUGAACCGGCAGCACUGUUCGCGUUGGCGGUUACAAUUGUCCAGAUCGGCGGCUCGACGCUCAUAAUCAUAGACCGUUUCGCAUGGCUCGGUGCUGGAGCGCUAGGG
env-20    GUUUUCUGGAGCGCAGGUCUCGCUGGCAUCUUCGAUUUUUCCGGGAAAGUCAUUGAGAUGGAAAUGGUCGGUCUUGAACCGGCAGCACUGUUCGCGUUGGCGGUUACAAUUGUCCAGAUCGGCGGCUCGACGCUCAUAAUCAUAGACCGUUUCGCAUGGCUCGGUGCUGGAGCGCUAGGG
env-21    GUUUUCUGGAGCGCAGGUCUCGCUGGCAUCUUCGAUUUUUCCGGGAAAGUCAUUGAGAUGGAAAUGGUCGGUCUUGAACCGGCAGCACUGUUCGCGUUGGCGGUUACAAUUGUCCAGAUCGGCGGCUCGACGCUCAUAAUCAUAGACCGUUUCGCAUGGCUCGGUGCUGGAGCGCUAGGA
env-22    GUUUUCUGGAGCGCAGGUCUCGCUGGCAUCUUCGAUUUUUCCGGGAAAGUCAUUGAGAUGGAAAUGGUCGGUCUUGAACCGGCAGCACUGUUCGCGUUGGCGGUUACAAUUGUCCAGAUCGGCGGCUCGACGCUCAUAAUCAUAGACCGUUUCGCAUGGCUCGGUGCUGGAGCGCUAGGA
env-23    GUUUUCUGGAGCGCAGGUCUCGCUGGCAUCUUCGAUUUUUCCGGGAAAGUCAUUGAGAUGGAAAUGGUCGGUCUUGAACCGGCAGCACUGUUCGCGUUGGCGGUUACAAUUGUCCAGAUCGGCGGCUCGACGCUCAUAAUCAUAGACCGUUUCGCAUGGCUCGGUGCUGGAGCGCUAGGG
env-24    GUUUUCUGGAGCGCAGGUCUCGCUGGCAUCUUCGAUUUUUCCGGGAAAAUCAUUGAGAUGGAAAUGGUCGGUCUUGAACCGGCAGCACUGUUCGCGUUGGCGGUUACAAUUGUCCAGAUCGGCGGCUCGACGCUCAUAAUCAUAGACCGUUUCGCAUGGCUCGGUGCUGGAGCGCUAGGG
env-25    .........
env-26    GUUUUCUGGAGCGCAGGUCUCGCUGGCAUCUUCGAUUUUUCCGGGAAAAUCAUUGAGAUGGAAAUGGUCGGUCUUGAACCGGCAGCACUGUUCGCGUUGGCGGUUACAAUUGUCCAGAUCGGCGGCUCGACGCUCAUAAUCAUAGACCGUUUCGCAUGGCUCGGUGCUGGAGCGCUAGGG
Cpa-1-1   GGUCUCACCCUGGGCCGUGACGACCUGCUUCAACUGCUGGCGAGUAUCGGCACCGACCCUACUUUCGAAAACUGCGUGUUCGAUGGCGAAAAUCUGUCUGGCCUCGAUUUUCGCAACGCUAGAUUCUCUCGCUGCUCAUUCGUGGCAGUUGAUUUCGAACGGGCGAAUCUUGCGAACACG
Ysp-1-1   GCCCUUGGCGGCACUCAACUGCGCCACGCCAUGCAAAAGGCCGCCCCGGGGGCGGCCUGUCGGUCGGCUCUCCACGACGGCCCUUGGGGGGCAGGAUCAGGGCAUGAUGAGAAUCGGCGUCGGCUUGUCGGUGCGGAUCAUCGCGUAGAUGUCCUCCAUCUCGCGGUCGGUCACCGCGAU
env-31    GAUGGAUGCCCGGCAUAUCCCGGCCGCGCAGCGUGGCGGAGCCUUGCGUCAUGUCCGCCCGAUCAUUCUCACCAUUGUCAUUCUGGCCCUUGUGCUGGGCGGGCUGUUCUGGUGGCGGACGCUGCGCCUGGCGCCGG.........
env-32    GACGGAUGCCCGGCAUAUCCCGGCCGGACGGCGUGGCGGGUCCGCGCGCCAUGUCCGCCCGAUCAUUCUCACCAUUGUCAUUCUGGCCGUCGUGCUGGGCGGGCUGUUCUGGUGGCGGGCGCUGCGCCUGGCGCCGGCCCAGAGCUGGUCGCGGGAGGCGCUGCCCGUGGCCGCCAU...
env-33    UGGCUCGUAUUGGUCGUCGUCUCGACCGCUCU.........
env-34    UGGCUCGUAUUGGUCGUCGUCUCGACCGCUCU.........
env-35    UUCUUCAUCAUCGGAGCAGUGGUAAACUGGAUCGCUCCGCCCAAGAUUGCGAACGACUAUGCACGGUGGGGCUAUCCUCCGCGCUUUCAUUAUCUGACUGCAGUUCUGGAGGGGCUUUCGGCCAUUCUCCUGCUUUUACCGACUACGCGGCUCUUUGGUGCAACACUGGGCGCUCUGGUC
Bsp-1-1   GCUUUGCUGACGAUCUUCGUAUUUUGGAUCUCAACAGCUGUCACUGAACUAUUCGCCUCUUCCUCGACCAUCGCAGAACUCAAGAUGGUGAUCCCGUGGGGGUUCCUUUGGUUGGUCCCUGCUUUGGCGAUCACAGGCGGAUCGGGCCUUCAAAUGGCCAAAGGCCGCCGGUCACGCAUC
env-36    AUCCUUUCAGCCUUGCCAUCCUGUCGUGGACCAAAGCCUUUCGCAUGGAGCAGGCCGCAUGACUGACGCCGUCACCCCAACGCCGCGCCCCCGCCAGACGCGCGCGAUCAUCCUGACGGUGAUCGUACUGCUGCUCCUGCUGGGUGGGCUGUAUCUGUGGCGCGGCUGGCGUGAAGGCCG
Ssp-11-1  GCUUUUCCAGGCUGGUGCCGAGCAUCAUCACGGCGAUUGCCUAUGCCAUCGCCUUUUAUUUCCUCUCGCAGACGCUGAAGACCAUUCCGACCGGCAUCGCUUAUGCGAUCUGGUCAGGCGCCGGCAUCGUCCUCAUCACCGCGAUCGCCUGGGUGUUUCAGGGCCAGAAGCUGGAUGUCG
Ssp-7-1   GCUUUUCCCGACUGGUGCCGAGCAUCAUCACGGCGAUUGCCUAUGCCAUCGCCUUUUAUUUCCUCUCGCAGACGCUGAAGACCAUUCCGACCGGCAUCGCUUAUGCGAUCUGGUCAGGCGCAGGCAUCGUCCUCAUCACCGCAAUCGCCUGGGUGUUUCAGGGCCAGAAGCUGGAUGUCG
Nsp-2-1   CGGAAGGGUUUACGAAGCCCGUCCCAAGCGUGAUUACGGCGAUUGCUUACGUGAUUGCAUUCUAUUUUUUAUCGCAAACCCUUAAGACGAUCCCGACGGGUAUAGCCUAUGCCAUCUGGUCCGGUUCAGGCAUUGUGCUCAUUGCUGCGAUUGCCUGGAUCUUUCAGGGGCAAAAAUUGG
Rsu-1-1   UCUUGCGGGAGCGAUCCUCUGCGAAGUCGCGGGCUCCAGUUUCCUUCUGAAAUCGGAGCAGUUCACCAGGCUUGGCCCGACUGCGGUCAUGGCCUUGUUCUACAUCGCCUCUUUCUAUCUCUUGUCGAUCGCUCUCAAGACGAUUCCUCUCGGCAUUGCCUAUGCGGUCUGGGGGGACU
env-37    CUUCACCAAACUUGUCCCCUCUCUCGUGAUGAUCGUGGGAUAUGCGACGGCCUUCUAUUUCCUGUCGCUGACGCUCCGCACCAUCCCUACGGGCGUGGCUUAUGCGAUCUGGUCUGGCGCAGGCGUUGUUCUGAUUGCAGCUAUCGCCUGGAUUUUCCAAGGGCAGAAGCUCGACGUACC
Sya-3-1   CUUUGACGGAGUCCUGCGCGACUUCGCGGCAGCCAUCUCGCCUAUAUUCCUCUUAUCCAACUGGAUUUUCGAUGAGCAUCGAUCCUUUCGGCCUUGCGGUUCUGCUCUGGCCCCACUUCGUUCGCCUGAAGCGCGGGGAGGUGAAAGCAUGAGCGAUCAUAAUCUGCCUGAACCAUCGCG
env-40    CUUUGACGGAGUCCUGCGCGACUUCGCGGCAGCCAUCUCGCCUAUAUUCCUCUUAUCCAACUGGAUUUUCGAUGAGCAUCGAUCCUUUCGGCCUUGCGGUUCUGCUCUGGCCCCACUUCGUUCGCCGGAAGCGCGGGGAGGUGAAAGCAUGAGCGAUCAUAAUCUGCCUGAACCAUCGCG
Sya-1-1   CUUUGACGGAGUCCUGCGCGACUUCGCGGCAGCCAUCUCGCCUAUAUUCCUCUUAUCCAACUGGAUUUUCGAUGAGCAUCGAUCCUUUCGGCCUUGCGGUUCUCCUCUGGCCCCACUUUGUUCGCCUGAAGCGGGGGGAGGUGAAAGCAUGAGCGAUCAUAAUCUGUCCGAACCAUCGCG
Sya-2-1   CUUUGACGAAGUCCUGCGCGACUUCGCGGCAGCCAUCUCGCCUAUAUUCCUCUUAUCCAACUGGAUUUUCGAUGAGCAUCGAUCCUUUCGGCCUUGCGGUUCUCCUCUGGCCCCACUUCGUUCGCCCGAAGCGGGGGAGGUGAAAGCAUGAGCGAUCAUAAUCUGCCCGAACCAUCGCG
Ssp-3-1   CUUUGACGAAGUCCUGCGUGACUUCGCGGCAGCCAUCUCGCCUAUAUUCCUCUUAUCCAACUGGAUUUUCGAUGAGCAUCGAUCCUUUCGGCCUUGCGGUUCUCCUCUGGCCCCACUUCGUUCGCCCGAAGCGGGGGAGGUGAAAGCAUGAGCGAUCAUAAUCUGUCCAAACCAUCGCG
env-42    CUUUGACGAAGUCCUGCGCGACUUCGCGGCAGCCAUCUCGCCUAUAUUCCUCUUAUCCAACUGGAUUUUCGAUGAGCAUCGAUCCUUUCGGCCUUGCGGUUCUCCUCUGGCCCCACUUC.........
env-43    AUGGCUUGUGUUGGUCGUCGUUUCGACCGCUCUUUUUCUCAUCAGCAUUGACAUGACUGUGCUGUAUACCGCUUUGCCGCGGUUGACCCACGAUCUUGCCGCCGACAGCGGCCAAAAACUUUGGAUCGUCGACACAUUUCCGCUUGUCAUGGCGGGUCUGCUUCCAACUAUGGGCAUGCU
env-44    AUGGCUCGUGCUGGUCGUUGUCUCUACCGCUCUUUUUCUCAUCAGCAUUGAUAUGACAGUGCUGUACACCGCUUUGCCACGGCUGACACACGAUCUCGCCGCCAGCAGCAGCCAAAAGCUCUGGAUCGUCAACGCGUUUCCACUGGUGAUGGCUGGUUUGCUCCCAACUAUGGGUUUGCU
env-45    AUGGCUCGUGCUGGUCGUUGUCUCUACCGCUCUUUUUCUCAUCAGCAUUGAUAUGACAGUGCUGUACACCGCUUUGCCACGACUGACACACGAUCUCGCCGCCAGCAGCAGCCAAAAGCUCUGGAUCGUCAAUGCGUUUCCACUUGUGAUGGCUGGUUUGCUCCCAACUAUGGGUUUGCU
env-46    AUGGCUCCUGCUGGUCGUUGUCUCUACCGCUCUUUUUCUCAUCAGCAUUGAUAUGACAGUGCUGUACACCGCUUUGCCACGACUGACACACGAUCUCGCCGCCAGCAGCAGCCAAAAGCUCUGGAUCGUCAAUGCGUUUCCACUU.........
env-47    AUGGCUCGUGCUGGUCGUCGUCUCGACCGCGCUUUUUCUCAUCAGCAUUGAUAUGA.........
env-48    AUGGCUCGUGCUGGUCGUCGUCUCGACCGCUCUCUUUCUCAUCAGCAUUGAUAUGACAGUGCUGUACACCGCUUUGCCACGGCUGACC.........
env-49    C.........
env-50    .........
env-51    ACGGGGCUUUUCCUCAUUC.........
env-52    ACGGGGCUUUU.........
env-53    ACGGGGCUUUUCCUCAUUCGAGCAGGCAUUACGAUUGCCUCGCUCACAAGCAGAGGGGUGAACUGCACCCCGAGACGCGCGAUAGACUUAGCAGCCGUUGCAGUGCGCGUGAAGAGCGUUGGUCGCCAACUUGCCGCAAGCAUUCUCUUUCUAGCGGUGAUGGCUCUGGCAGACGUUGCC
env-54    ACGGGGCUUUUCCUCAUUCAAGCAGGCAUAACGAUUGCCUCGCUCACAAGCAGAGGGGUGAACUGCACUCCGAGACGCGCGCUAGACUUAGCAGCCGUAACAGUGCGCGUGAAGAGCGUUGGUCGCCAACUUGCCGCAAGCAUUCUCUUUCUAGCGGUGAUGGCUCUGGCGGGCGUUGCC
env-55    ACGGGGCUUUUCCUCAUUCAAGCAGGCAUUACGAUUGCCUCGCUCACAAGCAGAGGGGUGAACUGCACUCCGAGACGCGCGCUAGACUUAGCAGCCAUUGCAGUGCGCGUGAAGAGCGUUGGUCGCCAACUUGCCGCGAGCGUUCUCUUUCUAGCGGUGAUGGCUCUGACGGACGUUGCC
env-56    ACGGGGCUUUUCCUCAUUCAAGCAGGCAUUACGAUUGCCUCGCUCACAAGCAGAGGGGUGAACUGCACUCCGAGACGCGCGCUAGACUUAGCAGCCAUUGCAGUGCGCGUGAAGAGCGUUGGUCGCCAACUUGCCGCAAGCAUUCUCUUUCUAGCGGUG.........
env-57    ACGGGGCUGUUCCUCAUUCAAGCAGGCAUAACGAUCGCCUCGCUCACAAGCAGAGGGGC.........
env-58    ACGGGGCUUUUCCU.........
env-59    GGUCUUUUCCUCAUUGAGGCAGGCGUAACGAUUGCAUCGCUCGCAAGCAUAUGUGUGAAGCGCGUACUGAGAUGUGCGCGGGACUUGGCGGUCCUUGCACGACGAGUGAAAAGCACCGGGCGUCGACCCGUCGCAACCAUUACGUUUCUAGUGGUGAUGGGUCCGCGGGGUUUCGCUAGC
env-60    .........
env-61    AAUGGCUCGUGCUGGUCGUCGUUUCGACCGCUCUCUUUCUCAUCAGCAUUGAUAUGACUGUGCUGUAUACCGCUUUGCCGCGGCUGACCCACGAUCUUGCCGCCAACAGCAGCCAAAAACUUUGGAUCGUCGACACAUUUCCGCUUGUCAUGGCGGGCCUGCUUCCAACUAUGGGUAUGC
env-62    .........
env-63    .........
env-64    UGGUUUCACGAAGCCGUUGCCCACCGCCAUCAUGGUCGCCUUUUAUGUGGUGACCUUCUACUUCCUGUCGCUGGCGCUGCGGACCAUGCCCACCGGCGUUGCCUAUGCGAUCUGGUCGGGCGUGGGCGUGGUCCUUAUCACCGCGAUCGCCUGGAUAUAUCAGGGCCAGAAACUGGACGC
Ssp-12-1  CUUGUCGAACACCCUGCCCGGCUCGUCCUGGACGAUGGGCCGCUGCUUCAACAAUGGCAGCCAAGCUACAGGCGAGCGUUACCCAUUCAGUCCGCCCUGGCGGUGAUCAGCGGCGUGGCCGGGCUGGCGGUCGCCUAUGCUUCGGGUGACUGGCGCUGGGCUGCGGGUGGCGCGGCCCUU
env-65    AAUGGUGGCGACGGCAUUAUAACACGGCGCGACCACACUCGUCCCUGGUCUACAAACCACCCU.........
env-66    CUACGGCUUCGCUCCGAAUCGGUUGGUACGGUGGAUUAGUGGAUUGAUAUCCGCCUGCGCGGGCGUCACGCUACUUAUGCCCGGCACUCGGCUAAUCGGUGCCACGAUCGGCGUCAUGAUCAUGGGUACCGCGCUGACUACCUCACUUCUGGAUCAGAGGUUUACACCAGCUUGCUGCUG
Ssp-10-1  CUACGGCUUCGCCCCGAAUUGGUUGGUACGGUGGAUUAGUGGAUUGAUAUGCGCCUGCGCGGGCGUCACGCUACUUAUGCCCGGCACUCGGCUAAUCGGUGCCACGAUCGGCGUCGUGAUUAUGGGCACCGCGCUGACUACCUCACUUCUGGAUCAGAGGUUUACAUCAGCGUGCUGCUG
Bst-1-1   GACGACAUUCAGCCGGGAUGAACUGGAGCGUGUCCUCCGGCAAUCCGGUGACGGGCCGACCUUCGAGCGUUGCAAUUUCGACGGCGAAGACCUUUCCAGCCUCGACUUCCGCGCGGCGCGAUUCGCGCAGUGCAAUUUCGAGCAGACCCGCUUCGAGCACAGCGAUCUUUCGCGGAGCCG
env-67    .........
She-1-1   ACAGUCCGAGGGCUUUACCAGGCUCGUGCCUAGCCUCGUUACGGCUUUGGCCUAUGCAAUCGCAUUCUUCUUCCUCUCACAAACUCUCAAAACUAUCCCCACCGGCGUCGCCUACGCCAUUUGGUCAGGGGCAGGCAUCGUUCUGAUCGCUACCGCCGCAGCAGUUUUCCAGGGUCAAAA
Ssp-13-1  GCAGUCCGAAGGCUUUACGAAGCUGGUGCCGAGCCUGAUCACGAUCACGGCCUAUGUCGCCGCGUUCUACUGUCUGUCGUUGACGUUGAAAACCAUUCCGACAGGCGUCGCCUAUGCGAUCUGGUCAGGGGUCGGGAUCGUUCUGAUCUCCGCAGUUGCCUGGAUUUUCCAGGGGCAGAA
env-68    .........
env-69    CGAGUUUCAAUCGCGACGACCUGACUCGCGCGCUCCGCGAGUCCGGCGACGAGCCGACUUUCGAGCGCUGCCAGUUCGACGGCGAAGAUCUUUCCAAUAUCGAUUUCC.........
Ptr-1-1   CGCAUUUCGAUCGCGACGACCUCGCUCGCGCGCUCCGUGAGUCCGGCGACGAGCCGACUUUCGAGCGCUGCCAGUUCGACGGCGAAGAUCUUUCCAAUAUCGAUUUCCACGCGGCGCGAUUCGCGCAAUGCACGAUCGGGCAAACCCGCUUCGAGCACAGCGAUUUAUCGCGCACCCGCU
Ptr-1-3   CGCAUUUCGAUCGCGACGACCUCGCUCGCGCGCUCCGCGAGUCCGGCGACGAGCCGACUUUCGAGCGCUGCCAGUUCGACGGCGAAGAUCUUUCCAAUAUCGAUUUCCACGCGGCGCGAUUCGCGCAAUGCACGAUCGGGCAAACCCGCUUCGAGCACAGCGAUUUAUCGCGCACCCGCU
Ssp-22-1  GACGGCUUUACCAAAUUGACGCCUUCGCUCGUCACGGUCGCAGGCUAUGCGACCUCCUUCUUUUUCCUGUCGCUAGCGCUCAGGGAUUUGCCGACCGGCAUCGCCUAUGCGAUCUGGUCGGGCGUCGGCAUCGUACUGAUCGCCGCGAUCGCCUGGCUGUUUCAGGGGCAAAGGCUCGAC
Bsp-2-1   CGUCCUUGAGCCGGGAAGAGGUAGCAGUGCUACUUCGAGACAGCGGCGCGGCGCCGACAUUCGAACACUGCAUGUUCGACGGCGAAGACUAUUCCGAUCUCGAUUUCCGGGGAGCCCGGUUCGCUCAUUGCUCGUUCGCAAAUUCUAUUCUUGAGAACUGCAGGCUGGCGGGUAGUCGCU
env-70    GCUUUACUUCAACGUCGUCGAACAUCCCGCUCGCUCCGGAUUGGGCGAUGCGGCCAUGUUGGCGCAAUGGCAGACGAGCUACGCGCGGGCCCUGCCGAUUCAGGGAGGCUUAGCGAUCGUCGGAGGGGGCGCUGGCCUGCUUGUGGUGGUAUGAAACGGGCGGCUGGCAGUGGCUGGCAGC
Kvu-2-1   CGCUGGCGGCGUUCUUUGUGCUGGGCGGCGUGGUGAAUUGGAUCGGCACCCCAUCCGCGCGGGCCGAUUACGCACGCUGGGGCUAUCCCAGGUGGUUCCAUUAUGUGACAGCGCUAUGCGAACUGACAGCCGCAGCCCUGCUGGUGUCGCCCUUUACCCAGAUCUGGGGCGCGGCGCUGG
env-71    GGGCUUCACUCGACUGGUGCCCAGUCUGGUCACGGUCAUUGCCUAUGCCGCUGCCUUUUACUGCCUCUCGCUCACCCUGCGGACGAUCCCGACGGGGAUCGCCUAUGCGAUCUGGUCCGGUGUGGGGAUCGUGCUGAUCGCUGCAAUUGCGUGGAUCUUCCAGGGGCAGAAGCUCGAUAU
env-72    GGGCUUCACUCGACUGGUGCCCAGUCUGGUCACGGUCAUUGCCUAUGCCGCUGCCUUUUACUGCCUCUCGCUCACCCUGCGGACGAUCCCGACGGGGAUCGCCUAUGCGAUCUGGUCCGGUGUGGGGAUCGUGCUGAUCGCUGCAAUUGCGUGGAUCUUCCAGGGGCAGAAGCUCGAUAU
env-73    GGGAUUCACUCGACUGGCGCCCAGCCUCGUCACUGUCAUCGCCUAUGCCGCUGCCUUCUAUUGCCUCUCGCUCACCCUGCGAACGAUCCCGACGGGUAUCGCCUAUGCGAUCUGGUCCGGUGUGGGGAUCGUACUGAUCACCACGAUCGCCUGGGUCUUUCAGGGCCAGAAGCUCGAUGC
env-74    GGGAUUCACUCGACUGGCGCCCAGCCUCGUCACUGUCAUCGCCUAUGCCGCUGCCUUCUAUUGCCUCUCGCUCACCCUGCGAACGAUCCCGACGGGUAUCGCCUAUGCGAUCUGGUCCGGUGUGGGGAUCGUACUGAUCACCACGAUCGCCUGGGUCUUUCAGGGCCAGAAGCUCGAUGC

          .........
          .........
          ooooooooo
          3'
```

```
env-75   ..........................................................................................................................................................................................................UGGGGGUUAUGGCAGCUG
Psp-1-1  UUGGCACCUUCACCAAUGGCUAUGGCCCGCAAACGAUCCGCGAGGAUACGCCCGCUGGGGCUAUCCAAGCUGGUUUCGCUAUGUGACCGCACUGCUGGAAUUGGCGGUGGCGUUGCUGCUUUUGUUCCCAGCGGGCAUCGGCUGGGGCGCGAUCCUGGGUAUGGGGGUUAUGGCAGCUG
Psa-1-1  CCUUGCCGGAGCCAUCAUCUUCGAGGUUGCAGGCUCCAGCUUCCUUCAGAAAUCCCAACAAUUCACGAAAGCUGGCCCGACUGCGGCCAUGGCGCUGUUCUACAUCUGUCCUUUUACCUUCUGUCAAUCGCCCUCAAGUCACUGCCCCUCGGGAUUGCCUACGCGAUCUGGGGUGGGCU
env-76   ACAUCAAUGUCGCCGAGCAACCCGCUCGGUUGGGUCUAGACGACCAAGCAAUGCUGACCGAGUGGAAGCCGGCCUACAAGCGCGGCACCAGAAUGCAGGCCCCGCUAGCGGUGCUCGGUUGUCUCUUUGGCAUGCUGGCUGGUGGCAAACCGAUAAUCUCAUUUGGAUCGCAAGCGCCG
env-77   ..........................................................................................................................................................................................................................
env-78   ACAUCAAUGUCGCCGAGCAACCCGCUCGGUUGGGUCUAGACGACCAAGCAAUGCUGACCGAGUGGAAGCCGGCCUACAAGCGCGGCACCAGAAUGCAGGCCCCGCUAGCGGUGCUCGGUUGUCUCUUUGGCAUGCUGGCUGGUGGCAAACCGAUAAUCUCAUUUGGAUCGCAAGCGCCG
Ssp-8-1  UCGCGGAGGUUUCCGCCAGCUCGUUCGUCAACAAAUCCGAAUAUUUGACCAAGGCGGCGCCCAGCAUCAUCGCUUGUCUUCUCUACCUGCUCGCACUGGUGCUCUUCAGCCAGGCGCUUCGGUCGAUUCCCCUGGGGUUGGGCUACGCCAUCUGGUCGGGACUCGGCAUCACUCUCGUCU
Osp-1-1  CCGGUGGAGCAAUCAUCUGGAGGUCAUCGGAUCCGGCUCCCUGCAGAAGAGCGAGCAGGUCACCAGGCUGGUGCCGACGGCGCUCACAGUGGCGCUCUACGCCCUGUCCUUCUGCCUGCUGCCCCAUGUGCUGAAGGUGCUGCCGCUUGGCAUCGCCCAGGCGAUCUGGGCGGGCUGG
env-79   GCAGUCCCAGGGCUUCUCCAAGCUCGUCCCCAGCCUGAUCACGGCCGUGGCCUACGCAGUUGCGUUCUUCUGCCUUUCGCAGACGCUCAAAACGAUCCCGACCGGCAUCGCCUAUGCGAUCUGGUCCGGGGUGGGGAUCGUCUUGAUCGCCACCGUCGCCGCAGUGUUCCAGGGGCAGAA
env-80   GCAGUCCCAGGGCUUCUCCAAGCUCGCCCCAGCCUGAUCACGGCCGUGGCCUACGCAGUUGCGUUCUUCUGCCUUUCGCAGACGCUCAAAACGAUCCCGACCGGCAUCGCCUAUGCGAUCUGGUCCGGGGUGGGGAUCGUCUUGAUCGCCACCGUCGCCGCAGUGUUCCAGGGGCAGAA
Rma-1-1  CCCUCGCCGGAGCCAUCAUCUCCGAAGUGACGGGUACGGCGUAUCUUCAAAAGUCCGCGCAGUUCACCAAGCUUGCCCCACGGCUAUCAUGGCCGUGUUCUACAUCCUGUCGUUCUAUUUGCUGUCGGUCGCACUGCGGACGCUCCCGCUAGGUGUGGCCUAUGCAGUUUGGGGUGGCU
Spa-1-2  GCUGGUCGAGCAUCCCGCGCGGCUUGGCCUGGGCGACGGGCCGAUGCUUCAACAAUGGCAGCCAAGCUAUAAGCGCGCGCUACCCAUUCAGUCUGGCUUGGCCGUGAUUGGCGGCGUGGCAGGCCUGAUCGCAGGCUAUCUCACGAAAGACUGGCUUUGGGUGGCAGGGAGUGUCGCCCU
Squ-1-1  GCUGGUCGAGCAUCCCGCGCGGCUUGGCCUGGGCGACGGGCCGAUGCUUCAACAAUGGCAGCCAAGCUAUAAGCGCGCGCUACCCAUUCAGUCUGGCUUGGCCGUGAUUGGCGGCCUGGCAGGCCUGAUCGCAGGCUAUCUCACGAAAGACUGGCUUUGGGUGGCAGGGAGUGUCGCCCU
env-81   GCGGUUCAACCUUCCUGCAGAAAUCAGCGCAAUUCACCGGCCUUGGCCGACCGCGGCCAGGCCCUCUGCUACGU.....................................................................................................................................
Sph-1-1  AUCAGUCUUGUCGAGCAUCCCGCGCGUUUACAGUUGGACGAUGCCCCCCUUCUUGCGCAAUGGCAGCCGAGCUAUAGCCGGGCCUUGCCGAUCCAAUCCGGGCUGGCGAUCGCCGGCGGCGUGGCGGGACUGGCCGCCUGGCAUAUGUCGGGCGAAUGGCUUUAUGUUCUGGGAAGCCUG
Pno-1-1  GAUCGACCUUCGACCGCGAAGCGCUGACCCGCCUGCUUCGGGAAUGCGGCGACGAACCGACGUUCGAGCAUUGCAGUUUCGACGGCGAAGAUCUCUCCGGUCUCGAUCUCGGCGCCGCCCGAUUCACACAGUGCACCUUCGAGCGCACCCUGCUCGAGCACUGCGAACUCGCAAGAAGCC
Bce-1-1  ACACGAACUUCAGCCGCGACGAACUGGCUCGCGUUCUUCAGCAAUCCGGCGACGGGCCGACCUUCGAGCGCUGCGACUUCGACGGCCAGGACCUUUCCGACCUUGACUUCCGAGCGGCACGAUUCGCGCAGUGCAACUUCGAGCGCACCCGCUUCGAGCACAGCGAUCUGACGGGCAGCC
env-82   GCAAGAUGGAUCACCGCGAACUUCUGAAUGACUGGUUCUGCCAUCAGGUUCUGCCGCUGGAACGCUCGCUCACCCGUUUCAUUCAAAGGAACUGGCGCGUGGAGGCGGAGGUGAGCGAUCUGCGCCAGGAAAUCUAUGAGCGGGUCUGGUCGGCGCGCGGGAUGCGCUGCCGCAGAACG
env-85   ..........................................................................................................................................................................................................................
env-86   CCCUCGCAGGCGCGAUUAUCUGUGAGGUCACAGGCUCCAGUUUCCUUCAGAAAUCCGCCCAGUUCACCAAGCUUGGUCCGACGGCGGUCAUGGCGAUAUUCUACAUCGCCUCCUUCUAUCUGCUGUCCAUCGCGCUCAGAACGCUUCCGCUCGGUAUCGCCUAUGCGGUCUGGGGAGGGC
env-87   AGCAGAGCGAAGGCUCCACGAGACUUGUUCCCAGCCUCCUUACCGCCCUCUCAUAUAUCGUGGCUUUCUACAGCCUGUCGCUGACCUUGAAGACCAUUCCCACCGGGAUAGCUUACGCAAUCUGGUCGGGCUGCGGCAUCGUGCUGAUCGCGGCCGUUGCCUGGAUCAUUCAGGGGCAGA
env-89   AAACAAAGCGAAGGCUUUACGAAGCUGGUCCCCAGCUUGCUUACUGCACUUGCAUAUGUAGUCGCGUUUUAUUGC.....................................................................................................................................
env-90   AAACAAAGCGAAGGCUUUACGAAGCUGGUCCCCAGCUUGCUUACUGCACUUGCAUAUGUAGUCGCGUUUUAUUGCCUAUCGCUAACUUUG.......................................................................................................................
env-91   ..........................................................................................................................................................................................................................
env-92   AAACAAAGCGAAGGCUUUACGAAGCUGGUCCCCAGCUUGCUUACUGCACUUGCAUAUGUAGUCGCGUUUUAUUGCCUAUCGCUAACUUUG.........................................................................................................AAAACUAUCCCAACUGGC
env-95   ..........................................................................................................................................................................................................................
env-96   ..........................................................................................................................................................................................................................
env-97   GACGGAGGGGUUUACAAGGCUUGGCCCGACCGUGAUCGCCUUGAUGGGCUAUCUGGUCACGUUCUAUUUUCUUGCGCUGGCAUUGCGGACCAUCCCGACUGGUGUUGCUUACGCGAUCUGGGCAGGCUUAGGCGUAGUGCUGGUAGCUGGUGUUGCAUGGGUGAUCCAAGGCCAGAAGCU
env-98   GACGGAGGGGUUUACAAGGCUUGGCCCGACCGUGAUCGCCUUGAUGGGCUAUCUGGUCACGUUCUAUUUUCUUGCGCUGGCAUUGCGGACCAUCCCGACUGGUGUUGCUUACGCGAUCUGGGCAGGCUUAGGCGUAGUGCUGGUAGCUGGUGUUGCAUGGGUGAUCCAAGGCCAGAAGCU
env-99   GACGGAGGGG................................................................................................................................................................................................................
env-100  GACGGAGGGGUUUACAAGGCUUGGCCCGACCGUGAUCGCCUUGAUGGGCUAUCUGGUCACGUUCUAUUUUCUUGCGCUGGCAUUGCGGACCAUCCCGACUGGUGUUGCUUACGCGAUCUGGGCAGGCUUAGGCGUAGUGCUGGUAGCUGGUGUUGCAUGGGUGAUCCAAGGCCAGAAGCU
env-101  GACGGAGGGGUUUACAAGGCUUGGCCCGACCGUGAUCGCCUUGAUGGGCUAUCUGGUCACGUUCUAUUUUCUUGCGCUGGCAUUGCGGACCAUCCCGACUGGUGUUGCUUACGCGAUCUGGGCAGGCUUAGGCGUAGUGCUGGUAGCUGGUGUUGCAUGGGUGA................
env-102  GACGGAGGGGUUUACAAGGCUUGGCCCGACCGUGAUCGCCUUGAUGGGCUAUCUGGUCACGUUCUAUUUUCUUGCGCUGGCAUUGCGGACCAUCCCGACUGGUGUUGCUUACGCGAUCUGGGCAGGCUUAGGCGUAGUGCUGGUAGCUGGUGUUGCAUGGGUGAUCCAAGGCCAGAAGCU
env-103  GACGGAGGGGUUUACAAGGCUUG...................................................................................................................................................................................................
env-104  GACGGAGGGGUUUACAAGGCUUGGCCCGACCGUGAUCGCCUUGAUGGGCUAUCUGGUCACGUUCUAUUUUCUUGCGCUGGCAUUGCGGACCAUCCCGACUGGUGUUGCUUACGCGAUCUGGGCAGGCUUAGGCGUAGUGCUGGUAGCUGGUGUUGCAUGGGUGAUCCAAGGCCAGAAGCU
env-118  GACGGAGGGGUUUACAAGGCUUGGCCCGACCGUGAUCGCCUUGAUGGGCUAUCUGGUCACGUUCUAUUUUCUUGCGCUGGCAUUGCGGACCAUCCCGACUGGUGUUGCUUACGCGAUCUGGGCAGGCUUAGGCGUAGUGCUGGUAGCUGGUGUUGCAUGGGUGAUCCAAGGCCAGAAGCU
env-119  GACGGAGGGGUUUACAAGGCUUGGCCCGACCGUGAUCGCCUUGAUGGGCUAUCUGGUCACGUUCUAUUUUCUUGCGCUGGCAUUGCGGACCAUCCCGACUGGUGUUGCUUA.........................................................................................................
env-120  GACGGAGGGGUUUACAAGGCUUGGCCCGACCGUGAUCGCCUUGAUGGGCUAUCUGGUCACGUUCUAUUUUCUUGCGCUGGCAUUGCGGACCAUCCCGACUGGUGUUGCUUACGCGAUCUGGGCAGGCUUAGGCGUAGUGCUGGUAGCUGGUGUUGCAUGGGUGAUCCAAGGCCAGAAGCU
env-121  GACGGAGGGGUUUACAAGGCUUGGCCCGACCGUGAUCGCCUUGAUGGGCUAUCUGGUCACGUUCUAUUUUCUUGCGCUGGCAUUGCGGACCAUCCCGACUGGUGUUGCUUACGCGAUCUGGGCAGGCUUAGGCGUAGUGCUGGUAGCUGGUGUUGCAUGGGUGAUCCAAGGCCAGAAGCU
Ssp-9-1  UUGGUUGAGCAUCCCACCCGGCUCGGUUUGGACGAUGGCCCGCUGCUUCAGCAAUGGCAGCCGAGCUACAAGCGCGCACUGCCCAUCCAGUCCGGCCUGGCCGUGAUUGGCGGCGUGGCGGGGCUGCUGGCCGGCUACCUCACGAAAGAUUGGCGCUGGGUGGCAGGGAGUGUCGCUCUA
Ssp-2-1  CUGGUCGAGCAUCCCGCGCGGCUUGGCCUGGGCGACGGGCCGAUGCUUCAACAAUGGCAGCCAAGCUAUAAGCGCGCACUACCCAUCCAGUCUGGCUUGGCCGUGAUUGGCGGAGUGGCAGGCCUGAUCGCAGGCUAUCUCACGAAAGACUGGCUCUGGGUGGCAGGGAGUGUCGCCCUU
env-122  UCGGACGGCUUUUCGAAGCUGCUUCCCAGCGCCGUCACGGCCCUGGCCUAUAUCGUCGCAUUCUAUUGCCUGUCGCUGACGCUCAAGACCAUUCCCACCGGCAUUGCCUAUGCGAUCUGGUCGGGUUCGGGCAUCGUCCUGAUUGCGGCGGCGGCCUGGAUAUUUCAGGGCCAGAAGCUC
Npa-1-1  UCGGACGGCUUUUCGAAGCUGCUUCCCAGCGCCGUCACGGCCCUGGCCUAUAUCGUCGCAUUCUAUUGCCUGUCGCUGACGCUCAAGACCAUUCCCACCGGCAUUGCCUAUGCGAUCUGGUCGGGUUCGGGCAUCGUCCUGAUUGCGGCGGCGGCCUGGAUAUUUCAGGGCCAGAAGCUC
Pba-1-1  AUCGACCUUCGAUCGCGACGAUCUGGCGCUGCUGCUUCGCGAUGGCGGCGACACGCCGACCUUCGAGCAAUGUGAUUUCAGCGGUGAAGACCUUUCGCAUCUCGACUUCGGGGCGCGCGAUUCACGCGCUGCAAUUUCGGCAAGCCGUCUGGAGCACAGCGAACUCGCCAGGACGCG
env-124  CUUCGUCGUGGGCGCCGCCGGGAACUUCAUGGCGCCGCCCGACAUCGCGGCCGACUAUGCCCGGUGGGGGUAUCCCCUCCUUCUUUCACUACGUCACCGCCGCCUUGGAGAUCACGACCGCCGGUCUGCUGAUAUUCCGGCGAACGCGCGUCACGGGCGCGCUGCUGGGCUGCGUCGUCAU
env-125  CUUCGUCGUGGGCGCCGCCGGGAACUUCAUGGCGCCGCCCGACAUCGCGGCCGACUAUGCCCGGUGGGGGUAUCCCCUCCUUCUUUCACUACGUCACCGCCGCCUUGGAGAUCACGACCGCCGGUCUGCUGAUAUUCCGGCGAACGCGCGUCACGGGCGCGCUGCUGGGCUGCGUCGUCAU
env-126  AAGCAGAGCGAAGGUUUCACCAAACUAGCGCCCAGCCUUCUCACUGCCCUCUCGUACAUCGUGGCUUUCUACUGCCUGUCGCUGACAUUGAAGACCAUUCCCACCGGGAUAGCUUACGCAAUUUGGUCGGGCUGCGGCAUCGUAUUGAUCGCAGCCGUUGCCUGGAUCAUUCAGGGGCAA
env-127  CCCCGGAUCCCAAAAGGAAGGCCCCCCCUUUUGCGGGGAUGAACAAGAAAGGUCCCCUGCUGGGAAUAAGGUUUUGCUCUCCUUGGGUUUGCCCAAGGAAGGAGACCAAAUCCCGCGGAAACCAAUGAAAAUAUCCAACGGUUCUCCAAAUUUUUAGGAUUCCUUUAACUGGGCGGCCCU
env-128  ..........................................................................................................................................................................................................................
Ssp-3-2  CCCCUGAUUCCGAGAUGAUGUCGCCCCCUUUGCGGGGAUGACGAUGAAGGUCCGCUG.................................................................................................................................................................
env-129  CCCCUGAUUCCGAGAUGAUGUCGCCCCCUUUGCGGGGAUGACGAUGAAGGUCCGCUGCUGGGAUUAGGUUUGCUUCCUUUGGGUUGUCCGAGGGAGGGAGCCGAUGUCGGCGGAGCGAUUGAGUAUGCGACGGUUCGCGAGAUUUUACGGUACCGGUUUGAGCAGGGGCUGGGUC....
Ssp-6-1  CCCCUGAUUCCGAGAUGAUGUCGCCCCCUUUGCGGGGAUGACGAUGAAGGUCCGCUGCUGGGAUUAGGUUUGCUUCCUUUGGGUUGUCCGAGGGAGGGAGCCGAUGUCGGCGGAGCGAUUGAGUAUGCGACGGUUCGCGAGAUUUUACGGUACCGGUUUGAGCAGGGGCUGGGUCACAA
Peb-1-1  CAGGAAACGGCGGCCGCGGAGGGAGGGCUGUUCAGCGAAUUGACCUUCGACCGCGAAGGUCUCGCCCGCCUGCUUCGAGAAUGUGGUGACGCACCGACGUUCGAACACUGCGAUUUCAGCGGUGAAGAUCUAUCCAGGCUAGACUUCCGUGGCGCCCGAUUCACACGGUGCAGCUUCGAG
env-130  GUCGGACGGCUUUUCGAAGCUGUUUCCCAGCGCCGUCACGGCCCUGGCCUAUAUCGUCGCAUUCUAUUGCCUGUCGCUGACGCUCAAGACUAUUCCCACCGGCAUUGCCUAUGCGAUCUGGUCGGGUUCGGGCAUCGUCCUGAUUGCGGUGGUGGCCUGGAUAUUUCAGGGCCAGAAGCU
env-131  GUCGGACGGCUUUUCGAAGCUGCUUCCCAGCGCCGUCACGGCCCUGGCCUAUAUCGUCGCAUUCUAUUGCCUGUCGCUGACGCUCAAGACCAUUCCCACCGGCAUUGCAUAUGCGAUCUGGUCGGGUUCGGGCAUCGUCCUGAUUGCGGCGGCGGCCUGGAUAUUUCAGGGCCAGAAGCU
env-132  GUCGGACGGCUUUUCGAAGCUGCUUCCCAGCGCCGUCACGGCCCUGGCCUAUAUCGUCGCAUUCUAUUGCCUGUCGCUGACGCUCAAGACCAUUCCCACCGGCAUUGCCUAUGCGAUCUGGUCGGGUUCGGGCAUCGUCCUGAUUGCGGUGGUGGCCUGGAUAUUUCAGGGCCAGAAGCU
Sxe-1-1  CAGCCUGGUCGAACAUCCGGCUCGGCUGGCGCUGAGCGACAGCGCCUCCUCACGCAGUGGCAACCCAGCUACAAGCGAGCACUUCCCUUCCAGUCCGGGCUGGCCGUCGCAGGCGGCCUGUCGGGGGUAAUCUGUUUCUAUCUGACGGCGGAUUUCAGGUGGCUUGCGGGGAGCAUCGC
Ssp-23-1 GAGACAAUCGGAAGGCUUCACGAAGCUGGUUCCGUCGCUGAUUACCGCCGGCGGCUAUAUCGCGCCUUCUACUUCCUGUCGCUGACCCUCAGAACCUUGCCCACGGGAAUCGCCUACGCGAUCUGGUCCGGCGUCGGGAUCGUCCUGAUCGCGGCGAUCGCGUGGUGUUUCAGGGCCA
Esp-2-1  AGUCGGAAGGUUUCACGAAGCUGGUGCCGGGUCUAAUCACCGGUGCCGCCUAUGUGGUCGCUUUUUACUUUCUAUCACUGACACUGAAAACGAUCCCGACGGGUGUUGCUUAUGCAAUCUGGUCAGGCGUCGGCAUAGUUCUGAUCGCAGCCGUGGCCUGGGUUUUGCAAGGACAGAAAC
Esp-1-1  AGUCGGAAGGUUUCACGAAGCUGGUGCCGGGUCUAAUCACCGGUGCCGCCUAUGUGGUCGCUUUUUACUUUCUAUCACUGACACUGAAAACGAUCCCGACGGGUGUUGCUUAUGCAAUCUGGUCAGGCGUCGGCAUAGUUCUGAUCGCAGCCGUGGCCUGGGUUUUGCAAGGACAGAAAC
env-133  AGUCGGAUGGUUUCACCAAGCUGGUGCCGG............................................................................................................................................................................................
Efl-1-1  AAUCGGACGGUUUCACCAAGCUGGUGCCGGGUCUGAUCACUGCUGCCGCCUAUGUGGUCGCUUUUUACUUUCUAUCACUGACACUGAAAACGAUUCCGACAGGAAUUGCUUAUGCAAUUUGGUCAGGCGUCGGCAUAAUUCUGAUCGCAGCCAUAGCCUGGGUUUUCCAAGGACAGAAAC
env-134  AAUCGGAAGGUUUCACCAAGCUGGUGCCCGGUCUGAUCACUGUUGCCGCCUAUGUGGUGGCUUUUUACUUUCUAUCACUGACACUGAAAACGAUUCCGACAGGUAUUGCUUAUGCAAUUUGGUCAGGUGUCGGCAUAGUUCUGAUCGCAGCCGUAGCAUGGCUUUUCCAGGGACAGAAAC
env-135  AUCGGAAGGUUUCACCAAGCUGGUGCCCGGUCUGAUCACUGCUGCCGCCUAUGUGGUGGCUUUUUACUUUCUAUCACUGACACUGAAAACGAUUCCGACAGGUAUUGCUUAUGCAAUUUGGUCAGGCGUCGGCAUAGUUCUGAUCGCAGCCGUAGCAUGGCUUUUCCAGGGACAGAAACU
env-136  AUCGGAAGGUUUCACCAAGCUGGUGCCCGGUCUGAUCACUGCUGCCGCCUAUGUGGUGGCUUUUUACUUUCUAUCACUGACACUGAAAACGAUUCCGACAGGUAUUGCUUAUGCAAUUUGGUCAGGCGUCGGCAUAGUUCUGAUCGCAGCCGUAGCAUGGCUUUUCCAGGGACAGAAACU
env-137  AUCGGAAGGUUUCACCAAGCUGGUGCCCGGUCUGAUCACUGCUGCCGCCUAUGUGGUGGCUUUUUACUUUCUAUCACUGACACUGAAAACGAUUCCGACAGGUAUUGCUUAUGCAAUUUGGUCAGGCGUCGGCAUAGUUCUGAUCGCAGCCGUAGCAUGGCUUUUCCAGGGACAGAAACU
env-138  AUCGGAAGGUUUCACCAAGCUGGUGCCCGGUCUGAUCACUGCUGCCGCCUAUGUGGUGGCUUUUUACUUUCUAUCACUGACACUGAAAACGAUUCCGACAGGUAUUGCUUAUGCAAUUUGGUCAGGCGUCGGCAUAGUUCUGAUCGCAGCCGUAGCAUGGCUUUUCCAGGGACAGAAACU
env-139  AUCGGAAGGUUUCACCAAGCUGGUGCCCGGUCUGAUCACUGCUGCCGCCUAUGUGGUGGCUUUUUACUUUCUAUCACUGACACUGAAAACGAUUCCGACAGGUAUUGCUUAUGCAAUUUGGUCAGGCGUCGGCAUAGUUCUGAUCGCAGCCGUAGCAUGGCUUUUCCAGGGACAGAAACU
env-155  UAUAUCAGUCUGGUCGAACACCCGGCCCGCCUGUUGCUGGAAGACAGGCCGCUACUCACACAGUGGCAAUCAAGCUACGCGAAAGCGCUCCCGAUACAGUCCGGCUUGGCUAUUAUCGGCGGUG..............................................................................
env-156  UAUAUCAGUCUGGUCGAACACCCGGCCCGCCUGUUGCUGGAAGACAGGCCGCUACUCACACAGUGGCAAUCAAGCUACGCGAAAGCGCUCCCGAUACAGUCCGGCUUGGCUAUUAUCGGCGGUG............................................................UAUGCGGAUUGGCGGCUU
env-157  UAUAUCAGUCUGGUCGAACACCCGGCCCGCCUGUUGCUGGAAGACAGGCCGCU.....................................................................................................................................................
env-158  UAUAUCAGUCUGGUCGAACACCCGGCCCGCCUGUUGCUGGAAGACAGGCCGCUACUCACACAGUGGCAAUCAAGCUACGCGAAAGCACUACCGAUACAGUCCGGCCUGGCUAUCAUCGGCGGUGUAUGCGGAGUGGCAGCUUGGUAUUUAUCGACCCAAUGGUUGUGGAUCGCUGGAAGC
env-160  ..........................................................................................................................................................................................................................
env-161  ...................................................................................................................................................................................................GAGGCGCUGGCUGUUUUU
env-162  AUAUCAGUCUGGUCGAACACCCGGCCCGCCUGUUGCUGGAAGACAGGCCGCUACUUACACAGUGGCAAUCAAGCUACGCGAAAGCGCUACCGAUACAGUCCGGCCUGGCUAUUAUCGGCGGUGUAUGCGGAGUGGCGGCUUGGUAUUUAUCGACCCAAUGGUUGUGGAUCGCUGGAAGCA
env-163  AUAUCAGUCUGGUCGAACACCCGGCCCGCCUGUUGCUGGAAGACAGGCCGCUACUUACACAGUGGCAAUCAAGCUACGCGAAAGCGCUACCGAUACAGUCCGGCCUGGCUAUUAUCGGCGGUGUAUGCGGAGUGGCGGCUUGGUAUUUAUCGACCCAAUGGUUGUGGAUCGCUGGAAGCA
env-165  AUAUCAGUCUGGUCGAACACCCGGCCCGCCUGUUGCUGGAAGACAGGCCGCUACUUACACAGUGGCAAUCAAGCUACGCGAAAGCGCUACCGAUACAGUCCGGCCUGGCUAUUAUCGGCGGUGUAUGCGGAGUGGCGGCUUGGUAUUUAUCGACCCAAUGGUUGUGGAUCGCUGGAAGCA
env-166  ..........................................................................................................................................................................................................................
env-168  AUAUCAGUCUGGUCGAACACCCGGCCCGCCUGUUGCUGGAAGACAGGCCGCUACUCACACAGUGGCAAUCAAGCUACGCGAAAGCGCUCCCGAUACAGUCCGGCUUGGCUAUUAUCGGCGGUGUAUGCGGAUUGGCGGCUUGGUAUUUAUCGACCCAAUGGUUGUGGAUCGCUGGAAGCA
env-169  UAUCAGUCUGGUCGAACACCCG....................................................................................................................................................................................................
env-170  UAUCAGUCUGGUCGAACACCCGGCCGCGCCUGUUGCUGGAAGACAGGCCGCUACUCACACAGUGGCAAUCAAGCUACGCGAAAGCGCUACCGAUACAGUCCGGCCUGGCUGUUAUCGGCGGUGUAUGCGGAGUGGCGGCUUGGUAUUUAUCGACCCAAUGGUUGUGGAUCGCUGGAAGCAU
env-171  UAUCAGUCUGGUCGAACACCCGGCCGCGCCUAUUGCUGGAAGACACGCCGCUACUCACACAGUGGCAAUCAAGCUACGCGAAAGCGCUCCCGAUACAGUCCGGCUUGGCUAUUAUCGGCGGUGUAUGCGGAGUGGCGGCUUGGUAUUUAUCGACCCAAUGGUUGUGGAUCGCUGGAAGCAU
env-172  AUAUCAGUCUGGUCGAACACCCGGCCCGCCUGUUGCUGGAAGACAGGCCGCUACUUACACAGUGGCAAUCAAGCUACGCGAAAGCGCUACCGAUACAGUCCGGCCUGGCUGUUAUCGGCGGUGUAUGCGGAUUGGCGGCUUGGUAUUUAUCGACCCAAUGGUUGUGGAUCGCUGGAAGCA

..........................................................................................................................................................................................................................
..........................................................................................................................................................................................................................
°°°°°°°°°°°°°°°°°°°°°°°°°°°°°°°°°°°°°°°°°°°°°°°°°°°°°°°°°°°°°°°°°°°°°°°°°°°°°°°°°°°°°°°°°°°°°°°°°°°°°°°°°°°°°°°°°°°°°°°°°°°°°°°°°°°°°°°°°°°°°°°°°°°°°°°°°°°°°°°°°°°°°°°°°°°°°°°°°°°°°°°°°°°°°°°°°°°°°°°°°°°°°°
                                                                                                                                                                                                    3'
```

env-173 UAUCAGUCUGGUCGAACACCCGGCCCGCCUGNUGCUGGAAGACAGGCCGCUNCUCACACANUGGCAAUCAAGCUACGCGAAAGCGCUNCCGAUACAGUCCGGCNUGGCUNUUAUCGGCGGUGUAUGCGGAUUGGCGGCUUGGUAUUUAUCGACCCAAUGGUUGUGGAUCGCUGGAAGCAU
env-174 AUAUCAGUCUGGUCGAACACCCGGCCCGCCUGUUGCUGGAAGACAGGCCGCUACUCACACAGUGGCAAUCAAG......
env-175 AUAUCAGUCUGGUCGAACACCCGGCCCGCCUGUUGCUGGAAGACAGGCCGCUACUCACACAGUGGCAAUCAAGCUACGCGAAAGCGCUCCCGAUACAGUCCGGCUUGGCUAUUAUCGGCGGUGUAUGCGGAUUGGCGGCUUGGUAUUUAUCGACCCAAUGGUUGUGGAUCGCUGGAAGCA
Esp-3-1 AUAUCAGUCUGGUCGAACACCCGGCGCGCCUGUUGCUGGAAGACAGGCCGCUACUCACACAGUGGCAAUCAAGCUACGCGAAAGCGCUACCGAUACAGUCCGGCCUGGCUAUUAUCGGCGGUGUAUGCGGAGUGGCGGCUUGGUAUUUAUCGACCCAAUGGUUGUGGAUCGCUGGAAGCA
env-176 UAUAUCAGUCUGGUCGAACACCCGGCCCGCUUGUUGCUGGAAGACAGGCCGCUACUCACACAGUGGCAAUCAAGCUACGCGAAAGCGCUCCCGAUACAGUCCGGCUUGGCUAUUAUCGGCGGUGUAUGCGGAUUGGCGGCUUGGUAUUUAUCGACCCAAUGGUUGUGGAUCGCUGGAAGC
env-179 UAUAUCAGUCUGGUCGAACACCCGGCCCGCUUGUUGCUGGAAGACAGGCCGCUACUCACACAGUGGCAAUCAAGCUACGCGAAAGCGCUCCCGAUACAGUCCGGCUUGGCUAUUAUCGGCGGUGUAUGCGGAUUGGCGGCUUGGUAUUUAUCGACCCAAUGGUUGUGGAUCGCUGGAAGC
env-180 UAUAUCAGUCUGGUCGAACACCCGGCCCGCUUG......
env-181 UAUAUCAGUCUGGUCGAACACCCGGCCCGCUUG......UUGCUGGAAGACAGGCCG
env-182 ......
env-183 ......UCGCGUCUCCCCGGCUCA
env-184 ......
env-185 ......UCGCGUCUCCCCGGCUCA
env-186 AUAUCAGUCUGGUCGAACACCCGGCCCGCCUGUUGCUGGAAGA......
env-187 AUAUCAGUCUGGUCGAACACCCGGCCCGCCUGUUGCUGGAAGA......CAGGCCGCUACUCACACA
env-192 ......
env-194 AUAUCAGUCUGGUCGAACACCCGGCCCGCCUGUUGCUGGAAGA......CAGGCCGCUACUCACACA
env-195 AUAUCAGUCUGGUCGAACACCCGGCCCGCCUGUUGCUGGAAGA......CAGGCCGCUACUCACACA
env-196 ......UCGCGUCUCCCCGGCUCA
Ssp-1-1 UGGUCGAGCAUCCCGCCCGGCUGGGCCUAGACGAUGGCCCGCUGCUCCAACAAUGGCAGCCGAGCUACAAGCGCGCACUACCCAUCCAGUCCGGCCUGGCUGUGAUUGGCGGAGUAGCCGGGCUGCUGGCCGCUUAUUUCACGAAAGAUUGGCGCUGGGUGGCAGGGAGCGUGGCUCUGC
Nsp-1-1 AGACAGAGGGGUUCACAAAGAUCGGCCCGACCAUGCUCGCCCUUGGGGGCUAUUUAGUCACGUUCUACUUCCUUGCGCUGGCAUUACGGACCAUCCCACCGGAGUUGCCUACGCGAUUUGGGCAGGCUUGGGCGUAGUGUUGGUCGCGGCUGUCGCGUGGCUGAUCCAAGGCCAGAAGC
env-197 AGACAGAGGGUUUCACGAAGCUCGGCCCGACAGUGAUCGCCCUCGGUGGCUAUCUAGUCACAUUCUAUUUUCUUGCGCUGGCAUUGCGGACCAUCCCGACCGGUGUCGCUUACGCGAUCUGGGCAGGCUUAGGCGUAGUGUUGGUUGCGGGUGUUGCAUGGCUGAUCCAAGGCCAGAAGC
Ssp-15-1 UCAUGAAACAAUCGGAAGGCUUCACGAAGCUCGUCCCGUCGCUGAUUACCGCCGGCGGCUAUAUUGUGGCCUUCUAUUUCCUCUCGCUGACCCUCCGGACCUUGCCCACGGGAAUCGCCUAUGCGAUCUGGUCCGGCGUCGGGAUCGUGCUGAUCGCGGCGAUCGCGUGGGUGUUUCAGG
Ssp-18-1 CGACCAGUUUCAUGAAACAAUCGGAAGGCUUCACGAAGCUCGUCCCGUCGCUGAUUACCGCCGGCGGCUAUAUUGUGGCCUUCUAUUUCCUCUCGCUGACCCUCCGGACCUUGCCCACGGGAAUCGCCUAUGCGAUCUGGUCCGGCGUCGGGAUCGUGCUGAUCGCGGCGAUCGCGUGGG
env-198 CAUCUCGCUUGUCGAGCAUCCCGCGCGCCUUCGGCUGGACGACGCAUCAAUGC......
Ssp-16-1 UGAAACAAUCGGAAGGCUUCACGAAGCUCGUCCCGUCGCUGAUUACCGCCGGCGGCUAUAUUGUGGCCUUCUAUUUCCUCUCGCUGACCCUCCGGACCUUGCCCACGGGAAUCGCCUAUGCGAUCUGGUCCGGCGUCGGGAUCGUGCUGAUCGCGGCGAUCGCGUGGGUGUUUCAGGCC
env-199 CGAUCUACAUCAGUCUCGUUGAGCAUCCUGCCCGACUG......
env-200 CAUCACGCUGGUCGAGCAUCCGGCACGGCUCCAACUGGCGGAUGGCUCCC......
Sdo-1-1 CAUCACGCUGGUCGAGCAUCCGGCACGGCUCCAAUUGGCGGAUGGCCCCCUGCUCGCGCAAUGGAAGCCCAGUUAUGACAGGGCGCUGCCGAUCCAGUCCGGCCUAGCUAUUGUCGGAGGCCUCAGCGGAAUGGGCGCCUGGUAUCUGUCGGGCACUGUGCUCUGGGUAUUGGGAAGCCU
env-201 CAUCACGCUGGUCGAGCAUCCGGCACGGCUCCAACUGGCGGAUGGCUCCCUGCUCGCGCAAUGGAAGCCCAGCUAUGACAGGGCGCUGCCGAUCCAGUCCGGCCUAGCCAUUGCCGGAGGCCUCAGCGGAAUGGGCGCUUGGUAUCUAUCGGGCACUGUGCUCUGGGUAUUGGGGAGCCU
Bro-1-1 ACAUCAGUAUCGUCGAGCAUCCCGCUCGGAUGAGCCUGGAGGACGCGCCCCUGCUCGCGCAGUGGCAGCUCAGCUACAAGCGCGCCCUGCCCAUUCAGGCUGGCUUGGCCAUGCUGGGAGGGGCCGCAGGGCUCCUGGCUUGGUAUGGCUCCCGCGAGUGGCAGUGGAUGGCAGGGAGCC
Bro-1-2 ACAUCAGUAUCGUCGAGCAUCCCGCUCGGAUGAGCCUGGAGGACGCGCCCCUGCUCGCGCAGUGGCAGCUCAGCUACAAGCGCGCCCUGCCCAUUCAGGCUGGCUUGGCCAUGCUGGGAGGGGCCGCAGGGCUCCUGGCUUGGUAUGGCUCCCGCGAGUGGCAGUGGAUGGCAGGGAGCC
env-202 ......
env-203 UUUACAUCACGUUCGUCGAGCAUCCUGCCCGCCUGACACUCGACGAUGCUUCGGCGCUGGCCCAGUGGAAGCCCAGCUACGAUCGCGCUUUGCCGGCACAAGGCAGCUUGGCAAUCCUGGGUGCGCUUUUCGGAGCGGUCUCUUGGUAUUAUUCGGCUGACUGGCGCUGGCUGGCCGGAA
env-204 UUUACAUCACGUUUGUCGAGCAUCCUGCCCGGCUGACACUCGACGACGCUUCGGCCCUUGCUCAAUGGAAACCCAGCUAUGACCGCGCCUUACCUAUACAGGGCAGCUUGGCGAUUCUGGGCGGGCUUGUCGGAGCGAUCAUGUCGUAUCAC......
env-205 ......
env-206 ......UUUCGCGCCUUCCCGGCU
env-208 ......UUUCGCGCCUUCCCGGCU
env-209 ......UUUCGCGCCUUCCCGGCU
env-210 AUAAGCCUCGUCGAACAUCCGGCGCGUGUCGGACUAGUAGACGGUCCUCUGCUGAUGCAGUGGCAGCCCAGCUACAAGCGGGCGCUUCCAAUCCAGUCUGGGCUGGCCGUCGCCAGCGGCUUGGCGGAUUGAUCGUCGGAUAUUACU......
env-211 CAUCAGCCUUGUCGAACACCCGGCCCGCCUCGCCUUGGCCGAUGGUCCGUCGCUCGCGCAGUGGCAGCCCAGCUACAAGCGGGCGCUUCCGAUCCAGUCUGGUCUGGCCAUCGCUGGCGGCGUGGCGGGGCUGGUGGCGGGAUACCUUGCGGCAGACUGGCGAUGGUUUGCCGGAAGCGC
env-212 CAUCAGCCUCGUCGAACAUCCUGCGCG......
env-213 CAUCAGCCUCGUCGAACAUCCUGCGCGUCUUGGAUUGGAAGAUGGUCCUUUGCUCGCGCAGUGGCAGCCCAGCUACAAGCGAGCGUUACCGAUCCAGUCCGGCUUAGCGGUGGCCGGGGGCGCGGCGGGACUGAUCGUCGGGUAUCUCUCGGCCGAUUGGCCGUGGUUUGCCGGAAGCGC
env-214 UAUAACCCUGGUCGAACACCCUGCACGUCUCGGGUUGGCCGAUGGGCCUCUGCUGGCCCAGUGGCAGCCCAGCUAUAAGCGAGCGCUACCCAUUCAGUCUGGGUUAGCGAUUGCCGGGGGCGCGGCGGGACUGAUCGUCGGGUACCCGUCGGCGGAUUGGCGAUGGUUUGCCGGAAGCGC
env-223 UAUAACCCUGGUCGAACACCCUGCACGUCUCGGGUUGGCCGAUGGGCCUCUGCUGGCCCAGUGGCAG......
env-224 UAUAACCCUGGUCGAACACCCUGCACGUCUCGGGUUGGCCGAUGGGCCUCUGCUGGCCCAGUGGCAGCCCAGCUAUAAGCGAGCGCUACCCAUUCAGUCUGGGUUAGCGAUUGCCGGGGGCGCGGCGGGACUGAUCGUCGGGUACCCGUCGGCGGAUUGGCGAUGGUUUGCCGGAAGCGC
env-225 CAUAAGCCUCGUUGAACAUCCUGCGCGUCUCGGUUUGGCAGAUGGCCCUCUGUUGGCGCAGUGGCAGCCCAGCUACAAGCGAGCGCUACCGAUUCAGUCCGGGUUAGCGGUCGCCGGGGGCGUGGCGGGGCUUGUCGUCGGGUAUCUCUCGGCCGAUUGGCGAUGGUUUGCUGGAAGCAC
env-226 CAUAAGCCUGGUUGAACAUCCUGCGCGUCUCGGUCUAGCAGAUGGUCCGCUGUUGGCGCAGUGGCAGCCCAGCUACAAGCGAGCGCUGCCAAUCCAGUCCGGUCUGGCGGUCGCCGGGGGCGUGGCGGGGCUGAUCGUUGGGUAUCUCUCGGCCGAUUGGCGAUGGUUUGCCGGAAGCGC
Ssp-8-2 CAUCAGCCUCGUCGAACAUCCUGCGCGGCUCGGAUUGGCAGACGGCCCCCUGCUUGCGCAGUGGCAACCCAGCUACAGGCGAGCGUUACCUGUUCAGUCCGGGCUGUCGGUCCUCGGCGGCGUCGCGGGACUGAUCGUCGCUUAUCUUUCGGCGGAUUGGCGAUGGCUUGCCGGAAGCGU
env-227 CAUAAGUCUCGUGGAGCAUCCUUCACGUCUCGGAUUGUCGGAUGGCCCUUUGCUGGCGCAAUGGCAGCCCAGCUACAAGCGAGCGCUACCGAUCCAAUCCGGGUUGGCNAUUGCCGGGGGCGCGGCNGGACUGAUCGNCGGGUAUCUUUCGGCGGAUUGGCGGUGGUUUGCCGGAAGCAU
env-228 CAUAAGUCUCGUGGAGCAUCCUUCACGUCUCGGAUUGUCGGAUGGCCCUUUGCUGGCGCAAUGGCAGCCCAGCUACAAGCGAGCGCUACCGAUCCAAUCCGGGUUGGCAAUUGCCGGGGGCGCGGCGGGACUGAUCGCCGGGUAUCUUUCGGCGGAUUGGCGGUGGUUUGCCGGGAGCAU
env-229 CAUAAGUCUCGUGGAGCAUCCUUCACGUCUCGGAUUGUCGGAUGGCCCUUUGCUGGCGCAAUGGCAGCCCAGCUACAAGCGAGCGCUACCGAUCCAAUCCGGGUUGGCAAUUGCCGGGGGCGCGGCGGGACUGAUCGCCGGGUAUCUUUCGGCGGAUUGGCGGUGGUUUGCCGGGAGCAU
env-235 CAUAAGUCUCGUGGAGCAUCCUUCACGUCUCGGAUUGUCGGAUGGCCCUUUGCUGGCGCAAUGGCAGCCCAGCUACAAGCGAGCGCUACCGAUCCAAUCCGGGUUGGCAAUUGCCGGGGGCGCGGCGGGACUGAUCGCCGGGUAUCUUUCGGCGGAUUGGCGGUGGUUUGCCGGGAGCAU
env-236 CAUAAGUCUCGUGGAACAUCCUUCACGUCUCGGAUUGUCGGAUGGCCCUUUGCUGGCGCAAUGGCAGCCCAGCUACAAGCGAGCGCUACCGAUCCAAUCCGGGUUGGCAAUUGCCGGGGGCGCGGCGGGACUGAUCGCCGGGUAUCUUUCGGCGGAUUGGCGGUGGUUUGCCGGGAGCAU
env-237 CAUAAGUCUCGUGGAACAUCCUUCACGUCUCGGAUUGUCGGAUGGCCCUUUGCUGGCGCAAUGGCAGCCCAGCUACAAGCGAGCGCUACCGAUCCAAUCCGGGUUGGCAAUUGCCGGGGGCGCGGCGGGACUGAUCGCCGGGUAUCUUUCGGCGGAUUGGCGGUGGUUUGCCGGGAGCAU
env-238 CAUAAGUCUCGUGGAGCAUCCUUCACGUCUCGGAUUGUCGGAUGGCCCUUUGCUGGCGCAAUGGCAGCCCAGCUACAAGCGAGCGCUACCGAUCCAAUCCGGGUUGGCGAUUGCCGGGGGCGCGGCGGGACUGAUCGCCGGGUAUCUUUCGGCGGAUUGGCGGUGGUUUGCCGGAAGCAU
env-239 CAUAAGUCUCGUGGAGCAUCCUUCACGUCUCGGAUUGUCGGAUGGCCCUUUGCUGGCGCAAUGGCAGCCCAGCUACAAGCGAGCGCUACCGAUCCAAUCCGGGUUGGCGAUUGCCGGGGGCGCGGCGGGACUGAUCGCCGGGUAUCUUUCGGCGGAUUGGCGGUGGUUUGCCGGAAGCAU
env-240 CAUAAGUCUCGUGGAGCAUCCUUCACGUCUCGGAUUGUCGGAUG......
env-241 CAUAA......GUCUCGUGGAGCAUCCUU
env-242 CAUAAGUCUCGUGGAGCAUCCUUCACGUCUCGGAUUGUCGGAUGGCCCUUUGCUGGCGCAAUGGCAGCCCAGCUACAAGCGAGCGCUACCGAUCCAAUCCGGGUUGGCGAUUGCCGGGGGCGCGGCAGGACUGAUCGCCGGGUAUCUUUCGGCGGAUUGGCGGUGGUUUGCCGGAAGCAU
env-243 CAUAA......
env-244 CAUAAGCCUCGUCGAACAUCCUGCACGGCUCGGAUUGGCCGAUGGUCCUCUGCUGGCGCAGUGGCAGCCCAGCUACAAGCGAGCGCUACCGAUCCAAUCCGGGUUAGCGGUUGUUGGAGGCGCGGCGGGACUGAUCGUGGGCUAUCUUUCGGCGGAUUGGCGAUGGUUUGCCGGCAGCAU
Swi-1-1 CAUCAGUCUCGUCGAACAUCCGGCGCGUCUCAGAUUGGCAGAUGGUCCCCUGCUGGCCCAGUGGCAGCCCAGCUACAGGCGAGCGCUACCGAUGCAGUCCGGGUUAGCGGUCGCCGGGGGGCAGCGGGAUCAUCGUUGGGUACCUUUCGGCGGAUUGGCGGUGGUUUGCCGGGAGCAU
Ssp-5-1 UACAUAAGUCUCGUCGAACAUCCUGCGCGUCUUGGUUUAGCAGAUGGUCCAUUGCUGGCGCAGUGGCAGCCCAGCUACAAGCGAGCGCUUCCGAUCCAGUCGGGCUUAGCGGUCCUCGGGGGCGCGGCGGGGCUGAUCGUUGGGUAUUACUCGGCUGAUUGGCGCUGGUUUGCUGGAAGC
env-245 CUACAUCACCCUUGUUGAACAUCCCGCCAGGAUGGGUUUGGACGAUGGGGCCCUGCUGGCGCAGUGGCAGCCCAGCUACAAACGUGC......
env-246 GGCGCGGCGCUCUACAUCAGCCUGGUCGAGCACCCCGCCCGUUUGGGCCUCGCCGACGGGCCUUUGCUCGCCCAGUGGCAGCCGAGCUACAAAAGGGCGCUUCCGAUCCAGGCGGGCCUUGCGAUCGCGGGAGGGGUGGCGGGACUGAUUGCCGGCUAUCGGAGCGGGGACUGGCAGUGG
env-247 UACAUCAGCCUCGUUGAGCAUCCCGCUAGGAUGAGCUUG......
env-248 ......
env-249 ......
env-250 ......
env-251 ......
env-252 ......
env-253 ......
env-254 ......
env-255 CGACCGUCGUCGCGCUCGCCGGCUACGGGGUGUCCUUCUAUUGCCUGUCCCUCGCGCUGCGGACCCUGUCCACCGGCAUCGCCUAUGCCAUCUGGUCGGGCGUGGGCAUCGUGCUGGUCGCGACCGUGGCCUGGGUGUUCCAGGGACAGAAGCUCGACAUCGCGGCCAUGGCCGGCAUGG
env-256 UUACAUCACCCUUGUCGAACAUCCCGCCAGGAUGGGGUUGGACGAUGGGCCUCUGCUGGCGCAGUGGCAGCCUAGCUACAAACGUGCUCUGCCUAUCCAGGCUGGCCUGGCAGUAUUGGGAGGGGCUGCAGGGCUCCUGGCUUGGUAUGGCUCCCGCGA......

3′

alignment positions 541 · · · 720

env-1	UGUUCUUAGUCCUCACAGUUCCGAUUGCGCACCCGUUUUGGCGAAUGCCCGAGCCGCAGCGCACUUUCGAGUUCUAUGUCGUUCUCGAACACGUUUCGCUUGUAGGAGGAUUGAUGGUCGCGGCCGCCUUGUGUCAUCGGCGCGCCCAGAAGACAGCCUGACCGGCGCUUUGGAUCCAAU
env-2	AGAUGCCCGCCGCGCUGGAGGCGGUGGGGACGCUGACGGCCGUGCGCGAAGUGACGCUCUCGCCGGAGGUGGCGGGCCGCGUCUCCGCCAUCCGGCUCGAGGCGGGAUCGCGGGUCGGUGCCGGGACGCUGCUCGUGCAGCUUUAUGAUGGCCCCGAGCAGGCCGACCGCCGCGCGGCGC
env-4	GAUGCGCG..
Ral-1-1	GCUGCGGCAGCGACGCUUCUUCGAGCGAAGGAAUAUGCGCAUCUCGCAGGGCCUGUUGUUAUCUUCAUCCUGACAGCAAUUGUGUUCUGGGAUGCCGGACUAGCGUCGUAAAACAACAAGUUCCCACCUGAGAAUCCGAGCAAUUUCGAACCCUUCACCCGGGGCGCAAGACAGCUCUGG
env-5	....
env-6	CUGCAAUUAUAACUCUCAUUUACCAUAAAGAAUUUAAGCACGCUAUUCCCGCAGGAGUGGUUGCGUUUCUCUGCCUGGUUUUAAUUUUUACUCUUCAGCUUCAAUCAUAGUUCUCAGAGUCAAAACAUCUAAAAAAAAAGGCUUCACCACAGCCAGGGGAAGCCUUGAUUCAAAUAAUCAA
Spa-1-1	GCCGGAGCGCAGCCGUUUCGGGGGAAGCCAGACCCGGACCCUCAUUGUCACCCUCGUGAUCCUGGCCGUCGUCCUGGGCGGGCUGUUCCUGUGGCGCACGAUGCGCACCGGGCAGCAACAAGGCUGGCAGCAGCAGGCGACGUCCGUCGCCGCGACCGUCGUGACGCCGCAAGACGUGCC
env-7	CGACGGUCGUGCAGCCGCGCGUGGUGCCGGCGUCGCUCGACGCGGUCGGCACGCUGACGGCGGUGCGCGAAGUGACGCUGUCGCCCGAAGUCGCCGGACGCAUCUCGGCGAUCCGGUUCGAGGCGGGGCAGCAGG..
env-8	....
env-9	....
env-10	....
env-11	....
env-12	UCGUGCAGCCGCGCAUGGUGCCGGCGUCGCUCGACGCGGUCGGCACGCUGACGGCGGUGCGCGAAGUGACGCUGUCGCCCGAAGUCGCCGGACGCAUCUCGGCGAUCCGGUUCGAGGCGGGGCAGCAGGUCGGCGCCGGCGCGCUGCUCGUCCAGCUGUUCGACGGACCGGAGCGGGCCG
env-13	CGUGGUUGAUCCGCGCGAUGUGCCCGCCUCGCUGGAUGCGGUCGGCACGUUGACGGCGGUGCGCGAAGUGUCGCUGUCGCCGGAGGUGGCGGGGCGCGUCUCCGCGAUCCAUUUCAAGGCCGGCUCGCGCGUCGCGGCGGGCGCAUUGCUCGUGCAAUUGUUCGAUGGGCCGGAGCAAGC
env-14	CGUCGUCAAUCCGCGCGACGUGCCCGCAUCUUUGGACGCCGUCGGCUCGCUGACGGCCGUGCGCGAAGUGUCGCUGUCGCCCGAAGUGGCGGGGCGCGUUUCCGCGAUCCGCUUCAAGGCCGGUUCGCAGGUCGCGGCGGGGGCGUUGCUGGUGCAGUUGUUCGACGGUCCCGAGCAGGC
env-15	GAGCCAUGCAUGGGUGAUCGCCGACGGCCCAGUGCGUGCGAUCGUAAUUGUCAACUUUGUUCACCAUAAGCCGGCGAUAACGAAUAAUGCCGUUUCCCAGACCGAUACAAUAGGAAUGCACCGUGGAAGAUCUGAUGCGCAUGGCGAUCUUCGCCCGAGUGGUAGAGGCGAAGAGCUUUU
env-16	....
env-17	UGCCUCCACCGACCCAUGGUGCAUCGCGGCCCAUCCGUACCAUCGCAUUGACCGUCGCGAUUCUGCUCCUGCUGCUCGGCGCACUCUAUCUGUGGCGCGGCUGGCGCGCCGGGCAGGCGCAGGCGUGGCAGCCGCAGGCUGUGCCGGUCGCCGCCACGCUGGUGCAGCCGCGCGAUAUCC
env-18	GUGUUUCUGGCACUCACGAUACCUAUCGCCCAUCCCUUCUGGCAGAUGUCCGAGCCGAACCGCACCUUCGAGUUCUACGUUGUUCUUGAGCAUAUCUCCCUGAUCGGCGGACUGAUGAUUGCAGCAGCACUCAGCGGACGCAAGACGAUCUCGUGAGGUGAUCAGGAUGUAGGCCAGCCA
env-20	GUGUUUCUGGCACUCACGAUACCUAUCGCCCAUCCCUUCUGGCAGAUGUCCGAGCCGAACCGCACCUUCGAGUUCUACGUUGUUCUUGAGCAUAUCUCCCUGAUCGGCGGACUGAUGAUUGCAGCAGCACUCAGCGGACGCAAGACGAUCUCGUGAGGUGAUCAGGAUGUAGGCCAGCCA
env-21	GUGUUUCUGGCACUCACGAUACCUAUCGCCCAUCCCUUCUGGCAGAUGUCCGAGCCGAACCGCACCUUCGAGUUCUACGUUGUUCUUGAGCAUAUCUCCCUGAUCGGCGGACUGAUGAUUGCAGCAGCACUCAGCGGACGCAAGACGAUCUCGUGAGGUGAUCAGGAUGUAGGCCAGCCA
env-22	GUGUUUCUGGCACUCACGAUACCUAUCGCCCAUCCCUUCUGGCAGAUGUCCGAGCCGAACCGCACCUUCGAGUUCUACGUUGUUCUUGAGCAUAUCUCCCUGAUCGGCGGACUGAUGAUUGCAGCAGCACUCAGCGGACGCAAGACGAUCUCGUGAGGUGAUCAGGAUGUAGGCCAGCCA
env-23	GUGUUUCUGGCACUCGCGAUACCUAUCGCCCAUCCCUUCUGGCAGAUGUCCGAGCCGAACCGCACCUUCGAGUUCUACGUUGUUCUUGAGCAUAUCUCCCUGAUCGGCGGACUGAUGAUUGCAGCAGCACUCAGCGGACGCAAGACGAUCUCGUGAGGUGAUCAGGAUGUAGGCCAGCCA
env-24	GUGUUUCUGGCACUCGCGAUACCUAUCGCCCAUCCCUUCUGGCAGAUGUCCGAGCCGAACCGCACCUUCGAGUUCUACGUUGUUCUUGAGCAUAUCUCCCUGAUCGGCGGACUGAUGAUUGCAGCAGCACUCAGCGGACGCAAGACGAUCUCGUGAGGUGAUCAGGAUGUAGGCCAGCCA
env-25	....
env-26	GUGUUUCUGGCACUCACGAUACCUAUCGCCCAUCCCUUCUGGCAGAUGUCCGAGCCGAACCGCACCUUCGAGUUCUACGUUGUUCUUGAGCAUAUCUCCCUGAUCGGCGGACUGAUGAUUGCAGCAGCACUCAGCGGACGCAAGACGAUCUCGUGAGGUGAUCAGGAUGUAGGCCAGCCA
Cpa-1-1	CGCUGGCUCGGUUGCAAGGCUGGUGCGGCCAAAUUUCGCUACGCGGAUCUGACGGACGCACGUCUUUGCCGGUGCGAUUUGAACAACUCGCAGUGGUCUGGAGCCAAGCUCGCAUCGGCAAUGUUUGUGGAAGUAAAGCUCACCGGUGCGCGUCUCAACGAUGUGCAUUCCCUCGGGCUC
Ysp-1-1	GCAACCCCAGGUCCAGUCCCAGACCCCGUUCUGAUAUGGCGCCGGACGACCGUGAAUGAAGAUGUCGCCACCCGGGCUCUUGCCGAGAUAGCGUGCCUCGGCGCGGUCGGCGGCAUUGGGAUAGUCGAUCCCGAGACUCAGGUGGAACCGCGAGUUCGGGUUGCGCCGGUCGAUCAUGUA
env-31	....
env-32	....
env-33	....
env-34	....
env-35	AUGUCCGCUGCAGCGCUGACCUUGCUCCGACACAGGGAAAACGGCCAUGCGGUAGUGCCGACCGUUGUCUUGGGUCUAUGCAUUGCGGCGGCCGCUUUGCAAUAGAUGCUCUAGUCGAUGCCUUGCAGAAGCCAUGAAGGCUUUCUGCAAGGCAUUGGCCUAAUCCGGGCCCGUCAGGCU
Bsp-1-1	AUCGCAUCUAAGAAGAGACGAAUGCCGAUUGUCGCGGGCGUCAGGCUUCUGAUUCUCAUUCCGGCCGCCUUGUUUCUCAGCGUCAAAGCGCACAGCGGGGCGUUCGACGCCGCAUUUUAUGCGGUGCAGGCGAUCGAACUUAUCGCAGGCGCCUCAAACAUCCUGCUUCUCGCAGCCAGC
env-36	GGCGCAGGGCUGGCAGCCGCAGGCGGUGCCGGUCGCCGCCAUGCAGGUCAUGGCCCGCGACCUGCCCGCCGCGCUGGAAGCGGUCGGCAACCUGCGCGCCGUCCGCGAAGUCACGCUGUCGCCCGAAGUCGCGGGCCGCGUGUCCGCCCUGCAUUUCGAGGCGGGGGCGCAGGUCGGCGC
Ssp-11-1	CCGCGAUGCUGGGCAUGGCGCUGAUCGUCGCGGGCGUGAUCGUGAUGAACGUGUUUUCGAAGGCUGCGGGGCAUUAGGGAUUUGAUCCAGGCUUCUGGAAGCACCUCCCCGGCGAAGGCCGGGGGCCAGGUGGGAGAGCGUUGGCAGAACCCGGUGCGCCUGCCAGCUAUGUCGCGCAAC
Ssp-7-1	CCGCGAUGCUGGGCAUGGCGCUGAUCGUCGCGGGCGUGAUCGUGAUGAACGUGUUUUCGAAGGCUGCGGGGCAUUAGGGGUUUGCGGGUGUUGCCAAUGCUUUUGCACCUCCACAUAGUGGUGCGGUGAUCGAAAGCCGGCUUGGCUUAGAGGAUGUCGGCGGUGGCUGCGGAGAAGGCC
Nsp-2-1	ACGCGCCCGCGCUCCUUGGGAUUGGCUUGAUAGUGGCCGGCGUUGUCGUGAUGAACGUGUUUUCCAAGGCGGCAACUCACUAAGGCCGCUCGUUUGUGGGUUCACAACCUAGUUUGUCGACUUUGGCUUCGCGUUGGCGCGAUUCAGUUCGAUGAGAAGCCAAUCCCGAAACUUGCCCAU
Rsu-1-1	GGGCACCGUGCUGAUCGCGCUGGUCAGCGUCACGAUCUUCCGGCAAUCGCUGGAUGCGGCGGCCAUAGUGGGGAUCGCCAUGAUCGUGGGCGGCGUCGUCAUCAUGAACGCCUUUUCGACGGUCUCUCACUAGCAGAGGCCGCCGCCAAGGGCUUGGCGACAGAGCAAAGAUGGCCCGCG
env-37	CGCAAUCUUGGGGAUGGGUCUGAUCGUCUCCGGCGUGGUUGUCUUGAACCUCUUCUCGAAGUCCUCCGCGCAUUAGCAAUGAGGUGCUGGUUGGGCCUUGAAACGCUCUCCCAUCGCUAAGCGGCCUUCUUUUGCGCCACCCAUUUUUUCACGAAGCCGAGAAACGCGGCGUCGGGAUUU
Sya-3-1	CGUUAUCCCGCGAUUGAUCCGCCCAACACGCACCAUGUUGCUGACGAUCGUCCUGCUUUUCGUGCUUCUGGGGACCCUUUAUCUUUGGCGUGGAUGGCGCGCGAGCCAGGCGCAGGCCUGGCAGCCCCAAGCGGUAUCGGUCGCUGCCAUGGUCGUGCAGUCGCGCGACAUUCCUGCCGC
env-40	CGUUAUCCCGCGAUUGAUCCGCCCAACACGCACCAUGUUGCUGACGAUCGUCCUGCUUUUCGUGCUUCUGGGGACCCUUUAUCUUUGGCGUGGAUGGCGCGCGAGCCAGGCGCAGGCCUGGCAGCCCCAAGCGGUAUCGGUCGCUGCCAUGGUCGUGCAGUCGCGCGACAUUCCUGCCGC
Sya-1-1	CGUUGUCCCGCGAUUGAUCCGCCCGACACGCACCAUGUUGCUGACGAUCGUCCUGCUUUUCGUGCUUCUGGGGACCCUGUAUCUUUGGCGUGGAUGGCGCGCGAGCCAGGCGCAGGCCUGGCAGCCCCAAGCGGUACCGGUCGCUGCCAUGGUCGUGCAGUCGCGCGACAUUCCUGCCGC
Sya-2-1	CGUUGCCCCGCGAUUGAUCCGCCCGACACGCACCAUGUUGCUGACGAUCGUCCUGCUUUUCGUGCUUCUGGGGACCCUGUAUCUUUGGCGUGGAUGGCGUGCGAGCCAGGCGCAAGCCUGGGAACCCAAAGCGGUACCGGUCGCUGCCAUGGUCGUGCAGUCGCGCGACAUUCCUGCCGC
Ssp-3-1	CGUUGCCCCGCGAUUGAUCCGCCCGACACGCACCAUGUUGCUGACGAUCGUCCUGCUUUUCGUGCUUCUGGGGACCCUGUAUCUUUGGCGUGGAUGGCGCGCGAGCCAGGCGCAAGCCUGGCAGCCCCAAGCGGUACCGGUCGCUGCCAUGGUCGUGCAGUCGCGCGACAUUCCUGCCGC
env-42	....
env-43	UGGCGACCGGAUUGGGCACCGCCGAACAUUUCUUUGGGGUCUGCUUGCUUUUGCCCUUGCUUCUCUGAUCGCGGCUUAUGCCCCCUCAGCGAACGUCCUGAUCGCGGCACGUGCCUUUCUUGCCGUGGGCGCAUCCAUGAUGAUGCCCGCGACGCUUUCGCUUCUGCGGCUGACAUUUAC
env-44	UGGCGACCGGAUUGGGCACCGCCGGAUAUUCCUUUGGGGGCUCGUUGCGUUCGCUUCUGCCUCUUUGCUCGCAGCUUAUGCCCCGUCU..
env-45	UGGCGACCGGAUUGGGCACCGCC..
env-46	....
env-47	....
env-48	....
env-49	....
env-50	....
env-51	....
env-52	....
env-53	GGCGCCCAGGAUACGAGGAACAUGCCGGAUGAGGCGAAGCCGCCUCAAGCCGUGCCUUUCUUUCUUCUAGUCGACAAUCGCGGUACUUUCAGUUAUCAGUCCACCGCAACACUACCAGGCGUCACCAGCAAGACAGCCCAGCAGACUUUCGAGUUUUCUCAUCUUGAUAUCUGGCGCUAC
env-54	GGCGCCCAGGAUACGAGGAACAUGCCGGAUGAGGCGAAGCCGCCUCAAGCCGUGCCUUUCUUUCUGCUAGUCGACAAUCGCGCUACUUUCGCUUAUCAGUCCACCGCAACACAACCAGG......................................
env-55	GGCGCCCAGGAUACGAGGAACAUGCCGGAUGAGGCGAAGCCGCCUCAAGCCGUGCCUGUCUUUCUGCUAGUCGACAAUCGCGCUACUUUCGCUUAUCAGUCCACCGCAACACAACCAGGCGUCACCAGCAAGACGGCCAAGCAGAUUUUCGAGUUUUCUCAUCU...............
env-56	....
env-57	....
env-58	....
env-59	GCAUCGGACACGACGAGCACUUCGGAUGAGGCGAAACCUGAUGACGAUGUGCCCUUCUUCCUUUUAGUCGACAACCGCGCUAGCUUCGCCUAUCAAUCCACCGCAACACAACCAGGCAUCACCAGCAAGACGGCCAAGCAGAUUUUCGAGUUUUCUCAUCUUGAUAUCUGGCGCUACGGC
env-60	....
env-61	UUGGCGACCGGAUUGGGCAUCGCCGAGUCUUUCUUUGGGGCCUGAUUGCUUUUGCCUCUGCUUCUCUGAUCGCGGCUUAUGCCCCUUCUGCGAGCGUCCUGAUCGCGGCGCGGGCCUUUCUUGCUGUGGGCGCAUCCAUGAUGAUGCCAGCCACGCUUUCGCUUCUGCGGUUGACCUUUA
env-62	....
env-63	....
env-64	UGCCGCCGUUGCCGGCAUCGCGCUGAUCGUGGCCGGCGCGGUAGUGCUGAACCUGUUCUCAAAGACGGUCGGGCACUGAGGCUGCAUCGGCGCGGUAUAGCCGACAUGGGCUAUACUGAUCAGGCGAGUGGUGGCGCCUCUCGGGGGUCGG..........................
Ssp-12-1	CUGGCCAACUGGCCGUUCACGCUCCUGAUCAUCAUGCCCGUCAACAAGCGCCUGAUGGCCAUGAGCCCGGGUCAGGCUGGUCCCGAAAGCCGCCGGAUGCUGGUGCGAUGGGGCAAGCUGCACGAUGUUCGAAGCGCGCUCGGGCUCGUGGCCACGCUGCUGUUCGCUUGGGCGUCGGCG
env-65	....
env-66	UAUGCUCAUUUCGGUUUGGCUGACGAUCGCUGUCGCUUACGGCGGCUGAACCGAACAGGCGCUCCUCAAGGAAAGACCUAAACCCGACCCGCUUAAGCUAUCGCGCCUUAUCGUUUCGGAUGGUGGCACUCGGCAAGCCGCCAUGUUGCUCAGGCAGAUCCUCCAAAGCGAGCGCUGCGU
Ssp-10-1	UAUGCUCAUCUCAGUCUGGCUAACGGUCGCUGUUGCUUACGGCGGCUAACCUGAACAGGCGCUCCUCGGGGAAGGCUUAAACUCAAUCCGCCUAAGCGAUCGCGCCUGAUCGUUUCGGAUGGUGGCACUCGGCAAGUCGCCAUGUUUCUCAGGCAGAUCCUCCCAAGCCAGCGCUGCGCA
Bst-1-1	CUGGCUGGGCUGCAAGGGCGCCACGGCAAAUUUCCGGUAUUCCAAUGCGACUGACGCCCGAUUCUGCCGGAGCGAUUUGAACAAUACCGACUGGACCGGCGCCAAGCUGGCAUCGACCGUCUUUACGGAGGUUCGGCUGACGGGCGCCCGCUUCGCGGACAUCCGGGCGCUGGGGCUCAG
env-67	....
She-1-1	ACUCGACGCUCCAGCCAUGAUCGGGAUGGGACUUAUCAUCUGCGGUGUGAUCGUGAUGAACGUCUUUUCCAAAGCGACAGGGCACUAAAAUGCGGAGUUAGCAGAUACCGUCGCUCAACUUCCAUUGAACCUGUUGUUCCAAAGCAGCAUUCUAAUCGAGAUGCUAGGAUAGUCAGAUCA
Ssp-13-1	ACUCGAUGCCCCCGCGAUGAUCGGGAUGGGGUUGAUCAUCUCGGGCGUGGUCGUGAUGAACCUGUUUUCGAAGGCAGCCGCGCACUAGUCGCAACGCCGCCCUAGAGAAACGAUGAUAGCGUUGUCAUAGCUAUGCAAUCGCUGGUGGCUUGCAUUGCUGCGGCGUUUGCCUGAUUGGGA
env-68	....
env-69	....
Ptr-1-1	GGAUUGGCUGUCGAGGCGCGGCGGCCAGUUUCCGCUAUGCGAAUGCGGGCGACGCACGCUUUAUUCGCAGCGAUCUGAACAAUACCGACUGGACCGGCGCGAAACUCGCUUCAACCGUAUUUACCGAAGUGAAAUUGACAGGCGCGCGUUUCAUCGACACCCGCGCAUUGGGCCUGAGCU
Ptr-1-3	GGAUUGGCUGUCGAGGCGCGGCGGCCAGUUUCCGCUAUGCGAAUGCGGGCGACGCACGCUUUAUUCGCAGCGAUCUGAACAAUACCGACUGGACCGGCGCGAAACUCGCUUCAACCGUAUUUACCGAAGUGAAAUUGACAGGCGCGCGUUUCAUCGACACCCGCGCAUUGGGCCUGAGCU
Ssp-22-1	GCGCCGGCGCUGUUCGGCAUGGCCCUCAUCACCGCCGGGGUGGUCGUGAUGAACGUCUUCUCAAAGAGCGUGGCGCACUGAGAAGGCGAUGGCGGCCGCCGGGCCGGCUAGGCCAGCUCGACGGUCGUCACCAUAUAACCUGCGUCGCGGAGUGAGGCGACAAGACUGUCGAGAUGCUCG
Bsp-2-1	GGCUGAGGUGCAAGGCGCCCGCAGCGAAAUUGCGCUACGCCGACCUGACAGAGGCACGUUUCUGCCACAGCGACCUGAACAAUACGGACUGGACCGGUGCAAAUCUCGCGUCGGCUGUGUUUUCCGAGGCGAAGCUGACGGGCGCUCGUUUCACGGAAGCGCGCACGCUCGGUCUUAGCU
env-70	GAGUGUCGUCCUGAUAGCGAAUUGGCCAUUCACCCUGUCGAUGAUCAUGCCCGUCAACAAAUGCCUUCAAGCGCGGGUACCGGAAAAUAUCGAUCCGGACAGCCGCUCGCUUUUGGAUCGCUGGGCACGGCUUCAUGCCGCAAGGAGUGCCUUGGGCGCACUAUCAGCGCUGAUGUUCGU
Kvu-2-1	CAGCGCUGGUGAUGGCGGCGGCCUGCACAACAGUGAUAAAACAUAAGGAACUGGGCCACCUGCCCGCGCCUGCCAUUGUGCUGGUGCUGUGUUUGAUCUGUAUGUUGGGCAAUUUGAUUUAACGACAUGGCCCCCGCAGCGCGUAUUGCGGGGCCAUGAUUUCACAUCUGCAAUCAGAU
	....
	....
	oooooooooooooooooooooooooooooooooooooooooooooooooooooooooooooooooooooooooooooooooooooooooooooooooooooooooooooooooooooooo

env-71	GCCCGCAAUGCUGGGGCUGGCGCUGAUUAUCGCCGGGGUGGUGGUGAUGAACCUGUUUUCGAAGGCAGUGGCGCACUAGCAACUCUUCAAACUCCAGUGAGCCCUGAUCUGGCCUUACCGGAGGAUCGCCAGCUUUAGUCUAUCAGCACACCGAAAGCGGAUCGGCGACGUAGCGGUGUA
env-72	GCCCGCAAUGCUGGGGCUGGCGCUGAUUAUCGCCGGGGUGGUGGUGAUGAACCUGUUUUCGAAGGCAGUGGCGCACUAGCAACUCUUCAAACUCCAGUGAGCCCUGAUCUGGCCUUACCGGAGGAUCGCCAGCUUUAGUCUAUCAGCACACCGAAAGCGGAUCGGCGACGUAGCGGUGUA
env-73	ACCGGCGAUGGUCGGACUGGCGCUGAUCAUCGCCGGCGUGGUGGUGAUGAACCUGUUUUCCAAGGCAGUGGCGCACUAGGAGGCACCAGCCCGUGAAACGGAAAAGGCCGGGCAAUGCCCGGCCUUCCCUCUCCUCCCGGCAAGCACGGGAGAGUUGGUCAUAGCUUCAGAAGAAGCUGG
env-74	ACCGGCGAUGGUCGGACUGGCGCUGAUCAUCGCCGGCGUGGUGGUGAUGAACCUGUUUUCCAAGGCAGUGGCGCACUAGGAGGCACCAGCCCGUGAAACGGAAAAGGCCGGGCAAUGCCCGGCCUUCCCUCUCCUCCCGGCAAGCACGGGAGAGUUGGUCAUAGCUUCAGAAGAAGCUGG
env-75	....
Psp-1-1	CACAUCUGACCCUGCUGCACCAUCGCGAAACCCUGCAGUCCAUUGGCCCAGCAGUGGUUUUUGCUUUGCUUGCCCUUGUUCUGUGGCAGGUUGCAUUGCCAAGCUGACAGUCCGCUAUCCUUGGCAAGGGGUCCGGAUCAACCGGAUCCCUUACAGCAUGUCCCAGAUCAGCUUGAAGGC
Psa-1-1	UGGCAUCGUGCUCACGGCAAUCGUCAGCGUCGUCCUGUUCCGGCAGACUCUCGACGCAAUGGCGUUGGUUGGUAUCGCAAUGAUCGUUGGCGGUGUCAUCGUCAUCAACGCAUUCUCGAACGCUUCUCAUUGACGUGAUCGCGCAGAAGGACAUGAGCACUCGACUGUCCGCUGCGGGCU
env-76	UCAUAAUGAUCGCCAACUUGCCGGUGACCUUUGCGAUCAUUUGGCCAACCAACACCCGUUUGAUGCAUACCCAGAUCCAGGCAGCGGGGCCGGACAGCCGUGCGCUGAUAGUAAAGUGGGGCAAGCUCCACGGCAUCAGGACCGCACUAGGGUUUGGAGCCGCCGCGGCCUCUCUUCUGG
env-77	....
env-78	UCAUGAUGAUCGCCAACUUGCCGGUGACCUUUGCGAUCAUUUGGCCAACCAACACCCGUUUGAUGCAUACCCAGAUCCAGGCAGCGGGGCCGGACAGCCGUGCGCUGAUAGUAAAGUGGGGCAAGCUCCACGGCAUCAGGACCGCACUAGGGUUUGGAGCCGCCGCGGCCUCUCUUCUGG
Ssp-8-1	CGCUCGUGAGCGUGUUCUAUUUCAAGCAGUCGCUUGACCUUCCGGCGAUCGCGGGGCUGGCGCUGAUCAUUGCGGGCGUCGUUCUCAUCAAUGGAUUUUCCAACUCGCUUCAUACGCAGUGACCGAACGGGCGGCUUCCUGAUCGGAGGCCGCCCAAGGCCUUGCAGCGACCAUGGCGGC
Osp-1-1	GCAUUGUCCUGACCUCUCUGGUCAGCGUCUUCGUCUUCCGGCAGAGCCUCGACGCGAUGGCGCUGCUCGGCAUCGGGCGGAUCGUCAGCGGUGUGCUGGUGAUCAACCUCUUCUCGGGCUCCCCGCACCAGGCGGGGCUCAGCCCGGUGCCGGGGCGUCGUCGCGGCCCGGCUCGUCGG
env-79	GCUCGACGCGCCGGCGAUCGUCGGCAUGGGCCUCAUCAUCUGUGGCG..
env-80	GCUGGAUGCCCCCGCCGUGGUCGGCAUGGGCCUCAUCAUCUGUGGCGUGGUCGUGAUGAACGUCUUCUCGAAAGCCGCAGUGCACUAGGACAGGCGUGGUGGA..
Rma-1-1	UGGGCAUUGUCCUCACGGCGAUCAUCAGUGUCGCCGUUUUCAAGCAGACACUCGACGCGAUGGCUAUUUUGGGGAUUGCUAUGAUCGUUGGCGGUGUCGUCGUUGUGAACGUAUUUUCGAACGCGUCUCACUGAAUGCUCGCCCUACUCAGAGCGGAUCCAAGCGUCUGCUCUGGGCUCG
Spa-1-2	UCUUGCGAACUGGCCCUUCACGCUCUUGGCCAUCUUUCCCGUCAAUAAGCGGCUCAUGGCCCUGAACCCACGUCUCGCCGACUUCGAAAGUCGCGCGAUGCUCGUCCAUUGGGGCCGGCUUCAUGGUGUCAGAAGUUUUCUGGGAGCCGCAGCCAUGCUUUUGUUUGCCUUGCCAUUCGU
Squ-1-1	UCUUGCGAACUGGCCCUUCACGCUCUUGGCCAUCUUCCCCGUCAAUAAGCGGCUCAUGGCCCUGAACCCACGUCUCGCCGGCUUCGAAAGUCGCGCGAUGCUCGUCCAGUGGGGCCGGCUUCAUGGUGUCAGAAGUUUUCUGGGAGCCGCAGCCAUGCUUUUUUUUGCCUUGCCAUUCAU
env-81	....
Sph-1-1	GUGCUGCUCGCCAAUUGGCCGUUCACGCUUCUGGCGAUCAUGCCGGUCAACAACCGCCUGAAGGCGAUAACGCCGGAUCAGGCCGGGCCGGAAAGCCGGACCCUGCUGCUGAAAUGGGGAAGCUCCACAAUGUCAGGAGCGGCCUUGGCUGCGCGGCGGCCCUGCUCUUUGCCGUGGCG
Pno-1-1	GCUGGUUGGCGUGCAAAUGCGCAGCCGCAAGAUUUCGCUACGCCAAUCUGAGUGACGCGCGUUUCUACCGCAGCGAUCUAAACAAUACCGAUUGGACAGGCUCGAAGCUCGCCUCGGCGGCGUUUACCGAGGUGAAGCUCACCGGCGCCCGCUUUGUCGACGCUCGCACGCUAGGGCUUA
Bce-1-1	GCUGGCUGAGUUGCAAGGGCGCCACGGCAAAUUUCCGGUAUGCCAAUGCGAGUGAUGCCCGAUUCUGCCGGAGCGAUUUGAACAAUACCGACUGGACCGGCGCCAAGCUUGCAUCGACCGUCUUUACGGAGGUCAGGCUGACGGGAGCCCGUUUCACGGACAUCCGUGCGCUGGGGCUCA
env-82	UCUCCGCAUAUGUCUACGCGGUUGCGCGCAAUCACCUGAUAAACCGUGCGAAGCGCGAGCGUAUCAUCUCCUUCACGCUGGUCGCGGAUCUAGAGGGGGGCUUGCCGGAUCACGAGCCGAUCCAGCCGGAACGACAGCUGGAUGCGCGCGAUCAGCUUCGGCGAACACAAGAAGGCCUCG
env-85	....
env-86	UUGGGACCGUGCUGGUCGCCAUCGUCAGCGUCGUGGUGUUCCGGCAGACGCUGGACGCCAUGGCCAUUCUUGGGAUCGCGAUGAUUGUCGGCGGCGUCGUCAUCAUGAACGCGUUCUCAAGCGUCUCUCACUGAGGGCUGCGCCGGUUUCUGUGCCGGGCUCGGAGCGGACUGAUCGCUU
env-87	AGCUGGACAUACCGGCGAUGGCGGGAAUGGGCUUGAUCGUGACUGGUGUCAUAGUGAUGAAUGUGUUCUCGCAGACUGCUGCUCACUGACCGGUCGGGAUCGGCAGCUUUCGGGAAUAGCGGCGUAGCGGUUGAGCGGCGGGAAUGGGGCGCAUUGCAGCCAACGAACUGCCGGCAGUCU
env-89	....
env-90	....
env-91	....
env-92	GUUGCCUACGCCAUUUGGUCGGGCUGCGGUAUUGUGCUGAUCGCAGCAGUAGCAUGGAUAAUUCAGGGCCAGAAGCUCGACGUUCCAGCAAUGGCUGGAAUGGGAUUGAUCGUUGCCGGUGUCAUCGUAAUGAACGCGUUUUCAAAGACCGCUGCACACUAAACCAGAAAGCGGAUCGUC
env-95	....
env-96	....
env-97	CGAUACCGCCGCCAUCAUUGGCAUGAGCUUCAUUGUCGCCGGCGUGGUCAUCCUUAACGGCUUUUCCGACGCGGUGUCGCAUUGAACGAUUUCCGCUAUCCACCCCAUUCGCAGCCAUUGAGCGCCCGCAGCAGGACCAGUCAGGAACGAUCCACAUGUACGCCUGUUGAGAUUUCUGCU
env-98	CGAUACCGCCGCCAUCAUUGGCAUGAGCUUCAUUGUCGCCGGCGUGGUCAUCCUUAACGGCUUUUCCGACGCGGUGUCGCAUUGAACGAUUUCCGCUAUCCACCCCAUUCGCAGCCAUUGAGCGCCCGCAGCAGGACCAGUCAGGAACGAUCCACAUGUACGCCUGUUGAGAUUUCUGCU
env-99	....
env-100	CGAUACCGCCGCCAUCAUUGGCAUGAGCUUCAUUGUCGCCGGCGUGGUCAUCCUUAACGGCUUUUCCGACGCGGUGUCGCAUUGAACGAUUUCCGCUAUCCACCCCAUUCGCAGCCAUUGAGCGCCCGCAGCAGGACCAGUCAGGAACGAUCCACAUGUACGCCUGUUGAGAUUUCUGCU
env-101	....
env-102	CGAUACCGCCGCCAUCAUUGGCAUGAGCUUCAUUGUCGCCGGCGUGGUCAUCCUUAACGGCUUUUCCGACGCGGUGUCGCAUU..
env-103	....
env-104	CGAUACCGCCGCCAUCAUUGGCAUGAGCUUCAUUGUCGCCGGCGUGGUCAUCCUUAACGGCUUUUCCGACGCGGUGUCGCAUUGAACGAUUUCCGCUAUCCACCCCAUUCGCAGCCAUUGAGCGCCCGCAGCAGGACCAGUCAGGAACGAUCCACAUGUACGCCUGUUGAGAUUUCUGCU
env-118	CGAUACCGCCGCCAUCAUUGGCAUGAGCUUCAUUGUCGCCGGCGUGGUCAUCCUUAACGGCUUUUCCGACGCGGUGUCGCAUUGAACGAUUUCCGCUAUCCACCCCAUUCGCAGCCAUUGAGCGCCCGCAGCAGGACCAGUCAGGAACG............................
env-119	....
env-120	CGAUACCGCCGCCAUCAUUGGCAUGAGCUUCAUUGUCGCCGGCGUGGUCAUCCUUAACGGCUUUUCCGACGCGGUGUCGCAUUGAACGAUUUCCGCUAUCCACCCCAUUCGCAGCCAUUGAGCGCCCGCAGCAGGACCAGUCAGGAACGAUCCACAUGUACGCCUGUUGAGAUUUCUGCU
env-121	CGAUACCGCCGCCAUCAUUGGCAUGAGCUUCAUUGUCGCCGGCGUGGUCAUCCUUAACGGCUUUUCCGACGCGGUGUCGCAUUGAACGAUUUCCGCUAUCCACCCCAUUCGCAGCCAUUGAGCGCCCGCAGCAGGACCAGUCAGGAACGAUCCACAUGUACGCCUGUUGAGAUUUCUGCU
Ssp-9-1	CUUGCGAACUGGCCGUUCACGCUUCUGGUCAUCCUGCCCGUCAACAAGCGCCUCAUGGCGUUGGACCCUCGUCACGCCAACUCUGAAAGCCGCGGGAUGCUCGUCCAAUGGGGCAAACUCCACAAUGUCAGAAGCGUGUUGGGAGCCAUCGCCAUGCUCCUCUUCGCGGCGGCGUUCGCA
Ssp-2-1	CUUGCGAACUGGCCCUUCACGCUGUGGGCCAUCUUCCCCGUCAAUAAGCGGCUCAUGGCCCUGAACCCACGUCUCGCCGACUUCGAAAGUCGCGCGAUGCUCGUCCAGUGGGGCCGGCUUCAUGGUGUCAGAAGUUUUCUGGGAGCCGCAGCCAUGCUUUUGUUUGCCUUGCCAUUCAUG
env-122	GACCUGCCCGCCAUGCUGGGCAUGGCGCUGAUCGUCGCGGGCGUCGUCGUCAUGAACGCCUUUUCCGCCUCAUCGGCGCACUGAGUUCGCACUCAGAACGAAGCCUGAAGCAGCACGGCCGGGCCAUGGAGCUUGUAGCGCACGCGGCCGUUCCAGCGCUCCUUGUCGAUGCCCAGGCGG
Npa-1-1	GACCUGCCCGCCAUGCUGGGCAUGGCGCUGAUCGUCGCGGGCGUCGUCGUCAUGAACGCCUUUUCCGCCUCAUCGGCGCACUGAGUUCGCACUCAGAACGAAGCCUGAAGCAGCACGGCCGGGCCAUGGAGCUUGUAGCGCACGCGGCCGUUCCAGCGCUCCUUGUCGAUGCCCAGGCGG
Pba-1-1	CUGGCUGGCGUGCAAAGGCGCCGGAGCGCGGUUUCGCUAUGCCAACGCGAGCGACGCGCGCUUCUGCCAGAGCGACCUGAACAAUACCGACUGGACUGGAUCGAAACUCGCUUCGGCGACAUUCACCGGCGUGAAACUGACGGGCGCGCGUUUUGUCGACGCGCGCACGCUCGGGCUGGU
env-124	GGCGUCUGCCGUCGCGACGCUGGUAUGGCACGUGGAAUAUCUUCACGCGCUCGCGCCGAUCGUCGUGUUGGCCGCUUGCGU..
env-125	GGCGUCUGCCGUCGCGACGCUGGUAUGGCACGUGGAAUAUCUUCACGCGCUCGCGCCGAUCGUCGUGUUGGCCGCUUGCGUGGCGAUCGUCGCGAUCAACCGUCGUCGCGGAUGAUACAGGGGCGGCCGCCGAUCAGGUCUUCCCGCAAACGAGGGGCAGCGAGAGGGCCGGGGCUUUCC
env-126	AAGCUGGACUUACCGGCAAUGGCGGGAAUGGGUUUGAUCGUGACUGGUGUCAUCGUGAUGAACGUGUUCUCGCAGACUGCUGCUCACUGAUCGGUCGGGGUCGGCAGCUUCCGGGGAUUGCGGUGCAUCGGCUGAGCGACCGGAAUGGGCGCGUAACAGACAUUUCCCAAUCUGUCGGCG
env-127	CUAAAUAGGUCUUAAAGGGGCAAUUCCCCCAUAAGGGAGCCAAUUUAAUUCCCUGGGCCGCCCCUUUUAAAGGCCGGGACUGGAAAAACCUGGGGUUUCCAAAUAAAACCCUUUGGACCAAUCCCCCUGCUCCCGGUGGGAAAAAAGAAAAAGCCCCCCCAUCCCCUUUCAAAAUUGG
env-128	....
Ssp-3-2	....
env-129	....
Ssp-6-1	GGCGAUUUCGUAUCGGGUGGGCGCCGCGCCCUCGACGGUGCGCGAGACGCUGAGGCGGGCAGCGGUCGCGGGUCUGGCUUGGCCGCUGGGCGCGGAUGUCAGCGAUGCCGUUCUGGAGGCGGCGCUUUACCGCGCGGCUGGCACCAAGACCGGCCAUCGCCGCUGCCCUGAACCGGACUG
Peb-1-1	CAAACCGUCCUUGAACACUGCGAACUCGUGAGAAGCCGCUGGCUGGCGUGCAAAGGCGCGGGCGCCAGGUUUCGAUACGCCAAUGCGAGUGACGCCCGCUUCUGCCAAAGCGAUCUGAACAAUACCGACUGGACCGGCUCGAAACUCGCAUCGGCGACCUUUACCGGCGUGAAGCUCACG
env-130	CGACCUGCCCGCCAUGCUGGGCAUGGCGCUGAUCGUCGCGGGCGUCGUCGUCAUGAACGUCUUUUCCGCCUCAUCCGCGCACUGAGUUCGCACUCAGAACGAAGCCUGAAGCAGCACGGCGGGGCCAUGGAGCUUGUAGCGCACGCGGCCGUUCCAGCGCUCCUUGUCGAUGCCCAGGCG
env-131	CGAUCUGCCCGCCAUGCUGGGCAUGGCGCUGAUCGUUGCGGGCGUCGUCGUCAUGAACGUCUUUUCCGCCUCAUCCGCGCACUGAGUUCGCACUCAGAACGAAGCCUGAAGCAGCACGGCCGGGCCAUGGAGCUUGUAGCGCACGCGGCCGUUCCAGCGCUCCUUGUCGAUGCCCAGGCG
env-132	CGAUCUGCCUGCUAUGCUGGGCAUGGCGCUGAUCGUUGCGGGCGUCGUCGUCAUGAACGUCUUUUCCGCCUCAUCCGCGCACUGAGUUCGCACUCAGAACGAAGCCUGAAGCAGCACGGCCGGGCCAUGGAGCUUGUAGCGCACGCGGCCGUUCCAGCGCUCCUUGUCGAUGCCCAGGCG
Sxe-1-1	GAUGCUCGCCAACUGGCCAUUCACCUUGUUUAUCAUCUUCCCAGUCAACAAGCGUCUUCUCGCCAUUCGCCCGGUCGAGGCCGAUCGCGAGACACGCGGACUGCUGCGCAAAUGGGGGCACUGCAUAACGUGCGAAGCGCGCUGGGCGCCGCUGCGGUGCUCCUUUUCGCAUGGGCGGC
Ssp-23-1	GAAGCUCGACACCCCCGCCAUGGUCGGCAUGGCGCUGAUCAUAGGCGGCGUGGUGGUGAUGAACGGCUUUUCGAACAGCACCGCACACUGAUCCGGACAGGACCUACAGCUGCGGUCGCGCGCUACGGAGCGACCGGCCCCUAGUCGGGCGCCAUCUGGCAAUUGGGGCCGACCCACU
Esp-2-1	UUGAUGCUCCCGCCAUGCUGGGCAUGGGUUUGAUAGUAGCUG..
Esp-1-1	UUGAUGCUCCCGCCAUGCUGGGCAUGGGUUUGAUAGUAGCUGGUGUCGUAGUGAUGAACGUAUUUUCGAAGGUGCCGGCUCAUUAAGAUUAUGAAAUUCCGCUUGCUUACAAGAGCGUCGCAAGCGGUUAGUCGGCAAACCACCCUAAUCGGACAUUCGCGGUCAUCAUUACCGGAGCAA
env-133	....
Efl-1-1	UUGAUGCUCCCGCCAUGCUGGGCAUGGGGUUGAUAGUAGCUGGCGUCGUGGUGAUGAACGUAUUUUCGAAGGUGUCGGCUCACUAAAGUGCGACUGUCCGCUUGCGACGCUCUUUGUCAUCACGCGGACAGUCGGCAAACCACCCCAUCUCGGUCAUUCCGCUGACAAUGAUGUUAGCCC
env-134	UCGAUGCUCCCGCUAUGGUGGGUAUGGGAUUGAUUGUAGCUGGUGUCGUGGUGAUGAACGUAUUUUCGAAGGUGCCGGCUCAUUAAAGUGCGACUGUCCGCUUGCUGACAAAGCAGCGUCGCAAGUGGACAGUCGGCAUCCACUCCCAUUCACGACAUAGCGAUCCCGCGCAAACUGCGU
env-135	CGAUGCUCCCGCUAUGGUGGGUAUGGGAUUGAUUGUAGCUGGUGUCGUGGUGAUGAACGUAUUUUCGAAGGUGUCGGCUCAUUAAAGUGCGACUG..
env-136	CGAUGCUCCCGCUAUGGUGGGUAUGGGAUUGAUUGUAGCUGGUGUCGUGGUGAUGAACGUAUUUUCGAAGGUGUCGGCUCAUUAAAGUGCGACUGUCCGCUUG..
env-137	CGAUGCUCCCGCUAUGGUGGGUAUGGGAUUGAUUGUAGCUGGUGUCGUGGUGAUGAACGUAUUUUCGAAGGUGUCGGCUCAUUAAAGUGCGACUGUCCGCUUGGUGACAAAGCAGCGUCGCAAGUGGACAGUCGGCAUCCACCCACAUUCACGACAUAGCGAUCCUGCGCAAACUGCGUC
env-138	CGAUGCUCCCGCUAUGGUGGGUAUGGGAUUGAUUGUAGCUGGUGUCGUGGUGAUGAACGUAUUUUCGAAGGUGUCGGCUCAUUAAAGUGCGACUGUCCGCUUGGUGACAAAGCAGCGUCGCAAGUGGACAGUCGGCAUCCACCC............................
env-139	CGAUGCUCCCGCUAUGGUGGGUAUGGGAUUGAUUGUAGCUGGUGUCGUGGUGAUGAACGUAUUUUCGAAGGUGUCGGCUCAUUAAAGUGCGACUGUCCGCUUGGUGACAAAGCAGCGUCGCAAGUGGACAGUCGGCAUCCACCCACAUUCACGACAUAGCGAUCCUGCGCAAACUGCGUC
env-155	....
env-156	GGUAUUUAUCGACCCAAUGGUUGUGGAUCGUUGGAAGCAUCGCACUUCUUGGAAACUGGCCGUUUACCAUCAUAGCAAUCAUGCCGACGAACAAACGACUUCAGGCCGUUCAUCCAGAACAGGCAGGCUCCGACACAAGGGCCCAGCUUGUGGCCUGGGGGCGCCUUCACGAUAUAAGAA
env-157	....
env-158	AUCGCACUUCUUGGUAACUGGCCGUUUACCAUCAUAGCAAUUAUGCCGACGAACAAGCGACUUCAGGCCGUUUAUCCAGAACAGGCAGGCUCCGACACAAGGGCCCAGCUUGUGGCCUGGGGGCGCCUUCACGAUAUAAGAAGCCUGCUCGGGCUGGCCUCGACAGUGCUUUUCGCGAUA
env-160	....
env-161	UUCGCGUCUCCCCGGCUCAUGCCGAGGAACCCUAAAUGUCGCUUUUCUAUAUCAUUGCACUGGUGUCUGCCGCGUUGUUUGCCGGUGCGGCAAGUUAUAUCAGUCUGGUCGAACACCCGGCGCGCCUGUUGCUGGAAGACAGGCCGCUACUCACACAGUGGCAAUCAAGCUACGCGAAAG
env-162	UCGCACUUCUUGGAAACUGGCCGUUUACCAUCAUAGCGAUCAUGCCGACGAACAAGCGACUUCAGGCCGUUCAACCGGAACAGGCAGGCCCCGACACAAGGGCCCAGCUUGUGGCCUGGGGGCGCCUUCACGAUAUAAGAAGCCUGCUCGGGCUGGCCUCGACAGUGCUUUUCGCGAUAG
env-163	UCGCACUUCUUGG..
env-165	UCGCACUUCUUGGAAACUGGCCGUUUACCAUCAUAGCGAUCAUGCCGACGAACAAGCGACUUCAGGCCGUUCAACCGGAACAGGCAGGCCCCGACACAAGGGCCCAGCUUGUGGCCUGGGGGCGCCUUCACGAUAUAAGAAGCCUGCUCGGGCUGGCCUCGACAGUGCUUUUCGCGAUAG
env-166	....
env-168	UCGCACUUCUUGGUAACUGGCCGUUUACCAUCAUAGCAAUCAUGCCGACGAACAAACGACUUCAGGCCGUUCAUCCAGAACAGGCAGGCCCCGACAUAAGGGCCCAACUUGUGUUCUGGGGGCGCCUUCACGAUAUAAGAAGCCUGCUCGGUCUGGCCUCGACAGUGCUUUUCGCGAUAG
env-169	....
	....
	....
	oooooooooooooooooooooooooooooooooooooooooooooooooooooooooooooooooooooooooooooooooooooooooooooooooo

env-170 CGCACUUCUUGGNAACUGGCCGUUUACCAUCAUAGCAAUCAUGCCGACGAACAANCGACUUCAGGCCGUUCAUUCGGAACAGGCAGGCCCCGACAUAAGGGCCCAACUUGUGUCCUGGGGGCGCCUUCACGAUAUAAGAAGCCUGCUCGGGCUGGCCUCGACAGUGCUUUUCGCGAUAGC
env-171 CGCACUUCUUGGAAACUGGCCGUUUACCAUCAUAGCAAUCAUGCCGACGAACAAACGACUUCAGGCCGUUCAUUCGGAACAGGCAGGCCCCGACAUAAGGGCCCAACUUGUGUCCUGGGGGCGCCUUCACGAUAUAAGAAGCCUGCUCGGGCUGGCCUCGGCAGUGCUUUUCGCGAUAGC
env-172 UCGCACUUCUUGGUAACUGGCCGUUUACCAUCAUAGCAAUCAUGCCGACGAACAAACGACUUCAGGCCGUUCAUCCAGAACAGGCAGGCCCCGACAUAAGGGCCCAACUUGUGUUCUGGGGGCGCCUUCACGAUAUAAGAAGCCUGCUCGGUCUGGCCUCGACAGUGCUUUUCGCGAUAG
env-173 CGCACUUCUUGGUAACUGGCCGUUUACCAUCAUAGCAAUCAUGCCGACGAACAAACGACUUCAGGCCGUUCAUCCAGAACAGGCAGGCCCNGACAUNAGGGCNCANCUUGUGNNCUGGGGGCGCCUUCACGAUAUAAGAAGCCUGCUCGGNCUGGCCUCGACAGUGCUUUUCGCGAUAGC
env-174 ....
env-175 UCGCACUUCUUGGUAACUGGCCGUUUACCAUCAUAGCAAUCAUGCCGACGAACAAACGACUUCAGGCCGUUCAUCCAGAACAGGCAGGCCCCGACAUAAGGGCCCAACUUGUGUUCUGGGGGCGCCUUCACGAUAUAAGAAGCCUGCUCGGUCUGGCCUCGACAGUGCUUUUCGCGAUAG
Esp-3-1 UCGCACUUCUUGGAAACUGGCCGUUUACCAUCAUAGCAAUCAUGCCGACGAACAAACGACUUCAGGCCGUUCAUUCGGAACAGGCAGGCCCCGACAUAAGGGCCCAACUUGUGUCCUGGGGGCGCCUUCACGAUAUAAGAAGCCUGCUCGGGCUGGCCUCGACAGUGCUUUUCGCGAUAG
env-176 AUCGCACUUCUUGGCAACUGGCCGUUUACCAUCAUAGCAAUCAUGCCGACGAACAAACGACUUCAGGCCGUUCAUCCAGAACAGGCAGGCCCUGACAUUAGGGCUCAGCUUGUGGCCUGGGGGCGCCUUCACGAUAUAAGAAGCCUGCUCGGGCUGGCCUCCACAGUGCUUUUCGCGAUA
env-179 AUCGCACUUCUUGGCAACUGGCCGUUUACCAUCAUAGCAAUCAUGCCGACGAACAAACGACUUCAGGCCGUUCAUCCAGAACAGGCAGGCCCUGACAUUAGGGCUCAGCUUGUGGCCUGGGGGCGCCUUCACGAUAUAAGAAGCCUGCUCGGGCUGGCCUCCACAGUGCUUUUCGCGAUA
env-180 ....
env-181 CUACUCACACAGUGGCAAUCAAGCUACGCGAAAGCGCUCCCGA...
env-182 ....
env-183 UGCCGAGGAACCCUGAUGUCGCUUUUCUAUAUCAUUGCACUGGCGUCUGCCGCGUUGUUUGCCGGUGCGGCAAGUUAUAUCAGUCUGGUCGAACACCCGGCCCGCCUGUUGCUGGAAGACAGGCCGCUACUUACACAGUGGCAAUCAAGCUACGCGAAAGCGCUACCGAUACAGUCCGGC
env-184 ....
env-185 UGCCGAGGAACCCUGAUGUCGCUUUUCUAUAUCAUUGCACUGGUGUCCGCCGCCUUGUUUGCCGGUGCGGCAGCUUAUAUCAGUCUGGUCGAACACCCGGCCCGCCUGUUGCUGGAAGA...
env-186 ....
env-187 GUGGCAAUCAAGCUACGCGAAAGCGCUCCCGAUACAGUCCGGCUUGGCUAUUAUCGGCGGUGUAUGCGGAUUGGCGGCUUGGUAUUUAUCGACCCAAUGGUUGUGGAUCGCUGGAAGCAUCGCACUUCUUGGUAACUGGCCGUUUACCAUCAUAGCAAUCAUGCCGACGAACAAACGACU
env-192 ....
env-194 GUGGCAAUCAAGCUACGCGAAAGCGCUCCCGAUACAGUCCGGCUUGGCUAUUAUCGGCGGUGUAUGCGGAUUGGCGGCUUGGUAUUUAUCGACCCAAUGGUUGUGGAUCGCUGGAAGCAUCGCACUUCUUGGUAACUGGCCGUUUACCAUCAUAGCAAUCAUGCCGACGAACAAACGACU
env-195 GUGGCAAUCAAGCUACGCGAAAGCGCUCCCGAUACAGUCCGGCUUGGCUGUUAUCGGCGGUGUAUGCGGAUUGGCGGCUUGGUAUUUAUCGACCCAAUGGUUGUGGAUCGCUGGAAGCAUCGCACUUCUUGGUAACUGGCCGUUUACCAUCAUAGCAAUCAUGCCGACGAACAAACGACU
env-196 UGCCGAGGAACCCUGAUGUCGCUUUUCUAUAUCAUUGCA...
Ssp-1-1 UUGCGAACUGGCCAUUCACGCUUCUGGUCAUCCUGCCCGUCAACAAGCGCCUUAUGGCCAUGGAUUCACAUCACGCCGACUUGGAAAGUCGCCGGAUGCUCAUCCAAUGGGGCAAACUUCACAAUAUCAGAAGCAUCUUGGGAGCCGUUGCCAUGCUUCUUCUUCGCCGCGUCUUUCGUAA
Nsp-1-1 UCGAUUCCGCCGCCAUGAUCGGCAUGGGCUUCAUCGUUGUUGGCGUGGUAAUCAUGAACGGCUUUUCCAGCGCGGUGUCGCACUGAGCGAUGUCCGCUUCCCACCCAACAGUCGCCAUCAGCGAGGGCGCGCCGUGAUCCUAGAAGCGGCCAUUGCGCGCCACGGCCCUGAUCGGACGCU
env-197 UCGAUACCGCCGCUAUGAUCGGCAUGGGCUUCAUCGUCGUGGGCGUGGUCAUUAUGAACGGCUUUUCCAGCUCGGUGUCGCAUUGAGCGAACAUCCACUUUCGACCCUUAGCGGCCGCCGGGCGGAAACGGAUUGGGAGUUCAGGCCGCCGUCUGCCCAUAGGCCAGUUUCCGGAACGG
Ssp-15-1 GCCAGAAGCUCGAUACGCCCGCCAUGGUCGGCAUGGCGCUGAUCAUAUGCGGCGUGGUGGUGAUGAACGGCUUUUCGAACAGCACCGCACACUGAUCGCCGGAUUCGACGGAUGCCUUCGGGUCCGAUGAGAUUGCUGCGCUUCGCGGCAAGCUCCUUUUCCGACCAGAAGCCCCGGAAG
Ssp-18-1 UGUUUCAGGGCCAGAAGCUCGAUACGCCCGCCAUGGUCGGCAUGGCGCUGAUCAUAUGCGGCGUGGUGGUGAUGAACGGCUUUUCGAACAGCACCGCACACUGAUCGCCGGAUUCGACGGAUGCCUUCGGGUCCGAUGAGAUUGCUGCGCUUCGCGGCAAGCUCCUUUUCCGACCAGAAG
env-198 ....
Ssp-16-1 AGAAGCUCGAUACGCCCGCCAUGGUCGGCAUGGCGCUGAUCAUAUGCGGCGUGGUGGUGAUGAACGGCUUUUCGAACAGCACCGCACACUGAUCGCCGGAUUCGACGGAUGCCUUCGGGUCCGAUGAGAUUGCUGCGCUUCGCGGCAAGCUCCUUUUCCGACCAGAAGCCCCGGAAGUCC
env-199 ....
env-200 ....
Sdo-1-1 UGUCCUGCUCGCCAACUGGCCGUUCACGCUGCUUGCGAUCAUGCCGACCAACAAGCAGCUUAAGAUGAUCCGGCCCGAGAAAGCCGGGCCGCGACCCGUGCGCUAUUGGUUUCGUGGGGAAAGCUGCACGCGGCAGGAGCGUGCUGGGCGUCGUGUCGACGGCCUUGUUUGCAGCCGC
env-201 UAUCCUGCUCGCCAACUGGCCGUUCACCCUGCUUGCGAUCAUGCCGACCAACAAGCGGCUCAAGAUGAUCGGGCCCGAGGAAGCCGGGCCGGCGACCCGUGCACUGUUGGUAUCGUGGGGAAAGCUGCACGCGAUCAGGAGCGUGCUGGGCGUCGUGUCGACGGUCUUGUUUGCAGCCGC
Bro-1-1 UGCUCCUGCUCGCAAACUGGCCGUUCACGAUGUUGGUUAUCAUGCCCGUAAACAAGCGCUUGAUGGCGAUGACGCCGCAGGAAGCGGGGGCGAGAAGCCGUGACCUACUGCGUCGCUGGGGUAACCUGCACGGCGUCCGGAGCGUACUCGGCUCCGCCGCUUCCCUCCUGUUCGCCUGCG
Bro-1-2 UGCUCCUGCUCGCAAACUGGCCGUUCACGAUGUUGGUUAUCAUGCCCGUAAACAAGCGCUUGAUGGCGAUGACGCCGCAGGAAGCGGGGGCGAGAAGCCGUGACCUACUGCGUCGCUGGGGUAACCUGCACGGCGUCCGGAGCGUGCUCGGCUCCGCCGCUUCCCUCCUGUUCGCCUGCG
env-202 ....
env-203 GUAUCAUCCUGUUUGCGAACUGGCCGUUCACGUUGCUGGUGAUCAUGUCGGUAAAUCGUCGUUUGAAGACCAUCGAGCCCGCCGACGCCGGGGCACAGUGCCGUGUACUGUUGCAGCGGUGGGGAACCUUGCACAGCGUCAGGAGUGUACUCGGAGCACUGGCAGCGAUCCUAUUCGCGU
env-204 ....
env-205 ....
env-206 GAUGCCGAGGAAUGUUCAUGUUUCAGCUCUCGAUAGCUGCCCUUGUUUUCGCCGCGCUGUUUGCCGGCGCUGCACUCUAUAUAAGCCUCGUCGAACAUCCGGCGCGUGUCGGACUAGCAGACGGUCCUCUGCUGAUGCAGUGGCAGCCCAGCUACAAGCGGGCGCUUCCAAUCCAGUCUG
env-208 GAUG..
env-209 GAUGCCGAGGAAUGUUCAUGUUUCAGCUCU..
env-210 ....
env-211 GCUGUUGCUGGCCAACUGGCCGUUCACUCUGGCCAUCAUCUUUCCCGUCAACAAACGGUUGAUGGCGAUGCGCGAAGCCGAUGCGGGCGCAGAGAGCCGGGCGCUGCUGGUGCAGUGGGGCAAGCUGCACAAUGUCAGAAGCGCACUGGGCGCGGCUACGACACUGGUCUUUGCAUGGGC
env-212 ....
env-213 CUUAUUGCUCGCCAAUUGGCCGCUCACGCUGUUCGUCAUCAUGCCAGUCAACAAACGCUUGAUGGCGAUGCAGGAAGGUGAGGCGGGCGCCGGGAGCCGGGCGAUGCUAGUGCAGUGGGGAAAGCUGCACAACGUCAGGAGUGUAUUGGGCGCAGCCACGGCGCUGCUCUUCGCAUUGGC
env-214 CCUGUUGCUCGCCAACUGGCCAUUCACACUGCUCGUUAUGAUGCCAAUCAACAAGCGGCUGAUGGCGAUGCACGAAUGUGAGGCGGGUGCUGAAAGCCGGGCGAUGCUGGUAAAGUGGGGAAAGCUGCACAACAUCCGGAGCGUAUUGGGCGCAGCCACGAUGCUGCUUUAUGGUGGGC
env-223 ....
env-224 CCUGUUGCUCGCCAACUGGCCAUUCACACUGCUCGUUAUGAUGCCAAUCAACAAGCGGCUGAUGGCGAUGCACGAAUGUGAGGCGGGUGCUGAAAGCCGGGCGAUGCUGGUAAAGUGGGGAAAGCUGCACAACAUCCGGAGCGUAUUGGGCGCAGCCACGAUGCUGCUUUAUGGUGGGC
env-225 CCUGUUACUCGCCAACUGGCCGUUUACGCUGUUCGUUAUCAUGCCGGUCAACAAACGCCUGAUGGCGAUGCCGGAACGUGAGGCGGGCGGUGGAAGUCGAGCGAUGCUGGUACAGUGGGGAAAGCUGCACAACGUCAGGAGCGCACUGGGAGCAGCCACGGCGCUGCUCUUCGCAUGGGC
env-226 CCUGUUGCUUGCCAACUGGCCGUUCACGCUAUUGGUCAUCCUGCCAGUCAACAAACGCUUGAUGGCGAUGCCGGAACGUGAGGCGGGCGCUGGAAGUCGCGCGAUGCUGAUACAAUGGGGCAAGCUGCACAACGUCAGGAGCGCACUGGGAGCAGCCGCAGCGCUGUUCUUUGCUUGGGG
Ssp-8-2 CCUGUUGCUCGCCAAUUGGCCGUUCACGCUGUUCGUCAUCAUGCCGGUCAACAAACGCCUGAUGGCGAUGCCGGAAAGUGAGGCGGGUGCCGAAAGCCGUGCGAUGCUGGUGCAAUGGGGAAACCUGCACAACGUCAGGAGCGCGCUGGGCGCAGCAAUGGCGCUGCUCUUUGCGUGGGC
env-227 CCUGUUGCUCGCCAACUGGCCGUUCACGCUAUUCGUCAUCAUGCCCGUCAACAAACGCUUGAUGGCGACGCCGGUAAGCGAGGCGGGCGCUGAAAGCCGCGCGAUGCUGGUUCAGUGGGGAAAGCUGCACAACCUCAGGAGCGCACUGGGCGCAGCCACGGCGCUGCUCUUUGUCUGGGC
env-228 CCUGUUGCUCGCCAACUGGCCGAUCACGCUAUUCGUCAUCAUGCCCGUCAACAAACGCUUGAUGGCGAUGCCGGUAAGCGAGGCGGGCGCUGAAAGCCGCGCGAUGCUGGUUCAGUGGGGA..
env-229 CCUGUUGCUCGCCAACUGGCCGAUCACGCUAUUCGUCAUCAUGCCCGUCAACAAACGCUUGAUGGCGAUGCCGGUAAGCGAGGCGGGCGCUGAAAGCCGCGCGAUGCUGGUUCAGUGGGGAAAGCUGCACAACGUCAGGAGCGCACUGGGCGCAGCCACAGCGCUGCUCUUUGUCUGGGC
env-235 CCUGUUGCUCGCCAACUGGCCGAUCACGCUAUUCGUCAUCAUGCCCGUCAACAAACGCUUGAUGGCGAUGCCGGUAAGCGAGGCGGGCGCUGAAAGCCGCGCGAUGCUGGUUCAGUGGGGAAAGCUGCACAACGUCAGGAGCGCACUGGGCGCAGCCACAGCGCUGCUCUUUGUCUGGGC
env-236 CCUGUUGCUCGCCAACUGGCCGAUCACGCUAUUCGUCAUCAUGCCCGUCAACAAACGCUUGAUGGCGAUGCCGGUAAGCGAGGCGGGCGCUGAAAGCCGCGCGAUGCUGGUUCAGUGGGGAAAGCUGCACAACGUCAGGAGCGCACUGGGCGCAGCCACAGCGCUGCUCUUUGUCUGGGC
env-237 CCUGUUGCUCGCCAACUGGCCGAUCACGCUAUUCGUCAUCAUGCCCGUCAACAAACGCUUGAUGGCGAUGCCGGUAAGCGAGGCGGGCGCUGAAAGCCGCGCGAUGCUGGUUCAGUGGGGAAAGCUGCACAACGUCAGGAGCGCACUGGGCGCAGCCACAGCGCUGCUCUUUGUCUGGGC
env-238 CCUGUUGCUCGCCAACUGGCCGUUCACGCUAUUCGUCAUCAUGCCCGUCAACAAACGCUUGAUGGCGACGCCGGUAAGCGAGGCGGGCGCUGAAAGCCGCGCGAUGCUGGUUCAGUGGGGAAAGCUGCACAACCUCAGGAGCGCACUGGGCGCAGCCACGGCGCUGCUCUUUGUCUGGGC
env-239 CCUGUUGCUCGCCAACUGGCCGUUCACGCUAUUCGUCAUCAUGCCCGUCAACAAACGCUUGAUGGCGACGCCGGUAAGCGAGGCGGGCGCUGAAAGCCGCGCGAUGCUGGUUCAGUGGGGAAAGCUGCACAACCUCAGGAGCGCACUGGGCGCAGCCACGGCGCUGCUCUUUGUCUGGGC
env-240 ....
env-241 CACGUCUCGGAUUGUCGGAUGGCCCUUUGCUGGCGCAAUGGCAGCCCAGCUACAAGCGAGCGCUACCGAUCCAAUCCGGGUUGGCAAUUGCCGGGGGAGCGGCGGGACUGAUCGCCGGGUAUCUUUCGGCUGAUUGGCGGUGGUUUGCCGGGAGCAUCCUGUUGCUCGCCAACUGGCCGA
env-242 CCUGUUGCUCGCCAACUGGCCGUUCACGCUAU..
env-243 ....
env-244 CCUGUUACUCGCCAACUGGCCAUUCACGCUAUUCGUCAUCAUGCCAGUCAACAAACGCCUGAUGGCGAUGCAGGAACGUGAGGCCGGCGCUGAAAGCCGAGCGAUGCUGGUGCGGUGGGGAAAGCUGCACAACGUCAGAAGCGCACUGGGCGCAGCCACGACGCUGCUCUUUGCGUGGGC
Swi-1-1 CCUUUUGCUCGCCAACUGGCCGUUCACGCUAAUUUUCAUCAUGCCCGUCAACAAGUGCUUGAUGGCGAUCCCGCAACGUGAGGCGGGUGCAGGAAAGCCGAGCGAUGCUGGUGCAGUGGGGAAAGUUGCACAACUUCAGGAGCGCACUGGGCGCAGCCUCGGUGCUGUUCUUUACGUGGAC
Ssp-5-1 AUCCUGUUGCUCGCCAACUGGCCAUUCACGCUCCUCGCUAUCAUGCCGGUCAACAAACGCUUGAUGGCGAUGCAGGUUGGUGAGGCGGGCCCUGGAAGCCGAGCGAUGCUGGUGCGUUGGGGAAAGCUGCACAACGUCAGGAGUGCGCUUGGAGCAGCCUCGACGCUGCUCUUCGCAUUG
env-245 ....
env-246 UUCGCGGGCGCUGUCCUGCUGCUGGCCAAUUGGCCGUUCACCCUGUUCGUGAUCAUGCCGGUCAAUAAGCGGCUGCUUGCGAUGGAGGCAAAGGAUGCAGGGCCGGAAAGCCGGCGGAUGCUGCUGGAUUGGGGCAAGCUCCACAACGUCCGAAGCGCGCUCGGGAGCGCCGCUACCCUC
env-247 ....
env-248 ....
env-249 ....
env-250 ....
env-251 ....
env-252 ....
env-253 ....
env-254 ....
env-255 CCAUGAUCGUCGGCGGGGUGCUGGUGAUGAACCUGUCCUCGACCGCGAUCGCAGCAUUAGGGACUGGGAUGCGCUCGCCACACCCCAGGCCGCGACGCUGGACCCGGCCCCCCGAACCGCGUAGACGACCGCCCCUUCACGGACUGCAUCCCAUGGCUGACAAGAAGAAGGCGGGGCUCA
env-256 ....
....
....
oooooooooooooooooooooooooooooooooooooooooooooooooooooooooooooooooooooooooooooooooooooooooooooooooooooooooooooooooooooooooooooooooooooooooooooooooooooooooooooooooooooooooooooooooooooooooooooooooo

alignment positions 721 · · · 822

```
env-1     CCAGUCAGCCGGGAAGGCGGCGGGACGGAGCGGCCUCUUUCCUUCGCGGUGAGUCCCAGGUCCACCCGCAGCGGUCGUAAGGAAGGUCCGGAGCGACGCACG
env-2     AGGCCAAGGCGGCCUUUGCGCAGGCGCAGCUUGCCCGCUCCGAGCAGCUUGCGCCGACCGGGGCGGAAUCGCGCGAACUGCUCCAGCAGCGCCGGGCCGAGC
env-4     ......................................................................................................
Ral-1-1   UGGGAACCUUAGUUUCAUCUCGAGACCGAUCUUGGAAUUGCAACCCUGCGCUCGCCAUCAUAGCUUUAAGUUAUGCGAUAUGCGGCCCAUCACCUAUUGACC
env-5     ......................................................................................................
env-6     AAGUUGAGUGGGGUAAUAAGAACUUGUGGCACAUAAUAAUACGAUGAAUUAAUAAUCCAAGCUUUAAUUCUGAUUAUUUUGGCAUUCAAAAUUCCUGGAAA
Spa-1-1   GGCCGCUCUGCAAGCGGUCGGGGGCUUGCGCGCGGUUCGGGAGGUGACGCUCUCGCCGGAGAUCGCGGGGCGCGUGACCGCCAUCCGGUUCGAGGCCGGUUC
env-7     ......................................................................................................
env-8     ......................................................................................................
env-9     ......................................................................................................
env-10    ......................................................................................................
env-11    ......................................................................................................
env-12    ACCGCCAGGCAGCGCAGGCCAAGGCCGCCUUCGCCGGUGUGCAGCUCGCGCGUUCGGAGCAGCUCGCCCCGACCGGUGCCGAGCCGCGCGAGCUGCUCCAGC
env-13    GGACCGGCAGGCGGCACAGGCGCAGGCGGCCUUUGCCAAGGCGCAACUUGCGCGAUCGGAGCAGCUUGCCCCCUCCGGCGCCGAAUCGCGUGAAUUGCUGCA
env-14    GGAUCGGCAGGCGGCGCUGGCGCAGGCGGCCUUCGCGAAGGCGCAACUGGCGCGAUCGGAGCAGCUUGCUCCUUCCGGCGCCGAAUCGCGCGAAUUGCUGCA
env-15    CCGAGGCGGCGCGCCGCCUGCGGAUGUCCAAGUCGCAGGUGAGCAAGCAGGUGGCCAGCCUCGAGCGAUCAUUGGGAGCGAGACUGCUCAACCGCACGACUC
env-16    ......................................................................................................
env-17    CCGCGGCAUUGGAGGCUGUCGGCAAUUUGCGCGCCGUGCGGGAGGUGACAUUGUCACCCGAAGUGGCCGGCCGGGUUUCGGCCCUGCAUUUCGAGGCUGGGC
env-18    UAACGGAGCGUCGAUUUUGGCGCUCCGAACGGCCCAAAUGGGGCGAUUGCUGCCGAACGAUUUUUAUCGCGCAAGGUCAGCUAUCGCCGAUCAUGUCUCCAA
env-20    UAACGGAGCGUCGAUUUUGGCGCUCCGAACGGCCCAAAUGGGGCGAUUGCUGCCGAACGAUUUUUAUCGCGCAAGGUCAGCUAUCGCCGAUCAUGUCUCCAA
env-21    UAACGGAGCGUCGAUUUUGGCGCUCCGAACGGCCCAAAUGGGGCGAUUGCUGCCGAACGAUUUUUAUCGCGCAAGGUCAGCUAUCGCCGAUCAUGUCUCCAA
env-22    UAACGGAGCGUCGAUUUUGGCGCUCCGAACGGCCCAAAUGGGGCGAUUGCUGCCGAACGAUUUUUAUCGCGCAAGGUCAGCUAUCGCCGAUCAUGUCUCCAA
env-23    UAACGGAGCGUCGAUUUUGGCGCUCCGAACGGCCCAAAUGGGGCGAUUGCUGCCGAACGAUUUUUAUCGCGCAAGGUCAGCUAUCGCCGAUCAUGUCUCCAA
env-24    UAACGGAGCGUCGAUUUUGGCGCUCCGAACGGCCCAAAUGGGGCGAUUGCUGCCGAACGAUUUUUAUCGCGCAAGGUCAGCUAUCGCCGAUCAUGUCUCCAA
env-25    ......................................................................................................
env-26    UAACGGAGCGUCGAUUUUGGCGCUCCGAACGGCCCAAAUGGGGCGAUUGCUGCCGAACGAUUUUUAUCGCGCAAGGUCAGCUAUCGCCGAUCAUGUCUCCAA
Cpa-1-1   AGCUUUCACGACUCACAACUGGUGGCAGCCGACCUCAAGGCGUUUUCCUUUCGAAAGCAGAGAAUCGAGCUCCUGAAUCUAUCCGAAGCAGAUUUGUCAGGU
Ysp-1-1   GAGACCCUCGGGCGUCUUGCCGUCGCCCUCGAUCUUCUUGUCGCCGAUGGGGUCGAAACCGAGCCCGACGUUGUAGAUCUUCACGAUCGAAUCAUGGUGAAG
env-31    ......................................................................................................
env-32    ......................................................................................................
env-33    ......................................................................................................
env-34    ......................................................................................................
env-35    GCGCGCCGUGGCCGCCGGAACAUUGAAAUGGCGGUGGUAUUCGCGGCUGAACUGCGAAACGCUUUCAUAUCCGACCCGCCUUGCCGCCUCGCCGAUCCUCGC
Bsp-1-1   UUGAAAGAUGGCCUGAAGAUAUCGGGUCGGUUUCAGCGUUUUGUAAAACAGGACGGUGCUUCGCCCGGAUAGCUUCCUACACCAACUCCACGCCCAUCGCGG
env-36    CGGCGCGUUGCUGGUGCAACUGUUCGACGGGCCGGAACGCGCCGACCGCCGCGCCGCGCAGGCCAGGUCGGCCUUUGCGGGCAUCCAGCUCGCCCGGUCUCG
Ssp-11-1  UGGGCCCCGGCCUUCGCCGGGGAGGUGAAUCAAUCUGGGUGGAUCAAACUACGAAGGGUCCGGGUGUGGGGGAGGGAUGCCUUGGCGCGCGAGGGCGUCGUU
Ssp-7-1   GUGGAGUUCAUGUCGUGAUUGAAUCAUUCUUUGCUCUUGUUGGCGCGAUCAUCAUCAAGGGCUUGCUAGAGUUCUUCCGGCCAUCCGACGGGGAGCGGAAAA
Nsp-2-1   AUAGCUGCCCGUAUCGGCGGCGAUCUGCGCCAGCAGUUCUUCGCUUAGGUCAUCUUGUUUGAGAUCGAGAUUGUCAACCGGCGCGCAAAACUGACCCCCUAU
Rsu-1-1   AAGGGCCGCGGCUCUUCAGCGGGUCCGGACAGGUCGGCGCCCCGGCAGGAGCGCCGACCCGGUUCAGGCUGCCGCCUCGGCGGCGUCGGGUCCUAGAAGCCC
env-37    UUCGGAGGAUCCCCGCCGCUGUCGGCCAUCCACACGCGCCAGUCGUUCUCGAUCACGUACACGUCCCAGCCGCGGGCAAGUCCGCGGGCCUUCUCUAUCGCA
Sya-3-1   GCUGGAGGCUGUCGGUUCUCUGCGUGCCGUCCGCGAAGUCACUUUGUCGCCUGAAAUUGCCGGCAGAGUUUCCGCGAUUCAUUUCGAAGCGGGGCAACCGGU
env-40    GCUGGAGGCUGUCGGUUCUCUGCGUGCCGUCCGCGAAGUCACUUUGUCGCCUGAAAUUGCCGGCAGAGUUUCCGCGAUUCAUUUCGAAGCGGGGCAACCGGU
Sya-1-1   GCUGGAGGCUGUCGGUUCUCUGCGUGCCGUGCGCGAAGUCACUUUGUCGCCCGAAAUUGCCGGCAGAGUUUCAGCGAUUCAUUUCGAAGCGGGGCAACCGGU
Sya-2-1   GCUGGAGGCUGUCGGUUCUCUGCGUGCCGUGCGCGAAGUCACUUUGUCGCCCGAAAUUGCCGGCAGAGUUUCAGCGAUUCAUUUCGAAGCGGGGCAACCGGU
Ssp-3-1   GCUGGAGGCUGUCGGUUCUCUGCGUGCUGUGCGCGAAGUCACUUUGUCGCCCGAAAUUGCCGGCAGAGUUUCAGCGAUUCAUUUCGAAGCGGGGCAACCGGU
env-42    ......................................................................................................
env-43    GACCGACAAAGAACGCGGC...................................................................................
env-44    ......................................................................................................
env-45    ......................................................................................................
env-46    ......................................................................................................
env-47    ......................................................................................................
env-48    ......................................................................................................
env-49    ......................................................................................................
env-50    ......................................................................................................
env-51    ......................................................................................................
env-52    ......................................................................................................
env-53    GGCACAAAUCUUGUCCUCGUUGAUUUGCUGAAGUCAGAUCAGGCGGACGCGGCAUCGCCAUCCCUCUCGCCGAGUGCGCCAACGACAGGCAUUGCUGGCGCU
env-54    ......................................................................................................
env-55    ......................................................................................................
env-56    ......................................................................................................
env-57    ......................................................................................................
env-58    ......................................................................................................
env-59    ACGAAUCUUGUCAUCGUCGAUUCGCUGAAAUCAGAUCAUGCGGACCCGGCAGCUCCGUGUCUUUCGCCGACUGGUCCAACGACAGGCUGUGCCGGCGCUACG
env-60    ......................................................................................................
env-61    CGACCGAUAAAGAACGCGGCCAAGCGAUCGGUAUCUGGGCUGCUGUCUUUUCAGGAGGGGUUGCGAUCGGCCCGUUGAUCGGUGGAGCUCUACUCAGCCAUU
env-62    ......................................................................................................
env-63    ......................................................................................................
env-64    ......................................................................................................
Ssp-12-1  GGAUGACGGCCGGCCCGCGACACUCGAUAAUCAGAGGCCAGCCGCGCCAGCGAUCGCGAAGUUCAGCGCAUUGCGUAGCCGCGCCGUGUCAUAUUCGGGGGC
env-65    ......................................................................................................
env-66    AGAUUGUCGUCGCCAAGCGGCGCCUCAACCACCGGCCCCCCACCGGCAGCCCCGCUGGUACCGCCUUGUCCACUGGAUCCGCUCUGGGCACCGGUGUCGCC
Ssp-10-1  GAUUGGCUUCGCCAAGCGGCGCCUCAACCACCGGACCCGCCACCGGCAGCCCCGCUGGUACCGCCCUGUCCACUGGAUCCGCUCUGGGCGCCGGUGUCACCC
Bst-1-1   CUUUCACGAUUCCCUGCUCGUCGGCGCCAGUUUGAGAGGGCUGUCGUUCCGGAAGCAGACGCUCGAAGCAUUGAAUUUCUCCGAGGCGGAUCUUGCCGAUUG
env-67    ......................................................................................................
She-1-1   AAAGUUUUGCAUCGAGGCUGACCCGGCAACAAUUUGGACCUUGCCCAUACAGAAAUCUGACGCGGAUUCACGCGCCGUUCUAUGUGUGAUAAUGUCCUUUAU
Ssp-13-1  CGAGCGAGAAGGAGACAGCCCCAUGAAGACCCGCGCCGCCGUUGCGUUCGAAGCGAAGAAGCCGCUCGAAAUCGUCGAACUCGAUCUCGAAGUCCCCAAGGC
env-68    ......................................................................................................
env-69    ......................................................................................................
Ptr-1-1   UUCACGAUUCGCUGCUCGUGGGCGCCACGCUUCGAGGUUUGUCGUUUAGAAAACAGACGCUCGAACAAUUGAAUUUCUCCGAUGCCGAUCUCGCCGAUUGCG
Ptr-1-3   UUCACGAUUCGCUGCUCGUGGGCGCCACGCUUCGAGGUUUGUCGUUUAGAAAACAGACGCUCGAACAAUUGAAUUUCUCCGAUGCCGAUCUCGCCGAUUGCG
Ssp-22-1  CGGUCGCGCGCCUCGCACUCGAUGUCGGUGAUCAGCCCCUUCGCGGGCAGCGUCGUGAAGAUCCGCUGGUGAUAAAUUUCGAUGAUGUUGACCUGCUUCUCG

          ......................................................................................................
          ......................................................................................................
          oooooooooooooooooooooooooooooooooooooooooooooooooooooooooooooooooooooooooooooooooooooooooooooooooooooo
```

```
Bsp-2-1   UUCACGAUUCGUUGCUUGUCGCUGCCGACGUGAAGGGCAUGUCGUUUCGCAGGAAGAGGCUUGAAGGGCUGAAUUUCACCGAUGCGGAUUUGUCGGACUGCG
env-70    GGCUGCGCUCGCCACUCGAUUGGGGCCGGCGAAUUGCUGUCUUUGUAGGUCGGAACCUUCGGUAAUCCUUGGACAGUUCUUCGAGUCAAAUCGUGAUCGAAC
Kvu-2-1   CGCGUGGCGGAAUUGCAGGUUGAUCAACAGACCAUUGAUGAAGACCGCAACCGCACUGUUGAACACAAUCCCCGUAUUCCGGCGGGACCGGCGCUCGUGGCG
env-71    CGAAAUCGGUGAAGUUGUCGGCCACCCAGCGAUCGUCGCUGCCGAUAACGGCGACCUUCCGUAGAACUCGCCUUCCUCGCAGCAGAUCACCCAAGCCCAACA
env-72    CGAAAUCGGUGAAGUUGUCGGCCACCCAGCGAUCGUCGCUGCCGAUAACGGCGACCUUCCGUAGAACUCGCCUUCCUCGCAGCAGAUCACCCAAGCCCAACA
env-73    UGAUCCCCGGCGUCUCAUGGGUGCCGACCCAGCCUUCAUAGAUCAUGCGGAACGCCACGAAGACGAUCACGAUCAGGCCGAUAUAGGCGAUCCAGGGAUAGC
env-74    UGAUCCCCGGCGUCUCAUGGGUGCCGACCCAGCCUUCAUAGAUCAUGCGGAACGCCACGAAGACGAUCACGAUCAGGCCGAUAUAGGCGAUCCAGGGAUA..
env-75    ......................................................................................................
Psp-1-1   CAGCGACAGGGAGGUCAGCACCAACAGGGGUUUGAUCAACCCGGCGCCUUUGGUCUGGGCCAGGGUGGCGCCAGCCUGUGCGCCGGCAAUCUGCGCUACCCC
Psa-1-1   CGACGCAAUCAGUGCACAAUGAGAUACCGCCGCCCCGGAGGGACGGCGGCCAUCGACUUUUCGGGAUCCGAUUCGUCAGGCUGCGGGGAGCUUGUACACGCG
env-76    CGCUGCUGGGCUAGCCUUCGCCAUUUGGCCAAGCCCAGGACCCGGUAUGCAACAGGACAGGGCCCGACACACGGCCUCAAUCAAAGUCCGCAAACCACCCCG
env-77    ......................................................................................................
env-78    CGCUGCUGGGCUAGCCUUCGCCAUUUGGCCAAGACCCGGUAUGCAACAGGACAGGGCCCGACAC......................................
Ssp-8-1   CAGUGGGUGCGGCGGGUCCCGGAACAAGCCCCCGCCCGUUGACAACCCCGUGCGGCGAUGGGCAAAUGGCGUCAUGGGGAUCGCGAUCUCCCCAUGAGGCGG
Osp-1-1   CUGGCUCGAGAAGAGGGCCAGCUUGUUGCCCUGCGGGUCGCGCAGGUAACAGACGUAGAAGCGCGGCCCGUAGGCGGCGCGAAAGCCGGGGGCGCCCUCAUC
env-79    ......................................................................................................
env-80    ......................................................................................................
Rma-1-1   AAGCGGUCAUGCGGCGACGCAGCGGCAGCCCUCGGGCGCGCAACGCCACGCCCGGCGCGAAGCGGUCACUCGCGAAUCGCAAGGCAAGCGACGCGAUGCGUA
Spa-1-2   GCGAUGACACGCAGAUUCGCCACGCUCUUUAUGUGUCACAGUCUCGCUGACCGUGAUCGUCUUACACGGAAGCCUCCGCAUCAAGGCUCGCUAUCCAUCAUC
Squ-1-1   GCGAUGACACGCAGAUUCGCCACGCUUUUUAUGUGUCACAGUCUCGCUGACCGUGAUCGUCUUGUACGGAAGCCUCCGCAUCAAGGCUCGCUAUCCAUCAUC
env-81    ......................................................................................................
Sph-1-1   CUGGCGAGCGGGAACUAGCGGCUGGUUCCACAAUGUUCGCCAAGGGCAAGCGGCCGGACGGGCAUCCAUUGGCUCUGCUAUGGAGCAUUCAUGCAAUGGUUG
Pno-1-1   GCUUUCACGAUUCGCUGCUCGUCGGCGCAGACCUGAAGGGGCUCUCCUUCCGGAAACAAACGCUCGAGGCGCUCAAUUUUGCCGACGCGGACCUGUCCGACU
Bce-1-1   GCUUCCACGAUUCGCUGCUCGUGGGCGCCAAUCUCAGCGGGUUGUCGUUCCGGAAGCAGACCCUCGAAGCAUUGAAUUUCUCCGAGGCCGACCUGUCCGAUU
env-82    AUCGGCUUCCGCCGCGCUGUCGCGAGGUCGUCCGUCUACGCAAGCUUGAAGGCCUGUCCACAAAGGAAGUCGCGAGCAGGCUUGGCGUCAGCGUCAAUACCG
env-85    ......................................................................................................
env-86    CGAGCCGCAGGGGCAUUCGUCAGGACGCCAAGCGCCAAGGCCCGCAGUCCUGCUGCCCCUUUCGCAAAUGCUGCGGAACCGACGCUUUUGGCCUCUUGUGCG
env-87    AUAUGUCAGGAGCAUCAGCCUAGUUAUGCCAUGCGUUCGUAACCGGACCGAGCUAUACGUCCCAAAGUUUGCCGCAAAACGGCGCCUGUGCUGGCAGCAGCA
env-89    ......................................................................................................
env-90    ......................................................................................................
env-91    ......................................................................................................
env-92    UGUUUCAUAUGGUAAAUCGCUGGAACCGGACGUUCCGCUUUCCACCCAUCCAAGCCAUCUUCUGUCACCGGAAACCAGGUGAAUUGCGACGGGUUCCAUUAA
env-95    ......................................................................................................
env-96    ......................................................................................................
env-97    GGUAGGGUGAACUCCCACGCCUUGCACCAUUUUACGAUGCGACCCGUCCGAUGGUGCGCCCUGUCGUGUCUGCCUCCUCAAGGCGACAGGGCACCAGCACCC
env-98    GGUAGGGUGAACUCCCACGCCUUGCACCAUUUUACGAUGCGACCCGUCCGAUGGUGCGCCCUGU......................................
env-99    ......................................................................................................
env-100   GGUAGGGUGAACUCCCACGCCUUGCACCAUUUUACGAUGCGACCCGUCCGAUGGUGCGCCCUGUCGUGUCUGCCUCCUCAAGGCGACAGGGCACCAGCACCC
env-101   ......................................................................................................
env-102   ......................................................................................................
env-103   ......................................................................................................
env-104   GGUAGGGUGAACUCCCACGCCUUGCACCAUUUUACGAUGCGACCCGUCCGAUGGUGCGCCCUGUCGUGUCUGCCUCCUCAAGGCGACAGGGCACCAGCACCC
env-118   ......................................................................................................
env-119   ......................................................................................................
env-120   GGUAGGGUGAACUCCCACGCCUUGCACCAUUUUACGAUGCGACCCGUCCGAUGGUGCGCCCUGUCGUGUCUGCCUCCUCAAGGCGACAGGGCACCAGCACCC
env-121   GGUAGGGUGAACUCCCACGCCUUGCACCAUUUUACGAUGCGACCCGUCCGAUGGUGCGCCCUGUCGUGUCUGCCUCCUCAAGGCGACAGGGCACCAGCACCC
Ssp-9-1   AGAUAGCUCAUGGUUUCCCCGGGGCCGAUCGACAUUCCGGUUGAGGGAAUGAUUUGGCGGGCCGGAACCUUUCGAACCAAGACACCUCUUCCGUCAUCCCCG
Ssp-2-1   CGAUGACACGCAGAUUCGCCACGCUUUUUAUGUGUCACAGUCUCGCUGACCGUGAUCGUCUUGUACGGAAGCCUCCGCAUCAAGGCUCGCUAUCCAUCAUCC
env-122   UAGUCCACGUAGCGGUAGGCCACGCCCAGCGCGAGGUUCUUCGCCACGGCAUAGUUGGCGCCGACCUGGGCGUUGAGCAGGCGGCCGUCGUAGUCGUCGAUC
Npa-1-1   UAGUCCACGUAGCGGUAGGCCACGCCCAGCGCGAGGUUCUUCGCCACGGCAUAGUUGGCGCCGACCUGGGCGUUGAGCAGGCGGCCGUCGUAGUCGUCGAUC
Pba-1-1   CUUUCACGAUUCCCUGCUGGUCGGCGCAGACCUGCGCGGACUCUCGUUUCGCAAACAGACGCUCGAAGGACUGAACUUCUCCGAUGCGGAUCUAUCCGACUG
env-124   ......................................................................................................
env-125   CAUUGGAUGGUGAAGCGGGAAUCGGCUUGAGCACGGCAUUGGGCGAUGUUACGAAACUCGCAUGAUCCGCGUGGCUGCACACAUCCAACAACAACGCCGCAA
env-126   CAACGAUCAAGGCCGCAUCAGCAAGUCCCGACGGGCAAUGGCAAGUCAGUUUCUUCCGCUCAGCAAAGUCAUUCCGGAUGCCGCCGCGACGAGUCGCACCGC
env-127   CCACCAAUCUUGGGGGUUUGAGUUACCCUAAAAAAAACCCCUUUCCCUUUUUGGAAUACAAAGAAAUAAUAACCCCGGCACGAAGGGACCCCGGCCCGCGCU
env-128   ......................................................................................................
Ssp-3-2   ......................................................................................................
env-129   ......................................................................................................
Ssp-6-1   GGCAGCGAUCCAUCGCGAGUUGAAGCGCAAGCAUGUGACGCUGCAGAUCGUGUGGGACGAAUAUAUCGCUCAGCAUCCGGAAGGUUAUCGCUACAGCCGGUU
Peb-1-1   GGCGCCCGCUUCGUCGACGCCCGUACGCUGGGGCUUAACUUUCACGAUUCCCUGCUCGUCGGGGCCGAUCUGCGCGGACUGUCCUUUCGCAAACAGACGCUC
env-130   GUAGUCCACGUAGCGGUAGGCCACGCCCAGCGCGAGGUUCUUCGCCACGGCAUAGUUGGCGCCGACCUGGGCGUUGAGCAGGCGGCCGUGGUAGUCGUCGAU
env-131   GUAGUCCACGUAGCGGUAGGCCACGCCCAGCGCGAGGUUCUUCGCCACGGCAUAGUUGGCGCCGACCUGGGCGUUGAGCAGGCGGCCGUCGUAGUCGUCGAU
env-132   GUAGUCCACGUAGCGGUAGGCCACACCCAGCGCGAGGUUCUUCGCCACGGCAUAGUUGGCGCCGACCUGGGCGUUGAGCAGGCGGCCGUCGUAGUCGUCGAU
Sxe-1-1   AGUAACCGCCGGCUAGUGCCGAGACUUCGCGCGAACGCGCCACCGUCCAUGAUCAACGCAAUCCUCCCUCGCCAAACGUCGCAAAGGAAAUUUAUGUCUAUC
Ssp-23-1  UGUUGACCGACGUGUCGAGCUCGUUAUCCAUCAUGGAAGCAGUGCACACCGGAUACCGGUCAUCGCCACGCCGGGCCAUGCUGAGAUCUUUAUAUCGGCGAA
Esp-2-1   ......................................................................................................
Esp-1-1   AGUUGCCGGCCCGCUUUCAGGCAUCCGCAGGGGAAGCCUGAACGUCCCGAUGUAGGGCGUAUUGUGUUGAAAAACUCGGCCUGACCCGAUCCGCAGCCGAUU
env-133   ......................................................................................................
Efl-1-1   AUAAACUGCCGAAAAGCUGCCGUUCACAGUUAAUUCGCGUUGUAAUACAACACCUUAGUCAAAAAUCGGAAUCGAUAACAAUUUCAUCCGCGAAGGCGCGGU
env-134   CCGCAUUUGGGAAUUAGGUAUCUCGCAUUUUAUGGCCGAGAUCGGGUC......................................................
env-135   ......................................................................................................
env-136   ......................................................................................................
env-137   CGCUUUUGGGAAUUAGGUAUCUCGCAAUUUAUGACCGAGAUCGGGUCGGAAGCAGAAUGGCAGAUUUUCGCGCUUUUUUGACUGGAACCGGACAGUCAGCUC
env-138   ......................................................................................................
env-139   CGCUUUUGGGAAUUAGGUAUCUCGCAAUUUAUGACCGAGAUCGGGUCGGAAGCAGAAUGGCAGAUUUUCGCGCUUUUUUGACUGGAACCGGACAGUCAGCUC
env-155   ......................................................................................................
env-156   GCCUGCUCGGGCUGGCCUCGACAGUGCUUUUCGCGAUAGCCUAGAUAGCGACGAUGUAGCGGCAGAUCCCCCACAGAUAUUCGCCGGAUCAGUCGCCGACGA
env-157   ......................................................................................................
env-158   GCCUUGAUGGCGACGAUGUAGCGGCAGAUCCGCACAGAUAUUCGCCGGAUCAGUUGCUGGCGACAAGUCAGCCGAGAAUUUGGCCGAAACCUGAAACCCCCA
env-160   ......................................................................................................
env-161   CGCUACCGAUACAGUCCGGCCUGGCUGUUAUCGGCGGUGUAUGCGGAGUGGCGGCUUGGUAUUUAUCGACCCAAUGGUUGUGGAUCGCUGGAAGCAUCGCAC
env-162   CCUUGAUGGCGACGAUGUAGCGGCAGAUCCGCACAGAUCUUCGCCCGAUCAGUCGCUGACGACAAGUCAGCCGAGAAUUUGGCCGAAACCUGAAACCCCCAA
env-163   ......................................................................................................
env-165   CCUUGAUGGCGACGAUGUAGCGGCAGAUCCGCACAGAUCUUCGCCCGAUCAGUCGCUGACGACAAGUCAGCCGAGAAUUUGGCCGAAACCUGAAACCCCCAA

          ......................................................................................................
          ......................................................................................................
          oooooooooooooooooooooooooooooooooooooooooooooooooooooooooooooooooooooooooooooooooooooooooooooooooooooo
```

```
env-166   ......................................................................................................
env-168   CCUUGAUGGCGACGAUGUAGCGGCAGAUCCCCACAGAUCUUCGCCCGAUCAGUCGCUGAAGACAAGUCUGCCGAGAAUUUGGCCGAAACCUGCCAGUCCGAA
env-169   ......................................................................................................
env-170   CUUGAUGGCGACGAUGUAGCGGCAGAUCCGCACAGAUAUUCGCCGGAUCAGUUGCUGGCGACAAGUCAGCCGAGAAUUUGGCCGAAACCUGCCAGUCCGAAC
env-171   CUUGAUGGCGACGAUGUAGCGGCAGAUCCCCACAGAUAUUCGCCGGAUCAGUCGCCGACGACAAGUCAGCCGAGAAUUUGGCCGAAACCUGCCAGUCCGAA
env-172   CCUUGAUGGCGACGAUGUAGCGGCAGAUCC........................................................................
env-173   CUUGAUGGCGACGAUGUAGCGGCAGAUCCNCACAGAUCUUCGCCCGAUCAGUCGCUGANGACAAGUCNGCCGAGAAUUUGGCCGAAACCUGCCAGUCCGAAC
env-174   ......................................................................................................
env-175   CCUUGAUGGCGACGAUGUAGCGGCAGAUCCG.......................................................................
Esp-3-1   CCUUGAUGGCGACGAUGUAGCGGCAGAUCCCCACAGAUAUUCGCCGGAUCAGUCGCUGAUGACAAGUCAGCCGAGAAUUUGGCCGAAACCUGACAGUCCGA
env-176   GCCUUGAUGGCGACGAUGUAGCGGCAGAUCCGCACAGAUAUUCGCCGGAUCAGUUGCUGGCGACAAGUCAGCCGAGAAUUUGGCCGAAACCUGCCAGUCCGA
env-179   GCCUUGAUGGCGACGAUGUAGCGGCAGAUCCGCACAGAUAUUCGCCGGAUCAGUUGCUGGCGACAAGUCAGCCGAGAAUUUGGCCGAAACCUGCCAGUCCGA
env-180   ......................................................................................................
env-181   ......................................................................................................
env-182   ......................................................................................................
env-183   CUGGCUGUUAUCGGCGGUGUAUGCGGAGUGGCGGCUUGGUAUUUAUCGACCCAAUGGUUGUGGAUCGCUGGAAGCAUCGCACUUCUUGG.............
env-184   ......................................................................................................
env-185   ......................................................................................................
env-186   ......................................................................................................
env-187   UCAGGCCGUUCAUCCAGAACAGGCAGGCCCCGACAUAAGGGCCCAACUUGUGUUCUGGGGGCGCCUUCACGAUAUAAGAAGCCUGCUCGGUCUGGCCUCGAC
env-192   ......................................................................................................
env-194   UCAGGCCGUUCAUCCAGAACAGGCAGGCUCCGACACAAGGGCCCAGCUUGUGGCCUGGGGGCGCCUUCACGAUAUAAGAAGCCUGCUCGGGCUGGCCUCGAC
env-195   UCAGGCCGUUCAUCCAGAACAGGCAGGCCCUGACAUUAGGGCUCAGCUUGUGUCCUGGGGGCGCCUUCACGAUAUAAGAAGCCUGCUCGGGCUGGCUUCGAC
env-196   ......................................................................................................
Ssp-1-1   GAUAGCACAUGGUGGCGCUCCCCAUUUAUGGGUUCACGGCGUAAGUGAGCCGUGACGGAAAGAGAGCCGCCGCCGCCGCCGCUCUCGCUUCCGGAUAGGCCA
Nsp-1-1   UACUAAUUAUCCGAUAAAGCGCGGCUGGCAAGUGCGAUUGUUCGCCCUUGUUCCUUCCUGCCUGAAGGGCAUCGGCACCCUGCGCGGUCUUGUCCGCUACCG
env-197   GUGGCUCGCGGAGAGGCCGCCGUCCACCACCAUGGCGUGUCCGUUAACGUAGCUGCUCUCGUCCGAGGCGAGGAAGAGGGCGGCCUUGGCGAUCUCGUCGGG
Ssp-15-1  UCCGCUAGCGACCGGAAGCUGACCCCAUCCCCAUAACCCGUUCGCUCGACACGAACGGCAUUUGAGAACGGCUUGAACCAACCCUCCCCUUCUUCGUCACCC
Ssp-18-1  CCCCGGAAGUCCGCUAGCGACCGGAAGCUGACCCCAUCCCCAUAACCCGUUCGCUCGACACGAACGGCAUUUGAGAACGGCUUGAACCAACCCUCCCCUUCU
env-198   ......................................................................................................
Ssp-16-1  GCUAGCGACCGGAAGCUGACCCCAUCCCCAUAACCCGUUCGCUCGACACGAACGGCAUUUGAGAACGGCUUGAACCAACCCUCCCCUUCUUCGUCACCCCGG
env-199   ......................................................................................................
env-200   ......................................................................................................
Sdo-1-1   GCUUGCCGUGGCCGCAUAACCCGGCUGUCCCGAUCAACCAUCGUUUCUGGGGAAAAGCGGCUAGGUUCAGGGUCGGCUAUGCAGGUCGGCGUUCCUGUAAGC
env-201   GCUUGCCGUGACCGCAUAACGCGGCUGUCCCGAUCAACCAUCGUUUUCGGGAAAGGCGAUGCCACGCUAUGUCGGCUAUUGCCACCGGCGCUCCCGUAAGCG
Bro-1-1   GCUUCCUCGCCACCCCUUAGGUCCGCUCUCCACCCAGCCCGGCGGUAGGCCGGGUCGGGGUAUCACCGCCCGGGCCCACCCGAACCGGCCAUAGAACCGAAA
Bro-1-2   GCUUCCUCGCCACCCCUUAGGUCCGCUCUCCACCCAGCCCGGCGGUAGGCCGGGUCGGGGUAUCACCGCCCGGGCCCACCCGAACCGGCCAUAGAACCGAAA
env-202   ......................................................................................................
env-203   GGGGUUUGCUGGCUUAAUGACGGUUCGUGAUGUUUGCUUGCCGGAACCUGCUGUUCCGGAUUGAUCGACAUUGCGACCUUUCAGCCCUCAAUCAGUUCGGCC
env-204   ......................................................................................................
env-205   ......................................................................................................
env-206   GGCUGGCCGUCGCCAGCGGCUUGGCGGGACUGAUUGUCGGAUAUUACUCAGCCGAUUGGCGGUGGUUUGCGGGAAGCAUCCUGAUACUCGCCAACUGGCCGU
env-208   ......................................................................................................
env-209   ......................................................................................................
env-210   ......................................................................................................
env-211   CUUGGCCGGGGCUGGCUAGGGACCGUAAUCAAAUCGGCCACCCGGCAGGCCUCACCCCUUCCGGUUAACCGGCGGCUCG.......................
env-212   ......................................................................................................
env-213   GUUGGCAGGAUAACAGCAGCUCUCCACCCUAAACUCGCCGUCGCCCCGGACUUGGUCCGGGGCCCUGCUUGCUGUGGCUUCGCUCGAAAAAGCAAGCUUAGG
env-214   AUUGGCCAGCGCGGGCUAACGACCUGCUUUCCAUUCAGCCCGCAGGUCGCAUUCACUGAAAUCGGCCGCACGUCGACAUGAACGAGUUUUCGCUACUCCACC
env-223   ......................................................................................................
env-224   AUUGGCCAGCGCGGGCUAACGACCUGCUUUCCAUUCAGCCCGCAGGUCGCAUUCACUGAAAUCGGCCGCACGUCGACAUGAACGAGUUUUCGCUACUCCACC
env-225   AUCGGCAGGCGCCGGCUAACGACCGCUUCCCACCCCAGCUGCCAUAGUGCCCCGCCGUGCUUCCUUGUAAGCCGACGAUCCGCCCAAUUCGCAAUUUGUCCA
env-226   CUCGGCAGGCGCCGGCUAACGCCUGAUUGGUCACGUUCCCGACAGGCGGCUUUUCGCGGCGCGAUCAGCAAUCAUGCCGUUCAUUGAGAGCCAGCCUGUACG
Ssp-8-2   GUUGGCGGCUGCAGGCUGACGCGCCACUCACCCCUCAAUCCUCCCCCGCCCGGGGGGGUGGGCAUACGAAGCAGACGGAGGGGAGGAUGCACGACGGCUGAU
env-227   GUUGGCAGGCGCAGGCUAAACGGCCGCUUCGACCAUUUUUCUCGUGCAGCGGCUGAACAGCGGGCGCCGGGCCUGAUAGUCGACCGGCGGGUUUCGGGUGGG
env-228   ......................................................................................................
env-229   GUUGGCAGGCGCAGGCUAAACGGCCGCUUCGACCAUUUUUCUCGUGCAGCGGCUGAACAGCGGGCGCCGGGCCUGAUAGUCGACCGACGGGUUUCGGGGUGG
env-235   GUUGGCAGGCGCAGGCUAAACGGCCGCUUCGACCAUUUUUCUCGUGCAGCGGCUGAACAGCGGGCGCCGGGCCUGAUAGUCGACCG................
env-236   GUUGGCAGGCGCAGGCUAAACGGCCGCUUCGACCAUUUUUCUCGUGCAGCGGCUGAACAGCGGGCGCCGGGCCUGAUAGUCGACCGACGGGUUUCGGGGUGG
env-237   GUUGGCAGGCGCAGGCUAAACGGCCGCUUCGACCAUUUUUCUCGUGCAGCGGCUGAACAGCGGGCGCCGGGCCUGAUAGUCGACCGACGGGUUUCGGGUGGG
env-238   GUUGGCAGGCGCAGGCUAAACGGCCGCUUCGACCAUUUUUCUCGUGCAGCGGCUGAACAGCGGGCGCCGGGCCUGAUAGUCGACCGGCGGGUUUCGGGUGGG
env-239   GUUGGCAGGCGCAGGCUAAACGGCCGCUUCGACCAUUUUUCUCGUGCAGCGGCUGAACAGCGGGCGCCGGGCCUGAUAGUCGACCGACGGGUUUCGGGGUGG
env-240   ......................................................................................................
env-241   UCACGCUAUUCGUCAUCAUGCCCGUCAACAAACGCUUGAUGGCGAUGCCGGUAAGCGAGGCGGGCGCUGAAAGCCGCGCGAUGCUGGUUCAGUGGGGAAAGC
env-242   ......................................................................................................
env-243   ......................................................................................................
env-244   GUUGGCAGGCGCAGGCUGACGACCACGACGUAUUGAUCUCGGCGGUAGACGAUUCGGCCUGCAAAUCGCCGCUCGUCCCGCGAUCAACCUGAUUCGCUCAAC
Swi-1-1   AUUAGCAAGCGCAGGCUAACGACCGCUUCCCCUAAAUACGCCGAUAGCGUCGCCGGGUACACCGCCGCUGAGUAUCCUACCUGAUUGAAGCCGGCGCGGACG
Ssp-5-1   GCGUUGGCUGGCUAAUGGCAGCUCUCCACCCGUUGUCGGCCGUUCCGCAUCGACGCUGUCGACUCCAAAAGCGGCCGUUCUGCUUCCGCCCCAAUUGCGGAC
env-245   ......................................................................................................
env-246   UUCUUUAUCUGGGCGCUGCUCGCUGCCGGGUGAACCUCAUUCGCAGUCGUCGGCGAGCUCCGGCGUGAUCUCGCGCGGGAGGAGGGCGAAGCGGAAGCGGUA
env-247   ......................................................................................................
env-248   ......................................................................................................
env-249   ......................................................................................................
env-250   ......................................................................................................
env-251   ......................................................................................................
env-252   ......................................................................................................
env-253   ......................................................................................................
env-254   ......................................................................................................
env-255   UCUCGCACGGCAUCGCCGCGCAGAACCGCAAGGCCCGCUACGACUACAAGAUCGGCGAGACCAUCGAGGCCGGACUGGCGCUGGUCGGGCCGGAGGUGAAAU
env-256   ......................................................................................................
          ......................................................................................................
          ......................................................................................................
          oooooooooooooooooooooooooooooooooooooooooooooooooooooooooooooooooooooooooooooooooooooooooooooooooooooo
```

6 GGAM-6

6.1 Taxa

The taxonomy of each organism containing a putative GGAM-6 RNA is listed, with abbreviations identifying each hit (e.g., "Eco-1-1" and "Eco-1-2" might hypothetically represent two distinct RNAs in *E. coli*). The abbreviations will be used to identify each individual GGAM-6 RNA in Sections 6.2 and 6.4:.

abbrev. of hits	taxonomy of species
Sru-1-1 to Sru-1-5	Bacteria Firmicutes Negativicutes Selenomonadales Selenomonadaceae *Selenomonas ruminantium*
env-1 to env-3	environmental samples

6.2 Gene contexts

Each GGAM-6 RNA (indicated by "RNA→") is listed. For each hit, the downstream genes predicted to reside in a regulated operon are listed. If the nearest downstream gene is encoding in the opposite strand (and therefore presumed to not be a part of a regulated operon), then that gene is still depicted. Some environmental sequences and some RefSeq entries lack gene annotations, and so no genes are available for such sequences. The direction of each gene is indicated with an arrow (→), and each predicted conserved domain in the gene is named. Conserved domains associated with more than one GGAM-6 RNA are assigned a color; other domains are gray. Information about these conserved domains is given in Section 6.3. The accession of the sequence containing each GGAM-6 RNA is given in the column named "Seq. accession". Accessions beginning with "NC_", "NS_", "NW_" or "NZ_" are contained in RefSeq. Other accession refer to environmental samples. Nucleotide coordinates are given for the 5′ and 3′ boundaries of each GGAM-6 RNA. If the 5′ coordinate is greater than the 3′ coordinate, the RNA is present on the reverse-complement strand of the containing genomic DNA sequence. Each hit is denoted by an abbreviation (like "Eco-1-1") that refers to a taxonomy given in Section 6.1.

abbrev.	Seq. accession		5′ at	3′ at	genes
Sru-1-1	NZ_FNCM01000005.1	+	68373	68911	**RNA**→ emrE (PRK09541)EmrE (COG2076)→ acidPPc (smart00014)PAP2_like_2 (cd03392)→
env-1	Ga0256405_10361186	-	839	354	**RNA**→ EmrE (COG2076)→
Sru-1-2	NZ_FOQK01000026.1	+	26558	27100	**RNA**→ emrE (PRK09541)EmrE (COG2076)→
Sru-1-3	NZ_FPAX01000001.1	+	436504	437042	**RNA**→ emrE (PRK09541)EmrE (COG2076)→
env-2	Ga0256404_1172595	-	784	246	**RNA**→ emrE (PRK09541)EmrE (COG2076)→
env-3	Ga0256405_10313215	+	107	645	**RNA**→ emrE (PRK09541)EmrE (COG2076)→
Sru-1-4	NZ_FNCM01000005.1	+	30556	31094	**RNA**→ hypo→
Sru-1-5	NZ_FOJX01000001.1	+	174807	175343	**RNA**→ emrE (PRK09541)EmrE (COG2076)→

6.3 Conserved domains

Conserved domains found in protein-coding genes listed in Section 6.2 are shown below, with the first sentence in their description from the Conserved Domain Database (if any). Conserved domains associated with more than one GGAM-6 RNA are assigned a color, while others are shown in gray. The number in parentheses after the colored domain name is the number of occurrences in Section 6.2.

cd03392 (1) PAP2_like_2 proteins.

COG2076 (7) Multidrug transporter EmrE and related cation transporters [Defense mechanisms]

PRK09541 (6) multidrug efflux protein; Reviewed

smart00014 (1) Acid phosphatase homologues.

6.4 Multiple-sequence alignment

Each GGAM-6 RNA is denoted by an abbreviation (like "Eco-1-1") that refers to a taxonomy given in Section 6.1. The alignment may include sequences containing the RNA motif, as well as flanking sequence. The GGAM-6 RNA itself is denoted by the line underneath marked 5′ and 3′ on either end. Nucleotides in flanking sequences (i.e., not part of the motif) are written in gray letters. Stems of predicted rho-independent transcription terminators, if any, are shaded yellow. (Note: terminator predictions have not been analyzed manually, and many are likely to be false positives. Terminator predictions are those of the RNIE software.) Nucleotides predicted to function (as DNA) as transcription-factor binding sites, if any, are shaded in green. (But note: these predictions are manually annotated, so they might be under-predicted.) Annotated start codons, if any, are shaded green. (Note: start codons are frequently misannotated, especially in environmental samples.) Nucleotides proposed to basepair as part of the consensus structure are shaded in color when they comprise Watson-Crick or G-U pairs. Otherwise they are shaded gray. Conserved stems are also indicated at the bottom of the alignment by angle brackets, where matching < and > denote base-paired columns. Below these angle brackets, the symbol "2" denotes base pairs exhibiting covariation according to the statistically well-founded R-scape method. "1" denotes base pairs exhibiting covariation according to R2R's simplistic method. "0" denotes base pairs that are not observed to mutate and "?" denotes base pairs that have a significant frequency of non-canonical nucleotides for Watson-Crick or G-U pairs (> 5%). Below these base pair annotation is the consensus sequence: "R" = "A" or "G", "Y" = "C" or "U", red nucleotides: nucleotide identity conserved more than 97% of the time, black nucleotides: 90%, gray nucleotides: 75%, red circle (•): nucleotide is present 97% of the time, black circle (•): 90%, gray circle (•): 75%, white circle (◦): 50%. All percentages of sequences just described (e.g. 97% conserved) assume that sequences have been weighted by the GSC algorithm implemented by the Infernal software package.

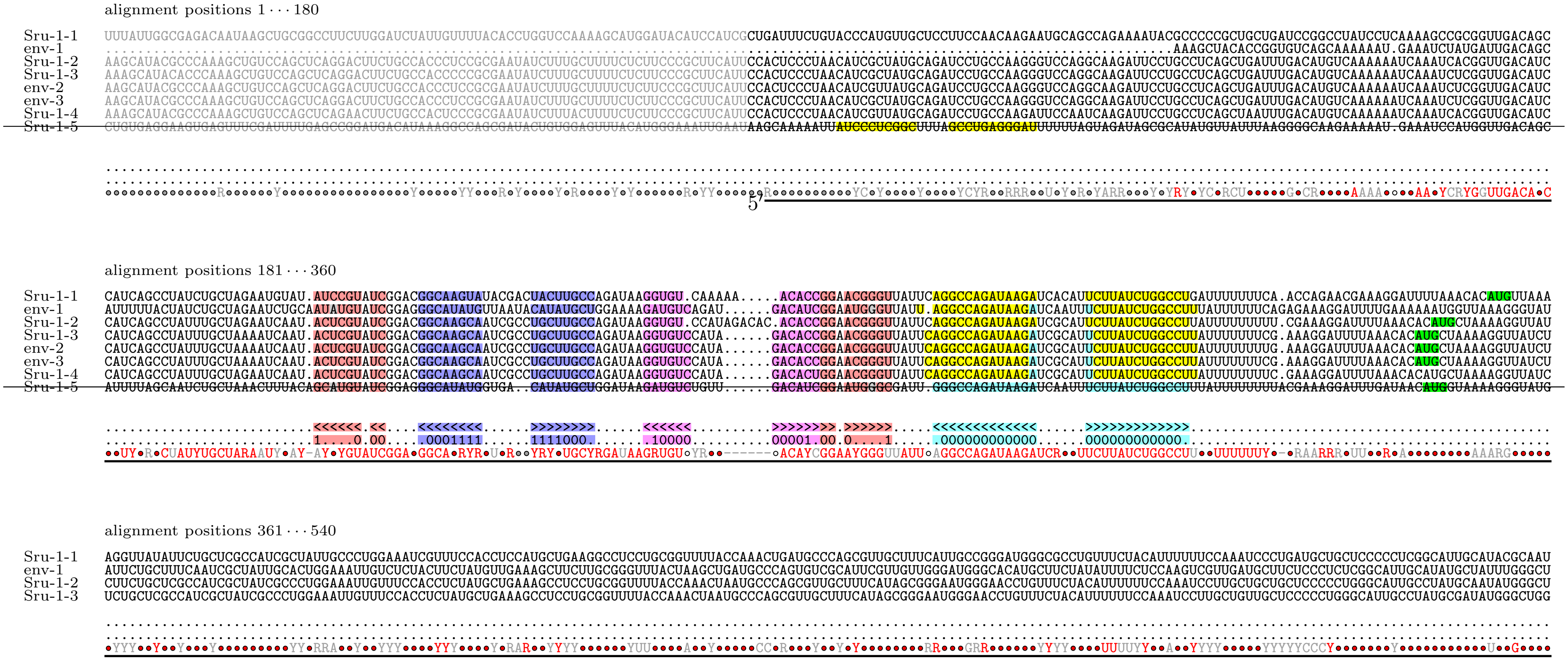

```
env-2     UCUGCUCGCCAUCGCUAUCGCCCUGGAAAUUGUUUCCACCUCUAUGCUGAAAGCCUCCUGCGGUUUUACCAAACUAAUGCCCAGCGUUGCUUUCAUAGCGGGAAUGGGAACCUGUUUCUACAUUUUUUCCAAAUCCUUGCUGCUGCUCCCCCUGGGCAUUGCCUAUGCAAUAUGGGCUGG
env-3     UCUGCUCGCCAUCGCUAUCGCCCUGGAAAUUGUUUCCACCUCUAUGCUGAAAGCCUCCUGCGGUUUUACCAAACUAAUGCCCAGCGUUGCUUUCAUAGCGGGAAUGGGAACCUGUUUCUACAUUUUUUCCAAAUCCUUGCUGCUGCUCCCCCUGGGCAUUGCCUAUGCAAUAUGGGCUGG
Sru-1-4   UUCUGCUCGCCAUCGCUAUCGCCCUAGAAAUUGUUUCCACCUCUAUGCUGAAAGCCUCCUGCGGUUUUACCAAACUAAUGCCCAGCGUUGCUUUCAUAGCGGGAAUGGGAACCUGUUUCUACAUUUUUUCCAAAUCCUUGCUGCUGCUCCCCCUGGGCAUUGCCUAUGCAAUAUGGGCUG
Sru-1-5   UUCUGCUUGCGGUCGCUAUUGCAUUGGAAAUCGUCUCUACGUCUAUGCUGAAGGCAUCCUGCGGUUUUACUAAGCUGAUGCCCAGCGCUGCUUUUAUUAUCGGAAUGGGGACGUGCUUCUAUAUUUUCUCCAAGUCGUUGAUGCUUCUCCCUCUCGGCAUUGCAUAUGCCAUUUGGGCUG

          ....................................................................................................................................................................................
          ....................................................................................................................................................................................
          •YYY••Y••Y•••Y•••••••••YY•RRA••Y••YYY••••YYY••••Y•RAR••YYYY••••••YUU••••A••Y•••••CC•R•••Y••Y•Y•••••••••RR•••GRR••••••YYYY••••UUUUYY••A••YYYY•••••YYYYYCCCY•••••••Y•••••••••••U••G••••
```

alignment positions 541 · · · 720

```
Sru-1-1   AUGGGCCGGCGUAGGAACAGCCCUUACAGCACUAGUAGCAGUAUUUAUCUGGCAUGAGCAGAUGGACUUCACCACCGGACUAGGCAUCGUCUUUAUCAUCGUCGGAGUCAUAUUAUUGAACAUCCGCAGUAUCACCAAUUAAUACAUGACCAGAAUCGUCUGCACAUUGACAAGGGUAUC
env-1     GGCUGGAACGGCCUUGACGGCAUUGGUUGCUGUAGUAAUUUGGCAUGAGCAGUUAGAUUUAACUACGGGGCUGGAAAUUUUGCUCAUCAUCGUCGGUGUAAUCUUGCUGAACAUUCGCAGCAUAACUAAUUGAGUAUGGAAGCACCAUUACGCAAAAAAUAUUAGUAAAAUAGAACUAUG
Sru-1-2   GGCGUCGGCACAGCUCUUACGGCGCUGGUGGCGGUAUUUAUCUGGCAUGAACAGAUGGACCUUACCACCGGCUUGGGCAUCUUGUUCAUCAUCAUAGGAGUGAUCCUGUUAAACAUCCGCAGCAUCACCAGUUGAUAGCGGCACAUCAUCCCAGCCAGGACGCCCCAUAGUGCCCUGAAU
Sru-1-3   CGUCGGGACAGCUCUUACGGCGCUGGUGGCGGUAUUUAUCUGGCAUGAACAGAUGGACCUUACCACCGGCUUGGGCAUCUUGUUCAUCAUCAUAGGAGUGAUCCUGUUAAACAUCCGCAGCAUCACCAGUUGAUAGCGGGGCAUCAUGCCCUGCACCACCAUGCGCAUCAACAUUCCCCC
env-2     CGUCGGCACAGCUCUUACGGCGCUGGUGGCGGUAUUUAUCUGGCAUGAACAGAUGGACCUUACCACCGGCUUGGGCAUCUUGUUCAUCAUCAUAGGAGUGAUCCUGUUAAACAUCCGCAGCAUCACCAGUUGAUAGCGGCACAUCAUCCCAGCCAGGACGCCCCAUAGCGCAUCAUGCCC
env-3     CGUCGGCACAGCUCUUACGGCGCUGGUGGCGGUAUUUAUCUGGCAUGAACAGAUGGACCUUACCACCGGCUUGGGCAUCUUGUUCAUCAUCAUAGGAGUGAUCCUGUUAAACAUCCGCAGCAUCACCAGUUGAUAGCGGCACAUCAUCCCAGCCAGGACGCCCCAUAGCGCAUCAUGCCC
Sru-1-4   GCGUCGGGACAGCUCUUACGGCGCUGGUGGCGGUAUUUAUCUAGCAUGAACAGAUGGACCUUACCACCGGCUUGGGCAUCUUGUUCAUCAUCAUAGGAGUGAUCCUGUUAAACAUCCGCAGCAUCACCAGUUGAUAGCGGCACAUCAUCCCAGCCAGGACGCCCCAUAGCGCAUCAUGCC
Sru-1-5   GUGUUGGAACGGCCUUGACAGCAUUGGUUGCUGUUGUUAUUUGGCAUGAGCAAAUGGAUUUAACAACGGGGCUGGGGAUUGUAUUCAUCAUUGUGGGCGUCAUUUUGCUUAACAUUCGUAGCAUUACUAGCUGAUUGUGGGGACACCGUUAAACAUAGAUGUUUAGCGGUGUUUUUUUAU

          ....................................................................................................................................................................................
          ....................................................................................................................................................................................
          R••••••R••R••YY••••R•••Y•R••••••••••••••Y•R•••••R••R•••GR•YY•••••••••••Y•RR•R••YY•••Y••Y••Y•••RR•R••••YYY••Y••A••••Y•••R••••••Y•R••••R••••••R•••••••YY•••••••R••••Y•••••R•••••••••••Y
                                                                                                   3′
```

alignment positions 721 · · · 900

```
Sru-1-1   CCCUAUAAUAGACCACCAUGUCUUUCUGACAUAGUACAUAAUACGAGGUAAUUUAUGGACGAAUCACUACUCCUAUUCCUACAGCAAAAUAUCCGCACCGAUUUCCUGACGCCUGUAUUGAUGCUAAUCACCAGUAUUGGCCAUUAUGGAGCCAUCUGGCUACUUAUCGCAGUCCUUUUU
env-1     GCUACGAUUUCAGGCUAUGUGCGAAGAUAUUGCGCAUAGCUUUUUUCUUGCGGUAAAGCAGGUUUUUGGCGCAGGAAUAUUGAACAUAUUUAAAAUAUGUCUCAGAGGAGGCGCGUGUUAUAGACGAGGGCAUUUUCCUAUAUGUUCCGGGUAAUCUGAUGAAUGUAGAUGAAAAACAUU
Sru-1-2   CGGCAACUCCCUAGACCUCGUCUCCCCGUGCUAAGUUCCCUUAAAGCAGCCUCUUCUUUUAUUUCAUAUUCUCCCACCAGCACGCAAGGUUCUGUUUUUUCCCACGGGCCAGAACGGCCUUCCAGGAAGGUACCGAGGCAGCCCGCAAGGAUAUUUUAGUAGAGCAGCGUGCCCGCCUGC
Sru-1-3   GCUAGCUUCAAUGGUCAAUUCUCCAUCAUGGGUCCUGGUACAAUUCCAGCAAACAGCAAUCUUGUUUGACUCGAUUACACAGUCAUUGAGAUGCCACUGGAUAUCGGAGAAGGCCGCGUGCAUCAUAUGGACGAUGAAACGAUAACCUUUUGGUCCUUUGAGUGGUUCCGACUGUGUUAA
env-2     UGCACCACCAUGCGCAUCAACAUUCCCCCGCUAGCUUCAAUGGUCAAUUCUCCAUCAUGGGUCCUGGUACAAUUCCAGCAAACAGCAAUCUUGUUUGACUCGAAAUCAAGUGUGGUUAAUAGCCACCGUGGGUUCGAAUCCCACCCUCUCUG.............................
env-3     UGCACCACCAUGCGCAUCAACAUUCCCCCGCUAGCUUCAAUGGUCAAUUCUCCAUCAUGGGUCCUGGUACAAUUCCAGCAAACAGCAAUCUUGUUUGACUCGAAAUCAAGUGUGGUUAAUAGCCACCGUGGGUUCGAAUCCCACCCUCUCUGCCAUUGAGAUUAAAGGGCUCGCGCGUUU
Sru-1-4   CUGCACCACCAUGCGCAUCAACAUUCCCCCGCUAGCUUCAAUGGUCAAUUCUUCAUCAUGGGUCCUGAUACAAUUCCAGCAAACAACAAUCUUCAGGUACAUAUCCCGCCAGUCAUCCUCGGUUUGUAUUUUCUCUGGCUCGAUAAUCCCCUGCUGGGUAAGCUGGACGUAUAUUUCGUU
Sru-1-5   ACGUAAAACAUUCUUGCCAGAAGAAAUAUUUCGUGUUACACUAUAUGUAAAUAAAUUACACAUAAGGUGAAAGGCUAUGAAGAAAGCAAGUUGGAUAGCUAUUGACCUGAAAUCGUUUUACGCGUCCGUCGAGUGCGUCGAAAGGAACCUCGACCCGCUAACUACCAAUUUGGUGGUAGC

          ....................................................................................................................................................................................
          ....................................................................................................................................................................................
          ••••••••Y•••••••••••••••••Y•••••••••Y••••UR••••••••••••••••••Y•Y•••••••••••••••R•••R•R•••Y•••••••••Y•••••R••R••••••YY••••••••••••••••••••••••••••••••YY••••R••••••••Y••••••Y••Y
```

alignment positions 901 · · · 927

```
Sru-1-1   UCCCUGCACCAAUCUACCCGCCAGCAG
env-1     UUAUGCAGGUUGUGAAGAAACUGACUA
Sru-1-2   AGGAACAGCUGGAAACCUUGCAACCCA
Sru-1-3   UCAUCGUUUCAAAUUUAGAUAUAAGUU
env-2     ...........................
env-3     UUGUGCGGGUCUUUUUUGCU.......
Sru-1-4   GGCCAGCUCAUGCUUGUCCUUAUAGCA
Sru-1-5   GGAUGAGACACGGACAGAAAAGACAAU

          ...........................
          ...........................
          •••Y•••••••R••••••••○○○○○○○
```
